# Lab perceptual training and robot-assisted training in improving speech prosody of autistic children

**DOI:** 10.1038/s41539-026-00425-7

**Published:** 2026-05-06

**Authors:** Si Chen, Bruce Xiao Wang, James Chung-Wai Cheung, Fang Zhou, Yitian Hong, Bei Li, Angel Chan, Tempo Po Yi Tang, Bin Li, Zhuoming Chen, Chunyi Wen

**Affiliations:** 1 Department of Language Science and Technology, The Hong Kong Polytechnic University, Hong Kong, China; 2 Research Centre for Language, Cognition, and Neuroscience, The Hong Kong Polytechnic University, Hong Kong, China; 3 The Hong Kong Polytechnic University—Peking University Research Centre on Chinese Linguistics, Hong Kong, China; 4 Research Institute for Smart Ageing, The Hong Kong Polytechnic University, Hong Kong, China; 5 Department of English and Communication, The Hong Kong Polytechnic University, Hong Kong, China; 6 Department of Biomedical Engineering, The Hong Kong Polytechnic University, Hong Kong, China; 7 The Hong Kong Child and Youth Services, Hong Kong, China; 8 Department of Linguistics and Translation, City University of Hong Kong, Hong Kong, China; 9 The First Hospital of Jinan University, Guangzhou, China

**Keywords:** Neuroscience, Psychology, Psychology

## Abstract

Children with autism spectrum disorder are known to exhibit both social and language difficulties. Speech prosody is known to be easily noticeable, which has been shown to have far-reaching influences in the academic and social life of autistic individuals. This study examined two training programs on the speech prosody of autistic children, who tend to avoid social speech signals. The first program is a lab perceptual training program without social interaction, while the second utilizes a social robot to provide training with controlled, simulated social interaction. Ninety-two children in total were recruited with sixty-nine participants formally diagnosed with ASD and twenty-three children were typically developing children without any language or speech disorder. Our results showed that both lab perceptual training and robot-assisted training with simulated social interactions led to improvement in the use of speech prosody by autistic children. Although social interaction is considered critical in language acquisition for typical population, autistic individuals tend not to prefer social speech signals, which is hypothesized to lead to their social and language deficits. This study hence proposes two successful alternative ways to facilitate their learning of language through lab perceptual training and simulated human-robot interaction.

## Introduction

In the DSM-5^1^, Autism Spectrum Disorder (henceforth ASD) is a neurodevelopmental disorder and patients are characterized by deficits in social communications and interactions as well as repetitive and restrictive behavior. It is known that autistic children may show deficits in using prosodic cues to mark information structure. Information structure is the structure reflecting pragmatic functions and it has categories of topic and focus, where topic can be considered as previously given information and focus as new information^2^. Prosodic prominence may be used to mark new information^3^.

Autistic children may also show abnormal speech prosody in general^4^ and in using communicative functions^5–7^. Tonal-speaking autistic children are facing the difficulties in using the same acoustic cues for multiple communicative functions such as lexical contrasts and focus marking^8^. It is also known there is a high heterogeneity in the use of prosody by autistic population^9^. Some established methods have also been reported to be effective in improving speech prosody of autistic children such as antecedent-based intervention, vivo modeling, pivotal response training, video modeling and technology-aided intervention. Due to the limited number of studies, it can be only concluded that established interventions targeting at prosody for a period of time is likely to result in improvements^10^.

In addition, social interaction is known to be a key factor in language acquisition as language evolved in communication and speakers make subtle adjustments in considering audience. People engaged in social interaction may be more aroused and attentive so that the abilities to store information and generate output might be enhanced^11,12^. As proposed by Mayer et al.^13^, social agency theory indicates that human or human-like characteristics will be more appealing to learners, which may elicit more social-affective responses and deeper cognitive processing. In turn, better learning performance can be achieved. A recent study also showed that onscreen presence of the instructor led to better learning results compared to no-instructor presence condition^14^.

Early studies reported the importance of social interaction in language learning. For example, babies show a decline in the ability to discriminate foreign sounds between 6 and 12 moths, and live interactions may reverse this decline more than presentation of pre-recorded stimuli^11^. For the learning of phonemes or words, displays of videos and audio recordings may not be very effective^15,16^. Theories such as the levels of processing theory^17^ and dual encoding theory^18^ suggest an advantage of multimodal sensory information to help improve the memory quality and lead to better learning results in adults. Training with social interactions might bring positive behavioral outcome and brain changes and it has positive effects in language acquisition by typically developing population. Neural imaging studies also supported the advantage of social learning^19^.

However, for the population with social communication deficits, social interaction may be demanding as multisensory information needs to be processed during the interaction and social speech signals may not be favored. Neuroimaging studies revealed that autistic individuals showed a significant increase in interregional temporal correlations while carrying out spontaneous conversations compared to controls, which may be due to greater sensory demands with social components^20^. The increase found in their neural connections may account for their frustrations in social interactions. In addition, autistic children have been shown to prefer non-social speech signals over social signals (child-directed speech) supported by both behavioral and neural evidence, different from typically developing children, which may lead to decreased social and language abilities^21,22^. It is thus argued that social impairments in children with autism may, in fact, influence their ability to acquire language. In a social setting, the speaker uses these prosodic cues to guide the listener’s heightened attention toward the most critical part of the message. To be able to use speech prosody to mark focus, children need to learn that the prominence of acoustic cues, such as the lengthening of the syllables on focus represents that these syllables deliver new or contrastive information. Without social interaction, it might be challenging for them to establish such an integration of acoustic cues and information structure from conversations and understand the function they can achieve in using prosodic cues. Whether training with no interaction and simulated interaction lead to improvement in language acquisition is unknown.

Prosodic cues are employed in focus marking by the typical population cross-linguistically in addition to morphosyntactic structures. Three types of focus are often examined in studies of focus marking, including broad, narrow focus, and contrastive focus^23^. On-focus words usually show higher f0 mean values or wider f0 range, longer duration and higher intensity. Post-focus compression (PFC) is also found in languages, which refers to the compression of acoustic cues on post-focus words, though it is not a universal phenomenon^24^. PFC was not found in Cantonese for typical adult speakers^25^.

Typically developing (TD) children may show different strategies in using acoustic cues. Four-to-five-year-olds could not mark focus in Dutch like adults but 7-to-8-year-olds were found to have developed the ability in using accent types to mark focus^26^. Similarly, 4-to-5-year-olds used duration to mark focus in Mandarin, but they failed to use pitch span like adults. By the age of seven to eight, they have further developed the ability to use pitch span in some situations. By the age of ten to eleven, their usage become adult-like in most situations^27^.

Children with ASD speaking non-tonal languages have difficulties in producing and perceiving stress in pragmatic and affective contexts^6^. Topic and focus were usually produced equally or the beginning of a sentence was produced with prosodic prominence by autistic children even if the focus was not at the beginning^5,6,28,29^. In addition, tonal-language-speaking autistic individuals have also exhibited abnormal speech prosody and focus marking. Higher f0 variability was found in Cantonese-speaking autistic adults compared to neurotypical population^30^. Regarding prosodic focus marking, it is revealed that acoustic contrasts between focused and non-focused syllables are often less distinct in autistic speech compared to TD speech^31,32^. Studies using PEPS-C, a battery designed to assess prosody across functional communicative domains such as interaction and affect have consistently identified significant impairments in both the perception and production of prosody among autistic individuals^32–34^. DePape et al.^34^ noted that children with moderate language skills—rather than those with high proficiency—were more likely to use f0 range to signal information structure. However, their use of f0 was not always accurate and may have been influenced by prior clinical interventions. Chen et al. reveal that children with ASD exhibit reduced on-focus expansion in f0 and duration and produce less distinct lexical tone shapes compared to typically developing (TD) peers in marking focus of Cantonese^35^. Autistic children were not able to distinguish among information structure categories using prosodic cues perceptually^36^. They were reported to be less accurate in producing focus in initial and final positions compared to TD children. These results may suggest that autistic individuals have difficulties in integrating information from multiple levels of language in general, namely the mapping between acoustic cues and information structure. In turn, they may have difficulties in using prosody to mark focus in their speech production.

Atypical speech prosody is a critical sign of autism that sets autistic individuals apart from their typically developing peers^37,38^. Atypical prosody may exert negative influence in many aspects of autistic individuals’ life^20,39^. General low quality of studies on interventions targeting at speech prosody has been reported and perceptual ratings of the training results may have problems in accuracy and reliability^32^. Limited studies have been conducted on intervention methods in improving speech prosody, it is summarized that autistic individuals improved in speech prosody more in structured, targeted and discrete tasks compared to naturalistic tasks^10,32^.

Short-term lab perceptual training has been shown to be effective in improving perception of segmentals (vowel and consonants) and suprasegmentals (tones) in language acquisition by typically developing population. It has been demonstrated to be effective in modifying perceptual mechanisms and in turn improve speech production^40,41^. This linguistically based training method is also structured and targeted, which is likely to modify the mapping between acoustic cues and information structure by autistic children.

However, lab perceptual training does not include a social component, which is shown to be critical in language learning by typical population. Li and Jeong^19^ reviewed extensive studies showing the positive effects of training with social interaction in language acquisition by typically developing population. Autistic children are known to have deficits in social interaction and tend not to prefer social speech signals^21^. It is yet unknown how social interaction plays a role in the intervention for autistic children to improve their speech prosody.

Moreover, autistic children are known to feel frustrated interacting with people since their behavior is not entirely predictable and less consistent compared to robots^1,42,43^. Using robots to enhance social skills in autistic children assisting children with ASD to improve their social skills is based on the concept of human–robotic interaction (HRI), which is considered as the dynamic relationship between robots and humans^44^. It has been reported that autistic children showed increased activity engagement^45,46^, eye-contact engagement^47,48^ and speech engagement^45,49^ when interacting with robots compared to humans. Studies also show that social robots are generally effective for instructing a variety of social skills such as eye contact, basic greetings, and general social interaction^50^. A variety of robots such as Nao, Lego Mindstorms, Probo and Kspar have been used to help children improve their social and communication skills. The social skills improved include imitation, attention, eye contact, emotion recognition and regulation, social routines, turn taking, help requesting and conflict management^51–55^. It is argued that robots can help in the social development because it may motivate autistic children to learn^56^ and it may provide a chance to model and practice behaviors with the robot^57^.

However, it is noted that the number of studies conducted on the training effects of robots is still limited and most studies focused on social skills without including a control group^55,58^. Although training with social interaction has been shown to improve language learning for typical population and human-robot interaction has been shown to be effective in improving various social skills, the role of simulated social interaction in robot-assisted training in improving the acquisition of prosody by autistic children remains to be explored.

What lab perceptual training provides is a chance to expose children with fixed matched and mismatched conversations so that they may compare and learn how acoustic cues are used to mark information structure. However, it does not contain the social component, which is key in acquiring subtle aspects of language and for generalizing the use of prosodic cues in a social setting.

Interventions for children with ASD are often complicated by the communicative frustration arising from the inherent unpredictability and inconsistency of human social behavior. Robots may offer a more structured and predictable interface, which might be better than a decontextualized lab perceptual training, as social interaction remains a fundamental pillar of language acquisition. We propose that robot-assisted intervention can bridge this gap by providing a simulated social environment with controlled exposure. This approach maintains the necessary social components of learning while mitigating the stressors of human-to-human interaction, potentially offering a more effective method. It is expected that prosodic cues might be better acquired and mapped with information structure in the controlled simulated social interaction.

The current study proposes three hypotheses to be tested as follows:

**H1:** Lab perceptual training improves the mapping between acoustic cues and information structure by autistic children and leads to better usage of prosodic cues in speech production in human-human interaction.

**H2:** Robot-assisted training improves the usage of prosodic cues in encoding communicative functions in human-human interaction through simulated social interaction with the Furhat robot.

**H3:** Robot-assisted training with SSI leads to more improvement in using prosodic cues compared to lab perceptual training due to its social component, which is a key factor in language acquisition.

## Results

### Cantonese typically-developing (CTD) group

**Fig. 1:**
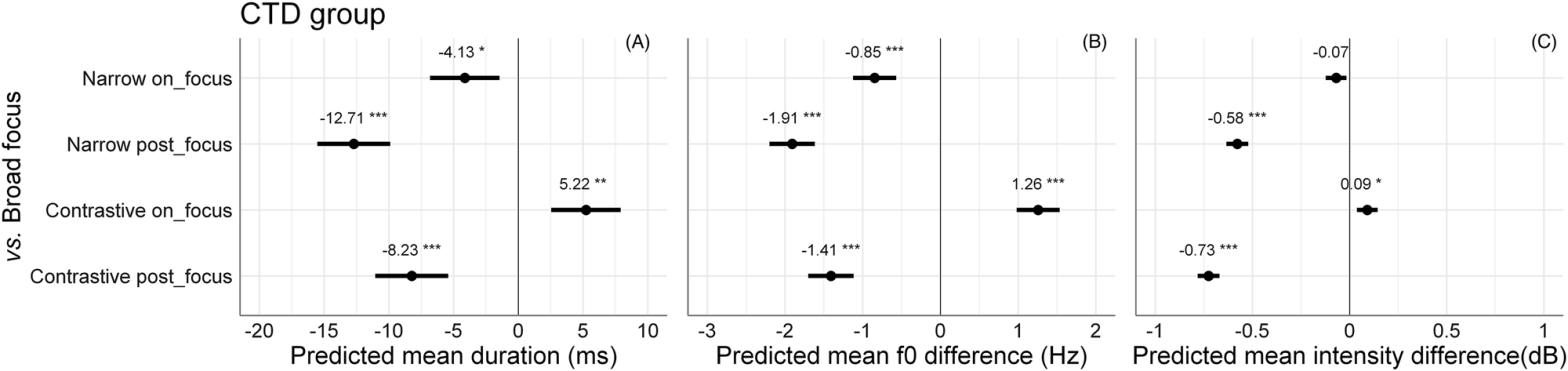
Focus marking by CTD group. Differences in predicted mean duration (**A**), f0 (**B**) and intensity (**C**) between broad focus, narrow and contrastive on- and post-focus syllables for the CTD group.

For children in the CTD group, results from the linear mixed effects models (LMM) showed that focus condition reached significance for mean duration (*χ*^2^ = 301.18; df = 6; *p* < 0.001), mean f0 (χ^2^ = 1553.2; df = 6; *p* < 0.001), mean intensity (*χ*^2^ = 1193.3; df = 6; *p* < 0.001). Post-hoc comparisons showed that the TD group produced post-focus words with significantly shorter duration, lower mean f0 and intensity than the broad focus words under both narrow and contrastive conditions, whereas they only produced contrastive on-focus words with significantly longer duration and higher f0 than broad focus words. Figure 1 presents the results from this group.

### Cantonese autistic group

**Fig. 2:**
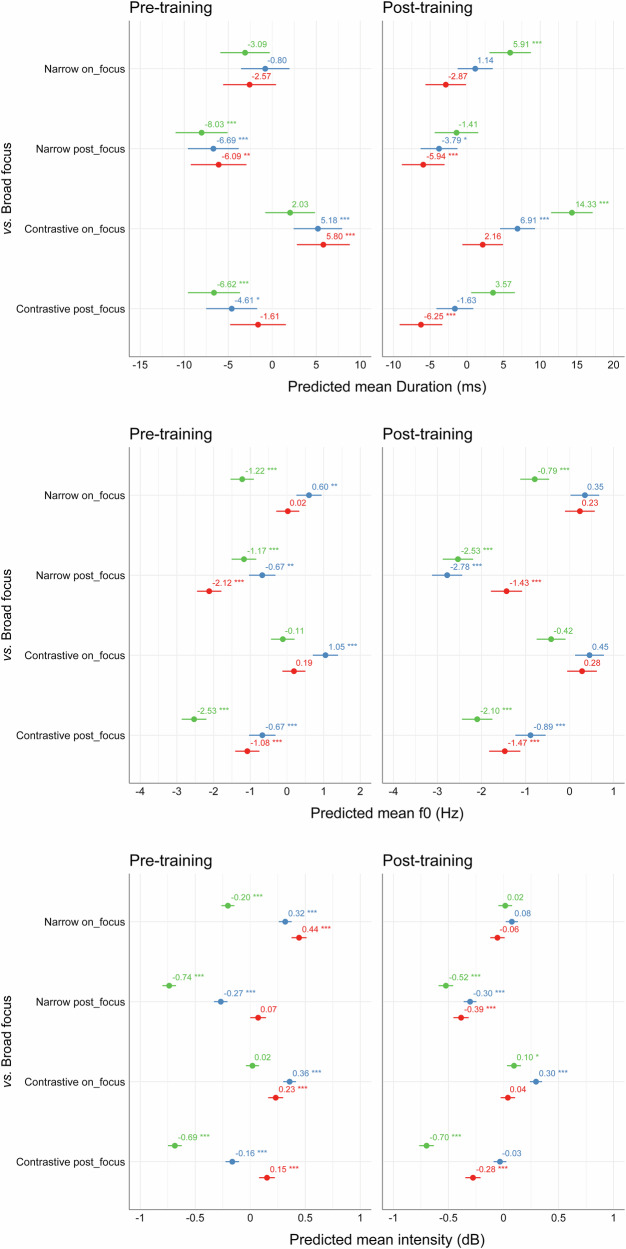
Focus marking before and after training. Differences in predicted mean duration, f0 and intensity between broad focus, narrow and contrastive on- and post-focus syllables. Green dots represent the lab perceptual training group. Blue dots represent the robot-assisted training group. Red dots represent the control group.

The training effects were examined by comparing the strategies used in focus marking before and after training. For two training groups and the autistic control group, LMM results showed that focus condition was significant for mean duration, f0 and intensity in both pre- and post-training production (Table 1). The strategy used before and after training was further examined based on the results from post-hoc comparisons reported below. The significant value was indicated in Fig. 2.

**Table 1:** LMM statistics of the main effect of focus condition on duration, f0 and intensity across three autistic groups

Duration	Pre-training			Post-training		
	χ^2^	df	*p*	χ^2^	df	*p*
Robot-assisted Training SSI	137.25	6	<0.001	116.93	6	<0.001
Lab perceptual Training	107.24	6	<0.001	200.08	6	<0.001
Control Group	116.19	6	<0.001	80.46	6	<0.001

F0	Pre-training			Post-training		
	χ^2^	df	*p*	χ^2^	df	*p*
Robot-assisted Training SSI	817.68	6	<0.001	713.47	6	<0.001
Lab perceptual Training	861.48	6	<0.001	867.72	6	<0.001
Control Group	951.92	6	<0.001	390.04	6	<0.001

Intensity	Pre-training			Post-training		
	χ^2^	df	*p*	χ^2^	df	*p*
Robot-assisted Training SSI	650.32	6	<0.001	456.64	6	<0.001
Lab perceptual Training	1006.1	6	<0.001	896.01	6	<0.001
Control Group	335.36	6	<0.001	209.4	6	<0.001

For the mean duration, a post-hoc comparison revealed some differences in speech production patterns before and after training across different training groups. For the lab perceptual training group, participants produced both narrow and contrastive on-focus words with significantly longer duration than broad focus words in post-training sessions, while this pattern is absent in pre-training sessions.

For the robot-assisted SSI training group, participants produced contrastive on-focus words with longer duration than broad focus words in both pre- and post-training sessions, but the difference is more statistically significant in the post-training session, while this pattern of on-focus expansion was not observed under neither narrow conditions in pre-training sessions. In contrast, no durational expansion was observed for narrow on-focus words in robot-assisted SSI training group. For post-focus words, both the lab perceptual and robot-assisted training groups had a similar pattern, namely, participants produced post-focus words with significantly shorter durations compared to broad focus words in pre-training sessions, but not in post-training sessions.

For the control group, they produced contrastive on-focus words with significantly longer duration than those of broad focus words in the first speech production experiment, but not in the second speech production experiment. Meanwhile, they produced post-focus words with shorter duration than those of broad focus words in both experiments. These patterns suggest that both types of training led to changes in how participants use duration to mark focus in speech, with notable differences observed between the groups and across training conditions.

As for mean f0, in the pre-training session, the lab perceptual training group produced on-focus words with a lower mean f0 compared to broad focus words. Although the post-training production showed a similar pattern, the f0 differences between on-focus and broad focus words decreased. Additionally, this group consistently produced post-focus words with a significantly lower mean f0 than broad focus words in both pre- and post-training speech production.

Conversely, the robot-assisted training group initially produced on-focus words with a significantly higher mean f0 than broad focus words. However, this difference was not observed in the post-training session. Interestingly, post-focus words have significantly lower f0 than broad focus words in both pre- and post-training sessions, but the difference in f0 between post-focus and broad focus words is larger in post-sessions. For the control group, they had similar patterns for on-focus and post-focus words in the first and second production sessions. Namely, narrow and contrastive post-focus words had significantly lower f0 than broad focus words, and there was no significant difference in f0 between narrow and contrastive on-focus words and broad focus words.

As for mean intensity, for the lab perceptual training group, the narrow and contrastive on-focus words had higher mean intensity than broad focus words with a significant difference between contrastive on-focus and broad focus words in post-training sessions, while this pattern is not observed in pre-training sessions. The robot-assisted training group produced both narrow and contrastive on-focus words with significantly higher mean intensity than broad focus words in pre-training production; while in post-training, they only produced contrastive on-focus words with significantly higher mean intensity. The lab perceptual training group produced post-focus words with significantly lower intensity than broad focus words in both pre- and post-training productions. The same pattern was not observed in the robot-assisted group. For the control group, both narrow and contrastive on-focus words had significantly higher mean intensity than that of the broad focus words in the first production session, but not in the second production. Meanwhile, they produced post-focus words with higher mean intensity than that of broad focus words in first production session, but lower in the second production with a significant difference under contrastive condition.

Overall, these results suggest that there is no significant improvement in on-focus expansion for the control group in using acoustic cues such as f0, duration and intensity. Meanwhile, the robot speech training may improve significantly in f0 in the post-focus position, on-focus expansion in using the acoustic cue of duration and it leads to some improvement in the use of intensity. The training effect of lab perceptual training is more significant on the use of duration and intensity for on-focus words.

## Discussion

Our results showed that both lab perceptual training and robot-assisted training with simulated social interaction led to improvement in using prosodic cues in focus marking by autistic children. Specifically, lab perceptual training helped children in producing more prominent on-focus words by significantly increasing the duration and intensity and there was also a trend in increasing mean f0. Robot-assisted training with simulated social interaction improved significantly in children’s use of duration for on-focus expansion, but for other cues intensity and mean f0, on-focus words did not show significant improvement in terms of on-focus expansion, but the post-focus words started to show post-focus compression. The control group did not demonstrate significant changes.

Many studies demonstrate that lab perceptual training significantly improves speech production in both typical adults and children^59^. In contrast, speech production training may not necessarily provide extra help in improving speech production^60^. Similar to the positive effects of lab perceptual training on typical adults^40,41,61^ and children^59^, our positive results on the effects of lab perceptual training indicate that it may modify perceptual mechanisms in autistic children and improve their speech production. Specifically, it may help the implicit learning of the mapping between acoustic cues and information structure by autistic children. In turn, their use of prosodic cues in focus marking in speech production has also significantly improved. Lab perceptual training can be a potentially effective intervention method in improving the encoding of other communicative functions using speech prosody for autistic children.

Moreover, social interaction is known as a key factor in language acquisition. However, autistic children have difficulties in processing and integrating multisensory information such as facial expressions and gestures during social interaction^62,63^. They feel frustrated interacting with people since people’s behavior are not entirely predictable and less consistent compared to robots^1,42^. Human-robot interaction provides a controlled and consistent social interaction across participants, which provide a great chance to test the training effects of simulated social interactions with a robot. Similar to the results from earlier studies that human-robot interaction was proved to be useful in improving social skills^55^, our results confirmed that acquisition of speech prosody may be facilitated by interacting with the social robot. Robots may motivate them in their acquisition of speech prosody as autistic children are more interested in interacting with robots compared to humans^45^, and interacting with robots provides them with a chance in practicing the mapping between acoustic cues and information structure in targeted, discrete and structured tasks, which may be easier than real-life situations. As more personalized training is being designed for this population^64^, it is also possible to personalize human-robot interaction to fit the abilities of each individual in the future. More social cues may be incorporated for those who have better abilities in integrating these cues than others.

It is interesting to note that for the ASD group undergoing robot-assisted training, the on-focus expansion including longer duration and higher mean intensity were observed for the on-focus words in the contrastive focus, but not the narrow focus. Contrastive focus might be functionally stronger than narrow focus because it involves highlighting a specific item in opposition to another. For children with ASD, the contrastive focus condition might have provided a clearer social goal during their interaction with the robot. This suggests that the simulated social setting of the robot might have helped children identify the need for emphasis more effectively when there was a clear contrast to be made, whereas narrow focus signaling new information may remain harder to distinguish from broad focus after the training.

Lab perceptual training does not include a social component, but mainly focuses on exposure of conversations with matched and mismatched prosody to train the mapping between prosodic cues and information structure. In discriminating pairs of conversations, it might be easier for children to master changes in duration and intensity cues, especially for on-focus positions because during the discrimination process, it is relatively easy to generalize that the on-focus words are longer and louder. However, the cue f0 is harder to acquire in this type of training because it can signal both lexical meaning and information structure, and participants need to pay more attention to the conversation flow to capture the changes in the f0 contour in the entire sentence. In contrast, during the robot-assisted training, participants may be able to better capture not only the changes in acoustic cues in the on-focus position, but also the changes during the entire sentence. Therefore, participants tend to show post-focus compression for mean f0 after this type of training.

In sum, the study found two effective ways in improving the use of speech prosody to mark focus by autistic children, which can potentially tackle the problem that autistic children have difficulties in social interaction, which may affect their language acquisition. The two methods lead to improvement of speech prosody in slightly different aspects. One limitation of the current study is that the sentences are limited to the structure of five syllables containing the subject, verb and object. For future studies, various sentence types can be further tested to understand better about the generalizability of the training effects on different sentence types. The proposed methods might be useful in improving skills that involve integration from multiple level of languages such as speech acts (e.g., complaint, suspect and celebration) distinguishable by prosodic cues. The simulated social interaction may also be used to improve learning of multimodal cues such as facial expressions and gestures in addition to speech. With a design to increase the complexity and the amount of cues, autistic children might learn to be better integrate visual and audio information or various cues exhibited at the same time.

## Methods

### Study design

Written consent was obtained from all participants’ parents for their participation and they were compensated in compliance with a protocol approved by the Human Subjects Ethics Sub-Committee (IRB number: HSEARS20210920005) at the Hong Kong Polytechnic University. We recruited 92 children in total, with 69 participants formally diagnosed with ASD. Twenty-three participants were typically developing children (CTD) without any suspected or diagnosed language or speech disorder. For the 60 autistic participants, 20 were randomly assigned to either the lab perceptual training (CASD LPT), robot-assisted speech training with simulated social interaction (CASD SSI) or the control (CASD control) groups, respectively. No training was provided for the CASD and CTD groups.

A questionnaire regarding children’s information, such as gender, age and language background, was filled out by the parents. Further, all participants were assessed using the Hong Kong Cantonese Oral Language Assessment Scale^65^ for the narrative and vocabulary test and Raven’s Matrices and Progressive Matrices^66^ for IQ evaluation. Table 2 presents the statistics of participants’ information.

**Table 2:** Statistics of participants’ gender distribution, age and test scores

Group	CASD PT	CASD RS	CASD Control	CTD
Gender	2F21M	2F21M	2F21M	2F21M
Chronological Age	8.76 ± 1.52	9.10 ± 1.44	9.01 ± 1.57	8.55 ± 1.17
English Acquisition (Age)	3.01 ± 1.40	3.72 ± 1.97	3.66 ± 1.44	2.74 ± 0.87
Mandarin Acquisition (Age)	4.02 ± 1.83	3.80 ± 1.96	4.46 ± 1.61	3.35 ± 1.01
Raven Test	108.04 ± 16.64	109.43 ± 19.96	108.00 ± 16.03	115.78 ± 12.24
HKCOLAS (Narrative Test)	87.09 ± 29.64	76.34 ± 23.86	89.87 ± 25.11	99.56 ± 19.56
HKCOLAS (Vocabulary Test)	65.26 ± 20.11	60.96 ± 20.63	61.52 ± 21.08	67.56 ± 18.93

The overall objective of this study is to examine whether lab perceptual training and robot-assisted training with simulated social interaction lead to significant improvement in the use of speech prosody by autistic children.

### Pre-training speech production tasks

Fifteen target sentences and prompt questions were designed to elicit the desired types of focus, including broad, narrow and contrastive focus on subject, verb and object. Each sentence describes an on-going action with the corresponding picture depicting the content of the target sentence. The participants familiarized themselves with all the pictures and target sentences in the practice session.

**Fig. 3:**
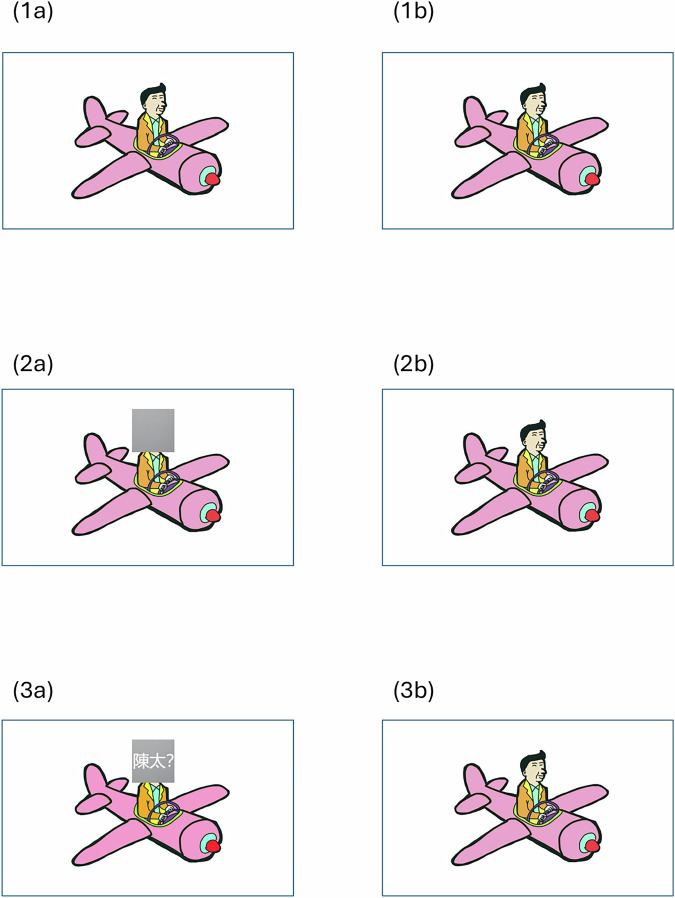
Production test elicitation. An example eliciting broad, narrow and contrastive focus in the production test without the gray area (**a**); An example eliciting narrow focus with the gray area (**b**); An example eliciting contrastive focus with the gray area (**c**).

During the experimental sessions, the participants were instructed to answer questions using the target sentences and corresponding pictures. If the precursor question elicited a broad focus condition where the experimenter may ask “What is happening?” (Fig. 3a), participants were required to answer “[Mr. Cheung is flying the plane]_broad focus_”. If the picture covered with a gray area (Fig. 3b) was first shown to participants with a question “Who is flying the plane?”, a picture removing the gray area was then presented and participants were expected to answer “[Mr. Cheung]_narrow focus_ is flying the plane” with a narrow focus on the subject. If the first picture covered with a gray area and text with incorrect subject (Fig. 3c) was shown to participants with a question “Ms. Chan is flying the plane?”, a picture removing the gray area and text was then presented and participants were expected to answer “[Mr. Cheung]_contrastive focus_ is flying the plane” with a contrastive focus on the subject.

Each participant produced 210 stimuli (15 target sentences * 7 focus types (broad focus, narrow focus on the subject, verb and object, contrastive focus on the subject, verb and object)* 2 repetitions). The focus marking production test was recorded once for the CTD group, while the autistic groups were recorded both before and after the training.

**Fig. 4:**
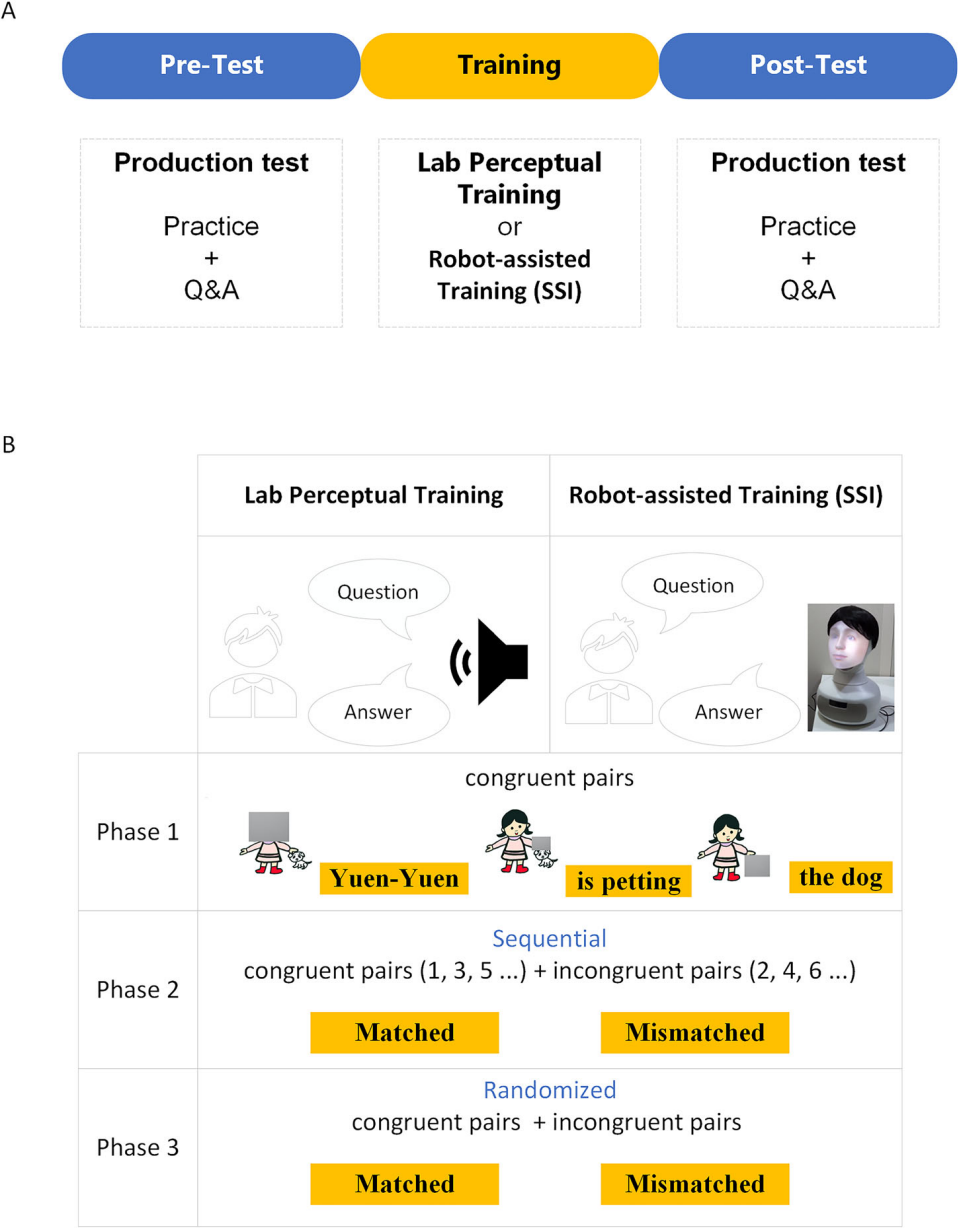
Experimental procedure. An example of the main experimental procedure (**A**) and details of pre- and post-test and the training (**B**).

The speech production task and two types of training were conducted in a soundproof booth at the speech lab at a University. Audio Technica AT2035 condenser microphone and Steinberg UR22mkII USB Audio Interface were used to record participants’ speech in the two production tasks with the sample rate of 44100 Hz in Audacity^67^. The entire procedure is presented in Fig. 4.

### Lab perceptual training (LPT)

Six sentences with subject, verb and object in various focus conditions were used for lab perceptual training. The lab perceptual training has three training phases presented in the software Eprime 2.0^68^. In these training phases, stimuli with matched and mismatched question-answer pairs were used. Specifically, in a congruent pair, a precursor question elicited a focus type and focus position that matched with those in the answer. In an incongruent pair, they were not matched. An example is shown below.

Congruent pair:

Question: Who is petting the dog?

Answer: [Yuen-Yuen]_narrow focus_ is petting the dog.

Incongruent pair:

Question: Who is petting the dog?

Answer: [Yuen-Yuen is petting the dog.]_broad focus_

In training phase one, only audios of congruent pairs were played during the first training period. A webpage featuring pictures and buttons of six selected sentences was made. From left to right, three pictures of each sentence were displayed, where the illustrations of the subject, the verb, and the object were covered with gray area, respectively. Three buttons with the text of the subject (“Yuen-Yuen”), the verb (“is petting”), and the object (“the dog”) were placed next to the pictures. After hearing the precursor questions, participants were told to identify the focus position (subject, verb or object) and focus type (broad, narrow or contrastive) by clicking on corresponding buttons while hearing the answers. If the question elicited a broad focus condition, the participant did not need to click any button while the answer was being played. If the question was “Who is petting the dog?” eliciting a narrow focus, the participant was required to *single click* the button “Yuen-Yuen” while listening to the answer “[Yuen-Yuen]_narrow focus_ is petting the dog”. If the question was “Big brother is petting the dog?” eliciting a contrastive focus, the participant was required to *double click* the button “Yuen-Yuen” while listening to the answer “[Yuen-Yuen]_contrastive focus_ is petting the dog”.

In training phase two, both congruent and incongruent pairs were used and were assigned to odd and even number trials, respectively. Participants were informed about the trial arrangement before this phase started. After listening to the audio of each pair, the participants need to press the key Y or N standing for “Yes, they are congruent” or “No, they are not congruent” to reinforce the matchedness.

In training phase three, the congruent and incongruent pairs were presented in a randomized trial order. After listening to the audio of each pair, participants needed to make a judgment whether the pairs are congruent or incongruent by pressing the key Y or N and feedback was provided on the screen.

There were two training sessions in total, and the phases of these two sessions were identical. Materials of six sentences were assigned to separate training sessions with three sentences for each session. Depending on the participants, it took 1–1.5 h to complete each session.

### Robot speech training

**Fig. 5:**
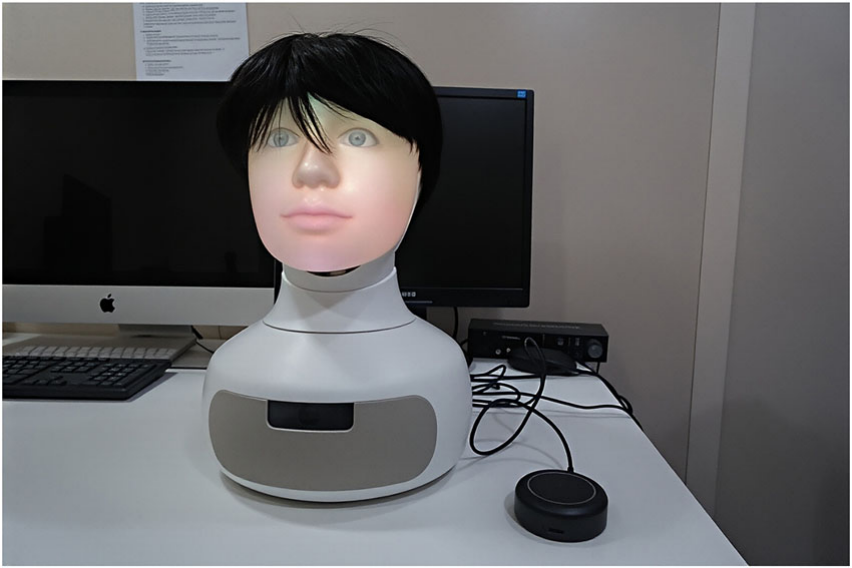
Social robot. A pic of the social robot Furhat used in the current study.

The social robot Furhat was used for the SSI training sessions, as shown in Fig. 5. This Furhat robot can be programmed to interact with autistic children and provide effective training. Furhat uses cloud-based speech recognition services powered by both Google ASR (automatic speech recognition) and Microsoft Azure Cognitive Services ASR. Its speech synthesis technology is powered by both Amazon Polly TTS and Acapela TTS (text-to-speech). The Furhat robot utilizes a back-projected approach to project its face. The robot will automatically add synchronized lip movements for any texts and audio files of speech. For visual recognition, Furhat has a high-quality built-in camera to detect and track users’ faces. Moreover, Furhat SDK (Software Development kit) for programming Furhat Robot can be used in a virtual environment using Kotlin language.

**Fig. 6:**
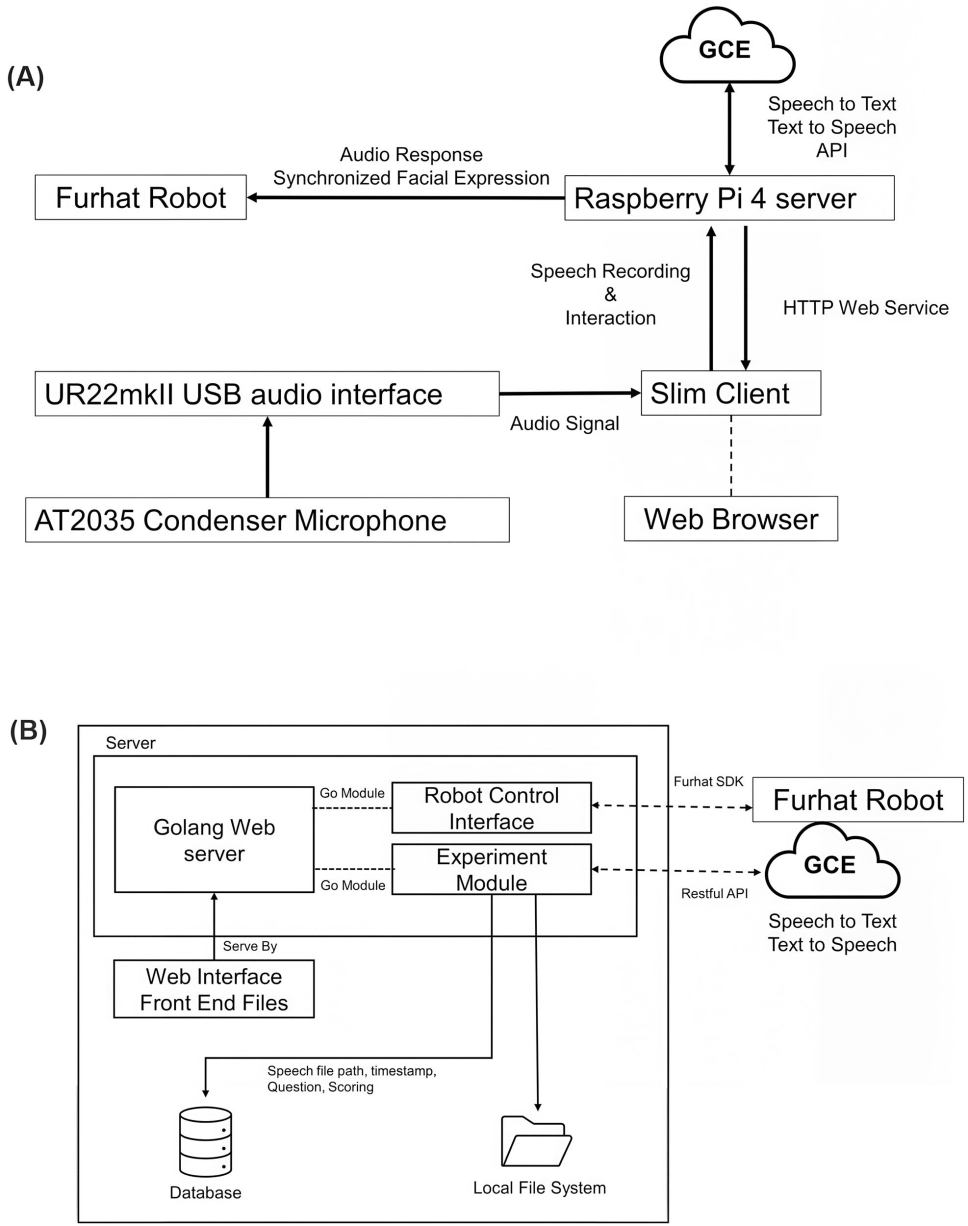
The system architecture. The system components (**A**) and the web server (**B**).

A system and a web-application-based user interface were developed for the training session. The system architecture deployed for this study is shown in Fig. 6. The system was developed to manage the interaction and synchronization of the image display, audio files from a computer unit (slim client, which can be any computing unit featured with an internet browser and a display unit) and facial expressions of the Furhat robot. A web server was installed on a Raspberry Pi 4B single-board computer for building lightweight client-server architecture. The web server was developed using Golang 1.14 and was responsible for the interaction control, data processing and communication with the slim client and the Furhat robot using Furhat SDK. Interaction with the web interface was conducted using a slim client. A web-based graphical user interface (GUI) was coded using React.js. The web-based GUI was designed with two modes, which dedicated to performing interactions with test subjects and instructors respectively.

In the robot-assisted training, the same six sentences with various focus conditions in LPT were used. Similar to LPT, there were three phases of training. Instead of being exposed to pairs of dialogs with no social interaction as in the lab perceptual training, participants were instructed to interact with the robot during the training. Specifically, in training phase one, participants were instructed to ask the robot a question eliciting focus based on a picture either covered with a gray area or not, and the robot provided the congruent answers with matched prosody. Participants needed to identify the focus position and focus type by clicking buttons as in LPT while the robot was answering. In the second phase, participants continued to ask the robot questions, and they were informed that the odd trials of answers provided by the robot had matched focus types and positions with their questions and in the even trials, the robot provided mismatched answers. The participants needed to press the keys Y or N to keep track of the odd and even trials. In the third phase, participants asked the robot questions and the robot provided matched and mismatched answers randomly. Participants needed to make a judgment and feedback was provided by the robot.

Same as LPT, there were two identical training sessions in robot speech training. Materials of six sentences were assigned to separate training sessions and each session took 1 to 1.5 h to complete.

### Segmentation, feature extraction, and statistical analyses

The recorded sentences were manually segmented following the procedure of segmentation^41^ in Praat^69^. Acoustic measurements such as word duration, mean f0 and intensity were extracted using ProsodyPro^70^.

Linear mixed-effects models (LMM) were then fitted with word duration, mean f0, mean intensity and f0 range as the response variables in each model, while groups (i.e., training groups, ASD and TD control groups) and focus conditions (i.e., narrow/contrastive pre-, on- and post-focus) were assigned as explanatory variables. Forward selection procedure was employed for the optimal model and post-hoc comparisons were conducted for significant main effects. The statistical analysis was done using the package lmer4^71^ in R. The LMM analyses were conducted for both pre- and post-training sessions for the autistic training and control groups.

## Materials availability

All data containing the values of extracted acoustic data for all groups are available in the supplementary materials. The raw datasets of recordings generated during the current study are not publicly available due to privacy issues, but are available from the corresponding author on reasonable request.

## Supplementary information

code_merged

## Data Availability

All data containing the values of extracted acoustic data for all groups are available in the supplementary materials. The raw datasets of recordings generated during the current study are not publicly available due to privacy issues, but are available from the corresponding author on reasonable request.

## Code availability

The code is available in the supplementary materials. The programming language R has been used in data analysis.
