# Supplementary material for: Lab perceptual training and robot-assisted training in improving speech prosody of autistic children
Source: NPJ Sci Learn. 2026 May 6;11:44. doi: 10.1038/s41539-026-00425-7 (PMC13350709; doi:10.1038/s41539-026-00425-7)
Supplement: Supplementary file 1 — code_merged [file 41539_2026_425_MOESM1_ESM.pdf]

Code

1 Plots
1.1 CTD
1.2 CASD

CASD Training By Focus Condition (science robotics)

Combine newly added speakers

Hide

```
#remove syllableposition col, newly added data doesn't have this col, and it's not going to be used anyway.
CASD.HS.dur <- dplyr::select(CASD.HS.dur, -SyllablePosition)
CASD.HS.dur <- rbind(CASD.HS.dur, CASD.HS.dur.add)

CASD.HS.f0Int<- dplyr::select(CASD.HS.f0Int,  -SyllablePoint, -SentencePoint)
CASD.HS.f0Int<- rbind(CASD.HS.f0Int,CASD.HS.f0Int.add)

CASD.RS.dur.add <- dplyr::select(CASD.RS.dur.add, -X)
CASD.RS.dur <- rbind(CASD.RS.dur, CASD.RS.dur.add)

CASD.RS.f0Int.add <- dplyr::select(CASD.RS.f0Int.add, -X, -Group)
CASD.RS.f0Int <- rbind(CASD.RS.f0Int, CASD.RS.f0Int.add)

CASD.Ctrl.dur <- dplyr::select(CASD.Ctrl.dur, -maxf0, -minf0)
CASD.Ctrl.dur <- rbind(CASD.Ctrl.dur, CASD.Ctrl.dur.add)

CASD.Ctrl.f0Int.add <- dplyr::select(CASD.Ctrl.f0Int.add, -X)
CASD.Ctrl.f0Int <- rbind(CASD.Ctrl.f0Int, CASD.Ctrl.f0Int.add)

CTD.dur <- dplyr::select(CTD.dur, -SyllablePosition)
CTD.dur <- rbind(CTD.dur, CTD.dur.add)


CTD.f0Int <- dplyr::select(CTD.f0Int, -SyllablePoint, -SentencePoint)
CTD.f0Int <-rbind(CTD.f0Int, CTD.f0Int.add)
```

Remove unwanted speakers

Hide

```
#CASD HS: delete ASD9, 2020108
unWantedspks <- c("ASD9", "2020108")
[illegible]
```

```
CASD.HS.dur <- filter(CASD.HS.dur, !(Subject %in% unWan
tedspks))
CASD.HS.f0Int <- filter(CASD.HS.f0Int, !(Subject %in% u
nWantedspks))

#CASD RS: delete 2023310, 2023312
unWantedspks <- c("2023310", "2023312")
CASD.RS.dur <- filter(CASD.RS.dur, !(Subject %in% unWan
tedspks))
CASD.RS.f0Int <- filter(CASD.RS.f0Int, !(Subject %in% u
nWantedspks))

#CASD Ctrl: delete 2020211, 2023412
unWantedspks <- c("2020211", "2023412")
CASD.Ctrl.dur <- filter(CASD.Ctrl.dur, !(Subject %in% u
nWantedspks))
CASD.Ctrl.f0Int <- filter(CASD.Ctrl.f0Int, !(Subject %i
n% unWantedspks))

#TD Ctrl: delete "2020301", "2020304", "2020305", "2020
308", "2020315", "2020317", "2020318"
unWantedspks <- c("2020301", "2020304", "2020305", "202
0308", "2020315", "2020317", "2020318")
CTD.dur <- filter(CTD.dur, !(Subject %in% unWantedspks)
)
CTD.f0Int <- filter(CTD.f0Int, !(Subject %in% unWanteds
pks))
```

Check number of participants

Hide

```
length(unique(CASD.HS.dur$Subject))
```

```
## [1] 23
```

Hide

```
length(unique(CASD.HS.f0Int$Subject))
```

```
## [1] 23
```

Hide

```
length(unique(CASD.RS.dur$Subject))
```

```
## [1] 23
```

Hide

```
length(unique(CASD.RS.f0Int$Subject))
```

```
## [1] 23
```

Hide

```
length(unique(CASD.Ctrl.dur$Subject))
```

```
## [1] 23
```

Hide

```
length(unique(CASD.Ctrl.f0Int$Subject))
```

```
## [1] 23
```

Hide

```
length(unique(CTD.dur$Subject))
```

```
## [1] 23
```

Hide

```
length(unique(CTD.f0Int$Subject))
```

```
## [1] 23
```

Hide

```
#load MAD function

#' @title Identify outliers using robust median absolut
e deviation approach
#' @name outliersMAD
#'
#' @description outliersMAD is used to identify outlier
s in vectors using Leys et al.'s (2003) median absolute
deviation approach.
#'
#' @param x a vector of numbers
#' @param MADCutOff value to use as cutoff (Leys e tal.
recommend 2.5 or 3.0 as default)
#' @param replaceOutliersWith if value is an outlier, w
hat to replace it with? NA by default
#' @param showMADValues if TRUE, will show deviation sc
ore of each value
#' @param outlierIndices return index/position of outli
er
#' @param bConstant a constant linked to the assumption
of normality of the data, disregarding the abnormality
induced by outliers
```

```
induced by outliers
#' @param digits how many digits/decimals to round outp
ut to
#'
#' @return A vector with outliers identified (default c
onverts outliers to NA)
#'
#' @details We can identify and remove outliers in our
data by identifying data points that are too extreme—ei
ther too many standard deviations (SD) away from the me
an or too many median absolute deviations (MAD) away fr
om the median. The SD approach might not be ideal with
extreme outliers, whereas the MAD approach is much more
robust (for comparison of both approaches, see Leys et
al., 2013, Journal of Experimental Social Psychology).
#'
#' @references \itemize{
#' \item Leys, C., Ley, C., Klein, O., Bernard, P., & L
icata, L. (2013). Detecting outliers: Do not use standa
rd deviation around the mean, use absolute deviation ar
ound the median. Journal of Experimental Social Psychol
ogy, 49(4), 764-766. doi:10.1016/j.jesp.2013.03.013 (\u
rl{https://www.sciencedirect.com/science/article/pii/S0
022103113000668})}
#' @seealso \code{\link{outliersZ}}
#'
#' @author Hause Lin
#'
#' @export
#'
#' @usage
#' outliersMAD(x, MADCutOff = 3.0, replaceOutliersWith
= NA,
#' showMADValues = FALSE, outlierIndices = FALSE, bCons
tant = 1.4826, digits = 2)
#'
#' @examples
#' example <- c(1, 3, 3, 6, 8, 10, 10, 1000, -1000) # 1
000 is an outlier
#' outliersMAD(example)
#' outliersMAD(example, MADCutOff = 3.0)
#' outliersMAD(example, MADCutOff = 2.5, replaceOutlier
sWith = -999)
#' outliersMAD(example, MADCutOff = 1.5, outlierIndices
= TRUE)
#' outliersMAD(example, MADCutOff = 1.5, showMADValues
= TRUE)
#' outliersMAD(example, MADCutOff = 1.5, showMADValues
= TRUE, replaceOutliersWith = -88)
outliersMAD <- function(x, MADCutOff = 3.0, replaceOutl
iersWith = NA, showMADValues = FALSE, outlierIndices =
FALSE, bConstant = 1.4826, digits = 2) {
  # bConstant: usually, b = 1.4826, a constant linked t
o the assumption of normality of the data, disregarding
the abnormality induced by out- liers (Rousseeuw & Crou
```

```
x, 1993).

  # compute number of absolute MADs away for each value
: formula: abs( ( x - median(x) ) )/ mad(x)
  MADAway <- (x - stats::median(x, na.rm = T)) / stats:
:mad(x, constant = bConstant, na.rm = T)
  absMADAway <- abs(MADAway)
  # subset data that has absMADAway greater than the MA
DCutOff and replace them with replace
  x[absMADAway > MADCutOff] <- replaceOutliersWith
  outliers <- length(x[absMADAway > MADCutOff])
  if (showMADValues) { # if values == TRUE, return numb
er of mads for each value
    message("Showing MAD from median for each value.")
    message(paste0(outliers, " outliers detected."))
    return(round(MADAway, digits))
  } else if (outlierIndices) {
    message("Showing indices of outliers.")
    if (is.na(replaceOutliersWith)) {
      return(which(is.na(x)))
    } else {
      return(x[x == replaceOutliersWith])
    }

  } else {
    message(paste0(outliers, " outliers detected."))
    message(paste0("Outliers replaced with ", replaceOu
tliersWith))
    return(round(x, digits)) # otherwise, return origin
al with outliers replaced
  }
}
```

#LMM

0.1 HumanSpeech by focus condition

0.1.1 mean duration

Hide

```
df <- filter(bob.mad, TrainingOrder == "Pre")

m1 <- lmer(Duration ~ 1  + (1|word) + (1|Subject) + (1|
word_type) + (1|Block), data = df, REML = FALSE)
```

```
## boundary (singular) fit: see help('isSingular')
```

Hide

```
casd.hs.dur.pre <- lmer(Duration ~  focus_condition + (
1|word) + (1|Subject)+ (1|word_type) + (1|Block), data
= df, REML = FALSE)
```

```
## boundary (singular) fit: see help('isSingular')
```

Hide

```
anova(m1, casd.hs.dur.pre)
```

```
## Data: df
## Models:
## m1: Duration ~ 1 + (1 | word) + (1 | Subject) + (1 |
word_type) + (1 | Block)
## casd.hs.dur.pre: Duration ~ focus_condition + (1 | w
ord) + (1 | Subject) + (1 | word_type) + (1 | Block)
##                 npar    AIC    BIC  logLik deviance
Chisq Df Pr(>Chisq)
## m1                 6 255789 255837 -127889   255777
## casd.hs.dur.pre   12 255694 255791 -127835   255670
107.24  6  < 2.2e-16 ***
## ---
## Signif. codes:  0 '***' 0.001 '**' 0.01 '*' 0.05 '.'
0.1 ' ' 1
```

Hide

```
emmeans(casd.hs.dur.pre, pairwise~focus_condition)
```

```
## Note: D.f. calculations have been disabled because t
he number of observations exceeds 3000.
## To enable adjustments, add the argument 'pbkrtest.li
mit = 23255' (or larger)
## [or, globally, 'set emm_options(pbkrtest.limit = 232
55)' or larger];
## but be warned that this may result in large computat
ion time and memory use.
```

```
## Note: D.f. calculations have been disabled because t
he number of observations exceeds 3000.
## To enable adjustments, add the argument 'lmerTest.li
mit = 23255' (or larger)
## [or, globally, 'set emm_options(lmerTest.limit = 232
55)' or larger];
## but be warned that this may result in large computat
ion time and memory use.
```

```
## $emmeans
##  focus_condition        emmean   SE  df asymp.LCL as
ymp.UCL
##  Broad focus               208 10.3 Inf       188
229
##  Narrow pre_focus          200 10.4 Inf       180
221
##  Narrow on_focus           205 10.3 Inf       185
226
##  Narrow post_focus         200 10.4 Inf       180
221
##  Contrastive pre_focus     210 10.4 Inf       190
230
##  Contrastive on_focus      210 10.3 Inf       190
231
##  Contrastive post_focus    202 10.4 Inf       181
222
##
## Degrees-of-freedom method: asymptotic
## Confidence level used: 0.95
##
## $contrasts
##  contrast                                       esti
mate   SE  df z.ratio
##  Broad focus - Narrow pre_focus                    7
.946 1.50 Inf   5.286
##  Broad focus - Narrow on_focus                     3
.091 1.43 Inf   2.160
##  Broad focus - Narrow post_focus                   8
.028 1.51 Inf   5.327
##  Broad focus - Contrastive pre_focus              -1
.686 1.51 Inf  -1.117
##  Broad focus - Contrastive on_focus               -2
.027 1.44 Inf  -1.410
##  Broad focus - Contrastive post_focus              6
.622 1.51 Inf   4.382
##  Narrow pre_focus - Narrow on_focus               -4
.855 1.50 Inf  -3.239
##  Narrow pre_focus - Narrow post_focus              0
.082 1.70 Inf   0.048
##  Narrow pre_focus - Contrastive pre_focus         -9
.632 1.43 Inf  -6.714
##  Narrow pre_focus - Contrastive on_focus          -9
.973 1.51 Inf  -6.623
##  Narrow pre_focus - Contrastive post_focus        -1
.324 1.71 Inf  -0.775
##  Narrow on_focus - Narrow post_focus               4
.937 1.51 Inf   3.279
##  Narrow on_focus - Contrastive pre_focus          -4
.777 1.50 Inf  -3.175
##  Narrow on_focus - Contrastive on_focus           -5
.119 1.44 Inf  -3.567
##  Narrow on_focus - Contrastive post_focus          3
.531 1.51 Inf   2.339
```

```
[illegible]
##  Narrow post_focus - Contrastive pre_focus        -9
.714 1.71 Inf  -5.684
##  Narrow post_focus - Contrastive on_focus        -10
.055 1.51 Inf  -6.650
##  Narrow post_focus - Contrastive post_focus       -1
.406 1.44 Inf  -0.979
##  Contrastive pre_focus - Contrastive on_focus     -0
.341 1.51 Inf  -0.226
##  Contrastive pre_focus - Contrastive post_focus    8
.308 1.71 Inf   4.852
##  Contrastive on_focus - Contrastive post_focus     8
.649 1.52 Inf   5.706
##  p.value
##   <.0001
##   0.3175
##   <.0001
##   0.9230
##   0.7967
##   0.0002
##   0.0205
##   1.0000
##   <.0001
##   <.0001
##   0.9873
##   0.0180
##   0.0252
##   0.0066
##   0.2255
##   <.0001
##   <.0001
##   0.9586
##   1.0000
##   <.0001
##   <.0001
##
## Degrees-of-freedom method: asymptotic
## P value adjustment: tukey method for comparing a fam
ily of 7 estimates
```

Hide

```
df <- filter(bob.mad, TrainingOrder == "Post")

m1 <- lmer(Duration ~ 1  + (1|word) + (1|Subject) + (1|
word_type) + (1|Block), data = df, REML = FALSE)
casd.hs.dur.post <- lmer(Duration ~  focus_condition +
(1|word) + (1|Subject)+ (1|word_type) + (1|Block), data
= df, REML = FALSE)

anova(m1, casd.hs.dur.post)
```

```
## Data: df
## Models:
```

```
## m1: Duration ~ 1 + (1 | word) + (1 | Subject) + (1 |
word_type) + (1 | Block)
## casd.hs.dur.post: Duration ~ focus_condition + (1 |
word) + (1 | Subject) + (1 | word_type) + (1 | Block)
##                    npar    AIC    BIC  logLik deviance
Chisq Df Pr(>Chisq)
## m1                   6 244009 244057 -121998   243997
## casd.hs.dur.post   12 243821 243917 -121898   243797
200.08  6  < 2.2e-16 ***
## ---
## Signif. codes:  0 '***' 0.001 '**' 0.01 '*' 0.05 '.'
0.1 ' ' 1
```

Hide

```
emmeans(casd.hs.dur.post, pairwise~focus_condition)
```

```
## Note: D.f. calculations have been disabled because t
he number of observations exceeds 3000.
## To enable adjustments, add the argument 'pbkrtest.li
mit = 22258' (or larger)
## [or, globally, 'set emm_options(pbkrtest.limit = 222
58)' or larger];
## but be warned that this may result in large computat
ion time and memory use.
```

```
## Note: D.f. calculations have been disabled because t
he number of observations exceeds 3000.
## To enable adjustments, add the argument 'lmerTest.li
mit = 22258' (or larger)
## [or, globally, 'set emm_options(lmerTest.limit = 222
58)' or larger];
## but be warned that this may result in large computat
ion time and memory use.
```

```
## $emmeans
##  focus_condition        emmean   SE  df asymp.LCL as
ymp.UCL
##  Broad focus               181 12.7 Inf       156
205
##  Narrow pre_focus          177 12.7 Inf       152
202
##  Narrow on_focus           187 12.7 Inf       162
211
```

```
##  Narrow post_focus         179 12.7 Inf       154
204
##  Contrastive pre_focus     182 12.7 Inf       157
207
##  Contrastive on_focus      195 12.7 Inf       170
220
##  Contrastive post_focus    184 12.7 Inf       159
209
##
## Degrees-of-freedom method: asymptotic
## Confidence level used: 0.95
##
## $contrasts
##  contrast                                        esti
mate   SE  df z.ratio
##  Broad focus - Narrow pre_focus
3.40 1.50 Inf   2.261
##  Broad focus - Narrow on_focus                      -
5.91 1.44 Inf  -4.109
##  Broad focus - Narrow post_focus
1.41 1.51 Inf   0.930
##  Broad focus - Contrastive pre_focus                -
1.12 1.51 Inf  -0.745
##  Broad focus - Contrastive on_focus                -1
4.33 1.44 Inf  -9.955
##  Broad focus - Contrastive post_focus               -
3.57 1.52 Inf  -2.358
##  Narrow pre_focus - Narrow on_focus                 -
9.31 1.50 Inf  -6.191
##  Narrow pre_focus - Narrow post_focus               -
1.99 1.71 Inf  -1.166
##  Narrow pre_focus - Contrastive pre_focus           -
4.52 1.43 Inf  -3.162
##  Narrow pre_focus - Contrastive on_focus           -1
7.72 1.51 Inf -11.776
##  Narrow pre_focus - Contrastive post_focus          -
6.97 1.71 Inf  -4.078
##  Narrow on_focus - Narrow post_focus
7.32 1.51 Inf   4.829
##  Narrow on_focus - Contrastive pre_focus
4.79 1.51 Inf   3.178
##  Narrow on_focus - Contrastive on_focus             -
8.42 1.44 Inf  -5.848
##  Narrow on_focus - Contrastive post_focus
2.34 1.52 Inf   1.540
##  Narrow post_focus - Contrastive pre_focus          -
2.53 1.71 Inf  -1.479
##  Narrow post_focus - Contrastive on_focus          -1
5.73 1.52 Inf -10.380
##  Narrow post_focus - Contrastive post_focus         -
4.98 1.44 Inf  -3.459
##  Contrastive pre_focus - Contrastive on_focus      -1
3.20 1.51 Inf  -8.758
##  Contrastive pre_focus - Contrastive post_focus     -
```

```
2.45 1.71 Inf  -1.431
##  Contrastive on_focus - Contrastive post_focus     1
0.75 1.52 Inf   7.088
##  p.value
##   0.2632
##   0.0008
##   0.9679
##   0.9897
##   <.0001
##   0.2168
##   <.0001
##   0.9070
##   0.0263
##   <.0001
##   0.0009
##   <.0001
##   0.0250
##   <.0001
##   0.7205
##   0.7576
##   <.0001
##   0.0098
##   <.0001
##   0.7850
##   <.0001
##
## Degrees-of-freedom method: asymptotic
## P value adjustment: tukey method for comparing a fam
ily of 7 estimates
```

0.1.2 mean f0

Hide

```
df <- filter(bob.mad, TrainingOrder == "Pre")

m1 <- lmer(F0 ~ 1  + (1|word) + (1|Subject) + (1|word_t
ype) + (1|Block), data = df, REML = FALSE)
casd.hs.f0.pre <- lmer(F0 ~  focus_condition + (1|word)
+ (1|Subject)+ (1|word_type) + (1|Block), data = df, RE
ML = FALSE)

anova(m1, casd.hs.f0.pre)
```

```
## Data: df
## Models:
## m1: F0 ~ 1 + (1 | word) + (1 | Subject) + (1 | word_
type) + (1 | Block)
## casd.hs.f0.pre: F0 ~ focus_condition + (1 | word) +
(1 | Subject) + (1 | word_type) + (1 | Block)
##                npar     AIC     BIC   logLik devianc
e  Chisq Df Pr(>Chisq)
## m1                6 3869984 3870049 -1934986  386997
```

```
2
## casd.hs.f0.pre   12 3869134 3869265 -1934555  386911
0 861.48  6  < 2.2e-16 ***
## ---
## Signif. codes:  0 '***' 0.001 '**' 0.01 '*' 0.05 '.'
0.1 ' ' 1
```

Hide

```
emmeans(casd.hs.f0.pre, pairwise~focus_condition)
```

```
## Note: D.f. calculations have been disabled because t
he number of observations exceeds 3000.
## To enable adjustments, add the argument 'pbkrtest.li
mit = 407496' (or larger)
## [or, globally, 'set emm_options(pbkrtest.limit = 407
496)' or larger];
## but be warned that this may result in large computat
ion time and memory use.
```

```
## Note: D.f. calculations have been disabled because t
he number of observations exceeds 3000.
## To enable adjustments, add the argument 'lmerTest.li
mit = 407496' (or larger)
## [or, globally, 'set emm_options(lmerTest.limit = 407
496)' or larger];
## but be warned that this may result in large computat
ion time and memory use.
```

```
## $emmeans
##  focus_condition        emmean   SE  df asymp.LCL as
ymp.UCL
##  Broad focus               229 8.95 Inf       212
247
##  Narrow pre_focus          230 8.95 Inf       213
248
##  Narrow on_focus           228 8.95 Inf       211
246
##  Narrow post_focus         228 8.95 Inf       211
246
##  Contrastive pre_focus     232 8.95 Inf       215
250
##  Contrastive on_focus      229 8.95 Inf       212
247
##  Contrastive post_focus    227 8.95 Inf       209
244
##
## Degrees-of-freedom method: asymptotic
```

```
## Degrees of freedom method: asymptotic
## Confidence level used: 0.95
##
## $contrasts
##  contrast                                               esti
mate    SE  df z.ratio
##  Broad focus - Narrow pre_focus                           -1
.033 0.171 Inf  -6.049
##  Broad focus - Narrow on_focus                             1
.219 0.163 Inf   7.501
##  Broad focus - Narrow post_focus                           1
.172 0.171 Inf   6.843
##  Broad focus - Contrastive pre_focus                      -2
.811 0.172 Inf -16.386
##  Broad focus - Contrastive on_focus                        0
.112 0.163 Inf   0.686
##  Broad focus - Contrastive post_focus                      2
.531 0.172 Inf  14.699
##  Narrow pre_focus - Narrow on_focus                        2
.252 0.171 Inf  13.198
##  Narrow pre_focus - Narrow post_focus                      2
.205 0.194 Inf  11.365
##  Narrow pre_focus - Contrastive pre_focus                 -1
.778 0.163 Inf -10.915
##  Narrow pre_focus - Contrastive on_focus                   1
.145 0.172 Inf   6.676
##  Narrow pre_focus - Contrastive post_focus                 3
.564 0.195 Inf  18.292
##  Narrow on_focus - Narrow post_focus                      -0
.047 0.171 Inf  -0.274
##  Narrow on_focus - Contrastive pre_focus                  -4
.031 0.171 Inf -23.514
##  Narrow on_focus - Contrastive on_focus                   -1
.107 0.163 Inf  -6.778
##  Narrow on_focus - Contrastive post_focus                  1
.312 0.172 Inf   7.614
##  Narrow post_focus - Contrastive pre_focus                -3
.984 0.195 Inf -20.454
##  Narrow post_focus - Contrastive on_focus                 -1
.060 0.172 Inf  -6.161
##  Narrow post_focus - Contrastive post_focus                1
.359 0.164 Inf   8.263
##  Contrastive pre_focus - Contrastive on_focus              2
.923 0.172 Inf  16.974
##  Contrastive pre_focus - Contrastive post_focus            5
.342 0.196 Inf  27.324
##  Contrastive on_focus - Contrastive post_focus             2
.419 0.173 Inf  13.989
##  p.value
##   <.0001
##   <.0001
##   <.0001
##   <.0001
##   0.9934
##   <.0001
```

```
##   <.0001
##   <.0001
##   <.0001
##   <.0001
##   <.0001
##   1.0000
##   <.0001
##   <.0001
##   <.0001
##   <.0001
##   <.0001
##   <.0001
##   <.0001
##   <.0001
##   <.0001
##
## Degrees-of-freedom method: asymptotic
## P value adjustment: tukey method for comparing a fam
ily of 7 estimates
```

Hide

```
df <- filter(bob.mad, TrainingOrder == "Post")

m1 <- lmer(F0 ~ 1  + (1|word) + (1|Subject) + (1|word_t
ype) + (1|Block), data = df, REML = FALSE)
casd.hs.f0.post <- lmer(F0 ~  focus_condition + (1|word
) + (1|Subject)+ (1|word_type) + (1|Block), data = df,
REML = FALSE)

anova(m1, casd.hs.f0.post)
```

```
## Data: df
## Models:
## m1: F0 ~ 1 + (1 | word) + (1 | Subject) + (1 | word_
type) + (1 | Block)
## casd.hs.f0.post: F0 ~ focus_condition + (1 | word) +
(1 | Subject) + (1 | word_type) + (1 | Block)
##                  npar     AIC     BIC   logLik devian
ce  Chisq Df Pr(>Chisq)
## m1                  6 3687007 3687073 -1843498  36869
95
## casd.hs.f0.post   12 3686152 3686282 -1843064  36861
28 867.72  6  < 2.2e-16 ***
## ---
## Signif. codes:  0 '***' 0.001 '**' 0.01 '*' 0.05 '.'
0.1 ' ' 1
```

Hide

```
emmeans(casd.hs.f0.post, pairwise~focus_condition)
```

```
## Note: D.f. calculations have been disabled because t
he number of observations exceeds 3000.
## To enable adjustments, add the argument 'pbkrtest.li
mit = 388286' (or larger)
## [or, globally, 'set emm_options(pbkrtest.limit = 388
286)' or larger];
## but be warned that this may result in large computat
ion time and memory use.
```

```
## Note: D.f. calculations have been disabled because t
he number of observations exceeds 3000.
## To enable adjustments, add the argument 'lmerTest.li
mit = 388286' (or larger)
## [or, globally, 'set emm_options(lmerTest.limit = 388
286)' or larger];
## but be warned that this may result in large computat
ion time and memory use.
```

```
## $emmeans
##  focus_condition        emmean   SE  df asymp.LCL as
ymp.UCL
##  Broad focus               233 9.55 Inf       214
251
##  Narrow pre_focus          234 9.55 Inf       216
253
##  Narrow on_focus           232 9.55 Inf       213
251
##  Narrow post_focus         230 9.55 Inf       211
249
##  Contrastive pre_focus     235 9.55 Inf       216
254
##  Contrastive on_focus      232 9.55 Inf       213
251
##  Contrastive post_focus    230 9.55 Inf       212
249
##
## Degrees-of-freedom method: asymptotic
## Confidence level used: 0.95
##
## $contrasts
##  contrast                                        esti
mate    SE  df z.ratio
##  Broad focus - Narrow pre_focus                    -1
.833 0.175 Inf -10.449
##  Broad focus - Narrow on_focus                      0
.791 0.167 Inf   4.723
[illegible]
```

```
##  Broad focus - Narrow post_focus                        2
.535 0.176 Inf  14.391
##  Broad focus - Contrastive pre_focus                   -2
.615 0.176 Inf -14.881
##  Broad focus - Contrastive on_focus                     0
.419 0.167 Inf   2.504
##  Broad focus - Contrastive post_focus                   2
.098 0.176 Inf  11.914
##  Narrow pre_focus - Narrow on_focus                     2
.624 0.175 Inf  14.958
##  Narrow pre_focus - Narrow post_focus                   4
.368 0.199 Inf  21.929
##  Narrow pre_focus - Contrastive pre_focus              -0
.782 0.166 Inf  -4.695
##  Narrow pre_focus - Contrastive on_focus                2
.251 0.175 Inf  12.856
##  Narrow pre_focus - Contrastive post_focus              3
.931 0.199 Inf  19.742
##  Narrow on_focus - Narrow post_focus                    1
.744 0.176 Inf   9.895
##  Narrow on_focus - Contrastive pre_focus               -3
.405 0.176 Inf -19.383
##  Narrow on_focus - Contrastive on_focus                -0
.372 0.167 Inf  -2.225
##  Narrow on_focus - Contrastive post_focus               1
.307 0.176 Inf   7.419
##  Narrow post_focus - Contrastive pre_focus             -5
.150 0.199 Inf -25.823
##  Narrow post_focus - Contrastive on_focus              -2
.116 0.176 Inf -12.020
##  Narrow post_focus - Contrastive post_focus            -0
.437 0.168 Inf  -2.600
##  Contrastive pre_focus - Contrastive on_focus           3
.033 0.175 Inf  17.291
##  Contrastive pre_focus - Contrastive post_focus         4
.713 0.199 Inf  23.639
##  Contrastive on_focus - Contrastive post_focus          1
.680 0.176 Inf   9.542
##  p.value
##   <.0001
##   <.0001
##   <.0001
##   <.0001
##   0.1576
##   <.0001
##   <.0001
##   <.0001
##   0.0001
##   <.0001
##   <.0001
##   <.0001
##   <.0001
##   0.2816
##   <.0001
[illegible]
```

```
##   <.0001
##   <.0001
##   0.1259
##   <.0001
##   <.0001
##   <.0001
##
## Degrees-of-freedom method: asymptotic
## P value adjustment: tukey method for comparing a fam
ily of 7 estimates
```

0.1.3 mean intensity

Hide

```
df <- filter(bob.mad, TrainingOrder == "Pre")

m1 <- lmer(Intensity ~ 1  + (1|word) + (1|Subject) + (1
|word_type) + (1|Block), data = df, REML = FALSE)
casd.hs.int.pre <- lmer(Intensity ~  focus_condition +
(1|word) + (1|Subject)+ (1|word_type) + (1|Block), data
= df, REML = FALSE)

anova(m1, casd.hs.int.pre)
```

```
## Data: df
## Models:
## m1: Intensity ~ 1 + (1 | word) + (1 | Subject) + (1
| word_type) + (1 | Block)
## casd.hs.int.pre: Intensity ~ focus_condition + (1 |
word) + (1 | Subject) + (1 | word_type) + (1 | Block)
##                 npar     AIC     BIC   logLik devian
ce  Chisq Df Pr(>Chisq)
## m1                 6 2487944 2488009 -1243966  24879
32
## casd.hs.int.pre   12 2486950 2487081 -1243463  24869
26 1006.1  6  < 2.2e-16 ***
## ---
## Signif. codes:  0 '***' 0.001 '**' 0.01 '*' 0.05 '.'
0.1 ' ' 1
```

Hide

```
emmeans(casd.hs.int.pre, pairwise~focus_condition)
```

```
## Note: D.f. calculations have been disabled because t
he number of observations exceeds 3000.
## To enable adjustments, add the argument 'pbkrtest.li
```

```
mit = 406471' (or larger)
## [or, globally, 'set emm_options(pbkrtest.limit = 406
471)' or larger];
## but be warned that this may result in large computat
ion time and memory use.
```

```
## Note: D.f. calculations have been disabled because t
he number of observations exceeds 3000.
## To enable adjustments, add the argument 'lmerTest.li
mit = 406471' (or larger)
## [or, globally, 'set emm_options(lmerTest.limit = 406
471)' or larger];
## but be warned that this may result in large computat
ion time and memory use.
```

```
## $emmeans
##  focus_condition        emmean   SE  df asymp.LCL as
ymp.UCL
##  Broad focus              58.3 1.81 Inf      54.7
61.8
##  Narrow pre_focus         58.0 1.81 Inf      54.4
61.6
##  Narrow on_focus          58.1 1.81 Inf      54.5
61.6
##  Narrow post_focus        57.6 1.81 Inf      54.0
61.1
##  Contrastive pre_focus    58.0 1.81 Inf      54.5
61.6
##  Contrastive on_focus     58.3 1.81 Inf      54.8
61.9
##  Contrastive post_focus   57.6 1.81 Inf      54.1
61.2
##
## Degrees-of-freedom method: asymptotic
## Confidence level used: 0.95
##
## $contrasts
##  contrast                                       esti
mate     SE  df z.ratio
##  Broad focus - Narrow pre_focus                  0.2
9759 0.0318 Inf   9.367
##  Broad focus - Narrow on_focus                   0.2
0312 0.0301 Inf   6.743
##  Broad focus - Narrow post_focus                 0.7
3644 0.0317 Inf  23.206
##  Broad focus - Contrastive pre_focus             0.2
9211 0.0318 Inf   9.179
##  Broad focus - Contrastive on_focus             -0.0
2014 0.0302 Inf  -0.666
##  Broad focus - Contrastive post_focus            0.6
8519 0.0318 Inf  21.530
##  Narrow pre_focus - Narrow on_focus             -0.0
9448 0.0317 Inf  -2.978
```

```
##  Narrow pre_focus - Narrow post_focus             0.4
3885 0.0360 Inf  12.180
##  Narrow pre_focus - Contrastive pre_focus       -0.0
0548 0.0303 Inf  -0.181
##  Narrow pre_focus - Contrastive on_focus        -0.3
1773 0.0318 Inf  -9.979
##  Narrow pre_focus - Contrastive post_focus       0.3
8760 0.0361 Inf  10.735
##  Narrow on_focus - Narrow post_focus             0.5
3333 0.0317 Inf  16.839
##  Narrow on_focus - Contrastive pre_focus         0.0
8900 0.0318 Inf   2.800
##  Narrow on_focus - Contrastive on_focus         -0.2
2325 0.0302 Inf  -7.395
##  Narrow on_focus - Contrastive post_focus        0.4
8208 0.0318 Inf  15.177
##  Narrow post_focus - Contrastive pre_focus      -0.4
4433 0.0361 Inf -12.315
##  Narrow post_focus - Contrastive on_focus       -0.7
5658 0.0318 Inf -23.802
##  Narrow post_focus - Contrastive post_focus     -0.0
5125 0.0303 Inf  -1.692
##  Contrastive pre_focus - Contrastive on_focus   -0.3
1225 0.0319 Inf  -9.793
##  Contrastive pre_focus - Contrastive post_focus  0.3
9308 0.0362 Inf  10.873
##  Contrastive on_focus - Contrastive post_focus   0.7
0533 0.0319 Inf  22.131
##  p.value
##   <.0001
##   <.0001
##   <.0001
##   <.0001
##   0.9944
##   <.0001
##   0.0459
##   <.0001
##   1.0000
##   <.0001
##   <.0001
##   <.0001
##   0.0755
##   <.0001
##   <.0001
##   <.0001
##   <.0001
##   0.6212
##   <.0001
##   <.0001
##   <.0001
##
## Degrees-of-freedom method: asymptotic
## P value adjustment: tukey method for comparing a fam
ily of 7 estimates
```

Hide

```
df <- filter(bob.mad, TrainingOrder == "Post")

m1 <- lmer(Intensity ~ 1  + (1|word) + (1|Subject) + (1
|word_type) + (1|Block), data = df, REML = FALSE)
casd.hs.int.post <- lmer(Intensity ~  focus_condition +
(1|word) + (1|Subject)+ (1|word_type) + (1|Block), data
= df, REML = FALSE)

anova(m1, casd.hs.int.post)
```

```
## Data: df
## Models:
## m1: Intensity ~ 1 + (1 | word) + (1 | Subject) + (1
| word_type) + (1 | Block)
## casd.hs.int.post: Intensity ~ focus_condition + (1 |
word) + (1 | Subject) + (1 | word_type) + (1 | Block)
##                  npar     AIC     BIC   logLik devia
nce  Chisq Df Pr(>Chisq)
## m1                  6 2490740 2490805 -1245364  2490
728
## casd.hs.int.post   12 2489856 2489987 -1244916  2489
832 896.01  6  < 2.2e-16
##
## m1
## casd.hs.int.post ***
## ---
## Signif. codes:  0 '***' 0.001 '**' 0.01 '*' 0.05 '.'
0.1 ' ' 1
```

Hide

```
emmeans(casd.hs.int.post, pairwise~focus_condition)
```

```
## Note: D.f. calculations have been disabled because t
he number of observations exceeds 3000.
## To enable adjustments, add the argument 'pbkrtest.li
mit = 400277' (or larger)
## [or, globally, 'set emm_options(pbkrtest.limit = 400
277)' or larger];
## but be warned that this may result in large computat
ion time and memory use.
```

```
## Note: D.f. calculations have been disabled because t
he number of observations exceeds 3000.
## To enable adjustments, add the argument 'lmerTest.li
```

```
mit = 400277' (or larger)
## [or, globally, 'set emm_options(lmerTest.limit = 400
277)' or larger];
## but be warned that this may result in large computat
ion time and memory use.
```

```
## $emmeans
##  focus_condition        emmean   SE  df asymp.LCL as
ymp.UCL
##  Broad focus              59.4 1.91 Inf      55.6
63.1
##  Narrow pre_focus         59.2 1.91 Inf      55.5
63.0
##  Narrow on_focus          59.4 1.91 Inf      55.6
63.1
##  Narrow post_focus        58.8 1.91 Inf      55.1
62.6
##  Contrastive pre_focus    59.1 1.91 Inf      55.3
62.8
##  Contrastive on_focus     59.5 1.91 Inf      55.7
63.2
##  Contrastive post_focus   58.7 1.91 Inf      54.9
62.4
##
## Degrees-of-freedom method: asymptotic
## Confidence level used: 0.95
##
## $contrasts
##  contrast                                       esti
mate     SE  df z.ratio
##  Broad focus - Narrow pre_focus                   0.
1431 0.0337 Inf   4.250
##  Broad focus - Narrow on_focus                   -0.
0158 0.0320 Inf  -0.492
##  Broad focus - Narrow post_focus                  0.
5237 0.0337 Inf  15.538
##  Broad focus - Contrastive pre_focus              0.
2799 0.0337 Inf   8.304
##  Broad focus - Contrastive on_focus              -0.
0955 0.0320 Inf  -2.980
##  Broad focus - Contrastive post_focus             0.
6995 0.0337 Inf  20.736
##  Narrow pre_focus - Narrow on_focus              -0.
1588 0.0337 Inf  -4.717
##  Narrow pre_focus - Narrow post_focus             0.
3807 0.0382 Inf   9.964
##  Narrow pre_focus - Contrastive pre_focus         0.
1368 0.0321 Inf   4.265
##  Narrow pre_focus - Contrastive on_focus         -0.
2385 0.0337 Inf  -7.081
##  Narrow pre_focus - Contrastive post_focus        0.
5565 0.0382 Inf  14.555
##  Narrow on_focus - Narrow post_focus              0.
[illegible]
```

```
5395 0.0337 Inf  16.009
##  Narrow on_focus - Contrastive pre_focus          0.
2956 0.0337 Inf   8.769
##  Narrow on_focus - Contrastive on_focus          -0.
0797 0.0321 Inf  -2.487
##  Narrow on_focus - Contrastive post_focus         0.
7153 0.0337 Inf  21.208
##  Narrow post_focus - Contrastive pre_focus       -0.
2439 0.0382 Inf  -6.377
##  Narrow post_focus - Contrastive on_focus        -0.
6192 0.0337 Inf -18.364
##  Narrow post_focus - Contrastive post_focus       0.
1758 0.0321 Inf   5.478
##  Contrastive pre_focus - Contrastive on_focus    -0.
3753 0.0337 Inf -11.128
##  Contrastive pre_focus - Contrastive post_focus   0.
4197 0.0383 Inf  10.967
##  Contrastive on_focus - Contrastive post_focus    0.
7950 0.0337 Inf  23.558
##  p.value
##   0.0004
##   0.9990
##   <.0001
##   <.0001
##   0.0457
##   <.0001
##   <.0001
##   <.0001
##   0.0004
##   <.0001
##   <.0001
##   <.0001
##   <.0001
##   0.1638
##   <.0001
##   <.0001
##   <.0001
##   <.0001
##   <.0001
##   <.0001
##   <.0001
##
## Degrees-of-freedom method: asymptotic
## P value adjustment: tukey method for comparing a fam
ily of 7 estimates
```

0.2 RobotSpeech by focus condition

0.2.1 mean duration

Hide

```
df <- filter(bob.mad, TrainingOrder == "Pre")

m1 <- lmer(Duration ~ 1  + (1|word) + (1|Subject) + (1|
word_type) + (1|Block), data = df, REML = FALSE)
```

```
## boundary (singular) fit: see help('isSingular')
```

Hide

```
casd.rs.dur.pre <- lmer(Duration ~  focus_condition + (
1|word) + (1|Subject)+ (1|word_type) + (1|Block), data
= df, REML = FALSE)
```

```
## Warning in checkConv(attr(opt, "derivs"), opt$par, c
trl = control$checkConv, :
## Model failed to converge with max|grad| = 0.00507113
(tol = 0.002, component 1)
```

Hide

```
anova(m1, casd.rs.dur.pre)
```

```
## Data: df
## Models:
## m1: Duration ~ 1 + (1 | word) + (1 | Subject) + (1 |
word_type) + (1 | Block)
## casd.rs.dur.pre: Duration ~ focus_condition + (1 | w
ord) + (1 | Subject) + (1 | word_type) + (1 | Block)
##                    npar    AIC    BIC  logLik deviance
Chisq Df Pr(>Chisq)
## m1                    6 249222 249270 -124605   249210
## casd.rs.dur.pre   12 249097 249193 -124536   249073
137.25  6  < 2.2e-16 ***
## ---
## Signif. codes:  0 '***' 0.001 '**' 0.01 '*' 0.05 '.'
0.1 ' ' 1
```

Hide

```
emmeans(casd.rs.dur.pre, pairwise~focus_condition)
```

```
## Note: D.f. calculations have been disabled because t
he number of observations exceeds 3000.
```

```
## To enable adjustments, add the argument 'pbkrtest.li
mit = 22777' (or larger)
## [or, globally, 'set emm_options(pbkrtest.limit = 227
77)' or larger];
## but be warned that this may result in large computat
ion time and memory use.
```

```
## Note: D.f. calculations have been disabled because t
he number of observations exceeds 3000.
## To enable adjustments, add the argument 'lmerTest.li
mit = 22777' (or larger)
## [or, globally, 'set emm_options(lmerTest.limit = 227
77)' or larger];
## but be warned that this may result in large computat
ion time and memory use.
```

```
## $emmeans
##  focus_condition        emmean   SE  df asymp.LCL as
ymp.UCL
##  Broad focus               204 10.7 Inf       183
225
##  Narrow pre_focus          196 10.7 Inf       175
217
##  Narrow on_focus           203 10.7 Inf       182
224
##  Narrow post_focus         197 10.7 Inf       176
218
##  Contrastive pre_focus     207 10.7 Inf       186
228
##  Contrastive on_focus      209 10.7 Inf       188
230
##  Contrastive post_focus    200 10.7 Inf       179
221
##
## Degrees-of-freedom method: asymptotic
## Confidence level used: 0.95
##
## $contrasts
##  contrast                                       esti
mate   SE  df z.ratio
##  Broad focus - Narrow pre_focus                    7
.981 1.48 Inf   5.396
##  Broad focus - Narrow on_focus                     0
.795 1.40 Inf   0.566
##  Broad focus - Narrow post_focus                   6
.693 1.47 Inf   4.540
##  Broad focus - Contrastive pre_focus              -2
.349 1.48 Inf  -1.585
##  Broad focus - Contrastive on_focus               -5
.184 1.40 Inf  -3.691
##  Broad focus - Contrastive post_focus              4
.608 1.48 Inf   3.120
##  Narrow pre_focus - Narrow on_focus               -7
```

```
.186 1.48 Inf  -4.860
##  Narrow pre_focus - Narrow post_focus              -1
.288 1.67 Inf  -0.770
##  Narrow pre_focus - Contrastive pre_focus         -10
.330 1.41 Inf  -7.303
##  Narrow pre_focus - Contrastive on_focus          -13
.166 1.48 Inf  -8.904
##  Narrow pre_focus - Contrastive post_focus         -3
.374 1.68 Inf  -2.012
##  Narrow on_focus - Narrow post_focus                5
.897 1.48 Inf   3.997
##  Narrow on_focus - Contrastive pre_focus           -3
.144 1.48 Inf  -2.123
##  Narrow on_focus - Contrastive on_focus            -5
.980 1.40 Inf  -4.256
##  Narrow on_focus - Contrastive post_focus           3
.812 1.48 Inf   2.580
##  Narrow post_focus - Contrastive pre_focus         -9
.042 1.68 Inf  -5.393
##  Narrow post_focus - Contrastive on_focus         -11
.877 1.48 Inf  -8.047
##  Narrow post_focus - Contrastive post_focus        -2
.085 1.40 Inf  -1.486
##  Contrastive pre_focus - Contrastive on_focus      -2
.835 1.48 Inf  -1.914
##  Contrastive pre_focus - Contrastive post_focus     6
.957 1.68 Inf   4.145
##  Contrastive on_focus - Contrastive post_focus      9
.792 1.48 Inf   6.624
##  p.value
##   <.0001
##   0.9977
##   0.0001
##   0.6920
##   0.0042
##   0.0299
##   <.0001
##   0.9878
##   <.0001
##   <.0001
##   0.4066
##   0.0013
##   0.3392
##   0.0004
##   0.1322
##   <.0001
##   <.0001
##   0.7537
##   0.4708
##   0.0007
##   <.0001
##
## Degrees-of-freedom method: asymptotic
## P value adjustment: tukey method for comparing a fam
```

```
ily of 7 estimates
```

Hide

```
df <- filter(bob.mad, TrainingOrder == "Post")

m1 <- lmer(Duration ~ 1  + (1|word) + (1|Subject) + (1|
word_type) + (1|Block), data = df, REML = FALSE)
casd.rs.dur.post <- lmer(Duration ~  focus_condition +
(1|word) + (1|Subject)+ (1|word_type) + (1|Block), data
= df, REML = FALSE)

anova(m1, casd.rs.dur.post)
```

```
## Data: df
## Models:
## m1: Duration ~ 1 + (1 | word) + (1 | Subject) + (1 |
word_type) + (1 | Block)
## casd.rs.dur.post: Duration ~ focus_condition + (1 |
word) + (1 | Subject) + (1 | word_type) + (1 | Block)
##                   npar    AIC    BIC  logLik deviance
Chisq Df Pr(>Chisq)
## m1                   6 251569 251617 -125779   251557
## casd.rs.dur.post   12 251464 251561 -125720   251440
116.93  6  < 2.2e-16 ***
## ---
## Signif. codes:  0 '***' 0.001 '**' 0.01 '*' 0.05 '.'
0.1 ' ' 1
```

Hide

```
emmeans(casd.rs.dur.post, pairwise~focus_condition)
```

```
## Note: D.f. calculations have been disabled because t
he number of observations exceeds 3000.
## To enable adjustments, add the argument 'pbkrtest.li
mit = 23547' (or larger)
## [or, globally, 'set emm_options(pbkrtest.limit = 235
47)' or larger];
## but be warned that this may result in large computat
ion time and memory use.
```

```
## Note: D.f. calculations have been disabled because t
he number of observations exceeds 3000.
```

```
## To enable adjustments, add the argument 'lmerTest.li
mit = 23547' (or larger)
## [or, globally, 'set emm_options(lmerTest.limit = 235
47)' or larger];
## but be warned that this may result in large computat
ion time and memory use.
```

```
## $emmeans
##  focus_condition        emmean   SE  df asymp.LCL as
ymp.UCL
##  Broad focus               183 13.1 Inf       158
209
##  Narrow pre_focus          181 13.1 Inf       155
207
##  Narrow on_focus           185 13.1 Inf       159
210
##  Narrow post_focus         180 13.1 Inf       154
205
##  Contrastive pre_focus     189 13.1 Inf       163
214
##  Contrastive on_focus      190 13.1 Inf       165
216
##  Contrastive post_focus    182 13.1 Inf       156
207
##
## Degrees-of-freedom method: asymptotic
## Confidence level used: 0.95
##
## $contrasts
##  contrast                                       esti
mate   SE  df z.ratio
##  Broad focus - Narrow pre_focus
2.43 1.28 Inf   1.905
##  Broad focus - Narrow on_focus                     -
1.14 1.22 Inf  -0.937
##  Broad focus - Narrow post_focus
3.79 1.28 Inf   2.957
##  Broad focus - Contrastive pre_focus               -
5.15 1.28 Inf  -4.025
##  Broad focus - Contrastive on_focus                -
6.91 1.22 Inf  -5.673
##  Broad focus - Contrastive post_focus
1.63 1.28 Inf   1.274
##  Narrow pre_focus - Narrow on_focus                -
3.57 1.28 Inf  -2.802
##  Narrow pre_focus - Narrow post_focus
1.36 1.45 Inf   0.937
##  Narrow pre_focus - Contrastive pre_focus          -
7.59 1.22 Inf  -6.232
##  Narrow pre_focus - Contrastive on_focus           -
9.34 1.28 Inf  -7.321
##  Narrow pre_focus - Contrastive post_focus         -
0.80 1.45 Inf  -0.553
##  Narrow on_focus - Narrow post_focus
```

```
4.93 1.28 Inf   3.850
##  Narrow on_focus - Contrastive pre_focus         -
4.01 1.28 Inf  -3.137
##  Narrow on_focus - Contrastive on_focus          -
5.77 1.22 Inf  -4.741
##  Narrow on_focus - Contrastive post_focus
2.77 1.28 Inf   2.167
##  Narrow post_focus - Contrastive pre_focus       -
8.94 1.45 Inf  -6.162
##  Narrow post_focus - Contrastive on_focus       -1
0.70 1.28 Inf  -8.356
##  Narrow post_focus - Contrastive post_focus      -
2.16 1.22 Inf  -1.774
##  Contrastive pre_focus - Contrastive on_focus    -
1.75 1.28 Inf  -1.370
##  Contrastive pre_focus - Contrastive post_focus
6.79 1.45 Inf   4.677
##  Contrastive on_focus - Contrastive post_focus
8.54 1.28 Inf   6.674
##  p.value
##   0.4767
##   0.9665
##   0.0488
##   0.0011
##   <.0001
##   0.8640
##   0.0751
##   0.9665
##   <.0001
##   <.0001
##   0.9980
##   0.0023
##   0.0284
##   <.0001
##   0.3138
##   <.0001
##   <.0001
##   0.5657
##   0.8180
##   0.0001
##   <.0001
##
## Degrees-of-freedom method: asymptotic
## P value adjustment: tukey method for comparing a fam
ily of 7 estimates
```

0.2.2 mean f0

Hide

```
df <- filter(bob.mad, TrainingOrder == "Pre")

m1 <- lmer(F0 ~ 1  + (1|word) + (1|Subject) + (1|word_t
```

```
ype) + (1|Block), data = df, REML = FALSE)
casd.rs.f0.pre <- lmer(F0 ~  focus_condition + (1|word)
+ (1|Subject)+ (1|word_type) + (1|Block), data = df, RE
ML = FALSE)

anova(m1, casd.rs.f0.pre)
```

```
## Data: df
## Models:
## m1: F0 ~ 1 + (1 | word) + (1 | Subject) + (1 | word_
type) + (1 | Block)
## casd.rs.f0.pre: F0 ~ focus_condition + (1 | word) +
(1 | Subject) + (1 | word_type) + (1 | Block)
##                npar     AIC     BIC   logLik devianc
e  Chisq Df Pr(>Chisq)
## m1                6 3849431 3849497 -1924710  384941
9
## casd.rs.f0.pre   12 3848626 3848756 -1924301  384860
2 817.68  6  < 2.2e-16 ***
## ---
## Signif. codes:  0 '***' 0.001 '**' 0.01 '*' 0.05 '.'
0.1 ' ' 1
```

Hide

```
emmeans(casd.rs.f0.pre, pairwise~focus_condition)
```

```
## Note: D.f. calculations have been disabled because t
he number of observations exceeds 3000.
## To enable adjustments, add the argument 'pbkrtest.li
mit = 400029' (or larger)
## [or, globally, 'set emm_options(pbkrtest.limit = 400
029)' or larger];
## but be warned that this may result in large computat
ion time and memory use.
```

```
## Note: D.f. calculations have been disabled because t
he number of observations exceeds 3000.
## To enable adjustments, add the argument 'lmerTest.li
mit = 400029' (or larger)
## [or, globally, 'set emm_options(lmerTest.limit = 400
029)' or larger];
## but be warned that this may result in large computat
ion time and memory use.
```

```
## $emmeans
```

```
##  focus_condition        emmean   SE  df asymp.LCL as
ymp.UCL
##  Broad focus               228 8.63 Inf       211
245
##  Narrow pre_focus          232 8.63 Inf       215
249
##  Narrow on_focus           229 8.63 Inf       212
246
##  Narrow post_focus         227 8.63 Inf       210
244
##  Contrastive pre_focus     231 8.63 Inf       214
248
##  Contrastive on_focus      229 8.63 Inf       212
246
##  Contrastive post_focus    227 8.63 Inf       210
244
##
## Degrees-of-freedom method: asymptotic
## Confidence level used: 0.95
##
## $contrasts
##  contrast                                      esti
mate    SE  df z.ratio
##  Broad focus - Narrow pre_focus                 -4.
0490 0.185 Inf -21.927
##  Broad focus - Narrow on_focus                  -0.
6017 0.175 Inf  -3.434
##  Broad focus - Narrow post_focus                 0.
6733 0.185 Inf   3.648
##  Broad focus - Contrastive pre_focus            -3.
2987 0.184 Inf -17.920
##  Broad focus - Contrastive on_focus             -1.
0495 0.175 Inf  -5.991
##  Broad focus - Contrastive post_focus            0.
6710 0.184 Inf   3.640
##  Narrow pre_focus - Narrow on_focus              3.
4473 0.185 Inf  18.653
##  Narrow pre_focus - Narrow post_focus            4.
7223 0.210 Inf  22.500
##  Narrow pre_focus - Contrastive pre_focus        0.
7503 0.176 Inf   4.274
##  Narrow pre_focus - Contrastive on_focus         2.
9995 0.185 Inf  16.224
##  Narrow pre_focus - Contrastive post_focus       4.
7200 0.210 Inf  22.514
##  Narrow on_focus - Narrow post_focus             1.
2750 0.185 Inf   6.892
##  Narrow on_focus - Contrastive pre_focus        -2.
6970 0.184 Inf -14.640
##  Narrow on_focus - Contrastive on_focus         -0.
4478 0.176 Inf  -2.551
##  Narrow on_focus - Contrastive post_focus        1.
2727 0.185 Inf   6.888
##  Narrow post_focus - Contrastive pre_focus      -3.
[illegible]
```

```
9720 0.209 Inf -18.973
##  Narrow post_focus - Contrastive on_focus        -1.
7228 0.185 Inf  -9.319
##  Narrow post_focus - Contrastive post_focus      -0.
0023 0.176 Inf  -0.013
##  Contrastive pre_focus - Contrastive on_focus     2.
2492 0.184 Inf  12.205
##  Contrastive pre_focus - Contrastive post_focus   3.
9697 0.209 Inf  18.982
##  Contrastive on_focus - Contrastive post_focus    1.
7205 0.185 Inf   9.319
##  p.value
##   <.0001
##   0.0107
##   0.0049
##   <.0001
##   <.0001
##   0.0051
##   <.0001
##   <.0001
##   0.0004
##   <.0001
##   <.0001
##   <.0001
##   <.0001
##   0.1412
##   <.0001
##   <.0001
##   <.0001
##   1.0000
##   <.0001
##   <.0001
##   <.0001
##
## Degrees-of-freedom method: asymptotic
## P value adjustment: tukey method for comparing a fam
ily of 7 estimates
```

Hide

```
df <- filter(bob.mad, TrainingOrder == "Post")

m1 <- lmer(F0 ~ 1  + (1|word) + (1|Subject) + (1|word_t
ype) + (1|Block), data = df, REML = FALSE)
casd.rs.f0.post <- lmer(F0 ~  focus_condition + (1|word
) + (1|Subject)+ (1|word_type) + (1|Block), data = df,
REML = FALSE)

anova(m1, casd.rs.f0.post)
```

```
## Data: df
## Models:
## m1: F0 ~ 1 + (1 | word) + (1 | Subject) + (1 | word_
type) + (1 | Block)
## casd.rs.f0.post: F0 ~ focus_condition + (1 | word) +
(1 | Subject) + (1 | word_type) + (1 | Block)
##                  npar     AIC     BIC   logLik devian
ce  Chisq Df Pr(>Chisq)
## m1                  6 3932352 3932418 -1966170  39323
40
## casd.rs.f0.post   12 3931651 3931782 -1965813  39316
27 713.47  6  < 2.2e-16 ***
## ---
## Signif. codes:  0 '***' 0.001 '**' 0.01 '*' 0.05 '.'
0.1 ' ' 1
```

Hide

```
emmeans(casd.rs.f0.post, pairwise~focus_condition)
```

```
## Note: D.f. calculations have been disabled because t
he number of observations exceeds 3000.
## To enable adjustments, add the argument 'pbkrtest.li
mit = 412153' (or larger)
## [or, globally, 'set emm_options(pbkrtest.limit = 412
153)' or larger];
## but be warned that this may result in large computat
ion time and memory use.
```

```
## Note: D.f. calculations have been disabled because t
he number of observations exceeds 3000.
## To enable adjustments, add the argument 'lmerTest.li
mit = 412153' (or larger)
## [or, globally, 'set emm_options(lmerTest.limit = 412
153)' or larger];
## but be warned that this may result in large computat
ion time and memory use.
```

```
## $emmeans
##  focus_condition        emmean   SE  df asymp.LCL as
ymp.UCL
##  Broad focus               232 10.4 Inf       212
252
##  Narrow pre_focus          234 10.4 Inf       214
255
##  Narrow on_focus           232 10.4 Inf       212
253
##  Narrow post_focus         229 10.4 Inf       209
250
```

```
[illegible]
##  Contrastive pre_focus     234 10.4 Inf       213
254
##  Contrastive on_focus      232 10.4 Inf       212
253
##  Contrastive post_focus    231 10.4 Inf       211
251
##
## Degrees-of-freedom method: asymptotic
## Confidence level used: 0.95
##
## $contrasts
##  contrast                                       esti
mate    SE  df z.ratio
##  Broad focus - Narrow pre_focus                   -2
.252 0.174 Inf -12.918
##  Broad focus - Narrow on_focus                    -0
.349 0.166 Inf  -2.095
##  Broad focus - Narrow post_focus                   2
.780 0.175 Inf  15.857
##  Broad focus - Contrastive pre_focus              -1
.588 0.174 Inf  -9.105
##  Broad focus - Contrastive on_focus               -0
.453 0.166 Inf  -2.725
##  Broad focus - Contrastive post_focus              0
.887 0.175 Inf   5.056
##  Narrow pre_focus - Narrow on_focus                1
.903 0.174 Inf  10.928
##  Narrow pre_focus - Narrow post_focus              5
.032 0.198 Inf  25.436
##  Narrow pre_focus - Contrastive pre_focus          0
.664 0.165 Inf   4.016
##  Narrow pre_focus - Contrastive on_focus           1
.799 0.174 Inf  10.332
##  Narrow pre_focus - Contrastive post_focus         3
.139 0.198 Inf  15.864
##  Narrow on_focus - Narrow post_focus               3
.129 0.175 Inf  17.878
##  Narrow on_focus - Contrastive pre_focus          -1
.239 0.174 Inf  -7.113
##  Narrow on_focus - Contrastive on_focus           -0
.104 0.166 Inf  -0.629
##  Narrow on_focus - Contrastive post_focus          1
.236 0.175 Inf   7.057
##  Narrow post_focus - Contrastive pre_focus        -4
.368 0.198 Inf -22.070
##  Narrow post_focus - Contrastive on_focus         -3
.233 0.175 Inf -18.491
##  Narrow post_focus - Contrastive post_focus       -1
.893 0.167 Inf -11.360
##  Contrastive pre_focus - Contrastive on_focus      1
.135 0.174 Inf   6.516
##  Contrastive pre_focus - Contrastive post_focus    2
.475 0.198 Inf  12.503
##  Contrastive on_focus - Contrastive post_focus     1
```

```
.340 0.175 Inf   7.661
##  p.value
##   <.0001
##   0.3554
##   <.0001
##   <.0001
##   0.0922
##   <.0001
##   <.0001
##   <.0001
##   0.0012
##   <.0001
##   <.0001
##   <.0001
##   <.0001
##   0.9959
##   <.0001
##   <.0001
##   <.0001
##   <.0001
##   <.0001
##   <.0001
##   <.0001
##
## Degrees-of-freedom method: asymptotic
## P value adjustment: tukey method for comparing a fam
ily of 7 estimates
```

0.2.3 mean intensity

Hide

```
df <- filter(bob.mad, TrainingOrder == "Pre")

m1 <- lmer(Intensity ~ 1  + (1|word) + (1|Subject) + (1
|word_type) + (1|Block), data = df, REML = FALSE)
casd.rs.int.pre <- lmer(Intensity ~  focus_condition +
(1|word) + (1|Subject)+ (1|word_type) + (1|Block), data
= df, REML = FALSE)

anova(m1, casd.rs.int.pre)
```

```
## Data: df
## Models:
## m1: Intensity ~ 1 + (1 | word) + (1 | Subject) + (1
| word_type) + (1 | Block)
## casd.rs.int.pre: Intensity ~ focus_condition + (1 |
word) + (1 | Subject) + (1 | word_type) + (1 | Block)
##                 npar    AIC    BIC  logLik devian
```

```
##                    npar     AIC     BIC   logLik devian
ce  Chisq Df Pr(>Chisq)
## m1                  6 2521363 2521429 -1260676  25213
51
## casd.rs.int.pre   12 2520725 2520856 -1260351  25207
01 650.32  6  < 2.2e-16 ***
## ---
## Signif. codes:  0 '***' 0.001 '**' 0.01 '*' 0.05 '.'
0.1 ' ' 1
```

Hide

```
emmeans(casd.rs.int.pre, pairwise~focus_condition)
```

```
## Note: D.f. calculations have been disabled because t
he number of observations exceeds 3000.
## To enable adjustments, add the argument 'pbkrtest.li
mit = 412550' (or larger)
## [or, globally, 'set emm_options(pbkrtest.limit = 412
550)' or larger];
## but be warned that this may result in large computat
ion time and memory use.
```

```
## Note: D.f. calculations have been disabled because t
he number of observations exceeds 3000.
## To enable adjustments, add the argument 'lmerTest.li
mit = 412550' (or larger)
## [or, globally, 'set emm_options(lmerTest.limit = 412
550)' or larger];
## but be warned that this may result in large computat
ion time and memory use.
```

```
## $emmeans
##  focus_condition        emmean   SE  df asymp.LCL as
ymp.UCL
##  Broad focus              60.4 2.67 Inf      55.2
65.6
##  Narrow pre_focus         60.7 2.67 Inf      55.5
65.9
##  Narrow on_focus          60.7 2.67 Inf      55.5
66.0
##  Narrow post_focus        60.1 2.67 Inf      54.9
65.4
##  Contrastive pre_focus    60.5 2.67 Inf      55.3
65.7
##  Contrastive on_focus     60.8 2.67 Inf      55.5
66.0
##  Contrastive post_focus   60.3 2.67 Inf      55.0
65.5
##
## Degrees-of-freedom method: asymptotic
## Confidence level used: 0.95
##
```

```
##
## $contrasts
##  contrast                                           esti
mate     SE  df z.ratio
##  Broad focus - Narrow pre_focus                      -0.
2908 0.0314 Inf  -9.259
##  Broad focus - Narrow on_focus                       -0.
3181 0.0299 Inf -10.639
##  Broad focus - Narrow post_focus                      0.
2680 0.0314 Inf   8.524
##  Broad focus - Contrastive pre_focus                 -0.
0781 0.0314 Inf  -2.492
##  Broad focus - Contrastive on_focus                  -0.
3576 0.0299 Inf -11.969
##  Broad focus - Contrastive post_focus                 0.
1634 0.0315 Inf   5.196
##  Narrow pre_focus - Narrow on_focus                  -0.
0272 0.0314 Inf  -0.867
##  Narrow pre_focus - Narrow post_focus                 0.
5588 0.0357 Inf  15.675
##  Narrow pre_focus - Contrastive pre_focus             0.
2127 0.0299 Inf   7.122
##  Narrow pre_focus - Contrastive on_focus             -0.
0668 0.0314 Inf  -2.124
##  Narrow pre_focus - Contrastive post_focus            0.
4543 0.0357 Inf  12.737
##  Narrow on_focus - Narrow post_focus                  0.
5861 0.0315 Inf  18.623
##  Narrow on_focus - Contrastive pre_focus              0.
2400 0.0314 Inf   7.649
##  Narrow on_focus - Contrastive on_focus              -0.
0395 0.0299 Inf  -1.322
##  Narrow on_focus - Contrastive post_focus             0.
4815 0.0315 Inf  15.293
##  Narrow post_focus - Contrastive pre_focus           -0.
3461 0.0356 Inf  -9.724
##  Narrow post_focus - Contrastive on_focus            -0.
6256 0.0314 Inf -19.902
##  Narrow post_focus - Contrastive post_focus          -0.
1045 0.0299 Inf  -3.494
##  Contrastive pre_focus - Contrastive on_focus        -0.
2795 0.0314 Inf  -8.909
##  Contrastive pre_focus - Contrastive post_focus       0.
2416 0.0356 Inf   6.784
##  Contrastive on_focus - Contrastive post_focus        0.
5210 0.0314 Inf  16.568
##  p.value
##   <.0001
##   <.0001
##   <.0001
##   0.1622
##   <.0001
##   <.0001
##   0.9774
##   <.0001
```

```
## <.0001
## <.0001
## 0.3383
## <.0001
## <.0001
## <.0001
## 0.8421
## <.0001
## <.0001
## <.0001
## 0.0086
## <.0001
## <.0001
## <.0001
##
## Degrees-of-freedom method: asymptotic
## P value adjustment: tukey method for comparing a fam
ily of 7 estimates
```

Hide

```
df <- filter(bob.mad, TrainingOrder == "Post")

m1 <- lmer(Intensity ~ 1  + (1|word) + (1|Subject) + (1
|word_type) + (1|Block), data = df, REML = FALSE)
casd.rs.int.post <- lmer(Intensity ~  focus_condition +
(1|word) + (1|Subject)+ (1|word_type) + (1|Block), data
= df, REML = FALSE)

anova(m1, casd.rs.int.post)
```

```
## Data: df
## Models:
## m1: Intensity ~ 1 + (1 | word) + (1 | Subject) + (1
| word_type) + (1 | Block)
## casd.rs.int.post: Intensity ~ focus_condition + (1 |
word) + (1 | Subject) + (1 | word_type) + (1 | Block)
##                  npar     AIC     BIC   logLik devia
nce  Chisq Df Pr(>Chisq)
## m1                  6 2599076 2599142 -1299532  2599
064
## casd.rs.int.post   12 2598632 2598763 -1299304  2598
608 456.64  6  < 2.2e-16
##
## m1
## casd.rs.int.post ***
## ---
```

```
## Signif. codes:  0 '***' 0.001 '**' 0.01 '*' 0.05 '.'
0.1 ' ' 1
```

Hide

```
emmeans(casd.rs.int.post, pairwise~focus_condition)
```

```
## Note: D.f. calculations have been disabled because t
he number of observations exceeds 3000.
## To enable adjustments, add the argument 'pbkrtest.li
mit = 430305' (or larger)
## [or, globally, 'set emm_options(pbkrtest.limit = 430
305)' or larger];
## but be warned that this may result in large computat
ion time and memory use.
```

```
## Note: D.f. calculations have been disabled because t
he number of observations exceeds 3000.
## To enable adjustments, add the argument 'lmerTest.li
mit = 430305' (or larger)
## [or, globally, 'set emm_options(lmerTest.limit = 430
305)' or larger];
## but be warned that this may result in large computat
ion time and memory use.
```

```
## $emmeans
##  focus_condition        emmean   SE  df asymp.LCL as
ymp.UCL
##  Broad focus              61.7 2.68 Inf      56.4
66.9
##  Narrow pre_focus         61.7 2.68 Inf      56.5
67.0
##  Narrow on_focus          61.7 2.68 Inf      56.5
67.0
##  Narrow post_focus        61.4 2.68 Inf      56.1
66.6
##  Contrastive pre_focus    61.6 2.68 Inf      56.4
66.9
##  Contrastive on_focus     62.0 2.68 Inf      56.7
67.2
##  Contrastive post_focus   61.6 2.68 Inf      56.4
66.9
##
## Degrees-of-freedom method: asymptotic
## Confidence level used: 0.95
##
## $contrasts
##  contrast                                          esti
mate     SE  df z.ratio
##  Broad focus - Narrow pre_focus                     -0.
0376 0.0297 Inf  -1.266
##  Broad focus - Narrow on_focus                      -0.
```

```
0759 0.0282 Inf  -2.688
##  Broad focus - Narrow post_focus                   0.
3040 0.0297 Inf  10.238
##  Broad focus - Contrastive pre_focus               0.
0709 0.0297 Inf   2.387
##  Broad focus - Contrastive on_focus               -0.
2953 0.0282 Inf -10.458
##  Broad focus - Contrastive post_focus              0.
0324 0.0297 Inf   1.090
##  Narrow pre_focus - Narrow on_focus               -0.
0383 0.0297 Inf  -1.290
##  Narrow pre_focus - Narrow post_focus              0.
3416 0.0337 Inf  10.141
##  Narrow pre_focus - Contrastive pre_focus          0.
1084 0.0282 Inf   3.840
##  Narrow pre_focus - Contrastive on_focus          -0.
2577 0.0297 Inf  -8.676
##  Narrow pre_focus - Contrastive post_focus         0.
0699 0.0337 Inf   2.077
##  Narrow on_focus - Narrow post_focus               0.
3799 0.0297 Inf  12.786
##  Narrow on_focus - Contrastive pre_focus           0.
1468 0.0297 Inf   4.942
##  Narrow on_focus - Contrastive on_focus           -0.
2194 0.0283 Inf  -7.764
##  Narrow on_focus - Contrastive post_focus          0.
1083 0.0297 Inf   3.644
##  Narrow post_focus - Contrastive pre_focus        -0.
2331 0.0337 Inf  -6.924
##  Narrow post_focus - Contrastive on_focus         -0.
5993 0.0297 Inf -20.175
##  Narrow post_focus - Contrastive post_focus       -0.
2716 0.0283 Inf  -9.613
##  Contrastive pre_focus - Contrastive on_focus     -0.
3661 0.0297 Inf -12.331
##  Contrastive pre_focus - Contrastive post_focus  -0.
0385 0.0337 Inf  -1.144
##  Contrastive on_focus - Contrastive post_focus    0.
3276 0.0297 Inf  11.032
##  p.value
##   0.8673
##   0.1013
##   <.0001
##   0.2038
##   <.0001
##   0.9313
##   0.8570
##   <.0001
##   0.0024
##   <.0001
##   0.3664
##   <.0001
##   <.0001
##   <.0001
```

```
##   0.0050
##   <.0001
##   <.0001
##   <.0001
##   <.0001
##   0.9146
##   <.0001
##
## Degrees-of-freedom method: asymptotic
## P value adjustment: tukey method for comparing a fam
ily of 7 estimates
```

0.3 CASD Contrl by focus condition

0.3.1 mean duration

Hide

```
df <- filter(bob.mad, TrainingOrder == "Pre")

m1 <- lmer(Duration ~ 1  + (1|word) + (1|Subject) + (1|
word_type), data = df, REML = FALSE)
casd.ctrl.dur.pre <- lmer(Duration ~  focus_condition +
(1|word) + (1|Subject)+ (1|word_type), data = df, REML
= FALSE)

anova(m1, casd.ctrl.dur.pre)
```

```
## Data: df
## Models:
## m1: Duration ~ 1 + (1 | word) + (1 | Subject) + (1 |
word_type)
## casd.ctrl.dur.pre: Duration ~ focus_condition + (1 |
word) + (1 | Subject) + (1 | word_type)
##                   npar    AIC    BIC  logLik devianc
e  Chisq Df Pr(>Chisq)
## m1                   5 200569 200608 -100280   20055
9
## casd.ctrl.dur.pre   11 200465 200551 -100222   20044
3 116.19  6  < 2.2e-16 ***
## ---
## Signif. codes:  0 '***' 0.001 '**' 0.01 '*' 0.05 '.'
0.1 ' ' 1
```

Hide

```
emmeans(casd.ctrl.dur.pre, pairwise~focus_condition)
```

```
## Note: D.f. calculations have been disabled because t
he number of observations exceeds 3000.
## To enable adjustments, add the argument 'pbkrtest.li
mit = 18395' (or larger)
## [or, globally, 'set emm_options(pbkrtest.limit = 183
95)' or larger];
## but be warned that this may result in large computat
ion time and memory use.
```

```
## Note: D.f. calculations have been disabled because t
he number of observations exceeds 3000.
## To enable adjustments, add the argument 'lmerTest.li
mit = 18395' (or larger)
## [or, globally, 'set emm_options(lmerTest.limit = 183
95)' or larger];
## but be warned that this may result in large computat
ion time and memory use.
```

```
## $emmeans
##  focus_condition        emmean   SE  df asymp.LCL as
ymp.UCL
##  Broad focus               208 13.8 Inf       181
235
##  Narrow pre_focus          198 13.8 Inf       171
225
##  Narrow on_focus           205 13.8 Inf       178
232
##  Narrow post_focus         202 13.8 Inf       175
229
##  Contrastive pre_focus     206 13.8 Inf       178
233
##  Contrastive on_focus      214 13.8 Inf       186
241
##  Contrastive post_focus    206 13.8 Inf       179
233
##
## Degrees-of-freedom method: asymptotic
## Confidence level used: 0.95
##
## $contrasts
##  contrast                                       esti
mate   SE  df z.ratio
##  Broad focus - Narrow pre_focus                    9
.560 1.61 Inf   5.948
##  Broad focus - Narrow on_focus                     2
.573 1.53 Inf   1.679
##  Broad focus - Narrow post_focus                   6
```

```
.090 1.61 Inf   3.779
##  Broad focus - Contrastive pre_focus                 2
.168 1.61 Inf   1.349
##  Broad focus - Contrastive on_focus                 -5
.804 1.53 Inf  -3.788
##  Broad focus - Contrastive post_focus                1
.613 1.61 Inf   0.999
##  Narrow pre_focus - Narrow on_focus                 -6
.987 1.61 Inf  -4.347
##  Narrow pre_focus - Narrow post_focus               -3
.470 1.82 Inf  -1.903
##  Narrow pre_focus - Contrastive pre_focus           -7
.393 1.53 Inf  -4.831
##  Narrow pre_focus - Contrastive on_focus           -15
.364 1.61 Inf  -9.556
##  Narrow pre_focus - Contrastive post_focus          -7
.947 1.83 Inf  -4.351
##  Narrow on_focus - Narrow post_focus                 3
.517 1.61 Inf   2.179
##  Narrow on_focus - Contrastive pre_focus            -0
.405 1.61 Inf  -0.252

##  Narrow on_focus - Contrastive on_focus             -8
.377 1.53 Inf  -5.463
##  Narrow on_focus - Contrastive post_focus           -0
.960 1.62 Inf  -0.594
##  Narrow post_focus - Contrastive pre_focus          -3
.923 1.82 Inf  -2.151
##  Narrow post_focus - Contrastive on_focus          -11
.895 1.61 Inf  -7.371
##  Narrow post_focus - Contrastive post_focus         -4
.477 1.54 Inf  -2.912
##  Contrastive pre_focus - Contrastive on_focus       -7
.972 1.61 Inf  -4.958
##  Contrastive pre_focus - Contrastive post_focus     -0
.554 1.83 Inf  -0.303
##  Contrastive on_focus - Contrastive post_focus       7
.418 1.62 Inf   4.588
##  p.value
##   <.0001
##   0.6299
##   0.0030
##   0.8287
##   0.0029
##   0.9543
##   0.0003
##   0.4785
##   <.0001
##   <.0001
##   0.0003
##   0.3068
##   1.0000
##   <.0001
##   0.9970
##   0.3228
```

```
##   <.0001
##   0.0554
##   <.0001
##   0.9999
##   0.0001
##
## Degrees-of-freedom method: asymptotic
## P value adjustment: tukey method for comparing a fam
ily of 7 estimates
```

Hide

```
df <- filter(bob.mad, TrainingOrder == "Post")

m1 <- lmer(Duration ~ 1  + (1|word) + (1|Subject) + (1|
word_type), data = df, REML = FALSE)
casd.ctrl.dur.post <- lmer(Duration ~  focus_condition
+ (1|word) + (1|Subject)+ (1|word_type), data = df, REM
L = FALSE)

anova(m1, casd.ctrl.dur.post)
```

```
## Data: df
## Models:
## m1: Duration ~ 1 + (1 | word) + (1 | Subject) + (1 |
word_type)
## casd.ctrl.dur.post: Duration ~ focus_condition + (1
| word) + (1 | Subject) + (1 | word_type)
##                    npar    AIC    BIC logLik devianc
e  Chisq Df Pr(>Chisq)
## m1                    5 199903 199943 -99947   19989
3
## casd.ctrl.dur.post   11 199835 199921 -99906   19981
3 80.464  6  2.866e-15 ***
## ---
## Signif. codes:  0 '***' 0.001 '**' 0.01 '*' 0.05 '.'
0.1 ' ' 1
```

Hide

```
emmeans(casd.ctrl.dur.post, pairwise~focus_condition)
```

```
## Note: D.f. calculations have been disabled because t
he number of observations exceeds 3000.
## To enable adjustments, add the argument 'pbkrtest.li
mit = 18603' (or larger)
```

```
## [or, globally, 'set emm_options(pbkrtest.limit = 186
03)' or larger];
## but be warned that this may result in large computat
ion time and memory use.
```

```
## Note: D.f. calculations have been disabled because t
he number of observations exceeds 3000.
## To enable adjustments, add the argument 'lmerTest.li
mit = 18603' (or larger)
## [or, globally, 'set emm_options(lmerTest.limit = 186
03)' or larger];
## but be warned that this may result in large computat
ion time and memory use.
```

```
## $emmeans
##  focus_condition        emmean   SE  df asymp.LCL as
ymp.UCL
##  Broad focus               209 14.7 Inf       180
238
##  Narrow pre_focus          201 14.7 Inf       172
229
##  Narrow on_focus           206 14.7 Inf       177
235
##  Narrow post_focus         203 14.7 Inf       174
232
##  Contrastive pre_focus     206 14.7 Inf       178
235
##  Contrastive on_focus      211 14.7 Inf       182
240
##  Contrastive post_focus    203 14.7 Inf       174
231
##
## Degrees-of-freedom method: asymptotic
## Confidence level used: 0.95
##
## $contrasts
##  contrast                                       esti
mate   SE  df z.ratio
##  Broad focus - Narrow pre_focus                    8
.113 1.48 Inf   5.486
##  Broad focus - Narrow on_focus                     2
.871 1.41 Inf   2.036
##  Broad focus - Narrow post_focus                   5
.940 1.48 Inf   4.008
##  Broad focus - Contrastive pre_focus               2
.469 1.48 Inf   1.666
##  Broad focus - Contrastive on_focus               -2
.165 1.41 Inf  -1.535
##  Broad focus - Contrastive post_focus              6
.251 1.48 Inf   4.217
##  Narrow pre_focus - Narrow on_focus               -5
.243 1.48 Inf  -3.544
##  Narrow pre_focus - Narrow post_focus             -2
```

```
.174 1.68 Inf  -1.295
##  Narrow pre_focus - Contrastive pre_focus          -5
.645 1.41 Inf  -4.006
##  Narrow pre_focus - Contrastive on_focus          -10
.278 1.48 Inf  -6.947
##  Narrow pre_focus - Contrastive post_focus         -1
.862 1.68 Inf  -1.110
##  Narrow on_focus - Narrow post_focus                3
.069 1.48 Inf   2.068
##  Narrow on_focus - Contrastive pre_focus           -0
.402 1.48 Inf  -0.271

##  Narrow on_focus - Contrastive on_focus            -5
.035 1.41 Inf  -3.567
##  Narrow on_focus - Contrastive post_focus           3
.381 1.48 Inf   2.277
##  Narrow post_focus - Contrastive pre_focus         -3
.471 1.68 Inf  -2.065
##  Narrow post_focus - Contrastive on_focus          -8
.105 1.49 Inf  -5.457
##  Narrow post_focus - Contrastive post_focus         0
.311 1.41 Inf   0.221
##  Contrastive pre_focus - Contrastive on_focus      -4
.634 1.48 Inf  -3.127
##  Contrastive pre_focus - Contrastive post_focus     3
.782 1.68 Inf   2.251
##  Contrastive on_focus - Contrastive post_focus      8
.416 1.49 Inf   5.666
##  p.value
##   <.0001
##   0.3918
##   0.0012
##   0.6386
##   0.7239
##   0.0005
##   0.0072
##   0.8545
##   0.0012
##   <.0001
##   0.9254
##   0.3720
##   1.0000
##   0.0067
##   0.2549
##   0.3735
##   <.0001
##   1.0000
##   0.0293
##   0.2685
##   <.0001
##
## Degrees-of-freedom method: asymptotic
## P value adjustment: tukey method for comparing a fam
ily of 7 estimates
```

0.3.2 mean f0

Hide

```
df <- filter(bob.mad, TrainingOrder == "Pre")

m1 <- lmer(F0 ~ 1  + (1|word) + (1|Subject) + (1|word_t
ype) , data = df, REML = FALSE)
casd.ctrl.f0.pre <- lmer(F0 ~  focus_condition + (1|wor
d) + (1|Subject)+ (1|word_type), data = df, REML = FALS
E)

anova(m1, casd.ctrl.f0.pre)
```

```
## Data: df
## Models:
## m1: F0 ~ 1 + (1 | word) + (1 | Subject) + (1 | word_
type)
## casd.ctrl.f0.pre: F0 ~ focus_condition + (1 | word)
+ (1 | Subject) + (1 | word_type)
##                  npar     AIC     BIC   logLik devia
nce  Chisq Df Pr(>Chisq)
## m1                  5 3385611 3385665 -1692801  3385
601
## casd.ctrl.f0.pre   11 3384671 3384790 -1692325  3384
649 951.92  6  < 2.2e-16
##
## m1
## casd.ctrl.f0.pre ***
## ---
## Signif. codes:  0 '***' 0.001 '**' 0.01 '*' 0.05 '.'
0.1 ' ' 1
```

Hide

```
emmeans(casd.ctrl.f0.pre, pairwise~focus_condition)
```

```
## Note: D.f. calculations have been disabled because t
he number of observations exceeds 3000.
## To enable adjustments, add the argument 'pbkrtest.li
mit = 362434' (or larger)
## [or, globally, 'set emm_options(pbkrtest.limit = 362
434)' or larger];
## but be warned that this may result in large computat
ion time and memory use.
```

```
## Note: D.f. calculations have been disabled because t
he number of observations exceeds 3000.
## To enable adjustments, add the argument 'lmerTest.li
mit = 362434' (or larger)
## [or, globally, 'set emm_options(lmerTest.limit = 362
434)' or larger];
## but be warned that this may result in large computat
ion time and memory use.
```

```
## $emmeans
##  focus_condition        emmean   SE  df asymp.LCL as
ymp.UCL
##  Broad focus               235 9.35 Inf       217
254
##  Narrow pre_focus          238 9.35 Inf       219
256
##  Narrow on_focus           235 9.35 Inf       217
254
##  Narrow post_focus         233 9.35 Inf       215
252
##  Contrastive pre_focus     239 9.35 Inf       220
257
##  Contrastive on_focus      236 9.35 Inf       217
254
##  Contrastive post_focus    234 9.35 Inf       216
253
##
## Degrees-of-freedom method: asymptotic
## Confidence level used: 0.95
##
## $contrasts
##  contrast                                       esti
mate    SE  df z.ratio
##  Broad focus - Narrow pre_focus                   -2.
3210 0.168 Inf -13.796
##  Broad focus - Narrow on_focus                    -0.
0238 0.160 Inf  -0.149
##  Broad focus - Narrow post_focus                   2.
1187 0.169 Inf  12.560
##  Broad focus - Contrastive pre_focus              -3.
3294 0.168 Inf -19.809
##  Broad focus - Contrastive on_focus               -0.
1911 0.160 Inf  -1.194
##  Broad focus - Contrastive post_focus              1.
0808 0.169 Inf   6.411
##  Narrow pre_focus - Narrow on_focus                2.
2972 0.168 Inf  13.654
##  Narrow pre_focus - Narrow post_focus              4.
```

```
##  Narrow pre_focus - Narrow post_focus            [illegible]
4397 0.191 Inf  23.239

##  Narrow pre_focus - Contrastive pre_focus        -1.
0084 0.160 Inf  -6.309
##  Narrow pre_focus - Contrastive on_focus          2.
1299 0.168 Inf  12.657
##  Narrow pre_focus - Contrastive post_focus        3.
4019 0.191 Inf  17.811
##  Narrow on_focus - Narrow post_focus              2.
1425 0.169 Inf  12.698
##  Narrow on_focus - Contrastive pre_focus         -3.
3056 0.168 Inf -19.667
##  Narrow on_focus - Contrastive on_focus          -0.
1673 0.160 Inf  -1.045
##  Narrow on_focus - Contrastive post_focus         1.
1047 0.169 Inf   6.551
##  Narrow post_focus - Contrastive pre_focus       -5.
4481 0.191 Inf -28.542
##  Narrow post_focus - Contrastive on_focus        -2.
3098 0.169 Inf -13.704
##  Narrow post_focus - Contrastive post_focus      -1.
0378 0.161 Inf  -6.454
##  Contrastive pre_focus - Contrastive on_focus     3.
1383 0.168 Inf  18.668
##  Contrastive pre_focus - Contrastive post_focus   4.
4103 0.191 Inf  23.110
##  Contrastive on_focus - Contrastive post_focus    1.
2720 0.168 Inf   7.551
##  p.value
##   <.0001
##   1.0000
##   <.0001
##   <.0001
##   0.8966
##   <.0001
##   <.0001
##   <.0001
##   <.0001
##   <.0001
##   <.0001
##   <.0001
##   <.0001
##   0.9434
##   <.0001
##   <.0001
##   <.0001
##   <.0001
##   <.0001
##   <.0001
##   <.0001
##
## Degrees-of-freedom method: asymptotic
## P value adjustment: tukey method for comparing a fam
ily of 7 estimates
```

Hide

```
df <- filter(bob.mad, TrainingOrder == "Post")

m1 <- lmer(F0 ~ 1  + (1|word) + (1|Subject) + (1|word_t
ype), data = df, REML = FALSE)
casd.ctrl.f0.post <- lmer(F0 ~  focus_condition + (1|wo
rd) + (1|Subject)+ (1|word_type) , data = df, REML = FA
LSE)

anova(m1, casd.ctrl.f0.post)
```

```
## Data: df
## Models:
## m1: F0 ~ 1 + (1 | word) + (1 | Subject) + (1 | word_
type)
## casd.ctrl.f0.post: F0 ~ focus_condition + (1 | word)
+ (1 | Subject) + (1 | word_type)
##                   npar     AIC     BIC   logLik devi
ance  Chisq Df Pr(>Chisq)
## m1                   5 3442829 3442883 -1721409  344
2819
## casd.ctrl.f0.post   11 3442451 3442569 -1721214  344
2429 390.04  6  < 2.2e-16
##
## m1
## casd.ctrl.f0.post ***
## ---
## Signif. codes:  0 '***' 0.001 '**' 0.01 '*' 0.05 '.'
0.1 ' ' 1
```

Hide

```
emmeans(casd.ctrl.f0.post, pairwise~focus_condition)
```

```
## Note: D.f. calculations have been disabled because t
he number of observations exceeds 3000.
## To enable adjustments, add the argument 'pbkrtest.li
mit = 363059' (or larger)
## [or, globally, 'set emm_options(pbkrtest.limit = 363
059)' or larger];
## but be warned that this may result in large computat
ion time and memory use.
```

```
## Note: D.f. calculations have been disabled because t
he number of observations exceeds 3000.
## To enable adjustments, add the argument 'lmerTest.li
mit = 363059' (or larger)
```

```
mit = 363059' (or larger)
## [or, globally, 'set emm_options(lmerTest.limit = 363
059)' or larger];
## but be warned that this may result in large computat
ion time and memory use.
```

```
## $emmeans
##  focus_condition        emmean   SE  df asymp.LCL as
ymp.UCL
##  Broad focus               238 10.4 Inf       218
258
##  Narrow pre_focus          239 10.4 Inf       219
259
##  Narrow on_focus           238 10.4 Inf       218
259
##  Narrow post_focus         237 10.4 Inf       216
257
##  Contrastive pre_focus     240 10.4 Inf       220
261
##  Contrastive on_focus      238 10.4 Inf       218
259
##  Contrastive post_focus    237 10.4 Inf       216
257
##
## Degrees-of-freedom method: asymptotic
## Confidence level used: 0.95
##
## $contrasts
##  contrast                                       esti
mate    SE  df z.ratio
##  Broad focus - Narrow pre_focus                  -1.
0616 0.180 Inf  -5.882
##  Broad focus - Narrow on_focus                   -0.
2346 0.172 Inf  -1.365
##  Broad focus - Narrow post_focus                  1.
4313 0.181 Inf   7.925
##  Broad focus - Contrastive pre_focus             -2.
1678 0.181 Inf -12.007
##  Broad focus - Contrastive on_focus              -0.
2843 0.172 Inf  -1.653
##  Broad focus - Contrastive post_focus             1.
4715 0.181 Inf   8.143
##  Narrow pre_focus - Narrow on_focus               0.
8269 0.181 Inf   4.575
##  Narrow pre_focus - Narrow post_focus             2.
4928 0.205 Inf  12.161

##  Narrow pre_focus - Contrastive pre_focus        -1.
1062 0.172 Inf  -6.449
##  Narrow pre_focus - Contrastive on_focus          0.
7773 0.181 Inf   4.295
##  Narrow pre_focus - Contrastive post_focus        2.
5331 0.205 Inf  12.350
##  Narrow on_focus - Narrow post_focus              1.
6659 0.181 Inf   9.207
```

```
6659 0.181 Inf   9.207
##  Narrow on_focus - Contrastive pre_focus         -1.
9331 0.181 Inf -10.691
##  Narrow on_focus - Contrastive on_focus          -0.
0496 0.172 Inf  -0.288
##  Narrow on_focus - Contrastive post_focus         1.
7061 0.181 Inf   9.423
##  Narrow post_focus - Contrastive pre_focus       -3.
5990 0.205 Inf -17.552
##  Narrow post_focus - Contrastive on_focus        -1.
7155 0.181 Inf  -9.482
##  Narrow post_focus - Contrastive post_focus       0.
0402 0.172 Inf   0.234
##  Contrastive pre_focus - Contrastive on_focus     1.
8835 0.181 Inf  10.404
##  Contrastive pre_focus - Contrastive post_focus   3.
6393 0.205 Inf  17.738
##  Contrastive on_focus - Contrastive post_focus    1.
7558 0.181 Inf   9.698
##  p.value
##   <.0001
##   0.8203
##   <.0001
##   <.0001
##   0.6474
##   <.0001
##   0.0001
##   <.0001
##   <.0001
##   0.0004
##   <.0001
##   <.0001
##   <.0001
##   1.0000
##   <.0001
##   <.0001
##   <.0001
##   1.0000
##   <.0001
##   <.0001
##   <.0001
##
## Degrees-of-freedom method: asymptotic
## P value adjustment: tukey method for comparing a fam
ily of 7 estimates
```

0.3.3 mean intensity

Hide

```
df <- filter(bob.mad, TrainingOrder == "Pre")

m1 <- lmer(Intensity ~ 1  + (1|word) + (1|Subject) + (1
|word_type) + (1|Block), data = df, REML = FALSE)
```

```
casd.ctrl.int.pre <- lmer(Intensity ~  focus_condition
+ (1|word) + (1|Subject)+ (1|word_type) + (1|Block), da
ta = df, REML = FALSE)

anova(m1, casd.ctrl.int.pre)
```

```
## Data: df
## Models:
## m1: Intensity ~ 1 + (1 | word) + (1 | Subject) + (1
| word_type) + (1 | Block)
## casd.ctrl.int.pre: Intensity ~ focus_condition + (1
| word) + (1 | Subject) + (1 | word_type) + (1 | Block)
##                   npar     AIC     BIC   logLik devi
ance  Chisq Df Pr(>Chisq)
## m1                   6 2374293 2374358 -1187141  237
4281
## casd.ctrl.int.pre   12 2373970 2374100 -1186973  237
3946 335.36  6  < 2.2e-16
##
## m1
## casd.ctrl.int.pre ***
## ---
## Signif. codes:  0 '***' 0.001 '**' 0.01 '*' 0.05 '.'
0.1 ' ' 1
```

Hide

```
emmeans(casd.ctrl.int.pre, pairwise~focus_condition)
```

```
## Note: D.f. calculations have been disabled because t
he number of observations exceeds 3000.
## To enable adjustments, add the argument 'pbkrtest.li
mit = 376064' (or larger)
## [or, globally, 'set emm_options(pbkrtest.limit = 376
064)' or larger];
## but be warned that this may result in large computat
ion time and memory use.
```

```
## Note: D.f. calculations have been disabled because t
he number of observations exceeds 3000.
## To enable adjustments, add the argument 'lmerTest.li
mit = 376064' (or larger)
## [or, globally, 'set emm_options(lmerTest.limit = 376
064)' or larger];
## but be warned that this may result in large computat
ion time and memory use.
```

```
## $emmeans
##  focus_condition        emmean   SE  df asymp.LCL as
ymp.UCL
##  Broad focus              62.5 1.66 Inf      59.3
```

```
##  Broad focus              62.5 1.66 Inf      59.3
65.7
##  Narrow pre_focus         62.7 1.66 Inf      59.4
65.9
##  Narrow on_focus          62.9 1.66 Inf      59.7
66.2
##  Narrow post_focus        62.6 1.66 Inf      59.3
65.8
##  Contrastive pre_focus    62.4 1.66 Inf      59.1
65.6
##  Contrastive on_focus     62.7 1.66 Inf      59.5
66.0
##  Contrastive post_focus   62.7 1.66 Inf      59.4
65.9
##
## Degrees-of-freedom method: asymptotic
## Confidence level used: 0.95
##
## $contrasts
##  contrast                                              esti
mate     SE  df z.ratio
##  Broad focus - Narrow pre_focus                         -0.
1840 0.0364 Inf  -5.053
##  Broad focus - Narrow on_focus                          -0.
4436 0.0346 Inf -12.805
##  Broad focus - Narrow post_focus                        -0.
0716 0.0365 Inf  -1.965
##  Broad focus - Contrastive pre_focus                     0.
1392 0.0364 Inf   3.826
##  Broad focus - Contrastive on_focus                     -0.
2314 0.0346 Inf  -6.684
##  Broad focus - Contrastive post_focus                   -0.
1522 0.0365 Inf  -4.173
##  Narrow pre_focus - Narrow on_focus                     -0.
2596 0.0364 Inf  -7.131
##  Narrow pre_focus - Narrow post_focus                    0.
1124 0.0413 Inf   2.720

##  Narrow pre_focus - Contrastive pre_focus                0.
3232 0.0346 Inf   9.337
##  Narrow pre_focus - Contrastive on_focus                -0.
0474 0.0364 Inf  -1.303
##  Narrow pre_focus - Contrastive post_focus               0.
0319 0.0413 Inf   0.771
##  Narrow on_focus - Narrow post_focus                     0.
3720 0.0364 Inf  10.207
##  Narrow on_focus - Contrastive pre_focus                 0.
5828 0.0364 Inf  16.024
##  Narrow on_focus - Contrastive on_focus                  0.
2122 0.0346 Inf   6.130
##  Narrow on_focus - Contrastive post_focus                0.
2915 0.0364 Inf   7.999
##  Narrow post_focus - Contrastive pre_focus               0.
2109 0.0413 Inf   5.109
##  Narrow post_focus - Contrastive on_focus               -0.
```

```
1598 0.0364 Inf  -4.387
##  Narrow post_focus - Contrastive post_focus      -0.
0805 0.0347 Inf  -2.320
##  Contrastive pre_focus - Contrastive on_focus    -0.
3707 0.0364 Inf -10.194
##  Contrastive pre_focus - Contrastive post_focus  -0.
2914 0.0413 Inf  -7.059
##  Contrastive on_focus - Contrastive post_focus    0.
0793 0.0364 Inf   2.177
##  p.value
##   <.0001
##   <.0001
##   0.4373
##   0.0025
##   <.0001
##   0.0006
##   <.0001
##   0.0932
##   <.0001
##   0.8507
##   0.9876
##   <.0001
##   <.0001
##   <.0001
##   <.0001
##   <.0001
##   0.0002
##   0.2342
##   <.0001
##   <.0001
##   0.3079
##
## Degrees-of-freedom method: asymptotic
## P value adjustment: tukey method for comparing a fam
ily of 7 estimates
```

Hide

```
df <- filter(bob.mad, TrainingOrder == "Post")

m1 <- lmer(Intensity ~ 1  + (1|word) + (1|Subject) + (1
|word_type) + (1|Block), data = df, REML = FALSE)
casd.ctrl.int.post <- lmer(Intensity ~  focus_condition
+ (1|word) + (1|Subject)+ (1|word_type) + (1|Block), da
ta = df, REML = FALSE)

anova(m1, casd.ctrl.int.post)
```

```
## Data: df
## Models:
## m1: Intensity ~ 1 + (1 | word) + (1 | Subject) + (1
| word_type) + (1 | Block)
## casd.ctrl.int.post: Intensity ~ focus_condition + (1
[illegible]
```

```
| word) + (1 | Subject) + (1 | word_type) + (1 | Block)
##                          npar     AIC     BIC   logLik dev
iance Chisq Df Pr(>Chisq)
## m1                         6 2290717 2290782 -1145353  22
90705
## casd.ctrl.int.post   12 2290520 2290650 -1145248  22
90496 209.4  6  < 2.2e-16
##
## m1
## casd.ctrl.int.post ***
## ---
## Signif. codes:  0 '***' 0.001 '**' 0.01 '*' 0.05 '.'
0.1 ' ' 1
```

Hide

```
emmeans(casd.ctrl.int.post, pairwise~focus_condition)
```

```
## Note: D.f. calculations have been disabled because t
he number of observations exceeds 3000.
## To enable adjustments, add the argument 'pbkrtest.li
mit = 367753' (or larger)
## [or, globally, 'set emm_options(pbkrtest.limit = 367
753)' or larger];
## but be warned that this may result in large computat
ion time and memory use.
```

```
## Note: D.f. calculations have been disabled because t
he number of observations exceeds 3000.
## To enable adjustments, add the argument 'lmerTest.li
mit = 367753' (or larger)
## [or, globally, 'set emm_options(lmerTest.limit = 367
753)' or larger];
## but be warned that this may result in large computat
ion time and memory use.
```

```
## $emmeans
##  focus_condition        emmean   SE  df asymp.LCL as
ymp.UCL
##  Broad focus              60.8 1.91 Inf      57.1
64.6
##  Narrow pre_focus         60.8 1.91 Inf      57.0
64.5
##  Narrow on_focus          60.8 1.91 Inf      57.0
64.5
##  Narrow post_focus        60.4 1.91 Inf      56.7
64.2
##  Contrastive pre_focus    60.7 1.91 Inf      56.9
64.4
```

```
##  Contrastive on_focus     60.9 1.91 Inf      57.1
64.6
##  Contrastive post_focus   60.5 1.91 Inf      56.8
64.3
##
## Degrees-of-freedom method: asymptotic
## Confidence level used: 0.95
##
## $contrasts
##  contrast                                          esti
mate     SE  df z.ratio
##  Broad focus - Narrow pre_focus                     0.0
4958 0.0353 Inf   1.405
##  Broad focus - Narrow on_focus                      0.0
5519 0.0336 Inf   1.645
##  Broad focus - Narrow post_focus                    0.3
8535 0.0353 Inf  10.931
##  Broad focus - Contrastive pre_focus                0.1
3401 0.0353 Inf   3.798
##  Broad focus - Contrastive on_focus                -0.0
3961 0.0336 Inf  -1.180
##  Broad focus - Contrastive post_focus               0.2
7743 0.0353 Inf   7.865
##  Narrow pre_focus - Narrow on_focus                 0.0
0562 0.0353 Inf   0.159
##  Narrow pre_focus - Narrow post_focus               0.3
3578 0.0400 Inf   8.385

##  Narrow pre_focus - Contrastive pre_focus           0.0
8443 0.0336 Inf   2.511
##  Narrow pre_focus - Contrastive on_focus           -0.0
8918 0.0353 Inf  -2.523
##  Narrow pre_focus - Contrastive post_focus          0.2
2785 0.0401 Inf   5.688
##  Narrow on_focus - Narrow post_focus                0.3
3016 0.0353 Inf   9.350
##  Narrow on_focus - Contrastive pre_focus            0.0
7881 0.0353 Inf   2.231
##  Narrow on_focus - Contrastive on_focus            -0.0
9480 0.0336 Inf  -2.820
##  Narrow on_focus - Contrastive post_focus           0.2
2223 0.0353 Inf   6.290
##  Narrow post_focus - Contrastive pre_focus         -0.2
5135 0.0400 Inf  -6.278
##  Narrow post_focus - Contrastive on_focus          -0.4
2496 0.0353 Inf -12.034
##  Narrow post_focus - Contrastive post_focus        -0.1
0793 0.0336 Inf  -3.216
##  Contrastive pre_focus - Contrastive on_focus      -0.1
7361 0.0353 Inf  -4.913
##  Contrastive pre_focus - Contrastive post_focus  0.1
4342 0.0401 Inf   3.581
##  Contrastive on_focus - Contrastive post_focus   0.3
1703 0.0353 Inf   8.973
##  p.value
```

```
##   0.7995
##   0.6530
##   <.0001
##   0.0028
##   0.9018
##   <.0001
##   1.0000
##   <.0001
##   0.1553
##   0.1508
##   <.0001
##   <.0001
##   0.2787
##   0.0716
##   <.0001
##   <.0001
##   <.0001
##   0.0221
##   <.0001
##   0.0063
##   <.0001
##
## Degrees-of-freedom method: asymptotic
## P value adjustment: tukey method for comparing a fam
ily of 7 estimates
```

0.4 CTD by focus condition

0.4.1 mean duration

Hide

```
df <- bob.mad

m1 <- lmer(Duration ~ 1  + (1|word) + (1|Subject) + (1|
word_type) + (1|Block), data = df, REML = FALSE)
ctd.dur <- lmer(Duration ~  focus_condition + (1|word)
+ (1|Subject)+ (1|word_type) + (1|Block), data = df, RE
ML = FALSE)

anova(m1, ctd.dur)
```

```
## Data: df
## Models:
## m1: Duration ~ 1 + (1 | word) + (1 | Subject) + (1 |
word_type) + (1 | Block)
## ctd.dur: Duration ~ focus_condition + (1 | word) + (
1 | Subject) + (1 | word_type) + (1 | Block)
##         npar    AIC    BIC  logLik deviance  Chisq D
f Pr(>Chisq)
## m1         6 259264 259313 -129626   259252
## ctd.dur   12 258975 259072 -129475   258951 301.18
```

```
[illegible]
6  < 2.2e-16 ***
## ---
## Signif. codes:  0 '***' 0.001 '**' 0.01 '*' 0.05 '.'
0.1 ' ' 1
```

Hide

```
emmeans(ctd.dur, pairwise~focus_condition)
```

```
## Note: D.f. calculations have been disabled because t
he number of observations exceeds 3000.
## To enable adjustments, add the argument 'pbkrtest.li
mit = 23711' (or larger)
## [or, globally, 'set emm_options(pbkrtest.limit = 237
11)' or larger];
## but be warned that this may result in large computat
ion time and memory use.
```

```
## Note: D.f. calculations have been disabled because t
he number of observations exceeds 3000.
## To enable adjustments, add the argument 'lmerTest.li
mit = 23711' (or larger)
## [or, globally, 'set emm_options(lmerTest.limit = 237
11)' or larger];
## but be warned that this may result in large computat
ion time and memory use.
```

```
## $emmeans
##  focus_condition        emmean   SE  df asymp.LCL as
ymp.UCL
##  Broad focus               219 11.2 Inf       197
241
##  Narrow pre_focus          206 11.2 Inf       184
228
##  Narrow on_focus           215 11.2 Inf       193
237
##  Narrow post_focus         206 11.2 Inf       185
228
##  Contrastive pre_focus     220 11.2 Inf       198
242
##  Contrastive on_focus      224 11.2 Inf       202
246
##  Contrastive post_focus    211 11.2 Inf       189
233
##
## Degrees-of-freedom method: asymptotic
## Confidence level used: 0.95
##
## $contrasts
##  contrast                                         esti
```

```
mate   SE  df z.ratio
##  Broad focus - Narrow pre_focus                      12
.816 1.44 Inf   8.888
##  Broad focus - Narrow on_focus                        4
.134 1.37 Inf   3.018
##  Broad focus - Narrow post_focus                     12
.705 1.44 Inf   8.831
##  Broad focus - Contrastive pre_focus                 -0
.752 1.45 Inf  -0.520
##  Broad focus - Contrastive on_focus                  -5
.224 1.37 Inf  -3.814
##  Broad focus - Contrastive post_focus                 8
.229 1.44 Inf   5.721
##  Narrow pre_focus - Narrow on_focus                  -8
.683 1.44 Inf  -6.039
##  Narrow pre_focus - Narrow post_focus                -0
.111 1.63 Inf  -0.068

##  Narrow pre_focus - Contrastive pre_focus           -13
.568 1.37 Inf  -9.872
##  Narrow pre_focus - Contrastive on_focus            -18
.041 1.44 Inf -12.543
##  Narrow pre_focus - Contrastive post_focus           -4
.587 1.63 Inf  -2.815
##  Narrow on_focus - Narrow post_focus                  8
.571 1.44 Inf   5.969
##  Narrow on_focus - Contrastive pre_focus             -4
.886 1.44 Inf  -3.387
##  Narrow on_focus - Contrastive on_focus              -9
.358 1.37 Inf  -6.850
##  Narrow on_focus - Contrastive post_focus             4
.095 1.44 Inf   2.852
##  Narrow post_focus - Contrastive pre_focus          -13
.457 1.63 Inf  -8.233
##  Narrow post_focus - Contrastive on_focus           -17
.930 1.44 Inf -12.484
##  Narrow post_focus - Contrastive post_focus          -4
.476 1.37 Inf  -3.277
##  Contrastive pre_focus - Contrastive on_focus        -4
.472 1.44 Inf  -3.099
##  Contrastive pre_focus - Contrastive post_focus       8
.981 1.63 Inf   5.495
##  Contrastive on_focus - Contrastive post_focus       13
.453 1.44 Inf   9.369
##  p.value
##   <.0001
##   0.0407
##   <.0001
##   0.9986
##   0.0026
##   <.0001
##   <.0001
##   1.0000
##   <.0001
##   <.0001
```

```
##   <.0001
##   0.0726
##   <.0001
##   0.0125
##   <.0001
##   0.0656
##   <.0001
##   <.0001
##   0.0181
##   0.0319
##   <.0001
##   <.0001
##
## Degrees-of-freedom method: asymptotic
## P value adjustment: tukey method for comparing a fam
ily of 7 estimates
```

0.4.2 mean f0

Hide

```
df <- bob.mad

m1 <- lmer(F0 ~ 1  + (1|word) + (1|Subject) + (1|word_t
ype) + (1|Block), data = df, REML = FALSE)
ctd.f0 <- lmer(F0 ~  focus_condition + (1|word) + (1|Su
bject)+ (1|word_type) + (1|Block), data = df, REML = FA
LSE)

anova(m1, ctd.f0)
```

```
## Data: df
## Models:
## m1: F0 ~ 1 + (1 | word) + (1 | Subject) + (1 | word_
type) + (1 | Block)
## ctd.f0: F0 ~ focus_condition + (1 | word) + (1 | Sub
ject) + (1 | word_type) + (1 | Block)
##        npar     AIC     BIC   logLik deviance  Chisq
Df Pr(>Chisq)
## m1        6 3548984 3549049 -1774486  3548972
## ctd.f0   12 3547443 3547573 -1773709  3547419 1553.2
6  < 2.2e-16 ***
## ---
## Signif. codes:  0 '***' 0.001 '**' 0.01 '*' 0.05 '.'
0.1 ' ' 1
```

Hide

```
emmeans(ctd.f0, pairwise~focus_condition)
```

```
## Note: D.f. calculations have been disabled because t
he number of observations exceeds 3000.
[illegible]
```

```
## To enable adjustments, add the argument 'pbkrtest.li
mit = 387465' (or larger)
## [or, globally, 'set emm_options(pbkrtest.limit = 387
465)' or larger];
## but be warned that this may result in large computat
ion time and memory use.
```

```
## Note: D.f. calculations have been disabled because t
he number of observations exceeds 3000.
## To enable adjustments, add the argument 'lmerTest.li
mit = 387465' (or larger)
## [or, globally, 'set emm_options(lmerTest.limit = 387
465)' or larger];
## but be warned that this may result in large computat
ion time and memory use.
```

```
## $emmeans
##  focus_condition        emmean  SE  df asymp.LCL asy
mp.UCL
##  Broad focus               223 7.8 Inf       207
238
##  Narrow pre_focus          225 7.8 Inf       210
240
##  Narrow on_focus           222 7.8 Inf       207
237
##  Narrow post_focus         221 7.8 Inf       206
236
##  Contrastive pre_focus     226 7.8 Inf       211
242
##  Contrastive on_focus      224 7.8 Inf       209
239
##  Contrastive post_focus    221 7.8 Inf       206
237
##
## Degrees-of-freedom method: asymptotic
## Confidence level used: 0.95
##
## $contrasts
##  contrast                                       esti
mate    SE  df z.ratio
##  Broad focus - Narrow pre_focus                   -2
.123 0.149 Inf -14.292
##  Broad focus - Narrow on_focus                     0
.847 0.142 Inf   5.987
##  Broad focus - Narrow post_focus                   1
.909 0.149 Inf  12.811
##  Broad focus - Contrastive pre_focus              -3
.736 0.148 Inf -25.189
##  Broad focus - Contrastive on_focus               -1
```

```
##  Broad focus - Contrastive on_focus                -1
.258 0.141 Inf  -8.903
##  Broad focus - Contrastive post_focus               1
.409 0.149 Inf   9.449
##  Narrow pre_focus - Narrow on_focus                 2
.970 0.149 Inf  19.995
##  Narrow pre_focus - Narrow post_focus               4
.031 0.169 Inf  23.906

##  Narrow pre_focus - Contrastive pre_focus          -1
.613 0.141 Inf -11.450
##  Narrow pre_focus - Contrastive on_focus            0
.865 0.148 Inf   5.831
##  Narrow pre_focus - Contrastive post_focus          3
.531 0.169 Inf  20.929
##  Narrow on_focus - Narrow post_focus                1
.061 0.149 Inf   7.126
##  Narrow on_focus - Contrastive pre_focus           -4
.583 0.148 Inf -30.899
##  Narrow on_focus - Contrastive on_focus            -2
.105 0.141 Inf -14.900
##  Narrow on_focus - Contrastive post_focus           0
.561 0.149 Inf   3.767
##  Narrow post_focus - Contrastive pre_focus         -5
.645 0.168 Inf -33.508
##  Narrow post_focus - Contrastive on_focus          -3
.167 0.149 Inf -21.282
##  Narrow post_focus - Contrastive post_focus        -0
.500 0.142 Inf  -3.523
##  Contrastive pre_focus - Contrastive on_focus       2
.478 0.148 Inf  16.735
##  Contrastive pre_focus - Contrastive post_focus     5
.145 0.169 Inf  30.523
##  Contrastive on_focus - Contrastive post_focus      2
.667 0.149 Inf  17.910
##  p.value
##   <.0001
##   <.0001
##   <.0001
##   <.0001
##   <.0001
##   <.0001
##   <.0001
##   <.0001
##   <.0001
##   <.0001
##   <.0001
##   <.0001
##   <.0001
##   <.0001
##   0.0031
##   <.0001
##   <.0001
##   0.0078
##   <.0001
```

```
##   <.0001
##   <.0001
##
## Degrees-of-freedom method: asymptotic
## P value adjustment: tukey method for comparing a fam
ily of 7 estimates
```

0.4.3 mean intensity

Hide

```
df <- bob.mad

m1 <- lmer(Intensity ~ 1  + (1|word) + (1|Subject) + (1
|word_type) + (1|Block), data = df, REML = FALSE)
ctd.int <- lmer(Intensity ~  focus_condition + (1|word)
+ (1|Subject)+ (1|word_type) + (1|Block), data = df, RE
ML = FALSE)

anova(m1, ctd.int)
```

```
## Data: df
## Models:
## m1: Intensity ~ 1 + (1 | word) + (1 | Subject) + (1
| word_type) + (1 | Block)
## ctd.int: Intensity ~ focus_condition + (1 | word) +
(1 | Subject) + (1 | word_type) + (1 | Block)
##         npar     AIC     BIC   logLik deviance  Chis
q Df Pr(>Chisq)
## m1         6 2399015 2399080 -1199501  2399003
## ctd.int   12 2397834 2397965 -1198905  2397810 1193.
3  6  < 2.2e-16 ***
## ---
## Signif. codes:  0 '***' 0.001 '**' 0.01 '*' 0.05 '.'
0.1 ' ' 1
```

Hide

```
emmeans(ctd.int, pairwise~focus_condition)
```

```
## Note: D.f. calculations have been disabled because t
he number of observations exceeds 3000.
## To enable adjustments, add the argument 'pbkrtest.li
mit = 405670' (or larger)
## [or, globally, 'set emm_options(pbkrtest.limit = 405
670)' or larger];
## but be warned that this may result in large computat
ion time and memory use.
```

```
## Note: D.f. calculations have been disabled because t
he number of observations exceeds 3000.
## To enable adjustments, add the argument 'lmerTest.li
mit = 405670' (or larger)
## [or, globally, 'set emm_options(lmerTest.limit = 405
670)' or larger];
## but be warned that this may result in large computat
ion time and memory use.
```

```
## $emmeans
##  focus_condition        emmean  SE  df asymp.LCL asy
mp.UCL
##  Broad focus              59.4 2.1 Inf      55.3
63.6
##  Narrow pre_focus         59.4 2.1 Inf      55.3
63.5
##  Narrow on_focus          59.4 2.1 Inf      55.2
63.5
##  Narrow post_focus        58.9 2.1 Inf      54.7
63.0
##  Contrastive pre_focus    59.3 2.1 Inf      55.2
63.4
##  Contrastive on_focus     59.5 2.1 Inf      55.4
63.6
##  Contrastive post_focus   58.7 2.1 Inf      54.6
62.8
##
## Degrees-of-freedom method: asymptotic
## Confidence level used: 0.95
##
## $contrasts
##  contrast                                       esti
mate     SE  df z.ratio
##  Broad focus - Narrow pre_focus                   0.
0546 0.0286 Inf   1.905
##  Broad focus - Narrow on_focus                    0.
0691 0.0273 Inf   2.534
##  Broad focus - Narrow post_focus                  0.
5784 0.0287 Inf  20.135
##  Broad focus - Contrastive pre_focus              0.
1493 0.0287 Inf   5.209
##  Broad focus - Contrastive on_focus              -0.
0912 0.0273 Inf  -3.343
##  Broad focus - Contrastive post_focus             0.
7261 0.0287 Inf  25.277
##  Narrow pre_focus - Narrow on_focus               0.
0146 0.0286 Inf   0.508
##  Narrow pre_focus - Narrow post_focus             0.
5238 0.0325 Inf  16.107

##  Narrow pre_focus - Contrastive pre_focus         0.
[illegible]
```

```
0947 0.0272 Inf   3.479
##  Narrow pre_focus - Contrastive on_focus         -0.
1458 0.0286 Inf  -5.090
##  Narrow pre_focus - Contrastive post_focus        0.
6715 0.0325 Inf  20.651
##  Narrow on_focus - Narrow post_focus              0.
5093 0.0287 Inf  17.731
##  Narrow on_focus - Contrastive pre_focus          0.
0801 0.0286 Inf   2.797
##  Narrow on_focus - Contrastive on_focus          -0.
1603 0.0273 Inf  -5.878
##  Narrow on_focus - Contrastive post_focus         0.
6570 0.0287 Inf  22.874
##  Narrow post_focus - Contrastive pre_focus       -0.
4292 0.0325 Inf -13.191
##  Narrow post_focus - Contrastive on_focus        -0.
6696 0.0287 Inf -23.317
##  Narrow post_focus - Contrastive post_focus       0.
1477 0.0274 Inf   5.397
##  Contrastive pre_focus - Contrastive on_focus    -0.
2404 0.0286 Inf  -8.393
##  Contrastive pre_focus - Contrastive post_focus   0.
5768 0.0325 Inf  17.733
##  Contrastive on_focus - Contrastive post_focus    0.
8173 0.0287 Inf  28.462
##  p.value
##   0.4766
##   0.1473
##   <.0001
##   <.0001
##   0.0146
##   <.0001
##   0.9988
##   <.0001
##   0.0091
##   <.0001
##   <.0001
##   <.0001
##   0.0762
##   <.0001
##   <.0001
##   <.0001
##   <.0001
##   <.0001
##   <.0001
##   <.0001
##   <.0001
##
## Degrees-of-freedom method: asymptotic
## P value adjustment: tukey method for comparing a fam
ily of 7 estimates
```

1 Plots

CASD_Control_duration_clean_add

Subject	Block	TrainingType	TrainingOrder	word	word_type	focus_type	repetition	Duration	SyllablePosition	syllable	syll_focus	tone	focus_condition
2023404	block1	Control	post	zoeng1	Subject	Narrow	r1	333.3816421	1	1	pre_focus	1	Narrow pre_focus
2023404	block1	Control	post	saang1	Subject	Narrow	r1	358.5138324	2	2	pre_focus	1	Narrow pre_focus
2023404	block1	Control	post	tsa1	Verb	Narrow	r1	332.9849516	3	1	pre_focus	1	Narrow pre_focus
2023404	block1	Control	post	fei1	Object	Narrow	r1	446.7751323	4	1	on_focus	1	Narrow on_focus
2023404	block1	Control	post	gei1	Object	Narrow	r1	263.3381065	5	2	on_focus	1	Narrow on_focus
2023404	block1	Control	post	sau3	Subject	Contrastive	r1	268.0412898	1	1	pre_focus	3	Contrastive pre_focus
2023404	block1	Control	post	sau3	Subject	Contrastive	r1	295.9718941	2	2	pre_focus	3	Contrastive pre_focus
2023404	block1	Control	post	sik3	Verb	Contrastive	r1	126.6803083	3	1	pre_focus	3	Contrastive pre_focus
2023404	block1	Control	post	baak3	Object	Contrastive	r1	181.9068015	4	1	on_focus	3	Contrastive on_focus
2023404	block1	Control	post	baak3	Object	Contrastive	r1	166.0652166	5	2	on_focus	3	Contrastive on_focus
2023404	block1	Control	post	jyun2	Subject	Contrastive	r1	322.8397451	1	1	pre_focus	2	Contrastive pre_focus
2023404	block1	Control	post	jyun2	Subject	Contrastive	r1	323.7900554	2	2	pre_focus	2	Contrastive pre_focus
2023404	block1	Control	post	mo2	Verb	Contrastive	r1	343.0131244	3	1	pre_focus	2	Contrastive pre_focus
2023404	block1	Control	post	gau2	Object	Contrastive	r1	416.9533258	4	1	on_focus	2	Contrastive on_focus
2023404	block1	Control	post	zai2	Object	Contrastive	r1	347.564059	5	2	on_focus	2	Contrastive on_focus
2023404	block1	Control	post	jyun2	Subject	Narrow	r1	313.8123583	1	1	on_focus	2	Narrow on_focus
2023404	block1	Control	post	jyun2	Subject	Narrow	r1	299.3862377	2	2	on_focus	2	Narrow on_focus
2023404	block1	Control	post	mo2	Verb	Narrow	r1	383.1867268	3	1	post_focus	2	Narrow post_focus
2023404	block1	Control	post	gau2	Object	Narrow	r1	412.2110147	4	1	post_focus	2	Narrow post_focus
2023404	block1	Control	post	zai2	Object	Narrow	r1	331.5073725	5	2	post_focus	2	Narrow post_focus
2023404	block1	Control	post	jyun2	Subject	Broad	r1	315.734279	1	1	broad_focus	2	Broad focus
2023404	block1	Control	post	jyun2	Subject	Broad	r1	209.7438741	2	2	broad_focus	2	Broad focus
2023404	block1	Control	post	mo2	Verb	Broad	r1	368.6959943	3	1	broad_focus	2	Broad focus
2023404	block1	Control	post	gau2	Object	Broad	r1	386.0946756	4	1	broad_focus	2	Broad focus
2023404	block1	Control	post	zai2	Object	Broad	r1	355.5677013	5	2	broad_focus	2	Broad focus
2023404	block1	Control	post	sau3	Subject	Contrastive	r1	304.3499569	1	1	pre_focus	3	Contrastive pre_focus
2023404	block1	Control	post	sau3	Subject	Contrastive	r1	329.519518	2	2	pre_focus	3	Contrastive pre_focus
2023404	block1	Control	post	sik3	Verb	Contrastive	r1	138.7315374	3	1	on_focus	3	Contrastive on_focus
2023404	block1	Control	post	baak3	Object	Contrastive	r1	186.1767333	4	1	post_focus	3	Contrastive post_focus
2023404	block1	Control	post	baak3	Object	Contrastive	r1	200.7259789	5	2	post_focus	3	Contrastive post_focus
2023404	block1	Control	post	zoeng1	Subject	Contrastive	r1	347.2498182	1	1	on_focus	1	Contrastive on_focus
2023404	block1	Control	post	saang1	Subject	Contrastive	r1	371.750018	2	2	on_focus	1	Contrastive on_focus
2023404	block1	Control	post	tsa1	Verb	Contrastive	r1	322.6683634	3	1	post_focus	1	Contrastive post_focus
2023404	block1	Control	post	fei1	Object	Contrastive	r1	396.8170824	4	1	post_focus	1	Contrastive post_focus
2023404	block1	Control	post	gei1	Object	Contrastive	r1	279.1647309	5	2	post_focus	1	Contrastive post_focus
2023404	block1	Control	post	jyun2	Subject	Narrow	r1	328.0037793	1	1	pre_focus	2	Narrow pre_focus
2023404	block1	Control	post	jyun2	Subject	Narrow	r1	302.5213987	2	2	pre_focus	2	Narrow pre_focus
2023404	block1	Control	post	mo2	Verb	Narrow	r1	330.5095762	3	1	pre_focus	2	Narrow pre_focus
2023404	block1	Control	post	gau2	Object	Narrow	r1	427.5479476	4	1	on_focus	2	Narrow on_focus
2023404	block1	Control	post	zai2	Object	Narrow	r1	372.2535147	5	2	on_focus	2	Narrow on_focus
2023404	block1	Control	post	sau3	Subject	Broad	r1	337.7834467	1	1	broad_focus	3	Broad focus
2023404	block1	Control	post	sau3	Subject	Broad	r1	283.6866854	2	2	broad_focus	3	Broad focus
2023404	block1	Control	post	sik3	Verb	Broad	r1	85.13929381	3	1	broad_focus	3	Broad focus
2023404	block1	Control	post	baak3	Object	Broad	r1	164.0390659	4	1	broad_focus	3	Broad focus
2023404	block1	Control	post	baak3	Object	Broad	r1	281.4955821	5	2	broad_focus	3	Broad focus
2023404	block1	Control	post	zoeng1	Subject	Broad	r1	340.7992066	1	1	broad_focus	1	Broad focus
2023404	block1	Control	post	saang1	Subject	Broad	r1	409.235974	2	2	broad_focus	1	Broad focus
2023404	block1	Control	post	tsa1	Verb	Broad	r1	335.4761014	3	1	broad_focus	1	Broad focus
2023404	block1	Control	post	fei1	Object	Broad	r1	366.9977722	4	1	broad_focus	1	Broad focus
2023404	block1	Control	post	gei1	Object	Broad	r1	236.3752704	5	2	broad_focus	1	Broad focus
2023404	block1	Control	post	jyun2	Subject	Contrastive	r1	312.762525	1	1	on_focus	2	Contrastive on_focus
2023404	block1	Control	post	jyun2	Subject	Contrastive	r1	301.2039602	2	2	on_focus	2	Contrastive on_focus
2023404	block1	Control	post	mo2	Verb	Contrastive	r1	283.4723143	3	1	post_focus	2	Contrastive post_focus
2023404	block1	Control	post	gau2	Object	Contrastive	r1	385.6145688	4	1	post_focus	2	Contrastive post_focus
2023404	block1	Control	post	zai2	Object	Contrastive	r1	323.4538566	5	2	post_focus	2	Contrastive post_focus
2023404	block1	Control	post	zoeng1	Subject	Narrow	r1	293.2627267	1	1	pre_focus	1	Narrow pre_focus
2023404	block1	Control	post	saang1	Subject	Narrow	r1	341.2450903	2	2	pre_focus	1	Narrow pre_focus
2023404	block1	Control	post	tsa1	Verb	Narrow	r1	320.763048	3	1	on_focus	1	Narrow on_focus
2023404	block1	Control	post	fei1	Object	Narrow	r1	365.1549769	4	1	post_focus	1	Narrow post_focus
2023404	block1	Control	post	gei1	Object	Narrow	r1	279.5758245	5	2	post_focus	1	Narrow post_focus
2023404	block1	Control	post	zoeng1	Subject	Contrastive	r1	388.9508692	1	1	pre_focus	1	Contrastive pre_focus
2023404	block1	Control	post	saang1	Subject	Contrastive	r1	344.5796049	2	2	pre_focus	1	Contrastive pre_focus
2023404	block1	Control	post	tsa1	Verb	Contrastive	r1	269.8441945	3	1	pre_focus	1	Contrastive pre_focus
2023404	block1	Control	post	fei1	Object	Contrastive	r1	380.6231849	4	1	on_focus	1	Contrastive on_focus

2023404	block1	Control	post	gei1	Object	Contrastive	r1	269.3016713	5	2	on_focus	1	Contrastive on_focus
2023404	block1	Control	post	zoeng1	Subject	Contrastive	r1	334.3484956	1	1	pre_focus	1	Contrastive pre_focus
2023404	block1	Control	post	saang1	Subject	Contrastive	r1	358.515927	2	2	pre_focus	1	Contrastive pre_focus
2023404	block1	Control	post	tsa1	Verb	Contrastive	r1	352.6118904	3	1	on_focus	1	Contrastive on_focus
2023404	block1	Control	post	fei1	Object	Contrastive	r1	348.239025	4	1	post_focus	1	Contrastive post_focus
2023404	block1	Control	post	gei1	Object	Contrastive	r1	276.5945452	5	2	post_focus	1	Contrastive post_focus
2023404	block1	Control	post	sau3	Subject	Narrow	r1	271.9344203	1	1	pre_focus	3	Narrow pre_focus
2023404	block1	Control	post	sau3	Subject	Narrow	r1	275.8610111	2	2	pre_focus	3	Narrow pre_focus
2023404	block1	Control	post	sik3	Verb	Narrow	r1	132.7183589	3	1	pre_focus	3	Narrow pre_focus
2023404	block1	Control	post	baak3	Object	Narrow	r1	142.575565	4	1	on_focus	3	Narrow on_focus
2023404	block1	Control	post	baak3	Object	Narrow	r1	81.3018881	5	2	on_focus	3	Narrow on_focus
2023404	block1	Control	post	jyun2	Subject	Contrastive	r1	249.8630702	1	1	pre_focus	2	Contrastive pre_focus
2023404	block1	Control	post	jyun2	Subject	Contrastive	r1	222.3028771	2	2	pre_focus	2	Contrastive pre_focus
2023404	block1	Control	post	mo2	Verb	Contrastive	r1	431.4049509	3	1	on_focus	2	Contrastive on_focus
2023404	block1	Control	post	gau2	Object	Contrastive	r1	399.7284336	4	1	post_focus	2	Contrastive post_focus
2023404	block1	Control	post	zai2	Object	Contrastive	r1	370.3995664	5	2	post_focus	2	Contrastive post_focus
2023404	block1	Control	post	sau3	Subject	Contrastive	r1	344.7729492	1	1	on_focus	3	Contrastive on_focus
2023404	block1	Control	post	sau3	Subject	Contrastive	r1	301.1989558	2	2	on_focus	3	Contrastive on_focus
2023404	block1	Control	post	sik3	Verb	Contrastive	r1	140.054155	3	1	post_focus	3	Contrastive post_focus
2023404	block1	Control	post	baak3	Object	Contrastive	r1	256.4321899	4	1	post_focus	3	Contrastive post_focus
2023404	block1	Control	post	baak3	Object	Contrastive	r1	104.5241021	5	2	post_focus	3	Contrastive post_focus
2023404	block1	Control	post	zoeng1	Subject	Narrow	r1	327.8257259	1	1	on_focus	1	Narrow on_focus
2023404	block1	Control	post	saang1	Subject	Narrow	r1	377.4952799	2	2	on_focus	1	Narrow on_focus
2023404	block1	Control	post	tsa1	Verb	Narrow	r1	328.7952312	3	1	post_focus	1	Narrow post_focus
2023404	block1	Control	post	fei1	Object	Narrow	r1	365.5903927	4	1	post_focus	1	Narrow post_focus
2023404	block1	Control	post	gei1	Object	Narrow	r1	237.8709103	5	2	post_focus	1	Narrow post_focus
2023404	block1	Control	post	jyun2	Subject	Narrow	r1	248.9323119	1	1	pre_focus	2	Narrow pre_focus
2023404	block1	Control	post	jyun2	Subject	Narrow	r1	250.8795844	2	2	pre_focus	2	Narrow pre_focus
2023404	block1	Control	post	mo2	Verb	Narrow	r1	372.9686156	3	1	on_focus	2	Narrow on_focus
2023404	block1	Control	post	gau2	Object	Narrow	r1	484.1803981	4	1	post_focus	2	Narrow post_focus
2023404	block1	Control	post	zai2	Object	Narrow	r1	365.7532605	5	2	post_focus	2	Narrow post_focus
2023404	block1	Control	post	sau3	Subject	Narrow	r1	290.379275	1	1	on_focus	3	Narrow on_focus
2023404	block1	Control	post	sau3	Subject	Narrow	r1	299.6459737	2	2	on_focus	3	Narrow on_focus
2023404	block1	Control	post	sik3	Verb	Narrow	r1	200.8411505	3	1	post_focus	3	Narrow post_focus
2023404	block1	Control	post	baak3	Object	Narrow	r1	154.762178	4	1	post_focus	3	Narrow post_focus
2023404	block1	Control	post	baak3	Object	Narrow	r1	126.7706682	5	2	post_focus	3	Narrow post_focus
2023404	block1	Control	post	sau3	Subject	Narrow	r1	297.8218548	1	1	pre_focus	3	Narrow pre_focus
2023404	block1	Control	post	sau3	Subject	Narrow	r1	319.5369025	2	2	pre_focus	3	Narrow pre_focus
2023404	block1	Control	post	sik3	Verb	Narrow	r1	226.6584436	3	1	on_focus	3	Narrow on_focus
2023404	block1	Control	post	baak3	Object	Narrow	r1	149.9118481	4	1	post_focus	3	Narrow post_focus
2023404	block1	Control	post	baak3	Object	Narrow	r1	171.9976064	5	2	post_focus	3	Narrow post_focus
2023404	block1	Control	post	zoeng1	Subject	Narrow	r2	350.5083576	1	1	pre_focus	1	Narrow pre_focus
2023404	block1	Control	post	saang1	Subject	Narrow	r2	389.9016763	2	2	pre_focus	1	Narrow pre_focus
2023404	block1	Control	post	tsa1	Verb	Narrow	r2	357.6850592	3	1	on_focus	1	Narrow on_focus
2023404	block1	Control	post	fei1	Object	Narrow	r2	373.4586168	4	1	post_focus	1	Narrow post_focus
2023404	block1	Control	post	gei1	Object	Narrow	r2	255.5981535	5	2	post_focus	1	Narrow post_focus
2023404	block1	Control	post	jyun2	Subject	Narrow	r2	258.777502	1	1	pre_focus	2	Narrow pre_focus
2023404	block1	Control	post	jyun2	Subject	Narrow	r2	277.242975	2	2	pre_focus	2	Narrow pre_focus
2023404	block1	Control	post	mo2	Verb	Narrow	r2	413.7028659	3	1	pre_focus	2	Narrow pre_focus
2023404	block1	Control	post	gau2	Object	Narrow	r2	399.921767	4	1	on_focus	2	Narrow on_focus
2023404	block1	Control	post	zai2	Object	Narrow	r2	343.1365403	5	2	on_focus	2	Narrow on_focus
2023404	block1	Control	post	zoeng1	Subject	Narrow	r2	283.8560212	1	1	pre_focus	1	Narrow pre_focus
2023404	block1	Control	post	saang1	Subject	Narrow	r2	328.8547608	2	2	pre_focus	1	Narrow pre_focus
2023404	block1	Control	post	tsa1	Verb	Narrow	r2	325.0851985	3	1	pre_focus	1	Narrow pre_focus
2023404	block1	Control	post	fei1	Object	Narrow	r2	336.952788	4	1	on_focus	1	Narrow on_focus
2023404	block1	Control	post	gei1	Object	Narrow	r2	223.0216464	5	2	on_focus	1	Narrow on_focus
2023404	block1	Control	post	sau3	Subject	Narrow	r2	289.3503846	1	1	on_focus	3	Narrow on_focus
2023404	block1	Control	post	sau3	Subject	Narrow	r2	247.4458123	2	2	on_focus	3	Narrow on_focus
2023404	block1	Control	post	sik3	Verb	Narrow	r2	140.8566165	3	1	post_focus	3	Narrow post_focus
2023404	block1	Control	post	baak3	Object	Narrow	r2	145.18714	4	1	post_focus	3	Narrow post_focus
2023404	block1	Control	post	baak3	Object	Narrow	r2	227.5874636	5	2	post_focus	3	Narrow post_focus
2023404	block1	Control	post	zoeng1	Subject	Broad	r2	345.4580499	1	1	broad_focus	1	Broad focus
2023404	block1	Control	post	saang1	Subject	Broad	r2	359.3594192	2	2	broad_focus	1	Broad focus
2023404	block1	Control	post	tsa1	Verb	Broad	r2	269.2067219	3	1	broad_focus	1	Broad focus
2023404	block1	Control	post	fei1	Object	Broad	r2	369.0776112	4	1	broad_focus	1	Broad focus
2023404	block1	Control	post	gei1	Object	Broad	r2	229.4840785	5	2	broad_focus	1	Broad focus

2023404	block1	Control	post	sau3	Subject	Narrow	r2	311.4835009	1	1	pre_focus	3	Narrow pre_focus
2023404	block1	Control	post	sau3	Subject	Narrow	r2	272.347141	2	2	pre_focus	3	Narrow pre_focus
2023404	block1	Control	post	sik3	Verb	Narrow	r2	154.2263722	3	1	pre_focus	3	Narrow pre_focus
2023404	block1	Control	post	baak3	Object	Narrow	r2	237.4829932	4	1	on_focus	3	Narrow on_focus
2023404	block1	Control	post	baak3	Object	Narrow	r2	234.4681134	5	2	on_focus	3	Narrow on_focus
2023404	block1	Control	post	sau3	Subject	Broad	r2	266.7443902	1	1	broad_focus	3	Broad focus
2023404	block1	Control	post	sau3	Subject	Broad	r2	235.0976997	2	2	broad_focus	3	Broad focus
2023404	block1	Control	post	sik3	Verb	Broad	r2	103.4537667	3	1	broad_focus	3	Broad focus
2023404	block1	Control	post	baak3	Object	Broad	r2	89.77600875	4	1	broad_focus	3	Broad focus
2023404	block1	Control	post	baak3	Object	Broad	r2	85.94372845	5	2	broad_focus	3	Broad focus
2023404	block1	Control	post	zoeng1	Subject	Contrastive	r2	318.1611657	1	1	pre_focus	1	Contrastive pre_focus
2023404	block1	Control	post	saang1	Subject	Contrastive	r2	368.3494246	2	2	pre_focus	1	Contrastive pre_focus
2023404	block1	Control	post	tsa1	Verb	Contrastive	r2	284.4313721	3	1	pre_focus	1	Contrastive pre_focus
2023404	block1	Control	post	fei1	Object	Contrastive	r2	378.4711194	4	1	on_focus	1	Contrastive on_focus
2023404	block1	Control	post	gei1	Object	Contrastive	r2	235.7198943	5	2	on_focus	1	Contrastive on_focus
2023404	block1	Control	post	sau3	Subject	Contrastive	r2	296.9494281	1	1	pre_focus	3	Contrastive pre_focus
2023404	block1	Control	post	sau3	Subject	Contrastive	r2	282.6861395	2	2	pre_focus	3	Contrastive pre_focus
2023404	block1	Control	post	sik3	Verb	Contrastive	r2	247.602044	3	1	on_focus	3	Contrastive on_focus
2023404	block1	Control	post	baak3	Object	Contrastive	r2	221.8098073	4	1	post_focus	3	Contrastive post_focus
2023404	block1	Control	post	baak3	Object	Contrastive	r2	139.2065553	5	2	post_focus	3	Contrastive post_focus
2023404	block1	Control	post	sau3	Subject	Contrastive	r2	297.2996437	1	1	on_focus	3	Contrastive on_focus
2023404	block1	Control	post	sau3	Subject	Contrastive	r2	290.8594492	2	2	on_focus	3	Contrastive on_focus
2023404	block1	Control	post	sik3	Verb	Contrastive	r2	218.1638309	3	1	post_focus	3	Contrastive post_focus
2023404	block1	Control	post	baak3	Object	Contrastive	r2	137.7756182	4	1	post_focus	3	Contrastive post_focus
2023404	block1	Control	post	baak3	Object	Contrastive	r2	174.5162509	5	2	post_focus	3	Contrastive post_focus
2023404	block1	Control	post	zoeng1	Subject	Narrow	r2	326.2966774	1	1	on_focus	1	Narrow on_focus
2023404	block1	Control	post	saang1	Subject	Narrow	r2	334.3776552	2	2	on_focus	1	Narrow on_focus
2023404	block1	Control	post	tsa1	Verb	Narrow	r2	315.9973477	3	1	post_focus	1	Narrow post_focus
2023404	block1	Control	post	fei1	Object	Narrow	r2	365.3059783	4	1	post_focus	1	Narrow post_focus
2023404	block1	Control	post	gei1	Object	Narrow	r2	233.5327424	5	2	post_focus	1	Narrow post_focus
2023404	block1	Control	post	zoeng1	Subject	Contrastive	r2	313.2034663	1	1	on_focus	1	Contrastive on_focus
2023404	block1	Control	post	saang1	Subject	Contrastive	r2	333.0911849	2	2	on_focus	1	Contrastive on_focus
2023404	block1	Control	post	tsa1	Verb	Contrastive	r2	337.9312268	3	1	post_focus	1	Contrastive post_focus
2023404	block1	Control	post	fei1	Object	Contrastive	r2	340.9706752	4	1	post_focus	1	Contrastive post_focus
2023404	block1	Control	post	gei1	Object	Contrastive	r2	258.0958195	5	2	post_focus	1	Contrastive post_focus
2023404	block1	Control	post	jyun2	Subject	Narrow	r2	93.64001915	1	1	pre_focus	2	Narrow pre_focus
2023404	block1	Control	post	jyun2	Subject	Narrow	r2	288.9997062	2	2	pre_focus	2	Narrow pre_focus
2023404	block1	Control	post	mo2	Verb	Narrow	r2	417.5174322	3	1	on_focus	2	Narrow on_focus
2023404	block1	Control	post	gau2	Object	Narrow	r2	430.5470466	4	1	post_focus	2	Narrow post_focus
2023404	block1	Control	post	zai2	Object	Narrow	r2	355.642235	5	2	post_focus	2	Narrow post_focus
2023404	block1	Control	post	zoeng1	Subject	Contrastive	r2	351.4304611	1	1	pre_focus	1	Contrastive pre_focus
2023404	block1	Control	post	saang1	Subject	Contrastive	r2	342.5840211	2	2	pre_focus	1	Contrastive pre_focus
2023404	block1	Control	post	tsa1	Verb	Contrastive	r2	338.3522298	3	1	on_focus	1	Contrastive on_focus
2023404	block1	Control	post	fei1	Object	Contrastive	r2	458.7281802	4	1	post_focus	1	Contrastive post_focus
2023404	block1	Control	post	gei1	Object	Contrastive	r2	251.3295143	5	2	post_focus	1	Contrastive post_focus
2023404	block1	Control	post	jyun2	Subject	Contrastive	r2	221.6888362	1	1	pre_focus	2	Contrastive pre_focus
2023404	block1	Control	post	jyun2	Subject	Contrastive	r2	222.0077724	2	2	pre_focus	2	Contrastive pre_focus
2023404	block1	Control	post	mo2	Verb	Contrastive	r2	381.1551128	3	1	pre_focus	2	Contrastive pre_focus
2023404	block1	Control	post	gau2	Object	Contrastive	r2	493.9657344	4	1	on_focus	2	Contrastive on_focus
2023404	block1	Control	post	zai2	Object	Contrastive	r2	321.8192718	5	2	on_focus	2	Contrastive on_focus
2023404	block1	Control	post	sau3	Subject	Narrow	r2	251.0762744	1	1	pre_focus	3	Narrow pre_focus
2023404	block1	Control	post	sau3	Subject	Narrow	r2	300.4995631	2	2	pre_focus	3	Narrow pre_focus
2023404	block1	Control	post	sik3	Verb	Narrow	r2	126.4306329	3	1	on_focus	3	Narrow on_focus
2023404	block1	Control	post	baak3	Object	Narrow	r2	243.8917234	4	1	post_focus	3	Narrow post_focus
2023404	block1	Control	post	baak3	Object	Narrow	r2	130.9656085	5	2	post_focus	3	Narrow post_focus
2023404	block1	Control	post	jyun2	Subject	Narrow	r2	381.8999519	1	1	on_focus	2	Narrow on_focus
2023404	block1	Control	post	jyun2	Subject	Narrow	r2	295.3307934	2	2	on_focus	2	Narrow on_focus
2023404	block1	Control	post	mo2	Verb	Narrow	r2	318.3030278	3	1	post_focus	2	Narrow post_focus
2023404	block1	Control	post	gau2	Object	Narrow	r2	374.6257581	4	1	post_focus	2	Narrow post_focus
2023404	block1	Control	post	zai2	Object	Narrow	r2	350.401011	5	2	post_focus	2	Narrow post_focus
2023404	block1	Control	post	jyun2	Subject	Contrastive	r2	275.3162722	1	1	pre_focus	2	Contrastive pre_focus
2023404	block1	Control	post	jyun2	Subject	Contrastive	r2	345.8608309	2	2	pre_focus	2	Contrastive pre_focus
2023404	block1	Control	post	mo2	Verb	Contrastive	r2	348.9254561	3	1	on_focus	2	Contrastive on_focus
2023404	block1	Control	post	gau2	Object	Contrastive	r2	392.1750567	4	1	post_focus	2	Contrastive post_focus
2023404	block1	Control	post	zai2	Object	Contrastive	r2	340.1711607	5	2	post_focus	2	Contrastive post_focus
2023404	block1	Control	post	sau3	Subject	Contrastive	r2	278.5683841	1	1	pre_focus	3	Contrastive pre_focus

2023404	block1	Control	post	sau3	Subject	Contrastive	r2	277.9077605	2	2	pre_focus	3	Contrastive pre_focus
2023404	block1	Control	post	sik3	Verb	Contrastive	r2	107.9121614	3	1	pre_focus	3	Contrastive pre_focus
2023404	block1	Control	post	baak3	Object	Contrastive	r2	171.5516536	4	1	on_focus	3	Contrastive on_focus
2023404	block1	Control	post	baak3	Object	Contrastive	r2	100.9262755	5	2	on_focus	3	Contrastive on_focus
2023404	block1	Control	post	jyun2	Subject	Broad	r2	248.5609725	1	1	broad_focus	2	Broad focus
2023404	block1	Control	post	jyun2	Subject	Broad	r2	302.9631387	2	2	broad_focus	2	Broad focus
2023404	block1	Control	post	mo2	Verb	Broad	r2	379.8388499	3	1	broad_focus	2	Broad focus
2023404	block1	Control	post	gau2	Object	Broad	r2	437.7273965	4	1	broad_focus	2	Broad focus
2023404	block1	Control	post	zai2	Object	Broad	r2	339.0277178	5	2	broad_focus	2	Broad focus
2023404	block1	Control	post	jyun2	Subject	Contrastive	r2	408.9092087	1	1	on_focus	2	Contrastive on_focus
2023404	block1	Control	post	jyun2	Subject	Contrastive	r2	288.42695	2	2	on_focus	2	Contrastive on_focus
2023404	block1	Control	post	mo2	Verb	Contrastive	r2	404.491547	3	1	post_focus	2	Contrastive post_focus
2023404	block1	Control	post	gau2	Object	Contrastive	r2	403.7489472	4	1	post_focus	2	Contrastive post_focus
2023404	block1	Control	post	zai2	Object	Contrastive	r2	323.9037698	5	2	post_focus	2	Contrastive post_focus
2023404	block1	Control	pre	sau3	Subject	Broad	r1	238.4934928	1	1	broad_focus	3	Broad focus
2023404	block1	Control	pre	sau3	Subject	Broad	r1	324.4996941	2	2	broad_focus	3	Broad focus
2023404	block1	Control	pre	sik3	Verb	Broad	r1	113.9711056	3	1	broad_focus	3	Broad focus
2023404	block1	Control	pre	baak3	Object	Broad	r1	213.0101384	4	1	broad_focus	3	Broad focus
2023404	block1	Control	pre	baak3	Object	Broad	r1	201.7552163	5	2	broad_focus	3	Broad focus
2023404	block1	Control	pre	sau3	Subject	Narrow	r1	335.1504844	1	1	on_focus	3	Narrow on_focus
2023404	block1	Control	pre	sau3	Subject	Narrow	r1	172.1202477	2	2	on_focus	3	Narrow on_focus
2023404	block1	Control	pre	sik3	Verb	Narrow	r1	197.6314162	3	1	post_focus	3	Narrow post_focus
2023404	block1	Control	pre	baak3	Object	Narrow	r1	93.08843537	4	1	post_focus	3	Narrow post_focus
2023404	block1	Control	pre	baak3	Object	Narrow	r1	105.6842737	5	2	post_focus	3	Narrow post_focus
2023404	block1	Control	pre	jyun2	Subject	Contrastive	r1	193.481909	1	1	pre_focus	2	Contrastive pre_focus
2023404	block1	Control	pre	jyun2	Subject	Contrastive	r1	288.4475094	2	2	pre_focus	2	Contrastive pre_focus
2023404	block1	Control	pre	mo2	Verb	Contrastive	r1	441.5088999	3	1	pre_focus	2	Contrastive pre_focus
2023404	block1	Control	pre	gau2	Object	Contrastive	r1	532.9193392	4	1	on_focus	2	Contrastive on_focus
2023404	block1	Control	pre	zai2	Object	Contrastive	r1	132.1709001	5	2	on_focus	2	Contrastive on_focus
2023404	block1	Control	pre	zoeng1	Subject	Narrow	r1	275.7920898	1	1	pre_focus	1	Narrow pre_focus
2023404	block1	Control	pre	saang1	Subject	Narrow	r1	235.2979066	2	2	pre_focus	1	Narrow pre_focus
2023404	block1	Control	pre	tsa1	Verb	Narrow	r1	298.4735106	3	1	pre_focus	1	Narrow pre_focus
2023404	block1	Control	pre	fei1	Object	Narrow	r1	177.3701544	4	1	on_focus	1	Narrow on_focus
2023404	block1	Control	pre	gei1	Object	Narrow	r1	268.2123811	5	2	on_focus	1	Narrow on_focus
2023404	block1	Control	pre	jyun2	Subject	Contrastive	r1	346.1428571	1	1	pre_focus	2	Contrastive pre_focus
2023404	block1	Control	pre	jyun2	Subject	Contrastive	r1	381.7106264	2	2	pre_focus	2	Contrastive pre_focus
2023404	block1	Control	pre	mo2	Verb	Contrastive	r1	408.1632653	3	1	on_focus	2	Contrastive on_focus
2023404	block1	Control	pre	gau2	Object	Contrastive	r1	420.9294455	4	1	post_focus	2	Contrastive post_focus
2023404	block1	Control	pre	zai2	Object	Contrastive	r1	344.5003084	5	2	post_focus	2	Contrastive post_focus
2023404	block1	Control	pre	zoeng1	Subject	Narrow	r1	285.1098759	1	1	pre_focus	1	Narrow pre_focus
2023404	block1	Control	pre	saang1	Subject	Narrow	r1	335.0808768	2	2	pre_focus	1	Narrow pre_focus
2023404	block1	Control	pre	tsa1	Verb	Narrow	r1	338.4315949	3	1	on_focus	1	Narrow on_focus
2023404	block1	Control	pre	fei1	Object	Narrow	r1	358.2270224	4	1	post_focus	1	Narrow post_focus
2023404	block1	Control	pre	gei1	Object	Narrow	r1	227.9032502	5	2	post_focus	1	Narrow post_focus
2023404	block1	Control	pre	sau3	Subject	Narrow	r1	246.031746	1	1	pre_focus	3	Narrow pre_focus
2023404	block1	Control	pre	sau3	Subject	Narrow	r1	297.496484	2	2	pre_focus	3	Narrow pre_focus
2023404	block1	Control	pre	sik3	Verb	Narrow	r1	250.3075397	3	1	pre_focus	3	Narrow pre_focus
2023404	block1	Control	pre	baak3	Object	Narrow	r1	278.8034188	4	1	on_focus	3	Narrow on_focus
2023404	block1	Control	pre	baak3	Object	Narrow	r1	152.069161	5	2	on_focus	3	Narrow on_focus
2023404	block1	Control	pre	jyun2	Subject	Narrow	r1	268.2570437	1	1	on_focus	2	Narrow on_focus
2023404	block1	Control	pre	jyun2	Subject	Narrow	r1	212.5564809	2	2	on_focus	2	Narrow on_focus
2023404	block1	Control	pre	mo2	Verb	Narrow	r1	333.1145259	3	1	post_focus	2	Narrow post_focus
2023404	block1	Control	pre	gau2	Object	Narrow	r1	400.6980649	4	1	post_focus	2	Narrow post_focus
2023404	block1	Control	pre	zai2	Object	Narrow	r1	160.8874459	5	2	post_focus	2	Narrow post_focus
2023404	block1	Control	pre	zoeng1	Subject	Contrastive	r1	304.7322854	1	1	pre_focus	1	Contrastive pre_focus
2023404	block1	Control	pre	saang1	Subject	Contrastive	r1	318.6326531	2	2	pre_focus	1	Contrastive pre_focus
2023404	block1	Control	pre	tsa1	Verb	Contrastive	r1	317.0873519	3	1	on_focus	1	Contrastive on_focus
2023404	block1	Control	pre	fei1	Object	Contrastive	r1	374.5391966	4	1	post_focus	1	Contrastive post_focus
2023404	block1	Control	pre	gei1	Object	Contrastive	r1	233.0472702	5	2	post_focus	1	Contrastive post_focus
2023404	block1	Control	pre	zoeng1	Subject	Narrow	r1	293.6696654	1	1	on_focus	1	Narrow on_focus
2023404	block1	Control	pre	saang1	Subject	Narrow	r1	315.2505768	2	2	on_focus	1	Narrow on_focus
2023404	block1	Control	pre	tsa1	Verb	Narrow	r1	257.7445086	3	1	post_focus	1	Narrow post_focus
2023404	block1	Control	pre	fei1	Object	Narrow	r1	369.6779687	4	1	post_focus	1	Narrow post_focus
2023404	block1	Control	pre	gei1	Object	Narrow	r1	255.6556777	5	2	post_focus	1	Narrow post_focus
2023404	block1	Control	pre	jyun2	Subject	Narrow	r1	211.9159301	1	1	pre_focus	2	Narrow pre_focus
2023404	block1	Control	pre	jyun2	Subject	Narrow	r1	238.5907285	2	2	pre_focus	2	Narrow pre_focus

**2023404**	block1	Control	pre	mo2	Verb	Narrow	r1	342.1311757	3	1	pre_focus	2	Narrow pre_focus
**2023404**	block1	Control	pre	gau2	Object	Narrow	r1	475.9795231	4	1	on_focus	2	Narrow on_focus
**2023404**	block1	Control	pre	zai2	Object	Narrow	r1	373.3586531	5	2	on_focus	2	Narrow on_focus
**2023404**	block1	Control	pre	zoeng1	Subject	Contrastive	r1	305.1685175	1	1	on_focus	1	Contrastive on_focus
**2023404**	block1	Control	pre	saang1	Subject	Contrastive	r1	358.2494012	2	2	on_focus	1	Contrastive on_focus
**2023404**	block1	Control	pre	tsa1	Verb	Contrastive	r1	274.6986961	3	1	post_focus	1	Contrastive post_focus
**2023404**	block1	Control	pre	fei1	Object	Contrastive	r1	384.7371449	4	1	post_focus	1	Contrastive post_focus
**2023404**	block1	Control	pre	gei1	Object	Contrastive	r1	256.7624913	5	2	post_focus	1	Contrastive post_focus
**2023404**	block1	Control	pre	zoeng1	Subject	Contrastive	r1	251.4741184	1	1	pre_focus	1	Contrastive pre_focus
**2023404**	block1	Control	pre	saang1	Subject	Contrastive	r1	439.1602583	2	2	pre_focus	1	Contrastive pre_focus
**2023404**	block1	Control	pre	tsa1	Verb	Contrastive	r1	347.2409478	3	1	pre_focus	1	Contrastive pre_focus
**2023404**	block1	Control	pre	fei1	Object	Contrastive	r1	355.5660491	4	1	on_focus	1	Contrastive on_focus
**2023404**	block1	Control	pre	gei1	Object	Contrastive	r1	275.3072687	5	2	on_focus	1	Contrastive on_focus
**2023404**	block1	Control	pre	sau3	Subject	Narrow	r1	251.3495843	1	1	pre_focus	3	Narrow pre_focus
**2023404**	block1	Control	pre	sau3	Subject	Narrow	r1	305.1043166	2	2	pre_focus	3	Narrow pre_focus
**2023404**	block1	Control	pre	sik3	Verb	Narrow	r1	123.7707231	3	1	on_focus	3	Narrow on_focus
**2023404**	block1	Control	pre	baak3	Object	Narrow	r1	204.3276499	4	1	post_focus	3	Narrow post_focus
**2023404**	block1	Control	pre	baak3	Object	Narrow	r1	199.0926269	5	2	post_focus	3	Narrow post_focus
**2023404**	block1	Control	pre	sau3	Subject	Contrastive	r1	318.5569409	1	1	pre_focus	3	Contrastive pre_focus
**2023404**	block1	Control	pre	sau3	Subject	Contrastive	r1	380.0387359	2	2	pre_focus	3	Contrastive pre_focus
**2023404**	block1	Control	pre	sik3	Verb	Contrastive	r1	146.9194201	3	1	pre_focus	3	Contrastive pre_focus
**2023404**	block1	Control	pre	baak3	Object	Contrastive	r1	181.0289308	4	1	on_focus	3	Contrastive on_focus
**2023404**	block1	Control	pre	baak3	Object	Contrastive	r1	148.0190476	5	2	on_focus	3	Contrastive on_focus
**2023404**	block1	Control	pre	jyun2	Subject	Contrastive	r1	272.5553697	1	1	on_focus	2	Contrastive on_focus
**2023404**	block1	Control	pre	jyun2	Subject	Contrastive	r1	353.6671599	2	2	on_focus	2	Contrastive on_focus
**2023404**	block1	Control	pre	mo2	Verb	Contrastive	r1	412.8153958	3	1	post_focus	2	Contrastive post_focus
**2023404**	block1	Control	pre	gau2	Object	Contrastive	r1	435.4124485	4	1	post_focus	2	Contrastive post_focus
**2023404**	block1	Control	pre	zai2	Object	Contrastive	r1	179.3954855	5	2	post_focus	2	Contrastive post_focus
**2023404**	block1	Control	pre	zoeng1	Subject	Broad	r1	280.4901043	1	1	broad_focus	1	Broad focus
**2023404**	block1	Control	pre	saang1	Subject	Broad	r1	339.4600807	2	2	broad_focus	1	Broad focus
**2023404**	block1	Control	pre	tsa1	Verb	Broad	r1	347.3740998	3	1	broad_focus	1	Broad focus
**2023404**	block1	Control	pre	fei1	Object	Broad	r1	311.7420334	4	1	broad_focus	1	Broad focus
**2023404**	block1	Control	pre	gei1	Object	Broad	r1	243.7799362	5	2	broad_focus	1	Broad focus
**2023404**	block1	Control	pre	jyun2	Subject	Narrow	r1	345.0718638	1	1	pre_focus	2	Narrow pre_focus
**2023404**	block1	Control	pre	jyun2	Subject	Narrow	r1	366.8028426	2	2	pre_focus	2	Narrow pre_focus
**2023404**	block1	Control	pre	mo2	Verb	Narrow	r1	476.4805111	3	1	on_focus	2	Narrow on_focus
**2023404**	block1	Control	pre	gau2	Object	Narrow	r1	424.6058921	4	1	post_focus	2	Narrow post_focus
**2023404**	block1	Control	pre	zai2	Object	Narrow	r1	273.6753106	5	2	post_focus	2	Narrow post_focus
**2023404**	block1	Control	pre	sau3	Subject	Contrastive	r1	359.8892968	1	1	pre_focus	3	Contrastive pre_focus
**2023404**	block1	Control	pre	sau3	Subject	Contrastive	r1	590.9057576	2	2	pre_focus	3	Contrastive pre_focus
**2023404**	block1	Control	pre	sik3	Verb	Contrastive	r1	252.8380305	3	1	on_focus	3	Contrastive on_focus
**2023404**	block1	Control	pre	baak3	Object	Contrastive	r1	313.3907785	4	1	post_focus	3	Contrastive post_focus
**2023404**	block1	Control	pre	baak3	Object	Contrastive	r1	140.1527265	5	2	post_focus	3	Contrastive post_focus
**2023404**	block1	Control	pre	sau3	Subject	Contrastive	r1	228.271029	1	1	on_focus	3	Contrastive on_focus
**2023404**	block1	Control	pre	sau3	Subject	Contrastive	r1	371.5929705	2	2	on_focus	3	Contrastive on_focus
**2023404**	block1	Control	pre	sik3	Verb	Contrastive	r1	125.1868049	3	1	post_focus	3	Contrastive post_focus
**2023404**	block1	Control	pre	baak3	Object	Contrastive	r1	175.9941211	4	1	post_focus	3	Contrastive post_focus
**2023404**	block1	Control	pre	baak3	Object	Contrastive	r1	90.58314437	5	2	post_focus	3	Contrastive post_focus
**2023404**	block1	Control	pre	jyun2	Subject	Broad	r1	172.5095038	1	1	broad_focus	2	Broad focus
**2023404**	block1	Control	pre	jyun2	Subject	Broad	r1	222.7634071	2	2	broad_focus	2	Broad focus
**2023404**	block1	Control	pre	mo2	Verb	Broad	r1	497.8361145	3	1	broad_focus	2	Broad focus
**2023404**	block1	Control	pre	gau2	Object	Broad	r1	389.2164045	4	1	broad_focus	2	Broad focus
**2023404**	block1	Control	pre	zai2	Object	Broad	r1	256.277458	5	2	broad_focus	2	Broad focus
**2023404**	block1	Control	pre	zoeng1	Subject	Contrastive	r2	273.775703	1	1	pre_focus	1	Contrastive pre_focus
**2023404**	block1	Control	pre	saang1	Subject	Contrastive	r2	317.9910792	2	2	pre_focus	1	Contrastive pre_focus
**2023404**	block1	Control	pre	tsa1	Verb	Contrastive	r2	283.2514972	3	1	pre_focus	1	Contrastive pre_focus
**2023404**	block1	Control	pre	fei1	Object	Contrastive	r2	304.4205882	4	1	on_focus	1	Contrastive on_focus
**2023404**	block1	Control	pre	gei1	Object	Contrastive	r2	272.3532376	5	2	on_focus	1	Contrastive on_focus
**2023404**	block1	Control	pre	jyun2	Subject	Broad	r2	237.0048671	1	1	broad_focus	2	Broad focus
**2023404**	block1	Control	pre	jyun2	Subject	Broad	r2	211.920007	2	2	broad_focus	2	Broad focus
**2023404**	block1	Control	pre	mo2	Verb	Broad	r2	281.8614379	3	1	broad_focus	2	Broad focus
**2023404**	block1	Control	pre	gau2	Object	Broad	r2	388.8732809	4	1	broad_focus	2	Broad focus
**2023404**	block1	Control	pre	zai2	Object	Broad	r2	308.6281179	5	2	broad_focus	2	Broad focus
**2023404**	block1	Control	pre	zoeng1	Subject	Broad	r2	323.7472555	1	1	broad_focus	1	Broad focus
**2023404**	block1	Control	pre	saang1	Subject	Broad	r2	321.4819398	2	2	broad_focus	1	Broad focus
**2023404**	block1	Control	pre	tsa1	Verb	Broad	r2	284.4625595	3	1	broad_focus	1	Broad focus

**2023404**	block1	Control	pre	fei1	Object	Broad	r2	404.0230537	4	1	broad_focus	1	Broad focus
**2023404**	block1	Control	pre	gei1	Object	Broad	r2	246.4810967	5	2	broad_focus	1	Broad focus
**2023404**	block1	Control	pre	jyun2	Subject	Contrastive	r2	261.9671568	1	1	pre_focus	2	Contrastive pre_focus
**2023404**	block1	Control	pre	jyun2	Subject	Contrastive	r2	303.0384549	2	2	pre_focus	2	Contrastive pre_focus
**2023404**	block1	Control	pre	mo2	Verb	Contrastive	r2	371.8653239	3	1	pre_focus	2	Contrastive pre_focus
**2023404**	block1	Control	pre	gau2	Object	Contrastive	r2	387.3363568	4	1	on_focus	2	Contrastive on_focus
**2023404**	block1	Control	pre	zai2	Object	Contrastive	r2	291.3465608	5	2	on_focus	2	Contrastive on_focus
**2023404**	block1	Control	pre	sau3	Subject	Narrow	r2	314.4808151	1	1	on_focus	3	Narrow on_focus
**2023404**	block1	Control	pre	sau3	Subject	Narrow	r2	274.2990426	2	2	on_focus	3	Narrow on_focus
**2023404**	block1	Control	pre	sik3	Verb	Narrow	r2	119.4490104	3	1	post_focus	3	Narrow post_focus
**2023404**	block1	Control	pre	baak3	Object	Narrow	r2	136.0502825	4	1	post_focus	3	Narrow post_focus
**2023404**	block1	Control	pre	baak3	Object	Narrow	r2	139.271461	5	2	post_focus	3	Narrow post_focus
**2023404**	block1	Control	pre	zoeng1	Subject	Narrow	r2	259.3535697	1	1	pre_focus	1	Narrow pre_focus
**2023404**	block1	Control	pre	saang1	Subject	Narrow	r2	350.9801214	2	2	pre_focus	1	Narrow pre_focus
**2023404**	block1	Control	pre	tsa1	Verb	Narrow	r2	265.7137308	3	1	pre_focus	1	Narrow pre_focus
**2023404**	block1	Control	pre	fei1	Object	Narrow	r2	360.2972895	4	1	on_focus	1	Narrow on_focus
**2023404**	block1	Control	pre	gei1	Object	Narrow	r2	263.4509265	5	2	on_focus	1	Narrow on_focus
**2023404**	block1	Control	pre	sau3	Subject	Contrastive	r2	274.0349564	1	1	pre_focus	3	Contrastive pre_focus
**2023404**	block1	Control	pre	sau3	Subject	Contrastive	r2	267.2253716	2	2	pre_focus	3	Contrastive pre_focus
**2023404**	block1	Control	pre	sik3	Verb	Contrastive	r2	134.8617351	3	1	on_focus	3	Contrastive on_focus
**2023404**	block1	Control	pre	baak3	Object	Contrastive	r2	140.2357415	4	1	post_focus	3	Contrastive post_focus
**2023404**	block1	Control	pre	baak3	Object	Contrastive	r2	126.5846021	5	2	post_focus	3	Contrastive post_focus
**2023404**	block1	Control	pre	jyun2	Subject	Narrow	r2	307.6920364	1	1	pre_focus	2	Narrow pre_focus
**2023404**	block1	Control	pre	jyun2	Subject	Narrow	r2	249.6073635	2	2	pre_focus	2	Narrow pre_focus
**2023404**	block1	Control	pre	mo2	Verb	Narrow	r2	331.5109723	3	1	pre_focus	2	Narrow pre_focus
**2023404**	block1	Control	pre	gau2	Object	Narrow	r2	448.3221277	4	1	on_focus	2	Narrow on_focus
**2023404**	block1	Control	pre	zai2	Object	Narrow	r2	322.2336159	5	2	on_focus	2	Narrow on_focus
**2023404**	block1	Control	pre	zoeng1	Subject	Narrow	r2	287.3940804	1	1	pre_focus	1	Narrow pre_focus
**2023404**	block1	Control	pre	saang1	Subject	Narrow	r2	364.6964676	2	2	pre_focus	1	Narrow pre_focus
**2023404**	block1	Control	pre	tsa1	Verb	Narrow	r2	330.8585066	3	1	on_focus	1	Narrow on_focus
**2023404**	block1	Control	pre	fei1	Object	Narrow	r2	318.0342482	4	1	post_focus	1	Narrow post_focus
**2023404**	block1	Control	pre	gei1	Object	Narrow	r2	265.5810944	5	2	post_focus	1	Narrow post_focus
**2023404**	block1	Control	pre	sau3	Subject	Broad	r2	296.6352207	1	1	broad_focus	3	Broad focus
**2023404**	block1	Control	pre	sau3	Subject	Broad	r2	296.1832044	2	2	broad_focus	3	Broad focus
**2023404**	block1	Control	pre	sik3	Verb	Broad	r2	204.8294772	3	1	broad_focus	3	Broad focus
**2023404**	block1	Control	pre	baak3	Object	Broad	r2	163.1090055	4	1	broad_focus	3	Broad focus
**2023404**	block1	Control	pre	baak3	Object	Broad	r2	165.32158	5	2	broad_focus	3	Broad focus
**2023404**	block1	Control	pre	zoeng1	Subject	Contrastive	r2	380.1319064	1	1	on_focus	1	Contrastive on_focus
**2023404**	block1	Control	pre	saang1	Subject	Contrastive	r2	354.3164105	2	2	on_focus	1	Contrastive on_focus
**2023404**	block1	Control	pre	tsa1	Verb	Contrastive	r2	294.1244513	3	1	post_focus	1	Contrastive post_focus
**2023404**	block1	Control	pre	fei1	Object	Contrastive	r2	356.0431889	4	1	post_focus	1	Contrastive post_focus
**2023404**	block1	Control	pre	gei1	Object	Contrastive	r2	213.6896663	5	2	post_focus	1	Contrastive post_focus
**2023404**	block1	Control	pre	sau3	Subject	Contrastive	r2	303.4976866	1	1	on_focus	3	Contrastive on_focus
**2023404**	block1	Control	pre	sau3	Subject	Contrastive	r2	283.6779922	2	2	on_focus	3	Contrastive on_focus
**2023404**	block1	Control	pre	sik3	Verb	Contrastive	r2	126.0358403	3	1	post_focus	3	Contrastive post_focus
**2023404**	block1	Control	pre	baak3	Object	Contrastive	r2	237.1134818	4	1	post_focus	3	Contrastive post_focus
**2023404**	block1	Control	pre	baak3	Object	Contrastive	r2	104.9158611	5	2	post_focus	3	Contrastive post_focus
**2023404**	block1	Control	pre	zoeng1	Subject	Narrow	r2	359.6195786	1	1	on_focus	1	Narrow on_focus
**2023404**	block1	Control	pre	saang1	Subject	Narrow	r2	275.7543784	2	2	on_focus	1	Narrow on_focus
**2023404**	block1	Control	pre	tsa1	Verb	Narrow	r2	344.8681267	3	1	post_focus	1	Narrow post_focus
**2023404**	block1	Control	pre	fei1	Object	Narrow	r2	337.3185941	4	1	post_focus	1	Narrow post_focus
**2023404**	block1	Control	pre	gei1	Object	Narrow	r2	325.8453798	5	2	post_focus	1	Narrow post_focus
**2023404**	block1	Control	pre	sau3	Subject	Narrow	r2	302.3255325	1	1	pre_focus	3	Narrow pre_focus
**2023404**	block1	Control	pre	sau3	Subject	Narrow	r2	282.1947324	2	2	pre_focus	3	Narrow pre_focus
**2023404**	block1	Control	pre	sik3	Verb	Narrow	r2	106.0183296	3	1	on_focus	3	Narrow on_focus
**2023404**	block1	Control	pre	baak3	Object	Narrow	r2	244.8088367	4	1	post_focus	3	Narrow post_focus
**2023404**	block1	Control	pre	baak3	Object	Narrow	r2	110.2859204	5	2	post_focus	3	Narrow post_focus
**2023404**	block1	Control	pre	sau3	Subject	Contrastive	r2	240.1246266	1	1	pre_focus	3	Contrastive pre_focus
**2023404**	block1	Control	pre	sau3	Subject	Contrastive	r2	300.6600259	2	2	pre_focus	3	Contrastive pre_focus
**2023404**	block1	Control	pre	sik3	Verb	Contrastive	r2	225.031854	3	1	pre_focus	3	Contrastive pre_focus
**2023404**	block1	Control	pre	baak3	Object	Contrastive	r2	159.2187178	4	1	on_focus	3	Contrastive on_focus
**2023404**	block1	Control	pre	baak3	Object	Contrastive	r2	105.8308088	5	2	on_focus	3	Contrastive on_focus
**2023404**	block1	Control	pre	jyun2	Subject	Narrow	r2	208.6712241	1	1	pre_focus	2	Narrow pre_focus
**2023404**	block1	Control	pre	jyun2	Subject	Narrow	r2	306.4050037	2	2	pre_focus	2	Narrow pre_focus
**2023404**	block1	Control	pre	mo2	Verb	Narrow	r2	403.4096322	3	1	on_focus	2	Narrow on_focus
**2023404**	block1	Control	pre	gau2	Object	Narrow	r2	487.3786953	4	1	post_focus	2	Narrow post_focus

**2023404**	block1	Control	pre	zai2	Object	Narrow	r2	225.4918177	5	2	post_focus	2	Narrow post_focus
**2023404**	block1	Control	pre	zoeng1	Subject	Contrastive	r2	332.6110106	1	1	pre_focus	1	Contrastive pre_focus
**2023404**	block1	Control	pre	saang1	Subject	Contrastive	r2	368.8084266	2	2	pre_focus	1	Contrastive pre_focus
**2023404**	block1	Control	pre	tsa1	Verb	Contrastive	r2	359.0038979	3	1	on_focus	1	Contrastive on_focus
**2023404**	block1	Control	pre	fei1	Object	Contrastive	r2	391.0725993	4	1	post_focus	1	Contrastive post_focus
**2023404**	block1	Control	pre	gei1	Object	Contrastive	r2	207.8612615	5	2	post_focus	1	Contrastive post_focus
**2023404**	block1	Control	pre	jyun2	Subject	Narrow	r2	196.1565522	1	1	on_focus	2	Narrow on_focus
**2023404**	block1	Control	pre	jyun2	Subject	Narrow	r2	283.2966742	2	2	on_focus	2	Narrow on_focus
**2023404**	block1	Control	pre	mo2	Verb	Narrow	r2	500.2307526	3	1	post_focus	2	Narrow post_focus
**2023404**	block1	Control	pre	gau2	Object	Narrow	r2	425.92906	4	1	post_focus	2	Narrow post_focus
**2023404**	block1	Control	pre	zai2	Object	Narrow	r2	241.3371417	5	2	post_focus	2	Narrow post_focus
**2023404**	block1	Control	pre	jyun2	Subject	Contrastive	r2	306.3759472	1	1	on_focus	2	Contrastive on_focus
**2023404**	block1	Control	pre	jyun2	Subject	Contrastive	r2	300.1416959	2	2	on_focus	2	Contrastive on_focus
**2023404**	block1	Control	pre	mo2	Verb	Contrastive	r2	326.5410472	3	1	post_focus	2	Contrastive post_focus
**2023404**	block1	Control	pre	gau2	Object	Contrastive	r2	378.5486844	4	1	post_focus	2	Contrastive post_focus
**2023404**	block1	Control	pre	zai2	Object	Contrastive	r2	335.7031998	5	2	post_focus	2	Contrastive post_focus
**2023404**	block1	Control	pre	sau3	Subject	Narrow	r2	276.5126561	1	1	pre_focus	3	Narrow pre_focus
**2023404**	block1	Control	pre	sau3	Subject	Narrow	r2	289.3818984	2	2	pre_focus	3	Narrow pre_focus
**2023404**	block1	Control	pre	sik3	Verb	Narrow	r2	130.9806361	3	1	pre_focus	3	Narrow pre_focus
**2023404**	block1	Control	pre	baak3	Object	Narrow	r2	170.6482701	4	1	on_focus	3	Narrow on_focus
**2023404**	block1	Control	pre	baak3	Object	Narrow	r2	117.7288544	5	2	on_focus	3	Narrow on_focus
**2023404**	block1	Control	pre	jyun2	Subject	Contrastive	r2	196.3335203	1	1	pre_focus	2	Contrastive pre_focus
**2023404**	block1	Control	pre	jyun2	Subject	Contrastive	r2	238.0488372	2	2	pre_focus	2	Contrastive pre_focus
**2023404**	block1	Control	pre	mo2	Verb	Contrastive	r2	436.6122663	3	1	on_focus	2	Contrastive on_focus
**2023404**	block1	Control	pre	gau2	Object	Contrastive	r2	407.4864289	4	1	post_focus	2	Contrastive post_focus
**2023404**	block1	Control	pre	zai2	Object	Contrastive	r2	287.7141871	5	2	post_focus	2	Contrastive post_focus
**2023404**	block2	Control	post	ngaa5	Subject	Narrow	r1	540.4421139	1	1	on_focus	5	Narrow on_focus
**2023404**	block2	Control	post	ngaa5	Subject	Narrow	r1	394.6324822	2	2	on_focus	5	Narrow on_focus
**2023404**	block2	Control	post	maai5	Verb	Narrow	r1	371.2934716	3	1	post_focus	5	Narrow post_focus
**2023404**	block2	Control	post	pou5	Object	Narrow	r1	74.67139078	4	1	post_focus	5	Narrow post_focus
**2023404**	block2	Control	post	pou5	Object	Narrow	r1	78.90011223	5	2	post_focus	5	Narrow post_focus
**2023404**	block2	Control	post	ngaa5	Subject	Narrow	r1	113.1776754	1	1	pre_focus	5	Narrow pre_focus
**2023404**	block2	Control	post	ngaa5	Subject	Narrow	r1	245.3187893	2	2	pre_focus	5	Narrow pre_focus
**2023404**	block2	Control	post	maai5	Verb	Narrow	r1	175.2609986	3	1	pre_focus	5	Narrow pre_focus
**2023404**	block2	Control	post	pou5	Object	Narrow	r1	347.1020637	4	1	on_focus	5	Narrow on_focus
**2023404**	block2	Control	post	pou5	Object	Narrow	r1	122.8982954	5	2	on_focus	5	Narrow on_focus
**2023404**	block2	Control	post	ma4	Subject	Contrastive	r1	229.0077516	1	1	pre_focus	4	Contrastive pre_focus
**2023404**	block2	Control	post	ma4	Subject	Contrastive	r1	284.5007413	2	2	pre_focus	4	Contrastive pre_focus
**2023404**	block2	Control	post	fu4	Verb	Contrastive	r1	115.8396321	3	1	on_focus	4	Contrastive on_focus
**2023404**	block2	Control	post	maang4	Object	Contrastive	r1	341.9486675	4	1	post_focus	4	Contrastive post_focus
**2023404**	block2	Control	post	Jan-04	Object	Contrastive	r1	246.4187321	5	2	post_focus	4	Contrastive post_focus
**2023404**	block2	Control	post	ma4	Subject	Broad	r1	389.5790834	1	1	broad_focus	4	Broad focus
**2023404**	block2	Control	post	ma4	Subject	Broad	r1	344.8878301	2	2	broad_focus	4	Broad focus
**2023404**	block2	Control	post	fu4	Verb	Broad	r1	208.7936118	3	1	broad_focus	4	Broad focus
**2023404**	block2	Control	post	maang4	Object	Broad	r1	457.8288288	4	1	broad_focus	4	Broad focus
**2023404**	block2	Control	post	Jan-04	Object	Broad	r1	158.7083525	5	2	broad_focus	4	Broad focus
**2023404**	block2	Control	post	lok6	Subject	Contrastive	r1	85.57236393	1	1	pre_focus	6	Contrastive pre_focus
**2023404**	block2	Control	post	lok6	Subject	Contrastive	r1	207.6807561	2	2	pre_focus	6	Contrastive pre_focus
**2023404**	block2	Control	post	waa6	Verb	Contrastive	r1	225.2972361	3	1	pre_focus	6	Contrastive pre_focus
**2023404**	block2	Control	post	jyut6	Object	Contrastive	r1	208.0249433	4	1	on_focus	6	Contrastive on_focus
**2023404**	block2	Control	post	loeng6	Object	Contrastive	r1	277.194532	5	2	on_focus	6	Contrastive on_focus
**2023404**	block2	Control	post	lok6	Subject	Contrastive	r1	123.2664597	1	1	pre_focus	6	Contrastive pre_focus
**2023404**	block2	Control	post	lok6	Subject	Contrastive	r1	138.9457124	2	2	pre_focus	6	Contrastive pre_focus
**2023404**	block2	Control	post	waa6	Verb	Contrastive	r1	158.5447387	3	1	on_focus	6	Contrastive on_focus
**2023404**	block2	Control	post	jyut6	Object	Contrastive	r1	206.5022027	4	1	post_focus	6	Contrastive post_focus
**2023404**	block2	Control	post	loeng6	Object	Contrastive	r1	296.7048867	5	2	post_focus	6	Contrastive post_focus
**2023404**	block2	Control	post	lok6	Subject	Contrastive	r1	211.9491416	1	1	on_focus	6	Contrastive on_focus
**2023404**	block2	Control	post	lok6	Subject	Contrastive	r1	227.7566269	2	2	on_focus	6	Contrastive on_focus
**2023404**	block2	Control	post	waa6	Verb	Contrastive	r1	223.7523638	3	1	post_focus	6	Contrastive post_focus
**2023404**	block2	Control	post	jyut6	Object	Contrastive	r1	195.2976477	4	1	post_focus	6	Contrastive post_focus
**2023404**	block2	Control	post	loeng6	Object	Contrastive	r1	288.9619204	5	2	post_focus	6	Contrastive post_focus
**2023404**	block2	Control	post	ngaa5	Subject	Contrastive	r1	286.0142668	1	1	on_focus	5	Contrastive on_focus
**2023404**	block2	Control	post	ngaa5	Subject	Contrastive	r1	242.3671465	2	2	on_focus	5	Contrastive on_focus
**2023404**	block2	Control	post	maai5	Verb	Contrastive	r1	397.8426147	3	1	post_focus	5	Contrastive post_focus
**2023404**	block2	Control	post	pou5	Object	Contrastive	r1	178.0502402	4	1	post_focus	5	Contrastive post_focus
**2023404**	block2	Control	post	pou5	Object	Contrastive	r1	151.31387	5	2	post_focus	5	Contrastive post_focus

2023404	block2	Control	post	lok6	Subject	Narrow	r1	179.3166044	1	1	on_focus	6	Narrow on_focus
2023404	block2	Control	post	lok6	Subject	Narrow	r1	271.0092128	2	2	on_focus	6	Narrow on_focus
2023404	block2	Control	post	waa6	Verb	Narrow	r1	250.7069661	3	1	post_focus	6	Narrow post_focus
2023404	block2	Control	post	jyut6	Object	Narrow	r1	180.9246253	4	1	post_focus	6	Narrow post_focus
2023404	block2	Control	post	loeng6	Object	Narrow	r1	199.1256805	5	2	post_focus	6	Narrow post_focus
2023404	block2	Control	post	lok6	Subject	Broad	r1	160.2388881	1	1	broad_focus	6	Broad focus
2023404	block2	Control	post	lok6	Subject	Broad	r1	196.2539879	2	2	broad_focus	6	Broad focus
2023404	block2	Control	post	waa6	Verb	Broad	r1	193.177221	3	1	broad_focus	6	Broad focus
2023404	block2	Control	post	jyut6	Object	Broad	r1	163.0633393	4	1	broad_focus	6	Broad focus
2023404	block2	Control	post	loeng6	Object	Broad	r1	297.3969277	5	2	broad_focus	6	Broad focus
2023404	block2	Control	post	ngaa5	Subject	Contrastive	r1	228.4313002	1	1	pre_focus	5	Contrastive pre_focus
2023404	block2	Control	post	ngaa5	Subject	Contrastive	r1	338.8223063	2	2	pre_focus	5	Contrastive pre_focus
2023404	block2	Control	post	maai5	Verb	Contrastive	r1	421.312463	3	1	on_focus	5	Contrastive on_focus
2023404	block2	Control	post	pou5	Object	Contrastive	r1	391.6789137	4	1	post_focus	5	Contrastive post_focus
2023404	block2	Control	post	pou5	Object	Contrastive	r1	154.7849127	5	2	post_focus	5	Contrastive post_focus
2023404	block2	Control	post	lok6	Subject	Narrow	r1	157.8210821	1	1	pre_focus	6	Narrow pre_focus
2023404	block2	Control	post	lok6	Subject	Narrow	r1	307.4311945	2	2	pre_focus	6	Narrow pre_focus
2023404	block2	Control	post	waa6	Verb	Narrow	r1	282.6752331	3	1	on_focus	6	Narrow on_focus
2023404	block2	Control	post	jyut6	Object	Narrow	r1	192.6085521	4	1	post_focus	6	Narrow post_focus
2023404	block2	Control	post	loeng6	Object	Narrow	r1	390.0231845	5	2	post_focus	6	Narrow post_focus
2023404	block2	Control	post	ngaa5	Subject	Contrastive	r1	307.4540177	1	1	pre_focus	5	Contrastive pre_focus
2023404	block2	Control	post	ngaa5	Subject	Contrastive	r1	280.9392244	2	2	pre_focus	5	Contrastive pre_focus
2023404	block2	Control	post	maai5	Verb	Contrastive	r1	383.5870308	3	1	pre_focus	5	Contrastive pre_focus
2023404	block2	Control	post	pou5	Object	Contrastive	r1	391.9537272	4	1	on_focus	5	Contrastive on_focus
2023404	block2	Control	post	pou5	Object	Contrastive	r1	300.4874693	5	2	on_focus	5	Contrastive on_focus
2023404	block2	Control	post	ma4	Subject	Contrastive	r1	263.8730159	1	1	pre_focus	4	Contrastive pre_focus
2023404	block2	Control	post	ma4	Subject	Contrastive	r1	270.6814716	2	2	pre_focus	4	Contrastive pre_focus
2023404	block2	Control	post	fu4	Verb	Contrastive	r1	218.9074978	3	1	pre_focus	4	Contrastive pre_focus
2023404	block2	Control	post	maang4	Object	Contrastive	r1	454.8656833	4	1	on_focus	4	Contrastive on_focus
2023404	block2	Control	post	Jan-04	Object	Contrastive	r1	264.2964608	5	2	on_focus	4	Contrastive on_focus
2023404	block2	Control	post	ma4	Subject	Narrow	r1	289.3501942	1	1	pre_focus	4	Narrow pre_focus
2023404	block2	Control	post	ma4	Subject	Narrow	r1	368.3775995	2	2	pre_focus	4	Narrow pre_focus
2023404	block2	Control	post	fu4	Verb	Narrow	r1	214.053288	3	1	pre_focus	4	Narrow pre_focus
2023404	block2	Control	post	maang4	Object	Narrow	r1	483.0643642	4	1	on_focus	4	Narrow on_focus
2023404	block2	Control	post	Jan-04	Object	Narrow	r1	180.4960683	5	2	on_focus	4	Narrow on_focus
2023404	block2	Control	post	ma4	Subject	Contrastive	r1	351.4750271	1	1	on_focus	4	Contrastive on_focus
2023404	block2	Control	post	ma4	Subject	Contrastive	r1	366.8039535	2	2	on_focus	4	Contrastive on_focus
2023404	block2	Control	post	fu4	Verb	Contrastive	r1	135.5618994	3	1	post_focus	4	Contrastive post_focus
2023404	block2	Control	post	maang4	Object	Contrastive	r1	329.8776947	4	1	post_focus	4	Contrastive post_focus
2023404	block2	Control	post	Jan-04	Object	Contrastive	r1	106.5384714	5	2	post_focus	4	Contrastive post_focus
2023404	block2	Control	post	lok6	Subject	Narrow	r1	192.3420257	1	1	pre_focus	6	Narrow pre_focus
2023404	block2	Control	post	lok6	Subject	Narrow	r1	226.3144512	2	2	pre_focus	6	Narrow pre_focus
2023404	block2	Control	post	waa6	Verb	Narrow	r1	199.7414261	3	1	pre_focus	6	Narrow pre_focus
2023404	block2	Control	post	jyut6	Object	Narrow	r1	161.1371056	4	1	on_focus	6	Narrow on_focus
2023404	block2	Control	post	loeng6	Object	Narrow	r1	341.9221563	5	2	on_focus	6	Narrow on_focus
2023404	block2	Control	post	ma4	Subject	Narrow	r1	302.5758293	1	1	on_focus	4	Narrow on_focus
2023404	block2	Control	post	ma4	Subject	Narrow	r1	356.2585599	2	2	on_focus	4	Narrow on_focus
2023404	block2	Control	post	fu4	Verb	Narrow	r1	157.0075113	3	1	post_focus	4	Narrow post_focus
2023404	block2	Control	post	maang4	Object	Narrow	r1	387.9510582	4	1	post_focus	4	Narrow post_focus
2023404	block2	Control	post	Jan-04	Object	Narrow	r1	226.8128952	5	2	post_focus	4	Narrow post_focus
2023404	block2	Control	post	ma4	Subject	Narrow	r1	277.5791252	1	1	pre_focus	4	Narrow pre_focus
2023404	block2	Control	post	ma4	Subject	Narrow	r1	340.5934938	2	2	pre_focus	4	Narrow pre_focus
2023404	block2	Control	post	fu4	Verb	Narrow	r1	200.8273485	3	1	on_focus	4	Narrow on_focus
2023404	block2	Control	post	maang4	Object	Narrow	r1	428.7938762	4	1	post_focus	4	Narrow post_focus
2023404	block2	Control	post	Jan-04	Object	Narrow	r1	221.8463683	5	2	post_focus	4	Narrow post_focus
2023404	block2	Control	post	ngaa5	Subject	Narrow	r1	258.1118429	1	1	pre_focus	5	Narrow pre_focus
2023404	block2	Control	post	ngaa5	Subject	Narrow	r1	331.0070275	2	2	pre_focus	5	Narrow pre_focus
2023404	block2	Control	post	maai5	Verb	Narrow	r1	474.4649033	3	1	on_focus	5	Narrow on_focus
2023404	block2	Control	post	pou5	Object	Narrow	r1	222.1014686	4	1	post_focus	5	Narrow post_focus
2023404	block2	Control	post	pou5	Object	Narrow	r1	244.8252006	5	2	post_focus	5	Narrow post_focus
2023404	block2	Control	post	ngaa5	Subject	Broad	r1	279.7871339	1	1	broad_focus	5	Broad focus
2023404	block2	Control	post	ngaa5	Subject	Broad	r1	308.8599064	2	2	broad_focus	5	Broad focus
2023404	block2	Control	post	maai5	Verb	Broad	r1	456.8884635	3	1	broad_focus	5	Broad focus
2023404	block2	Control	post	pou5	Object	Broad	r1	203.3765605	4	1	broad_focus	5	Broad focus
2023404	block2	Control	post	pou5	Object	Broad	r1	219.4049481	5	2	broad_focus	5	Broad focus
2023404	block2	Control	post	ngaa5	Subject	Narrow	r2	298.1193674	1	1	on_focus	5	Narrow on_focus

**2023404**	block2	Control	post	ngaa5	Subject	Narrow	r2	278.4393518	2	2	on_focus	5	Narrow on_focus
**2023404**	block2	Control	post	maai5	Verb	Narrow	r2	402.258924	3	1	post_focus	5	Narrow post_focus
**2023404**	block2	Control	post	pou5	Object	Narrow	r2	134.5612107	4	1	post_focus	5	Narrow post_focus
**2023404**	block2	Control	post	pou5	Object	Narrow	r2	149.7190058	5	2	post_focus	5	Narrow post_focus
**2023404**	block2	Control	post	ma4	Subject	Broad	r2	283.8689982	1	1	broad_focus	4	Broad focus
**2023404**	block2	Control	post	ma4	Subject	Broad	r2	335.833751	2	2	broad_focus	4	Broad focus
**2023404**	block2	Control	post	fu4	Verb	Broad	r2	204.2603133	3	1	broad_focus	4	Broad focus
**2023404**	block2	Control	post	maang4	Object	Broad	r2	439.6003769	4	1	broad_focus	4	Broad focus
**2023404**	block2	Control	post	Jan-04	Object	Broad	r2	216.6765219	5	2	broad_focus	4	Broad focus
**2023404**	block2	Control	post	lok6	Subject	Contrastive	r2	157.8367576	1	1	pre_focus	6	Contrastive pre_focus
**2023404**	block2	Control	post	lok6	Subject	Contrastive	r2	237.8854875	2	2	pre_focus	6	Contrastive pre_focus
**2023404**	block2	Control	post	waa6	Verb	Contrastive	r2	233.9765239	3	1	on_focus	6	Contrastive on_focus
**2023404**	block2	Control	post	jyut6	Object	Contrastive	r2	202.172996	4	1	post_focus	6	Contrastive post_focus
**2023404**	block2	Control	post	loeng6	Object	Contrastive	r2	282.8001654	5	2	post_focus	6	Contrastive post_focus
**2023404**	block2	Control	post	ma4	Subject	Contrastive	r2	242.5831322	1	1	pre_focus	4	Contrastive pre_focus
**2023404**	block2	Control	post	ma4	Subject	Contrastive	r2	314.5196987	2	2	pre_focus	4	Contrastive pre_focus
**2023404**	block2	Control	post	fu4	Verb	Contrastive	r2	187.9179551	3	1	pre_focus	4	Contrastive pre_focus
**2023404**	block2	Control	post	maang4	Object	Contrastive	r2	317.7649969	4	1	on_focus	4	Contrastive on_focus
**2023404**	block2	Control	post	Jan-04	Object	Contrastive	r2	162.3015873	5	2	on_focus	4	Contrastive on_focus
**2023404**	block2	Control	post	ngaa5	Subject	Broad	r2	268.0151993	1	1	broad_focus	5	Broad focus
**2023404**	block2	Control	post	ngaa5	Subject	Broad	r2	322.4968971	2	2	broad_focus	5	Broad focus
**2023404**	block2	Control	post	maai5	Verb	Broad	r2	510.3059291	3	1	broad_focus	5	Broad focus
**2023404**	block2	Control	post	pou5	Object	Broad	r2	262.0265637	4	1	broad_focus	5	Broad focus
**2023404**	block2	Control	post	pou5	Object	Broad	r2	144.4451278	5	2	broad_focus	5	Broad focus
**2023404**	block2	Control	post	ma4	Subject	Narrow	r2	270.0108277	1	1	on_focus	4	Narrow on_focus
**2023404**	block2	Control	post	ma4	Subject	Narrow	r2	338.0238095	2	2	on_focus	4	Narrow on_focus
**2023404**	block2	Control	post	fu4	Verb	Narrow	r2	184.3163535	3	1	post_focus	4	Narrow post_focus
**2023404**	block2	Control	post	maang4	Object	Narrow	r2	371.0333494	4	1	post_focus	4	Narrow post_focus
**2023404**	block2	Control	post	Jan-04	Object	Narrow	r2	181.4468632	5	2	post_focus	4	Narrow post_focus
**2023404**	block2	Control	post	lok6	Subject	Narrow	r2	155.1925526	1	1	pre_focus	6	Narrow pre_focus
**2023404**	block2	Control	post	lok6	Subject	Narrow	r2	189.6488696	2	2	pre_focus	6	Narrow pre_focus
**2023404**	block2	Control	post	waa6	Verb	Narrow	r2	225.2715878	3	1	pre_focus	6	Narrow pre_focus
**2023404**	block2	Control	post	jyut6	Object	Narrow	r2	202.6995493	4	1	on_focus	6	Narrow on_focus
**2023404**	block2	Control	post	loeng6	Object	Narrow	r2	372.615359	5	2	on_focus	6	Narrow on_focus
**2023404**	block2	Control	post	lok6	Subject	Narrow	r2	137.7245713	1	1	pre_focus	6	Narrow pre_focus
**2023404**	block2	Control	post	lok6	Subject	Narrow	r2	144.7793673	2	2	pre_focus	6	Narrow pre_focus
**2023404**	block2	Control	post	waa6	Verb	Narrow	r2	254.8817898	3	1	on_focus	6	Narrow on_focus
**2023404**	block2	Control	post	jyut6	Object	Narrow	r2	205.4300309	4	1	post_focus	6	Narrow post_focus
**2023404**	block2	Control	post	loeng6	Object	Narrow	r2	291.2185869	5	2	post_focus	6	Narrow post_focus
**2023404**	block2	Control	post	ngaa5	Subject	Contrastive	r2	204.4161907	1	1	pre_focus	5	Contrastive pre_focus
**2023404**	block2	Control	post	ngaa5	Subject	Contrastive	r2	172.167909	2	2	pre_focus	5	Contrastive pre_focus
**2023404**	block2	Control	post	maai5	Verb	Contrastive	r2	439.1266823	3	1	on_focus	5	Contrastive on_focus
**2023404**	block2	Control	post	pou5	Object	Contrastive	r2	400.1829034	4	1	post_focus	5	Contrastive post_focus
**2023404**	block2	Control	post	pou5	Object	Contrastive	r2	298.9293813	5	2	post_focus	5	Contrastive post_focus
**2023404**	block2	Control	post	ma4	Subject	Narrow	r2	249.6239538	1	1	pre_focus	4	Narrow pre_focus
**2023404**	block2	Control	post	ma4	Subject	Narrow	r2	350.7183142	2	2	pre_focus	4	Narrow pre_focus
**2023404**	block2	Control	post	fu4	Verb	Narrow	r2	241.0532156	3	1	pre_focus	4	Narrow pre_focus
**2023404**	block2	Control	post	maang4	Object	Narrow	r2	408.3116085	4	1	on_focus	4	Narrow on_focus
**2023404**	block2	Control	post	Jan-04	Object	Narrow	r2	280.3909522	5	2	on_focus	4	Narrow on_focus
**2023404**	block2	Control	post	lok6	Subject	Contrastive	r2	166.6841331	1	1	pre_focus	6	Contrastive pre_focus
**2023404**	block2	Control	post	lok6	Subject	Contrastive	r2	218.2914421	2	2	pre_focus	6	Contrastive pre_focus
**2023404**	block2	Control	post	waa6	Verb	Contrastive	r2	230.826457	3	1	pre_focus	6	Contrastive pre_focus
**2023404**	block2	Control	post	jyut6	Object	Contrastive	r2	224.0052512	4	1	on_focus	6	Contrastive on_focus
**2023404**	block2	Control	post	loeng6	Object	Contrastive	r2	246.1039765	5	2	on_focus	6	Contrastive on_focus
**2023404**	block2	Control	post	ma4	Subject	Contrastive	r2	189.0654848	1	1	pre_focus	4	Contrastive pre_focus
**2023404**	block2	Control	post	ma4	Subject	Contrastive	r2	388.0610092	2	2	pre_focus	4	Contrastive pre_focus
**2023404**	block2	Control	post	fu4	Verb	Contrastive	r2	188.1820538	3	1	on_focus	4	Contrastive on_focus
**2023404**	block2	Control	post	maang4	Object	Contrastive	r2	404.2699809	4	1	post_focus	4	Contrastive post_focus
**2023404**	block2	Control	post	Jan-04	Object	Contrastive	r2	158.8887777	5	2	post_focus	4	Contrastive post_focus
**2023404**	block2	Control	post	ma4	Subject	Contrastive	r2	303.0650038	1	1	on_focus	4	Contrastive on_focus
**2023404**	block2	Control	post	ma4	Subject	Contrastive	r2	392.5287226	2	2	on_focus	4	Contrastive on_focus
**2023404**	block2	Control	post	fu4	Verb	Contrastive	r2	262.1548344	3	1	post_focus	4	Contrastive post_focus
**2023404**	block2	Control	post	maang4	Object	Contrastive	r2	264.3393667	4	1	post_focus	4	Contrastive post_focus
**2023404**	block2	Control	post	Jan-04	Object	Contrastive	r2	149.0332218	5	2	post_focus	4	Contrastive post_focus
**2023404**	block2	Control	post	lok6	Subject	Broad	r2	183.0413832	1	1	broad_focus	6	Broad focus
**2023404**	block2	Control	post	lok6	Subject	Broad	r2	227.0804989	2	2	broad_focus	6	Broad focus

**2023404**	block2	Control	post	waa6	Verb	Broad	r2	331.9816075	3	1	broad_focus	6	Broad focus
**2023404**	block2	Control	post	jyut6	Object	Broad	r2	230.0642678	4	1	broad_focus	6	Broad focus
**2023404**	block2	Control	post	loeng6	Object	Broad	r2	317.0159251	5	2	broad_focus	6	Broad focus
**2023404**	block2	Control	post	lok6	Subject	Narrow	r2	194.5559335	1	1	on_focus	6	Narrow on_focus
**2023404**	block2	Control	post	lok6	Subject	Narrow	r2	212.3750544	2	2	on_focus	6	Narrow on_focus
**2023404**	block2	Control	post	waa6	Verb	Narrow	r2	207.3425079	3	1	post_focus	6	Narrow post_focus
**2023404**	block2	Control	post	jyut6	Object	Narrow	r2	187.127833	4	1	post_focus	6	Narrow post_focus
**2023404**	block2	Control	post	loeng6	Object	Narrow	r2	294.6284628	5	2	post_focus	6	Narrow post_focus
**2023404**	block2	Control	post	ngaa5	Subject	Contrastive	r2	376.4627768	1	1	on_focus	5	Contrastive on_focus
**2023404**	block2	Control	post	ngaa5	Subject	Contrastive	r2	389.9011992	2	2	on_focus	5	Contrastive on_focus
**2023404**	block2	Control	post	maai5	Verb	Contrastive	r2	429.0868655	3	1	post_focus	5	Contrastive post_focus
**2023404**	block2	Control	post	pou5	Object	Contrastive	r2	388.4820907	4	1	post_focus	5	Contrastive post_focus
**2023404**	block2	Control	post	pou5	Object	Contrastive	r2	144.1354367	5	2	post_focus	5	Contrastive post_focus
**2023404**	block2	Control	post	ngaa5	Subject	Contrastive	r2	244.9912577	1	1	pre_focus	5	Contrastive pre_focus
**2023404**	block2	Control	post	ngaa5	Subject	Contrastive	r2	255.6111354	2	2	pre_focus	5	Contrastive pre_focus
**2023404**	block2	Control	post	maai5	Verb	Contrastive	r2	393.6787604	3	1	pre_focus	5	Contrastive pre_focus
**2023404**	block2	Control	post	pou5	Object	Contrastive	r2	393.1866485	4	1	on_focus	5	Contrastive on_focus
**2023404**	block2	Control	post	pou5	Object	Contrastive	r2	260.4283729	5	2	on_focus	5	Contrastive on_focus
**2023404**	block2	Control	post	lok6	Subject	Contrastive	r2	210.2271956	1	1	on_focus	6	Contrastive on_focus
**2023404**	block2	Control	post	lok6	Subject	Contrastive	r2	165.1676997	2	2	on_focus	6	Contrastive on_focus
**2023404**	block2	Control	post	waa6	Verb	Contrastive	r2	174.7906544	3	1	post_focus	6	Contrastive post_focus
**2023404**	block2	Control	post	jyut6	Object	Contrastive	r2	170.723194	4	1	post_focus	6	Contrastive post_focus
**2023404**	block2	Control	post	loeng6	Object	Contrastive	r2	225.5885724	5	2	post_focus	6	Contrastive post_focus
**2023404**	block2	Control	post	ma4	Subject	Narrow	r2	280.1449647	1	1	pre_focus	4	Narrow pre_focus
**2023404**	block2	Control	post	ma4	Subject	Narrow	r2	320.0004724	2	2	pre_focus	4	Narrow pre_focus
**2023404**	block2	Control	post	fu4	Verb	Narrow	r2	195.0354308	3	1	on_focus	4	Narrow on_focus
**2023404**	block2	Control	post	maang4	Object	Narrow	r2	413.3529169	4	1	post_focus	4	Narrow post_focus
**2023404**	block2	Control	post	Jan-04	Object	Narrow	r2	112.3997636	5	2	post_focus	4	Narrow post_focus
**2023404**	block2	Control	post	ngaa5	Subject	Narrow	r2	226.9912806	1	1	pre_focus	5	Narrow pre_focus
**2023404**	block2	Control	post	ngaa5	Subject	Narrow	r2	328.817064	2	2	pre_focus	5	Narrow pre_focus
**2023404**	block2	Control	post	maai5	Verb	Narrow	r2	352.4617749	3	1	pre_focus	5	Narrow pre_focus
**2023404**	block2	Control	post	pou5	Object	Narrow	r2	354.800152	4	1	on_focus	5	Narrow on_focus
**2023404**	block2	Control	post	pou5	Object	Narrow	r2	266.1335169	5	2	on_focus	5	Narrow on_focus
**2023404**	block2	Control	post	ngaa5	Subject	Narrow	r2	247.8449664	1	1	pre_focus	5	Narrow pre_focus
**2023404**	block2	Control	post	ngaa5	Subject	Narrow	r2	286.1378326	2	2	pre_focus	5	Narrow pre_focus
**2023404**	block2	Control	post	maai5	Verb	Narrow	r2	423.0579271	3	1	on_focus	5	Narrow on_focus
**2023404**	block2	Control	post	pou5	Object	Narrow	r2	351.4315623	4	1	post_focus	5	Narrow post_focus
**2023404**	block2	Control	post	pou5	Object	Narrow	r2	177.0991254	5	2	post_focus	5	Narrow post_focus
**2023404**	block2	Control	pre	ma4	Subject	Narrow	r1	193.4699919	1	1	pre_focus	4	Narrow pre_focus
**2023404**	block2	Control	pre	ma4	Subject	Narrow	r1	305.1350826	2	2	pre_focus	4	Narrow pre_focus
**2023404**	block2	Control	pre	fu4	Verb	Narrow	r1	173.8973923	3	1	pre_focus	4	Narrow pre_focus
**2023404**	block2	Control	pre	maang4	Object	Narrow	r1	489.9837578	4	1	on_focus	4	Narrow on_focus
**2023404**	block2	Control	pre	Jan-04	Object	Narrow	r1	289.9773243	5	2	on_focus	4	Narrow on_focus
**2023404**	block2	Control	pre	ma4	Subject	Contrastive	r1	280.2666019	1	1	pre_focus	4	Contrastive pre_focus
**2023404**	block2	Control	pre	ma4	Subject	Contrastive	r1	238.5118238	2	2	pre_focus	4	Contrastive pre_focus
**2023404**	block2	Control	pre	fu4	Verb	Contrastive	r1	48.29365079	3	1	pre_focus	4	Contrastive pre_focus
**2023404**	block2	Control	pre	maang4	Object	Contrastive	r1	464.1424991	4	1	on_focus	4	Contrastive on_focus
**2023404**	block2	Control	pre	Jan-04	Object	Contrastive	r1	308.4140066	5	2	on_focus	4	Contrastive on_focus
**2023404**	block2	Control	pre	ma4	Subject	Contrastive	r1	339.0778534	1	1	pre_focus	4	Contrastive pre_focus
**2023404**	block2	Control	pre	ma4	Subject	Contrastive	r1	400.5903832	2	2	pre_focus	4	Contrastive pre_focus
**2023404**	block2	Control	pre	fu4	Verb	Contrastive	r1	177.9280045	3	1	on_focus	4	Contrastive on_focus
**2023404**	block2	Control	pre	maang4	Object	Contrastive	r1	448.8344671	4	1	post_focus	4	Contrastive post_focus
**2023404**	block2	Control	pre	Jan-04	Object	Contrastive	r1	236.2522046	5	2	post_focus	4	Contrastive post_focus
**2023404**	block2	Control	pre	ngaa5	Subject	Narrow	r1	163.7095671	1	1	pre_focus	5	Narrow pre_focus
**2023404**	block2	Control	pre	ngaa5	Subject	Narrow	r1	286.7853665	2	2	pre_focus	5	Narrow pre_focus
**2023404**	block2	Control	pre	maai5	Verb	Narrow	r1	192.6867862	3	1	pre_focus	5	Narrow pre_focus
**2023404**	block2	Control	pre	pou5	Object	Narrow	r1	88.99422799	4	1	on_focus	5	Narrow on_focus
**2023404**	block2	Control	pre	pou5	Object	Narrow	r1	230.8981874	5	2	on_focus	5	Narrow on_focus
**2023404**	block2	Control	pre	lok6	Subject	Contrastive	r1	170.367011	1	1	pre_focus	6	Contrastive pre_focus
**2023404**	block2	Control	pre	lok6	Subject	Contrastive	r1	220.1472636	2	2	pre_focus	6	Contrastive pre_focus
**2023404**	block2	Control	pre	waa6	Verb	Contrastive	r1	296.0285066	3	1	pre_focus	6	Contrastive pre_focus
**2023404**	block2	Control	pre	jyut6	Object	Contrastive	r1	199.3785168	4	1	on_focus	6	Contrastive on_focus
**2023404**	block2	Control	pre	loeng6	Object	Contrastive	r1	483.606576	5	2	on_focus	6	Contrastive on_focus
**2023404**	block2	Control	pre	ma4	Subject	Broad	r1	210.2045016	1	1	broad_focus	4	Broad focus
**2023404**	block2	Control	pre	ma4	Subject	Broad	r1	462.1756258	2	2	broad_focus	4	Broad focus
**2023404**	block2	Control	pre	fu4	Verb	Broad	r1	151.040697	3	1	broad_focus	4	Broad focus

**2023404**	block2	Control	pre	maang4	Object	Broad	r1	447.4390689	4	1	broad_focus	4	Broad focus
**2023404**	block2	Control	pre	Jan-04	Object	Broad	r1	353.8864128	5	2	broad_focus	4	Broad focus
**2023404**	block2	Control	pre	lok6	Subject	Narrow	r1	146.9880143	1	1	pre_focus	6	Narrow pre_focus
**2023404**	block2	Control	pre	lok6	Subject	Narrow	r1	224.1558327	2	2	pre_focus	6	Narrow pre_focus
**2023404**	block2	Control	pre	waa6	Verb	Narrow	r1	210.2806763	3	1	on_focus	6	Narrow on_focus
**2023404**	block2	Control	pre	jyut6	Object	Narrow	r1	189.7675669	4	1	post_focus	6	Narrow post_focus
**2023404**	block2	Control	pre	loeng6	Object	Narrow	r1	337.1524811	5	2	post_focus	6	Narrow post_focus
**2023404**	block2	Control	pre	ngaa5	Subject	Narrow	r1	419.4223356	1	1	on_focus	5	Narrow on_focus
**2023404**	block2	Control	pre	ngaa5	Subject	Narrow	r1	360.3966166	2	2	on_focus	5	Narrow on_focus
**2023404**	block2	Control	pre	maai5	Verb	Narrow	r1	117.7392786	3	1	post_focus	5	Narrow post_focus
**2023404**	block2	Control	pre	pou5	Object	Narrow	r1	233.6580877	4	1	post_focus	5	Narrow post_focus
**2023404**	block2	Control	pre	pou5	Object	Narrow	r1	225.1247166	5	2	post_focus	5	Narrow post_focus
**2023404**	block2	Control	pre	ngaa5	Subject	Broad	r1	275.9870087	1	1	broad_focus	5	Broad focus
**2023404**	block2	Control	pre	ngaa5	Subject	Broad	r1	483.9038486	2	2	broad_focus	5	Broad focus
**2023404**	block2	Control	pre	maai5	Verb	Broad	r1	161.161205	3	1	broad_focus	5	Broad focus
**2023404**	block2	Control	pre	pou5	Object	Broad	r1	340.8545513	4	1	broad_focus	5	Broad focus
**2023404**	block2	Control	pre	pou5	Object	Broad	r1	307.5363684	5	2	broad_focus	5	Broad focus
**2023404**	block2	Control	pre	ngaa5	Subject	Contrastive	r1	412.5059781	1	1	pre_focus	5	Contrastive pre_focus
**2023404**	block2	Control	pre	ngaa5	Subject	Contrastive	r1	266.0627745	2	2	pre_focus	5	Contrastive pre_focus
**2023404**	block2	Control	pre	maai5	Verb	Contrastive	r1	377.2765066	3	1	pre_focus	5	Contrastive pre_focus
**2023404**	block2	Control	pre	pou5	Object	Contrastive	r1	271.3137656	4	1	on_focus	5	Contrastive on_focus
**2023404**	block2	Control	pre	pou5	Object	Contrastive	r1	320.7931973	5	2	on_focus	5	Contrastive on_focus
**2023404**	block2	Control	pre	ma4	Subject	Contrastive	r1	325.1919455	1	1	on_focus	4	Contrastive on_focus
**2023404**	block2	Control	pre	ma4	Subject	Contrastive	r1	347.3642605	2	2	on_focus	4	Contrastive on_focus
**2023404**	block2	Control	pre	fu4	Verb	Contrastive	r1	136.6855435	3	1	post_focus	4	Contrastive post_focus
**2023404**	block2	Control	pre	maang4	Object	Contrastive	r1	153.755102	4	1	post_focus	4	Contrastive post_focus
**2023404**	block2	Control	pre	Jan-04	Object	Contrastive	r1	252.8676453	5	2	post_focus	4	Contrastive post_focus
**2023404**	block2	Control	pre	ma4	Subject	Narrow	r1	301.2584577	1	1	pre_focus	4	Narrow pre_focus
**2023404**	block2	Control	pre	ma4	Subject	Narrow	r1	350.436184	2	2	pre_focus	4	Narrow pre_focus
**2023404**	block2	Control	pre	fu4	Verb	Narrow	r1	132.4335475	3	1	on_focus	4	Narrow on_focus
**2023404**	block2	Control	pre	maang4	Object	Narrow	r1	384.0346615	4	1	post_focus	4	Narrow post_focus
**2023404**	block2	Control	pre	Jan-04	Object	Narrow	r1	181.7496599	5	2	post_focus	4	Narrow post_focus
**2023404**	block2	Control	pre	lok6	Subject	Contrastive	r1	210.9137654	1	1	on_focus	6	Contrastive on_focus
**2023404**	block2	Control	pre	lok6	Subject	Contrastive	r1	233.8956555	2	2	on_focus	6	Contrastive on_focus
**2023404**	block2	Control	pre	waa6	Verb	Contrastive	r1	319.4698197	3	1	post_focus	6	Contrastive post_focus
**2023404**	block2	Control	pre	jyut6	Object	Contrastive	r1	237.324263	4	1	post_focus	6	Contrastive post_focus
**2023404**	block2	Control	pre	loeng6	Object	Contrastive	r1	310.9604241	5	2	post_focus	6	Contrastive post_focus
**2023404**	block2	Control	pre	ngaa5	Subject	Narrow	r1	201.8662132	1	1	pre_focus	5	Narrow pre_focus
**2023404**	block2	Control	pre	ngaa5	Subject	Narrow	r1	294.7173811	2	2	pre_focus	5	Narrow pre_focus
**2023404**	block2	Control	pre	maai5	Verb	Narrow	r1	203.8238538	3	1	on_focus	5	Narrow on_focus
**2023404**	block2	Control	pre	pou5	Object	Narrow	r1	247.3812763	4	1	post_focus	5	Narrow post_focus
**2023404**	block2	Control	pre	pou5	Object	Narrow	r1	244.8703241	5	2	post_focus	5	Narrow post_focus
**2023404**	block2	Control	pre	ma4	Subject	Narrow	r1	313.4395023	1	1	on_focus	4	Narrow on_focus
**2023404**	block2	Control	pre	ma4	Subject	Narrow	r1	411.8879018	2	2	on_focus	4	Narrow on_focus
**2023404**	block2	Control	pre	fu4	Verb	Narrow	r1	202.8321248	3	1	post_focus	4	Narrow post_focus
**2023404**	block2	Control	pre	maang4	Object	Narrow	r1	271.2152175	4	1	post_focus	4	Narrow post_focus
**2023404**	block2	Control	pre	Jan-04	Object	Narrow	r1	264.4050024	5	2	post_focus	4	Narrow post_focus
**2023404**	block2	Control	pre	ngaa5	Subject	Contrastive	r1	275.1518499	1	1	pre_focus	5	Contrastive pre_focus
**2023404**	block2	Control	pre	ngaa5	Subject	Contrastive	r1	206.4925674	2	2	pre_focus	5	Contrastive pre_focus
**2023404**	block2	Control	pre	maai5	Verb	Contrastive	r1	204.3366951	3	1	on_focus	5	Contrastive on_focus
**2023404**	block2	Control	pre	pou5	Object	Contrastive	r1	236.4218314	4	1	post_focus	5	Contrastive post_focus
**2023404**	block2	Control	pre	pou5	Object	Contrastive	r1	325.5661784	5	2	post_focus	5	Contrastive post_focus
**2023404**	block2	Control	pre	ngaa5	Subject	Contrastive	r1	254.8849811	1	1	on_focus	5	Contrastive on_focus
**2023404**	block2	Control	pre	ngaa5	Subject	Contrastive	r1	297.0045645	2	2	on_focus	5	Contrastive on_focus
**2023404**	block2	Control	pre	maai5	Verb	Contrastive	r1	386.5381422	3	1	post_focus	5	Contrastive post_focus
**2023404**	block2	Control	pre	pou5	Object	Contrastive	r1	228.9592205	4	1	post_focus	5	Contrastive post_focus
**2023404**	block2	Control	pre	pou5	Object	Contrastive	r1	317.1467818	5	2	post_focus	5	Contrastive post_focus
**2023404**	block2	Control	pre	lok6	Subject	Narrow	r1	128.3645755	1	1	on_focus	6	Narrow on_focus
**2023404**	block2	Control	pre	lok6	Subject	Narrow	r1	231.1980348	2	2	on_focus	6	Narrow on_focus
**2023404**	block2	Control	pre	waa6	Verb	Narrow	r1	199.2883979	3	1	post_focus	6	Narrow post_focus
**2023404**	block2	Control	pre	jyut6	Object	Narrow	r1	187.9635444	4	1	post_focus	6	Narrow post_focus
**2023404**	block2	Control	pre	loeng6	Object	Narrow	r1	278.4283064	5	2	post_focus	6	Narrow post_focus
**2023404**	block2	Control	pre	lok6	Subject	Narrow	r1	112.317637	1	1	pre_focus	6	Narrow pre_focus
**2023404**	block2	Control	pre	lok6	Subject	Narrow	r1	259.9311076	2	2	pre_focus	6	Narrow pre_focus
**2023404**	block2	Control	pre	waa6	Verb	Narrow	r1	157.6681164	3	1	pre_focus	6	Narrow pre_focus
**2023404**	block2	Control	pre	jyut6	Object	Narrow	r1	263.6360667	4	1	on_focus	6	Narrow on_focus

**2023404**	block2	Control	pre	loeng6	Object	Narrow	r1	312.7041564	5	2	on_focus	6	Narrow on_focus
**2023404**	block2	Control	pre	lok6	Subject	Contrastive	r1	134.8314437	1	1	pre_focus	6	Contrastive pre_focus
**2023404**	block2	Control	pre	lok6	Subject	Contrastive	r1	217.7878946	2	2	pre_focus	6	Contrastive pre_focus
**2023404**	block2	Control	pre	waa6	Verb	Contrastive	r1	277.1801587	3	1	on_focus	6	Contrastive on_focus
**2023404**	block2	Control	pre	jyut6	Object	Contrastive	r1	177.9875283	4	1	post_focus	6	Contrastive post_focus
**2023404**	block2	Control	pre	loeng6	Object	Contrastive	r1	247.0537499	5	2	post_focus	6	Contrastive post_focus
**2023404**	block2	Control	pre	ngaa5	Subject	Contrastive	r2	323.8600491	1	1	pre_focus	5	Contrastive pre_focus
**2023404**	block2	Control	pre	ngaa5	Subject	Contrastive	r2	291.4992005	2	2	pre_focus	5	Contrastive pre_focus
**2023404**	block2	Control	pre	maai5	Verb	Contrastive	r2	541.7729656	3	1	on_focus	5	Contrastive on_focus
**2023404**	block2	Control	pre	pou5	Object	Contrastive	r2	220.6857338	4	1	post_focus	5	Contrastive post_focus
**2023404**	block2	Control	pre	pou5	Object	Contrastive	r2	239.5529116	5	2	post_focus	5	Contrastive post_focus
**2023404**	block2	Control	pre	ma4	Subject	Broad	r2	256.2615242	1	1	broad_focus	4	Broad focus
**2023404**	block2	Control	pre	ma4	Subject	Broad	r2	352.5729088	2	2	broad_focus	4	Broad focus
**2023404**	block2	Control	pre	fu4	Verb	Broad	r2	206.6039934	3	1	broad_focus	4	Broad focus
**2023404**	block2	Control	pre	maang4	Object	Broad	r2	385.3460515	4	1	broad_focus	4	Broad focus
**2023404**	block2	Control	pre	Jan-04	Object	Broad	r2	240.5881889	5	2	broad_focus	4	Broad focus
**2023404**	block2	Control	pre	lok6	Subject	Contrastive	r2	201.2909209	1	1	pre_focus	6	Contrastive pre_focus
**2023404**	block2	Control	pre	lok6	Subject	Contrastive	r2	227.6295659	2	2	pre_focus	6	Contrastive pre_focus
**2023404**	block2	Control	pre	waa6	Verb	Contrastive	r2	308.2955345	3	1	on_focus	6	Contrastive on_focus
**2023404**	block2	Control	pre	jyut6	Object	Contrastive	r2	204.2295918	4	1	post_focus	6	Contrastive post_focus
**2023404**	block2	Control	pre	loeng6	Object	Contrastive	r2	289.7565058	5	2	post_focus	6	Contrastive post_focus
**2023404**	block2	Control	pre	lok6	Subject	Contrastive	r2	150.5166124	1	1	on_focus	6	Contrastive on_focus
**2023404**	block2	Control	pre	lok6	Subject	Contrastive	r2	226.2641723	2	2	on_focus	6	Contrastive on_focus
**2023404**	block2	Control	pre	waa6	Verb	Contrastive	r2	287.8228053	3	1	post_focus	6	Contrastive post_focus
**2023404**	block2	Control	pre	jyut6	Object	Contrastive	r2	178.3952938	4	1	post_focus	6	Contrastive post_focus
**2023404**	block2	Control	pre	loeng6	Object	Contrastive	r2	299.5261286	5	2	post_focus	6	Contrastive post_focus
**2023404**	block2	Control	pre	ngaa5	Subject	Narrow	r2	288.3630278	1	1	pre_focus	5	Narrow pre_focus
**2023404**	block2	Control	pre	ngaa5	Subject	Narrow	r2	315.4772928	2	2	pre_focus	5	Narrow pre_focus
**2023404**	block2	Control	pre	maai5	Verb	Narrow	r2	371.9003993	3	1	pre_focus	5	Narrow pre_focus
**2023404**	block2	Control	pre	pou5	Object	Narrow	r2	284.0225787	4	1	on_focus	5	Narrow on_focus
**2023404**	block2	Control	pre	pou5	Object	Narrow	r2	272.4177293	5	2	on_focus	5	Narrow on_focus
**2023404**	block2	Control	pre	ma4	Subject	Contrastive	r2	311.4421085	1	1	on_focus	4	Contrastive on_focus
**2023404**	block2	Control	pre	ma4	Subject	Contrastive	r2	395.1031498	2	2	on_focus	4	Contrastive on_focus
**2023404**	block2	Control	pre	fu4	Verb	Contrastive	r2	233.7885643	3	1	post_focus	4	Contrastive post_focus
**2023404**	block2	Control	pre	maang4	Object	Contrastive	r2	477.4561946	4	1	post_focus	4	Contrastive post_focus
**2023404**	block2	Control	pre	Jan-04	Object	Contrastive	r2	158.3375305	5	2	post_focus	4	Contrastive post_focus
**2023404**	block2	Control	pre	ngaa5	Subject	Contrastive	r2	276.6237799	1	1	pre_focus	5	Contrastive pre_focus
**2023404**	block2	Control	pre	ngaa5	Subject	Contrastive	r2	314.0650734	2	2	pre_focus	5	Contrastive pre_focus
**2023404**	block2	Control	pre	maai5	Verb	Contrastive	r2	252.0053597	3	1	pre_focus	5	Contrastive pre_focus
**2023404**	block2	Control	pre	pou5	Object	Contrastive	r2	334.3341076	4	1	on_focus	5	Contrastive on_focus
**2023404**	block2	Control	pre	pou5	Object	Contrastive	r2	279.6335111	5	2	on_focus	5	Contrastive on_focus
**2023404**	block2	Control	pre	lok6	Subject	Narrow	r2	96.26922938	1	1	pre_focus	6	Narrow pre_focus
**2023404**	block2	Control	pre	lok6	Subject	Narrow	r2	196.3138414	2	2	pre_focus	6	Narrow pre_focus
**2023404**	block2	Control	pre	waa6	Verb	Narrow	r2	253.2795083	3	1	on_focus	6	Narrow on_focus
**2023404**	block2	Control	pre	jyut6	Object	Narrow	r2	179.7917008	4	1	post_focus	6	Narrow post_focus
**2023404**	block2	Control	pre	loeng6	Object	Narrow	r2	294.0971088	5	2	post_focus	6	Narrow post_focus
**2023404**	block2	Control	pre	lok6	Subject	Contrastive	r2	192.5913394	1	1	pre_focus	6	Contrastive pre_focus
**2023404**	block2	Control	pre	lok6	Subject	Contrastive	r2	180.6087793	2	2	pre_focus	6	Contrastive pre_focus
**2023404**	block2	Control	pre	waa6	Verb	Contrastive	r2	241.1043581	3	1	pre_focus	6	Contrastive pre_focus
**2023404**	block2	Control	pre	jyut6	Object	Contrastive	r2	190.3588102	4	1	on_focus	6	Contrastive on_focus
**2023404**	block2	Control	pre	loeng6	Object	Contrastive	r2	299.4242882	5	2	on_focus	6	Contrastive on_focus
**2023404**	block2	Control	pre	ma4	Subject	Contrastive	r2	272.090655	1	1	pre_focus	4	Contrastive pre_focus
**2023404**	block2	Control	pre	ma4	Subject	Contrastive	r2	348.6152271	2	2	pre_focus	4	Contrastive pre_focus
**2023404**	block2	Control	pre	fu4	Verb	Contrastive	r2	244.9070772	3	1	pre_focus	4	Contrastive pre_focus
**2023404**	block2	Control	pre	maang4	Object	Contrastive	r2	494.1406528	4	1	on_focus	4	Contrastive on_focus
**2023404**	block2	Control	pre	Jan-04	Object	Contrastive	r2	279.2658319	5	2	on_focus	4	Contrastive on_focus
**2023404**	block2	Control	pre	ngaa5	Subject	Narrow	r2	271.3690476	1	1	on_focus	5	Narrow on_focus
**2023404**	block2	Control	pre	ngaa5	Subject	Narrow	r2	371.5565935	2	2	on_focus	5	Narrow on_focus
**2023404**	block2	Control	pre	maai5	Verb	Narrow	r2	169.7725585	3	1	post_focus	5	Narrow post_focus
**2023404**	block2	Control	pre	pou5	Object	Narrow	r2	213.8355442	4	1	post_focus	5	Narrow post_focus
**2023404**	block2	Control	pre	pou5	Object	Narrow	r2	333.7854149	5	2	post_focus	5	Narrow post_focus
**2023404**	block2	Control	pre	lok6	Subject	Narrow	r2	149.6012935	1	1	pre_focus	6	Narrow pre_focus
**2023404**	block2	Control	pre	lok6	Subject	Narrow	r2	217.8358319	2	2	pre_focus	6	Narrow pre_focus
**2023404**	block2	Control	pre	waa6	Verb	Narrow	r2	226.7827692	3	1	pre_focus	6	Narrow pre_focus
**2023404**	block2	Control	pre	jyut6	Object	Narrow	r2	202.0244765	4	1	on_focus	6	Narrow on_focus
**2023404**	block2	Control	pre	loeng6	Object	Narrow	r2	332.6795967	5	2	on_focus	6	Narrow on_focus

2023404	block2	Control	pre	lok6	Subject	Narrow	r2	182.0137646	1	1	on_focus	6	Narrow on_focus
2023404	block2	Control	pre	lok6	Subject	Narrow	r2	164.5666415	2	2	on_focus	6	Narrow on_focus
2023404	block2	Control	pre	waa6	Verb	Narrow	r2	229.9688472	3	1	post_focus	6	Narrow post_focus
2023404	block2	Control	pre	jyut6	Object	Narrow	r2	153.0871396	4	1	post_focus	6	Narrow post_focus
2023404	block2	Control	pre	loeng6	Object	Narrow	r2	157.4287776	5	2	post_focus	6	Narrow post_focus
2023404	block2	Control	pre	ma4	Subject	Narrow	r2	251.6178008	1	1	pre_focus	4	Narrow pre_focus
2023404	block2	Control	pre	ma4	Subject	Narrow	r2	390.9165358	2	2	pre_focus	4	Narrow pre_focus
2023404	block2	Control	pre	fu4	Verb	Narrow	r2	167.1565571	3	1	pre_focus	4	Narrow pre_focus
2023404	block2	Control	pre	maang4	Object	Narrow	r2	290.7451041	4	1	on_focus	4	Narrow on_focus
2023404	block2	Control	pre	Jan-04	Object	Narrow	r2	203.1438403	5	2	on_focus	4	Narrow on_focus
2023404	block2	Control	pre	ma4	Subject	Narrow	r2	173.5511149	1	1	pre_focus	4	Narrow pre_focus
2023404	block2	Control	pre	ma4	Subject	Narrow	r2	353.5425091	2	2	pre_focus	4	Narrow pre_focus
2023404	block2	Control	pre	fu4	Verb	Narrow	r2	119.8441043	3	1	on_focus	4	Narrow on_focus
2023404	block2	Control	pre	maang4	Object	Narrow	r2	503.1344846	4	1	post_focus	4	Narrow post_focus
2023404	block2	Control	pre	Jan-04	Object	Narrow	r2	174.6155432	5	2	post_focus	4	Narrow post_focus
2023404	block2	Control	pre	ngaa5	Subject	Narrow	r2	288.5696064	1	1	pre_focus	5	Narrow pre_focus
2023404	block2	Control	pre	ngaa5	Subject	Narrow	r2	312.6997455	2	2	pre_focus	5	Narrow pre_focus
2023404	block2	Control	pre	maai5	Verb	Narrow	r2	506.9833602	3	1	on_focus	5	Narrow on_focus
2023404	block2	Control	pre	pou5	Object	Narrow	r2	222.6391069	4	1	post_focus	5	Narrow post_focus
2023404	block2	Control	pre	pou5	Object	Narrow	r2	282.9903628	5	2	post_focus	5	Narrow post_focus
2023404	block2	Control	pre	ngaa5	Subject	Broad	r2	249.4930896	1	1	broad_focus	5	Broad focus
2023404	block2	Control	pre	ngaa5	Subject	Broad	r2	331.6090996	2	2	broad_focus	5	Broad focus
2023404	block2	Control	pre	maai5	Verb	Broad	r2	391.3703573	3	1	broad_focus	5	Broad focus
2023404	block2	Control	pre	pou5	Object	Broad	r2	245.5128676	4	1	broad_focus	5	Broad focus
2023404	block2	Control	pre	pou5	Object	Broad	r2	121.3756144	5	2	broad_focus	5	Broad focus
2023404	block2	Control	pre	ma4	Subject	Contrastive	r2	291.1569381	1	1	pre_focus	4	Contrastive pre_focus
2023404	block2	Control	pre	ma4	Subject	Contrastive	r2	405.7442013	2	2	pre_focus	4	Contrastive pre_focus
2023404	block2	Control	pre	fu4	Verb	Contrastive	r2	252.2011145	3	1	on_focus	4	Contrastive on_focus
2023404	block2	Control	pre	maang4	Object	Contrastive	r2	365.7709751	4	1	post_focus	4	Contrastive post_focus
2023404	block2	Control	pre	Jan-04	Object	Contrastive	r2	145.1318972	5	2	post_focus	4	Contrastive post_focus
2023404	block2	Control	pre	ngaa5	Subject	Contrastive	r2	291.7494059	1	1	on_focus	5	Contrastive on_focus
2023404	block2	Control	pre	ngaa5	Subject	Contrastive	r2	266.3582743	2	2	on_focus	5	Contrastive on_focus
2023404	block2	Control	pre	maai5	Verb	Contrastive	r2	512.7011482	3	1	post_focus	5	Contrastive post_focus
2023404	block2	Control	pre	pou5	Object	Contrastive	r2	401.6890725	4	1	post_focus	5	Contrastive post_focus
2023404	block2	Control	pre	pou5	Object	Contrastive	r2	154.861489	5	2	post_focus	5	Contrastive post_focus
2023404	block2	Control	pre	ma4	Subject	Narrow	r2	243.7704077	1	1	on_focus	4	Narrow on_focus
2023404	block2	Control	pre	ma4	Subject	Narrow	r2	294.9792139	2	2	on_focus	4	Narrow on_focus
2023404	block2	Control	pre	fu4	Verb	Narrow	r2	146.3385556	3	1	post_focus	4	Narrow post_focus
2023404	block2	Control	pre	maang4	Object	Narrow	r2	322.8769934	4	1	post_focus	4	Narrow post_focus
2023404	block2	Control	pre	Jan-04	Object	Narrow	r2	125.6239058	5	2	post_focus	4	Narrow post_focus
2023404	block2	Control	pre	lok6	Subject	Broad	r2	159.8608931	1	1	broad_focus	6	Broad focus
2023404	block2	Control	pre	lok6	Subject	Broad	r2	169.5695399	2	2	broad_focus	6	Broad focus
2023404	block2	Control	pre	waa6	Verb	Broad	r2	237.852267	3	1	broad_focus	6	Broad focus
2023404	block2	Control	pre	jyut6	Object	Broad	r2	167.8550912	4	1	broad_focus	6	Broad focus
2023404	block2	Control	pre	loeng6	Object	Broad	r2	256.4008181	5	2	broad_focus	6	Broad focus
2023404	block3	Control	post	suk1	Subject	Narrow	r1	59.30103035	1	1	pre_focus	1	Narrow pre_focus
2023404	block3	Control	post	suk1	Subject	Narrow	r1	111.6907357	2	2	pre_focus	1	Narrow pre_focus
2023404	block3	Control	post	sei2	Verb	Narrow	r1	411.2073641	3	1	pre_focus	2	Narrow pre_focus
2023404	block3	Control	post	svy2	Object	Narrow	r1	251.720143	4	1	on_focus	2	Narrow on_focus
2023404	block3	Control	post	kwo2	Object	Narrow	r1	262.6999654	5	2	on_focus	2	Narrow on_focus
2023404	block3	Control	post	piu2	Subject	Contrastive	r1	358.6859184	1	1	on_focus	2	Contrastive on_focus
2023404	block3	Control	post	tse2	Subject	Contrastive	r1	264.3099521	2	2	on_focus	2	Contrastive on_focus
2023404	block3	Control	post	tsap1	Verb	Contrastive	r1	112.602132	3	1	post_focus	1	Contrastive post_focus
2023404	block3	Control	post	sy1	Object	Contrastive	r1	370.9037421	4	1	post_focus	1	Contrastive post_focus
2023404	block3	Control	post	pau1	Object	Contrastive	r1	243.8328166	5	2	post_focus	1	Contrastive post_focus
2023404	block3	Control	post	piu2	Subject	Contrastive	r1	287.102063	1	1	pre_focus	2	Contrastive pre_focus
2023404	block3	Control	post	tse2	Subject	Contrastive	r1	258.8607397	2	2	pre_focus	2	Contrastive pre_focus
2023404	block3	Control	post	tsap1	Verb	Contrastive	r1	147.2482326	3	1	on_focus	1	Contrastive on_focus
2023404	block3	Control	post	sy1	Object	Contrastive	r1	196.8233618	4	1	post_focus	1	Contrastive post_focus
2023404	block3	Control	post	pau1	Object	Contrastive	r1	236.6926314	5	2	post_focus	1	Contrastive post_focus
2023404	block3	Control	post	piu2	Subject	Contrastive	r1	267.0972033	1	1	pre_focus	2	Contrastive pre_focus
2023404	block3	Control	post	tse2	Subject	Contrastive	r1	242.003675	2	2	pre_focus	2	Contrastive pre_focus
2023404	block3	Control	post	tsap1	Verb	Contrastive	r1	104.5151533	3	1	pre_focus	1	Contrastive pre_focus
2023404	block3	Control	post	sy1	Object	Contrastive	r1	371.2429903	4	1	on_focus	1	Contrastive on_focus
2023404	block3	Control	post	pau1	Object	Contrastive	r1	310.5598846	5	2	on_focus	1	Contrastive on_focus
2023404	block3	Control	post	bui3	Subject	Narrow	r1	292.9841851	1	1	pre_focus	3	Narrow pre_focus

2023404	block3	Control	post	bui3	Subject	Narrow	r1	281.2310249	2	2	pre_focus	3	Narrow pre_focus
2023404	block3	Control	post	tsv1	Verb	Narrow	r1	357.5053706	3	1	on_focus	1	Narrow on_focus
2023404	block3	Control	post	fug1	Object	Narrow	r1	400.4354772	4	1	post_focus	1	Narrow post_focus
2023404	block3	Control	post	tshe1	Object	Narrow	r1	141.9140552	5	2	post_focus	1	Narrow post_focus
2023404	block3	Control	post	suk1	Subject	Contrastive	r1	52.67106365	1	1	pre_focus	1	Contrastive pre_focus
2023404	block3	Control	post	suk1	Subject	Contrastive	r1	264.861966	2	2	pre_focus	1	Contrastive pre_focus
2023404	block3	Control	post	sei2	Verb	Contrastive	r1	324.329554	3	1	pre_focus	2	Contrastive pre_focus
2023404	block3	Control	post	svy2	Object	Contrastive	r1	451.5708743	4	1	on_focus	2	Contrastive on_focus
2023404	block3	Control	post	kwo2	Object	Contrastive	r1	306.9672106	5	2	on_focus	2	Contrastive on_focus
2023404	block3	Control	post	bui3	Subject	Contrastive	r1	260.2748631	1	1	on_focus	3	Contrastive on_focus
2023404	block3	Control	post	bui3	Subject	Contrastive	r1	241.408308	2	2	on_focus	3	Contrastive on_focus
2023404	block3	Control	post	tsv1	Verb	Contrastive	r1	288.1967451	3	1	post_focus	1	Contrastive post_focus
2023404	block3	Control	post	fug1	Object	Contrastive	r1	321.8495843	4	1	post_focus	1	Contrastive post_focus
2023404	block3	Control	post	tshe1	Object	Contrastive	r1	267.8075811	5	2	post_focus	1	Contrastive post_focus
2023404	block3	Control	post	suk1	Subject	Narrow	r1	101.0963255	1	1	on_focus	1	Narrow on_focus
2023404	block3	Control	post	suk1	Subject	Narrow	r1	135.1892247	2	2	on_focus	1	Narrow on_focus
2023404	block3	Control	post	sei2	Verb	Narrow	r1	352.5379598	3	1	post_focus	2	Narrow post_focus
2023404	block3	Control	post	svy2	Object	Narrow	r1	190.156587	4	1	post_focus	2	Narrow post_focus
2023404	block3	Control	post	kwo2	Object	Narrow	r1	180.2785779	5	2	post_focus	2	Narrow post_focus
2023404	block3	Control	post	suk1	Subject	Narrow	r1	75.66153763	1	1	pre_focus	1	Narrow pre_focus
2023404	block3	Control	post	suk1	Subject	Narrow	r1	116.2586025	2	2	pre_focus	1	Narrow pre_focus
2023404	block3	Control	post	sei2	Verb	Narrow	r1	403.3560091	3	1	on_focus	2	Narrow on_focus
2023404	block3	Control	post	svy2	Object	Narrow	r1	321.2213094	4	1	post_focus	2	Narrow post_focus
2023404	block3	Control	post	kwo2	Object	Narrow	r1	177.931049	5	2	post_focus	2	Narrow post_focus
2023404	block3	Control	post	bui3	Subject	Contrastive	r1	248.3551433	1	1	pre_focus	3	Contrastive pre_focus
2023404	block3	Control	post	bui3	Subject	Contrastive	r1	231.142629	2	2	pre_focus	3	Contrastive pre_focus
2023404	block3	Control	post	tsv1	Verb	Contrastive	r1	272.1612175	3	1	pre_focus	1	Contrastive pre_focus
2023404	block3	Control	post	fug1	Object	Contrastive	r1	389.9304985	4	1	on_focus	1	Contrastive on_focus
2023404	block3	Control	post	tshe1	Object	Contrastive	r1	228.4332977	5	2	on_focus	1	Contrastive on_focus
2023404	block3	Control	post	bui3	Subject	Broad	r1	348.7842971	1	1	broad_focus	3	Broad focus
2023404	block3	Control	post	bui3	Subject	Broad	r1	238.8758722	2	2	broad_focus	3	Broad focus
2023404	block3	Control	post	tsv1	Verb	Broad	r1	291.8189498	3	1	broad_focus	1	Broad focus
2023404	block3	Control	post	fug1	Object	Broad	r1	379.4589039	4	1	broad_focus	1	Broad focus
2023404	block3	Control	post	tshe1	Object	Broad	r1	221.5037908	5	2	broad_focus	1	Broad focus
2023404	block3	Control	post	piu2	Subject	Narrow	r1	257.4297857	1	1	pre_focus	2	Narrow pre_focus
2023404	block3	Control	post	tse2	Subject	Narrow	r1	244.5543462	2	2	pre_focus	2	Narrow pre_focus
2023404	block3	Control	post	tsap1	Verb	Narrow	r1	122.1442177	3	1	pre_focus	1	Narrow pre_focus
2023404	block3	Control	post	sy1	Object	Narrow	r1	413.2813681	4	1	on_focus	1	Narrow on_focus
2023404	block3	Control	post	pau1	Object	Narrow	r1	293.8774376	5	2	on_focus	1	Narrow on_focus
2023404	block3	Control	post	suk1	Subject	Contrastive	r1	113.1083815	1	1	on_focus	1	Contrastive on_focus
2023404	block3	Control	post	suk1	Subject	Contrastive	r1	117.8188557	2	2	on_focus	1	Contrastive on_focus
2023404	block3	Control	post	sei2	Verb	Contrastive	r1	333.9628312	3	1	post_focus	2	Contrastive post_focus
2023404	block3	Control	post	svy2	Object	Contrastive	r1	272.2221194	4	1	post_focus	2	Contrastive post_focus
2023404	block3	Control	post	kwo2	Object	Contrastive	r1	192.3241437	5	2	post_focus	2	Contrastive post_focus
2023404	block3	Control	post	piu2	Subject	Broad	r1	316.5499433	1	1	broad_focus	2	Broad focus
2023404	block3	Control	post	tse2	Subject	Broad	r1	278.9871504	2	2	broad_focus	2	Broad focus
2023404	block3	Control	post	tsap1	Verb	Broad	r1	261.5594258	3	1	broad_focus	1	Broad focus
2023404	block3	Control	post	sy1	Object	Broad	r1	423.888701	4	1	broad_focus	1	Broad focus
2023404	block3	Control	post	pau1	Object	Broad	r1	340.9812376	5	2	broad_focus	1	Broad focus
2023404	block3	Control	post	piu2	Subject	Narrow	r1	220.9569575	1	1	pre_focus	2	Narrow pre_focus
2023404	block3	Control	post	tse2	Subject	Narrow	r1	228.2314683	2	2	pre_focus	2	Narrow pre_focus
2023404	block3	Control	post	tsap1	Verb	Narrow	r1	110.8534549	3	1	on_focus	1	Narrow on_focus
2023404	block3	Control	post	sy1	Object	Narrow	r1	348.0767633	4	1	post_focus	1	Narrow post_focus
2023404	block3	Control	post	pau1	Object	Narrow	r1	265.1999588	5	2	post_focus	1	Narrow post_focus
2023404	block3	Control	post	bui3	Subject	Narrow	r1	243.2000407	1	1	on_focus	3	Narrow on_focus
2023404	block3	Control	post	bui3	Subject	Narrow	r1	226.3416809	2	2	on_focus	3	Narrow on_focus
2023404	block3	Control	post	tsv1	Verb	Narrow	r1	230.9072164	3	1	post_focus	1	Narrow post_focus
2023404	block3	Control	post	fug1	Object	Narrow	r1	347.3539549	4	1	post_focus	1	Narrow post_focus
2023404	block3	Control	post	tshe1	Object	Narrow	r1	195.8568189	5	2	post_focus	1	Narrow post_focus
2023404	block3	Control	post	bui3	Subject	Contrastive	r1	220.1401394	1	1	pre_focus	3	Contrastive pre_focus
2023404	block3	Control	post	bui3	Subject	Contrastive	r1	289.2688874	2	2	pre_focus	3	Contrastive pre_focus
2023404	block3	Control	post	tsv1	Verb	Contrastive	r1	394.3282479	3	1	on_focus	1	Contrastive on_focus
2023404	block3	Control	post	fug1	Object	Contrastive	r1	314.3671428	4	1	post_focus	1	Contrastive post_focus
2023404	block3	Control	post	tshe1	Object	Contrastive	r1	206.0605934	5	2	post_focus	1	Contrastive post_focus
2023404	block3	Control	post	piu2	Subject	Narrow	r1	333.8128769	1	1	on_focus	2	Narrow on_focus
2023404	block3	Control	post	tse2	Subject	Narrow	r1	242.1159065	2	2	on_focus	2	Narrow on_focus

2023404	block3	Control	post	tsap1	Verb	Narrow	r1	255.8136754	3	1	post_focus	1	Narrow post_focus
2023404	block3	Control	post	sy1	Object	Narrow	r1	294.660767	4	1	post_focus	1	Narrow post_focus
2023404	block3	Control	post	pau1	Object	Narrow	r1	345.4480638	5	2	post_focus	1	Narrow post_focus
2023404	block3	Control	post	bui3	Subject	Narrow	r1	278.7359393	1	1	pre_focus	3	Narrow pre_focus
2023404	block3	Control	post	bui3	Subject	Narrow	r1	271.6193434	2	2	pre_focus	3	Narrow pre_focus
2023404	block3	Control	post	tsv1	Verb	Narrow	r1	275.7232264	3	1	pre_focus	1	Narrow pre_focus
2023404	block3	Control	post	fug1	Object	Narrow	r1	385.0256621	4	1	on_focus	1	Narrow on_focus
2023404	block3	Control	post	tshe1	Object	Narrow	r1	222.5374442	5	2	on_focus	1	Narrow on_focus
2023404	block3	Control	post	suk1	Subject	Broad	r1	77.9122807	1	1	broad_focus	1	Broad focus
2023404	block3	Control	post	suk1	Subject	Broad	r1	112.6040467	2	2	broad_focus	1	Broad focus
2023404	block3	Control	post	sei2	Verb	Broad	r1	326.384402	3	1	broad_focus	2	Broad focus
2023404	block3	Control	post	svy2	Object	Broad	r1	408.7070225	4	1	broad_focus	2	Broad focus
2023404	block3	Control	post	kwo2	Object	Broad	r1	271.6900564	5	2	broad_focus	2	Broad focus
2023404	block3	Control	post	suk1	Subject	Contrastive	r1	65.70364556	1	1	pre_focus	1	Contrastive pre_focus
2023404	block3	Control	post	suk1	Subject	Contrastive	r1	117.3723309	2	2	pre_focus	1	Contrastive pre_focus
2023404	block3	Control	post	sei2	Verb	Contrastive	r1	332.7522582	3	1	on_focus	2	Contrastive on_focus
2023404	block3	Control	post	svy2	Object	Contrastive	r1	427.6912505	4	1	post_focus	2	Contrastive post_focus
2023404	block3	Control	post	kwo2	Object	Contrastive	r1	312.9945991	5	2	post_focus	2	Contrastive post_focus
2023404	block3	Control	post	bui3	Subject	Contrastive	r2	279.6571191	1	1	pre_focus	3	Contrastive pre_focus
2023404	block3	Control	post	bui3	Subject	Contrastive	r2	298.87078	2	2	pre_focus	3	Contrastive pre_focus
2023404	block3	Control	post	tsv1	Verb	Contrastive	r2	318.0760202	3	1	on_focus	1	Contrastive on_focus
2023404	block3	Control	post	fug1	Object	Contrastive	r2	416.8005909	4	1	post_focus	1	Contrastive post_focus
2023404	block3	Control	post	tshe1	Object	Contrastive	r2	200.9204334	5	2	post_focus	1	Contrastive post_focus
2023404	block3	Control	post	piu2	Subject	Narrow	r2	326.0438937	1	1	pre_focus	2	Narrow pre_focus
2023404	block3	Control	post	tse2	Subject	Narrow	r2	315.4383232	2	2	pre_focus	2	Narrow pre_focus
2023404	block3	Control	post	tsap1	Verb	Narrow	r2	224.9969475	3	1	pre_focus	1	Narrow pre_focus
2023404	block3	Control	post	sy1	Object	Narrow	r2	388.6457546	4	1	on_focus	1	Narrow on_focus
2023404	block3	Control	post	pau1	Object	Narrow	r2	336.5537608	5	2	on_focus	1	Narrow on_focus
2023404	block3	Control	post	piu2	Subject	Contrastive	r2	321.1682573	1	1	pre_focus	2	Contrastive pre_focus
2023404	block3	Control	post	tse2	Subject	Contrastive	r2	251.3544281	2	2	pre_focus	2	Contrastive pre_focus
2023404	block3	Control	post	tsap1	Verb	Contrastive	r2	225.9069076	3	1	pre_focus	1	Contrastive pre_focus
2023404	block3	Control	post	sy1	Object	Contrastive	r2	385.4402608	4	1	on_focus	1	Contrastive on_focus
2023404	block3	Control	post	pau1	Object	Contrastive	r2	355.9695838	5	2	on_focus	1	Contrastive on_focus
2023404	block3	Control	post	suk1	Subject	Narrow	r2	86.56026513	1	1	on_focus	1	Narrow on_focus
2023404	block3	Control	post	suk1	Subject	Narrow	r2	157.3163997	2	2	on_focus	1	Narrow on_focus
2023404	block3	Control	post	sei2	Verb	Narrow	r2	281.113544	3	1	post_focus	2	Narrow post_focus
2023404	block3	Control	post	svy2	Object	Narrow	r2	437.3590325	4	1	post_focus	2	Narrow post_focus
2023404	block3	Control	post	kwo2	Object	Narrow	r2	177.4184846	5	2	post_focus	2	Narrow post_focus
2023404	block3	Control	post	piu2	Subject	Broad	r2	323.1321936	1	1	broad_focus	2	Broad focus
2023404	block3	Control	post	tse2	Subject	Broad	r2	323.1391201	2	2	broad_focus	2	Broad focus
2023404	block3	Control	post	tsap1	Verb	Broad	r2	213.4504913	3	1	broad_focus	1	Broad focus
2023404	block3	Control	post	sy1	Object	Broad	r2	400.9437229	4	1	broad_focus	1	Broad focus
2023404	block3	Control	post	pau1	Object	Broad	r2	310.0125526	5	2	broad_focus	1	Broad focus
2023404	block3	Control	post	suk1	Subject	Contrastive	r2	74.8749616	1	1	on_focus	1	Contrastive on_focus
2023404	block3	Control	post	suk1	Subject	Contrastive	r2	132.3780801	2	2	on_focus	1	Contrastive on_focus
2023404	block3	Control	post	sei2	Verb	Contrastive	r2	335.0833087	3	1	post_focus	2	Contrastive post_focus
2023404	block3	Control	post	svy2	Object	Contrastive	r2	199.2803888	4	1	post_focus	2	Contrastive post_focus
2023404	block3	Control	post	kwo2	Object	Contrastive	r2	228.7540942	5	2	post_focus	2	Contrastive post_focus
2023404	block3	Control	post	piu2	Subject	Narrow	r2	340.8077005	1	1	on_focus	2	Narrow on_focus
2023404	block3	Control	post	tse2	Subject	Narrow	r2	325.6923556	2	2	on_focus	2	Narrow on_focus
2023404	block3	Control	post	tsap1	Verb	Narrow	r2	118.5894113	3	1	post_focus	1	Narrow post_focus
2023404	block3	Control	post	sy1	Object	Narrow	r2	404.5555556	4	1	post_focus	1	Narrow post_focus
2023404	block3	Control	post	pau1	Object	Narrow	r2	300.0722384	5	2	post_focus	1	Narrow post_focus
2023404	block3	Control	post	suk1	Subject	Contrastive	r2	57.83664307	1	1	pre_focus	1	Contrastive pre_focus
2023404	block3	Control	post	suk1	Subject	Contrastive	r2	127.4603326	2	2	pre_focus	1	Contrastive pre_focus
2023404	block3	Control	post	sei2	Verb	Contrastive	r2	304.5568709	3	1	pre_focus	2	Contrastive pre_focus
2023404	block3	Control	post	svy2	Object	Contrastive	r2	218.9579284	4	1	on_focus	2	Contrastive on_focus
2023404	block3	Control	post	kwo2	Object	Contrastive	r2	337.8522838	5	2	on_focus	2	Contrastive on_focus
2023404	block3	Control	post	bui3	Subject	Narrow	r2	309.521959	1	1	pre_focus	3	Narrow pre_focus
2023404	block3	Control	post	bui3	Subject	Narrow	r2	269.1010595	2	2	pre_focus	3	Narrow pre_focus
2023404	block3	Control	post	tsv1	Verb	Narrow	r2	313.9122182	3	1	on_focus	1	Narrow on_focus
2023404	block3	Control	post	fug1	Object	Narrow	r2	444.8628658	4	1	post_focus	1	Narrow post_focus
2023404	block3	Control	post	tshe1	Object	Narrow	r2	219.2301151	5	2	post_focus	1	Narrow post_focus
2023404	block3	Control	post	bui3	Subject	Narrow	r2	306.5459786	1	1	on_focus	3	Narrow on_focus
2023404	block3	Control	post	bui3	Subject	Narrow	r2	286.5295722	2	2	on_focus	3	Narrow on_focus
2023404	block3	Control	post	tsv1	Verb	Narrow	r2	287.3287556	3	1	post_focus	1	Narrow post_focus

**2023404**	block3	Control	post	fug1	Object	Narrow	r2	392.6435738	4	1	post_focus	1	Narrow post_focus
**2023404**	block3	Control	post	tshe1	Object	Narrow	r2	241.6524792	5	2	post_focus	1	Narrow post_focus
**2023404**	block3	Control	post	bui3	Subject	Broad	r2	251.0863883	1	1	broad_focus	3	Broad focus
**2023404**	block3	Control	post	bui3	Subject	Broad	r2	300.9057337	2	2	broad_focus	3	Broad focus
**2023404**	block3	Control	post	tsv1	Verb	Broad	r2	274.9385246	3	1	broad_focus	1	Broad focus
**2023404**	block3	Control	post	fug1	Object	Broad	r2	385.9822042	4	1	broad_focus	1	Broad focus
**2023404**	block3	Control	post	tshe1	Object	Broad	r2	228.7462858	5	2	broad_focus	1	Broad focus
**2023404**	block3	Control	post	bui3	Subject	Narrow	r2	277.1465628	1	1	pre_focus	3	Narrow pre_focus
**2023404**	block3	Control	post	bui3	Subject	Narrow	r2	236.3282313	2	2	pre_focus	3	Narrow pre_focus
**2023404**	block3	Control	post	tsv1	Verb	Narrow	r2	259.0017994	3	1	pre_focus	1	Narrow pre_focus
**2023404**	block3	Control	post	fug1	Object	Narrow	r2	373.4439987	4	1	on_focus	1	Narrow on_focus
**2023404**	block3	Control	post	tshe1	Object	Narrow	r2	276.6942702	5	2	on_focus	1	Narrow on_focus
**2023404**	block3	Control	post	suk1	Subject	Broad	r2	76.1686726	1	1	broad_focus	1	Broad focus
**2023404**	block3	Control	post	suk1	Subject	Broad	r2	252.046564	2	2	broad_focus	1	Broad focus
**2023404**	block3	Control	post	sei2	Verb	Broad	r2	359.3727573	3	1	broad_focus	2	Broad focus
**2023404**	block3	Control	post	svy2	Object	Broad	r2	279.5043732	4	1	broad_focus	2	Broad focus
**2023404**	block3	Control	post	kwo2	Object	Broad	r2	240.895463	5	2	broad_focus	2	Broad focus
**2023404**	block3	Control	post	suk1	Subject	Narrow	r2	97.30532595	1	1	pre_focus	1	Narrow pre_focus
**2023404**	block3	Control	post	suk1	Subject	Narrow	r2	434.250475	2	2	pre_focus	1	Narrow pre_focus
**2023404**	block3	Control	post	sei2	Verb	Narrow	r2	387.0850171	3	1	on_focus	2	Narrow on_focus
**2023404**	block3	Control	post	svy2	Object	Narrow	r2	343.3630952	4	1	post_focus	2	Narrow post_focus
**2023404**	block3	Control	post	kwo2	Object	Narrow	r2	130.0484436	5	2	post_focus	2	Narrow post_focus
**2023404**	block3	Control	post	piu2	Subject	Contrastive	r2	345.8992255	1	1	pre_focus	2	Contrastive pre_focus
**2023404**	block3	Control	post	tse2	Subject	Contrastive	r2	273.2510053	2	2	pre_focus	2	Contrastive pre_focus
**2023404**	block3	Control	post	tsap1	Verb	Contrastive	r2	130.9889379	3	1	on_focus	1	Contrastive on_focus
**2023404**	block3	Control	post	sy1	Object	Contrastive	r2	400.4241677	4	1	post_focus	1	Contrastive post_focus
**2023404**	block3	Control	post	pau1	Object	Contrastive	r2	301.8165424	5	2	post_focus	1	Contrastive post_focus
**2023404**	block3	Control	post	bui3	Subject	Contrastive	r2	332.7512329	1	1	on_focus	3	Contrastive on_focus
**2023404**	block3	Control	post	bui3	Subject	Contrastive	r2	274.8702084	2	2	on_focus	3	Contrastive on_focus
**2023404**	block3	Control	post	tsv1	Verb	Contrastive	r2	317.1195502	3	1	post_focus	1	Contrastive post_focus
**2023404**	block3	Control	post	fug1	Object	Contrastive	r2	435.9160299	4	1	post_focus	1	Contrastive post_focus
**2023404**	block3	Control	post	tshe1	Object	Contrastive	r2	199.6733277	5	2	post_focus	1	Contrastive post_focus
**2023404**	block3	Control	post	suk1	Subject	Contrastive	r2	85.78394721	1	1	pre_focus	1	Contrastive pre_focus
**2023404**	block3	Control	post	suk1	Subject	Contrastive	r2	173.8873133	2	2	pre_focus	1	Contrastive pre_focus
**2023404**	block3	Control	post	sei2	Verb	Contrastive	r2	345.9434858	3	1	on_focus	2	Contrastive on_focus
**2023404**	block3	Control	post	svy2	Object	Contrastive	r2	284.4352825	4	1	post_focus	2	Contrastive post_focus
**2023404**	block3	Control	post	kwo2	Object	Contrastive	r2	180.6982237	5	2	post_focus	2	Contrastive post_focus
**2023404**	block3	Control	post	bui3	Subject	Contrastive	r2	259.9953664	1	1	pre_focus	3	Contrastive pre_focus
**2023404**	block3	Control	post	bui3	Subject	Contrastive	r2	274.2110716	2	2	pre_focus	3	Contrastive pre_focus
**2023404**	block3	Control	post	tsv1	Verb	Contrastive	r2	282.1010973	3	1	pre_focus	1	Contrastive pre_focus
**2023404**	block3	Control	post	fug1	Object	Contrastive	r2	403.7862182	4	1	on_focus	1	Contrastive on_focus
**2023404**	block3	Control	post	tshe1	Object	Contrastive	r2	267.1400333	5	2	on_focus	1	Contrastive on_focus
**2023404**	block3	Control	post	piu2	Subject	Contrastive	r2	461.9600251	1	1	on_focus	2	Contrastive on_focus
**2023404**	block3	Control	post	tse2	Subject	Contrastive	r2	297.1618035	2	2	on_focus	2	Contrastive on_focus
**2023404**	block3	Control	post	tsap1	Verb	Contrastive	r2	112.7140023	3	1	post_focus	1	Contrastive post_focus
**2023404**	block3	Control	post	sy1	Object	Contrastive	r2	400.821675	4	1	post_focus	1	Contrastive post_focus
**2023404**	block3	Control	post	pau1	Object	Contrastive	r2	295.494655	5	2	post_focus	1	Contrastive post_focus
**2023404**	block3	Control	post	suk1	Subject	Narrow	r2	99.569161	1	1	pre_focus	1	Narrow pre_focus
**2023404**	block3	Control	post	suk1	Subject	Narrow	r2	120.2693603	2	2	pre_focus	1	Narrow pre_focus
**2023404**	block3	Control	post	sei2	Verb	Narrow	r2	416.5343036	3	1	pre_focus	2	Narrow pre_focus
**2023404**	block3	Control	post	svy2	Object	Narrow	r2	384.8098702	4	1	on_focus	2	Narrow on_focus
**2023404**	block3	Control	post	kwo2	Object	Narrow	r2	291.7146669	5	2	on_focus	2	Narrow on_focus
**2023404**	block3	Control	post	piu2	Subject	Narrow	r2	336.4456914	1	1	pre_focus	2	Narrow pre_focus
**2023404**	block3	Control	post	tse2	Subject	Narrow	r2	258.1891595	2	2	pre_focus	2	Narrow pre_focus
**2023404**	block3	Control	post	tsap1	Verb	Narrow	r2	116.6562126	3	1	on_focus	1	Narrow on_focus
**2023404**	block3	Control	post	sy1	Object	Narrow	r2	357.4358974	4	1	post_focus	1	Narrow post_focus
**2023404**	block3	Control	post	pau1	Object	Narrow	r2	284.9076497	5	2	post_focus	1	Narrow post_focus
**2023404**	block3	Control	pre	bui3	Subject	Narrow	r1	195.6235525	1	1	pre_focus	3	Narrow pre_focus
**2023404**	block3	Control	pre	bui3	Subject	Narrow	r1	203.5971817	2	2	pre_focus	3	Narrow pre_focus
**2023404**	block3	Control	pre	tsv1	Verb	Narrow	r1	232.0421186	3	1	pre_focus	1	Narrow pre_focus
**2023404**	block3	Control	pre	fug1	Object	Narrow	r1	413.9440646	4	1	on_focus	1	Narrow on_focus
**2023404**	block3	Control	pre	tshe1	Object	Narrow	r1	202.0692889	5	2	on_focus	1	Narrow on_focus
**2023404**	block3	Control	pre	piu2	Subject	Contrastive	r1	261.5979725	1	1	pre_focus	2	Contrastive pre_focus
**2023404**	block3	Control	pre	tse2	Subject	Contrastive	r1	253.2331951	2	2	pre_focus	2	Contrastive pre_focus
**2023404**	block3	Control	pre	tsap1	Verb	Contrastive	r1	99.43303786	3	1	on_focus	1	Contrastive on_focus
**2023404**	block3	Control	pre	sy1	Object	Contrastive	r1	454.5587283	4	1	post_focus	1	Contrastive post_focus

**2023404**	block3	Control	pre	pau1	Object	Contrastive	r1	311.7136315	5	2	post_focus	1	Contrastive post_focus
**2023404**	block3	Control	pre	bui3	Subject	Narrow	r1	295.138322	1	1	pre_focus	3	Narrow pre_focus
**2023404**	block3	Control	pre	bui3	Subject	Narrow	r1	305.8492628	2	2	pre_focus	3	Narrow pre_focus
**2023404**	block3	Control	pre	tsv1	Verb	Narrow	r1	401.3855141	3	1	on_focus	1	Narrow on_focus
**2023404**	block3	Control	pre	fug1	Object	Narrow	r1	460.1880373	4	1	post_focus	1	Narrow post_focus
**2023404**	block3	Control	pre	tshe1	Object	Narrow	r1	115.5658557	5	2	post_focus	1	Narrow post_focus
**2023404**	block3	Control	pre	piu2	Subject	Narrow	r1	232.3810199	1	1	pre_focus	2	Narrow pre_focus
**2023404**	block3	Control	pre	tse2	Subject	Narrow	r1	358.0164892	2	2	pre_focus	2	Narrow pre_focus
**2023404**	block3	Control	pre	tsap1	Verb	Narrow	r1	283.1139178	3	1	on_focus	1	Narrow on_focus
**2023404**	block3	Control	pre	sy1	Object	Narrow	r1	275.5247438	4	1	post_focus	1	Narrow post_focus
**2023404**	block3	Control	pre	pau1	Object	Narrow	r1	379.4728886	5	2	post_focus	1	Narrow post_focus
**2023404**	block3	Control	pre	suk1	Subject	Narrow	r1	88.31519274	1	1	pre_focus	1	Narrow pre_focus
**2023404**	block3	Control	pre	suk1	Subject	Narrow	r1	224.1643827	2	2	pre_focus	1	Narrow pre_focus
**2023404**	block3	Control	pre	sei2	Verb	Narrow	r1	371.8331579	3	1	on_focus	2	Narrow on_focus
**2023404**	block3	Control	pre	svy2	Object	Narrow	r1	378.6059399	4	1	post_focus	2	Narrow post_focus
**2023404**	block3	Control	pre	kwo2	Object	Narrow	r1	272.9363452	5	2	post_focus	2	Narrow post_focus
**2023404**	block3	Control	pre	bui3	Subject	Contrastive	r1	297.1362953	1	1	on_focus	3	Contrastive on_focus
**2023404**	block3	Control	pre	bui3	Subject	Contrastive	r1	249.367001	2	2	on_focus	3	Contrastive on_focus
**2023404**	block3	Control	pre	tsv1	Verb	Contrastive	r1	379.1462585	3	1	post_focus	1	Contrastive post_focus
**2023404**	block3	Control	pre	fug1	Object	Contrastive	r1	392.9767896	4	1	post_focus	1	Contrastive post_focus
**2023404**	block3	Control	pre	tshe1	Object	Contrastive	r1	240.4995798	5	2	post_focus	1	Contrastive post_focus
**2023404**	block3	Control	pre	piu2	Subject	Narrow	r1	275.742158	1	1	on_focus	2	Narrow on_focus
**2023404**	block3	Control	pre	tse2	Subject	Narrow	r1	238.0656805	2	2	on_focus	2	Narrow on_focus
**2023404**	block3	Control	pre	tsap1	Verb	Narrow	r1	261.4051447	3	1	post_focus	1	Narrow post_focus
**2023404**	block3	Control	pre	sy1	Object	Narrow	r1	343.7143926	4	1	post_focus	1	Narrow post_focus
**2023404**	block3	Control	pre	pau1	Object	Narrow	r1	260.6440989	5	2	post_focus	1	Narrow post_focus
**2023404**	block3	Control	pre	suk1	Subject	Narrow	r1	65.30899471	1	1	pre_focus	1	Narrow pre_focus
**2023404**	block3	Control	pre	suk1	Subject	Narrow	r1	187.8521038	2	2	pre_focus	1	Narrow pre_focus
**2023404**	block3	Control	pre	sei2	Verb	Narrow	r1	302.0329321	3	1	pre_focus	2	Narrow pre_focus
**2023404**	block3	Control	pre	svy2	Object	Narrow	r1	381.047997	4	1	on_focus	2	Narrow on_focus
**2023404**	block3	Control	pre	kwo2	Object	Narrow	r1	252.5914264	5	2	on_focus	2	Narrow on_focus
**2023404**	block3	Control	pre	piu2	Subject	Contrastive	r1	350.3976663	1	1	on_focus	2	Contrastive on_focus
**2023404**	block3	Control	pre	tse2	Subject	Contrastive	r1	221.7291488	2	2	on_focus	2	Contrastive on_focus
**2023404**	block3	Control	pre	tsap1	Verb	Contrastive	r1	112.8129103	3	1	post_focus	1	Contrastive post_focus
**2023404**	block3	Control	pre	sy1	Object	Contrastive	r1	351.1995815	4	1	post_focus	1	Contrastive post_focus
**2023404**	block3	Control	pre	pau1	Object	Contrastive	r1	228.5325019	5	2	post_focus	1	Contrastive post_focus
**2023404**	block3	Control	pre	piu2	Subject	Broad	r1	135.7320598	1	1	broad_focus	2	Broad focus
**2023404**	block3	Control	pre	tse2	Subject	Broad	r1	148.4568171	2	2	broad_focus	2	Broad focus
**2023404**	block3	Control	pre	tsap1	Verb	Broad	r1	175.8834834	3	1	broad_focus	1	Broad focus
**2023404**	block3	Control	pre	sy1	Object	Broad	r1	383.5187408	4	1	broad_focus	1	Broad focus
**2023404**	block3	Control	pre	pau1	Object	Broad	r1	271.3355278	5	2	broad_focus	1	Broad focus
**2023404**	block3	Control	pre	bui3	Subject	Contrastive	r1	233.0565998	1	1	pre_focus	3	Contrastive pre_focus
**2023404**	block3	Control	pre	bui3	Subject	Contrastive	r1	244.9208123	2	2	pre_focus	3	Contrastive pre_focus
**2023404**	block3	Control	pre	tsv1	Verb	Contrastive	r1	338.6107753	3	1	pre_focus	1	Contrastive pre_focus
**2023404**	block3	Control	pre	fug1	Object	Contrastive	r1	425.3480925	4	1	on_focus	1	Contrastive on_focus
**2023404**	block3	Control	pre	tshe1	Object	Contrastive	r1	274.3137392	5	2	on_focus	1	Contrastive on_focus
**2023404**	block3	Control	pre	bui3	Subject	Narrow	r1	260.103458	1	1	on_focus	3	Narrow on_focus
**2023404**	block3	Control	pre	bui3	Subject	Narrow	r1	236.0668187	2	2	on_focus	3	Narrow on_focus
**2023404**	block3	Control	pre	tsv1	Verb	Narrow	r1	243.9403817	3	1	post_focus	1	Narrow post_focus
**2023404**	block3	Control	pre	fug1	Object	Narrow	r1	383.4503385	4	1	post_focus	1	Narrow post_focus
**2023404**	block3	Control	pre	tshe1	Object	Narrow	r1	198.2121997	5	2	post_focus	1	Narrow post_focus
**2023404**	block3	Control	pre	suk1	Subject	Broad	r1	67.26838354	1	1	broad_focus	1	Broad focus
**2023404**	block3	Control	pre	suk1	Subject	Broad	r1	114.6898935	2	2	broad_focus	1	Broad focus
**2023404**	block3	Control	pre	sei2	Verb	Broad	r1	149.7765143	3	1	broad_focus	2	Broad focus
**2023404**	block3	Control	pre	svy2	Object	Broad	r1	325.7944308	4	1	broad_focus	2	Broad focus
**2023404**	block3	Control	pre	kwo2	Object	Broad	r1	247.7051434	5	2	broad_focus	2	Broad focus
**2023404**	block3	Control	pre	bui3	Subject	Contrastive	r1	269.9897259	1	1	pre_focus	3	Contrastive pre_focus
**2023404**	block3	Control	pre	bui3	Subject	Contrastive	r1	230.6615177	2	2	pre_focus	3	Contrastive pre_focus
**2023404**	block3	Control	pre	tsv1	Verb	Contrastive	r1	305.9771015	3	1	on_focus	1	Contrastive on_focus
**2023404**	block3	Control	pre	fug1	Object	Contrastive	r1	353.4764895	4	1	post_focus	1	Contrastive post_focus
**2023404**	block3	Control	pre	tshe1	Object	Contrastive	r1	202.7122673	5	2	post_focus	1	Contrastive post_focus
**2023404**	block3	Control	pre	suk1	Subject	Narrow	r1	251.0190525	1	1	on_focus	1	Narrow on_focus
**2023404**	block3	Control	pre	suk1	Subject	Narrow	r1	271.6441182	2	2	on_focus	1	Narrow on_focus
**2023404**	block3	Control	pre	sei2	Verb	Narrow	r1	184.4010938	3	1	post_focus	2	Narrow post_focus
**2023404**	block3	Control	pre	svy2	Object	Narrow	r1	239.9201625	4	1	post_focus	2	Narrow post_focus
**2023404**	block3	Control	pre	kwo2	Object	Narrow	r1	126.5131741	5	2	post_focus	2	Narrow post_focus

**2023404**	block3	Control	pre	suk1	Subject	Contrastive	r1	57.34847849	1	1	pre_focus	1	Contrastive pre_focus
**2023404**	block3	Control	pre	suk1	Subject	Contrastive	r1	238.239229	2	2	pre_focus	1	Contrastive pre_focus
**2023404**	block3	Control	pre	sei2	Verb	Contrastive	r1	354.1576533	3	1	on_focus	2	Contrastive on_focus
**2023404**	block3	Control	pre	svy2	Object	Contrastive	r1	165.5336809	4	1	post_focus	2	Contrastive post_focus
**2023404**	block3	Control	pre	kwo2	Object	Contrastive	r1	160.9852443	5	2	post_focus	2	Contrastive post_focus
**2023404**	block3	Control	pre	bui3	Subject	Broad	r1	223.8504089	1	1	broad_focus	3	Broad focus
**2023404**	block3	Control	pre	bui3	Subject	Broad	r1	328.9832307	2	2	broad_focus	3	Broad focus
**2023404**	block3	Control	pre	tsv1	Verb	Broad	r1	317.8044264	3	1	broad_focus	1	Broad focus
**2023404**	block3	Control	pre	fug1	Object	Broad	r1	338.2768089	4	1	broad_focus	1	Broad focus
**2023404**	block3	Control	pre	tshe1	Object	Broad	r1	285.9458234	5	2	broad_focus	1	Broad focus
**2023404**	block3	Control	pre	suk1	Subject	Contrastive	r1	70.03207423	1	1	pre_focus	1	Contrastive pre_focus
**2023404**	block3	Control	pre	suk1	Subject	Contrastive	r1	87.30936819	2	2	pre_focus	1	Contrastive pre_focus
**2023404**	block3	Control	pre	sei2	Verb	Contrastive	r1	172.1598639	3	1	pre_focus	2	Contrastive pre_focus
**2023404**	block3	Control	pre	svy2	Object	Contrastive	r1	393.5875936	4	1	on_focus	2	Contrastive on_focus
**2023404**	block3	Control	pre	kwo2	Object	Contrastive	r1	254.984127	5	2	on_focus	2	Contrastive on_focus
**2023404**	block3	Control	pre	piu2	Subject	Narrow	r1	272.0456607	1	1	pre_focus	2	Narrow pre_focus
**2023404**	block3	Control	pre	tse2	Subject	Narrow	r1	283.119864	2	2	pre_focus	2	Narrow pre_focus
**2023404**	block3	Control	pre	tsap1	Verb	Narrow	r1	112.3314041	3	1	pre_focus	1	Narrow pre_focus
**2023404**	block3	Control	pre	sy1	Object	Narrow	r1	389.0097764	4	1	on_focus	1	Narrow on_focus
**2023404**	block3	Control	pre	pau1	Object	Narrow	r1	352.6681784	5	2	on_focus	1	Narrow on_focus
**2023404**	block3	Control	pre	piu2	Subject	Contrastive	r1	168.7106042	1	1	pre_focus	2	Contrastive pre_focus
**2023404**	block3	Control	pre	tse2	Subject	Contrastive	r1	254.8116861	2	2	pre_focus	2	Contrastive pre_focus
**2023404**	block3	Control	pre	tsap1	Verb	Contrastive	r1	121.2994126	3	1	pre_focus	1	Contrastive pre_focus
**2023404**	block3	Control	pre	sy1	Object	Contrastive	r1	304.0438908	4	1	on_focus	1	Contrastive on_focus
**2023404**	block3	Control	pre	pau1	Object	Contrastive	r1	334.6151004	5	2	on_focus	1	Contrastive on_focus
**2023404**	block3	Control	pre	suk1	Subject	Contrastive	r1	100.7830001	1	1	on_focus	1	Contrastive on_focus
**2023404**	block3	Control	pre	suk1	Subject	Contrastive	r1	145.2785403	2	2	on_focus	1	Contrastive on_focus
**2023404**	block3	Control	pre	sei2	Verb	Contrastive	r1	168.7622827	3	1	post_focus	2	Contrastive post_focus
**2023404**	block3	Control	pre	svy2	Object	Contrastive	r1	367.1626638	4	1	post_focus	2	Contrastive post_focus
**2023404**	block3	Control	pre	kwo2	Object	Contrastive	r1	249.3528694	5	2	post_focus	2	Contrastive post_focus
**2023404**	block3	Control	pre	bui3	Subject	Contrastive	r2	251.3418097	1	1	pre_focus	3	Contrastive pre_focus
**2023404**	block3	Control	pre	bui3	Subject	Contrastive	r2	212.0459241	2	2	pre_focus	3	Contrastive pre_focus
**2023404**	block3	Control	pre	tsv1	Verb	Contrastive	r2	242.7864352	3	1	pre_focus	1	Contrastive pre_focus
**2023404**	block3	Control	pre	fug1	Object	Contrastive	r2	416.0297452	4	1	on_focus	1	Contrastive on_focus
**2023404**	block3	Control	pre	tshe1	Object	Contrastive	r2	318.7951869	5	2	on_focus	1	Contrastive on_focus
**2023404**	block3	Control	pre	piu2	Subject	Narrow	r2	274.7399885	1	1	on_focus	2	Narrow on_focus
**2023404**	block3	Control	pre	tse2	Subject	Narrow	r2	253.4440064	2	2	on_focus	2	Narrow on_focus
**2023404**	block3	Control	pre	tsap1	Verb	Narrow	r2	241.9778581	3	1	post_focus	1	Narrow post_focus
**2023404**	block3	Control	pre	sy1	Object	Narrow	r2	345.5865273	4	1	post_focus	1	Narrow post_focus
**2023404**	block3	Control	pre	pau1	Object	Narrow	r2	316.5640291	5	2	post_focus	1	Narrow post_focus
**2023404**	block3	Control	pre	suk1	Subject	Narrow	r2	89.14121945	1	1	on_focus	1	Narrow on_focus
**2023404**	block3	Control	pre	suk1	Subject	Narrow	r2	126.1111111	2	2	on_focus	1	Narrow on_focus
**2023404**	block3	Control	pre	sei2	Verb	Narrow	r2	290.178712	3	1	post_focus	2	Narrow post_focus
**2023404**	block3	Control	pre	svy2	Object	Narrow	r2	421.9078983	4	1	post_focus	2	Narrow post_focus
**2023404**	block3	Control	pre	kwo2	Object	Narrow	r2	292.7067393	5	2	post_focus	2	Narrow post_focus
**2023404**	block3	Control	pre	suk1	Subject	Broad	r2	103.9659082	1	1	broad_focus	1	Broad focus
**2023404**	block3	Control	pre	suk1	Subject	Broad	r2	136.0607388	2	2	broad_focus	1	Broad focus
**2023404**	block3	Control	pre	sei2	Verb	Broad	r2	400.2774097	3	1	broad_focus	2	Broad focus
**2023404**	block3	Control	pre	svy2	Object	Broad	r2	158.726339	4	1	broad_focus	2	Broad focus
**2023404**	block3	Control	pre	kwo2	Object	Broad	r2	333.4772952	5	2	broad_focus	2	Broad focus
**2023404**	block3	Control	pre	suk1	Subject	Narrow	r2	75.12898493	1	1	pre_focus	1	Narrow pre_focus
**2023404**	block3	Control	pre	suk1	Subject	Narrow	r2	166.149311	2	2	pre_focus	1	Narrow pre_focus
**2023404**	block3	Control	pre	sei2	Verb	Narrow	r2	398.1036359	3	1	on_focus	2	Narrow on_focus
**2023404**	block3	Control	pre	svy2	Object	Narrow	r2	210.5910982	4	1	post_focus	2	Narrow post_focus
**2023404**	block3	Control	pre	kwo2	Object	Narrow	r2	174.2418745	5	2	post_focus	2	Narrow post_focus
**2023404**	block3	Control	pre	piu2	Subject	Broad	r2	316.900639	1	1	broad_focus	2	Broad focus
**2023404**	block3	Control	pre	tse2	Subject	Broad	r2	229.7317784	2	2	broad_focus	2	Broad focus
**2023404**	block3	Control	pre	tsap1	Verb	Broad	r2	126.7410835	3	1	broad_focus	1	Broad focus
**2023404**	block3	Control	pre	sy1	Object	Broad	r2	312.8239166	4	1	broad_focus	1	Broad focus
**2023404**	block3	Control	pre	pau1	Object	Broad	r2	388.0209121	5	2	broad_focus	1	Broad focus
**2023404**	block3	Control	pre	suk1	Subject	Contrastive	r2	78.14328783	1	1	on_focus	1	Contrastive on_focus
**2023404**	block3	Control	pre	suk1	Subject	Contrastive	r2	216.8460727	2	2	on_focus	1	Contrastive on_focus
**2023404**	block3	Control	pre	sei2	Verb	Contrastive	r2	280.4704586	3	1	post_focus	2	Contrastive post_focus
**2023404**	block3	Control	pre	svy2	Object	Contrastive	r2	387.7816764	4	1	post_focus	2	Contrastive post_focus
**2023404**	block3	Control	pre	kwo2	Object	Contrastive	r2	183.3099773	5	2	post_focus	2	Contrastive post_focus
**2023404**	block3	Control	pre	piu2	Subject	Contrastive	r2	172.8051611	1	1	pre_focus	2	Contrastive pre_focus

2023404	block3	Control	pre	tse2	Subject	Contrastive	r2	232.4435107	2	2	pre_focus	2	Contrastive pre_focus
2023404	block3	Control	pre	tsap1	Verb	Contrastive	r2	137.5421987	3	1	on_focus	1	Contrastive on_focus
2023404	block3	Control	pre	sy1	Object	Contrastive	r2	351.6806981	4	1	post_focus	1	Contrastive post_focus
2023404	block3	Control	pre	pau1	Object	Contrastive	r2	317.9583591	5	2	post_focus	1	Contrastive post_focus
2023404	block3	Control	pre	suk1	Subject	Contrastive	r2	137.1219257	1	1	pre_focus	1	Contrastive pre_focus
2023404	block3	Control	pre	suk1	Subject	Contrastive	r2	240.5858729	2	2	pre_focus	1	Contrastive pre_focus
2023404	block3	Control	pre	sei2	Verb	Contrastive	r2	437.8501242	3	1	pre_focus	2	Contrastive pre_focus
2023404	block3	Control	pre	svy2	Object	Contrastive	r2	429.0953461	4	1	on_focus	2	Contrastive on_focus
2023404	block3	Control	pre	kwo2	Object	Contrastive	r2	271.7757214	5	2	on_focus	2	Contrastive on_focus
2023404	block3	Control	pre	bui3	Subject	Contrastive	r2	227.5462693	1	1	pre_focus	3	Contrastive pre_focus
2023404	block3	Control	pre	bui3	Subject	Contrastive	r2	289.2613499	2	2	pre_focus	3	Contrastive pre_focus
2023404	block3	Control	pre	tsv1	Verb	Contrastive	r2	279.9029804	3	1	on_focus	1	Contrastive on_focus
2023404	block3	Control	pre	fug1	Object	Contrastive	r2	356.1704526	4	1	post_focus	1	Contrastive post_focus
2023404	block3	Control	pre	tshe1	Object	Contrastive	r2	221.9474943	5	2	post_focus	1	Contrastive post_focus
2023404	block3	Control	pre	bui3	Subject	Narrow	r2	238.7139326	1	1	on_focus	3	Narrow on_focus
2023404	block3	Control	pre	bui3	Subject	Narrow	r2	217.2011662	2	2	on_focus	3	Narrow on_focus
2023404	block3	Control	pre	tsv1	Verb	Narrow	r2	242.2545729	3	1	post_focus	1	Narrow post_focus
2023404	block3	Control	pre	fug1	Object	Narrow	r2	269.7542015	4	1	post_focus	1	Narrow post_focus
2023404	block3	Control	pre	tshe1	Object	Narrow	r2	211.4988891	5	2	post_focus	1	Narrow post_focus
2023404	block3	Control	pre	suk1	Subject	Contrastive	r2	71.65673287	1	1	pre_focus	1	Contrastive pre_focus
2023404	block3	Control	pre	suk1	Subject	Contrastive	r2	105.0476776	2	2	pre_focus	1	Contrastive pre_focus
2023404	block3	Control	pre	sei2	Verb	Contrastive	r2	232.6400158	3	1	on_focus	2	Contrastive on_focus
2023404	block3	Control	pre	svy2	Object	Contrastive	r2	153.5100781	4	1	post_focus	2	Contrastive post_focus
2023404	block3	Control	pre	kwo2	Object	Contrastive	r2	241.0512544	5	2	post_focus	2	Contrastive post_focus
2023404	block3	Control	pre	piu2	Subject	Contrastive	r2	219.2829608	1	1	pre_focus	2	Contrastive pre_focus
2023404	block3	Control	pre	tse2	Subject	Contrastive	r2	352.9744003	2	2	pre_focus	2	Contrastive pre_focus
2023404	block3	Control	pre	tsap1	Verb	Contrastive	r2	122.7186904	3	1	pre_focus	1	Contrastive pre_focus
2023404	block3	Control	pre	sy1	Object	Contrastive	r2	502.9914883	4	1	on_focus	1	Contrastive on_focus
2023404	block3	Control	pre	pau1	Object	Contrastive	r2	329.5409513	5	2	on_focus	1	Contrastive on_focus
2023404	block3	Control	pre	piu2	Subject	Narrow	r2	237.3082566	1	1	pre_focus	2	Narrow pre_focus
2023404	block3	Control	pre	tse2	Subject	Narrow	r2	216.1970913	2	2	pre_focus	2	Narrow pre_focus
2023404	block3	Control	pre	tsap1	Verb	Narrow	r2	167.8385371	3	1	pre_focus	1	Narrow pre_focus
2023404	block3	Control	pre	sy1	Object	Narrow	r2	352.2570933	4	1	on_focus	1	Narrow on_focus
2023404	block3	Control	pre	pau1	Object	Narrow	r2	331.2773998	5	2	on_focus	1	Narrow on_focus
2023404	block3	Control	pre	piu2	Subject	Contrastive	r2	317.2033258	1	1	on_focus	2	Contrastive on_focus
2023404	block3	Control	pre	tse2	Subject	Contrastive	r2	261.5808657	2	2	on_focus	2	Contrastive on_focus
2023404	block3	Control	pre	tsap1	Verb	Contrastive	r2	130.8129322	3	1	post_focus	1	Contrastive post_focus
2023404	block3	Control	pre	sy1	Object	Contrastive	r2	347.8344671	4	1	post_focus	1	Contrastive post_focus
2023404	block3	Control	pre	pau1	Object	Contrastive	r2	268.1097662	5	2	post_focus	1	Contrastive post_focus
2023404	block3	Control	pre	bui3	Subject	Broad	r2	258.0575802	1	1	broad_focus	3	Broad focus
2023404	block3	Control	pre	bui3	Subject	Broad	r2	265.5419501	2	2	broad_focus	3	Broad focus
2023404	block3	Control	pre	tsv1	Verb	Broad	r2	331.3934057	3	1	broad_focus	1	Broad focus
2023404	block3	Control	pre	fug1	Object	Broad	r2	297.6552437	4	1	broad_focus	1	Broad focus
2023404	block3	Control	pre	tshe1	Object	Broad	r2	229.9982557	5	2	broad_focus	1	Broad focus
2023404	block3	Control	pre	bui3	Subject	Narrow	r2	260.1543419	1	1	pre_focus	3	Narrow pre_focus
2023404	block3	Control	pre	bui3	Subject	Narrow	r2	230.8634223	2	2	pre_focus	3	Narrow pre_focus
2023404	block3	Control	pre	tsv1	Verb	Narrow	r2	348.6231688	3	1	on_focus	1	Narrow on_focus
2023404	block3	Control	pre	fug1	Object	Narrow	r2	365.7490552	4	1	post_focus	1	Narrow post_focus
2023404	block3	Control	pre	tshe1	Object	Narrow	r2	203.9359107	5	2	post_focus	1	Narrow post_focus
2023404	block3	Control	pre	bui3	Subject	Narrow	r2	273.2332548	1	1	pre_focus	3	Narrow pre_focus
2023404	block3	Control	pre	bui3	Subject	Narrow	r2	205.290563	2	2	pre_focus	3	Narrow pre_focus
2023404	block3	Control	pre	tsv1	Verb	Narrow	r2	325.5504052	3	1	pre_focus	1	Narrow pre_focus
2023404	block3	Control	pre	fug1	Object	Narrow	r2	333.0357356	4	1	on_focus	1	Narrow on_focus
2023404	block3	Control	pre	tshe1	Object	Narrow	r2	221.087315	5	2	on_focus	1	Narrow on_focus
2023404	block3	Control	pre	bui3	Subject	Contrastive	r2	346.7317286	1	1	on_focus	3	Contrastive on_focus
2023404	block3	Control	pre	bui3	Subject	Contrastive	r2	268.4780601	2	2	on_focus	3	Contrastive on_focus
2023404	block3	Control	pre	tsv1	Verb	Contrastive	r2	222.3034014	3	1	post_focus	1	Contrastive post_focus
2023404	block3	Control	pre	fug1	Object	Contrastive	r2	398.0660116	4	1	post_focus	1	Contrastive post_focus
2023404	block3	Control	pre	tshe1	Object	Contrastive	r2	129.2798719	5	2	post_focus	1	Contrastive post_focus
2023404	block3	Control	pre	suk1	Subject	Narrow	r2	101.2374474	1	1	pre_focus	1	Narrow pre_focus
2023404	block3	Control	pre	suk1	Subject	Narrow	r2	279.4488426	2	2	pre_focus	1	Narrow pre_focus
2023404	block3	Control	pre	sei2	Verb	Narrow	r2	325.4151585	3	1	pre_focus	2	Narrow pre_focus
2023404	block3	Control	pre	svy2	Object	Narrow	r2	411.0592436	4	1	on_focus	2	Narrow on_focus
2023404	block3	Control	pre	kwo2	Object	Narrow	r2	272.5309902	5	2	on_focus	2	Narrow on_focus
2023404	block3	Control	pre	piu2	Subject	Narrow	r2	295.1182918	1	1	pre_focus	2	Narrow pre_focus
2023404	block3	Control	pre	tse2	Subject	Narrow	r2	237.3355654	2	2	pre_focus	2	Narrow pre_focus

2023404	block3	Control	pre	tsap1	Verb	Narrow	r2	135.0353813	3	1	on_focus	1	Narrow on_focus
2023404	block3	Control	pre	sy1	Object	Narrow	r2	426.3713667	4	1	post_focus	1	Narrow post_focus
2023404	block3	Control	pre	pau1	Object	Narrow	r2	310.0443208	5	2	post_focus	1	Narrow post_focus
2023404	block4	Control	post	pak3	Subject	Contrastive	r1	284.9630425	1	1	on_focus	3	Contrastive on_focus
2023404	block4	Control	post	pak3	Subject	Contrastive	r1	228.9053288	2	2	on_focus	3	Contrastive on_focus
2023404	block4	Control	post	tsing2	Verb	Contrastive	r1	347.4810361	3	1	post_focus	2	Contrastive post_focus
2023404	block4	Control	post	kau2	Object	Contrastive	r1	422.1521137	4	1	post_focus	2	Contrastive post_focus
2023404	block4	Control	post	tsi2	Object	Contrastive	r1	53.95827664	5	2	post_focus	2	Contrastive post_focus
2023404	block4	Control	post	piu35	Subject	Broad	r1	228.8324135	1	1	broad_focus	35	Broad focus
2023404	block4	Control	post	mui35	Subject	Broad	r1	411.7082969	2	2	broad_focus	35	Broad focus
2023404	block4	Control	post	tsan3	Verb	Broad	r1	434.9150539	3	1	broad_focus	3	Broad focus
2023404	block4	Control	post	jin3	Object	Broad	r1	441.6698256	4	1	broad_focus	3	Broad focus
2023404	block4	Control	post	jin3	Object	Broad	r1	284.515618	5	2	broad_focus	3	Broad focus
2023404	block4	Control	post	pak3	Subject	Narrow	r1	221.6513216	1	1	pre_focus	3	Narrow pre_focus
2023404	block4	Control	post	pak3	Subject	Narrow	r1	149.9911817	2	2	pre_focus	3	Narrow pre_focus
2023404	block4	Control	post	tsing2	Verb	Narrow	r1	359.2170055	3	1	on_focus	2	Narrow on_focus
2023404	block4	Control	post	kau2	Object	Narrow	r1	491.7421232	4	1	post_focus	2	Narrow post_focus
2023404	block4	Control	post	tsi2	Object	Narrow	r1	168.6498488	5	2	post_focus	2	Narrow post_focus
2023404	block4	Control	post	Jan-01	Subject	Contrastive	r1	208.1442147	1	1	pre_focus	1	Contrastive pre_focus
2023404	block4	Control	post	Jan-01	Subject	Contrastive	r1	211.8917666	2	2	pre_focus	1	Contrastive pre_focus
2023404	block4	Control	post	wei3	Verb	Contrastive	r1	242.900958	3	1	pre_focus	3	Contrastive pre_focus
2023404	block4	Control	post	tsam3	Object	Contrastive	r1	339.1933521	4	1	on_focus	3	Contrastive on_focus
2023404	block4	Control	post	tsam3	Object	Contrastive	r1	245.2527605	5	2	on_focus	3	Contrastive on_focus
2023404	block4	Control	post	Jan-01	Subject	Narrow	r1	229.6480601	1	1	on_focus	1	Narrow on_focus
2023404	block4	Control	post	Jan-01	Subject	Narrow	r1	206.1092422	2	2	on_focus	1	Narrow on_focus
2023404	block4	Control	post	wei3	Verb	Narrow	r1	277.074551	3	1	post_focus	3	Narrow post_focus
2023404	block4	Control	post	tsam3	Object	Narrow	r1	349.8344144	4	1	post_focus	3	Narrow post_focus
2023404	block4	Control	post	tsam3	Object	Narrow	r1	201.5668991	5	2	post_focus	3	Narrow post_focus
2023404	block4	Control	post	Jan-01	Subject	Narrow	r1	215.6001544	1	1	pre_focus	1	Narrow pre_focus
2023404	block4	Control	post	Jan-01	Subject	Narrow	r1	251.8059253	2	2	pre_focus	1	Narrow pre_focus
2023404	block4	Control	post	wei3	Verb	Narrow	r1	316.809319	3	1	pre_focus	3	Narrow pre_focus
2023404	block4	Control	post	tsam3	Object	Narrow	r1	423.1770723	4	1	on_focus	3	Narrow on_focus
2023404	block4	Control	post	tsam3	Object	Narrow	r1	269.9160806	5	2	on_focus	3	Narrow on_focus
2023404	block4	Control	post	pak3	Subject	Contrastive	r1	119.8366053	1	1	pre_focus	3	Contrastive pre_focus
2023404	block4	Control	post	pak3	Subject	Contrastive	r1	231.7162698	2	2	pre_focus	3	Contrastive pre_focus
2023404	block4	Control	post	tsing2	Verb	Contrastive	r1	335.7409473	3	1	pre_focus	2	Contrastive pre_focus
2023404	block4	Control	post	kau2	Object	Contrastive	r1	362.7482157	4	1	on_focus	2	Contrastive on_focus
2023404	block4	Control	post	tsi2	Object	Contrastive	r1	139.8960695	5	2	on_focus	2	Contrastive on_focus
2023404	block4	Control	post	Jan-01	Subject	Contrastive	r1	318.5535628	1	1	on_focus	1	Contrastive on_focus
2023404	block4	Control	post	Jan-01	Subject	Contrastive	r1	294.7781412	2	2	on_focus	1	Contrastive on_focus
2023404	block4	Control	post	wei3	Verb	Contrastive	r1	433.5630466	3	1	post_focus	3	Contrastive post_focus
2023404	block4	Control	post	tsam3	Object	Contrastive	r1	423.9046579	4	1	post_focus	3	Contrastive post_focus
2023404	block4	Control	post	tsam3	Object	Contrastive	r1	279.9083522	5	2	post_focus	3	Contrastive post_focus
2023404	block4	Control	post	Jan-01	Subject	Contrastive	r1	305.1261519	1	1	pre_focus	1	Contrastive pre_focus
2023404	block4	Control	post	Jan-01	Subject	Contrastive	r1	240.8120883	2	2	pre_focus	1	Contrastive pre_focus
2023404	block4	Control	post	wei3	Verb	Contrastive	r1	368.3715415	3	1	on_focus	3	Contrastive on_focus
2023404	block4	Control	post	tsam3	Object	Contrastive	r1	449.8943741	4	1	post_focus	3	Contrastive post_focus
2023404	block4	Control	post	tsam3	Object	Contrastive	r1	237.5604487	5	2	post_focus	3	Contrastive post_focus
2023404	block4	Control	post	Jan-01	Subject	Narrow	r1	229.6149617	1	1	pre_focus	1	Narrow pre_focus
2023404	block4	Control	post	Jan-01	Subject	Narrow	r1	260.0268088	2	2	pre_focus	1	Narrow pre_focus
2023404	block4	Control	post	wei3	Verb	Narrow	r1	440.74165	3	1	on_focus	3	Narrow on_focus
2023404	block4	Control	post	tsam3	Object	Narrow	r1	469.2351571	4	1	post_focus	3	Narrow post_focus
2023404	block4	Control	post	tsam3	Object	Narrow	r1	238.3411619	5	2	post_focus	3	Narrow post_focus
2023404	block4	Control	post	piu35	Subject	Contrastive	r1	245.7469178	1	1	on_focus	35	Contrastive on_focus
2023404	block4	Control	post	mui35	Subject	Contrastive	r1	394.4929656	2	2	on_focus	35	Contrastive on_focus
2023404	block4	Control	post	tsan3	Verb	Contrastive	r1	304.2735554	3	1	post_focus	3	Contrastive post_focus
2023404	block4	Control	post	jin3	Object	Contrastive	r1	364.849073	4	1	post_focus	3	Contrastive post_focus
2023404	block4	Control	post	jin3	Object	Contrastive	r1	288.9457697	5	2	post_focus	3	Contrastive post_focus
2023404	block4	Control	post	piu35	Subject	Narrow	r1	234.5474007	1	1	on_focus	35	Narrow on_focus
2023404	block4	Control	post	mui35	Subject	Narrow	r1	421.2854773	2	2	on_focus	35	Narrow on_focus
2023404	block4	Control	post	tsan3	Verb	Narrow	r1	332.4445229	3	1	post_focus	3	Narrow post_focus
2023404	block4	Control	post	jin3	Object	Narrow	r1	323.2321514	4	1	post_focus	3	Narrow post_focus
2023404	block4	Control	post	jin3	Object	Narrow	r1	266.9109738	5	2	post_focus	3	Narrow post_focus
2023404	block4	Control	post	Jan-01	Subject	Broad	r1	232.2804589	1	1	broad_focus	1	Broad focus
2023404	block4	Control	post	Jan-01	Subject	Broad	r1	208.5493318	2	2	broad_focus	1	Broad focus
2023404	block4	Control	post	wei3	Verb	Broad	r1	272.6068675	3	1	broad_focus	3	Broad focus

**2023404**	block4	Control	post	tsam3	Object	Broad	r1	433.8302633	4	1	broad_focus	3	Broad focus
**2023404**	block4	Control	post	tsam3	Object	Broad	r1	218.3926085	5	2	broad_focus	3	Broad focus
**2023404**	block4	Control	post	piu35	Subject	Narrow	r1	204.5334982	1	1	pre_focus	35	Narrow pre_focus
**2023404**	block4	Control	post	mui35	Subject	Narrow	r1	269.9241743	2	2	pre_focus	35	Narrow pre_focus
**2023404**	block4	Control	post	tsan3	Verb	Narrow	r1	327.4777407	3	1	on_focus	3	Narrow on_focus
**2023404**	block4	Control	post	jin3	Object	Narrow	r1	361.5971438	4	1	post_focus	3	Narrow post_focus
**2023404**	block4	Control	post	jin3	Object	Narrow	r1	243.6669906	5	2	post_focus	3	Narrow post_focus
**2023404**	block4	Control	post	piu35	Subject	Narrow	r1	190.0151252	1	1	pre_focus	35	Narrow pre_focus
**2023404**	block4	Control	post	mui35	Subject	Narrow	r1	352.2947846	2	2	pre_focus	35	Narrow pre_focus
**2023404**	block4	Control	post	tsan3	Verb	Narrow	r1	368.6132718	3	1	pre_focus	3	Narrow pre_focus
**2023404**	block4	Control	post	jin3	Object	Narrow	r1	339.6989181	4	1	on_focus	3	Narrow on_focus
**2023404**	block4	Control	post	jin3	Object	Narrow	r1	262.015584	5	2	on_focus	3	Narrow on_focus
**2023404**	block4	Control	post	pak3	Subject	Narrow	r1	148.4605466	1	1	pre_focus	3	Narrow pre_focus
**2023404**	block4	Control	post	pak3	Subject	Narrow	r1	198.5152166	2	2	pre_focus	3	Narrow pre_focus
**2023404**	block4	Control	post	tsing2	Verb	Narrow	r1	321.5769085	3	1	pre_focus	2	Narrow pre_focus
**2023404**	block4	Control	post	kau2	Object	Narrow	r1	462.0265616	4	1	on_focus	2	Narrow on_focus
**2023404**	block4	Control	post	tsi2	Object	Narrow	r1	105.8892325	5	2	on_focus	2	Narrow on_focus
**2023404**	block4	Control	post	piu35	Subject	Contrastive	r1	155.3856589	1	1	pre_focus	35	Contrastive pre_focus
**2023404**	block4	Control	post	mui35	Subject	Contrastive	r1	409.4479655	2	2	pre_focus	35	Contrastive pre_focus
**2023404**	block4	Control	post	tsan3	Verb	Contrastive	r1	332.8546648	3	1	pre_focus	3	Contrastive pre_focus
**2023404**	block4	Control	post	jin3	Object	Contrastive	r1	391.2489521	4	1	on_focus	3	Contrastive on_focus
**2023404**	block4	Control	post	jin3	Object	Contrastive	r1	300.6502394	5	2	on_focus	3	Contrastive on_focus
**2023404**	block4	Control	post	pak3	Subject	Contrastive	r1	206.1932477	1	1	pre_focus	3	Contrastive pre_focus
**2023404**	block4	Control	post	pak3	Subject	Contrastive	r1	278.2186319	2	2	pre_focus	3	Contrastive pre_focus
**2023404**	block4	Control	post	tsing2	Verb	Contrastive	r1	423.5891059	3	1	on_focus	2	Contrastive on_focus
**2023404**	block4	Control	post	kau2	Object	Contrastive	r1	635.1074398	4	1	post_focus	2	Contrastive post_focus
**2023404**	block4	Control	post	tsi2	Object	Contrastive	r1	142.6472811	5	2	post_focus	2	Contrastive post_focus
**2023404**	block4	Control	post	pak3	Subject	Broad	r1	211.6697261	1	1	broad_focus	3	Broad focus
**2023404**	block4	Control	post	pak3	Subject	Broad	r1	244.0846217	2	2	broad_focus	3	Broad focus
**2023404**	block4	Control	post	tsing2	Verb	Broad	r1	312.1669501	3	1	broad_focus	2	Broad focus
**2023404**	block4	Control	post	kau2	Object	Broad	r1	417.5001718	4	1	broad_focus	2	Broad focus
**2023404**	block4	Control	post	tsi2	Object	Broad	r1	188.5997732	5	2	broad_focus	2	Broad focus
**2023404**	block4	Control	post	piu35	Subject	Contrastive	r1	227.7889658	1	1	pre_focus	35	Contrastive pre_focus
**2023404**	block4	Control	post	mui35	Subject	Contrastive	r1	308.9953542	2	2	pre_focus	35	Contrastive pre_focus
**2023404**	block4	Control	post	tsan3	Verb	Contrastive	r1	395.6472531	3	1	on_focus	3	Contrastive on_focus
**2023404**	block4	Control	post	jin3	Object	Contrastive	r1	361.0336134	4	1	post_focus	3	Contrastive post_focus
**2023404**	block4	Control	post	jin3	Object	Contrastive	r1	266.0554077	5	2	post_focus	3	Contrastive post_focus
**2023404**	block4	Control	post	pak3	Subject	Narrow	r1	158.9100353	1	1	on_focus	3	Narrow on_focus
**2023404**	block4	Control	post	pak3	Subject	Narrow	r1	233.5837412	2	2	on_focus	3	Narrow on_focus
**2023404**	block4	Control	post	tsing2	Verb	Narrow	r1	311.3080661	3	1	post_focus	2	Narrow post_focus
**2023404**	block4	Control	post	kau2	Object	Narrow	r1	465.5124717	4	1	post_focus	2	Narrow post_focus
**2023404**	block4	Control	post	tsi2	Object	Narrow	r1	267.3735828	5	2	post_focus	2	Narrow post_focus
**2023404**	block4	Control	post	piu35	Subject	Narrow	r2	186.9687075	1	1	pre_focus	35	Narrow pre_focus
**2023404**	block4	Control	post	mui35	Subject	Narrow	r2	298.7485153	2	2	pre_focus	35	Narrow pre_focus
**2023404**	block4	Control	post	tsan3	Verb	Narrow	r2	388.3961388	3	1	on_focus	3	Narrow on_focus
**2023404**	block4	Control	post	jin3	Object	Narrow	r2	413.3656094	4	1	post_focus	3	Narrow post_focus
**2023404**	block4	Control	post	jin3	Object	Narrow	r2	248.8178268	5	2	post_focus	3	Narrow post_focus
**2023404**	block4	Control	post	Jan-01	Subject	Contrastive	r2	253.1105557	1	1	pre_focus	1	Contrastive pre_focus
**2023404**	block4	Control	post	Jan-01	Subject	Contrastive	r2	290.2898232	2	2	pre_focus	1	Contrastive pre_focus
**2023404**	block4	Control	post	wei3	Verb	Contrastive	r2	329.7323081	3	1	on_focus	3	Contrastive on_focus
**2023404**	block4	Control	post	tsam3	Object	Contrastive	r2	478.99104	4	1	post_focus	3	Contrastive post_focus
**2023404**	block4	Control	post	tsam3	Object	Contrastive	r2	208.2052048	5	2	post_focus	3	Contrastive post_focus
**2023404**	block4	Control	post	piu35	Subject	Narrow	r2	239.93186	1	1	on_focus	35	Narrow on_focus
**2023404**	block4	Control	post	mui35	Subject	Narrow	r2	420.3250301	2	2	on_focus	35	Narrow on_focus
**2023404**	block4	Control	post	tsan3	Verb	Narrow	r2	325.6358625	3	1	post_focus	3	Narrow post_focus
**2023404**	block4	Control	post	jin3	Object	Narrow	r2	362.8049381	4	1	post_focus	3	Narrow post_focus
**2023404**	block4	Control	post	jin3	Object	Narrow	r2	222.4733302	5	2	post_focus	3	Narrow post_focus
**2023404**	block4	Control	post	Jan-01	Subject	Contrastive	r2	186.6443715	1	1	pre_focus	1	Contrastive pre_focus
**2023404**	block4	Control	post	Jan-01	Subject	Contrastive	r2	224.7489535	2	2	pre_focus	1	Contrastive pre_focus
**2023404**	block4	Control	post	wei3	Verb	Contrastive	r2	257.7435842	3	1	pre_focus	3	Contrastive pre_focus
**2023404**	block4	Control	post	tsam3	Object	Contrastive	r2	446.7733866	4	1	on_focus	3	Contrastive on_focus
**2023404**	block4	Control	post	tsam3	Object	Contrastive	r2	228.3433215	5	2	on_focus	3	Contrastive on_focus
**2023404**	block4	Control	post	piu35	Subject	Contrastive	r2	192.2832037	1	1	pre_focus	35	Contrastive pre_focus
**2023404**	block4	Control	post	mui35	Subject	Contrastive	r2	388.0937601	2	2	pre_focus	35	Contrastive pre_focus
**2023404**	block4	Control	post	tsan3	Verb	Contrastive	r2	409.0106421	3	1	on_focus	3	Contrastive on_focus
**2023404**	block4	Control	post	jin3	Object	Contrastive	r2	370.8397645	4	1	post_focus	3	Contrastive post_focus

2023404	block4	Control	post	jin3	Object	Contrastive	r2	250.6817043	5	2	post_focus	3	Contrastive post_focus
2023404	block4	Control	post	piu35	Subject	Broad	r2	239.9339015	1	1	broad_focus	35	Broad focus
2023404	block4	Control	post	mui35	Subject	Broad	r2	336.0909621	2	2	broad_focus	35	Broad focus
2023404	block4	Control	post	tsan3	Verb	Broad	r2	353.4079246	3	1	broad_focus	3	Broad focus
2023404	block4	Control	post	jin3	Object	Broad	r2	406.1346316	4	1	broad_focus	3	Broad focus
2023404	block4	Control	post	jin3	Object	Broad	r2	261.3673743	5	2	broad_focus	3	Broad focus
2023404	block4	Control	post	pak3	Subject	Contrastive	r2	290.1816097	1	1	pre_focus	3	Contrastive pre_focus
2023404	block4	Control	post	pak3	Subject	Contrastive	r2	312.1988434	2	2	pre_focus	3	Contrastive pre_focus
2023404	block4	Control	post	tsing2	Verb	Contrastive	r2	393.458961	3	1	on_focus	2	Contrastive on_focus
2023404	block4	Control	post	kau2	Object	Contrastive	r2	525.2829577	4	1	post_focus	2	Contrastive post_focus
2023404	block4	Control	post	tsi2	Object	Contrastive	r2	220.5348267	5	2	post_focus	2	Contrastive post_focus
2023404	block4	Control	post	piu35	Subject	Contrastive	r2	341.0950059	1	1	on_focus	35	Contrastive on_focus
2023404	block4	Control	post	mui35	Subject	Contrastive	r2	451.8209671	2	2	on_focus	35	Contrastive on_focus
2023404	block4	Control	post	tsan3	Verb	Contrastive	r2	422.8905634	3	1	post_focus	3	Contrastive post_focus
2023404	block4	Control	post	jin3	Object	Contrastive	r2	261.11678	4	1	post_focus	3	Contrastive post_focus
2023404	block4	Control	post	jin3	Object	Contrastive	r2	274.648068	5	2	post_focus	3	Contrastive post_focus
2023404	block4	Control	post	pak3	Subject	Narrow	r2	226.2557901	1	1	on_focus	3	Narrow on_focus
2023404	block4	Control	post	pak3	Subject	Narrow	r2	199.7054044	2	2	on_focus	3	Narrow on_focus
2023404	block4	Control	post	tsing2	Verb	Narrow	r2	339.7692887	3	1	post_focus	2	Narrow post_focus
2023404	block4	Control	post	kau2	Object	Narrow	r2	410.6804693	4	1	post_focus	2	Narrow post_focus
2023404	block4	Control	post	tsi2	Object	Narrow	r2	174.5238095	5	2	post_focus	2	Narrow post_focus
2023404	block4	Control	post	Jan-01	Subject	Narrow	r2	176.3928349	1	1	pre_focus	1	Narrow pre_focus
2023404	block4	Control	post	Jan-01	Subject	Narrow	r2	243.1502281	2	2	pre_focus	1	Narrow pre_focus
2023404	block4	Control	post	wei3	Verb	Narrow	r2	307.9823061	3	1	pre_focus	3	Narrow pre_focus
2023404	block4	Control	post	tsam3	Object	Narrow	r2	374.970105	4	1	on_focus	3	Narrow on_focus
2023404	block4	Control	post	tsam3	Object	Narrow	r2	207.5702408	5	2	on_focus	3	Narrow on_focus
2023404	block4	Control	post	pak3	Subject	Contrastive	r2	90.89020342	1	1	pre_focus	3	Contrastive pre_focus
2023404	block4	Control	post	pak3	Subject	Contrastive	r2	219.1351903	2	2	pre_focus	3	Contrastive pre_focus
2023404	block4	Control	post	tsing2	Verb	Contrastive	r2	332.5977276	3	1	pre_focus	2	Contrastive pre_focus
2023404	block4	Control	post	kau2	Object	Contrastive	r2	478.1603384	4	1	on_focus	2	Contrastive on_focus
2023404	block4	Control	post	tsi2	Object	Contrastive	r2	264.6765615	5	2	on_focus	2	Contrastive on_focus
2023404	block4	Control	post	Jan-01	Subject	Contrastive	r2	268.1172469	1	1	on_focus	1	Contrastive on_focus
2023404	block4	Control	post	Jan-01	Subject	Contrastive	r2	284.4478458	2	2	on_focus	1	Contrastive on_focus
2023404	block4	Control	post	wei3	Verb	Contrastive	r2	278.7267394	3	1	post_focus	3	Contrastive post_focus
2023404	block4	Control	post	tsam3	Object	Contrastive	r2	408.5427368	4	1	post_focus	3	Contrastive post_focus
2023404	block4	Control	post	tsam3	Object	Contrastive	r2	222.0745615	5	2	post_focus	3	Contrastive post_focus
2023404	block4	Control	post	piu35	Subject	Narrow	r2	247.6493551	1	1	pre_focus	35	Narrow pre_focus
2023404	block4	Control	post	mui35	Subject	Narrow	r2	317.7081568	2	2	pre_focus	35	Narrow pre_focus
2023404	block4	Control	post	tsan3	Verb	Narrow	r2	294.9456954	3	1	pre_focus	3	Narrow pre_focus
2023404	block4	Control	post	jin3	Object	Narrow	r2	342.4219179	4	1	on_focus	3	Narrow on_focus
2023404	block4	Control	post	jin3	Object	Narrow	r2	297.5808942	5	2	on_focus	3	Narrow on_focus
2023404	block4	Control	post	pak3	Subject	Narrow	r2	118.8021875	1	1	pre_focus	3	Narrow pre_focus
2023404	block4	Control	post	pak3	Subject	Narrow	r2	171.2662338	2	2	pre_focus	3	Narrow pre_focus
2023404	block4	Control	post	tsing2	Verb	Narrow	r2	361.5724544	3	1	pre_focus	2	Narrow pre_focus
2023404	block4	Control	post	kau2	Object	Narrow	r2	495.4877276	4	1	on_focus	2	Narrow on_focus
2023404	block4	Control	post	tsi2	Object	Narrow	r2	209.5613276	5	2	on_focus	2	Narrow on_focus
2023404	block4	Control	post	Jan-01	Subject	Narrow	r2	231.7032502	1	1	pre_focus	1	Narrow pre_focus
2023404	block4	Control	post	Jan-01	Subject	Narrow	r2	310.5402848	2	2	pre_focus	1	Narrow pre_focus
2023404	block4	Control	post	wei3	Verb	Narrow	r2	367.7732571	3	1	on_focus	3	Narrow on_focus
2023404	block4	Control	post	tsam3	Object	Narrow	r2	403.5228377	4	1	post_focus	3	Narrow post_focus
2023404	block4	Control	post	tsam3	Object	Narrow	r2	208.4808156	5	2	post_focus	3	Narrow post_focus
2023404	block4	Control	post	piu35	Subject	Contrastive	r2	168.4309334	1	1	pre_focus	35	Contrastive pre_focus
2023404	block4	Control	post	mui35	Subject	Contrastive	r2	270.6848655	2	2	pre_focus	35	Contrastive pre_focus
2023404	block4	Control	post	tsan3	Verb	Contrastive	r2	325.155102	3	1	pre_focus	3	Contrastive pre_focus
2023404	block4	Control	post	jin3	Object	Contrastive	r2	352.5472124	4	1	on_focus	3	Contrastive on_focus
2023404	block4	Control	post	jin3	Object	Contrastive	r2	196.925548	5	2	on_focus	3	Contrastive on_focus
2023404	block4	Control	post	pak3	Subject	Contrastive	r2	177.4435315	1	1	on_focus	3	Contrastive on_focus
2023404	block4	Control	post	pak3	Subject	Contrastive	r2	225.7176922	2	2	on_focus	3	Contrastive on_focus
2023404	block4	Control	post	tsing2	Verb	Contrastive	r2	320.491702	3	1	post_focus	2	Contrastive post_focus
2023404	block4	Control	post	kau2	Object	Contrastive	r2	489.587218	4	1	post_focus	2	Contrastive post_focus
2023404	block4	Control	post	tsi2	Object	Contrastive	r2	152.515711	5	2	post_focus	2	Contrastive post_focus
2023404	block4	Control	post	pak3	Subject	Broad	r2	171.0546107	1	1	broad_focus	3	Broad focus
2023404	block4	Control	post	pak3	Subject	Broad	r2	155.4041183	2	2	broad_focus	3	Broad focus
2023404	block4	Control	post	tsing2	Verb	Broad	r2	504.9384282	3	1	broad_focus	2	Broad focus
2023404	block4	Control	post	kau2	Object	Broad	r2	501.3219094	4	1	broad_focus	2	Broad focus
2023404	block4	Control	post	tsi2	Object	Broad	r2	120.058309	5	2	broad_focus	2	Broad focus

**2023404**	block4	Control	post	Jan-01	Subject	Broad	r2	309.2871832	1	1	broad_focus	1	Broad focus
**2023404**	block4	Control	post	Jan-01	Subject	Broad	r2	271.9053967	2	2	broad_focus	1	Broad focus
**2023404**	block4	Control	post	wei3	Verb	Broad	r2	276.4336821	3	1	broad_focus	3	Broad focus
**2023404**	block4	Control	post	tsam3	Object	Broad	r2	428.8447743	4	1	broad_focus	3	Broad focus
**2023404**	block4	Control	post	tsam3	Object	Broad	r2	266.3681903	5	2	broad_focus	3	Broad focus
**2023404**	block4	Control	post	pak3	Subject	Narrow	r2	185.9361472	1	1	pre_focus	3	Narrow pre_focus
**2023404**	block4	Control	post	pak3	Subject	Narrow	r2	241.1141345	2	2	pre_focus	3	Narrow pre_focus
**2023404**	block4	Control	post	tsing2	Verb	Narrow	r2	321.9706295	3	1	on_focus	2	Narrow on_focus
**2023404**	block4	Control	post	kau2	Object	Narrow	r2	478.8684998	4	1	post_focus	2	Narrow post_focus
**2023404**	block4	Control	post	tsi2	Object	Narrow	r2	201.404006	5	2	post_focus	2	Narrow post_focus
**2023404**	block4	Control	post	Jan-01	Subject	Narrow	r2	323.2812387	1	1	on_focus	1	Narrow on_focus
**2023404**	block4	Control	post	Jan-01	Subject	Narrow	r2	320.1542743	2	2	on_focus	1	Narrow on_focus
**2023404**	block4	Control	post	wei3	Verb	Narrow	r2	338.5218011	3	1	post_focus	3	Narrow post_focus
**2023404**	block4	Control	post	tsam3	Object	Narrow	r2	432.3446257	4	1	post_focus	3	Narrow post_focus
**2023404**	block4	Control	post	tsam3	Object	Narrow	r2	245.2541016	5	2	post_focus	3	Narrow post_focus
**2023404**	block4	Control	pre	piu35	Subject	Contrastive	r1	412.2252187	1	1	on_focus	35	Contrastive on_focus
**2023404**	block4	Control	pre	mui35	Subject	Contrastive	r1	392.616948	2	2	on_focus	35	Contrastive on_focus
**2023404**	block4	Control	pre	tsan3	Verb	Contrastive	r1	341.1753919	3	1	post_focus	3	Contrastive post_focus
**2023404**	block4	Control	pre	jin3	Object	Contrastive	r1	224.9383632	4	1	post_focus	3	Contrastive post_focus
**2023404**	block4	Control	pre	jin3	Object	Contrastive	r1	367.5180375	5	2	post_focus	3	Contrastive post_focus
**2023404**	block4	Control	pre	Jan-01	Subject	Broad	r1	360.7050158	1	1	broad_focus	1	Broad focus
**2023404**	block4	Control	pre	Jan-01	Subject	Broad	r1	246.4232242	2	2	broad_focus	1	Broad focus
**2023404**	block4	Control	pre	wei3	Verb	Broad	r1	306.6874256	3	1	broad_focus	3	Broad focus
**2023404**	block4	Control	pre	tsam3	Object	Broad	r1	425.7867909	4	1	broad_focus	3	Broad focus
**2023404**	block4	Control	pre	tsam3	Object	Broad	r1	248.3911565	5	2	broad_focus	3	Broad focus
**2023404**	block4	Control	pre	piu35	Subject	Contrastive	r1	230.2105006	1	1	pre_focus	35	Contrastive pre_focus
**2023404**	block4	Control	pre	mui35	Subject	Contrastive	r1	346.0835223	2	2	pre_focus	35	Contrastive pre_focus
**2023404**	block4	Control	pre	tsan3	Verb	Contrastive	r1	367.1099773	3	1	pre_focus	3	Contrastive pre_focus
**2023404**	block4	Control	pre	jin3	Object	Contrastive	r1	381.8662559	4	1	on_focus	3	Contrastive on_focus
**2023404**	block4	Control	pre	jin3	Object	Contrastive	r1	208.9410644	5	2	on_focus	3	Contrastive on_focus
**2023404**	block4	Control	pre	pak3	Subject	Contrastive	r1	124.4112407	1	1	pre_focus	3	Contrastive pre_focus
**2023404**	block4	Control	pre	pak3	Subject	Contrastive	r1	138.5575712	2	2	pre_focus	3	Contrastive pre_focus
**2023404**	block4	Control	pre	tsing2	Verb	Contrastive	r1	433.8685024	3	1	on_focus	2	Contrastive on_focus
**2023404**	block4	Control	pre	kau2	Object	Contrastive	r1	421.2878585	4	1	post_focus	2	Contrastive post_focus
**2023404**	block4	Control	pre	tsi2	Object	Contrastive	r1	261.4577259	5	2	post_focus	2	Contrastive post_focus
**2023404**	block4	Control	pre	pak3	Subject	Contrastive	r1	77.72616839	1	1	pre_focus	3	Contrastive pre_focus
**2023404**	block4	Control	pre	pak3	Subject	Contrastive	r1	239.547516	2	2	pre_focus	3	Contrastive pre_focus
**2023404**	block4	Control	pre	tsing2	Verb	Contrastive	r1	379.7567145	3	1	pre_focus	2	Contrastive pre_focus
**2023404**	block4	Control	pre	kau2	Object	Contrastive	r1	511.5551776	4	1	on_focus	2	Contrastive on_focus
**2023404**	block4	Control	pre	tsi2	Object	Contrastive	r1	64.04856387	5	2	on_focus	2	Contrastive on_focus
**2023404**	block4	Control	pre	piu35	Subject	Narrow	r1	265.9183673	1	1	on_focus	35	Narrow on_focus
**2023404**	block4	Control	pre	mui35	Subject	Narrow	r1	292.0477585	2	2	on_focus	35	Narrow on_focus
**2023404**	block4	Control	pre	tsan3	Verb	Narrow	r1	372.8835979	3	1	post_focus	3	Narrow post_focus
**2023404**	block4	Control	pre	jin3	Object	Narrow	r1	313.0681874	4	1	post_focus	3	Narrow post_focus
**2023404**	block4	Control	pre	jin3	Object	Narrow	r1	198.2368355	5	2	post_focus	3	Narrow post_focus
**2023404**	block4	Control	pre	Jan-01	Subject	Narrow	r1	284.0293742	1	1	pre_focus	1	Narrow pre_focus
**2023404**	block4	Control	pre	Jan-01	Subject	Narrow	r1	236.5886801	2	2	pre_focus	1	Narrow pre_focus
**2023404**	block4	Control	pre	wei3	Verb	Narrow	r1	298.7284009	3	1	pre_focus	3	Narrow pre_focus
**2023404**	block4	Control	pre	tsam3	Object	Narrow	r1	391.120854	4	1	on_focus	3	Narrow on_focus
**2023404**	block4	Control	pre	tsam3	Object	Narrow	r1	212.7244131	5	2	on_focus	3	Narrow on_focus
**2023404**	block4	Control	pre	Jan-01	Subject	Contrastive	r1	280.2272807	1	1	on_focus	1	Contrastive on_focus
**2023404**	block4	Control	pre	Jan-01	Subject	Contrastive	r1	247.2326795	2	2	on_focus	1	Contrastive on_focus
**2023404**	block4	Control	pre	wei3	Verb	Contrastive	r1	240.0367851	3	1	post_focus	3	Contrastive post_focus
**2023404**	block4	Control	pre	tsam3	Object	Contrastive	r1	369.2117067	4	1	post_focus	3	Contrastive post_focus
**2023404**	block4	Control	pre	tsam3	Object	Contrastive	r1	236.8253311	5	2	post_focus	3	Contrastive post_focus
**2023404**	block4	Control	pre	pak3	Subject	Narrow	r1	89.75037793	1	1	pre_focus	3	Narrow pre_focus
**2023404**	block4	Control	pre	pak3	Subject	Narrow	r1	143.4291113	2	2	pre_focus	3	Narrow pre_focus
**2023404**	block4	Control	pre	tsing2	Verb	Narrow	r1	361.0461208	3	1	pre_focus	2	Narrow pre_focus
**2023404**	block4	Control	pre	kau2	Object	Narrow	r1	390.2017625	4	1	on_focus	2	Narrow on_focus
**2023404**	block4	Control	pre	tsi2	Object	Narrow	r1	110.5701328	5	2	on_focus	2	Narrow on_focus
**2023404**	block4	Control	pre	pak3	Subject	Narrow	r1	163.2242837	1	1	pre_focus	3	Narrow pre_focus
**2023404**	block4	Control	pre	pak3	Subject	Narrow	r1	156.8323129	2	2	pre_focus	3	Narrow pre_focus
**2023404**	block4	Control	pre	tsing2	Verb	Narrow	r1	374.2019687	3	1	on_focus	2	Narrow on_focus
**2023404**	block4	Control	pre	kau2	Object	Narrow	r1	429.307421	4	1	post_focus	2	Narrow post_focus
**2023404**	block4	Control	pre	tsi2	Object	Narrow	r1	235.8272016	5	2	post_focus	2	Narrow post_focus
**2023404**	block4	Control	pre	piu35	Subject	Narrow	r1	204.0161797	1	1	pre_focus	35	Narrow pre_focus

**2023404**	block4	Control	pre	mui35	Subject	Narrow	r1	260.614564	2	2	pre_focus	35	Narrow pre_focus
**2023404**	block4	Control	pre	tsan3	Verb	Narrow	r1	383.441421	3	1	pre_focus	3	Narrow pre_focus
**2023404**	block4	Control	pre	jin3	Object	Narrow	r1	396.3253968	4	1	on_focus	3	Narrow on_focus
**2023404**	block4	Control	pre	jin3	Object	Narrow	r1	318.0461073	5	2	on_focus	3	Narrow on_focus
**2023404**	block4	Control	pre	pak3	Subject	Contrastive	r1	178.2523486	1	1	on_focus	3	Contrastive on_focus
**2023404**	block4	Control	pre	pak3	Subject	Contrastive	r1	250.521542	2	2	on_focus	3	Contrastive on_focus
**2023404**	block4	Control	pre	tsing2	Verb	Contrastive	r1	335.0037348	3	1	post_focus	2	Contrastive post_focus
**2023404**	block4	Control	pre	kau2	Object	Contrastive	r1	572.6398337	4	1	post_focus	2	Contrastive post_focus
**2023404**	block4	Control	pre	tsi2	Object	Contrastive	r1	143.5884354	5	2	post_focus	2	Contrastive post_focus
**2023404**	block4	Control	pre	Jan-01	Subject	Narrow	r1	236.3278585	1	1	pre_focus	1	Narrow pre_focus
**2023404**	block4	Control	pre	Jan-01	Subject	Narrow	r1	327.9726673	2	2	pre_focus	1	Narrow pre_focus
**2023404**	block4	Control	pre	wei3	Verb	Narrow	r1	431.0662878	3	1	on_focus	3	Narrow on_focus
**2023404**	block4	Control	pre	tsam3	Object	Narrow	r1	245.3736458	4	1	post_focus	3	Narrow post_focus
**2023404**	block4	Control	pre	tsam3	Object	Narrow	r1	182.2306446	5	2	post_focus	3	Narrow post_focus
**2023404**	block4	Control	pre	pak3	Subject	Broad	r1	244.9300831	1	1	broad_focus	3	Broad focus
**2023404**	block4	Control	pre	pak3	Subject	Broad	r1	236.4134543	2	2	broad_focus	3	Broad focus
**2023404**	block4	Control	pre	tsing2	Verb	Broad	r1	388.180364	3	1	broad_focus	2	Broad focus
**2023404**	block4	Control	pre	kau2	Object	Broad	r1	481.8957636	4	1	broad_focus	2	Broad focus
**2023404**	block4	Control	pre	tsi2	Object	Broad	r1	156.0447036	5	2	broad_focus	2	Broad focus
**2023404**	block4	Control	pre	piu35	Subject	Broad	r1	188.9730892	1	1	broad_focus	35	Broad focus
**2023404**	block4	Control	pre	mui35	Subject	Broad	r1	312.9754174	2	2	broad_focus	35	Broad focus
**2023404**	block4	Control	pre	tsan3	Verb	Broad	r1	407.0535157	3	1	broad_focus	3	Broad focus
**2023404**	block4	Control	pre	jin3	Object	Broad	r1	411.8185941	4	1	broad_focus	3	Broad focus
**2023404**	block4	Control	pre	jin3	Object	Broad	r1	225.5782313	5	2	broad_focus	3	Broad focus
**2023404**	block4	Control	pre	piu35	Subject	Narrow	r1	189.1626858	1	1	pre_focus	35	Narrow pre_focus
**2023404**	block4	Control	pre	mui35	Subject	Narrow	r1	252.4243772	2	2	pre_focus	35	Narrow pre_focus
**2023404**	block4	Control	pre	tsan3	Verb	Narrow	r1	407.223356	3	1	on_focus	3	Narrow on_focus
**2023404**	block4	Control	pre	jin3	Object	Narrow	r1	368.5551459	4	1	post_focus	3	Narrow post_focus
**2023404**	block4	Control	pre	jin3	Object	Narrow	r1	325.7750979	5	2	post_focus	3	Narrow post_focus
**2023404**	block4	Control	pre	pak3	Subject	Narrow	r1	255.599686	1	1	on_focus	3	Narrow on_focus
**2023404**	block4	Control	pre	pak3	Subject	Narrow	r1	290.1715378	2	2	on_focus	3	Narrow on_focus
**2023404**	block4	Control	pre	tsing2	Verb	Narrow	r1	391.9113922	3	1	post_focus	2	Narrow post_focus
**2023404**	block4	Control	pre	kau2	Object	Narrow	r1	558.6744772	4	1	post_focus	2	Narrow post_focus
**2023404**	block4	Control	pre	tsi2	Object	Narrow	r1	142.5160691	5	2	post_focus	2	Narrow post_focus
**2023404**	block4	Control	pre	Jan-01	Subject	Contrastive	r1	340.2870271	1	1	pre_focus	1	Contrastive pre_focus
**2023404**	block4	Control	pre	Jan-01	Subject	Contrastive	r1	287.4433389	2	2	pre_focus	1	Contrastive pre_focus
**2023404**	block4	Control	pre	wei3	Verb	Contrastive	r1	436.5391871	3	1	pre_focus	3	Contrastive pre_focus
**2023404**	block4	Control	pre	tsam3	Object	Contrastive	r1	383.3417641	4	1	on_focus	3	Contrastive on_focus
**2023404**	block4	Control	pre	tsam3	Object	Contrastive	r1	261.4018066	5	2	on_focus	3	Contrastive on_focus
**2023404**	block4	Control	pre	Jan-01	Subject	Contrastive	r1	247.5928244	1	1	pre_focus	1	Contrastive pre_focus
**2023404**	block4	Control	pre	Jan-01	Subject	Contrastive	r1	256.3357089	2	2	pre_focus	1	Contrastive pre_focus
**2023404**	block4	Control	pre	wei3	Verb	Contrastive	r1	371.5330653	3	1	on_focus	3	Contrastive on_focus
**2023404**	block4	Control	pre	tsam3	Object	Contrastive	r1	411.8820862	4	1	post_focus	3	Contrastive post_focus
**2023404**	block4	Control	pre	tsam3	Object	Contrastive	r1	130.7578929	5	2	post_focus	3	Contrastive post_focus
**2023404**	block4	Control	pre	piu35	Subject	Contrastive	r1	214.2033258	1	1	pre_focus	35	Contrastive pre_focus
**2023404**	block4	Control	pre	mui35	Subject	Contrastive	r1	339.2187953	2	2	pre_focus	35	Contrastive pre_focus
**2023404**	block4	Control	pre	tsan3	Verb	Contrastive	r1	417.8846777	3	1	on_focus	3	Contrastive on_focus
**2023404**	block4	Control	pre	jin3	Object	Contrastive	r1	219.963806	4	1	post_focus	3	Contrastive post_focus
**2023404**	block4	Control	pre	jin3	Object	Contrastive	r1	204.5304233	5	2	post_focus	3	Contrastive post_focus
**2023404**	block4	Control	pre	Jan-01	Subject	Narrow	r1	315.3749055	1	1	on_focus	1	Narrow on_focus
**2023404**	block4	Control	pre	Jan-01	Subject	Narrow	r1	247.8581736	2	2	on_focus	1	Narrow on_focus
**2023404**	block4	Control	pre	wei3	Verb	Narrow	r1	311.1782895	3	1	post_focus	3	Narrow post_focus
**2023404**	block4	Control	pre	tsam3	Object	Narrow	r1	321.4294926	4	1	post_focus	3	Narrow post_focus
**2023404**	block4	Control	pre	tsam3	Object	Narrow	r1	197.5384228	5	2	post_focus	3	Narrow post_focus
**2023404**	block4	Control	pre	Jan-01	Subject	Contrastive	r2	350.2686535	1	1	on_focus	1	Contrastive on_focus
**2023404**	block4	Control	pre	Jan-01	Subject	Contrastive	r2	248.3262935	2	2	on_focus	1	Contrastive on_focus
**2023404**	block4	Control	pre	wei3	Verb	Contrastive	r2	335.9035201	3	1	post_focus	3	Contrastive post_focus
**2023404**	block4	Control	pre	tsam3	Object	Contrastive	r2	400.8843537	4	1	post_focus	3	Contrastive post_focus
**2023404**	block4	Control	pre	tsam3	Object	Contrastive	r2	176.0408163	5	2	post_focus	3	Contrastive post_focus
**2023404**	block4	Control	pre	pak3	Subject	Contrastive	r2	143.8387828	1	1	pre_focus	3	Contrastive pre_focus
**2023404**	block4	Control	pre	pak3	Subject	Contrastive	r2	221.3727639	2	2	pre_focus	3	Contrastive pre_focus
**2023404**	block4	Control	pre	tsing2	Verb	Contrastive	r2	356.6809017	3	1	pre_focus	2	Contrastive pre_focus
**2023404**	block4	Control	pre	kau2	Object	Contrastive	r2	539.8335006	4	1	on_focus	2	Contrastive on_focus
**2023404**	block4	Control	pre	tsi2	Object	Contrastive	r2	145.0118778	5	2	on_focus	2	Contrastive on_focus
**2023404**	block4	Control	pre	Jan-01	Subject	Narrow	r2	250.4267954	1	1	on_focus	1	Narrow on_focus
**2023404**	block4	Control	pre	Jan-01	Subject	Narrow	r2	218.6509765	2	2	on_focus	1	Narrow on_focus

2023404	block4	Control	pre	wei3	Verb	Narrow	r2	303.9498299	3	1	post_focus	3	Narrow post_focus
2023404	block4	Control	pre	tsam3	Object	Narrow	r2	315.4141956	4	1	post_focus	3	Narrow post_focus
2023404	block4	Control	pre	tsam3	Object	Narrow	r2	241.2622827	5	2	post_focus	3	Narrow post_focus
2023404	block4	Control	pre	piu35	Subject	Narrow	r2	293.5008314	1	1	on_focus	35	Narrow on_focus
2023404	block4	Control	pre	mui35	Subject	Narrow	r2	351.4405842	2	2	on_focus	35	Narrow on_focus
2023404	block4	Control	pre	tsan3	Verb	Narrow	r2	437.064032	3	1	post_focus	3	Narrow post_focus
2023404	block4	Control	pre	jin3	Object	Narrow	r2	315.1919879	4	1	post_focus	3	Narrow post_focus
2023404	block4	Control	pre	jin3	Object	Narrow	r2	298.1101753	5	2	post_focus	3	Narrow post_focus
2023404	block4	Control	pre	pak3	Subject	Contrastive	r2	239.7252396	1	1	on_focus	3	Contrastive on_focus
2023404	block4	Control	pre	pak3	Subject	Contrastive	r2	250.2534347	2	2	on_focus	3	Contrastive on_focus
2023404	block4	Control	pre	tsing2	Verb	Contrastive	r2	446.739229	3	1	post_focus	2	Contrastive post_focus
2023404	block4	Control	pre	kau2	Object	Contrastive	r2	494.7943194	4	1	post_focus	2	Contrastive post_focus
2023404	block4	Control	pre	tsi2	Object	Contrastive	r2	195.0292895	5	2	post_focus	2	Contrastive post_focus
2023404	block4	Control	pre	piu35	Subject	Contrastive	r2	274.2513228	1	1	on_focus	35	Contrastive on_focus
2023404	block4	Control	pre	mui35	Subject	Contrastive	r2	377.6406436	2	2	on_focus	35	Contrastive on_focus
2023404	block4	Control	pre	tsan3	Verb	Contrastive	r2	363.3060385	3	1	post_focus	3	Contrastive post_focus
2023404	block4	Control	pre	jin3	Object	Contrastive	r2	274.3537415	4	1	post_focus	3	Contrastive post_focus
2023404	block4	Control	pre	jin3	Object	Contrastive	r2	211.8302559	5	2	post_focus	3	Contrastive post_focus
2023404	block4	Control	pre	Jan-01	Subject	Contrastive	r2	286.680839	1	1	pre_focus	1	Contrastive pre_focus
2023404	block4	Control	pre	Jan-01	Subject	Contrastive	r2	296.0295791	2	2	pre_focus	1	Contrastive pre_focus
2023404	block4	Control	pre	wei3	Verb	Contrastive	r2	481.0253887	3	1	on_focus	3	Contrastive on_focus
2023404	block4	Control	pre	tsam3	Object	Contrastive	r2	218.0772719	4	1	post_focus	3	Contrastive post_focus
2023404	block4	Control	pre	tsam3	Object	Contrastive	r2	248.7926888	5	2	post_focus	3	Contrastive post_focus
2023404	block4	Control	pre	piu35	Subject	Contrastive	r2	151.2717309	1	1	pre_focus	35	Contrastive pre_focus
2023404	block4	Control	pre	mui35	Subject	Contrastive	r2	646.6397077	2	2	pre_focus	35	Contrastive pre_focus
2023404	block4	Control	pre	tsan3	Verb	Contrastive	r2	548.0066427	3	1	on_focus	3	Contrastive on_focus
2023404	block4	Control	pre	jin3	Object	Contrastive	r2	323.7697169	4	1	post_focus	3	Contrastive post_focus
2023404	block4	Control	pre	jin3	Object	Contrastive	r2	299.1300286	5	2	post_focus	3	Contrastive post_focus
2023404	block4	Control	pre	pak3	Subject	Narrow	r2	107.1794218	1	1	pre_focus	3	Narrow pre_focus
2023404	block4	Control	pre	pak3	Subject	Narrow	r2	210.8680364	2	2	pre_focus	3	Narrow pre_focus
2023404	block4	Control	pre	tsing2	Verb	Narrow	r2	321.3342636	3	1	pre_focus	2	Narrow pre_focus
2023404	block4	Control	pre	kau2	Object	Narrow	r2	464.1848231	4	1	on_focus	2	Narrow on_focus
2023404	block4	Control	pre	tsi2	Object	Narrow	r2	217.0007559	5	2	on_focus	2	Narrow on_focus
2023404	block4	Control	pre	piu35	Subject	Narrow	r2	179.7403051	1	1	pre_focus	35	Narrow pre_focus
2023404	block4	Control	pre	mui35	Subject	Narrow	r2	305.5051082	2	2	pre_focus	35	Narrow pre_focus
2023404	block4	Control	pre	tsan3	Verb	Narrow	r2	415.466942	3	1	pre_focus	3	Narrow pre_focus
2023404	block4	Control	pre	jin3	Object	Narrow	r2	307.0306122	4	1	on_focus	3	Narrow on_focus
2023404	block4	Control	pre	jin3	Object	Narrow	r2	230.4622827	5	2	on_focus	3	Narrow on_focus
2023404	block4	Control	pre	Jan-01	Subject	Narrow	r2	229.6663427	1	1	pre_focus	1	Narrow pre_focus
2023404	block4	Control	pre	Jan-01	Subject	Narrow	r2	240.2883111	2	2	pre_focus	1	Narrow pre_focus
2023404	block4	Control	pre	wei3	Verb	Narrow	r2	257.3970161	3	1	pre_focus	3	Narrow pre_focus
2023404	block4	Control	pre	tsam3	Object	Narrow	r2	351.546038	4	1	on_focus	3	Narrow on_focus
2023404	block4	Control	pre	tsam3	Object	Narrow	r2	237.1313111	5	2	on_focus	3	Narrow on_focus
2023404	block4	Control	pre	Jan-01	Subject	Broad	r2	295.6712902	1	1	broad_focus	1	Broad focus
2023404	block4	Control	pre	Jan-01	Subject	Broad	r2	242.0574917	2	2	broad_focus	1	Broad focus
2023404	block4	Control	pre	wei3	Verb	Broad	r2	359.0310052	3	1	broad_focus	3	Broad focus
2023404	block4	Control	pre	tsam3	Object	Broad	r2	405.7686576	4	1	broad_focus	3	Broad focus
2023404	block4	Control	pre	tsam3	Object	Broad	r2	221.1250091	5	2	broad_focus	3	Broad focus
2023404	block4	Control	pre	pak3	Subject	Narrow	r2	215.5078057	1	1	on_focus	3	Narrow on_focus
2023404	block4	Control	pre	pak3	Subject	Narrow	r2	260.2648679	2	2	on_focus	3	Narrow on_focus
2023404	block4	Control	pre	tsing2	Verb	Narrow	r2	352.3454571	3	1	post_focus	2	Narrow post_focus
2023404	block4	Control	pre	kau2	Object	Narrow	r2	235.4953231	4	1	post_focus	2	Narrow post_focus
2023404	block4	Control	pre	tsi2	Object	Narrow	r2	230.667609	5	2	post_focus	2	Narrow post_focus
2023404	block4	Control	pre	Jan-01	Subject	Contrastive	r2	251.2891576	1	1	pre_focus	1	Contrastive pre_focus
2023404	block4	Control	pre	Jan-01	Subject	Contrastive	r2	264.8157218	2	2	pre_focus	1	Contrastive pre_focus
2023404	block4	Control	pre	wei3	Verb	Contrastive	r2	362.1556514	3	1	pre_focus	3	Contrastive pre_focus
2023404	block4	Control	pre	tsam3	Object	Contrastive	r2	421.1857807	4	1	on_focus	3	Contrastive on_focus
2023404	block4	Control	pre	tsam3	Object	Contrastive	r2	233.6148904	5	2	on_focus	3	Contrastive on_focus
2023404	block4	Control	pre	piu35	Subject	Narrow	r2	316.1949771	1	1	pre_focus	35	Narrow pre_focus
2023404	block4	Control	pre	mui35	Subject	Narrow	r2	360.9615352	2	2	pre_focus	35	Narrow pre_focus
2023404	block4	Control	pre	tsan3	Verb	Narrow	r2	422.1678373	3	1	on_focus	3	Narrow on_focus
2023404	block4	Control	pre	jin3	Object	Narrow	r2	257.8319566	4	1	post_focus	3	Narrow post_focus
2023404	block4	Control	pre	jin3	Object	Narrow	r2	322.1253501	5	2	post_focus	3	Narrow post_focus
2023404	block4	Control	pre	pak3	Subject	Contrastive	r2	148.9697366	1	1	pre_focus	3	Contrastive pre_focus
2023404	block4	Control	pre	pak3	Subject	Contrastive	r2	319.3899311	2	2	pre_focus	3	Contrastive pre_focus
2023404	block4	Control	pre	tsing2	Verb	Contrastive	r2	395.7622605	3	1	on_focus	2	Contrastive on_focus

**2023404**	block4	Control	pre	kau2	Object	Contrastive	r2	538.2688793	4	1	post_focus	2	Contrastive post_focus
**2023404**	block4	Control	pre	tsi2	Object	Contrastive	r2	227.9052791	5	2	post_focus	2	Contrastive post_focus
**2023404**	block4	Control	pre	Jan-01	Subject	Narrow	r2	308.7269609	1	1	pre_focus	1	Narrow pre_focus
**2023404**	block4	Control	pre	Jan-01	Subject	Narrow	r2	244.8630417	2	2	pre_focus	1	Narrow pre_focus
**2023404**	block4	Control	pre	wei3	Verb	Narrow	r2	432.0945276	3	1	on_focus	3	Narrow on_focus
**2023404**	block4	Control	pre	tsam3	Object	Narrow	r2	425.3716916	4	1	post_focus	3	Narrow post_focus
**2023404**	block4	Control	pre	tsam3	Object	Narrow	r2	246.76713	5	2	post_focus	3	Narrow post_focus
**2023404**	block4	Control	pre	pak3	Subject	Narrow	r2	204.2015754	1	1	pre_focus	3	Narrow pre_focus
**2023404**	block4	Control	pre	pak3	Subject	Narrow	r2	219.3181082	2	2	pre_focus	3	Narrow pre_focus
**2023404**	block4	Control	pre	tsing2	Verb	Narrow	r2	329.1761149	3	1	on_focus	2	Narrow on_focus
**2023404**	block4	Control	pre	kau2	Object	Narrow	r2	430.1302898	4	1	post_focus	2	Narrow post_focus
**2023404**	block4	Control	pre	tsi2	Object	Narrow	r2	156.4227712	5	2	post_focus	2	Narrow post_focus
**2023404**	block4	Control	pre	piu35	Subject	Contrastive	r2	230.7702427	1	1	pre_focus	35	Contrastive pre_focus
**2023404**	block4	Control	pre	mui35	Subject	Contrastive	r2	317.7713452	2	2	pre_focus	35	Contrastive pre_focus
**2023404**	block4	Control	pre	tsan3	Verb	Contrastive	r2	364.2726985	3	1	pre_focus	3	Contrastive pre_focus
**2023404**	block4	Control	pre	jin3	Object	Contrastive	r2	301.5983155	4	1	on_focus	3	Contrastive on_focus
**2023404**	block4	Control	pre	jin3	Object	Contrastive	r2	255.3757195	5	2	on_focus	3	Contrastive on_focus
**2023404**	block4	Control	pre	pak3	Subject	Broad	r2	178.404195	1	1	broad_focus	3	Broad focus
**2023404**	block4	Control	pre	pak3	Subject	Broad	r2	173.2512197	2	2	broad_focus	3	Broad focus
**2023404**	block4	Control	pre	tsing2	Verb	Broad	r2	241.8037518	3	1	broad_focus	2	Broad focus
**2023404**	block4	Control	pre	kau2	Object	Broad	r2	252.2119401	4	1	broad_focus	2	Broad focus
**2023404**	block4	Control	pre	tsi2	Object	Broad	r2	173.4815502	5	2	broad_focus	2	Broad focus
**2023404**	block4	Control	pre	piu35	Subject	Broad	r2	339.8504993	1	1	broad_focus	35	Broad focus
**2023404**	block4	Control	pre	mui35	Subject	Broad	r2	241.4187376	2	2	broad_focus	35	Broad focus
**2023404**	block4	Control	pre	tsan3	Verb	Broad	r2	319.5735335	3	1	broad_focus	3	Broad focus
**2023404**	block4	Control	pre	jin3	Object	Broad	r2	272.1904282	4	1	broad_focus	3	Broad focus
**2023404**	block4	Control	pre	jin3	Object	Broad	r2	280.175359	5	2	broad_focus	3	Broad focus
**2023404**	block5	Control	post	ceoi3	Subject	Narrow	r1	181.3821001	1	1	pre_focus	3	Narrow pre_focus
**2023404**	block5	Control	post	ceoi3	Subject	Narrow	r1	235.6330521	2	2	pre_focus	3	Narrow pre_focus
**2023404**	block5	Control	post	caa4	Verb	Narrow	r1	287.1885921	3	1	pre_focus	4	Narrow pre_focus
**2023404**	block5	Control	post	ngau4	Object	Narrow	r1	308.8748515	4	1	on_focus	4	Narrow on_focus
**2023404**	block5	Control	post	jau4	Object	Narrow	r1	213.2211915	5	2	on_focus	4	Narrow on_focus
**2023404**	block5	Control	post	wai5	Subject	Narrow	r1	227.5907808	1	1	pre_focus	5	Narrow pre_focus
**2023404**	block5	Control	post	wai5	Subject	Narrow	r1	260.7588443	2	2	pre_focus	5	Narrow pre_focus
**2023404**	block5	Control	post	waat3	Verb	Narrow	r1	184.5685034	3	1	pre_focus	3	Narrow pre_focus
**2023404**	block5	Control	post	bui3	Object	Narrow	r1	360.0398804	4	1	on_focus	3	Narrow on_focus
**2023404**	block5	Control	post	hok3	Object	Narrow	r1	162.1845901	5	2	on_focus	3	Narrow on_focus
**2023404**	block5	Control	post	wai5	Subject	Contrastive	r1	249.9702507	1	1	pre_focus	5	Contrastive pre_focus
**2023404**	block5	Control	post	wai5	Subject	Contrastive	r1	227.9523481	2	2	pre_focus	5	Contrastive pre_focus
**2023404**	block5	Control	post	waat3	Verb	Contrastive	r1	159.4679222	3	1	pre_focus	3	Contrastive pre_focus
**2023404**	block5	Control	post	bui3	Object	Contrastive	r1	405.955102	4	1	on_focus	3	Contrastive on_focus
**2023404**	block5	Control	post	hok3	Object	Contrastive	r1	87.19707195	5	2	on_focus	3	Contrastive on_focus
**2023404**	block5	Control	post	ceoi3	Subject	Contrastive	r1	207.518104	1	1	pre_focus	3	Contrastive pre_focus
**2023404**	block5	Control	post	ceoi3	Subject	Contrastive	r1	262.6858242	2	2	pre_focus	3	Contrastive pre_focus
**2023404**	block5	Control	post	caa4	Verb	Contrastive	r1	236.4858671	3	1	pre_focus	4	Contrastive pre_focus
**2023404**	block5	Control	post	ngau4	Object	Contrastive	r1	292.1008403	4	1	on_focus	4	Contrastive on_focus
**2023404**	block5	Control	post	jau4	Object	Contrastive	r1	207.5444276	5	2	on_focus	4	Contrastive on_focus
**2023404**	block5	Control	post	wai5	Subject	Narrow	r1	211.0063376	1	1	pre_focus	5	Narrow pre_focus
**2023404**	block5	Control	post	wai5	Subject	Narrow	r1	262.0152763	2	2	pre_focus	5	Narrow pre_focus
**2023404**	block5	Control	post	waat3	Verb	Narrow	r1	178.5914787	3	1	on_focus	3	Narrow on_focus
**2023404**	block5	Control	post	bui3	Object	Narrow	r1	406.9713314	4	1	post_focus	3	Narrow post_focus
**2023404**	block5	Control	post	hok3	Object	Narrow	r1	132.3783187	5	2	post_focus	3	Narrow post_focus
**2023404**	block5	Control	post	wai5	Subject	Contrastive	r1	265.2430211	1	1	on_focus	5	Contrastive on_focus
**2023404**	block5	Control	post	wai5	Subject	Contrastive	r1	333.3346413	2	2	on_focus	5	Contrastive on_focus
**2023404**	block5	Control	post	waat3	Verb	Contrastive	r1	214.8237889	3	1	post_focus	3	Contrastive post_focus
**2023404**	block5	Control	post	bui3	Object	Contrastive	r1	359.4494456	4	1	post_focus	3	Contrastive post_focus
**2023404**	block5	Control	post	hok3	Object	Contrastive	r1	147.1800447	5	2	post_focus	3	Contrastive post_focus
**2023404**	block5	Control	post	siu2	Subject	Broad	r1	147.1187348	1	1	broad_focus	2	Broad focus
**2023404**	block5	Control	post	gwong2	Subject	Broad	r1	367.9412142	2	2	broad_focus	2	Broad focus
**2023404**	block5	Control	post	cyun4	Verb	Broad	r1	388.7282961	3	1	broad_focus	4	Broad focus
**2023404**	block5	Control	post	laam4	Object	Broad	r1	257.8921352	4	1	broad_focus	4	Broad focus
**2023404**	block5	Control	post	kau4	Object	Broad	r1	136.0808228	5	2	broad_focus	4	Broad focus
**2023404**	block5	Control	post	siu2	Subject	Contrastive	r1	254.6015549	1	1	pre_focus	2	Contrastive pre_focus
**2023404**	block5	Control	post	gwong2	Subject	Contrastive	r1	335.1393538	2	2	pre_focus	2	Contrastive pre_focus
**2023404**	block5	Control	post	cyun4	Verb	Contrastive	r1	242.3642155	3	1	pre_focus	4	Contrastive pre_focus
**2023404**	block5	Control	post	laam4	Object	Contrastive	r1	266.3124673	4	1	on_focus	4	Contrastive on_focus

**2023404**	block5	Control	post	kau4	Object	Contrastive	r1	201.4890401	5	2	on_focus	4	Contrastive on_focus
**2023404**	block5	Control	post	siu2	Subject	Narrow	r1	173.8912284	1	1	pre_focus	2	Narrow pre_focus
**2023404**	block5	Control	post	gwong2	Subject	Narrow	r1	332.7378488	2	2	pre_focus	2	Narrow pre_focus
**2023404**	block5	Control	post	cyun4	Verb	Narrow	r1	271.6750851	3	1	pre_focus	4	Narrow pre_focus
**2023404**	block5	Control	post	laam4	Object	Narrow	r1	268.2417582	4	1	on_focus	4	Narrow on_focus
**2023404**	block5	Control	post	kau4	Object	Narrow	r1	242.079007	5	2	on_focus	4	Narrow on_focus
**2023404**	block5	Control	post	ceoi3	Subject	Contrastive	r1	284.5792174	1	1	on_focus	3	Contrastive on_focus
**2023404**	block5	Control	post	ceoi3	Subject	Contrastive	r1	299.7478505	2	2	on_focus	3	Contrastive on_focus
**2023404**	block5	Control	post	caa4	Verb	Contrastive	r1	294.3467058	3	1	post_focus	4	Contrastive post_focus
**2023404**	block5	Control	post	ngau4	Object	Contrastive	r1	246.4237614	4	1	post_focus	4	Contrastive post_focus
**2023404**	block5	Control	post	jau4	Object	Contrastive	r1	146.1594416	5	2	post_focus	4	Contrastive post_focus
**2023404**	block5	Control	post	siu2	Subject	Narrow	r1	273.1921364	1	1	pre_focus	2	Narrow pre_focus
**2023404**	block5	Control	post	gwong2	Subject	Narrow	r1	334.0569008	2	2	pre_focus	2	Narrow pre_focus
**2023404**	block5	Control	post	cyun4	Verb	Narrow	r1	275.5739121	3	1	on_focus	4	Narrow on_focus
**2023404**	block5	Control	post	laam4	Object	Narrow	r1	374.0408345	4	1	post_focus	4	Narrow post_focus
**2023404**	block5	Control	post	kau4	Object	Narrow	r1	128.2884294	5	2	post_focus	4	Narrow post_focus
**2023404**	block5	Control	post	ceoi3	Subject	Narrow	r1	200.1921497	1	1	pre_focus	3	Narrow pre_focus
**2023404**	block5	Control	post	ceoi3	Subject	Narrow	r1	249.5471082	2	2	pre_focus	3	Narrow pre_focus
**2023404**	block5	Control	post	caa4	Verb	Narrow	r1	260.594583	3	1	on_focus	4	Narrow on_focus
**2023404**	block5	Control	post	ngau4	Object	Narrow	r1	281.0479747	4	1	post_focus	4	Narrow post_focus
**2023404**	block5	Control	post	jau4	Object	Narrow	r1	186.2939706	5	2	post_focus	4	Narrow post_focus
**2023404**	block5	Control	post	wai5	Subject	Contrastive	r1	446.7556269	1	1	pre_focus	5	Contrastive pre_focus
**2023404**	block5	Control	post	wai5	Subject	Contrastive	r1	306.3098971	2	2	pre_focus	5	Contrastive pre_focus
**2023404**	block5	Control	post	waat3	Verb	Contrastive	r1	204.5985804	3	1	on_focus	3	Contrastive on_focus
**2023404**	block5	Control	post	bui3	Object	Contrastive	r1	376.7351107	4	1	post_focus	3	Contrastive post_focus
**2023404**	block5	Control	post	hok3	Object	Contrastive	r1	100.700949	5	2	post_focus	3	Contrastive post_focus
**2023404**	block5	Control	post	ceoi3	Subject	Contrastive	r1	283.7367435	1	1	pre_focus	3	Contrastive pre_focus
**2023404**	block5	Control	post	ceoi3	Subject	Contrastive	r1	283.1054746	2	2	pre_focus	3	Contrastive pre_focus
**2023404**	block5	Control	post	caa4	Verb	Contrastive	r1	296.446628	3	1	on_focus	4	Contrastive on_focus
**2023404**	block5	Control	post	ngau4	Object	Contrastive	r1	274.0243612	4	1	post_focus	4	Contrastive post_focus
**2023404**	block5	Control	post	jau4	Object	Contrastive	r1	181.9957113	5	2	post_focus	4	Contrastive post_focus
**2023404**	block5	Control	post	siu2	Subject	Narrow	r1	240.6004155	1	1	on_focus	2	Narrow on_focus
**2023404**	block5	Control	post	gwong2	Subject	Narrow	r1	381.0561872	2	2	on_focus	2	Narrow on_focus
**2023404**	block5	Control	post	cyun4	Verb	Narrow	r1	240.0274457	3	1	post_focus	4	Narrow post_focus
**2023404**	block5	Control	post	laam4	Object	Narrow	r1	449.5175378	4	1	post_focus	4	Narrow post_focus
**2023404**	block5	Control	post	kau4	Object	Narrow	r1	65.71510067	5	2	post_focus	4	Narrow post_focus
**2023404**	block5	Control	post	ceoi3	Subject	Narrow	r1	191.5435755	1	1	on_focus	3	Narrow on_focus
**2023404**	block5	Control	post	ceoi3	Subject	Narrow	r1	282.7839925	2	2	on_focus	3	Narrow on_focus
**2023404**	block5	Control	post	caa4	Verb	Narrow	r1	260.8728897	3	1	post_focus	4	Narrow post_focus
**2023404**	block5	Control	post	ngau4	Object	Narrow	r1	384.3822373	4	1	post_focus	4	Narrow post_focus
**2023404**	block5	Control	post	jau4	Object	Narrow	r1	186.7299428	5	2	post_focus	4	Narrow post_focus
**2023404**	block5	Control	post	ceoi3	Subject	Broad	r1	263.8704289	1	1	broad_focus	3	Broad focus
**2023404**	block5	Control	post	ceoi3	Subject	Broad	r1	295.0972708	2	2	broad_focus	3	Broad focus
**2023404**	block5	Control	post	caa4	Verb	Broad	r1	262.8115116	3	1	broad_focus	4	Broad focus
**2023404**	block5	Control	post	ngau4	Object	Broad	r1	395.0663156	4	1	broad_focus	4	Broad focus
**2023404**	block5	Control	post	jau4	Object	Broad	r1	210.6045081	5	2	broad_focus	4	Broad focus
**2023404**	block5	Control	post	wai5	Subject	Broad	r1	322.1606307	1	1	broad_focus	5	Broad focus
**2023404**	block5	Control	post	wai5	Subject	Broad	r1	363.3435051	2	2	broad_focus	5	Broad focus
**2023404**	block5	Control	post	waat3	Verb	Broad	r1	328.9840855	3	1	broad_focus	3	Broad focus
**2023404**	block5	Control	post	bui3	Object	Broad	r1	342.6208485	4	1	broad_focus	3	Broad focus
**2023404**	block5	Control	post	hok3	Object	Broad	r1	207.9683725	5	2	broad_focus	3	Broad focus
**2023404**	block5	Control	post	siu2	Subject	Contrastive	r1	717.9269841	1	1	on_focus	2	Contrastive on_focus
**2023404**	block5	Control	post	gwong2	Subject	Contrastive	r1	371.7056333	2	2	on_focus	2	Contrastive on_focus
**2023404**	block5	Control	post	cyun4	Verb	Contrastive	r1	324.3483108	3	1	post_focus	4	Contrastive post_focus
**2023404**	block5	Control	post	laam4	Object	Contrastive	r1	392.3429972	4	1	post_focus	4	Contrastive post_focus
**2023404**	block5	Control	post	kau4	Object	Contrastive	r1	175.0203961	5	2	post_focus	4	Contrastive post_focus
**2023404**	block5	Control	post	siu2	Subject	Contrastive	r1	291.0238924	1	1	pre_focus	2	Contrastive pre_focus
**2023404**	block5	Control	post	gwong2	Subject	Contrastive	r1	283.4719319	2	2	pre_focus	2	Contrastive pre_focus
**2023404**	block5	Control	post	cyun4	Verb	Contrastive	r1	259.892288	3	1	on_focus	4	Contrastive on_focus
**2023404**	block5	Control	post	laam4	Object	Contrastive	r1	431.4183134	4	1	post_focus	4	Contrastive post_focus
**2023404**	block5	Control	post	kau4	Object	Contrastive	r1	140.1933487	5	2	post_focus	4	Contrastive post_focus
**2023404**	block5	Control	post	wai5	Subject	Narrow	r1	270.9894243	1	1	on_focus	5	Narrow on_focus
**2023404**	block5	Control	post	wai5	Subject	Narrow	r1	383.7014137	2	2	on_focus	5	Narrow on_focus
**2023404**	block5	Control	post	waat3	Verb	Narrow	r1	230.078302	3	1	post_focus	3	Narrow post_focus
**2023404**	block5	Control	post	bui3	Object	Narrow	r1	441.2105045	4	1	post_focus	3	Narrow post_focus
**2023404**	block5	Control	post	hok3	Object	Narrow	r1	171.7491798	5	2	post_focus	3	Narrow post_focus

**2023404**	block5	Control	post	siu2	Subject	Contrastive	r2	187.4217562	1	1	pre_focus	2	Contrastive pre_focus
**2023404**	block5	Control	post	gwong2	Subject	Contrastive	r2	368.7885584	2	2	pre_focus	2	Contrastive pre_focus
**2023404**	block5	Control	post	cyun4	Verb	Contrastive	r2	293.935168	3	1	pre_focus	4	Contrastive pre_focus
**2023404**	block5	Control	post	laam4	Object	Contrastive	r2	441.6607292	4	1	on_focus	4	Contrastive on_focus
**2023404**	block5	Control	post	kau4	Object	Contrastive	r2	242.6680667	5	2	on_focus	4	Contrastive on_focus
**2023404**	block5	Control	post	siu2	Subject	Narrow	r2	327.0863993	1	1	pre_focus	2	Narrow pre_focus
**2023404**	block5	Control	post	gwong2	Subject	Narrow	r2	380.6411567	2	2	pre_focus	2	Narrow pre_focus
**2023404**	block5	Control	post	cyun4	Verb	Narrow	r2	235.193946	3	1	on_focus	4	Narrow on_focus
**2023404**	block5	Control	post	laam4	Object	Narrow	r2	533.5287149	4	1	post_focus	4	Narrow post_focus
**2023404**	block5	Control	post	kau4	Object	Narrow	r2	193.2205302	5	2	post_focus	4	Narrow post_focus
**2023404**	block5	Control	post	siu2	Subject	Broad	r2	381.8802505	1	1	broad_focus	2	Broad focus
**2023404**	block5	Control	post	gwong2	Subject	Broad	r2	365.1566188	2	2	broad_focus	2	Broad focus
**2023404**	block5	Control	post	cyun4	Verb	Broad	r2	302.6952901	3	1	broad_focus	4	Broad focus
**2023404**	block5	Control	post	laam4	Object	Broad	r2	472.4168156	4	1	broad_focus	4	Broad focus
**2023404**	block5	Control	post	kau4	Object	Broad	r2	113.5121472	5	2	broad_focus	4	Broad focus
**2023404**	block5	Control	post	siu2	Subject	Narrow	r2	318.7712172	1	1	on_focus	2	Narrow on_focus
**2023404**	block5	Control	post	gwong2	Subject	Narrow	r2	375.6831935	2	2	on_focus	2	Narrow on_focus
**2023404**	block5	Control	post	cyun4	Verb	Narrow	r2	248.1542421	3	1	post_focus	4	Narrow post_focus
**2023404**	block5	Control	post	laam4	Object	Narrow	r2	389.8764114	4	1	post_focus	4	Narrow post_focus
**2023404**	block5	Control	post	kau4	Object	Narrow	r2	118.8828406	5	2	post_focus	4	Narrow post_focus
**2023404**	block5	Control	post	wai5	Subject	Contrastive	r2	265.9179059	1	1	pre_focus	5	Contrastive pre_focus
**2023404**	block5	Control	post	wai5	Subject	Contrastive	r2	352.4147389	2	2	pre_focus	5	Contrastive pre_focus
**2023404**	block5	Control	post	waat3	Verb	Contrastive	r2	238.2624122	3	1	on_focus	3	Contrastive on_focus
**2023404**	block5	Control	post	bui3	Object	Contrastive	r2	395.1421997	4	1	post_focus	3	Contrastive post_focus
**2023404**	block5	Control	post	hok3	Object	Contrastive	r2	208.7806329	5	2	post_focus	3	Contrastive post_focus
**2023404**	block5	Control	post	ceoi3	Subject	Narrow	r2	312.667811	1	1	pre_focus	3	Narrow pre_focus
**2023404**	block5	Control	post	ceoi3	Subject	Narrow	r2	353.4891475	2	2	pre_focus	3	Narrow pre_focus
**2023404**	block5	Control	post	caa4	Verb	Narrow	r2	217.1666137	3	1	on_focus	4	Narrow on_focus
**2023404**	block5	Control	post	ngau4	Object	Narrow	r2	521.7788475	4	1	post_focus	4	Narrow post_focus
**2023404**	block5	Control	post	jau4	Object	Narrow	r2	233.611738	5	2	post_focus	4	Narrow post_focus
**2023404**	block5	Control	post	ceoi3	Subject	Broad	r2	345.752813	1	1	broad_focus	3	Broad focus
**2023404**	block5	Control	post	ceoi3	Subject	Broad	r2	357.3001901	2	2	broad_focus	3	Broad focus
**2023404**	block5	Control	post	caa4	Verb	Broad	r2	312.3306128	3	1	broad_focus	4	Broad focus
**2023404**	block5	Control	post	ngau4	Object	Broad	r2	486.103719	4	1	broad_focus	4	Broad focus
**2023404**	block5	Control	post	jau4	Object	Broad	r2	251.0431615	5	2	broad_focus	4	Broad focus
**2023404**	block5	Control	post	wai5	Subject	Narrow	r2	221.2180113	1	1	pre_focus	5	Narrow pre_focus
**2023404**	block5	Control	post	wai5	Subject	Narrow	r2	301.0198571	2	2	pre_focus	5	Narrow pre_focus
**2023404**	block5	Control	post	waat3	Verb	Narrow	r2	216.3717695	3	1	pre_focus	3	Narrow pre_focus
**2023404**	block5	Control	post	bui3	Object	Narrow	r2	381.4124997	4	1	on_focus	3	Narrow on_focus
**2023404**	block5	Control	post	hok3	Object	Narrow	r2	187.2123597	5	2	on_focus	3	Narrow on_focus
**2023404**	block5	Control	post	wai5	Subject	Contrastive	r2	320.8369581	1	1	pre_focus	5	Contrastive pre_focus
**2023404**	block5	Control	post	wai5	Subject	Contrastive	r2	367.5816534	2	2	pre_focus	5	Contrastive pre_focus
**2023404**	block5	Control	post	waat3	Verb	Contrastive	r2	190.9261357	3	1	pre_focus	3	Contrastive pre_focus
**2023404**	block5	Control	post	bui3	Object	Contrastive	r2	436.0792552	4	1	on_focus	3	Contrastive on_focus
**2023404**	block5	Control	post	hok3	Object	Contrastive	r2	131.9767664	5	2	on_focus	3	Contrastive on_focus
**2023404**	block5	Control	post	ceoi3	Subject	Narrow	r2	290.4429353	1	1	on_focus	3	Narrow on_focus
**2023404**	block5	Control	post	ceoi3	Subject	Narrow	r2	323.9161386	2	2	on_focus	3	Narrow on_focus
**2023404**	block5	Control	post	caa4	Verb	Narrow	r2	258.239103	3	1	post_focus	4	Narrow post_focus
**2023404**	block5	Control	post	ngau4	Object	Narrow	r2	411.8542359	4	1	post_focus	4	Narrow post_focus
**2023404**	block5	Control	post	jau4	Object	Narrow	r2	195.4917066	5	2	post_focus	4	Narrow post_focus
**2023404**	block5	Control	post	ceoi3	Subject	Narrow	r2	251.4029849	1	1	pre_focus	3	Narrow pre_focus
**2023404**	block5	Control	post	ceoi3	Subject	Narrow	r2	288.7306358	2	2	pre_focus	3	Narrow pre_focus
**2023404**	block5	Control	post	caa4	Verb	Narrow	r2	247.3731514	3	1	pre_focus	4	Narrow pre_focus
**2023404**	block5	Control	post	ngau4	Object	Narrow	r2	380.8753724	4	1	on_focus	4	Narrow on_focus
**2023404**	block5	Control	post	jau4	Object	Narrow	r2	178.5498866	5	2	on_focus	4	Narrow on_focus
**2023404**	block5	Control	post	ceoi3	Subject	Contrastive	r2	184.068952	1	1	pre_focus	3	Contrastive pre_focus
**2023404**	block5	Control	post	ceoi3	Subject	Contrastive	r2	268.5970585	2	2	pre_focus	3	Contrastive pre_focus
**2023404**	block5	Control	post	caa4	Verb	Contrastive	r2	338.6755664	3	1	on_focus	4	Contrastive on_focus
**2023404**	block5	Control	post	ngau4	Object	Contrastive	r2	400.2864642	4	1	post_focus	4	Contrastive post_focus
**2023404**	block5	Control	post	jau4	Object	Contrastive	r2	155.5715048	5	2	post_focus	4	Contrastive post_focus
**2023404**	block5	Control	post	wai5	Subject	Broad	r2	296.9605814	1	1	broad_focus	5	Broad focus
**2023404**	block5	Control	post	wai5	Subject	Broad	r2	342.0801079	2	2	broad_focus	5	Broad focus
**2023404**	block5	Control	post	waat3	Verb	Broad	r2	254.2699903	3	1	broad_focus	3	Broad focus
**2023404**	block5	Control	post	bui3	Object	Broad	r2	341.0349398	4	1	broad_focus	3	Broad focus
**2023404**	block5	Control	post	hok3	Object	Broad	r2	146.7533172	5	2	broad_focus	3	Broad focus
**2023404**	block5	Control	post	siu2	Subject	Contrastive	r2	348.1864102	1	1	on_focus	2	Contrastive on_focus

**2023404**	block5	Control	post	gwong2	Subject	Contrastive	r2	412.7381097	2	2	on_focus	2	Contrastive on_focus
**2023404**	block5	Control	post	cyun4	Verb	Contrastive	r2	294.2455483	3	1	post_focus	4	Contrastive post_focus
**2023404**	block5	Control	post	laam4	Object	Contrastive	r2	433.6223403	4	1	post_focus	4	Contrastive post_focus
**2023404**	block5	Control	post	kau4	Object	Contrastive	r2	189.7018894	5	2	post_focus	4	Contrastive post_focus
**2023404**	block5	Control	post	ceoi3	Subject	Contrastive	r2	236.9038597	1	1	pre_focus	3	Contrastive pre_focus
**2023404**	block5	Control	post	ceoi3	Subject	Contrastive	r2	250.5019279	2	2	pre_focus	3	Contrastive pre_focus
**2023404**	block5	Control	post	caa4	Verb	Contrastive	r2	236.8483606	3	1	pre_focus	4	Contrastive pre_focus
**2023404**	block5	Control	post	ngau4	Object	Contrastive	r2	364.364676	4	1	on_focus	4	Contrastive on_focus
**2023404**	block5	Control	post	jau4	Object	Contrastive	r2	271.1750666	5	2	on_focus	4	Contrastive on_focus
**2023404**	block5	Control	post	wai5	Subject	Narrow	r2	307.8763627	1	1	on_focus	5	Narrow on_focus
**2023404**	block5	Control	post	wai5	Subject	Narrow	r2	274.5716618	2	2	on_focus	5	Narrow on_focus
**2023404**	block5	Control	post	waat3	Verb	Narrow	r2	257.5096406	3	1	post_focus	3	Narrow post_focus
**2023404**	block5	Control	post	bui3	Object	Narrow	r2	266.2808588	4	1	post_focus	3	Narrow post_focus
**2023404**	block5	Control	post	hok3	Object	Narrow	r2	139.7085445	5	2	post_focus	3	Narrow post_focus
**2023404**	block5	Control	post	wai5	Subject	Contrastive	r2	254.2693702	1	1	on_focus	5	Contrastive on_focus
**2023404**	block5	Control	post	wai5	Subject	Contrastive	r2	276.4418639	2	2	on_focus	5	Contrastive on_focus
**2023404**	block5	Control	post	waat3	Verb	Contrastive	r2	208.508049	3	1	post_focus	3	Contrastive post_focus
**2023404**	block5	Control	post	bui3	Object	Contrastive	r2	342.7557306	4	1	post_focus	3	Contrastive post_focus
**2023404**	block5	Control	post	hok3	Object	Contrastive	r2	141.5994173	5	2	post_focus	3	Contrastive post_focus
**2023404**	block5	Control	post	siu2	Subject	Contrastive	r2	237.79456	1	1	pre_focus	2	Contrastive pre_focus
**2023404**	block5	Control	post	gwong2	Subject	Contrastive	r2	336.9406695	2	2	pre_focus	2	Contrastive pre_focus
**2023404**	block5	Control	post	cyun4	Verb	Contrastive	r2	219.9868809	3	1	on_focus	4	Contrastive on_focus
**2023404**	block5	Control	post	laam4	Object	Contrastive	r2	455.1250533	4	1	post_focus	4	Contrastive post_focus
**2023404**	block5	Control	post	kau4	Object	Contrastive	r2	261.8011198	5	2	post_focus	4	Contrastive post_focus
**2023404**	block5	Control	post	ceoi3	Subject	Contrastive	r2	238.6189088	1	1	on_focus	3	Contrastive on_focus
**2023404**	block5	Control	post	ceoi3	Subject	Contrastive	r2	290.0282385	2	2	on_focus	3	Contrastive on_focus
**2023404**	block5	Control	post	caa4	Verb	Contrastive	r2	259.6490735	3	1	post_focus	4	Contrastive post_focus
**2023404**	block5	Control	post	ngau4	Object	Contrastive	r2	417.2114087	4	1	post_focus	4	Contrastive post_focus
**2023404**	block5	Control	post	jau4	Object	Contrastive	r2	136.427531	5	2	post_focus	4	Contrastive post_focus
**2023404**	block5	Control	post	wai5	Subject	Narrow	r2	226.9755085	1	1	pre_focus	5	Narrow pre_focus
**2023404**	block5	Control	post	wai5	Subject	Narrow	r2	263.5945232	2	2	pre_focus	5	Narrow pre_focus
**2023404**	block5	Control	post	waat3	Verb	Narrow	r2	238.3240715	3	1	on_focus	3	Narrow on_focus
**2023404**	block5	Control	post	bui3	Object	Narrow	r2	347.0268869	4	1	post_focus	3	Narrow post_focus
**2023404**	block5	Control	post	hok3	Object	Narrow	r2	199.9702537	5	2	post_focus	3	Narrow post_focus
**2023404**	block5	Control	post	siu2	Subject	Narrow	r2	172.4142188	1	1	pre_focus	2	Narrow pre_focus
**2023404**	block5	Control	post	gwong2	Subject	Narrow	r2	382.1188264	2	2	pre_focus	2	Narrow pre_focus
**2023404**	block5	Control	post	cyun4	Verb	Narrow	r2	307.2010054	3	1	pre_focus	4	Narrow pre_focus
**2023404**	block5	Control	post	laam4	Object	Narrow	r2	476.8587086	4	1	on_focus	4	Narrow on_focus
**2023404**	block5	Control	post	kau4	Object	Narrow	r2	247.2681034	5	2	on_focus	4	Narrow on_focus
**2023404**	block5	Control	pre	siu2	Subject	Contrastive	r1	266.1428821	1	1	pre_focus	2	Contrastive pre_focus
**2023404**	block5	Control	pre	gwong2	Subject	Contrastive	r1	298.5100006	2	2	pre_focus	2	Contrastive pre_focus
**2023404**	block5	Control	pre	cyun4	Verb	Contrastive	r1	201.6627299	3	1	pre_focus	4	Contrastive pre_focus
**2023404**	block5	Control	pre	laam4	Object	Contrastive	r1	523.7210884	4	1	on_focus	4	Contrastive on_focus
**2023404**	block5	Control	pre	kau4	Object	Contrastive	r1	150.2494331	5	2	on_focus	4	Contrastive on_focus
**2023404**	block5	Control	pre	wai5	Subject	Contrastive	r1	277.379201	1	1	pre_focus	5	Contrastive pre_focus
**2023404**	block5	Control	pre	wai5	Subject	Contrastive	r1	311.6130362	2	2	pre_focus	5	Contrastive pre_focus
**2023404**	block5	Control	pre	waat3	Verb	Contrastive	r1	253.0107885	3	1	pre_focus	3	Contrastive pre_focus
**2023404**	block5	Control	pre	bui3	Object	Contrastive	r1	343.3665911	4	1	on_focus	3	Contrastive on_focus
**2023404**	block5	Control	pre	hok3	Object	Contrastive	r1	137.8167492	5	2	on_focus	3	Contrastive on_focus
**2023404**	block5	Control	pre	ceoi3	Subject	Contrastive	r1	354.1099898	1	1	pre_focus	3	Contrastive pre_focus
**2023404**	block5	Control	pre	ceoi3	Subject	Contrastive	r1	208.9645542	2	2	pre_focus	3	Contrastive pre_focus
**2023404**	block5	Control	pre	caa4	Verb	Contrastive	r1	251.255438	3	1	pre_focus	4	Contrastive pre_focus
**2023404**	block5	Control	pre	ngau4	Object	Contrastive	r1	339.8309131	4	1	on_focus	4	Contrastive on_focus
**2023404**	block5	Control	pre	jau4	Object	Contrastive	r1	232.9606954	5	2	on_focus	4	Contrastive on_focus
**2023404**	block5	Control	pre	wai5	Subject	Narrow	r1	232.4206349	1	1	pre_focus	5	Narrow pre_focus
**2023404**	block5	Control	pre	wai5	Subject	Narrow	r1	250.5174927	2	2	pre_focus	5	Narrow pre_focus
**2023404**	block5	Control	pre	waat3	Verb	Narrow	r1	284.1751701	3	1	pre_focus	3	Narrow pre_focus
**2023404**	block5	Control	pre	bui3	Object	Narrow	r1	323.5698504	4	1	on_focus	3	Narrow on_focus
**2023404**	block5	Control	pre	hok3	Object	Narrow	r1	172.0626331	5	2	on_focus	3	Narrow on_focus
**2023404**	block5	Control	pre	ceoi3	Subject	Narrow	r1	194.3783069	1	1	pre_focus	3	Narrow pre_focus
**2023404**	block5	Control	pre	ceoi3	Subject	Narrow	r1	238.5059929	2	2	pre_focus	3	Narrow pre_focus
**2023404**	block5	Control	pre	caa4	Verb	Narrow	r1	213.4986116	3	1	pre_focus	4	Narrow pre_focus
**2023404**	block5	Control	pre	ngau4	Object	Narrow	r1	313.7514547	4	1	on_focus	4	Narrow on_focus
**2023404**	block5	Control	pre	jau4	Object	Narrow	r1	144.8009574	5	2	on_focus	4	Narrow on_focus
**2023404**	block5	Control	pre	siu2	Subject	Contrastive	r1	283.4348015	1	1	pre_focus	2	Contrastive pre_focus
**2023404**	block5	Control	pre	gwong2	Subject	Contrastive	r1	321.4535659	2	2	pre_focus	2	Contrastive pre_focus

**2023404**	block5	Control	pre	cyun4	Verb	Contrastive	r1	296.8041383	3	1	on_focus	4	Contrastive on_focus
**2023404**	block5	Control	pre	laam4	Object	Contrastive	r1	380.9966422	4	1	post_focus	4	Contrastive post_focus
**2023404**	block5	Control	pre	kau4	Object	Contrastive	r1	216.9761905	5	2	post_focus	4	Contrastive post_focus
**2023404**	block5	Control	pre	ceoi3	Subject	Narrow	r1	318.313897	1	1	pre_focus	3	Narrow pre_focus
**2023404**	block5	Control	pre	ceoi3	Subject	Narrow	r1	239.7067659	2	2	pre_focus	3	Narrow pre_focus
**2023404**	block5	Control	pre	caa4	Verb	Narrow	r1	268.6874115	3	1	on_focus	4	Narrow on_focus
**2023404**	block5	Control	pre	ngau4	Object	Narrow	r1	409.0246804	4	1	post_focus	4	Narrow post_focus
**2023404**	block5	Control	pre	jau4	Object	Narrow	r1	259.6643631	5	2	post_focus	4	Narrow post_focus
**2023404**	block5	Control	pre	ceoi3	Subject	Contrastive	r1	274.7929705	1	1	pre_focus	3	Contrastive pre_focus
**2023404**	block5	Control	pre	ceoi3	Subject	Contrastive	r1	189.3224765	2	2	pre_focus	3	Contrastive pre_focus
**2023404**	block5	Control	pre	caa4	Verb	Contrastive	r1	250.4708995	3	1	on_focus	4	Contrastive on_focus
**2023404**	block5	Control	pre	ngau4	Object	Contrastive	r1	328.1090791	4	1	post_focus	4	Contrastive post_focus
**2023404**	block5	Control	pre	jau4	Object	Contrastive	r1	265.6386471	5	2	post_focus	4	Contrastive post_focus
**2023404**	block5	Control	pre	wai5	Subject	Narrow	r1	373.4567901	1	1	on_focus	5	Narrow on_focus
**2023404**	block5	Control	pre	wai5	Subject	Narrow	r1	269.9067925	2	2	on_focus	5	Narrow on_focus
**2023404**	block5	Control	pre	waat3	Verb	Narrow	r1	341.0286281	3	1	post_focus	3	Narrow post_focus
**2023404**	block5	Control	pre	bui3	Object	Narrow	r1	202.3582766	4	1	post_focus	3	Narrow post_focus
**2023404**	block5	Control	pre	hok3	Object	Narrow	r1	160.3789465	5	2	post_focus	3	Narrow post_focus
**2023404**	block5	Control	pre	siu2	Subject	Narrow	r1	155.6500378	1	1	pre_focus	2	Narrow pre_focus
**2023404**	block5	Control	pre	gwong2	Subject	Narrow	r1	376.8178382	2	2	pre_focus	2	Narrow pre_focus
**2023404**	block5	Control	pre	cyun4	Verb	Narrow	r1	304.5091896	3	1	on_focus	4	Narrow on_focus
**2023404**	block5	Control	pre	laam4	Object	Narrow	r1	490.3708785	4	1	post_focus	4	Narrow post_focus
**2023404**	block5	Control	pre	kau4	Object	Narrow	r1	183.6281179	5	2	post_focus	4	Narrow post_focus
**2023404**	block5	Control	pre	siu2	Subject	Contrastive	r1	262.8093826	1	1	on_focus	2	Contrastive on_focus
**2023404**	block5	Control	pre	gwong2	Subject	Contrastive	r1	275.7215797	2	2	on_focus	2	Contrastive on_focus
**2023404**	block5	Control	pre	cyun4	Verb	Contrastive	r1	258.8270975	3	1	post_focus	4	Contrastive post_focus
**2023404**	block5	Control	pre	laam4	Object	Contrastive	r1	342.8620019	4	1	post_focus	4	Contrastive post_focus
**2023404**	block5	Control	pre	kau4	Object	Contrastive	r1	163.9247663	5	2	post_focus	4	Contrastive post_focus
**2023404**	block5	Control	pre	siu2	Subject	Narrow	r1	244.5253212	1	1	pre_focus	2	Narrow pre_focus
**2023404**	block5	Control	pre	gwong2	Subject	Narrow	r1	318.4446703	2	2	pre_focus	2	Narrow pre_focus
**2023404**	block5	Control	pre	cyun4	Verb	Narrow	r1	211.7845805	3	1	pre_focus	4	Narrow pre_focus
**2023404**	block5	Control	pre	laam4	Object	Narrow	r1	269.1824059	4	1	on_focus	4	Narrow on_focus
**2023404**	block5	Control	pre	kau4	Object	Narrow	r1	197.7134162	5	2	on_focus	4	Narrow on_focus
**2023404**	block5	Control	pre	ceoi3	Subject	Broad	r1	275.6362275	1	1	broad_focus	3	Broad focus
**2023404**	block5	Control	pre	ceoi3	Subject	Broad	r1	277.8849389	2	2	broad_focus	3	Broad focus
**2023404**	block5	Control	pre	caa4	Verb	Broad	r1	299.8928571	3	1	broad_focus	4	Broad focus
**2023404**	block5	Control	pre	ngau4	Object	Broad	r1	269.4104308	4	1	broad_focus	4	Broad focus
**2023404**	block5	Control	pre	jau4	Object	Broad	r1	231.7704744	5	2	broad_focus	4	Broad focus
**2023404**	block5	Control	pre	siu2	Subject	Broad	r1	266.5443797	1	1	broad_focus	2	Broad focus
**2023404**	block5	Control	pre	gwong2	Subject	Broad	r1	335.6304509	2	2	broad_focus	2	Broad focus
**2023404**	block5	Control	pre	cyun4	Verb	Broad	r1	228.8643746	3	1	broad_focus	4	Broad focus
**2023404**	block5	Control	pre	laam4	Object	Broad	r1	511.4729781	4	1	broad_focus	4	Broad focus
**2023404**	block5	Control	pre	kau4	Object	Broad	r1	96.44979697	5	2	broad_focus	4	Broad focus
**2023404**	block5	Control	pre	ceoi3	Subject	Narrow	r1	264.5279866	1	1	on_focus	3	Narrow on_focus
**2023404**	block5	Control	pre	ceoi3	Subject	Narrow	r1	247.7502541	2	2	on_focus	3	Narrow on_focus
**2023404**	block5	Control	pre	caa4	Verb	Narrow	r1	298.197027	3	1	post_focus	4	Narrow post_focus
**2023404**	block5	Control	pre	ngau4	Object	Narrow	r1	276.7616057	4	1	post_focus	4	Narrow post_focus
**2023404**	block5	Control	pre	jau4	Object	Narrow	r1	168.6009757	5	2	post_focus	4	Narrow post_focus
**2023404**	block5	Control	pre	wai5	Subject	Contrastive	r1	390.2999779	1	1	on_focus	5	Contrastive on_focus
**2023404**	block5	Control	pre	wai5	Subject	Contrastive	r1	280.3087877	2	2	on_focus	5	Contrastive on_focus
**2023404**	block5	Control	pre	waat3	Verb	Contrastive	r1	348.0025615	3	1	post_focus	3	Contrastive post_focus
**2023404**	block5	Control	pre	bui3	Object	Contrastive	r1	366.9720333	4	1	post_focus	3	Contrastive post_focus
**2023404**	block5	Control	pre	hok3	Object	Contrastive	r1	150.1479592	5	2	post_focus	3	Contrastive post_focus
**2023404**	block5	Control	pre	siu2	Subject	Narrow	r1	201.7950672	1	1	on_focus	2	Narrow on_focus
**2023404**	block5	Control	pre	gwong2	Subject	Narrow	r1	374.5650646	2	2	on_focus	2	Narrow on_focus
**2023404**	block5	Control	pre	cyun4	Verb	Narrow	r1	297.7581255	3	1	post_focus	4	Narrow post_focus
**2023404**	block5	Control	pre	laam4	Object	Narrow	r1	385.6979088	4	1	post_focus	4	Narrow post_focus
**2023404**	block5	Control	pre	kau4	Object	Narrow	r1	153.8824101	5	2	post_focus	4	Narrow post_focus
**2023404**	block5	Control	pre	wai5	Subject	Contrastive	r1	313.6935646	1	1	pre_focus	5	Contrastive pre_focus
**2023404**	block5	Control	pre	wai5	Subject	Contrastive	r1	277.1159293	2	2	pre_focus	5	Contrastive pre_focus
**2023404**	block5	Control	pre	waat3	Verb	Contrastive	r1	331.4219749	3	1	on_focus	3	Contrastive on_focus
**2023404**	block5	Control	pre	bui3	Object	Contrastive	r1	374.5089768	4	1	post_focus	3	Contrastive post_focus
**2023404**	block5	Control	pre	hok3	Object	Contrastive	r1	157.6952772	5	2	post_focus	3	Contrastive post_focus
**2023404**	block5	Control	pre	ceoi3	Subject	Contrastive	r1	248.8385459	1	1	on_focus	3	Contrastive on_focus
**2023404**	block5	Control	pre	ceoi3	Subject	Contrastive	r1	312.8416291	2	2	on_focus	3	Contrastive on_focus
**2023404**	block5	Control	pre	caa4	Verb	Contrastive	r1	307.6194485	3	1	post_focus	4	Contrastive post_focus

2023404	block5	Control	pre	ngau4	Object	Contrastive	r1	385.1284958	4	1	post_focus	4	Contrastive post_focus
2023404	block5	Control	pre	jau4	Object	Contrastive	r1	253.5769257	5	2	post_focus	4	Contrastive post_focus
2023404	block5	Control	pre	wai5	Subject	Broad	r1	273.8894703	1	1	broad_focus	5	Broad focus
2023404	block5	Control	pre	wai5	Subject	Broad	r1	313.3594502	2	2	broad_focus	5	Broad focus
2023404	block5	Control	pre	waat3	Verb	Broad	r1	187.4271661	3	1	broad_focus	3	Broad focus
2023404	block5	Control	pre	bui3	Object	Broad	r1	311.962585	4	1	broad_focus	3	Broad focus
2023404	block5	Control	pre	hok3	Object	Broad	r1	164.832433	5	2	broad_focus	3	Broad focus
2023404	block5	Control	pre	wai5	Subject	Narrow	r1	201.2305367	1	1	pre_focus	5	Narrow pre_focus
2023404	block5	Control	pre	wai5	Subject	Narrow	r1	246.7557663	2	2	pre_focus	5	Narrow pre_focus
2023404	block5	Control	pre	waat3	Verb	Narrow	r1	234.2672378	3	1	on_focus	3	Narrow on_focus
2023404	block5	Control	pre	bui3	Object	Narrow	r1	283.1282051	4	1	post_focus	3	Narrow post_focus
2023404	block5	Control	pre	hok3	Object	Narrow	r1	172.1012192	5	2	post_focus	3	Narrow post_focus
2023404	block5	Control	pre	siu2	Subject	Narrow	r2	203.919741	1	1	pre_focus	2	Narrow pre_focus
2023404	block5	Control	pre	gwong2	Subject	Narrow	r2	369.7140885	2	2	pre_focus	2	Narrow pre_focus
2023404	block5	Control	pre	cyun4	Verb	Narrow	r2	326.5986395	3	1	pre_focus	4	Narrow pre_focus
2023404	block5	Control	pre	laam4	Object	Narrow	r2	442.6807086	4	1	on_focus	4	Narrow on_focus
2023404	block5	Control	pre	kau4	Object	Narrow	r2	79.74025974	5	2	on_focus	4	Narrow on_focus
2023404	block5	Control	pre	siu2	Subject	Narrow	r2	325.8008658	1	1	pre_focus	2	Narrow pre_focus
2023404	block5	Control	pre	gwong2	Subject	Narrow	r2	404.137767	2	2	pre_focus	2	Narrow pre_focus
2023404	block5	Control	pre	cyun4	Verb	Narrow	r2	272.3719402	3	1	on_focus	4	Narrow on_focus
2023404	block5	Control	pre	laam4	Object	Narrow	r2	350.1238632	4	1	post_focus	4	Narrow post_focus
2023404	block5	Control	pre	kau4	Object	Narrow	r2	167.1345872	5	2	post_focus	4	Narrow post_focus
2023404	block5	Control	pre	wai5	Subject	Contrastive	r2	319.5509011	1	1	on_focus	5	Contrastive on_focus
2023404	block5	Control	pre	wai5	Subject	Contrastive	r2	348.3920923	2	2	on_focus	5	Contrastive on_focus
2023404	block5	Control	pre	waat3	Verb	Contrastive	r2	300.0932225	3	1	post_focus	3	Contrastive post_focus
2023404	block5	Control	pre	bui3	Object	Contrastive	r2	233.0409783	4	1	post_focus	3	Contrastive post_focus
2023404	block5	Control	pre	hok3	Object	Contrastive	r2	192.6775814	5	2	post_focus	3	Contrastive post_focus
2023404	block5	Control	pre	ceoi3	Subject	Contrastive	r2	238.2967102	1	1	pre_focus	3	Contrastive pre_focus
2023404	block5	Control	pre	ceoi3	Subject	Contrastive	r2	288.2114015	2	2	pre_focus	3	Contrastive pre_focus
2023404	block5	Control	pre	caa4	Verb	Contrastive	r2	249.00869	3	1	pre_focus	4	Contrastive pre_focus
2023404	block5	Control	pre	ngau4	Object	Contrastive	r2	300.8064317	4	1	on_focus	4	Contrastive on_focus
2023404	block5	Control	pre	jau4	Object	Contrastive	r2	284.4341725	5	2	on_focus	4	Contrastive on_focus
2023404	block5	Control	pre	ceoi3	Subject	Narrow	r2	234.104696	1	1	on_focus	3	Narrow on_focus
2023404	block5	Control	pre	ceoi3	Subject	Narrow	r2	241.6841586	2	2	on_focus	3	Narrow on_focus
2023404	block5	Control	pre	caa4	Verb	Narrow	r2	252.0997438	3	1	post_focus	4	Narrow post_focus
2023404	block5	Control	pre	ngau4	Object	Narrow	r2	322.5369195	4	1	post_focus	4	Narrow post_focus
2023404	block5	Control	pre	jau4	Object	Narrow	r2	217.4580499	5	2	post_focus	4	Narrow post_focus
2023404	block5	Control	pre	siu2	Subject	Broad	r2	185.4242364	1	1	broad_focus	2	Broad focus
2023404	block5	Control	pre	gwong2	Subject	Broad	r2	307.0954061	2	2	broad_focus	2	Broad focus
2023404	block5	Control	pre	cyun4	Verb	Broad	r2	175.2391075	3	1	broad_focus	4	Broad focus
2023404	block5	Control	pre	laam4	Object	Broad	r2	418.3781449	4	1	broad_focus	4	Broad focus
2023404	block5	Control	pre	kau4	Object	Broad	r2	179.7642917	5	2	broad_focus	4	Broad focus
2023404	block5	Control	pre	wai5	Subject	Narrow	r2	196.9078262	1	1	pre_focus	5	Narrow pre_focus
2023404	block5	Control	pre	wai5	Subject	Narrow	r2	230.9913477	2	2	pre_focus	5	Narrow pre_focus
2023404	block5	Control	pre	waat3	Verb	Narrow	r2	272.8966014	3	1	pre_focus	3	Narrow pre_focus
2023404	block5	Control	pre	bui3	Object	Narrow	r2	394.2130489	4	1	on_focus	3	Narrow on_focus
2023404	block5	Control	pre	hok3	Object	Narrow	r2	170.7946056	5	2	on_focus	3	Narrow on_focus
2023404	block5	Control	pre	wai5	Subject	Broad	r2	246.5985675	1	1	broad_focus	5	Broad focus
2023404	block5	Control	pre	wai5	Subject	Broad	r2	261.6994205	2	2	broad_focus	5	Broad focus
2023404	block5	Control	pre	waat3	Verb	Broad	r2	246.9817115	3	1	broad_focus	3	Broad focus
2023404	block5	Control	pre	bui3	Object	Broad	r2	427.5817586	4	1	broad_focus	3	Broad focus
2023404	block5	Control	pre	hok3	Object	Broad	r2	194.6874528	5	2	broad_focus	3	Broad focus
2023404	block5	Control	pre	ceoi3	Subject	Contrastive	r2	262.0544218	1	1	on_focus	3	Contrastive on_focus
2023404	block5	Control	pre	ceoi3	Subject	Contrastive	r2	255.6544876	2	2	on_focus	3	Contrastive on_focus
2023404	block5	Control	pre	caa4	Verb	Contrastive	r2	227.9876395	3	1	post_focus	4	Contrastive post_focus
2023404	block5	Control	pre	ngau4	Object	Contrastive	r2	334.3008314	4	1	post_focus	4	Contrastive post_focus
2023404	block5	Control	pre	jau4	Object	Contrastive	r2	259.8378955	5	2	post_focus	4	Contrastive post_focus
2023404	block5	Control	pre	wai5	Subject	Narrow	r2	256.9689018	1	1	pre_focus	5	Narrow pre_focus
2023404	block5	Control	pre	wai5	Subject	Narrow	r2	311.3852608	2	2	pre_focus	5	Narrow pre_focus
2023404	block5	Control	pre	waat3	Verb	Narrow	r2	312.0853234	3	1	on_focus	3	Narrow on_focus
2023404	block5	Control	pre	bui3	Object	Narrow	r2	419.9427139	4	1	post_focus	3	Narrow post_focus
2023404	block5	Control	pre	hok3	Object	Narrow	r2	171.0330058	5	2	post_focus	3	Narrow post_focus
2023404	block5	Control	pre	ceoi3	Subject	Narrow	r2	218.5433563	1	1	pre_focus	3	Narrow pre_focus
2023404	block5	Control	pre	ceoi3	Subject	Narrow	r2	283.6587722	2	2	pre_focus	3	Narrow pre_focus
2023404	block5	Control	pre	caa4	Verb	Narrow	r2	250.9430677	3	1	on_focus	4	Narrow on_focus
2023404	block5	Control	pre	ngau4	Object	Narrow	r2	358.4353741	4	1	post_focus	4	Narrow post_focus

**2023404**	block5	Control	pre	jau4	Object	Narrow	r2	228.4051398	5	2	post_focus	4	Narrow post_focus
**2023404**	block5	Control	pre	wai5	Subject	Contrastive	r2	254.1288487	1	1	pre_focus	5	Contrastive pre_focus
**2023404**	block5	Control	pre	wai5	Subject	Contrastive	r2	250.9439405	2	2	pre_focus	5	Contrastive pre_focus
**2023404**	block5	Control	pre	waat3	Verb	Contrastive	r2	266.2365853	3	1	pre_focus	3	Contrastive pre_focus
**2023404**	block5	Control	pre	bui3	Object	Contrastive	r2	390.3221518	4	1	on_focus	3	Contrastive on_focus
**2023404**	block5	Control	pre	hok3	Object	Contrastive	r2	176.0821867	5	2	on_focus	3	Contrastive on_focus
**2023404**	block5	Control	pre	ceoi3	Subject	Contrastive	r2	213.3727459	1	1	pre_focus	3	Contrastive pre_focus
**2023404**	block5	Control	pre	ceoi3	Subject	Contrastive	r2	248.0496256	2	2	pre_focus	3	Contrastive pre_focus
**2023404**	block5	Control	pre	caa4	Verb	Contrastive	r2	237.2014607	3	1	on_focus	4	Contrastive on_focus
**2023404**	block5	Control	pre	ngau4	Object	Contrastive	r2	374.6014303	4	1	post_focus	4	Contrastive post_focus
**2023404**	block5	Control	pre	jau4	Object	Contrastive	r2	179.4407378	5	2	post_focus	4	Contrastive post_focus
**2023404**	block5	Control	pre	siu2	Subject	Narrow	r2	289.1272991	1	1	on_focus	2	Narrow on_focus
**2023404**	block5	Control	pre	gwong2	Subject	Narrow	r2	332.1838479	2	2	on_focus	2	Narrow on_focus
**2023404**	block5	Control	pre	cyun4	Verb	Narrow	r2	243.6437075	3	1	post_focus	4	Narrow post_focus
**2023404**	block5	Control	pre	laam4	Object	Narrow	r2	279.9546485	4	1	post_focus	4	Narrow post_focus
**2023404**	block5	Control	pre	kau4	Object	Narrow	r2	219.6821348	5	2	post_focus	4	Narrow post_focus
**2023404**	block5	Control	pre	ceoi3	Subject	Narrow	r2	233.6206943	1	1	pre_focus	3	Narrow pre_focus
**2023404**	block5	Control	pre	ceoi3	Subject	Narrow	r2	234.9405315	2	2	pre_focus	3	Narrow pre_focus
**2023404**	block5	Control	pre	caa4	Verb	Narrow	r2	221.0923382	3	1	pre_focus	4	Narrow pre_focus
**2023404**	block5	Control	pre	ngau4	Object	Narrow	r2	310.4928193	4	1	on_focus	4	Narrow on_focus
**2023404**	block5	Control	pre	jau4	Object	Narrow	r2	292.7567781	5	2	on_focus	4	Narrow on_focus
**2023404**	block5	Control	pre	ceoi3	Subject	Broad	r2	279.1365777	1	1	broad_focus	3	Broad focus
**2023404**	block5	Control	pre	ceoi3	Subject	Broad	r2	271.2979477	2	2	broad_focus	3	Broad focus
**2023404**	block5	Control	pre	caa4	Verb	Broad	r2	274.2535903	3	1	broad_focus	4	Broad focus
**2023404**	block5	Control	pre	ngau4	Object	Broad	r2	285.2380952	4	1	broad_focus	4	Broad focus
**2023404**	block5	Control	pre	jau4	Object	Broad	r2	158.4546142	5	2	broad_focus	4	Broad focus
**2023404**	block5	Control	pre	siu2	Subject	Contrastive	r2	216.7334845	1	1	pre_focus	2	Contrastive pre_focus
**2023404**	block5	Control	pre	gwong2	Subject	Contrastive	r2	332.4958076	2	2	pre_focus	2	Contrastive pre_focus
**2023404**	block5	Control	pre	cyun4	Verb	Contrastive	r2	289.3657643	3	1	on_focus	4	Contrastive on_focus
**2023404**	block5	Control	pre	laam4	Object	Contrastive	r2	248.9753401	4	1	post_focus	4	Contrastive post_focus
**2023404**	block5	Control	pre	kau4	Object	Contrastive	r2	141.5738616	5	2	post_focus	4	Contrastive post_focus
**2023404**	block5	Control	pre	wai5	Subject	Contrastive	r2	353.9548547	1	1	pre_focus	5	Contrastive pre_focus
**2023404**	block5	Control	pre	wai5	Subject	Contrastive	r2	366.1495595	2	2	pre_focus	5	Contrastive pre_focus
**2023404**	block5	Control	pre	waat3	Verb	Contrastive	r2	306.1565869	3	1	on_focus	3	Contrastive on_focus
**2023404**	block5	Control	pre	bui3	Object	Contrastive	r2	451.5155815	4	1	post_focus	3	Contrastive post_focus
**2023404**	block5	Control	pre	hok3	Object	Contrastive	r2	211.7676002	5	2	post_focus	3	Contrastive post_focus
**2023404**	block5	Control	pre	siu2	Subject	Contrastive	r2	368.9880568	1	1	on_focus	2	Contrastive on_focus
**2023404**	block5	Control	pre	gwong2	Subject	Contrastive	r2	352.9235954	2	2	on_focus	2	Contrastive on_focus
**2023404**	block5	Control	pre	cyun4	Verb	Contrastive	r2	286.2512884	3	1	post_focus	4	Contrastive post_focus
**2023404**	block5	Control	pre	laam4	Object	Contrastive	r2	407.3415398	4	1	post_focus	4	Contrastive post_focus
**2023404**	block5	Control	pre	kau4	Object	Contrastive	r2	97.52985639	5	2	post_focus	4	Contrastive post_focus
**2023404**	block5	Control	pre	siu2	Subject	Contrastive	r2	239.1768419	1	1	pre_focus	2	Contrastive pre_focus
**2023404**	block5	Control	pre	gwong2	Subject	Contrastive	r2	281.9703119	2	2	pre_focus	2	Contrastive pre_focus
**2023404**	block5	Control	pre	cyun4	Verb	Contrastive	r2	218.0395347	3	1	pre_focus	4	Contrastive pre_focus
**2023404**	block5	Control	pre	laam4	Object	Contrastive	r2	428.814956	4	1	on_focus	4	Contrastive on_focus
**2023404**	block5	Control	pre	kau4	Object	Contrastive	r2	100.5350953	5	2	on_focus	4	Contrastive on_focus
**2023404**	block5	Control	pre	wai5	Subject	Narrow	r2	267.9039116	1	1	on_focus	5	Narrow on_focus
**2023404**	block5	Control	pre	wai5	Subject	Narrow	r2	271.818748	2	2	on_focus	5	Narrow on_focus
**2023404**	block5	Control	pre	waat3	Verb	Narrow	r2	204.324557	3	1	post_focus	3	Narrow post_focus
**2023404**	block5	Control	pre	bui3	Object	Narrow	r2	354.0842782	4	1	post_focus	3	Narrow post_focus
**2023404**	block5	Control	pre	hok3	Object	Narrow	r2	160.0303172	5	2	post_focus	3	Narrow post_focus
**2023405**	block1	Control	post	sau3	Subject	Contrastive	r1	295.7727418	1	1	on_focus	3	Contrastive on_focus
**2023405**	block1	Control	post	sau3	Subject	Contrastive	r1	184.369257	2	2	on_focus	3	Contrastive on_focus
**2023405**	block1	Control	post	sik3	Verb	Contrastive	r1	123.8676421	3	1	post_focus	3	Contrastive post_focus
**2023405**	block1	Control	post	baak3	Object	Contrastive	r1	167.5284727	4	1	post_focus	3	Contrastive post_focus
**2023405**	block1	Control	post	baak3	Object	Contrastive	r1	182.0365118	5	2	post_focus	3	Contrastive post_focus
**2023405**	block1	Control	post	jyun2	Subject	Narrow	r1	317.3868238	1	1	pre_focus	2	Narrow pre_focus
**2023405**	block1	Control	post	jyun2	Subject	Narrow	r1	266.9752488	2	2	pre_focus	2	Narrow pre_focus
**2023405**	block1	Control	post	mo2	Verb	Narrow	r1	350.2167693	3	1	pre_focus	2	Narrow pre_focus
**2023405**	block1	Control	post	gau2	Object	Narrow	r1	268.1254677	4	1	on_focus	2	Narrow on_focus
**2023405**	block1	Control	post	zai2	Object	Narrow	r1	357.268097	5	2	on_focus	2	Narrow on_focus
**2023405**	block1	Control	post	sau3	Subject	Contrastive	r1	144.6816499	1	1	pre_focus	3	Contrastive pre_focus
**2023405**	block1	Control	post	sau3	Subject	Contrastive	r1	184.644987	2	2	pre_focus	3	Contrastive pre_focus
**2023405**	block1	Control	post	sik3	Verb	Contrastive	r1	122.1615822	3	1	on_focus	3	Contrastive on_focus
**2023405**	block1	Control	post	baak3	Object	Contrastive	r1	168.4381983	4	1	post_focus	3	Contrastive post_focus
**2023405**	block1	Control	post	baak3	Object	Contrastive	r1	198.1633449	5	2	post_focus	3	Contrastive post_focus

2023405	block1	Control	post	jyun2	Subject	Contrastive	r1	258.9543672	1	1	on_focus	2	Contrastive on_focus
2023405	block1	Control	post	jyun2	Subject	Contrastive	r1	203.7971053	2	2	on_focus	2	Contrastive on_focus
2023405	block1	Control	post	mo2	Verb	Contrastive	r1	262.6437866	3	1	post_focus	2	Contrastive post_focus
2023405	block1	Control	post	gau2	Object	Contrastive	r1	231.2961142	4	1	post_focus	2	Contrastive post_focus
2023405	block1	Control	post	zai2	Object	Contrastive	r1	327.333855	5	2	post_focus	2	Contrastive post_focus
2023405	block1	Control	post	sau3	Subject	Broad	r1	248.3194975	1	1	broad_focus	3	Broad focus
2023405	block1	Control	post	sau3	Subject	Broad	r1	242.615759	2	2	broad_focus	3	Broad focus
2023405	block1	Control	post	sik3	Verb	Broad	r1	140.6844608	3	1	broad_focus	3	Broad focus
2023405	block1	Control	post	baak3	Object	Broad	r1	155.7780492	4	1	broad_focus	3	Broad focus
2023405	block1	Control	post	baak3	Object	Broad	r1	224.408742	5	2	broad_focus	3	Broad focus
2023405	block1	Control	post	zoeng1	Subject	Broad	r1	228.997378	1	1	broad_focus	1	Broad focus
2023405	block1	Control	post	saang1	Subject	Broad	r1	261.8183038	2	2	broad_focus	1	Broad focus
2023405	block1	Control	post	tsa1	Verb	Broad	r1	229.4183757	3	1	broad_focus	1	Broad focus
2023405	block1	Control	post	fei1	Object	Broad	r1	248.035369	4	1	broad_focus	1	Broad focus
2023405	block1	Control	post	gei1	Object	Broad	r1	329.3666117	5	2	broad_focus	1	Broad focus
2023405	block1	Control	post	jyun2	Subject	Narrow	r1	255.5638333	1	1	pre_focus	2	Narrow pre_focus
2023405	block1	Control	post	jyun2	Subject	Narrow	r1	223.4712079	2	2	pre_focus	2	Narrow pre_focus
2023405	block1	Control	post	mo2	Verb	Narrow	r1	282.3534634	3	1	on_focus	2	Narrow on_focus
2023405	block1	Control	post	gau2	Object	Narrow	r1	217.504396	4	1	post_focus	2	Narrow post_focus
2023405	block1	Control	post	zai2	Object	Narrow	r1	309.9714699	5	2	post_focus	2	Narrow post_focus
2023405	block1	Control	post	zoeng1	Subject	Narrow	r1	216.737122	1	1	pre_focus	1	Narrow pre_focus
2023405	block1	Control	post	saang1	Subject	Narrow	r1	220.4734063	2	2	pre_focus	1	Narrow pre_focus
2023405	block1	Control	post	tsa1	Verb	Narrow	r1	225.6844575	3	1	pre_focus	1	Narrow pre_focus
2023405	block1	Control	post	fei1	Object	Narrow	r1	282.4690426	4	1	on_focus	1	Narrow on_focus
2023405	block1	Control	post	gei1	Object	Narrow	r1	346.8689241	5	2	on_focus	1	Narrow on_focus
2023405	block1	Control	post	jyun2	Subject	Broad	r1	273.2687056	1	1	broad_focus	2	Broad focus
2023405	block1	Control	post	jyun2	Subject	Broad	r1	217.3525737	2	2	broad_focus	2	Broad focus
2023405	block1	Control	post	mo2	Verb	Broad	r1	282.6582253	3	1	broad_focus	2	Broad focus
2023405	block1	Control	post	gau2	Object	Broad	r1	283.6217034	4	1	broad_focus	2	Broad focus
2023405	block1	Control	post	zai2	Object	Broad	r1	371.3570746	5	2	broad_focus	2	Broad focus
2023405	block1	Control	post	sau3	Subject	Narrow	r1	188.121886	1	1	pre_focus	3	Narrow pre_focus
2023405	block1	Control	post	sau3	Subject	Narrow	r1	214.7892723	2	2	pre_focus	3	Narrow pre_focus
2023405	block1	Control	post	sik3	Verb	Narrow	r1	138.372222	3	1	on_focus	3	Narrow on_focus
2023405	block1	Control	post	baak3	Object	Narrow	r1	148.4836685	4	1	post_focus	3	Narrow post_focus
2023405	block1	Control	post	baak3	Object	Narrow	r1	147.9102438	5	2	post_focus	3	Narrow post_focus
2023405	block1	Control	post	sau3	Subject	Contrastive	r1	113.1153103	1	1	pre_focus	3	Contrastive pre_focus
2023405	block1	Control	post	sau3	Subject	Contrastive	r1	170.4367054	2	2	pre_focus	3	Contrastive pre_focus
2023405	block1	Control	post	sik3	Verb	Contrastive	r1	126.2551271	3	1	pre_focus	3	Contrastive pre_focus
2023405	block1	Control	post	baak3	Object	Contrastive	r1	193.2307851	4	1	on_focus	3	Contrastive on_focus
2023405	block1	Control	post	baak3	Object	Contrastive	r1	399.854808	5	2	on_focus	3	Contrastive on_focus
2023405	block1	Control	post	zoeng1	Subject	Contrastive	r1	187.3017814	1	1	pre_focus	1	Contrastive pre_focus
2023405	block1	Control	post	saang1	Subject	Contrastive	r1	213.5022785	2	2	pre_focus	1	Contrastive pre_focus
2023405	block1	Control	post	tsa1	Verb	Contrastive	r1	169.8042669	3	1	pre_focus	1	Contrastive pre_focus
2023405	block1	Control	post	fei1	Object	Contrastive	r1	189.9433253	4	1	on_focus	1	Contrastive on_focus
2023405	block1	Control	post	gei1	Object	Contrastive	r1	270.9232697	5	2	on_focus	1	Contrastive on_focus
2023405	block1	Control	post	zoeng1	Subject	Narrow	r1	182.5422336	1	1	on_focus	1	Narrow on_focus
2023405	block1	Control	post	saang1	Subject	Narrow	r1	179.1075109	2	2	on_focus	1	Narrow on_focus
2023405	block1	Control	post	tsa1	Verb	Narrow	r1	161.5047615	3	1	post_focus	1	Narrow post_focus
2023405	block1	Control	post	fei1	Object	Narrow	r1	122.758317	4	1	post_focus	1	Narrow post_focus
2023405	block1	Control	post	gei1	Object	Narrow	r1	292.3814434	5	2	post_focus	1	Narrow post_focus
2023405	block1	Control	post	jyun2	Subject	Contrastive	r1	105.2705043	1	1	pre_focus	2	Contrastive pre_focus
2023405	block1	Control	post	jyun2	Subject	Contrastive	r1	178.6286531	2	2	pre_focus	2	Contrastive pre_focus
2023405	block1	Control	post	mo2	Verb	Contrastive	r1	303.22058	3	1	on_focus	2	Contrastive on_focus
2023405	block1	Control	post	gau2	Object	Contrastive	r1	201.7546441	4	1	post_focus	2	Contrastive post_focus
2023405	block1	Control	post	zai2	Object	Contrastive	r1	420.0172413	5	2	post_focus	2	Contrastive post_focus
2023405	block1	Control	post	sau3	Subject	Narrow	r1	192.3483418	1	1	on_focus	3	Narrow on_focus
2023405	block1	Control	post	sau3	Subject	Narrow	r1	188.0620764	2	2	on_focus	3	Narrow on_focus
2023405	block1	Control	post	sik3	Verb	Narrow	r1	100.9470548	3	1	post_focus	3	Narrow post_focus
2023405	block1	Control	post	baak3	Object	Narrow	r1	120.7056061	4	1	post_focus	3	Narrow post_focus
2023405	block1	Control	post	baak3	Object	Narrow	r1	194.9140041	5	2	post_focus	3	Narrow post_focus
2023405	block1	Control	post	jyun2	Subject	Narrow	r1	263.0268737	1	1	on_focus	2	Narrow on_focus
2023405	block1	Control	post	jyun2	Subject	Narrow	r1	215.9559669	2	2	on_focus	2	Narrow on_focus
2023405	block1	Control	post	mo2	Verb	Narrow	r1	267.3194262	3	1	post_focus	2	Narrow post_focus
2023405	block1	Control	post	gau2	Object	Narrow	r1	233.5199684	4	1	post_focus	2	Narrow post_focus
2023405	block1	Control	post	zai2	Object	Narrow	r1	301.364488	5	2	post_focus	2	Narrow post_focus
2023405	block1	Control	post	jyun2	Subject	Contrastive	r1	228.6895823	1	1	pre_focus	2	Contrastive pre_focus

**2023405**	block1	Control	post	jyun2	Subject	Contrastive	r1	233.4207471	2	2	pre_focus	2	Contrastive pre_focus
**2023405**	block1	Control	post	mo2	Verb	Contrastive	r1	254.477643	3	1	pre_focus	2	Contrastive pre_focus
**2023405**	block1	Control	post	gau2	Object	Contrastive	r1	257.8239493	4	1	on_focus	2	Contrastive on_focus
**2023405**	block1	Control	post	zai2	Object	Contrastive	r1	412.8444835	5	2	on_focus	2	Contrastive on_focus
**2023405**	block1	Control	post	zoeng1	Subject	Contrastive	r1	201.9525216	1	1	pre_focus	1	Contrastive pre_focus
**2023405**	block1	Control	post	saang1	Subject	Contrastive	r1	227.1111073	2	2	pre_focus	1	Contrastive pre_focus
**2023405**	block1	Control	post	tsa1	Verb	Contrastive	r1	195.573378	3	1	on_focus	1	Contrastive on_focus
**2023405**	block1	Control	post	fei1	Object	Contrastive	r1	168.851237	4	1	post_focus	1	Contrastive post_focus
**2023405**	block1	Control	post	gei1	Object	Contrastive	r1	288.3889233	5	2	post_focus	1	Contrastive post_focus
**2023405**	block1	Control	post	zoeng1	Subject	Contrastive	r1	163.0391008	1	1	on_focus	1	Contrastive on_focus
**2023405**	block1	Control	post	saang1	Subject	Contrastive	r1	203.0243663	2	2	on_focus	1	Contrastive on_focus
**2023405**	block1	Control	post	tsa1	Verb	Contrastive	r1	175.0207802	3	1	post_focus	1	Contrastive post_focus
**2023405**	block1	Control	post	fei1	Object	Contrastive	r1	160.0783135	4	1	post_focus	1	Contrastive post_focus
**2023405**	block1	Control	post	gei1	Object	Contrastive	r1	216.091627	5	2	post_focus	1	Contrastive post_focus
**2023405**	block1	Control	post	sau3	Subject	Narrow	r1	175.3857949	1	1	pre_focus	3	Narrow pre_focus
**2023405**	block1	Control	post	sau3	Subject	Narrow	r1	193.6561014	2	2	pre_focus	3	Narrow pre_focus
**2023405**	block1	Control	post	sik3	Verb	Narrow	r1	120.6879085	3	1	pre_focus	3	Narrow pre_focus
**2023405**	block1	Control	post	baak3	Object	Narrow	r1	162.3735928	4	1	on_focus	3	Narrow on_focus
**2023405**	block1	Control	post	baak3	Object	Narrow	r1	169.9750115	5	2	on_focus	3	Narrow on_focus
**2023405**	block1	Control	post	zoeng1	Subject	Narrow	r1	184.4452107	1	1	pre_focus	1	Narrow pre_focus
**2023405**	block1	Control	post	saang1	Subject	Narrow	r1	208.5650891	2	2	pre_focus	1	Narrow pre_focus
**2023405**	block1	Control	post	tsa1	Verb	Narrow	r1	178.0922919	3	1	on_focus	1	Narrow on_focus
**2023405**	block1	Control	post	fei1	Object	Narrow	r1	160.4695089	4	1	post_focus	1	Narrow post_focus
**2023405**	block1	Control	post	gei1	Object	Narrow	r1	148.0025186	5	2	post_focus	1	Narrow post_focus
**2023405**	block1	Control	post	sau3	Subject	Contrastive	r2	158.1044636	1	1	pre_focus	3	Contrastive pre_focus
**2023405**	block1	Control	post	sau3	Subject	Contrastive	r2	205.561157	2	2	pre_focus	3	Contrastive pre_focus
**2023405**	block1	Control	post	sik3	Verb	Contrastive	r2	156.4820399	3	1	on_focus	3	Contrastive on_focus
**2023405**	block1	Control	post	baak3	Object	Contrastive	r2	161.7310764	4	1	post_focus	3	Contrastive post_focus
**2023405**	block1	Control	post	baak3	Object	Contrastive	r2	188.2687765	5	2	post_focus	3	Contrastive post_focus
**2023405**	block1	Control	post	sau3	Subject	Narrow	r2	212.7740877	1	1	on_focus	3	Narrow on_focus
**2023405**	block1	Control	post	sau3	Subject	Narrow	r2	224.3948964	2	2	on_focus	3	Narrow on_focus
**2023405**	block1	Control	post	sik3	Verb	Narrow	r2	102.7107495	3	1	post_focus	3	Narrow post_focus
**2023405**	block1	Control	post	baak3	Object	Narrow	r2	149.6415251	4	1	post_focus	3	Narrow post_focus
**2023405**	block1	Control	post	baak3	Object	Narrow	r2	154.0916616	5	2	post_focus	3	Narrow post_focus
**2023405**	block1	Control	post	zoeng1	Subject	Contrastive	r2	226.8062316	1	1	pre_focus	1	Contrastive pre_focus
**2023405**	block1	Control	post	saang1	Subject	Contrastive	r2	234.5187954	2	2	pre_focus	1	Contrastive pre_focus
**2023405**	block1	Control	post	tsa1	Verb	Contrastive	r2	199.7673541	3	1	pre_focus	1	Contrastive pre_focus
**2023405**	block1	Control	post	fei1	Object	Contrastive	r2	171.0233344	4	1	on_focus	1	Contrastive on_focus
**2023405**	block1	Control	post	gei1	Object	Contrastive	r2	284.9631881	5	2	on_focus	1	Contrastive on_focus
**2023405**	block1	Control	post	jyun2	Subject	Narrow	r2	261.3934983	1	1	pre_focus	2	Narrow pre_focus
**2023405**	block1	Control	post	jyun2	Subject	Narrow	r2	139.9023276	2	2	pre_focus	2	Narrow pre_focus
**2023405**	block1	Control	post	mo2	Verb	Narrow	r2	257.4808076	3	1	on_focus	2	Narrow on_focus
**2023405**	block1	Control	post	gau2	Object	Narrow	r2	199.8895238	4	1	post_focus	2	Narrow post_focus
**2023405**	block1	Control	post	zai2	Object	Narrow	r2	364.2332786	5	2	post_focus	2	Narrow post_focus
**2023405**	block1	Control	post	zoeng1	Subject	Narrow	r2	196.1890811	1	1	on_focus	1	Narrow on_focus
**2023405**	block1	Control	post	saang1	Subject	Narrow	r2	237.4706711	2	2	on_focus	1	Narrow on_focus
**2023405**	block1	Control	post	tsa1	Verb	Narrow	r2	187.8762645	3	1	post_focus	1	Narrow post_focus
**2023405**	block1	Control	post	fei1	Object	Narrow	r2	176.2682869	4	1	post_focus	1	Narrow post_focus
**2023405**	block1	Control	post	gei1	Object	Narrow	r2	333.643595	5	2	post_focus	1	Narrow post_focus
**2023405**	block1	Control	post	zoeng1	Subject	Contrastive	r2	203.1282863	1	1	pre_focus	1	Contrastive pre_focus
**2023405**	block1	Control	post	saang1	Subject	Contrastive	r2	194.5739675	2	2	pre_focus	1	Contrastive pre_focus
**2023405**	block1	Control	post	tsa1	Verb	Contrastive	r2	178.6966266	3	1	on_focus	1	Contrastive on_focus
**2023405**	block1	Control	post	fei1	Object	Contrastive	r2	190.9408613	4	1	post_focus	1	Contrastive post_focus
**2023405**	block1	Control	post	gei1	Object	Contrastive	r2	419.0998308	5	2	post_focus	1	Contrastive post_focus
**2023405**	block1	Control	post	zoeng1	Subject	Narrow	r2	224.8043471	1	1	pre_focus	1	Narrow pre_focus
**2023405**	block1	Control	post	saang1	Subject	Narrow	r2	222.7428268	2	2	pre_focus	1	Narrow pre_focus
**2023405**	block1	Control	post	tsa1	Verb	Narrow	r2	168.3747578	3	1	on_focus	1	Narrow on_focus
**2023405**	block1	Control	post	fei1	Object	Narrow	r2	175.32368	4	1	post_focus	1	Narrow post_focus
**2023405**	block1	Control	post	gei1	Object	Narrow	r2	353.9526598	5	2	post_focus	1	Narrow post_focus
**2023405**	block1	Control	post	jyun2	Subject	Contrastive	r2	261.7353239	1	1	pre_focus	2	Contrastive pre_focus
**2023405**	block1	Control	post	jyun2	Subject	Contrastive	r2	213.7428264	2	2	pre_focus	2	Contrastive pre_focus
**2023405**	block1	Control	post	mo2	Verb	Contrastive	r2	232.2064007	3	1	on_focus	2	Contrastive on_focus
**2023405**	block1	Control	post	gau2	Object	Contrastive	r2	205.9275516	4	1	post_focus	2	Contrastive post_focus
**2023405**	block1	Control	post	zai2	Object	Contrastive	r2	351.4018577	5	2	post_focus	2	Contrastive post_focus
**2023405**	block1	Control	post	jyun2	Subject	Contrastive	r2	161.9158518	1	1	pre_focus	2	Contrastive pre_focus
**2023405**	block1	Control	post	jyun2	Subject	Contrastive	r2	224.3657732	2	2	pre_focus	2	Contrastive pre_focus

2023405	block1	Control	post	mo2	Verb	Contrastive	r2	267.3513552	3	1	pre_focus	2	Contrastive pre_focus
2023405	block1	Control	post	gau2	Object	Contrastive	r2	197.9634451	4	1	on_focus	2	Contrastive on_focus
2023405	block1	Control	post	zai2	Object	Contrastive	r2	364.7977554	5	2	on_focus	2	Contrastive on_focus
2023405	block1	Control	post	jyun2	Subject	Contrastive	r2	255.1241625	1	1	on_focus	2	Contrastive on_focus
2023405	block1	Control	post	jyun2	Subject	Contrastive	r2	224.7430202	2	2	on_focus	2	Contrastive on_focus
2023405	block1	Control	post	mo2	Verb	Contrastive	r2	209.2273164	3	1	post_focus	2	Contrastive post_focus
2023405	block1	Control	post	gau2	Object	Contrastive	r2	206.1644855	4	1	post_focus	2	Contrastive post_focus
2023405	block1	Control	post	zai2	Object	Contrastive	r2	319.4994183	5	2	post_focus	2	Contrastive post_focus
2023405	block1	Control	post	zoeng1	Subject	Broad	r2	185.5038085	1	1	broad_focus	1	Broad focus
2023405	block1	Control	post	saang1	Subject	Broad	r2	231.0647626	2	2	broad_focus	1	Broad focus
2023405	block1	Control	post	tsa1	Verb	Broad	r2	162.1806941	3	1	broad_focus	1	Broad focus
2023405	block1	Control	post	fei1	Object	Broad	r2	172.5830791	4	1	broad_focus	1	Broad focus
2023405	block1	Control	post	gei1	Object	Broad	r2	398.5634837	5	2	broad_focus	1	Broad focus
2023405	block1	Control	post	jyun2	Subject	Narrow	r2	258.1980942	1	1	on_focus	2	Narrow on_focus
2023405	block1	Control	post	jyun2	Subject	Narrow	r2	201.8263027	2	2	on_focus	2	Narrow on_focus
2023405	block1	Control	post	mo2	Verb	Narrow	r2	219.0242645	3	1	post_focus	2	Narrow post_focus
2023405	block1	Control	post	gau2	Object	Narrow	r2	216.3246005	4	1	post_focus	2	Narrow post_focus
2023405	block1	Control	post	zai2	Object	Narrow	r2	245.0035001	5	2	post_focus	2	Narrow post_focus
2023405	block1	Control	post	jyun2	Subject	Narrow	r2	263.7235313	1	1	pre_focus	2	Narrow pre_focus
2023405	block1	Control	post	jyun2	Subject	Narrow	r2	219.7919506	2	2	pre_focus	2	Narrow pre_focus
2023405	block1	Control	post	mo2	Verb	Narrow	r2	246.0738569	3	1	pre_focus	2	Narrow pre_focus
2023405	block1	Control	post	gau2	Object	Narrow	r2	183.2184498	4	1	on_focus	2	Narrow on_focus
2023405	block1	Control	post	zai2	Object	Narrow	r2	346.5899249	5	2	on_focus	2	Narrow on_focus
2023405	block1	Control	post	zoeng1	Subject	Contrastive	r2	228.6762719	1	1	on_focus	1	Contrastive on_focus
2023405	block1	Control	post	saang1	Subject	Contrastive	r2	240.9059134	2	2	on_focus	1	Contrastive on_focus
2023405	block1	Control	post	tsa1	Verb	Contrastive	r2	251.1974977	3	1	post_focus	1	Contrastive post_focus
2023405	block1	Control	post	fei1	Object	Contrastive	r2	281.0957024	4	1	post_focus	1	Contrastive post_focus
2023405	block1	Control	post	gei1	Object	Contrastive	r2	795.9691229	5	2	post_focus	1	Contrastive post_focus
2023405	block1	Control	post	sau3	Subject	Contrastive	r2	152.285742	1	1	pre_focus	3	Contrastive pre_focus
2023405	block1	Control	post	sau3	Subject	Contrastive	r2	162.298635	2	2	pre_focus	3	Contrastive pre_focus
2023405	block1	Control	post	sik3	Verb	Contrastive	r2	117.5306476	3	1	pre_focus	3	Contrastive pre_focus
2023405	block1	Control	post	baak3	Object	Contrastive	r2	139.5426699	4	1	on_focus	3	Contrastive on_focus
2023405	block1	Control	post	baak3	Object	Contrastive	r2	212.661844	5	2	on_focus	3	Contrastive on_focus
2023405	block1	Control	post	sau3	Subject	Narrow	r2	151.2041483	1	1	pre_focus	3	Narrow pre_focus
2023405	block1	Control	post	sau3	Subject	Narrow	r2	177.2854968	2	2	pre_focus	3	Narrow pre_focus
2023405	block1	Control	post	sik3	Verb	Narrow	r2	96.9288771	3	1	on_focus	3	Narrow on_focus
2023405	block1	Control	post	baak3	Object	Narrow	r2	117.5134487	4	1	post_focus	3	Narrow post_focus
2023405	block1	Control	post	baak3	Object	Narrow	r2	188.011832	5	2	post_focus	3	Narrow post_focus
2023405	block1	Control	post	sau3	Subject	Broad	r2	150.1918612	1	1	broad_focus	3	Broad focus
2023405	block1	Control	post	sau3	Subject	Broad	r2	174.7414669	2	2	broad_focus	3	Broad focus
2023405	block1	Control	post	sik3	Verb	Broad	r2	125.8268477	3	1	broad_focus	3	Broad focus
2023405	block1	Control	post	baak3	Object	Broad	r2	124.2859745	4	1	broad_focus	3	Broad focus
2023405	block1	Control	post	baak3	Object	Broad	r2	179.3100589	5	2	broad_focus	3	Broad focus
2023405	block1	Control	post	sau3	Subject	Narrow	r2	144.1284643	1	1	pre_focus	3	Narrow pre_focus
2023405	block1	Control	post	sau3	Subject	Narrow	r2	161.8516846	2	2	pre_focus	3	Narrow pre_focus
2023405	block1	Control	post	sik3	Verb	Narrow	r2	107.6595378	3	1	pre_focus	3	Narrow pre_focus
2023405	block1	Control	post	baak3	Object	Narrow	r2	130.535409	4	1	on_focus	3	Narrow on_focus
2023405	block1	Control	post	baak3	Object	Narrow	r2	198.5458678	5	2	on_focus	3	Narrow on_focus
2023405	block1	Control	post	jyun2	Subject	Broad	r2	242.7281854	1	1	broad_focus	2	Broad focus
2023405	block1	Control	post	jyun2	Subject	Broad	r2	210.2128275	2	2	broad_focus	2	Broad focus
2023405	block1	Control	post	mo2	Verb	Broad	r2	253.4969208	3	1	broad_focus	2	Broad focus
2023405	block1	Control	post	gau2	Object	Broad	r2	205.321549	4	1	broad_focus	2	Broad focus
2023405	block1	Control	post	zai2	Object	Broad	r2	409.2445263	5	2	broad_focus	2	Broad focus
2023405	block1	Control	post	zoeng1	Subject	Narrow	r2	181.2724112	1	1	pre_focus	1	Narrow pre_focus
2023405	block1	Control	post	saang1	Subject	Narrow	r2	207.5770715	2	2	pre_focus	1	Narrow pre_focus
2023405	block1	Control	post	tsa1	Verb	Narrow	r2	168.3639526	3	1	pre_focus	1	Narrow pre_focus
2023405	block1	Control	post	fei1	Object	Narrow	r2	208.8435	4	1	on_focus	1	Narrow on_focus
2023405	block1	Control	post	gei1	Object	Narrow	r2	317.488567	5	2	on_focus	1	Narrow on_focus
2023405	block1	Control	post	sau3	Subject	Contrastive	r2	182.8105344	1	1	on_focus	3	Contrastive on_focus
2023405	block1	Control	post	sau3	Subject	Contrastive	r2	180.3521172	2	2	on_focus	3	Contrastive on_focus
2023405	block1	Control	post	sik3	Verb	Contrastive	r2	132.4493251	3	1	post_focus	3	Contrastive post_focus
2023405	block1	Control	post	baak3	Object	Contrastive	r2	131.7266362	4	1	post_focus	3	Contrastive post_focus
2023405	block1	Control	post	baak3	Object	Contrastive	r2	253.2536904	5	2	post_focus	3	Contrastive post_focus
2023405	block1	Control	pre	jyun2	Subject	Broad	r1	330.9704031	1	1	broad_focus	2	Broad focus
2023405	block1	Control	pre	jyun2	Subject	Broad	r1	245.5141184	2	2	broad_focus	2	Broad focus
2023405	block1	Control	pre	mo2	Verb	Broad	r1	224.5787428	3	1	broad_focus	2	Broad focus

**2023405**	block1	Control	pre	gau2	Object	Broad	r1	188.8031185	4	1	broad_focus	2	Broad focus
**2023405**	block1	Control	pre	zai2	Object	Broad	r1	261.1968169	5	2	broad_focus	2	Broad focus
**2023405**	block1	Control	pre	zoeng1	Subject	Contrastive	r1	260.8444489	1	1	on_focus	1	Contrastive on_focus
**2023405**	block1	Control	pre	saang1	Subject	Contrastive	r1	291.9190512	2	2	on_focus	1	Contrastive on_focus
**2023405**	block1	Control	pre	tsa1	Verb	Contrastive	r1	259.987871	3	1	post_focus	1	Contrastive post_focus
**2023405**	block1	Control	pre	fei1	Object	Contrastive	r1	184.0225259	4	1	post_focus	1	Contrastive post_focus
**2023405**	block1	Control	pre	gei1	Object	Contrastive	r1	301.1813477	5	2	post_focus	1	Contrastive post_focus
**2023405**	block1	Control	pre	sau3	Subject	Contrastive	r1	245.7717176	1	1	pre_focus	3	Contrastive pre_focus
**2023405**	block1	Control	pre	sau3	Subject	Contrastive	r1	208.1912625	2	2	pre_focus	3	Contrastive pre_focus
**2023405**	block1	Control	pre	sik3	Verb	Contrastive	r1	149.9517691	3	1	pre_focus	3	Contrastive pre_focus
**2023405**	block1	Control	pre	baak3	Object	Contrastive	r1	152.4049202	4	1	on_focus	3	Contrastive on_focus
**2023405**	block1	Control	pre	baak3	Object	Contrastive	r1	284.8953497	5	2	on_focus	3	Contrastive on_focus
**2023405**	block1	Control	pre	jyun2	Subject	Narrow	r1	426.7800454	1	1	pre_focus	2	Narrow pre_focus
**2023405**	block1	Control	pre	jyun2	Subject	Narrow	r1	293.7958402	2	2	pre_focus	2	Narrow pre_focus
**2023405**	block1	Control	pre	mo2	Verb	Narrow	r1	408.2784689	3	1	on_focus	2	Narrow on_focus
**2023405**	block1	Control	pre	gau2	Object	Narrow	r1	268.9436868	4	1	post_focus	2	Narrow post_focus
**2023405**	block1	Control	pre	zai2	Object	Narrow	r1	382.9862181	5	2	post_focus	2	Narrow post_focus
**2023405**	block1	Control	pre	sau3	Subject	Narrow	r1	234.996179	1	1	on_focus	3	Narrow on_focus
**2023405**	block1	Control	pre	sau3	Subject	Narrow	r1	217.653582	2	2	on_focus	3	Narrow on_focus
**2023405**	block1	Control	pre	sik3	Verb	Narrow	r1	128.637323	3	1	post_focus	3	Narrow post_focus
**2023405**	block1	Control	pre	baak3	Object	Narrow	r1	170.1509607	4	1	post_focus	3	Narrow post_focus
**2023405**	block1	Control	pre	baak3	Object	Narrow	r1	246.3489901	5	2	post_focus	3	Narrow post_focus
**2023405**	block1	Control	pre	jyun2	Subject	Narrow	r1	326.920738	1	1	pre_focus	2	Narrow pre_focus
**2023405**	block1	Control	pre	jyun2	Subject	Narrow	r1	284.1988429	2	2	pre_focus	2	Narrow pre_focus
**2023405**	block1	Control	pre	mo2	Verb	Narrow	r1	304.8567401	3	1	pre_focus	2	Narrow pre_focus
**2023405**	block1	Control	pre	gau2	Object	Narrow	r1	301.4207454	4	1	on_focus	2	Narrow on_focus
**2023405**	block1	Control	pre	zai2	Object	Narrow	r1	381.2334148	5	2	on_focus	2	Narrow on_focus
**2023405**	block1	Control	pre	jyun2	Subject	Narrow	r1	417.023374	1	1	on_focus	2	Narrow on_focus
**2023405**	block1	Control	pre	jyun2	Subject	Narrow	r1	302.2594928	2	2	on_focus	2	Narrow on_focus
**2023405**	block1	Control	pre	mo2	Verb	Narrow	r1	368.5823571	3	1	post_focus	2	Narrow post_focus
**2023405**	block1	Control	pre	gau2	Object	Narrow	r1	277.3647699	4	1	post_focus	2	Narrow post_focus
**2023405**	block1	Control	pre	zai2	Object	Narrow	r1	335.2091086	5	2	post_focus	2	Narrow post_focus
**2023405**	block1	Control	pre	zoeng1	Subject	Narrow	r1	189.25125	1	1	pre_focus	1	Narrow pre_focus
**2023405**	block1	Control	pre	saang1	Subject	Narrow	r1	267.9516123	2	2	pre_focus	1	Narrow pre_focus
**2023405**	block1	Control	pre	tsa1	Verb	Narrow	r1	242.9725506	3	1	pre_focus	1	Narrow pre_focus
**2023405**	block1	Control	pre	fei1	Object	Narrow	r1	290.9908906	4	1	on_focus	1	Narrow on_focus
**2023405**	block1	Control	pre	gei1	Object	Narrow	r1	312.3858746	5	2	on_focus	1	Narrow on_focus
**2023405**	block1	Control	pre	jyun2	Subject	Contrastive	r1	393.194565	1	1	on_focus	2	Contrastive on_focus
**2023405**	block1	Control	pre	jyun2	Subject	Contrastive	r1	341.9489711	2	2	on_focus	2	Contrastive on_focus
**2023405**	block1	Control	pre	mo2	Verb	Contrastive	r1	351.9191607	3	1	post_focus	2	Contrastive post_focus
**2023405**	block1	Control	pre	gau2	Object	Contrastive	r1	305.6160909	4	1	post_focus	2	Contrastive post_focus
**2023405**	block1	Control	pre	zai2	Object	Contrastive	r1	329.1690879	5	2	post_focus	2	Contrastive post_focus
**2023405**	block1	Control	pre	zoeng1	Subject	Contrastive	r1	249.5921174	1	1	pre_focus	1	Contrastive pre_focus
**2023405**	block1	Control	pre	saang1	Subject	Contrastive	r1	305.4423724	2	2	pre_focus	1	Contrastive pre_focus
**2023405**	block1	Control	pre	tsa1	Verb	Contrastive	r1	252.6482512	3	1	pre_focus	1	Contrastive pre_focus
**2023405**	block1	Control	pre	fei1	Object	Contrastive	r1	312.4849177	4	1	on_focus	1	Contrastive on_focus
**2023405**	block1	Control	pre	gei1	Object	Contrastive	r1	357.1077339	5	2	on_focus	1	Contrastive on_focus
**2023405**	block1	Control	pre	zoeng1	Subject	Broad	r1	395.6657165	1	1	broad_focus	1	Broad focus
**2023405**	block1	Control	pre	saang1	Subject	Broad	r1	358.0389296	2	2	broad_focus	1	Broad focus
**2023405**	block1	Control	pre	tsa1	Verb	Broad	r1	329.5144963	3	1	broad_focus	1	Broad focus
**2023405**	block1	Control	pre	fei1	Object	Broad	r1	423.7839097	4	1	broad_focus	1	Broad focus
**2023405**	block1	Control	pre	gei1	Object	Broad	r1	470.3145532	5	2	broad_focus	1	Broad focus
**2023405**	block1	Control	pre	sau3	Subject	Contrastive	r1	168.4306364	1	1	pre_focus	3	Contrastive pre_focus
**2023405**	block1	Control	pre	sau3	Subject	Contrastive	r1	189.0258539	2	2	pre_focus	3	Contrastive pre_focus
**2023405**	block1	Control	pre	sik3	Verb	Contrastive	r1	387.6279441	3	1	on_focus	3	Contrastive on_focus
**2023405**	block1	Control	pre	baak3	Object	Contrastive	r1	164.7491057	4	1	post_focus	3	Contrastive post_focus
**2023405**	block1	Control	pre	baak3	Object	Contrastive	r1	683.0741202	5	2	post_focus	3	Contrastive post_focus
**2023405**	block1	Control	pre	sau3	Subject	Narrow	r1	203.2732213	1	1	pre_focus	3	Narrow pre_focus
**2023405**	block1	Control	pre	sau3	Subject	Narrow	r1	195.961166	2	2	pre_focus	3	Narrow pre_focus
**2023405**	block1	Control	pre	sik3	Verb	Narrow	r1	113.6385016	3	1	pre_focus	3	Narrow pre_focus
**2023405**	block1	Control	pre	baak3	Object	Narrow	r1	162.2806542	4	1	on_focus	3	Narrow on_focus
**2023405**	block1	Control	pre	baak3	Object	Narrow	r1	235.8326615	5	2	on_focus	3	Narrow on_focus
**2023405**	block1	Control	pre	jyun2	Subject	Contrastive	r1	334.6161912	1	1	pre_focus	2	Contrastive pre_focus
**2023405**	block1	Control	pre	jyun2	Subject	Contrastive	r1	232.6814023	2	2	pre_focus	2	Contrastive pre_focus
**2023405**	block1	Control	pre	mo2	Verb	Contrastive	r1	288.9896853	3	1	pre_focus	2	Contrastive pre_focus
**2023405**	block1	Control	pre	gau2	Object	Contrastive	r1	265.5746958	4	1	on_focus	2	Contrastive on_focus

2023405	block1	Control	pre	zai2	Object	Contrastive	r1	326.5616839	5	2	on_focus	2	Contrastive on_focus
2023405	block1	Control	pre	zoeng1	Subject	Narrow	r1	223.2742271	1	1	pre_focus	1	Narrow pre_focus
2023405	block1	Control	pre	saang1	Subject	Narrow	r1	238.737154	2	2	pre_focus	1	Narrow pre_focus
2023405	block1	Control	pre	tsa1	Verb	Narrow	r1	239.3770171	3	1	on_focus	1	Narrow on_focus
2023405	block1	Control	pre	fei1	Object	Narrow	r1	263.3872199	4	1	post_focus	1	Narrow post_focus
2023405	block1	Control	pre	gei1	Object	Narrow	r1	313.5458981	5	2	post_focus	1	Narrow post_focus
2023405	block1	Control	pre	zoeng1	Subject	Contrastive	r1	232.4426876	1	1	pre_focus	1	Contrastive pre_focus
2023405	block1	Control	pre	saang1	Subject	Contrastive	r1	289.3390968	2	2	pre_focus	1	Contrastive pre_focus
2023405	block1	Control	pre	tsa1	Verb	Contrastive	r1	268.7646582	3	1	on_focus	1	Contrastive on_focus
2023405	block1	Control	pre	fei1	Object	Contrastive	r1	194.54867	4	1	post_focus	1	Contrastive post_focus
2023405	block1	Control	pre	gei1	Object	Contrastive	r1	344.4524109	5	2	post_focus	1	Contrastive post_focus
2023405	block1	Control	pre	sau3	Subject	Contrastive	r1	216.3802188	1	1	on_focus	3	Contrastive on_focus
2023405	block1	Control	pre	sau3	Subject	Contrastive	r1	263.9341601	2	2	on_focus	3	Contrastive on_focus
2023405	block1	Control	pre	sik3	Verb	Contrastive	r1	139.9897612	3	1	post_focus	3	Contrastive post_focus
2023405	block1	Control	pre	baak3	Object	Contrastive	r1	147.7276204	4	1	post_focus	3	Contrastive post_focus
2023405	block1	Control	pre	baak3	Object	Contrastive	r1	245.7522435	5	2	post_focus	3	Contrastive post_focus
2023405	block1	Control	pre	zoeng1	Subject	Narrow	r1	283.1560449	1	1	on_focus	1	Narrow on_focus
2023405	block1	Control	pre	saang1	Subject	Narrow	r1	308.04635	2	2	on_focus	1	Narrow on_focus
2023405	block1	Control	pre	tsa1	Verb	Narrow	r1	239.4521794	3	1	post_focus	1	Narrow post_focus
2023405	block1	Control	pre	fei1	Object	Narrow	r1	254.5806034	4	1	post_focus	1	Narrow post_focus
2023405	block1	Control	pre	gei1	Object	Narrow	r1	294.3931452	5	2	post_focus	1	Narrow post_focus
2023405	block1	Control	pre	sau3	Subject	Narrow	r1	216.0102939	1	1	pre_focus	3	Narrow pre_focus
2023405	block1	Control	pre	sau3	Subject	Narrow	r1	187.8164955	2	2	pre_focus	3	Narrow pre_focus
2023405	block1	Control	pre	sik3	Verb	Narrow	r1	115.6560363	3	1	on_focus	3	Narrow on_focus
2023405	block1	Control	pre	baak3	Object	Narrow	r1	150.7603192	4	1	post_focus	3	Narrow post_focus
2023405	block1	Control	pre	baak3	Object	Narrow	r1	224.204565	5	2	post_focus	3	Narrow post_focus
2023405	block1	Control	pre	jyun2	Subject	Contrastive	r1	363.8508883	1	1	pre_focus	2	Contrastive pre_focus
2023405	block1	Control	pre	jyun2	Subject	Contrastive	r1	296.1340837	2	2	pre_focus	2	Contrastive pre_focus
2023405	block1	Control	pre	mo2	Verb	Contrastive	r1	439.2101215	3	1	on_focus	2	Contrastive on_focus
2023405	block1	Control	pre	gau2	Object	Contrastive	r1	267.8220154	4	1	post_focus	2	Contrastive post_focus
2023405	block1	Control	pre	zai2	Object	Contrastive	r1	327.0931886	5	2	post_focus	2	Contrastive post_focus
2023405	block1	Control	pre	sau3	Subject	Broad	r1	267.5551737	1	1	broad_focus	3	Broad focus
2023405	block1	Control	pre	sau3	Subject	Broad	r1	230.6876528	2	2	broad_focus	3	Broad focus
2023405	block1	Control	pre	sik3	Verb	Broad	r1	136.8991622	3	1	broad_focus	3	Broad focus
2023405	block1	Control	pre	baak3	Object	Broad	r1	157.2414377	4	1	broad_focus	3	Broad focus
2023405	block1	Control	pre	baak3	Object	Broad	r1	281.7525191	5	2	broad_focus	3	Broad focus
2023405	block1	Control	pre	sau3	Subject	Contrastive	r2	296.8751895	1	1	on_focus	3	Contrastive on_focus
2023405	block1	Control	pre	sau3	Subject	Contrastive	r2	301.4080089	2	2	on_focus	3	Contrastive on_focus
2023405	block1	Control	pre	sik3	Verb	Contrastive	r2	186.3636769	3	1	post_focus	3	Contrastive post_focus
2023405	block1	Control	pre	baak3	Object	Contrastive	r2	248.6566623	4	1	post_focus	3	Contrastive post_focus
2023405	block1	Control	pre	baak3	Object	Contrastive	r2	409.7039891	5	2	post_focus	3	Contrastive post_focus
2023405	block1	Control	pre	zoeng1	Subject	Narrow	r2	310.904021	1	1	pre_focus	1	Narrow pre_focus
2023405	block1	Control	pre	saang1	Subject	Narrow	r2	342.0398225	2	2	pre_focus	1	Narrow pre_focus
2023405	block1	Control	pre	tsa1	Verb	Narrow	r2	281.6123883	3	1	pre_focus	1	Narrow pre_focus
2023405	block1	Control	pre	fei1	Object	Narrow	r2	237.5506404	4	1	on_focus	1	Narrow on_focus
2023405	block1	Control	pre	gei1	Object	Narrow	r2	490.7370807	5	2	on_focus	1	Narrow on_focus
2023405	block1	Control	pre	zoeng1	Subject	Narrow	r2	348.9761343	1	1	pre_focus	1	Narrow pre_focus
2023405	block1	Control	pre	saang1	Subject	Narrow	r2	253.1664638	2	2	pre_focus	1	Narrow pre_focus
2023405	block1	Control	pre	tsa1	Verb	Narrow	r2	293.472727	3	1	on_focus	1	Narrow on_focus
2023405	block1	Control	pre	fei1	Object	Narrow	r2	219.115637	4	1	post_focus	1	Narrow post_focus
2023405	block1	Control	pre	gei1	Object	Narrow	r2	583.8969029	5	2	post_focus	1	Narrow post_focus
2023405	block1	Control	pre	zoeng1	Subject	Contrastive	r2	304.8607144	1	1	pre_focus	1	Contrastive pre_focus
2023405	block1	Control	pre	saang1	Subject	Contrastive	r2	302.9087762	2	2	pre_focus	1	Contrastive pre_focus
2023405	block1	Control	pre	tsa1	Verb	Contrastive	r2	280.5545462	3	1	on_focus	1	Contrastive on_focus
2023405	block1	Control	pre	fei1	Object	Contrastive	r2	164.0658622	4	1	post_focus	1	Contrastive post_focus
2023405	block1	Control	pre	gei1	Object	Contrastive	r2	674.5998691	5	2	post_focus	1	Contrastive post_focus
2023405	block1	Control	pre	sau3	Subject	Narrow	r2	218.8923255	1	1	pre_focus	3	Narrow pre_focus
2023405	block1	Control	pre	sau3	Subject	Narrow	r2	215.6957925	2	2	pre_focus	3	Narrow pre_focus
2023405	block1	Control	pre	sik3	Verb	Narrow	r2	161.2006684	3	1	pre_focus	3	Narrow pre_focus
2023405	block1	Control	pre	baak3	Object	Narrow	r2	178.4573357	4	1	on_focus	3	Narrow on_focus
2023405	block1	Control	pre	baak3	Object	Narrow	r2	347.1234283	5	2	on_focus	3	Narrow on_focus
2023405	block1	Control	pre	sau3	Subject	Narrow	r2	216.4816599	1	1	on_focus	3	Narrow on_focus
2023405	block1	Control	pre	sau3	Subject	Narrow	r2	139.9838454	2	2	on_focus	3	Narrow on_focus
2023405	block1	Control	pre	sik3	Verb	Narrow	r2	135.2490622	3	1	post_focus	3	Narrow post_focus
2023405	block1	Control	pre	baak3	Object	Narrow	r2	117.8305493	4	1	post_focus	3	Narrow post_focus
2023405	block1	Control	pre	baak3	Object	Narrow	r2	302.7991639	5	2	post_focus	3	Narrow post_focus

**2023405**	block1	Control	pre	zoeng1	Subject	Contrastive	r2	293.210854	1	1	on_focus	1	Contrastive on_focus
**2023405**	block1	Control	pre	saang1	Subject	Contrastive	r2	313.2086963	2	2	on_focus	1	Contrastive on_focus
**2023405**	block1	Control	pre	tsa1	Verb	Contrastive	r2	282.1676959	3	1	post_focus	1	Contrastive post_focus
**2023405**	block1	Control	pre	fei1	Object	Contrastive	r2	261.5611385	4	1	post_focus	1	Contrastive post_focus
**2023405**	block1	Control	pre	gei1	Object	Contrastive	r2	592.4585459	5	2	post_focus	1	Contrastive post_focus
**2023405**	block1	Control	pre	jyun2	Subject	Narrow	r2	325.8506234	1	1	pre_focus	2	Narrow pre_focus
**2023405**	block1	Control	pre	jyun2	Subject	Narrow	r2	292.6989513	2	2	pre_focus	2	Narrow pre_focus
**2023405**	block1	Control	pre	mo2	Verb	Narrow	r2	373.7947659	3	1	on_focus	2	Narrow on_focus
**2023405**	block1	Control	pre	gau2	Object	Narrow	r2	231.4456891	4	1	post_focus	2	Narrow post_focus
**2023405**	block1	Control	pre	zai2	Object	Narrow	r2	880.2298352	5	2	post_focus	2	Narrow post_focus
**2023405**	block1	Control	pre	jyun2	Subject	Broad	r2	345.5734371	1	1	broad_focus	2	Broad focus
**2023405**	block1	Control	pre	jyun2	Subject	Broad	r2	248.5528383	2	2	broad_focus	2	Broad focus
**2023405**	block1	Control	pre	mo2	Verb	Broad	r2	279.0179502	3	1	broad_focus	2	Broad focus
**2023405**	block1	Control	pre	gau2	Object	Broad	r2	255.4261679	4	1	broad_focus	2	Broad focus
**2023405**	block1	Control	pre	zai2	Object	Broad	r2	407.5346022	5	2	broad_focus	2	Broad focus
**2023405**	block1	Control	pre	sau3	Subject	Contrastive	r2	222.1528329	1	1	pre_focus	3	Contrastive pre_focus
**2023405**	block1	Control	pre	sau3	Subject	Contrastive	r2	159.7946619	2	2	pre_focus	3	Contrastive pre_focus
**2023405**	block1	Control	pre	sik3	Verb	Contrastive	r2	135.4211945	3	1	pre_focus	3	Contrastive pre_focus
**2023405**	block1	Control	pre	baak3	Object	Contrastive	r2	117.44558	4	1	on_focus	3	Contrastive on_focus
**2023405**	block1	Control	pre	baak3	Object	Contrastive	r2	341.9550219	5	2	on_focus	3	Contrastive on_focus
**2023405**	block1	Control	pre	jyun2	Subject	Narrow	r2	286.2112623	1	1	on_focus	2	Narrow on_focus
**2023405**	block1	Control	pre	jyun2	Subject	Narrow	r2	173.2542242	2	2	on_focus	2	Narrow on_focus
**2023405**	block1	Control	pre	mo2	Verb	Narrow	r2	218.6079975	3	1	post_focus	2	Narrow post_focus
**2023405**	block1	Control	pre	gau2	Object	Narrow	r2	157.2769108	4	1	post_focus	2	Narrow post_focus
**2023405**	block1	Control	pre	zai2	Object	Narrow	r2	388.5340584	5	2	post_focus	2	Narrow post_focus
**2023405**	block1	Control	pre	sau3	Subject	Narrow	r2	179.8083932	1	1	pre_focus	3	Narrow pre_focus
**2023405**	block1	Control	pre	sau3	Subject	Narrow	r2	169.0527231	2	2	pre_focus	3	Narrow pre_focus
**2023405**	block1	Control	pre	sik3	Verb	Narrow	r2	114.4529415	3	1	on_focus	3	Narrow on_focus
**2023405**	block1	Control	pre	baak3	Object	Narrow	r2	144.8918322	4	1	post_focus	3	Narrow post_focus
**2023405**	block1	Control	pre	baak3	Object	Narrow	r2	228.9661045	5	2	post_focus	3	Narrow post_focus
**2023405**	block1	Control	pre	jyun2	Subject	Contrastive	r2	305.6216931	1	1	on_focus	2	Contrastive on_focus
**2023405**	block1	Control	pre	jyun2	Subject	Contrastive	r2	211.2513528	2	2	on_focus	2	Contrastive on_focus
**2023405**	block1	Control	pre	mo2	Verb	Contrastive	r2	226.8273405	3	1	post_focus	2	Contrastive post_focus
**2023405**	block1	Control	pre	gau2	Object	Contrastive	r2	211.502174	4	1	post_focus	2	Contrastive post_focus
**2023405**	block1	Control	pre	zai2	Object	Contrastive	r2	284.9851008	5	2	post_focus	2	Contrastive post_focus
**2023405**	block1	Control	pre	zoeng1	Subject	Contrastive	r2	267.9157566	1	1	pre_focus	1	Contrastive pre_focus
**2023405**	block1	Control	pre	saang1	Subject	Contrastive	r2	230.6623722	2	2	pre_focus	1	Contrastive pre_focus
**2023405**	block1	Control	pre	tsa1	Verb	Contrastive	r2	245.7659091	3	1	pre_focus	1	Contrastive pre_focus
**2023405**	block1	Control	pre	fei1	Object	Contrastive	r2	216.3144369	4	1	on_focus	1	Contrastive on_focus
**2023405**	block1	Control	pre	gei1	Object	Contrastive	r2	410.2878668	5	2	on_focus	1	Contrastive on_focus
**2023405**	block1	Control	pre	jyun2	Subject	Contrastive	r2	280.2492616	1	1	pre_focus	2	Contrastive pre_focus
**2023405**	block1	Control	pre	jyun2	Subject	Contrastive	r2	278.867104	2	2	pre_focus	2	Contrastive pre_focus
**2023405**	block1	Control	pre	mo2	Verb	Contrastive	r2	546.8182158	3	1	on_focus	2	Contrastive on_focus
**2023405**	block1	Control	pre	gau2	Object	Contrastive	r2	229.0381208	4	1	post_focus	2	Contrastive post_focus
**2023405**	block1	Control	pre	zai2	Object	Contrastive	r2	1034.379201	5	2	post_focus	2	Contrastive post_focus
**2023405**	block1	Control	pre	jyun2	Subject	Narrow	r2	465.675737	1	1	pre_focus	2	Narrow pre_focus
**2023405**	block1	Control	pre	jyun2	Subject	Narrow	r2	298.9119522	2	2	pre_focus	2	Narrow pre_focus
**2023405**	block1	Control	pre	mo2	Verb	Narrow	r2	602.8896645	3	1	pre_focus	2	Narrow pre_focus
**2023405**	block1	Control	pre	gau2	Object	Narrow	r2	468.1215962	4	1	on_focus	2	Narrow on_focus
**2023405**	block1	Control	pre	zai2	Object	Narrow	r2	501.5232684	5	2	on_focus	2	Narrow on_focus
**2023405**	block1	Control	pre	sau3	Subject	Broad	r2	251.2107414	1	1	broad_focus	3	Broad focus
**2023405**	block1	Control	pre	sau3	Subject	Broad	r2	193.3738325	2	2	broad_focus	3	Broad focus
**2023405**	block1	Control	pre	sik3	Verb	Broad	r2	163.750306	3	1	broad_focus	3	Broad focus
**2023405**	block1	Control	pre	baak3	Object	Broad	r2	152.3610164	4	1	broad_focus	3	Broad focus
**2023405**	block1	Control	pre	baak3	Object	Broad	r2	323.3196773	5	2	broad_focus	3	Broad focus
**2023405**	block1	Control	pre	jyun2	Subject	Contrastive	r2	325.7079365	1	1	pre_focus	2	Contrastive pre_focus
**2023405**	block1	Control	pre	jyun2	Subject	Contrastive	r2	232.6307256	2	2	pre_focus	2	Contrastive pre_focus
**2023405**	block1	Control	pre	mo2	Verb	Contrastive	r2	283.2802783	3	1	pre_focus	2	Contrastive pre_focus
**2023405**	block1	Control	pre	gau2	Object	Contrastive	r2	265.2664824	4	1	on_focus	2	Contrastive on_focus
**2023405**	block1	Control	pre	zai2	Object	Contrastive	r2	374.7674968	5	2	on_focus	2	Contrastive on_focus
**2023405**	block1	Control	pre	zoeng1	Subject	Broad	r2	270.860636	1	1	broad_focus	1	Broad focus
**2023405**	block1	Control	pre	saang1	Subject	Broad	r2	271.9962033	2	2	broad_focus	1	Broad focus
**2023405**	block1	Control	pre	tsa1	Verb	Broad	r2	212.0234356	3	1	broad_focus	1	Broad focus
**2023405**	block1	Control	pre	fei1	Object	Broad	r2	185.0931982	4	1	broad_focus	1	Broad focus
**2023405**	block1	Control	pre	gei1	Object	Broad	r2	351.1923694	5	2	broad_focus	1	Broad focus
**2023405**	block1	Control	pre	sau3	Subject	Contrastive	r2	170.5525723	1	1	pre_focus	3	Contrastive pre_focus

2023405	block1	Control	pre	sau3	Subject	Contrastive	r2	208.3757644	2	2	pre_focus	3	Contrastive pre_focus
2023405	block1	Control	pre	sik3	Verb	Contrastive	r2	423.3238787	3	1	on_focus	3	Contrastive on_focus
2023405	block1	Control	pre	baak3	Object	Contrastive	r2	229.4188198	4	1	post_focus	3	Contrastive post_focus
2023405	block1	Control	pre	baak3	Object	Contrastive	r2	1019.075401	5	2	post_focus	3	Contrastive post_focus
2023405	block1	Control	pre	zoeng1	Subject	Narrow	r2	267.487988	1	1	on_focus	1	Narrow on_focus
2023405	block1	Control	pre	saang1	Subject	Narrow	r2	186.0934391	2	2	on_focus	1	Narrow on_focus
2023405	block1	Control	pre	tsa1	Verb	Narrow	r2	161.8941117	3	1	post_focus	1	Narrow post_focus
2023405	block1	Control	pre	fei1	Object	Narrow	r2	133.1365329	4	1	post_focus	1	Narrow post_focus
2023405	block1	Control	pre	gei1	Object	Narrow	r2	401.4872119	5	2	post_focus	1	Narrow post_focus
2023405	block2	Control	post	ma4	Subject	Broad	r1	212.5164981	1	1	broad_focus	4	Broad focus
2023405	block2	Control	post	ma4	Subject	Broad	r1	255.1067221	2	2	broad_focus	4	Broad focus
2023405	block2	Control	post	fu4	Verb	Broad	r1	130.5101523	3	1	broad_focus	4	Broad focus
2023405	block2	Control	post	maang4	Object	Broad	r1	353.43519	4	1	broad_focus	4	Broad focus
2023405	block2	Control	post	Jan-04	Object	Broad	r1	279.0136043	5	2	broad_focus	4	Broad focus
2023405	block2	Control	post	ma4	Subject	Narrow	r1	201.7171852	1	1	on_focus	4	Narrow on_focus
2023405	block2	Control	post	ma4	Subject	Narrow	r1	236.652532	2	2	on_focus	4	Narrow on_focus
2023405	block2	Control	post	fu4	Verb	Narrow	r1	104.8619276	3	1	post_focus	4	Narrow post_focus
2023405	block2	Control	post	maang4	Object	Narrow	r1	291.1532486	4	1	post_focus	4	Narrow post_focus
2023405	block2	Control	post	Jan-04	Object	Narrow	r1	242.0552974	5	2	post_focus	4	Narrow post_focus
2023405	block2	Control	post	ngaa5	Subject	Broad	r1	138.8294855	1	1	broad_focus	5	Broad focus
2023405	block2	Control	post	ngaa5	Subject	Broad	r1	163.0564415	2	2	broad_focus	5	Broad focus
2023405	block2	Control	post	maai5	Verb	Broad	r1	162.0552171	3	1	broad_focus	5	Broad focus
2023405	block2	Control	post	pou5	Object	Broad	r1	110.8004666	4	1	broad_focus	5	Broad focus
2023405	block2	Control	post	pou5	Object	Broad	r1	135.9651723	5	2	broad_focus	5	Broad focus
2023405	block2	Control	post	ma4	Subject	Contrastive	r1	104.013268	1	1	pre_focus	4	Contrastive pre_focus
2023405	block2	Control	post	ma4	Subject	Contrastive	r1	133.648796	2	2	pre_focus	4	Contrastive pre_focus
2023405	block2	Control	post	fu4	Verb	Contrastive	r1	77.48404208	3	1	pre_focus	4	Contrastive pre_focus
2023405	block2	Control	post	maang4	Object	Contrastive	r1	232.5378705	4	1	on_focus	4	Contrastive on_focus
2023405	block2	Control	post	Jan-04	Object	Contrastive	r1	172.616521	5	2	on_focus	4	Contrastive on_focus
2023405	block2	Control	post	ma4	Subject	Contrastive	r1	99.11464054	1	1	pre_focus	4	Contrastive pre_focus
2023405	block2	Control	post	ma4	Subject	Contrastive	r1	198.4329211	2	2	pre_focus	4	Contrastive pre_focus
2023405	block2	Control	post	fu4	Verb	Contrastive	r1	125.3286485	3	1	on_focus	4	Contrastive on_focus
2023405	block2	Control	post	maang4	Object	Contrastive	r1	306.9627763	4	1	post_focus	4	Contrastive post_focus
2023405	block2	Control	post	Jan-04	Object	Contrastive	r1	245.7400342	5	2	post_focus	4	Contrastive post_focus
2023405	block2	Control	post	ma4	Subject	Narrow	r1	135.1548046	1	1	pre_focus	4	Narrow pre_focus
2023405	block2	Control	post	ma4	Subject	Narrow	r1	174.9934813	2	2	pre_focus	4	Narrow pre_focus
2023405	block2	Control	post	fu4	Verb	Narrow	r1	79.95669594	3	1	on_focus	4	Narrow on_focus
2023405	block2	Control	post	maang4	Object	Narrow	r1	301.0126056	4	1	post_focus	4	Narrow post_focus
2023405	block2	Control	post	Jan-04	Object	Narrow	r1	180.7652341	5	2	post_focus	4	Narrow post_focus
2023405	block2	Control	post	lok6	Subject	Narrow	r1	124.4752899	1	1	pre_focus	6	Narrow pre_focus
2023405	block2	Control	post	lok6	Subject	Narrow	r1	129.9070533	2	2	pre_focus	6	Narrow pre_focus
2023405	block2	Control	post	waa6	Verb	Narrow	r1	122.1603703	3	1	on_focus	6	Narrow on_focus
2023405	block2	Control	post	jyut6	Object	Narrow	r1	125.2625766	4	1	post_focus	6	Narrow post_focus
2023405	block2	Control	post	loeng6	Object	Narrow	r1	358.2880303	5	2	post_focus	6	Narrow post_focus
2023405	block2	Control	post	ngaa5	Subject	Narrow	r1	153.2933667	1	1	pre_focus	5	Narrow pre_focus
2023405	block2	Control	post	ngaa5	Subject	Narrow	r1	170.4943168	2	2	pre_focus	5	Narrow pre_focus
2023405	block2	Control	post	maai5	Verb	Narrow	r1	197.4154244	3	1	on_focus	5	Narrow on_focus
2023405	block2	Control	post	pou5	Object	Narrow	r1	120.9128233	4	1	post_focus	5	Narrow post_focus
2023405	block2	Control	post	pou5	Object	Narrow	r1	223.3847258	5	2	post_focus	5	Narrow post_focus
2023405	block2	Control	post	lok6	Subject	Broad	r1	155.0021759	1	1	broad_focus	6	Broad focus
2023405	block2	Control	post	lok6	Subject	Broad	r1	133.4464249	2	2	broad_focus	6	Broad focus
2023405	block2	Control	post	waa6	Verb	Broad	r1	153.0021836	3	1	broad_focus	6	Broad focus
2023405	block2	Control	post	jyut6	Object	Broad	r1	130.7914574	4	1	broad_focus	6	Broad focus
2023405	block2	Control	post	loeng6	Object	Broad	r1	296.4427782	5	2	broad_focus	6	Broad focus
2023405	block2	Control	post	lok6	Subject	Contrastive	r1	89.38017815	1	1	pre_focus	6	Contrastive pre_focus
2023405	block2	Control	post	lok6	Subject	Contrastive	r1	74.19539393	2	2	pre_focus	6	Contrastive pre_focus
2023405	block2	Control	post	waa6	Verb	Contrastive	r1	175.9417215	3	1	pre_focus	6	Contrastive pre_focus
2023405	block2	Control	post	jyut6	Object	Contrastive	r1	158.0878907	4	1	on_focus	6	Contrastive on_focus
2023405	block2	Control	post	loeng6	Object	Contrastive	r1	303.1776503	5	2	on_focus	6	Contrastive on_focus
2023405	block2	Control	post	lok6	Subject	Narrow	r1	80.86262799	1	1	pre_focus	6	Narrow pre_focus
2023405	block2	Control	post	lok6	Subject	Narrow	r1	123.7432057	2	2	pre_focus	6	Narrow pre_focus
2023405	block2	Control	post	waa6	Verb	Narrow	r1	159.3287822	3	1	pre_focus	6	Narrow pre_focus
2023405	block2	Control	post	jyut6	Object	Narrow	r1	126.4795085	4	1	on_focus	6	Narrow on_focus
2023405	block2	Control	post	loeng6	Object	Narrow	r1	330.6757459	5	2	on_focus	6	Narrow on_focus
2023405	block2	Control	post	ma4	Subject	Narrow	r1	112.4372004	1	1	pre_focus	4	Narrow pre_focus
2023405	block2	Control	post	ma4	Subject	Narrow	r1	163.1575108	2	2	pre_focus	4	Narrow pre_focus

2023405	block2	Control	post	fu4	Verb	Narrow	r1	77.36445007	3	1	pre_focus	4	Narrow pre_focus
2023405	block2	Control	post	maang4	Object	Narrow	r1	148.5113893	4	1	on_focus	4	Narrow on_focus
2023405	block2	Control	post	Jan-04	Object	Narrow	r1	298.9979345	5	2	on_focus	4	Narrow on_focus
2023405	block2	Control	post	ngaa5	Subject	Narrow	r1	148.2178932	1	1	pre_focus	5	Narrow pre_focus
2023405	block2	Control	post	ngaa5	Subject	Narrow	r1	113.3935867	2	2	pre_focus	5	Narrow pre_focus
2023405	block2	Control	post	maai5	Verb	Narrow	r1	156.8550119	3	1	pre_focus	5	Narrow pre_focus
2023405	block2	Control	post	pou5	Object	Narrow	r1	87.70493621	4	1	on_focus	5	Narrow on_focus
2023405	block2	Control	post	pou5	Object	Narrow	r1	221.1643389	5	2	on_focus	5	Narrow on_focus
2023405	block2	Control	post	ngaa5	Subject	Contrastive	r1	145.2651208	1	1	pre_focus	5	Contrastive pre_focus
2023405	block2	Control	post	ngaa5	Subject	Contrastive	r1	112.746709	2	2	pre_focus	5	Contrastive pre_focus
2023405	block2	Control	post	maai5	Verb	Contrastive	r1	179.2898075	3	1	pre_focus	5	Contrastive pre_focus
2023405	block2	Control	post	pou5	Object	Contrastive	r1	64.23098817	4	1	on_focus	5	Contrastive on_focus
2023405	block2	Control	post	pou5	Object	Contrastive	r1	289.0814099	5	2	on_focus	5	Contrastive on_focus
2023405	block2	Control	post	ngaa5	Subject	Contrastive	r1	151.2586284	1	1	pre_focus	5	Contrastive pre_focus
2023405	block2	Control	post	ngaa5	Subject	Contrastive	r1	162.0923837	2	2	pre_focus	5	Contrastive pre_focus
2023405	block2	Control	post	maai5	Verb	Contrastive	r1	179.8908159	3	1	on_focus	5	Contrastive on_focus
2023405	block2	Control	post	pou5	Object	Contrastive	r1	103.0122675	4	1	post_focus	5	Contrastive post_focus
2023405	block2	Control	post	pou5	Object	Contrastive	r1	240.2817519	5	2	post_focus	5	Contrastive post_focus
2023405	block2	Control	post	ngaa5	Subject	Contrastive	r1	181.7401617	1	1	on_focus	5	Contrastive on_focus
2023405	block2	Control	post	ngaa5	Subject	Contrastive	r1	157.2380656	2	2	on_focus	5	Contrastive on_focus
2023405	block2	Control	post	maai5	Verb	Contrastive	r1	179.5437807	3	1	post_focus	5	Contrastive post_focus
2023405	block2	Control	post	pou5	Object	Contrastive	r1	122.7292271	4	1	post_focus	5	Contrastive post_focus
2023405	block2	Control	post	pou5	Object	Contrastive	r1	246.3133264	5	2	post_focus	5	Contrastive post_focus
2023405	block2	Control	post	lok6	Subject	Contrastive	r1	96.32050028	1	1	on_focus	6	Contrastive on_focus
2023405	block2	Control	post	lok6	Subject	Contrastive	r1	106.1014383	2	2	on_focus	6	Contrastive on_focus
2023405	block2	Control	post	waa6	Verb	Contrastive	r1	154.1927216	3	1	post_focus	6	Contrastive post_focus
2023405	block2	Control	post	jyut6	Object	Contrastive	r1	76.3495282	4	1	post_focus	6	Contrastive post_focus
2023405	block2	Control	post	loeng6	Object	Contrastive	r1	247.4019453	5	2	post_focus	6	Contrastive post_focus
2023405	block2	Control	post	ngaa5	Subject	Narrow	r1	177.0680192	1	1	on_focus	5	Narrow on_focus
2023405	block2	Control	post	ngaa5	Subject	Narrow	r1	106.2981062	2	2	on_focus	5	Narrow on_focus
2023405	block2	Control	post	maai5	Verb	Narrow	r1	191.6547236	3	1	post_focus	5	Narrow post_focus
2023405	block2	Control	post	pou5	Object	Narrow	r1	106.1303266	4	1	post_focus	5	Narrow post_focus
2023405	block2	Control	post	pou5	Object	Narrow	r1	261.3419701	5	2	post_focus	5	Narrow post_focus
2023405	block2	Control	post	ma4	Subject	Contrastive	r1	177.4470478	1	1	on_focus	4	Contrastive on_focus
2023405	block2	Control	post	ma4	Subject	Contrastive	r1	136.4778974	2	2	on_focus	4	Contrastive on_focus
2023405	block2	Control	post	fu4	Verb	Contrastive	r1	138.042485	3	1	post_focus	4	Contrastive post_focus
2023405	block2	Control	post	maang4	Object	Contrastive	r1	170.6536711	4	1	post_focus	4	Contrastive post_focus
2023405	block2	Control	post	Jan-04	Object	Contrastive	r1	137.2673	5	2	post_focus	4	Contrastive post_focus
2023405	block2	Control	post	lok6	Subject	Contrastive	r1	173.4589593	1	1	pre_focus	6	Contrastive pre_focus
2023405	block2	Control	post	lok6	Subject	Contrastive	r1	118.5500343	2	2	pre_focus	6	Contrastive pre_focus
2023405	block2	Control	post	waa6	Verb	Contrastive	r1	158.7570872	3	1	on_focus	6	Contrastive on_focus
2023405	block2	Control	post	jyut6	Object	Contrastive	r1	139.3562099	4	1	post_focus	6	Contrastive post_focus
2023405	block2	Control	post	loeng6	Object	Contrastive	r1	191.0883478	5	2	post_focus	6	Contrastive post_focus
2023405	block2	Control	post	lok6	Subject	Narrow	r1	146.3148271	1	1	on_focus	6	Narrow on_focus
2023405	block2	Control	post	lok6	Subject	Narrow	r1	143.4107909	2	2	on_focus	6	Narrow on_focus
2023405	block2	Control	post	waa6	Verb	Narrow	r1	143.0844647	3	1	post_focus	6	Narrow post_focus
2023405	block2	Control	post	jyut6	Object	Narrow	r1	128.4119375	4	1	post_focus	6	Narrow post_focus
2023405	block2	Control	post	loeng6	Object	Narrow	r1	336.4088114	5	2	post_focus	6	Narrow post_focus
2023405	block2	Control	post	ma4	Subject	Broad	r2	114.9733154	1	1	broad_focus	4	Broad focus
2023405	block2	Control	post	ma4	Subject	Broad	r2	161.4438857	2	2	broad_focus	4	Broad focus
2023405	block2	Control	post	fu4	Verb	Broad	r2	128.2057696	3	1	broad_focus	4	Broad focus
2023405	block2	Control	post	maang4	Object	Broad	r2	193.4718529	4	1	broad_focus	4	Broad focus
2023405	block2	Control	post	Jan-04	Object	Broad	r2	145.7885994	5	2	broad_focus	4	Broad focus
2023405	block2	Control	post	ma4	Subject	Contrastive	r2	118.1294187	1	1	pre_focus	4	Contrastive pre_focus
2023405	block2	Control	post	ma4	Subject	Contrastive	r2	125.4963515	2	2	pre_focus	4	Contrastive pre_focus
2023405	block2	Control	post	fu4	Verb	Contrastive	r2	148.0757061	3	1	pre_focus	4	Contrastive pre_focus
2023405	block2	Control	post	maang4	Object	Contrastive	r2	300.5275454	4	1	on_focus	4	Contrastive on_focus
2023405	block2	Control	post	Jan-04	Object	Contrastive	r2	261.8736569	5	2	on_focus	4	Contrastive on_focus
2023405	block2	Control	post	ngaa5	Subject	Narrow	r2	134.9314719	1	1	pre_focus	5	Narrow pre_focus
2023405	block2	Control	post	ngaa5	Subject	Narrow	r2	159.3716814	2	2	pre_focus	5	Narrow pre_focus
2023405	block2	Control	post	maai5	Verb	Narrow	r2	177.9160736	3	1	pre_focus	5	Narrow pre_focus
2023405	block2	Control	post	pou5	Object	Narrow	r2	96.13621271	4	1	on_focus	5	Narrow on_focus
2023405	block2	Control	post	pou5	Object	Narrow	r2	301.266399	5	2	on_focus	5	Narrow on_focus
2023405	block2	Control	post	ma4	Subject	Narrow	r2	155.0004445	1	1	pre_focus	4	Narrow pre_focus
2023405	block2	Control	post	ma4	Subject	Narrow	r2	174.672049	2	2	pre_focus	4	Narrow pre_focus
2023405	block2	Control	post	fu4	Verb	Narrow	r2	83.87234274	3	1	pre_focus	4	Narrow pre_focus

2023405	block2	Control	post	maang4	Object	Narrow	r2	284.7805771	4	1	on_focus	4	Narrow on_focus
2023405	block2	Control	post	Jan-04	Object	Narrow	r2	255.3412319	5	2	on_focus	4	Narrow on_focus
2023405	block2	Control	post	ngaa5	Subject	Contrastive	r2	131.8851252	1	1	pre_focus	5	Contrastive pre_focus
2023405	block2	Control	post	ngaa5	Subject	Contrastive	r2	173.6552589	2	2	pre_focus	5	Contrastive pre_focus
2023405	block2	Control	post	maai5	Verb	Contrastive	r2	183.7584203	3	1	pre_focus	5	Contrastive pre_focus
2023405	block2	Control	post	pou5	Object	Contrastive	r2	120.7248663	4	1	on_focus	5	Contrastive on_focus
2023405	block2	Control	post	pou5	Object	Contrastive	r2	299.9185569	5	2	on_focus	5	Contrastive on_focus
2023405	block2	Control	post	ngaa5	Subject	Narrow	r2	137.1136223	1	1	pre_focus	5	Narrow pre_focus
2023405	block2	Control	post	ngaa5	Subject	Narrow	r2	168.5259942	2	2	pre_focus	5	Narrow pre_focus
2023405	block2	Control	post	maai5	Verb	Narrow	r2	203.8256916	3	1	on_focus	5	Narrow on_focus
2023405	block2	Control	post	pou5	Object	Narrow	r2	91.96579251	4	1	post_focus	5	Narrow post_focus
2023405	block2	Control	post	pou5	Object	Narrow	r2	269.6576993	5	2	post_focus	5	Narrow post_focus
2023405	block2	Control	post	lok6	Subject	Narrow	r2	125.2177396	1	1	pre_focus	6	Narrow pre_focus
2023405	block2	Control	post	lok6	Subject	Narrow	r2	141.4510304	2	2	pre_focus	6	Narrow pre_focus
2023405	block2	Control	post	waa6	Verb	Narrow	r2	151.0786278	3	1	on_focus	6	Narrow on_focus
2023405	block2	Control	post	jyut6	Object	Narrow	r2	106.1316143	4	1	post_focus	6	Narrow post_focus
2023405	block2	Control	post	loeng6	Object	Narrow	r2	338.2926234	5	2	post_focus	6	Narrow post_focus
2023405	block2	Control	post	lok6	Subject	Contrastive	r2	138.6590033	1	1	pre_focus	6	Contrastive pre_focus
2023405	block2	Control	post	lok6	Subject	Contrastive	r2	132.2231284	2	2	pre_focus	6	Contrastive pre_focus
2023405	block2	Control	post	waa6	Verb	Contrastive	r2	122.1294818	3	1	pre_focus	6	Contrastive pre_focus
2023405	block2	Control	post	jyut6	Object	Contrastive	r2	100.6796028	4	1	on_focus	6	Contrastive on_focus
2023405	block2	Control	post	loeng6	Object	Contrastive	r2	315.2760956	5	2	on_focus	6	Contrastive on_focus
2023405	block2	Control	post	ma4	Subject	Narrow	r2	179.5583195	1	1	on_focus	4	Narrow on_focus
2023405	block2	Control	post	ma4	Subject	Narrow	r2	162.0704943	2	2	on_focus	4	Narrow on_focus
2023405	block2	Control	post	fu4	Verb	Narrow	r2	103.1465366	3	1	post_focus	4	Narrow post_focus
2023405	block2	Control	post	maang4	Object	Narrow	r2	214.6485241	4	1	post_focus	4	Narrow post_focus
2023405	block2	Control	post	Jan-04	Object	Narrow	r2	240.9026214	5	2	post_focus	4	Narrow post_focus
2023405	block2	Control	post	ngaa5	Subject	Broad	r2	205.4620605	1	1	broad_focus	5	Broad focus
2023405	block2	Control	post	ngaa5	Subject	Broad	r2	143.9834152	2	2	broad_focus	5	Broad focus
2023405	block2	Control	post	maai5	Verb	Broad	r2	195.3962082	3	1	broad_focus	5	Broad focus
2023405	block2	Control	post	pou5	Object	Broad	r2	95.49868432	4	1	broad_focus	5	Broad focus
2023405	block2	Control	post	pou5	Object	Broad	r2	312.56787	5	2	broad_focus	5	Broad focus
2023405	block2	Control	post	lok6	Subject	Broad	r2	105.4786325	1	1	broad_focus	6	Broad focus
2023405	block2	Control	post	lok6	Subject	Broad	r2	155.6438559	2	2	broad_focus	6	Broad focus
2023405	block2	Control	post	waa6	Verb	Broad	r2	165.5026447	3	1	broad_focus	6	Broad focus
2023405	block2	Control	post	jyut6	Object	Broad	r2	89.7992477	4	1	broad_focus	6	Broad focus
2023405	block2	Control	post	loeng6	Object	Broad	r2	343.3664775	5	2	broad_focus	6	Broad focus
2023405	block2	Control	post	lok6	Subject	Narrow	r2	137.0696646	1	1	on_focus	6	Narrow on_focus
2023405	block2	Control	post	lok6	Subject	Narrow	r2	129.3189407	2	2	on_focus	6	Narrow on_focus
2023405	block2	Control	post	waa6	Verb	Narrow	r2	171.9167596	3	1	post_focus	6	Narrow post_focus
2023405	block2	Control	post	jyut6	Object	Narrow	r2	158.2232692	4	1	post_focus	6	Narrow post_focus
2023405	block2	Control	post	loeng6	Object	Narrow	r2	298.6548238	5	2	post_focus	6	Narrow post_focus
2023405	block2	Control	post	lok6	Subject	Narrow	r2	146.4656916	1	1	pre_focus	6	Narrow pre_focus
2023405	block2	Control	post	lok6	Subject	Narrow	r2	117.3338797	2	2	pre_focus	6	Narrow pre_focus
2023405	block2	Control	post	waa6	Verb	Narrow	r2	142.7297663	3	1	pre_focus	6	Narrow pre_focus
2023405	block2	Control	post	jyut6	Object	Narrow	r2	122.9620516	4	1	on_focus	6	Narrow on_focus
2023405	block2	Control	post	loeng6	Object	Narrow	r2	349.6408605	5	2	on_focus	6	Narrow on_focus
2023405	block2	Control	post	ma4	Subject	Narrow	r2	184.1789966	1	1	pre_focus	4	Narrow pre_focus
2023405	block2	Control	post	ma4	Subject	Narrow	r2	162.8401143	2	2	pre_focus	4	Narrow pre_focus
2023405	block2	Control	post	fu4	Verb	Narrow	r2	119.7363494	3	1	on_focus	4	Narrow on_focus
2023405	block2	Control	post	maang4	Object	Narrow	r2	191.6792508	4	1	post_focus	4	Narrow post_focus
2023405	block2	Control	post	Jan-04	Object	Narrow	r2	265.3138162	5	2	post_focus	4	Narrow post_focus
2023405	block2	Control	post	ma4	Subject	Contrastive	r2	125.2666711	1	1	pre_focus	4	Contrastive pre_focus
2023405	block2	Control	post	ma4	Subject	Contrastive	r2	182.4574343	2	2	pre_focus	4	Contrastive pre_focus
2023405	block2	Control	post	fu4	Verb	Contrastive	r2	115.966292	3	1	on_focus	4	Contrastive on_focus
2023405	block2	Control	post	maang4	Object	Contrastive	r2	238.6319429	4	1	post_focus	4	Contrastive post_focus
2023405	block2	Control	post	Jan-04	Object	Contrastive	r2	346.8696217	5	2	post_focus	4	Contrastive post_focus
2023405	block2	Control	post	ngaa5	Subject	Contrastive	r2	155.5648527	1	1	pre_focus	5	Contrastive pre_focus
2023405	block2	Control	post	ngaa5	Subject	Contrastive	r2	156.2698492	2	2	pre_focus	5	Contrastive pre_focus
2023405	block2	Control	post	maai5	Verb	Contrastive	r2	232.5413388	3	1	on_focus	5	Contrastive on_focus
2023405	block2	Control	post	pou5	Object	Contrastive	r2	125.590103	4	1	post_focus	5	Contrastive post_focus
2023405	block2	Control	post	pou5	Object	Contrastive	r2	267.6986182	5	2	post_focus	5	Contrastive post_focus
2023405	block2	Control	post	lok6	Subject	Contrastive	r2	239.8940867	1	1	pre_focus	6	Contrastive pre_focus
2023405	block2	Control	post	lok6	Subject	Contrastive	r2	197.3460758	2	2	pre_focus	6	Contrastive pre_focus
2023405	block2	Control	post	waa6	Verb	Contrastive	r2	227.6017617	3	1	on_focus	6	Contrastive on_focus
2023405	block2	Control	post	jyut6	Object	Contrastive	r2	118.1271514	4	1	post_focus	6	Contrastive post_focus

2023405	block2	Control	post	loeng6	Object	Contrastive	r2	318.0683272	5	2	post_focus	6	Contrastive post_focus
2023405	block2	Control	post	lok6	Subject	Contrastive	r2	187.222684	1	1	on_focus	6	Contrastive on_focus
2023405	block2	Control	post	lok6	Subject	Contrastive	r2	166.4323559	2	2	on_focus	6	Contrastive on_focus
2023405	block2	Control	post	waa6	Verb	Contrastive	r2	184.4788896	3	1	post_focus	6	Contrastive post_focus
2023405	block2	Control	post	jyut6	Object	Contrastive	r2	112.5384994	4	1	post_focus	6	Contrastive post_focus
2023405	block2	Control	post	loeng6	Object	Contrastive	r2	351.5282806	5	2	post_focus	6	Contrastive post_focus
2023405	block2	Control	post	ma4	Subject	Contrastive	r2	189.6616706	1	1	on_focus	4	Contrastive on_focus
2023405	block2	Control	post	ma4	Subject	Contrastive	r2	176.5824186	2	2	on_focus	4	Contrastive on_focus
2023405	block2	Control	post	fu4	Verb	Contrastive	r2	120.5702849	3	1	post_focus	4	Contrastive post_focus
2023405	block2	Control	post	maang4	Object	Contrastive	r2	208.2511846	4	1	post_focus	4	Contrastive post_focus
2023405	block2	Control	post	Jan-04	Object	Contrastive	r2	260.9524628	5	2	post_focus	4	Contrastive post_focus
2023405	block2	Control	post	ngaa5	Subject	Contrastive	r2	172.8369458	1	1	on_focus	5	Contrastive on_focus
2023405	block2	Control	post	ngaa5	Subject	Contrastive	r2	143.0769304	2	2	on_focus	5	Contrastive on_focus
2023405	block2	Control	post	maai5	Verb	Contrastive	r2	245.0395081	3	1	post_focus	5	Contrastive post_focus
2023405	block2	Control	post	pou5	Object	Contrastive	r2	82.00000515	4	1	post_focus	5	Contrastive post_focus
2023405	block2	Control	post	pou5	Object	Contrastive	r2	318.1506994	5	2	post_focus	5	Contrastive post_focus
2023405	block2	Control	post	ngaa5	Subject	Narrow	r2	307.7099498	1	1	on_focus	5	Narrow on_focus
2023405	block2	Control	post	ngaa5	Subject	Narrow	r2	152.8502137	2	2	on_focus	5	Narrow on_focus
2023405	block2	Control	post	maai5	Verb	Narrow	r2	233.9651436	3	1	post_focus	5	Narrow post_focus
2023405	block2	Control	post	pou5	Object	Narrow	r2	149.8714515	4	1	post_focus	5	Narrow post_focus
2023405	block2	Control	post	pou5	Object	Narrow	r2	254.7583898	5	2	post_focus	5	Narrow post_focus
2023405	block2	Control	pre	ma4	Subject	Narrow	r1	247.7104996	1	1	on_focus	4	Narrow on_focus
2023405	block2	Control	pre	ma4	Subject	Narrow	r1	248.8241686	2	2	on_focus	4	Narrow on_focus
2023405	block2	Control	pre	fu4	Verb	Narrow	r1	133.9879181	3	1	post_focus	4	Narrow post_focus
2023405	block2	Control	pre	maang4	Object	Narrow	r1	367.8169635	4	1	post_focus	4	Narrow post_focus
2023405	block2	Control	pre	Jan-04	Object	Narrow	r1	175.2353741	5	2	post_focus	4	Narrow post_focus
2023405	block2	Control	pre	ngaa5	Subject	Contrastive	r1	219.9579065	1	1	pre_focus	5	Contrastive pre_focus
2023405	block2	Control	pre	ngaa5	Subject	Contrastive	r1	232.1921487	2	2	pre_focus	5	Contrastive pre_focus
2023405	block2	Control	pre	maai5	Verb	Contrastive	r1	661.0383936	3	1	on_focus	5	Contrastive on_focus
2023405	block2	Control	pre	pou5	Object	Contrastive	r1	242.9501443	4	1	post_focus	5	Contrastive post_focus
2023405	block2	Control	pre	pou5	Object	Contrastive	r1	336.3846669	5	2	post_focus	5	Contrastive post_focus
2023405	block2	Control	pre	ngaa5	Subject	Narrow	r1	447.723095	1	1	on_focus	5	Narrow on_focus
2023405	block2	Control	pre	ngaa5	Subject	Narrow	r1	320.272661	2	2	on_focus	5	Narrow on_focus
2023405	block2	Control	pre	maai5	Verb	Narrow	r1	341.1500626	3	1	post_focus	5	Narrow post_focus
2023405	block2	Control	pre	pou5	Object	Narrow	r1	322.8690476	4	1	post_focus	5	Narrow post_focus
2023405	block2	Control	pre	pou5	Object	Narrow	r1	291.489955	5	2	post_focus	5	Narrow post_focus
2023405	block2	Control	pre	lok6	Subject	Broad	r1	293.6849721	1	1	broad_focus	6	Broad focus
2023405	block2	Control	pre	lok6	Subject	Broad	r1	305.8947582	2	2	broad_focus	6	Broad focus
2023405	block2	Control	pre	waa6	Verb	Broad	r1	219.3382394	3	1	broad_focus	6	Broad focus
2023405	block2	Control	pre	jyut6	Object	Broad	r1	198.7161101	4	1	broad_focus	6	Broad focus
2023405	block2	Control	pre	loeng6	Object	Broad	r1	509.9297739	5	2	broad_focus	6	Broad focus
2023405	block2	Control	pre	ngaa5	Subject	Broad	r1	308.4199477	1	1	broad_focus	5	Broad focus
2023405	block2	Control	pre	ngaa5	Subject	Broad	r1	232.2558213	2	2	broad_focus	5	Broad focus
2023405	block2	Control	pre	maai5	Verb	Broad	r1	263.1012395	3	1	broad_focus	5	Broad focus
2023405	block2	Control	pre	pou5	Object	Broad	r1	150.5309885	4	1	broad_focus	5	Broad focus
2023405	block2	Control	pre	pou5	Object	Broad	r1	369.8422777	5	2	broad_focus	5	Broad focus
2023405	block2	Control	pre	lok6	Subject	Narrow	r1	97.69198939	1	1	pre_focus	6	Narrow pre_focus
2023405	block2	Control	pre	lok6	Subject	Narrow	r1	105.523129	2	2	pre_focus	6	Narrow pre_focus
2023405	block2	Control	pre	waa6	Verb	Narrow	r1	193.7017812	3	1	on_focus	6	Narrow on_focus
2023405	block2	Control	pre	jyut6	Object	Narrow	r1	124.2926755	4	1	post_focus	6	Narrow post_focus
2023405	block2	Control	pre	loeng6	Object	Narrow	r1	322.3008903	5	2	post_focus	6	Narrow post_focus
2023405	block2	Control	pre	lok6	Subject	Contrastive	r1	176.6251542	1	1	on_focus	6	Contrastive on_focus
2023405	block2	Control	pre	lok6	Subject	Contrastive	r1	101.3986001	2	2	on_focus	6	Contrastive on_focus
2023405	block2	Control	pre	waa6	Verb	Contrastive	r1	177.9629752	3	1	post_focus	6	Contrastive post_focus
2023405	block2	Control	pre	jyut6	Object	Contrastive	r1	164.5616542	4	1	post_focus	6	Contrastive post_focus
2023405	block2	Control	pre	loeng6	Object	Contrastive	r1	363.2021627	5	2	post_focus	6	Contrastive post_focus
2023405	block2	Control	pre	ngaa5	Subject	Contrastive	r1	243.3999449	1	1	pre_focus	5	Contrastive pre_focus
2023405	block2	Control	pre	ngaa5	Subject	Contrastive	r1	150.9572809	2	2	pre_focus	5	Contrastive pre_focus
2023405	block2	Control	pre	maai5	Verb	Contrastive	r1	198.4037135	3	1	pre_focus	5	Contrastive pre_focus
2023405	block2	Control	pre	pou5	Object	Contrastive	r1	146.9258464	4	1	on_focus	5	Contrastive on_focus
2023405	block2	Control	pre	pou5	Object	Contrastive	r1	342.2243791	5	2	on_focus	5	Contrastive on_focus
2023405	block2	Control	pre	ma4	Subject	Contrastive	r1	150.8887233	1	1	pre_focus	4	Contrastive pre_focus
2023405	block2	Control	pre	ma4	Subject	Contrastive	r1	154.7600552	2	2	pre_focus	4	Contrastive pre_focus
2023405	block2	Control	pre	fu4	Verb	Contrastive	r1	110.0896955	3	1	pre_focus	4	Contrastive pre_focus
2023405	block2	Control	pre	maang4	Object	Contrastive	r1	285.116674	4	1	on_focus	4	Contrastive on_focus
2023405	block2	Control	pre	Jan-04	Object	Contrastive	r1	292.2725013	5	2	on_focus	4	Contrastive on_focus

**2023405**	block2	Control	pre	lok6	Subject	Narrow	r1	170.5663061	1	1	pre_focus	6	Narrow pre_focus
**2023405**	block2	Control	pre	lok6	Subject	Narrow	r1	148.61365	2	2	pre_focus	6	Narrow pre_focus
**2023405**	block2	Control	pre	waa6	Verb	Narrow	r1	133.732716	3	1	pre_focus	6	Narrow pre_focus
**2023405**	block2	Control	pre	jyut6	Object	Narrow	r1	183.4482713	4	1	on_focus	6	Narrow on_focus
**2023405**	block2	Control	pre	loeng6	Object	Narrow	r1	389.4446798	5	2	on_focus	6	Narrow on_focus
**2023405**	block2	Control	pre	ngaa5	Subject	Narrow	r1	234.8549939	1	1	pre_focus	5	Narrow pre_focus
**2023405**	block2	Control	pre	ngaa5	Subject	Narrow	r1	221.3257	2	2	pre_focus	5	Narrow pre_focus
**2023405**	block2	Control	pre	maai5	Verb	Narrow	r1	281.5104158	3	1	on_focus	5	Narrow on_focus
**2023405**	block2	Control	pre	pou5	Object	Narrow	r1	160.3597572	4	1	post_focus	5	Narrow post_focus
**2023405**	block2	Control	pre	pou5	Object	Narrow	r1	251.0111509	5	2	post_focus	5	Narrow post_focus
**2023405**	block2	Control	pre	ngaa5	Subject	Contrastive	r1	424.5676177	1	1	on_focus	5	Contrastive on_focus
**2023405**	block2	Control	pre	ngaa5	Subject	Contrastive	r1	247.9426018	2	2	on_focus	5	Contrastive on_focus
**2023405**	block2	Control	pre	maai5	Verb	Contrastive	r1	251.9772172	3	1	post_focus	5	Contrastive post_focus
**2023405**	block2	Control	pre	pou5	Object	Contrastive	r1	151.1601901	4	1	post_focus	5	Contrastive post_focus
**2023405**	block2	Control	pre	pou5	Object	Contrastive	r1	305.2164017	5	2	post_focus	5	Contrastive post_focus
**2023405**	block2	Control	pre	lok6	Subject	Narrow	r1	121.6299072	1	1	on_focus	6	Narrow on_focus
**2023405**	block2	Control	pre	lok6	Subject	Narrow	r1	121.0475897	2	2	on_focus	6	Narrow on_focus
**2023405**	block2	Control	pre	waa6	Verb	Narrow	r1	122.154929	3	1	post_focus	6	Narrow post_focus
**2023405**	block2	Control	pre	jyut6	Object	Narrow	r1	129.4570881	4	1	post_focus	6	Narrow post_focus
**2023405**	block2	Control	pre	loeng6	Object	Narrow	r1	364.978355	5	2	post_focus	6	Narrow post_focus
**2023405**	block2	Control	pre	ma4	Subject	Broad	r1	184.5074506	1	1	broad_focus	4	Broad focus
**2023405**	block2	Control	pre	ma4	Subject	Broad	r1	182.8155107	2	2	broad_focus	4	Broad focus
**2023405**	block2	Control	pre	fu4	Verb	Broad	r1	89.3866117	3	1	broad_focus	4	Broad focus
**2023405**	block2	Control	pre	maang4	Object	Broad	r1	302.4897196	4	1	broad_focus	4	Broad focus
**2023405**	block2	Control	pre	Jan-04	Object	Broad	r1	366.8201681	5	2	broad_focus	4	Broad focus
**2023405**	block2	Control	pre	ma4	Subject	Contrastive	r1	191.1202779	1	1	on_focus	4	Contrastive on_focus
**2023405**	block2	Control	pre	ma4	Subject	Contrastive	r1	146.1646795	2	2	on_focus	4	Contrastive on_focus
**2023405**	block2	Control	pre	fu4	Verb	Contrastive	r1	134.0951751	3	1	post_focus	4	Contrastive post_focus
**2023405**	block2	Control	pre	maang4	Object	Contrastive	r1	203.3885353	4	1	post_focus	4	Contrastive post_focus
**2023405**	block2	Control	pre	Jan-04	Object	Contrastive	r1	315.8980857	5	2	post_focus	4	Contrastive post_focus
**2023405**	block2	Control	pre	ma4	Subject	Narrow	r1	152.4276106	1	1	pre_focus	4	Narrow pre_focus
**2023405**	block2	Control	pre	ma4	Subject	Narrow	r1	165.6553489	2	2	pre_focus	4	Narrow pre_focus
**2023405**	block2	Control	pre	fu4	Verb	Narrow	r1	147.1944028	3	1	on_focus	4	Narrow on_focus
**2023405**	block2	Control	pre	maang4	Object	Narrow	r1	232.7549231	4	1	post_focus	4	Narrow post_focus
**2023405**	block2	Control	pre	Jan-04	Object	Narrow	r1	293.3466683	5	2	post_focus	4	Narrow post_focus
**2023405**	block2	Control	pre	ngaa5	Subject	Narrow	r1	256.7360863	1	1	pre_focus	5	Narrow pre_focus
**2023405**	block2	Control	pre	ngaa5	Subject	Narrow	r1	152.2536416	2	2	pre_focus	5	Narrow pre_focus
**2023405**	block2	Control	pre	maai5	Verb	Narrow	r1	216.7623755	3	1	pre_focus	5	Narrow pre_focus
**2023405**	block2	Control	pre	pou5	Object	Narrow	r1	79.49487841	4	1	on_focus	5	Narrow on_focus
**2023405**	block2	Control	pre	pou5	Object	Narrow	r1	304.8323255	5	2	on_focus	5	Narrow on_focus
**2023405**	block2	Control	pre	lok6	Subject	Contrastive	r1	217.2036415	1	1	pre_focus	6	Contrastive pre_focus
**2023405**	block2	Control	pre	lok6	Subject	Contrastive	r1	155.4907864	2	2	pre_focus	6	Contrastive pre_focus
**2023405**	block2	Control	pre	waa6	Verb	Contrastive	r1	213.2251377	3	1	on_focus	6	Contrastive on_focus
**2023405**	block2	Control	pre	jyut6	Object	Contrastive	r1	141.4092684	4	1	post_focus	6	Contrastive post_focus
**2023405**	block2	Control	pre	loeng6	Object	Contrastive	r1	329.6977943	5	2	post_focus	6	Contrastive post_focus
**2023405**	block2	Control	pre	ma4	Subject	Narrow	r1	166.7124781	1	1	pre_focus	4	Narrow pre_focus
**2023405**	block2	Control	pre	ma4	Subject	Narrow	r1	196.6631089	2	2	pre_focus	4	Narrow pre_focus
**2023405**	block2	Control	pre	fu4	Verb	Narrow	r1	177.6541572	3	1	pre_focus	4	Narrow pre_focus
**2023405**	block2	Control	pre	maang4	Object	Narrow	r1	310.5565164	4	1	on_focus	4	Narrow on_focus
**2023405**	block2	Control	pre	Jan-04	Object	Narrow	r1	316.200705	5	2	on_focus	4	Narrow on_focus
**2023405**	block2	Control	pre	lok6	Subject	Contrastive	r1	216.1474217	1	1	pre_focus	6	Contrastive pre_focus
**2023405**	block2	Control	pre	lok6	Subject	Contrastive	r1	141.5231812	2	2	pre_focus	6	Contrastive pre_focus
**2023405**	block2	Control	pre	waa6	Verb	Contrastive	r1	201.7463339	3	1	pre_focus	6	Contrastive pre_focus
**2023405**	block2	Control	pre	jyut6	Object	Contrastive	r1	110.2283397	4	1	on_focus	6	Contrastive on_focus
**2023405**	block2	Control	pre	loeng6	Object	Contrastive	r1	236.6803835	5	2	on_focus	6	Contrastive on_focus
**2023405**	block2	Control	pre	ma4	Subject	Contrastive	r1	227.6327464	1	1	pre_focus	4	Contrastive pre_focus
**2023405**	block2	Control	pre	ma4	Subject	Contrastive	r1	210.8685046	2	2	pre_focus	4	Contrastive pre_focus
**2023405**	block2	Control	pre	fu4	Verb	Contrastive	r1	78.14261188	3	1	on_focus	4	Contrastive on_focus
**2023405**	block2	Control	pre	maang4	Object	Contrastive	r1	332.4078598	4	1	post_focus	4	Contrastive post_focus
**2023405**	block2	Control	pre	Jan-04	Object	Contrastive	r1	346.0768494	5	2	post_focus	4	Contrastive post_focus
**2023405**	block2	Control	pre	lok6	Subject	Broad	r2	131.2655531	1	1	broad_focus	6	Broad focus
**2023405**	block2	Control	pre	lok6	Subject	Broad	r2	109.303051	2	2	broad_focus	6	Broad focus
**2023405**	block2	Control	pre	waa6	Verb	Broad	r2	189.5350132	3	1	broad_focus	6	Broad focus
**2023405**	block2	Control	pre	jyut6	Object	Broad	r2	117.9310318	4	1	broad_focus	6	Broad focus
**2023405**	block2	Control	pre	loeng6	Object	Broad	r2	336.6418452	5	2	broad_focus	6	Broad focus
**2023405**	block2	Control	pre	ngaa5	Subject	Narrow	r2	221.7180108	1	1	pre_focus	5	Narrow pre_focus

2023405	block2	Control	pre	ngaa5	Subject	Narrow	r2	152.6244946	2	2	pre_focus	5	Narrow pre_focus
2023405	block2	Control	pre	maai5	Verb	Narrow	r2	228.2712837	3	1	pre_focus	5	Narrow pre_focus
2023405	block2	Control	pre	pou5	Object	Narrow	r2	151.3050802	4	1	on_focus	5	Narrow on_focus
2023405	block2	Control	pre	pou5	Object	Narrow	r2	421.553506	5	2	on_focus	5	Narrow on_focus
2023405	block2	Control	pre	ngaa5	Subject	Narrow	r2	227.0599395	1	1	pre_focus	5	Narrow pre_focus
2023405	block2	Control	pre	ngaa5	Subject	Narrow	r2	159.9907309	2	2	pre_focus	5	Narrow pre_focus
2023405	block2	Control	pre	maai5	Verb	Narrow	r2	242.797499	3	1	on_focus	5	Narrow on_focus
2023405	block2	Control	pre	pou5	Object	Narrow	r2	104.5604572	4	1	post_focus	5	Narrow post_focus
2023405	block2	Control	pre	pou5	Object	Narrow	r2	361.4638605	5	2	post_focus	5	Narrow post_focus
2023405	block2	Control	pre	lok6	Subject	Narrow	r2	107.0603234	1	1	pre_focus	6	Narrow pre_focus
2023405	block2	Control	pre	lok6	Subject	Narrow	r2	109.8179916	2	2	pre_focus	6	Narrow pre_focus
2023405	block2	Control	pre	waa6	Verb	Narrow	r2	175.3887425	3	1	pre_focus	6	Narrow pre_focus
2023405	block2	Control	pre	jyut6	Object	Narrow	r2	166.9477359	4	1	on_focus	6	Narrow on_focus
2023405	block2	Control	pre	loeng6	Object	Narrow	r2	259.2094889	5	2	on_focus	6	Narrow on_focus
2023405	block2	Control	pre	ma4	Subject	Narrow	r2	241.1288723	1	1	on_focus	4	Narrow on_focus
2023405	block2	Control	pre	ma4	Subject	Narrow	r2	206.9183407	2	2	on_focus	4	Narrow on_focus
2023405	block2	Control	pre	fu4	Verb	Narrow	r2	102.1899093	3	1	post_focus	4	Narrow post_focus
2023405	block2	Control	pre	maang4	Object	Narrow	r2	289.1372422	4	1	post_focus	4	Narrow post_focus
2023405	block2	Control	pre	Jan-04	Object	Narrow	r2	238.6079209	5	2	post_focus	4	Narrow post_focus
2023405	block2	Control	pre	ma4	Subject	Narrow	r2	192.0162746	1	1	pre_focus	4	Narrow pre_focus
2023405	block2	Control	pre	ma4	Subject	Narrow	r2	214.3157794	2	2	pre_focus	4	Narrow pre_focus
2023405	block2	Control	pre	fu4	Verb	Narrow	r2	115.8951434	3	1	pre_focus	4	Narrow pre_focus
2023405	block2	Control	pre	maang4	Object	Narrow	r2	238.0284455	4	1	on_focus	4	Narrow on_focus
2023405	block2	Control	pre	Jan-04	Object	Narrow	r2	278.7647588	5	2	on_focus	4	Narrow on_focus
2023405	block2	Control	pre	ngaa5	Subject	Narrow	r2	279.1610504	1	1	on_focus	5	Narrow on_focus
2023405	block2	Control	pre	ngaa5	Subject	Narrow	r2	213.6615362	2	2	on_focus	5	Narrow on_focus
2023405	block2	Control	pre	maai5	Verb	Narrow	r2	312.1313745	3	1	post_focus	5	Narrow post_focus
2023405	block2	Control	pre	pou5	Object	Narrow	r2	186.5629827	4	1	post_focus	5	Narrow post_focus
2023405	block2	Control	pre	pou5	Object	Narrow	r2	244.121594	5	2	post_focus	5	Narrow post_focus
2023405	block2	Control	pre	ngaa5	Subject	Contrastive	r2	295.2227558	1	1	pre_focus	5	Contrastive pre_focus
2023405	block2	Control	pre	ngaa5	Subject	Contrastive	r2	234.7771643	2	2	pre_focus	5	Contrastive pre_focus
2023405	block2	Control	pre	maai5	Verb	Contrastive	r2	314.3496749	3	1	on_focus	5	Contrastive on_focus
2023405	block2	Control	pre	pou5	Object	Contrastive	r2	248.2683296	4	1	post_focus	5	Contrastive post_focus
2023405	block2	Control	pre	pou5	Object	Contrastive	r2	395.7777581	5	2	post_focus	5	Contrastive post_focus
2023405	block2	Control	pre	ma4	Subject	Broad	r2	221.4166368	1	1	broad_focus	4	Broad focus
2023405	block2	Control	pre	ma4	Subject	Broad	r2	186.6796761	2	2	broad_focus	4	Broad focus
2023405	block2	Control	pre	fu4	Verb	Broad	r2	131.3175871	3	1	broad_focus	4	Broad focus
2023405	block2	Control	pre	maang4	Object	Broad	r2	351.7585034	4	1	broad_focus	4	Broad focus
2023405	block2	Control	pre	Jan-04	Object	Broad	r2	265.1736218	5	2	broad_focus	4	Broad focus
2023405	block2	Control	pre	lok6	Subject	Narrow	r2	208.415373	1	1	on_focus	6	Narrow on_focus
2023405	block2	Control	pre	lok6	Subject	Narrow	r2	198.2826168	2	2	on_focus	6	Narrow on_focus
2023405	block2	Control	pre	waa6	Verb	Narrow	r2	202.9719139	3	1	post_focus	6	Narrow post_focus
2023405	block2	Control	pre	jyut6	Object	Narrow	r2	124.3444968	4	1	post_focus	6	Narrow post_focus
2023405	block2	Control	pre	loeng6	Object	Narrow	r2	272.0560087	5	2	post_focus	6	Narrow post_focus
2023405	block2	Control	pre	ma4	Subject	Contrastive	r2	205.8917063	1	1	pre_focus	4	Contrastive pre_focus
2023405	block2	Control	pre	ma4	Subject	Contrastive	r2	193.5853966	2	2	pre_focus	4	Contrastive pre_focus
2023405	block2	Control	pre	fu4	Verb	Contrastive	r2	89.53509073	3	1	pre_focus	4	Contrastive pre_focus
2023405	block2	Control	pre	maang4	Object	Contrastive	r2	300.5072064	4	1	on_focus	4	Contrastive on_focus
2023405	block2	Control	pre	Jan-04	Object	Contrastive	r2	334.0273422	5	2	on_focus	4	Contrastive on_focus
2023405	block2	Control	pre	ma4	Subject	Contrastive	r2	234.4607073	1	1	pre_focus	4	Contrastive pre_focus
2023405	block2	Control	pre	ma4	Subject	Contrastive	r2	198.0186445	2	2	pre_focus	4	Contrastive pre_focus
2023405	block2	Control	pre	fu4	Verb	Contrastive	r2	130.1100347	3	1	on_focus	4	Contrastive on_focus
2023405	block2	Control	pre	maang4	Object	Contrastive	r2	266.0996444	4	1	post_focus	4	Contrastive post_focus
2023405	block2	Control	pre	Jan-04	Object	Contrastive	r2	110.4154901	5	2	post_focus	4	Contrastive post_focus
2023405	block2	Control	pre	ngaa5	Subject	Contrastive	r2	337.3987052	1	1	pre_focus	5	Contrastive pre_focus
2023405	block2	Control	pre	ngaa5	Subject	Contrastive	r2	210.7690404	2	2	pre_focus	5	Contrastive pre_focus
2023405	block2	Control	pre	maai5	Verb	Contrastive	r2	282.5647398	3	1	pre_focus	5	Contrastive pre_focus
2023405	block2	Control	pre	pou5	Object	Contrastive	r2	204.1536293	4	1	on_focus	5	Contrastive on_focus
2023405	block2	Control	pre	pou5	Object	Contrastive	r2	260.580692	5	2	on_focus	5	Contrastive on_focus
2023405	block2	Control	pre	lok6	Subject	Contrastive	r2	158.9215282	1	1	pre_focus	6	Contrastive pre_focus
2023405	block2	Control	pre	lok6	Subject	Contrastive	r2	187.2189246	2	2	pre_focus	6	Contrastive pre_focus
2023405	block2	Control	pre	waa6	Verb	Contrastive	r2	218.223145	3	1	on_focus	6	Contrastive on_focus
2023405	block2	Control	pre	jyut6	Object	Contrastive	r2	90.89527169	4	1	post_focus	6	Contrastive post_focus
2023405	block2	Control	pre	loeng6	Object	Contrastive	r2	393.1952175	5	2	post_focus	6	Contrastive post_focus
2023405	block2	Control	pre	ngaa5	Subject	Contrastive	r2	385.8048375	1	1	on_focus	5	Contrastive on_focus
2023405	block2	Control	pre	ngaa5	Subject	Contrastive	r2	156.0455128	2	2	on_focus	5	Contrastive on_focus

2023405	block2	Control	pre	maai5	Verb	Contrastive	r2	295.1954899	3	1	post_focus	5	Contrastive post_focus
2023405	block2	Control	pre	pou5	Object	Contrastive	r2	113.74204	4	1	post_focus	5	Contrastive post_focus
2023405	block2	Control	pre	pou5	Object	Contrastive	r2	261.852724	5	2	post_focus	5	Contrastive post_focus
2023405	block2	Control	pre	lok6	Subject	Contrastive	r2	180.5734442	1	1	pre_focus	6	Contrastive pre_focus
2023405	block2	Control	pre	lok6	Subject	Contrastive	r2	100.3243452	2	2	pre_focus	6	Contrastive pre_focus
2023405	block2	Control	pre	waa6	Verb	Contrastive	r2	179.775929	3	1	pre_focus	6	Contrastive pre_focus
2023405	block2	Control	pre	jyut6	Object	Contrastive	r2	82.72617594	4	1	on_focus	6	Contrastive on_focus
2023405	block2	Control	pre	loeng6	Object	Contrastive	r2	421.5500361	5	2	on_focus	6	Contrastive on_focus
2023405	block2	Control	pre	ma4	Subject	Contrastive	r2	308.6198948	1	1	on_focus	4	Contrastive on_focus
2023405	block2	Control	pre	ma4	Subject	Contrastive	r2	260.3215232	2	2	on_focus	4	Contrastive on_focus
2023405	block2	Control	pre	fu4	Verb	Contrastive	r2	187.1699348	3	1	post_focus	4	Contrastive post_focus
2023405	block2	Control	pre	maang4	Object	Contrastive	r2	290.9306145	4	1	post_focus	4	Contrastive post_focus
2023405	block2	Control	pre	Jan-04	Object	Contrastive	r2	187.6533447	5	2	post_focus	4	Contrastive post_focus
2023405	block2	Control	pre	lok6	Subject	Contrastive	r2	104.2513652	1	1	on_focus	6	Contrastive on_focus
2023405	block2	Control	pre	lok6	Subject	Contrastive	r2	143.3163559	2	2	on_focus	6	Contrastive on_focus
2023405	block2	Control	pre	waa6	Verb	Contrastive	r2	146.9083598	3	1	post_focus	6	Contrastive post_focus
2023405	block2	Control	pre	jyut6	Object	Contrastive	r2	110.5636154	4	1	post_focus	6	Contrastive post_focus
2023405	block2	Control	pre	loeng6	Object	Contrastive	r2	413.6280099	5	2	post_focus	6	Contrastive post_focus
2023405	block2	Control	pre	ma4	Subject	Narrow	r2	186.1151127	1	1	pre_focus	4	Narrow pre_focus
2023405	block2	Control	pre	ma4	Subject	Narrow	r2	209.7340525	2	2	pre_focus	4	Narrow pre_focus
2023405	block2	Control	pre	fu4	Verb	Narrow	r2	178.9687709	3	1	on_focus	4	Narrow on_focus
2023405	block2	Control	pre	maang4	Object	Narrow	r2	292.6126736	4	1	post_focus	4	Narrow post_focus
2023405	block2	Control	pre	Jan-04	Object	Narrow	r2	210.688219	5	2	post_focus	4	Narrow post_focus
2023405	block2	Control	pre	lok6	Subject	Narrow	r2	152.8217744	1	1	pre_focus	6	Narrow pre_focus
2023405	block2	Control	pre	lok6	Subject	Narrow	r2	133.0199477	2	2	pre_focus	6	Narrow pre_focus
2023405	block2	Control	pre	waa6	Verb	Narrow	r2	207.7376165	3	1	on_focus	6	Narrow on_focus
2023405	block2	Control	pre	jyut6	Object	Narrow	r2	131.4156487	4	1	post_focus	6	Narrow post_focus
2023405	block2	Control	pre	loeng6	Object	Narrow	r2	410.8472278	5	2	post_focus	6	Narrow post_focus
2023405	block2	Control	pre	ngaa5	Subject	Broad	r2	331.4891704	1	1	broad_focus	5	Broad focus
2023405	block2	Control	pre	ngaa5	Subject	Broad	r2	279.2232824	2	2	broad_focus	5	Broad focus
2023405	block2	Control	pre	maai5	Verb	Broad	r2	275.0190608	3	1	broad_focus	5	Broad focus
2023405	block2	Control	pre	pou5	Object	Broad	r2	160.1336985	4	1	broad_focus	5	Broad focus
2023405	block2	Control	pre	pou5	Object	Broad	r2	159.9721277	5	2	broad_focus	5	Broad focus
2023405	block3	Control	post	bui3	Subject	Contrastive	r1	171.045829	1	1	pre_focus	3	Contrastive pre_focus
2023405	block3	Control	post	bui3	Subject	Contrastive	r1	184.4932999	2	2	pre_focus	3	Contrastive pre_focus
2023405	block3	Control	post	tsv1	Verb	Contrastive	r1	153.0973521	3	1	pre_focus	1	Contrastive pre_focus
2023405	block3	Control	post	fug1	Object	Contrastive	r1	224.8635726	4	1	on_focus	1	Contrastive on_focus
2023405	block3	Control	post	tshe1	Object	Contrastive	r1	308.9309846	5	2	on_focus	1	Contrastive on_focus
2023405	block3	Control	post	suk1	Subject	Narrow	r1	64.70112899	1	1	on_focus	1	Narrow on_focus
2023405	block3	Control	post	suk1	Subject	Narrow	r1	85.55165868	2	2	on_focus	1	Narrow on_focus
2023405	block3	Control	post	sei2	Verb	Narrow	r1	181.032255	3	1	post_focus	2	Narrow post_focus
2023405	block3	Control	post	svy2	Object	Narrow	r1	186.3793675	4	1	post_focus	2	Narrow post_focus
2023405	block3	Control	post	kwo2	Object	Narrow	r1	255.6645291	5	2	post_focus	2	Narrow post_focus
2023405	block3	Control	post	bui3	Subject	Contrastive	r1	157.2044783	1	1	pre_focus	3	Contrastive pre_focus
2023405	block3	Control	post	bui3	Subject	Contrastive	r1	93.99219771	2	2	pre_focus	3	Contrastive pre_focus
2023405	block3	Control	post	tsv1	Verb	Contrastive	r1	105.7878987	3	1	on_focus	1	Contrastive on_focus
2023405	block3	Control	post	fug1	Object	Contrastive	r1	149.2459486	4	1	post_focus	1	Contrastive post_focus
2023405	block3	Control	post	tshe1	Object	Contrastive	r1	217.8898282	5	2	post_focus	1	Contrastive post_focus
2023405	block3	Control	post	piu2	Subject	Narrow	r1	128.8399033	1	1	pre_focus	2	Narrow pre_focus
2023405	block3	Control	post	tse2	Subject	Narrow	r1	123.8063716	2	2	pre_focus	2	Narrow pre_focus
2023405	block3	Control	post	tsap1	Verb	Narrow	r1	118.4811475	3	1	pre_focus	1	Narrow pre_focus
2023405	block3	Control	post	sy1	Object	Narrow	r1	135.2848926	4	1	on_focus	1	Narrow on_focus
2023405	block3	Control	post	pau1	Object	Narrow	r1	305.0030359	5	2	on_focus	1	Narrow on_focus
2023405	block3	Control	post	piu2	Subject	Contrastive	r1	142.9767037	1	1	pre_focus	2	Contrastive pre_focus
2023405	block3	Control	post	tse2	Subject	Contrastive	r1	114.7251456	2	2	pre_focus	2	Contrastive pre_focus
2023405	block3	Control	post	tsap1	Verb	Contrastive	r1	77.71906594	3	1	pre_focus	1	Contrastive pre_focus
2023405	block3	Control	post	sy1	Object	Contrastive	r1	146.8621613	4	1	on_focus	1	Contrastive on_focus
2023405	block3	Control	post	pau1	Object	Contrastive	r1	309.7978101	5	2	on_focus	1	Contrastive on_focus
2023405	block3	Control	post	bui3	Subject	Broad	r1	143.3617642	1	1	broad_focus	3	Broad focus
2023405	block3	Control	post	bui3	Subject	Broad	r1	146.9994491	2	2	broad_focus	3	Broad focus
2023405	block3	Control	post	tsv1	Verb	Broad	r1	106.7265715	3	1	broad_focus	1	Broad focus
2023405	block3	Control	post	fug1	Object	Broad	r1	201.0713972	4	1	broad_focus	1	Broad focus
2023405	block3	Control	post	tshe1	Object	Broad	r1	220.1669686	5	2	broad_focus	1	Broad focus
2023405	block3	Control	post	piu2	Subject	Contrastive	r1	160.5928545	1	1	on_focus	2	Contrastive on_focus
2023405	block3	Control	post	tse2	Subject	Contrastive	r1	152.50143	2	2	on_focus	2	Contrastive on_focus
2023405	block3	Control	post	tsap1	Verb	Contrastive	r1	79.78523586	3	1	post_focus	1	Contrastive post_focus

**2023405**	block3	Control	post	sy1	Object	Contrastive	r1	131.0944655	4	1	post_focus	1	Contrastive post_focus
**2023405**	block3	Control	post	pau1	Object	Contrastive	r1	300.873366	5	2	post_focus	1	Contrastive post_focus
**2023405**	block3	Control	post	bui3	Subject	Narrow	r1	144.9944235	1	1	pre_focus	3	Narrow pre_focus
**2023405**	block3	Control	post	bui3	Subject	Narrow	r1	122.6736969	2	2	pre_focus	3	Narrow pre_focus
**2023405**	block3	Control	post	tsv1	Verb	Narrow	r1	141.9851077	3	1	on_focus	1	Narrow on_focus
**2023405**	block3	Control	post	fug1	Object	Narrow	r1	183.276261	4	1	post_focus	1	Narrow post_focus
**2023405**	block3	Control	post	tshe1	Object	Narrow	r1	238.587803	5	2	post_focus	1	Narrow post_focus
**2023405**	block3	Control	post	piu2	Subject	Narrow	r1	212.9677425	1	1	on_focus	2	Narrow on_focus
**2023405**	block3	Control	post	tse2	Subject	Narrow	r1	139.5262267	2	2	on_focus	2	Narrow on_focus
**2023405**	block3	Control	post	tsap1	Verb	Narrow	r1	127.2425414	3	1	post_focus	1	Narrow post_focus
**2023405**	block3	Control	post	sy1	Object	Narrow	r1	121.3799296	4	1	post_focus	1	Narrow post_focus
**2023405**	block3	Control	post	pau1	Object	Narrow	r1	317.1321372	5	2	post_focus	1	Narrow post_focus
**2023405**	block3	Control	post	piu2	Subject	Broad	r1	170.1100772	1	1	broad_focus	2	Broad focus
**2023405**	block3	Control	post	tse2	Subject	Broad	r1	137.8100335	2	2	broad_focus	2	Broad focus
**2023405**	block3	Control	post	tsap1	Verb	Broad	r1	163.8450167	3	1	broad_focus	1	Broad focus
**2023405**	block3	Control	post	sy1	Object	Broad	r1	135.4141699	4	1	broad_focus	1	Broad focus
**2023405**	block3	Control	post	pau1	Object	Broad	r1	284.0114834	5	2	broad_focus	1	Broad focus
**2023405**	block3	Control	post	bui3	Subject	Narrow	r1	146.8189369	1	1	on_focus	3	Narrow on_focus
**2023405**	block3	Control	post	bui3	Subject	Narrow	r1	134.5114358	2	2	on_focus	3	Narrow on_focus
**2023405**	block3	Control	post	tsv1	Verb	Narrow	r1	120.9919427	3	1	post_focus	1	Narrow post_focus
**2023405**	block3	Control	post	fug1	Object	Narrow	r1	227.1793235	4	1	post_focus	1	Narrow post_focus
**2023405**	block3	Control	post	tshe1	Object	Narrow	r1	214.6250282	5	2	post_focus	1	Narrow post_focus
**2023405**	block3	Control	post	suk1	Subject	Narrow	r1	93.25956418	1	1	pre_focus	1	Narrow pre_focus
**2023405**	block3	Control	post	suk1	Subject	Narrow	r1	120.6264473	2	2	pre_focus	1	Narrow pre_focus
**2023405**	block3	Control	post	sei2	Verb	Narrow	r1	158.5981887	3	1	on_focus	2	Narrow on_focus
**2023405**	block3	Control	post	svy2	Object	Narrow	r1	159.2011111	4	1	post_focus	2	Narrow post_focus
**2023405**	block3	Control	post	kwo2	Object	Narrow	r1	302.9860767	5	2	post_focus	2	Narrow post_focus
**2023405**	block3	Control	post	suk1	Subject	Narrow	r1	87.98152067	1	1	pre_focus	1	Narrow pre_focus
**2023405**	block3	Control	post	suk1	Subject	Narrow	r1	114.1031738	2	2	pre_focus	1	Narrow pre_focus
**2023405**	block3	Control	post	sei2	Verb	Narrow	r1	122.1624774	3	1	pre_focus	2	Narrow pre_focus
**2023405**	block3	Control	post	svy2	Object	Narrow	r1	158.6535356	4	1	on_focus	2	Narrow on_focus
**2023405**	block3	Control	post	kwo2	Object	Narrow	r1	254.6872563	5	2	on_focus	2	Narrow on_focus
**2023405**	block3	Control	post	piu2	Subject	Narrow	r1	155.7275153	1	1	pre_focus	2	Narrow pre_focus
**2023405**	block3	Control	post	tse2	Subject	Narrow	r1	137.6755641	2	2	pre_focus	2	Narrow pre_focus
**2023405**	block3	Control	post	tsap1	Verb	Narrow	r1	84.27257263	3	1	on_focus	1	Narrow on_focus
**2023405**	block3	Control	post	sy1	Object	Narrow	r1	122.7300076	4	1	post_focus	1	Narrow post_focus
**2023405**	block3	Control	post	pau1	Object	Narrow	r1	289.5426814	5	2	post_focus	1	Narrow post_focus
**2023405**	block3	Control	post	bui3	Subject	Narrow	r1	142.5824827	1	1	pre_focus	3	Narrow pre_focus
**2023405**	block3	Control	post	bui3	Subject	Narrow	r1	125.3840138	2	2	pre_focus	3	Narrow pre_focus
**2023405**	block3	Control	post	tsv1	Verb	Narrow	r1	152.3928285	3	1	pre_focus	1	Narrow pre_focus
**2023405**	block3	Control	post	fug1	Object	Narrow	r1	174.6026084	4	1	on_focus	1	Narrow on_focus
**2023405**	block3	Control	post	tshe1	Object	Narrow	r1	214.8721873	5	2	on_focus	1	Narrow on_focus
**2023405**	block3	Control	post	suk1	Subject	Contrastive	r1	99.63010509	1	1	on_focus	1	Contrastive on_focus
**2023405**	block3	Control	post	suk1	Subject	Contrastive	r1	92.33484788	2	2	on_focus	1	Contrastive on_focus
**2023405**	block3	Control	post	sei2	Verb	Contrastive	r1	170.5484026	3	1	post_focus	2	Contrastive post_focus
**2023405**	block3	Control	post	svy2	Object	Contrastive	r1	172.4477344	4	1	post_focus	2	Contrastive post_focus
**2023405**	block3	Control	post	kwo2	Object	Contrastive	r1	285.7526146	5	2	post_focus	2	Contrastive post_focus
**2023405**	block3	Control	post	suk1	Subject	Contrastive	r1	96.94763858	1	1	pre_focus	1	Contrastive pre_focus
**2023405**	block3	Control	post	suk1	Subject	Contrastive	r1	112.8098097	2	2	pre_focus	1	Contrastive pre_focus
**2023405**	block3	Control	post	sei2	Verb	Contrastive	r1	132.6622006	3	1	pre_focus	2	Contrastive pre_focus
**2023405**	block3	Control	post	svy2	Object	Contrastive	r1	126.6473339	4	1	on_focus	2	Contrastive on_focus
**2023405**	block3	Control	post	kwo2	Object	Contrastive	r1	261.8720009	5	2	on_focus	2	Contrastive on_focus
**2023405**	block3	Control	post	bui3	Subject	Contrastive	r1	123.5282522	1	1	on_focus	3	Contrastive on_focus
**2023405**	block3	Control	post	bui3	Subject	Contrastive	r1	145.5502602	2	2	on_focus	3	Contrastive on_focus
**2023405**	block3	Control	post	tsv1	Verb	Contrastive	r1	129.7330892	3	1	post_focus	1	Contrastive post_focus
**2023405**	block3	Control	post	fug1	Object	Contrastive	r1	157.2689607	4	1	post_focus	1	Contrastive post_focus
**2023405**	block3	Control	post	tshe1	Object	Contrastive	r1	233.885737	5	2	post_focus	1	Contrastive post_focus
**2023405**	block3	Control	post	suk1	Subject	Contrastive	r1	107.5480727	1	1	pre_focus	1	Contrastive pre_focus
**2023405**	block3	Control	post	suk1	Subject	Contrastive	r1	141.7168188	2	2	pre_focus	1	Contrastive pre_focus
**2023405**	block3	Control	post	sei2	Verb	Contrastive	r1	144.8081133	3	1	on_focus	2	Contrastive on_focus
**2023405**	block3	Control	post	svy2	Object	Contrastive	r1	179.1539499	4	1	post_focus	2	Contrastive post_focus
**2023405**	block3	Control	post	kwo2	Object	Contrastive	r1	237.1368436	5	2	post_focus	2	Contrastive post_focus
**2023405**	block3	Control	post	suk1	Subject	Broad	r1	98.74691013	1	1	broad_focus	1	Broad focus
**2023405**	block3	Control	post	suk1	Subject	Broad	r1	62.53582835	2	2	broad_focus	1	Broad focus
**2023405**	block3	Control	post	sei2	Verb	Broad	r1	164.6961312	3	1	broad_focus	2	Broad focus
**2023405**	block3	Control	post	svy2	Object	Broad	r1	169.6621967	4	1	broad_focus	2	Broad focus

**2023405**	block3	Control	post	kwo2	Object	Broad	r1	262.5518282	5	2	broad_focus	2	Broad focus
**2023405**	block3	Control	post	piu2	Subject	Contrastive	r1	117.0297656	1	1	pre_focus	2	Contrastive pre_focus
**2023405**	block3	Control	post	tse2	Subject	Contrastive	r1	129.9371089	2	2	pre_focus	2	Contrastive pre_focus
**2023405**	block3	Control	post	tsap1	Verb	Contrastive	r1	120.5632899	3	1	on_focus	1	Contrastive on_focus
**2023405**	block3	Control	post	sy1	Object	Contrastive	r1	121.956705	4	1	post_focus	1	Contrastive post_focus
**2023405**	block3	Control	post	pau1	Object	Contrastive	r1	251.0213581	5	2	post_focus	1	Contrastive post_focus
**2023405**	block3	Control	post	suk1	Subject	Contrastive	r2	86.08179893	1	1	pre_focus	1	Contrastive pre_focus
**2023405**	block3	Control	post	suk1	Subject	Contrastive	r2	112.2681745	2	2	pre_focus	1	Contrastive pre_focus
**2023405**	block3	Control	post	sei2	Verb	Contrastive	r2	159.8537512	3	1	pre_focus	2	Contrastive pre_focus
**2023405**	block3	Control	post	svy2	Object	Contrastive	r2	146.0477434	4	1	on_focus	2	Contrastive on_focus
**2023405**	block3	Control	post	kwo2	Object	Contrastive	r2	213.7299038	5	2	on_focus	2	Contrastive on_focus
**2023405**	block3	Control	post	suk1	Subject	Broad	r2	82.4119557	1	1	broad_focus	1	Broad focus
**2023405**	block3	Control	post	suk1	Subject	Broad	r2	84.32971686	2	2	broad_focus	1	Broad focus
**2023405**	block3	Control	post	sei2	Verb	Broad	r2	128.4507136	3	1	broad_focus	2	Broad focus
**2023405**	block3	Control	post	svy2	Object	Broad	r2	158.7275388	4	1	broad_focus	2	Broad focus
**2023405**	block3	Control	post	kwo2	Object	Broad	r2	244.7077939	5	2	broad_focus	2	Broad focus
**2023405**	block3	Control	post	piu2	Subject	Contrastive	r2	125.5655516	1	1	pre_focus	2	Contrastive pre_focus
**2023405**	block3	Control	post	tse2	Subject	Contrastive	r2	133.4290292	2	2	pre_focus	2	Contrastive pre_focus
**2023405**	block3	Control	post	tsap1	Verb	Contrastive	r2	80.21690797	3	1	pre_focus	1	Contrastive pre_focus
**2023405**	block3	Control	post	sy1	Object	Contrastive	r2	153.7884579	4	1	on_focus	1	Contrastive on_focus
**2023405**	block3	Control	post	pau1	Object	Contrastive	r2	274.1552966	5	2	on_focus	1	Contrastive on_focus
**2023405**	block3	Control	post	piu2	Subject	Contrastive	r2	180.0033142	1	1	on_focus	2	Contrastive on_focus
**2023405**	block3	Control	post	tse2	Subject	Contrastive	r2	126.5992705	2	2	on_focus	2	Contrastive on_focus
**2023405**	block3	Control	post	tsap1	Verb	Contrastive	r2	126.5069192	3	1	post_focus	1	Contrastive post_focus
**2023405**	block3	Control	post	sy1	Object	Contrastive	r2	175.9459029	4	1	post_focus	1	Contrastive post_focus
**2023405**	block3	Control	post	pau1	Object	Contrastive	r2	266.4645779	5	2	post_focus	1	Contrastive post_focus
**2023405**	block3	Control	post	bui3	Subject	Contrastive	r2	115.5141352	1	1	on_focus	3	Contrastive on_focus
**2023405**	block3	Control	post	bui3	Subject	Contrastive	r2	142.8104113	2	2	on_focus	3	Contrastive on_focus
**2023405**	block3	Control	post	tsv1	Verb	Contrastive	r2	131.0178975	3	1	post_focus	1	Contrastive post_focus
**2023405**	block3	Control	post	fug1	Object	Contrastive	r2	187.5598996	4	1	post_focus	1	Contrastive post_focus
**2023405**	block3	Control	post	tshe1	Object	Contrastive	r2	196.8207692	5	2	post_focus	1	Contrastive post_focus
**2023405**	block3	Control	post	bui3	Subject	Broad	r2	139.256271	1	1	broad_focus	3	Broad focus
**2023405**	block3	Control	post	bui3	Subject	Broad	r2	122.4265093	2	2	broad_focus	3	Broad focus
**2023405**	block3	Control	post	tsv1	Verb	Broad	r2	109.1270843	3	1	broad_focus	1	Broad focus
**2023405**	block3	Control	post	fug1	Object	Broad	r2	147.8385812	4	1	broad_focus	1	Broad focus
**2023405**	block3	Control	post	tshe1	Object	Broad	r2	198.026777	5	2	broad_focus	1	Broad focus
**2023405**	block3	Control	post	piu2	Subject	Contrastive	r2	105.4002197	1	1	pre_focus	2	Contrastive pre_focus
**2023405**	block3	Control	post	tse2	Subject	Contrastive	r2	126.353214	2	2	pre_focus	2	Contrastive pre_focus
**2023405**	block3	Control	post	tsap1	Verb	Contrastive	r2	95.07765196	3	1	on_focus	1	Contrastive on_focus
**2023405**	block3	Control	post	sy1	Object	Contrastive	r2	136.546328	4	1	post_focus	1	Contrastive post_focus
**2023405**	block3	Control	post	pau1	Object	Contrastive	r2	314.3893106	5	2	post_focus	1	Contrastive post_focus
**2023405**	block3	Control	post	suk1	Subject	Narrow	r2	86.7790701	1	1	on_focus	1	Narrow on_focus
**2023405**	block3	Control	post	suk1	Subject	Narrow	r2	131.7696492	2	2	on_focus	1	Narrow on_focus
**2023405**	block3	Control	post	sei2	Verb	Narrow	r2	136.6877828	3	1	post_focus	2	Narrow post_focus
**2023405**	block3	Control	post	svy2	Object	Narrow	r2	152.8240771	4	1	post_focus	2	Narrow post_focus
**2023405**	block3	Control	post	kwo2	Object	Narrow	r2	271.7629125	5	2	post_focus	2	Narrow post_focus
**2023405**	block3	Control	post	suk1	Subject	Narrow	r2	82.75929839	1	1	pre_focus	1	Narrow pre_focus
**2023405**	block3	Control	post	suk1	Subject	Narrow	r2	81.84678851	2	2	pre_focus	1	Narrow pre_focus
**2023405**	block3	Control	post	sei2	Verb	Narrow	r2	121.2725938	3	1	pre_focus	2	Narrow pre_focus
**2023405**	block3	Control	post	svy2	Object	Narrow	r2	106.3519313	4	1	on_focus	2	Narrow on_focus
**2023405**	block3	Control	post	kwo2	Object	Narrow	r2	269.161137	5	2	on_focus	2	Narrow on_focus
**2023405**	block3	Control	post	piu2	Subject	Broad	r2	102.1377719	1	1	broad_focus	2	Broad focus
**2023405**	block3	Control	post	tse2	Subject	Broad	r2	129.2050116	2	2	broad_focus	2	Broad focus
**2023405**	block3	Control	post	tsap1	Verb	Broad	r2	112.5138363	3	1	broad_focus	1	Broad focus
**2023405**	block3	Control	post	sy1	Object	Broad	r2	128.4302318	4	1	broad_focus	1	Broad focus
**2023405**	block3	Control	post	pau1	Object	Broad	r2	290.4652997	5	2	broad_focus	1	Broad focus
**2023405**	block3	Control	post	bui3	Subject	Contrastive	r2	106.9562655	1	1	pre_focus	3	Contrastive pre_focus
**2023405**	block3	Control	post	bui3	Subject	Contrastive	r2	113.6047848	2	2	pre_focus	3	Contrastive pre_focus
**2023405**	block3	Control	post	tsv1	Verb	Contrastive	r2	140.9540809	3	1	on_focus	1	Contrastive on_focus
**2023405**	block3	Control	post	fug1	Object	Contrastive	r2	157.5403418	4	1	post_focus	1	Contrastive post_focus
**2023405**	block3	Control	post	tshe1	Object	Contrastive	r2	201.2003496	5	2	post_focus	1	Contrastive post_focus
**2023405**	block3	Control	post	suk1	Subject	Contrastive	r2	93.72792279	1	1	pre_focus	1	Contrastive pre_focus
**2023405**	block3	Control	post	suk1	Subject	Contrastive	r2	86.2343347	2	2	pre_focus	1	Contrastive pre_focus
**2023405**	block3	Control	post	sei2	Verb	Contrastive	r2	157.8282478	3	1	on_focus	2	Contrastive on_focus
**2023405**	block3	Control	post	svy2	Object	Contrastive	r2	184.3599707	4	1	post_focus	2	Contrastive post_focus
**2023405**	block3	Control	post	kwo2	Object	Contrastive	r2	268.210729	5	2	post_focus	2	Contrastive post_focus

2023405	block3	Control	post	bui3	Subject	Contrastive	r2	95.54620798	1	1	pre_focus	3	Contrastive pre_focus
2023405	block3	Control	post	bui3	Subject	Contrastive	r2	119.4783274	2	2	pre_focus	3	Contrastive pre_focus
2023405	block3	Control	post	tsv1	Verb	Contrastive	r2	179.4632938	3	1	pre_focus	1	Contrastive pre_focus
2023405	block3	Control	post	fug1	Object	Contrastive	r2	180.2734843	4	1	on_focus	1	Contrastive on_focus
2023405	block3	Control	post	tshe1	Object	Contrastive	r2	254.4159979	5	2	on_focus	1	Contrastive on_focus
2023405	block3	Control	post	piu2	Subject	Narrow	r2	105.3983616	1	1	pre_focus	2	Narrow pre_focus
2023405	block3	Control	post	tse2	Subject	Narrow	r2	124.9980652	2	2	pre_focus	2	Narrow pre_focus
2023405	block3	Control	post	tsap1	Verb	Narrow	r2	108.1021356	3	1	pre_focus	1	Narrow pre_focus
2023405	block3	Control	post	sy1	Object	Narrow	r2	133.1336669	4	1	on_focus	1	Narrow on_focus
2023405	block3	Control	post	pau1	Object	Narrow	r2	257.0963934	5	2	on_focus	1	Narrow on_focus
2023405	block3	Control	post	suk1	Subject	Contrastive	r2	96.42257267	1	1	on_focus	1	Contrastive on_focus
2023405	block3	Control	post	suk1	Subject	Contrastive	r2	124.5235476	2	2	on_focus	1	Contrastive on_focus
2023405	block3	Control	post	sei2	Verb	Contrastive	r2	130.61356	3	1	post_focus	2	Contrastive post_focus
2023405	block3	Control	post	svy2	Object	Contrastive	r2	141.9045948	4	1	post_focus	2	Contrastive post_focus
2023405	block3	Control	post	kwo2	Object	Contrastive	r2	256.9645344	5	2	post_focus	2	Contrastive post_focus
2023405	block3	Control	post	bui3	Subject	Narrow	r2	98.82653993	1	1	pre_focus	3	Narrow pre_focus
2023405	block3	Control	post	bui3	Subject	Narrow	r2	140.9618493	2	2	pre_focus	3	Narrow pre_focus
2023405	block3	Control	post	tsv1	Verb	Narrow	r2	100.2011462	3	1	on_focus	1	Narrow on_focus
2023405	block3	Control	post	fug1	Object	Narrow	r2	168.7566433	4	1	post_focus	1	Narrow post_focus
2023405	block3	Control	post	tshe1	Object	Narrow	r2	187.8429588	5	2	post_focus	1	Narrow post_focus
2023405	block3	Control	post	bui3	Subject	Narrow	r2	124.4827403	1	1	on_focus	3	Narrow on_focus
2023405	block3	Control	post	bui3	Subject	Narrow	r2	135.9195344	2	2	on_focus	3	Narrow on_focus
2023405	block3	Control	post	tsv1	Verb	Narrow	r2	105.1786198	3	1	post_focus	1	Narrow post_focus
2023405	block3	Control	post	fug1	Object	Narrow	r2	150.0183482	4	1	post_focus	1	Narrow post_focus
2023405	block3	Control	post	tshe1	Object	Narrow	r2	227.0031668	5	2	post_focus	1	Narrow post_focus
2023405	block3	Control	post	piu2	Subject	Narrow	r2	87.963228	1	1	pre_focus	2	Narrow pre_focus
2023405	block3	Control	post	tse2	Subject	Narrow	r2	133.0305596	2	2	pre_focus	2	Narrow pre_focus
2023405	block3	Control	post	tsap1	Verb	Narrow	r2	91.55173061	3	1	on_focus	1	Narrow on_focus
2023405	block3	Control	post	sy1	Object	Narrow	r2	113.4669753	4	1	post_focus	1	Narrow post_focus
2023405	block3	Control	post	pau1	Object	Narrow	r2	257.3063332	5	2	post_focus	1	Narrow post_focus
2023405	block3	Control	post	suk1	Subject	Narrow	r2	89.89941687	1	1	pre_focus	1	Narrow pre_focus
2023405	block3	Control	post	suk1	Subject	Narrow	r2	116.6734216	2	2	pre_focus	1	Narrow pre_focus
2023405	block3	Control	post	sei2	Verb	Narrow	r2	162.5764405	3	1	on_focus	2	Narrow on_focus
2023405	block3	Control	post	svy2	Object	Narrow	r2	134.9133349	4	1	post_focus	2	Narrow post_focus
2023405	block3	Control	post	kwo2	Object	Narrow	r2	248.4314601	5	2	post_focus	2	Narrow post_focus
2023405	block3	Control	post	bui3	Subject	Narrow	r2	113.8477659	1	1	pre_focus	3	Narrow pre_focus
2023405	block3	Control	post	bui3	Subject	Narrow	r2	105.8658376	2	2	pre_focus	3	Narrow pre_focus
2023405	block3	Control	post	tsv1	Verb	Narrow	r2	94.26362986	3	1	pre_focus	1	Narrow pre_focus
2023405	block3	Control	post	fug1	Object	Narrow	r2	167.4805568	4	1	on_focus	1	Narrow on_focus
2023405	block3	Control	post	tshe1	Object	Narrow	r2	223.8285355	5	2	on_focus	1	Narrow on_focus
2023405	block3	Control	post	piu2	Subject	Narrow	r2	131.3332137	1	1	on_focus	2	Narrow on_focus
2023405	block3	Control	post	tse2	Subject	Narrow	r2	118.240243	2	2	on_focus	2	Narrow on_focus
2023405	block3	Control	post	tsap1	Verb	Narrow	r2	78.16202664	3	1	post_focus	1	Narrow post_focus
2023405	block3	Control	post	sy1	Object	Narrow	r2	129.7956031	4	1	post_focus	1	Narrow post_focus
2023405	block3	Control	post	pau1	Object	Narrow	r2	213.0280283	5	2	post_focus	1	Narrow post_focus
2023405	block3	Control	pre	piu2	Subject	Contrastive	r1	233.2114187	1	1	on_focus	2	Contrastive on_focus
2023405	block3	Control	pre	tse2	Subject	Contrastive	r1	178.8035512	2	2	on_focus	2	Contrastive on_focus
2023405	block3	Control	pre	tsap1	Verb	Contrastive	r1	77.45793278	3	1	post_focus	1	Contrastive post_focus
2023405	block3	Control	pre	sy1	Object	Contrastive	r1	248.561521	4	1	post_focus	1	Contrastive post_focus
2023405	block3	Control	pre	pau1	Object	Contrastive	r1	531.4156907	5	2	post_focus	1	Contrastive post_focus
2023405	block3	Control	pre	piu2	Subject	Narrow	r1	367.3840427	1	1	pre_focus	2	Narrow pre_focus
2023405	block3	Control	pre	tse2	Subject	Narrow	r1	411.2874088	2	2	pre_focus	2	Narrow pre_focus
2023405	block3	Control	pre	tsap1	Verb	Narrow	r1	175.210086	3	1	pre_focus	1	Narrow pre_focus
2023405	block3	Control	pre	sy1	Object	Narrow	r1	377.0762317	4	1	on_focus	1	Narrow on_focus
2023405	block3	Control	pre	pau1	Object	Narrow	r1	553.0520334	5	2	on_focus	1	Narrow on_focus
2023405	block3	Control	pre	bui3	Subject	Contrastive	r1	192.4877881	1	1	on_focus	3	Contrastive on_focus
2023405	block3	Control	pre	bui3	Subject	Contrastive	r1	202.5900166	2	2	on_focus	3	Contrastive on_focus
2023405	block3	Control	pre	tsv1	Verb	Contrastive	r1	159.0718065	3	1	post_focus	1	Contrastive post_focus
2023405	block3	Control	pre	fug1	Object	Contrastive	r1	214.0400065	4	1	post_focus	1	Contrastive post_focus
2023405	block3	Control	pre	tshe1	Object	Contrastive	r1	302.0785071	5	2	post_focus	1	Contrastive post_focus
2023405	block3	Control	pre	piu2	Subject	Contrastive	r1	208.5827267	1	1	pre_focus	2	Contrastive pre_focus
2023405	block3	Control	pre	tse2	Subject	Contrastive	r1	114.7303207	2	2	pre_focus	2	Contrastive pre_focus
2023405	block3	Control	pre	tsap1	Verb	Contrastive	r1	117.1023108	3	1	pre_focus	1	Contrastive pre_focus
2023405	block3	Control	pre	sy1	Object	Contrastive	r1	123.5966064	4	1	on_focus	1	Contrastive on_focus
2023405	block3	Control	pre	pau1	Object	Contrastive	r1	358.0768168	5	2	on_focus	1	Contrastive on_focus
2023405	block3	Control	pre	suk1	Subject	Narrow	r1	64.82575486	1	1	on_focus	1	Narrow on_focus

**2023405**	block3	Control	pre	suk1	Subject	Narrow	r1	96.75392933	2	2	on_focus	1	Narrow on_focus
**2023405**	block3	Control	pre	sei2	Verb	Narrow	r1	151.57928	3	1	post_focus	2	Narrow post_focus
**2023405**	block3	Control	pre	svy2	Object	Narrow	r1	178.7301587	4	1	post_focus	2	Narrow post_focus
**2023405**	block3	Control	pre	kwo2	Object	Narrow	r1	83.69308191	5	2	post_focus	2	Narrow post_focus
**2023405**	block3	Control	pre	bui3	Subject	Contrastive	r1	152.2532292	1	1	pre_focus	3	Contrastive pre_focus
**2023405**	block3	Control	pre	bui3	Subject	Contrastive	r1	158.515711	2	2	pre_focus	3	Contrastive pre_focus
**2023405**	block3	Control	pre	tsv1	Verb	Contrastive	r1	152.1631033	3	1	on_focus	1	Contrastive on_focus
**2023405**	block3	Control	pre	fug1	Object	Contrastive	r1	207.7136723	4	1	post_focus	1	Contrastive post_focus
**2023405**	block3	Control	pre	tshe1	Object	Contrastive	r1	367.5452991	5	2	post_focus	1	Contrastive post_focus
**2023405**	block3	Control	pre	bui3	Subject	Narrow	r1	180.8219955	1	1	pre_focus	3	Narrow pre_focus
**2023405**	block3	Control	pre	bui3	Subject	Narrow	r1	197.0054689	2	2	pre_focus	3	Narrow pre_focus
**2023405**	block3	Control	pre	tsv1	Verb	Narrow	r1	138.667937	3	1	on_focus	1	Narrow on_focus
**2023405**	block3	Control	pre	fug1	Object	Narrow	r1	216.7791635	4	1	post_focus	1	Narrow post_focus
**2023405**	block3	Control	pre	tshe1	Object	Narrow	r1	344.1640752	5	2	post_focus	1	Narrow post_focus
**2023405**	block3	Control	pre	suk1	Subject	Narrow	r1	72.06330329	1	1	pre_focus	1	Narrow pre_focus
**2023405**	block3	Control	pre	suk1	Subject	Narrow	r1	73.57524955	2	2	pre_focus	1	Narrow pre_focus
**2023405**	block3	Control	pre	sei2	Verb	Narrow	r1	174.696577	3	1	pre_focus	2	Narrow pre_focus
**2023405**	block3	Control	pre	svy2	Object	Narrow	r1	250.9432325	4	1	on_focus	2	Narrow on_focus
**2023405**	block3	Control	pre	kwo2	Object	Narrow	r1	320.0129368	5	2	on_focus	2	Narrow on_focus
**2023405**	block3	Control	pre	piu2	Subject	Broad	r1	142.553099	1	1	broad_focus	2	Broad focus
**2023405**	block3	Control	pre	tse2	Subject	Broad	r1	141.6670541	2	2	broad_focus	2	Broad focus
**2023405**	block3	Control	pre	tsap1	Verb	Broad	r1	57.47455838	3	1	broad_focus	1	Broad focus
**2023405**	block3	Control	pre	sy1	Object	Broad	r1	179.238846	4	1	broad_focus	1	Broad focus
**2023405**	block3	Control	pre	pau1	Object	Broad	r1	523.3202832	5	2	broad_focus	1	Broad focus
**2023405**	block3	Control	pre	piu2	Subject	Narrow	r1	60.18709206	1	1	on_focus	2	Narrow on_focus
**2023405**	block3	Control	pre	tse2	Subject	Narrow	r1	141.6472224	2	2	on_focus	2	Narrow on_focus
**2023405**	block3	Control	pre	tsap1	Verb	Narrow	r1	56.7499695	3	1	post_focus	1	Narrow post_focus
**2023405**	block3	Control	pre	sy1	Object	Narrow	r1	170.8748967	4	1	post_focus	1	Narrow post_focus
**2023405**	block3	Control	pre	pau1	Object	Narrow	r1	152.0116983	5	2	post_focus	1	Narrow post_focus
**2023405**	block3	Control	pre	bui3	Subject	Contrastive	r1	446.0429092	1	1	pre_focus	3	Contrastive pre_focus
**2023405**	block3	Control	pre	bui3	Subject	Contrastive	r1	488.0236963	2	2	pre_focus	3	Contrastive pre_focus
**2023405**	block3	Control	pre	tsv1	Verb	Contrastive	r1	197.9832982	3	1	pre_focus	1	Contrastive pre_focus
**2023405**	block3	Control	pre	fug1	Object	Contrastive	r1	248.4093423	4	1	on_focus	1	Contrastive on_focus
**2023405**	block3	Control	pre	tshe1	Object	Contrastive	r1	370.7392245	5	2	on_focus	1	Contrastive on_focus
**2023405**	block3	Control	pre	suk1	Subject	Narrow	r1	91.44391137	1	1	pre_focus	1	Narrow pre_focus
**2023405**	block3	Control	pre	suk1	Subject	Narrow	r1	86.59634307	2	2	pre_focus	1	Narrow pre_focus
**2023405**	block3	Control	pre	sei2	Verb	Narrow	r1	178.4394016	3	1	on_focus	2	Narrow on_focus
**2023405**	block3	Control	pre	svy2	Object	Narrow	r1	219.3763647	4	1	post_focus	2	Narrow post_focus
**2023405**	block3	Control	pre	kwo2	Object	Narrow	r1	243.2377239	5	2	post_focus	2	Narrow post_focus
**2023405**	block3	Control	pre	suk1	Subject	Broad	r1	82.78584784	1	1	broad_focus	1	Broad focus
**2023405**	block3	Control	pre	suk1	Subject	Broad	r1	76.95043922	2	2	broad_focus	1	Broad focus
**2023405**	block3	Control	pre	sei2	Verb	Broad	r1	115.1881813	3	1	broad_focus	2	Broad focus
**2023405**	block3	Control	pre	svy2	Object	Broad	r1	159.8342408	4	1	broad_focus	2	Broad focus
**2023405**	block3	Control	pre	kwo2	Object	Broad	r1	63.42664807	5	2	broad_focus	2	Broad focus
**2023405**	block3	Control	pre	bui3	Subject	Broad	r1	153.9839911	1	1	broad_focus	3	Broad focus
**2023405**	block3	Control	pre	bui3	Subject	Broad	r1	170.2840337	2	2	broad_focus	3	Broad focus
**2023405**	block3	Control	pre	tsv1	Verb	Broad	r1	180.7633696	3	1	broad_focus	1	Broad focus
**2023405**	block3	Control	pre	fug1	Object	Broad	r1	234.2209388	4	1	broad_focus	1	Broad focus
**2023405**	block3	Control	pre	tshe1	Object	Broad	r1	369.641126	5	2	broad_focus	1	Broad focus
**2023405**	block3	Control	pre	suk1	Subject	Contrastive	r1	73.87850438	1	1	pre_focus	1	Contrastive pre_focus
**2023405**	block3	Control	pre	suk1	Subject	Contrastive	r1	150.9348717	2	2	pre_focus	1	Contrastive pre_focus
**2023405**	block3	Control	pre	sei2	Verb	Contrastive	r1	163.521762	3	1	pre_focus	2	Contrastive pre_focus
**2023405**	block3	Control	pre	svy2	Object	Contrastive	r1	197.6990407	4	1	on_focus	2	Contrastive on_focus
**2023405**	block3	Control	pre	kwo2	Object	Contrastive	r1	280.9975849	5	2	on_focus	2	Contrastive on_focus
**2023405**	block3	Control	pre	suk1	Subject	Contrastive	r1	76.17710213	1	1	on_focus	1	Contrastive on_focus
**2023405**	block3	Control	pre	suk1	Subject	Contrastive	r1	82.18482432	2	2	on_focus	1	Contrastive on_focus
**2023405**	block3	Control	pre	sei2	Verb	Contrastive	r1	149.728832	3	1	post_focus	2	Contrastive post_focus
**2023405**	block3	Control	pre	svy2	Object	Contrastive	r1	140.2855726	4	1	post_focus	2	Contrastive post_focus
**2023405**	block3	Control	pre	kwo2	Object	Contrastive	r1	119.6678506	5	2	post_focus	2	Contrastive post_focus
**2023405**	block3	Control	pre	piu2	Subject	Narrow	r1	210.3641631	1	1	pre_focus	2	Narrow pre_focus
**2023405**	block3	Control	pre	tse2	Subject	Narrow	r1	172.8404805	2	2	pre_focus	2	Narrow pre_focus
**2023405**	block3	Control	pre	tsap1	Verb	Narrow	r1	58.18522011	3	1	on_focus	1	Narrow on_focus
**2023405**	block3	Control	pre	sy1	Object	Narrow	r1	224.4551986	4	1	post_focus	1	Narrow post_focus
**2023405**	block3	Control	pre	pau1	Object	Narrow	r1	421.5954957	5	2	post_focus	1	Narrow post_focus
**2023405**	block3	Control	pre	suk1	Subject	Contrastive	r1	127.7945108	1	1	pre_focus	1	Contrastive pre_focus
**2023405**	block3	Control	pre	suk1	Subject	Contrastive	r1	57.85110606	2	2	pre_focus	1	Contrastive pre_focus

**2023405**	block3	Control	pre	sei2	Verb	Contrastive	r1	209.6695652	3	1	on_focus	2	Contrastive on_focus
**2023405**	block3	Control	pre	svy2	Object	Contrastive	r1	174.1424981	4	1	post_focus	2	Contrastive post_focus
**2023405**	block3	Control	pre	kwo2	Object	Contrastive	r1	107.6606814	5	2	post_focus	2	Contrastive post_focus
**2023405**	block3	Control	pre	bui3	Subject	Narrow	r1	165.5391918	1	1	on_focus	3	Narrow on_focus
**2023405**	block3	Control	pre	bui3	Subject	Narrow	r1	91.22404843	2	2	on_focus	3	Narrow on_focus
**2023405**	block3	Control	pre	tsv1	Verb	Narrow	r1	127.9598321	3	1	post_focus	1	Narrow post_focus
**2023405**	block3	Control	pre	fug1	Object	Narrow	r1	226.4581186	4	1	post_focus	1	Narrow post_focus
**2023405**	block3	Control	pre	tshe1	Object	Narrow	r1	347.5966476	5	2	post_focus	1	Narrow post_focus
**2023405**	block3	Control	pre	piu2	Subject	Contrastive	r1	188.5427421	1	1	pre_focus	2	Contrastive pre_focus
**2023405**	block3	Control	pre	tse2	Subject	Contrastive	r1	199.3275043	2	2	pre_focus	2	Contrastive pre_focus
**2023405**	block3	Control	pre	tsap1	Verb	Contrastive	r1	61.01768644	3	1	on_focus	1	Contrastive on_focus
**2023405**	block3	Control	pre	sy1	Object	Contrastive	r1	165.822295	4	1	post_focus	1	Contrastive post_focus
**2023405**	block3	Control	pre	pau1	Object	Contrastive	r1	460.4524613	5	2	post_focus	1	Contrastive post_focus
**2023405**	block3	Control	pre	bui3	Subject	Narrow	r1	205.0650104	1	1	pre_focus	3	Narrow pre_focus
**2023405**	block3	Control	pre	bui3	Subject	Narrow	r1	172.7056101	2	2	pre_focus	3	Narrow pre_focus
**2023405**	block3	Control	pre	tsv1	Verb	Narrow	r1	187.9171511	3	1	pre_focus	1	Narrow pre_focus
**2023405**	block3	Control	pre	fug1	Object	Narrow	r1	220.0874116	4	1	on_focus	1	Narrow on_focus
**2023405**	block3	Control	pre	tshe1	Object	Narrow	r1	456.6438491	5	2	on_focus	1	Narrow on_focus
**2023405**	block3	Control	pre	bui3	Subject	Contrastive	r2	66.67934898	1	1	pre_focus	3	Contrastive pre_focus
**2023405**	block3	Control	pre	bui3	Subject	Contrastive	r2	80.09434416	2	2	pre_focus	3	Contrastive pre_focus
**2023405**	block3	Control	pre	tsv1	Verb	Contrastive	r2	127.586424	3	1	pre_focus	1	Contrastive pre_focus
**2023405**	block3	Control	pre	fug1	Object	Contrastive	r2	153.3265383	4	1	on_focus	1	Contrastive on_focus
**2023405**	block3	Control	pre	tshe1	Object	Contrastive	r2	292.5045316	5	2	on_focus	1	Contrastive on_focus
**2023405**	block3	Control	pre	bui3	Subject	Contrastive	r2	99.76632494	1	1	pre_focus	3	Contrastive pre_focus
**2023405**	block3	Control	pre	bui3	Subject	Contrastive	r2	104.4479783	2	2	pre_focus	3	Contrastive pre_focus
**2023405**	block3	Control	pre	tsv1	Verb	Contrastive	r2	127.2856803	3	1	on_focus	1	Contrastive on_focus
**2023405**	block3	Control	pre	fug1	Object	Contrastive	r2	168.0480677	4	1	post_focus	1	Contrastive post_focus
**2023405**	block3	Control	pre	tshe1	Object	Contrastive	r2	298.8854599	5	2	post_focus	1	Contrastive post_focus
**2023405**	block3	Control	pre	suk1	Subject	Narrow	r2	63.47961929	1	1	on_focus	1	Narrow on_focus
**2023405**	block3	Control	pre	suk1	Subject	Narrow	r2	60.91033936	2	2	on_focus	1	Narrow on_focus
**2023405**	block3	Control	pre	sei2	Verb	Narrow	r2	83.43016351	3	1	post_focus	2	Narrow post_focus
**2023405**	block3	Control	pre	svy2	Object	Narrow	r2	231.0033064	4	1	post_focus	2	Narrow post_focus
**2023405**	block3	Control	pre	kwo2	Object	Narrow	r2	95.31591803	5	2	post_focus	2	Narrow post_focus
**2023405**	block3	Control	pre	piu2	Subject	Narrow	r2	61.13858058	1	1	on_focus	2	Narrow on_focus
**2023405**	block3	Control	pre	tse2	Subject	Narrow	r2	64.55467101	2	2	on_focus	2	Narrow on_focus
**2023405**	block3	Control	pre	tsap1	Verb	Narrow	r2	103.6137202	3	1	post_focus	1	Narrow post_focus
**2023405**	block3	Control	pre	sy1	Object	Narrow	r2	72.10618746	4	1	post_focus	1	Narrow post_focus
**2023405**	block3	Control	pre	pau1	Object	Narrow	r2	370.9929458	5	2	post_focus	1	Narrow post_focus
**2023405**	block3	Control	pre	piu2	Subject	Narrow	r2	116.0444062	1	1	pre_focus	2	Narrow pre_focus
**2023405**	block3	Control	pre	tse2	Subject	Narrow	r2	146.2114509	2	2	pre_focus	2	Narrow pre_focus
**2023405**	block3	Control	pre	tsap1	Verb	Narrow	r2	107.5419677	3	1	pre_focus	1	Narrow pre_focus
**2023405**	block3	Control	pre	sy1	Object	Narrow	r2	234.7994663	4	1	on_focus	1	Narrow on_focus
**2023405**	block3	Control	pre	pau1	Object	Narrow	r2	415.2896465	5	2	on_focus	1	Narrow on_focus
**2023405**	block3	Control	pre	piu2	Subject	Broad	r2	179.2508608	1	1	broad_focus	2	Broad focus
**2023405**	block3	Control	pre	tse2	Subject	Broad	r2	195.9584826	2	2	broad_focus	2	Broad focus
**2023405**	block3	Control	pre	tsap1	Verb	Broad	r2	111.4072787	3	1	broad_focus	1	Broad focus
**2023405**	block3	Control	pre	sy1	Object	Broad	r2	249.2084568	4	1	broad_focus	1	Broad focus
**2023405**	block3	Control	pre	pau1	Object	Broad	r2	457.023869	5	2	broad_focus	1	Broad focus
**2023405**	block3	Control	pre	bui3	Subject	Contrastive	r2	122.6248502	1	1	on_focus	3	Contrastive on_focus
**2023405**	block3	Control	pre	bui3	Subject	Contrastive	r2	142.0131406	2	2	on_focus	3	Contrastive on_focus
**2023405**	block3	Control	pre	tsv1	Verb	Contrastive	r2	138.7829125	3	1	post_focus	1	Contrastive post_focus
**2023405**	block3	Control	pre	fug1	Object	Contrastive	r2	188.0964918	4	1	post_focus	1	Contrastive post_focus
**2023405**	block3	Control	pre	tshe1	Object	Contrastive	r2	193.9791932	5	2	post_focus	1	Contrastive post_focus
**2023405**	block3	Control	pre	bui3	Subject	Narrow	r2	144.3014841	1	1	on_focus	3	Narrow on_focus
**2023405**	block3	Control	pre	bui3	Subject	Narrow	r2	144.9926053	2	2	on_focus	3	Narrow on_focus
**2023405**	block3	Control	pre	tsv1	Verb	Narrow	r2	154.8880072	3	1	post_focus	1	Narrow post_focus
**2023405**	block3	Control	pre	fug1	Object	Narrow	r2	216.9619632	4	1	post_focus	1	Narrow post_focus
**2023405**	block3	Control	pre	tshe1	Object	Narrow	r2	257.0620755	5	2	post_focus	1	Narrow post_focus
**2023405**	block3	Control	pre	bui3	Subject	Narrow	r2	153.8584503	1	1	pre_focus	3	Narrow pre_focus
**2023405**	block3	Control	pre	bui3	Subject	Narrow	r2	150.9974823	2	2	pre_focus	3	Narrow pre_focus
**2023405**	block3	Control	pre	tsv1	Verb	Narrow	r2	185.9950623	3	1	pre_focus	1	Narrow pre_focus
**2023405**	block3	Control	pre	fug1	Object	Narrow	r2	165.1562124	4	1	on_focus	1	Narrow on_focus
**2023405**	block3	Control	pre	tshe1	Object	Narrow	r2	230.9112556	5	2	on_focus	1	Narrow on_focus
**2023405**	block3	Control	pre	suk1	Subject	Narrow	r2	80.01395388	1	1	pre_focus	1	Narrow pre_focus
**2023405**	block3	Control	pre	suk1	Subject	Narrow	r2	80.56972349	2	2	pre_focus	1	Narrow pre_focus
**2023405**	block3	Control	pre	sei2	Verb	Narrow	r2	190.3217072	3	1	on_focus	2	Narrow on_focus

2023405	block3	Control	pre	svy2	Object	Narrow	r2	237.2016493	4	1	post_focus	2	Narrow post_focus
2023405	block3	Control	pre	kwo2	Object	Narrow	r2	224.0727572	5	2	post_focus	2	Narrow post_focus
2023405	block3	Control	pre	suk1	Subject	Contrastive	r2	93.08973432	1	1	on_focus	1	Contrastive on_focus
2023405	block3	Control	pre	suk1	Subject	Contrastive	r2	120.8849239	2	2	on_focus	1	Contrastive on_focus
2023405	block3	Control	pre	sei2	Verb	Contrastive	r2	161.9479016	3	1	post_focus	2	Contrastive post_focus
2023405	block3	Control	pre	svy2	Object	Contrastive	r2	86.99056304	4	1	post_focus	2	Contrastive post_focus
2023405	block3	Control	pre	kwo2	Object	Contrastive	r2	265.6922357	5	2	post_focus	2	Contrastive post_focus
2023405	block3	Control	pre	piu2	Subject	Contrastive	r2	141.6357237	1	1	pre_focus	2	Contrastive pre_focus
2023405	block3	Control	pre	tse2	Subject	Contrastive	r2	145.2044441	2	2	pre_focus	2	Contrastive pre_focus
2023405	block3	Control	pre	tsap1	Verb	Contrastive	r2	108.1274653	3	1	pre_focus	1	Contrastive pre_focus
2023405	block3	Control	pre	sy1	Object	Contrastive	r2	103.8205465	4	1	on_focus	1	Contrastive on_focus
2023405	block3	Control	pre	pau1	Object	Contrastive	r2	314.6316769	5	2	on_focus	1	Contrastive on_focus
2023405	block3	Control	pre	piu2	Subject	Contrastive	r2	176.1920603	1	1	pre_focus	2	Contrastive pre_focus
2023405	block3	Control	pre	tse2	Subject	Contrastive	r2	137.8152728	2	2	pre_focus	2	Contrastive pre_focus
2023405	block3	Control	pre	tsap1	Verb	Contrastive	r2	88.9229185	3	1	on_focus	1	Contrastive on_focus
2023405	block3	Control	pre	sy1	Object	Contrastive	r2	163.6264262	4	1	post_focus	1	Contrastive post_focus
2023405	block3	Control	pre	pau1	Object	Contrastive	r2	360.2426971	5	2	post_focus	1	Contrastive post_focus
2023405	block3	Control	pre	piu2	Subject	Narrow	r2	184.5118195	1	1	pre_focus	2	Narrow pre_focus
2023405	block3	Control	pre	tse2	Subject	Narrow	r2	165.7622883	2	2	pre_focus	2	Narrow pre_focus
2023405	block3	Control	pre	tsap1	Verb	Narrow	r2	146.4469976	3	1	on_focus	1	Narrow on_focus
2023405	block3	Control	pre	sy1	Object	Narrow	r2	158.3889771	4	1	post_focus	1	Narrow post_focus
2023405	block3	Control	pre	pau1	Object	Narrow	r2	456.5148668	5	2	post_focus	1	Narrow post_focus
2023405	block3	Control	pre	suk1	Subject	Contrastive	r2	81.67955333	1	1	pre_focus	1	Contrastive pre_focus
2023405	block3	Control	pre	suk1	Subject	Contrastive	r2	125.3334121	2	2	pre_focus	1	Contrastive pre_focus
2023405	block3	Control	pre	sei2	Verb	Contrastive	r2	316.1627635	3	1	on_focus	2	Contrastive on_focus
2023405	block3	Control	pre	svy2	Object	Contrastive	r2	237.1474058	4	1	post_focus	2	Contrastive post_focus
2023405	block3	Control	pre	kwo2	Object	Contrastive	r2	296.8725394	5	2	post_focus	2	Contrastive post_focus
2023405	block3	Control	pre	piu2	Subject	Contrastive	r2	142.7088976	1	1	on_focus	2	Contrastive on_focus
2023405	block3	Control	pre	tse2	Subject	Contrastive	r2	148.0905713	2	2	on_focus	2	Contrastive on_focus
2023405	block3	Control	pre	tsap1	Verb	Contrastive	r2	75.13808454	3	1	post_focus	1	Contrastive post_focus
2023405	block3	Control	pre	sy1	Object	Contrastive	r2	159.7385794	4	1	post_focus	1	Contrastive post_focus
2023405	block3	Control	pre	pau1	Object	Contrastive	r2	350.6423966	5	2	post_focus	1	Contrastive post_focus
2023405	block3	Control	pre	suk1	Subject	Contrastive	r2	142.4145263	1	1	pre_focus	1	Contrastive pre_focus
2023405	block3	Control	pre	suk1	Subject	Contrastive	r2	156.6005909	2	2	pre_focus	1	Contrastive pre_focus
2023405	block3	Control	pre	sei2	Verb	Contrastive	r2	258.3731271	3	1	pre_focus	2	Contrastive pre_focus
2023405	block3	Control	pre	svy2	Object	Contrastive	r2	299.0180057	4	1	on_focus	2	Contrastive on_focus
2023405	block3	Control	pre	kwo2	Object	Contrastive	r2	331.2784323	5	2	on_focus	2	Contrastive on_focus
2023405	block3	Control	pre	suk1	Subject	Narrow	r2	52.48650451	1	1	pre_focus	1	Narrow pre_focus
2023405	block3	Control	pre	suk1	Subject	Narrow	r2	125.9673397	2	2	pre_focus	1	Narrow pre_focus
2023405	block3	Control	pre	sei2	Verb	Narrow	r2	170.4364727	3	1	pre_focus	2	Narrow pre_focus
2023405	block3	Control	pre	svy2	Object	Narrow	r2	193.8318521	4	1	on_focus	2	Narrow on_focus
2023405	block3	Control	pre	kwo2	Object	Narrow	r2	158.7020095	5	2	on_focus	2	Narrow on_focus
2023405	block3	Control	pre	bui3	Subject	Narrow	r2	163.5232075	1	1	pre_focus	3	Narrow pre_focus
2023405	block3	Control	pre	bui3	Subject	Narrow	r2	84.54276784	2	2	pre_focus	3	Narrow pre_focus
2023405	block3	Control	pre	tsv1	Verb	Narrow	r2	152.0117406	3	1	on_focus	1	Narrow on_focus
2023405	block3	Control	pre	fug1	Object	Narrow	r2	222.0119997	4	1	post_focus	1	Narrow post_focus
2023405	block3	Control	pre	tshe1	Object	Narrow	r2	258.3970885	5	2	post_focus	1	Narrow post_focus
2023405	block3	Control	pre	suk1	Subject	Broad	r2	103.1713671	1	1	broad_focus	1	Broad focus
2023405	block3	Control	pre	suk1	Subject	Broad	r2	87.89373249	2	2	broad_focus	1	Broad focus
2023405	block3	Control	pre	sei2	Verb	Broad	r2	131.2832756	3	1	broad_focus	2	Broad focus
2023405	block3	Control	pre	svy2	Object	Broad	r2	169.74394	4	1	broad_focus	2	Broad focus
2023405	block3	Control	pre	kwo2	Object	Broad	r2	284.8691575	5	2	broad_focus	2	Broad focus
2023405	block3	Control	pre	bui3	Subject	Broad	r2	161.1328576	1	1	broad_focus	3	Broad focus
2023405	block3	Control	pre	bui3	Subject	Broad	r2	173.8380844	2	2	broad_focus	3	Broad focus
2023405	block3	Control	pre	tsv1	Verb	Broad	r2	167.4253652	3	1	broad_focus	1	Broad focus
2023405	block3	Control	pre	fug1	Object	Broad	r2	238.8780045	4	1	broad_focus	1	Broad focus
2023405	block3	Control	pre	tshe1	Object	Broad	r2	335.9409773	5	2	broad_focus	1	Broad focus
2023405	block4	Control	post	pak3	Subject	Narrow	r1	118.8958589	1	1	pre_focus	3	Narrow pre_focus
2023405	block4	Control	post	pak3	Subject	Narrow	r1	154.1657289	2	2	pre_focus	3	Narrow pre_focus
2023405	block4	Control	post	tsing2	Verb	Narrow	r1	256.1580817	3	1	on_focus	2	Narrow on_focus
2023405	block4	Control	post	kau2	Object	Narrow	r1	329.489732	4	1	post_focus	2	Narrow post_focus
2023405	block4	Control	post	tsi2	Object	Narrow	r1	158.1591298	5	2	post_focus	2	Narrow post_focus
2023405	block4	Control	post	Jan-01	Subject	Narrow	r1	282.4302291	1	1	pre_focus	1	Narrow pre_focus
2023405	block4	Control	post	Jan-01	Subject	Narrow	r1	260.8417368	2	2	pre_focus	1	Narrow pre_focus
2023405	block4	Control	post	wei3	Verb	Narrow	r1	398.3019864	3	1	on_focus	3	Narrow on_focus
2023405	block4	Control	post	tsam3	Object	Narrow	r1	259.4635187	4	1	post_focus	3	Narrow post_focus

2023405	block4	Control	post	tsam3	Object	Narrow	r1	253.5484924	5	2	post_focus	3	Narrow post_focus
2023405	block4	Control	post	pak3	Subject	Contrastive	r1	97.68124701	1	1	pre_focus	3	Contrastive pre_focus
2023405	block4	Control	post	pak3	Subject	Contrastive	r1	136.7737659	2	2	pre_focus	3	Contrastive pre_focus
2023405	block4	Control	post	tsing2	Verb	Contrastive	r1	208.03177	3	1	pre_focus	2	Contrastive pre_focus
2023405	block4	Control	post	kau2	Object	Contrastive	r1	323.8169115	4	1	on_focus	2	Contrastive on_focus
2023405	block4	Control	post	tsi2	Object	Contrastive	r1	357.5033257	5	2	on_focus	2	Contrastive on_focus
2023405	block4	Control	post	piu35	Subject	Contrastive	r1	238.1637755	1	1	on_focus	35	Contrastive on_focus
2023405	block4	Control	post	mui35	Subject	Contrastive	r1	392.8925489	2	2	on_focus	35	Contrastive on_focus
2023405	block4	Control	post	tsan3	Verb	Contrastive	r1	314.1332508	3	1	post_focus	3	Contrastive post_focus
2023405	block4	Control	post	jin3	Object	Contrastive	r1	293.7821964	4	1	post_focus	3	Contrastive post_focus
2023405	block4	Control	post	jin3	Object	Contrastive	r1	693.0046347	5	2	post_focus	3	Contrastive post_focus
2023405	block4	Control	post	pak3	Subject	Broad	r1	130.9134719	1	1	broad_focus	3	Broad focus
2023405	block4	Control	post	pak3	Subject	Broad	r1	151.5230233	2	2	broad_focus	3	Broad focus
2023405	block4	Control	post	tsing2	Verb	Broad	r1	252.8879748	3	1	broad_focus	2	Broad focus
2023405	block4	Control	post	kau2	Object	Broad	r1	298.9935519	4	1	broad_focus	2	Broad focus
2023405	block4	Control	post	tsi2	Object	Broad	r1	331.0917722	5	2	broad_focus	2	Broad focus
2023405	block4	Control	post	piu35	Subject	Narrow	r1	250.3228979	1	1	on_focus	35	Narrow on_focus
2023405	block4	Control	post	mui35	Subject	Narrow	r1	320.5696068	2	2	on_focus	35	Narrow on_focus
2023405	block4	Control	post	tsan3	Verb	Narrow	r1	284.2674068	3	1	post_focus	3	Narrow post_focus
2023405	block4	Control	post	jin3	Object	Narrow	r1	284.1480279	4	1	post_focus	3	Narrow post_focus
2023405	block4	Control	post	jin3	Object	Narrow	r1	347.2504987	5	2	post_focus	3	Narrow post_focus
2023405	block4	Control	post	piu35	Subject	Broad	r1	214.9497626	1	1	broad_focus	35	Broad focus
2023405	block4	Control	post	mui35	Subject	Broad	r1	320.5066909	2	2	broad_focus	35	Broad focus
2023405	block4	Control	post	tsan3	Verb	Broad	r1	282.2883258	3	1	broad_focus	3	Broad focus
2023405	block4	Control	post	jin3	Object	Broad	r1	234.5092859	4	1	broad_focus	3	Broad focus
2023405	block4	Control	post	jin3	Object	Broad	r1	387.8593858	5	2	broad_focus	3	Broad focus
2023405	block4	Control	post	Jan-01	Subject	Narrow	r1	323.9021798	1	1	on_focus	1	Narrow on_focus
2023405	block4	Control	post	Jan-01	Subject	Narrow	r1	334.0849451	2	2	on_focus	1	Narrow on_focus
2023405	block4	Control	post	wei3	Verb	Narrow	r1	350.9412361	3	1	post_focus	3	Narrow post_focus
2023405	block4	Control	post	tsam3	Object	Narrow	r1	322.4439233	4	1	post_focus	3	Narrow post_focus
2023405	block4	Control	post	tsam3	Object	Narrow	r1	481.1389671	5	2	post_focus	3	Narrow post_focus
2023405	block4	Control	post	piu35	Subject	Narrow	r1	255.1785994	1	1	pre_focus	35	Narrow pre_focus
2023405	block4	Control	post	mui35	Subject	Narrow	r1	305.4233919	2	2	pre_focus	35	Narrow pre_focus
2023405	block4	Control	post	tsan3	Verb	Narrow	r1	374.0663171	3	1	on_focus	3	Narrow on_focus
2023405	block4	Control	post	jin3	Object	Narrow	r1	308.9044668	4	1	post_focus	3	Narrow post_focus
2023405	block4	Control	post	jin3	Object	Narrow	r1	375.9784992	5	2	post_focus	3	Narrow post_focus
2023405	block4	Control	post	piu35	Subject	Contrastive	r1	225.2265691	1	1	pre_focus	35	Contrastive pre_focus
2023405	block4	Control	post	mui35	Subject	Contrastive	r1	305.0118611	2	2	pre_focus	35	Contrastive pre_focus
2023405	block4	Control	post	tsan3	Verb	Contrastive	r1	374.850693	3	1	pre_focus	3	Contrastive pre_focus
2023405	block4	Control	post	jin3	Object	Contrastive	r1	358.0004314	4	1	on_focus	3	Contrastive on_focus
2023405	block4	Control	post	jin3	Object	Contrastive	r1	490.1895612	5	2	on_focus	3	Contrastive on_focus
2023405	block4	Control	post	Jan-01	Subject	Broad	r1	318.2866207	1	1	broad_focus	1	Broad focus
2023405	block4	Control	post	Jan-01	Subject	Broad	r1	255.5149125	2	2	broad_focus	1	Broad focus
2023405	block4	Control	post	wei3	Verb	Broad	r1	435.8687743	3	1	broad_focus	3	Broad focus
2023405	block4	Control	post	tsam3	Object	Broad	r1	326.2570816	4	1	broad_focus	3	Broad focus
2023405	block4	Control	post	tsam3	Object	Broad	r1	497.6737779	5	2	broad_focus	3	Broad focus
2023405	block4	Control	post	piu35	Subject	Narrow	r1	223.1963021	1	1	pre_focus	35	Narrow pre_focus
2023405	block4	Control	post	mui35	Subject	Narrow	r1	298.8551286	2	2	pre_focus	35	Narrow pre_focus
2023405	block4	Control	post	tsan3	Verb	Narrow	r1	287.1165558	3	1	pre_focus	3	Narrow pre_focus
2023405	block4	Control	post	jin3	Object	Narrow	r1	235.2266528	4	1	on_focus	3	Narrow on_focus
2023405	block4	Control	post	jin3	Object	Narrow	r1	446.878551	5	2	on_focus	3	Narrow on_focus
2023405	block4	Control	post	Jan-01	Subject	Contrastive	r1	249.8782911	1	1	pre_focus	1	Contrastive pre_focus
2023405	block4	Control	post	Jan-01	Subject	Contrastive	r1	136.366152	2	2	pre_focus	1	Contrastive pre_focus
2023405	block4	Control	post	wei3	Verb	Contrastive	r1	230.7658358	3	1	pre_focus	3	Contrastive pre_focus
2023405	block4	Control	post	tsam3	Object	Contrastive	r1	298.7155655	4	1	on_focus	3	Contrastive on_focus
2023405	block4	Control	post	tsam3	Object	Contrastive	r1	404.6990185	5	2	on_focus	3	Contrastive on_focus
2023405	block4	Control	post	Jan-01	Subject	Contrastive	r1	292.7639183	1	1	pre_focus	1	Contrastive pre_focus
2023405	block4	Control	post	Jan-01	Subject	Contrastive	r1	273.538956	2	2	pre_focus	1	Contrastive pre_focus
2023405	block4	Control	post	wei3	Verb	Contrastive	r1	463.847583	3	1	on_focus	3	Contrastive on_focus
2023405	block4	Control	post	tsam3	Object	Contrastive	r1	329.5723174	4	1	post_focus	3	Contrastive post_focus
2023405	block4	Control	post	tsam3	Object	Contrastive	r1	419.0617149	5	2	post_focus	3	Contrastive post_focus
2023405	block4	Control	post	Jan-01	Subject	Narrow	r1	223.8752084	1	1	pre_focus	1	Narrow pre_focus
2023405	block4	Control	post	Jan-01	Subject	Narrow	r1	212.9425121	2	2	pre_focus	1	Narrow pre_focus
2023405	block4	Control	post	wei3	Verb	Narrow	r1	218.3141907	3	1	pre_focus	3	Narrow pre_focus
2023405	block4	Control	post	tsam3	Object	Narrow	r1	220.4262789	4	1	on_focus	3	Narrow on_focus
2023405	block4	Control	post	tsam3	Object	Narrow	r1	379.3753975	5	2	on_focus	3	Narrow on_focus

**2023405**	block4	Control	post	pak3	Subject	Narrow	r1	99.20908978	1	1	pre_focus	3	Narrow pre_focus
**2023405**	block4	Control	post	pak3	Subject	Narrow	r1	117.2185281	2	2	pre_focus	3	Narrow pre_focus
**2023405**	block4	Control	post	tsing2	Verb	Narrow	r1	210.7291005	3	1	pre_focus	2	Narrow pre_focus
**2023405**	block4	Control	post	kau2	Object	Narrow	r1	298.9376161	4	1	on_focus	2	Narrow on_focus
**2023405**	block4	Control	post	tsi2	Object	Narrow	r1	268.403232	5	2	on_focus	2	Narrow on_focus
**2023405**	block4	Control	post	pak3	Subject	Contrastive	r1	126.2397107	1	1	pre_focus	3	Contrastive pre_focus
**2023405**	block4	Control	post	pak3	Subject	Contrastive	r1	131.7528473	2	2	pre_focus	3	Contrastive pre_focus
**2023405**	block4	Control	post	tsing2	Verb	Contrastive	r1	312.1638842	3	1	on_focus	2	Contrastive on_focus
**2023405**	block4	Control	post	kau2	Object	Contrastive	r1	311.84946	4	1	post_focus	2	Contrastive post_focus
**2023405**	block4	Control	post	tsi2	Object	Contrastive	r1	368.7913849	5	2	post_focus	2	Contrastive post_focus
**2023405**	block4	Control	post	pak3	Subject	Contrastive	r1	110.6465129	1	1	on_focus	3	Contrastive on_focus
**2023405**	block4	Control	post	pak3	Subject	Contrastive	r1	133.9744988	2	2	on_focus	3	Contrastive on_focus
**2023405**	block4	Control	post	tsing2	Verb	Contrastive	r1	211.5165036	3	1	post_focus	2	Contrastive post_focus
**2023405**	block4	Control	post	kau2	Object	Contrastive	r1	286.0861625	4	1	post_focus	2	Contrastive post_focus
**2023405**	block4	Control	post	tsi2	Object	Contrastive	r1	315.7327916	5	2	post_focus	2	Contrastive post_focus
**2023405**	block4	Control	post	piu35	Subject	Contrastive	r1	172.7338076	1	1	pre_focus	35	Contrastive pre_focus
**2023405**	block4	Control	post	mui35	Subject	Contrastive	r1	320.8871836	2	2	pre_focus	35	Contrastive pre_focus
**2023405**	block4	Control	post	tsan3	Verb	Contrastive	r1	326.4277505	3	1	on_focus	3	Contrastive on_focus
**2023405**	block4	Control	post	jin3	Object	Contrastive	r1	308.0067637	4	1	post_focus	3	Contrastive post_focus
**2023405**	block4	Control	post	jin3	Object	Contrastive	r1	421.418425	5	2	post_focus	3	Contrastive post_focus
**2023405**	block4	Control	post	pak3	Subject	Narrow	r1	112.2883797	1	1	on_focus	3	Narrow on_focus
**2023405**	block4	Control	post	pak3	Subject	Narrow	r1	111.5514128	2	2	on_focus	3	Narrow on_focus
**2023405**	block4	Control	post	tsing2	Verb	Narrow	r1	219.6744623	3	1	post_focus	2	Narrow post_focus
**2023405**	block4	Control	post	kau2	Object	Narrow	r1	284.1429698	4	1	post_focus	2	Narrow post_focus
**2023405**	block4	Control	post	tsi2	Object	Narrow	r1	309.4851696	5	2	post_focus	2	Narrow post_focus
**2023405**	block4	Control	post	Jan-01	Subject	Contrastive	r1	384.5543365	1	1	on_focus	1	Contrastive on_focus
**2023405**	block4	Control	post	Jan-01	Subject	Contrastive	r1	262.7828215	2	2	on_focus	1	Contrastive on_focus
**2023405**	block4	Control	post	wei3	Verb	Contrastive	r1	308.2594716	3	1	post_focus	3	Contrastive post_focus
**2023405**	block4	Control	post	tsam3	Object	Contrastive	r1	324.5553147	4	1	post_focus	3	Contrastive post_focus
**2023405**	block4	Control	post	tsam3	Object	Contrastive	r1	335.0110208	5	2	post_focus	3	Contrastive post_focus
**2023405**	block4	Control	post	Jan-01	Subject	Contrastive	r2	303.3087048	1	1	pre_focus	1	Contrastive pre_focus
**2023405**	block4	Control	post	Jan-01	Subject	Contrastive	r2	284.1785615	2	2	pre_focus	1	Contrastive pre_focus
**2023405**	block4	Control	post	wei3	Verb	Contrastive	r2	506.4520139	3	1	on_focus	3	Contrastive on_focus
**2023405**	block4	Control	post	tsam3	Object	Contrastive	r2	312.1973319	4	1	post_focus	3	Contrastive post_focus
**2023405**	block4	Control	post	tsam3	Object	Contrastive	r2	451.9881558	5	2	post_focus	3	Contrastive post_focus
**2023405**	block4	Control	post	Jan-01	Subject	Narrow	r2	305.0460842	1	1	pre_focus	1	Narrow pre_focus
**2023405**	block4	Control	post	Jan-01	Subject	Narrow	r2	284.021496	2	2	pre_focus	1	Narrow pre_focus
**2023405**	block4	Control	post	wei3	Verb	Narrow	r2	331.4276602	3	1	pre_focus	3	Narrow pre_focus
**2023405**	block4	Control	post	tsam3	Object	Narrow	r2	242.9014752	4	1	on_focus	3	Narrow on_focus
**2023405**	block4	Control	post	tsam3	Object	Narrow	r2	419.6403268	5	2	on_focus	3	Narrow on_focus
**2023405**	block4	Control	post	pak3	Subject	Narrow	r2	105.3031104	1	1	on_focus	3	Narrow on_focus
**2023405**	block4	Control	post	pak3	Subject	Narrow	r2	143.0502243	2	2	on_focus	3	Narrow on_focus
**2023405**	block4	Control	post	tsing2	Verb	Narrow	r2	186.6516289	3	1	post_focus	2	Narrow post_focus
**2023405**	block4	Control	post	kau2	Object	Narrow	r2	255.4612955	4	1	post_focus	2	Narrow post_focus
**2023405**	block4	Control	post	tsi2	Object	Narrow	r2	314.2909244	5	2	post_focus	2	Narrow post_focus
**2023405**	block4	Control	post	pak3	Subject	Broad	r2	126.3477367	1	1	broad_focus	3	Broad focus
**2023405**	block4	Control	post	pak3	Subject	Broad	r2	151.8371205	2	2	broad_focus	3	Broad focus
**2023405**	block4	Control	post	tsing2	Verb	Broad	r2	211.9268879	3	1	broad_focus	2	Broad focus
**2023405**	block4	Control	post	kau2	Object	Broad	r2	258.0578929	4	1	broad_focus	2	Broad focus
**2023405**	block4	Control	post	tsi2	Object	Broad	r2	346.4719293	5	2	broad_focus	2	Broad focus
**2023405**	block4	Control	post	piu35	Subject	Contrastive	r2	140.3951272	1	1	pre_focus	35	Contrastive pre_focus
**2023405**	block4	Control	post	mui35	Subject	Contrastive	r2	202.4662591	2	2	pre_focus	35	Contrastive pre_focus
**2023405**	block4	Control	post	tsan3	Verb	Contrastive	r2	246.3566725	3	1	pre_focus	3	Contrastive pre_focus
**2023405**	block4	Control	post	jin3	Object	Contrastive	r2	225.1531664	4	1	on_focus	3	Contrastive on_focus
**2023405**	block4	Control	post	jin3	Object	Contrastive	r2	293.5954776	5	2	on_focus	3	Contrastive on_focus
**2023405**	block4	Control	post	piu35	Subject	Contrastive	r2	159.4112182	1	1	pre_focus	35	Contrastive pre_focus
**2023405**	block4	Control	post	mui35	Subject	Contrastive	r2	296.7120671	2	2	pre_focus	35	Contrastive pre_focus
**2023405**	block4	Control	post	tsan3	Verb	Contrastive	r2	224.9895011	3	1	on_focus	3	Contrastive on_focus
**2023405**	block4	Control	post	jin3	Object	Contrastive	r2	202.3708744	4	1	post_focus	3	Contrastive post_focus
**2023405**	block4	Control	post	jin3	Object	Contrastive	r2	347.4888775	5	2	post_focus	3	Contrastive post_focus
**2023405**	block4	Control	post	Jan-01	Subject	Contrastive	r2	252.4041139	1	1	pre_focus	1	Contrastive pre_focus
**2023405**	block4	Control	post	Jan-01	Subject	Contrastive	r2	208.4236647	2	2	pre_focus	1	Contrastive pre_focus
**2023405**	block4	Control	post	wei3	Verb	Contrastive	r2	313.2323103	3	1	pre_focus	3	Contrastive pre_focus
**2023405**	block4	Control	post	tsam3	Object	Contrastive	r2	277.119736	4	1	on_focus	3	Contrastive on_focus
**2023405**	block4	Control	post	tsam3	Object	Contrastive	r2	272.0242549	5	2	on_focus	3	Contrastive on_focus
**2023405**	block4	Control	post	pak3	Subject	Narrow	r2	111.0263071	1	1	pre_focus	3	Narrow pre_focus

**2023405**	block4	Control	post	pak3	Subject	Narrow	r2	124.4184101	2	2	pre_focus	3	Narrow pre_focus
**2023405**	block4	Control	post	tsing2	Verb	Narrow	r2	266.5948846	3	1	on_focus	2	Narrow on_focus
**2023405**	block4	Control	post	kau2	Object	Narrow	r2	358.74589	4	1	post_focus	2	Narrow post_focus
**2023405**	block4	Control	post	tsi2	Object	Narrow	r2	282.6346472	5	2	post_focus	2	Narrow post_focus
**2023405**	block4	Control	post	Jan-01	Subject	Broad	r2	205.0015985	1	1	broad_focus	1	Broad focus
**2023405**	block4	Control	post	Jan-01	Subject	Broad	r2	240.3901463	2	2	broad_focus	1	Broad focus
**2023405**	block4	Control	post	wei3	Verb	Broad	r2	201.3640924	3	1	broad_focus	3	Broad focus
**2023405**	block4	Control	post	tsam3	Object	Broad	r2	257.8260502	4	1	broad_focus	3	Broad focus
**2023405**	block4	Control	post	tsam3	Object	Broad	r2	289.1039236	5	2	broad_focus	3	Broad focus
**2023405**	block4	Control	post	Jan-01	Subject	Contrastive	r2	267.7363693	1	1	on_focus	1	Contrastive on_focus
**2023405**	block4	Control	post	Jan-01	Subject	Contrastive	r2	202.0574944	2	2	on_focus	1	Contrastive on_focus
**2023405**	block4	Control	post	wei3	Verb	Contrastive	r2	313.9945467	3	1	post_focus	3	Contrastive post_focus
**2023405**	block4	Control	post	tsam3	Object	Contrastive	r2	238.6957231	4	1	post_focus	3	Contrastive post_focus
**2023405**	block4	Control	post	tsam3	Object	Contrastive	r2	466.4794825	5	2	post_focus	3	Contrastive post_focus
**2023405**	block4	Control	post	pak3	Subject	Contrastive	r2	111.136418	1	1	pre_focus	3	Contrastive pre_focus
**2023405**	block4	Control	post	pak3	Subject	Contrastive	r2	137.7318979	2	2	pre_focus	3	Contrastive pre_focus
**2023405**	block4	Control	post	tsing2	Verb	Contrastive	r2	199.5588663	3	1	pre_focus	2	Contrastive pre_focus
**2023405**	block4	Control	post	kau2	Object	Contrastive	r2	268.9801152	4	1	on_focus	2	Contrastive on_focus
**2023405**	block4	Control	post	tsi2	Object	Contrastive	r2	302.7784829	5	2	on_focus	2	Contrastive on_focus
**2023405**	block4	Control	post	Jan-01	Subject	Narrow	r2	207.4276389	1	1	pre_focus	1	Narrow pre_focus
**2023405**	block4	Control	post	Jan-01	Subject	Narrow	r2	252.3877006	2	2	pre_focus	1	Narrow pre_focus
**2023405**	block4	Control	post	wei3	Verb	Narrow	r2	204.3087753	3	1	on_focus	3	Narrow on_focus
**2023405**	block4	Control	post	tsam3	Object	Narrow	r2	245.6367195	4	1	post_focus	3	Narrow post_focus
**2023405**	block4	Control	post	tsam3	Object	Narrow	r2	290.6830034	5	2	post_focus	3	Narrow post_focus
**2023405**	block4	Control	post	piu35	Subject	Narrow	r2	219.1813934	1	1	pre_focus	35	Narrow pre_focus
**2023405**	block4	Control	post	mui35	Subject	Narrow	r2	349.2001027	2	2	pre_focus	35	Narrow pre_focus
**2023405**	block4	Control	post	tsan3	Verb	Narrow	r2	335.7709	3	1	pre_focus	3	Narrow pre_focus
**2023405**	block4	Control	post	jin3	Object	Narrow	r2	315.1427207	4	1	on_focus	3	Narrow on_focus
**2023405**	block4	Control	post	jin3	Object	Narrow	r2	371.9402519	5	2	on_focus	3	Narrow on_focus
**2023405**	block4	Control	post	piu35	Subject	Contrastive	r2	196.8075034	1	1	on_focus	35	Contrastive on_focus
**2023405**	block4	Control	post	mui35	Subject	Contrastive	r2	353.8822221	2	2	on_focus	35	Contrastive on_focus
**2023405**	block4	Control	post	tsan3	Verb	Contrastive	r2	311.6283899	3	1	post_focus	3	Contrastive post_focus
**2023405**	block4	Control	post	jin3	Object	Contrastive	r2	241.3739301	4	1	post_focus	3	Contrastive post_focus
**2023405**	block4	Control	post	jin3	Object	Contrastive	r2	345.3737129	5	2	post_focus	3	Contrastive post_focus
**2023405**	block4	Control	post	piu35	Subject	Broad	r2	273.1530614	1	1	broad_focus	35	Broad focus
**2023405**	block4	Control	post	mui35	Subject	Broad	r2	248.6648208	2	2	broad_focus	35	Broad focus
**2023405**	block4	Control	post	tsan3	Verb	Broad	r2	303.7231687	3	1	broad_focus	3	Broad focus
**2023405**	block4	Control	post	jin3	Object	Broad	r2	286.0964856	4	1	broad_focus	3	Broad focus
**2023405**	block4	Control	post	jin3	Object	Broad	r2	404.5550136	5	2	broad_focus	3	Broad focus
**2023405**	block4	Control	post	pak3	Subject	Narrow	r2	132.028778	1	1	pre_focus	3	Narrow pre_focus
**2023405**	block4	Control	post	pak3	Subject	Narrow	r2	150.9056261	2	2	pre_focus	3	Narrow pre_focus
**2023405**	block4	Control	post	tsing2	Verb	Narrow	r2	275.2746124	3	1	pre_focus	2	Narrow pre_focus
**2023405**	block4	Control	post	kau2	Object	Narrow	r2	285.4914695	4	1	on_focus	2	Narrow on_focus
**2023405**	block4	Control	post	tsi2	Object	Narrow	r2	366.99396	5	2	on_focus	2	Narrow on_focus
**2023405**	block4	Control	post	Jan-01	Subject	Narrow	r2	268.4711475	1	1	on_focus	1	Narrow on_focus
**2023405**	block4	Control	post	Jan-01	Subject	Narrow	r2	221.3459966	2	2	on_focus	1	Narrow on_focus
**2023405**	block4	Control	post	wei3	Verb	Narrow	r2	351.0886769	3	1	post_focus	3	Narrow post_focus
**2023405**	block4	Control	post	tsam3	Object	Narrow	r2	234.1666308	4	1	post_focus	3	Narrow post_focus
**2023405**	block4	Control	post	tsam3	Object	Narrow	r2	344.5653194	5	2	post_focus	3	Narrow post_focus
**2023405**	block4	Control	post	piu35	Subject	Narrow	r2	149.346517	1	1	on_focus	35	Narrow on_focus
**2023405**	block4	Control	post	mui35	Subject	Narrow	r2	282.3158003	2	2	on_focus	35	Narrow on_focus
**2023405**	block4	Control	post	tsan3	Verb	Narrow	r2	269.3458178	3	1	post_focus	3	Narrow post_focus
**2023405**	block4	Control	post	jin3	Object	Narrow	r2	301.1557214	4	1	post_focus	3	Narrow post_focus
**2023405**	block4	Control	post	jin3	Object	Narrow	r2	279.454929	5	2	post_focus	3	Narrow post_focus
**2023405**	block4	Control	post	pak3	Subject	Contrastive	r2	101.847326	1	1	pre_focus	3	Contrastive pre_focus
**2023405**	block4	Control	post	pak3	Subject	Contrastive	r2	107.5659332	2	2	pre_focus	3	Contrastive pre_focus
**2023405**	block4	Control	post	tsing2	Verb	Contrastive	r2	229.7811703	3	1	on_focus	2	Contrastive on_focus
**2023405**	block4	Control	post	kau2	Object	Contrastive	r2	269.0153111	4	1	post_focus	2	Contrastive post_focus
**2023405**	block4	Control	post	tsi2	Object	Contrastive	r2	315.7273248	5	2	post_focus	2	Contrastive post_focus
**2023405**	block4	Control	post	piu35	Subject	Narrow	r2	204.9355221	1	1	pre_focus	35	Narrow pre_focus
**2023405**	block4	Control	post	mui35	Subject	Narrow	r2	262.9048441	2	2	pre_focus	35	Narrow pre_focus
**2023405**	block4	Control	post	tsan3	Verb	Narrow	r2	254.9470293	3	1	on_focus	3	Narrow on_focus
**2023405**	block4	Control	post	jin3	Object	Narrow	r2	269.2632219	4	1	post_focus	3	Narrow post_focus
**2023405**	block4	Control	post	jin3	Object	Narrow	r2	353.4402127	5	2	post_focus	3	Narrow post_focus
**2023405**	block4	Control	post	pak3	Subject	Contrastive	r2	160.4467525	1	1	on_focus	3	Contrastive on_focus
**2023405**	block4	Control	post	pak3	Subject	Contrastive	r2	164.3911623	2	2	on_focus	3	Contrastive on_focus

2023405	block4	Control	post	tsing2	Verb	Contrastive	r2	252.9415066	3	1	post_focus	2	Contrastive post_focus
2023405	block4	Control	post	kau2	Object	Contrastive	r2	289.520993	4	1	post_focus	2	Contrastive post_focus
2023405	block4	Control	post	tsi2	Object	Contrastive	r2	85.90974089	5	2	post_focus	2	Contrastive post_focus
2023405	block4	Control	pre	piu35	Subject	Contrastive	r1	187.015233	1	1	pre_focus	35	Contrastive pre_focus
2023405	block4	Control	pre	mui35	Subject	Contrastive	r1	266.2675242	2	2	pre_focus	35	Contrastive pre_focus
2023405	block4	Control	pre	tsan3	Verb	Contrastive	r1	324.0380841	3	1	pre_focus	3	Contrastive pre_focus
2023405	block4	Control	pre	jin3	Object	Contrastive	r1	252.0359048	4	1	on_focus	3	Contrastive on_focus
2023405	block4	Control	pre	jin3	Object	Contrastive	r1	381.1841339	5	2	on_focus	3	Contrastive on_focus
2023405	block4	Control	pre	piu35	Subject	Contrastive	r1	200.8774808	1	1	pre_focus	35	Contrastive pre_focus
2023405	block4	Control	pre	mui35	Subject	Contrastive	r1	247.7828995	2	2	pre_focus	35	Contrastive pre_focus
2023405	block4	Control	pre	tsan3	Verb	Contrastive	r1	323.9438767	3	1	on_focus	3	Contrastive on_focus
2023405	block4	Control	pre	jin3	Object	Contrastive	r1	258.5347682	4	1	post_focus	3	Contrastive post_focus
2023405	block4	Control	pre	jin3	Object	Contrastive	r1	361.5269633	5	2	post_focus	3	Contrastive post_focus
2023405	block4	Control	pre	piu35	Subject	Narrow	r1	142.374107	1	1	on_focus	35	Narrow on_focus
2023405	block4	Control	pre	mui35	Subject	Narrow	r1	262.7887641	2	2	on_focus	35	Narrow on_focus
2023405	block4	Control	pre	tsan3	Verb	Narrow	r1	260.0151541	3	1	post_focus	3	Narrow post_focus
2023405	block4	Control	pre	jin3	Object	Narrow	r1	271.0503625	4	1	post_focus	3	Narrow post_focus
2023405	block4	Control	pre	jin3	Object	Narrow	r1	346.4031703	5	2	post_focus	3	Narrow post_focus
2023405	block4	Control	pre	piu35	Subject	Narrow	r1	170.7651297	1	1	pre_focus	35	Narrow pre_focus
2023405	block4	Control	pre	mui35	Subject	Narrow	r1	213.7025988	2	2	pre_focus	35	Narrow pre_focus
2023405	block4	Control	pre	tsan3	Verb	Narrow	r1	264.7596849	3	1	pre_focus	3	Narrow pre_focus
2023405	block4	Control	pre	jin3	Object	Narrow	r1	274.1013812	4	1	on_focus	3	Narrow on_focus
2023405	block4	Control	pre	jin3	Object	Narrow	r1	409.6357295	5	2	on_focus	3	Narrow on_focus
2023405	block4	Control	pre	Jan-01	Subject	Contrastive	r1	251.5421187	1	1	on_focus	1	Contrastive on_focus
2023405	block4	Control	pre	Jan-01	Subject	Contrastive	r1	199.1429389	2	2	on_focus	1	Contrastive on_focus
2023405	block4	Control	pre	wei3	Verb	Contrastive	r1	326.5113112	3	1	post_focus	3	Contrastive post_focus
2023405	block4	Control	pre	tsam3	Object	Contrastive	r1	185.3610457	4	1	post_focus	3	Contrastive post_focus
2023405	block4	Control	pre	tsam3	Object	Contrastive	r1	171.481612	5	2	post_focus	3	Contrastive post_focus
2023405	block4	Control	pre	Jan-01	Subject	Contrastive	r1	183.001411	1	1	pre_focus	1	Contrastive pre_focus
2023405	block4	Control	pre	Jan-01	Subject	Contrastive	r1	219.7912453	2	2	pre_focus	1	Contrastive pre_focus
2023405	block4	Control	pre	wei3	Verb	Contrastive	r1	193.2658802	3	1	pre_focus	3	Contrastive pre_focus
2023405	block4	Control	pre	tsam3	Object	Contrastive	r1	200.5438877	4	1	on_focus	3	Contrastive on_focus
2023405	block4	Control	pre	tsam3	Object	Contrastive	r1	355.1022402	5	2	on_focus	3	Contrastive on_focus
2023405	block4	Control	pre	piu35	Subject	Contrastive	r1	161.2008096	1	1	on_focus	35	Contrastive on_focus
2023405	block4	Control	pre	mui35	Subject	Contrastive	r1	257.9875283	2	2	on_focus	35	Contrastive on_focus
2023405	block4	Control	pre	tsan3	Verb	Contrastive	r1	270.7903644	3	1	post_focus	3	Contrastive post_focus
2023405	block4	Control	pre	jin3	Object	Contrastive	r1	266.1041771	4	1	post_focus	3	Contrastive post_focus
2023405	block4	Control	pre	jin3	Object	Contrastive	r1	391.5935442	5	2	post_focus	3	Contrastive post_focus
2023405	block4	Control	pre	Jan-01	Subject	Narrow	r1	254.6849793	1	1	on_focus	1	Narrow on_focus
2023405	block4	Control	pre	Jan-01	Subject	Narrow	r1	230.6245997	2	2	on_focus	1	Narrow on_focus
2023405	block4	Control	pre	wei3	Verb	Narrow	r1	232.9488947	3	1	post_focus	3	Narrow post_focus
2023405	block4	Control	pre	tsam3	Object	Narrow	r1	255.1719863	4	1	post_focus	3	Narrow post_focus
2023405	block4	Control	pre	tsam3	Object	Narrow	r1	276.5986395	5	2	post_focus	3	Narrow post_focus
2023405	block4	Control	pre	Jan-01	Subject	Contrastive	r1	192.1868991	1	1	pre_focus	1	Contrastive pre_focus
2023405	block4	Control	pre	Jan-01	Subject	Contrastive	r1	227.8755608	2	2	pre_focus	1	Contrastive pre_focus
2023405	block4	Control	pre	wei3	Verb	Contrastive	r1	390.8987584	3	1	on_focus	3	Contrastive on_focus
2023405	block4	Control	pre	tsam3	Object	Contrastive	r1	235.1286758	4	1	post_focus	3	Contrastive post_focus
2023405	block4	Control	pre	tsam3	Object	Contrastive	r1	325.8027548	5	2	post_focus	3	Contrastive post_focus
2023405	block4	Control	pre	pak3	Subject	Narrow	r1	91.44236137	1	1	pre_focus	3	Narrow pre_focus
2023405	block4	Control	pre	pak3	Subject	Narrow	r1	132.5460076	2	2	pre_focus	3	Narrow pre_focus
2023405	block4	Control	pre	tsing2	Verb	Narrow	r1	252.856153	3	1	on_focus	2	Narrow on_focus
2023405	block4	Control	pre	kau2	Object	Narrow	r1	157.2100078	4	1	post_focus	2	Narrow post_focus
2023405	block4	Control	pre	tsi2	Object	Narrow	r1	80.49588253	5	2	post_focus	2	Narrow post_focus
2023405	block4	Control	pre	Jan-01	Subject	Broad	r1	213.9125403	1	1	broad_focus	1	Broad focus
2023405	block4	Control	pre	Jan-01	Subject	Broad	r1	226.9501944	2	2	broad_focus	1	Broad focus
2023405	block4	Control	pre	wei3	Verb	Broad	r1	315.675517	3	1	broad_focus	3	Broad focus
2023405	block4	Control	pre	tsam3	Object	Broad	r1	267.1509712	4	1	broad_focus	3	Broad focus
2023405	block4	Control	pre	tsam3	Object	Broad	r1	405.9272523	5	2	broad_focus	3	Broad focus
2023405	block4	Control	pre	pak3	Subject	Contrastive	r1	142.3877386	1	1	on_focus	3	Contrastive on_focus
2023405	block4	Control	pre	pak3	Subject	Contrastive	r1	122.3520851	2	2	on_focus	3	Contrastive on_focus
2023405	block4	Control	pre	tsing2	Verb	Contrastive	r1	133.8363736	3	1	post_focus	2	Contrastive post_focus
2023405	block4	Control	pre	kau2	Object	Contrastive	r1	264.3392298	4	1	post_focus	2	Contrastive post_focus
2023405	block4	Control	pre	tsi2	Object	Contrastive	r1	340.7493244	5	2	post_focus	2	Contrastive post_focus
2023405	block4	Control	pre	pak3	Subject	Broad	r1	75.87688758	1	1	broad_focus	3	Broad focus
2023405	block4	Control	pre	pak3	Subject	Broad	r1	116.5670435	2	2	broad_focus	3	Broad focus
2023405	block4	Control	pre	tsing2	Verb	Broad	r1	199.8929408	3	1	broad_focus	2	Broad focus

2023405	block4	Control	pre	kau2	Object	Broad	r1	274.7690558	4	1	broad_focus	2	Broad focus
2023405	block4	Control	pre	tsi2	Object	Broad	r1	356.9311697	5	2	broad_focus	2	Broad focus
2023405	block4	Control	pre	pak3	Subject	Narrow	r1	101.3878202	1	1	on_focus	3	Narrow on_focus
2023405	block4	Control	pre	pak3	Subject	Narrow	r1	118.8846244	2	2	on_focus	3	Narrow on_focus
2023405	block4	Control	pre	tsing2	Verb	Narrow	r1	207.885162	3	1	post_focus	2	Narrow post_focus
2023405	block4	Control	pre	kau2	Object	Narrow	r1	245.1188538	4	1	post_focus	2	Narrow post_focus
2023405	block4	Control	pre	tsi2	Object	Narrow	r1	105.6970236	5	2	post_focus	2	Narrow post_focus
2023405	block4	Control	pre	piu35	Subject	Broad	r1	158.4841786	1	1	broad_focus	35	Broad focus
2023405	block4	Control	pre	mui35	Subject	Broad	r1	353.4636503	2	2	broad_focus	35	Broad focus
2023405	block4	Control	pre	tsan3	Verb	Broad	r1	285.6745789	3	1	broad_focus	3	Broad focus
2023405	block4	Control	pre	jin3	Object	Broad	r1	275.7838465	4	1	broad_focus	3	Broad focus
2023405	block4	Control	pre	jin3	Object	Broad	r1	333.4823147	5	2	broad_focus	3	Broad focus
2023405	block4	Control	pre	pak3	Subject	Contrastive	r1	134.5950577	1	1	pre_focus	3	Contrastive pre_focus
2023405	block4	Control	pre	pak3	Subject	Contrastive	r1	124.7697741	2	2	pre_focus	3	Contrastive pre_focus
2023405	block4	Control	pre	tsing2	Verb	Contrastive	r1	205.2965472	3	1	pre_focus	2	Contrastive pre_focus
2023405	block4	Control	pre	kau2	Object	Contrastive	r1	267.1806416	4	1	on_focus	2	Contrastive on_focus
2023405	block4	Control	pre	tsi2	Object	Contrastive	r1	323.7146283	5	2	on_focus	2	Contrastive on_focus
2023405	block4	Control	pre	piu35	Subject	Narrow	r1	244.2128396	1	1	pre_focus	35	Narrow pre_focus
2023405	block4	Control	pre	mui35	Subject	Narrow	r1	176.3735869	2	2	pre_focus	35	Narrow pre_focus
2023405	block4	Control	pre	tsan3	Verb	Narrow	r1	263.1481395	3	1	on_focus	3	Narrow on_focus
2023405	block4	Control	pre	jin3	Object	Narrow	r1	277.1421324	4	1	post_focus	3	Narrow post_focus
2023405	block4	Control	pre	jin3	Object	Narrow	r1	341.6761234	5	2	post_focus	3	Narrow post_focus
2023405	block4	Control	pre	Jan-01	Subject	Narrow	r1	192.0574307	1	1	pre_focus	1	Narrow pre_focus
2023405	block4	Control	pre	Jan-01	Subject	Narrow	r1	250.2464019	2	2	pre_focus	1	Narrow pre_focus
2023405	block4	Control	pre	wei3	Verb	Narrow	r1	104.9271295	3	1	pre_focus	3	Narrow pre_focus
2023405	block4	Control	pre	tsam3	Object	Narrow	r1	199.4487488	4	1	on_focus	3	Narrow on_focus
2023405	block4	Control	pre	tsam3	Object	Narrow	r1	407.7468442	5	2	on_focus	3	Narrow on_focus
2023405	block4	Control	pre	Jan-01	Subject	Narrow	r1	178.2380082	1	1	pre_focus	1	Narrow pre_focus
2023405	block4	Control	pre	Jan-01	Subject	Narrow	r1	233.4129106	2	2	pre_focus	1	Narrow pre_focus
2023405	block4	Control	pre	wei3	Verb	Narrow	r1	239.2266445	3	1	on_focus	3	Narrow on_focus
2023405	block4	Control	pre	tsam3	Object	Narrow	r1	214.2316368	4	1	post_focus	3	Narrow post_focus
2023405	block4	Control	pre	tsam3	Object	Narrow	r1	371.7342304	5	2	post_focus	3	Narrow post_focus
2023405	block4	Control	pre	pak3	Subject	Narrow	r1	118.7842267	1	1	pre_focus	3	Narrow pre_focus
2023405	block4	Control	pre	pak3	Subject	Narrow	r1	111.8098372	2	2	pre_focus	3	Narrow pre_focus
2023405	block4	Control	pre	tsing2	Verb	Narrow	r1	220.1614526	3	1	pre_focus	2	Narrow pre_focus
2023405	block4	Control	pre	kau2	Object	Narrow	r1	279.0426139	4	1	on_focus	2	Narrow on_focus
2023405	block4	Control	pre	tsi2	Object	Narrow	r1	256.3458393	5	2	on_focus	2	Narrow on_focus
2023405	block4	Control	pre	pak3	Subject	Contrastive	r1	147.1874436	1	1	pre_focus	3	Contrastive pre_focus
2023405	block4	Control	pre	pak3	Subject	Contrastive	r1	164.466049	2	2	pre_focus	3	Contrastive pre_focus
2023405	block4	Control	pre	tsing2	Verb	Contrastive	r1	233.861029	3	1	on_focus	2	Contrastive on_focus
2023405	block4	Control	pre	kau2	Object	Contrastive	r1	283.311709	4	1	post_focus	2	Contrastive post_focus
2023405	block4	Control	pre	tsi2	Object	Contrastive	r1	272.7044554	5	2	post_focus	2	Contrastive post_focus
2023405	block4	Control	pre	pak3	Subject	Narrow	r2	117.6350374	1	1	pre_focus	3	Narrow pre_focus
2023405	block4	Control	pre	pak3	Subject	Narrow	r2	143.2947296	2	2	pre_focus	3	Narrow pre_focus
2023405	block4	Control	pre	tsing2	Verb	Narrow	r2	174.4339023	3	1	on_focus	2	Narrow on_focus
2023405	block4	Control	pre	kau2	Object	Narrow	r2	215.927346	4	1	post_focus	2	Narrow post_focus
2023405	block4	Control	pre	tsi2	Object	Narrow	r2	289.1017532	5	2	post_focus	2	Narrow post_focus
2023405	block4	Control	pre	Jan-01	Subject	Broad	r2	217.0068361	1	1	broad_focus	1	Broad focus
2023405	block4	Control	pre	Jan-01	Subject	Broad	r2	112.8495612	2	2	broad_focus	1	Broad focus
2023405	block4	Control	pre	wei3	Verb	Broad	r2	295.0252671	3	1	broad_focus	3	Broad focus
2023405	block4	Control	pre	tsam3	Object	Broad	r2	277.6986792	4	1	broad_focus	3	Broad focus
2023405	block4	Control	pre	tsam3	Object	Broad	r2	360.8376065	5	2	broad_focus	3	Broad focus
2023405	block4	Control	pre	piu35	Subject	Contrastive	r2	176.2852677	1	1	pre_focus	35	Contrastive pre_focus
2023405	block4	Control	pre	mui35	Subject	Contrastive	r2	252.1701814	2	2	pre_focus	35	Contrastive pre_focus
2023405	block4	Control	pre	tsan3	Verb	Contrastive	r2	314.2100451	3	1	on_focus	3	Contrastive on_focus
2023405	block4	Control	pre	jin3	Object	Contrastive	r2	232.3707955	4	1	post_focus	3	Contrastive post_focus
2023405	block4	Control	pre	jin3	Object	Contrastive	r2	405.6575244	5	2	post_focus	3	Contrastive post_focus
2023405	block4	Control	pre	Jan-01	Subject	Contrastive	r2	182.2754636	1	1	on_focus	1	Contrastive on_focus
2023405	block4	Control	pre	Jan-01	Subject	Contrastive	r2	222.281505	2	2	on_focus	1	Contrastive on_focus
2023405	block4	Control	pre	wei3	Verb	Contrastive	r2	250.7723464	3	1	post_focus	3	Contrastive post_focus
2023405	block4	Control	pre	tsam3	Object	Contrastive	r2	252.3927942	4	1	post_focus	3	Contrastive post_focus
2023405	block4	Control	pre	tsam3	Object	Contrastive	r2	385.357055	5	2	post_focus	3	Contrastive post_focus
2023405	block4	Control	pre	Jan-01	Subject	Narrow	r2	216.8603361	1	1	pre_focus	1	Narrow pre_focus
2023405	block4	Control	pre	Jan-01	Subject	Narrow	r2	280.6957931	2	2	pre_focus	1	Narrow pre_focus
2023405	block4	Control	pre	wei3	Verb	Narrow	r2	245.1633568	3	1	on_focus	3	Narrow on_focus
2023405	block4	Control	pre	tsam3	Object	Narrow	r2	279.4803381	4	1	post_focus	3	Narrow post_focus

**2023405**	block4	Control	pre	tsam3	Object	Narrow	r2	432.9437702	5	2	post_focus	3	Narrow post_focus
**2023405**	block4	Control	pre	pak3	Subject	Contrastive	r2	123.2963103	1	1	pre_focus	3	Contrastive pre_focus
**2023405**	block4	Control	pre	pak3	Subject	Contrastive	r2	133.7120342	2	2	pre_focus	3	Contrastive pre_focus
**2023405**	block4	Control	pre	tsing2	Verb	Contrastive	r2	203.0749169	3	1	pre_focus	2	Contrastive pre_focus
**2023405**	block4	Control	pre	kau2	Object	Contrastive	r2	301.5012364	4	1	on_focus	2	Contrastive on_focus
**2023405**	block4	Control	pre	tsi2	Object	Contrastive	r2	299.3077441	5	2	on_focus	2	Contrastive on_focus
**2023405**	block4	Control	pre	Jan-01	Subject	Contrastive	r2	260.0068554	1	1	pre_focus	1	Contrastive pre_focus
**2023405**	block4	Control	pre	Jan-01	Subject	Contrastive	r2	188.2692906	2	2	pre_focus	1	Contrastive pre_focus
**2023405**	block4	Control	pre	wei3	Verb	Contrastive	r2	250.414268	3	1	pre_focus	3	Contrastive pre_focus
**2023405**	block4	Control	pre	tsam3	Object	Contrastive	r2	205.6513816	4	1	on_focus	3	Contrastive on_focus
**2023405**	block4	Control	pre	tsam3	Object	Contrastive	r2	317.3095763	5	2	on_focus	3	Contrastive on_focus
**2023405**	block4	Control	pre	pak3	Subject	Broad	r2	130.3361019	1	1	broad_focus	3	Broad focus
**2023405**	block4	Control	pre	pak3	Subject	Broad	r2	134.0508639	2	2	broad_focus	3	Broad focus
**2023405**	block4	Control	pre	tsing2	Verb	Broad	r2	226.9121117	3	1	broad_focus	2	Broad focus
**2023405**	block4	Control	pre	kau2	Object	Broad	r2	269.9164028	4	1	broad_focus	2	Broad focus
**2023405**	block4	Control	pre	tsi2	Object	Broad	r2	290.9127626	5	2	broad_focus	2	Broad focus
**2023405**	block4	Control	pre	Jan-01	Subject	Narrow	r2	221.6089882	1	1	pre_focus	1	Narrow pre_focus
**2023405**	block4	Control	pre	Jan-01	Subject	Narrow	r2	246.0892715	2	2	pre_focus	1	Narrow pre_focus
**2023405**	block4	Control	pre	wei3	Verb	Narrow	r2	237.0490323	3	1	pre_focus	3	Narrow pre_focus
**2023405**	block4	Control	pre	tsam3	Object	Narrow	r2	254.0368054	4	1	on_focus	3	Narrow on_focus
**2023405**	block4	Control	pre	tsam3	Object	Narrow	r2	391.135083	5	2	on_focus	3	Narrow on_focus
**2023405**	block4	Control	pre	Jan-01	Subject	Contrastive	r2	180.2171951	1	1	pre_focus	1	Contrastive pre_focus
**2023405**	block4	Control	pre	Jan-01	Subject	Contrastive	r2	245.3061179	2	2	pre_focus	1	Contrastive pre_focus
**2023405**	block4	Control	pre	wei3	Verb	Contrastive	r2	273.0109665	3	1	on_focus	3	Contrastive on_focus
**2023405**	block4	Control	pre	tsam3	Object	Contrastive	r2	217.4021808	4	1	post_focus	3	Contrastive post_focus
**2023405**	block4	Control	pre	tsam3	Object	Contrastive	r2	208.2946836	5	2	post_focus	3	Contrastive post_focus
**2023405**	block4	Control	pre	pak3	Subject	Contrastive	r2	114.3027863	1	1	pre_focus	3	Contrastive pre_focus
**2023405**	block4	Control	pre	pak3	Subject	Contrastive	r2	142.2582873	2	2	pre_focus	3	Contrastive pre_focus
**2023405**	block4	Control	pre	tsing2	Verb	Contrastive	r2	232.3761264	3	1	on_focus	2	Contrastive on_focus
**2023405**	block4	Control	pre	kau2	Object	Contrastive	r2	291.8283181	4	1	post_focus	2	Contrastive post_focus
**2023405**	block4	Control	pre	tsi2	Object	Contrastive	r2	282.9125267	5	2	post_focus	2	Contrastive post_focus
**2023405**	block4	Control	pre	piu35	Subject	Narrow	r2	253.9860001	1	1	pre_focus	35	Narrow pre_focus
**2023405**	block4	Control	pre	mui35	Subject	Narrow	r2	330.4903304	2	2	pre_focus	35	Narrow pre_focus
**2023405**	block4	Control	pre	tsan3	Verb	Narrow	r2	321.6554831	3	1	pre_focus	3	Narrow pre_focus
**2023405**	block4	Control	pre	jin3	Object	Narrow	r2	271.3617797	4	1	on_focus	3	Narrow on_focus
**2023405**	block4	Control	pre	jin3	Object	Narrow	r2	375.7607673	5	2	on_focus	3	Narrow on_focus
**2023405**	block4	Control	pre	piu35	Subject	Narrow	r2	216.1976988	1	1	on_focus	35	Narrow on_focus
**2023405**	block4	Control	pre	mui35	Subject	Narrow	r2	339.4353497	2	2	on_focus	35	Narrow on_focus
**2023405**	block4	Control	pre	tsan3	Verb	Narrow	r2	315.5716912	3	1	post_focus	3	Narrow post_focus
**2023405**	block4	Control	pre	jin3	Object	Narrow	r2	243.6247808	4	1	post_focus	3	Narrow post_focus
**2023405**	block4	Control	pre	jin3	Object	Narrow	r2	371.5045475	5	2	post_focus	3	Narrow post_focus
**2023405**	block4	Control	pre	Jan-01	Subject	Narrow	r2	232.4460864	1	1	on_focus	1	Narrow on_focus
**2023405**	block4	Control	pre	Jan-01	Subject	Narrow	r2	197.3908659	2	2	on_focus	1	Narrow on_focus
**2023405**	block4	Control	pre	wei3	Verb	Narrow	r2	233.4906145	3	1	post_focus	3	Narrow post_focus
**2023405**	block4	Control	pre	tsam3	Object	Narrow	r2	268.4715025	4	1	post_focus	3	Narrow post_focus
**2023405**	block4	Control	pre	tsam3	Object	Narrow	r2	334.0125214	5	2	post_focus	3	Narrow post_focus
**2023405**	block4	Control	pre	piu35	Subject	Broad	r2	152.0545706	1	1	broad_focus	35	Broad focus
**2023405**	block4	Control	pre	mui35	Subject	Broad	r2	277.7264595	2	2	broad_focus	35	Broad focus
**2023405**	block4	Control	pre	tsan3	Verb	Broad	r2	306.7286857	3	1	broad_focus	3	Broad focus
**2023405**	block4	Control	pre	jin3	Object	Broad	r2	276.5107029	4	1	broad_focus	3	Broad focus
**2023405**	block4	Control	pre	jin3	Object	Broad	r2	428.4455305	5	2	broad_focus	3	Broad focus
**2023405**	block4	Control	pre	pak3	Subject	Narrow	r2	121.9275691	1	1	pre_focus	3	Narrow pre_focus
**2023405**	block4	Control	pre	pak3	Subject	Narrow	r2	138.5185761	2	2	pre_focus	3	Narrow pre_focus
**2023405**	block4	Control	pre	tsing2	Verb	Narrow	r2	219.7977143	3	1	pre_focus	2	Narrow pre_focus
**2023405**	block4	Control	pre	kau2	Object	Narrow	r2	287.4346023	4	1	on_focus	2	Narrow on_focus
**2023405**	block4	Control	pre	tsi2	Object	Narrow	r2	120.8327807	5	2	on_focus	2	Narrow on_focus
**2023405**	block4	Control	pre	piu35	Subject	Contrastive	r2	172.9778818	1	1	pre_focus	35	Contrastive pre_focus
**2023405**	block4	Control	pre	mui35	Subject	Contrastive	r2	310.3271578	2	2	pre_focus	35	Contrastive pre_focus
**2023405**	block4	Control	pre	tsan3	Verb	Contrastive	r2	275.2219551	3	1	pre_focus	3	Contrastive pre_focus
**2023405**	block4	Control	pre	jin3	Object	Contrastive	r2	311.4628684	4	1	on_focus	3	Contrastive on_focus
**2023405**	block4	Control	pre	jin3	Object	Contrastive	r2	270.6383014	5	2	on_focus	3	Contrastive on_focus
**2023405**	block4	Control	pre	pak3	Subject	Narrow	r2	109.6687758	1	1	on_focus	3	Narrow on_focus
**2023405**	block4	Control	pre	pak3	Subject	Narrow	r2	135.4679231	2	2	on_focus	3	Narrow on_focus
**2023405**	block4	Control	pre	tsing2	Verb	Narrow	r2	181.2023023	3	1	post_focus	2	Narrow post_focus
**2023405**	block4	Control	pre	kau2	Object	Narrow	r2	244.8061757	4	1	post_focus	2	Narrow post_focus
**2023405**	block4	Control	pre	tsi2	Object	Narrow	r2	259.6213988	5	2	post_focus	2	Narrow post_focus

**2023405**	block4	Control	pre	piu35	Subject	Narrow	r2	191.0292746	1	1	pre_focus	35	Narrow pre_focus
**2023405**	block4	Control	pre	mui35	Subject	Narrow	r2	283.1940265	2	2	pre_focus	35	Narrow pre_focus
**2023405**	block4	Control	pre	tsan3	Verb	Narrow	r2	312.382861	3	1	on_focus	3	Narrow on_focus
**2023405**	block4	Control	pre	jin3	Object	Narrow	r2	240.8805215	4	1	post_focus	3	Narrow post_focus
**2023405**	block4	Control	pre	jin3	Object	Narrow	r2	429.7407997	5	2	post_focus	3	Narrow post_focus
**2023405**	block4	Control	pre	pak3	Subject	Contrastive	r2	139.2458005	1	1	on_focus	3	Contrastive on_focus
**2023405**	block4	Control	pre	pak3	Subject	Contrastive	r2	141.5124522	2	2	on_focus	3	Contrastive on_focus
**2023405**	block4	Control	pre	tsing2	Verb	Contrastive	r2	229.6731935	3	1	post_focus	2	Contrastive post_focus
**2023405**	block4	Control	pre	kau2	Object	Contrastive	r2	281.6906081	4	1	post_focus	2	Contrastive post_focus
**2023405**	block4	Control	pre	tsi2	Object	Contrastive	r2	216.1518291	5	2	post_focus	2	Contrastive post_focus
**2023405**	block4	Control	pre	piu35	Subject	Contrastive	r2	132.2297992	1	1	on_focus	35	Contrastive on_focus
**2023405**	block4	Control	pre	mui35	Subject	Contrastive	r2	301.3257403	2	2	on_focus	35	Contrastive on_focus
**2023405**	block4	Control	pre	tsan3	Verb	Contrastive	r2	254.8011396	3	1	post_focus	3	Contrastive post_focus
**2023405**	block4	Control	pre	jin3	Object	Contrastive	r2	252.0886284	4	1	post_focus	3	Contrastive post_focus
**2023405**	block4	Control	pre	jin3	Object	Contrastive	r2	363.0274714	5	2	post_focus	3	Contrastive post_focus
**2023405**	block5	Control	post	siu2	Subject	Narrow	r1	255.9007232	1	1	pre_focus	2	Narrow pre_focus
**2023405**	block5	Control	post	gwong2	Subject	Narrow	r1	367.2487606	2	2	pre_focus	2	Narrow pre_focus
**2023405**	block5	Control	post	cyun4	Verb	Narrow	r1	214.3530803	3	1	pre_focus	4	Narrow pre_focus
**2023405**	block5	Control	post	laam4	Object	Narrow	r1	280.2413051	4	1	on_focus	4	Narrow on_focus
**2023405**	block5	Control	post	kau4	Object	Narrow	r1	304.0057187	5	2	on_focus	4	Narrow on_focus
**2023405**	block5	Control	post	siu2	Subject	Contrastive	r1	456.2513055	1	1	on_focus	2	Contrastive on_focus
**2023405**	block5	Control	post	gwong2	Subject	Contrastive	r1	389.1801338	2	2	on_focus	2	Contrastive on_focus
**2023405**	block5	Control	post	cyun4	Verb	Contrastive	r1	313.1869469	3	1	post_focus	4	Contrastive post_focus
**2023405**	block5	Control	post	laam4	Object	Contrastive	r1	297.3374889	4	1	post_focus	4	Contrastive post_focus
**2023405**	block5	Control	post	kau4	Object	Contrastive	r1	498.9824669	5	2	post_focus	4	Contrastive post_focus
**2023405**	block5	Control	post	wai5	Subject	Narrow	r1	206.0569315	1	1	pre_focus	5	Narrow pre_focus
**2023405**	block5	Control	post	wai5	Subject	Narrow	r1	309.6130117	2	2	pre_focus	5	Narrow pre_focus
**2023405**	block5	Control	post	waat3	Verb	Narrow	r1	206.2755132	3	1	on_focus	3	Narrow on_focus
**2023405**	block5	Control	post	bui3	Object	Narrow	r1	241.1220219	4	1	post_focus	3	Narrow post_focus
**2023405**	block5	Control	post	hok3	Object	Narrow	r1	158.8643093	5	2	post_focus	3	Narrow post_focus
**2023405**	block5	Control	post	siu2	Subject	Contrastive	r1	167.8536966	1	1	pre_focus	2	Contrastive pre_focus
**2023405**	block5	Control	post	gwong2	Subject	Contrastive	r1	284.182002	2	2	pre_focus	2	Contrastive pre_focus
**2023405**	block5	Control	post	cyun4	Verb	Contrastive	r1	185.4830772	3	1	on_focus	4	Contrastive on_focus
**2023405**	block5	Control	post	laam4	Object	Contrastive	r1	249.4024313	4	1	post_focus	4	Contrastive post_focus
**2023405**	block5	Control	post	kau4	Object	Contrastive	r1	209.8570629	5	2	post_focus	4	Contrastive post_focus
**2023405**	block5	Control	post	ceoi3	Subject	Contrastive	r1	190.5352321	1	1	pre_focus	3	Contrastive pre_focus
**2023405**	block5	Control	post	ceoi3	Subject	Contrastive	r1	196.8604327	2	2	pre_focus	3	Contrastive pre_focus
**2023405**	block5	Control	post	caa4	Verb	Contrastive	r1	139.4726361	3	1	on_focus	4	Contrastive on_focus
**2023405**	block5	Control	post	ngau4	Object	Contrastive	r1	267.3633253	4	1	post_focus	4	Contrastive post_focus
**2023405**	block5	Control	post	jau4	Object	Contrastive	r1	303.8234147	5	2	post_focus	4	Contrastive post_focus
**2023405**	block5	Control	post	siu2	Subject	Narrow	r1	264.5344272	1	1	on_focus	2	Narrow on_focus
**2023405**	block5	Control	post	gwong2	Subject	Narrow	r1	286.3766484	2	2	on_focus	2	Narrow on_focus
**2023405**	block5	Control	post	cyun4	Verb	Narrow	r1	202.039841	3	1	post_focus	4	Narrow post_focus
**2023405**	block5	Control	post	laam4	Object	Narrow	r1	201.8451056	4	1	post_focus	4	Narrow post_focus
**2023405**	block5	Control	post	kau4	Object	Narrow	r1	191.912568	5	2	post_focus	4	Narrow post_focus
**2023405**	block5	Control	post	wai5	Subject	Contrastive	r1	132.9072176	1	1	pre_focus	5	Contrastive pre_focus
**2023405**	block5	Control	post	wai5	Subject	Contrastive	r1	259.2399949	2	2	pre_focus	5	Contrastive pre_focus
**2023405**	block5	Control	post	waat3	Verb	Contrastive	r1	221.2930995	3	1	on_focus	3	Contrastive on_focus
**2023405**	block5	Control	post	bui3	Object	Contrastive	r1	190.3046284	4	1	post_focus	3	Contrastive post_focus
**2023405**	block5	Control	post	hok3	Object	Contrastive	r1	159.287068	5	2	post_focus	3	Contrastive post_focus
**2023405**	block5	Control	post	wai5	Subject	Contrastive	r1	175.3637645	1	1	pre_focus	5	Contrastive pre_focus
**2023405**	block5	Control	post	wai5	Subject	Contrastive	r1	223.5910602	2	2	pre_focus	5	Contrastive pre_focus
**2023405**	block5	Control	post	waat3	Verb	Contrastive	r1	163.7719986	3	1	pre_focus	3	Contrastive pre_focus
**2023405**	block5	Control	post	bui3	Object	Contrastive	r1	168.8331304	4	1	on_focus	3	Contrastive on_focus
**2023405**	block5	Control	post	hok3	Object	Contrastive	r1	177.2099141	5	2	on_focus	3	Contrastive on_focus
**2023405**	block5	Control	post	siu2	Subject	Broad	r1	184.8019091	1	1	broad_focus	2	Broad focus
**2023405**	block5	Control	post	gwong2	Subject	Broad	r1	251.1351253	2	2	broad_focus	2	Broad focus
**2023405**	block5	Control	post	cyun4	Verb	Broad	r1	197.9758768	3	1	broad_focus	4	Broad focus
**2023405**	block5	Control	post	laam4	Object	Broad	r1	141.2223935	4	1	broad_focus	4	Broad focus
**2023405**	block5	Control	post	kau4	Object	Broad	r1	199.2011383	5	2	broad_focus	4	Broad focus
**2023405**	block5	Control	post	ceoi3	Subject	Broad	r1	176.6017436	1	1	broad_focus	3	Broad focus
**2023405**	block5	Control	post	ceoi3	Subject	Broad	r1	166.1639882	2	2	broad_focus	3	Broad focus
**2023405**	block5	Control	post	caa4	Verb	Broad	r1	126.4400155	3	1	broad_focus	4	Broad focus
**2023405**	block5	Control	post	ngau4	Object	Broad	r1	281.7292631	4	1	broad_focus	4	Broad focus
**2023405**	block5	Control	post	jau4	Object	Broad	r1	280.9503999	5	2	broad_focus	4	Broad focus
**2023405**	block5	Control	post	wai5	Subject	Broad	r1	246.4270246	1	1	broad_focus	5	Broad focus

**2023405**	block5	Control	post	wai5	Subject	Broad	r1	388.3375143	2	2	broad_focus	5	Broad focus
**2023405**	block5	Control	post	waat3	Verb	Broad	r1	208.9629363	3	1	broad_focus	3	Broad focus
**2023405**	block5	Control	post	bui3	Object	Broad	r1	231.456197	4	1	broad_focus	3	Broad focus
**2023405**	block5	Control	post	hok3	Object	Broad	r1	162.9360061	5	2	broad_focus	3	Broad focus
**2023405**	block5	Control	post	wai5	Subject	Narrow	r1	223.9167242	1	1	pre_focus	5	Narrow pre_focus
**2023405**	block5	Control	post	wai5	Subject	Narrow	r1	256.3878178	2	2	pre_focus	5	Narrow pre_focus
**2023405**	block5	Control	post	waat3	Verb	Narrow	r1	215.4419584	3	1	pre_focus	3	Narrow pre_focus
**2023405**	block5	Control	post	bui3	Object	Narrow	r1	265.2732824	4	1	on_focus	3	Narrow on_focus
**2023405**	block5	Control	post	hok3	Object	Narrow	r1	207.0213566	5	2	on_focus	3	Narrow on_focus
**2023405**	block5	Control	post	siu2	Subject	Narrow	r1	214.1489259	1	1	pre_focus	2	Narrow pre_focus
**2023405**	block5	Control	post	gwong2	Subject	Narrow	r1	248.2615645	2	2	pre_focus	2	Narrow pre_focus
**2023405**	block5	Control	post	cyun4	Verb	Narrow	r1	66.19258124	3	1	on_focus	4	Narrow on_focus
**2023405**	block5	Control	post	laam4	Object	Narrow	r1	184.7774403	4	1	post_focus	4	Narrow post_focus
**2023405**	block5	Control	post	kau4	Object	Narrow	r1	44.93569727	5	2	post_focus	4	Narrow post_focus
**2023405**	block5	Control	post	ceoi3	Subject	Narrow	r1	229.3977779	1	1	on_focus	3	Narrow on_focus
**2023405**	block5	Control	post	ceoi3	Subject	Narrow	r1	217.8707617	2	2	on_focus	3	Narrow on_focus
**2023405**	block5	Control	post	caa4	Verb	Narrow	r1	158.829261	3	1	post_focus	4	Narrow post_focus
**2023405**	block5	Control	post	ngau4	Object	Narrow	r1	207.1538615	4	1	post_focus	4	Narrow post_focus
**2023405**	block5	Control	post	jau4	Object	Narrow	r1	225.0499794	5	2	post_focus	4	Narrow post_focus
**2023405**	block5	Control	post	wai5	Subject	Narrow	r1	312.4486887	1	1	on_focus	5	Narrow on_focus
**2023405**	block5	Control	post	wai5	Subject	Narrow	r1	326.4418571	2	2	on_focus	5	Narrow on_focus
**2023405**	block5	Control	post	waat3	Verb	Narrow	r1	208.9200026	3	1	post_focus	3	Narrow post_focus
**2023405**	block5	Control	post	bui3	Object	Narrow	r1	251.7034461	4	1	post_focus	3	Narrow post_focus
**2023405**	block5	Control	post	hok3	Object	Narrow	r1	138.9025799	5	2	post_focus	3	Narrow post_focus
**2023405**	block5	Control	post	ceoi3	Subject	Contrastive	r1	151.2215817	1	1	pre_focus	3	Contrastive pre_focus
**2023405**	block5	Control	post	ceoi3	Subject	Contrastive	r1	240.6325738	2	2	pre_focus	3	Contrastive pre_focus
**2023405**	block5	Control	post	caa4	Verb	Contrastive	r1	160.2175241	3	1	pre_focus	4	Contrastive pre_focus
**2023405**	block5	Control	post	ngau4	Object	Contrastive	r1	285.9647291	4	1	on_focus	4	Contrastive on_focus
**2023405**	block5	Control	post	jau4	Object	Contrastive	r1	190.8408878	5	2	on_focus	4	Contrastive on_focus
**2023405**	block5	Control	post	ceoi3	Subject	Narrow	r1	143.7424622	1	1	pre_focus	3	Narrow pre_focus
**2023405**	block5	Control	post	ceoi3	Subject	Narrow	r1	192.6971876	2	2	pre_focus	3	Narrow pre_focus
**2023405**	block5	Control	post	caa4	Verb	Narrow	r1	135.371226	3	1	pre_focus	4	Narrow pre_focus
**2023405**	block5	Control	post	ngau4	Object	Narrow	r1	216.5835357	4	1	on_focus	4	Narrow on_focus
**2023405**	block5	Control	post	jau4	Object	Narrow	r1	276.9648006	5	2	on_focus	4	Narrow on_focus
**2023405**	block5	Control	post	wai5	Subject	Contrastive	r1	252.4171772	1	1	on_focus	5	Contrastive on_focus
**2023405**	block5	Control	post	wai5	Subject	Contrastive	r1	304.0479665	2	2	on_focus	5	Contrastive on_focus
**2023405**	block5	Control	post	waat3	Verb	Contrastive	r1	191.0942219	3	1	post_focus	3	Contrastive post_focus
**2023405**	block5	Control	post	bui3	Object	Contrastive	r1	259.2041698	4	1	post_focus	3	Contrastive post_focus
**2023405**	block5	Control	post	hok3	Object	Contrastive	r1	308.868165	5	2	post_focus	3	Contrastive post_focus
**2023405**	block5	Control	post	ceoi3	Subject	Contrastive	r1	150.2294778	1	1	on_focus	3	Contrastive on_focus
**2023405**	block5	Control	post	ceoi3	Subject	Contrastive	r1	198.0505278	2	2	on_focus	3	Contrastive on_focus
**2023405**	block5	Control	post	caa4	Verb	Contrastive	r1	155.988354	3	1	post_focus	4	Contrastive post_focus
**2023405**	block5	Control	post	ngau4	Object	Contrastive	r1	253.7213051	4	1	post_focus	4	Contrastive post_focus
**2023405**	block5	Control	post	jau4	Object	Contrastive	r1	313.2476025	5	2	post_focus	4	Contrastive post_focus
**2023405**	block5	Control	post	ceoi3	Subject	Narrow	r1	154.6466174	1	1	pre_focus	3	Narrow pre_focus
**2023405**	block5	Control	post	ceoi3	Subject	Narrow	r1	205.9538801	2	2	pre_focus	3	Narrow pre_focus
**2023405**	block5	Control	post	caa4	Verb	Narrow	r1	203.4510109	3	1	on_focus	4	Narrow on_focus
**2023405**	block5	Control	post	ngau4	Object	Narrow	r1	171.6845049	4	1	post_focus	4	Narrow post_focus
**2023405**	block5	Control	post	jau4	Object	Narrow	r1	178.3334654	5	2	post_focus	4	Narrow post_focus
**2023405**	block5	Control	post	siu2	Subject	Contrastive	r1	109.2206574	1	1	pre_focus	2	Contrastive pre_focus
**2023405**	block5	Control	post	gwong2	Subject	Contrastive	r1	223.6287218	2	2	pre_focus	2	Contrastive pre_focus
**2023405**	block5	Control	post	cyun4	Verb	Contrastive	r1	133.3548794	3	1	pre_focus	4	Contrastive pre_focus
**2023405**	block5	Control	post	laam4	Object	Contrastive	r1	175.350167	4	1	on_focus	4	Contrastive on_focus
**2023405**	block5	Control	post	kau4	Object	Contrastive	r1	224.3120517	5	2	on_focus	4	Contrastive on_focus
**2023405**	block5	Control	post	wai5	Subject	Contrastive	r2	94.24650828	1	1	pre_focus	5	Contrastive pre_focus
**2023405**	block5	Control	post	wai5	Subject	Contrastive	r2	222.6000588	2	2	pre_focus	5	Contrastive pre_focus
**2023405**	block5	Control	post	waat3	Verb	Contrastive	r2	139.1165754	3	1	on_focus	3	Contrastive on_focus
**2023405**	block5	Control	post	bui3	Object	Contrastive	r2	190.0399735	4	1	post_focus	3	Contrastive post_focus
**2023405**	block5	Control	post	hok3	Object	Contrastive	r2	125.1370433	5	2	post_focus	3	Contrastive post_focus
**2023405**	block5	Control	post	ceoi3	Subject	Narrow	r2	132.6335997	1	1	pre_focus	3	Narrow pre_focus
**2023405**	block5	Control	post	ceoi3	Subject	Narrow	r2	175.4616066	2	2	pre_focus	3	Narrow pre_focus
**2023405**	block5	Control	post	caa4	Verb	Narrow	r2	191.7586181	3	1	pre_focus	4	Narrow pre_focus
**2023405**	block5	Control	post	ngau4	Object	Narrow	r2	146.8433045	4	1	on_focus	4	Narrow on_focus
**2023405**	block5	Control	post	jau4	Object	Narrow	r2	200.4666824	5	2	on_focus	4	Narrow on_focus
**2023405**	block5	Control	post	ceoi3	Subject	Contrastive	r2	126.790725	1	1	pre_focus	3	Contrastive pre_focus
**2023405**	block5	Control	post	ceoi3	Subject	Contrastive	r2	176.7742543	2	2	pre_focus	3	Contrastive pre_focus

**2023405**	block5	Control	post	caa4	Verb	Contrastive	r2	152.0883892	3	1	on_focus	4	Contrastive on_focus
**2023405**	block5	Control	post	ngau4	Object	Contrastive	r2	239.8184462	4	1	post_focus	4	Contrastive post_focus
**2023405**	block5	Control	post	jau4	Object	Contrastive	r2	239.6626854	5	2	post_focus	4	Contrastive post_focus
**2023405**	block5	Control	post	siu2	Subject	Narrow	r2	206.7247874	1	1	pre_focus	2	Narrow pre_focus
**2023405**	block5	Control	post	gwong2	Subject	Narrow	r2	250.977655	2	2	pre_focus	2	Narrow pre_focus
**2023405**	block5	Control	post	cyun4	Verb	Narrow	r2	242.9933773	3	1	on_focus	4	Narrow on_focus
**2023405**	block5	Control	post	laam4	Object	Narrow	r2	261.489173	4	1	post_focus	4	Narrow post_focus
**2023405**	block5	Control	post	kau4	Object	Narrow	r2	299.5321518	5	2	post_focus	4	Narrow post_focus
**2023405**	block5	Control	post	ceoi3	Subject	Narrow	r2	127.3631584	1	1	on_focus	3	Narrow on_focus
**2023405**	block5	Control	post	ceoi3	Subject	Narrow	r2	163.6326247	2	2	on_focus	3	Narrow on_focus
**2023405**	block5	Control	post	caa4	Verb	Narrow	r2	155.7642602	3	1	post_focus	4	Narrow post_focus
**2023405**	block5	Control	post	ngau4	Object	Narrow	r2	201.1424954	4	1	post_focus	4	Narrow post_focus
**2023405**	block5	Control	post	jau4	Object	Narrow	r2	286.7260966	5	2	post_focus	4	Narrow post_focus
**2023405**	block5	Control	post	siu2	Subject	Narrow	r2	228.0845434	1	1	on_focus	2	Narrow on_focus
**2023405**	block5	Control	post	gwong2	Subject	Narrow	r2	244.4516878	2	2	on_focus	2	Narrow on_focus
**2023405**	block5	Control	post	cyun4	Verb	Narrow	r2	148.0710227	3	1	post_focus	4	Narrow post_focus
**2023405**	block5	Control	post	laam4	Object	Narrow	r2	202.1266559	4	1	post_focus	4	Narrow post_focus
**2023405**	block5	Control	post	kau4	Object	Narrow	r2	76.73037128	5	2	post_focus	4	Narrow post_focus
**2023405**	block5	Control	post	ceoi3	Subject	Broad	r2	137.9767394	1	1	broad_focus	3	Broad focus
**2023405**	block5	Control	post	ceoi3	Subject	Broad	r2	193.4356393	2	2	broad_focus	3	Broad focus
**2023405**	block5	Control	post	caa4	Verb	Broad	r2	112.7671243	3	1	broad_focus	4	Broad focus
**2023405**	block5	Control	post	ngau4	Object	Broad	r2	228.9111624	4	1	broad_focus	4	Broad focus
**2023405**	block5	Control	post	jau4	Object	Broad	r2	274.7400323	5	2	broad_focus	4	Broad focus
**2023405**	block5	Control	post	wai5	Subject	Narrow	r2	238.8012633	1	1	on_focus	5	Narrow on_focus
**2023405**	block5	Control	post	wai5	Subject	Narrow	r2	224.3671541	2	2	on_focus	5	Narrow on_focus
**2023405**	block5	Control	post	waat3	Verb	Narrow	r2	159.2990559	3	1	post_focus	3	Narrow post_focus
**2023405**	block5	Control	post	bui3	Object	Narrow	r2	251.6716969	4	1	post_focus	3	Narrow post_focus
**2023405**	block5	Control	post	hok3	Object	Narrow	r2	156.8428676	5	2	post_focus	3	Narrow post_focus
**2023405**	block5	Control	post	siu2	Subject	Contrastive	r2	170.6788527	1	1	pre_focus	2	Contrastive pre_focus
**2023405**	block5	Control	post	gwong2	Subject	Contrastive	r2	215.3352741	2	2	pre_focus	2	Contrastive pre_focus
**2023405**	block5	Control	post	cyun4	Verb	Contrastive	r2	187.4604527	3	1	pre_focus	4	Contrastive pre_focus
**2023405**	block5	Control	post	laam4	Object	Contrastive	r2	243.5534407	4	1	on_focus	4	Contrastive on_focus
**2023405**	block5	Control	post	kau4	Object	Contrastive	r2	181.1945424	5	2	on_focus	4	Contrastive on_focus
**2023405**	block5	Control	post	siu2	Subject	Narrow	r2	108.3095984	1	1	pre_focus	2	Narrow pre_focus
**2023405**	block5	Control	post	gwong2	Subject	Narrow	r2	190.2272527	2	2	pre_focus	2	Narrow pre_focus
**2023405**	block5	Control	post	cyun4	Verb	Narrow	r2	116.090118	3	1	pre_focus	4	Narrow pre_focus
**2023405**	block5	Control	post	laam4	Object	Narrow	r2	210.895867	4	1	on_focus	4	Narrow on_focus
**2023405**	block5	Control	post	kau4	Object	Narrow	r2	109.7027844	5	2	on_focus	4	Narrow on_focus
**2023405**	block5	Control	post	wai5	Subject	Contrastive	r2	209.1940401	1	1	on_focus	5	Contrastive on_focus
**2023405**	block5	Control	post	wai5	Subject	Contrastive	r2	217.4034568	2	2	on_focus	5	Contrastive on_focus
**2023405**	block5	Control	post	waat3	Verb	Contrastive	r2	174.8033213	3	1	post_focus	3	Contrastive post_focus
**2023405**	block5	Control	post	bui3	Object	Contrastive	r2	193.2805342	4	1	post_focus	3	Contrastive post_focus
**2023405**	block5	Control	post	hok3	Object	Contrastive	r2	169.7752126	5	2	post_focus	3	Contrastive post_focus
**2023405**	block5	Control	post	siu2	Subject	Contrastive	r2	207.4675038	1	1	on_focus	2	Contrastive on_focus
**2023405**	block5	Control	post	gwong2	Subject	Contrastive	r2	232.8829128	2	2	on_focus	2	Contrastive on_focus
**2023405**	block5	Control	post	cyun4	Verb	Contrastive	r2	173.0585246	3	1	post_focus	4	Contrastive post_focus
**2023405**	block5	Control	post	laam4	Object	Contrastive	r2	180.8638365	4	1	post_focus	4	Contrastive post_focus
**2023405**	block5	Control	post	kau4	Object	Contrastive	r2	183.630037	5	2	post_focus	4	Contrastive post_focus
**2023405**	block5	Control	post	wai5	Subject	Narrow	r2	194.7791326	1	1	pre_focus	5	Narrow pre_focus
**2023405**	block5	Control	post	wai5	Subject	Narrow	r2	192.0049352	2	2	pre_focus	5	Narrow pre_focus
**2023405**	block5	Control	post	waat3	Verb	Narrow	r2	189.1954178	3	1	pre_focus	3	Narrow pre_focus
**2023405**	block5	Control	post	bui3	Object	Narrow	r2	210.7903534	4	1	on_focus	3	Narrow on_focus
**2023405**	block5	Control	post	hok3	Object	Narrow	r2	158.7261997	5	2	on_focus	3	Narrow on_focus
**2023405**	block5	Control	post	siu2	Subject	Broad	r2	170.8841012	1	1	broad_focus	2	Broad focus
**2023405**	block5	Control	post	gwong2	Subject	Broad	r2	205.2067851	2	2	broad_focus	2	Broad focus
**2023405**	block5	Control	post	cyun4	Verb	Broad	r2	164.4297457	3	1	broad_focus	4	Broad focus
**2023405**	block5	Control	post	laam4	Object	Broad	r2	212.0379191	4	1	broad_focus	4	Broad focus
**2023405**	block5	Control	post	kau4	Object	Broad	r2	196.9420109	5	2	broad_focus	4	Broad focus
**2023405**	block5	Control	post	wai5	Subject	Narrow	r2	192.488552	1	1	pre_focus	5	Narrow pre_focus
**2023405**	block5	Control	post	wai5	Subject	Narrow	r2	149.0927581	2	2	pre_focus	5	Narrow pre_focus
**2023405**	block5	Control	post	waat3	Verb	Narrow	r2	146.5253741	3	1	on_focus	3	Narrow on_focus
**2023405**	block5	Control	post	bui3	Object	Narrow	r2	181.6806509	4	1	post_focus	3	Narrow post_focus
**2023405**	block5	Control	post	hok3	Object	Narrow	r2	170.7835188	5	2	post_focus	3	Narrow post_focus
**2023405**	block5	Control	post	ceoi3	Subject	Contrastive	r2	182.7873641	1	1	on_focus	3	Contrastive on_focus
**2023405**	block5	Control	post	ceoi3	Subject	Contrastive	r2	171.4542532	2	2	on_focus	3	Contrastive on_focus
**2023405**	block5	Control	post	caa4	Verb	Contrastive	r2	140.1749625	3	1	post_focus	4	Contrastive post_focus

**2023405**	block5	Control	post	ngau4	Object	Contrastive	r2	175.5196523	4	1	post_focus	4	Contrastive post_focus
**2023405**	block5	Control	post	jau4	Object	Contrastive	r2	222.2834035	5	2	post_focus	4	Contrastive post_focus
**2023405**	block5	Control	post	wai5	Subject	Contrastive	r2	145.203197	1	1	pre_focus	5	Contrastive pre_focus
**2023405**	block5	Control	post	wai5	Subject	Contrastive	r2	217.6610236	2	2	pre_focus	5	Contrastive pre_focus
**2023405**	block5	Control	post	waat3	Verb	Contrastive	r2	183.6966056	3	1	pre_focus	3	Contrastive pre_focus
**2023405**	block5	Control	post	bui3	Object	Contrastive	r2	217.4454965	4	1	on_focus	3	Contrastive on_focus
**2023405**	block5	Control	post	hok3	Object	Contrastive	r2	157.6343034	5	2	on_focus	3	Contrastive on_focus
**2023405**	block5	Control	post	siu2	Subject	Contrastive	r2	228.6015563	1	1	pre_focus	2	Contrastive pre_focus
**2023405**	block5	Control	post	gwong2	Subject	Contrastive	r2	269.3286583	2	2	pre_focus	2	Contrastive pre_focus
**2023405**	block5	Control	post	cyun4	Verb	Contrastive	r2	135.4278156	3	1	on_focus	4	Contrastive on_focus
**2023405**	block5	Control	post	laam4	Object	Contrastive	r2	319.0787621	4	1	post_focus	4	Contrastive post_focus
**2023405**	block5	Control	post	kau4	Object	Contrastive	r2	155.9683953	5	2	post_focus	4	Contrastive post_focus
**2023405**	block5	Control	post	ceoi3	Subject	Contrastive	r2	199.7676131	1	1	pre_focus	3	Contrastive pre_focus
**2023405**	block5	Control	post	ceoi3	Subject	Contrastive	r2	186.2710246	2	2	pre_focus	3	Contrastive pre_focus
**2023405**	block5	Control	post	caa4	Verb	Contrastive	r2	160.056419	3	1	pre_focus	4	Contrastive pre_focus
**2023405**	block5	Control	post	ngau4	Object	Contrastive	r2	239.4008749	4	1	on_focus	4	Contrastive on_focus
**2023405**	block5	Control	post	jau4	Object	Contrastive	r2	279.1472641	5	2	on_focus	4	Contrastive on_focus
**2023405**	block5	Control	post	ceoi3	Subject	Narrow	r2	148.6614645	1	1	pre_focus	3	Narrow pre_focus
**2023405**	block5	Control	post	ceoi3	Subject	Narrow	r2	177.0792658	2	2	pre_focus	3	Narrow pre_focus
**2023405**	block5	Control	post	caa4	Verb	Narrow	r2	159.8926646	3	1	on_focus	4	Narrow on_focus
**2023405**	block5	Control	post	ngau4	Object	Narrow	r2	255.6890427	4	1	post_focus	4	Narrow post_focus
**2023405**	block5	Control	post	jau4	Object	Narrow	r2	189.3209598	5	2	post_focus	4	Narrow post_focus
**2023405**	block5	Control	post	wai5	Subject	Broad	r2	255.0902358	1	1	broad_focus	5	Broad focus
**2023405**	block5	Control	post	wai5	Subject	Broad	r2	265.1703243	2	2	broad_focus	5	Broad focus
**2023405**	block5	Control	post	waat3	Verb	Broad	r2	186.577433	3	1	broad_focus	3	Broad focus
**2023405**	block5	Control	post	bui3	Object	Broad	r2	239.0573353	4	1	broad_focus	3	Broad focus
**2023405**	block5	Control	post	hok3	Object	Broad	r2	166.1989276	5	2	broad_focus	3	Broad focus
**2023405**	block5	Control	pre	siu2	Subject	Narrow	r1	161.3379119	1	1	on_focus	2	Narrow on_focus
**2023405**	block5	Control	pre	gwong2	Subject	Narrow	r1	212.1566525	2	2	on_focus	2	Narrow on_focus
**2023405**	block5	Control	pre	cyun4	Verb	Narrow	r1	164.5151515	3	1	post_focus	4	Narrow post_focus
**2023405**	block5	Control	pre	laam4	Object	Narrow	r1	292.2220259	4	1	post_focus	4	Narrow post_focus
**2023405**	block5	Control	pre	kau4	Object	Narrow	r1	195.7859715	5	2	post_focus	4	Narrow post_focus
**2023405**	block5	Control	pre	ceoi3	Subject	Narrow	r1	178.5653997	1	1	pre_focus	3	Narrow pre_focus
**2023405**	block5	Control	pre	ceoi3	Subject	Narrow	r1	194.5607303	2	2	pre_focus	3	Narrow pre_focus
**2023405**	block5	Control	pre	caa4	Verb	Narrow	r1	198.7065884	3	1	pre_focus	4	Narrow pre_focus
**2023405**	block5	Control	pre	ngau4	Object	Narrow	r1	212.1373374	4	1	on_focus	4	Narrow on_focus
**2023405**	block5	Control	pre	jau4	Object	Narrow	r1	393.2928997	5	2	on_focus	4	Narrow on_focus
**2023405**	block5	Control	pre	ceoi3	Subject	Contrastive	r1	239.6968126	1	1	pre_focus	3	Contrastive pre_focus
**2023405**	block5	Control	pre	ceoi3	Subject	Contrastive	r1	222.8587491	2	2	pre_focus	3	Contrastive pre_focus
**2023405**	block5	Control	pre	caa4	Verb	Contrastive	r1	234.4067055	3	1	on_focus	4	Contrastive on_focus
**2023405**	block5	Control	pre	ngau4	Object	Contrastive	r1	353.7794931	4	1	post_focus	4	Contrastive post_focus
**2023405**	block5	Control	pre	jau4	Object	Contrastive	r1	343.5046693	5	2	post_focus	4	Contrastive post_focus
**2023405**	block5	Control	pre	wai5	Subject	Narrow	r1	216.6147053	1	1	on_focus	5	Narrow on_focus
**2023405**	block5	Control	pre	wai5	Subject	Narrow	r1	239.6808921	2	2	on_focus	5	Narrow on_focus
**2023405**	block5	Control	pre	waat3	Verb	Narrow	r1	213.3144679	3	1	post_focus	3	Narrow post_focus
**2023405**	block5	Control	pre	bui3	Object	Narrow	r1	172.7562729	4	1	post_focus	3	Narrow post_focus
**2023405**	block5	Control	pre	hok3	Object	Narrow	r1	195.8605006	5	2	post_focus	3	Narrow post_focus
**2023405**	block5	Control	pre	ceoi3	Subject	Broad	r1	165.2738999	1	1	broad_focus	3	Broad focus
**2023405**	block5	Control	pre	ceoi3	Subject	Broad	r1	213.6890028	2	2	broad_focus	3	Broad focus
**2023405**	block5	Control	pre	caa4	Verb	Broad	r1	114.0478357	3	1	broad_focus	4	Broad focus
**2023405**	block5	Control	pre	ngau4	Object	Broad	r1	239.3436648	4	1	broad_focus	4	Broad focus
**2023405**	block5	Control	pre	jau4	Object	Broad	r1	266.7621008	5	2	broad_focus	4	Broad focus
**2023405**	block5	Control	pre	ceoi3	Subject	Narrow	r1	152.605874	1	1	pre_focus	3	Narrow pre_focus
**2023405**	block5	Control	pre	ceoi3	Subject	Narrow	r1	203.9176459	2	2	pre_focus	3	Narrow pre_focus
**2023405**	block5	Control	pre	caa4	Verb	Narrow	r1	221.4622415	3	1	on_focus	4	Narrow on_focus
**2023405**	block5	Control	pre	ngau4	Object	Narrow	r1	292.3919748	4	1	post_focus	4	Narrow post_focus
**2023405**	block5	Control	pre	jau4	Object	Narrow	r1	205.8215946	5	2	post_focus	4	Narrow post_focus
**2023405**	block5	Control	pre	siu2	Subject	Narrow	r1	107.1762537	1	1	pre_focus	2	Narrow pre_focus
**2023405**	block5	Control	pre	gwong2	Subject	Narrow	r1	232.8261453	2	2	pre_focus	2	Narrow pre_focus
**2023405**	block5	Control	pre	cyun4	Verb	Narrow	r1	68.90791353	3	1	pre_focus	4	Narrow pre_focus
**2023405**	block5	Control	pre	laam4	Object	Narrow	r1	182.9517986	4	1	on_focus	4	Narrow on_focus
**2023405**	block5	Control	pre	kau4	Object	Narrow	r1	134.5622545	5	2	on_focus	4	Narrow on_focus
**2023405**	block5	Control	pre	siu2	Subject	Contrastive	r1	272.0476259	1	1	on_focus	2	Contrastive on_focus
**2023405**	block5	Control	pre	gwong2	Subject	Contrastive	r1	286.1499519	2	2	on_focus	2	Contrastive on_focus
**2023405**	block5	Control	pre	cyun4	Verb	Contrastive	r1	178.664584	3	1	post_focus	4	Contrastive post_focus
**2023405**	block5	Control	pre	laam4	Object	Contrastive	r1	174.4610668	4	1	post_focus	4	Contrastive post_focus

**2023405**	block5	Control	pre	kau4	Object	Contrastive	r1	122.9308046	5	2	post_focus	4	Contrastive post_focus
**2023405**	block5	Control	pre	ceoi3	Subject	Narrow	r1	141.1363952	1	1	on_focus	3	Narrow on_focus
**2023405**	block5	Control	pre	ceoi3	Subject	Narrow	r1	163.1086024	2	2	on_focus	3	Narrow on_focus
**2023405**	block5	Control	pre	caa4	Verb	Narrow	r1	95.63103022	3	1	post_focus	4	Narrow post_focus
**2023405**	block5	Control	pre	ngau4	Object	Narrow	r1	119.300425	4	1	post_focus	4	Narrow post_focus
**2023405**	block5	Control	pre	jau4	Object	Narrow	r1	120.238356	5	2	post_focus	4	Narrow post_focus
**2023405**	block5	Control	pre	wai5	Subject	Contrastive	r1	205.4584949	1	1	pre_focus	5	Contrastive pre_focus
**2023405**	block5	Control	pre	wai5	Subject	Contrastive	r1	259.5509993	2	2	pre_focus	5	Contrastive pre_focus
**2023405**	block5	Control	pre	waat3	Verb	Contrastive	r1	202.5403055	3	1	pre_focus	3	Contrastive pre_focus
**2023405**	block5	Control	pre	bui3	Object	Contrastive	r1	163.4650672	4	1	on_focus	3	Contrastive on_focus
**2023405**	block5	Control	pre	hok3	Object	Contrastive	r1	202.8945305	5	2	on_focus	3	Contrastive on_focus
**2023405**	block5	Control	pre	siu2	Subject	Contrastive	r1	204.7959442	1	1	pre_focus	2	Contrastive pre_focus
**2023405**	block5	Control	pre	gwong2	Subject	Contrastive	r1	259.0190354	2	2	pre_focus	2	Contrastive pre_focus
**2023405**	block5	Control	pre	cyun4	Verb	Contrastive	r1	122.1589866	3	1	on_focus	4	Contrastive on_focus
**2023405**	block5	Control	pre	laam4	Object	Contrastive	r1	124.1494953	4	1	post_focus	4	Contrastive post_focus
**2023405**	block5	Control	pre	kau4	Object	Contrastive	r1	131.5207857	5	2	post_focus	4	Contrastive post_focus
**2023405**	block5	Control	pre	wai5	Subject	Broad	r1	193.6766894	1	1	broad_focus	5	Broad focus
**2023405**	block5	Control	pre	wai5	Subject	Broad	r1	284.4791589	2	2	broad_focus	5	Broad focus
**2023405**	block5	Control	pre	waat3	Verb	Broad	r1	245.4511458	3	1	broad_focus	3	Broad focus
**2023405**	block5	Control	pre	bui3	Object	Broad	r1	232.4307334	4	1	broad_focus	3	Broad focus
**2023405**	block5	Control	pre	hok3	Object	Broad	r1	114.8140128	5	2	broad_focus	3	Broad focus
**2023405**	block5	Control	pre	siu2	Subject	Broad	r1	227.3003483	1	1	broad_focus	2	Broad focus
**2023405**	block5	Control	pre	gwong2	Subject	Broad	r1	249.7945006	2	2	broad_focus	2	Broad focus
**2023405**	block5	Control	pre	cyun4	Verb	Broad	r1	77.67229798	3	1	broad_focus	4	Broad focus
**2023405**	block5	Control	pre	laam4	Object	Broad	r1	97.99738969	4	1	broad_focus	4	Broad focus
**2023405**	block5	Control	pre	kau4	Object	Broad	r1	44.07902211	5	2	broad_focus	4	Broad focus
**2023405**	block5	Control	pre	wai5	Subject	Contrastive	r1	226.9180322	1	1	on_focus	5	Contrastive on_focus
**2023405**	block5	Control	pre	wai5	Subject	Contrastive	r1	251.0911922	2	2	on_focus	5	Contrastive on_focus
**2023405**	block5	Control	pre	waat3	Verb	Contrastive	r1	204.5302492	3	1	post_focus	3	Contrastive post_focus
**2023405**	block5	Control	pre	bui3	Object	Contrastive	r1	150.0805732	4	1	post_focus	3	Contrastive post_focus
**2023405**	block5	Control	pre	hok3	Object	Contrastive	r1	289.330415	5	2	post_focus	3	Contrastive post_focus
**2023405**	block5	Control	pre	wai5	Subject	Contrastive	r1	184.6952826	1	1	pre_focus	5	Contrastive pre_focus
**2023405**	block5	Control	pre	wai5	Subject	Contrastive	r1	263.183504	2	2	pre_focus	5	Contrastive pre_focus
**2023405**	block5	Control	pre	waat3	Verb	Contrastive	r1	172.6097889	3	1	on_focus	3	Contrastive on_focus
**2023405**	block5	Control	pre	bui3	Object	Contrastive	r1	181.3293962	4	1	post_focus	3	Contrastive post_focus
**2023405**	block5	Control	pre	hok3	Object	Contrastive	r1	174.6637475	5	2	post_focus	3	Contrastive post_focus
**2023405**	block5	Control	pre	siu2	Subject	Narrow	r1	123.4269661	1	1	pre_focus	2	Narrow pre_focus
**2023405**	block5	Control	pre	gwong2	Subject	Narrow	r1	158.6329094	2	2	pre_focus	2	Narrow pre_focus
**2023405**	block5	Control	pre	cyun4	Verb	Narrow	r1	155.4618492	3	1	on_focus	4	Narrow on_focus
**2023405**	block5	Control	pre	laam4	Object	Narrow	r1	150.6817894	4	1	post_focus	4	Narrow post_focus
**2023405**	block5	Control	pre	kau4	Object	Narrow	r1	66.82924456	5	2	post_focus	4	Narrow post_focus
**2023405**	block5	Control	pre	wai5	Subject	Narrow	r1	183.7386485	1	1	pre_focus	5	Narrow pre_focus
**2023405**	block5	Control	pre	wai5	Subject	Narrow	r1	257.276632	2	2	pre_focus	5	Narrow pre_focus
**2023405**	block5	Control	pre	waat3	Verb	Narrow	r1	122.9926736	3	1	on_focus	3	Narrow on_focus
**2023405**	block5	Control	pre	bui3	Object	Narrow	r1	200.9687683	4	1	post_focus	3	Narrow post_focus
**2023405**	block5	Control	pre	hok3	Object	Narrow	r1	131.1344031	5	2	post_focus	3	Narrow post_focus
**2023405**	block5	Control	pre	ceoi3	Subject	Contrastive	r1	124.4739696	1	1	on_focus	3	Contrastive on_focus
**2023405**	block5	Control	pre	ceoi3	Subject	Contrastive	r1	188.5218242	2	2	on_focus	3	Contrastive on_focus
**2023405**	block5	Control	pre	caa4	Verb	Contrastive	r1	144.0703328	3	1	post_focus	4	Contrastive post_focus
**2023405**	block5	Control	pre	ngau4	Object	Contrastive	r1	103.5092997	4	1	post_focus	4	Contrastive post_focus
**2023405**	block5	Control	pre	jau4	Object	Contrastive	r1	69.84127254	5	2	post_focus	4	Contrastive post_focus
**2023405**	block5	Control	pre	wai5	Subject	Narrow	r1	243.1423148	1	1	pre_focus	5	Narrow pre_focus
**2023405**	block5	Control	pre	wai5	Subject	Narrow	r1	294.6929283	2	2	pre_focus	5	Narrow pre_focus
**2023405**	block5	Control	pre	waat3	Verb	Narrow	r1	198.5999614	3	1	pre_focus	3	Narrow pre_focus
**2023405**	block5	Control	pre	bui3	Object	Narrow	r1	181.5679303	4	1	on_focus	3	Narrow on_focus
**2023405**	block5	Control	pre	hok3	Object	Narrow	r1	257.4936229	5	2	on_focus	3	Narrow on_focus
**2023405**	block5	Control	pre	ceoi3	Subject	Contrastive	r1	149.7171765	1	1	pre_focus	3	Contrastive pre_focus
**2023405**	block5	Control	pre	ceoi3	Subject	Contrastive	r1	239.0502936	2	2	pre_focus	3	Contrastive pre_focus
**2023405**	block5	Control	pre	caa4	Verb	Contrastive	r1	68.59475896	3	1	pre_focus	4	Contrastive pre_focus
**2023405**	block5	Control	pre	ngau4	Object	Contrastive	r1	273.7427159	4	1	on_focus	4	Contrastive on_focus
**2023405**	block5	Control	pre	jau4	Object	Contrastive	r1	157.9998903	5	2	on_focus	4	Contrastive on_focus
**2023405**	block5	Control	pre	siu2	Subject	Contrastive	r1	184.7527553	1	1	pre_focus	2	Contrastive pre_focus
**2023405**	block5	Control	pre	gwong2	Subject	Contrastive	r1	224.1527785	2	2	pre_focus	2	Contrastive pre_focus
**2023405**	block5	Control	pre	cyun4	Verb	Contrastive	r1	78.20801471	3	1	pre_focus	4	Contrastive pre_focus
**2023405**	block5	Control	pre	laam4	Object	Contrastive	r1	192.8440341	4	1	on_focus	4	Contrastive on_focus
**2023405**	block5	Control	pre	kau4	Object	Contrastive	r1	66.25027586	5	2	on_focus	4	Contrastive on_focus

**2023405**	block5	Control	pre	siu2	Subject	Narrow	r2	146.0493484	1	1	on_focus	2	Narrow on_focus
**2023405**	block5	Control	pre	gwong2	Subject	Narrow	r2	181.6141562	2	2	on_focus	2	Narrow on_focus
**2023405**	block5	Control	pre	cyun4	Verb	Narrow	r2	174.4751832	3	1	post_focus	4	Narrow post_focus
**2023405**	block5	Control	pre	laam4	Object	Narrow	r2	89.17940705	4	1	post_focus	4	Narrow post_focus
**2023405**	block5	Control	pre	kau4	Object	Narrow	r2	141.6492035	5	2	post_focus	4	Narrow post_focus
**2023405**	block5	Control	pre	ceoi3	Subject	Narrow	r2	156.0697286	1	1	pre_focus	3	Narrow pre_focus
**2023405**	block5	Control	pre	ceoi3	Subject	Narrow	r2	166.8447855	2	2	pre_focus	3	Narrow pre_focus
**2023405**	block5	Control	pre	caa4	Verb	Narrow	r2	144.2747602	3	1	on_focus	4	Narrow on_focus
**2023405**	block5	Control	pre	ngau4	Object	Narrow	r2	79.07787128	4	1	post_focus	4	Narrow post_focus
**2023405**	block5	Control	pre	jau4	Object	Narrow	r2	104.4205498	5	2	post_focus	4	Narrow post_focus
**2023405**	block5	Control	pre	siu2	Subject	Contrastive	r2	208.1258356	1	1	on_focus	2	Contrastive on_focus
**2023405**	block5	Control	pre	gwong2	Subject	Contrastive	r2	241.8412974	2	2	on_focus	2	Contrastive on_focus
**2023405**	block5	Control	pre	cyun4	Verb	Contrastive	r2	81.68126088	3	1	post_focus	4	Contrastive post_focus
**2023405**	block5	Control	pre	laam4	Object	Contrastive	r2	141.1119014	4	1	post_focus	4	Contrastive post_focus
**2023405**	block5	Control	pre	kau4	Object	Contrastive	r2	56.50622848	5	2	post_focus	4	Contrastive post_focus
**2023405**	block5	Control	pre	ceoi3	Subject	Contrastive	r2	196.8459072	1	1	on_focus	3	Contrastive on_focus
**2023405**	block5	Control	pre	ceoi3	Subject	Contrastive	r2	212.0510312	2	2	on_focus	3	Contrastive on_focus
**2023405**	block5	Control	pre	caa4	Verb	Contrastive	r2	143.6553782	3	1	post_focus	4	Contrastive post_focus
**2023405**	block5	Control	pre	ngau4	Object	Contrastive	r2	126.9617681	4	1	post_focus	4	Contrastive post_focus
**2023405**	block5	Control	pre	jau4	Object	Contrastive	r2	180.9106244	5	2	post_focus	4	Contrastive post_focus
**2023405**	block5	Control	pre	wai5	Subject	Narrow	r2	262.8103835	1	1	on_focus	5	Narrow on_focus
**2023405**	block5	Control	pre	wai5	Subject	Narrow	r2	223.2515614	2	2	on_focus	5	Narrow on_focus
**2023405**	block5	Control	pre	waat3	Verb	Narrow	r2	160.5187316	3	1	post_focus	3	Narrow post_focus
**2023405**	block5	Control	pre	bui3	Object	Narrow	r2	186.8540649	4	1	post_focus	3	Narrow post_focus
**2023405**	block5	Control	pre	hok3	Object	Narrow	r2	195.8559038	5	2	post_focus	3	Narrow post_focus
**2023405**	block5	Control	pre	wai5	Subject	Contrastive	r2	171.1275039	1	1	pre_focus	5	Contrastive pre_focus
**2023405**	block5	Control	pre	wai5	Subject	Contrastive	r2	225.6655371	2	2	pre_focus	5	Contrastive pre_focus
**2023405**	block5	Control	pre	waat3	Verb	Contrastive	r2	215.6315419	3	1	on_focus	3	Contrastive on_focus
**2023405**	block5	Control	pre	bui3	Object	Contrastive	r2	176.9072278	4	1	post_focus	3	Contrastive post_focus
**2023405**	block5	Control	pre	hok3	Object	Contrastive	r2	166.2759537	5	2	post_focus	3	Contrastive post_focus
**2023405**	block5	Control	pre	siu2	Subject	Contrastive	r2	145.9719706	1	1	pre_focus	2	Contrastive pre_focus
**2023405**	block5	Control	pre	gwong2	Subject	Contrastive	r2	263.5823073	2	2	pre_focus	2	Contrastive pre_focus
**2023405**	block5	Control	pre	cyun4	Verb	Contrastive	r2	104.3045403	3	1	pre_focus	4	Contrastive pre_focus
**2023405**	block5	Control	pre	laam4	Object	Contrastive	r2	97.36785794	4	1	on_focus	4	Contrastive on_focus
**2023405**	block5	Control	pre	kau4	Object	Contrastive	r2	36.75521002	5	2	on_focus	4	Contrastive on_focus
**2023405**	block5	Control	pre	ceoi3	Subject	Narrow	r2	222.7159798	1	1	pre_focus	3	Narrow pre_focus
**2023405**	block5	Control	pre	ceoi3	Subject	Narrow	r2	207.9471705	2	2	pre_focus	3	Narrow pre_focus
**2023405**	block5	Control	pre	caa4	Verb	Narrow	r2	68.22861555	3	1	pre_focus	4	Narrow pre_focus
**2023405**	block5	Control	pre	ngau4	Object	Narrow	r2	72.56150489	4	1	on_focus	4	Narrow on_focus
**2023405**	block5	Control	pre	jau4	Object	Narrow	r2	69.49217034	5	2	on_focus	4	Narrow on_focus
**2023405**	block5	Control	pre	siu2	Subject	Broad	r2	243.609871	1	1	broad_focus	2	Broad focus
**2023405**	block5	Control	pre	gwong2	Subject	Broad	r2	259.6571581	2	2	broad_focus	2	Broad focus
**2023405**	block5	Control	pre	cyun4	Verb	Broad	r2	39.79606681	3	1	broad_focus	4	Broad focus
**2023405**	block5	Control	pre	laam4	Object	Broad	r2	119.8976403	4	1	broad_focus	4	Broad focus
**2023405**	block5	Control	pre	kau4	Object	Broad	r2	121.9360899	5	2	broad_focus	4	Broad focus
**2023405**	block5	Control	pre	wai5	Subject	Contrastive	r2	197.3535936	1	1	on_focus	5	Contrastive on_focus
**2023405**	block5	Control	pre	wai5	Subject	Contrastive	r2	241.4311448	2	2	on_focus	5	Contrastive on_focus
**2023405**	block5	Control	pre	waat3	Verb	Contrastive	r2	188.1265693	3	1	post_focus	3	Contrastive post_focus
**2023405**	block5	Control	pre	bui3	Object	Contrastive	r2	179.8353737	4	1	post_focus	3	Contrastive post_focus
**2023405**	block5	Control	pre	hok3	Object	Contrastive	r2	277.8010774	5	2	post_focus	3	Contrastive post_focus
**2023405**	block5	Control	pre	siu2	Subject	Narrow	r2	111.5577415	1	1	pre_focus	2	Narrow pre_focus
**2023405**	block5	Control	pre	gwong2	Subject	Narrow	r2	254.7175373	2	2	pre_focus	2	Narrow pre_focus
**2023405**	block5	Control	pre	cyun4	Verb	Narrow	r2	103.613616	3	1	pre_focus	4	Narrow pre_focus
**2023405**	block5	Control	pre	laam4	Object	Narrow	r2	138.4615875	4	1	on_focus	4	Narrow on_focus
**2023405**	block5	Control	pre	kau4	Object	Narrow	r2	51.44861277	5	2	on_focus	4	Narrow on_focus
**2023405**	block5	Control	pre	wai5	Subject	Narrow	r2	87.81441309	1	1	pre_focus	5	Narrow pre_focus
**2023405**	block5	Control	pre	wai5	Subject	Narrow	r2	152.6767048	2	2	pre_focus	5	Narrow pre_focus
**2023405**	block5	Control	pre	waat3	Verb	Narrow	r2	123.6241975	3	1	on_focus	3	Narrow on_focus
**2023405**	block5	Control	pre	bui3	Object	Narrow	r2	166.8650334	4	1	post_focus	3	Narrow post_focus
**2023405**	block5	Control	pre	hok3	Object	Narrow	r2	136.1035805	5	2	post_focus	3	Narrow post_focus
**2023405**	block5	Control	pre	ceoi3	Subject	Narrow	r2	142.96734	1	1	on_focus	3	Narrow on_focus
**2023405**	block5	Control	pre	ceoi3	Subject	Narrow	r2	139.2465201	2	2	on_focus	3	Narrow on_focus
**2023405**	block5	Control	pre	caa4	Verb	Narrow	r2	110.4469133	3	1	post_focus	4	Narrow post_focus
**2023405**	block5	Control	pre	ngau4	Object	Narrow	r2	110.8257667	4	1	post_focus	4	Narrow post_focus
**2023405**	block5	Control	pre	jau4	Object	Narrow	r2	104.4923229	5	2	post_focus	4	Narrow post_focus
**2023405**	block5	Control	pre	siu2	Subject	Contrastive	r2	233.9949119	1	1	pre_focus	2	Contrastive pre_focus

**2023405**	block5	Control	pre	gwong2	Subject	Contrastive	r2	130.9111594	2	2	pre_focus	2	Contrastive pre_focus
**2023405**	block5	Control	pre	cyun4	Verb	Contrastive	r2	59.9059909	3	1	on_focus	4	Contrastive on_focus
**2023405**	block5	Control	pre	laam4	Object	Contrastive	r2	115.8957652	4	1	post_focus	4	Contrastive post_focus
**2023405**	block5	Control	pre	kau4	Object	Contrastive	r2	122.3304395	5	2	post_focus	4	Contrastive post_focus
**2023405**	block5	Control	pre	ceoi3	Subject	Contrastive	r2	128.8327388	1	1	pre_focus	3	Contrastive pre_focus
**2023405**	block5	Control	pre	ceoi3	Subject	Contrastive	r2	231.4583378	2	2	pre_focus	3	Contrastive pre_focus
**2023405**	block5	Control	pre	caa4	Verb	Contrastive	r2	78.56574989	3	1	pre_focus	4	Contrastive pre_focus
**2023405**	block5	Control	pre	ngau4	Object	Contrastive	r2	104.3681575	4	1	on_focus	4	Contrastive on_focus
**2023405**	block5	Control	pre	jau4	Object	Contrastive	r2	113.0390863	5	2	on_focus	4	Contrastive on_focus
**2023405**	block5	Control	pre	wai5	Subject	Contrastive	r2	210.928252	1	1	pre_focus	5	Contrastive pre_focus
**2023405**	block5	Control	pre	wai5	Subject	Contrastive	r2	276.099572	2	2	pre_focus	5	Contrastive pre_focus
**2023405**	block5	Control	pre	waat3	Verb	Contrastive	r2	158.8240239	3	1	pre_focus	3	Contrastive pre_focus
**2023405**	block5	Control	pre	bui3	Object	Contrastive	r2	87.76402632	4	1	on_focus	3	Contrastive on_focus
**2023405**	block5	Control	pre	hok3	Object	Contrastive	r2	254.0521906	5	2	on_focus	3	Contrastive on_focus
**2023405**	block5	Control	pre	wai5	Subject	Narrow	r2	169.8048755	1	1	pre_focus	5	Narrow pre_focus
**2023405**	block5	Control	pre	wai5	Subject	Narrow	r2	231.6950908	2	2	pre_focus	5	Narrow pre_focus
**2023405**	block5	Control	pre	waat3	Verb	Narrow	r2	180.6715381	3	1	pre_focus	3	Narrow pre_focus
**2023405**	block5	Control	pre	bui3	Object	Narrow	r2	180.5314209	4	1	on_focus	3	Narrow on_focus
**2023405**	block5	Control	pre	hok3	Object	Narrow	r2	167.5782946	5	2	on_focus	3	Narrow on_focus
**2023405**	block5	Control	pre	siu2	Subject	Narrow	r2	171.1225592	1	1	pre_focus	2	Narrow pre_focus
**2023405**	block5	Control	pre	gwong2	Subject	Narrow	r2	262.4845916	2	2	pre_focus	2	Narrow pre_focus
**2023405**	block5	Control	pre	cyun4	Verb	Narrow	r2	185.4870656	3	1	on_focus	4	Narrow on_focus
**2023405**	block5	Control	pre	laam4	Object	Narrow	r2	202.6140909	4	1	post_focus	4	Narrow post_focus
**2023405**	block5	Control	pre	kau4	Object	Narrow	r2	80.56660537	5	2	post_focus	4	Narrow post_focus
**2023405**	block5	Control	pre	ceoi3	Subject	Broad	r2	161.2586512	1	1	broad_focus	3	Broad focus
**2023405**	block5	Control	pre	ceoi3	Subject	Broad	r2	193.5470766	2	2	broad_focus	3	Broad focus
**2023405**	block5	Control	pre	caa4	Verb	Broad	r2	130.8302842	3	1	broad_focus	4	Broad focus
**2023405**	block5	Control	pre	ngau4	Object	Broad	r2	124.1565694	4	1	broad_focus	4	Broad focus
**2023405**	block5	Control	pre	jau4	Object	Broad	r2	196.6876671	5	2	broad_focus	4	Broad focus
**2023405**	block5	Control	pre	wai5	Subject	Broad	r2	186.9725229	1	1	broad_focus	5	Broad focus
**2023405**	block5	Control	pre	wai5	Subject	Broad	r2	306.9554011	2	2	broad_focus	5	Broad focus
**2023405**	block5	Control	pre	waat3	Verb	Broad	r2	163.5700528	3	1	broad_focus	3	Broad focus
**2023405**	block5	Control	pre	bui3	Object	Broad	r2	193.3057392	4	1	broad_focus	3	Broad focus
**2023405**	block5	Control	pre	hok3	Object	Broad	r2	161.9182716	5	2	broad_focus	3	Broad focus
**2023405**	block5	Control	pre	ceoi3	Subject	Contrastive	r2	166.5344003	1	1	pre_focus	3	Contrastive pre_focus
**2023405**	block5	Control	pre	ceoi3	Subject	Contrastive	r2	193.4557733	2	2	pre_focus	3	Contrastive pre_focus
**2023405**	block5	Control	pre	caa4	Verb	Contrastive	r2	38.61139742	3	1	on_focus	4	Contrastive on_focus
**2023405**	block5	Control	pre	ngau4	Object	Contrastive	r2	105.6742987	4	1	post_focus	4	Contrastive post_focus
**2023405**	block5	Control	pre	jau4	Object	Contrastive	r2	106.7509115	5	2	post_focus	4	Contrastive post_focus
**2023407**	block1	Control	post	sau3	Subject	Contrastive	r1	76.71237009	1	1	pre_focus	3	Contrastive pre_focus
**2023407**	block1	Control	post	sau3	Subject	Contrastive	r1	249.3774835	2	2	pre_focus	3	Contrastive pre_focus
**2023407**	block1	Control	post	sik3	Verb	Contrastive	r1	75.86601307	3	1	pre_focus	3	Contrastive pre_focus
**2023407**	block1	Control	post	baak3	Object	Contrastive	r1	75.58843829	4	1	on_focus	3	Contrastive on_focus
**2023407**	block1	Control	post	baak3	Object	Contrastive	r1	115.9756431	5	2	on_focus	3	Contrastive on_focus
**2023407**	block1	Control	post	sau3	Subject	Contrastive	r1	75.74425008	1	1	pre_focus	3	Contrastive pre_focus
**2023407**	block1	Control	post	sau3	Subject	Contrastive	r1	254.4592467	2	2	pre_focus	3	Contrastive pre_focus
**2023407**	block1	Control	post	sik3	Verb	Contrastive	r1	71.21308172	3	1	on_focus	3	Contrastive on_focus
**2023407**	block1	Control	post	baak3	Object	Contrastive	r1	74.79876903	4	1	post_focus	3	Contrastive post_focus
**2023407**	block1	Control	post	baak3	Object	Contrastive	r1	199.3679138	5	2	post_focus	3	Contrastive post_focus
**2023407**	block1	Control	post	zoeng1	Subject	Narrow	r1	124.6394558	1	1	on_focus	1	Narrow on_focus
**2023407**	block1	Control	post	saang1	Subject	Narrow	r1	228.0464869	2	2	on_focus	1	Narrow on_focus
**2023407**	block1	Control	post	tsa1	Verb	Narrow	r1	168.3334667	3	1	post_focus	1	Narrow post_focus
**2023407**	block1	Control	post	fei1	Object	Narrow	r1	123.7564248	4	1	post_focus	1	Narrow post_focus
**2023407**	block1	Control	post	gei1	Object	Narrow	r1	444.9194654	5	2	post_focus	1	Narrow post_focus
**2023407**	block1	Control	post	jyun2	Subject	Narrow	r1	178.462801	1	1	pre_focus	2	Narrow pre_focus
**2023407**	block1	Control	post	jyun2	Subject	Narrow	r1	213.1862078	2	2	pre_focus	2	Narrow pre_focus
**2023407**	block1	Control	post	mo2	Verb	Narrow	r1	240.6290258	3	1	on_focus	2	Narrow on_focus
**2023407**	block1	Control	post	gau2	Object	Narrow	r1	116.6080877	4	1	post_focus	2	Narrow post_focus
**2023407**	block1	Control	post	zai2	Object	Narrow	r1	443.3434114	5	2	post_focus	2	Narrow post_focus
**2023407**	block1	Control	post	jyun2	Subject	Narrow	r1	214.5330202	1	1	on_focus	2	Narrow on_focus
**2023407**	block1	Control	post	jyun2	Subject	Narrow	r1	243.2603201	2	2	on_focus	2	Narrow on_focus
**2023407**	block1	Control	post	mo2	Verb	Narrow	r1	277.4928676	3	1	post_focus	2	Narrow post_focus
**2023407**	block1	Control	post	gau2	Object	Narrow	r1	148.5033506	4	1	post_focus	2	Narrow post_focus
**2023407**	block1	Control	post	zai2	Object	Narrow	r1	522.742892	5	2	post_focus	2	Narrow post_focus
**2023407**	block1	Control	post	jyun2	Subject	Contrastive	r1	210.7573327	1	1	pre_focus	2	Contrastive pre_focus
**2023407**	block1	Control	post	jyun2	Subject	Contrastive	r1	364.1509279	2	2	pre_focus	2	Contrastive pre_focus

**2023407**	block1	Control	post	mo2	Verb	Contrastive	r1	223.2010582	3	1	on_focus	2	Contrastive on_focus
**2023407**	block1	Control	post	gau2	Object	Contrastive	r1	180.4771133	4	1	post_focus	2	Contrastive post_focus
**2023407**	block1	Control	post	zai2	Object	Contrastive	r1	436.4279319	5	2	post_focus	2	Contrastive post_focus
**2023407**	block1	Control	post	jyun2	Subject	Broad	r1	271.4724415	1	1	broad_focus	2	Broad focus
**2023407**	block1	Control	post	jyun2	Subject	Broad	r1	339.1406536	2	2	broad_focus	2	Broad focus
**2023407**	block1	Control	post	mo2	Verb	Broad	r1	240.6587302	3	1	broad_focus	2	Broad focus
**2023407**	block1	Control	post	gau2	Object	Broad	r1	221.51882	4	1	broad_focus	2	Broad focus
**2023407**	block1	Control	post	zai2	Object	Broad	r1	375.7552765	5	2	broad_focus	2	Broad focus
**2023407**	block1	Control	post	zoeng1	Subject	Contrastive	r1	161.6362606	1	1	pre_focus	1	Contrastive pre_focus
**2023407**	block1	Control	post	saang1	Subject	Contrastive	r1	316.7798834	2	2	pre_focus	1	Contrastive pre_focus
**2023407**	block1	Control	post	tsa1	Verb	Contrastive	r1	210.6175804	3	1	pre_focus	1	Contrastive pre_focus
**2023407**	block1	Control	post	fei1	Object	Contrastive	r1	151.4204793	4	1	on_focus	1	Contrastive on_focus
**2023407**	block1	Control	post	gei1	Object	Contrastive	r1	444.1237447	5	2	on_focus	1	Contrastive on_focus
**2023407**	block1	Control	post	zoeng1	Subject	Broad	r1	132.5459814	1	1	broad_focus	1	Broad focus
**2023407**	block1	Control	post	saang1	Subject	Broad	r1	111.7039666	2	2	broad_focus	1	Broad focus
**2023407**	block1	Control	post	tsa1	Verb	Broad	r1	102.4788563	3	1	broad_focus	1	Broad focus
**2023407**	block1	Control	post	fei1	Object	Broad	r1	109.197434	4	1	broad_focus	1	Broad focus
**2023407**	block1	Control	post	gei1	Object	Broad	r1	1258.61507	5	2	broad_focus	1	Broad focus
**2023407**	block1	Control	post	sau3	Subject	Narrow	r1	278.2143687	1	1	pre_focus	3	Narrow pre_focus
**2023407**	block1	Control	post	sau3	Subject	Narrow	r1	263.8553473	2	2	pre_focus	3	Narrow pre_focus
**2023407**	block1	Control	post	sik3	Verb	Narrow	r1	188.3743309	3	1	pre_focus	3	Narrow pre_focus
**2023407**	block1	Control	post	baak3	Object	Narrow	r1	172.5408702	4	1	on_focus	3	Narrow on_focus
**2023407**	block1	Control	post	baak3	Object	Narrow	r1	1127.166799	5	2	on_focus	3	Narrow on_focus
**2023407**	block1	Control	post	zoeng1	Subject	Narrow	r1	97.81632653	1	1	pre_focus	1	Narrow pre_focus
**2023407**	block1	Control	post	saang1	Subject	Narrow	r1	99.68792517	2	2	pre_focus	1	Narrow pre_focus
**2023407**	block1	Control	post	tsa1	Verb	Narrow	r1	85.69555724	3	1	pre_focus	1	Narrow pre_focus
**2023407**	block1	Control	post	fei1	Object	Narrow	r1	94.64191232	4	1	on_focus	1	Narrow on_focus
**2023407**	block1	Control	post	gei1	Object	Narrow	r1	901.6122044	5	2	on_focus	1	Narrow on_focus
**2023407**	block1	Control	post	jyun2	Subject	Contrastive	r1	118.1448853	1	1	pre_focus	2	Contrastive pre_focus
**2023407**	block1	Control	post	jyun2	Subject	Contrastive	r1	96.79772489	2	2	pre_focus	2	Contrastive pre_focus
**2023407**	block1	Control	post	mo2	Verb	Contrastive	r1	124.5595813	3	1	pre_focus	2	Contrastive pre_focus
**2023407**	block1	Control	post	gau2	Object	Contrastive	r1	76.54672395	4	1	on_focus	2	Contrastive on_focus
**2023407**	block1	Control	post	zai2	Object	Contrastive	r1	508.1292517	5	2	on_focus	2	Contrastive on_focus
**2023407**	block1	Control	post	sau3	Subject	Contrastive	r1	234.3966634	1	1	on_focus	3	Contrastive on_focus
**2023407**	block1	Control	post	sau3	Subject	Contrastive	r1	217.707294	2	2	on_focus	3	Contrastive on_focus
**2023407**	block1	Control	post	sik3	Verb	Contrastive	r1	101.2687321	3	1	post_focus	3	Contrastive post_focus
**2023407**	block1	Control	post	baak3	Object	Contrastive	r1	197.5154075	4	1	post_focus	3	Contrastive post_focus
**2023407**	block1	Control	post	baak3	Object	Contrastive	r1	524.0903365	5	2	post_focus	3	Contrastive post_focus
**2023407**	block1	Control	post	sau3	Subject	Narrow	r1	195.751634	1	1	on_focus	3	Narrow on_focus
**2023407**	block1	Control	post	sau3	Subject	Narrow	r1	203.6324967	2	2	on_focus	3	Narrow on_focus
**2023407**	block1	Control	post	sik3	Verb	Narrow	r1	105.7609329	3	1	post_focus	3	Narrow post_focus
**2023407**	block1	Control	post	baak3	Object	Narrow	r1	123.5658627	4	1	post_focus	3	Narrow post_focus
**2023407**	block1	Control	post	baak3	Object	Narrow	r1	203.5400314	5	2	post_focus	3	Narrow post_focus
**2023407**	block1	Control	post	sau3	Subject	Narrow	r1	151.2849584	1	1	pre_focus	3	Narrow pre_focus
**2023407**	block1	Control	post	sau3	Subject	Narrow	r1	252.1322751	2	2	pre_focus	3	Narrow pre_focus
**2023407**	block1	Control	post	sik3	Verb	Narrow	r1	129.8190793	3	1	on_focus	3	Narrow on_focus
**2023407**	block1	Control	post	baak3	Object	Narrow	r1	105.0458914	4	1	post_focus	3	Narrow post_focus
**2023407**	block1	Control	post	baak3	Object	Narrow	r1	236.6867104	5	2	post_focus	3	Narrow post_focus
**2023407**	block1	Control	post	zoeng1	Subject	Contrastive	r1	199.4110607	1	1	pre_focus	1	Contrastive pre_focus
**2023407**	block1	Control	post	saang1	Subject	Contrastive	r1	305.076993	2	2	pre_focus	1	Contrastive pre_focus
**2023407**	block1	Control	post	tsa1	Verb	Contrastive	r1	199.0996129	3	1	on_focus	1	Contrastive on_focus
**2023407**	block1	Control	post	fei1	Object	Contrastive	r1	157.7622628	4	1	post_focus	1	Contrastive post_focus
**2023407**	block1	Control	post	gei1	Object	Contrastive	r1	305.8423091	5	2	post_focus	1	Contrastive post_focus
**2023407**	block1	Control	post	sau3	Subject	Broad	r1	233.5727624	1	1	broad_focus	3	Broad focus
**2023407**	block1	Control	post	sau3	Subject	Broad	r1	316.0252344	2	2	broad_focus	3	Broad focus
**2023407**	block1	Control	post	sik3	Verb	Broad	r1	129.259815	3	1	broad_focus	3	Broad focus
**2023407**	block1	Control	post	baak3	Object	Broad	r1	135.787352	4	1	broad_focus	3	Broad focus
**2023407**	block1	Control	post	baak3	Object	Broad	r1	244.9961262	5	2	broad_focus	3	Broad focus
**2023407**	block1	Control	post	jyun2	Subject	Contrastive	r1	184.5408163	1	1	on_focus	2	Contrastive on_focus
**2023407**	block1	Control	post	jyun2	Subject	Contrastive	r1	124.7257001	2	2	on_focus	2	Contrastive on_focus
**2023407**	block1	Control	post	mo2	Verb	Contrastive	r1	101.2471271	3	1	post_focus	2	Contrastive post_focus
**2023407**	block1	Control	post	gau2	Object	Contrastive	r1	86.46258503	4	1	post_focus	2	Contrastive post_focus
**2023407**	block1	Control	post	zai2	Object	Contrastive	r1	490.3800609	5	2	post_focus	2	Contrastive post_focus
**2023407**	block1	Control	post	zoeng1	Subject	Contrastive	r1	211.0140882	1	1	on_focus	1	Contrastive on_focus
**2023407**	block1	Control	post	saang1	Subject	Contrastive	r1	270.8971596	2	2	on_focus	1	Contrastive on_focus
**2023407**	block1	Control	post	tsa1	Verb	Contrastive	r1	173.5071087	3	1	post_focus	1	Contrastive post_focus

**2023407**	block1	Control	post	fei1	Object	Contrastive	r1	142.8950732	4	1	post_focus	1	Contrastive post_focus
**2023407**	block1	Control	post	gei1	Object	Contrastive	r1	78.23582766	5	2	post_focus	1	Contrastive post_focus
**2023407**	block1	Control	post	zoeng1	Subject	Narrow	r1	120.5778815	1	1	pre_focus	1	Narrow pre_focus
**2023407**	block1	Control	post	saang1	Subject	Narrow	r1	184.941691	2	2	pre_focus	1	Narrow pre_focus
**2023407**	block1	Control	post	tsa1	Verb	Narrow	r1	143.5758408	3	1	on_focus	1	Narrow on_focus
**2023407**	block1	Control	post	fei1	Object	Narrow	r1	142.3633803	4	1	post_focus	1	Narrow post_focus
**2023407**	block1	Control	post	gei1	Object	Narrow	r1	375.4665533	5	2	post_focus	1	Narrow post_focus
**2023407**	block1	Control	post	jyun2	Subject	Narrow	r1	110.6713212	1	1	pre_focus	2	Narrow pre_focus
**2023407**	block1	Control	post	jyun2	Subject	Narrow	r1	134.3066893	2	2	pre_focus	2	Narrow pre_focus
**2023407**	block1	Control	post	mo2	Verb	Narrow	r1	67.64444213	3	1	pre_focus	2	Narrow pre_focus
**2023407**	block1	Control	post	gau2	Object	Narrow	r1	69.38539052	4	1	on_focus	2	Narrow on_focus
**2023407**	block1	Control	post	zai2	Object	Narrow	r1	367.5380811	5	2	on_focus	2	Narrow on_focus
**2023407**	block1	Control	post	zoeng1	Subject	Contrastive	r2	81.59263276	1	1	on_focus	1	Contrastive on_focus
**2023407**	block1	Control	post	saang1	Subject	Contrastive	r2	114.691129	2	2	on_focus	1	Contrastive on_focus
**2023407**	block1	Control	post	tsa1	Verb	Contrastive	r2	62.57034014	3	1	post_focus	1	Contrastive post_focus
**2023407**	block1	Control	post	fei1	Object	Contrastive	r2	91.85356183	4	1	post_focus	1	Contrastive post_focus
**2023407**	block1	Control	post	gei1	Object	Contrastive	r2	307.9936754	5	2	post_focus	1	Contrastive post_focus
**2023407**	block1	Control	post	jyun2	Subject	Contrastive	r2	138.8110355	1	1	pre_focus	2	Contrastive pre_focus
**2023407**	block1	Control	post	jyun2	Subject	Contrastive	r2	233.3072562	2	2	pre_focus	2	Contrastive pre_focus
**2023407**	block1	Control	post	mo2	Verb	Contrastive	r2	111.7334436	3	1	on_focus	2	Contrastive on_focus
**2023407**	block1	Control	post	gau2	Object	Contrastive	r2	60.75743878	4	1	post_focus	2	Contrastive post_focus
**2023407**	block1	Control	post	zai2	Object	Contrastive	r2	368.6013389	5	2	post_focus	2	Contrastive post_focus
**2023407**	block1	Control	post	zoeng1	Subject	Contrastive	r2	74.29135583	1	1	pre_focus	1	Contrastive pre_focus
**2023407**	block1	Control	post	saang1	Subject	Contrastive	r2	114.4378307	2	2	pre_focus	1	Contrastive pre_focus
**2023407**	block1	Control	post	tsa1	Verb	Contrastive	r2	44.71585922	3	1	pre_focus	1	Contrastive pre_focus
**2023407**	block1	Control	post	fei1	Object	Contrastive	r2	84.45059341	4	1	on_focus	1	Contrastive on_focus
**2023407**	block1	Control	post	gei1	Object	Contrastive	r2	343.0500216	5	2	on_focus	1	Contrastive on_focus
**2023407**	block1	Control	post	zoeng1	Subject	Contrastive	r2	70.4744898	1	1	pre_focus	1	Contrastive pre_focus
**2023407**	block1	Control	post	saang1	Subject	Contrastive	r2	66.84278156	2	2	pre_focus	1	Contrastive pre_focus
**2023407**	block1	Control	post	tsa1	Verb	Contrastive	r2	57.74599935	3	1	on_focus	1	Contrastive on_focus
**2023407**	block1	Control	post	fei1	Object	Contrastive	r2	118.6895314	4	1	post_focus	1	Contrastive post_focus
**2023407**	block1	Control	post	gei1	Object	Contrastive	r2	428.2637909	5	2	post_focus	1	Contrastive post_focus
**2023407**	block1	Control	post	jyun2	Subject	Narrow	r2	71.68708575	1	1	pre_focus	2	Narrow pre_focus
**2023407**	block1	Control	post	jyun2	Subject	Narrow	r2	96.7069958	2	2	pre_focus	2	Narrow pre_focus
**2023407**	block1	Control	post	mo2	Verb	Narrow	r2	132.2547241	3	1	on_focus	2	Narrow on_focus
**2023407**	block1	Control	post	gau2	Object	Narrow	r2	56.21089352	4	1	post_focus	2	Narrow post_focus
**2023407**	block1	Control	post	zai2	Object	Narrow	r2	325.8010294	5	2	post_focus	2	Narrow post_focus
**2023407**	block1	Control	post	zoeng1	Subject	Broad	r2	81.07698298	1	1	broad_focus	1	Broad focus
**2023407**	block1	Control	post	saang1	Subject	Broad	r2	95.16973456	2	2	broad_focus	1	Broad focus
**2023407**	block1	Control	post	tsa1	Verb	Broad	r2	77.4021164	3	1	broad_focus	1	Broad focus
**2023407**	block1	Control	post	fei1	Object	Broad	r2	86.52250395	4	1	broad_focus	1	Broad focus
**2023407**	block1	Control	post	gei1	Object	Broad	r2	340.3955437	5	2	broad_focus	1	Broad focus
**2023407**	block1	Control	post	zoeng1	Subject	Narrow	r2	83.01098217	1	1	pre_focus	1	Narrow pre_focus
**2023407**	block1	Control	post	saang1	Subject	Narrow	r2	126.7689594	2	2	pre_focus	1	Narrow pre_focus
**2023407**	block1	Control	post	tsa1	Verb	Narrow	r2	65.42723968	3	1	on_focus	1	Narrow on_focus
**2023407**	block1	Control	post	fei1	Object	Narrow	r2	79.80011758	4	1	post_focus	1	Narrow post_focus
**2023407**	block1	Control	post	gei1	Object	Narrow	r2	362.399575	5	2	post_focus	1	Narrow post_focus
**2023407**	block1	Control	post	zoeng1	Subject	Narrow	r2	92.32873248	1	1	on_focus	1	Narrow on_focus
**2023407**	block1	Control	post	saang1	Subject	Narrow	r2	122.4075903	2	2	on_focus	1	Narrow on_focus
**2023407**	block1	Control	post	tsa1	Verb	Narrow	r2	70.10868957	3	1	post_focus	1	Narrow post_focus
**2023407**	block1	Control	post	fei1	Object	Narrow	r2	100.3854875	4	1	post_focus	1	Narrow post_focus
**2023407**	block1	Control	post	gei1	Object	Narrow	r2	324.456328	5	2	post_focus	1	Narrow post_focus
**2023407**	block1	Control	post	sau3	Subject	Narrow	r2	80.76169259	1	1	pre_focus	3	Narrow pre_focus
**2023407**	block1	Control	post	sau3	Subject	Narrow	r2	103.3479106	2	2	pre_focus	3	Narrow pre_focus
**2023407**	block1	Control	post	sik3	Verb	Narrow	r2	98.87104685	3	1	on_focus	3	Narrow on_focus
**2023407**	block1	Control	post	baak3	Object	Narrow	r2	57.01992494	4	1	post_focus	3	Narrow post_focus
**2023407**	block1	Control	post	baak3	Object	Narrow	r2	254.951885	5	2	post_focus	3	Narrow post_focus
**2023407**	block1	Control	post	sau3	Subject	Contrastive	r2	71.49366817	1	1	on_focus	3	Contrastive on_focus
**2023407**	block1	Control	post	sau3	Subject	Contrastive	r2	79.67971537	2	2	on_focus	3	Contrastive on_focus
**2023407**	block1	Control	post	sik3	Verb	Contrastive	r2	53.27671809	3	1	post_focus	3	Contrastive post_focus
**2023407**	block1	Control	post	baak3	Object	Contrastive	r2	60.55978836	4	1	post_focus	3	Contrastive post_focus
**2023407**	block1	Control	post	baak3	Object	Contrastive	r2	204.7991446	5	2	post_focus	3	Contrastive post_focus
**2023407**	block1	Control	post	sau3	Subject	Broad	r2	110.2892315	1	1	broad_focus	3	Broad focus
**2023407**	block1	Control	post	sau3	Subject	Broad	r2	133.3124204	2	2	broad_focus	3	Broad focus
**2023407**	block1	Control	post	sik3	Verb	Broad	r2	90.36545557	3	1	broad_focus	3	Broad focus
**2023407**	block1	Control	post	baak3	Object	Broad	r2	97.89863919	4	1	broad_focus	3	Broad focus

2023407	block1	Control	post	baak3	Object	Broad	r2	244.2607219	5	2	broad_focus	3	Broad focus
2023407	block1	Control	post	sau3	Subject	Contrastive	r2	85.03360701	1	1	pre_focus	3	Contrastive pre_focus
2023407	block1	Control	post	sau3	Subject	Contrastive	r2	107.3373967	2	2	pre_focus	3	Contrastive pre_focus
2023407	block1	Control	post	sik3	Verb	Contrastive	r2	81.06337272	3	1	on_focus	3	Contrastive on_focus
2023407	block1	Control	post	baak3	Object	Contrastive	r2	77.38560758	4	1	post_focus	3	Contrastive post_focus
2023407	block1	Control	post	baak3	Object	Contrastive	r2	205.1159769	5	2	post_focus	3	Contrastive post_focus
2023407	block1	Control	post	jyun2	Subject	Narrow	r2	112.9708332	1	1	on_focus	2	Narrow on_focus
2023407	block1	Control	post	jyun2	Subject	Narrow	r2	119.9453512	2	2	on_focus	2	Narrow on_focus
2023407	block1	Control	post	mo2	Verb	Narrow	r2	75.13441032	3	1	post_focus	2	Narrow post_focus
2023407	block1	Control	post	gau2	Object	Narrow	r2	82.46031387	4	1	post_focus	2	Narrow post_focus
2023407	block1	Control	post	zai2	Object	Narrow	r2	296.6851222	5	2	post_focus	2	Narrow post_focus
2023407	block1	Control	post	sau3	Subject	Narrow	r2	90.37399404	1	1	on_focus	3	Narrow on_focus
2023407	block1	Control	post	sau3	Subject	Narrow	r2	136.7323928	2	2	on_focus	3	Narrow on_focus
2023407	block1	Control	post	sik3	Verb	Narrow	r2	85.81146744	3	1	post_focus	3	Narrow post_focus
2023407	block1	Control	post	baak3	Object	Narrow	r2	86.69073783	4	1	post_focus	3	Narrow post_focus
2023407	block1	Control	post	baak3	Object	Narrow	r2	185.3256938	5	2	post_focus	3	Narrow post_focus
2023407	block1	Control	post	zoeng1	Subject	Narrow	r2	130.4662005	1	1	pre_focus	1	Narrow pre_focus
2023407	block1	Control	post	saang1	Subject	Narrow	r2	136.3412994	2	2	pre_focus	1	Narrow pre_focus
2023407	block1	Control	post	tsa1	Verb	Narrow	r2	110.3500029	3	1	pre_focus	1	Narrow pre_focus
2023407	block1	Control	post	fei1	Object	Narrow	r2	119.7591418	4	1	on_focus	1	Narrow on_focus
2023407	block1	Control	post	gei1	Object	Narrow	r2	237.8673469	5	2	on_focus	1	Narrow on_focus
2023407	block1	Control	post	jyun2	Subject	Broad	r2	598.6823777	1	1	broad_focus	2	Broad focus
2023407	block1	Control	post	jyun2	Subject	Broad	r2	418.2549761	2	2	broad_focus	2	Broad focus
2023407	block1	Control	post	mo2	Verb	Broad	r2	300.6657848	3	1	broad_focus	2	Broad focus
2023407	block1	Control	post	gau2	Object	Broad	r2	183.2511338	4	1	broad_focus	2	Broad focus
2023407	block1	Control	post	zai2	Object	Broad	r2	255.6002986	5	2	broad_focus	2	Broad focus
2023407	block1	Control	post	sau3	Subject	Narrow	r2	300.8526696	1	1	pre_focus	3	Narrow pre_focus
2023407	block1	Control	post	sau3	Subject	Narrow	r2	341.739544	2	2	pre_focus	3	Narrow pre_focus
2023407	block1	Control	post	sik3	Verb	Narrow	r2	124.4579726	3	1	pre_focus	3	Narrow pre_focus
2023407	block1	Control	post	baak3	Object	Narrow	r2	113.2913226	4	1	on_focus	3	Narrow on_focus
2023407	block1	Control	post	baak3	Object	Narrow	r2	150.4259299	5	2	on_focus	3	Narrow on_focus
2023407	block1	Control	post	jyun2	Subject	Contrastive	r2	297.5741686	1	1	pre_focus	2	Contrastive pre_focus
2023407	block1	Control	post	jyun2	Subject	Contrastive	r2	408.9734588	2	2	pre_focus	2	Contrastive pre_focus
2023407	block1	Control	post	mo2	Verb	Contrastive	r2	258.5379079	3	1	pre_focus	2	Contrastive pre_focus
2023407	block1	Control	post	gau2	Object	Contrastive	r2	210.9942762	4	1	on_focus	2	Contrastive on_focus
2023407	block1	Control	post	zai2	Object	Contrastive	r2	330.4584278	5	2	on_focus	2	Contrastive on_focus
2023407	block1	Control	post	jyun2	Subject	Narrow	r2	398.1670446	1	1	pre_focus	2	Narrow pre_focus
2023407	block1	Control	post	jyun2	Subject	Narrow	r2	283.3757503	2	2	pre_focus	2	Narrow pre_focus
2023407	block1	Control	post	mo2	Verb	Narrow	r2	247.7542922	3	1	pre_focus	2	Narrow pre_focus
2023407	block1	Control	post	gau2	Object	Narrow	r2	234.7014361	4	1	on_focus	2	Narrow on_focus
2023407	block1	Control	post	zai2	Object	Narrow	r2	339.3816173	5	2	on_focus	2	Narrow on_focus
2023407	block1	Control	post	sau3	Subject	Contrastive	r2	210.5236206	1	1	pre_focus	3	Contrastive pre_focus
2023407	block1	Control	post	sau3	Subject	Contrastive	r2	233.4038686	2	2	pre_focus	3	Contrastive pre_focus
2023407	block1	Control	post	sik3	Verb	Contrastive	r2	63.61780832	3	1	pre_focus	3	Contrastive pre_focus
2023407	block1	Control	post	baak3	Object	Contrastive	r2	70.71678888	4	1	on_focus	3	Contrastive on_focus
2023407	block1	Control	post	baak3	Object	Contrastive	r2	112.9977935	5	2	on_focus	3	Contrastive on_focus
2023407	block1	Control	post	jyun2	Subject	Contrastive	r2	98.49548285	1	1	on_focus	2	Contrastive on_focus
2023407	block1	Control	post	jyun2	Subject	Contrastive	r2	103.4546737	2	2	on_focus	2	Contrastive on_focus
2023407	block1	Control	post	mo2	Verb	Contrastive	r2	94.21201814	3	1	post_focus	2	Contrastive post_focus
2023407	block1	Control	post	gau2	Object	Contrastive	r2	30.91005291	4	1	post_focus	2	Contrastive post_focus
2023407	block1	Control	post	zai2	Object	Contrastive	r2	282.0952381	5	2	post_focus	2	Contrastive post_focus
2023407	block1	Control	pre	zoeng1	Subject	Contrastive	r1	178.9277143	1	1	pre_focus	1	Contrastive pre_focus
2023407	block1	Control	pre	saang1	Subject	Contrastive	r1	194.5327948	2	2	pre_focus	1	Contrastive pre_focus
2023407	block1	Control	pre	tsa1	Verb	Contrastive	r1	135.7494912	3	1	on_focus	1	Contrastive on_focus
2023407	block1	Control	pre	fei1	Object	Contrastive	r1	129.9096636	4	1	post_focus	1	Contrastive post_focus
2023407	block1	Control	pre	gei1	Object	Contrastive	r1	604.3378535	5	2	post_focus	1	Contrastive post_focus
2023407	block1	Control	pre	jyun2	Subject	Narrow	r1	152.3387061	1	1	pre_focus	2	Narrow pre_focus
2023407	block1	Control	pre	jyun2	Subject	Narrow	r1	189.1128991	2	2	pre_focus	2	Narrow pre_focus
2023407	block1	Control	pre	mo2	Verb	Narrow	r1	144.0870284	3	1	on_focus	2	Narrow on_focus
2023407	block1	Control	pre	gau2	Object	Narrow	r1	109.5002971	4	1	post_focus	2	Narrow post_focus
2023407	block1	Control	pre	zai2	Object	Narrow	r1	292.1055017	5	2	post_focus	2	Narrow post_focus
2023407	block1	Control	pre	zoeng1	Subject	Narrow	r1	199.2784949	1	1	pre_focus	1	Narrow pre_focus
2023407	block1	Control	pre	saang1	Subject	Narrow	r1	180.0516139	2	2	pre_focus	1	Narrow pre_focus
2023407	block1	Control	pre	tsa1	Verb	Narrow	r1	135.6602876	3	1	on_focus	1	Narrow on_focus
2023407	block1	Control	pre	fei1	Object	Narrow	r1	106.6612619	4	1	post_focus	1	Narrow post_focus
2023407	block1	Control	pre	gei1	Object	Narrow	r1	245.7949893	5	2	post_focus	1	Narrow post_focus

2023407	block1	Control	pre	zoeng1	Subject	Broad	r1	202.7932996	1	1	broad_focus	1	Broad focus
2023407	block1	Control	pre	saang1	Subject	Broad	r1	242.5041786	2	2	broad_focus	1	Broad focus
2023407	block1	Control	pre	tsa1	Verb	Broad	r1	156.3780215	3	1	broad_focus	1	Broad focus
2023407	block1	Control	pre	fei1	Object	Broad	r1	129.1044242	4	1	broad_focus	1	Broad focus
2023407	block1	Control	pre	gei1	Object	Broad	r1	498.0905985	5	2	broad_focus	1	Broad focus
2023407	block1	Control	pre	jyun2	Subject	Contrastive	r1	223.4871517	1	1	pre_focus	2	Contrastive pre_focus
2023407	block1	Control	pre	jyun2	Subject	Contrastive	r1	220.9031595	2	2	pre_focus	2	Contrastive pre_focus
2023407	block1	Control	pre	mo2	Verb	Contrastive	r1	208.4531433	3	1	on_focus	2	Contrastive on_focus
2023407	block1	Control	pre	gau2	Object	Contrastive	r1	122.7556211	4	1	post_focus	2	Contrastive post_focus
2023407	block1	Control	pre	zai2	Object	Contrastive	r1	436.9145676	5	2	post_focus	2	Contrastive post_focus
2023407	block1	Control	pre	jyun2	Subject	Narrow	r1	295.2429414	1	1	on_focus	2	Narrow on_focus
2023407	block1	Control	pre	jyun2	Subject	Narrow	r1	215.6954786	2	2	on_focus	2	Narrow on_focus
2023407	block1	Control	pre	mo2	Verb	Narrow	r1	174.9572769	3	1	post_focus	2	Narrow post_focus
2023407	block1	Control	pre	gau2	Object	Narrow	r1	79.097426	4	1	post_focus	2	Narrow post_focus
2023407	block1	Control	pre	zai2	Object	Narrow	r1	351.2028939	5	2	post_focus	2	Narrow post_focus
2023407	block1	Control	pre	sau3	Subject	Contrastive	r1	158.6077128	1	1	on_focus	3	Contrastive on_focus
2023407	block1	Control	pre	sau3	Subject	Contrastive	r1	166.7393593	2	2	on_focus	3	Contrastive on_focus
2023407	block1	Control	pre	sik3	Verb	Contrastive	r1	72.71785182	3	1	post_focus	3	Contrastive post_focus
2023407	block1	Control	pre	baak3	Object	Contrastive	r1	48.18858482	4	1	post_focus	3	Contrastive post_focus
2023407	block1	Control	pre	baak3	Object	Contrastive	r1	168.8647389	5	2	post_focus	3	Contrastive post_focus
2023407	block1	Control	pre	sau3	Subject	Narrow	r1	145.245333	1	1	pre_focus	3	Narrow pre_focus
2023407	block1	Control	pre	sau3	Subject	Narrow	r1	143.7882347	2	2	pre_focus	3	Narrow pre_focus
2023407	block1	Control	pre	sik3	Verb	Narrow	r1	63.58871882	3	1	pre_focus	3	Narrow pre_focus
2023407	block1	Control	pre	baak3	Object	Narrow	r1	35.69587793	4	1	on_focus	3	Narrow on_focus
2023407	block1	Control	pre	baak3	Object	Narrow	r1	121.3656172	5	2	on_focus	3	Narrow on_focus
2023407	block1	Control	pre	jyun2	Subject	Contrastive	r1	206.8983292	1	1	pre_focus	2	Contrastive pre_focus
2023407	block1	Control	pre	jyun2	Subject	Contrastive	r1	225.7675107	2	2	pre_focus	2	Contrastive pre_focus
2023407	block1	Control	pre	mo2	Verb	Contrastive	r1	200.0730231	3	1	pre_focus	2	Contrastive pre_focus
2023407	block1	Control	pre	gau2	Object	Contrastive	r1	62.59269974	4	1	on_focus	2	Contrastive on_focus
2023407	block1	Control	pre	zai2	Object	Contrastive	r1	355.9973415	5	2	on_focus	2	Contrastive on_focus
2023407	block1	Control	pre	zoeng1	Subject	Contrastive	r1	192.2868061	1	1	on_focus	1	Contrastive on_focus
2023407	block1	Control	pre	saang1	Subject	Contrastive	r1	188.6514186	2	2	on_focus	1	Contrastive on_focus
2023407	block1	Control	pre	tsa1	Verb	Contrastive	r1	118.6872236	3	1	post_focus	1	Contrastive post_focus
2023407	block1	Control	pre	fei1	Object	Contrastive	r1	105.6152145	4	1	post_focus	1	Contrastive post_focus
2023407	block1	Control	pre	gei1	Object	Contrastive	r1	262.6733958	5	2	post_focus	1	Contrastive post_focus
2023407	block1	Control	pre	sau3	Subject	Contrastive	r1	161.9419986	1	1	pre_focus	3	Contrastive pre_focus
2023407	block1	Control	pre	sau3	Subject	Contrastive	r1	117.7306828	2	2	pre_focus	3	Contrastive pre_focus
2023407	block1	Control	pre	sik3	Verb	Contrastive	r1	93.77181574	3	1	on_focus	3	Contrastive on_focus
2023407	block1	Control	pre	baak3	Object	Contrastive	r1	63.2090124	4	1	post_focus	3	Contrastive post_focus
2023407	block1	Control	pre	baak3	Object	Contrastive	r1	172.5236063	5	2	post_focus	3	Contrastive post_focus
2023407	block1	Control	pre	jyun2	Subject	Broad	r1	216.718177	1	1	broad_focus	2	Broad focus
2023407	block1	Control	pre	jyun2	Subject	Broad	r1	192.9879635	2	2	broad_focus	2	Broad focus
2023407	block1	Control	pre	mo2	Verb	Broad	r1	219.8067163	3	1	broad_focus	2	Broad focus
2023407	block1	Control	pre	gau2	Object	Broad	r1	114.2932267	4	1	broad_focus	2	Broad focus
2023407	block1	Control	pre	zai2	Object	Broad	r1	286.5160545	5	2	broad_focus	2	Broad focus
2023407	block1	Control	pre	sau3	Subject	Broad	r1	172.445158	1	1	broad_focus	3	Broad focus
2023407	block1	Control	pre	sau3	Subject	Broad	r1	157.3859923	2	2	broad_focus	3	Broad focus
2023407	block1	Control	pre	sik3	Verb	Broad	r1	101.9959254	3	1	broad_focus	3	Broad focus
2023407	block1	Control	pre	baak3	Object	Broad	r1	46.39575311	4	1	broad_focus	3	Broad focus
2023407	block1	Control	pre	baak3	Object	Broad	r1	114.3495612	5	2	broad_focus	3	Broad focus
2023407	block1	Control	pre	zoeng1	Subject	Narrow	r1	208.6982225	1	1	on_focus	1	Narrow on_focus
2023407	block1	Control	pre	saang1	Subject	Narrow	r1	193.226762	2	2	on_focus	1	Narrow on_focus
2023407	block1	Control	pre	tsa1	Verb	Narrow	r1	173.5682197	3	1	post_focus	1	Narrow post_focus
2023407	block1	Control	pre	fei1	Object	Narrow	r1	113.8977735	4	1	post_focus	1	Narrow post_focus
2023407	block1	Control	pre	gei1	Object	Narrow	r1	327.2027015	5	2	post_focus	1	Narrow post_focus
2023407	block1	Control	pre	jyun2	Subject	Narrow	r1	256.1108591	1	1	pre_focus	2	Narrow pre_focus
2023407	block1	Control	pre	jyun2	Subject	Narrow	r1	191.8048225	2	2	pre_focus	2	Narrow pre_focus
2023407	block1	Control	pre	mo2	Verb	Narrow	r1	206.2652401	3	1	pre_focus	2	Narrow pre_focus
2023407	block1	Control	pre	gau2	Object	Narrow	r1	108.2897442	4	1	on_focus	2	Narrow on_focus
2023407	block1	Control	pre	zai2	Object	Narrow	r1	318.1964804	5	2	on_focus	2	Narrow on_focus
2023407	block1	Control	pre	zoeng1	Subject	Contrastive	r1	161.6675118	1	1	pre_focus	1	Contrastive pre_focus
2023407	block1	Control	pre	saang1	Subject	Contrastive	r1	208.7027302	2	2	pre_focus	1	Contrastive pre_focus
2023407	block1	Control	pre	tsa1	Verb	Contrastive	r1	140.4025206	3	1	pre_focus	1	Contrastive pre_focus
2023407	block1	Control	pre	fei1	Object	Contrastive	r1	138.352174	4	1	on_focus	1	Contrastive on_focus
2023407	block1	Control	pre	gei1	Object	Contrastive	r1	229.1301191	5	2	on_focus	1	Contrastive on_focus
2023407	block1	Control	pre	zoeng1	Subject	Narrow	r1	106.4956932	1	1	pre_focus	1	Narrow pre_focus

**2023407**	block1	Control	pre	saang1	Subject	Narrow	r1	125.1066125	2	2	pre_focus	1	Narrow pre_focus
**2023407**	block1	Control	pre	tsa1	Verb	Narrow	r1	97.15173632	3	1	pre_focus	1	Narrow pre_focus
**2023407**	block1	Control	pre	fei1	Object	Narrow	r1	71.59142445	4	1	on_focus	1	Narrow on_focus
**2023407**	block1	Control	pre	gei1	Object	Narrow	r1	127.2543152	5	2	on_focus	1	Narrow on_focus
**2023407**	block1	Control	pre	sau3	Subject	Narrow	r1	94.19885656	1	1	on_focus	3	Narrow on_focus
**2023407**	block1	Control	pre	sau3	Subject	Narrow	r1	106.4829351	2	2	on_focus	3	Narrow on_focus
**2023407**	block1	Control	pre	sik3	Verb	Narrow	r1	46.59073374	3	1	post_focus	3	Narrow post_focus
**2023407**	block1	Control	pre	baak3	Object	Narrow	r1	58.49891922	4	1	post_focus	3	Narrow post_focus
**2023407**	block1	Control	pre	baak3	Object	Narrow	r1	299.0858549	5	2	post_focus	3	Narrow post_focus
**2023407**	block1	Control	pre	sau3	Subject	Narrow	r1	94.18916244	1	1	pre_focus	3	Narrow pre_focus
**2023407**	block1	Control	pre	sau3	Subject	Narrow	r1	79.27595112	2	2	pre_focus	3	Narrow pre_focus
**2023407**	block1	Control	pre	sik3	Verb	Narrow	r1	69.66385089	3	1	on_focus	3	Narrow on_focus
**2023407**	block1	Control	pre	baak3	Object	Narrow	r1	54.37246964	4	1	post_focus	3	Narrow post_focus
**2023407**	block1	Control	pre	baak3	Object	Narrow	r1	172.847561	5	2	post_focus	3	Narrow post_focus
**2023407**	block1	Control	pre	sau3	Subject	Contrastive	r1	58.63797693	1	1	pre_focus	3	Contrastive pre_focus
**2023407**	block1	Control	pre	sau3	Subject	Contrastive	r1	45.6619401	2	2	pre_focus	3	Contrastive pre_focus
**2023407**	block1	Control	pre	sik3	Verb	Contrastive	r1	64.6613269	3	1	pre_focus	3	Contrastive pre_focus
**2023407**	block1	Control	pre	baak3	Object	Contrastive	r1	60.69846309	4	1	on_focus	3	Contrastive on_focus
**2023407**	block1	Control	pre	baak3	Object	Contrastive	r1	208.8821725	5	2	on_focus	3	Contrastive on_focus
**2023407**	block1	Control	pre	jyun2	Subject	Contrastive	r1	246.5353209	1	1	on_focus	2	Contrastive on_focus
**2023407**	block1	Control	pre	jyun2	Subject	Contrastive	r1	219.7610055	2	2	on_focus	2	Contrastive on_focus
**2023407**	block1	Control	pre	mo2	Verb	Contrastive	r1	206.1032125	3	1	post_focus	2	Contrastive post_focus
**2023407**	block1	Control	pre	gau2	Object	Contrastive	r1	125.6560604	4	1	post_focus	2	Contrastive post_focus
**2023407**	block1	Control	pre	zai2	Object	Contrastive	r1	220.4684837	5	2	post_focus	2	Contrastive post_focus
**2023407**	block1	Control	pre	sau3	Subject	Narrow	r2	162.171071	1	1	on_focus	3	Narrow on_focus
**2023407**	block1	Control	pre	sau3	Subject	Narrow	r2	135.3860206	2	2	on_focus	3	Narrow on_focus
**2023407**	block1	Control	pre	sik3	Verb	Narrow	r2	77.10001894	3	1	post_focus	3	Narrow post_focus
**2023407**	block1	Control	pre	baak3	Object	Narrow	r2	67.36917844	4	1	post_focus	3	Narrow post_focus
**2023407**	block1	Control	pre	baak3	Object	Narrow	r2	144.0358016	5	2	post_focus	3	Narrow post_focus
**2023407**	block1	Control	pre	sau3	Subject	Contrastive	r2	182.250661	1	1	on_focus	3	Contrastive on_focus
**2023407**	block1	Control	pre	sau3	Subject	Contrastive	r2	174.0268182	2	2	on_focus	3	Contrastive on_focus
**2023407**	block1	Control	pre	sik3	Verb	Contrastive	r2	88.81945828	3	1	post_focus	3	Contrastive post_focus
**2023407**	block1	Control	pre	baak3	Object	Contrastive	r2	58.23119334	4	1	post_focus	3	Contrastive post_focus
**2023407**	block1	Control	pre	baak3	Object	Contrastive	r2	133.6274841	5	2	post_focus	3	Contrastive post_focus
**2023407**	block1	Control	pre	sau3	Subject	Contrastive	r2	157.830474	1	1	pre_focus	3	Contrastive pre_focus
**2023407**	block1	Control	pre	sau3	Subject	Contrastive	r2	164.7277081	2	2	pre_focus	3	Contrastive pre_focus
**2023407**	block1	Control	pre	sik3	Verb	Contrastive	r2	97.15879913	3	1	pre_focus	3	Contrastive pre_focus
**2023407**	block1	Control	pre	baak3	Object	Contrastive	r2	88.38560241	4	1	on_focus	3	Contrastive on_focus
**2023407**	block1	Control	pre	baak3	Object	Contrastive	r2	149.3412118	5	2	on_focus	3	Contrastive on_focus
**2023407**	block1	Control	pre	zoeng1	Subject	Narrow	r2	161.5743252	1	1	pre_focus	1	Narrow pre_focus
**2023407**	block1	Control	pre	saang1	Subject	Narrow	r2	195.9035129	2	2	pre_focus	1	Narrow pre_focus
**2023407**	block1	Control	pre	tsa1	Verb	Narrow	r2	153.3367007	3	1	on_focus	1	Narrow on_focus
**2023407**	block1	Control	pre	fei1	Object	Narrow	r2	101.9772299	4	1	post_focus	1	Narrow post_focus
**2023407**	block1	Control	pre	gei1	Object	Narrow	r2	290.6622357	5	2	post_focus	1	Narrow post_focus
**2023407**	block1	Control	pre	zoeng1	Subject	Contrastive	r2	185.1361719	1	1	pre_focus	1	Contrastive pre_focus
**2023407**	block1	Control	pre	saang1	Subject	Contrastive	r2	246.9249429	2	2	pre_focus	1	Contrastive pre_focus
**2023407**	block1	Control	pre	tsa1	Verb	Contrastive	r2	188.7527464	3	1	pre_focus	1	Contrastive pre_focus
**2023407**	block1	Control	pre	fei1	Object	Contrastive	r2	125.0815051	4	1	on_focus	1	Contrastive on_focus
**2023407**	block1	Control	pre	gei1	Object	Contrastive	r2	192.6760913	5	2	on_focus	1	Contrastive on_focus
**2023407**	block1	Control	pre	jyun2	Subject	Contrastive	r2	204.0648148	1	1	on_focus	2	Contrastive on_focus
**2023407**	block1	Control	pre	jyun2	Subject	Contrastive	r2	157.110471	2	2	on_focus	2	Contrastive on_focus
**2023407**	block1	Control	pre	mo2	Verb	Contrastive	r2	145.0803012	3	1	post_focus	2	Contrastive post_focus
**2023407**	block1	Control	pre	gau2	Object	Contrastive	r2	89.48558489	4	1	post_focus	2	Contrastive post_focus
**2023407**	block1	Control	pre	zai2	Object	Contrastive	r2	259.8564044	5	2	post_focus	2	Contrastive post_focus
**2023407**	block1	Control	pre	jyun2	Subject	Contrastive	r2	244.908965	1	1	pre_focus	2	Contrastive pre_focus
**2023407**	block1	Control	pre	jyun2	Subject	Contrastive	r2	163.4217702	2	2	pre_focus	2	Contrastive pre_focus
**2023407**	block1	Control	pre	mo2	Verb	Contrastive	r2	167.2194418	3	1	on_focus	2	Contrastive on_focus
**2023407**	block1	Control	pre	gau2	Object	Contrastive	r2	92.86913019	4	1	post_focus	2	Contrastive post_focus
**2023407**	block1	Control	pre	zai2	Object	Contrastive	r2	197.6662726	5	2	post_focus	2	Contrastive post_focus
**2023407**	block1	Control	pre	jyun2	Subject	Broad	r2	188.249431	1	1	broad_focus	2	Broad focus
**2023407**	block1	Control	pre	jyun2	Subject	Broad	r2	142.373606	2	2	broad_focus	2	Broad focus
**2023407**	block1	Control	pre	mo2	Verb	Broad	r2	118.9283906	3	1	broad_focus	2	Broad focus
**2023407**	block1	Control	pre	gau2	Object	Broad	r2	54.51984143	4	1	broad_focus	2	Broad focus
**2023407**	block1	Control	pre	zai2	Object	Broad	r2	184.4059371	5	2	broad_focus	2	Broad focus
**2023407**	block1	Control	pre	jyun2	Subject	Narrow	r2	214.4744944	1	1	pre_focus	2	Narrow pre_focus
**2023407**	block1	Control	pre	jyun2	Subject	Narrow	r2	143.4927024	2	2	pre_focus	2	Narrow pre_focus

**2023407**	block1	Control	pre	mo2	Verb	Narrow	r2	116.5228822	3	1	pre_focus	2	Narrow pre_focus
**2023407**	block1	Control	pre	gau2	Object	Narrow	r2	80.03358031	4	1	on_focus	2	Narrow on_focus
**2023407**	block1	Control	pre	zai2	Object	Narrow	r2	231.859015	5	2	on_focus	2	Narrow on_focus
**2023407**	block1	Control	pre	zoeng1	Subject	Contrastive	r2	131.3126295	1	1	on_focus	1	Contrastive on_focus
**2023407**	block1	Control	pre	saang1	Subject	Contrastive	r2	139.3079608	2	2	on_focus	1	Contrastive on_focus
**2023407**	block1	Control	pre	tsa1	Verb	Contrastive	r2	108.9659353	3	1	post_focus	1	Contrastive post_focus
**2023407**	block1	Control	pre	fei1	Object	Contrastive	r2	89.22763837	4	1	post_focus	1	Contrastive post_focus
**2023407**	block1	Control	pre	gei1	Object	Contrastive	r2	303.1201555	5	2	post_focus	1	Contrastive post_focus
**2023407**	block1	Control	pre	jyun2	Subject	Narrow	r2	200.174872	1	1	on_focus	2	Narrow on_focus
**2023407**	block1	Control	pre	jyun2	Subject	Narrow	r2	166.8751838	2	2	on_focus	2	Narrow on_focus
**2023407**	block1	Control	pre	mo2	Verb	Narrow	r2	145.6922971	3	1	post_focus	2	Narrow post_focus
**2023407**	block1	Control	pre	gau2	Object	Narrow	r2	91.10352454	4	1	post_focus	2	Narrow post_focus
**2023407**	block1	Control	pre	zai2	Object	Narrow	r2	262.1182151	5	2	post_focus	2	Narrow post_focus
**2023407**	block1	Control	pre	sau3	Subject	Broad	r2	131.0132206	1	1	broad_focus	3	Broad focus
**2023407**	block1	Control	pre	sau3	Subject	Broad	r2	145.2401715	2	2	broad_focus	3	Broad focus
**2023407**	block1	Control	pre	sik3	Verb	Broad	r2	69.34792721	3	1	broad_focus	3	Broad focus
**2023407**	block1	Control	pre	baak3	Object	Broad	r2	59.56846684	4	1	broad_focus	3	Broad focus
**2023407**	block1	Control	pre	baak3	Object	Broad	r2	143.4524224	5	2	broad_focus	3	Broad focus
**2023407**	block1	Control	pre	zoeng1	Subject	Contrastive	r2	166.2179013	1	1	pre_focus	1	Contrastive pre_focus
**2023407**	block1	Control	pre	saang1	Subject	Contrastive	r2	156.6268579	2	2	pre_focus	1	Contrastive pre_focus
**2023407**	block1	Control	pre	tsa1	Verb	Contrastive	r2	126.008069	3	1	on_focus	1	Contrastive on_focus
**2023407**	block1	Control	pre	fei1	Object	Contrastive	r2	120.1940994	4	1	post_focus	1	Contrastive post_focus
**2023407**	block1	Control	pre	gei1	Object	Contrastive	r2	197.853273	5	2	post_focus	1	Contrastive post_focus
**2023407**	block1	Control	pre	jyun2	Subject	Narrow	r2	203.3796937	1	1	pre_focus	2	Narrow pre_focus
**2023407**	block1	Control	pre	jyun2	Subject	Narrow	r2	215.450757	2	2	pre_focus	2	Narrow pre_focus
**2023407**	block1	Control	pre	mo2	Verb	Narrow	r2	161.4776931	3	1	on_focus	2	Narrow on_focus
**2023407**	block1	Control	pre	gau2	Object	Narrow	r2	94.87679211	4	1	post_focus	2	Narrow post_focus
**2023407**	block1	Control	pre	zai2	Object	Narrow	r2	235.5604783	5	2	post_focus	2	Narrow post_focus
**2023407**	block1	Control	pre	zoeng1	Subject	Narrow	r2	134.0264222	1	1	pre_focus	1	Narrow pre_focus
**2023407**	block1	Control	pre	saang1	Subject	Narrow	r2	200.7714625	2	2	pre_focus	1	Narrow pre_focus
**2023407**	block1	Control	pre	tsa1	Verb	Narrow	r2	113.2021986	3	1	pre_focus	1	Narrow pre_focus
**2023407**	block1	Control	pre	fei1	Object	Narrow	r2	108.4239766	4	1	on_focus	1	Narrow on_focus
**2023407**	block1	Control	pre	gei1	Object	Narrow	r2	185.4780324	5	2	on_focus	1	Narrow on_focus
**2023407**	block1	Control	pre	sau3	Subject	Narrow	r2	118.9119256	1	1	pre_focus	3	Narrow pre_focus
**2023407**	block1	Control	pre	sau3	Subject	Narrow	r2	116.9324196	2	2	pre_focus	3	Narrow pre_focus
**2023407**	block1	Control	pre	sik3	Verb	Narrow	r2	79.46218781	3	1	pre_focus	3	Narrow pre_focus
**2023407**	block1	Control	pre	baak3	Object	Narrow	r2	64.10266904	4	1	on_focus	3	Narrow on_focus
**2023407**	block1	Control	pre	baak3	Object	Narrow	r2	109.6681737	5	2	on_focus	3	Narrow on_focus
**2023407**	block1	Control	pre	zoeng1	Subject	Broad	r2	180.5173958	1	1	broad_focus	1	Broad focus
**2023407**	block1	Control	pre	saang1	Subject	Broad	r2	291.4016256	2	2	broad_focus	1	Broad focus
**2023407**	block1	Control	pre	tsa1	Verb	Broad	r2	113.5941329	3	1	broad_focus	1	Broad focus
**2023407**	block1	Control	pre	fei1	Object	Broad	r2	117.7031354	4	1	broad_focus	1	Broad focus
**2023407**	block1	Control	pre	gei1	Object	Broad	r2	157.9905698	5	2	broad_focus	1	Broad focus
**2023407**	block1	Control	pre	sau3	Subject	Contrastive	r2	161.3778457	1	1	pre_focus	3	Contrastive pre_focus
**2023407**	block1	Control	pre	sau3	Subject	Contrastive	r2	122.2598771	2	2	pre_focus	3	Contrastive pre_focus
**2023407**	block1	Control	pre	sik3	Verb	Contrastive	r2	59.29844603	3	1	on_focus	3	Contrastive on_focus
**2023407**	block1	Control	pre	baak3	Object	Contrastive	r2	55.12906592	4	1	post_focus	3	Contrastive post_focus
**2023407**	block1	Control	pre	baak3	Object	Contrastive	r2	106.1914183	5	2	post_focus	3	Contrastive post_focus
**2023407**	block1	Control	pre	sau3	Subject	Narrow	r2	169.0326183	1	1	pre_focus	3	Narrow pre_focus
**2023407**	block1	Control	pre	sau3	Subject	Narrow	r2	125.9166892	2	2	pre_focus	3	Narrow pre_focus
**2023407**	block1	Control	pre	sik3	Verb	Narrow	r2	81.90198072	3	1	on_focus	3	Narrow on_focus
**2023407**	block1	Control	pre	baak3	Object	Narrow	r2	49.27385768	4	1	post_focus	3	Narrow post_focus
**2023407**	block1	Control	pre	baak3	Object	Narrow	r2	147.3377879	5	2	post_focus	3	Narrow post_focus
**2023407**	block1	Control	pre	zoeng1	Subject	Narrow	r2	124.2965342	1	1	on_focus	1	Narrow on_focus
**2023407**	block1	Control	pre	saang1	Subject	Narrow	r2	101.1667325	2	2	on_focus	1	Narrow on_focus
**2023407**	block1	Control	pre	tsa1	Verb	Narrow	r2	86.63566735	3	1	post_focus	1	Narrow post_focus
**2023407**	block1	Control	pre	fei1	Object	Narrow	r2	61.72985664	4	1	post_focus	1	Narrow post_focus
**2023407**	block1	Control	pre	gei1	Object	Narrow	r2	249.7302228	5	2	post_focus	1	Narrow post_focus
**2023407**	block1	Control	pre	jyun2	Subject	Contrastive	r2	175.7619692	1	1	pre_focus	2	Contrastive pre_focus
**2023407**	block1	Control	pre	jyun2	Subject	Contrastive	r2	180.8463805	2	2	pre_focus	2	Contrastive pre_focus
**2023407**	block1	Control	pre	mo2	Verb	Contrastive	r2	188.6463826	3	1	pre_focus	2	Contrastive pre_focus
**2023407**	block1	Control	pre	gau2	Object	Contrastive	r2	137.8189943	4	1	on_focus	2	Contrastive on_focus
**2023407**	block1	Control	pre	zai2	Object	Contrastive	r2	337.5976803	5	2	on_focus	2	Contrastive on_focus
**2023407**	block2	Control	post	ma4	Subject	Contrastive	r1	137.9117372	1	1	pre_focus	4	Contrastive pre_focus
**2023407**	block2	Control	post	ma4	Subject	Contrastive	r1	98.69427506	2	2	pre_focus	4	Contrastive pre_focus
**2023407**	block2	Control	post	fu4	Verb	Contrastive	r1	65.18586948	3	1	pre_focus	4	Contrastive pre_focus

**2023407**	block2	Control	post	maang4	Object	Contrastive	r1	150.5915211	4	1	on_focus	4	Contrastive on_focus
**2023407**	block2	Control	post	Jan-04	Object	Contrastive	r1	172.4979194	5	2	on_focus	4	Contrastive on_focus
**2023407**	block2	Control	post	lok6	Subject	Broad	r1	128.6310013	1	1	broad_focus	6	Broad focus
**2023407**	block2	Control	post	lok6	Subject	Broad	r1	140.283093	2	2	broad_focus	6	Broad focus
**2023407**	block2	Control	post	waa6	Verb	Broad	r1	111.9803759	3	1	broad_focus	6	Broad focus
**2023407**	block2	Control	post	jyut6	Object	Broad	r1	90.47345807	4	1	broad_focus	6	Broad focus
**2023407**	block2	Control	post	loeng6	Object	Broad	r1	185.4602663	5	2	broad_focus	6	Broad focus
**2023407**	block2	Control	post	lok6	Subject	Contrastive	r1	128.6972327	1	1	pre_focus	6	Contrastive pre_focus
**2023407**	block2	Control	post	lok6	Subject	Contrastive	r1	79.14488198	2	2	pre_focus	6	Contrastive pre_focus
**2023407**	block2	Control	post	waa6	Verb	Contrastive	r1	101.9430489	3	1	pre_focus	6	Contrastive pre_focus
**2023407**	block2	Control	post	jyut6	Object	Contrastive	r1	107.8360281	4	1	on_focus	6	Contrastive on_focus
**2023407**	block2	Control	post	loeng6	Object	Contrastive	r1	224.3062304	5	2	on_focus	6	Contrastive on_focus
**2023407**	block2	Control	post	ma4	Subject	Narrow	r1	105.4478458	1	1	pre_focus	4	Narrow pre_focus
**2023407**	block2	Control	post	ma4	Subject	Narrow	r1	95.72323821	2	2	pre_focus	4	Narrow pre_focus
**2023407**	block2	Control	post	fu4	Verb	Narrow	r1	112.8033892	3	1	on_focus	4	Narrow on_focus
**2023407**	block2	Control	post	maang4	Object	Narrow	r1	120.4815628	4	1	post_focus	4	Narrow post_focus
**2023407**	block2	Control	post	Jan-04	Object	Narrow	r1	223.9717425	5	2	post_focus	4	Narrow post_focus
**2023407**	block2	Control	post	ma4	Subject	Narrow	r1	74.45966958	1	1	pre_focus	4	Narrow pre_focus
**2023407**	block2	Control	post	ma4	Subject	Narrow	r1	111.4811464	2	2	pre_focus	4	Narrow pre_focus
**2023407**	block2	Control	post	fu4	Verb	Narrow	r1	44.69652305	3	1	pre_focus	4	Narrow pre_focus
**2023407**	block2	Control	post	maang4	Object	Narrow	r1	208.8271578	4	1	on_focus	4	Narrow on_focus
**2023407**	block2	Control	post	Jan-04	Object	Narrow	r1	255.1490015	5	2	on_focus	4	Narrow on_focus
**2023407**	block2	Control	post	ma4	Subject	Contrastive	r1	85.19958248	1	1	pre_focus	4	Contrastive pre_focus
**2023407**	block2	Control	post	ma4	Subject	Contrastive	r1	104.5481447	2	2	pre_focus	4	Contrastive pre_focus
**2023407**	block2	Control	post	fu4	Verb	Contrastive	r1	49.46466232	3	1	on_focus	4	Contrastive on_focus
**2023407**	block2	Control	post	maang4	Object	Contrastive	r1	197.4316812	4	1	post_focus	4	Contrastive post_focus
**2023407**	block2	Control	post	Jan-04	Object	Contrastive	r1	203.4103115	5	2	post_focus	4	Contrastive post_focus
**2023407**	block2	Control	post	ngaa5	Subject	Narrow	r1	121.2214025	1	1	on_focus	5	Narrow on_focus
**2023407**	block2	Control	post	ngaa5	Subject	Narrow	r1	130.6934947	2	2	on_focus	5	Narrow on_focus
**2023407**	block2	Control	post	maai5	Verb	Narrow	r1	123.3840506	3	1	post_focus	5	Narrow post_focus
**2023407**	block2	Control	post	pou5	Object	Narrow	r1	97.4727204	4	1	post_focus	5	Narrow post_focus
**2023407**	block2	Control	post	pou5	Object	Narrow	r1	233.7064784	5	2	post_focus	5	Narrow post_focus
**2023407**	block2	Control	post	ngaa5	Subject	Narrow	r1	60.7381296	1	1	pre_focus	5	Narrow pre_focus
**2023407**	block2	Control	post	ngaa5	Subject	Narrow	r1	125.9715024	2	2	pre_focus	5	Narrow pre_focus
**2023407**	block2	Control	post	maai5	Verb	Narrow	r1	126.1274155	3	1	pre_focus	5	Narrow pre_focus
**2023407**	block2	Control	post	pou5	Object	Narrow	r1	59.69429747	4	1	on_focus	5	Narrow on_focus
**2023407**	block2	Control	post	pou5	Object	Narrow	r1	171.6417158	5	2	on_focus	5	Narrow on_focus
**2023407**	block2	Control	post	ma4	Subject	Narrow	r1	96.0174108	1	1	on_focus	4	Narrow on_focus
**2023407**	block2	Control	post	ma4	Subject	Narrow	r1	109.8348815	2	2	on_focus	4	Narrow on_focus
**2023407**	block2	Control	post	fu4	Verb	Narrow	r1	43.0697327	3	1	post_focus	4	Narrow post_focus
**2023407**	block2	Control	post	maang4	Object	Narrow	r1	162.7415215	4	1	post_focus	4	Narrow post_focus
**2023407**	block2	Control	post	Jan-04	Object	Narrow	r1	206.2537147	5	2	post_focus	4	Narrow post_focus
**2023407**	block2	Control	post	lok6	Subject	Narrow	r1	138.8464045	1	1	pre_focus	6	Narrow pre_focus
**2023407**	block2	Control	post	lok6	Subject	Narrow	r1	101.7704622	2	2	pre_focus	6	Narrow pre_focus
**2023407**	block2	Control	post	waa6	Verb	Narrow	r1	101.9150451	3	1	on_focus	6	Narrow on_focus
**2023407**	block2	Control	post	jyut6	Object	Narrow	r1	123.6357615	4	1	post_focus	6	Narrow post_focus
**2023407**	block2	Control	post	loeng6	Object	Narrow	r1	204.3152338	5	2	post_focus	6	Narrow post_focus
**2023407**	block2	Control	post	lok6	Subject	Narrow	r1	109.862742	1	1	on_focus	6	Narrow on_focus
**2023407**	block2	Control	post	lok6	Subject	Narrow	r1	109.8263956	2	2	on_focus	6	Narrow on_focus
**2023407**	block2	Control	post	waa6	Verb	Narrow	r1	91.57518208	3	1	post_focus	6	Narrow post_focus
**2023407**	block2	Control	post	jyut6	Object	Narrow	r1	85.81445803	4	1	post_focus	6	Narrow post_focus
**2023407**	block2	Control	post	loeng6	Object	Narrow	r1	241.9393634	5	2	post_focus	6	Narrow post_focus
**2023407**	block2	Control	post	ngaa5	Subject	Contrastive	r1	148.6099773	1	1	pre_focus	5	Contrastive pre_focus
**2023407**	block2	Control	post	ngaa5	Subject	Contrastive	r1	102.0096208	2	2	pre_focus	5	Contrastive pre_focus
**2023407**	block2	Control	post	maai5	Verb	Contrastive	r1	106.2015862	3	1	on_focus	5	Contrastive on_focus
**2023407**	block2	Control	post	pou5	Object	Contrastive	r1	86.81508238	4	1	post_focus	5	Contrastive post_focus
**2023407**	block2	Control	post	pou5	Object	Contrastive	r1	242.7337795	5	2	post_focus	5	Contrastive post_focus
**2023407**	block2	Control	post	ma4	Subject	Broad	r1	99.40413883	1	1	broad_focus	4	Broad focus
**2023407**	block2	Control	post	ma4	Subject	Broad	r1	133.8331397	2	2	broad_focus	4	Broad focus
**2023407**	block2	Control	post	fu4	Verb	Broad	r1	69.5393586	3	1	broad_focus	4	Broad focus
**2023407**	block2	Control	post	maang4	Object	Broad	r1	152.6696931	4	1	broad_focus	4	Broad focus
**2023407**	block2	Control	post	Jan-04	Object	Broad	r1	232.0662248	5	2	broad_focus	4	Broad focus
**2023407**	block2	Control	post	ngaa5	Subject	Narrow	r1	91.44737168	1	1	pre_focus	5	Narrow pre_focus
**2023407**	block2	Control	post	ngaa5	Subject	Narrow	r1	131.0985623	2	2	pre_focus	5	Narrow pre_focus
**2023407**	block2	Control	post	maai5	Verb	Narrow	r1	107.2262296	3	1	on_focus	5	Narrow on_focus
**2023407**	block2	Control	post	pou5	Object	Narrow	r1	87.29828202	4	1	post_focus	5	Narrow post_focus

2023407	block2	Control	post	pou5	Object	Narrow	r1	201.5110899	5	2	post_focus	5	Narrow post_focus
2023407	block2	Control	post	lok6	Subject	Narrow	r1	107.4653083	1	1	pre_focus	6	Narrow pre_focus
2023407	block2	Control	post	lok6	Subject	Narrow	r1	186.633516	2	2	pre_focus	6	Narrow pre_focus
2023407	block2	Control	post	waa6	Verb	Narrow	r1	108.0122563	3	1	pre_focus	6	Narrow pre_focus
2023407	block2	Control	post	jyut6	Object	Narrow	r1	136.0070504	4	1	on_focus	6	Narrow on_focus
2023407	block2	Control	post	loeng6	Object	Narrow	r1	237.7710011	5	2	on_focus	6	Narrow on_focus
2023407	block2	Control	post	ngaa5	Subject	Broad	r1	107.8123105	1	1	broad_focus	5	Broad focus
2023407	block2	Control	post	ngaa5	Subject	Broad	r1	137.963125	2	2	broad_focus	5	Broad focus
2023407	block2	Control	post	maai5	Verb	Broad	r1	120.0630069	3	1	broad_focus	5	Broad focus
2023407	block2	Control	post	pou5	Object	Broad	r1	72.66553288	4	1	broad_focus	5	Broad focus
2023407	block2	Control	post	pou5	Object	Broad	r1	234.0663154	5	2	broad_focus	5	Broad focus
2023407	block2	Control	post	ngaa5	Subject	Contrastive	r1	71.31590437	1	1	pre_focus	5	Contrastive pre_focus
2023407	block2	Control	post	ngaa5	Subject	Contrastive	r1	87.24480546	2	2	pre_focus	5	Contrastive pre_focus
2023407	block2	Control	post	maai5	Verb	Contrastive	r1	98.24848219	3	1	pre_focus	5	Contrastive pre_focus
2023407	block2	Control	post	pou5	Object	Contrastive	r1	53.08862434	4	1	on_focus	5	Contrastive on_focus
2023407	block2	Control	post	pou5	Object	Contrastive	r1	184.1747543	5	2	on_focus	5	Contrastive on_focus
2023407	block2	Control	post	ma4	Subject	Contrastive	r1	60.78841793	1	1	on_focus	4	Contrastive on_focus
2023407	block2	Control	post	ma4	Subject	Contrastive	r1	137.3409685	2	2	on_focus	4	Contrastive on_focus
2023407	block2	Control	post	fu4	Verb	Contrastive	r1	85.76344593	3	1	post_focus	4	Contrastive post_focus
2023407	block2	Control	post	maang4	Object	Contrastive	r1	87.37580716	4	1	post_focus	4	Contrastive post_focus
2023407	block2	Control	post	Jan-04	Object	Contrastive	r1	211.6831014	5	2	post_focus	4	Contrastive post_focus
2023407	block2	Control	post	ngaa5	Subject	Contrastive	r1	104.6543691	1	1	on_focus	5	Contrastive on_focus
2023407	block2	Control	post	ngaa5	Subject	Contrastive	r1	131.390011	2	2	on_focus	5	Contrastive on_focus
2023407	block2	Control	post	maai5	Verb	Contrastive	r1	93.36406238	3	1	post_focus	5	Contrastive post_focus
2023407	block2	Control	post	pou5	Object	Contrastive	r1	62.43337162	4	1	post_focus	5	Contrastive post_focus
2023407	block2	Control	post	pou5	Object	Contrastive	r1	186.0058626	5	2	post_focus	5	Contrastive post_focus
2023407	block2	Control	post	lok6	Subject	Contrastive	r1	129.8393961	1	1	on_focus	6	Contrastive on_focus
2023407	block2	Control	post	lok6	Subject	Contrastive	r1	102.3795526	2	2	on_focus	6	Contrastive on_focus
2023407	block2	Control	post	waa6	Verb	Contrastive	r1	92.96871351	3	1	post_focus	6	Contrastive post_focus
2023407	block2	Control	post	jyut6	Object	Contrastive	r1	94.72696141	4	1	post_focus	6	Contrastive post_focus
2023407	block2	Control	post	loeng6	Object	Contrastive	r1	245.2167938	5	2	post_focus	6	Contrastive post_focus
2023407	block2	Control	post	lok6	Subject	Contrastive	r1	114.839831	1	1	pre_focus	6	Contrastive pre_focus
2023407	block2	Control	post	lok6	Subject	Contrastive	r1	107.1629534	2	2	pre_focus	6	Contrastive pre_focus
2023407	block2	Control	post	waa6	Verb	Contrastive	r1	82.78940092	3	1	on_focus	6	Contrastive on_focus
2023407	block2	Control	post	jyut6	Object	Contrastive	r1	139.0920507	4	1	post_focus	6	Contrastive post_focus
2023407	block2	Control	post	loeng6	Object	Contrastive	r1	271.396741	5	2	post_focus	6	Contrastive post_focus
2023407	block2	Control	post	ngaa5	Subject	Contrastive	r2	174.7171832	1	1	on_focus	5	Contrastive on_focus
2023407	block2	Control	post	ngaa5	Subject	Contrastive	r2	111.270235	2	2	on_focus	5	Contrastive on_focus
2023407	block2	Control	post	maai5	Verb	Contrastive	r2	132.2262469	3	1	post_focus	5	Contrastive post_focus
2023407	block2	Control	post	pou5	Object	Contrastive	r2	105.7875711	4	1	post_focus	5	Contrastive post_focus
2023407	block2	Control	post	pou5	Object	Contrastive	r2	154.1918762	5	2	post_focus	5	Contrastive post_focus
2023407	block2	Control	post	lok6	Subject	Contrastive	r2	137.5765204	1	1	on_focus	6	Contrastive on_focus
2023407	block2	Control	post	lok6	Subject	Contrastive	r2	120.5922321	2	2	on_focus	6	Contrastive on_focus
2023407	block2	Control	post	waa6	Verb	Contrastive	r2	125.5561857	3	1	post_focus	6	Contrastive post_focus
2023407	block2	Control	post	jyut6	Object	Contrastive	r2	159.0878857	4	1	post_focus	6	Contrastive post_focus
2023407	block2	Control	post	loeng6	Object	Contrastive	r2	247.8508396	5	2	post_focus	6	Contrastive post_focus
2023407	block2	Control	post	lok6	Subject	Narrow	r2	143.5637013	1	1	pre_focus	6	Narrow pre_focus
2023407	block2	Control	post	lok6	Subject	Narrow	r2	131.590448	2	2	pre_focus	6	Narrow pre_focus
2023407	block2	Control	post	waa6	Verb	Narrow	r2	122.1824002	3	1	pre_focus	6	Narrow pre_focus
2023407	block2	Control	post	jyut6	Object	Narrow	r2	137.2748623	4	1	on_focus	6	Narrow on_focus
2023407	block2	Control	post	loeng6	Object	Narrow	r2	309.7950456	5	2	on_focus	6	Narrow on_focus
2023407	block2	Control	post	ma4	Subject	Contrastive	r2	103.790913	1	1	pre_focus	4	Contrastive pre_focus
2023407	block2	Control	post	ma4	Subject	Contrastive	r2	153.7565751	2	2	pre_focus	4	Contrastive pre_focus
2023407	block2	Control	post	fu4	Verb	Contrastive	r2	109.9319728	3	1	pre_focus	4	Contrastive pre_focus
2023407	block2	Control	post	maang4	Object	Contrastive	r2	181.1822712	4	1	on_focus	4	Contrastive on_focus
2023407	block2	Control	post	Jan-04	Object	Contrastive	r2	168.2105064	5	2	on_focus	4	Contrastive on_focus
2023407	block2	Control	post	ma4	Subject	Contrastive	r2	135.9547679	1	1	on_focus	4	Contrastive on_focus
2023407	block2	Control	post	ma4	Subject	Contrastive	r2	105.5993654	2	2	on_focus	4	Contrastive on_focus
2023407	block2	Control	post	fu4	Verb	Contrastive	r2	86.43580421	3	1	post_focus	4	Contrastive post_focus
2023407	block2	Control	post	maang4	Object	Contrastive	r2	157.6885229	4	1	post_focus	4	Contrastive post_focus
2023407	block2	Control	post	Jan-04	Object	Contrastive	r2	173.2700367	5	2	post_focus	4	Contrastive post_focus
2023407	block2	Control	post	ngaa5	Subject	Contrastive	r2	76.24968861	1	1	pre_focus	5	Contrastive pre_focus
2023407	block2	Control	post	ngaa5	Subject	Contrastive	r2	120.1542298	2	2	pre_focus	5	Contrastive pre_focus
2023407	block2	Control	post	maai5	Verb	Contrastive	r2	114.6079464	3	1	on_focus	5	Contrastive on_focus
2023407	block2	Control	post	pou5	Object	Contrastive	r2	136.0147225	4	1	post_focus	5	Contrastive post_focus
2023407	block2	Control	post	pou5	Object	Contrastive	r2	198.9407277	5	2	post_focus	5	Contrastive post_focus

2023407	block2	Control	post	lok6	Subject	Broad	r2	116.5269296	1	1	broad_focus	6	Broad focus
2023407	block2	Control	post	lok6	Subject	Broad	r2	134.1284345	2	2	broad_focus	6	Broad focus
2023407	block2	Control	post	waa6	Verb	Broad	r2	95.4986785	3	1	broad_focus	6	Broad focus
2023407	block2	Control	post	jyut6	Object	Broad	r2	102.2728667	4	1	broad_focus	6	Broad focus
2023407	block2	Control	post	loeng6	Object	Broad	r2	232.0431402	5	2	broad_focus	6	Broad focus
2023407	block2	Control	post	lok6	Subject	Narrow	r2	106.6230562	1	1	pre_focus	6	Narrow pre_focus
2023407	block2	Control	post	lok6	Subject	Narrow	r2	74.873383	2	2	pre_focus	6	Narrow pre_focus
2023407	block2	Control	post	waa6	Verb	Narrow	r2	110.1772714	3	1	on_focus	6	Narrow on_focus
2023407	block2	Control	post	jyut6	Object	Narrow	r2	113.5283447	4	1	post_focus	6	Narrow post_focus
2023407	block2	Control	post	loeng6	Object	Narrow	r2	298.7968362	5	2	post_focus	6	Narrow post_focus
2023407	block2	Control	post	ngaa5	Subject	Narrow	r2	95.3844533	1	1	pre_focus	5	Narrow pre_focus
2023407	block2	Control	post	ngaa5	Subject	Narrow	r2	151.3756001	2	2	pre_focus	5	Narrow pre_focus
2023407	block2	Control	post	maai5	Verb	Narrow	r2	125.2491982	3	1	on_focus	5	Narrow on_focus
2023407	block2	Control	post	pou5	Object	Narrow	r2	37.72127288	4	1	post_focus	5	Narrow post_focus
2023407	block2	Control	post	pou5	Object	Narrow	r2	243.9115646	5	2	post_focus	5	Narrow post_focus
2023407	block2	Control	post	ngaa5	Subject	Narrow	r2	105.4387532	1	1	on_focus	5	Narrow on_focus
2023407	block2	Control	post	ngaa5	Subject	Narrow	r2	125.8512937	2	2	on_focus	5	Narrow on_focus
2023407	block2	Control	post	maai5	Verb	Narrow	r2	125.5289283	3	1	post_focus	5	Narrow post_focus
2023407	block2	Control	post	pou5	Object	Narrow	r2	64.06451016	4	1	post_focus	5	Narrow post_focus
2023407	block2	Control	post	pou5	Object	Narrow	r2	179.4552111	5	2	post_focus	5	Narrow post_focus
2023407	block2	Control	post	ma4	Subject	Contrastive	r2	107.1805018	1	1	pre_focus	4	Contrastive pre_focus
2023407	block2	Control	post	ma4	Subject	Contrastive	r2	101.1638721	2	2	pre_focus	4	Contrastive pre_focus
2023407	block2	Control	post	fu4	Verb	Contrastive	r2	63.63705843	3	1	on_focus	4	Contrastive on_focus
2023407	block2	Control	post	maang4	Object	Contrastive	r2	208.7174192	4	1	post_focus	4	Contrastive post_focus
2023407	block2	Control	post	Jan-04	Object	Contrastive	r2	198.7533997	5	2	post_focus	4	Contrastive post_focus
2023407	block2	Control	post	ma4	Subject	Narrow	r2	116.9101784	1	1	on_focus	4	Narrow on_focus
2023407	block2	Control	post	ma4	Subject	Narrow	r2	156.7721961	2	2	on_focus	4	Narrow on_focus
2023407	block2	Control	post	fu4	Verb	Narrow	r2	75.99572217	3	1	post_focus	4	Narrow post_focus
2023407	block2	Control	post	maang4	Object	Narrow	r2	247.8962203	4	1	post_focus	4	Narrow post_focus
2023407	block2	Control	post	Jan-04	Object	Narrow	r2	234.0801457	5	2	post_focus	4	Narrow post_focus
2023407	block2	Control	post	ngaa5	Subject	Broad	r2	142.3971356	1	1	broad_focus	5	Broad focus
2023407	block2	Control	post	ngaa5	Subject	Broad	r2	120.0600011	2	2	broad_focus	5	Broad focus
2023407	block2	Control	post	maai5	Verb	Broad	r2	132.8696943	3	1	broad_focus	5	Broad focus
2023407	block2	Control	post	pou5	Object	Broad	r2	74.35492387	4	1	broad_focus	5	Broad focus
2023407	block2	Control	post	pou5	Object	Broad	r2	145.0931256	5	2	broad_focus	5	Broad focus
2023407	block2	Control	post	ngaa5	Subject	Contrastive	r2	168.669589	1	1	pre_focus	5	Contrastive pre_focus
2023407	block2	Control	post	ngaa5	Subject	Contrastive	r2	103.3390578	2	2	pre_focus	5	Contrastive pre_focus
2023407	block2	Control	post	maai5	Verb	Contrastive	r2	134.9399723	3	1	pre_focus	5	Contrastive pre_focus
2023407	block2	Control	post	pou5	Object	Contrastive	r2	73.16507555	4	1	on_focus	5	Contrastive on_focus
2023407	block2	Control	post	pou5	Object	Contrastive	r2	222.5762458	5	2	on_focus	5	Contrastive on_focus
2023407	block2	Control	post	lok6	Subject	Contrastive	r2	139.7588904	1	1	pre_focus	6	Contrastive pre_focus
2023407	block2	Control	post	lok6	Subject	Contrastive	r2	155.6034374	2	2	pre_focus	6	Contrastive pre_focus
2023407	block2	Control	post	waa6	Verb	Contrastive	r2	142.2873469	3	1	on_focus	6	Contrastive on_focus
2023407	block2	Control	post	jyut6	Object	Contrastive	r2	106.3071712	4	1	post_focus	6	Contrastive post_focus
2023407	block2	Control	post	loeng6	Object	Contrastive	r2	366.0452885	5	2	post_focus	6	Contrastive post_focus
2023407	block2	Control	post	lok6	Subject	Broad	r2	119.338777	1	1	broad_focus	6	Broad focus
2023407	block2	Control	post	lok6	Subject	Broad	r2	155.6605183	2	2	broad_focus	6	Broad focus
2023407	block2	Control	post	waa6	Verb	Broad	r2	130.5173686	3	1	broad_focus	6	Broad focus
2023407	block2	Control	post	jyut6	Object	Broad	r2	116.0846239	4	1	broad_focus	6	Broad focus
2023407	block2	Control	post	loeng6	Object	Broad	r2	293.6891602	5	2	broad_focus	6	Broad focus
2023407	block2	Control	post	ngaa5	Subject	Narrow	r2	185.4982835	1	1	pre_focus	5	Narrow pre_focus
2023407	block2	Control	post	ngaa5	Subject	Narrow	r2	261.0783722	2	2	pre_focus	5	Narrow pre_focus
2023407	block2	Control	post	maai5	Verb	Narrow	r2	361.0204969	3	1	pre_focus	5	Narrow pre_focus
2023407	block2	Control	post	pou5	Object	Narrow	r2	140.344782	4	1	on_focus	5	Narrow on_focus
2023407	block2	Control	post	pou5	Object	Narrow	r2	227.0845481	5	2	on_focus	5	Narrow on_focus
2023407	block2	Control	post	lok6	Subject	Narrow	r2	132.9948791	1	1	on_focus	6	Narrow on_focus
2023407	block2	Control	post	lok6	Subject	Narrow	r2	103.3240987	2	2	on_focus	6	Narrow on_focus
2023407	block2	Control	post	waa6	Verb	Narrow	r2	103.5470584	3	1	post_focus	6	Narrow post_focus
2023407	block2	Control	post	jyut6	Object	Narrow	r2	90.49979846	4	1	post_focus	6	Narrow post_focus
2023407	block2	Control	post	loeng6	Object	Narrow	r2	393.899119	5	2	post_focus	6	Narrow post_focus
2023407	block2	Control	post	ma4	Subject	Narrow	r2	75.96058863	1	1	pre_focus	4	Narrow pre_focus
2023407	block2	Control	post	ma4	Subject	Narrow	r2	123.3849095	2	2	pre_focus	4	Narrow pre_focus
2023407	block2	Control	post	fu4	Verb	Narrow	r2	86.63847593	3	1	pre_focus	4	Narrow pre_focus
2023407	block2	Control	post	maang4	Object	Narrow	r2	116.9720884	4	1	on_focus	4	Narrow on_focus
2023407	block2	Control	post	Jan-04	Object	Narrow	r2	258.3273732	5	2	on_focus	4	Narrow on_focus
2023407	block2	Control	post	lok6	Subject	Contrastive	r2	100.1273463	1	1	pre_focus	6	Contrastive pre_focus

**2023407**	block2	Control	post	lok6	Subject	Contrastive	r2	114.5342275	2	2	pre_focus	6	Contrastive pre_focus
**2023407**	block2	Control	post	waa6	Verb	Contrastive	r2	103.7893745	3	1	pre_focus	6	Contrastive pre_focus
**2023407**	block2	Control	post	jyut6	Object	Contrastive	r2	87.88926276	4	1	on_focus	6	Contrastive on_focus
**2023407**	block2	Control	post	loeng6	Object	Contrastive	r2	255.6643065	5	2	on_focus	6	Contrastive on_focus
**2023407**	block2	Control	post	ma4	Subject	Narrow	r2	79.70817423	1	1	pre_focus	4	Narrow pre_focus
**2023407**	block2	Control	post	ma4	Subject	Narrow	r2	130.5815606	2	2	pre_focus	4	Narrow pre_focus
**2023407**	block2	Control	post	fu4	Verb	Narrow	r2	61.75642612	3	1	on_focus	4	Narrow on_focus
**2023407**	block2	Control	post	maang4	Object	Narrow	r2	185.7774151	4	1	post_focus	4	Narrow post_focus
**2023407**	block2	Control	post	Jan-04	Object	Narrow	r2	375.0835558	5	2	post_focus	4	Narrow post_focus
**2023407**	block2	Control	pre	lok6	Subject	Contrastive	r1	220.8249064	1	1	on_focus	6	Contrastive on_focus
**2023407**	block2	Control	pre	lok6	Subject	Contrastive	r1	153.1440616	2	2	on_focus	6	Contrastive on_focus
**2023407**	block2	Control	pre	waa6	Verb	Contrastive	r1	102.2569352	3	1	post_focus	6	Contrastive post_focus
**2023407**	block2	Control	pre	jyut6	Object	Contrastive	r1	121.8942962	4	1	post_focus	6	Contrastive post_focus
**2023407**	block2	Control	pre	loeng6	Object	Contrastive	r1	594.0961479	5	2	post_focus	6	Contrastive post_focus
**2023407**	block2	Control	pre	ma4	Subject	Narrow	r1	215.8887581	1	1	pre_focus	4	Narrow pre_focus
**2023407**	block2	Control	pre	ma4	Subject	Narrow	r1	279.5297043	2	2	pre_focus	4	Narrow pre_focus
**2023407**	block2	Control	pre	fu4	Verb	Narrow	r1	274.3230827	3	1	on_focus	4	Narrow on_focus
**2023407**	block2	Control	pre	maang4	Object	Narrow	r1	270.2306596	4	1	post_focus	4	Narrow post_focus
**2023407**	block2	Control	pre	Jan-04	Object	Narrow	r1	468.5082559	5	2	post_focus	4	Narrow post_focus
**2023407**	block2	Control	pre	lok6	Subject	Contrastive	r1	142.2987386	1	1	pre_focus	6	Contrastive pre_focus
**2023407**	block2	Control	pre	lok6	Subject	Contrastive	r1	163.7003121	2	2	pre_focus	6	Contrastive pre_focus
**2023407**	block2	Control	pre	waa6	Verb	Contrastive	r1	108.3667106	3	1	pre_focus	6	Contrastive pre_focus
**2023407**	block2	Control	pre	jyut6	Object	Contrastive	r1	140.5064806	4	1	on_focus	6	Contrastive on_focus
**2023407**	block2	Control	pre	loeng6	Object	Contrastive	r1	314.0881654	5	2	on_focus	6	Contrastive on_focus
**2023407**	block2	Control	pre	ngaa5	Subject	Narrow	r1	238.7249949	1	1	pre_focus	5	Narrow pre_focus
**2023407**	block2	Control	pre	ngaa5	Subject	Narrow	r1	290.3192149	2	2	pre_focus	5	Narrow pre_focus
**2023407**	block2	Control	pre	maai5	Verb	Narrow	r1	176.3642171	3	1	pre_focus	5	Narrow pre_focus
**2023407**	block2	Control	pre	pou5	Object	Narrow	r1	106.0023168	4	1	on_focus	5	Narrow on_focus
**2023407**	block2	Control	pre	pou5	Object	Narrow	r1	241.7496525	5	2	on_focus	5	Narrow on_focus
**2023407**	block2	Control	pre	ngaa5	Subject	Contrastive	r1	192.3792824	1	1	pre_focus	5	Contrastive pre_focus
**2023407**	block2	Control	pre	ngaa5	Subject	Contrastive	r1	256.3813906	2	2	pre_focus	5	Contrastive pre_focus
**2023407**	block2	Control	pre	maai5	Verb	Contrastive	r1	176.3842066	3	1	pre_focus	5	Contrastive pre_focus
**2023407**	block2	Control	pre	pou5	Object	Contrastive	r1	90.84169869	4	1	on_focus	5	Contrastive on_focus
**2023407**	block2	Control	pre	pou5	Object	Contrastive	r1	231.3852411	5	2	on_focus	5	Contrastive on_focus
**2023407**	block2	Control	pre	lok6	Subject	Broad	r1	165.1410071	1	1	broad_focus	6	Broad focus
**2023407**	block2	Control	pre	lok6	Subject	Broad	r1	212.8936588	2	2	broad_focus	6	Broad focus
**2023407**	block2	Control	pre	waa6	Verb	Broad	r1	137.6593029	3	1	broad_focus	6	Broad focus
**2023407**	block2	Control	pre	jyut6	Object	Broad	r1	125.3934906	4	1	broad_focus	6	Broad focus
**2023407**	block2	Control	pre	loeng6	Object	Broad	r1	404.4011447	5	2	broad_focus	6	Broad focus
**2023407**	block2	Control	pre	lok6	Subject	Narrow	r1	156.2972836	1	1	pre_focus	6	Narrow pre_focus
**2023407**	block2	Control	pre	lok6	Subject	Narrow	r1	210.9006025	2	2	pre_focus	6	Narrow pre_focus
**2023407**	block2	Control	pre	waa6	Verb	Narrow	r1	143.3568549	3	1	on_focus	6	Narrow on_focus
**2023407**	block2	Control	pre	jyut6	Object	Narrow	r1	107.7912339	4	1	post_focus	6	Narrow post_focus
**2023407**	block2	Control	pre	loeng6	Object	Narrow	r1	392.7387881	5	2	post_focus	6	Narrow post_focus
**2023407**	block2	Control	pre	ngaa5	Subject	Contrastive	r1	334.1172582	1	1	pre_focus	5	Contrastive pre_focus
**2023407**	block2	Control	pre	ngaa5	Subject	Contrastive	r1	412.2819931	2	2	pre_focus	5	Contrastive pre_focus
**2023407**	block2	Control	pre	maai5	Verb	Contrastive	r1	303.459837	3	1	on_focus	5	Contrastive on_focus
**2023407**	block2	Control	pre	pou5	Object	Contrastive	r1	110.5108872	4	1	post_focus	5	Contrastive post_focus
**2023407**	block2	Control	pre	pou5	Object	Contrastive	r1	239.7432762	5	2	post_focus	5	Contrastive post_focus
**2023407**	block2	Control	pre	lok6	Subject	Contrastive	r1	184.8229885	1	1	pre_focus	6	Contrastive pre_focus
**2023407**	block2	Control	pre	lok6	Subject	Contrastive	r1	230.6758966	2	2	pre_focus	6	Contrastive pre_focus
**2023407**	block2	Control	pre	waa6	Verb	Contrastive	r1	165.0149693	3	1	on_focus	6	Contrastive on_focus
**2023407**	block2	Control	pre	jyut6	Object	Contrastive	r1	122.0680233	4	1	post_focus	6	Contrastive post_focus
**2023407**	block2	Control	pre	loeng6	Object	Contrastive	r1	374.1303697	5	2	post_focus	6	Contrastive post_focus
**2023407**	block2	Control	pre	ngaa5	Subject	Narrow	r1	279.5313555	1	1	on_focus	5	Narrow on_focus
**2023407**	block2	Control	pre	ngaa5	Subject	Narrow	r1	310.5556528	2	2	on_focus	5	Narrow on_focus
**2023407**	block2	Control	pre	maai5	Verb	Narrow	r1	244.5185382	3	1	post_focus	5	Narrow post_focus
**2023407**	block2	Control	pre	pou5	Object	Narrow	r1	110.7278217	4	1	post_focus	5	Narrow post_focus
**2023407**	block2	Control	pre	pou5	Object	Narrow	r1	239.185129	5	2	post_focus	5	Narrow post_focus
**2023407**	block2	Control	pre	ma4	Subject	Contrastive	r1	165.2128427	1	1	pre_focus	4	Contrastive pre_focus
**2023407**	block2	Control	pre	ma4	Subject	Contrastive	r1	283.3751575	2	2	pre_focus	4	Contrastive pre_focus
**2023407**	block2	Control	pre	fu4	Verb	Contrastive	r1	166.260603	3	1	on_focus	4	Contrastive on_focus
**2023407**	block2	Control	pre	maang4	Object	Contrastive	r1	305.5660878	4	1	post_focus	4	Contrastive post_focus
**2023407**	block2	Control	pre	Jan-04	Object	Contrastive	r1	306.8063785	5	2	post_focus	4	Contrastive post_focus
**2023407**	block2	Control	pre	lok6	Subject	Narrow	r1	172.8788856	1	1	pre_focus	6	Narrow pre_focus
**2023407**	block2	Control	pre	lok6	Subject	Narrow	r1	225.3084905	2	2	pre_focus	6	Narrow pre_focus

**2023407**	block2	Control	pre	waa6	Verb	Narrow	r1	186.291871	3	1	pre_focus	6	Narrow pre_focus
**2023407**	block2	Control	pre	jyut6	Object	Narrow	r1	165.4391534	4	1	on_focus	6	Narrow on_focus
**2023407**	block2	Control	pre	loeng6	Object	Narrow	r1	384.1851852	5	2	on_focus	6	Narrow on_focus
**2023407**	block2	Control	pre	ngaa5	Subject	Broad	r1	197.2482831	1	1	broad_focus	5	Broad focus
**2023407**	block2	Control	pre	ngaa5	Subject	Broad	r1	330.2865252	2	2	broad_focus	5	Broad focus
**2023407**	block2	Control	pre	maai5	Verb	Broad	r1	1015.114355	3	1	broad_focus	5	Broad focus
**2023407**	block2	Control	pre	pou5	Object	Broad	r1	55.72205425	4	1	broad_focus	5	Broad focus
**2023407**	block2	Control	pre	pou5	Object	Broad	r1	40.19532789	5	2	broad_focus	5	Broad focus
**2023407**	block2	Control	pre	ma4	Subject	Broad	r1	145.1505291	1	1	broad_focus	4	Broad focus
**2023407**	block2	Control	pre	ma4	Subject	Broad	r1	235.4092141	2	2	broad_focus	4	Broad focus
**2023407**	block2	Control	pre	fu4	Verb	Broad	r1	148.0455074	3	1	broad_focus	4	Broad focus
**2023407**	block2	Control	pre	maang4	Object	Broad	r1	282.5483405	4	1	broad_focus	4	Broad focus
**2023407**	block2	Control	pre	Jan-04	Object	Broad	r1	79.67045607	5	2	broad_focus	4	Broad focus
**2023407**	block2	Control	pre	ngaa5	Subject	Narrow	r1	224.2671614	1	1	pre_focus	5	Narrow pre_focus
**2023407**	block2	Control	pre	ngaa5	Subject	Narrow	r1	376.5480459	2	2	pre_focus	5	Narrow pre_focus
**2023407**	block2	Control	pre	maai5	Verb	Narrow	r1	236.6202523	3	1	on_focus	5	Narrow on_focus
**2023407**	block2	Control	pre	pou5	Object	Narrow	r1	58.97966856	4	1	post_focus	5	Narrow post_focus
**2023407**	block2	Control	pre	pou5	Object	Narrow	r1	110.8284486	5	2	post_focus	5	Narrow post_focus
**2023407**	block2	Control	pre	ma4	Subject	Contrastive	r1	200.7594434	1	1	pre_focus	4	Contrastive pre_focus
**2023407**	block2	Control	pre	ma4	Subject	Contrastive	r1	268.409423	2	2	pre_focus	4	Contrastive pre_focus
**2023407**	block2	Control	pre	fu4	Verb	Contrastive	r1	129.2885157	3	1	pre_focus	4	Contrastive pre_focus
**2023407**	block2	Control	pre	maang4	Object	Contrastive	r1	294.8080644	4	1	on_focus	4	Contrastive on_focus
**2023407**	block2	Control	pre	Jan-04	Object	Contrastive	r1	126.4143991	5	2	on_focus	4	Contrastive on_focus
**2023407**	block2	Control	pre	ma4	Subject	Narrow	r1	176.5098782	1	1	on_focus	4	Narrow on_focus
**2023407**	block2	Control	pre	ma4	Subject	Narrow	r1	255.7717777	2	2	on_focus	4	Narrow on_focus
**2023407**	block2	Control	pre	fu4	Verb	Narrow	r1	270.9181028	3	1	post_focus	4	Narrow post_focus
**2023407**	block2	Control	pre	maang4	Object	Narrow	r1	274.7687623	4	1	post_focus	4	Narrow post_focus
**2023407**	block2	Control	pre	Jan-04	Object	Narrow	r1	195.7962445	5	2	post_focus	4	Narrow post_focus
**2023407**	block2	Control	pre	lok6	Subject	Narrow	r1	211.3394156	1	1	on_focus	6	Narrow on_focus
**2023407**	block2	Control	pre	lok6	Subject	Narrow	r1	245.2113408	2	2	on_focus	6	Narrow on_focus
**2023407**	block2	Control	pre	waa6	Verb	Narrow	r1	178.7663909	3	1	post_focus	6	Narrow post_focus
**2023407**	block2	Control	pre	jyut6	Object	Narrow	r1	153.1125265	4	1	post_focus	6	Narrow post_focus
**2023407**	block2	Control	pre	loeng6	Object	Narrow	r1	342.6716969	5	2	post_focus	6	Narrow post_focus
**2023407**	block2	Control	pre	ma4	Subject	Contrastive	r1	159.8418249	1	1	on_focus	4	Contrastive on_focus
**2023407**	block2	Control	pre	ma4	Subject	Contrastive	r1	286.3193156	2	2	on_focus	4	Contrastive on_focus
**2023407**	block2	Control	pre	fu4	Verb	Contrastive	r1	154.637633	3	1	post_focus	4	Contrastive post_focus
**2023407**	block2	Control	pre	maang4	Object	Contrastive	r1	288.2025302	4	1	post_focus	4	Contrastive post_focus
**2023407**	block2	Control	pre	Jan-04	Object	Contrastive	r1	192.359565	5	2	post_focus	4	Contrastive post_focus
**2023407**	block2	Control	pre	ma4	Subject	Narrow	r1	184.1018717	1	1	pre_focus	4	Narrow pre_focus
**2023407**	block2	Control	pre	ma4	Subject	Narrow	r1	251.7358817	2	2	pre_focus	4	Narrow pre_focus
**2023407**	block2	Control	pre	fu4	Verb	Narrow	r1	440.8164345	3	1	pre_focus	4	Narrow pre_focus
**2023407**	block2	Control	pre	maang4	Object	Narrow	r1	268.6702993	4	1	on_focus	4	Narrow on_focus
**2023407**	block2	Control	pre	Jan-04	Object	Narrow	r1	120.999748	5	2	on_focus	4	Narrow on_focus
**2023407**	block2	Control	pre	ngaa5	Subject	Contrastive	r1	311.847907	1	1	on_focus	5	Contrastive on_focus
**2023407**	block2	Control	pre	ngaa5	Subject	Contrastive	r1	385.147102	2	2	on_focus	5	Contrastive on_focus
**2023407**	block2	Control	pre	maai5	Verb	Contrastive	r1	318.1649767	3	1	post_focus	5	Contrastive post_focus
**2023407**	block2	Control	pre	pou5	Object	Contrastive	r1	110.8868631	4	1	post_focus	5	Contrastive post_focus
**2023407**	block2	Control	pre	pou5	Object	Contrastive	r1	276.1770142	5	2	post_focus	5	Contrastive post_focus
**2023407**	block2	Control	pre	ngaa5	Subject	Narrow	r2	222.2260322	1	1	pre_focus	5	Narrow pre_focus
**2023407**	block2	Control	pre	ngaa5	Subject	Narrow	r2	334.7571319	2	2	pre_focus	5	Narrow pre_focus
**2023407**	block2	Control	pre	maai5	Verb	Narrow	r2	281.8024743	3	1	pre_focus	5	Narrow pre_focus
**2023407**	block2	Control	pre	pou5	Object	Narrow	r2	84.64764078	4	1	on_focus	5	Narrow on_focus
**2023407**	block2	Control	pre	pou5	Object	Narrow	r2	165.7234678	5	2	on_focus	5	Narrow on_focus
**2023407**	block2	Control	pre	ma4	Subject	Contrastive	r2	224.3424936	1	1	on_focus	4	Contrastive on_focus
**2023407**	block2	Control	pre	ma4	Subject	Contrastive	r2	252.0988895	2	2	on_focus	4	Contrastive on_focus
**2023407**	block2	Control	pre	fu4	Verb	Contrastive	r2	204.917467	3	1	post_focus	4	Contrastive post_focus
**2023407**	block2	Control	pre	maang4	Object	Contrastive	r2	248.5927936	4	1	post_focus	4	Contrastive post_focus
**2023407**	block2	Control	pre	Jan-04	Object	Contrastive	r2	144.8851114	5	2	post_focus	4	Contrastive post_focus
**2023407**	block2	Control	pre	lok6	Subject	Narrow	r2	180.7692617	1	1	pre_focus	6	Narrow pre_focus
**2023407**	block2	Control	pre	lok6	Subject	Narrow	r2	181.3094086	2	2	pre_focus	6	Narrow pre_focus
**2023407**	block2	Control	pre	waa6	Verb	Narrow	r2	214.4842188	3	1	pre_focus	6	Narrow pre_focus
**2023407**	block2	Control	pre	jyut6	Object	Narrow	r2	166.0610309	4	1	on_focus	6	Narrow on_focus
**2023407**	block2	Control	pre	loeng6	Object	Narrow	r2	459.8885213	5	2	on_focus	6	Narrow on_focus
**2023407**	block2	Control	pre	lok6	Subject	Contrastive	r2	160.7395182	1	1	pre_focus	6	Contrastive pre_focus
**2023407**	block2	Control	pre	lok6	Subject	Contrastive	r2	236.8176887	2	2	pre_focus	6	Contrastive pre_focus
**2023407**	block2	Control	pre	waa6	Verb	Contrastive	r2	157.2188109	3	1	pre_focus	6	Contrastive pre_focus

**2023407**	block2	Control	pre	jyut6	Object	Contrastive	r2	129.2814159	4	1	on_focus	6	Contrastive on_focus
**2023407**	block2	Control	pre	loeng6	Object	Contrastive	r2	295.2886412	5	2	on_focus	6	Contrastive on_focus
**2023407**	block2	Control	pre	ma4	Subject	Contrastive	r2	212.3965304	1	1	pre_focus	4	Contrastive pre_focus
**2023407**	block2	Control	pre	ma4	Subject	Contrastive	r2	299.1589962	2	2	pre_focus	4	Contrastive pre_focus
**2023407**	block2	Control	pre	fu4	Verb	Contrastive	r2	247.4224036	3	1	pre_focus	4	Contrastive pre_focus
**2023407**	block2	Control	pre	maang4	Object	Contrastive	r2	284.1536629	4	1	on_focus	4	Contrastive on_focus
**2023407**	block2	Control	pre	Jan-04	Object	Contrastive	r2	361.8324065	5	2	on_focus	4	Contrastive on_focus
**2023407**	block2	Control	pre	ma4	Subject	Contrastive	r2	237.3195804	1	1	pre_focus	4	Contrastive pre_focus
**2023407**	block2	Control	pre	ma4	Subject	Contrastive	r2	411.2581564	2	2	pre_focus	4	Contrastive pre_focus
**2023407**	block2	Control	pre	fu4	Verb	Contrastive	r2	298.0570308	3	1	on_focus	4	Contrastive on_focus
**2023407**	block2	Control	pre	maang4	Object	Contrastive	r2	328.5907811	4	1	post_focus	4	Contrastive post_focus
**2023407**	block2	Control	pre	Jan-04	Object	Contrastive	r2	282.9713111	5	2	post_focus	4	Contrastive post_focus
**2023407**	block2	Control	pre	ma4	Subject	Narrow	r2	220.0005374	1	1	on_focus	4	Narrow on_focus
**2023407**	block2	Control	pre	ma4	Subject	Narrow	r2	353.6825397	2	2	on_focus	4	Narrow on_focus
**2023407**	block2	Control	pre	fu4	Verb	Narrow	r2	267.4567271	3	1	post_focus	4	Narrow post_focus
**2023407**	block2	Control	pre	maang4	Object	Narrow	r2	293.9855861	4	1	post_focus	4	Narrow post_focus
**2023407**	block2	Control	pre	Jan-04	Object	Narrow	r2	370.5742812	5	2	post_focus	4	Narrow post_focus
**2023407**	block2	Control	pre	ma4	Subject	Narrow	r2	193.0058169	1	1	pre_focus	4	Narrow pre_focus
**2023407**	block2	Control	pre	ma4	Subject	Narrow	r2	350.7750977	2	2	pre_focus	4	Narrow pre_focus
**2023407**	block2	Control	pre	fu4	Verb	Narrow	r2	318.7096426	3	1	pre_focus	4	Narrow pre_focus
**2023407**	block2	Control	pre	maang4	Object	Narrow	r2	322.1874321	4	1	on_focus	4	Narrow on_focus
**2023407**	block2	Control	pre	Jan-04	Object	Narrow	r2	406.9786645	5	2	on_focus	4	Narrow on_focus
**2023407**	block2	Control	pre	lok6	Subject	Narrow	r2	208.5244024	1	1	pre_focus	6	Narrow pre_focus
**2023407**	block2	Control	pre	lok6	Subject	Narrow	r2	255.6960386	2	2	pre_focus	6	Narrow pre_focus
**2023407**	block2	Control	pre	waa6	Verb	Narrow	r2	236.6125472	3	1	on_focus	6	Narrow on_focus
**2023407**	block2	Control	pre	jyut6	Object	Narrow	r2	138.2160832	4	1	post_focus	6	Narrow post_focus
**2023407**	block2	Control	pre	loeng6	Object	Narrow	r2	444.500648	5	2	post_focus	6	Narrow post_focus
**2023407**	block2	Control	pre	ngaa5	Subject	Narrow	r2	240.8255013	1	1	pre_focus	5	Narrow pre_focus
**2023407**	block2	Control	pre	ngaa5	Subject	Narrow	r2	333.3322679	2	2	pre_focus	5	Narrow pre_focus
**2023407**	block2	Control	pre	maai5	Verb	Narrow	r2	345.4294581	3	1	on_focus	5	Narrow on_focus
**2023407**	block2	Control	pre	pou5	Object	Narrow	r2	116.5751681	4	1	post_focus	5	Narrow post_focus
**2023407**	block2	Control	pre	pou5	Object	Narrow	r2	261.4740718	5	2	post_focus	5	Narrow post_focus
**2023407**	block2	Control	pre	ngaa5	Subject	Contrastive	r2	244.1054422	1	1	pre_focus	5	Contrastive pre_focus
**2023407**	block2	Control	pre	ngaa5	Subject	Contrastive	r2	310.5944816	2	2	pre_focus	5	Contrastive pre_focus
**2023407**	block2	Control	pre	maai5	Verb	Contrastive	r2	224.3084201	3	1	pre_focus	5	Contrastive pre_focus
**2023407**	block2	Control	pre	pou5	Object	Contrastive	r2	106.098029	4	1	on_focus	5	Contrastive on_focus
**2023407**	block2	Control	pre	pou5	Object	Contrastive	r2	275.7106782	5	2	on_focus	5	Contrastive on_focus
**2023407**	block2	Control	pre	ngaa5	Subject	Contrastive	r2	207.6218265	1	1	pre_focus	5	Contrastive pre_focus
**2023407**	block2	Control	pre	ngaa5	Subject	Contrastive	r2	306.1283521	2	2	pre_focus	5	Contrastive pre_focus
**2023407**	block2	Control	pre	maai5	Verb	Contrastive	r2	298.2177849	3	1	on_focus	5	Contrastive on_focus
**2023407**	block2	Control	pre	pou5	Object	Contrastive	r2	173.3754465	4	1	post_focus	5	Contrastive post_focus
**2023407**	block2	Control	pre	pou5	Object	Contrastive	r2	251.722273	5	2	post_focus	5	Contrastive post_focus
**2023407**	block2	Control	pre	ngaa5	Subject	Narrow	r2	212.2214266	1	1	on_focus	5	Narrow on_focus
**2023407**	block2	Control	pre	ngaa5	Subject	Narrow	r2	284.110621	2	2	on_focus	5	Narrow on_focus
**2023407**	block2	Control	pre	maai5	Verb	Narrow	r2	275.2373975	3	1	post_focus	5	Narrow post_focus
**2023407**	block2	Control	pre	pou5	Object	Narrow	r2	97.29782839	4	1	post_focus	5	Narrow post_focus
**2023407**	block2	Control	pre	pou5	Object	Narrow	r2	216.7692131	5	2	post_focus	5	Narrow post_focus
**2023407**	block2	Control	pre	lok6	Subject	Contrastive	r2	189.5978899	1	1	on_focus	6	Contrastive on_focus
**2023407**	block2	Control	pre	lok6	Subject	Contrastive	r2	233.039075	2	2	on_focus	6	Contrastive on_focus
**2023407**	block2	Control	pre	waa6	Verb	Contrastive	r2	156.8854378	3	1	post_focus	6	Contrastive post_focus
**2023407**	block2	Control	pre	jyut6	Object	Contrastive	r2	125.4667409	4	1	post_focus	6	Contrastive post_focus
**2023407**	block2	Control	pre	loeng6	Object	Contrastive	r2	381.6972332	5	2	post_focus	6	Contrastive post_focus
**2023407**	block2	Control	pre	lok6	Subject	Narrow	r2	184.4836116	1	1	on_focus	6	Narrow on_focus
**2023407**	block2	Control	pre	lok6	Subject	Narrow	r2	220.3239843	2	2	on_focus	6	Narrow on_focus
**2023407**	block2	Control	pre	waa6	Verb	Narrow	r2	198.0966569	3	1	post_focus	6	Narrow post_focus
**2023407**	block2	Control	pre	jyut6	Object	Narrow	r2	158.4096955	4	1	post_focus	6	Narrow post_focus
**2023407**	block2	Control	pre	loeng6	Object	Narrow	r2	369.3291177	5	2	post_focus	6	Narrow post_focus
**2023407**	block2	Control	pre	ngaa5	Subject	Contrastive	r2	217.7495369	1	1	on_focus	5	Contrastive on_focus
**2023407**	block2	Control	pre	ngaa5	Subject	Contrastive	r2	298.466091	2	2	on_focus	5	Contrastive on_focus
**2023407**	block2	Control	pre	maai5	Verb	Contrastive	r2	420.2622838	3	1	post_focus	5	Contrastive post_focus
**2023407**	block2	Control	pre	pou5	Object	Contrastive	r2	62.48668175	4	1	post_focus	5	Contrastive post_focus
**2023407**	block2	Control	pre	pou5	Object	Contrastive	r2	266.8642695	5	2	post_focus	5	Contrastive post_focus
**2023407**	block2	Control	pre	lok6	Subject	Contrastive	r2	227.8199608	1	1	pre_focus	6	Contrastive pre_focus
**2023407**	block2	Control	pre	lok6	Subject	Contrastive	r2	290.8510979	2	2	pre_focus	6	Contrastive pre_focus
**2023407**	block2	Control	pre	waa6	Verb	Contrastive	r2	239.8073808	3	1	on_focus	6	Contrastive on_focus
**2023407**	block2	Control	pre	jyut6	Object	Contrastive	r2	133.7899616	4	1	post_focus	6	Contrastive post_focus

2023407	block2	Control	pre	loeng6	Object	Contrastive	r2	385.8598091	5	2	post_focus	6	Contrastive post_focus
2023407	block2	Control	pre	ngaa5	Subject	Broad	r2	221.4562212	1	1	broad_focus	5	Broad focus
2023407	block2	Control	pre	ngaa5	Subject	Broad	r2	432.7179968	2	2	broad_focus	5	Broad focus
2023407	block2	Control	pre	maai5	Verb	Broad	r2	343.0176952	3	1	broad_focus	5	Broad focus
2023407	block2	Control	pre	pou5	Object	Broad	r2	106.0999215	4	1	broad_focus	5	Broad focus
2023407	block2	Control	pre	pou5	Object	Broad	r2	274.9059156	5	2	broad_focus	5	Broad focus
2023407	block2	Control	pre	lok6	Subject	Broad	r2	291.690546	1	1	broad_focus	6	Broad focus
2023407	block2	Control	pre	lok6	Subject	Broad	r2	333.8649277	2	2	broad_focus	6	Broad focus
2023407	block2	Control	pre	waa6	Verb	Broad	r2	176.32977	3	1	broad_focus	6	Broad focus
2023407	block2	Control	pre	jyut6	Object	Broad	r2	141.746022	4	1	broad_focus	6	Broad focus
2023407	block2	Control	pre	loeng6	Object	Broad	r2	398.7109955	5	2	broad_focus	6	Broad focus
2023407	block2	Control	pre	ma4	Subject	Narrow	r2	211.6556037	1	1	pre_focus	4	Narrow pre_focus
2023407	block2	Control	pre	ma4	Subject	Narrow	r2	280.0968872	2	2	pre_focus	4	Narrow pre_focus
2023407	block2	Control	pre	fu4	Verb	Narrow	r2	196.7796254	3	1	on_focus	4	Narrow on_focus
2023407	block2	Control	pre	maang4	Object	Narrow	r2	312.6650305	4	1	post_focus	4	Narrow post_focus
2023407	block2	Control	pre	Jan-04	Object	Narrow	r2	190.6657249	5	2	post_focus	4	Narrow post_focus
2023407	block2	Control	pre	ma4	Subject	Broad	r2	247.9180434	1	1	broad_focus	4	Broad focus
2023407	block2	Control	pre	ma4	Subject	Broad	r2	319.2889378	2	2	broad_focus	4	Broad focus
2023407	block2	Control	pre	fu4	Verb	Broad	r2	192.2967025	3	1	broad_focus	4	Broad focus
2023407	block2	Control	pre	maang4	Object	Broad	r2	300.5140129	4	1	broad_focus	4	Broad focus
2023407	block2	Control	pre	Jan-04	Object	Broad	r2	196.721277	5	2	broad_focus	4	Broad focus
2023407	block3	Control	post	bui3	Subject	Narrow	r1	153.7904313	1	1	on_focus	3	Narrow on_focus
2023407	block3	Control	post	bui3	Subject	Narrow	r1	184.9038046	2	2	on_focus	3	Narrow on_focus
2023407	block3	Control	post	tsv1	Verb	Narrow	r1	160.8477097	3	1	post_focus	1	Narrow post_focus
2023407	block3	Control	post	fug1	Object	Narrow	r1	266.6874471	4	1	post_focus	1	Narrow post_focus
2023407	block3	Control	post	tshe1	Object	Narrow	r1	627.600907	5	2	post_focus	1	Narrow post_focus
2023407	block3	Control	post	suk1	Subject	Contrastive	r1	59.91213152	1	1	pre_focus	1	Contrastive pre_focus
2023407	block3	Control	post	suk1	Subject	Contrastive	r1	52.37969262	2	2	pre_focus	1	Contrastive pre_focus
2023407	block3	Control	post	sei2	Verb	Contrastive	r1	163.9002268	3	1	pre_focus	2	Contrastive pre_focus
2023407	block3	Control	post	svy2	Object	Contrastive	r1	160.542598	4	1	on_focus	2	Contrastive on_focus
2023407	block3	Control	post	kwo2	Object	Contrastive	r1	364.540155	5	2	on_focus	2	Contrastive on_focus
2023407	block3	Control	post	suk1	Subject	Broad	r1	66.47054809	1	1	broad_focus	1	Broad focus
2023407	block3	Control	post	suk1	Subject	Broad	r1	56.52209283	2	2	broad_focus	1	Broad focus
2023407	block3	Control	post	sei2	Verb	Broad	r1	140.4508152	3	1	broad_focus	2	Broad focus
2023407	block3	Control	post	svy2	Object	Broad	r1	123.0303885	4	1	broad_focus	2	Broad focus
2023407	block3	Control	post	kwo2	Object	Broad	r1	483.7154138	5	2	broad_focus	2	Broad focus
2023407	block3	Control	post	piu2	Subject	Broad	r1	365.0270907	1	1	broad_focus	2	Broad focus
2023407	block3	Control	post	tse2	Subject	Broad	r1	301.3204591	2	2	broad_focus	2	Broad focus
2023407	block3	Control	post	tsap1	Verb	Broad	r1	128.4591697	3	1	broad_focus	1	Broad focus
2023407	block3	Control	post	sy1	Object	Broad	r1	259.3986113	4	1	broad_focus	1	Broad focus
2023407	block3	Control	post	pau1	Object	Broad	r1	464.3310658	5	2	broad_focus	1	Broad focus
2023407	block3	Control	post	piu2	Subject	Narrow	r1	336.1226951	1	1	pre_focus	2	Narrow pre_focus
2023407	block3	Control	post	tse2	Subject	Narrow	r1	374.1430461	2	2	pre_focus	2	Narrow pre_focus
2023407	block3	Control	post	tsap1	Verb	Narrow	r1	162.3725349	3	1	pre_focus	1	Narrow pre_focus
2023407	block3	Control	post	sy1	Object	Narrow	r1	374.1560037	4	1	on_focus	1	Narrow on_focus
2023407	block3	Control	post	pau1	Object	Narrow	r1	803.0768325	5	2	on_focus	1	Narrow on_focus
2023407	block3	Control	post	piu2	Subject	Contrastive	r1	385.4444998	1	1	pre_focus	2	Contrastive pre_focus
2023407	block3	Control	post	tse2	Subject	Contrastive	r1	311.640438	2	2	pre_focus	2	Contrastive pre_focus
2023407	block3	Control	post	tsap1	Verb	Contrastive	r1	94.52398444	3	1	on_focus	1	Contrastive on_focus
2023407	block3	Control	post	sy1	Object	Contrastive	r1	256.6008727	4	1	post_focus	1	Contrastive post_focus
2023407	block3	Control	post	pau1	Object	Contrastive	r1	431.5317784	5	2	post_focus	1	Contrastive post_focus
2023407	block3	Control	post	bui3	Subject	Contrastive	r1	305.9863946	1	1	pre_focus	3	Contrastive pre_focus
2023407	block3	Control	post	bui3	Subject	Contrastive	r1	320.4581117	2	2	pre_focus	3	Contrastive pre_focus
2023407	block3	Control	post	tsv1	Verb	Contrastive	r1	273.2767501	3	1	pre_focus	1	Contrastive pre_focus
2023407	block3	Control	post	fug1	Object	Contrastive	r1	364.7600714	4	1	on_focus	1	Contrastive on_focus
2023407	block3	Control	post	tshe1	Object	Contrastive	r1	557.7862812	5	2	on_focus	1	Contrastive on_focus
2023407	block3	Control	post	bui3	Subject	Broad	r1	304.6296296	1	1	broad_focus	3	Broad focus
2023407	block3	Control	post	bui3	Subject	Broad	r1	315.5836903	2	2	broad_focus	3	Broad focus
2023407	block3	Control	post	tsv1	Verb	Broad	r1	288.5211526	3	1	broad_focus	1	Broad focus
2023407	block3	Control	post	fug1	Object	Broad	r1	346.688381	4	1	broad_focus	1	Broad focus
2023407	block3	Control	post	tshe1	Object	Broad	r1	578.3650306	5	2	broad_focus	1	Broad focus
2023407	block3	Control	post	bui3	Subject	Contrastive	r1	214.337716	1	1	pre_focus	3	Contrastive pre_focus
2023407	block3	Control	post	bui3	Subject	Contrastive	r1	280.097447	2	2	pre_focus	3	Contrastive pre_focus
2023407	block3	Control	post	tsv1	Verb	Contrastive	r1	275.6364282	3	1	on_focus	1	Contrastive on_focus
2023407	block3	Control	post	fug1	Object	Contrastive	r1	321.7010582	4	1	post_focus	1	Contrastive post_focus
2023407	block3	Control	post	tshe1	Object	Contrastive	r1	603.8619862	5	2	post_focus	1	Contrastive post_focus

**2023407**	block3	Control	post	bui3	Subject	Narrow	r1	223.6141474	1	1	pre_focus	3	Narrow pre_focus
**2023407**	block3	Control	post	bui3	Subject	Narrow	r1	223.5843861	2	2	pre_focus	3	Narrow pre_focus
**2023407**	block3	Control	post	tsv1	Verb	Narrow	r1	194.88067	3	1	pre_focus	1	Narrow pre_focus
**2023407**	block3	Control	post	fug1	Object	Narrow	r1	317.8364556	4	1	on_focus	1	Narrow on_focus
**2023407**	block3	Control	post	tshe1	Object	Narrow	r1	503.9461807	5	2	on_focus	1	Narrow on_focus
**2023407**	block3	Control	post	suk1	Subject	Narrow	r1	62.65592984	1	1	on_focus	1	Narrow on_focus
**2023407**	block3	Control	post	suk1	Subject	Narrow	r1	98.29595105	2	2	on_focus	1	Narrow on_focus
**2023407**	block3	Control	post	sei2	Verb	Narrow	r1	206.1433004	3	1	post_focus	2	Narrow post_focus
**2023407**	block3	Control	post	svy2	Object	Narrow	r1	312.0669068	4	1	post_focus	2	Narrow post_focus
**2023407**	block3	Control	post	kwo2	Object	Narrow	r1	553.895725	5	2	post_focus	2	Narrow post_focus
**2023407**	block3	Control	post	suk1	Subject	Narrow	r1	76.74745144	1	1	pre_focus	1	Narrow pre_focus
**2023407**	block3	Control	post	suk1	Subject	Narrow	r1	122.5709173	2	2	pre_focus	1	Narrow pre_focus
**2023407**	block3	Control	post	sei2	Verb	Narrow	r1	227.7280171	3	1	on_focus	2	Narrow on_focus
**2023407**	block3	Control	post	svy2	Object	Narrow	r1	253.4805629	4	1	post_focus	2	Narrow post_focus
**2023407**	block3	Control	post	kwo2	Object	Narrow	r1	463.2079689	5	2	post_focus	2	Narrow post_focus
**2023407**	block3	Control	post	bui3	Subject	Narrow	r1	248.9039616	1	1	pre_focus	3	Narrow pre_focus
**2023407**	block3	Control	post	bui3	Subject	Narrow	r1	225.0105734	2	2	pre_focus	3	Narrow pre_focus
**2023407**	block3	Control	post	tsv1	Verb	Narrow	r1	215.6812286	3	1	on_focus	1	Narrow on_focus
**2023407**	block3	Control	post	fug1	Object	Narrow	r1	294.0299824	4	1	post_focus	1	Narrow post_focus
**2023407**	block3	Control	post	tshe1	Object	Narrow	r1	520.6071182	5	2	post_focus	1	Narrow post_focus
**2023407**	block3	Control	post	suk1	Subject	Contrastive	r1	76.81096681	1	1	pre_focus	1	Contrastive pre_focus
**2023407**	block3	Control	post	suk1	Subject	Contrastive	r1	95.45007902	2	2	pre_focus	1	Contrastive pre_focus
**2023407**	block3	Control	post	sei2	Verb	Contrastive	r1	200.4310537	3	1	on_focus	2	Contrastive on_focus
**2023407**	block3	Control	post	svy2	Object	Contrastive	r1	231.0453515	4	1	post_focus	2	Contrastive post_focus
**2023407**	block3	Control	post	kwo2	Object	Contrastive	r1	399.4913117	5	2	post_focus	2	Contrastive post_focus
**2023407**	block3	Control	post	suk1	Subject	Narrow	r1	124.0550696	1	1	pre_focus	1	Narrow pre_focus
**2023407**	block3	Control	post	suk1	Subject	Narrow	r1	253.1738764	2	2	pre_focus	1	Narrow pre_focus
**2023407**	block3	Control	post	sei2	Verb	Narrow	r1	308.1353567	3	1	pre_focus	2	Narrow pre_focus
**2023407**	block3	Control	post	svy2	Object	Narrow	r1	389.2460317	4	1	on_focus	2	Narrow on_focus
**2023407**	block3	Control	post	kwo2	Object	Narrow	r1	473.8817281	5	2	on_focus	2	Narrow on_focus
**2023407**	block3	Control	post	piu2	Subject	Narrow	r1	177.5906706	1	1	on_focus	2	Narrow on_focus
**2023407**	block3	Control	post	tse2	Subject	Narrow	r1	143.6808153	2	2	on_focus	2	Narrow on_focus
**2023407**	block3	Control	post	tsap1	Verb	Narrow	r1	108.2961047	3	1	post_focus	1	Narrow post_focus
**2023407**	block3	Control	post	sy1	Object	Narrow	r1	98.0531074	4	1	post_focus	1	Narrow post_focus
**2023407**	block3	Control	post	pau1	Object	Narrow	r1	466.5929885	5	2	post_focus	1	Narrow post_focus
**2023407**	block3	Control	post	bui3	Subject	Contrastive	r1	147.1984432	1	1	on_focus	3	Contrastive on_focus
**2023407**	block3	Control	post	bui3	Subject	Contrastive	r1	165.2200302	2	2	on_focus	3	Contrastive on_focus
**2023407**	block3	Control	post	tsv1	Verb	Contrastive	r1	133.9621858	3	1	post_focus	1	Contrastive post_focus
**2023407**	block3	Control	post	fug1	Object	Contrastive	r1	178.1152712	4	1	post_focus	1	Contrastive post_focus
**2023407**	block3	Control	post	tshe1	Object	Contrastive	r1	250.251258	5	2	post_focus	1	Contrastive post_focus
**2023407**	block3	Control	post	suk1	Subject	Contrastive	r1	97.57095845	1	1	on_focus	1	Contrastive on_focus
**2023407**	block3	Control	post	suk1	Subject	Contrastive	r1	105.6746932	2	2	on_focus	1	Contrastive on_focus
**2023407**	block3	Control	post	sei2	Verb	Contrastive	r1	201.1836735	3	1	post_focus	2	Contrastive post_focus
**2023407**	block3	Control	post	svy2	Object	Contrastive	r1	200.7625526	4	1	post_focus	2	Contrastive post_focus
**2023407**	block3	Control	post	kwo2	Object	Contrastive	r1	635.0884943	5	2	post_focus	2	Contrastive post_focus
**2023407**	block3	Control	post	piu2	Subject	Contrastive	r1	247.5037793	1	1	pre_focus	2	Contrastive pre_focus
**2023407**	block3	Control	post	tse2	Subject	Contrastive	r1	235.2853903	2	2	pre_focus	2	Contrastive pre_focus
**2023407**	block3	Control	post	tsap1	Verb	Contrastive	r1	130.7437642	3	1	pre_focus	1	Contrastive pre_focus
**2023407**	block3	Control	post	sy1	Object	Contrastive	r1	241.1672037	4	1	on_focus	1	Contrastive on_focus
**2023407**	block3	Control	post	pau1	Object	Contrastive	r1	493.7556928	5	2	on_focus	1	Contrastive on_focus
**2023407**	block3	Control	post	piu2	Subject	Narrow	r1	344.365408	1	1	pre_focus	2	Narrow pre_focus
**2023407**	block3	Control	post	tse2	Subject	Narrow	r1	253.9708455	2	2	pre_focus	2	Narrow pre_focus
**2023407**	block3	Control	post	tsap1	Verb	Narrow	r1	175.2914031	3	1	on_focus	1	Narrow on_focus
**2023407**	block3	Control	post	sy1	Object	Narrow	r1	253.5355253	4	1	post_focus	1	Narrow post_focus
**2023407**	block3	Control	post	pau1	Object	Narrow	r1	400.2627146	5	2	post_focus	1	Narrow post_focus
**2023407**	block3	Control	post	piu2	Subject	Contrastive	r1	312.2494548	1	1	on_focus	2	Contrastive on_focus
**2023407**	block3	Control	post	tse2	Subject	Contrastive	r1	308.4457402	2	2	on_focus	2	Contrastive on_focus
**2023407**	block3	Control	post	tsap1	Verb	Contrastive	r1	92.91923118	3	1	post_focus	1	Contrastive post_focus
**2023407**	block3	Control	post	sy1	Object	Contrastive	r1	228.6401188	4	1	post_focus	1	Contrastive post_focus
**2023407**	block3	Control	post	pau1	Object	Contrastive	r1	351.4859277	5	2	post_focus	1	Contrastive post_focus
**2023407**	block3	Control	post	bui3	Subject	Broad	r2	165.2169412	1	1	broad_focus	3	Broad focus
**2023407**	block3	Control	post	bui3	Subject	Broad	r2	185.6742697	2	2	broad_focus	3	Broad focus
**2023407**	block3	Control	post	tsv1	Verb	Broad	r2	188.1831066	3	1	broad_focus	1	Broad focus
**2023407**	block3	Control	post	fug1	Object	Broad	r2	252.8518519	4	1	broad_focus	1	Broad focus
**2023407**	block3	Control	post	tshe1	Object	Broad	r2	516.6109491	5	2	broad_focus	1	Broad focus
**2023407**	block3	Control	post	suk1	Subject	Contrastive	r2	115.7842026	1	1	on_focus	1	Contrastive on_focus

**2023407**	block3	Control	post	suk1	Subject	Contrastive	r2	171.0846561	2	2	on_focus	1	Contrastive on_focus
**2023407**	block3	Control	post	sei2	Verb	Contrastive	r2	294.2510708	3	1	post_focus	2	Contrastive post_focus
**2023407**	block3	Control	post	svy2	Object	Contrastive	r2	518.4852608	4	1	post_focus	2	Contrastive post_focus
**2023407**	block3	Control	post	kwo2	Object	Contrastive	r2	445.5621196	5	2	post_focus	2	Contrastive post_focus
**2023407**	block3	Control	post	piu2	Subject	Contrastive	r2	374.5088482	1	1	pre_focus	2	Contrastive pre_focus
**2023407**	block3	Control	post	tse2	Subject	Contrastive	r2	308.0706454	2	2	pre_focus	2	Contrastive pre_focus
**2023407**	block3	Control	post	tsap1	Verb	Contrastive	r2	144.3274962	3	1	on_focus	1	Contrastive on_focus
**2023407**	block3	Control	post	sy1	Object	Contrastive	r2	266.4568725	4	1	post_focus	1	Contrastive post_focus
**2023407**	block3	Control	post	pau1	Object	Contrastive	r2	788.1321849	5	2	post_focus	1	Contrastive post_focus
**2023407**	block3	Control	post	suk1	Subject	Contrastive	r2	115.8944731	1	1	pre_focus	1	Contrastive pre_focus
**2023407**	block3	Control	post	suk1	Subject	Contrastive	r2	122.3905244	2	2	pre_focus	1	Contrastive pre_focus
**2023407**	block3	Control	post	sei2	Verb	Contrastive	r2	227.7078839	3	1	on_focus	2	Contrastive on_focus
**2023407**	block3	Control	post	svy2	Object	Contrastive	r2	276.690016	4	1	post_focus	2	Contrastive post_focus
**2023407**	block3	Control	post	kwo2	Object	Contrastive	r2	638.0700417	5	2	post_focus	2	Contrastive post_focus
**2023407**	block3	Control	post	piu2	Subject	Narrow	r2	340.4800793	1	1	pre_focus	2	Narrow pre_focus
**2023407**	block3	Control	post	tse2	Subject	Narrow	r2	371.2356215	2	2	pre_focus	2	Narrow pre_focus
**2023407**	block3	Control	post	tsap1	Verb	Narrow	r2	176.234372	3	1	on_focus	1	Narrow on_focus
**2023407**	block3	Control	post	sy1	Object	Narrow	r2	304.976223	4	1	post_focus	1	Narrow post_focus
**2023407**	block3	Control	post	pau1	Object	Narrow	r2	722.5085466	5	2	post_focus	1	Narrow post_focus
**2023407**	block3	Control	post	suk1	Subject	Narrow	r2	129.8124161	1	1	on_focus	1	Narrow on_focus
**2023407**	block3	Control	post	suk1	Subject	Narrow	r2	126.0473119	2	2	on_focus	1	Narrow on_focus
**2023407**	block3	Control	post	sei2	Verb	Narrow	r2	294.971106	3	1	post_focus	2	Narrow post_focus
**2023407**	block3	Control	post	svy2	Object	Narrow	r2	437.5907288	4	1	post_focus	2	Narrow post_focus
**2023407**	block3	Control	post	kwo2	Object	Narrow	r2	746.5458513	5	2	post_focus	2	Narrow post_focus
**2023407**	block3	Control	post	bui3	Subject	Narrow	r2	259.9408105	1	1	on_focus	3	Narrow on_focus
**2023407**	block3	Control	post	bui3	Subject	Narrow	r2	315.6357794	2	2	on_focus	3	Narrow on_focus
**2023407**	block3	Control	post	tsv1	Verb	Narrow	r2	273.2024917	3	1	post_focus	1	Narrow post_focus
**2023407**	block3	Control	post	fug1	Object	Narrow	r2	392.050332	4	1	post_focus	1	Narrow post_focus
**2023407**	block3	Control	post	tshe1	Object	Narrow	r2	518.0722211	5	2	post_focus	1	Narrow post_focus
**2023407**	block3	Control	post	bui3	Subject	Narrow	r2	259.9449721	1	1	pre_focus	3	Narrow pre_focus
**2023407**	block3	Control	post	bui3	Subject	Narrow	r2	231.1261887	2	2	pre_focus	3	Narrow pre_focus
**2023407**	block3	Control	post	tsv1	Verb	Narrow	r2	217.2003563	3	1	pre_focus	1	Narrow pre_focus
**2023407**	block3	Control	post	fug1	Object	Narrow	r2	302.063768	4	1	on_focus	1	Narrow on_focus
**2023407**	block3	Control	post	tshe1	Object	Narrow	r2	652.0742757	5	2	on_focus	1	Narrow on_focus
**2023407**	block3	Control	post	suk1	Subject	Contrastive	r2	48.16447626	1	1	pre_focus	1	Contrastive pre_focus
**2023407**	block3	Control	post	suk1	Subject	Contrastive	r2	49.36259662	2	2	pre_focus	1	Contrastive pre_focus
**2023407**	block3	Control	post	sei2	Verb	Contrastive	r2	210.840033	3	1	pre_focus	2	Contrastive pre_focus
**2023407**	block3	Control	post	svy2	Object	Contrastive	r2	223.8198696	4	1	on_focus	2	Contrastive on_focus
**2023407**	block3	Control	post	kwo2	Object	Contrastive	r2	405.9446111	5	2	on_focus	2	Contrastive on_focus
**2023407**	block3	Control	post	suk1	Subject	Narrow	r2	59.62630751	1	1	pre_focus	1	Narrow pre_focus
**2023407**	block3	Control	post	suk1	Subject	Narrow	r2	67.00151172	2	2	pre_focus	1	Narrow pre_focus
**2023407**	block3	Control	post	sei2	Verb	Narrow	r2	192.5972592	3	1	on_focus	2	Narrow on_focus
**2023407**	block3	Control	post	svy2	Object	Narrow	r2	242.3678149	4	1	post_focus	2	Narrow post_focus
**2023407**	block3	Control	post	kwo2	Object	Narrow	r2	751.3873428	5	2	post_focus	2	Narrow post_focus
**2023407**	block3	Control	post	piu2	Subject	Narrow	r2	236.1683404	1	1	pre_focus	2	Narrow pre_focus
**2023407**	block3	Control	post	tse2	Subject	Narrow	r2	204.0832523	2	2	pre_focus	2	Narrow pre_focus
**2023407**	block3	Control	post	tsap1	Verb	Narrow	r2	92.04495711	3	1	pre_focus	1	Narrow pre_focus
**2023407**	block3	Control	post	sy1	Object	Narrow	r2	211.2052821	4	1	on_focus	1	Narrow on_focus
**2023407**	block3	Control	post	pau1	Object	Narrow	r2	630.6258503	5	2	on_focus	1	Narrow on_focus
**2023407**	block3	Control	post	piu2	Subject	Contrastive	r2	376.0843984	1	1	on_focus	2	Contrastive on_focus
**2023407**	block3	Control	post	tse2	Subject	Contrastive	r2	259.1014889	2	2	on_focus	2	Contrastive on_focus
**2023407**	block3	Control	post	tsap1	Verb	Contrastive	r2	108.8142721	3	1	post_focus	1	Contrastive post_focus
**2023407**	block3	Control	post	sy1	Object	Contrastive	r2	248.3986928	4	1	post_focus	1	Contrastive post_focus
**2023407**	block3	Control	post	pau1	Object	Contrastive	r2	433.8934535	5	2	post_focus	1	Contrastive post_focus
**2023407**	block3	Control	post	piu2	Subject	Broad	r2	277.1612237	1	1	broad_focus	2	Broad focus
**2023407**	block3	Control	post	tse2	Subject	Broad	r2	389.054412	2	2	broad_focus	2	Broad focus
**2023407**	block3	Control	post	tsap1	Verb	Broad	r2	108.4491699	3	1	broad_focus	1	Broad focus
**2023407**	block3	Control	post	sy1	Object	Broad	r2	207.2840054	4	1	broad_focus	1	Broad focus
**2023407**	block3	Control	post	pau1	Object	Broad	r2	716.1228173	5	2	broad_focus	1	Broad focus
**2023407**	block3	Control	post	suk1	Subject	Broad	r2	160.6158706	1	1	broad_focus	1	Broad focus
**2023407**	block3	Control	post	suk1	Subject	Broad	r2	235.643206	2	2	broad_focus	1	Broad focus
**2023407**	block3	Control	post	sei2	Verb	Broad	r2	329.4555207	3	1	broad_focus	2	Broad focus
**2023407**	block3	Control	post	svy2	Object	Broad	r2	413.7197553	4	1	broad_focus	2	Broad focus
**2023407**	block3	Control	post	kwo2	Object	Broad	r2	711.6261252	5	2	broad_focus	2	Broad focus
**2023407**	block3	Control	post	bui3	Subject	Contrastive	r2	265.9836315	1	1	pre_focus	3	Contrastive pre_focus
**2023407**	block3	Control	post	bui3	Subject	Contrastive	r2	304.2710695	2	2	pre_focus	3	Contrastive pre_focus

2023407	block3	Control	post	tsv1	Verb	Contrastive	r2	340.6216931	3	1	on_focus	1	Contrastive on_focus
2023407	block3	Control	post	fug1	Object	Contrastive	r2	374.1426827	4	1	post_focus	1	Contrastive post_focus
2023407	block3	Control	post	tshe1	Object	Contrastive	r2	608.2588273	5	2	post_focus	1	Contrastive post_focus
2023407	block3	Control	post	piu2	Subject	Narrow	r2	400.0345521	1	1	on_focus	2	Narrow on_focus
2023407	block3	Control	post	tse2	Subject	Narrow	r2	371.1325673	2	2	on_focus	2	Narrow on_focus
2023407	block3	Control	post	tsap1	Verb	Narrow	r2	232.1184099	3	1	post_focus	1	Narrow post_focus
2023407	block3	Control	post	sy1	Object	Narrow	r2	371.1420722	4	1	post_focus	1	Narrow post_focus
2023407	block3	Control	post	pau1	Object	Narrow	r2	1473.075323	5	2	post_focus	1	Narrow post_focus
2023407	block3	Control	post	bui3	Subject	Narrow	r2	291.4559384	1	1	pre_focus	3	Narrow pre_focus
2023407	block3	Control	post	bui3	Subject	Narrow	r2	293.9880322	2	2	pre_focus	3	Narrow pre_focus
2023407	block3	Control	post	tsv1	Verb	Narrow	r2	288.1363448	3	1	on_focus	1	Narrow on_focus
2023407	block3	Control	post	fug1	Object	Narrow	r2	350.7303151	4	1	post_focus	1	Narrow post_focus
2023407	block3	Control	post	tshe1	Object	Narrow	r2	638.8055787	5	2	post_focus	1	Narrow post_focus
2023407	block3	Control	post	bui3	Subject	Contrastive	r2	262.2749298	1	1	on_focus	3	Contrastive on_focus
2023407	block3	Control	post	bui3	Subject	Contrastive	r2	291.1604308	2	2	on_focus	3	Contrastive on_focus
2023407	block3	Control	post	tsv1	Verb	Contrastive	r2	238.7471655	3	1	post_focus	1	Contrastive post_focus
2023407	block3	Control	post	fug1	Object	Contrastive	r2	389.7505669	4	1	post_focus	1	Contrastive post_focus
2023407	block3	Control	post	tshe1	Object	Contrastive	r2	562.9165808	5	2	post_focus	1	Contrastive post_focus
2023407	block3	Control	post	bui3	Subject	Contrastive	r2	312.5254587	1	1	pre_focus	3	Contrastive pre_focus
2023407	block3	Control	post	bui3	Subject	Contrastive	r2	318.8594256	2	2	pre_focus	3	Contrastive pre_focus
2023407	block3	Control	post	tsv1	Verb	Contrastive	r2	270.6836196	3	1	pre_focus	1	Contrastive pre_focus
2023407	block3	Control	post	fug1	Object	Contrastive	r2	332.9010342	4	1	on_focus	1	Contrastive on_focus
2023407	block3	Control	post	tshe1	Object	Contrastive	r2	1086.194485	5	2	on_focus	1	Contrastive on_focus
2023407	block3	Control	post	suk1	Subject	Narrow	r2	151.0728274	1	1	pre_focus	1	Narrow pre_focus
2023407	block3	Control	post	suk1	Subject	Narrow	r2	172.0748299	2	2	pre_focus	1	Narrow pre_focus
2023407	block3	Control	post	sei2	Verb	Narrow	r2	353.9855847	3	1	pre_focus	2	Narrow pre_focus
2023407	block3	Control	post	svy2	Object	Narrow	r2	413.1352986	4	1	on_focus	2	Narrow on_focus
2023407	block3	Control	post	kwo2	Object	Narrow	r2	545.0352734	5	2	on_focus	2	Narrow on_focus
2023407	block3	Control	post	piu2	Subject	Contrastive	r2	185.3583729	1	1	pre_focus	2	Contrastive pre_focus
2023407	block3	Control	post	tse2	Subject	Contrastive	r2	185.3885441	2	2	pre_focus	2	Contrastive pre_focus
2023407	block3	Control	post	tsap1	Verb	Contrastive	r2	87.75605655	3	1	pre_focus	1	Contrastive pre_focus
2023407	block3	Control	post	sy1	Object	Contrastive	r2	167.561625	4	1	on_focus	1	Contrastive on_focus
2023407	block3	Control	post	pau1	Object	Contrastive	r2	666.4461336	5	2	on_focus	1	Contrastive on_focus
2023407	block3	Control	pre	piu2	Subject	Narrow	r1	204.4470044	1	1	on_focus	2	Narrow on_focus
2023407	block3	Control	pre	tse2	Subject	Narrow	r1	149.4035801	2	2	on_focus	2	Narrow on_focus
2023407	block3	Control	pre	tsap1	Verb	Narrow	r1	47.18007793	3	1	post_focus	1	Narrow post_focus
2023407	block3	Control	pre	sy1	Object	Narrow	r1	90.4284827	4	1	post_focus	1	Narrow post_focus
2023407	block3	Control	pre	pau1	Object	Narrow	r1	306.3907759	5	2	post_focus	1	Narrow post_focus
2023407	block3	Control	pre	suk1	Subject	Narrow	r1	43.24840477	1	1	on_focus	1	Narrow on_focus
2023407	block3	Control	pre	suk1	Subject	Narrow	r1	55.04342425	2	2	on_focus	1	Narrow on_focus
2023407	block3	Control	pre	sei2	Verb	Narrow	r1	161.1985996	3	1	post_focus	2	Narrow post_focus
2023407	block3	Control	pre	svy2	Object	Narrow	r1	153.3352533	4	1	post_focus	2	Narrow post_focus
2023407	block3	Control	pre	kwo2	Object	Narrow	r1	428.6959569	5	2	post_focus	2	Narrow post_focus
2023407	block3	Control	pre	bui3	Subject	Narrow	r1	149.4035801	1	1	pre_focus	3	Narrow pre_focus
2023407	block3	Control	pre	bui3	Subject	Narrow	r1	176.9252922	2	2	pre_focus	3	Narrow pre_focus
2023407	block3	Control	pre	tsv1	Verb	Narrow	r1	129.7452143	3	1	pre_focus	1	Narrow pre_focus
2023407	block3	Control	pre	fug1	Object	Narrow	r1	206.4128409	4	1	on_focus	1	Narrow on_focus
2023407	block3	Control	pre	tshe1	Object	Narrow	r1	330.2605455	5	2	on_focus	1	Narrow on_focus
2023407	block3	Control	pre	suk1	Subject	Broad	r1	66.83844373	1	1	broad_focus	1	Broad focus
2023407	block3	Control	pre	suk1	Subject	Broad	r1	43.24840477	2	2	broad_focus	1	Broad focus
2023407	block3	Control	pre	sei2	Verb	Broad	r1	157.2669264	3	1	broad_focus	2	Broad focus
2023407	block3	Control	pre	svy2	Object	Broad	r1	161.1985996	4	1	broad_focus	2	Broad focus
2023407	block3	Control	pre	kwo2	Object	Broad	r1	416.757355	5	2	broad_focus	2	Broad focus
2023407	block3	Control	pre	piu2	Subject	Contrastive	r1	200.5153312	1	1	pre_focus	2	Contrastive pre_focus
2023407	block3	Control	pre	tse2	Subject	Contrastive	r1	206.4128409	2	2	pre_focus	2	Contrastive pre_focus
2023407	block3	Control	pre	tsap1	Verb	Contrastive	r1	51.11175109	3	1	pre_focus	1	Contrastive pre_focus
2023407	block3	Control	pre	sy1	Object	Contrastive	r1	135.642724	4	1	on_focus	1	Contrastive on_focus
2023407	block3	Control	pre	pau1	Object	Contrastive	r1	424.6207014	5	2	on_focus	1	Contrastive on_focus
2023407	block3	Control	pre	piu2	Subject	Contrastive	r1	198.5494946	1	1	on_focus	2	Contrastive on_focus
2023407	block3	Control	pre	tse2	Subject	Contrastive	r1	153.3352533	2	2	on_focus	2	Contrastive on_focus
2023407	block3	Control	pre	tsap1	Verb	Contrastive	r1	68.80428031	3	1	post_focus	1	Contrastive post_focus
2023407	block3	Control	pre	sy1	Object	Contrastive	r1	123.8477046	4	1	post_focus	1	Contrastive post_focus
2023407	block3	Control	pre	pau1	Object	Contrastive	r1	454.1082501	5	2	post_focus	1	Contrastive post_focus
2023407	block3	Control	pre	piu2	Subject	Contrastive	r1	192.6519849	1	1	pre_focus	2	Contrastive pre_focus
2023407	block3	Control	pre	tse2	Subject	Contrastive	r1	155.3010899	2	2	pre_focus	2	Contrastive pre_focus
2023407	block3	Control	pre	tsap1	Verb	Contrastive	r1	53.07758767	3	1	on_focus	1	Contrastive on_focus

**2023407**	block3	Control	pre	sy1	Object	Contrastive	r1	66.83844373	4	1	post_focus	1	Contrastive post_focus
**2023407**	block3	Control	pre	pau1	Object	Contrastive	r1	438.3815574	5	2	post_focus	1	Contrastive post_focus
**2023407**	block3	Control	pre	piu2	Subject	Broad	r1	208.3786775	1	1	broad_focus	2	Broad focus
**2023407**	block3	Control	pre	tse2	Subject	Broad	r1	151.3694167	2	2	broad_focus	2	Broad focus
**2023407**	block3	Control	pre	tsap1	Verb	Broad	r1	55.04342425	3	1	broad_focus	1	Broad focus
**2023407**	block3	Control	pre	sy1	Object	Broad	r1	47.18007793	4	1	broad_focus	1	Broad focus
**2023407**	block3	Control	pre	pau1	Object	Broad	r1	318.465526	5	2	broad_focus	1	Broad focus
**2023407**	block3	Control	pre	suk1	Subject	Contrastive	r1	49.14591451	1	1	on_focus	1	Contrastive on_focus
**2023407**	block3	Control	pre	suk1	Subject	Contrastive	r1	39.31673161	2	2	on_focus	1	Contrastive on_focus
**2023407**	block3	Control	pre	sei2	Verb	Contrastive	r1	167.0961093	3	1	post_focus	2	Contrastive post_focus
**2023407**	block3	Control	pre	svy2	Object	Contrastive	r1	169.0619459	4	1	post_focus	2	Contrastive post_focus
**2023407**	block3	Control	pre	kwo2	Object	Contrastive	r1	357.7822576	5	2	post_focus	2	Contrastive post_focus
**2023407**	block3	Control	pre	bui3	Subject	Contrastive	r1	157.2669264	1	1	on_focus	3	Contrastive on_focus
**2023407**	block3	Control	pre	bui3	Subject	Contrastive	r1	180.8569654	2	2	on_focus	3	Contrastive on_focus
**2023407**	block3	Control	pre	tsv1	Verb	Contrastive	r1	125.8135411	3	1	post_focus	1	Contrastive post_focus
**2023407**	block3	Control	pre	fug1	Object	Contrastive	r1	161.1985996	4	1	post_focus	1	Contrastive post_focus
**2023407**	block3	Control	pre	tshe1	Object	Contrastive	r1	446.2449038	5	2	post_focus	1	Contrastive post_focus
**2023407**	block3	Control	pre	bui3	Subject	Narrow	r1	213.2172339	1	1	on_focus	3	Narrow on_focus
**2023407**	block3	Control	pre	bui3	Subject	Narrow	r1	165.2999673	2	2	on_focus	3	Narrow on_focus
**2023407**	block3	Control	pre	tsv1	Verb	Narrow	r1	135.9519309	3	1	post_focus	1	Narrow post_focus
**2023407**	block3	Control	pre	fug1	Object	Narrow	r1	180.1832539	4	1	post_focus	1	Narrow post_focus
**2023407**	block3	Control	pre	tshe1	Object	Narrow	r1	332.2263821	5	2	post_focus	1	Narrow post_focus
**2023407**	block3	Control	pre	bui3	Subject	Contrastive	r1	117.9501948	1	1	pre_focus	3	Contrastive pre_focus
**2023407**	block3	Control	pre	bui3	Subject	Contrastive	r1	157.2669264	2	2	pre_focus	3	Contrastive pre_focus
**2023407**	block3	Control	pre	tsv1	Verb	Contrastive	r1	121.881868	3	1	pre_focus	1	Contrastive pre_focus
**2023407**	block3	Control	pre	fug1	Object	Contrastive	r1	176.9252922	4	1	on_focus	1	Contrastive on_focus
**2023407**	block3	Control	pre	tshe1	Object	Contrastive	r1	290.9438139	5	2	on_focus	1	Contrastive on_focus
**2023407**	block3	Control	pre	suk1	Subject	Contrastive	r1	64.87260715	1	1	pre_focus	1	Contrastive pre_focus
**2023407**	block3	Control	pre	suk1	Subject	Contrastive	r1	47.18007793	2	2	pre_focus	1	Contrastive pre_focus
**2023407**	block3	Control	pre	sei2	Verb	Contrastive	r1	125.8135411	3	1	pre_focus	2	Contrastive pre_focus
**2023407**	block3	Control	pre	svy2	Object	Contrastive	r1	114.0185217	4	1	on_focus	2	Contrastive on_focus
**2023407**	block3	Control	pre	kwo2	Object	Contrastive	r1	322.3971992	5	2	on_focus	2	Contrastive on_focus
**2023407**	block3	Control	pre	suk1	Subject	Contrastive	r1	43.24840477	1	1	pre_focus	1	Contrastive pre_focus
**2023407**	block3	Control	pre	suk1	Subject	Contrastive	r1	39.31673161	2	2	pre_focus	1	Contrastive pre_focus
**2023407**	block3	Control	pre	sei2	Verb	Contrastive	r1	171.0277825	3	1	on_focus	2	Contrastive on_focus
**2023407**	block3	Control	pre	svy2	Object	Contrastive	r1	139.5743972	4	1	post_focus	2	Contrastive post_focus
**2023407**	block3	Control	pre	kwo2	Object	Contrastive	r1	261.4562652	5	2	post_focus	2	Contrastive post_focus
**2023407**	block3	Control	pre	bui3	Subject	Broad	r1	115.9843582	1	1	broad_focus	3	Broad focus
**2023407**	block3	Control	pre	bui3	Subject	Broad	r1	174.9594557	2	2	broad_focus	3	Broad focus
**2023407**	block3	Control	pre	tsv1	Verb	Broad	r1	145.1033126	3	1	broad_focus	1	Broad focus
**2023407**	block3	Control	pre	fug1	Object	Broad	r1	182.822802	4	1	broad_focus	1	Broad focus
**2023407**	block3	Control	pre	tshe1	Object	Broad	r1	401.0306624	5	2	broad_focus	1	Broad focus
**2023407**	block3	Control	pre	piu2	Subject	Narrow	r1	190.6861483	1	1	pre_focus	2	Narrow pre_focus
**2023407**	block3	Control	pre	tse2	Subject	Narrow	r1	141.5402338	2	2	pre_focus	2	Narrow pre_focus
**2023407**	block3	Control	pre	tsap1	Verb	Narrow	r1	74.70179006	3	1	pre_focus	1	Narrow pre_focus
**2023407**	block3	Control	pre	sy1	Object	Narrow	r1	76.66762664	4	1	on_focus	1	Narrow on_focus
**2023407**	block3	Control	pre	pau1	Object	Narrow	r1	420.6890282	5	2	on_focus	1	Narrow on_focus
**2023407**	block3	Control	pre	bui3	Subject	Contrastive	r1	184.7886386	1	1	pre_focus	3	Contrastive pre_focus
**2023407**	block3	Control	pre	bui3	Subject	Contrastive	r1	102.2235022	2	2	pre_focus	3	Contrastive pre_focus
**2023407**	block3	Control	pre	tsv1	Verb	Contrastive	r1	125.8135411	3	1	on_focus	1	Contrastive on_focus
**2023407**	block3	Control	pre	fug1	Object	Contrastive	r1	165.1302728	4	1	post_focus	1	Contrastive post_focus
**2023407**	block3	Control	pre	tshe1	Object	Contrastive	r1	303.7380723	5	2	post_focus	1	Contrastive post_focus
**2023407**	block3	Control	pre	bui3	Subject	Narrow	r1	133.6768875	1	1	pre_focus	3	Narrow pre_focus
**2023407**	block3	Control	pre	bui3	Subject	Narrow	r1	151.3694167	2	2	pre_focus	3	Narrow pre_focus
**2023407**	block3	Control	pre	tsv1	Verb	Narrow	r1	112.0526851	3	1	on_focus	1	Narrow on_focus
**2023407**	block3	Control	pre	fug1	Object	Narrow	r1	169.6068035	4	1	post_focus	1	Narrow post_focus
**2023407**	block3	Control	pre	tshe1	Object	Narrow	r1	218.2078604	5	2	post_focus	1	Narrow post_focus
**2023407**	block3	Control	pre	suk1	Subject	Narrow	r1	26.92831208	1	1	pre_focus	1	Narrow pre_focus
**2023407**	block3	Control	pre	suk1	Subject	Narrow	r1	40.89304995	2	2	pre_focus	1	Narrow pre_focus
**2023407**	block3	Control	pre	sei2	Verb	Narrow	r1	125.8135411	3	1	pre_focus	2	Narrow pre_focus
**2023407**	block3	Control	pre	svy2	Object	Narrow	r1	117.9501948	4	1	on_focus	2	Narrow on_focus
**2023407**	block3	Control	pre	kwo2	Object	Narrow	r1	294.8754871	5	2	on_focus	2	Narrow on_focus
**2023407**	block3	Control	pre	suk1	Subject	Narrow	r1	55.04342425	1	1	pre_focus	1	Narrow pre_focus
**2023407**	block3	Control	pre	suk1	Subject	Narrow	r1	35.38505845	2	2	pre_focus	1	Narrow pre_focus
**2023407**	block3	Control	pre	sei2	Verb	Narrow	r1	190.6861483	3	1	on_focus	2	Narrow on_focus
**2023407**	block3	Control	pre	svy2	Object	Narrow	r1	155.3010899	4	1	post_focus	2	Narrow post_focus

**2023407**	block3	Control	pre	kwo2	Object	Narrow	r1	355.8164211	5	2	post_focus	2	Narrow post_focus
**2023407**	block3	Control	pre	piu2	Subject	Narrow	r1	200.5153312	1	1	pre_focus	2	Narrow pre_focus
**2023407**	block3	Control	pre	tse2	Subject	Narrow	r1	163.1644362	2	2	pre_focus	2	Narrow pre_focus
**2023407**	block3	Control	pre	tsap1	Verb	Narrow	r1	47.18007793	3	1	on_focus	1	Narrow on_focus
**2023407**	block3	Control	pre	sy1	Object	Narrow	r1	104.1893388	4	1	post_focus	1	Narrow post_focus
**2023407**	block3	Control	pre	pau1	Object	Narrow	r1	414.7915185	5	2	post_focus	1	Narrow post_focus
**2023407**	block3	Control	pre	bui3	Subject	Narrow	r2	226.0712067	1	1	pre_focus	3	Narrow pre_focus
**2023407**	block3	Control	pre	bui3	Subject	Narrow	r2	212.7040618	2	2	pre_focus	3	Narrow pre_focus
**2023407**	block3	Control	pre	tsv1	Verb	Narrow	r2	131.7110509	3	1	on_focus	1	Narrow on_focus
**2023407**	block3	Control	pre	fug1	Object	Narrow	r2	180.8569654	4	1	post_focus	1	Narrow post_focus
**2023407**	block3	Control	pre	tshe1	Object	Narrow	r2	218.2078604	5	2	post_focus	1	Narrow post_focus
**2023407**	block3	Control	pre	suk1	Subject	Contrastive	r2	62.90677057	1	1	pre_focus	1	Contrastive pre_focus
**2023407**	block3	Control	pre	suk1	Subject	Contrastive	r2	53.07758767	2	2	pre_focus	1	Contrastive pre_focus
**2023407**	block3	Control	pre	sei2	Verb	Contrastive	r2	143.5060704	3	1	pre_focus	2	Contrastive pre_focus
**2023407**	block3	Control	pre	svy2	Object	Contrastive	r2	108.1210119	4	1	on_focus	2	Contrastive on_focus
**2023407**	block3	Control	pre	kwo2	Object	Contrastive	r2	143.5060704	5	2	on_focus	2	Contrastive on_focus
**2023407**	block3	Control	pre	bui3	Subject	Contrastive	r2	125.8135411	1	1	pre_focus	3	Contrastive pre_focus
**2023407**	block3	Control	pre	bui3	Subject	Contrastive	r2	149.4035801	2	2	pre_focus	3	Contrastive pre_focus
**2023407**	block3	Control	pre	tsv1	Verb	Contrastive	r2	110.0868485	3	1	pre_focus	1	Contrastive pre_focus
**2023407**	block3	Control	pre	fug1	Object	Contrastive	r2	149.4035801	4	1	on_focus	1	Contrastive on_focus
**2023407**	block3	Control	pre	tshe1	Object	Contrastive	r2	279.1487944	5	2	on_focus	1	Contrastive on_focus
**2023407**	block3	Control	pre	piu2	Subject	Contrastive	r2	385.3039698	1	1	pre_focus	2	Contrastive pre_focus
**2023407**	block3	Control	pre	tse2	Subject	Contrastive	r2	298.8071602	2	2	pre_focus	2	Contrastive pre_focus
**2023407**	block3	Control	pre	tsap1	Verb	Contrastive	r2	62.90677057	3	1	pre_focus	1	Contrastive pre_focus
**2023407**	block3	Control	pre	sy1	Object	Contrastive	r2	162.5501122	4	1	on_focus	1	Contrastive on_focus
**2023407**	block3	Control	pre	pau1	Object	Contrastive	r2	440.347394	5	2	on_focus	1	Contrastive on_focus
**2023407**	block3	Control	pre	suk1	Subject	Contrastive	r2	51.11175109	1	1	on_focus	1	Contrastive on_focus
**2023407**	block3	Control	pre	suk1	Subject	Contrastive	r2	47.18007793	2	2	on_focus	1	Contrastive on_focus
**2023407**	block3	Control	pre	sei2	Verb	Contrastive	r2	165.1302728	3	1	post_focus	2	Contrastive post_focus
**2023407**	block3	Control	pre	svy2	Object	Contrastive	r2	161.1985996	4	1	post_focus	2	Contrastive post_focus
**2023407**	block3	Control	pre	kwo2	Object	Contrastive	r2	357.7822576	5	2	post_focus	2	Contrastive post_focus
**2023407**	block3	Control	pre	bui3	Subject	Narrow	r2	137.6085606	1	1	on_focus	3	Narrow on_focus
**2023407**	block3	Control	pre	bui3	Subject	Narrow	r2	165.1302728	2	2	on_focus	3	Narrow on_focus
**2023407**	block3	Control	pre	tsv1	Verb	Narrow	r2	161.1985996	3	1	post_focus	1	Narrow post_focus
**2023407**	block3	Control	pre	fug1	Object	Narrow	r2	176.9252922	4	1	post_focus	1	Narrow post_focus
**2023407**	block3	Control	pre	tshe1	Object	Narrow	r2	255.5587555	5	2	post_focus	1	Narrow post_focus
**2023407**	block3	Control	pre	piu2	Subject	Contrastive	r2	161.1985996	1	1	pre_focus	2	Contrastive pre_focus
**2023407**	block3	Control	pre	tse2	Subject	Contrastive	r2	163.1644362	2	2	pre_focus	2	Contrastive pre_focus
**2023407**	block3	Control	pre	tsap1	Verb	Contrastive	r2	41.28256819	3	1	on_focus	1	Contrastive on_focus
**2023407**	block3	Control	pre	sy1	Object	Contrastive	r2	62.90677057	4	1	post_focus	1	Contrastive post_focus
**2023407**	block3	Control	pre	pau1	Object	Contrastive	r2	369.5772771	5	2	post_focus	1	Contrastive post_focus
**2023407**	block3	Control	pre	piu2	Subject	Contrastive	r2	178.8911288	1	1	on_focus	2	Contrastive on_focus
**2023407**	block3	Control	pre	tse2	Subject	Contrastive	r2	143.5060704	2	2	on_focus	2	Contrastive on_focus
**2023407**	block3	Control	pre	tsap1	Verb	Contrastive	r2	47.76452516	3	1	post_focus	1	Contrastive post_focus
**2023407**	block3	Control	pre	sy1	Object	Contrastive	r2	75.38891804	4	1	post_focus	1	Contrastive post_focus
**2023407**	block3	Control	pre	pau1	Object	Contrastive	r2	305.0621335	5	2	post_focus	1	Contrastive post_focus
**2023407**	block3	Control	pre	suk1	Subject	Narrow	r2	73.63568738	1	1	pre_focus	1	Narrow pre_focus
**2023407**	block3	Control	pre	suk1	Subject	Narrow	r2	66.62276478	2	2	pre_focus	1	Narrow pre_focus
**2023407**	block3	Control	pre	sei2	Verb	Narrow	r2	150.7778361	3	1	pre_focus	2	Narrow pre_focus
**2023407**	block3	Control	pre	svy2	Object	Narrow	r2	140.2584522	4	1	on_focus	2	Narrow on_focus
**2023407**	block3	Control	pre	kwo2	Object	Narrow	r2	441.8141243	5	2	on_focus	2	Narrow on_focus
**2023407**	block3	Control	pre	bui3	Subject	Contrastive	r2	206.8812169	1	1	pre_focus	3	Contrastive pre_focus
**2023407**	block3	Control	pre	bui3	Subject	Contrastive	r2	133.2455296	2	2	pre_focus	3	Contrastive pre_focus
**2023407**	block3	Control	pre	tsv1	Verb	Contrastive	r2	115.713223	3	1	on_focus	1	Contrastive on_focus
**2023407**	block3	Control	pre	fug1	Object	Contrastive	r2	133.2455296	4	1	post_focus	1	Contrastive post_focus
**2023407**	block3	Control	pre	tshe1	Object	Contrastive	r2	266.4910591	5	2	post_focus	1	Contrastive post_focus
**2023407**	block3	Control	pre	bui3	Subject	Broad	r2	161.29722	1	1	broad_focus	3	Broad focus
**2023407**	block3	Control	pre	bui3	Subject	Broad	r2	150.7778361	2	2	broad_focus	3	Broad focus
**2023407**	block3	Control	pre	tsv1	Verb	Broad	r2	171.8166039	3	1	broad_focus	1	Broad focus
**2023407**	block3	Control	pre	fug1	Object	Broad	r2	168.3101426	4	1	broad_focus	1	Broad focus
**2023407**	block3	Control	pre	tshe1	Object	Broad	r2	515.4498117	5	2	broad_focus	1	Broad focus
**2023407**	block3	Control	pre	piu2	Subject	Narrow	r2	164.8036813	1	1	pre_focus	2	Narrow pre_focus
**2023407**	block3	Control	pre	tse2	Subject	Narrow	r2	203.3747556	2	2	pre_focus	2	Narrow pre_focus
**2023407**	block3	Control	pre	tsap1	Verb	Narrow	r2	66.62276478	3	1	pre_focus	1	Narrow pre_focus
**2023407**	block3	Control	pre	sy1	Object	Narrow	r2	98.18091651	4	1	on_focus	1	Narrow on_focus
**2023407**	block3	Control	pre	pau1	Object	Narrow	r2	504.9304278	5	2	on_focus	1	Narrow on_focus

**2023407**	block3	Control	pre	piu2	Subject	Narrow	r2	185.8424491	1	1	on_focus	2	Narrow on_focus
**2023407**	block3	Control	pre	tse2	Subject	Narrow	r2	140.2584522	2	2	on_focus	2	Narrow on_focus
**2023407**	block3	Control	pre	tsap1	Verb	Narrow	r2	56.10338086	3	1	post_focus	1	Narrow post_focus
**2023407**	block3	Control	pre	sy1	Object	Narrow	r2	87.6615326	4	1	post_focus	1	Narrow post_focus
**2023407**	block3	Control	pre	pau1	Object	Narrow	r2	248.5204449	5	2	post_focus	1	Narrow post_focus
**2023407**	block3	Control	pre	piu2	Subject	Broad	r2	162.2842239	1	1	broad_focus	2	Broad focus
**2023407**	block3	Control	pre	tse2	Subject	Broad	r2	153.0108397	2	2	broad_focus	2	Broad focus
**2023407**	block3	Control	pre	tsap1	Verb	Broad	r2	69.55038168	3	1	broad_focus	1	Broad focus
**2023407**	block3	Control	pre	sy1	Object	Broad	r2	69.55038168	4	1	broad_focus	1	Broad focus
**2023407**	block3	Control	pre	pau1	Object	Broad	r2	421.9389822	5	2	broad_focus	1	Broad focus
**2023407**	block3	Control	pre	suk1	Subject	Broad	r2	48.87491949	1	1	broad_focus	1	Broad focus
**2023407**	block3	Control	pre	suk1	Subject	Broad	r2	51.00361323	2	2	broad_focus	1	Broad focus
**2023407**	block3	Control	pre	sei2	Verb	Broad	r2	171.5576081	3	1	broad_focus	2	Broad focus
**2023407**	block3	Control	pre	svy2	Object	Broad	r2	162.4054574	4	1	broad_focus	2	Broad focus
**2023407**	block3	Control	pre	kwo2	Object	Broad	r2	355.142091	5	2	broad_focus	2	Broad focus
**2023407**	block3	Control	pre	suk1	Subject	Narrow	r2	67.23203562	1	1	pre_focus	1	Narrow pre_focus
**2023407**	block3	Control	pre	suk1	Subject	Narrow	r2	62.59534351	2	2	pre_focus	1	Narrow pre_focus
**2023407**	block3	Control	pre	sei2	Verb	Narrow	r2	150.6924936	3	1	on_focus	2	Narrow on_focus
**2023407**	block3	Control	pre	svy2	Object	Narrow	r2	139.1007634	4	1	post_focus	2	Narrow post_focus
**2023407**	block3	Control	pre	kwo2	Object	Narrow	r2	340.7968702	5	2	post_focus	2	Narrow post_focus
**2023407**	block3	Control	pre	suk1	Subject	Contrastive	r2	30.13849873	1	1	pre_focus	1	Contrastive pre_focus
**2023407**	block3	Control	pre	suk1	Subject	Contrastive	r2	60.27699745	2	2	pre_focus	1	Contrastive pre_focus
**2023407**	block3	Control	pre	sei2	Verb	Contrastive	r2	184.9128557	3	1	on_focus	2	Contrastive on_focus
**2023407**	block3	Control	pre	svy2	Object	Contrastive	r2	143.7374555	4	1	post_focus	2	Contrastive post_focus
**2023407**	block3	Control	pre	kwo2	Object	Contrastive	r2	319.9317557	5	2	post_focus	2	Contrastive post_focus
**2023407**	block3	Control	pre	bui3	Subject	Narrow	r2	148.3741476	1	1	pre_focus	3	Narrow pre_focus
**2023407**	block3	Control	pre	bui3	Subject	Narrow	r2	148.3741476	2	2	pre_focus	3	Narrow pre_focus
**2023407**	block3	Control	pre	tsv1	Verb	Narrow	r2	185.4676845	3	1	pre_focus	1	Narrow pre_focus
**2023407**	block3	Control	pre	fug1	Object	Narrow	r2	201.6961069	4	1	on_focus	1	Narrow on_focus
**2023407**	block3	Control	pre	tshe1	Object	Narrow	r2	472.9425954	5	2	on_focus	1	Narrow on_focus
**2023407**	block3	Control	pre	suk1	Subject	Narrow	r2	32.45684478	1	1	on_focus	1	Narrow on_focus
**2023407**	block3	Control	pre	suk1	Subject	Narrow	r2	32.45684478	2	2	on_focus	1	Narrow on_focus
**2023407**	block3	Control	pre	sei2	Verb	Narrow	r2	118.2356488	3	1	post_focus	2	Narrow post_focus
**2023407**	block3	Control	pre	svy2	Object	Narrow	r2	122.872341	4	1	post_focus	2	Narrow post_focus
**2023407**	block3	Control	pre	kwo2	Object	Narrow	r2	315.2950636	5	2	post_focus	2	Narrow post_focus
**2023407**	block3	Control	pre	bui3	Subject	Contrastive	r2	127.5090331	1	1	on_focus	3	Contrastive on_focus
**2023407**	block3	Control	pre	bui3	Subject	Contrastive	r2	159.9658779	2	2	on_focus	3	Contrastive on_focus
**2023407**	block3	Control	pre	tsv1	Verb	Contrastive	r2	113.5989567	3	1	post_focus	1	Contrastive post_focus
**2023407**	block3	Control	pre	fug1	Object	Contrastive	r2	150.6924936	4	1	post_focus	1	Contrastive post_focus
**2023407**	block3	Control	pre	tshe1	Object	Contrastive	r2	224.3724292	5	2	post_focus	1	Contrastive post_focus
**2023407**	block3	Control	pre	piu2	Subject	Narrow	r2	173.8759542	1	1	pre_focus	2	Narrow pre_focus
**2023407**	block3	Control	pre	tse2	Subject	Narrow	r2	139.1007634	2	2	pre_focus	2	Narrow pre_focus
**2023407**	block3	Control	pre	tsap1	Verb	Narrow	r2	57.9586514	3	1	on_focus	1	Narrow on_focus
**2023407**	block3	Control	pre	sy1	Object	Narrow	r2	81.14211195	4	1	post_focus	1	Narrow post_focus
**2023407**	block3	Control	pre	pau1	Object	Narrow	r2	375.572061	5	2	post_focus	1	Narrow post_focus
**2023407**	block4	Control	post	Jan-01	Subject	Narrow	r1	282.2619048	1	1	on_focus	1	Narrow on_focus
**2023407**	block4	Control	post	Jan-01	Subject	Narrow	r1	263.4271194	2	2	on_focus	1	Narrow on_focus
**2023407**	block4	Control	post	wei3	Verb	Narrow	r1	211.8314437	3	1	post_focus	3	Narrow post_focus
**2023407**	block4	Control	post	tsam3	Object	Narrow	r1	263.7643012	4	1	post_focus	3	Narrow post_focus
**2023407**	block4	Control	post	tsam3	Object	Narrow	r1	596.7938916	5	2	post_focus	3	Narrow post_focus
**2023407**	block4	Control	post	Jan-01	Subject	Contrastive	r1	250.4001924	1	1	pre_focus	1	Contrastive pre_focus
**2023407**	block4	Control	post	Jan-01	Subject	Contrastive	r1	207.9648526	2	2	pre_focus	1	Contrastive pre_focus
**2023407**	block4	Control	post	wei3	Verb	Contrastive	r1	250.6880416	3	1	pre_focus	3	Contrastive pre_focus
**2023407**	block4	Control	post	tsam3	Object	Contrastive	r1	286.7401361	4	1	on_focus	3	Contrastive on_focus
**2023407**	block4	Control	post	tsam3	Object	Contrastive	r1	604.9697279	5	2	on_focus	3	Contrastive on_focus
**2023407**	block4	Control	post	piu35	Subject	Narrow	r1	208.4405751	1	1	pre_focus	35	Narrow pre_focus
**2023407**	block4	Control	post	mui35	Subject	Narrow	r1	236.5991548	2	2	pre_focus	35	Narrow pre_focus
**2023407**	block4	Control	post	tsan3	Verb	Narrow	r1	283.8897908	3	1	pre_focus	3	Narrow pre_focus
**2023407**	block4	Control	post	jin3	Object	Narrow	r1	310.4787841	4	1	on_focus	3	Narrow on_focus
**2023407**	block4	Control	post	jin3	Object	Narrow	r1	460.8205988	5	2	on_focus	3	Narrow on_focus
**2023407**	block4	Control	post	piu35	Subject	Narrow	r1	195.526833	1	1	pre_focus	35	Narrow pre_focus
**2023407**	block4	Control	post	mui35	Subject	Narrow	r1	267.7123517	2	2	pre_focus	35	Narrow pre_focus
**2023407**	block4	Control	post	tsan3	Verb	Narrow	r1	266.7658972	3	1	on_focus	3	Narrow on_focus
**2023407**	block4	Control	post	jin3	Object	Narrow	r1	305.7586924	4	1	post_focus	3	Narrow post_focus
**2023407**	block4	Control	post	jin3	Object	Narrow	r1	425.2721088	5	2	post_focus	3	Narrow post_focus
**2023407**	block4	Control	post	piu35	Subject	Contrastive	r1	202.3365247	1	1	pre_focus	35	Contrastive pre_focus

2023407	block4	Control	post	mui35	Subject	Contrastive	r1	306.0500316	2	2	pre_focus	35	Contrastive pre_focus
2023407	block4	Control	post	tsan3	Verb	Contrastive	r1	287.1581523	3	1	pre_focus	3	Contrastive pre_focus
2023407	block4	Control	post	jin3	Object	Contrastive	r1	279.9692883	4	1	on_focus	3	Contrastive on_focus
2023407	block4	Control	post	jin3	Object	Contrastive	r1	530.3368967	5	2	on_focus	3	Contrastive on_focus
2023407	block4	Control	post	piu35	Subject	Broad	r1	168.399066	1	1	broad_focus	35	Broad focus
2023407	block4	Control	post	mui35	Subject	Broad	r1	253.901718	2	2	broad_focus	35	Broad focus
2023407	block4	Control	post	tsan3	Verb	Broad	r1	246.3511335	3	1	broad_focus	3	Broad focus
2023407	block4	Control	post	jin3	Object	Broad	r1	275.7142317	4	1	broad_focus	3	Broad focus
2023407	block4	Control	post	jin3	Object	Broad	r1	417.1369623	5	2	broad_focus	3	Broad focus
2023407	block4	Control	post	Jan-01	Subject	Narrow	r1	176.3845575	1	1	pre_focus	1	Narrow pre_focus
2023407	block4	Control	post	Jan-01	Subject	Narrow	r1	213.34178	2	2	pre_focus	1	Narrow pre_focus
2023407	block4	Control	post	wei3	Verb	Narrow	r1	202.4357909	3	1	pre_focus	3	Narrow pre_focus
2023407	block4	Control	post	tsam3	Object	Narrow	r1	247.8328474	4	1	on_focus	3	Narrow on_focus
2023407	block4	Control	post	tsam3	Object	Narrow	r1	589.4245266	5	2	on_focus	3	Narrow on_focus
2023407	block4	Control	post	pak3	Subject	Narrow	r1	94.05459747	1	1	pre_focus	3	Narrow pre_focus
2023407	block4	Control	post	pak3	Subject	Narrow	r1	121.985216	2	2	pre_focus	3	Narrow pre_focus
2023407	block4	Control	post	tsing2	Verb	Narrow	r1	243.9538076	3	1	on_focus	2	Narrow on_focus
2023407	block4	Control	post	kau2	Object	Narrow	r1	259.5975909	4	1	post_focus	2	Narrow post_focus
2023407	block4	Control	post	tsi2	Object	Narrow	r1	424.6936804	5	2	post_focus	2	Narrow post_focus
2023407	block4	Control	post	Jan-01	Subject	Broad	r1	276.42651	1	1	broad_focus	1	Broad focus
2023407	block4	Control	post	Jan-01	Subject	Broad	r1	246.9265121	2	2	broad_focus	1	Broad focus
2023407	block4	Control	post	wei3	Verb	Broad	r1	229.1310482	3	1	broad_focus	3	Broad focus
2023407	block4	Control	post	tsam3	Object	Broad	r1	248.3843881	4	1	broad_focus	3	Broad focus
2023407	block4	Control	post	tsam3	Object	Broad	r1	582.405281	5	2	broad_focus	3	Broad focus
2023407	block4	Control	post	pak3	Subject	Contrastive	r1	89.20952316	1	1	pre_focus	3	Contrastive pre_focus
2023407	block4	Control	post	pak3	Subject	Contrastive	r1	114.1259317	2	2	pre_focus	3	Contrastive pre_focus
2023407	block4	Control	post	tsing2	Verb	Contrastive	r1	231.6104362	3	1	pre_focus	2	Contrastive pre_focus
2023407	block4	Control	post	kau2	Object	Contrastive	r1	231.3920068	4	1	on_focus	2	Contrastive on_focus
2023407	block4	Control	post	tsi2	Object	Contrastive	r1	451.979003	5	2	on_focus	2	Contrastive on_focus
2023407	block4	Control	post	piu35	Subject	Contrastive	r1	180.2576417	1	1	pre_focus	35	Contrastive pre_focus
2023407	block4	Control	post	mui35	Subject	Contrastive	r1	321.7300299	2	2	pre_focus	35	Contrastive pre_focus
2023407	block4	Control	post	tsan3	Verb	Contrastive	r1	273.9402891	3	1	on_focus	3	Contrastive on_focus
2023407	block4	Control	post	jin3	Object	Contrastive	r1	273.0490588	4	1	post_focus	3	Contrastive post_focus
2023407	block4	Control	post	jin3	Object	Contrastive	r1	384.9563379	5	2	post_focus	3	Contrastive post_focus
2023407	block4	Control	post	piu35	Subject	Contrastive	r1	185.9994199	1	1	on_focus	35	Contrastive on_focus
2023407	block4	Control	post	mui35	Subject	Contrastive	r1	282.6437628	2	2	on_focus	35	Contrastive on_focus
2023407	block4	Control	post	tsan3	Verb	Contrastive	r1	306.0188871	3	1	post_focus	3	Contrastive post_focus
2023407	block4	Control	post	jin3	Object	Contrastive	r1	257.8115509	4	1	post_focus	3	Contrastive post_focus
2023407	block4	Control	post	jin3	Object	Contrastive	r1	406.7088599	5	2	post_focus	3	Contrastive post_focus
2023407	block4	Control	post	Jan-01	Subject	Contrastive	r1	213.5291669	1	1	on_focus	1	Contrastive on_focus
2023407	block4	Control	post	Jan-01	Subject	Contrastive	r1	265.1679318	2	2	on_focus	1	Contrastive on_focus
2023407	block4	Control	post	wei3	Verb	Contrastive	r1	178.5322427	3	1	post_focus	3	Contrastive post_focus
2023407	block4	Control	post	tsam3	Object	Contrastive	r1	251.1518002	4	1	post_focus	3	Contrastive post_focus
2023407	block4	Control	post	tsam3	Object	Contrastive	r1	535.5604424	5	2	post_focus	3	Contrastive post_focus
2023407	block4	Control	post	pak3	Subject	Contrastive	r1	110.0629184	1	1	on_focus	3	Contrastive on_focus
2023407	block4	Control	post	pak3	Subject	Contrastive	r1	129.5688591	2	2	on_focus	3	Contrastive on_focus
2023407	block4	Control	post	tsing2	Verb	Contrastive	r1	200.2629981	3	1	post_focus	2	Contrastive post_focus
2023407	block4	Control	post	kau2	Object	Contrastive	r1	212.3151846	4	1	post_focus	2	Contrastive post_focus
2023407	block4	Control	post	tsi2	Object	Contrastive	r1	349.8320106	5	2	post_focus	2	Contrastive post_focus
2023407	block4	Control	post	pak3	Subject	Narrow	r1	105.8125355	1	1	pre_focus	3	Narrow pre_focus
2023407	block4	Control	post	pak3	Subject	Narrow	r1	133.4699667	2	2	pre_focus	3	Narrow pre_focus
2023407	block4	Control	post	tsing2	Verb	Narrow	r1	204.7231173	3	1	pre_focus	2	Narrow pre_focus
2023407	block4	Control	post	kau2	Object	Narrow	r1	220.9494608	4	1	on_focus	2	Narrow on_focus
2023407	block4	Control	post	tsi2	Object	Narrow	r1	374.4278609	5	2	on_focus	2	Narrow on_focus
2023407	block4	Control	post	pak3	Subject	Narrow	r1	126.11911	1	1	on_focus	3	Narrow on_focus
2023407	block4	Control	post	pak3	Subject	Narrow	r1	160.4658516	2	2	on_focus	3	Narrow on_focus
2023407	block4	Control	post	tsing2	Verb	Narrow	r1	259.1871705	3	1	post_focus	2	Narrow post_focus
2023407	block4	Control	post	kau2	Object	Narrow	r1	220.3022468	4	1	post_focus	2	Narrow post_focus
2023407	block4	Control	post	tsi2	Object	Narrow	r1	515.4179621	5	2	post_focus	2	Narrow post_focus
2023407	block4	Control	post	pak3	Subject	Broad	r1	117.8720137	1	1	broad_focus	3	Broad focus
2023407	block4	Control	post	pak3	Subject	Broad	r1	114.0389795	2	2	broad_focus	3	Broad focus
2023407	block4	Control	post	tsing2	Verb	Broad	r1	196.0002795	3	1	broad_focus	2	Broad focus
2023407	block4	Control	post	kau2	Object	Broad	r1	232.2237652	4	1	broad_focus	2	Broad focus
2023407	block4	Control	post	tsi2	Object	Broad	r1	479.7856428	5	2	broad_focus	2	Broad focus
2023407	block4	Control	post	Jan-01	Subject	Contrastive	r1	267.0021454	1	1	pre_focus	1	Contrastive pre_focus
2023407	block4	Control	post	Jan-01	Subject	Contrastive	r1	226.2575815	2	2	pre_focus	1	Contrastive pre_focus

2023407	block4	Control	post	wei3	Verb	Contrastive	r1	239.8679483	3	1	on_focus	3	Contrastive on_focus
2023407	block4	Control	post	tsam3	Object	Contrastive	r1	267.3258532	4	1	post_focus	3	Contrastive post_focus
2023407	block4	Control	post	tsam3	Object	Contrastive	r1	520.5042854	5	2	post_focus	3	Contrastive post_focus
2023407	block4	Control	post	Jan-01	Subject	Narrow	r1	213.7052067	1	1	pre_focus	1	Narrow pre_focus
2023407	block4	Control	post	Jan-01	Subject	Narrow	r1	245.9483955	2	2	pre_focus	1	Narrow pre_focus
2023407	block4	Control	post	wei3	Verb	Narrow	r1	203.4130011	3	1	on_focus	3	Narrow on_focus
2023407	block4	Control	post	tsam3	Object	Narrow	r1	250.825211	4	1	post_focus	3	Narrow post_focus
2023407	block4	Control	post	tsam3	Object	Narrow	r1	501.0059762	5	2	post_focus	3	Narrow post_focus
2023407	block4	Control	post	piu35	Subject	Narrow	r1	170.40805	1	1	on_focus	35	Narrow on_focus
2023407	block4	Control	post	mui35	Subject	Narrow	r1	279.2922008	2	2	on_focus	35	Narrow on_focus
2023407	block4	Control	post	tsan3	Verb	Narrow	r1	228.0194856	3	1	post_focus	3	Narrow post_focus
2023407	block4	Control	post	jin3	Object	Narrow	r1	267.5894715	4	1	post_focus	3	Narrow post_focus
2023407	block4	Control	post	jin3	Object	Narrow	r1	501.8140977	5	2	post_focus	3	Narrow post_focus
2023407	block4	Control	post	pak3	Subject	Contrastive	r1	102.4071942	1	1	pre_focus	3	Contrastive pre_focus
2023407	block4	Control	post	pak3	Subject	Contrastive	r1	133.6303033	2	2	pre_focus	3	Contrastive pre_focus
2023407	block4	Control	post	tsing2	Verb	Contrastive	r1	213.1653378	3	1	on_focus	2	Contrastive on_focus
2023407	block4	Control	post	kau2	Object	Contrastive	r1	236.7167665	4	1	post_focus	2	Contrastive post_focus
2023407	block4	Control	post	tsi2	Object	Contrastive	r1	480.7209543	5	2	post_focus	2	Contrastive post_focus
2023407	block4	Control	post	pak3	Subject	Broad	r2	115.7154417	1	1	broad_focus	3	Broad focus
2023407	block4	Control	post	pak3	Subject	Broad	r2	129.6625332	2	2	broad_focus	3	Broad focus
2023407	block4	Control	post	tsing2	Verb	Broad	r2	197.1826685	3	1	broad_focus	2	Broad focus
2023407	block4	Control	post	kau2	Object	Broad	r2	226.1421013	4	1	broad_focus	2	Broad focus
2023407	block4	Control	post	tsi2	Object	Broad	r2	386.2369918	5	2	broad_focus	2	Broad focus
2023407	block4	Control	post	pak3	Subject	Narrow	r2	92.43539958	1	1	pre_focus	3	Narrow pre_focus
2023407	block4	Control	post	pak3	Subject	Narrow	r2	115.1758953	2	2	pre_focus	3	Narrow pre_focus
2023407	block4	Control	post	tsing2	Verb	Narrow	r2	214.6687535	3	1	pre_focus	2	Narrow pre_focus
2023407	block4	Control	post	kau2	Object	Narrow	r2	256.2806012	4	1	on_focus	2	Narrow on_focus
2023407	block4	Control	post	tsi2	Object	Narrow	r2	495.824263	5	2	on_focus	2	Narrow on_focus
2023407	block4	Control	post	piu35	Subject	Narrow	r2	196.3640254	1	1	pre_focus	35	Narrow pre_focus
2023407	block4	Control	post	mui35	Subject	Narrow	r2	260.3868286	2	2	pre_focus	35	Narrow pre_focus
2023407	block4	Control	post	tsan3	Verb	Narrow	r2	284.7935038	3	1	pre_focus	3	Narrow pre_focus
2023407	block4	Control	post	jin3	Object	Narrow	r2	260.2316836	4	1	on_focus	3	Narrow on_focus
2023407	block4	Control	post	jin3	Object	Narrow	r2	401.4487167	5	2	on_focus	3	Narrow on_focus
2023407	block4	Control	post	Jan-01	Subject	Broad	r2	218.9866848	1	1	broad_focus	1	Broad focus
2023407	block4	Control	post	Jan-01	Subject	Broad	r2	269.567171	2	2	broad_focus	1	Broad focus
2023407	block4	Control	post	wei3	Verb	Broad	r2	206.5801986	3	1	broad_focus	3	Broad focus
2023407	block4	Control	post	tsam3	Object	Broad	r2	250.1119119	4	1	broad_focus	3	Broad focus
2023407	block4	Control	post	tsam3	Object	Broad	r2	495.9601427	5	2	broad_focus	3	Broad focus
2023407	block4	Control	post	pak3	Subject	Contrastive	r2	105.9041797	1	1	pre_focus	3	Contrastive pre_focus
2023407	block4	Control	post	pak3	Subject	Contrastive	r2	142.2622827	2	2	pre_focus	3	Contrastive pre_focus
2023407	block4	Control	post	tsing2	Verb	Contrastive	r2	226.9852932	3	1	on_focus	2	Contrastive on_focus
2023407	block4	Control	post	kau2	Object	Contrastive	r2	239.0758441	4	1	post_focus	2	Contrastive post_focus
2023407	block4	Control	post	tsi2	Object	Contrastive	r2	499.0776644	5	2	post_focus	2	Contrastive post_focus
2023407	block4	Control	post	piu35	Subject	Contrastive	r2	148.9542129	1	1	pre_focus	35	Contrastive pre_focus
2023407	block4	Control	post	mui35	Subject	Contrastive	r2	223.5762942	2	2	pre_focus	35	Contrastive pre_focus
2023407	block4	Control	post	tsan3	Verb	Contrastive	r2	289.571534	3	1	on_focus	3	Contrastive on_focus
2023407	block4	Control	post	jin3	Object	Contrastive	r2	262.0206151	4	1	post_focus	3	Contrastive post_focus
2023407	block4	Control	post	jin3	Object	Contrastive	r2	502.5664208	5	2	post_focus	3	Contrastive post_focus
2023407	block4	Control	post	piu35	Subject	Narrow	r2	169.2514515	1	1	on_focus	35	Narrow on_focus
2023407	block4	Control	post	mui35	Subject	Narrow	r2	264.4350266	2	2	on_focus	35	Narrow on_focus
2023407	block4	Control	post	tsan3	Verb	Narrow	r2	227.1086714	3	1	post_focus	3	Narrow post_focus
2023407	block4	Control	post	jin3	Object	Narrow	r2	306.1889258	4	1	post_focus	3	Narrow post_focus
2023407	block4	Control	post	jin3	Object	Narrow	r2	545.3114339	5	2	post_focus	3	Narrow post_focus
2023407	block4	Control	post	Jan-01	Subject	Narrow	r2	181.5394202	1	1	pre_focus	1	Narrow pre_focus
2023407	block4	Control	post	Jan-01	Subject	Narrow	r2	209.1935453	2	2	pre_focus	1	Narrow pre_focus
2023407	block4	Control	post	wei3	Verb	Narrow	r2	184.1335062	3	1	pre_focus	3	Narrow pre_focus
2023407	block4	Control	post	tsam3	Object	Narrow	r2	256.7946905	4	1	on_focus	3	Narrow on_focus
2023407	block4	Control	post	tsam3	Object	Narrow	r2	427.1036591	5	2	on_focus	3	Narrow on_focus
2023407	block4	Control	post	pak3	Subject	Narrow	r2	84.03102304	1	1	pre_focus	3	Narrow pre_focus
2023407	block4	Control	post	pak3	Subject	Narrow	r2	125.7385171	2	2	pre_focus	3	Narrow pre_focus
2023407	block4	Control	post	tsing2	Verb	Narrow	r2	205.3889957	3	1	on_focus	2	Narrow on_focus
2023407	block4	Control	post	kau2	Object	Narrow	r2	185.9854211	4	1	post_focus	2	Narrow post_focus
2023407	block4	Control	post	tsi2	Object	Narrow	r2	403.0219199	5	2	post_focus	2	Narrow post_focus
2023407	block4	Control	post	Jan-01	Subject	Narrow	r2	217.4753144	1	1	pre_focus	1	Narrow pre_focus
2023407	block4	Control	post	Jan-01	Subject	Narrow	r2	240.7224267	2	2	pre_focus	1	Narrow pre_focus
2023407	block4	Control	post	wei3	Verb	Narrow	r2	186.731124	3	1	on_focus	3	Narrow on_focus

2023407	block4	Control	post	tsam3	Object	Narrow	r2	224.7937224	4	1	post_focus	3	Narrow post_focus
2023407	block4	Control	post	tsam3	Object	Narrow	r2	476.9371272	5	2	post_focus	3	Narrow post_focus
2023407	block4	Control	post	piu35	Subject	Narrow	r2	174.7642585	1	1	pre_focus	35	Narrow pre_focus
2023407	block4	Control	post	mui35	Subject	Narrow	r2	227.6813704	2	2	pre_focus	35	Narrow pre_focus
2023407	block4	Control	post	tsan3	Verb	Narrow	r2	247.8903091	3	1	on_focus	3	Narrow on_focus
2023407	block4	Control	post	jin3	Object	Narrow	r2	265.7576659	4	1	post_focus	3	Narrow post_focus
2023407	block4	Control	post	jin3	Object	Narrow	r2	395.0117549	5	2	post_focus	3	Narrow post_focus
2023407	block4	Control	post	Jan-01	Subject	Narrow	r2	185.2760959	1	1	on_focus	1	Narrow on_focus
2023407	block4	Control	post	Jan-01	Subject	Narrow	r2	236.4677054	2	2	on_focus	1	Narrow on_focus
2023407	block4	Control	post	wei3	Verb	Narrow	r2	223.6573848	3	1	post_focus	3	Narrow post_focus
2023407	block4	Control	post	tsam3	Object	Narrow	r2	255.7026253	4	1	post_focus	3	Narrow post_focus
2023407	block4	Control	post	tsam3	Object	Narrow	r2	524.7750952	5	2	post_focus	3	Narrow post_focus
2023407	block4	Control	post	pak3	Subject	Contrastive	r2	116.8439195	1	1	on_focus	3	Contrastive on_focus
2023407	block4	Control	post	pak3	Subject	Contrastive	r2	133.1636368	2	2	on_focus	3	Contrastive on_focus
2023407	block4	Control	post	tsing2	Verb	Contrastive	r2	220.6948542	3	1	post_focus	2	Contrastive post_focus
2023407	block4	Control	post	kau2	Object	Contrastive	r2	206.4693788	4	1	post_focus	2	Contrastive post_focus
2023407	block4	Control	post	tsi2	Object	Contrastive	r2	372.1250215	5	2	post_focus	2	Contrastive post_focus
2023407	block4	Control	post	piu35	Subject	Broad	r2	172.5768386	1	1	broad_focus	35	Broad focus
2023407	block4	Control	post	mui35	Subject	Broad	r2	256.2163718	2	2	broad_focus	35	Broad focus
2023407	block4	Control	post	tsan3	Verb	Broad	r2	253.9388747	3	1	broad_focus	3	Broad focus
2023407	block4	Control	post	jin3	Object	Broad	r2	379.5838178	4	1	broad_focus	3	Broad focus
2023407	block4	Control	post	jin3	Object	Broad	r2	366.9295201	5	2	broad_focus	3	Broad focus
2023407	block4	Control	post	Jan-01	Subject	Contrastive	r2	187.6197297	1	1	pre_focus	1	Contrastive pre_focus
2023407	block4	Control	post	Jan-01	Subject	Contrastive	r2	234.0171294	2	2	pre_focus	1	Contrastive pre_focus
2023407	block4	Control	post	wei3	Verb	Contrastive	r2	189.5020886	3	1	pre_focus	3	Contrastive pre_focus
2023407	block4	Control	post	tsam3	Object	Contrastive	r2	222.5063561	4	1	on_focus	3	Contrastive on_focus
2023407	block4	Control	post	tsam3	Object	Contrastive	r2	507.3300174	5	2	on_focus	3	Contrastive on_focus
2023407	block4	Control	post	pak3	Subject	Contrastive	r2	121.9209151	1	1	pre_focus	3	Contrastive pre_focus
2023407	block4	Control	post	pak3	Subject	Contrastive	r2	161.8775901	2	2	pre_focus	3	Contrastive pre_focus
2023407	block4	Control	post	tsing2	Verb	Contrastive	r2	224.1441571	3	1	pre_focus	2	Contrastive pre_focus
2023407	block4	Control	post	kau2	Object	Contrastive	r2	262.9440612	4	1	on_focus	2	Contrastive on_focus
2023407	block4	Control	post	tsi2	Object	Contrastive	r2	471.5646865	5	2	on_focus	2	Contrastive on_focus
2023407	block4	Control	post	Jan-01	Subject	Contrastive	r2	137.0617101	1	1	pre_focus	1	Contrastive pre_focus
2023407	block4	Control	post	Jan-01	Subject	Contrastive	r2	266.5978492	2	2	pre_focus	1	Contrastive pre_focus
2023407	block4	Control	post	wei3	Verb	Contrastive	r2	374.6142361	3	1	on_focus	3	Contrastive on_focus
2023407	block4	Control	post	tsam3	Object	Contrastive	r2	294.3779353	4	1	post_focus	3	Contrastive post_focus
2023407	block4	Control	post	tsam3	Object	Contrastive	r2	657.0722067	5	2	post_focus	3	Contrastive post_focus
2023407	block4	Control	post	pak3	Subject	Narrow	r2	116.8134863	1	1	on_focus	3	Narrow on_focus
2023407	block4	Control	post	pak3	Subject	Narrow	r2	196.4382423	2	2	on_focus	3	Narrow on_focus
2023407	block4	Control	post	tsing2	Verb	Narrow	r2	215.8508203	3	1	post_focus	2	Narrow post_focus
2023407	block4	Control	post	kau2	Object	Narrow	r2	231.8562706	4	1	post_focus	2	Narrow post_focus
2023407	block4	Control	post	tsi2	Object	Narrow	r2	417.4419106	5	2	post_focus	2	Narrow post_focus
2023407	block4	Control	post	piu35	Subject	Contrastive	r2	157.2548332	1	1	on_focus	35	Contrastive on_focus
2023407	block4	Control	post	mui35	Subject	Contrastive	r2	299.3237187	2	2	on_focus	35	Contrastive on_focus
2023407	block4	Control	post	tsan3	Verb	Contrastive	r2	231.63533	3	1	post_focus	3	Contrastive post_focus
2023407	block4	Control	post	jin3	Object	Contrastive	r2	259.7761107	4	1	post_focus	3	Contrastive post_focus
2023407	block4	Control	post	jin3	Object	Contrastive	r2	377.128784	5	2	post_focus	3	Contrastive post_focus
2023407	block4	Control	post	piu35	Subject	Contrastive	r2	193.2035361	1	1	pre_focus	35	Contrastive pre_focus
2023407	block4	Control	post	mui35	Subject	Contrastive	r2	284.9675208	2	2	pre_focus	35	Contrastive pre_focus
2023407	block4	Control	post	tsan3	Verb	Contrastive	r2	215.4167586	3	1	pre_focus	3	Contrastive pre_focus
2023407	block4	Control	post	jin3	Object	Contrastive	r2	275.3350942	4	1	on_focus	3	Contrastive on_focus
2023407	block4	Control	post	jin3	Object	Contrastive	r2	307.260735	5	2	on_focus	3	Contrastive on_focus
2023407	block4	Control	post	Jan-01	Subject	Contrastive	r2	306.9020912	1	1	on_focus	1	Contrastive on_focus
2023407	block4	Control	post	Jan-01	Subject	Contrastive	r2	263.2703073	2	2	on_focus	1	Contrastive on_focus
2023407	block4	Control	post	wei3	Verb	Contrastive	r2	286.3722156	3	1	post_focus	3	Contrastive post_focus
2023407	block4	Control	post	tsam3	Object	Contrastive	r2	311.0787172	4	1	post_focus	3	Contrastive post_focus
2023407	block4	Control	post	tsam3	Object	Contrastive	r2	486.748875	5	2	post_focus	3	Contrastive post_focus
2023407	block4	Control	pre	piu35	Subject	Narrow	r1	126.7964594	1	1	on_focus	35	Narrow on_focus
2023407	block4	Control	pre	mui35	Subject	Narrow	r1	267.3537749	2	2	on_focus	35	Narrow on_focus
2023407	block4	Control	pre	tsan3	Verb	Narrow	r1	231.9687165	3	1	post_focus	3	Narrow post_focus
2023407	block4	Control	pre	jin3	Object	Narrow	r1	255.5587555	4	1	post_focus	3	Narrow post_focus
2023407	block4	Control	pre	jin3	Object	Narrow	r1	427.5694562	5	2	post_focus	3	Narrow post_focus
2023407	block4	Control	pre	Jan-01	Subject	Contrastive	r1	212.3103507	1	1	on_focus	1	Contrastive on_focus
2023407	block4	Control	pre	Jan-01	Subject	Contrastive	r1	397.0989892	2	2	on_focus	1	Contrastive on_focus
2023407	block4	Control	pre	wei3	Verb	Contrastive	r1	255.5587555	3	1	post_focus	3	Contrastive post_focus
2023407	block4	Control	pre	tsam3	Object	Contrastive	r1	216.2420238	4	1	post_focus	3	Contrastive post_focus

2023407	block4	Control	pre	tsam3	Object	Contrastive	r1	290.9438139	5	2	post_focus	3	Contrastive post_focus
2023407	block4	Control	pre	pak3	Subject	Contrastive	r1	102.2235022	1	1	on_focus	3	Contrastive on_focus
2023407	block4	Control	pre	pak3	Subject	Contrastive	r1	86.49680954	2	2	on_focus	3	Contrastive on_focus
2023407	block4	Control	pre	tsing2	Verb	Contrastive	r1	176.9252922	3	1	post_focus	2	Contrastive post_focus
2023407	block4	Control	pre	kau2	Object	Contrastive	r1	228.0370433	4	1	post_focus	2	Contrastive post_focus
2023407	block4	Control	pre	tsi2	Object	Contrastive	r1	306.6705065	5	2	post_focus	2	Contrastive post_focus
2023407	block4	Control	pre	piu35	Subject	Contrastive	r1	147.4377435	1	1	pre_focus	35	Contrastive pre_focus
2023407	block4	Control	pre	mui35	Subject	Contrastive	r1	276.06462	2	2	pre_focus	35	Contrastive pre_focus
2023407	block4	Control	pre	tsan3	Verb	Contrastive	r1	288.9779773	3	1	on_focus	3	Contrastive on_focus
2023407	block4	Control	pre	jin3	Object	Contrastive	r1	270.9294085	4	1	post_focus	3	Contrastive post_focus
2023407	block4	Control	pre	jin3	Object	Contrastive	r1	281.2500506	5	2	post_focus	3	Contrastive post_focus
2023407	block4	Control	pre	pak3	Subject	Contrastive	r1	110.0868485	1	1	pre_focus	3	Contrastive pre_focus
2023407	block4	Control	pre	pak3	Subject	Contrastive	r1	114.0185217	2	2	pre_focus	3	Contrastive pre_focus
2023407	block4	Control	pre	tsing2	Verb	Contrastive	r1	196.583658	3	1	on_focus	2	Contrastive on_focus
2023407	block4	Control	pre	kau2	Object	Contrastive	r1	212.3103507	4	1	post_focus	2	Contrastive post_focus
2023407	block4	Control	pre	tsi2	Object	Contrastive	r1	432.4840477	5	2	post_focus	2	Contrastive post_focus
2023407	block4	Control	pre	Jan-01	Subject	Narrow	r1	200.5153312	1	1	on_focus	1	Narrow on_focus
2023407	block4	Control	pre	Jan-01	Subject	Narrow	r1	239.8320628	2	2	on_focus	1	Narrow on_focus
2023407	block4	Control	pre	wei3	Verb	Narrow	r1	184.7886386	3	1	post_focus	3	Narrow post_focus
2023407	block4	Control	pre	tsam3	Object	Narrow	r1	231.9687165	4	1	post_focus	3	Narrow post_focus
2023407	block4	Control	pre	tsam3	Object	Narrow	r1	338.1238918	5	2	post_focus	3	Narrow post_focus
2023407	block4	Control	pre	pak3	Subject	Narrow	r1	106.1551753	1	1	on_focus	3	Narrow on_focus
2023407	block4	Control	pre	pak3	Subject	Narrow	r1	121.881868	2	2	on_focus	3	Narrow on_focus
2023407	block4	Control	pre	tsing2	Verb	Narrow	r1	226.0712067	3	1	post_focus	2	Narrow post_focus
2023407	block4	Control	pre	kau2	Object	Narrow	r1	249.6612457	4	1	post_focus	2	Narrow post_focus
2023407	block4	Control	pre	tsi2	Object	Narrow	r1	418.7231916	5	2	post_focus	2	Narrow post_focus
2023407	block4	Control	pre	piu35	Subject	Contrastive	r1	169.0619459	1	1	pre_focus	35	Contrastive pre_focus
2023407	block4	Control	pre	mui35	Subject	Contrastive	r1	204.4470044	2	2	pre_focus	35	Contrastive pre_focus
2023407	block4	Control	pre	tsan3	Verb	Contrastive	r1	216.2420238	3	1	pre_focus	3	Contrastive pre_focus
2023407	block4	Control	pre	jin3	Object	Contrastive	r1	303.5349823	4	1	on_focus	3	Contrastive on_focus
2023407	block4	Control	pre	jin3	Object	Contrastive	r1	483.5957988	5	2	on_focus	3	Contrastive on_focus
2023407	block4	Control	pre	pak3	Subject	Broad	r1	94.36015586	1	1	broad_focus	3	Broad focus
2023407	block4	Control	pre	pak3	Subject	Broad	r1	121.881868	2	2	broad_focus	3	Broad focus
2023407	block4	Control	pre	tsing2	Verb	Broad	r1	184.7886386	3	1	broad_focus	2	Broad focus
2023407	block4	Control	pre	kau2	Object	Broad	r1	251.6270823	4	1	broad_focus	2	Broad focus
2023407	block4	Control	pre	tsi2	Object	Broad	r1	412.8256819	5	2	broad_focus	2	Broad focus
2023407	block4	Control	pre	Jan-01	Subject	Narrow	r1	172.9936191	1	1	pre_focus	1	Narrow pre_focus
2023407	block4	Control	pre	Jan-01	Subject	Narrow	r1	259.4904286	2	2	pre_focus	1	Narrow pre_focus
2023407	block4	Control	pre	wei3	Verb	Narrow	r1	224.1053702	3	1	pre_focus	3	Narrow pre_focus
2023407	block4	Control	pre	tsam3	Object	Narrow	r1	208.5741008	4	1	on_focus	3	Narrow on_focus
2023407	block4	Control	pre	tsam3	Object	Narrow	r1	328.2947089	5	2	on_focus	3	Narrow on_focus
2023407	block4	Control	pre	piu35	Subject	Narrow	r1	169.0619459	1	1	pre_focus	35	Narrow pre_focus
2023407	block4	Control	pre	mui35	Subject	Narrow	r1	267.3537749	2	2	pre_focus	35	Narrow pre_focus
2023407	block4	Control	pre	tsan3	Verb	Narrow	r1	314.5338529	3	1	pre_focus	3	Narrow pre_focus
2023407	block4	Control	pre	jin3	Object	Narrow	r1	271.2854481	4	1	on_focus	3	Narrow on_focus
2023407	block4	Control	pre	jin3	Object	Narrow	r1	456.0740867	5	2	on_focus	3	Narrow on_focus
2023407	block4	Control	pre	Jan-01	Subject	Contrastive	r1	275.2171213	1	1	pre_focus	1	Contrastive pre_focus
2023407	block4	Control	pre	Jan-01	Subject	Contrastive	r1	224.1053702	2	2	pre_focus	1	Contrastive pre_focus
2023407	block4	Control	pre	wei3	Verb	Contrastive	r1	192.6519849	3	1	pre_focus	3	Contrastive pre_focus
2023407	block4	Control	pre	tsam3	Object	Contrastive	r1	161.1985996	4	1	on_focus	3	Contrastive on_focus
2023407	block4	Control	pre	tsam3	Object	Contrastive	r1	491.4591451	5	2	on_focus	3	Contrastive on_focus
2023407	block4	Control	pre	Jan-01	Subject	Contrastive	r1	239.8320628	1	1	pre_focus	1	Contrastive pre_focus
2023407	block4	Control	pre	Jan-01	Subject	Contrastive	r1	357.7822576	2	2	pre_focus	1	Contrastive pre_focus
2023407	block4	Control	pre	wei3	Verb	Contrastive	r1	456.0740867	3	1	on_focus	3	Contrastive on_focus
2023407	block4	Control	pre	tsam3	Object	Contrastive	r1	165.1302728	4	1	post_focus	3	Contrastive post_focus
2023407	block4	Control	pre	tsam3	Object	Contrastive	r1	287.0121407	5	2	post_focus	3	Contrastive post_focus
2023407	block4	Control	pre	piu35	Subject	Narrow	r1	153.3352533	1	1	pre_focus	35	Narrow pre_focus
2023407	block4	Control	pre	mui35	Subject	Narrow	r1	239.8320628	2	2	pre_focus	35	Narrow pre_focus
2023407	block4	Control	pre	tsan3	Verb	Narrow	r1	322.3971992	3	1	on_focus	3	Narrow on_focus
2023407	block4	Control	pre	jin3	Object	Narrow	r1	238.8491445	4	1	post_focus	3	Narrow post_focus
2023407	block4	Control	pre	jin3	Object	Narrow	r1	339.2681199	5	2	post_focus	3	Narrow post_focus
2023407	block4	Control	pre	piu35	Subject	Contrastive	r1	141.5402338	1	1	on_focus	35	Contrastive on_focus
2023407	block4	Control	pre	mui35	Subject	Contrastive	r1	314.5338529	2	2	on_focus	35	Contrastive on_focus
2023407	block4	Control	pre	tsan3	Verb	Contrastive	r1	267.3537749	3	1	post_focus	3	Contrastive post_focus
2023407	block4	Control	pre	jin3	Object	Contrastive	r1	298.8071602	4	1	post_focus	3	Contrastive post_focus
2023407	block4	Control	pre	jin3	Object	Contrastive	r1	326.3288723	5	2	post_focus	3	Contrastive post_focus

2023407	block4	Control	pre	pak3	Subject	Narrow	r1	117.9501948	1	1	pre_focus	3	Narrow pre_focus
2023407	block4	Control	pre	pak3	Subject	Narrow	r1	196.583658	2	2	pre_focus	3	Narrow pre_focus
2023407	block4	Control	pre	tsing2	Verb	Narrow	r1	279.1487944	3	1	on_focus	2	Narrow on_focus
2023407	block4	Control	pre	kau2	Object	Narrow	r1	259.4904286	4	1	post_focus	2	Narrow post_focus
2023407	block4	Control	pre	tsi2	Object	Narrow	r1	373.5089503	5	2	post_focus	2	Narrow post_focus
2023407	block4	Control	pre	piu35	Subject	Broad	r1	157.2669264	1	1	broad_focus	35	Broad focus
2023407	block4	Control	pre	mui35	Subject	Broad	r1	239.8320628	2	2	broad_focus	35	Broad focus
2023407	block4	Control	pre	tsan3	Verb	Broad	r1	171.0277825	3	1	broad_focus	3	Broad focus
2023407	block4	Control	pre	jin3	Object	Broad	r1	260.694066	4	1	broad_focus	3	Broad focus
2023407	block4	Control	pre	jin3	Object	Broad	r1	240.5776424	5	2	broad_focus	3	Broad focus
2023407	block4	Control	pre	Jan-01	Subject	Broad	r1	188.7203117	1	1	broad_focus	1	Broad focus
2023407	block4	Control	pre	Jan-01	Subject	Broad	r1	377.4406234	2	2	broad_focus	1	Broad focus
2023407	block4	Control	pre	wei3	Verb	Broad	r1	306.6705065	3	1	broad_focus	3	Broad focus
2023407	block4	Control	pre	tsam3	Object	Broad	r1	180.8569654	4	1	broad_focus	3	Broad focus
2023407	block4	Control	pre	tsam3	Object	Broad	r1	361.7139308	5	2	broad_focus	3	Broad focus
2023407	block4	Control	pre	pak3	Subject	Contrastive	r1	94.36015586	1	1	pre_focus	3	Contrastive pre_focus
2023407	block4	Control	pre	pak3	Subject	Contrastive	r1	149.4035801	2	2	pre_focus	3	Contrastive pre_focus
2023407	block4	Control	pre	tsing2	Verb	Contrastive	r1	241.7978994	3	1	pre_focus	2	Contrastive pre_focus
2023407	block4	Control	pre	kau2	Object	Contrastive	r1	245.7295726	4	1	on_focus	2	Contrastive on_focus
2023407	block4	Control	pre	tsi2	Object	Contrastive	r1	357.7822576	5	2	on_focus	2	Contrastive on_focus
2023407	block4	Control	pre	Jan-01	Subject	Narrow	r1	263.4221018	1	1	pre_focus	1	Narrow pre_focus
2023407	block4	Control	pre	Jan-01	Subject	Narrow	r1	322.3971992	2	2	pre_focus	1	Narrow pre_focus
2023407	block4	Control	pre	wei3	Verb	Narrow	r1	204.4470044	3	1	on_focus	3	Narrow on_focus
2023407	block4	Control	pre	tsam3	Object	Narrow	r1	235.9003897	4	1	post_focus	3	Narrow post_focus
2023407	block4	Control	pre	tsam3	Object	Narrow	r1	428.5523745	5	2	post_focus	3	Narrow post_focus
2023407	block4	Control	pre	pak3	Subject	Narrow	r1	80.5992998	1	1	pre_focus	3	Narrow pre_focus
2023407	block4	Control	pre	pak3	Subject	Narrow	r1	96.32599244	2	2	pre_focus	3	Narrow pre_focus
2023407	block4	Control	pre	tsing2	Verb	Narrow	r1	250.644164	3	1	pre_focus	2	Narrow pre_focus
2023407	block4	Control	pre	kau2	Object	Narrow	r1	257.524592	4	1	on_focus	2	Narrow on_focus
2023407	block4	Control	pre	tsi2	Object	Narrow	r1	401.0306624	5	2	on_focus	2	Narrow on_focus
2023407	block4	Control	pre	Jan-01	Subject	Narrow	r2	263.4221018	1	1	pre_focus	1	Narrow pre_focus
2023407	block4	Control	pre	Jan-01	Subject	Narrow	r2	310.6021797	2	2	pre_focus	1	Narrow pre_focus
2023407	block4	Control	pre	wei3	Verb	Narrow	r2	180.8569654	3	1	pre_focus	3	Narrow pre_focus
2023407	block4	Control	pre	tsam3	Object	Narrow	r2	243.763736	4	1	on_focus	3	Narrow on_focus
2023407	block4	Control	pre	tsam3	Object	Narrow	r2	448.2107403	5	2	on_focus	3	Narrow on_focus
2023407	block4	Control	pre	Jan-01	Subject	Contrastive	r2	255.5587555	1	1	on_focus	1	Contrastive on_focus
2023407	block4	Control	pre	Jan-01	Subject	Contrastive	r2	228.0370433	2	2	on_focus	1	Contrastive on_focus
2023407	block4	Control	pre	wei3	Verb	Contrastive	r2	176.9252922	3	1	post_focus	3	Contrastive post_focus
2023407	block4	Control	pre	tsam3	Object	Contrastive	r2	196.583658	4	1	post_focus	3	Contrastive post_focus
2023407	block4	Control	pre	tsam3	Object	Contrastive	r2	334.1922187	5	2	post_focus	3	Contrastive post_focus
2023407	block4	Control	pre	Jan-01	Subject	Narrow	r2	235.3919992	1	1	on_focus	1	Narrow on_focus
2023407	block4	Control	pre	Jan-01	Subject	Narrow	r2	231.0696259	2	2	on_focus	1	Narrow on_focus
2023407	block4	Control	pre	wei3	Verb	Narrow	r2	165.1302728	3	1	post_focus	3	Narrow post_focus
2023407	block4	Control	pre	tsam3	Object	Narrow	r2	204.4470044	4	1	post_focus	3	Narrow post_focus
2023407	block4	Control	pre	tsam3	Object	Narrow	r2	449.8050631	5	2	post_focus	3	Narrow post_focus
2023407	block4	Control	pre	piu35	Subject	Narrow	r2	179.1103567	1	1	pre_focus	35	Narrow pre_focus
2023407	block4	Control	pre	mui35	Subject	Narrow	r2	306.9162361	2	2	pre_focus	35	Narrow pre_focus
2023407	block4	Control	pre	tsan3	Verb	Narrow	r2	236.3918488	3	1	pre_focus	3	Narrow pre_focus
2023407	block4	Control	pre	jin3	Object	Narrow	r2	362.2053899	4	1	on_focus	3	Narrow on_focus
2023407	block4	Control	pre	jin3	Object	Narrow	r2	546.0267111	5	2	on_focus	3	Narrow on_focus
2023407	block4	Control	pre	piu35	Subject	Broad	r2	164.1473545	1	1	broad_focus	35	Broad focus
2023407	block4	Control	pre	mui35	Subject	Broad	r2	335.2016386	2	2	broad_focus	35	Broad focus
2023407	block4	Control	pre	tsan3	Verb	Broad	r2	290.9438139	3	1	broad_focus	3	Broad focus
2023407	block4	Control	pre	jin3	Object	Broad	r2	287.0121407	4	1	broad_focus	3	Broad focus
2023407	block4	Control	pre	jin3	Object	Broad	r2	463.937433	5	2	broad_focus	3	Broad focus
2023407	block4	Control	pre	Jan-01	Subject	Broad	r2	348.935993	1	1	broad_focus	1	Broad focus
2023407	block4	Control	pre	Jan-01	Subject	Broad	r2	381.3722966	2	2	broad_focus	1	Broad focus
2023407	block4	Control	pre	wei3	Verb	Broad	r2	213.293269	3	1	broad_focus	3	Broad focus
2023407	block4	Control	pre	tsam3	Object	Broad	r2	265.3879384	4	1	broad_focus	3	Broad focus
2023407	block4	Control	pre	tsam3	Object	Broad	r2	471.8007793	5	2	broad_focus	3	Broad focus
2023407	block4	Control	pre	Jan-01	Subject	Contrastive	r2	298.8071602	1	1	pre_focus	1	Contrastive pre_focus
2023407	block4	Control	pre	Jan-01	Subject	Contrastive	r2	365.645604	2	2	pre_focus	1	Contrastive pre_focus
2023407	block4	Control	pre	wei3	Verb	Contrastive	r2	208.3786775	3	1	on_focus	3	Contrastive on_focus
2023407	block4	Control	pre	tsam3	Object	Contrastive	r2	279.1487944	4	1	post_focus	3	Contrastive post_focus
2023407	block4	Control	pre	tsam3	Object	Contrastive	r2	345.9872382	5	2	post_focus	3	Contrastive post_focus
2023407	block4	Control	pre	piu35	Subject	Contrastive	r2	137.6085606	1	1	pre_focus	35	Contrastive pre_focus

2023407	block4	Control	pre	mui35	Subject	Contrastive	r2	359.7480942	2	2	pre_focus	35	Contrastive pre_focus
2023407	block4	Control	pre	tsan3	Verb	Contrastive	r2	341.0726467	3	1	on_focus	3	Contrastive on_focus
2023407	block4	Control	pre	jin3	Object	Contrastive	r2	297.2475823	4	1	post_focus	3	Contrastive post_focus
2023407	block4	Control	pre	jin3	Object	Contrastive	r2	373.5089503	5	2	post_focus	3	Contrastive post_focus
2023407	block4	Control	pre	pak3	Subject	Contrastive	r2	70.7701169	1	1	pre_focus	3	Contrastive pre_focus
2023407	block4	Control	pre	pak3	Subject	Contrastive	r2	98.29182902	2	2	pre_focus	3	Contrastive pre_focus
2023407	block4	Control	pre	tsing2	Verb	Contrastive	r2	212.3103507	3	1	pre_focus	2	Contrastive pre_focus
2023407	block4	Control	pre	kau2	Object	Contrastive	r2	271.2854481	4	1	on_focus	2	Contrastive on_focus
2023407	block4	Control	pre	tsi2	Object	Contrastive	r2	369.5772771	5	2	on_focus	2	Contrastive on_focus
2023407	block4	Control	pre	piu35	Subject	Contrastive	r2	212.8868064	1	1	pre_focus	35	Contrastive pre_focus
2023407	block4	Control	pre	mui35	Subject	Contrastive	r2	331.9806525	2	2	pre_focus	35	Contrastive pre_focus
2023407	block4	Control	pre	tsan3	Verb	Contrastive	r2	225.5797476	3	1	pre_focus	3	Contrastive pre_focus
2023407	block4	Control	pre	jin3	Object	Contrastive	r2	288.4865182	4	1	on_focus	3	Contrastive on_focus
2023407	block4	Control	pre	jin3	Object	Contrastive	r2	232.4601756	5	2	on_focus	3	Contrastive on_focus
2023407	block4	Control	pre	pak3	Subject	Contrastive	r2	106.1551753	1	1	on_focus	3	Contrastive on_focus
2023407	block4	Control	pre	pak3	Subject	Contrastive	r2	129.7452143	2	2	on_focus	3	Contrastive on_focus
2023407	block4	Control	pre	tsing2	Verb	Contrastive	r2	180.8569654	3	1	post_focus	2	Contrastive post_focus
2023407	block4	Control	pre	kau2	Object	Contrastive	r2	235.9003897	4	1	post_focus	2	Contrastive post_focus
2023407	block4	Control	pre	tsi2	Object	Contrastive	r2	404.9623356	5	2	post_focus	2	Contrastive post_focus
2023407	block4	Control	pre	Jan-01	Subject	Contrastive	r2	220.173697	1	1	pre_focus	1	Contrastive pre_focus
2023407	block4	Control	pre	Jan-01	Subject	Contrastive	r2	271.2854481	2	2	pre_focus	1	Contrastive pre_focus
2023407	block4	Control	pre	wei3	Verb	Contrastive	r2	184.7886386	3	1	pre_focus	3	Contrastive pre_focus
2023407	block4	Control	pre	tsam3	Object	Contrastive	r2	259.4904286	4	1	on_focus	3	Contrastive on_focus
2023407	block4	Control	pre	tsam3	Object	Contrastive	r2	520.9466938	5	2	on_focus	3	Contrastive on_focus
2023407	block4	Control	pre	pak3	Subject	Broad	r2	90.4284827	1	1	broad_focus	3	Broad focus
2023407	block4	Control	pre	pak3	Subject	Broad	r2	114.0185217	2	2	broad_focus	3	Broad focus
2023407	block4	Control	pre	tsing2	Verb	Broad	r2	188.7203117	3	1	broad_focus	2	Broad focus
2023407	block4	Control	pre	kau2	Object	Broad	r2	239.8320628	4	1	broad_focus	2	Broad focus
2023407	block4	Control	pre	tsi2	Object	Broad	r2	475.7324525	5	2	broad_focus	2	Broad focus
2023407	block4	Control	pre	piu35	Subject	Contrastive	r2	127.7915312	1	1	on_focus	35	Contrastive on_focus
2023407	block4	Control	pre	mui35	Subject	Contrastive	r2	337.0181088	2	2	on_focus	35	Contrastive on_focus
2023407	block4	Control	pre	tsan3	Verb	Contrastive	r2	263.7005304	3	1	post_focus	3	Contrastive post_focus
2023407	block4	Control	pre	jin3	Object	Contrastive	r2	274.234203	4	1	post_focus	3	Contrastive post_focus
2023407	block4	Control	pre	jin3	Object	Contrastive	r2	271.1758975	5	2	post_focus	3	Contrastive post_focus
2023407	block4	Control	pre	piu35	Subject	Narrow	r2	143.5060704	1	1	pre_focus	35	Narrow pre_focus
2023407	block4	Control	pre	mui35	Subject	Narrow	r2	338.1238918	2	2	pre_focus	35	Narrow pre_focus
2023407	block4	Control	pre	tsan3	Verb	Narrow	r2	254.9574291	3	1	on_focus	3	Narrow on_focus
2023407	block4	Control	pre	jin3	Object	Narrow	r2	287.8853818	4	1	post_focus	3	Narrow post_focus
2023407	block4	Control	pre	jin3	Object	Narrow	r2	416.0201663	5	2	post_focus	3	Narrow post_focus
2023407	block4	Control	pre	pak3	Subject	Narrow	r2	94.36015586	1	1	pre_focus	3	Narrow pre_focus
2023407	block4	Control	pre	pak3	Subject	Narrow	r2	129.7452143	2	2	pre_focus	3	Narrow pre_focus
2023407	block4	Control	pre	tsing2	Verb	Narrow	r2	216.2420238	3	1	on_focus	2	Narrow on_focus
2023407	block4	Control	pre	kau2	Object	Narrow	r2	235.9003897	4	1	post_focus	2	Narrow post_focus
2023407	block4	Control	pre	tsi2	Object	Narrow	r2	393.1673161	5	2	post_focus	2	Narrow post_focus
2023407	block4	Control	pre	pak3	Subject	Narrow	r2	82.56513638	1	1	pre_focus	3	Narrow pre_focus
2023407	block4	Control	pre	pak3	Subject	Narrow	r2	110.0868485	2	2	pre_focus	3	Narrow pre_focus
2023407	block4	Control	pre	tsing2	Verb	Narrow	r2	186.7544751	3	1	pre_focus	2	Narrow pre_focus
2023407	block4	Control	pre	kau2	Object	Narrow	r2	176.9252922	4	1	on_focus	2	Narrow on_focus
2023407	block4	Control	pre	tsi2	Object	Narrow	r2	312.5680163	5	2	on_focus	2	Narrow on_focus
2023407	block4	Control	pre	pak3	Subject	Narrow	r2	90.4284827	1	1	on_focus	3	Narrow on_focus
2023407	block4	Control	pre	pak3	Subject	Narrow	r2	117.9501948	2	2	on_focus	3	Narrow on_focus
2023407	block4	Control	pre	tsing2	Verb	Narrow	r2	165.1302728	3	1	post_focus	2	Narrow post_focus
2023407	block4	Control	pre	kau2	Object	Narrow	r2	212.3103507	4	1	post_focus	2	Narrow post_focus
2023407	block4	Control	pre	tsi2	Object	Narrow	r2	377.4406234	5	2	post_focus	2	Narrow post_focus
2023407	block4	Control	pre	piu35	Subject	Narrow	r2	180.8569654	1	1	on_focus	35	Narrow on_focus
2023407	block4	Control	pre	mui35	Subject	Narrow	r2	298.8071602	2	2	on_focus	35	Narrow on_focus
2023407	block4	Control	pre	tsan3	Verb	Narrow	r2	231.1546726	3	1	post_focus	3	Narrow post_focus
2023407	block4	Control	pre	jin3	Object	Narrow	r2	298.3157011	4	1	post_focus	3	Narrow post_focus
2023407	block4	Control	pre	jin3	Object	Narrow	r2	371.5431137	5	2	post_focus	3	Narrow post_focus
2023407	block4	Control	pre	pak3	Subject	Contrastive	r2	70.7701169	1	1	pre_focus	3	Contrastive pre_focus
2023407	block4	Control	pre	pak3	Subject	Contrastive	r2	114.0185217	2	2	pre_focus	3	Contrastive pre_focus
2023407	block4	Control	pre	tsing2	Verb	Contrastive	r2	188.7203117	3	1	on_focus	2	Contrastive on_focus
2023407	block4	Control	pre	kau2	Object	Contrastive	r2	208.3786775	4	1	post_focus	2	Contrastive post_focus
2023407	block4	Control	pre	tsi2	Object	Contrastive	r2	428.5523745	5	2	post_focus	2	Contrastive post_focus
2023407	block4	Control	pre	Jan-01	Subject	Narrow	r2	149.9801	1	1	pre_focus	1	Narrow pre_focus
2023407	block4	Control	pre	Jan-01	Subject	Narrow	r2	235.9003897	2	2	pre_focus	1	Narrow pre_focus

2023407	block4	Control	pre	wei3	Verb	Narrow	r2	230.0028799	3	1	on_focus	3	Narrow on_focus
2023407	block4	Control	pre	tsam3	Object	Narrow	r2	261.4562652	4	1	post_focus	3	Narrow post_focus
2023407	block4	Control	pre	tsam3	Object	Narrow	r2	401.0306624	5	2	post_focus	3	Narrow post_focus
2023407	block5	Control	post	wai5	Subject	Contrastive	r1	290.2147639	1	1	pre_focus	5	Contrastive pre_focus
2023407	block5	Control	post	wai5	Subject	Contrastive	r1	368.6986493	2	2	pre_focus	5	Contrastive pre_focus
2023407	block5	Control	post	waat3	Verb	Contrastive	r1	227.7880377	3	1	pre_focus	3	Contrastive pre_focus
2023407	block5	Control	post	bui3	Object	Contrastive	r1	211.4850946	4	1	on_focus	3	Contrastive on_focus
2023407	block5	Control	post	hok3	Object	Contrastive	r1	322.0914881	5	2	on_focus	3	Contrastive on_focus
2023407	block5	Control	post	ceoi3	Subject	Contrastive	r1	172.8083215	1	1	pre_focus	3	Contrastive pre_focus
2023407	block5	Control	post	ceoi3	Subject	Contrastive	r1	338.3812073	2	2	pre_focus	3	Contrastive pre_focus
2023407	block5	Control	post	caa4	Verb	Contrastive	r1	275.3703704	3	1	pre_focus	4	Contrastive pre_focus
2023407	block5	Control	post	ngau4	Object	Contrastive	r1	164.7855108	4	1	on_focus	4	Contrastive on_focus
2023407	block5	Control	post	jau4	Object	Contrastive	r1	159.0867013	5	2	on_focus	4	Contrastive on_focus
2023407	block5	Control	post	siu2	Subject	Narrow	r1	444.4825155	1	1	pre_focus	2	Narrow pre_focus
2023407	block5	Control	post	gwong2	Subject	Narrow	r1	574.2236798	2	2	pre_focus	2	Narrow pre_focus
2023407	block5	Control	post	cyun4	Verb	Narrow	r1	523.6184415	3	1	pre_focus	4	Narrow pre_focus
2023407	block5	Control	post	laam4	Object	Narrow	r1	499.2793505	4	1	on_focus	4	Narrow on_focus
2023407	block5	Control	post	kau4	Object	Narrow	r1	488.0016865	5	2	on_focus	4	Narrow on_focus
2023407	block5	Control	post	ceoi3	Subject	Narrow	r1	227.23523	1	1	pre_focus	3	Narrow pre_focus
2023407	block5	Control	post	ceoi3	Subject	Narrow	r1	323.735126	2	2	pre_focus	3	Narrow pre_focus
2023407	block5	Control	post	caa4	Verb	Narrow	r1	193.0157014	3	1	pre_focus	4	Narrow pre_focus
2023407	block5	Control	post	ngau4	Object	Narrow	r1	226.1367902	4	1	on_focus	4	Narrow on_focus
2023407	block5	Control	post	jau4	Object	Narrow	r1	417.7545376	5	2	on_focus	4	Narrow on_focus
2023407	block5	Control	post	siu2	Subject	Contrastive	r1	177.5174705	1	1	on_focus	2	Contrastive on_focus
2023407	block5	Control	post	gwong2	Subject	Contrastive	r1	349.9845589	2	2	on_focus	2	Contrastive on_focus
2023407	block5	Control	post	cyun4	Verb	Contrastive	r1	290.8097343	3	1	post_focus	4	Contrastive post_focus
2023407	block5	Control	post	laam4	Object	Contrastive	r1	358.1238875	4	1	post_focus	4	Contrastive post_focus
2023407	block5	Control	post	kau4	Object	Contrastive	r1	451.1650232	5	2	post_focus	4	Contrastive post_focus
2023407	block5	Control	post	wai5	Subject	Contrastive	r1	208.0949368	1	1	on_focus	5	Contrastive on_focus
2023407	block5	Control	post	wai5	Subject	Contrastive	r1	330.5061381	2	2	on_focus	5	Contrastive on_focus
2023407	block5	Control	post	waat3	Verb	Contrastive	r1	181.5338058	3	1	post_focus	3	Contrastive post_focus
2023407	block5	Control	post	bui3	Object	Contrastive	r1	153.0675813	4	1	post_focus	3	Contrastive post_focus
2023407	block5	Control	post	hok3	Object	Contrastive	r1	247.526798	5	2	post_focus	3	Contrastive post_focus
2023407	block5	Control	post	ceoi3	Subject	Broad	r1	193.0020047	1	1	broad_focus	3	Broad focus
2023407	block5	Control	post	ceoi3	Subject	Broad	r1	428.4773333	2	2	broad_focus	3	Broad focus
2023407	block5	Control	post	caa4	Verb	Broad	r1	322.5550696	3	1	broad_focus	4	Broad focus
2023407	block5	Control	post	ngau4	Object	Broad	r1	295.4756074	4	1	broad_focus	4	Broad focus
2023407	block5	Control	post	jau4	Object	Broad	r1	333.5248173	5	2	broad_focus	4	Broad focus
2023407	block5	Control	post	wai5	Subject	Broad	r1	207.8858676	1	1	broad_focus	5	Broad focus
2023407	block5	Control	post	wai5	Subject	Broad	r1	413.2689758	2	2	broad_focus	5	Broad focus
2023407	block5	Control	post	waat3	Verb	Broad	r1	212.2852891	3	1	broad_focus	3	Broad focus
2023407	block5	Control	post	bui3	Object	Broad	r1	225.4750943	4	1	broad_focus	3	Broad focus
2023407	block5	Control	post	hok3	Object	Broad	r1	353.7746744	5	2	broad_focus	3	Broad focus
2023407	block5	Control	post	wai5	Subject	Narrow	r1	266.934173	1	1	on_focus	5	Narrow on_focus
2023407	block5	Control	post	wai5	Subject	Narrow	r1	416.5053922	2	2	on_focus	5	Narrow on_focus
2023407	block5	Control	post	waat3	Verb	Narrow	r1	177.4280166	3	1	post_focus	3	Narrow post_focus
2023407	block5	Control	post	bui3	Object	Narrow	r1	181.1403153	4	1	post_focus	3	Narrow post_focus
2023407	block5	Control	post	hok3	Object	Narrow	r1	369.1018071	5	2	post_focus	3	Narrow post_focus
2023407	block5	Control	post	siu2	Subject	Narrow	r1	239.6871565	1	1	pre_focus	2	Narrow pre_focus
2023407	block5	Control	post	gwong2	Subject	Narrow	r1	456.2602138	2	2	pre_focus	2	Narrow pre_focus
2023407	block5	Control	post	cyun4	Verb	Narrow	r1	415.4408255	3	1	on_focus	4	Narrow on_focus
2023407	block5	Control	post	laam4	Object	Narrow	r1	315.0144035	4	1	post_focus	4	Narrow post_focus
2023407	block5	Control	post	kau4	Object	Narrow	r1	254.6663996	5	2	post_focus	4	Narrow post_focus
2023407	block5	Control	post	siu2	Subject	Contrastive	r1	212.675737	1	1	pre_focus	2	Contrastive pre_focus
2023407	block5	Control	post	gwong2	Subject	Contrastive	r1	404.650984	2	2	pre_focus	2	Contrastive pre_focus
2023407	block5	Control	post	cyun4	Verb	Contrastive	r1	168.6598983	3	1	pre_focus	4	Contrastive pre_focus
2023407	block5	Control	post	laam4	Object	Contrastive	r1	260.8340757	4	1	on_focus	4	Contrastive on_focus
2023407	block5	Control	post	kau4	Object	Contrastive	r1	347.0151037	5	2	on_focus	4	Contrastive on_focus
2023407	block5	Control	post	siu2	Subject	Narrow	r1	123.5901679	1	1	on_focus	2	Narrow on_focus
2023407	block5	Control	post	gwong2	Subject	Narrow	r1	394.6150312	2	2	on_focus	2	Narrow on_focus
2023407	block5	Control	post	cyun4	Verb	Narrow	r1	174.5646259	3	1	post_focus	4	Narrow post_focus
2023407	block5	Control	post	laam4	Object	Narrow	r1	222.0358689	4	1	post_focus	4	Narrow post_focus
2023407	block5	Control	post	kau4	Object	Narrow	r1	144.4947189	5	2	post_focus	4	Narrow post_focus
2023407	block5	Control	post	wai5	Subject	Contrastive	r1	172.2215923	1	1	pre_focus	5	Contrastive pre_focus
2023407	block5	Control	post	wai5	Subject	Contrastive	r1	261.9980722	2	2	pre_focus	5	Contrastive pre_focus
2023407	block5	Control	post	waat3	Verb	Contrastive	r1	144.5074333	3	1	on_focus	3	Contrastive on_focus

2023407	block5	Control	post	bui3	Object	Contrastive	r1	145.0280502	4	1	post_focus	3	Contrastive post_focus
2023407	block5	Control	post	hok3	Object	Contrastive	r1	108.7355915	5	2	post_focus	3	Contrastive post_focus
2023407	block5	Control	post	wai5	Subject	Narrow	r1	124.547248	1	1	pre_focus	5	Narrow pre_focus
2023407	block5	Control	post	wai5	Subject	Narrow	r1	220.0162546	2	2	pre_focus	5	Narrow pre_focus
2023407	block5	Control	post	waat3	Verb	Narrow	r1	128.9223685	3	1	on_focus	3	Narrow on_focus
2023407	block5	Control	post	bui3	Object	Narrow	r1	152.4098058	4	1	post_focus	3	Narrow post_focus
2023407	block5	Control	post	hok3	Object	Narrow	r1	152.2234025	5	2	post_focus	3	Narrow post_focus
2023407	block5	Control	post	siu2	Subject	Contrastive	r1	118.458922	1	1	pre_focus	2	Contrastive pre_focus
2023407	block5	Control	post	gwong2	Subject	Contrastive	r1	445.5179516	2	2	pre_focus	2	Contrastive pre_focus
2023407	block5	Control	post	cyun4	Verb	Contrastive	r1	202.0505629	3	1	on_focus	4	Contrastive on_focus
2023407	block5	Control	post	laam4	Object	Contrastive	r1	348.2111178	4	1	post_focus	4	Contrastive post_focus
2023407	block5	Control	post	kau4	Object	Contrastive	r1	309.1529006	5	2	post_focus	4	Contrastive post_focus
2023407	block5	Control	post	ceoi3	Subject	Contrastive	r1	162.7025699	1	1	pre_focus	3	Contrastive pre_focus
2023407	block5	Control	post	ceoi3	Subject	Contrastive	r1	314.0306122	2	2	pre_focus	3	Contrastive pre_focus
2023407	block5	Control	post	caa4	Verb	Contrastive	r1	217.159486	3	1	on_focus	4	Contrastive on_focus
2023407	block5	Control	post	ngau4	Object	Contrastive	r1	212.154783	4	1	post_focus	4	Contrastive post_focus
2023407	block5	Control	post	jau4	Object	Contrastive	r1	361.466259	5	2	post_focus	4	Contrastive post_focus
2023407	block5	Control	post	ceoi3	Subject	Narrow	r1	131.8642605	1	1	pre_focus	3	Narrow pre_focus
2023407	block5	Control	post	ceoi3	Subject	Narrow	r1	268.7835362	2	2	pre_focus	3	Narrow pre_focus
2023407	block5	Control	post	caa4	Verb	Narrow	r1	179.6915069	3	1	on_focus	4	Narrow on_focus
2023407	block5	Control	post	ngau4	Object	Narrow	r1	222.9891946	4	1	post_focus	4	Narrow post_focus
2023407	block5	Control	post	jau4	Object	Narrow	r1	267.9608813	5	2	post_focus	4	Narrow post_focus
2023407	block5	Control	post	wai5	Subject	Narrow	r1	214.677551	1	1	pre_focus	5	Narrow pre_focus
2023407	block5	Control	post	wai5	Subject	Narrow	r1	308.7090526	2	2	pre_focus	5	Narrow pre_focus
2023407	block5	Control	post	waat3	Verb	Narrow	r1	170.8331054	3	1	pre_focus	3	Narrow pre_focus
2023407	block5	Control	post	bui3	Object	Narrow	r1	179.4629065	4	1	on_focus	3	Narrow on_focus
2023407	block5	Control	post	hok3	Object	Narrow	r1	230.7666804	5	2	on_focus	3	Narrow on_focus
2023407	block5	Control	post	ceoi3	Subject	Narrow	r1	177.9893235	1	1	on_focus	3	Narrow on_focus
2023407	block5	Control	post	ceoi3	Subject	Narrow	r1	373.2547279	2	2	on_focus	3	Narrow on_focus
2023407	block5	Control	post	caa4	Verb	Narrow	r1	287.1211989	3	1	post_focus	4	Narrow post_focus
2023407	block5	Control	post	ngau4	Object	Narrow	r1	281.9834985	4	1	post_focus	4	Narrow post_focus
2023407	block5	Control	post	jau4	Object	Narrow	r1	289.6056773	5	2	post_focus	4	Narrow post_focus
2023407	block5	Control	post	ceoi3	Subject	Contrastive	r1	158.515819	1	1	on_focus	3	Contrastive on_focus
2023407	block5	Control	post	ceoi3	Subject	Contrastive	r1	309.192314	2	2	on_focus	3	Contrastive on_focus
2023407	block5	Control	post	caa4	Verb	Contrastive	r1	289.4932751	3	1	post_focus	4	Contrastive post_focus
2023407	block5	Control	post	ngau4	Object	Contrastive	r1	214.6013749	4	1	post_focus	4	Contrastive post_focus
2023407	block5	Control	post	jau4	Object	Contrastive	r1	406.6030267	5	2	post_focus	4	Contrastive post_focus
2023407	block5	Control	post	siu2	Subject	Broad	r1	187.2922371	1	1	broad_focus	2	Broad focus
2023407	block5	Control	post	gwong2	Subject	Broad	r1	349.8331024	2	2	broad_focus	2	Broad focus
2023407	block5	Control	post	cyun4	Verb	Broad	r1	270.4066428	3	1	broad_focus	4	Broad focus
2023407	block5	Control	post	laam4	Object	Broad	r1	334.7612749	4	1	broad_focus	4	Broad focus
2023407	block5	Control	post	kau4	Object	Broad	r1	264.6943435	5	2	broad_focus	4	Broad focus
2023407	block5	Control	post	siu2	Subject	Narrow	r2	158.5896951	1	1	pre_focus	2	Narrow pre_focus
2023407	block5	Control	post	gwong2	Subject	Narrow	r2	380.8890779	2	2	pre_focus	2	Narrow pre_focus
2023407	block5	Control	post	cyun4	Verb	Narrow	r2	210.6111333	3	1	on_focus	4	Narrow on_focus
2023407	block5	Control	post	laam4	Object	Narrow	r2	369.2016251	4	1	post_focus	4	Narrow post_focus
2023407	block5	Control	post	kau4	Object	Narrow	r2	153.6069051	5	2	post_focus	4	Narrow post_focus
2023407	block5	Control	post	siu2	Subject	Narrow	r2	317.2799212	1	1	on_focus	2	Narrow on_focus
2023407	block5	Control	post	gwong2	Subject	Narrow	r2	492.9265306	2	2	on_focus	2	Narrow on_focus
2023407	block5	Control	post	cyun4	Verb	Narrow	r2	387.3264477	3	1	post_focus	4	Narrow post_focus
2023407	block5	Control	post	laam4	Object	Narrow	r2	444.9464442	4	1	post_focus	4	Narrow post_focus
2023407	block5	Control	post	kau4	Object	Narrow	r2	563.0119174	5	2	post_focus	4	Narrow post_focus
2023407	block5	Control	post	ceoi3	Subject	Contrastive	r2	315.4086887	1	1	pre_focus	3	Contrastive pre_focus
2023407	block5	Control	post	ceoi3	Subject	Contrastive	r2	338.4057312	2	2	pre_focus	3	Contrastive pre_focus
2023407	block5	Control	post	caa4	Verb	Contrastive	r2	412.7153083	3	1	pre_focus	4	Contrastive pre_focus
2023407	block5	Control	post	ngau4	Object	Contrastive	r2	310.6664576	4	1	on_focus	4	Contrastive on_focus
2023407	block5	Control	post	jau4	Object	Contrastive	r2	579.5134054	5	2	on_focus	4	Contrastive on_focus
2023407	block5	Control	post	wai5	Subject	Contrastive	r2	402.1935034	1	1	pre_focus	5	Contrastive pre_focus
2023407	block5	Control	post	wai5	Subject	Contrastive	r2	401.8723802	2	2	pre_focus	5	Contrastive pre_focus
2023407	block5	Control	post	waat3	Verb	Contrastive	r2	236.5423576	3	1	on_focus	3	Contrastive on_focus
2023407	block5	Control	post	bui3	Object	Contrastive	r2	273.6468918	4	1	post_focus	3	Contrastive post_focus
2023407	block5	Control	post	hok3	Object	Contrastive	r2	483.1701056	5	2	post_focus	3	Contrastive post_focus
2023407	block5	Control	post	ceoi3	Subject	Narrow	r2	343.707588	1	1	pre_focus	3	Narrow pre_focus
2023407	block5	Control	post	ceoi3	Subject	Narrow	r2	379.4372627	2	2	pre_focus	3	Narrow pre_focus
2023407	block5	Control	post	caa4	Verb	Narrow	r2	337.6571442	3	1	on_focus	4	Narrow on_focus
2023407	block5	Control	post	ngau4	Object	Narrow	r2	326.7656332	4	1	post_focus	4	Narrow post_focus

2023407	block5	Control	post	jau4	Object	Narrow	r2	467.0550483	5	2	post_focus	4	Narrow post_focus
2023407	block5	Control	post	wai5	Subject	Narrow	r2	376.0064475	1	1	on_focus	5	Narrow on_focus
2023407	block5	Control	post	wai5	Subject	Narrow	r2	455.8121688	2	2	on_focus	5	Narrow on_focus
2023407	block5	Control	post	waat3	Verb	Narrow	r2	284.3274214	3	1	post_focus	3	Narrow post_focus
2023407	block5	Control	post	bui3	Object	Narrow	r2	268.0429818	4	1	post_focus	3	Narrow post_focus
2023407	block5	Control	post	hok3	Object	Narrow	r2	456.1466946	5	2	post_focus	3	Narrow post_focus
2023407	block5	Control	post	wai5	Subject	Contrastive	r2	408.1753707	1	1	on_focus	5	Contrastive on_focus
2023407	block5	Control	post	wai5	Subject	Contrastive	r2	391.3206555	2	2	on_focus	5	Contrastive on_focus
2023407	block5	Control	post	waat3	Verb	Contrastive	r2	231.0124337	3	1	post_focus	3	Contrastive post_focus
2023407	block5	Control	post	bui3	Object	Contrastive	r2	301.1556287	4	1	post_focus	3	Contrastive post_focus
2023407	block5	Control	post	hok3	Object	Contrastive	r2	531.1850232	5	2	post_focus	3	Contrastive post_focus
2023407	block5	Control	post	ceoi3	Subject	Broad	r2	267.7428361	1	1	broad_focus	3	Broad focus
2023407	block5	Control	post	ceoi3	Subject	Broad	r2	397.7604772	2	2	broad_focus	3	Broad focus
2023407	block5	Control	post	caa4	Verb	Broad	r2	364.9054481	3	1	broad_focus	4	Broad focus
2023407	block5	Control	post	ngau4	Object	Broad	r2	336.776446	4	1	broad_focus	4	Broad focus
2023407	block5	Control	post	jau4	Object	Broad	r2	296.0245474	5	2	broad_focus	4	Broad focus
2023407	block5	Control	post	ceoi3	Subject	Contrastive	r2	258.0458393	1	1	pre_focus	3	Contrastive pre_focus
2023407	block5	Control	post	ceoi3	Subject	Contrastive	r2	240.0993118	2	2	pre_focus	3	Contrastive pre_focus
2023407	block5	Control	post	caa4	Verb	Contrastive	r2	305.1196744	3	1	on_focus	4	Contrastive on_focus
2023407	block5	Control	post	ngau4	Object	Contrastive	r2	237.8289394	4	1	post_focus	4	Contrastive post_focus
2023407	block5	Control	post	jau4	Object	Contrastive	r2	485.6559599	5	2	post_focus	4	Contrastive post_focus
2023407	block5	Control	post	ceoi3	Subject	Narrow	r2	289.5258161	1	1	on_focus	3	Narrow on_focus
2023407	block5	Control	post	ceoi3	Subject	Narrow	r2	327.6268398	2	2	on_focus	3	Narrow on_focus
2023407	block5	Control	post	caa4	Verb	Narrow	r2	402.5202461	3	1	post_focus	4	Narrow post_focus
2023407	block5	Control	post	ngau4	Object	Narrow	r2	284.7155071	4	1	post_focus	4	Narrow post_focus
2023407	block5	Control	post	jau4	Object	Narrow	r2	541.4956323	5	2	post_focus	4	Narrow post_focus
2023407	block5	Control	post	siu2	Subject	Contrastive	r2	209.8369781	1	1	pre_focus	2	Contrastive pre_focus
2023407	block5	Control	post	gwong2	Subject	Contrastive	r2	332.522228	2	2	pre_focus	2	Contrastive pre_focus
2023407	block5	Control	post	cyun4	Verb	Contrastive	r2	346.7191017	3	1	pre_focus	4	Contrastive pre_focus
2023407	block5	Control	post	laam4	Object	Contrastive	r2	326.0265923	4	1	on_focus	4	Contrastive on_focus
2023407	block5	Control	post	kau4	Object	Contrastive	r2	464.5988928	5	2	on_focus	4	Contrastive on_focus
2023407	block5	Control	post	ceoi3	Subject	Narrow	r2	83.06621028	1	1	pre_focus	3	Narrow pre_focus
2023407	block5	Control	post	ceoi3	Subject	Narrow	r2	197.6212097	2	2	pre_focus	3	Narrow pre_focus
2023407	block5	Control	post	caa4	Verb	Narrow	r2	263.2413587	3	1	pre_focus	4	Narrow pre_focus
2023407	block5	Control	post	ngau4	Object	Narrow	r2	165.734345	4	1	on_focus	4	Narrow on_focus
2023407	block5	Control	post	jau4	Object	Narrow	r2	236.6669826	5	2	on_focus	4	Narrow on_focus
2023407	block5	Control	post	wai5	Subject	Narrow	r2	276.7134276	1	1	pre_focus	5	Narrow pre_focus
2023407	block5	Control	post	wai5	Subject	Narrow	r2	312.556174	2	2	pre_focus	5	Narrow pre_focus
2023407	block5	Control	post	waat3	Verb	Narrow	r2	250.9258525	3	1	pre_focus	3	Narrow pre_focus
2023407	block5	Control	post	bui3	Object	Narrow	r2	274.1393527	4	1	on_focus	3	Narrow on_focus
2023407	block5	Control	post	hok3	Object	Narrow	r2	241.0543138	5	2	on_focus	3	Narrow on_focus
2023407	block5	Control	post	wai5	Subject	Broad	r2	181.5180146	1	1	broad_focus	5	Broad focus
2023407	block5	Control	post	wai5	Subject	Broad	r2	268.8699924	2	2	broad_focus	5	Broad focus
2023407	block5	Control	post	waat3	Verb	Broad	r2	165.222202	3	1	broad_focus	3	Broad focus
2023407	block5	Control	post	bui3	Object	Broad	r2	203.7887512	4	1	broad_focus	3	Broad focus
2023407	block5	Control	post	hok3	Object	Broad	r2	214.3172084	5	2	broad_focus	3	Broad focus
2023407	block5	Control	post	ceoi3	Subject	Contrastive	r2	159.6156463	1	1	on_focus	3	Contrastive on_focus
2023407	block5	Control	post	ceoi3	Subject	Contrastive	r2	304.5334031	2	2	on_focus	3	Contrastive on_focus
2023407	block5	Control	post	caa4	Verb	Contrastive	r2	349.6982066	3	1	post_focus	4	Contrastive post_focus
2023407	block5	Control	post	ngau4	Object	Contrastive	r2	350.425594	4	1	post_focus	4	Contrastive post_focus
2023407	block5	Control	post	jau4	Object	Contrastive	r2	453.7455782	5	2	post_focus	4	Contrastive post_focus
2023407	block5	Control	post	siu2	Subject	Contrastive	r2	129.5445956	1	1	on_focus	2	Contrastive on_focus
2023407	block5	Control	post	gwong2	Subject	Contrastive	r2	381.0670857	2	2	on_focus	2	Contrastive on_focus
2023407	block5	Control	post	cyun4	Verb	Contrastive	r2	225.7665327	3	1	post_focus	4	Contrastive post_focus
2023407	block5	Control	post	laam4	Object	Contrastive	r2	448.6356584	4	1	post_focus	4	Contrastive post_focus
2023407	block5	Control	post	kau4	Object	Contrastive	r2	504.184196	5	2	post_focus	4	Contrastive post_focus
2023407	block5	Control	post	siu2	Subject	Contrastive	r2	219.3983371	1	1	pre_focus	2	Contrastive pre_focus
2023407	block5	Control	post	gwong2	Subject	Contrastive	r2	431.7087787	2	2	pre_focus	2	Contrastive pre_focus
2023407	block5	Control	post	cyun4	Verb	Contrastive	r2	232.9061742	3	1	on_focus	4	Contrastive on_focus
2023407	block5	Control	post	laam4	Object	Contrastive	r2	408.77677	4	1	post_focus	4	Contrastive post_focus
2023407	block5	Control	post	kau4	Object	Contrastive	r2	204.6995465	5	2	post_focus	4	Contrastive post_focus
2023407	block5	Control	post	siu2	Subject	Narrow	r2	162.9838883	1	1	pre_focus	2	Narrow pre_focus
2023407	block5	Control	post	gwong2	Subject	Narrow	r2	326.2618381	2	2	pre_focus	2	Narrow pre_focus
2023407	block5	Control	post	cyun4	Verb	Narrow	r2	294.5866039	3	1	pre_focus	4	Narrow pre_focus
2023407	block5	Control	post	laam4	Object	Narrow	r2	268.4332791	4	1	on_focus	4	Narrow on_focus
2023407	block5	Control	post	kau4	Object	Narrow	r2	185.9602578	5	2	on_focus	4	Narrow on_focus

2023407	block5	Control	post	siu2	Subject	Broad	r2	189.9036281	1	1	broad_focus	2	Broad focus
2023407	block5	Control	post	gwong2	Subject	Broad	r2	569.6058741	2	2	broad_focus	2	Broad focus
2023407	block5	Control	post	cyun4	Verb	Broad	r2	406.5759637	3	1	broad_focus	4	Broad focus
2023407	block5	Control	post	laam4	Object	Broad	r2	317.1397351	4	1	broad_focus	4	Broad focus
2023407	block5	Control	post	kau4	Object	Broad	r2	223.1146744	5	2	broad_focus	4	Broad focus
2023407	block5	Control	post	wai5	Subject	Contrastive	r2	140.9829932	1	1	pre_focus	5	Contrastive pre_focus
2023407	block5	Control	post	wai5	Subject	Contrastive	r2	326.371944	2	2	pre_focus	5	Contrastive pre_focus
2023407	block5	Control	post	waat3	Verb	Contrastive	r2	173.5990077	3	1	pre_focus	3	Contrastive pre_focus
2023407	block5	Control	post	bui3	Object	Contrastive	r2	183.8288451	4	1	on_focus	3	Contrastive on_focus
2023407	block5	Control	post	hok3	Object	Contrastive	r2	243.8219191	5	2	on_focus	3	Contrastive on_focus
2023407	block5	Control	post	wai5	Subject	Narrow	r2	129.6030116	1	1	pre_focus	5	Narrow pre_focus
2023407	block5	Control	post	wai5	Subject	Narrow	r2	114.2768959	2	2	pre_focus	5	Narrow pre_focus
2023407	block5	Control	post	waat3	Verb	Narrow	r2	150.3051531	3	1	on_focus	3	Narrow on_focus
2023407	block5	Control	post	bui3	Object	Narrow	r2	109.6193093	4	1	post_focus	3	Narrow post_focus
2023407	block5	Control	post	hok3	Object	Narrow	r2	381.2010611	5	2	post_focus	3	Narrow post_focus
2023407	block5	Control	pre	ceoi3	Subject	Contrastive	r1	157.2282004	1	1	pre_focus	3	Contrastive pre_focus
2023407	block5	Control	pre	ceoi3	Subject	Contrastive	r1	152.2572039	2	2	pre_focus	3	Contrastive pre_focus
2023407	block5	Control	pre	caa4	Verb	Contrastive	r1	106.5641485	3	1	pre_focus	4	Contrastive pre_focus
2023407	block5	Control	pre	ngau4	Object	Contrastive	r1	164.585855	4	1	on_focus	4	Contrastive on_focus
2023407	block5	Control	pre	jau4	Object	Contrastive	r1	177.5667045	5	2	on_focus	4	Contrastive on_focus
2023407	block5	Control	pre	siu2	Subject	Contrastive	r1	173.3164653	1	1	pre_focus	2	Contrastive pre_focus
2023407	block5	Control	pre	gwong2	Subject	Contrastive	r1	250.7373071	2	2	pre_focus	2	Contrastive pre_focus
2023407	block5	Control	pre	cyun4	Verb	Contrastive	r1	180.5424044	3	1	on_focus	4	Contrastive on_focus
2023407	block5	Control	pre	laam4	Object	Contrastive	r1	263.6691502	4	1	post_focus	4	Contrastive post_focus
2023407	block5	Control	pre	kau4	Object	Contrastive	r1	105.8884069	5	2	post_focus	4	Contrastive post_focus
2023407	block5	Control	pre	ceoi3	Subject	Broad	r1	160.9495732	1	1	broad_focus	3	Broad focus
2023407	block5	Control	pre	ceoi3	Subject	Broad	r1	244.5152684	2	2	broad_focus	3	Broad focus
2023407	block5	Control	pre	caa4	Verb	Broad	r1	155.6272959	3	1	broad_focus	4	Broad focus
2023407	block5	Control	pre	ngau4	Object	Broad	r1	161.3403952	4	1	broad_focus	4	Broad focus
2023407	block5	Control	pre	jau4	Object	Broad	r1	164.7374271	5	2	broad_focus	4	Broad focus
2023407	block5	Control	pre	siu2	Subject	Narrow	r1	123.2789116	1	1	pre_focus	2	Narrow pre_focus
2023407	block5	Control	pre	gwong2	Subject	Narrow	r1	267.7348804	2	2	pre_focus	2	Narrow pre_focus
2023407	block5	Control	pre	cyun4	Verb	Narrow	r1	109.8008658	3	1	pre_focus	4	Narrow pre_focus
2023407	block5	Control	pre	laam4	Object	Narrow	r1	190.7560123	4	1	on_focus	4	Narrow on_focus
2023407	block5	Control	pre	kau4	Object	Narrow	r1	168.2415357	5	2	on_focus	4	Narrow on_focus
2023407	block5	Control	pre	wai5	Subject	Contrastive	r1	246.9203151	1	1	pre_focus	5	Contrastive pre_focus
2023407	block5	Control	pre	wai5	Subject	Contrastive	r1	326.2089187	2	2	pre_focus	5	Contrastive pre_focus
2023407	block5	Control	pre	waat3	Verb	Contrastive	r1	147.29917	3	1	pre_focus	3	Contrastive pre_focus
2023407	block5	Control	pre	bui3	Object	Contrastive	r1	183.752703	4	1	on_focus	3	Contrastive on_focus
2023407	block5	Control	pre	hok3	Object	Contrastive	r1	275.0256957	5	2	on_focus	3	Contrastive on_focus
2023407	block5	Control	pre	siu2	Subject	Contrastive	r1	193.0049947	1	1	on_focus	2	Contrastive on_focus
2023407	block5	Control	pre	gwong2	Subject	Contrastive	r1	362.2399106	2	2	on_focus	2	Contrastive on_focus
2023407	block5	Control	pre	cyun4	Verb	Contrastive	r1	141.6918545	3	1	post_focus	4	Contrastive post_focus
2023407	block5	Control	pre	laam4	Object	Contrastive	r1	289.7041588	4	1	post_focus	4	Contrastive post_focus
2023407	block5	Control	pre	kau4	Object	Contrastive	r1	121.7893824	5	2	post_focus	4	Contrastive post_focus
2023407	block5	Control	pre	ceoi3	Subject	Narrow	r1	134.6544002	1	1	on_focus	3	Narrow on_focus
2023407	block5	Control	pre	ceoi3	Subject	Narrow	r1	236.7623093	2	2	on_focus	3	Narrow on_focus
2023407	block5	Control	pre	caa4	Verb	Narrow	r1	104.5423554	3	1	post_focus	4	Narrow post_focus
2023407	block5	Control	pre	ngau4	Object	Narrow	r1	122.2649691	4	1	post_focus	4	Narrow post_focus
2023407	block5	Control	pre	jau4	Object	Narrow	r1	138.7256609	5	2	post_focus	4	Narrow post_focus
2023407	block5	Control	pre	wai5	Subject	Contrastive	r1	188.2903971	1	1	on_focus	5	Contrastive on_focus
2023407	block5	Control	pre	wai5	Subject	Contrastive	r1	372.5459787	2	2	on_focus	5	Contrastive on_focus
2023407	block5	Control	pre	waat3	Verb	Contrastive	r1	137.2161396	3	1	post_focus	3	Contrastive post_focus
2023407	block5	Control	pre	bui3	Object	Contrastive	r1	154.2651079	4	1	post_focus	3	Contrastive post_focus
2023407	block5	Control	pre	hok3	Object	Contrastive	r1	117.6356765	5	2	post_focus	3	Contrastive post_focus
2023407	block5	Control	pre	wai5	Subject	Narrow	r1	151.2038781	1	1	pre_focus	5	Narrow pre_focus
2023407	block5	Control	pre	wai5	Subject	Narrow	r1	228.0258265	2	2	pre_focus	5	Narrow pre_focus
2023407	block5	Control	pre	waat3	Verb	Narrow	r1	162.2103707	3	1	on_focus	3	Narrow on_focus
2023407	block5	Control	pre	bui3	Object	Narrow	r1	153.95825	4	1	post_focus	3	Narrow post_focus
2023407	block5	Control	pre	hok3	Object	Narrow	r1	105.9844416	5	2	post_focus	3	Narrow post_focus
2023407	block5	Control	pre	ceoi3	Subject	Contrastive	r1	125.2618968	1	1	on_focus	3	Contrastive on_focus
2023407	block5	Control	pre	ceoi3	Subject	Contrastive	r1	212.4488651	2	2	on_focus	3	Contrastive on_focus
2023407	block5	Control	pre	caa4	Verb	Contrastive	r1	129.6668052	3	1	post_focus	4	Contrastive post_focus
2023407	block5	Control	pre	ngau4	Object	Contrastive	r1	185.0088461	4	1	post_focus	4	Contrastive post_focus
2023407	block5	Control	pre	jau4	Object	Contrastive	r1	172.8549365	5	2	post_focus	4	Contrastive post_focus
2023407	block5	Control	pre	wai5	Subject	Narrow	r1	123.8450719	1	1	pre_focus	5	Narrow pre_focus

2023407	block5	Control	pre	wai5	Subject	Narrow	r1	270.6540626	2	2	pre_focus	5	Narrow pre_focus
2023407	block5	Control	pre	waat3	Verb	Narrow	r1	134.4061266	3	1	pre_focus	3	Narrow pre_focus
2023407	block5	Control	pre	bui3	Object	Narrow	r1	158.9791761	4	1	on_focus	3	Narrow on_focus
2023407	block5	Control	pre	hok3	Object	Narrow	r1	113.1864243	5	2	on_focus	3	Narrow on_focus
2023407	block5	Control	pre	ceoi3	Subject	Contrastive	r1	176.0468327	1	1	pre_focus	3	Contrastive pre_focus
2023407	block5	Control	pre	ceoi3	Subject	Contrastive	r1	326.178852	2	2	pre_focus	3	Contrastive pre_focus
2023407	block5	Control	pre	caa4	Verb	Contrastive	r1	132.2252059	3	1	on_focus	4	Contrastive on_focus
2023407	block5	Control	pre	ngau4	Object	Contrastive	r1	99.27261571	4	1	post_focus	4	Contrastive post_focus
2023407	block5	Control	pre	jau4	Object	Contrastive	r1	145.6673364	5	2	post_focus	4	Contrastive post_focus
2023407	block5	Control	pre	wai5	Subject	Contrastive	r1	212.2724183	1	1	pre_focus	5	Contrastive pre_focus
2023407	block5	Control	pre	wai5	Subject	Contrastive	r1	230.2814007	2	2	pre_focus	5	Contrastive pre_focus
2023407	block5	Control	pre	waat3	Verb	Contrastive	r1	119.526005	3	1	on_focus	3	Contrastive on_focus
2023407	block5	Control	pre	bui3	Object	Contrastive	r1	167.0157327	4	1	post_focus	3	Contrastive post_focus
2023407	block5	Control	pre	hok3	Object	Contrastive	r1	125.5645682	5	2	post_focus	3	Contrastive post_focus
2023407	block5	Control	pre	wai5	Subject	Narrow	r1	198.6838231	1	1	on_focus	5	Narrow on_focus
2023407	block5	Control	pre	wai5	Subject	Narrow	r1	183.0333183	2	2	on_focus	5	Narrow on_focus
2023407	block5	Control	pre	waat3	Verb	Narrow	r1	148.0322963	3	1	post_focus	3	Narrow post_focus
2023407	block5	Control	pre	bui3	Object	Narrow	r1	143.233968	4	1	post_focus	3	Narrow post_focus
2023407	block5	Control	pre	hok3	Object	Narrow	r1	93.69068966	5	2	post_focus	3	Narrow post_focus
2023407	block5	Control	pre	wai5	Subject	Broad	r1	166.7119748	1	1	broad_focus	5	Broad focus
2023407	block5	Control	pre	wai5	Subject	Broad	r1	273.5241299	2	2	broad_focus	5	Broad focus
2023407	block5	Control	pre	waat3	Verb	Broad	r1	131.7601476	3	1	broad_focus	3	Broad focus
2023407	block5	Control	pre	bui3	Object	Broad	r1	131.4966393	4	1	broad_focus	3	Broad focus
2023407	block5	Control	pre	hok3	Object	Broad	r1	112.5443635	5	2	broad_focus	3	Broad focus
2023407	block5	Control	pre	siu2	Subject	Narrow	r1	155.4870418	1	1	pre_focus	2	Narrow pre_focus
2023407	block5	Control	pre	gwong2	Subject	Narrow	r1	307.8331444	2	2	pre_focus	2	Narrow pre_focus
2023407	block5	Control	pre	cyun4	Verb	Narrow	r1	150.2266805	3	1	on_focus	4	Narrow on_focus
2023407	block5	Control	pre	laam4	Object	Narrow	r1	251.8730107	4	1	post_focus	4	Narrow post_focus
2023407	block5	Control	pre	kau4	Object	Narrow	r1	101.4974954	5	2	post_focus	4	Narrow post_focus
2023407	block5	Control	pre	ceoi3	Subject	Narrow	r1	131.4541844	1	1	pre_focus	3	Narrow pre_focus
2023407	block5	Control	pre	ceoi3	Subject	Narrow	r1	307.5910089	2	2	pre_focus	3	Narrow pre_focus
2023407	block5	Control	pre	caa4	Verb	Narrow	r1	144.4870192	3	1	on_focus	4	Narrow on_focus
2023407	block5	Control	pre	ngau4	Object	Narrow	r1	111.5386432	4	1	post_focus	4	Narrow post_focus
2023407	block5	Control	pre	jau4	Object	Narrow	r1	175.765446	5	2	post_focus	4	Narrow post_focus
2023407	block5	Control	pre	ceoi3	Subject	Narrow	r1	102.2896086	1	1	pre_focus	3	Narrow pre_focus
2023407	block5	Control	pre	ceoi3	Subject	Narrow	r1	214.4900331	2	2	pre_focus	3	Narrow pre_focus
2023407	block5	Control	pre	caa4	Verb	Narrow	r1	171.0628746	3	1	pre_focus	4	Narrow pre_focus
2023407	block5	Control	pre	ngau4	Object	Narrow	r1	151.0026731	4	1	on_focus	4	Narrow on_focus
2023407	block5	Control	pre	jau4	Object	Narrow	r1	240.1655848	5	2	on_focus	4	Narrow on_focus
2023407	block5	Control	pre	siu2	Subject	Broad	r1	184.8625958	1	1	broad_focus	2	Broad focus
2023407	block5	Control	pre	gwong2	Subject	Broad	r1	290.9267109	2	2	broad_focus	2	Broad focus
2023407	block5	Control	pre	cyun4	Verb	Broad	r1	189.3759846	3	1	broad_focus	4	Broad focus
2023407	block5	Control	pre	laam4	Object	Broad	r1	247.6205522	4	1	broad_focus	4	Broad focus
2023407	block5	Control	pre	kau4	Object	Broad	r1	115.6089127	5	2	broad_focus	4	Broad focus
2023407	block5	Control	pre	siu2	Subject	Narrow	r1	136.8240886	1	1	on_focus	2	Narrow on_focus
2023407	block5	Control	pre	gwong2	Subject	Narrow	r1	316.566083	2	2	on_focus	2	Narrow on_focus
2023407	block5	Control	pre	cyun4	Verb	Narrow	r1	141.1845875	3	1	post_focus	4	Narrow post_focus
2023407	block5	Control	pre	laam4	Object	Narrow	r1	150.3279638	4	1	post_focus	4	Narrow post_focus
2023407	block5	Control	pre	kau4	Object	Narrow	r1	103.984858	5	2	post_focus	4	Narrow post_focus
2023407	block5	Control	pre	siu2	Subject	Contrastive	r1	163.7547968	1	1	pre_focus	2	Contrastive pre_focus
2023407	block5	Control	pre	gwong2	Subject	Contrastive	r1	294.5300684	2	2	pre_focus	2	Contrastive pre_focus
2023407	block5	Control	pre	cyun4	Verb	Contrastive	r1	251.898244	3	1	pre_focus	4	Contrastive pre_focus
2023407	block5	Control	pre	laam4	Object	Contrastive	r1	283.0684075	4	1	on_focus	4	Contrastive on_focus
2023407	block5	Control	pre	kau4	Object	Contrastive	r1	64.41747406	5	2	on_focus	4	Contrastive on_focus
2023407	block5	Control	pre	wai5	Subject	Narrow	r2	137.7602501	1	1	pre_focus	5	Narrow pre_focus
2023407	block5	Control	pre	wai5	Subject	Narrow	r2	279.2412025	2	2	pre_focus	5	Narrow pre_focus
2023407	block5	Control	pre	waat3	Verb	Narrow	r2	156.9825731	3	1	on_focus	3	Narrow on_focus
2023407	block5	Control	pre	bui3	Object	Narrow	r2	167.50032	4	1	post_focus	3	Narrow post_focus
2023407	block5	Control	pre	hok3	Object	Narrow	r2	146.4758455	5	2	post_focus	3	Narrow post_focus
2023407	block5	Control	pre	siu2	Subject	Narrow	r2	108.5447805	1	1	pre_focus	2	Narrow pre_focus
2023407	block5	Control	pre	gwong2	Subject	Narrow	r2	249.5234856	2	2	pre_focus	2	Narrow pre_focus
2023407	block5	Control	pre	cyun4	Verb	Narrow	r2	190.3000597	3	1	on_focus	4	Narrow on_focus
2023407	block5	Control	pre	laam4	Object	Narrow	r2	282.4632075	4	1	post_focus	4	Narrow post_focus
2023407	block5	Control	pre	kau4	Object	Narrow	r2	198.4416615	5	2	post_focus	4	Narrow post_focus
2023407	block5	Control	pre	ceoi3	Subject	Narrow	r2	148.8601768	1	1	pre_focus	3	Narrow pre_focus
2023407	block5	Control	pre	ceoi3	Subject	Narrow	r2	260.7293191	2	2	pre_focus	3	Narrow pre_focus

2023407	block5	Control	pre	caa4	Verb	Narrow	r2	172.7627503	3	1	on_focus	4	Narrow on_focus
2023407	block5	Control	pre	ngau4	Object	Narrow	r2	145.8215778	4	1	post_focus	4	Narrow post_focus
2023407	block5	Control	pre	jau4	Object	Narrow	r2	267.7389124	5	2	post_focus	4	Narrow post_focus
2023407	block5	Control	pre	wai5	Subject	Narrow	r2	164.9584487	1	1	pre_focus	5	Narrow pre_focus
2023407	block5	Control	pre	wai5	Subject	Narrow	r2	207.5542322	2	2	pre_focus	5	Narrow pre_focus
2023407	block5	Control	pre	waat3	Verb	Narrow	r2	142.3340255	3	1	pre_focus	3	Narrow pre_focus
2023407	block5	Control	pre	bui3	Object	Narrow	r2	150.4781147	4	1	on_focus	3	Narrow on_focus
2023407	block5	Control	pre	hok3	Object	Narrow	r2	250.6962508	5	2	on_focus	3	Narrow on_focus
2023407	block5	Control	pre	wai5	Subject	Narrow	r2	182.6883125	1	1	on_focus	5	Narrow on_focus
2023407	block5	Control	pre	wai5	Subject	Narrow	r2	314.2188145	2	2	on_focus	5	Narrow on_focus
2023407	block5	Control	pre	waat3	Verb	Narrow	r2	139.5938503	3	1	post_focus	3	Narrow post_focus
2023407	block5	Control	pre	bui3	Object	Narrow	r2	207.715289	4	1	post_focus	3	Narrow post_focus
2023407	block5	Control	pre	hok3	Object	Narrow	r2	188.5284265	5	2	post_focus	3	Narrow post_focus
2023407	block5	Control	pre	ceoi3	Subject	Contrastive	r2	125.8773935	1	1	pre_focus	3	Contrastive pre_focus
2023407	block5	Control	pre	ceoi3	Subject	Contrastive	r2	258.6332963	2	2	pre_focus	3	Contrastive pre_focus
2023407	block5	Control	pre	caa4	Verb	Contrastive	r2	165.1152989	3	1	on_focus	4	Contrastive on_focus
2023407	block5	Control	pre	ngau4	Object	Contrastive	r2	185.9877619	4	1	post_focus	4	Contrastive post_focus
2023407	block5	Control	pre	jau4	Object	Contrastive	r2	172.4193051	5	2	post_focus	4	Contrastive post_focus
2023407	block5	Control	pre	wai5	Subject	Broad	r2	278.5227335	1	1	broad_focus	5	Broad focus
2023407	block5	Control	pre	wai5	Subject	Broad	r2	491.590918	2	2	broad_focus	5	Broad focus
2023407	block5	Control	pre	waat3	Verb	Broad	r2	197.9630559	3	1	broad_focus	3	Broad focus
2023407	block5	Control	pre	bui3	Object	Broad	r2	166.3780961	4	1	broad_focus	3	Broad focus
2023407	block5	Control	pre	hok3	Object	Broad	r2	117.7348457	5	2	broad_focus	3	Broad focus
2023407	block5	Control	pre	ceoi3	Subject	Broad	r2	149.0324885	1	1	broad_focus	3	Broad focus
2023407	block5	Control	pre	ceoi3	Subject	Broad	r2	263.0083144	2	2	broad_focus	3	Broad focus
2023407	block5	Control	pre	caa4	Verb	Broad	r2	157.2243352	3	1	broad_focus	4	Broad focus
2023407	block5	Control	pre	ngau4	Object	Broad	r2	204.9231339	4	1	broad_focus	4	Broad focus
2023407	block5	Control	pre	jau4	Object	Broad	r2	148.3811225	5	2	broad_focus	4	Broad focus
2023407	block5	Control	pre	siu2	Subject	Contrastive	r2	134.2148038	1	1	on_focus	2	Contrastive on_focus
2023407	block5	Control	pre	gwong2	Subject	Contrastive	r2	322.8004698	2	2	on_focus	2	Contrastive on_focus
2023407	block5	Control	pre	cyun4	Verb	Contrastive	r2	191.7751557	3	1	post_focus	4	Contrastive post_focus
2023407	block5	Control	pre	laam4	Object	Contrastive	r2	247.8421853	4	1	post_focus	4	Contrastive post_focus
2023407	block5	Control	pre	kau4	Object	Contrastive	r2	104.8114138	5	2	post_focus	4	Contrastive post_focus
2023407	block5	Control	pre	siu2	Subject	Contrastive	r2	109.8495678	1	1	pre_focus	2	Contrastive pre_focus
2023407	block5	Control	pre	gwong2	Subject	Contrastive	r2	319.4157318	2	2	pre_focus	2	Contrastive pre_focus
2023407	block5	Control	pre	cyun4	Verb	Contrastive	r2	165.2432577	3	1	pre_focus	4	Contrastive pre_focus
2023407	block5	Control	pre	laam4	Object	Contrastive	r2	225.6547063	4	1	on_focus	4	Contrastive on_focus
2023407	block5	Control	pre	kau4	Object	Contrastive	r2	117.8886536	5	2	on_focus	4	Contrastive on_focus
2023407	block5	Control	pre	siu2	Subject	Contrastive	r2	125.7938215	1	1	pre_focus	2	Contrastive pre_focus
2023407	block5	Control	pre	gwong2	Subject	Contrastive	r2	326.1612461	2	2	pre_focus	2	Contrastive pre_focus
2023407	block5	Control	pre	cyun4	Verb	Contrastive	r2	198.3890092	3	1	on_focus	4	Contrastive on_focus
2023407	block5	Control	pre	laam4	Object	Contrastive	r2	312.506414	4	1	post_focus	4	Contrastive post_focus
2023407	block5	Control	pre	kau4	Object	Contrastive	r2	107.4206107	5	2	post_focus	4	Contrastive post_focus
2023407	block5	Control	pre	wai5	Subject	Contrastive	r2	135.2877402	1	1	pre_focus	5	Contrastive pre_focus
2023407	block5	Control	pre	wai5	Subject	Contrastive	r2	244.9754568	2	2	pre_focus	5	Contrastive pre_focus
2023407	block5	Control	pre	waat3	Verb	Contrastive	r2	194.18472	3	1	on_focus	3	Contrastive on_focus
2023407	block5	Control	pre	bui3	Object	Contrastive	r2	178.0680548	4	1	post_focus	3	Contrastive post_focus
2023407	block5	Control	pre	hok3	Object	Contrastive	r2	141.8726154	5	2	post_focus	3	Contrastive post_focus
2023407	block5	Control	pre	ceoi3	Subject	Contrastive	r2	163.3835478	1	1	on_focus	3	Contrastive on_focus
2023407	block5	Control	pre	ceoi3	Subject	Contrastive	r2	249.9646989	2	2	on_focus	3	Contrastive on_focus
2023407	block5	Control	pre	caa4	Verb	Contrastive	r2	157.2720103	3	1	post_focus	4	Contrastive post_focus
2023407	block5	Control	pre	ngau4	Object	Contrastive	r2	202.2676057	4	1	post_focus	4	Contrastive post_focus
2023407	block5	Control	pre	jau4	Object	Contrastive	r2	369.9447502	5	2	post_focus	4	Contrastive post_focus
2023407	block5	Control	pre	ceoi3	Subject	Narrow	r2	114.2607072	1	1	on_focus	3	Narrow on_focus
2023407	block5	Control	pre	ceoi3	Subject	Narrow	r2	286.6503401	2	2	on_focus	3	Narrow on_focus
2023407	block5	Control	pre	caa4	Verb	Narrow	r2	141.9062882	3	1	post_focus	4	Narrow post_focus
2023407	block5	Control	pre	ngau4	Object	Narrow	r2	118.1538972	4	1	post_focus	4	Narrow post_focus
2023407	block5	Control	pre	jau4	Object	Narrow	r2	134.0173401	5	2	post_focus	4	Narrow post_focus
2023407	block5	Control	pre	wai5	Subject	Contrastive	r2	127.5304143	1	1	on_focus	5	Contrastive on_focus
2023407	block5	Control	pre	wai5	Subject	Contrastive	r2	273.7209873	2	2	on_focus	5	Contrastive on_focus
2023407	block5	Control	pre	waat3	Verb	Contrastive	r2	129.7945484	3	1	post_focus	3	Contrastive post_focus
2023407	block5	Control	pre	bui3	Object	Contrastive	r2	146.4804836	4	1	post_focus	3	Contrastive post_focus
2023407	block5	Control	pre	hok3	Object	Contrastive	r2	181.6771005	5	2	post_focus	3	Contrastive post_focus
2023407	block5	Control	pre	siu2	Subject	Broad	r2	190.0924877	1	1	broad_focus	2	Broad focus
2023407	block5	Control	pre	gwong2	Subject	Broad	r2	419.0519976	2	2	broad_focus	2	Broad focus
2023407	block5	Control	pre	cyun4	Verb	Broad	r2	166.5183526	3	1	broad_focus	4	Broad focus

2023407	block5	Control	pre	laam4	Object	Broad	r2	284.6468594	4	1	broad_focus	4	Broad focus
2023407	block5	Control	pre	kau4	Object	Broad	r2	107.1709788	5	2	broad_focus	4	Broad focus
2023407	block5	Control	pre	siu2	Subject	Narrow	r2	142.1470108	1	1	on_focus	2	Narrow on_focus
2023407	block5	Control	pre	gwong2	Subject	Narrow	r2	370.4127101	2	2	on_focus	2	Narrow on_focus
2023407	block5	Control	pre	cyun4	Verb	Narrow	r2	219.9841013	3	1	post_focus	4	Narrow post_focus
2023407	block5	Control	pre	laam4	Object	Narrow	r2	262.189711	4	1	post_focus	4	Narrow post_focus
2023407	block5	Control	pre	kau4	Object	Narrow	r2	176.5316163	5	2	post_focus	4	Narrow post_focus
2023407	block5	Control	pre	wai5	Subject	Contrastive	r2	180.849259	1	1	pre_focus	5	Contrastive pre_focus
2023407	block5	Control	pre	wai5	Subject	Contrastive	r2	248.3898821	2	2	pre_focus	5	Contrastive pre_focus
2023407	block5	Control	pre	waat3	Verb	Contrastive	r2	136.5569423	3	1	pre_focus	3	Contrastive pre_focus
2023407	block5	Control	pre	bui3	Object	Contrastive	r2	160.9441663	4	1	on_focus	3	Contrastive on_focus
2023407	block5	Control	pre	hok3	Object	Contrastive	r2	130.5439404	5	2	on_focus	3	Contrastive on_focus
2023407	block5	Control	pre	siu2	Subject	Narrow	r2	110.6332089	1	1	pre_focus	2	Narrow pre_focus
2023407	block5	Control	pre	gwong2	Subject	Narrow	r2	302.8775797	2	2	pre_focus	2	Narrow pre_focus
2023407	block5	Control	pre	cyun4	Verb	Narrow	r2	116.4313503	3	1	pre_focus	4	Narrow pre_focus
2023407	block5	Control	pre	laam4	Object	Narrow	r2	314.7798853	4	1	on_focus	4	Narrow on_focus
2023407	block5	Control	pre	kau4	Object	Narrow	r2	223.6761451	5	2	on_focus	4	Narrow on_focus
2023407	block5	Control	pre	ceoi3	Subject	Narrow	r2	118.1968896	1	1	pre_focus	3	Narrow pre_focus
2023407	block5	Control	pre	ceoi3	Subject	Narrow	r2	235.8321858	2	2	pre_focus	3	Narrow pre_focus
2023407	block5	Control	pre	caa4	Verb	Narrow	r2	212.0620451	3	1	pre_focus	4	Narrow pre_focus
2023407	block5	Control	pre	ngau4	Object	Narrow	r2	168.930033	4	1	on_focus	4	Narrow on_focus
2023407	block5	Control	pre	jau4	Object	Narrow	r2	318.4220436	5	2	on_focus	4	Narrow on_focus
2023407	block5	Control	pre	ceoi3	Subject	Contrastive	r2	120.8863854	1	1	pre_focus	3	Contrastive pre_focus
2023407	block5	Control	pre	ceoi3	Subject	Contrastive	r2	318.7923485	2	2	pre_focus	3	Contrastive pre_focus
2023407	block5	Control	pre	caa4	Verb	Contrastive	r2	189.2383392	3	1	pre_focus	4	Contrastive pre_focus
2023407	block5	Control	pre	ngau4	Object	Contrastive	r2	128.8197602	4	1	on_focus	4	Contrastive on_focus
2023407	block5	Control	pre	jau4	Object	Contrastive	r2	249.2769051	5	2	on_focus	4	Contrastive on_focus
2023408	block1	Control	post	zoeng1	Subject	Broad	r1	302.6014109	1	1	broad_focus	1	Broad focus
2023408	block1	Control	post	saang1	Subject	Broad	r1	295.191736	2	2	broad_focus	1	Broad focus
2023408	block1	Control	post	tsa1	Verb	Broad	r1	220.0226757	3	1	broad_focus	1	Broad focus
2023408	block1	Control	post	fei1	Object	Broad	r1	92.69549725	4	1	broad_focus	1	Broad focus
2023408	block1	Control	post	gei1	Object	Broad	r1	139.4580499	5	2	broad_focus	1	Broad focus
2023408	block1	Control	post	jyun2	Subject	Broad	r1	292.0498866	1	1	broad_focus	2	Broad focus
2023408	block1	Control	post	jyun2	Subject	Broad	r1	222.9988662	2	2	broad_focus	2	Broad focus
2023408	block1	Control	post	mo2	Verb	Broad	r1	194.2003023	3	1	broad_focus	2	Broad focus
2023408	block1	Control	post	gau2	Object	Broad	r1	159.9433107	4	1	broad_focus	2	Broad focus
2023408	block1	Control	post	zai2	Object	Broad	r1	234.5512094	5	2	broad_focus	2	Broad focus
2023408	block1	Control	post	sau3	Subject	Contrastive	r1	188.5099521	1	1	pre_focus	3	Contrastive pre_focus
2023408	block1	Control	post	sau3	Subject	Contrastive	r1	163.5524634	2	2	pre_focus	3	Contrastive pre_focus
2023408	block1	Control	post	sik3	Verb	Contrastive	r1	102.6411419	3	1	on_focus	3	Contrastive on_focus
2023408	block1	Control	post	baak3	Object	Contrastive	r1	109.5067291	4	1	post_focus	3	Contrastive post_focus
2023408	block1	Control	post	baak3	Object	Contrastive	r1	102.3407029	5	2	post_focus	3	Contrastive post_focus
2023408	block1	Control	post	zoeng1	Subject	Contrastive	r1	246.6575964	1	1	on_focus	1	Contrastive on_focus
2023408	block1	Control	post	saang1	Subject	Contrastive	r1	244.1399417	2	2	on_focus	1	Contrastive on_focus
2023408	block1	Control	post	tsa1	Verb	Contrastive	r1	176.7528345	3	1	post_focus	1	Contrastive post_focus
2023408	block1	Control	post	fei1	Object	Contrastive	r1	167.814059	4	1	post_focus	1	Contrastive post_focus
2023408	block1	Control	post	gei1	Object	Contrastive	r1	240.5814707	5	2	post_focus	1	Contrastive post_focus
2023408	block1	Control	post	sau3	Subject	Contrastive	r1	231.5950263	1	1	on_focus	3	Contrastive on_focus
2023408	block1	Control	post	sau3	Subject	Contrastive	r1	173.5759637	2	2	on_focus	3	Contrastive on_focus
2023408	block1	Control	post	sik3	Verb	Contrastive	r1	96.72335601	3	1	post_focus	3	Contrastive post_focus
2023408	block1	Control	post	baak3	Object	Contrastive	r1	124.1146155	4	1	post_focus	3	Contrastive post_focus
2023408	block1	Control	post	baak3	Object	Contrastive	r1	143.7559266	5	2	post_focus	3	Contrastive post_focus
2023408	block1	Control	post	jyun2	Subject	Narrow	r1	287.994817	1	1	on_focus	2	Narrow on_focus
2023408	block1	Control	post	jyun2	Subject	Narrow	r1	233.6180398	2	2	on_focus	2	Narrow on_focus
2023408	block1	Control	post	mo2	Verb	Narrow	r1	181.2872317	3	1	post_focus	2	Narrow post_focus
2023408	block1	Control	post	gau2	Object	Narrow	r1	150.4988662	4	1	post_focus	2	Narrow post_focus
2023408	block1	Control	post	zai2	Object	Narrow	r1	264.1780045	5	2	post_focus	2	Narrow post_focus
2023408	block1	Control	post	sau3	Subject	Narrow	r1	128.9705215	1	1	on_focus	3	Narrow on_focus
2023408	block1	Control	post	sau3	Subject	Narrow	r1	131.4037361	2	2	on_focus	3	Narrow on_focus
2023408	block1	Control	post	sik3	Verb	Narrow	r1	101.6865079	3	1	post_focus	3	Narrow post_focus
2023408	block1	Control	post	baak3	Object	Narrow	r1	91.28968254	4	1	post_focus	3	Narrow post_focus
2023408	block1	Control	post	baak3	Object	Narrow	r1	102.5793651	5	2	post_focus	3	Narrow post_focus
2023408	block1	Control	post	zoeng1	Subject	Narrow	r1	189.6696271	1	1	pre_focus	1	Narrow pre_focus
2023408	block1	Control	post	saang1	Subject	Narrow	r1	245.1080877	2	2	pre_focus	1	Narrow pre_focus
2023408	block1	Control	post	tsa1	Verb	Narrow	r1	199.952381	3	1	on_focus	1	Narrow on_focus
2023408	block1	Control	post	fei1	Object	Narrow	r1	165.2222222	4	1	post_focus	1	Narrow post_focus

**2023408**	block1	Control	post	gei1	Object	Narrow	r1	276.6869129	5	2	post_focus	1	Narrow post_focus
**2023408**	block1	Control	post	jyun2	Subject	Contrastive	r1	248.5345805	1	1	on_focus	2	Contrastive on_focus
**2023408**	block1	Control	post	jyun2	Subject	Contrastive	r1	253.4441925	2	2	on_focus	2	Contrastive on_focus
**2023408**	block1	Control	post	mo2	Verb	Contrastive	r1	175.1795163	3	1	post_focus	2	Contrastive post_focus
**2023408**	block1	Control	post	gau2	Object	Contrastive	r1	139.0208941	4	1	post_focus	2	Contrastive post_focus
**2023408**	block1	Control	post	zai2	Object	Contrastive	r1	296.8064059	5	2	post_focus	2	Contrastive post_focus
**2023408**	block1	Control	post	jyun2	Subject	Narrow	r1	244.7513399	1	1	pre_focus	2	Narrow pre_focus
**2023408**	block1	Control	post	jyun2	Subject	Narrow	r1	285.0365331	2	2	pre_focus	2	Narrow pre_focus
**2023408**	block1	Control	post	mo2	Verb	Narrow	r1	204.6976568	3	1	pre_focus	2	Narrow pre_focus
**2023408**	block1	Control	post	gau2	Object	Narrow	r1	167.8124213	4	1	on_focus	2	Narrow on_focus
**2023408**	block1	Control	post	zai2	Object	Narrow	r1	170.2091837	5	2	on_focus	2	Narrow on_focus
**2023408**	block1	Control	post	zoeng1	Subject	Narrow	r1	250.5687831	1	1	on_focus	1	Narrow on_focus
**2023408**	block1	Control	post	saang1	Subject	Narrow	r1	267.7569917	2	2	on_focus	1	Narrow on_focus
**2023408**	block1	Control	post	tsa1	Verb	Narrow	r1	171.9835871	3	1	post_focus	1	Narrow post_focus
**2023408**	block1	Control	post	fei1	Object	Narrow	r1	178.1472892	4	1	post_focus	1	Narrow post_focus
**2023408**	block1	Control	post	gei1	Object	Narrow	r1	235.8211856	5	2	post_focus	1	Narrow post_focus
**2023408**	block1	Control	post	zoeng1	Subject	Contrastive	r1	252.2157434	1	1	pre_focus	1	Contrastive pre_focus
**2023408**	block1	Control	post	saang1	Subject	Contrastive	r1	250.4182414	2	2	pre_focus	1	Contrastive pre_focus
**2023408**	block1	Control	post	tsa1	Verb	Contrastive	r1	213.1227729	3	1	on_focus	1	Contrastive on_focus
**2023408**	block1	Control	post	fei1	Object	Contrastive	r1	212.5192744	4	1	post_focus	1	Contrastive post_focus
**2023408**	block1	Control	post	gei1	Object	Contrastive	r1	244.584735	5	2	post_focus	1	Contrastive post_focus
**2023408**	block1	Control	post	jyun2	Subject	Narrow	r1	250.2380952	1	1	pre_focus	2	Narrow pre_focus
**2023408**	block1	Control	post	jyun2	Subject	Narrow	r1	281.8075082	2	2	pre_focus	2	Narrow pre_focus
**2023408**	block1	Control	post	mo2	Verb	Narrow	r1	163.3786848	3	1	on_focus	2	Narrow on_focus
**2023408**	block1	Control	post	gau2	Object	Narrow	r1	129.5011338	4	1	post_focus	2	Narrow post_focus
**2023408**	block1	Control	post	zai2	Object	Narrow	r1	237.4279236	5	2	post_focus	2	Narrow post_focus
**2023408**	block1	Control	post	sau3	Subject	Contrastive	r1	123.6937831	1	1	pre_focus	3	Contrastive pre_focus
**2023408**	block1	Control	post	sau3	Subject	Contrastive	r1	148.9427151	2	2	pre_focus	3	Contrastive pre_focus
**2023408**	block1	Control	post	sik3	Verb	Contrastive	r1	92.81405896	3	1	pre_focus	3	Contrastive pre_focus
**2023408**	block1	Control	post	baak3	Object	Contrastive	r1	121.9677501	4	1	on_focus	3	Contrastive on_focus
**2023408**	block1	Control	post	baak3	Object	Contrastive	r1	126.2183673	5	2	on_focus	3	Contrastive on_focus
**2023408**	block1	Control	post	sau3	Subject	Narrow	r1	146.5694849	1	1	pre_focus	3	Narrow pre_focus
**2023408**	block1	Control	post	sau3	Subject	Narrow	r1	174.5442177	2	2	pre_focus	3	Narrow pre_focus
**2023408**	block1	Control	post	sik3	Verb	Narrow	r1	125.8654573	3	1	pre_focus	3	Narrow pre_focus
**2023408**	block1	Control	post	baak3	Object	Narrow	r1	96.49659864	4	1	on_focus	3	Narrow on_focus
**2023408**	block1	Control	post	baak3	Object	Narrow	r1	55.94671202	5	2	on_focus	3	Narrow on_focus
**2023408**	block1	Control	post	zoeng1	Subject	Narrow	r1	205.085034	1	1	pre_focus	1	Narrow pre_focus
**2023408**	block1	Control	post	saang1	Subject	Narrow	r1	176.4777022	2	2	pre_focus	1	Narrow pre_focus
**2023408**	block1	Control	post	tsa1	Verb	Narrow	r1	133.6946334	3	1	pre_focus	1	Narrow pre_focus
**2023408**	block1	Control	post	fei1	Object	Narrow	r1	146.9193392	4	1	on_focus	1	Narrow on_focus
**2023408**	block1	Control	post	gei1	Object	Narrow	r1	147.2691934	5	2	on_focus	1	Narrow on_focus
**2023408**	block1	Control	post	jyun2	Subject	Contrastive	r1	181.2877929	1	1	pre_focus	2	Contrastive pre_focus
**2023408**	block1	Control	post	jyun2	Subject	Contrastive	r1	207.5503725	2	2	pre_focus	2	Contrastive pre_focus
**2023408**	block1	Control	post	mo2	Verb	Contrastive	r1	148.9514341	3	1	pre_focus	2	Contrastive pre_focus
**2023408**	block1	Control	post	gau2	Object	Contrastive	r1	138.2653061	4	1	on_focus	2	Contrastive on_focus
**2023408**	block1	Control	post	zai2	Object	Contrastive	r1	142.9387755	5	2	on_focus	2	Contrastive on_focus
**2023408**	block1	Control	post	sau3	Subject	Broad	r1	117.8004535	1	1	broad_focus	3	Broad focus
**2023408**	block1	Control	post	sau3	Subject	Broad	r1	153.8359788	2	2	broad_focus	3	Broad focus
**2023408**	block1	Control	post	sik3	Verb	Broad	r1	99.06349206	3	1	broad_focus	3	Broad focus
**2023408**	block1	Control	post	baak3	Object	Broad	r1	103.8435374	4	1	broad_focus	3	Broad focus
**2023408**	block1	Control	post	baak3	Object	Broad	r1	47.98469388	5	2	broad_focus	3	Broad focus
**2023408**	block1	Control	post	zoeng1	Subject	Contrastive	r1	153.7052154	1	1	pre_focus	1	Contrastive pre_focus
**2023408**	block1	Control	post	saang1	Subject	Contrastive	r1	241.5724004	2	2	pre_focus	1	Contrastive pre_focus
**2023408**	block1	Control	post	tsa1	Verb	Contrastive	r1	168.4965986	3	1	pre_focus	1	Contrastive pre_focus
**2023408**	block1	Control	post	fei1	Object	Contrastive	r1	192.7532124	4	1	on_focus	1	Contrastive on_focus
**2023408**	block1	Control	post	gei1	Object	Contrastive	r1	181.9229025	5	2	on_focus	1	Contrastive on_focus
**2023408**	block1	Control	post	sau3	Subject	Narrow	r1	133.5600907	1	1	pre_focus	3	Narrow pre_focus
**2023408**	block1	Control	post	sau3	Subject	Narrow	r1	206.0471331	2	2	pre_focus	3	Narrow pre_focus
**2023408**	block1	Control	post	sik3	Verb	Narrow	r1	110.1638837	3	1	on_focus	3	Narrow on_focus
**2023408**	block1	Control	post	baak3	Object	Narrow	r1	108.442642	4	1	post_focus	3	Narrow post_focus
**2023408**	block1	Control	post	baak3	Object	Narrow	r1	88.71396178	5	2	post_focus	3	Narrow post_focus
**2023408**	block1	Control	post	jyun2	Subject	Contrastive	r1	244.6872638	1	1	pre_focus	2	Contrastive pre_focus
**2023408**	block1	Control	post	jyun2	Subject	Contrastive	r1	230.7655526	2	2	pre_focus	2	Contrastive pre_focus
**2023408**	block1	Control	post	mo2	Verb	Contrastive	r1	147.2315666	3	1	on_focus	2	Contrastive on_focus
**2023408**	block1	Control	post	gau2	Object	Contrastive	r1	158.4761905	4	1	post_focus	2	Contrastive post_focus
**2023408**	block1	Control	post	zai2	Object	Contrastive	r1	185.4667423	5	2	post_focus	2	Contrastive post_focus

**2023408**	block1	Control	post	jyun2	Subject	Narrow	r2	194.3643235	1	1	pre_focus	2	Narrow pre_focus
**2023408**	block1	Control	post	jyun2	Subject	Narrow	r2	201.8633787	2	2	pre_focus	2	Narrow pre_focus
**2023408**	block1	Control	post	mo2	Verb	Narrow	r2	135.8621819	3	1	on_focus	2	Narrow on_focus
**2023408**	block1	Control	post	gau2	Object	Narrow	r2	142.8412698	4	1	post_focus	2	Narrow post_focus
**2023408**	block1	Control	post	zai2	Object	Narrow	r2	191.7532027	5	2	post_focus	2	Narrow post_focus
**2023408**	block1	Control	post	zoeng1	Subject	Contrastive	r2	173.7346939	1	1	pre_focus	1	Contrastive pre_focus
**2023408**	block1	Control	post	saang1	Subject	Contrastive	r2	184.1005291	2	2	pre_focus	1	Contrastive pre_focus
**2023408**	block1	Control	post	tsa1	Verb	Contrastive	r2	176.9765684	3	1	on_focus	1	Contrastive on_focus
**2023408**	block1	Control	post	fei1	Object	Contrastive	r2	153.6079617	4	1	post_focus	1	Contrastive post_focus
**2023408**	block1	Control	post	gei1	Object	Contrastive	r2	162.7503053	5	2	post_focus	1	Contrastive post_focus
**2023408**	block1	Control	post	sau3	Subject	Contrastive	r2	149.3866528	1	1	pre_focus	3	Contrastive pre_focus
**2023408**	block1	Control	post	sau3	Subject	Contrastive	r2	144.8962919	2	2	pre_focus	3	Contrastive pre_focus
**2023408**	block1	Control	post	sik3	Verb	Contrastive	r2	94.17311084	3	1	pre_focus	3	Contrastive pre_focus
**2023408**	block1	Control	post	baak3	Object	Contrastive	r2	102.6280851	4	1	on_focus	3	Contrastive on_focus
**2023408**	block1	Control	post	baak3	Object	Contrastive	r2	118.6470144	5	2	on_focus	3	Contrastive on_focus
**2023408**	block1	Control	post	sau3	Subject	Contrastive	r2	116.0119048	1	1	pre_focus	3	Contrastive pre_focus
**2023408**	block1	Control	post	sau3	Subject	Contrastive	r2	131.056263	2	2	pre_focus	3	Contrastive pre_focus
**2023408**	block1	Control	post	sik3	Verb	Contrastive	r2	109.5408163	3	1	on_focus	3	Contrastive on_focus
**2023408**	block1	Control	post	baak3	Object	Contrastive	r2	89.62207105	4	1	post_focus	3	Contrastive post_focus
**2023408**	block1	Control	post	baak3	Object	Contrastive	r2	90.73582766	5	2	post_focus	3	Contrastive post_focus
**2023408**	block1	Control	post	sau3	Subject	Contrastive	r2	107.1468164	1	1	on_focus	3	Contrastive on_focus
**2023408**	block1	Control	post	sau3	Subject	Contrastive	r2	97.87981859	2	2	on_focus	3	Contrastive on_focus
**2023408**	block1	Control	post	sik3	Verb	Contrastive	r2	87.07383112	3	1	post_focus	3	Contrastive post_focus
**2023408**	block1	Control	post	baak3	Object	Contrastive	r2	88.59410431	4	1	post_focus	3	Contrastive post_focus
**2023408**	block1	Control	post	baak3	Object	Contrastive	r2	64.78911565	5	2	post_focus	3	Contrastive post_focus
**2023408**	block1	Control	post	zoeng1	Subject	Contrastive	r2	161.4961937	1	1	pre_focus	1	Contrastive pre_focus
**2023408**	block1	Control	post	saang1	Subject	Contrastive	r2	187.5245654	2	2	pre_focus	1	Contrastive pre_focus
**2023408**	block1	Control	post	tsa1	Verb	Contrastive	r2	121.5345805	3	1	pre_focus	1	Contrastive pre_focus
**2023408**	block1	Control	post	fei1	Object	Contrastive	r2	161.3973923	4	1	on_focus	1	Contrastive on_focus
**2023408**	block1	Control	post	gei1	Object	Contrastive	r2	119.4042465	5	2	on_focus	1	Contrastive on_focus
**2023408**	block1	Control	post	jyun2	Subject	Narrow	r2	187.9039373	1	1	pre_focus	2	Narrow pre_focus
**2023408**	block1	Control	post	jyun2	Subject	Narrow	r2	188.1273621	2	2	pre_focus	2	Narrow pre_focus
**2023408**	block1	Control	post	mo2	Verb	Narrow	r2	145.4081633	3	1	pre_focus	2	Narrow pre_focus
**2023408**	block1	Control	post	gau2	Object	Narrow	r2	114.3159486	4	1	on_focus	2	Narrow on_focus
**2023408**	block1	Control	post	zai2	Object	Narrow	r2	251.2693374	5	2	on_focus	2	Narrow on_focus
**2023408**	block1	Control	post	sau3	Subject	Narrow	r2	106.30345	1	1	pre_focus	3	Narrow pre_focus
**2023408**	block1	Control	post	sau3	Subject	Narrow	r2	122.8713152	2	2	pre_focus	3	Narrow pre_focus
**2023408**	block1	Control	post	sik3	Verb	Narrow	r2	89.82867221	3	1	pre_focus	3	Narrow pre_focus
**2023408**	block1	Control	post	baak3	Object	Narrow	r2	77.46107332	4	1	on_focus	3	Narrow on_focus
**2023408**	block1	Control	post	baak3	Object	Narrow	r2	70.47619048	5	2	on_focus	3	Narrow on_focus
**2023408**	block1	Control	post	jyun2	Subject	Contrastive	r2	224.7773655	1	1	pre_focus	2	Contrastive pre_focus
**2023408**	block1	Control	post	jyun2	Subject	Contrastive	r2	187.4991902	2	2	pre_focus	2	Contrastive pre_focus
**2023408**	block1	Control	post	mo2	Verb	Contrastive	r2	123.5147392	3	1	pre_focus	2	Contrastive pre_focus
**2023408**	block1	Control	post	gau2	Object	Contrastive	r2	120.8057985	4	1	on_focus	2	Contrastive on_focus
**2023408**	block1	Control	post	zai2	Object	Contrastive	r2	184.9077853	5	2	on_focus	2	Contrastive on_focus
**2023408**	block1	Control	post	zoeng1	Subject	Narrow	r2	164.5144963	1	1	pre_focus	1	Narrow pre_focus
**2023408**	block1	Control	post	saang1	Subject	Narrow	r2	180.547997	2	2	pre_focus	1	Narrow pre_focus
**2023408**	block1	Control	post	tsa1	Verb	Narrow	r2	115.5623583	3	1	pre_focus	1	Narrow pre_focus
**2023408**	block1	Control	post	fei1	Object	Narrow	r2	128.2199546	4	1	on_focus	1	Narrow on_focus
**2023408**	block1	Control	post	gei1	Object	Narrow	r2	125.5180146	5	2	on_focus	1	Narrow on_focus
**2023408**	block1	Control	post	sau3	Subject	Broad	r2	129.1080877	1	1	broad_focus	3	Broad focus
**2023408**	block1	Control	post	sau3	Subject	Broad	r2	153.6406926	2	2	broad_focus	3	Broad focus
**2023408**	block1	Control	post	sik3	Verb	Broad	r2	79.4739229	3	1	broad_focus	3	Broad focus
**2023408**	block1	Control	post	baak3	Object	Broad	r2	82.8239391	4	1	broad_focus	3	Broad focus
**2023408**	block1	Control	post	baak3	Object	Broad	r2	71.07407407	5	2	broad_focus	3	Broad focus
**2023408**	block1	Control	post	zoeng1	Subject	Narrow	r2	160.5498866	1	1	on_focus	1	Narrow on_focus
**2023408**	block1	Control	post	saang1	Subject	Narrow	r2	196.2029478	2	2	on_focus	1	Narrow on_focus
**2023408**	block1	Control	post	tsa1	Verb	Narrow	r2	134.7885488	3	1	post_focus	1	Narrow post_focus
**2023408**	block1	Control	post	fei1	Object	Narrow	r2	165.4848289	4	1	post_focus	1	Narrow post_focus
**2023408**	block1	Control	post	gei1	Object	Narrow	r2	229.914966	5	2	post_focus	1	Narrow post_focus
**2023408**	block1	Control	post	jyun2	Subject	Contrastive	r2	219.3968254	1	1	on_focus	2	Contrastive on_focus
**2023408**	block1	Control	post	jyun2	Subject	Contrastive	r2	236.9387755	2	2	on_focus	2	Contrastive on_focus
**2023408**	block1	Control	post	mo2	Verb	Contrastive	r2	154.457672	3	1	post_focus	2	Contrastive post_focus
**2023408**	block1	Control	post	gau2	Object	Contrastive	r2	157.8150668	4	1	post_focus	2	Contrastive post_focus
**2023408**	block1	Control	post	zai2	Object	Contrastive	r2	243.7220603	5	2	post_focus	2	Contrastive post_focus
**2023408**	block1	Control	post	jyun2	Subject	Contrastive	r2	245.4036281	1	1	pre_focus	2	Contrastive pre_focus

**2023408**	block1	Control	post	jyun2	Subject	Contrastive	r2	229.3323255	2	2	pre_focus	2	Contrastive pre_focus
**2023408**	block1	Control	post	mo2	Verb	Contrastive	r2	170.7709751	3	1	on_focus	2	Contrastive on_focus
**2023408**	block1	Control	post	gau2	Object	Contrastive	r2	149.6054422	4	1	post_focus	2	Contrastive post_focus
**2023408**	block1	Control	post	zai2	Object	Contrastive	r2	182.3051506	5	2	post_focus	2	Contrastive post_focus
**2023408**	block1	Control	post	zoeng1	Subject	Contrastive	r2	210.9297052	1	1	on_focus	1	Contrastive on_focus
**2023408**	block1	Control	post	saang1	Subject	Contrastive	r2	221.1133583	2	2	on_focus	1	Contrastive on_focus
**2023408**	block1	Control	post	tsa1	Verb	Contrastive	r2	136.9909297	3	1	post_focus	1	Contrastive post_focus
**2023408**	block1	Control	post	fei1	Object	Contrastive	r2	173.3994709	4	1	post_focus	1	Contrastive post_focus
**2023408**	block1	Control	post	gei1	Object	Contrastive	r2	224.1883513	5	2	post_focus	1	Contrastive post_focus
**2023408**	block1	Control	post	zoeng1	Subject	Broad	r2	172.1394558	1	1	broad_focus	1	Broad focus
**2023408**	block1	Control	post	saang1	Subject	Broad	r2	196.4865205	2	2	broad_focus	1	Broad focus
**2023408**	block1	Control	post	tsa1	Verb	Broad	r2	137.8760393	3	1	broad_focus	1	Broad focus
**2023408**	block1	Control	post	fei1	Object	Broad	r2	188.2296728	4	1	broad_focus	1	Broad focus
**2023408**	block1	Control	post	gei1	Object	Broad	r2	174.0136054	5	2	broad_focus	1	Broad focus
**2023408**	block1	Control	post	sau3	Subject	Narrow	r2	110.7515387	1	1	on_focus	3	Narrow on_focus
**2023408**	block1	Control	post	sau3	Subject	Narrow	r2	138.0699708	2	2	on_focus	3	Narrow on_focus
**2023408**	block1	Control	post	sik3	Verb	Narrow	r2	69.34996221	3	1	post_focus	3	Narrow post_focus
**2023408**	block1	Control	post	baak3	Object	Narrow	r2	91.09977324	4	1	post_focus	3	Narrow post_focus
**2023408**	block1	Control	post	baak3	Object	Narrow	r2	76.18795666	5	2	post_focus	3	Narrow post_focus
**2023408**	block1	Control	post	jyun2	Subject	Broad	r2	146.0695389	1	1	broad_focus	2	Broad focus
**2023408**	block1	Control	post	jyun2	Subject	Broad	r2	176.9349962	2	2	broad_focus	2	Broad focus
**2023408**	block1	Control	post	mo2	Verb	Broad	r2	134.2444121	3	1	broad_focus	2	Broad focus
**2023408**	block1	Control	post	gau2	Object	Broad	r2	140.7684555	4	1	broad_focus	2	Broad focus
**2023408**	block1	Control	post	zai2	Object	Broad	r2	112.3431595	5	2	broad_focus	2	Broad focus
**2023408**	block1	Control	post	jyun2	Subject	Narrow	r2	184.2426304	1	1	on_focus	2	Narrow on_focus
**2023408**	block1	Control	post	jyun2	Subject	Narrow	r2	176.183063	2	2	on_focus	2	Narrow on_focus
**2023408**	block1	Control	post	mo2	Verb	Narrow	r2	139.0221088	3	1	post_focus	2	Narrow post_focus
**2023408**	block1	Control	post	gau2	Object	Narrow	r2	118.0668934	4	1	post_focus	2	Narrow post_focus
**2023408**	block1	Control	post	zai2	Object	Narrow	r2	219.5804989	5	2	post_focus	2	Narrow post_focus
**2023408**	block1	Control	post	zoeng1	Subject	Narrow	r2	150.402756	1	1	pre_focus	1	Narrow pre_focus
**2023408**	block1	Control	post	saang1	Subject	Narrow	r2	213.7131519	2	2	pre_focus	1	Narrow pre_focus
**2023408**	block1	Control	post	tsa1	Verb	Narrow	r2	162.5729403	3	1	on_focus	1	Narrow on_focus
**2023408**	block1	Control	post	fei1	Object	Narrow	r2	166.8480726	4	1	post_focus	1	Narrow post_focus
**2023408**	block1	Control	post	gei1	Object	Narrow	r2	236.1211892	5	2	post_focus	1	Narrow post_focus
**2023408**	block1	Control	post	sau3	Subject	Narrow	r2	125.606576	1	1	pre_focus	3	Narrow pre_focus
**2023408**	block1	Control	post	sau3	Subject	Narrow	r2	140.0020614	2	2	pre_focus	3	Narrow pre_focus
**2023408**	block1	Control	post	sik3	Verb	Narrow	r2	81.74489796	3	1	on_focus	3	Narrow on_focus
**2023408**	block1	Control	post	baak3	Object	Narrow	r2	105.8730159	4	1	post_focus	3	Narrow post_focus
**2023408**	block1	Control	post	baak3	Object	Narrow	r2	170.8755354	5	2	post_focus	3	Narrow post_focus
**2023408**	block1	Control	pre	sau3	Subject	Contrastive	r1	244.0411403	1	1	on_focus	3	Contrastive on_focus
**2023408**	block1	Control	pre	sau3	Subject	Contrastive	r1	178.2698811	2	2	on_focus	3	Contrastive on_focus
**2023408**	block1	Control	pre	sik3	Verb	Contrastive	r1	78.43704499	3	1	post_focus	3	Contrastive post_focus
**2023408**	block1	Control	pre	baak3	Object	Contrastive	r1	78.56991686	4	1	post_focus	3	Contrastive post_focus
**2023408**	block1	Control	pre	baak3	Object	Contrastive	r1	63.77000324	5	2	post_focus	3	Contrastive post_focus
**2023408**	block1	Control	pre	jyun2	Subject	Contrastive	r1	358.1857836	1	1	on_focus	2	Contrastive on_focus
**2023408**	block1	Control	pre	jyun2	Subject	Contrastive	r1	254.4236266	2	2	on_focus	2	Contrastive on_focus
**2023408**	block1	Control	pre	mo2	Verb	Contrastive	r1	242.7829227	3	1	post_focus	2	Contrastive post_focus
**2023408**	block1	Control	pre	gau2	Object	Contrastive	r1	169.5820616	4	1	post_focus	2	Contrastive post_focus
**2023408**	block1	Control	pre	zai2	Object	Contrastive	r1	322.6417234	5	2	post_focus	2	Contrastive post_focus
**2023408**	block1	Control	pre	zoeng1	Subject	Broad	r1	226.7146097	1	1	broad_focus	1	Broad focus
**2023408**	block1	Control	pre	saang1	Subject	Broad	r1	260.4380197	2	2	broad_focus	1	Broad focus
**2023408**	block1	Control	pre	tsa1	Verb	Broad	r1	200.6099133	3	1	broad_focus	1	Broad focus
**2023408**	block1	Control	pre	fei1	Object	Broad	r1	192.9304029	4	1	broad_focus	1	Broad focus
**2023408**	block1	Control	pre	gei1	Object	Broad	r1	181.8153735	5	2	broad_focus	1	Broad focus
**2023408**	block1	Control	pre	jyun2	Subject	Contrastive	r1	166.0103383	1	1	pre_focus	2	Contrastive pre_focus
**2023408**	block1	Control	pre	jyun2	Subject	Contrastive	r1	239.0322899	2	2	pre_focus	2	Contrastive pre_focus
**2023408**	block1	Control	pre	mo2	Verb	Contrastive	r1	186.0705335	3	1	pre_focus	2	Contrastive pre_focus
**2023408**	block1	Control	pre	gau2	Object	Contrastive	r1	159.5790453	4	1	on_focus	2	Contrastive on_focus
**2023408**	block1	Control	pre	zai2	Object	Contrastive	r1	236.5684051	5	2	on_focus	2	Contrastive on_focus
**2023408**	block1	Control	pre	sau3	Subject	Narrow	r1	166.4886534	1	1	pre_focus	3	Narrow pre_focus
**2023408**	block1	Control	pre	sau3	Subject	Narrow	r1	145.8391108	2	2	pre_focus	3	Narrow pre_focus
**2023408**	block1	Control	pre	sik3	Verb	Narrow	r1	118.6228269	3	1	pre_focus	3	Narrow pre_focus
**2023408**	block1	Control	pre	baak3	Object	Narrow	r1	96.10355253	4	1	on_focus	3	Narrow on_focus
**2023408**	block1	Control	pre	baak3	Object	Narrow	r1	100.4509637	5	2	on_focus	3	Narrow on_focus
**2023408**	block1	Control	pre	sau3	Subject	Narrow	r1	154.2484613	1	1	on_focus	3	Narrow on_focus
**2023408**	block1	Control	pre	sau3	Subject	Narrow	r1	164.999783	2	2	on_focus	3	Narrow on_focus

**2023408**	block1	Control	pre	sik3	Verb	Narrow	r1	94.25413022	3	1	post_focus	3	Narrow post_focus
**2023408**	block1	Control	pre	baak3	Object	Narrow	r1	106.2296384	4	1	post_focus	3	Narrow post_focus
**2023408**	block1	Control	pre	baak3	Object	Narrow	r1	101.9501134	5	2	post_focus	3	Narrow post_focus
**2023408**	block1	Control	pre	jyun2	Subject	Narrow	r1	198.8280963	1	1	pre_focus	2	Narrow pre_focus
**2023408**	block1	Control	pre	jyun2	Subject	Narrow	r1	143.4884123	2	2	pre_focus	2	Narrow pre_focus
**2023408**	block1	Control	pre	mo2	Verb	Narrow	r1	197.6287658	3	1	pre_focus	2	Narrow pre_focus
**2023408**	block1	Control	pre	gau2	Object	Narrow	r1	168.957902	4	1	on_focus	2	Narrow on_focus
**2023408**	block1	Control	pre	zai2	Object	Narrow	r1	168.7296548	5	2	on_focus	2	Narrow on_focus
**2023408**	block1	Control	pre	jyun2	Subject	Narrow	r1	198.4846266	1	1	on_focus	2	Narrow on_focus
**2023408**	block1	Control	pre	jyun2	Subject	Narrow	r1	130.0262515	2	2	on_focus	2	Narrow on_focus
**2023408**	block1	Control	pre	mo2	Verb	Narrow	r1	203.3192909	3	1	post_focus	2	Narrow post_focus
**2023408**	block1	Control	pre	gau2	Object	Narrow	r1	151.303225	4	1	post_focus	2	Narrow post_focus
**2023408**	block1	Control	pre	zai2	Object	Narrow	r1	215.546071	5	2	post_focus	2	Narrow post_focus
**2023408**	block1	Control	pre	zoeng1	Subject	Contrastive	r1	150.1565421	1	1	pre_focus	1	Contrastive pre_focus
**2023408**	block1	Control	pre	saang1	Subject	Contrastive	r1	237.0309757	2	2	pre_focus	1	Contrastive pre_focus
**2023408**	block1	Control	pre	tsa1	Verb	Contrastive	r1	165.8647959	3	1	pre_focus	1	Contrastive pre_focus
**2023408**	block1	Control	pre	fei1	Object	Contrastive	r1	221.5627362	4	1	on_focus	1	Contrastive on_focus
**2023408**	block1	Control	pre	gei1	Object	Contrastive	r1	198.4949789	5	2	on_focus	1	Contrastive on_focus
**2023408**	block1	Control	pre	zoeng1	Subject	Narrow	r1	174.9706535	1	1	pre_focus	1	Narrow pre_focus
**2023408**	block1	Control	pre	saang1	Subject	Narrow	r1	209.4978944	2	2	pre_focus	1	Narrow pre_focus
**2023408**	block1	Control	pre	tsa1	Verb	Narrow	r1	136.9361078	3	1	pre_focus	1	Narrow pre_focus
**2023408**	block1	Control	pre	fei1	Object	Narrow	r1	151.2734856	4	1	on_focus	1	Narrow on_focus
**2023408**	block1	Control	pre	gei1	Object	Narrow	r1	135.8178382	5	2	on_focus	1	Narrow on_focus
**2023408**	block1	Control	pre	jyun2	Subject	Broad	r1	220.7678413	1	1	broad_focus	2	Broad focus
**2023408**	block1	Control	pre	jyun2	Subject	Broad	r1	271.032239	2	2	broad_focus	2	Broad focus
**2023408**	block1	Control	pre	mo2	Verb	Broad	r1	169.1886755	3	1	broad_focus	2	Broad focus
**2023408**	block1	Control	pre	gau2	Object	Broad	r1	172.7923315	4	1	broad_focus	2	Broad focus
**2023408**	block1	Control	pre	zai2	Object	Broad	r1	170.3401361	5	2	broad_focus	2	Broad focus
**2023408**	block1	Control	pre	sau3	Subject	Narrow	r1	134.5584461	1	1	pre_focus	3	Narrow pre_focus
**2023408**	block1	Control	pre	sau3	Subject	Narrow	r1	138.0767736	2	2	pre_focus	3	Narrow pre_focus
**2023408**	block1	Control	pre	sik3	Verb	Narrow	r1	98.44278737	3	1	on_focus	3	Narrow on_focus
**2023408**	block1	Control	pre	baak3	Object	Narrow	r1	74.67372134	4	1	post_focus	3	Narrow post_focus
**2023408**	block1	Control	pre	baak3	Object	Narrow	r1	100.1710128	5	2	post_focus	3	Narrow post_focus
**2023408**	block1	Control	pre	sau3	Subject	Contrastive	r1	140.153788	1	1	pre_focus	3	Contrastive pre_focus
**2023408**	block1	Control	pre	sau3	Subject	Contrastive	r1	136.3739435	2	2	pre_focus	3	Contrastive pre_focus
**2023408**	block1	Control	pre	sik3	Verb	Contrastive	r1	101.1750155	3	1	on_focus	3	Contrastive on_focus
**2023408**	block1	Control	pre	baak3	Object	Contrastive	r1	100.3764172	4	1	post_focus	3	Contrastive post_focus
**2023408**	block1	Control	pre	baak3	Object	Contrastive	r1	47.60916748	5	2	post_focus	3	Contrastive post_focus
**2023408**	block1	Control	pre	zoeng1	Subject	Narrow	r1	164.860688	1	1	pre_focus	1	Narrow pre_focus
**2023408**	block1	Control	pre	saang1	Subject	Narrow	r1	204.1204649	2	2	pre_focus	1	Narrow pre_focus
**2023408**	block1	Control	pre	tsa1	Verb	Narrow	r1	164.4038668	3	1	on_focus	1	Narrow on_focus
**2023408**	block1	Control	pre	fei1	Object	Narrow	r1	180.4944334	4	1	post_focus	1	Narrow post_focus
**2023408**	block1	Control	pre	gei1	Object	Narrow	r1	189.9886621	5	2	post_focus	1	Narrow post_focus
**2023408**	block1	Control	pre	jyun2	Subject	Narrow	r1	167.7244898	1	1	pre_focus	2	Narrow pre_focus
**2023408**	block1	Control	pre	jyun2	Subject	Narrow	r1	226.2038593	2	2	pre_focus	2	Narrow pre_focus
**2023408**	block1	Control	pre	mo2	Verb	Narrow	r1	167.617104	3	1	on_focus	2	Narrow on_focus
**2023408**	block1	Control	pre	gau2	Object	Narrow	r1	142.0914588	4	1	post_focus	2	Narrow post_focus
**2023408**	block1	Control	pre	zai2	Object	Narrow	r1	83.97051361	5	2	post_focus	2	Narrow post_focus
**2023408**	block1	Control	pre	sau3	Subject	Broad	r1	115.8463089	1	1	broad_focus	3	Broad focus
**2023408**	block1	Control	pre	sau3	Subject	Broad	r1	128.2549761	2	2	broad_focus	3	Broad focus
**2023408**	block1	Control	pre	sik3	Verb	Broad	r1	81.02406748	3	1	broad_focus	3	Broad focus
**2023408**	block1	Control	pre	baak3	Object	Broad	r1	108.6874871	4	1	broad_focus	3	Broad focus
**2023408**	block1	Control	pre	baak3	Object	Broad	r1	88.4224801	5	2	broad_focus	3	Broad focus
**2023408**	block1	Control	pre	jyun2	Subject	Contrastive	r1	193.5181929	1	1	pre_focus	2	Contrastive pre_focus
**2023408**	block1	Control	pre	jyun2	Subject	Contrastive	r1	222.2716737	2	2	pre_focus	2	Contrastive pre_focus
**2023408**	block1	Control	pre	mo2	Verb	Contrastive	r1	146.8642695	3	1	on_focus	2	Contrastive on_focus
**2023408**	block1	Control	pre	gau2	Object	Contrastive	r1	180.5245432	4	1	post_focus	2	Contrastive post_focus
**2023408**	block1	Control	pre	zai2	Object	Contrastive	r1	217.1989369	5	2	post_focus	2	Contrastive post_focus
**2023408**	block1	Control	pre	zoeng1	Subject	Narrow	r1	196.8178382	1	1	on_focus	1	Narrow on_focus
**2023408**	block1	Control	pre	saang1	Subject	Narrow	r1	205.202898	2	2	on_focus	1	Narrow on_focus
**2023408**	block1	Control	pre	tsa1	Verb	Narrow	r1	142.6827141	3	1	post_focus	1	Narrow post_focus
**2023408**	block1	Control	pre	fei1	Object	Narrow	r1	160.3756957	4	1	post_focus	1	Narrow post_focus
**2023408**	block1	Control	pre	gei1	Object	Narrow	r1	299.3689553	5	2	post_focus	1	Narrow post_focus
**2023408**	block1	Control	pre	zoeng1	Subject	Contrastive	r1	174.0712396	1	1	pre_focus	1	Contrastive pre_focus
**2023408**	block1	Control	pre	saang1	Subject	Contrastive	r1	221.9965446	2	2	pre_focus	1	Contrastive pre_focus
**2023408**	block1	Control	pre	tsa1	Verb	Contrastive	r1	156.8707483	3	1	on_focus	1	Contrastive on_focus

**2023408**	block1	Control	pre	fei1	Object	Contrastive	r1	147.016161	4	1	post_focus	1	Contrastive post_focus
**2023408**	block1	Control	pre	gei1	Object	Contrastive	r1	146.0238868	5	2	post_focus	1	Contrastive post_focus
**2023408**	block1	Control	pre	sau3	Subject	Contrastive	r1	116.6985579	1	1	pre_focus	3	Contrastive pre_focus
**2023408**	block1	Control	pre	sau3	Subject	Contrastive	r1	128.8885753	2	2	pre_focus	3	Contrastive pre_focus
**2023408**	block1	Control	pre	sik3	Verb	Contrastive	r1	66.97354497	3	1	pre_focus	3	Contrastive pre_focus
**2023408**	block1	Control	pre	baak3	Object	Contrastive	r1	95.10616368	4	1	on_focus	3	Contrastive on_focus
**2023408**	block1	Control	pre	baak3	Object	Contrastive	r1	84.38964475	5	2	on_focus	3	Contrastive on_focus
**2023408**	block1	Control	pre	zoeng1	Subject	Contrastive	r1	127.9700893	1	1	on_focus	1	Contrastive on_focus
**2023408**	block1	Control	pre	saang1	Subject	Contrastive	r1	213.6963771	2	2	on_focus	1	Contrastive on_focus
**2023408**	block1	Control	pre	tsa1	Verb	Contrastive	r1	143.8421202	3	1	post_focus	1	Contrastive post_focus
**2023408**	block1	Control	pre	fei1	Object	Contrastive	r1	181.820793	4	1	post_focus	1	Contrastive post_focus
**2023408**	block1	Control	pre	gei1	Object	Contrastive	r1	249.3213804	5	2	post_focus	1	Contrastive post_focus
**2023408**	block1	Control	pre	zoeng1	Subject	Broad	r2	185.4979214	1	1	broad_focus	1	Broad focus
**2023408**	block1	Control	pre	saang1	Subject	Broad	r2	243.9136958	2	2	broad_focus	1	Broad focus
**2023408**	block1	Control	pre	tsa1	Verb	Broad	r2	125.4898689	3	1	broad_focus	1	Broad focus
**2023408**	block1	Control	pre	fei1	Object	Broad	r2	163.9805637	4	1	broad_focus	1	Broad focus
**2023408**	block1	Control	pre	gei1	Object	Broad	r2	213.8800705	5	2	broad_focus	1	Broad focus
**2023408**	block1	Control	pre	zoeng1	Subject	Narrow	r2	140.1214772	1	1	pre_focus	1	Narrow pre_focus
**2023408**	block1	Control	pre	saang1	Subject	Narrow	r2	185.7131519	2	2	pre_focus	1	Narrow pre_focus
**2023408**	block1	Control	pre	tsa1	Verb	Narrow	r2	111.5262931	3	1	pre_focus	1	Narrow pre_focus
**2023408**	block1	Control	pre	fei1	Object	Narrow	r2	131.0251323	4	1	on_focus	1	Narrow on_focus
**2023408**	block1	Control	pre	gei1	Object	Narrow	r2	173.7743373	5	2	on_focus	1	Narrow on_focus
**2023408**	block1	Control	pre	jyun2	Subject	Narrow	r2	174.4901448	1	1	pre_focus	2	Narrow pre_focus
**2023408**	block1	Control	pre	jyun2	Subject	Narrow	r2	204.3212479	2	2	pre_focus	2	Narrow pre_focus
**2023408**	block1	Control	pre	mo2	Verb	Narrow	r2	150.2039971	3	1	on_focus	2	Narrow on_focus
**2023408**	block1	Control	pre	gau2	Object	Narrow	r2	146.739103	4	1	post_focus	2	Narrow post_focus
**2023408**	block1	Control	pre	zai2	Object	Narrow	r2	264.4287459	5	2	post_focus	2	Narrow post_focus
**2023408**	block1	Control	pre	jyun2	Subject	Contrastive	r2	163.3506341	1	1	pre_focus	2	Contrastive pre_focus
**2023408**	block1	Control	pre	jyun2	Subject	Contrastive	r2	203.3331212	2	2	pre_focus	2	Contrastive pre_focus
**2023408**	block1	Control	pre	mo2	Verb	Contrastive	r2	166.4917706	3	1	on_focus	2	Contrastive on_focus
**2023408**	block1	Control	pre	gau2	Object	Contrastive	r2	112.3946142	4	1	post_focus	2	Contrastive post_focus
**2023408**	block1	Control	pre	zai2	Object	Contrastive	r2	221.2044305	5	2	post_focus	2	Contrastive post_focus
**2023408**	block1	Control	pre	jyun2	Subject	Narrow	r2	197.7917015	1	1	pre_focus	2	Narrow pre_focus
**2023408**	block1	Control	pre	jyun2	Subject	Narrow	r2	200.134767	2	2	pre_focus	2	Narrow pre_focus
**2023408**	block1	Control	pre	mo2	Verb	Narrow	r2	150.4913076	3	1	pre_focus	2	Narrow pre_focus
**2023408**	block1	Control	pre	gau2	Object	Narrow	r2	161.7938364	4	1	on_focus	2	Narrow on_focus
**2023408**	block1	Control	pre	zai2	Object	Narrow	r2	215.7974857	5	2	on_focus	2	Narrow on_focus
**2023408**	block1	Control	pre	sau3	Subject	Contrastive	r2	138.6587722	1	1	pre_focus	3	Contrastive pre_focus
**2023408**	block1	Control	pre	sau3	Subject	Contrastive	r2	149.0070848	2	2	pre_focus	3	Contrastive pre_focus
**2023408**	block1	Control	pre	sik3	Verb	Contrastive	r2	59.78042328	3	1	on_focus	3	Contrastive on_focus
**2023408**	block1	Control	pre	baak3	Object	Contrastive	r2	62.30839614	4	1	post_focus	3	Contrastive post_focus
**2023408**	block1	Control	pre	baak3	Object	Contrastive	r2	87.0218254	5	2	post_focus	3	Contrastive post_focus
**2023408**	block1	Control	pre	sau3	Subject	Narrow	r2	165.461007	1	1	on_focus	3	Narrow on_focus
**2023408**	block1	Control	pre	sau3	Subject	Narrow	r2	149.8787507	2	2	on_focus	3	Narrow on_focus
**2023408**	block1	Control	pre	sik3	Verb	Narrow	r2	92.97044839	3	1	post_focus	3	Narrow post_focus
**2023408**	block1	Control	pre	baak3	Object	Narrow	r2	86.60005669	4	1	post_focus	3	Narrow post_focus
**2023408**	block1	Control	pre	baak3	Object	Narrow	r2	138.852058	5	2	post_focus	3	Narrow post_focus
**2023408**	block1	Control	pre	sau3	Subject	Narrow	r2	126.8530147	1	1	pre_focus	3	Narrow pre_focus
**2023408**	block1	Control	pre	sau3	Subject	Narrow	r2	130.0589569	2	2	pre_focus	3	Narrow pre_focus
**2023408**	block1	Control	pre	sik3	Verb	Narrow	r2	81.34021892	3	1	pre_focus	3	Narrow pre_focus
**2023408**	block1	Control	pre	baak3	Object	Narrow	r2	76.51378181	4	1	on_focus	3	Narrow on_focus
**2023408**	block1	Control	pre	baak3	Object	Narrow	r2	73.89543966	5	2	on_focus	3	Narrow on_focus
**2023408**	block1	Control	pre	jyun2	Subject	Contrastive	r2	163.1762229	1	1	on_focus	2	Contrastive on_focus
**2023408**	block1	Control	pre	jyun2	Subject	Contrastive	r2	156.982486	2	2	on_focus	2	Contrastive on_focus
**2023408**	block1	Control	pre	mo2	Verb	Contrastive	r2	99.89136261	3	1	post_focus	2	Contrastive post_focus
**2023408**	block1	Control	pre	gau2	Object	Contrastive	r2	125.1423359	4	1	post_focus	2	Contrastive post_focus
**2023408**	block1	Control	pre	zai2	Object	Contrastive	r2	208.5235576	5	2	post_focus	2	Contrastive post_focus
**2023408**	block1	Control	pre	sau3	Subject	Narrow	r2	119.704504	1	1	pre_focus	3	Narrow pre_focus
**2023408**	block1	Control	pre	sau3	Subject	Narrow	r2	113.2525993	2	2	pre_focus	3	Narrow pre_focus
**2023408**	block1	Control	pre	sik3	Verb	Narrow	r2	114.3195651	3	1	on_focus	3	Narrow on_focus
**2023408**	block1	Control	pre	baak3	Object	Narrow	r2	111.3384652	4	1	post_focus	3	Narrow post_focus
**2023408**	block1	Control	pre	baak3	Object	Narrow	r2	75.24036281	5	2	post_focus	3	Narrow post_focus
**2023408**	block1	Control	pre	zoeng1	Subject	Contrastive	r2	147.7949564	1	1	pre_focus	1	Contrastive pre_focus
**2023408**	block1	Control	pre	saang1	Subject	Contrastive	r2	194.1876491	2	2	pre_focus	1	Contrastive pre_focus
**2023408**	block1	Control	pre	tsa1	Verb	Contrastive	r2	140.0153871	3	1	pre_focus	1	Contrastive pre_focus
**2023408**	block1	Control	pre	fei1	Object	Contrastive	r2	147.915128	4	1	on_focus	1	Contrastive on_focus

2023408	block1	Control	pre	gei1	Object	Contrastive	r2	167.1427372	5	2	on_focus	1	Contrastive on_focus
2023408	block1	Control	pre	zoeng1	Subject	Contrastive	r2	148.2311931	1	1	pre_focus	1	Contrastive pre_focus
2023408	block1	Control	pre	saang1	Subject	Contrastive	r2	178.9701436	2	2	pre_focus	1	Contrastive pre_focus
2023408	block1	Control	pre	tsa1	Verb	Contrastive	r2	131.3654033	3	1	on_focus	1	Contrastive on_focus
2023408	block1	Control	pre	fei1	Object	Contrastive	r2	139.0646259	4	1	post_focus	1	Contrastive post_focus
2023408	block1	Control	pre	gei1	Object	Contrastive	r2	183.5566893	5	2	post_focus	1	Contrastive post_focus
2023408	block1	Control	pre	sau3	Subject	Contrastive	r2	105.2357083	1	1	on_focus	3	Contrastive on_focus
2023408	block1	Control	pre	sau3	Subject	Contrastive	r2	145.6014375	2	2	on_focus	3	Contrastive on_focus
2023408	block1	Control	pre	sik3	Verb	Contrastive	r2	109.1137566	3	1	post_focus	3	Contrastive post_focus
2023408	block1	Control	pre	baak3	Object	Contrastive	r2	107.9342404	4	1	post_focus	3	Contrastive post_focus
2023408	block1	Control	pre	baak3	Object	Contrastive	r2	62.15203542	5	2	post_focus	3	Contrastive post_focus
2023408	block1	Control	pre	zoeng1	Subject	Narrow	r2	122.4810089	1	1	on_focus	1	Narrow on_focus
2023408	block1	Control	pre	saang1	Subject	Narrow	r2	195.3230335	2	2	on_focus	1	Narrow on_focus
2023408	block1	Control	pre	tsa1	Verb	Narrow	r2	141.5386069	3	1	post_focus	1	Narrow post_focus
2023408	block1	Control	pre	fei1	Object	Narrow	r2	134.2068302	4	1	post_focus	1	Narrow post_focus
2023408	block1	Control	pre	gei1	Object	Narrow	r2	211.3467971	5	2	post_focus	1	Narrow post_focus
2023408	block1	Control	pre	sau3	Subject	Contrastive	r2	96.68948067	1	1	pre_focus	3	Contrastive pre_focus
2023408	block1	Control	pre	sau3	Subject	Contrastive	r2	107.7593132	2	2	pre_focus	3	Contrastive pre_focus
2023408	block1	Control	pre	sik3	Verb	Contrastive	r2	80.58988539	3	1	pre_focus	3	Contrastive pre_focus
2023408	block1	Control	pre	baak3	Object	Contrastive	r2	83.55099024	4	1	on_focus	3	Contrastive on_focus
2023408	block1	Control	pre	baak3	Object	Contrastive	r2	83.82936508	5	2	on_focus	3	Contrastive on_focus
2023408	block1	Control	pre	jyun2	Subject	Contrastive	r2	156.0486146	1	1	pre_focus	2	Contrastive pre_focus
2023408	block1	Control	pre	jyun2	Subject	Contrastive	r2	222.5381522	2	2	pre_focus	2	Contrastive pre_focus
2023408	block1	Control	pre	mo2	Verb	Contrastive	r2	151.3792895	3	1	pre_focus	2	Contrastive pre_focus
2023408	block1	Control	pre	gau2	Object	Contrastive	r2	140.4065436	4	1	on_focus	2	Contrastive on_focus
2023408	block1	Control	pre	zai2	Object	Contrastive	r2	236.1350466	5	2	on_focus	2	Contrastive on_focus
2023408	block1	Control	pre	sau3	Subject	Broad	r2	134.7990435	1	1	broad_focus	3	Broad focus
2023408	block1	Control	pre	sau3	Subject	Broad	r2	121.8333952	2	2	broad_focus	3	Broad focus
2023408	block1	Control	pre	sik3	Verb	Broad	r2	56.52545867	3	1	broad_focus	3	Broad focus
2023408	block1	Control	pre	baak3	Object	Broad	r2	118.53207	4	1	broad_focus	3	Broad focus
2023408	block1	Control	pre	baak3	Object	Broad	r2	41.64669042	5	2	broad_focus	3	Broad focus
2023408	block1	Control	pre	jyun2	Subject	Narrow	r2	210.5766923	1	1	on_focus	2	Narrow on_focus
2023408	block1	Control	pre	jyun2	Subject	Narrow	r2	203.3536911	2	2	on_focus	2	Narrow on_focus
2023408	block1	Control	pre	mo2	Verb	Narrow	r2	154.6389519	3	1	post_focus	2	Narrow post_focus
2023408	block1	Control	pre	gau2	Object	Narrow	r2	115.007842	4	1	post_focus	2	Narrow post_focus
2023408	block1	Control	pre	zai2	Object	Narrow	r2	202.4247921	5	2	post_focus	2	Narrow post_focus
2023408	block1	Control	pre	zoeng1	Subject	Narrow	r2	160.9493157	1	1	pre_focus	1	Narrow pre_focus
2023408	block1	Control	pre	saang1	Subject	Narrow	r2	186.4058957	2	2	pre_focus	1	Narrow pre_focus
2023408	block1	Control	pre	tsa1	Verb	Narrow	r2	141.3314728	3	1	on_focus	1	Narrow on_focus
2023408	block1	Control	pre	fei1	Object	Narrow	r2	146.458872	4	1	post_focus	1	Narrow post_focus
2023408	block1	Control	pre	gei1	Object	Narrow	r2	198.1679338	5	2	post_focus	1	Narrow post_focus
2023408	block1	Control	pre	jyun2	Subject	Broad	r2	170.1286848	1	1	broad_focus	2	Broad focus
2023408	block1	Control	pre	jyun2	Subject	Broad	r2	227.0596372	2	2	broad_focus	2	Broad focus
2023408	block1	Control	pre	mo2	Verb	Broad	r2	148.4418139	3	1	broad_focus	2	Broad focus
2023408	block1	Control	pre	gau2	Object	Broad	r2	153.4225246	4	1	broad_focus	2	Broad focus
2023408	block1	Control	pre	zai2	Object	Broad	r2	131.1444959	5	2	broad_focus	2	Broad focus
2023408	block1	Control	pre	zoeng1	Subject	Contrastive	r2	153.9418971	1	1	on_focus	1	Contrastive on_focus
2023408	block1	Control	pre	saang1	Subject	Contrastive	r2	178.8420257	2	2	on_focus	1	Contrastive on_focus
2023408	block1	Control	pre	tsa1	Verb	Contrastive	r2	128.9795918	3	1	post_focus	1	Contrastive post_focus
2023408	block1	Control	pre	fei1	Object	Contrastive	r2	170.0442717	4	1	post_focus	1	Contrastive post_focus
2023408	block1	Control	pre	gei1	Object	Contrastive	r2	202.5415211	5	2	post_focus	1	Contrastive post_focus
2023408	block2	Control	post	ngaa5	Subject	Narrow	r1	176.9252922	1	1	on_focus	5	Narrow on_focus
2023408	block2	Control	post	ngaa5	Subject	Narrow	r1	169.0619459	2	2	on_focus	5	Narrow on_focus
2023408	block2	Control	post	maai5	Verb	Narrow	r1	220.1736969	3	1	post_focus	5	Narrow post_focus
2023408	block2	Control	post	pou5	Object	Narrow	r1	161.1985995	4	1	post_focus	5	Narrow post_focus
2023408	block2	Control	post	pou5	Object	Narrow	r1	133.6768874	5	2	post_focus	5	Narrow post_focus
2023408	block2	Control	post	ngaa5	Subject	Contrastive	r1	114.0185216	1	1	pre_focus	5	Contrastive pre_focus
2023408	block2	Control	post	ngaa5	Subject	Contrastive	r1	157.2669264	2	2	pre_focus	5	Contrastive pre_focus
2023408	block2	Control	post	maai5	Verb	Contrastive	r1	169.0619459	3	1	on_focus	5	Contrastive on_focus
2023408	block2	Control	post	pou5	Object	Contrastive	r1	102.2235021	4	1	post_focus	5	Contrastive post_focus
2023408	block2	Control	post	pou5	Object	Contrastive	r1	102.2235021	5	2	post_focus	5	Contrastive post_focus
2023408	block2	Control	post	ngaa5	Subject	Contrastive	r1	114.0185216	1	1	pre_focus	5	Contrastive pre_focus
2023408	block2	Control	post	ngaa5	Subject	Contrastive	r1	212.3103506	2	2	pre_focus	5	Contrastive pre_focus
2023408	block2	Control	post	maai5	Verb	Contrastive	r1	184.7886385	3	1	pre_focus	5	Contrastive pre_focus
2023408	block2	Control	post	pou5	Object	Contrastive	r1	114.0185216	4	1	on_focus	5	Contrastive on_focus
2023408	block2	Control	post	pou5	Object	Contrastive	r1	121.8818679	5	2	on_focus	5	Contrastive on_focus

**2023408**	block2	Control	post	lok6	Subject	Narrow	r1	137.6085606	1	1	on_focus	6	Narrow on_focus
**2023408**	block2	Control	post	lok6	Subject	Narrow	r1	75.49793971	2	2	on_focus	6	Narrow on_focus
**2023408**	block2	Control	post	waa6	Verb	Narrow	r1	70.77011687	3	1	post_focus	6	Narrow post_focus
**2023408**	block2	Control	post	jyut6	Object	Narrow	r1	102.2235021	4	1	post_focus	6	Narrow post_focus
**2023408**	block2	Control	post	loeng6	Object	Narrow	r1	266.1840905	5	2	post_focus	6	Narrow post_focus
**2023408**	block2	Control	post	lok6	Subject	Contrastive	r1	145.4719069	1	1	pre_focus	6	Contrastive pre_focus
**2023408**	block2	Control	post	lok6	Subject	Contrastive	r1	121.8818679	2	2	pre_focus	6	Contrastive pre_focus
**2023408**	block2	Control	post	waa6	Verb	Contrastive	r1	70.77011687	3	1	on_focus	6	Contrastive on_focus
**2023408**	block2	Control	post	jyut6	Object	Contrastive	r1	82.56513635	4	1	post_focus	6	Contrastive post_focus
**2023408**	block2	Control	post	loeng6	Object	Contrastive	r1	385.3039696	5	2	post_focus	6	Contrastive post_focus
**2023408**	block2	Control	post	ngaa5	Subject	Narrow	r1	110.0868485	1	1	pre_focus	5	Narrow pre_focus
**2023408**	block2	Control	post	ngaa5	Subject	Narrow	r1	106.1551753	2	2	pre_focus	5	Narrow pre_focus
**2023408**	block2	Control	post	maai5	Verb	Narrow	r1	228.1304266	3	1	on_focus	5	Narrow on_focus
**2023408**	block2	Control	post	pou5	Object	Narrow	r1	106.1551753	4	1	post_focus	5	Narrow post_focus
**2023408**	block2	Control	post	pou5	Object	Narrow	r1	184.7886385	5	2	post_focus	5	Narrow post_focus
**2023408**	block2	Control	post	lok6	Subject	Narrow	r1	86.49680951	1	1	pre_focus	6	Narrow pre_focus
**2023408**	block2	Control	post	lok6	Subject	Narrow	r1	114.0185216	2	2	pre_focus	6	Narrow pre_focus
**2023408**	block2	Control	post	waa6	Verb	Narrow	r1	70.77011687	3	1	pre_focus	6	Narrow pre_focus
**2023408**	block2	Control	post	jyut6	Object	Narrow	r1	106.1551753	4	1	on_focus	6	Narrow on_focus
**2023408**	block2	Control	post	loeng6	Object	Narrow	r1	153.3352532	5	2	on_focus	6	Narrow on_focus
**2023408**	block2	Control	post	lok6	Subject	Contrastive	r1	110.0868485	1	1	pre_focus	6	Contrastive pre_focus
**2023408**	block2	Control	post	lok6	Subject	Contrastive	r1	102.2235021	2	2	pre_focus	6	Contrastive pre_focus
**2023408**	block2	Control	post	waa6	Verb	Contrastive	r1	86.49680951	3	1	pre_focus	6	Contrastive pre_focus
**2023408**	block2	Control	post	jyut6	Object	Contrastive	r1	74.70179003	4	1	on_focus	6	Contrastive on_focus
**2023408**	block2	Control	post	loeng6	Object	Contrastive	r1	228.0370432	5	2	on_focus	6	Contrastive on_focus
**2023408**	block2	Control	post	ma4	Subject	Broad	r1	141.5402337	1	1	broad_focus	4	Broad focus
**2023408**	block2	Control	post	ma4	Subject	Broad	r1	176.9252922	2	2	broad_focus	4	Broad focus
**2023408**	block2	Control	post	fu4	Verb	Broad	r1	114.0185216	3	1	broad_focus	4	Broad focus
**2023408**	block2	Control	post	maang4	Object	Broad	r1	239.8320627	4	1	broad_focus	4	Broad focus
**2023408**	block2	Control	post	Jan-04	Object	Broad	r1	220.1736969	5	2	broad_focus	4	Broad focus
**2023408**	block2	Control	post	lok6	Subject	Contrastive	r1	145.4719069	1	1	on_focus	6	Contrastive on_focus
**2023408**	block2	Control	post	lok6	Subject	Contrastive	r1	145.4719069	2	2	on_focus	6	Contrastive on_focus
**2023408**	block2	Control	post	waa6	Verb	Contrastive	r1	129.7452143	3	1	post_focus	6	Contrastive post_focus
**2023408**	block2	Control	post	jyut6	Object	Contrastive	r1	86.49680951	4	1	post_focus	6	Contrastive post_focus
**2023408**	block2	Control	post	loeng6	Object	Contrastive	r1	365.6456038	5	2	post_focus	6	Contrastive post_focus
**2023408**	block2	Control	post	ma4	Subject	Narrow	r1	135.642724	1	1	on_focus	4	Narrow on_focus
**2023408**	block2	Control	post	ma4	Subject	Narrow	r1	202.4811677	2	2	on_focus	4	Narrow on_focus
**2023408**	block2	Control	post	fu4	Verb	Narrow	r1	180.8569653	3	1	post_focus	4	Narrow post_focus
**2023408**	block2	Control	post	maang4	Object	Narrow	r1	215.2591055	4	1	post_focus	4	Narrow post_focus
**2023408**	block2	Control	post	Jan-04	Object	Narrow	r1	109.1039302	5	2	post_focus	4	Narrow post_focus
**2023408**	block2	Control	post	ngaa5	Subject	Narrow	r1	96.19040718	1	1	pre_focus	5	Narrow pre_focus
**2023408**	block2	Control	post	ngaa5	Subject	Narrow	r1	159.3683482	2	2	pre_focus	5	Narrow pre_focus
**2023408**	block2	Control	post	maai5	Verb	Narrow	r1	235.9003896	3	1	pre_focus	5	Narrow pre_focus
**2023408**	block2	Control	post	pou5	Object	Narrow	r1	133.6768874	4	1	on_focus	5	Narrow on_focus
**2023408**	block2	Control	post	pou5	Object	Narrow	r1	114.0185216	5	2	on_focus	5	Narrow on_focus
**2023408**	block2	Control	post	lok6	Subject	Narrow	r1	171.0277824	1	1	pre_focus	6	Narrow pre_focus
**2023408**	block2	Control	post	lok6	Subject	Narrow	r1	147.4377435	2	2	pre_focus	6	Narrow pre_focus
**2023408**	block2	Control	post	waa6	Verb	Narrow	r1	74.70179003	3	1	on_focus	6	Narrow on_focus
**2023408**	block2	Control	post	jyut6	Object	Narrow	r1	66.83844371	4	1	post_focus	6	Narrow post_focus
**2023408**	block2	Control	post	loeng6	Object	Narrow	r1	220.1736969	5	2	post_focus	6	Narrow post_focus
**2023408**	block2	Control	post	ngaa5	Subject	Contrastive	r1	125.8135411	1	1	on_focus	5	Contrastive on_focus
**2023408**	block2	Control	post	ngaa5	Subject	Contrastive	r1	145.4719069	2	2	on_focus	5	Contrastive on_focus
**2023408**	block2	Control	post	maai5	Verb	Contrastive	r1	247.695409	3	1	post_focus	5	Contrastive post_focus
**2023408**	block2	Control	post	pou5	Object	Contrastive	r1	131.6176683	4	1	post_focus	5	Contrastive post_focus
**2023408**	block2	Control	post	pou5	Object	Contrastive	r1	212.3103506	5	2	post_focus	5	Contrastive post_focus
**2023408**	block2	Control	post	ma4	Subject	Contrastive	r1	165.1302727	1	1	pre_focus	4	Contrastive pre_focus
**2023408**	block2	Control	post	ma4	Subject	Contrastive	r1	227.189748	2	2	pre_focus	4	Contrastive pre_focus
**2023408**	block2	Control	post	fu4	Verb	Contrastive	r1	147.3021204	3	1	on_focus	4	Contrastive on_focus
**2023408**	block2	Control	post	maang4	Object	Contrastive	r1	276.2000395	4	1	post_focus	4	Contrastive post_focus
**2023408**	block2	Control	post	Jan-04	Object	Contrastive	r1	214.2761872	5	2	post_focus	4	Contrastive post_focus
**2023408**	block2	Control	post	ma4	Subject	Narrow	r1	208.3786775	1	1	pre_focus	4	Narrow pre_focus
**2023408**	block2	Control	post	ma4	Subject	Narrow	r1	200.5153311	2	2	pre_focus	4	Narrow pre_focus
**2023408**	block2	Control	post	fu4	Verb	Narrow	r1	159.232763	3	1	on_focus	4	Narrow on_focus
**2023408**	block2	Control	post	maang4	Object	Narrow	r1	269.3196114	4	1	post_focus	4	Narrow post_focus
**2023408**	block2	Control	post	Jan-04	Object	Narrow	r1	190.6861482	5	2	post_focus	4	Narrow post_focus
**2023408**	block2	Control	post	ma4	Subject	Contrastive	r1	172.993619	1	1	pre_focus	4	Contrastive pre_focus

2023408	block2	Control	post	ma4	Subject	Contrastive	r1	157.2669264	2	2	pre_focus	4	Contrastive pre_focus
2023408	block2	Control	post	fu4	Verb	Contrastive	r1	128.762296	3	1	pre_focus	4	Contrastive pre_focus
2023408	block2	Control	post	maang4	Object	Contrastive	r1	224.1053701	4	1	on_focus	4	Contrastive on_focus
2023408	block2	Control	post	Jan-04	Object	Contrastive	r1	172.0107007	5	2	on_focus	4	Contrastive on_focus
2023408	block2	Control	post	ma4	Subject	Contrastive	r1	153.3352532	1	1	on_focus	4	Contrastive on_focus
2023408	block2	Control	post	ma4	Subject	Contrastive	r1	204.4470043	2	2	on_focus	4	Contrastive on_focus
2023408	block2	Control	post	fu4	Verb	Contrastive	r1	180.8569653	3	1	post_focus	4	Contrastive post_focus
2023408	block2	Control	post	maang4	Object	Contrastive	r1	211.3274323	4	1	post_focus	4	Contrastive post_focus
2023408	block2	Control	post	Jan-04	Object	Contrastive	r1	210.344514	5	2	post_focus	4	Contrastive post_focus
2023408	block2	Control	post	lok6	Subject	Broad	r1	102.2235021	1	1	broad_focus	6	Broad focus
2023408	block2	Control	post	lok6	Subject	Broad	r1	133.6768874	2	2	broad_focus	6	Broad focus
2023408	block2	Control	post	waa6	Verb	Broad	r1	98.29182899	3	1	broad_focus	6	Broad focus
2023408	block2	Control	post	jyut6	Object	Broad	r1	121.8818679	4	1	broad_focus	6	Broad focus
2023408	block2	Control	post	loeng6	Object	Broad	r1	251.6270822	5	2	broad_focus	6	Broad focus
2023408	block2	Control	post	ngaa5	Subject	Broad	r1	90.42848267	1	1	broad_focus	5	Broad focus
2023408	block2	Control	post	ngaa5	Subject	Broad	r1	140.5573155	2	2	broad_focus	5	Broad focus
2023408	block2	Control	post	maai5	Verb	Broad	r1	155.3010898	3	1	broad_focus	5	Broad focus
2023408	block2	Control	post	pou5	Object	Broad	r1	94.36015583	4	1	broad_focus	5	Broad focus
2023408	block2	Control	post	pou5	Object	Broad	r1	57.9921791	5	2	broad_focus	5	Broad focus
2023408	block2	Control	post	ma4	Subject	Narrow	r1	139.5743972	1	1	pre_focus	4	Narrow pre_focus
2023408	block2	Control	post	ma4	Subject	Narrow	r1	180.8569653	2	2	pre_focus	4	Narrow pre_focus
2023408	block2	Control	post	fu4	Verb	Narrow	r1	131.7110508	3	1	pre_focus	4	Narrow pre_focus
2023408	block2	Control	post	maang4	Object	Narrow	r1	263.4221017	4	1	on_focus	4	Narrow on_focus
2023408	block2	Control	post	Jan-04	Object	Narrow	r1	125.8135411	5	2	on_focus	4	Narrow on_focus
2023408	block2	Control	post	ma4	Subject	Broad	r2	151.3694166	1	1	broad_focus	4	Broad focus
2023408	block2	Control	post	ma4	Subject	Broad	r2	231.9687164	2	2	broad_focus	4	Broad focus
2023408	block2	Control	post	fu4	Verb	Broad	r2	155.3010898	3	1	broad_focus	4	Broad focus
2023408	block2	Control	post	maang4	Object	Broad	r2	271.285448	4	1	broad_focus	4	Broad focus
2023408	block2	Control	post	Jan-04	Object	Broad	r2	123.8477045	5	2	broad_focus	4	Broad focus
2023408	block2	Control	post	lok6	Subject	Contrastive	r2	129.7452143	1	1	pre_focus	6	Contrastive pre_focus
2023408	block2	Control	post	lok6	Subject	Contrastive	r2	121.8818679	2	2	pre_focus	6	Contrastive pre_focus
2023408	block2	Control	post	waa6	Verb	Contrastive	r2	92.39431925	3	1	on_focus	6	Contrastive on_focus
2023408	block2	Control	post	jyut6	Object	Contrastive	r2	78.63346319	4	1	post_focus	6	Contrastive post_focus
2023408	block2	Control	post	loeng6	Object	Contrastive	r2	210.344514	5	2	post_focus	6	Contrastive post_focus
2023408	block2	Control	post	ma4	Subject	Contrastive	r2	176.9252922	1	1	on_focus	4	Contrastive on_focus
2023408	block2	Control	post	ma4	Subject	Contrastive	r2	214.2761872	2	2	on_focus	4	Contrastive on_focus
2023408	block2	Control	post	fu4	Verb	Contrastive	r2	141.5402337	3	1	post_focus	4	Contrastive post_focus
2023408	block2	Control	post	maang4	Object	Contrastive	r2	259.4904285	4	1	post_focus	4	Contrastive post_focus
2023408	block2	Control	post	Jan-04	Object	Contrastive	r2	172.993619	5	2	post_focus	4	Contrastive post_focus
2023408	block2	Control	post	ma4	Subject	Narrow	r2	172.993619	1	1	pre_focus	4	Narrow pre_focus
2023408	block2	Control	post	ma4	Subject	Narrow	r2	196.583658	2	2	pre_focus	4	Narrow pre_focus
2023408	block2	Control	post	fu4	Verb	Narrow	r2	180.8569653	3	1	pre_focus	4	Narrow pre_focus
2023408	block2	Control	post	maang4	Object	Narrow	r2	221.6480744	4	1	on_focus	4	Narrow on_focus
2023408	block2	Control	post	Jan-04	Object	Narrow	r2	196.0921988	5	2	on_focus	4	Narrow on_focus
2023408	block2	Control	post	ngaa5	Subject	Contrastive	r2	94.36015583	1	1	on_focus	5	Contrastive on_focus
2023408	block2	Control	post	ngaa5	Subject	Contrastive	r2	165.1302727	2	2	on_focus	5	Contrastive on_focus
2023408	block2	Control	post	maai5	Verb	Contrastive	r2	184.7886385	3	1	post_focus	5	Contrastive post_focus
2023408	block2	Control	post	pou5	Object	Contrastive	r2	114.0185216	4	1	post_focus	5	Contrastive post_focus
2023408	block2	Control	post	pou5	Object	Contrastive	r2	145.4719069	5	2	post_focus	5	Contrastive post_focus
2023408	block2	Control	post	ngaa5	Subject	Contrastive	r2	90.42848267	1	1	pre_focus	5	Contrastive pre_focus
2023408	block2	Control	post	ngaa5	Subject	Contrastive	r2	117.9501948	2	2	pre_focus	5	Contrastive pre_focus
2023408	block2	Control	post	maai5	Verb	Contrastive	r2	188.7203117	3	1	on_focus	5	Contrastive on_focus
2023408	block2	Control	post	pou5	Object	Contrastive	r2	117.9501948	4	1	post_focus	5	Contrastive post_focus
2023408	block2	Control	post	pou5	Object	Contrastive	r2	129.7452143	5	2	post_focus	5	Contrastive post_focus
2023408	block2	Control	post	ngaa5	Subject	Narrow	r2	102.2235021	1	1	pre_focus	5	Narrow pre_focus
2023408	block2	Control	post	ngaa5	Subject	Narrow	r2	137.6085606	2	2	pre_focus	5	Narrow pre_focus
2023408	block2	Control	post	maai5	Verb	Narrow	r2	201.4982494	3	1	on_focus	5	Narrow on_focus
2023408	block2	Control	post	pou5	Object	Narrow	r2	125.8135411	4	1	post_focus	5	Narrow post_focus
2023408	block2	Control	post	pou5	Object	Narrow	r2	137.6085606	5	2	post_focus	5	Narrow post_focus
2023408	block2	Control	post	ngaa5	Subject	Narrow	r2	110.0868485	1	1	pre_focus	5	Narrow pre_focus
2023408	block2	Control	post	ngaa5	Subject	Narrow	r2	129.7452143	2	2	pre_focus	5	Narrow pre_focus
2023408	block2	Control	post	maai5	Verb	Narrow	r2	180.8569653	3	1	pre_focus	5	Narrow pre_focus
2023408	block2	Control	post	pou5	Object	Narrow	r2	114.0185216	4	1	on_focus	5	Narrow on_focus
2023408	block2	Control	post	pou5	Object	Narrow	r2	125.8135411	5	2	on_focus	5	Narrow on_focus
2023408	block2	Control	post	ma4	Subject	Narrow	r2	239.8320627	1	1	pre_focus	4	Narrow pre_focus
2023408	block2	Control	post	ma4	Subject	Narrow	r2	224.1053701	2	2	pre_focus	4	Narrow pre_focus

2023408	block2	Control	post	fu4	Verb	Narrow	r2	90.42848267	3	1	on_focus	4	Narrow on_focus
2023408	block2	Control	post	maang4	Object	Narrow	r2	330.7520045	4	1	post_focus	4	Narrow post_focus
2023408	block2	Control	post	Jan-04	Object	Narrow	r2	165.1302727	5	2	post_focus	4	Narrow post_focus
2023408	block2	Control	post	ma4	Subject	Narrow	r2	149.4035801	1	1	on_focus	4	Narrow on_focus
2023408	block2	Control	post	ma4	Subject	Narrow	r2	223.1224518	2	2	on_focus	4	Narrow on_focus
2023408	block2	Control	post	fu4	Verb	Narrow	r2	161.6900587	3	1	post_focus	4	Narrow post_focus
2023408	block2	Control	post	maang4	Object	Narrow	r2	264.9898604	4	1	post_focus	4	Narrow post_focus
2023408	block2	Control	post	Jan-04	Object	Narrow	r2	117.8568136	5	2	post_focus	4	Narrow post_focus
2023408	block2	Control	post	ma4	Subject	Contrastive	r2	139.5743972	1	1	pre_focus	4	Contrastive pre_focus
2023408	block2	Control	post	ma4	Subject	Contrastive	r2	247.695409	2	2	pre_focus	4	Contrastive pre_focus
2023408	block2	Control	post	fu4	Verb	Contrastive	r2	178.8911288	3	1	on_focus	4	Contrastive on_focus
2023408	block2	Control	post	maang4	Object	Contrastive	r2	210.344514	4	1	post_focus	4	Contrastive post_focus
2023408	block2	Control	post	Jan-04	Object	Contrastive	r2	145.4719069	5	2	post_focus	4	Contrastive post_focus
2023408	block2	Control	post	lok6	Subject	Contrastive	r2	149.4035801	1	1	pre_focus	6	Contrastive pre_focus
2023408	block2	Control	post	lok6	Subject	Contrastive	r2	121.8818679	2	2	pre_focus	6	Contrastive pre_focus
2023408	block2	Control	post	waa6	Verb	Contrastive	r2	117.9501948	3	1	pre_focus	6	Contrastive pre_focus
2023408	block2	Control	post	jyut6	Object	Contrastive	r2	78.63346319	4	1	on_focus	6	Contrastive on_focus
2023408	block2	Control	post	loeng6	Object	Contrastive	r2	153.3352532	5	2	on_focus	6	Contrastive on_focus
2023408	block2	Control	post	ma4	Subject	Contrastive	r2	176.9252922	1	1	pre_focus	4	Contrastive pre_focus
2023408	block2	Control	post	ma4	Subject	Contrastive	r2	169.0619459	2	2	pre_focus	4	Contrastive pre_focus
2023408	block2	Control	post	fu4	Verb	Contrastive	r2	110.0868485	3	1	pre_focus	4	Contrastive pre_focus
2023408	block2	Control	post	maang4	Object	Contrastive	r2	214.5219168	4	1	on_focus	4	Contrastive on_focus
2023408	block2	Control	post	Jan-04	Object	Contrastive	r2	141.5402337	5	2	on_focus	4	Contrastive on_focus
2023408	block2	Control	post	lok6	Subject	Broad	r2	80.59929977	1	1	broad_focus	6	Broad focus
2023408	block2	Control	post	lok6	Subject	Broad	r2	85.51389122	2	2	broad_focus	6	Broad focus
2023408	block2	Control	post	waa6	Verb	Broad	r2	70.77011687	3	1	broad_focus	6	Broad focus
2023408	block2	Control	post	jyut6	Object	Broad	r2	86.49680951	4	1	broad_focus	6	Broad focus
2023408	block2	Control	post	loeng6	Object	Broad	r2	298.7137783	5	2	broad_focus	6	Broad focus
2023408	block2	Control	post	ngaa5	Subject	Narrow	r2	157.2669264	1	1	on_focus	5	Narrow on_focus
2023408	block2	Control	post	ngaa5	Subject	Narrow	r2	192.6519848	2	2	on_focus	5	Narrow on_focus
2023408	block2	Control	post	maai5	Verb	Narrow	r2	192.6519848	3	1	post_focus	5	Narrow post_focus
2023408	block2	Control	post	pou5	Object	Narrow	r2	125.8135411	4	1	post_focus	5	Narrow post_focus
2023408	block2	Control	post	pou5	Object	Narrow	r2	180.8569653	5	2	post_focus	5	Narrow post_focus
2023408	block2	Control	post	ngaa5	Subject	Contrastive	r2	143.5060703	1	1	pre_focus	5	Contrastive pre_focus
2023408	block2	Control	post	ngaa5	Subject	Contrastive	r2	174.9594556	2	2	pre_focus	5	Contrastive pre_focus
2023408	block2	Control	post	maai5	Verb	Contrastive	r2	198.5494946	3	1	pre_focus	5	Contrastive pre_focus
2023408	block2	Control	post	pou5	Object	Contrastive	r2	171.0277824	4	1	on_focus	5	Contrastive on_focus
2023408	block2	Control	post	pou5	Object	Contrastive	r2	170.7819021	5	2	on_focus	5	Contrastive on_focus
2023408	block2	Control	post	lok6	Subject	Contrastive	r2	184.7886385	1	1	on_focus	6	Contrastive on_focus
2023408	block2	Control	post	lok6	Subject	Contrastive	r2	106.1551753	2	2	on_focus	6	Contrastive on_focus
2023408	block2	Control	post	waa6	Verb	Contrastive	r2	84.48427993	3	1	post_focus	6	Contrastive post_focus
2023408	block2	Control	post	jyut6	Object	Contrastive	r2	70.72342481	4	1	post_focus	6	Contrastive post_focus
2023408	block2	Control	post	loeng6	Object	Contrastive	r2	275.2171212	5	2	post_focus	6	Contrastive post_focus
2023408	block2	Control	post	lok6	Subject	Narrow	r2	220.1736969	1	1	pre_focus	6	Narrow pre_focus
2023408	block2	Control	post	lok6	Subject	Narrow	r2	153.3352532	2	2	pre_focus	6	Narrow pre_focus
2023408	block2	Control	post	waa6	Verb	Narrow	r2	94.36015583	3	1	on_focus	6	Narrow on_focus
2023408	block2	Control	post	jyut6	Object	Narrow	r2	102.2235021	4	1	post_focus	6	Narrow post_focus
2023408	block2	Control	post	loeng6	Object	Narrow	r2	212.3103506	5	2	post_focus	6	Narrow post_focus
2023408	block2	Control	post	lok6	Subject	Narrow	r2	137.6085606	1	1	pre_focus	6	Narrow pre_focus
2023408	block2	Control	post	lok6	Subject	Narrow	r2	145.4719069	2	2	pre_focus	6	Narrow pre_focus
2023408	block2	Control	post	waa6	Verb	Narrow	r2	97.26221774	3	1	pre_focus	6	Narrow pre_focus
2023408	block2	Control	post	jyut6	Object	Narrow	r2	82.56513635	4	1	on_focus	6	Narrow on_focus
2023408	block2	Control	post	loeng6	Object	Narrow	r2	220.1736969	5	2	on_focus	6	Narrow on_focus
2023408	block2	Control	post	ngaa5	Subject	Broad	r2	149.4502721	1	1	broad_focus	5	Broad focus
2023408	block2	Control	post	ngaa5	Subject	Broad	r2	145.4719069	2	2	broad_focus	5	Broad focus
2023408	block2	Control	post	maai5	Verb	Broad	r2	259.4904285	3	1	broad_focus	5	Broad focus
2023408	block2	Control	post	pou5	Object	Broad	r2	156.7754672	4	1	broad_focus	5	Broad focus
2023408	block2	Control	post	pou5	Object	Broad	r2	170.5363233	5	2	broad_focus	5	Broad focus
2023408	block2	Control	post	lok6	Subject	Narrow	r2	110.0868485	1	1	on_focus	6	Narrow on_focus
2023408	block2	Control	post	lok6	Subject	Narrow	r2	133.6768874	2	2	on_focus	6	Narrow on_focus
2023408	block2	Control	post	waa6	Verb	Narrow	r2	63.88968884	3	1	post_focus	6	Narrow post_focus
2023408	block2	Control	post	jyut6	Object	Narrow	r2	76.66762661	4	1	post_focus	6	Narrow post_focus
2023408	block2	Control	post	loeng6	Object	Narrow	r2	286.5206815	5	2	post_focus	6	Narrow post_focus
2023408	block2	Control	pre	lok6	Subject	Broad	r1	135.7286012	1	1	broad_focus	6	Broad focus
2023408	block2	Control	pre	lok6	Subject	Broad	r1	153.3044428	2	2	broad_focus	6	Broad focus
2023408	block2	Control	pre	waa6	Verb	Broad	r1	159.7614372	3	1	broad_focus	6	Broad focus

2023408	block2	Control	pre	jyut6	Object	Broad	r1	188.2459145	4	1	broad_focus	6	Broad focus
2023408	block2	Control	pre	loeng6	Object	Broad	r1	316.5117158	5	2	broad_focus	6	Broad focus
2023408	block2	Control	pre	ngaa5	Subject	Contrastive	r1	174.9734545	1	1	pre_focus	5	Contrastive pre_focus
2023408	block2	Control	pre	ngaa5	Subject	Contrastive	r1	197.0009337	2	2	pre_focus	5	Contrastive pre_focus
2023408	block2	Control	pre	maai5	Verb	Contrastive	r1	294.9420509	3	1	on_focus	5	Contrastive on_focus
2023408	block2	Control	pre	pou5	Object	Contrastive	r1	130.5366591	4	1	post_focus	5	Contrastive post_focus
2023408	block2	Control	pre	pou5	Object	Contrastive	r1	120.6221655	5	2	post_focus	5	Contrastive post_focus
2023408	block2	Control	pre	ma4	Subject	Narrow	r1	150.1393891	1	1	on_focus	4	Narrow on_focus
2023408	block2	Control	pre	ma4	Subject	Narrow	r1	221.1572184	2	2	on_focus	4	Narrow on_focus
2023408	block2	Control	pre	fu4	Verb	Narrow	r1	182.3700562	3	1	post_focus	4	Narrow post_focus
2023408	block2	Control	pre	maang4	Object	Narrow	r1	226.1862434	4	1	post_focus	4	Narrow post_focus
2023408	block2	Control	pre	Jan-04	Object	Narrow	r1	208.5238827	5	2	post_focus	4	Narrow post_focus
2023408	block2	Control	pre	ngaa5	Subject	Narrow	r1	176.45199	1	1	pre_focus	5	Narrow pre_focus
2023408	block2	Control	pre	ngaa5	Subject	Narrow	r1	207.3449321	2	2	pre_focus	5	Narrow pre_focus
2023408	block2	Control	pre	maai5	Verb	Narrow	r1	296.8969874	3	1	on_focus	5	Narrow on_focus
2023408	block2	Control	pre	pou5	Object	Narrow	r1	109.1780782	4	1	post_focus	5	Narrow post_focus
2023408	block2	Control	pre	pou5	Object	Narrow	r1	231.0922147	5	2	post_focus	5	Narrow post_focus
2023408	block2	Control	pre	ma4	Subject	Contrastive	r1	135.0608001	1	1	pre_focus	4	Contrastive pre_focus
2023408	block2	Control	pre	ma4	Subject	Contrastive	r1	241.6735954	2	2	pre_focus	4	Contrastive pre_focus
2023408	block2	Control	pre	fu4	Verb	Contrastive	r1	225.3699415	3	1	on_focus	4	Contrastive on_focus
2023408	block2	Control	pre	maang4	Object	Contrastive	r1	254.2078281	4	1	post_focus	4	Contrastive post_focus
2023408	block2	Control	pre	Jan-04	Object	Contrastive	r1	207.0093629	5	2	post_focus	4	Contrastive post_focus
2023408	block2	Control	pre	ma4	Subject	Broad	r1	138.151197	1	1	broad_focus	4	Broad focus
2023408	block2	Control	pre	ma4	Subject	Broad	r1	261.7673364	2	2	broad_focus	4	Broad focus
2023408	block2	Control	pre	fu4	Verb	Broad	r1	108.8907371	3	1	broad_focus	4	Broad focus
2023408	block2	Control	pre	maang4	Object	Broad	r1	290.5743551	4	1	broad_focus	4	Broad focus
2023408	block2	Control	pre	Jan-04	Object	Broad	r1	255.7843285	5	2	broad_focus	4	Broad focus
2023408	block2	Control	pre	ma4	Subject	Contrastive	r1	211.0402862	1	1	on_focus	4	Contrastive on_focus
2023408	block2	Control	pre	ma4	Subject	Contrastive	r1	276.8300799	2	2	on_focus	4	Contrastive on_focus
2023408	block2	Control	pre	fu4	Verb	Contrastive	r1	176.3548753	3	1	post_focus	4	Contrastive post_focus
2023408	block2	Control	pre	maang4	Object	Contrastive	r1	278.4416687	4	1	post_focus	4	Contrastive post_focus
2023408	block2	Control	pre	Jan-04	Object	Contrastive	r1	242.9614512	5	2	post_focus	4	Contrastive post_focus
2023408	block2	Control	pre	lok6	Subject	Narrow	r1	207.9431217	1	1	pre_focus	6	Narrow pre_focus
2023408	block2	Control	pre	lok6	Subject	Narrow	r1	192.9026833	2	2	pre_focus	6	Narrow pre_focus
2023408	block2	Control	pre	waa6	Verb	Narrow	r1	151.4735143	3	1	on_focus	6	Narrow on_focus
2023408	block2	Control	pre	jyut6	Object	Narrow	r1	308.6285903	4	1	post_focus	6	Narrow post_focus
2023408	block2	Control	pre	loeng6	Object	Narrow	r1	269.3418024	5	2	post_focus	6	Narrow post_focus
2023408	block2	Control	pre	ngaa5	Subject	Contrastive	r1	115.3730576	1	1	pre_focus	5	Contrastive pre_focus
2023408	block2	Control	pre	ngaa5	Subject	Contrastive	r1	107.6145501	2	2	pre_focus	5	Contrastive pre_focus
2023408	block2	Control	pre	maai5	Verb	Contrastive	r1	228.8917234	3	1	pre_focus	5	Contrastive pre_focus
2023408	block2	Control	pre	pou5	Object	Contrastive	r1	103.3560091	4	1	on_focus	5	Contrastive on_focus
2023408	block2	Control	pre	pou5	Object	Contrastive	r1	174.3496923	5	2	on_focus	5	Contrastive on_focus
2023408	block2	Control	pre	lok6	Subject	Contrastive	r1	148.8978328	1	1	on_focus	6	Contrastive on_focus
2023408	block2	Control	pre	lok6	Subject	Contrastive	r1	172.8250864	2	2	on_focus	6	Contrastive on_focus
2023408	block2	Control	pre	waa6	Verb	Contrastive	r1	128.7819161	3	1	post_focus	6	Contrastive post_focus
2023408	block2	Control	pre	jyut6	Object	Contrastive	r1	101.9077313	4	1	post_focus	6	Contrastive post_focus
2023408	block2	Control	pre	loeng6	Object	Contrastive	r1	149.6098828	5	2	post_focus	6	Contrastive post_focus
2023408	block2	Control	pre	lok6	Subject	Contrastive	r1	186.5947139	1	1	pre_focus	6	Contrastive pre_focus
2023408	block2	Control	pre	lok6	Subject	Contrastive	r1	135.5365398	2	2	pre_focus	6	Contrastive pre_focus
2023408	block2	Control	pre	waa6	Verb	Contrastive	r1	128.9196876	3	1	on_focus	6	Contrastive on_focus
2023408	block2	Control	pre	jyut6	Object	Contrastive	r1	105.7401794	4	1	post_focus	6	Contrastive post_focus
2023408	block2	Control	pre	loeng6	Object	Contrastive	r1	408.5131195	5	2	post_focus	6	Contrastive post_focus
2023408	block2	Control	pre	ngaa5	Subject	Contrastive	r1	216.2811448	1	1	on_focus	5	Contrastive on_focus
2023408	block2	Control	pre	ngaa5	Subject	Contrastive	r1	176.0644999	2	2	on_focus	5	Contrastive on_focus
2023408	block2	Control	pre	maai5	Verb	Contrastive	r1	240.4131121	3	1	post_focus	5	Contrastive post_focus
2023408	block2	Control	pre	pou5	Object	Contrastive	r1	114.6900983	4	1	post_focus	5	Contrastive post_focus
2023408	block2	Control	pre	pou5	Object	Contrastive	r1	231.8234781	5	2	post_focus	5	Contrastive post_focus
2023408	block2	Control	pre	ngaa5	Subject	Narrow	r1	137.9867488	1	1	on_focus	5	Narrow on_focus
2023408	block2	Control	pre	ngaa5	Subject	Narrow	r1	141.1356009	2	2	on_focus	5	Narrow on_focus
2023408	block2	Control	pre	maai5	Verb	Narrow	r1	225.341768	3	1	post_focus	5	Narrow post_focus
2023408	block2	Control	pre	pou5	Object	Narrow	r1	83.64614222	4	1	post_focus	5	Narrow post_focus
2023408	block2	Control	pre	pou5	Object	Narrow	r1	169.4756163	5	2	post_focus	5	Narrow post_focus
2023408	block2	Control	pre	ma4	Subject	Narrow	r1	185.2148033	1	1	pre_focus	4	Narrow pre_focus
2023408	block2	Control	pre	ma4	Subject	Narrow	r1	229.0431507	2	2	pre_focus	4	Narrow pre_focus
2023408	block2	Control	pre	fu4	Verb	Narrow	r1	168.2476694	3	1	on_focus	4	Narrow on_focus
2023408	block2	Control	pre	maang4	Object	Narrow	r1	247.0634051	4	1	post_focus	4	Narrow post_focus

**2023408**	block2	Control	pre	Jan-04	Object	Narrow	r1	193.9799158	5	2	post_focus	4	Narrow post_focus
**2023408**	block2	Control	pre	ngaa5	Subject	Broad	r1	184.7937251	1	1	broad_focus	5	Broad focus
**2023408**	block2	Control	pre	ngaa5	Subject	Broad	r1	221.0303288	2	2	broad_focus	5	Broad focus
**2023408**	block2	Control	pre	maai5	Verb	Broad	r1	222.2940287	3	1	broad_focus	5	Broad focus
**2023408**	block2	Control	pre	pou5	Object	Broad	r1	126.2724444	4	1	broad_focus	5	Broad focus
**2023408**	block2	Control	pre	pou5	Object	Broad	r1	247.3864178	5	2	broad_focus	5	Broad focus
**2023408**	block2	Control	pre	lok6	Subject	Narrow	r1	98.23753744	1	1	pre_focus	6	Narrow pre_focus
**2023408**	block2	Control	pre	lok6	Subject	Narrow	r1	93.67252457	2	2	pre_focus	6	Narrow pre_focus
**2023408**	block2	Control	pre	waa6	Verb	Narrow	r1	127.824724	3	1	pre_focus	6	Narrow pre_focus
**2023408**	block2	Control	pre	jyut6	Object	Narrow	r1	184.1951058	4	1	on_focus	6	Narrow on_focus
**2023408**	block2	Control	pre	loeng6	Object	Narrow	r1	207.570789	5	2	on_focus	6	Narrow on_focus
**2023408**	block2	Control	pre	ma4	Subject	Narrow	r1	175.1145462	1	1	pre_focus	4	Narrow pre_focus
**2023408**	block2	Control	pre	ma4	Subject	Narrow	r1	211.313933	2	2	pre_focus	4	Narrow pre_focus
**2023408**	block2	Control	pre	fu4	Verb	Narrow	r1	188.3518228	3	1	pre_focus	4	Narrow pre_focus
**2023408**	block2	Control	pre	maang4	Object	Narrow	r1	247.2937245	4	1	on_focus	4	Narrow on_focus
**2023408**	block2	Control	pre	Jan-04	Object	Narrow	r1	215.3933299	5	2	on_focus	4	Narrow on_focus
**2023408**	block2	Control	pre	lok6	Subject	Narrow	r1	93.86750155	1	1	on_focus	6	Narrow on_focus
**2023408**	block2	Control	pre	lok6	Subject	Narrow	r1	138.7406815	2	2	on_focus	6	Narrow on_focus
**2023408**	block2	Control	pre	waa6	Verb	Narrow	r1	117.9247125	3	1	post_focus	6	Narrow post_focus
**2023408**	block2	Control	pre	jyut6	Object	Narrow	r1	183.2215108	4	1	post_focus	6	Narrow post_focus
**2023408**	block2	Control	pre	loeng6	Object	Narrow	r1	294.446395	5	2	post_focus	6	Narrow post_focus
**2023408**	block2	Control	pre	ma4	Subject	Contrastive	r1	153.3046107	1	1	pre_focus	4	Contrastive pre_focus
**2023408**	block2	Control	pre	ma4	Subject	Contrastive	r1	265.4500746	2	2	pre_focus	4	Contrastive pre_focus
**2023408**	block2	Control	pre	fu4	Verb	Contrastive	r1	166.5046971	3	1	pre_focus	4	Contrastive pre_focus
**2023408**	block2	Control	pre	maang4	Object	Contrastive	r1	270.5142983	4	1	on_focus	4	Contrastive on_focus
**2023408**	block2	Control	pre	Jan-04	Object	Contrastive	r1	262.3386931	5	2	on_focus	4	Contrastive on_focus
**2023408**	block2	Control	pre	ngaa5	Subject	Narrow	r1	125.7621955	1	1	pre_focus	5	Narrow pre_focus
**2023408**	block2	Control	pre	ngaa5	Subject	Narrow	r1	164.2598148	2	2	pre_focus	5	Narrow pre_focus
**2023408**	block2	Control	pre	maai5	Verb	Narrow	r1	262.1264172	3	1	pre_focus	5	Narrow pre_focus
**2023408**	block2	Control	pre	pou5	Object	Narrow	r1	78.4760645	4	1	on_focus	5	Narrow on_focus
**2023408**	block2	Control	pre	pou5	Object	Narrow	r1	92.37778712	5	2	on_focus	5	Narrow on_focus
**2023408**	block2	Control	pre	lok6	Subject	Contrastive	r1	102.3673469	1	1	pre_focus	6	Contrastive pre_focus
**2023408**	block2	Control	pre	lok6	Subject	Contrastive	r1	116.5835223	2	2	pre_focus	6	Contrastive pre_focus
**2023408**	block2	Control	pre	waa6	Verb	Contrastive	r1	141.3320301	3	1	pre_focus	6	Contrastive pre_focus
**2023408**	block2	Control	pre	jyut6	Object	Contrastive	r1	62.31122449	4	1	on_focus	6	Contrastive on_focus
**2023408**	block2	Control	pre	loeng6	Object	Contrastive	r1	240.6848073	5	2	on_focus	6	Contrastive on_focus
**2023408**	block2	Control	pre	lok6	Subject	Contrastive	r2	138.6731998	1	1	on_focus	6	Contrastive on_focus
**2023408**	block2	Control	pre	lok6	Subject	Contrastive	r2	188.9252565	2	2	on_focus	6	Contrastive on_focus
**2023408**	block2	Control	pre	waa6	Verb	Contrastive	r2	110.06783	3	1	post_focus	6	Contrastive post_focus
**2023408**	block2	Control	pre	jyut6	Object	Contrastive	r2	130.1322751	4	1	post_focus	6	Contrastive post_focus
**2023408**	block2	Control	pre	loeng6	Object	Contrastive	r2	224.1467022	5	2	post_focus	6	Contrastive post_focus
**2023408**	block2	Control	pre	ngaa5	Subject	Narrow	r2	115.2659193	1	1	on_focus	5	Narrow on_focus
**2023408**	block2	Control	pre	ngaa5	Subject	Narrow	r2	145.4976064	2	2	on_focus	5	Narrow on_focus
**2023408**	block2	Control	pre	maai5	Verb	Narrow	r2	170.0735244	3	1	post_focus	5	Narrow post_focus
**2023408**	block2	Control	pre	pou5	Object	Narrow	r2	104.3569024	4	1	post_focus	5	Narrow post_focus
**2023408**	block2	Control	pre	pou5	Object	Narrow	r2	50.59449867	5	2	post_focus	5	Narrow post_focus
**2023408**	block2	Control	pre	lok6	Subject	Narrow	r2	99.39185048	1	1	pre_focus	6	Narrow pre_focus
**2023408**	block2	Control	pre	lok6	Subject	Narrow	r2	183.8346291	2	2	pre_focus	6	Narrow pre_focus
**2023408**	block2	Control	pre	waa6	Verb	Narrow	r2	106.1035525	3	1	on_focus	6	Narrow on_focus
**2023408**	block2	Control	pre	jyut6	Object	Narrow	r2	106.4274978	4	1	post_focus	6	Narrow post_focus
**2023408**	block2	Control	pre	loeng6	Object	Narrow	r2	272.5186194	5	2	post_focus	6	Narrow post_focus
**2023408**	block2	Control	pre	ma4	Subject	Contrastive	r2	144.9404035	1	1	pre_focus	4	Contrastive pre_focus
**2023408**	block2	Control	pre	ma4	Subject	Contrastive	r2	251.0734345	2	2	pre_focus	4	Contrastive pre_focus
**2023408**	block2	Control	pre	fu4	Verb	Contrastive	r2	185.394645	3	1	on_focus	4	Contrastive on_focus
**2023408**	block2	Control	pre	maang4	Object	Contrastive	r2	218.8290681	4	1	post_focus	4	Contrastive post_focus
**2023408**	block2	Control	pre	Jan-04	Object	Contrastive	r2	175.3797314	5	2	post_focus	4	Contrastive post_focus
**2023408**	block2	Control	pre	ma4	Subject	Narrow	r2	155.1237638	1	1	pre_focus	4	Narrow pre_focus
**2023408**	block2	Control	pre	ma4	Subject	Narrow	r2	223.4207969	2	2	pre_focus	4	Narrow pre_focus
**2023408**	block2	Control	pre	fu4	Verb	Narrow	r2	149.9949166	3	1	pre_focus	4	Narrow pre_focus
**2023408**	block2	Control	pre	maang4	Object	Narrow	r2	233.3471749	4	1	on_focus	4	Narrow on_focus
**2023408**	block2	Control	pre	Jan-04	Object	Narrow	r2	217.7532124	5	2	on_focus	4	Narrow on_focus
**2023408**	block2	Control	pre	ma4	Subject	Contrastive	r2	191.2918084	1	1	pre_focus	4	Contrastive pre_focus
**2023408**	block2	Control	pre	ma4	Subject	Contrastive	r2	236.2425764	2	2	pre_focus	4	Contrastive pre_focus
**2023408**	block2	Control	pre	fu4	Verb	Contrastive	r2	180.160064	3	1	pre_focus	4	Contrastive pre_focus
**2023408**	block2	Control	pre	maang4	Object	Contrastive	r2	205.3664343	4	1	on_focus	4	Contrastive on_focus
**2023408**	block2	Control	pre	Jan-04	Object	Contrastive	r2	256.3422777	5	2	on_focus	4	Contrastive on_focus

**2023408**	block2	Control	pre	ngaa5	Subject	Narrow	r2	214.683751	1	1	pre_focus	5	Narrow pre_focus
**2023408**	block2	Control	pre	ngaa5	Subject	Narrow	r2	126.4321128	2	2	pre_focus	5	Narrow pre_focus
**2023408**	block2	Control	pre	maai5	Verb	Narrow	r2	246.740058	3	1	on_focus	5	Narrow on_focus
**2023408**	block2	Control	pre	pou5	Object	Narrow	r2	112.9190104	4	1	post_focus	5	Narrow post_focus
**2023408**	block2	Control	pre	pou5	Object	Narrow	r2	219.7347437	5	2	post_focus	5	Narrow post_focus
**2023408**	block2	Control	pre	ma4	Subject	Broad	r2	152.4936668	1	1	broad_focus	4	Broad focus
**2023408**	block2	Control	pre	ma4	Subject	Broad	r2	226.1678702	2	2	broad_focus	4	Broad focus
**2023408**	block2	Control	pre	fu4	Verb	Broad	r2	196.512295	3	1	broad_focus	4	Broad focus
**2023408**	block2	Control	pre	maang4	Object	Broad	r2	189.4188178	4	1	broad_focus	4	Broad focus
**2023408**	block2	Control	pre	Jan-04	Object	Broad	r2	207.0191232	5	2	broad_focus	4	Broad focus
**2023408**	block2	Control	pre	ngaa5	Subject	Contrastive	r2	192.9627308	1	1	pre_focus	5	Contrastive pre_focus
**2023408**	block2	Control	pre	ngaa5	Subject	Contrastive	r2	146.2059645	2	2	pre_focus	5	Contrastive pre_focus
**2023408**	block2	Control	pre	maai5	Verb	Contrastive	r2	291.9707541	3	1	on_focus	5	Contrastive on_focus
**2023408**	block2	Control	pre	pou5	Object	Contrastive	r2	112.3438355	4	1	post_focus	5	Contrastive post_focus
**2023408**	block2	Control	pre	pou5	Object	Contrastive	r2	159.3304611	5	2	post_focus	5	Contrastive post_focus
**2023408**	block2	Control	pre	ngaa5	Subject	Contrastive	r2	143.5604515	1	1	pre_focus	5	Contrastive pre_focus
**2023408**	block2	Control	pre	ngaa5	Subject	Contrastive	r2	151.1040106	2	2	pre_focus	5	Contrastive pre_focus
**2023408**	block2	Control	pre	maai5	Verb	Contrastive	r2	280.371222	3	1	pre_focus	5	Contrastive pre_focus
**2023408**	block2	Control	pre	pou5	Object	Contrastive	r2	87.83083604	4	1	on_focus	5	Contrastive on_focus
**2023408**	block2	Control	pre	pou5	Object	Contrastive	r2	130.7061872	5	2	on_focus	5	Contrastive on_focus
**2023408**	block2	Control	pre	lok6	Subject	Narrow	r2	142.3798186	1	1	pre_focus	6	Narrow pre_focus
**2023408**	block2	Control	pre	lok6	Subject	Narrow	r2	69.19567827	2	2	pre_focus	6	Narrow pre_focus
**2023408**	block2	Control	pre	waa6	Verb	Narrow	r2	75.16674683	3	1	pre_focus	6	Narrow pre_focus
**2023408**	block2	Control	pre	jyut6	Object	Narrow	r2	70.44070295	4	1	on_focus	6	Narrow on_focus
**2023408**	block2	Control	pre	loeng6	Object	Narrow	r2	298.5173557	5	2	on_focus	6	Narrow on_focus
**2023408**	block2	Control	pre	ma4	Subject	Contrastive	r2	121.1294677	1	1	on_focus	4	Contrastive on_focus
**2023408**	block2	Control	pre	ma4	Subject	Contrastive	r2	222.0970522	2	2	on_focus	4	Contrastive on_focus
**2023408**	block2	Control	pre	fu4	Verb	Contrastive	r2	134.457186	3	1	post_focus	4	Contrastive post_focus
**2023408**	block2	Control	pre	maang4	Object	Contrastive	r2	260.2182	4	1	post_focus	4	Contrastive post_focus
**2023408**	block2	Control	pre	Jan-04	Object	Contrastive	r2	198.2993197	5	2	post_focus	4	Contrastive post_focus
**2023408**	block2	Control	pre	ma4	Subject	Narrow	r2	176.7398453	1	1	on_focus	4	Narrow on_focus
**2023408**	block2	Control	pre	ma4	Subject	Narrow	r2	223.8097115	2	2	on_focus	4	Narrow on_focus
**2023408**	block2	Control	pre	fu4	Verb	Narrow	r2	172.6935841	3	1	post_focus	4	Narrow post_focus
**2023408**	block2	Control	pre	maang4	Object	Narrow	r2	245.6801494	4	1	post_focus	4	Narrow post_focus
**2023408**	block2	Control	pre	Jan-04	Object	Narrow	r2	199.3827403	5	2	post_focus	4	Narrow post_focus
**2023408**	block2	Control	pre	ngaa5	Subject	Narrow	r2	97.95609153	1	1	pre_focus	5	Narrow pre_focus
**2023408**	block2	Control	pre	ngaa5	Subject	Narrow	r2	170.2241769	2	2	pre_focus	5	Narrow pre_focus
**2023408**	block2	Control	pre	maai5	Verb	Narrow	r2	211.4658992	3	1	pre_focus	5	Narrow pre_focus
**2023408**	block2	Control	pre	pou5	Object	Narrow	r2	111.6696503	4	1	on_focus	5	Narrow on_focus
**2023408**	block2	Control	pre	pou5	Object	Narrow	r2	213.1860985	5	2	on_focus	5	Narrow on_focus
**2023408**	block2	Control	pre	lok6	Subject	Broad	r2	251.9498042	1	1	broad_focus	6	Broad focus
**2023408**	block2	Control	pre	lok6	Subject	Broad	r2	153.2618309	2	2	broad_focus	6	Broad focus
**2023408**	block2	Control	pre	waa6	Verb	Broad	r2	176.6357319	3	1	broad_focus	6	Broad focus
**2023408**	block2	Control	pre	jyut6	Object	Broad	r2	111.3600791	4	1	broad_focus	6	Broad focus
**2023408**	block2	Control	pre	loeng6	Object	Broad	r2	337.8609221	5	2	broad_focus	6	Broad focus
**2023408**	block2	Control	pre	ngaa5	Subject	Broad	r2	47.28945182	1	1	broad_focus	5	Broad focus
**2023408**	block2	Control	pre	ngaa5	Subject	Broad	r2	137.4196901	2	2	broad_focus	5	Broad focus
**2023408**	block2	Control	pre	maai5	Verb	Broad	r2	224.9294365	3	1	broad_focus	5	Broad focus
**2023408**	block2	Control	pre	pou5	Object	Broad	r2	109.69161	4	1	broad_focus	5	Broad focus
**2023408**	block2	Control	pre	pou5	Object	Broad	r2	97.98245614	5	2	broad_focus	5	Broad focus
**2023408**	block2	Control	pre	lok6	Subject	Contrastive	r2	177.1395692	1	1	pre_focus	6	Contrastive pre_focus
**2023408**	block2	Control	pre	lok6	Subject	Contrastive	r2	177.5931652	2	2	pre_focus	6	Contrastive pre_focus
**2023408**	block2	Control	pre	waa6	Verb	Contrastive	r2	120.9329176	3	1	on_focus	6	Contrastive on_focus
**2023408**	block2	Control	pre	jyut6	Object	Contrastive	r2	108.9335444	4	1	post_focus	6	Contrastive post_focus
**2023408**	block2	Control	pre	loeng6	Object	Contrastive	r2	407.9085142	5	2	post_focus	6	Contrastive post_focus
**2023408**	block2	Control	pre	lok6	Subject	Narrow	r2	142.8945811	1	1	on_focus	6	Narrow on_focus
**2023408**	block2	Control	pre	lok6	Subject	Narrow	r2	136.1680524	2	2	on_focus	6	Narrow on_focus
**2023408**	block2	Control	pre	waa6	Verb	Narrow	r2	112.7324811	3	1	post_focus	6	Narrow post_focus
**2023408**	block2	Control	pre	jyut6	Object	Narrow	r2	84.08044218	4	1	post_focus	6	Narrow post_focus
**2023408**	block2	Control	pre	loeng6	Object	Narrow	r2	303.5950902	5	2	post_focus	6	Narrow post_focus
**2023408**	block2	Control	pre	ma4	Subject	Narrow	r2	111.5319828	1	1	pre_focus	4	Narrow pre_focus
**2023408**	block2	Control	pre	ma4	Subject	Narrow	r2	181.0939535	2	2	pre_focus	4	Narrow pre_focus
**2023408**	block2	Control	pre	fu4	Verb	Narrow	r2	182.1133787	3	1	on_focus	4	Narrow on_focus
**2023408**	block2	Control	pre	maang4	Object	Narrow	r2	217.5717765	4	1	post_focus	4	Narrow post_focus
**2023408**	block2	Control	pre	Jan-04	Object	Narrow	r2	190.845967	5	2	post_focus	4	Narrow post_focus
**2023408**	block2	Control	pre	ngaa5	Subject	Contrastive	r2	114.0808673	1	1	on_focus	5	Contrastive on_focus

2023408	block2	Control	pre	ngaa5	Subject	Contrastive	r2	164.478836	2	2	on_focus	5	Contrastive on_focus
2023408	block2	Control	pre	maai5	Verb	Contrastive	r2	167.1861522	3	1	post_focus	5	Contrastive post_focus
2023408	block2	Control	pre	pou5	Object	Contrastive	r2	94.0818617	4	1	post_focus	5	Contrastive post_focus
2023408	block2	Control	pre	pou5	Object	Contrastive	r2	209.2832847	5	2	post_focus	5	Contrastive post_focus
2023408	block2	Control	pre	lok6	Subject	Contrastive	r2	90.95616024	1	1	pre_focus	6	Contrastive pre_focus
2023408	block2	Control	pre	lok6	Subject	Contrastive	r2	147.3346915	2	2	pre_focus	6	Contrastive pre_focus
2023408	block2	Control	pre	waa6	Verb	Contrastive	r2	87.74166457	3	1	pre_focus	6	Contrastive pre_focus
2023408	block2	Control	pre	jyut6	Object	Contrastive	r2	138.057689	4	1	on_focus	6	Contrastive on_focus
2023408	block2	Control	pre	loeng6	Object	Contrastive	r2	296.2622827	5	2	on_focus	6	Contrastive on_focus
2023408	block3	Control	post	piu2	Subject	Contrastive	r1	116.6226895	1	1	pre_focus	2	Contrastive pre_focus
2023408	block3	Control	post	tse2	Subject	Contrastive	r1	134.9807671	2	2	pre_focus	2	Contrastive pre_focus
2023408	block3	Control	post	tsap1	Verb	Contrastive	r1	49.74159955	3	1	on_focus	1	Contrastive on_focus
2023408	block3	Control	post	sy1	Object	Contrastive	r1	110.7003896	4	1	post_focus	1	Contrastive post_focus
2023408	block3	Control	post	pau1	Object	Contrastive	r1	226.4430924	5	2	post_focus	1	Contrastive post_focus
2023408	block3	Control	post	suk1	Subject	Contrastive	r1	39.39794275	1	1	pre_focus	1	Contrastive pre_focus
2023408	block3	Control	post	suk1	Subject	Contrastive	r1	60.21085167	2	2	pre_focus	1	Contrastive pre_focus
2023408	block3	Control	post	sei2	Verb	Contrastive	r1	149.4329888	3	1	pre_focus	2	Contrastive pre_focus
2023408	block3	Control	post	svy2	Object	Contrastive	r1	180.6478046	4	1	on_focus	2	Contrastive on_focus
2023408	block3	Control	post	kwo2	Object	Contrastive	r1	260.9437426	5	2	on_focus	2	Contrastive on_focus
2023408	block3	Control	post	bui3	Subject	Narrow	r1	176.166771	1	1	on_focus	3	Narrow on_focus
2023408	block3	Control	post	bui3	Subject	Narrow	r1	141.4746788	2	2	on_focus	3	Narrow on_focus
2023408	block3	Control	post	tsv1	Verb	Narrow	r1	115.1747921	3	1	post_focus	1	Narrow post_focus
2023408	block3	Control	post	fug1	Object	Narrow	r1	186.4671364	4	1	post_focus	1	Narrow post_focus
2023408	block3	Control	post	tshe1	Object	Narrow	r1	107.1087709	5	2	post_focus	1	Narrow post_focus
2023408	block3	Control	post	suk1	Subject	Narrow	r1	59.50445956	1	1	on_focus	1	Narrow on_focus
2023408	block3	Control	post	suk1	Subject	Narrow	r1	56.35116115	2	2	on_focus	1	Narrow on_focus
2023408	block3	Control	post	sei2	Verb	Narrow	r1	149.8194939	3	1	post_focus	2	Narrow post_focus
2023408	block3	Control	post	svy2	Object	Narrow	r1	174.2185731	4	1	post_focus	2	Narrow post_focus
2023408	block3	Control	post	kwo2	Object	Narrow	r1	181.6121687	5	2	post_focus	2	Narrow post_focus
2023408	block3	Control	post	suk1	Subject	Narrow	r1	30.10537548	1	1	pre_focus	1	Narrow pre_focus
2023408	block3	Control	post	suk1	Subject	Narrow	r1	47.72434209	2	2	pre_focus	1	Narrow pre_focus
2023408	block3	Control	post	sei2	Verb	Narrow	r1	167.5832474	3	1	on_focus	2	Narrow on_focus
2023408	block3	Control	post	svy2	Object	Narrow	r1	163.9741749	4	1	post_focus	2	Narrow post_focus
2023408	block3	Control	post	kwo2	Object	Narrow	r1	175.3769841	5	2	post_focus	2	Narrow post_focus
2023408	block3	Control	post	suk1	Subject	Contrastive	r1	57.25037279	1	1	pre_focus	1	Contrastive pre_focus
2023408	block3	Control	post	suk1	Subject	Contrastive	r1	62.20918367	2	2	pre_focus	1	Contrastive pre_focus
2023408	block3	Control	post	sei2	Verb	Contrastive	r1	155.3429851	3	1	on_focus	2	Contrastive on_focus
2023408	block3	Control	post	svy2	Object	Contrastive	r1	167.0311023	4	1	post_focus	2	Contrastive post_focus
2023408	block3	Control	post	kwo2	Object	Contrastive	r1	204.2466422	5	2	post_focus	2	Contrastive post_focus
2023408	block3	Control	post	piu2	Subject	Narrow	r1	119.1549104	1	1	pre_focus	2	Narrow pre_focus
2023408	block3	Control	post	tse2	Subject	Narrow	r1	140.5139834	2	2	pre_focus	2	Narrow pre_focus
2023408	block3	Control	post	tsap1	Verb	Narrow	r1	31.27362056	3	1	on_focus	1	Narrow on_focus
2023408	block3	Control	post	sy1	Object	Narrow	r1	93.03463203	4	1	post_focus	1	Narrow post_focus
2023408	block3	Control	post	pau1	Object	Narrow	r1	236.3673469	5	2	post_focus	1	Narrow post_focus
2023408	block3	Control	post	piu2	Subject	Broad	r1	141.7457737	1	1	broad_focus	2	Broad focus
2023408	block3	Control	post	tse2	Subject	Broad	r1	152.5542136	2	2	broad_focus	2	Broad focus
2023408	block3	Control	post	tsap1	Verb	Broad	r1	49.23335284	3	1	broad_focus	1	Broad focus
2023408	block3	Control	post	sy1	Object	Broad	r1	96.99496095	4	1	broad_focus	1	Broad focus
2023408	block3	Control	post	pau1	Object	Broad	r1	273.5207416	5	2	broad_focus	1	Broad focus
2023408	block3	Control	post	piu2	Subject	Contrastive	r1	137.0790816	1	1	on_focus	2	Contrastive on_focus
2023408	block3	Control	post	tse2	Subject	Contrastive	r1	166.8807904	2	2	on_focus	2	Contrastive on_focus
2023408	block3	Control	post	tsap1	Verb	Contrastive	r1	60.72058453	3	1	post_focus	1	Contrastive post_focus
2023408	block3	Control	post	sy1	Object	Contrastive	r1	83.75971817	4	1	post_focus	1	Contrastive post_focus
2023408	block3	Control	post	pau1	Object	Contrastive	r1	238.1089576	5	2	post_focus	1	Contrastive post_focus
2023408	block3	Control	post	suk1	Subject	Broad	r1	62.03653313	1	1	broad_focus	1	Broad focus
2023408	block3	Control	post	suk1	Subject	Broad	r1	61.84126984	2	2	broad_focus	1	Broad focus
2023408	block3	Control	post	sei2	Verb	Broad	r1	145.9348211	3	1	broad_focus	2	Broad focus
2023408	block3	Control	post	svy2	Object	Broad	r1	171.6074763	4	1	broad_focus	2	Broad focus
2023408	block3	Control	post	kwo2	Object	Broad	r1	60.31539889	5	2	broad_focus	2	Broad focus
2023408	block3	Control	post	bui3	Subject	Contrastive	r1	130.9075964	1	1	on_focus	3	Contrastive on_focus
2023408	block3	Control	post	bui3	Subject	Contrastive	r1	142.0870981	2	2	on_focus	3	Contrastive on_focus
2023408	block3	Control	post	tsv1	Verb	Contrastive	r1	120.7149239	3	1	post_focus	1	Contrastive post_focus
2023408	block3	Control	post	fug1	Object	Contrastive	r1	181.2333081	4	1	post_focus	1	Contrastive post_focus
2023408	block3	Control	post	tshe1	Object	Contrastive	r1	151.8495708	5	2	post_focus	1	Contrastive post_focus
2023408	block3	Control	post	bui3	Subject	Contrastive	r1	133.0655707	1	1	pre_focus	3	Contrastive pre_focus
2023408	block3	Control	post	bui3	Subject	Contrastive	r1	123.1875607	2	2	pre_focus	3	Contrastive pre_focus

2023408	block3	Control	post	tsv1	Verb	Contrastive	r1	96.69311224	3	1	pre_focus	1	Contrastive pre_focus
2023408	block3	Control	post	fug1	Object	Contrastive	r1	178.7357017	4	1	on_focus	1	Contrastive on_focus
2023408	block3	Control	post	tshe1	Object	Contrastive	r1	147.8479646	5	2	on_focus	1	Contrastive on_focus
2023408	block3	Control	post	bui3	Subject	Broad	r1	125.1023392	1	1	broad_focus	3	Broad focus
2023408	block3	Control	post	bui3	Subject	Broad	r1	90.63462614	2	2	broad_focus	3	Broad focus
2023408	block3	Control	post	tsv1	Verb	Broad	r1	96.70774158	3	1	broad_focus	1	Broad focus
2023408	block3	Control	post	fug1	Object	Broad	r1	193.7282691	4	1	broad_focus	1	Broad focus
2023408	block3	Control	post	tshe1	Object	Broad	r1	70.91747913	5	2	broad_focus	1	Broad focus
2023408	block3	Control	post	suk1	Subject	Narrow	r1	66.54956268	1	1	pre_focus	1	Narrow pre_focus
2023408	block3	Control	post	suk1	Subject	Narrow	r1	58.6196955	2	2	pre_focus	1	Narrow pre_focus
2023408	block3	Control	post	sei2	Verb	Narrow	r1	145.3072455	3	1	pre_focus	2	Narrow pre_focus
2023408	block3	Control	post	svy2	Object	Narrow	r1	171.3340892	4	1	on_focus	2	Narrow on_focus
2023408	block3	Control	post	kwo2	Object	Narrow	r1	150.4609702	5	2	on_focus	2	Narrow on_focus
2023408	block3	Control	post	piu2	Subject	Narrow	r1	169.8173255	1	1	on_focus	2	Narrow on_focus
2023408	block3	Control	post	tse2	Subject	Narrow	r1	130.5868697	2	2	on_focus	2	Narrow on_focus
2023408	block3	Control	post	tsap1	Verb	Narrow	r1	53.38435374	3	1	post_focus	1	Narrow post_focus
2023408	block3	Control	post	sy1	Object	Narrow	r1	125.9877238	4	1	post_focus	1	Narrow post_focus
2023408	block3	Control	post	pau1	Object	Narrow	r1	137.0555556	5	2	post_focus	1	Narrow post_focus
2023408	block3	Control	post	suk1	Subject	Contrastive	r1	55.16984177	1	1	on_focus	1	Contrastive on_focus
2023408	block3	Control	post	suk1	Subject	Contrastive	r1	48.08368427	2	2	on_focus	1	Contrastive on_focus
2023408	block3	Control	post	sei2	Verb	Contrastive	r1	198.7331822	3	1	post_focus	2	Contrastive post_focus
2023408	block3	Control	post	svy2	Object	Contrastive	r1	194.1266507	4	1	post_focus	2	Contrastive post_focus
2023408	block3	Control	post	kwo2	Object	Contrastive	r1	177.1156463	5	2	post_focus	2	Contrastive post_focus
2023408	block3	Control	post	bui3	Subject	Narrow	r1	133.4372638	1	1	pre_focus	3	Narrow pre_focus
2023408	block3	Control	post	bui3	Subject	Narrow	r1	117.245671	2	2	pre_focus	3	Narrow pre_focus
2023408	block3	Control	post	tsv1	Verb	Narrow	r1	101.2370244	3	1	on_focus	1	Narrow on_focus
2023408	block3	Control	post	fug1	Object	Narrow	r1	203.6615916	4	1	post_focus	1	Narrow post_focus
2023408	block3	Control	post	tshe1	Object	Narrow	r1	141.8457053	5	2	post_focus	1	Narrow post_focus
2023408	block3	Control	post	piu2	Subject	Contrastive	r1	132.8874312	1	1	pre_focus	2	Contrastive pre_focus
2023408	block3	Control	post	tse2	Subject	Contrastive	r1	157.6981535	2	2	pre_focus	2	Contrastive pre_focus
2023408	block3	Control	post	tsap1	Verb	Contrastive	r1	79.15319974	3	1	pre_focus	1	Contrastive pre_focus
2023408	block3	Control	post	sy1	Object	Contrastive	r1	105.542598	4	1	on_focus	1	Contrastive on_focus
2023408	block3	Control	post	pau1	Object	Contrastive	r1	238.060024	5	2	on_focus	1	Contrastive on_focus
2023408	block3	Control	post	bui3	Subject	Narrow	r1	150.0431163	1	1	pre_focus	3	Narrow pre_focus
2023408	block3	Control	post	bui3	Subject	Narrow	r1	154.5676493	2	2	pre_focus	3	Narrow pre_focus
2023408	block3	Control	post	tsv1	Verb	Narrow	r1	127.786428	3	1	pre_focus	1	Narrow pre_focus
2023408	block3	Control	post	fug1	Object	Narrow	r1	190.7505669	4	1	on_focus	1	Narrow on_focus
2023408	block3	Control	post	tshe1	Object	Narrow	r1	129.38322	5	2	on_focus	1	Narrow on_focus
2023408	block3	Control	post	bui3	Subject	Contrastive	r1	151.1012521	1	1	pre_focus	3	Contrastive pre_focus
2023408	block3	Control	post	bui3	Subject	Contrastive	r1	109.6212312	2	2	pre_focus	3	Contrastive pre_focus
2023408	block3	Control	post	tsv1	Verb	Contrastive	r1	84.75678271	3	1	on_focus	1	Contrastive on_focus
2023408	block3	Control	post	fug1	Object	Contrastive	r1	199.7510479	4	1	post_focus	1	Contrastive post_focus
2023408	block3	Control	post	tshe1	Object	Contrastive	r1	117.3527337	5	2	post_focus	1	Contrastive post_focus
2023408	block3	Control	post	piu2	Subject	Narrow	r1	128.2022664	1	1	pre_focus	2	Narrow pre_focus
2023408	block3	Control	post	tse2	Subject	Narrow	r1	132.4323551	2	2	pre_focus	2	Narrow pre_focus
2023408	block3	Control	post	tsap1	Verb	Narrow	r1	40.65665155	3	1	pre_focus	1	Narrow pre_focus
2023408	block3	Control	post	sy1	Object	Narrow	r1	105.0226757	4	1	on_focus	1	Narrow on_focus
2023408	block3	Control	post	pau1	Object	Narrow	r1	257.1153341	5	2	on_focus	1	Narrow on_focus
2023408	block3	Control	post	bui3	Subject	Contrastive	r2	113.6995645	1	1	pre_focus	3	Contrastive pre_focus
2023408	block3	Control	post	bui3	Subject	Contrastive	r2	88.77508886	2	2	pre_focus	3	Contrastive pre_focus
2023408	block3	Control	post	tsv1	Verb	Contrastive	r2	80.85876255	3	1	on_focus	1	Contrastive on_focus
2023408	block3	Control	post	fug1	Object	Contrastive	r2	114.4375007	4	1	post_focus	1	Contrastive post_focus
2023408	block3	Control	post	tshe1	Object	Contrastive	r2	164.4578774	5	2	post_focus	1	Contrastive post_focus
2023408	block3	Control	post	suk1	Subject	Contrastive	r2	61.88249109	1	1	pre_focus	1	Contrastive pre_focus
2023408	block3	Control	post	suk1	Subject	Contrastive	r2	61.22912801	2	2	pre_focus	1	Contrastive pre_focus
2023408	block3	Control	post	sei2	Verb	Contrastive	r2	147.1066168	3	1	on_focus	2	Contrastive on_focus
2023408	block3	Control	post	svy2	Object	Contrastive	r2	150.0132275	4	1	post_focus	2	Contrastive post_focus
2023408	block3	Control	post	kwo2	Object	Contrastive	r2	121.8559011	5	2	post_focus	2	Contrastive post_focus
2023408	block3	Control	post	piu2	Subject	Contrastive	r2	109.3092154	1	1	pre_focus	2	Contrastive pre_focus
2023408	block3	Control	post	tse2	Subject	Contrastive	r2	141.256058	2	2	pre_focus	2	Contrastive pre_focus
2023408	block3	Control	post	tsap1	Verb	Contrastive	r2	55.69023569	3	1	pre_focus	1	Contrastive pre_focus
2023408	block3	Control	post	sy1	Object	Contrastive	r2	90.46343537	4	1	on_focus	1	Contrastive on_focus
2023408	block3	Control	post	pau1	Object	Contrastive	r2	226.3422777	5	2	on_focus	1	Contrastive on_focus
2023408	block3	Control	post	piu2	Subject	Narrow	r2	118.5926703	1	1	pre_focus	2	Narrow pre_focus
2023408	block3	Control	post	tse2	Subject	Narrow	r2	84.25	2	2	pre_focus	2	Narrow pre_focus
2023408	block3	Control	post	tsap1	Verb	Narrow	r2	48.73825721	3	1	on_focus	1	Narrow on_focus

**2023408**	block3	Control	post	sy1	Object	Narrow	r2	108.4514035	4	1	post_focus	1	Narrow post_focus
**2023408**	block3	Control	post	pau1	Object	Narrow	r2	133.5830904	5	2	post_focus	1	Narrow post_focus
**2023408**	block3	Control	post	suk1	Subject	Narrow	r2	57.40551776	1	1	pre_focus	1	Narrow pre_focus
**2023408**	block3	Control	post	suk1	Subject	Narrow	r2	56.06802721	2	2	pre_focus	1	Narrow pre_focus
**2023408**	block3	Control	post	sei2	Verb	Narrow	r2	106.306078	3	1	pre_focus	2	Narrow pre_focus
**2023408**	block3	Control	post	svy2	Object	Narrow	r2	161.2386317	4	1	on_focus	2	Narrow on_focus
**2023408**	block3	Control	post	kwo2	Object	Narrow	r2	122.7100413	5	2	on_focus	2	Narrow on_focus
**2023408**	block3	Control	post	piu2	Subject	Contrastive	r2	114.1072478	1	1	pre_focus	2	Contrastive pre_focus
**2023408**	block3	Control	post	tse2	Subject	Contrastive	r2	106.098114	2	2	pre_focus	2	Contrastive pre_focus
**2023408**	block3	Control	post	tsap1	Verb	Contrastive	r2	39.87876106	3	1	on_focus	1	Contrastive on_focus
**2023408**	block3	Control	post	sy1	Object	Contrastive	r2	102.2714983	4	1	post_focus	1	Contrastive post_focus
**2023408**	block3	Control	post	pau1	Object	Contrastive	r2	196.088209	5	2	post_focus	1	Contrastive post_focus
**2023408**	block3	Control	post	bui3	Subject	Contrastive	r2	105.6019074	1	1	pre_focus	3	Contrastive pre_focus
**2023408**	block3	Control	post	bui3	Subject	Contrastive	r2	121.2414292	2	2	pre_focus	3	Contrastive pre_focus
**2023408**	block3	Control	post	tsv1	Verb	Contrastive	r2	84.63791072	3	1	pre_focus	1	Contrastive pre_focus
**2023408**	block3	Control	post	fug1	Object	Contrastive	r2	182.6478284	4	1	on_focus	1	Contrastive on_focus
**2023408**	block3	Control	post	tshe1	Object	Contrastive	r2	147.6158082	5	2	on_focus	1	Contrastive on_focus
**2023408**	block3	Control	post	piu2	Subject	Narrow	r2	120.7203402	1	1	on_focus	2	Narrow on_focus
**2023408**	block3	Control	post	tse2	Subject	Narrow	r2	108.2006604	2	2	on_focus	2	Narrow on_focus
**2023408**	block3	Control	post	tsap1	Verb	Narrow	r2	63.56478618	3	1	post_focus	1	Narrow post_focus
**2023408**	block3	Control	post	sy1	Object	Narrow	r2	107.7158556	4	1	post_focus	1	Narrow post_focus
**2023408**	block3	Control	post	pau1	Object	Narrow	r2	176.108298	5	2	post_focus	1	Narrow post_focus
**2023408**	block3	Control	post	bui3	Subject	Narrow	r2	76.36432351	1	1	pre_focus	3	Narrow pre_focus
**2023408**	block3	Control	post	bui3	Subject	Narrow	r2	124.3338358	2	2	pre_focus	3	Narrow pre_focus
**2023408**	block3	Control	post	tsv1	Verb	Narrow	r2	98.75137429	3	1	on_focus	1	Narrow on_focus
**2023408**	block3	Control	post	fug1	Object	Narrow	r2	191.1548429	4	1	post_focus	1	Narrow post_focus
**2023408**	block3	Control	post	tshe1	Object	Narrow	r2	110.776509	5	2	post_focus	1	Narrow post_focus
**2023408**	block3	Control	post	suk1	Subject	Narrow	r2	40.50080431	1	1	on_focus	1	Narrow on_focus
**2023408**	block3	Control	post	suk1	Subject	Narrow	r2	67.51669557	2	2	on_focus	1	Narrow on_focus
**2023408**	block3	Control	post	sei2	Verb	Narrow	r2	160.0570133	3	1	post_focus	2	Narrow post_focus
**2023408**	block3	Control	post	svy2	Object	Narrow	r2	147.4932473	4	1	post_focus	2	Narrow post_focus
**2023408**	block3	Control	post	kwo2	Object	Narrow	r2	89.58805745	5	2	post_focus	2	Narrow post_focus
**2023408**	block3	Control	post	bui3	Subject	Contrastive	r2	141.1560847	1	1	on_focus	3	Contrastive on_focus
**2023408**	block3	Control	post	bui3	Subject	Contrastive	r2	126.6601256	2	2	on_focus	3	Contrastive on_focus
**2023408**	block3	Control	post	tsv1	Verb	Contrastive	r2	91.50260104	3	1	post_focus	1	Contrastive post_focus
**2023408**	block3	Control	post	fug1	Object	Contrastive	r2	160.1814059	4	1	post_focus	1	Contrastive post_focus
**2023408**	block3	Control	post	tshe1	Object	Contrastive	r2	144.0177283	5	2	post_focus	1	Contrastive post_focus
**2023408**	block3	Control	post	piu2	Subject	Contrastive	r2	169.218227	1	1	on_focus	2	Contrastive on_focus
**2023408**	block3	Control	post	tse2	Subject	Contrastive	r2	123.1644566	2	2	on_focus	2	Contrastive on_focus
**2023408**	block3	Control	post	tsap1	Verb	Contrastive	r2	54.90083434	3	1	post_focus	1	Contrastive post_focus
**2023408**	block3	Control	post	sy1	Object	Contrastive	r2	132.5722022	4	1	post_focus	1	Contrastive post_focus
**2023408**	block3	Control	post	pau1	Object	Contrastive	r2	204.1782313	5	2	post_focus	1	Contrastive post_focus
**2023408**	block3	Control	post	bui3	Subject	Narrow	r2	159.7583478	1	1	pre_focus	3	Narrow pre_focus
**2023408**	block3	Control	post	bui3	Subject	Narrow	r2	145.1956072	2	2	pre_focus	3	Narrow pre_focus
**2023408**	block3	Control	post	tsv1	Verb	Narrow	r2	121.5954116	3	1	pre_focus	1	Narrow pre_focus
**2023408**	block3	Control	post	fug1	Object	Narrow	r2	209.7911913	4	1	on_focus	1	Narrow on_focus
**2023408**	block3	Control	post	tshe1	Object	Narrow	r2	95.98218335	5	2	on_focus	1	Narrow on_focus
**2023408**	block3	Control	post	piu2	Subject	Narrow	r2	126.2518053	1	1	pre_focus	2	Narrow pre_focus
**2023408**	block3	Control	post	tse2	Subject	Narrow	r2	126.5433179	2	2	pre_focus	2	Narrow pre_focus
**2023408**	block3	Control	post	tsap1	Verb	Narrow	r2	30.90419501	3	1	pre_focus	1	Narrow pre_focus
**2023408**	block3	Control	post	sy1	Object	Narrow	r2	69.43335576	4	1	on_focus	1	Narrow on_focus
**2023408**	block3	Control	post	pau1	Object	Narrow	r2	272.836393	5	2	on_focus	1	Narrow on_focus
**2023408**	block3	Control	post	piu2	Subject	Broad	r2	102.1209373	1	1	broad_focus	2	Broad focus
**2023408**	block3	Control	post	tse2	Subject	Broad	r2	111.092379	2	2	broad_focus	2	Broad focus
**2023408**	block3	Control	post	tsap1	Verb	Broad	r2	53.65362812	3	1	broad_focus	1	Broad focus
**2023408**	block3	Control	post	sy1	Object	Broad	r2	93.51230969	4	1	broad_focus	1	Broad focus
**2023408**	block3	Control	post	pau1	Object	Broad	r2	69.55366591	5	2	broad_focus	1	Broad focus
**2023408**	block3	Control	post	suk1	Subject	Contrastive	r2	46.10772448	1	1	on_focus	1	Contrastive on_focus
**2023408**	block3	Control	post	suk1	Subject	Contrastive	r2	59.6085286	2	2	on_focus	1	Contrastive on_focus
**2023408**	block3	Control	post	sei2	Verb	Contrastive	r2	146.2312925	3	1	post_focus	2	Contrastive post_focus
**2023408**	block3	Control	post	svy2	Object	Contrastive	r2	157.9521233	4	1	post_focus	2	Contrastive post_focus
**2023408**	block3	Control	post	kwo2	Object	Contrastive	r2	150.2341442	5	2	post_focus	2	Contrastive post_focus
**2023408**	block3	Control	post	suk1	Subject	Broad	r2	47.40101827	1	1	broad_focus	1	Broad focus
**2023408**	block3	Control	post	suk1	Subject	Broad	r2	45.84727496	2	2	broad_focus	1	Broad focus
**2023408**	block3	Control	post	sei2	Verb	Broad	r2	123.7428256	3	1	broad_focus	2	Broad focus
**2023408**	block3	Control	post	svy2	Object	Broad	r2	144.4095734	4	1	broad_focus	2	Broad focus

2023408	block3	Control	post	kwo2	Object	Broad	r2	122.4554584	5	2	broad_focus	2	Broad focus
2023408	block3	Control	post	bui3	Subject	Narrow	r2	153.2334521	1	1	on_focus	3	Narrow on_focus
2023408	block3	Control	post	bui3	Subject	Narrow	r2	108.3710726	2	2	on_focus	3	Narrow on_focus
2023408	block3	Control	post	tsv1	Verb	Narrow	r2	84.04754543	3	1	post_focus	1	Narrow post_focus
2023408	block3	Control	post	fug1	Object	Narrow	r2	179.3206893	4	1	post_focus	1	Narrow post_focus
2023408	block3	Control	post	tshe1	Object	Narrow	r2	217.9768204	5	2	post_focus	1	Narrow post_focus
2023408	block3	Control	post	suk1	Subject	Narrow	r2	39.73759201	1	1	pre_focus	1	Narrow pre_focus
2023408	block3	Control	post	suk1	Subject	Narrow	r2	45.95409881	2	2	pre_focus	1	Narrow pre_focus
2023408	block3	Control	post	sei2	Verb	Narrow	r2	142.033779	3	1	on_focus	2	Narrow on_focus
2023408	block3	Control	post	svy2	Object	Narrow	r2	160.0151172	4	1	post_focus	2	Narrow post_focus
2023408	block3	Control	post	kwo2	Object	Narrow	r2	99.96112731	5	2	post_focus	2	Narrow post_focus
2023408	block3	Control	post	bui3	Subject	Broad	r2	158.4822408	1	1	broad_focus	3	Broad focus
2023408	block3	Control	post	bui3	Subject	Broad	r2	106.6484776	2	2	broad_focus	3	Broad focus
2023408	block3	Control	post	tsv1	Verb	Broad	r2	105.5071554	3	1	broad_focus	1	Broad focus
2023408	block3	Control	post	fug1	Object	Broad	r2	190.9935212	4	1	broad_focus	1	Broad focus
2023408	block3	Control	post	tshe1	Object	Broad	r2	93.74420509	5	2	broad_focus	1	Broad focus
2023408	block3	Control	post	suk1	Subject	Contrastive	r2	62.02097506	1	1	pre_focus	1	Contrastive pre_focus
2023408	block3	Control	post	suk1	Subject	Contrastive	r2	72.21963302	2	2	pre_focus	1	Contrastive pre_focus
2023408	block3	Control	post	sei2	Verb	Contrastive	r2	134.9671493	3	1	pre_focus	2	Contrastive pre_focus
2023408	block3	Control	post	svy2	Object	Contrastive	r2	122.1753643	4	1	on_focus	2	Contrastive on_focus
2023408	block3	Control	post	kwo2	Object	Contrastive	r2	161.6587103	5	2	on_focus	2	Contrastive on_focus
2023408	block3	Control	pre	piu2	Subject	Broad	r1	192.510441	1	1	broad_focus	2	Broad focus
2023408	block3	Control	pre	tse2	Subject	Broad	r1	175.7753842	2	2	broad_focus	2	Broad focus
2023408	block3	Control	pre	tsap1	Verb	Broad	r1	65.91400162	3	1	broad_focus	1	Broad focus
2023408	block3	Control	pre	sy1	Object	Broad	r1	194.6889059	4	1	broad_focus	1	Broad focus
2023408	block3	Control	pre	pau1	Object	Broad	r1	334.9769631	5	2	broad_focus	1	Broad focus
2023408	block3	Control	pre	suk1	Subject	Narrow	r1	27.54192006	1	1	on_focus	1	Narrow on_focus
2023408	block3	Control	pre	suk1	Subject	Narrow	r1	54.59139235	2	2	on_focus	1	Narrow on_focus
2023408	block3	Control	pre	sei2	Verb	Narrow	r1	244.0361202	3	1	post_focus	2	Narrow post_focus
2023408	block3	Control	pre	svy2	Object	Narrow	r1	233.5066334	4	1	post_focus	2	Narrow post_focus
2023408	block3	Control	pre	kwo2	Object	Narrow	r1	244.1308182	5	2	post_focus	2	Narrow post_focus
2023408	block3	Control	pre	bui3	Subject	Contrastive	r1	184.3210719	1	1	pre_focus	3	Contrastive pre_focus
2023408	block3	Control	pre	bui3	Subject	Contrastive	r1	158.3053617	2	2	pre_focus	3	Contrastive pre_focus
2023408	block3	Control	pre	tsv1	Verb	Contrastive	r1	335.0951427	3	1	pre_focus	1	Contrastive pre_focus
2023408	block3	Control	pre	fug1	Object	Contrastive	r1	252.3129416	4	1	on_focus	1	Contrastive on_focus
2023408	block3	Control	pre	tshe1	Object	Contrastive	r1	253.3618727	5	2	on_focus	1	Contrastive on_focus
2023408	block3	Control	pre	bui3	Subject	Narrow	r1	181.3994254	1	1	pre_focus	3	Narrow pre_focus
2023408	block3	Control	pre	bui3	Subject	Narrow	r1	224.044076	2	2	pre_focus	3	Narrow pre_focus
2023408	block3	Control	pre	tsv1	Verb	Narrow	r1	516.036568	3	1	pre_focus	1	Narrow pre_focus
2023408	block3	Control	pre	fug1	Object	Narrow	r1	274.668526	4	1	on_focus	1	Narrow on_focus
2023408	block3	Control	pre	tshe1	Object	Narrow	r1	239.6861201	5	2	on_focus	1	Narrow on_focus
2023408	block3	Control	pre	bui3	Subject	Broad	r1	207.6503212	1	1	broad_focus	3	Broad focus
2023408	block3	Control	pre	bui3	Subject	Broad	r1	216.8343591	2	2	broad_focus	3	Broad focus
2023408	block3	Control	pre	tsv1	Verb	Broad	r1	602.0158215	3	1	broad_focus	1	Broad focus
2023408	block3	Control	pre	fug1	Object	Broad	r1	207.4932595	4	1	broad_focus	1	Broad focus
2023408	block3	Control	pre	tshe1	Object	Broad	r1	184.861678	5	2	broad_focus	1	Broad focus
2023408	block3	Control	pre	suk1	Subject	Contrastive	r1	59.39321579	1	1	on_focus	1	Contrastive on_focus
2023408	block3	Control	pre	suk1	Subject	Contrastive	r1	59.36540238	2	2	on_focus	1	Contrastive on_focus
2023408	block3	Control	pre	sei2	Verb	Contrastive	r1	210.1824857	3	1	post_focus	2	Contrastive post_focus
2023408	block3	Control	pre	svy2	Object	Contrastive	r1	223.3762537	4	1	post_focus	2	Contrastive post_focus
2023408	block3	Control	pre	kwo2	Object	Contrastive	r1	204.7193989	5	2	post_focus	2	Contrastive post_focus
2023408	block3	Control	pre	piu2	Subject	Contrastive	r1	139.2182685	1	1	on_focus	2	Contrastive on_focus
2023408	block3	Control	pre	tse2	Subject	Contrastive	r1	139.6050642	2	2	on_focus	2	Contrastive on_focus
2023408	block3	Control	pre	tsap1	Verb	Contrastive	r1	81.85676493	3	1	post_focus	1	Contrastive post_focus
2023408	block3	Control	pre	sy1	Object	Contrastive	r1	125.6027967	4	1	post_focus	1	Contrastive post_focus
2023408	block3	Control	pre	pau1	Object	Contrastive	r1	307.6344347	5	2	post_focus	1	Contrastive post_focus
2023408	block3	Control	pre	bui3	Subject	Contrastive	r1	140.6364741	1	1	pre_focus	3	Contrastive pre_focus
2023408	block3	Control	pre	bui3	Subject	Contrastive	r1	133.7113128	2	2	pre_focus	3	Contrastive pre_focus
2023408	block3	Control	pre	tsv1	Verb	Contrastive	r1	133.500889	3	1	on_focus	1	Contrastive on_focus
2023408	block3	Control	pre	fug1	Object	Contrastive	r1	188.2374768	4	1	post_focus	1	Contrastive post_focus
2023408	block3	Control	pre	tshe1	Object	Contrastive	r1	147.8235873	5	2	post_focus	1	Contrastive post_focus
2023408	block3	Control	pre	piu2	Subject	Narrow	r1	126.6989759	1	1	pre_focus	2	Narrow pre_focus
2023408	block3	Control	pre	tse2	Subject	Narrow	r1	160.4807796	2	2	pre_focus	2	Narrow pre_focus
2023408	block3	Control	pre	tsap1	Verb	Narrow	r1	43.58086036	3	1	pre_focus	1	Narrow pre_focus
2023408	block3	Control	pre	sy1	Object	Narrow	r1	126.2655062	4	1	on_focus	1	Narrow on_focus
2023408	block3	Control	pre	pau1	Object	Narrow	r1	163.3056212	5	2	on_focus	1	Narrow on_focus

2023408	block3	Control	pre	bui3	Subject	Contrastive	r1	138.2645786	1	1	on_focus	3	Contrastive on_focus
2023408	block3	Control	pre	bui3	Subject	Contrastive	r1	141.1355874	2	2	on_focus	3	Contrastive on_focus
2023408	block3	Control	pre	tsv1	Verb	Contrastive	r1	129.3316956	3	1	post_focus	1	Contrastive post_focus
2023408	block3	Control	pre	fug1	Object	Contrastive	r1	182.6778917	4	1	post_focus	1	Contrastive post_focus
2023408	block3	Control	pre	tshe1	Object	Contrastive	r1	151.9917935	5	2	post_focus	1	Contrastive post_focus
2023408	block3	Control	pre	piu2	Subject	Contrastive	r1	162.6044862	1	1	pre_focus	2	Contrastive pre_focus
2023408	block3	Control	pre	tse2	Subject	Contrastive	r1	136.7248257	2	2	pre_focus	2	Contrastive pre_focus
2023408	block3	Control	pre	tsap1	Verb	Contrastive	r1	67.2158418	3	1	pre_focus	1	Contrastive pre_focus
2023408	block3	Control	pre	sy1	Object	Contrastive	r1	114.3230626	4	1	on_focus	1	Contrastive on_focus
2023408	block3	Control	pre	pau1	Object	Contrastive	r1	240.1065953	5	2	on_focus	1	Contrastive on_focus
2023408	block3	Control	pre	suk1	Subject	Broad	r1	54.87734856	1	1	broad_focus	1	Broad focus
2023408	block3	Control	pre	suk1	Subject	Broad	r1	44.42617389	2	2	broad_focus	1	Broad focus
2023408	block3	Control	pre	sei2	Verb	Broad	r1	154.7832478	3	1	broad_focus	2	Broad focus
2023408	block3	Control	pre	svy2	Object	Broad	r1	189.7534014	4	1	broad_focus	2	Broad focus
2023408	block3	Control	pre	kwo2	Object	Broad	r1	149.7239973	5	2	broad_focus	2	Broad focus
2023408	block3	Control	pre	piu2	Subject	Narrow	r1	192.888259	1	1	pre_focus	2	Narrow pre_focus
2023408	block3	Control	pre	tse2	Subject	Narrow	r1	184.3744016	2	2	pre_focus	2	Narrow pre_focus
2023408	block3	Control	pre	tsap1	Verb	Narrow	r1	57.89087015	3	1	on_focus	1	Narrow on_focus
2023408	block3	Control	pre	sy1	Object	Narrow	r1	86.07664399	4	1	post_focus	1	Narrow post_focus
2023408	block3	Control	pre	pau1	Object	Narrow	r1	93.46416293	5	2	post_focus	1	Narrow post_focus
2023408	block3	Control	pre	bui3	Subject	Narrow	r1	122.6907814	1	1	pre_focus	3	Narrow pre_focus
2023408	block3	Control	pre	bui3	Subject	Narrow	r1	145.1787895	2	2	pre_focus	3	Narrow pre_focus
2023408	block3	Control	pre	tsv1	Verb	Narrow	r1	130.175611	3	1	on_focus	1	Narrow on_focus
2023408	block3	Control	pre	fug1	Object	Narrow	r1	192.425737	4	1	post_focus	1	Narrow post_focus
2023408	block3	Control	pre	tshe1	Object	Narrow	r1	127.4410707	5	2	post_focus	1	Narrow post_focus
2023408	block3	Control	pre	suk1	Subject	Contrastive	r1	66.00240096	1	1	pre_focus	1	Contrastive pre_focus
2023408	block3	Control	pre	suk1	Subject	Contrastive	r1	62.48157596	2	2	pre_focus	1	Contrastive pre_focus
2023408	block3	Control	pre	sei2	Verb	Contrastive	r1	275.470616	3	1	pre_focus	2	Contrastive pre_focus
2023408	block3	Control	pre	svy2	Object	Contrastive	r1	117.1198492	4	1	on_focus	2	Contrastive on_focus
2023408	block3	Control	pre	kwo2	Object	Contrastive	r1	129.0776981	5	2	on_focus	2	Contrastive on_focus
2023408	block3	Control	pre	suk1	Subject	Narrow	r1	40.03892668	1	1	pre_focus	1	Narrow pre_focus
2023408	block3	Control	pre	suk1	Subject	Narrow	r1	43.43386243	2	2	pre_focus	1	Narrow pre_focus
2023408	block3	Control	pre	sei2	Verb	Narrow	r1	229.1402149	3	1	on_focus	2	Narrow on_focus
2023408	block3	Control	pre	svy2	Object	Narrow	r1	192.3023432	4	1	post_focus	2	Narrow post_focus
2023408	block3	Control	pre	kwo2	Object	Narrow	r1	305.611489	5	2	post_focus	2	Narrow post_focus
2023408	block3	Control	pre	bui3	Subject	Narrow	r1	154.7558348	1	1	on_focus	3	Narrow on_focus
2023408	block3	Control	pre	bui3	Subject	Narrow	r1	156.8773826	2	2	on_focus	3	Narrow on_focus
2023408	block3	Control	pre	tsv1	Verb	Narrow	r1	148.0121286	3	1	post_focus	1	Narrow post_focus
2023408	block3	Control	pre	fug1	Object	Narrow	r1	196.534676	4	1	post_focus	1	Narrow post_focus
2023408	block3	Control	pre	tshe1	Object	Narrow	r1	169.6588174	5	2	post_focus	1	Narrow post_focus
2023408	block3	Control	pre	suk1	Subject	Narrow	r1	75.52869953	1	1	pre_focus	1	Narrow pre_focus
2023408	block3	Control	pre	suk1	Subject	Narrow	r1	62.17418905	2	2	pre_focus	1	Narrow pre_focus
2023408	block3	Control	pre	sei2	Verb	Narrow	r1	140.2548081	3	1	pre_focus	2	Narrow pre_focus
2023408	block3	Control	pre	svy2	Object	Narrow	r1	141.8091236	4	1	on_focus	2	Narrow on_focus
2023408	block3	Control	pre	kwo2	Object	Narrow	r1	67.13025951	5	2	on_focus	2	Narrow on_focus
2023408	block3	Control	pre	suk1	Subject	Contrastive	r1	45.62237339	1	1	pre_focus	1	Contrastive pre_focus
2023408	block3	Control	pre	suk1	Subject	Contrastive	r1	46.14824413	2	2	pre_focus	1	Contrastive pre_focus
2023408	block3	Control	pre	sei2	Verb	Contrastive	r1	160.8376066	3	1	on_focus	2	Contrastive on_focus
2023408	block3	Control	pre	svy2	Object	Contrastive	r1	173.6148547	4	1	post_focus	2	Contrastive post_focus
2023408	block3	Control	pre	kwo2	Object	Contrastive	r1	183.8584903	5	2	post_focus	2	Contrastive post_focus
2023408	block3	Control	pre	piu2	Subject	Narrow	r1	126.9182807	1	1	on_focus	2	Narrow on_focus
2023408	block3	Control	pre	tse2	Subject	Narrow	r1	137.3397401	2	2	on_focus	2	Narrow on_focus
2023408	block3	Control	pre	tsap1	Verb	Narrow	r1	47.2930929	3	1	post_focus	1	Narrow post_focus
2023408	block3	Control	pre	sy1	Object	Narrow	r1	115.8960695	4	1	post_focus	1	Narrow post_focus
2023408	block3	Control	pre	pau1	Object	Narrow	r1	202.4709168	5	2	post_focus	1	Narrow post_focus
2023408	block3	Control	pre	piu2	Subject	Contrastive	r1	82.4829932	1	1	pre_focus	2	Contrastive pre_focus
2023408	block3	Control	pre	tse2	Subject	Contrastive	r1	133.5697279	2	2	pre_focus	2	Contrastive pre_focus
2023408	block3	Control	pre	tsap1	Verb	Contrastive	r1	25.13524457	3	1	on_focus	1	Contrastive on_focus
2023408	block3	Control	pre	sy1	Object	Contrastive	r1	89.2452808	4	1	post_focus	1	Contrastive post_focus
2023408	block3	Control	pre	pau1	Object	Contrastive	r1	222.3769508	5	2	post_focus	1	Contrastive post_focus
2023408	block3	Control	pre	suk1	Subject	Broad	r2	35.53827483	1	1	broad_focus	1	Broad focus
2023408	block3	Control	pre	suk1	Subject	Broad	r2	31.89770923	2	2	broad_focus	1	Broad focus
2023408	block3	Control	pre	sei2	Verb	Broad	r2	161.5673319	3	1	broad_focus	2	Broad focus
2023408	block3	Control	pre	svy2	Object	Broad	r2	145.4776482	4	1	broad_focus	2	Broad focus
2023408	block3	Control	pre	kwo2	Object	Broad	r2	208.0972262	5	2	broad_focus	2	Broad focus
2023408	block3	Control	pre	piu2	Subject	Contrastive	r2	114.6237065	1	1	on_focus	2	Contrastive on_focus

2023408	block3	Control	pre	tse2	Subject	Contrastive	r2	125.8827867	2	2	on_focus	2	Contrastive on_focus
2023408	block3	Control	pre	tsap1	Verb	Contrastive	r2	37.72675737	3	1	post_focus	1	Contrastive post_focus
2023408	block3	Control	pre	sy1	Object	Contrastive	r2	78.92173174	4	1	post_focus	1	Contrastive post_focus
2023408	block3	Control	pre	pau1	Object	Contrastive	r2	121.4259845	5	2	post_focus	1	Contrastive post_focus
2023408	block3	Control	pre	bui3	Subject	Contrastive	r2	82.40264981	1	1	pre_focus	3	Contrastive pre_focus
2023408	block3	Control	pre	bui3	Subject	Contrastive	r2	65.16631522	2	2	pre_focus	3	Contrastive pre_focus
2023408	block3	Control	pre	tsv1	Verb	Contrastive	r2	683.654915	3	1	on_focus	1	Contrastive on_focus
2023408	block3	Control	pre	fug1	Object	Contrastive	r2	229.2156659	4	1	post_focus	1	Contrastive post_focus
2023408	block3	Control	pre	tshe1	Object	Contrastive	r2	258.1310161	5	2	post_focus	1	Contrastive post_focus
2023408	block3	Control	pre	bui3	Subject	Contrastive	r2	83.85596546	1	1	on_focus	3	Contrastive on_focus
2023408	block3	Control	pre	bui3	Subject	Contrastive	r2	123.0682932	2	2	on_focus	3	Contrastive on_focus
2023408	block3	Control	pre	tsv1	Verb	Contrastive	r2	916.2471719	3	1	post_focus	1	Contrastive post_focus
2023408	block3	Control	pre	fug1	Object	Contrastive	r2	106.2285875	4	1	post_focus	1	Contrastive post_focus
2023408	block3	Control	pre	tshe1	Object	Contrastive	r2	148.9267468	5	2	post_focus	1	Contrastive post_focus
2023408	block3	Control	pre	piu2	Subject	Narrow	r2	102.0392842	1	1	pre_focus	2	Narrow pre_focus
2023408	block3	Control	pre	tse2	Subject	Narrow	r2	98.27350798	2	2	pre_focus	2	Narrow pre_focus
2023408	block3	Control	pre	tsap1	Verb	Narrow	r2	19.40082544	3	1	on_focus	1	Narrow on_focus
2023408	block3	Control	pre	sy1	Object	Narrow	r2	126.196328	4	1	post_focus	1	Narrow post_focus
2023408	block3	Control	pre	pau1	Object	Narrow	r2	223.4968627	5	2	post_focus	1	Narrow post_focus
2023408	block3	Control	pre	suk1	Subject	Narrow	r2	37.14902029	1	1	on_focus	1	Narrow on_focus
2023408	block3	Control	pre	suk1	Subject	Narrow	r2	41.5310477	2	2	on_focus	1	Narrow on_focus
2023408	block3	Control	pre	sei2	Verb	Narrow	r2	175.1266062	3	1	post_focus	2	Narrow post_focus
2023408	block3	Control	pre	svy2	Object	Narrow	r2	171.0212327	4	1	post_focus	2	Narrow post_focus
2023408	block3	Control	pre	kwo2	Object	Narrow	r2	108.6800831	5	2	post_focus	2	Narrow post_focus
2023408	block3	Control	pre	piu2	Subject	Narrow	r2	56.0654357	1	1	pre_focus	2	Narrow pre_focus
2023408	block3	Control	pre	tse2	Subject	Narrow	r2	90.6851312	2	2	pre_focus	2	Narrow pre_focus
2023408	block3	Control	pre	tsap1	Verb	Narrow	r2	43.08739983	3	1	pre_focus	1	Narrow pre_focus
2023408	block3	Control	pre	sy1	Object	Narrow	r2	129.6662132	4	1	on_focus	1	Narrow on_focus
2023408	block3	Control	pre	pau1	Object	Narrow	r2	174.4088111	5	2	on_focus	1	Narrow on_focus
2023408	block3	Control	pre	bui3	Subject	Contrastive	r2	65.71642077	1	1	pre_focus	3	Contrastive pre_focus
2023408	block3	Control	pre	bui3	Subject	Contrastive	r2	86.73197279	2	2	pre_focus	3	Contrastive pre_focus
2023408	block3	Control	pre	tsv1	Verb	Contrastive	r2	76.87577151	3	1	pre_focus	1	Contrastive pre_focus
2023408	block3	Control	pre	fug1	Object	Contrastive	r2	124.2498595	4	1	on_focus	1	Contrastive on_focus
2023408	block3	Control	pre	tshe1	Object	Contrastive	r2	159.9458353	5	2	on_focus	1	Contrastive on_focus
2023408	block3	Control	pre	suk1	Subject	Narrow	r2	53.85089171	1	1	pre_focus	1	Narrow pre_focus
2023408	block3	Control	pre	suk1	Subject	Narrow	r2	72.71816808	2	2	pre_focus	1	Narrow pre_focus
2023408	block3	Control	pre	sei2	Verb	Narrow	r2	234.9397113	3	1	on_focus	2	Narrow on_focus
2023408	block3	Control	pre	svy2	Object	Narrow	r2	190.5648819	4	1	post_focus	2	Narrow post_focus
2023408	block3	Control	pre	kwo2	Object	Narrow	r2	202.0421309	5	2	post_focus	2	Narrow post_focus
2023408	block3	Control	pre	suk1	Subject	Contrastive	r2	41.80347695	1	1	pre_focus	1	Contrastive pre_focus
2023408	block3	Control	pre	suk1	Subject	Contrastive	r2	32.72281611	2	2	pre_focus	1	Contrastive pre_focus
2023408	block3	Control	pre	sei2	Verb	Contrastive	r2	345.6683427	3	1	pre_focus	2	Contrastive pre_focus
2023408	block3	Control	pre	svy2	Object	Contrastive	r2	230.5123637	4	1	on_focus	2	Contrastive on_focus
2023408	block3	Control	pre	kwo2	Object	Contrastive	r2	193.0730303	5	2	on_focus	2	Contrastive on_focus
2023408	block3	Control	pre	suk1	Subject	Contrastive	r2	46.4892076	1	1	pre_focus	1	Contrastive pre_focus
2023408	block3	Control	pre	suk1	Subject	Contrastive	r2	50.68455937	2	2	pre_focus	1	Contrastive pre_focus
2023408	block3	Control	pre	sei2	Verb	Contrastive	r2	243.9325935	3	1	on_focus	2	Contrastive on_focus
2023408	block3	Control	pre	svy2	Object	Contrastive	r2	217.0429019	4	1	post_focus	2	Contrastive post_focus
2023408	block3	Control	pre	kwo2	Object	Contrastive	r2	235.1313065	5	2	post_focus	2	Contrastive post_focus
2023408	block3	Control	pre	piu2	Subject	Narrow	r2	79.67168223	1	1	on_focus	2	Narrow on_focus
2023408	block3	Control	pre	tse2	Subject	Narrow	r2	127.0597437	2	2	on_focus	2	Narrow on_focus
2023408	block3	Control	pre	tsap1	Verb	Narrow	r2	55.35289944	3	1	post_focus	1	Narrow post_focus
2023408	block3	Control	pre	sy1	Object	Narrow	r2	106.5093843	4	1	post_focus	1	Narrow post_focus
2023408	block3	Control	pre	pau1	Object	Narrow	r2	219.6314298	5	2	post_focus	1	Narrow post_focus
2023408	block3	Control	pre	suk1	Subject	Narrow	r2	62.96753247	1	1	pre_focus	1	Narrow pre_focus
2023408	block3	Control	pre	suk1	Subject	Narrow	r2	67.83463761	2	2	pre_focus	1	Narrow pre_focus
2023408	block3	Control	pre	sei2	Verb	Narrow	r2	118.1719138	3	1	pre_focus	2	Narrow pre_focus
2023408	block3	Control	pre	svy2	Object	Narrow	r2	161.2997606	4	1	on_focus	2	Narrow on_focus
2023408	block3	Control	pre	kwo2	Object	Narrow	r2	124.7937274	5	2	on_focus	2	Narrow on_focus
2023408	block3	Control	pre	piu2	Subject	Contrastive	r2	118.3857035	1	1	pre_focus	2	Contrastive pre_focus
2023408	block3	Control	pre	tse2	Subject	Contrastive	r2	144.3271785	2	2	pre_focus	2	Contrastive pre_focus
2023408	block3	Control	pre	tsap1	Verb	Contrastive	r2	63.7515747	3	1	pre_focus	1	Contrastive pre_focus
2023408	block3	Control	pre	sy1	Object	Contrastive	r2	95.07785336	4	1	on_focus	1	Contrastive on_focus
2023408	block3	Control	pre	pau1	Object	Contrastive	r2	232.6639942	5	2	on_focus	1	Contrastive on_focus
2023408	block3	Control	pre	bui3	Subject	Broad	r2	111.0391743	1	1	broad_focus	3	Broad focus
2023408	block3	Control	pre	bui3	Subject	Broad	r2	110.7641723	2	2	broad_focus	3	Broad focus

2023408	block3	Control	pre	tsv1	Verb	Broad	r2	90.34595616	3	1	broad_focus	1	Broad focus
2023408	block3	Control	pre	fug1	Object	Broad	r2	148.4702599	4	1	broad_focus	1	Broad focus
2023408	block3	Control	pre	tshe1	Object	Broad	r2	129.050511	5	2	broad_focus	1	Broad focus
2023408	block3	Control	pre	bui3	Subject	Narrow	r2	67.93488824	1	1	pre_focus	3	Narrow pre_focus
2023408	block3	Control	pre	bui3	Subject	Narrow	r2	114.0037793	2	2	pre_focus	3	Narrow pre_focus
2023408	block3	Control	pre	tsv1	Verb	Narrow	r2	105.3461829	3	1	pre_focus	1	Narrow pre_focus
2023408	block3	Control	pre	fug1	Object	Narrow	r2	152.3044625	4	1	on_focus	1	Narrow on_focus
2023408	block3	Control	pre	tshe1	Object	Narrow	r2	139.9717813	5	2	on_focus	1	Narrow on_focus
2023408	block3	Control	pre	bui3	Subject	Narrow	r2	121.7374654	1	1	on_focus	3	Narrow on_focus
2023408	block3	Control	pre	bui3	Subject	Narrow	r2	116.4547745	2	2	on_focus	3	Narrow on_focus
2023408	block3	Control	pre	tsv1	Verb	Narrow	r2	101.2923301	3	1	post_focus	1	Narrow post_focus
2023408	block3	Control	pre	fug1	Object	Narrow	r2	152.4624433	4	1	post_focus	1	Narrow post_focus
2023408	block3	Control	pre	tshe1	Object	Narrow	r2	141.8594602	5	2	post_focus	1	Narrow post_focus
2023408	block3	Control	pre	piu2	Subject	Broad	r2	109.0502531	1	1	broad_focus	2	Broad focus
2023408	block3	Control	pre	tse2	Subject	Broad	r2	137.4205913	2	2	broad_focus	2	Broad focus
2023408	block3	Control	pre	tsap1	Verb	Broad	r2	71.24789701	3	1	broad_focus	1	Broad focus
2023408	block3	Control	pre	sy1	Object	Broad	r2	107.7345522	4	1	broad_focus	1	Broad focus
2023408	block3	Control	pre	pau1	Object	Broad	r2	223.6138167	5	2	broad_focus	1	Broad focus
2023408	block3	Control	pre	suk1	Subject	Contrastive	r2	63.69981643	1	1	on_focus	1	Contrastive on_focus
2023408	block3	Control	pre	suk1	Subject	Contrastive	r2	62.88781324	2	2	on_focus	1	Contrastive on_focus
2023408	block3	Control	pre	sei2	Verb	Contrastive	r2	148.358747	3	1	post_focus	2	Contrastive post_focus
2023408	block3	Control	pre	svy2	Object	Contrastive	r2	148.6232588	4	1	post_focus	2	Contrastive post_focus
2023408	block3	Control	pre	kwo2	Object	Contrastive	r2	158.5013128	5	2	post_focus	2	Contrastive post_focus
2023408	block3	Control	pre	piu2	Subject	Contrastive	r2	73.95677074	1	1	pre_focus	2	Contrastive pre_focus
2023408	block3	Control	pre	tse2	Subject	Contrastive	r2	106.8168934	2	2	pre_focus	2	Contrastive pre_focus
2023408	block3	Control	pre	tsap1	Verb	Contrastive	r2	64.08294087	3	1	on_focus	1	Contrastive on_focus
2023408	block3	Control	pre	sy1	Object	Contrastive	r2	78.20561479	4	1	post_focus	1	Contrastive post_focus
2023408	block3	Control	pre	pau1	Object	Contrastive	r2	167.7777103	5	2	post_focus	1	Contrastive post_focus
2023408	block3	Control	pre	bui3	Subject	Narrow	r2	74.45811097	1	1	pre_focus	3	Narrow pre_focus
2023408	block3	Control	pre	bui3	Subject	Narrow	r2	82.13448872	2	2	pre_focus	3	Narrow pre_focus
2023408	block3	Control	pre	tsv1	Verb	Narrow	r2	81.53723969	3	1	on_focus	1	Narrow on_focus
2023408	block3	Control	pre	fug1	Object	Narrow	r2	117.3256705	4	1	post_focus	1	Narrow post_focus
2023408	block3	Control	pre	tshe1	Object	Narrow	r2	100.3237962	5	2	post_focus	1	Narrow post_focus
2023408	block4	Control	post	piu35	Subject	Narrow	r1	107.9551796	1	1	on_focus	35	Narrow on_focus
2023408	block4	Control	post	mui35	Subject	Narrow	r1	213.1528817	2	2	on_focus	35	Narrow on_focus
2023408	block4	Control	post	tsan3	Verb	Narrow	r1	209.2245686	3	1	post_focus	3	Narrow post_focus
2023408	block4	Control	post	jin3	Object	Narrow	r1	240.1004637	4	1	post_focus	3	Narrow post_focus
2023408	block4	Control	post	jin3	Object	Narrow	r1	221.9450743	5	2	post_focus	3	Narrow post_focus
2023408	block4	Control	post	pak3	Subject	Narrow	r1	75.41602179	1	1	pre_focus	3	Narrow pre_focus
2023408	block4	Control	post	pak3	Subject	Narrow	r1	93.49397113	2	2	pre_focus	3	Narrow pre_focus
2023408	block4	Control	post	tsing2	Verb	Narrow	r1	178.4951814	3	1	pre_focus	2	Narrow pre_focus
2023408	block4	Control	post	kau2	Object	Narrow	r1	214.8591288	4	1	on_focus	2	Narrow on_focus
2023408	block4	Control	post	tsi2	Object	Narrow	r1	239.9942644	5	2	on_focus	2	Narrow on_focus
2023408	block4	Control	post	piu35	Subject	Narrow	r1	126.3821749	1	1	pre_focus	35	Narrow pre_focus
2023408	block4	Control	post	mui35	Subject	Narrow	r1	228.4739	2	2	pre_focus	35	Narrow pre_focus
2023408	block4	Control	post	tsan3	Verb	Narrow	r1	260.1716191	3	1	on_focus	3	Narrow on_focus
2023408	block4	Control	post	jin3	Object	Narrow	r1	251.6603091	4	1	post_focus	3	Narrow post_focus
2023408	block4	Control	post	jin3	Object	Narrow	r1	180.0161311	5	2	post_focus	3	Narrow post_focus
2023408	block4	Control	post	pak3	Subject	Contrastive	r1	104.2583604	1	1	pre_focus	3	Contrastive pre_focus
2023408	block4	Control	post	pak3	Subject	Contrastive	r1	120.7581795	2	2	pre_focus	3	Contrastive pre_focus
2023408	block4	Control	post	tsing2	Verb	Contrastive	r1	179.0414559	3	1	pre_focus	2	Contrastive pre_focus
2023408	block4	Control	post	kau2	Object	Contrastive	r1	161.3916031	4	1	on_focus	2	Contrastive on_focus
2023408	block4	Control	post	tsi2	Object	Contrastive	r1	162.6623641	5	2	on_focus	2	Contrastive on_focus
2023408	block4	Control	post	piu35	Subject	Contrastive	r1	126.2814222	1	1	pre_focus	35	Contrastive pre_focus
2023408	block4	Control	post	mui35	Subject	Contrastive	r1	236.7284571	2	2	pre_focus	35	Contrastive pre_focus
2023408	block4	Control	post	tsan3	Verb	Contrastive	r1	237.2227253	3	1	pre_focus	3	Contrastive pre_focus
2023408	block4	Control	post	jin3	Object	Contrastive	r1	263.2987689	4	1	on_focus	3	Contrastive on_focus
2023408	block4	Control	post	jin3	Object	Contrastive	r1	181.9904258	5	2	on_focus	3	Contrastive on_focus
2023408	block4	Control	post	piu35	Subject	Contrastive	r1	126.1737535	1	1	pre_focus	35	Contrastive pre_focus
2023408	block4	Control	post	mui35	Subject	Contrastive	r1	231.4194975	2	2	pre_focus	35	Contrastive pre_focus
2023408	block4	Control	post	tsan3	Verb	Contrastive	r1	200.5144558	3	1	on_focus	3	Contrastive on_focus
2023408	block4	Control	post	jin3	Object	Contrastive	r1	231.8617991	4	1	post_focus	3	Contrastive post_focus
2023408	block4	Control	post	jin3	Object	Contrastive	r1	156.7626025	5	2	post_focus	3	Contrastive post_focus
2023408	block4	Control	post	Jan-01	Subject	Narrow	r1	187.4872449	1	1	pre_focus	1	Narrow pre_focus
2023408	block4	Control	post	Jan-01	Subject	Narrow	r1	230.5646798	2	2	pre_focus	1	Narrow pre_focus
2023408	block4	Control	post	wei3	Verb	Narrow	r1	144.1166638	3	1	pre_focus	3	Narrow pre_focus

**2023408**	block4	Control	post	tsam3	Object	Narrow	r1	106.6203461	4	1	on_focus	3	Narrow on_focus
**2023408**	block4	Control	post	tsam3	Object	Narrow	r1	190.6964691	5	2	on_focus	3	Narrow on_focus
**2023408**	block4	Control	post	pak3	Subject	Contrastive	r1	86.22538489	1	1	pre_focus	3	Contrastive pre_focus
**2023408**	block4	Control	post	pak3	Subject	Contrastive	r1	94.39475673	2	2	pre_focus	3	Contrastive pre_focus
**2023408**	block4	Control	post	tsing2	Verb	Contrastive	r1	181.5427583	3	1	on_focus	2	Contrastive on_focus
**2023408**	block4	Control	post	kau2	Object	Contrastive	r1	178.7041868	4	1	post_focus	2	Contrastive post_focus
**2023408**	block4	Control	post	tsi2	Object	Contrastive	r1	196.8556311	5	2	post_focus	2	Contrastive post_focus
**2023408**	block4	Control	post	pak3	Subject	Broad	r1	36.29633829	1	1	broad_focus	3	Broad focus
**2023408**	block4	Control	post	pak3	Subject	Broad	r1	82.62338269	2	2	broad_focus	3	Broad focus
**2023408**	block4	Control	post	tsing2	Verb	Broad	r1	162.5720532	3	1	broad_focus	2	Broad focus
**2023408**	block4	Control	post	kau2	Object	Broad	r1	182.8966994	4	1	broad_focus	2	Broad focus
**2023408**	block4	Control	post	tsi2	Object	Broad	r1	210.771148	5	2	broad_focus	2	Broad focus
**2023408**	block4	Control	post	piu35	Subject	Narrow	r1	117.3646601	1	1	pre_focus	35	Narrow pre_focus
**2023408**	block4	Control	post	mui35	Subject	Narrow	r1	163.5708691	2	2	pre_focus	35	Narrow pre_focus
**2023408**	block4	Control	post	tsan3	Verb	Narrow	r1	266.9993505	3	1	pre_focus	3	Narrow pre_focus
**2023408**	block4	Control	post	jin3	Object	Narrow	r1	234.9815574	4	1	on_focus	3	Narrow on_focus
**2023408**	block4	Control	post	jin3	Object	Narrow	r1	217.5724446	5	2	on_focus	3	Narrow on_focus
**2023408**	block4	Control	post	pak3	Subject	Contrastive	r1	117.6679624	1	1	on_focus	3	Contrastive on_focus
**2023408**	block4	Control	post	pak3	Subject	Contrastive	r1	101.6462585	2	2	on_focus	3	Contrastive on_focus
**2023408**	block4	Control	post	tsing2	Verb	Contrastive	r1	94.61980348	3	1	post_focus	2	Contrastive post_focus
**2023408**	block4	Control	post	kau2	Object	Contrastive	r1	157.901607	4	1	post_focus	2	Contrastive post_focus
**2023408**	block4	Control	post	tsi2	Object	Contrastive	r1	145.0378586	5	2	post_focus	2	Contrastive post_focus
**2023408**	block4	Control	post	Jan-01	Subject	Narrow	r1	189.9658594	1	1	pre_focus	1	Narrow pre_focus
**2023408**	block4	Control	post	Jan-01	Subject	Narrow	r1	259.6399999	2	2	pre_focus	1	Narrow pre_focus
**2023408**	block4	Control	post	wei3	Verb	Narrow	r1	186.3556311	3	1	on_focus	3	Narrow on_focus
**2023408**	block4	Control	post	tsam3	Object	Narrow	r1	190.9839814	4	1	post_focus	3	Narrow post_focus
**2023408**	block4	Control	post	tsam3	Object	Narrow	r1	182.9058687	5	2	post_focus	3	Narrow post_focus
**2023408**	block4	Control	post	Jan-01	Subject	Broad	r1	176.4674981	1	1	broad_focus	1	Broad focus
**2023408**	block4	Control	post	Jan-01	Subject	Broad	r1	208.8245397	2	2	broad_focus	1	Broad focus
**2023408**	block4	Control	post	wei3	Verb	Broad	r1	125.7244838	3	1	broad_focus	3	Broad focus
**2023408**	block4	Control	post	tsam3	Object	Broad	r1	197.2459478	4	1	broad_focus	3	Broad focus
**2023408**	block4	Control	post	tsam3	Object	Broad	r1	161.8858655	5	2	broad_focus	3	Broad focus
**2023408**	block4	Control	post	pak3	Subject	Narrow	r1	77.87051476	1	1	pre_focus	3	Narrow pre_focus
**2023408**	block4	Control	post	pak3	Subject	Narrow	r1	107.0058378	2	2	pre_focus	3	Narrow pre_focus
**2023408**	block4	Control	post	tsing2	Verb	Narrow	r1	200.59754	3	1	on_focus	2	Narrow on_focus
**2023408**	block4	Control	post	kau2	Object	Narrow	r1	237.2229801	4	1	post_focus	2	Narrow post_focus
**2023408**	block4	Control	post	tsi2	Object	Narrow	r1	206.3816003	5	2	post_focus	2	Narrow post_focus
**2023408**	block4	Control	post	Jan-01	Subject	Contrastive	r1	187.6740191	1	1	pre_focus	1	Contrastive pre_focus
**2023408**	block4	Control	post	Jan-01	Subject	Contrastive	r1	240.3365841	2	2	pre_focus	1	Contrastive pre_focus
**2023408**	block4	Control	post	wei3	Verb	Contrastive	r1	140.9942572	3	1	on_focus	3	Contrastive on_focus
**2023408**	block4	Control	post	tsam3	Object	Contrastive	r1	208.1950822	4	1	post_focus	3	Contrastive post_focus
**2023408**	block4	Control	post	tsam3	Object	Contrastive	r1	192.6553408	5	2	post_focus	3	Contrastive post_focus
**2023408**	block4	Control	post	pak3	Subject	Narrow	r1	94.04824625	1	1	on_focus	3	Narrow on_focus
**2023408**	block4	Control	post	pak3	Subject	Narrow	r1	87.88151276	2	2	on_focus	3	Narrow on_focus
**2023408**	block4	Control	post	tsing2	Verb	Narrow	r1	173.8004535	3	1	post_focus	2	Narrow post_focus
**2023408**	block4	Control	post	kau2	Object	Narrow	r1	183.7000324	4	1	post_focus	2	Narrow post_focus
**2023408**	block4	Control	post	tsi2	Object	Narrow	r1	195.010101	5	2	post_focus	2	Narrow post_focus
**2023408**	block4	Control	post	piu35	Subject	Contrastive	r1	184.6412186	1	1	on_focus	35	Contrastive on_focus
**2023408**	block4	Control	post	mui35	Subject	Contrastive	r1	188.3034426	2	2	on_focus	35	Contrastive on_focus
**2023408**	block4	Control	post	tsan3	Verb	Contrastive	r1	204.772447	3	1	post_focus	3	Contrastive post_focus
**2023408**	block4	Control	post	jin3	Object	Contrastive	r1	234.9792338	4	1	post_focus	3	Contrastive post_focus
**2023408**	block4	Control	post	jin3	Object	Contrastive	r1	181.2682638	5	2	post_focus	3	Contrastive post_focus
**2023408**	block4	Control	post	piu35	Subject	Broad	r1	159.8326574	1	1	broad_focus	35	Broad focus
**2023408**	block4	Control	post	mui35	Subject	Broad	r1	207.332294	2	2	broad_focus	35	Broad focus
**2023408**	block4	Control	post	tsan3	Verb	Broad	r1	217.1432171	3	1	broad_focus	3	Broad focus
**2023408**	block4	Control	post	jin3	Object	Broad	r1	267.0598517	4	1	broad_focus	3	Broad focus
**2023408**	block4	Control	post	jin3	Object	Broad	r1	173.207357	5	2	broad_focus	3	Broad focus
**2023408**	block4	Control	post	Jan-01	Subject	Narrow	r1	169.8831828	1	1	on_focus	1	Narrow on_focus
**2023408**	block4	Control	post	Jan-01	Subject	Narrow	r1	305.7803444	2	2	on_focus	1	Narrow on_focus
**2023408**	block4	Control	post	wei3	Verb	Narrow	r1	144.8000126	3	1	post_focus	3	Narrow post_focus
**2023408**	block4	Control	post	tsam3	Object	Narrow	r1	240.4385567	4	1	post_focus	3	Narrow post_focus
**2023408**	block4	Control	post	tsam3	Object	Narrow	r1	224.9370233	5	2	post_focus	3	Narrow post_focus
**2023408**	block4	Control	post	Jan-01	Subject	Contrastive	r1	210.6280043	1	1	on_focus	1	Contrastive on_focus
**2023408**	block4	Control	post	Jan-01	Subject	Contrastive	r1	197.3223067	2	2	on_focus	1	Contrastive on_focus
**2023408**	block4	Control	post	wei3	Verb	Contrastive	r1	136.1845248	3	1	post_focus	3	Contrastive post_focus
**2023408**	block4	Control	post	tsam3	Object	Contrastive	r1	180.7525132	4	1	post_focus	3	Contrastive post_focus

2023408	block4	Control	post	tsam3	Object	Contrastive	r1	128.2252841	5	2	post_focus	3	Contrastive post_focus
2023408	block4	Control	post	Jan-01	Subject	Contrastive	r1	230.0037066	1	1	pre_focus	1	Contrastive pre_focus
2023408	block4	Control	post	Jan-01	Subject	Contrastive	r1	243.4801059	2	2	pre_focus	1	Contrastive pre_focus
2023408	block4	Control	post	wei3	Verb	Contrastive	r1	100.2750523	3	1	pre_focus	3	Contrastive pre_focus
2023408	block4	Control	post	tsam3	Object	Contrastive	r1	180.6223537	4	1	on_focus	3	Contrastive on_focus
2023408	block4	Control	post	tsam3	Object	Contrastive	r1	135.3055681	5	2	on_focus	3	Contrastive on_focus
2023408	block4	Control	post	pak3	Subject	Broad	r2	100.2344038	1	1	broad_focus	3	Broad focus
2023408	block4	Control	post	pak3	Subject	Broad	r2	100.4359926	2	2	broad_focus	3	Broad focus
2023408	block4	Control	post	tsing2	Verb	Broad	r2	176.4528849	3	1	broad_focus	2	Broad focus
2023408	block4	Control	post	kau2	Object	Broad	r2	177.4519003	4	1	broad_focus	2	Broad focus
2023408	block4	Control	post	tsi2	Object	Broad	r2	99.89274989	5	2	broad_focus	2	Broad focus
2023408	block4	Control	post	piu35	Subject	Narrow	r2	71.36948854	1	1	on_focus	35	Narrow on_focus
2023408	block4	Control	post	mui35	Subject	Narrow	r2	246.9313315	2	2	on_focus	35	Narrow on_focus
2023408	block4	Control	post	tsan3	Verb	Narrow	r2	226.909619	3	1	post_focus	3	Narrow post_focus
2023408	block4	Control	post	jin3	Object	Narrow	r2	245.8660872	4	1	post_focus	3	Narrow post_focus
2023408	block4	Control	post	jin3	Object	Narrow	r2	134.643487	5	2	post_focus	3	Narrow post_focus
2023408	block4	Control	post	Jan-01	Subject	Contrastive	r2	129.534216	1	1	pre_focus	1	Contrastive pre_focus
2023408	block4	Control	post	Jan-01	Subject	Contrastive	r2	151.8665986	2	2	pre_focus	1	Contrastive pre_focus
2023408	block4	Control	post	wei3	Verb	Contrastive	r2	147.3862137	3	1	pre_focus	3	Contrastive pre_focus
2023408	block4	Control	post	tsam3	Object	Contrastive	r2	246.4832349	4	1	on_focus	3	Contrastive on_focus
2023408	block4	Control	post	tsam3	Object	Contrastive	r2	140.9699495	5	2	on_focus	3	Contrastive on_focus
2023408	block4	Control	post	Jan-01	Subject	Broad	r2	159.1206871	1	1	broad_focus	1	Broad focus
2023408	block4	Control	post	Jan-01	Subject	Broad	r2	194.3368155	2	2	broad_focus	1	Broad focus
2023408	block4	Control	post	wei3	Verb	Broad	r2	138.0687471	3	1	broad_focus	3	Broad focus
2023408	block4	Control	post	tsam3	Object	Broad	r2	178.8542084	4	1	broad_focus	3	Broad focus
2023408	block4	Control	post	tsam3	Object	Broad	r2	181.8732678	5	2	broad_focus	3	Broad focus
2023408	block4	Control	post	piu35	Subject	Narrow	r2	104.624707	1	1	pre_focus	35	Narrow pre_focus
2023408	block4	Control	post	mui35	Subject	Narrow	r2	212.5905723	2	2	pre_focus	35	Narrow pre_focus
2023408	block4	Control	post	tsan3	Verb	Narrow	r2	188.9897362	3	1	pre_focus	3	Narrow pre_focus
2023408	block4	Control	post	jin3	Object	Narrow	r2	161.2586738	4	1	on_focus	3	Narrow on_focus
2023408	block4	Control	post	jin3	Object	Narrow	r2	98.08738257	5	2	on_focus	3	Narrow on_focus
2023408	block4	Control	post	pak3	Subject	Narrow	r2	121.9235896	1	1	pre_focus	3	Narrow pre_focus
2023408	block4	Control	post	pak3	Subject	Narrow	r2	107.6513105	2	2	pre_focus	3	Narrow pre_focus
2023408	block4	Control	post	tsing2	Verb	Narrow	r2	172.8042876	3	1	pre_focus	2	Narrow pre_focus
2023408	block4	Control	post	kau2	Object	Narrow	r2	206.8042328	4	1	on_focus	2	Narrow on_focus
2023408	block4	Control	post	tsi2	Object	Narrow	r2	212.3582766	5	2	on_focus	2	Narrow on_focus
2023408	block4	Control	post	Jan-01	Subject	Contrastive	r2	179.8911402	1	1	pre_focus	1	Contrastive pre_focus
2023408	block4	Control	post	Jan-01	Subject	Contrastive	r2	221.7816492	2	2	pre_focus	1	Contrastive pre_focus
2023408	block4	Control	post	wei3	Verb	Contrastive	r2	169.3509818	3	1	on_focus	3	Contrastive on_focus
2023408	block4	Control	post	tsam3	Object	Contrastive	r2	238.0175967	4	1	post_focus	3	Contrastive post_focus
2023408	block4	Control	post	tsam3	Object	Contrastive	r2	202.8919123	5	2	post_focus	3	Contrastive post_focus
2023408	block4	Control	post	pak3	Subject	Narrow	r2	101.1768458	1	1	on_focus	3	Narrow on_focus
2023408	block4	Control	post	pak3	Subject	Narrow	r2	90.33587224	2	2	on_focus	3	Narrow on_focus
2023408	block4	Control	post	tsing2	Verb	Narrow	r2	182.4821357	3	1	post_focus	2	Narrow post_focus
2023408	block4	Control	post	kau2	Object	Narrow	r2	198.9753347	4	1	post_focus	2	Narrow post_focus
2023408	block4	Control	post	tsi2	Object	Narrow	r2	157.1208093	5	2	post_focus	2	Narrow post_focus
2023408	block4	Control	post	Jan-01	Subject	Narrow	r2	195.9370118	1	1	on_focus	1	Narrow on_focus
2023408	block4	Control	post	Jan-01	Subject	Narrow	r2	232.779195	2	2	on_focus	1	Narrow on_focus
2023408	block4	Control	post	wei3	Verb	Narrow	r2	161.1387613	3	1	post_focus	3	Narrow post_focus
2023408	block4	Control	post	tsam3	Object	Narrow	r2	203.856811	4	1	post_focus	3	Narrow post_focus
2023408	block4	Control	post	tsam3	Object	Narrow	r2	178.1646396	5	2	post_focus	3	Narrow post_focus
2023408	block4	Control	post	piu35	Subject	Narrow	r2	207.4680082	1	1	pre_focus	35	Narrow pre_focus
2023408	block4	Control	post	mui35	Subject	Narrow	r2	126.1906118	2	2	pre_focus	35	Narrow pre_focus
2023408	block4	Control	post	tsan3	Verb	Narrow	r2	193.0731513	3	1	on_focus	3	Narrow on_focus
2023408	block4	Control	post	jin3	Object	Narrow	r2	275.6208819	4	1	post_focus	3	Narrow post_focus
2023408	block4	Control	post	jin3	Object	Narrow	r2	152.2790102	5	2	post_focus	3	Narrow post_focus
2023408	block4	Control	post	Jan-01	Subject	Narrow	r2	267.8069302	1	1	pre_focus	1	Narrow pre_focus
2023408	block4	Control	post	Jan-01	Subject	Narrow	r2	206.6519306	2	2	pre_focus	1	Narrow pre_focus
2023408	block4	Control	post	wei3	Verb	Narrow	r2	165.6845601	3	1	on_focus	3	Narrow on_focus
2023408	block4	Control	post	tsam3	Object	Narrow	r2	171.8432135	4	1	post_focus	3	Narrow post_focus
2023408	block4	Control	post	tsam3	Object	Narrow	r2	164.9347443	5	2	post_focus	3	Narrow post_focus
2023408	block4	Control	post	pak3	Subject	Narrow	r2	112.1570295	1	1	pre_focus	3	Narrow pre_focus
2023408	block4	Control	post	pak3	Subject	Narrow	r2	101.0144409	2	2	pre_focus	3	Narrow pre_focus
2023408	block4	Control	post	tsing2	Verb	Narrow	r2	132.2660038	3	1	on_focus	2	Narrow on_focus
2023408	block4	Control	post	kau2	Object	Narrow	r2	244.0485467	4	1	post_focus	2	Narrow post_focus
2023408	block4	Control	post	tsi2	Object	Narrow	r2	252.9211208	5	2	post_focus	2	Narrow post_focus

**2023408**	block4	Control	post	Jan-01	Subject	Contrastive	r2	193.4633409	1	1	on_focus	1	Contrastive on_focus
**2023408**	block4	Control	post	Jan-01	Subject	Contrastive	r2	212.9183285	2	2	on_focus	1	Contrastive on_focus
**2023408**	block4	Control	post	wei3	Verb	Contrastive	r2	168.7364053	3	1	post_focus	3	Contrastive post_focus
**2023408**	block4	Control	post	tsam3	Object	Contrastive	r2	161.4499514	4	1	post_focus	3	Contrastive post_focus
**2023408**	block4	Control	post	tsam3	Object	Contrastive	r2	143.7609965	5	2	post_focus	3	Contrastive post_focus
**2023408**	block4	Control	post	pak3	Subject	Contrastive	r2	94.00037793	1	1	on_focus	3	Contrastive on_focus
**2023408**	block4	Control	post	pak3	Subject	Contrastive	r2	75.92764378	2	2	on_focus	3	Contrastive on_focus
**2023408**	block4	Control	post	tsing2	Verb	Contrastive	r2	167.997114	3	1	post_focus	2	Contrastive post_focus
**2023408**	block4	Control	post	kau2	Object	Contrastive	r2	168.5120267	4	1	post_focus	2	Contrastive post_focus
**2023408**	block4	Control	post	tsi2	Object	Contrastive	r2	208.4334845	5	2	post_focus	2	Contrastive post_focus
**2023408**	block4	Control	post	piu35	Subject	Contrastive	r2	115.8003995	1	1	pre_focus	35	Contrastive pre_focus
**2023408**	block4	Control	post	mui35	Subject	Contrastive	r2	233.1110308	2	2	pre_focus	35	Contrastive pre_focus
**2023408**	block4	Control	post	tsan3	Verb	Contrastive	r2	210.8695802	3	1	on_focus	3	Contrastive on_focus
**2023408**	block4	Control	post	jin3	Object	Contrastive	r2	258.2656453	4	1	post_focus	3	Contrastive post_focus
**2023408**	block4	Control	post	jin3	Object	Contrastive	r2	140.4957741	5	2	post_focus	3	Contrastive post_focus
**2023408**	block4	Control	post	piu35	Subject	Contrastive	r2	119.0254157	1	1	pre_focus	35	Contrastive pre_focus
**2023408**	block4	Control	post	mui35	Subject	Contrastive	r2	219.3999454	2	2	pre_focus	35	Contrastive pre_focus
**2023408**	block4	Control	post	tsan3	Verb	Contrastive	r2	183.3857988	3	1	pre_focus	3	Contrastive pre_focus
**2023408**	block4	Control	post	jin3	Object	Contrastive	r2	214.7624201	4	1	on_focus	3	Contrastive on_focus
**2023408**	block4	Control	post	jin3	Object	Contrastive	r2	144.0381708	5	2	on_focus	3	Contrastive on_focus
**2023408**	block4	Control	post	piu35	Subject	Contrastive	r2	129.3248009	1	1	on_focus	35	Contrastive on_focus
**2023408**	block4	Control	post	mui35	Subject	Contrastive	r2	239.5313125	2	2	on_focus	35	Contrastive on_focus
**2023408**	block4	Control	post	tsan3	Verb	Contrastive	r2	162.6888002	3	1	post_focus	3	Contrastive post_focus
**2023408**	block4	Control	post	jin3	Object	Contrastive	r2	232.5402494	4	1	post_focus	3	Contrastive post_focus
**2023408**	block4	Control	post	jin3	Object	Contrastive	r2	116.6390614	5	2	post_focus	3	Contrastive post_focus
**2023408**	block4	Control	post	Jan-01	Subject	Narrow	r2	164.8305753	1	1	pre_focus	1	Narrow pre_focus
**2023408**	block4	Control	post	Jan-01	Subject	Narrow	r2	235.950351	2	2	pre_focus	1	Narrow pre_focus
**2023408**	block4	Control	post	wei3	Verb	Narrow	r2	123.8764826	3	1	pre_focus	3	Narrow pre_focus
**2023408**	block4	Control	post	tsam3	Object	Narrow	r2	158.4057623	4	1	on_focus	3	Narrow on_focus
**2023408**	block4	Control	post	tsam3	Object	Narrow	r2	199.9530901	5	2	on_focus	3	Narrow on_focus
**2023408**	block4	Control	post	piu35	Subject	Broad	r2	80.67081061	1	1	broad_focus	35	Broad focus
**2023408**	block4	Control	post	mui35	Subject	Broad	r2	202.3699915	2	2	broad_focus	35	Broad focus
**2023408**	block4	Control	post	tsan3	Verb	Broad	r2	212.0819476	3	1	broad_focus	3	Broad focus
**2023408**	block4	Control	post	jin3	Object	Broad	r2	215.7280286	4	1	broad_focus	3	Broad focus
**2023408**	block4	Control	post	jin3	Object	Broad	r2	97.24499655	5	2	broad_focus	3	Broad focus
**2023408**	block4	Control	post	pak3	Subject	Contrastive	r2	82.74479489	1	1	pre_focus	3	Contrastive pre_focus
**2023408**	block4	Control	post	pak3	Subject	Contrastive	r2	70.22968777	2	2	pre_focus	3	Contrastive pre_focus
**2023408**	block4	Control	post	tsing2	Verb	Contrastive	r2	138.0770975	3	1	on_focus	2	Contrastive on_focus
**2023408**	block4	Control	post	kau2	Object	Contrastive	r2	173.5579817	4	1	post_focus	2	Contrastive post_focus
**2023408**	block4	Control	post	tsi2	Object	Contrastive	r2	155.4181057	5	2	post_focus	2	Contrastive post_focus
**2023408**	block4	Control	post	pak3	Subject	Contrastive	r2	80.89514482	1	1	pre_focus	3	Contrastive pre_focus
**2023408**	block4	Control	post	pak3	Subject	Contrastive	r2	49.94309797	2	2	pre_focus	3	Contrastive pre_focus
**2023408**	block4	Control	post	tsing2	Verb	Contrastive	r2	134.5730262	3	1	pre_focus	2	Contrastive pre_focus
**2023408**	block4	Control	post	kau2	Object	Contrastive	r2	178.2294029	4	1	on_focus	2	Contrastive on_focus
**2023408**	block4	Control	post	tsi2	Object	Contrastive	r2	168.1638322	5	2	on_focus	2	Contrastive on_focus
**2023408**	block4	Control	pre	Jan-01	Subject	Narrow	r1	220.237458	1	1	on_focus	1	Narrow on_focus
**2023408**	block4	Control	pre	Jan-01	Subject	Narrow	r1	276.4000956	2	2	on_focus	1	Narrow on_focus
**2023408**	block4	Control	pre	wei3	Verb	Narrow	r1	133.1919781	3	1	post_focus	3	Narrow post_focus
**2023408**	block4	Control	pre	tsam3	Object	Narrow	r1	194.4938452	4	1	post_focus	3	Narrow post_focus
**2023408**	block4	Control	pre	tsam3	Object	Narrow	r1	177.8858809	5	2	post_focus	3	Narrow post_focus
**2023408**	block4	Control	pre	pak3	Subject	Contrastive	r1	135.0677288	1	1	pre_focus	3	Contrastive pre_focus
**2023408**	block4	Control	pre	pak3	Subject	Contrastive	r1	148.545356	2	2	pre_focus	3	Contrastive pre_focus
**2023408**	block4	Control	pre	tsing2	Verb	Contrastive	r1	214.8242007	3	1	pre_focus	2	Contrastive pre_focus
**2023408**	block4	Control	pre	kau2	Object	Contrastive	r1	271.2483859	4	1	on_focus	2	Contrastive on_focus
**2023408**	block4	Control	pre	tsi2	Object	Contrastive	r1	97.6225195	5	2	on_focus	2	Contrastive on_focus
**2023408**	block4	Control	pre	pak3	Subject	Narrow	r1	136.5730831	1	1	pre_focus	3	Narrow pre_focus
**2023408**	block4	Control	pre	pak3	Subject	Narrow	r1	176.680451	2	2	pre_focus	3	Narrow pre_focus
**2023408**	block4	Control	pre	tsing2	Verb	Narrow	r1	311.7924746	3	1	on_focus	2	Narrow on_focus
**2023408**	block4	Control	pre	kau2	Object	Narrow	r1	360.4211928	4	1	post_focus	2	Narrow post_focus
**2023408**	block4	Control	pre	tsi2	Object	Narrow	r1	200.2870271	5	2	post_focus	2	Narrow post_focus
**2023408**	block4	Control	pre	piu35	Subject	Narrow	r1	121.3243884	1	1	pre_focus	35	Narrow pre_focus
**2023408**	block4	Control	pre	mui35	Subject	Narrow	r1	262.3636472	2	2	pre_focus	35	Narrow pre_focus
**2023408**	block4	Control	pre	tsan3	Verb	Narrow	r1	322.5109831	3	1	pre_focus	3	Narrow pre_focus
**2023408**	block4	Control	pre	jin3	Object	Narrow	r1	368.8462369	4	1	on_focus	3	Narrow on_focus
**2023408**	block4	Control	pre	jin3	Object	Narrow	r1	206.9172021	5	2	on_focus	3	Narrow on_focus
**2023408**	block4	Control	pre	pak3	Subject	Broad	r1	125.8673164	1	1	broad_focus	3	Broad focus

2023408	block4	Control	pre	pak3	Subject	Broad	r1	113.8891366	2	2	broad_focus	3	Broad focus
2023408	block4	Control	pre	tsing2	Verb	Broad	r1	205.1539563	3	1	broad_focus	2	Broad focus
2023408	block4	Control	pre	kau2	Object	Broad	r1	220.4976362	4	1	broad_focus	2	Broad focus
2023408	block4	Control	pre	tsi2	Object	Broad	r1	236.7806122	5	2	broad_focus	2	Broad focus
2023408	block4	Control	pre	piu35	Subject	Narrow	r1	176.2088568	1	1	pre_focus	35	Narrow pre_focus
2023408	block4	Control	pre	mui35	Subject	Narrow	r1	196.0847821	2	2	pre_focus	35	Narrow pre_focus
2023408	block4	Control	pre	tsan3	Verb	Narrow	r1	400.9844461	3	1	on_focus	3	Narrow on_focus
2023408	block4	Control	pre	jin3	Object	Narrow	r1	202.8527705	4	1	post_focus	3	Narrow post_focus
2023408	block4	Control	pre	jin3	Object	Narrow	r1	224.3701537	5	2	post_focus	3	Narrow post_focus
2023408	block4	Control	pre	pak3	Subject	Contrastive	r1	129.8686258	1	1	pre_focus	3	Contrastive pre_focus
2023408	block4	Control	pre	pak3	Subject	Contrastive	r1	84.80875063	2	2	pre_focus	3	Contrastive pre_focus
2023408	block4	Control	pre	tsing2	Verb	Contrastive	r1	317.2411727	3	1	on_focus	2	Contrastive on_focus
2023408	block4	Control	pre	kau2	Object	Contrastive	r1	274.6087974	4	1	post_focus	2	Contrastive post_focus
2023408	block4	Control	pre	tsi2	Object	Contrastive	r1	157.3453641	5	2	post_focus	2	Contrastive post_focus
2023408	block4	Control	pre	Jan-01	Subject	Narrow	r1	198.1378129	1	1	pre_focus	1	Narrow pre_focus
2023408	block4	Control	pre	Jan-01	Subject	Narrow	r1	216.6750526	2	2	pre_focus	1	Narrow pre_focus
2023408	block4	Control	pre	wei3	Verb	Narrow	r1	203.9535787	3	1	pre_focus	3	Narrow pre_focus
2023408	block4	Control	pre	tsam3	Object	Narrow	r1	196.1110005	4	1	on_focus	3	Narrow on_focus
2023408	block4	Control	pre	tsam3	Object	Narrow	r1	168.1565166	5	2	on_focus	3	Narrow on_focus
2023408	block4	Control	pre	Jan-01	Subject	Contrastive	r1	260.1367849	1	1	pre_focus	1	Contrastive pre_focus
2023408	block4	Control	pre	Jan-01	Subject	Contrastive	r1	241.908636	2	2	pre_focus	1	Contrastive pre_focus
2023408	block4	Control	pre	wei3	Verb	Contrastive	r1	228.5529101	3	1	on_focus	3	Contrastive on_focus
2023408	block4	Control	pre	tsam3	Object	Contrastive	r1	224.4350862	4	1	post_focus	3	Contrastive post_focus
2023408	block4	Control	pre	tsam3	Object	Contrastive	r1	167.4959984	5	2	post_focus	3	Contrastive post_focus
2023408	block4	Control	pre	pak3	Subject	Narrow	r1	113.6925979	1	1	pre_focus	3	Narrow pre_focus
2023408	block4	Control	pre	pak3	Subject	Narrow	r1	94.35930736	2	2	pre_focus	3	Narrow pre_focus
2023408	block4	Control	pre	tsing2	Verb	Narrow	r1	190.068199	3	1	pre_focus	2	Narrow pre_focus
2023408	block4	Control	pre	kau2	Object	Narrow	r1	235.475944	4	1	on_focus	2	Narrow on_focus
2023408	block4	Control	pre	tsi2	Object	Narrow	r1	179.3326855	5	2	on_focus	2	Narrow on_focus
2023408	block4	Control	pre	piu35	Subject	Narrow	r1	217.9356083	1	1	on_focus	35	Narrow on_focus
2023408	block4	Control	pre	mui35	Subject	Narrow	r1	120.8019214	2	2	on_focus	35	Narrow on_focus
2023408	block4	Control	pre	tsan3	Verb	Narrow	r1	197.4993521	3	1	post_focus	3	Narrow post_focus
2023408	block4	Control	pre	jin3	Object	Narrow	r1	212.9433579	4	1	post_focus	3	Narrow post_focus
2023408	block4	Control	pre	jin3	Object	Narrow	r1	189.6103896	5	2	post_focus	3	Narrow post_focus
2023408	block4	Control	pre	piu35	Subject	Contrastive	r1	121.7687742	1	1	on_focus	35	Contrastive on_focus
2023408	block4	Control	pre	mui35	Subject	Contrastive	r1	178.4163174	2	2	on_focus	35	Contrastive on_focus
2023408	block4	Control	pre	tsan3	Verb	Contrastive	r1	198.271029	3	1	post_focus	3	Contrastive post_focus
2023408	block4	Control	pre	jin3	Object	Contrastive	r1	223.1402992	4	1	post_focus	3	Contrastive post_focus
2023408	block4	Control	pre	jin3	Object	Contrastive	r1	136.5106042	5	2	post_focus	3	Contrastive post_focus
2023408	block4	Control	pre	piu35	Subject	Contrastive	r1	165.2350583	1	1	pre_focus	35	Contrastive pre_focus
2023408	block4	Control	pre	mui35	Subject	Contrastive	r1	158.3319127	2	2	pre_focus	35	Contrastive pre_focus
2023408	block4	Control	pre	tsan3	Verb	Contrastive	r1	179.788242	3	1	pre_focus	3	Contrastive pre_focus
2023408	block4	Control	pre	jin3	Object	Contrastive	r1	193.7442452	4	1	on_focus	3	Contrastive on_focus
2023408	block4	Control	pre	jin3	Object	Contrastive	r1	167.1515786	5	2	on_focus	3	Contrastive on_focus
2023408	block4	Control	pre	piu35	Subject	Contrastive	r1	228.7676367	1	1	pre_focus	35	Contrastive pre_focus
2023408	block4	Control	pre	mui35	Subject	Contrastive	r1	86.78271309	2	2	pre_focus	35	Contrastive pre_focus
2023408	block4	Control	pre	tsan3	Verb	Contrastive	r1	228.1043084	3	1	on_focus	3	Contrastive on_focus
2023408	block4	Control	pre	jin3	Object	Contrastive	r1	209.0088183	4	1	post_focus	3	Contrastive post_focus
2023408	block4	Control	pre	jin3	Object	Contrastive	r1	214.9537037	5	2	post_focus	3	Contrastive post_focus
2023408	block4	Control	pre	piu35	Subject	Broad	r1	118.3485462	1	1	broad_focus	35	Broad focus
2023408	block4	Control	pre	mui35	Subject	Broad	r1	155.6821825	2	2	broad_focus	35	Broad focus
2023408	block4	Control	pre	tsan3	Verb	Broad	r1	173.276644	3	1	broad_focus	3	Broad focus
2023408	block4	Control	pre	jin3	Object	Broad	r1	224.5002436	4	1	broad_focus	3	Broad focus
2023408	block4	Control	pre	jin3	Object	Broad	r1	214.1036335	5	2	broad_focus	3	Broad focus
2023408	block4	Control	pre	Jan-01	Subject	Broad	r1	113.4650628	1	1	broad_focus	1	Broad focus
2023408	block4	Control	pre	Jan-01	Subject	Broad	r1	164.4737789	2	2	broad_focus	1	Broad focus
2023408	block4	Control	pre	wei3	Verb	Broad	r1	96.81999784	3	1	broad_focus	3	Broad focus
2023408	block4	Control	pre	tsam3	Object	Broad	r1	183.0110139	4	1	broad_focus	3	Broad focus
2023408	block4	Control	pre	tsam3	Object	Broad	r1	179.8825721	5	2	broad_focus	3	Broad focus
2023408	block4	Control	pre	pak3	Subject	Narrow	r1	76.34863946	1	1	on_focus	3	Narrow on_focus
2023408	block4	Control	pre	pak3	Subject	Narrow	r1	81.64134543	2	2	on_focus	3	Narrow on_focus
2023408	block4	Control	pre	tsing2	Verb	Narrow	r1	136.9297359	3	1	post_focus	2	Narrow post_focus
2023408	block4	Control	pre	kau2	Object	Narrow	r1	158.5200032	4	1	post_focus	2	Narrow post_focus
2023408	block4	Control	pre	tsi2	Object	Narrow	r1	170.4575241	5	2	post_focus	2	Narrow post_focus
2023408	block4	Control	pre	Jan-01	Subject	Contrastive	r1	170.5271114	1	1	pre_focus	1	Contrastive pre_focus
2023408	block4	Control	pre	Jan-01	Subject	Contrastive	r1	229.6555032	2	2	pre_focus	1	Contrastive pre_focus

**2023408**	block4	Control	pre	wei3	Verb	Contrastive	r1	117.5767196	3	1	pre_focus	3	Contrastive pre_focus
**2023408**	block4	Control	pre	tsam3	Object	Contrastive	r1	145.9758665	4	1	on_focus	3	Contrastive on_focus
**2023408**	block4	Control	pre	tsam3	Object	Contrastive	r1	231.3067747	5	2	on_focus	3	Contrastive on_focus
**2023408**	block4	Control	pre	Jan-01	Subject	Narrow	r1	186.6197155	1	1	pre_focus	1	Narrow pre_focus
**2023408**	block4	Control	pre	Jan-01	Subject	Narrow	r1	208.1913941	2	2	pre_focus	1	Narrow pre_focus
**2023408**	block4	Control	pre	wei3	Verb	Narrow	r1	108.4702696	3	1	on_focus	3	Narrow on_focus
**2023408**	block4	Control	pre	tsam3	Object	Narrow	r1	141.1422902	4	1	post_focus	3	Narrow post_focus
**2023408**	block4	Control	pre	tsam3	Object	Narrow	r1	148.7796044	5	2	post_focus	3	Narrow post_focus
**2023408**	block4	Control	pre	Jan-01	Subject	Contrastive	r1	181.0669573	1	1	on_focus	1	Contrastive on_focus
**2023408**	block4	Control	pre	Jan-01	Subject	Contrastive	r1	222.7869898	2	2	on_focus	1	Contrastive on_focus
**2023408**	block4	Control	pre	wei3	Verb	Contrastive	r1	98.5248647	3	1	post_focus	3	Contrastive post_focus
**2023408**	block4	Control	pre	tsam3	Object	Contrastive	r1	218.7133511	4	1	post_focus	3	Contrastive post_focus
**2023408**	block4	Control	pre	tsam3	Object	Contrastive	r1	241.0081255	5	2	post_focus	3	Contrastive post_focus
**2023408**	block4	Control	pre	pak3	Subject	Contrastive	r1	190.0227028	1	1	on_focus	3	Contrastive on_focus
**2023408**	block4	Control	pre	pak3	Subject	Contrastive	r1	198.7590287	2	2	on_focus	3	Contrastive on_focus
**2023408**	block4	Control	pre	tsing2	Verb	Contrastive	r1	280.146415	3	1	post_focus	2	Contrastive post_focus
**2023408**	block4	Control	pre	kau2	Object	Contrastive	r1	211.7554305	4	1	post_focus	2	Contrastive post_focus
**2023408**	block4	Control	pre	tsi2	Object	Contrastive	r1	136.3626822	5	2	post_focus	2	Contrastive post_focus
**2023408**	block4	Control	pre	pak3	Subject	Contrastive	r2	82.74716553	1	1	pre_focus	3	Contrastive pre_focus
**2023408**	block4	Control	pre	pak3	Subject	Contrastive	r2	86.03579254	2	2	pre_focus	3	Contrastive pre_focus
**2023408**	block4	Control	pre	tsing2	Verb	Contrastive	r2	148.9465231	3	1	pre_focus	2	Contrastive pre_focus
**2023408**	block4	Control	pre	kau2	Object	Contrastive	r2	175.5803909	4	1	on_focus	2	Contrastive on_focus
**2023408**	block4	Control	pre	tsi2	Object	Contrastive	r2	179.9107143	5	2	on_focus	2	Contrastive on_focus
**2023408**	block4	Control	pre	piu35	Subject	Narrow	r2	219.2661389	1	1	pre_focus	35	Narrow pre_focus
**2023408**	block4	Control	pre	mui35	Subject	Narrow	r2	245.5255635	2	2	pre_focus	35	Narrow pre_focus
**2023408**	block4	Control	pre	tsan3	Verb	Narrow	r2	207.2681371	3	1	pre_focus	3	Narrow pre_focus
**2023408**	block4	Control	pre	jin3	Object	Narrow	r2	247.5698089	4	1	on_focus	3	Narrow on_focus
**2023408**	block4	Control	pre	jin3	Object	Narrow	r2	244.0236476	5	2	on_focus	3	Narrow on_focus
**2023408**	block4	Control	pre	piu35	Subject	Broad	r2	177.4812295	1	1	broad_focus	35	Broad focus
**2023408**	block4	Control	pre	mui35	Subject	Broad	r2	143.5035292	2	2	broad_focus	35	Broad focus
**2023408**	block4	Control	pre	tsan3	Verb	Broad	r2	180.3966742	3	1	broad_focus	3	Broad focus
**2023408**	block4	Control	pre	jin3	Object	Broad	r2	224.4004404	4	1	broad_focus	3	Broad focus
**2023408**	block4	Control	pre	jin3	Object	Broad	r2	133.5547997	5	2	broad_focus	3	Broad focus
**2023408**	block4	Control	pre	pak3	Subject	Contrastive	r2	63.79566641	1	1	pre_focus	3	Contrastive pre_focus
**2023408**	block4	Control	pre	pak3	Subject	Contrastive	r2	97.2739632	2	2	pre_focus	3	Contrastive pre_focus
**2023408**	block4	Control	pre	tsing2	Verb	Contrastive	r2	154.992512	3	1	on_focus	2	Contrastive on_focus
**2023408**	block4	Control	pre	kau2	Object	Contrastive	r2	199.9254449	4	1	post_focus	2	Contrastive post_focus
**2023408**	block4	Control	pre	tsi2	Object	Contrastive	r2	172.9158672	5	2	post_focus	2	Contrastive post_focus
**2023408**	block4	Control	pre	pak3	Subject	Narrow	r2	118.6450732	1	1	pre_focus	3	Narrow pre_focus
**2023408**	block4	Control	pre	pak3	Subject	Narrow	r2	122.9047912	2	2	pre_focus	3	Narrow pre_focus
**2023408**	block4	Control	pre	tsing2	Verb	Narrow	r2	156.5810028	3	1	on_focus	2	Narrow on_focus
**2023408**	block4	Control	pre	kau2	Object	Narrow	r2	218.0580266	4	1	post_focus	2	Narrow post_focus
**2023408**	block4	Control	pre	tsi2	Object	Narrow	r2	207.8144591	5	2	post_focus	2	Narrow post_focus
**2023408**	block4	Control	pre	Jan-01	Subject	Narrow	r2	193.3477633	1	1	on_focus	1	Narrow on_focus
**2023408**	block4	Control	pre	Jan-01	Subject	Narrow	r2	180.8216971	2	2	on_focus	1	Narrow on_focus
**2023408**	block4	Control	pre	wei3	Verb	Narrow	r2	104.251104	3	1	post_focus	3	Narrow post_focus
**2023408**	block4	Control	pre	tsam3	Object	Narrow	r2	179.2749433	4	1	post_focus	3	Narrow post_focus
**2023408**	block4	Control	pre	tsam3	Object	Narrow	r2	191.2018141	5	2	post_focus	3	Narrow post_focus
**2023408**	block4	Control	pre	Jan-01	Subject	Contrastive	r2	154.5750788	1	1	pre_focus	1	Contrastive pre_focus
**2023408**	block4	Control	pre	Jan-01	Subject	Contrastive	r2	212.242328	2	2	pre_focus	1	Contrastive pre_focus
**2023408**	block4	Control	pre	wei3	Verb	Contrastive	r2	98.95171958	3	1	pre_focus	3	Contrastive pre_focus
**2023408**	block4	Control	pre	tsam3	Object	Contrastive	r2	124.4145537	4	1	on_focus	3	Contrastive on_focus
**2023408**	block4	Control	pre	tsam3	Object	Contrastive	r2	152.1838479	5	2	on_focus	3	Contrastive on_focus
**2023408**	block4	Control	pre	piu35	Subject	Contrastive	r2	235.6632528	1	1	pre_focus	35	Contrastive pre_focus
**2023408**	block4	Control	pre	mui35	Subject	Contrastive	r2	114.170446	2	2	pre_focus	35	Contrastive pre_focus
**2023408**	block4	Control	pre	tsan3	Verb	Contrastive	r2	225.8074182	3	1	on_focus	3	Contrastive on_focus
**2023408**	block4	Control	pre	jin3	Object	Contrastive	r2	159.5808458	4	1	post_focus	3	Contrastive post_focus
**2023408**	block4	Control	pre	jin3	Object	Contrastive	r2	135.2099296	5	2	post_focus	3	Contrastive post_focus
**2023408**	block4	Control	pre	Jan-01	Subject	Contrastive	r2	216.7549983	1	1	pre_focus	1	Contrastive pre_focus
**2023408**	block4	Control	pre	Jan-01	Subject	Contrastive	r2	203.3898967	2	2	pre_focus	1	Contrastive pre_focus
**2023408**	block4	Control	pre	wei3	Verb	Contrastive	r2	150.1284271	3	1	on_focus	3	Contrastive on_focus
**2023408**	block4	Control	pre	tsam3	Object	Contrastive	r2	175.244121	4	1	post_focus	3	Contrastive post_focus
**2023408**	block4	Control	pre	tsam3	Object	Contrastive	r2	136.33322	5	2	post_focus	3	Contrastive post_focus
**2023408**	block4	Control	pre	Jan-01	Subject	Contrastive	r2	279.8028955	1	1	on_focus	1	Contrastive on_focus
**2023408**	block4	Control	pre	Jan-01	Subject	Contrastive	r2	186.1867129	2	2	on_focus	1	Contrastive on_focus
**2023408**	block4	Control	pre	wei3	Verb	Contrastive	r2	118.4303721	3	1	post_focus	3	Contrastive post_focus

**2023408**	block4	Control	pre	tsam3	Object	Contrastive	r2	118.8203578	4	1	post_focus	3	Contrastive post_focus
**2023408**	block4	Control	pre	tsam3	Object	Contrastive	r2	100.6689342	5	2	post_focus	3	Contrastive post_focus
**2023408**	block4	Control	pre	piu35	Subject	Narrow	r2	98.15045088	1	1	on_focus	35	Narrow on_focus
**2023408**	block4	Control	pre	mui35	Subject	Narrow	r2	189.0245654	2	2	on_focus	35	Narrow on_focus
**2023408**	block4	Control	pre	tsan3	Verb	Narrow	r2	194.5383553	3	1	post_focus	3	Narrow post_focus
**2023408**	block4	Control	pre	jin3	Object	Narrow	r2	221.6106364	4	1	post_focus	3	Narrow post_focus
**2023408**	block4	Control	pre	jin3	Object	Narrow	r2	187.3349138	5	2	post_focus	3	Narrow post_focus
**2023408**	block4	Control	pre	piu35	Subject	Narrow	r2	110.8759872	1	1	pre_focus	35	Narrow pre_focus
**2023408**	block4	Control	pre	mui35	Subject	Narrow	r2	209.6741677	2	2	pre_focus	35	Narrow pre_focus
**2023408**	block4	Control	pre	tsan3	Verb	Narrow	r2	187.3269115	3	1	on_focus	3	Narrow on_focus
**2023408**	block4	Control	pre	jin3	Object	Narrow	r2	131.3879652	4	1	post_focus	3	Narrow post_focus
**2023408**	block4	Control	pre	jin3	Object	Narrow	r2	93.07018105	5	2	post_focus	3	Narrow post_focus
**2023408**	block4	Control	pre	piu35	Subject	Contrastive	r2	139.223302	1	1	pre_focus	35	Contrastive pre_focus
**2023408**	block4	Control	pre	mui35	Subject	Contrastive	r2	157.0685608	2	2	pre_focus	35	Contrastive pre_focus
**2023408**	block4	Control	pre	tsan3	Verb	Contrastive	r2	224.2782602	3	1	pre_focus	3	Contrastive pre_focus
**2023408**	block4	Control	pre	jin3	Object	Contrastive	r2	197.4501356	4	1	on_focus	3	Contrastive on_focus
**2023408**	block4	Control	pre	jin3	Object	Contrastive	r2	238.3394383	5	2	on_focus	3	Contrastive on_focus
**2023408**	block4	Control	pre	pak3	Subject	Broad	r2	97.13907785	1	1	broad_focus	3	Broad focus
**2023408**	block4	Control	pre	pak3	Subject	Broad	r2	116.3956028	2	2	broad_focus	3	Broad focus
**2023408**	block4	Control	pre	tsing2	Verb	Broad	r2	139.2726321	3	1	broad_focus	2	Broad focus
**2023408**	block4	Control	pre	kau2	Object	Broad	r2	176.7984019	4	1	broad_focus	2	Broad focus
**2023408**	block4	Control	pre	tsi2	Object	Broad	r2	296.9807256	5	2	broad_focus	2	Broad focus
**2023408**	block4	Control	pre	pak3	Subject	Narrow	r2	127.7744184	1	1	on_focus	3	Narrow on_focus
**2023408**	block4	Control	pre	pak3	Subject	Narrow	r2	152.7753482	2	2	on_focus	3	Narrow on_focus
**2023408**	block4	Control	pre	tsing2	Verb	Narrow	r2	169.2725229	3	1	post_focus	2	Narrow post_focus
**2023408**	block4	Control	pre	kau2	Object	Narrow	r2	234.8518121	4	1	post_focus	2	Narrow post_focus
**2023408**	block4	Control	pre	tsi2	Object	Narrow	r2	211.106387	5	2	post_focus	2	Narrow post_focus
**2023408**	block4	Control	pre	Jan-01	Subject	Narrow	r2	164.6279444	1	1	pre_focus	1	Narrow pre_focus
**2023408**	block4	Control	pre	Jan-01	Subject	Narrow	r2	207.8253153	2	2	pre_focus	1	Narrow pre_focus
**2023408**	block4	Control	pre	wei3	Verb	Narrow	r2	129.0535345	3	1	pre_focus	3	Narrow pre_focus
**2023408**	block4	Control	pre	tsam3	Object	Narrow	r2	191.031746	4	1	on_focus	3	Narrow on_focus
**2023408**	block4	Control	pre	tsam3	Object	Narrow	r2	213.2038927	5	2	on_focus	3	Narrow on_focus
**2023408**	block4	Control	pre	pak3	Subject	Narrow	r2	99.20686456	1	1	pre_focus	3	Narrow pre_focus
**2023408**	block4	Control	pre	pak3	Subject	Narrow	r2	117.7513228	2	2	pre_focus	3	Narrow pre_focus
**2023408**	block4	Control	pre	tsing2	Verb	Narrow	r2	142.1415974	3	1	pre_focus	2	Narrow pre_focus
**2023408**	block4	Control	pre	kau2	Object	Narrow	r2	189.9421115	4	1	on_focus	2	Narrow on_focus
**2023408**	block4	Control	pre	tsi2	Object	Narrow	r2	200.5243328	5	2	on_focus	2	Narrow on_focus
**2023408**	block4	Control	pre	Jan-01	Subject	Narrow	r2	204.6468769	1	1	pre_focus	1	Narrow pre_focus
**2023408**	block4	Control	pre	Jan-01	Subject	Narrow	r2	239.089727	2	2	pre_focus	1	Narrow pre_focus
**2023408**	block4	Control	pre	wei3	Verb	Narrow	r2	165.9633238	3	1	on_focus	3	Narrow on_focus
**2023408**	block4	Control	pre	tsam3	Object	Narrow	r2	176.6047474	4	1	post_focus	3	Narrow post_focus
**2023408**	block4	Control	pre	tsam3	Object	Narrow	r2	169.4718904	5	2	post_focus	3	Narrow post_focus
**2023408**	block4	Control	pre	Jan-01	Subject	Broad	r2	206.6043084	1	1	broad_focus	1	Broad focus
**2023408**	block4	Control	pre	Jan-01	Subject	Broad	r2	176.4571074	2	2	broad_focus	1	Broad focus
**2023408**	block4	Control	pre	wei3	Verb	Broad	r2	192.3129252	3	1	broad_focus	3	Broad focus
**2023408**	block4	Control	pre	tsam3	Object	Broad	r2	118.175919	4	1	broad_focus	3	Broad focus
**2023408**	block4	Control	pre	tsam3	Object	Broad	r2	181.2789833	5	2	broad_focus	3	Broad focus
**2023408**	block4	Control	pre	piu35	Subject	Contrastive	r2	229.0975057	1	1	on_focus	35	Contrastive on_focus
**2023408**	block4	Control	pre	mui35	Subject	Contrastive	r2	229.2519731	2	2	on_focus	35	Contrastive on_focus
**2023408**	block4	Control	pre	tsan3	Verb	Contrastive	r2	215.1026261	3	1	post_focus	3	Contrastive post_focus
**2023408**	block4	Control	pre	jin3	Object	Contrastive	r2	163.1101268	4	1	post_focus	3	Contrastive post_focus
**2023408**	block4	Control	pre	jin3	Object	Contrastive	r2	108.8225561	5	2	post_focus	3	Contrastive post_focus
**2023408**	block4	Control	pre	pak3	Subject	Contrastive	r2	102.2335601	1	1	on_focus	3	Contrastive on_focus
**2023408**	block4	Control	pre	pak3	Subject	Contrastive	r2	96.46788101	2	2	on_focus	3	Contrastive on_focus
**2023408**	block4	Control	pre	tsing2	Verb	Contrastive	r2	150.7620667	3	1	post_focus	2	Contrastive post_focus
**2023408**	block4	Control	pre	kau2	Object	Contrastive	r2	185.6282743	4	1	post_focus	2	Contrastive post_focus
**2023408**	block4	Control	pre	tsi2	Object	Contrastive	r2	176.5419501	5	2	post_focus	2	Contrastive post_focus
**2023408**	block5	Control	post	wai5	Subject	Narrow	r1	211.7547816	1	1	on_focus	5	Narrow on_focus
**2023408**	block5	Control	post	wai5	Subject	Narrow	r1	152.9802132	2	2	on_focus	5	Narrow on_focus
**2023408**	block5	Control	post	waat3	Verb	Narrow	r1	162.3809524	3	1	post_focus	3	Narrow post_focus
**2023408**	block5	Control	post	bui3	Object	Narrow	r1	80.42958952	4	1	post_focus	3	Narrow post_focus
**2023408**	block5	Control	post	hok3	Object	Narrow	r1	45.11639721	5	2	post_focus	3	Narrow post_focus
**2023408**	block5	Control	post	ceoi3	Subject	Narrow	r1	95.78030227	1	1	pre_focus	3	Narrow pre_focus
**2023408**	block5	Control	post	ceoi3	Subject	Narrow	r1	115.5846561	2	2	pre_focus	3	Narrow pre_focus
**2023408**	block5	Control	post	caa4	Verb	Narrow	r1	123.1577909	3	1	pre_focus	4	Narrow pre_focus
**2023408**	block5	Control	post	ngau4	Object	Narrow	r1	129.2146393	4	1	on_focus	4	Narrow on_focus

2023408	block5	Control	post	jau4	Object	Narrow	r1	123.8271605	5	2	on_focus	4	Narrow on_focus
2023408	block5	Control	post	siu2	Subject	Contrastive	r1	113.6696901	1	1	on_focus	2	Contrastive on_focus
2023408	block5	Control	post	gwong2	Subject	Contrastive	r1	194.3710709	2	2	on_focus	2	Contrastive on_focus
2023408	block5	Control	post	cyun4	Verb	Contrastive	r1	103.2588273	3	1	post_focus	4	Contrastive post_focus
2023408	block5	Control	post	laam4	Object	Contrastive	r1	253.8994059	4	1	post_focus	4	Contrastive post_focus
2023408	block5	Control	post	kau4	Object	Contrastive	r1	104.3108196	5	2	post_focus	4	Contrastive post_focus
2023408	block5	Control	post	wai5	Subject	Contrastive	r1	253.9689209	1	1	on_focus	5	Contrastive on_focus
2023408	block5	Control	post	wai5	Subject	Contrastive	r1	177.5409423	2	2	on_focus	5	Contrastive on_focus
2023408	block5	Control	post	waat3	Verb	Contrastive	r1	115.4726474	3	1	post_focus	3	Contrastive post_focus
2023408	block5	Control	post	bui3	Object	Contrastive	r1	147.4002088	4	1	post_focus	3	Contrastive post_focus
2023408	block5	Control	post	hok3	Object	Contrastive	r1	36.62419465	5	2	post_focus	3	Contrastive post_focus
2023408	block5	Control	post	siu2	Subject	Contrastive	r1	148.1033781	1	1	pre_focus	2	Contrastive pre_focus
2023408	block5	Control	post	gwong2	Subject	Contrastive	r1	210.3819242	2	2	pre_focus	2	Contrastive pre_focus
2023408	block5	Control	post	cyun4	Verb	Contrastive	r1	111.355592	3	1	on_focus	4	Contrastive on_focus
2023408	block5	Control	post	laam4	Object	Contrastive	r1	270.22323	4	1	post_focus	4	Contrastive post_focus
2023408	block5	Control	post	kau4	Object	Contrastive	r1	148.6824452	5	2	post_focus	4	Contrastive post_focus
2023408	block5	Control	post	siu2	Subject	Narrow	r1	147.6850707	1	1	on_focus	2	Narrow on_focus
2023408	block5	Control	post	gwong2	Subject	Narrow	r1	187.7553985	2	2	on_focus	2	Narrow on_focus
2023408	block5	Control	post	cyun4	Verb	Narrow	r1	111.9863946	3	1	post_focus	4	Narrow post_focus
2023408	block5	Control	post	laam4	Object	Narrow	r1	264.4402843	4	1	post_focus	4	Narrow post_focus
2023408	block5	Control	post	kau4	Object	Narrow	r1	128.9508692	5	2	post_focus	4	Narrow post_focus
2023408	block5	Control	post	ceoi3	Subject	Narrow	r1	103.9239452	1	1	pre_focus	3	Narrow pre_focus
2023408	block5	Control	post	ceoi3	Subject	Narrow	r1	101.7386955	2	2	pre_focus	3	Narrow pre_focus
2023408	block5	Control	post	caa4	Verb	Narrow	r1	102.507765	3	1	on_focus	4	Narrow on_focus
2023408	block5	Control	post	ngau4	Object	Narrow	r1	164.8728965	4	1	post_focus	4	Narrow post_focus
2023408	block5	Control	post	jau4	Object	Narrow	r1	260.6878307	5	2	post_focus	4	Narrow post_focus
2023408	block5	Control	post	siu2	Subject	Narrow	r1	139.6102914	1	1	pre_focus	2	Narrow pre_focus
2023408	block5	Control	post	gwong2	Subject	Narrow	r1	164.0528658	2	2	pre_focus	2	Narrow pre_focus
2023408	block5	Control	post	cyun4	Verb	Narrow	r1	116.574614	3	1	pre_focus	4	Narrow pre_focus
2023408	block5	Control	post	laam4	Object	Narrow	r1	243.0924404	4	1	on_focus	4	Narrow on_focus
2023408	block5	Control	post	kau4	Object	Narrow	r1	55.0470855	5	2	on_focus	4	Narrow on_focus
2023408	block5	Control	post	wai5	Subject	Narrow	r1	190.8142651	1	1	pre_focus	5	Narrow pre_focus
2023408	block5	Control	post	wai5	Subject	Narrow	r1	218.0094894	2	2	pre_focus	5	Narrow pre_focus
2023408	block5	Control	post	waat3	Verb	Narrow	r1	92.46065873	3	1	pre_focus	3	Narrow pre_focus
2023408	block5	Control	post	bui3	Object	Narrow	r1	163.0095005	4	1	on_focus	3	Narrow on_focus
2023408	block5	Control	post	hok3	Object	Narrow	r1	34.18389183	5	2	on_focus	3	Narrow on_focus
2023408	block5	Control	post	wai5	Subject	Broad	r1	201.7316686	1	1	broad_focus	5	Broad focus
2023408	block5	Control	post	wai5	Subject	Broad	r1	141.9601393	2	2	broad_focus	5	Broad focus
2023408	block5	Control	post	waat3	Verb	Broad	r1	123.0520912	3	1	broad_focus	3	Broad focus
2023408	block5	Control	post	bui3	Object	Broad	r1	115.8442351	4	1	broad_focus	3	Broad focus
2023408	block5	Control	post	hok3	Object	Broad	r1	48.59743008	5	2	broad_focus	3	Broad focus
2023408	block5	Control	post	ceoi3	Subject	Broad	r1	94.55395042	1	1	broad_focus	3	Broad focus
2023408	block5	Control	post	ceoi3	Subject	Broad	r1	97.92163996	2	2	broad_focus	3	Broad focus
2023408	block5	Control	post	caa4	Verb	Broad	r1	168.3719793	3	1	broad_focus	4	Broad focus
2023408	block5	Control	post	ngau4	Object	Broad	r1	155.3198872	4	1	broad_focus	4	Broad focus
2023408	block5	Control	post	jau4	Object	Broad	r1	160.6632653	5	2	broad_focus	4	Broad focus
2023408	block5	Control	post	ceoi3	Subject	Narrow	r1	85.16326531	1	1	on_focus	3	Narrow on_focus
2023408	block5	Control	post	ceoi3	Subject	Narrow	r1	97.42559524	2	2	on_focus	3	Narrow on_focus
2023408	block5	Control	post	caa4	Verb	Narrow	r1	35.13634597	3	1	post_focus	4	Narrow post_focus
2023408	block5	Control	post	ngau4	Object	Narrow	r1	149.6906797	4	1	post_focus	4	Narrow post_focus
2023408	block5	Control	post	jau4	Object	Narrow	r1	189.5328388	5	2	post_focus	4	Narrow post_focus
2023408	block5	Control	post	siu2	Subject	Contrastive	r1	156.5693037	1	1	pre_focus	2	Contrastive pre_focus
2023408	block5	Control	post	gwong2	Subject	Contrastive	r1	164.7407252	2	2	pre_focus	2	Contrastive pre_focus
2023408	block5	Control	post	cyun4	Verb	Contrastive	r1	90.36411728	3	1	pre_focus	4	Contrastive pre_focus
2023408	block5	Control	post	laam4	Object	Contrastive	r1	252.1756773	4	1	on_focus	4	Contrastive on_focus
2023408	block5	Control	post	kau4	Object	Contrastive	r1	69.32275132	5	2	on_focus	4	Contrastive on_focus
2023408	block5	Control	post	ceoi3	Subject	Contrastive	r1	104.438279	1	1	pre_focus	3	Contrastive pre_focus
2023408	block5	Control	post	ceoi3	Subject	Contrastive	r1	112.1581543	2	2	pre_focus	3	Contrastive pre_focus
2023408	block5	Control	post	caa4	Verb	Contrastive	r1	78.67654122	3	1	pre_focus	4	Contrastive pre_focus
2023408	block5	Control	post	ngau4	Object	Contrastive	r1	172.5497148	4	1	on_focus	4	Contrastive on_focus
2023408	block5	Control	post	jau4	Object	Contrastive	r1	161.9839245	5	2	on_focus	4	Contrastive on_focus
2023408	block5	Control	post	siu2	Subject	Broad	r1	127.9510852	1	1	broad_focus	2	Broad focus
2023408	block5	Control	post	gwong2	Subject	Broad	r1	212.1273964	2	2	broad_focus	2	Broad focus
2023408	block5	Control	post	cyun4	Verb	Broad	r1	163.1412644	3	1	broad_focus	4	Broad focus
2023408	block5	Control	post	laam4	Object	Broad	r1	210.6226764	4	1	broad_focus	4	Broad focus
2023408	block5	Control	post	kau4	Object	Broad	r1	116.2141642	5	2	broad_focus	4	Broad focus

**2023408**	block5	Control	post	ceoi3	Subject	Contrastive	r1	76.6553288	1	1	on_focus	3	Contrastive on_focus
**2023408**	block5	Control	post	ceoi3	Subject	Contrastive	r1	100.759904	2	2	on_focus	3	Contrastive on_focus
**2023408**	block5	Control	post	caa4	Verb	Contrastive	r1	83.23904006	3	1	post_focus	4	Contrastive post_focus
**2023408**	block5	Control	post	ngau4	Object	Contrastive	r1	129.8443068	4	1	post_focus	4	Contrastive post_focus
**2023408**	block5	Control	post	jau4	Object	Contrastive	r1	201.8884092	5	2	post_focus	4	Contrastive post_focus
**2023408**	block5	Control	post	ceoi3	Subject	Contrastive	r1	80.33677792	1	1	pre_focus	3	Contrastive pre_focus
**2023408**	block5	Control	post	ceoi3	Subject	Contrastive	r1	118.382553	2	2	pre_focus	3	Contrastive pre_focus
**2023408**	block5	Control	post	caa4	Verb	Contrastive	r1	94.57844725	3	1	on_focus	4	Contrastive on_focus
**2023408**	block5	Control	post	ngau4	Object	Contrastive	r1	164.0143215	4	1	post_focus	4	Contrastive post_focus
**2023408**	block5	Control	post	jau4	Object	Contrastive	r1	129.2410714	5	2	post_focus	4	Contrastive post_focus
**2023408**	block5	Control	post	wai5	Subject	Contrastive	r1	192.3971493	1	1	pre_focus	5	Contrastive pre_focus
**2023408**	block5	Control	post	wai5	Subject	Contrastive	r1	197.2286868	2	2	pre_focus	5	Contrastive pre_focus
**2023408**	block5	Control	post	waat3	Verb	Contrastive	r1	99.41009428	3	1	pre_focus	3	Contrastive pre_focus
**2023408**	block5	Control	post	bui3	Object	Contrastive	r1	126.9926251	4	1	on_focus	3	Contrastive on_focus
**2023408**	block5	Control	post	hok3	Object	Contrastive	r1	49.45362272	5	2	on_focus	3	Contrastive on_focus
**2023408**	block5	Control	post	wai5	Subject	Contrastive	r1	188.4763165	1	1	pre_focus	5	Contrastive pre_focus
**2023408**	block5	Control	post	wai5	Subject	Contrastive	r1	191.5286686	2	2	pre_focus	5	Contrastive pre_focus
**2023408**	block5	Control	post	waat3	Verb	Contrastive	r1	149.8383353	3	1	on_focus	3	Contrastive on_focus
**2023408**	block5	Control	post	bui3	Object	Contrastive	r1	118.0973218	4	1	post_focus	3	Contrastive post_focus
**2023408**	block5	Control	post	hok3	Object	Contrastive	r1	48.30099941	5	2	post_focus	3	Contrastive post_focus
**2023408**	block5	Control	post	siu2	Subject	Narrow	r1	172.4303739	1	1	pre_focus	2	Narrow pre_focus
**2023408**	block5	Control	post	gwong2	Subject	Narrow	r1	215.2928949	2	2	pre_focus	2	Narrow pre_focus
**2023408**	block5	Control	post	cyun4	Verb	Narrow	r1	166.1708282	3	1	on_focus	4	Narrow on_focus
**2023408**	block5	Control	post	laam4	Object	Narrow	r1	204.1703006	4	1	post_focus	4	Narrow post_focus
**2023408**	block5	Control	post	kau4	Object	Narrow	r1	76.98039421	5	2	post_focus	4	Narrow post_focus
**2023408**	block5	Control	post	wai5	Subject	Narrow	r1	119.7900236	1	1	pre_focus	5	Narrow pre_focus
**2023408**	block5	Control	post	wai5	Subject	Narrow	r1	194.1891804	2	2	pre_focus	5	Narrow pre_focus
**2023408**	block5	Control	post	waat3	Verb	Narrow	r1	158.6990311	3	1	on_focus	3	Narrow on_focus
**2023408**	block5	Control	post	bui3	Object	Narrow	r1	99.71941639	4	1	post_focus	3	Narrow post_focus
**2023408**	block5	Control	post	hok3	Object	Narrow	r1	36.72637944	5	2	post_focus	3	Narrow post_focus
**2023408**	block5	Control	post	wai5	Subject	Narrow	r2	134.97666	1	1	on_focus	5	Narrow on_focus
**2023408**	block5	Control	post	wai5	Subject	Narrow	r2	148.8475057	2	2	on_focus	5	Narrow on_focus
**2023408**	block5	Control	post	waat3	Verb	Narrow	r2	107.8576288	3	1	post_focus	3	Narrow post_focus
**2023408**	block5	Control	post	bui3	Object	Narrow	r2	107.223482	4	1	post_focus	3	Narrow post_focus
**2023408**	block5	Control	post	hok3	Object	Narrow	r2	23.85231194	5	2	post_focus	3	Narrow post_focus
**2023408**	block5	Control	post	siu2	Subject	Contrastive	r2	189.0239946	1	1	pre_focus	2	Contrastive pre_focus
**2023408**	block5	Control	post	gwong2	Subject	Contrastive	r2	204.0763416	2	2	pre_focus	2	Contrastive pre_focus
**2023408**	block5	Control	post	cyun4	Verb	Contrastive	r2	186.4866105	3	1	pre_focus	4	Contrastive pre_focus
**2023408**	block5	Control	post	laam4	Object	Contrastive	r2	245.3381643	4	1	on_focus	4	Contrastive on_focus
**2023408**	block5	Control	post	kau4	Object	Contrastive	r2	84.15973797	5	2	on_focus	4	Contrastive on_focus
**2023408**	block5	Control	post	siu2	Subject	Contrastive	r2	148.2982097	1	1	pre_focus	2	Contrastive pre_focus
**2023408**	block5	Control	post	gwong2	Subject	Contrastive	r2	185.2949551	2	2	pre_focus	2	Contrastive pre_focus
**2023408**	block5	Control	post	cyun4	Verb	Contrastive	r2	122.1161322	3	1	on_focus	4	Contrastive on_focus
**2023408**	block5	Control	post	laam4	Object	Contrastive	r2	233.641235	4	1	post_focus	4	Contrastive post_focus
**2023408**	block5	Control	post	kau4	Object	Contrastive	r2	98.61711895	5	2	post_focus	4	Contrastive post_focus
**2023408**	block5	Control	post	siu2	Subject	Narrow	r2	248.4450113	1	1	pre_focus	2	Narrow pre_focus
**2023408**	block5	Control	post	gwong2	Subject	Narrow	r2	249.1329506	2	2	pre_focus	2	Narrow pre_focus
**2023408**	block5	Control	post	cyun4	Verb	Narrow	r2	148.9796504	3	1	pre_focus	4	Narrow pre_focus
**2023408**	block5	Control	post	laam4	Object	Narrow	r2	242.0854592	4	1	on_focus	4	Narrow on_focus
**2023408**	block5	Control	post	kau4	Object	Narrow	r2	65.83576288	5	2	on_focus	4	Narrow on_focus
**2023408**	block5	Control	post	siu2	Subject	Contrastive	r2	137.8201448	1	1	on_focus	2	Contrastive on_focus
**2023408**	block5	Control	post	gwong2	Subject	Contrastive	r2	200.5462018	2	2	on_focus	2	Contrastive on_focus
**2023408**	block5	Control	post	cyun4	Verb	Contrastive	r2	118.9533468	3	1	post_focus	4	Contrastive post_focus
**2023408**	block5	Control	post	laam4	Object	Contrastive	r2	238.2295585	4	1	post_focus	4	Contrastive post_focus
**2023408**	block5	Control	post	kau4	Object	Contrastive	r2	86.29183824	5	2	post_focus	4	Contrastive post_focus
**2023408**	block5	Control	post	wai5	Subject	Narrow	r2	162.3482892	1	1	pre_focus	5	Narrow pre_focus
**2023408**	block5	Control	post	wai5	Subject	Narrow	r2	197.0074156	2	2	pre_focus	5	Narrow pre_focus
**2023408**	block5	Control	post	waat3	Verb	Narrow	r2	106.1076164	3	1	pre_focus	3	Narrow pre_focus
**2023408**	block5	Control	post	bui3	Object	Narrow	r2	119.1614493	4	1	on_focus	3	Narrow on_focus
**2023408**	block5	Control	post	hok3	Object	Narrow	r2	135.9365079	5	2	on_focus	3	Narrow on_focus
**2023408**	block5	Control	post	ceoi3	Subject	Contrastive	r2	83.73673094	1	1	pre_focus	3	Contrastive pre_focus
**2023408**	block5	Control	post	ceoi3	Subject	Contrastive	r2	119.2191775	2	2	pre_focus	3	Contrastive pre_focus
**2023408**	block5	Control	post	caa4	Verb	Contrastive	r2	99.23380059	3	1	pre_focus	4	Contrastive pre_focus
**2023408**	block5	Control	post	ngau4	Object	Contrastive	r2	115.1853346	4	1	on_focus	4	Contrastive on_focus
**2023408**	block5	Control	post	jau4	Object	Contrastive	r2	201.3385139	5	2	on_focus	4	Contrastive on_focus
**2023408**	block5	Control	post	siu2	Subject	Broad	r2	124.742493	1	1	broad_focus	2	Broad focus

**2023408**	block5	Control	post	gwong2	Subject	Broad	r2	217.734478	2	2	broad_focus	2	Broad focus
**2023408**	block5	Control	post	cyun4	Verb	Broad	r2	120.2067705	3	1	broad_focus	4	Broad focus
**2023408**	block5	Control	post	laam4	Object	Broad	r2	164.2109241	4	1	broad_focus	4	Broad focus
**2023408**	block5	Control	post	kau4	Object	Broad	r2	62.81767028	5	2	broad_focus	4	Broad focus
**2023408**	block5	Control	post	ceoi3	Subject	Narrow	r2	92.33617726	1	1	pre_focus	3	Narrow pre_focus
**2023408**	block5	Control	post	ceoi3	Subject	Narrow	r2	129.3695437	2	2	pre_focus	3	Narrow pre_focus
**2023408**	block5	Control	post	caa4	Verb	Narrow	r2	68.20889847	3	1	on_focus	4	Narrow on_focus
**2023408**	block5	Control	post	ngau4	Object	Narrow	r2	184.5144472	4	1	post_focus	4	Narrow post_focus
**2023408**	block5	Control	post	jau4	Object	Narrow	r2	212.9433996	5	2	post_focus	4	Narrow post_focus
**2023408**	block5	Control	post	ceoi3	Subject	Broad	r2	109.0967116	1	1	broad_focus	3	Broad focus
**2023408**	block5	Control	post	ceoi3	Subject	Broad	r2	126.8779746	2	2	broad_focus	3	Broad focus
**2023408**	block5	Control	post	caa4	Verb	Broad	r2	48.84185325	3	1	broad_focus	4	Broad focus
**2023408**	block5	Control	post	ngau4	Object	Broad	r2	196.9887015	4	1	broad_focus	4	Broad focus
**2023408**	block5	Control	post	jau4	Object	Broad	r2	231.3316755	5	2	broad_focus	4	Broad focus
**2023408**	block5	Control	post	siu2	Subject	Narrow	r2	138.0560456	1	1	on_focus	2	Narrow on_focus
**2023408**	block5	Control	post	gwong2	Subject	Narrow	r2	192.80428	2	2	on_focus	2	Narrow on_focus
**2023408**	block5	Control	post	cyun4	Verb	Narrow	r2	136.8645308	3	1	post_focus	4	Narrow post_focus
**2023408**	block5	Control	post	laam4	Object	Narrow	r2	184.8975813	4	1	post_focus	4	Narrow post_focus
**2023408**	block5	Control	post	kau4	Object	Narrow	r2	27.6878183	5	2	post_focus	4	Narrow post_focus
**2023408**	block5	Control	post	ceoi3	Subject	Contrastive	r2	114.6890756	1	1	pre_focus	3	Contrastive pre_focus
**2023408**	block5	Control	post	ceoi3	Subject	Contrastive	r2	173.8728825	2	2	pre_focus	3	Contrastive pre_focus
**2023408**	block5	Control	post	caa4	Verb	Contrastive	r2	121.6188824	3	1	on_focus	4	Contrastive on_focus
**2023408**	block5	Control	post	ngau4	Object	Contrastive	r2	145.3929411	4	1	post_focus	4	Contrastive post_focus
**2023408**	block5	Control	post	jau4	Object	Contrastive	r2	169.7279616	5	2	post_focus	4	Contrastive post_focus
**2023408**	block5	Control	post	wai5	Subject	Contrastive	r2	172.4356981	1	1	on_focus	5	Contrastive on_focus
**2023408**	block5	Control	post	wai5	Subject	Contrastive	r2	193.9673992	2	2	on_focus	5	Contrastive on_focus
**2023408**	block5	Control	post	waat3	Verb	Contrastive	r2	130.5747894	3	1	post_focus	3	Contrastive post_focus
**2023408**	block5	Control	post	bui3	Object	Contrastive	r2	82.92670831	4	1	post_focus	3	Contrastive post_focus
**2023408**	block5	Control	post	hok3	Object	Contrastive	r2	58.17571597	5	2	post_focus	3	Contrastive post_focus
**2023408**	block5	Control	post	wai5	Subject	Broad	r2	117.0030782	1	1	broad_focus	5	Broad focus
**2023408**	block5	Control	post	wai5	Subject	Broad	r2	203.3466106	2	2	broad_focus	5	Broad focus
**2023408**	block5	Control	post	waat3	Verb	Broad	r2	114.160685	3	1	broad_focus	3	Broad focus
**2023408**	block5	Control	post	bui3	Object	Broad	r2	91.76275894	4	1	broad_focus	3	Broad focus
**2023408**	block5	Control	post	hok3	Object	Broad	r2	76.32432118	5	2	broad_focus	3	Broad focus
**2023408**	block5	Control	post	wai5	Subject	Narrow	r2	117.3154016	1	1	pre_focus	5	Narrow pre_focus
**2023408**	block5	Control	post	wai5	Subject	Narrow	r2	178.4235883	2	2	pre_focus	5	Narrow pre_focus
**2023408**	block5	Control	post	waat3	Verb	Narrow	r2	109.0602209	3	1	on_focus	3	Narrow on_focus
**2023408**	block5	Control	post	bui3	Object	Narrow	r2	87.21479396	4	1	post_focus	3	Narrow post_focus
**2023408**	block5	Control	post	hok3	Object	Narrow	r2	101.604903	5	2	post_focus	3	Narrow post_focus
**2023408**	block5	Control	post	wai5	Subject	Contrastive	r2	133.1449141	1	1	pre_focus	5	Contrastive pre_focus
**2023408**	block5	Control	post	wai5	Subject	Contrastive	r2	158.4773798	2	2	pre_focus	5	Contrastive pre_focus
**2023408**	block5	Control	post	waat3	Verb	Contrastive	r2	108.6883358	3	1	pre_focus	3	Contrastive pre_focus
**2023408**	block5	Control	post	bui3	Object	Contrastive	r2	105.4801208	4	1	on_focus	3	Contrastive on_focus
**2023408**	block5	Control	post	hok3	Object	Contrastive	r2	49.38215366	5	2	on_focus	3	Contrastive on_focus
**2023408**	block5	Control	post	ceoi3	Subject	Narrow	r2	88.75146018	1	1	pre_focus	3	Narrow pre_focus
**2023408**	block5	Control	post	ceoi3	Subject	Narrow	r2	179.5044622	2	2	pre_focus	3	Narrow pre_focus
**2023408**	block5	Control	post	caa4	Verb	Narrow	r2	91.27791895	3	1	pre_focus	4	Narrow pre_focus
**2023408**	block5	Control	post	ngau4	Object	Narrow	r2	173.0937407	4	1	on_focus	4	Narrow on_focus
**2023408**	block5	Control	post	jau4	Object	Narrow	r2	109.1614629	5	2	on_focus	4	Narrow on_focus
**2023408**	block5	Control	post	siu2	Subject	Narrow	r2	156.6143714	1	1	pre_focus	2	Narrow pre_focus
**2023408**	block5	Control	post	gwong2	Subject	Narrow	r2	184.7806079	2	2	pre_focus	2	Narrow pre_focus
**2023408**	block5	Control	post	cyun4	Verb	Narrow	r2	171.8891539	3	1	on_focus	4	Narrow on_focus
**2023408**	block5	Control	post	laam4	Object	Narrow	r2	246.1434846	4	1	post_focus	4	Narrow post_focus
**2023408**	block5	Control	post	kau4	Object	Narrow	r2	155.4448764	5	2	post_focus	4	Narrow post_focus
**2023408**	block5	Control	post	ceoi3	Subject	Contrastive	r2	91.56979261	1	1	on_focus	3	Contrastive on_focus
**2023408**	block5	Control	post	ceoi3	Subject	Contrastive	r2	92.15824425	2	2	on_focus	3	Contrastive on_focus
**2023408**	block5	Control	post	caa4	Verb	Contrastive	r2	105.7606033	3	1	post_focus	4	Contrastive post_focus
**2023408**	block5	Control	post	ngau4	Object	Contrastive	r2	163.5519607	4	1	post_focus	4	Contrastive post_focus
**2023408**	block5	Control	post	jau4	Object	Contrastive	r2	205.2834467	5	2	post_focus	4	Contrastive post_focus
**2023408**	block5	Control	post	ceoi3	Subject	Narrow	r2	124.9514331	1	1	on_focus	3	Narrow on_focus
**2023408**	block5	Control	post	ceoi3	Subject	Narrow	r2	119.0192132	2	2	on_focus	3	Narrow on_focus
**2023408**	block5	Control	post	caa4	Verb	Narrow	r2	155.0040719	3	1	post_focus	4	Narrow post_focus
**2023408**	block5	Control	post	ngau4	Object	Narrow	r2	133.9129855	4	1	post_focus	4	Narrow post_focus
**2023408**	block5	Control	post	jau4	Object	Narrow	r2	155.6159515	5	2	post_focus	4	Narrow post_focus
**2023408**	block5	Control	post	wai5	Subject	Contrastive	r2	214.6485261	1	1	pre_focus	5	Contrastive pre_focus
**2023408**	block5	Control	post	wai5	Subject	Contrastive	r2	175.280721	2	2	pre_focus	5	Contrastive pre_focus

**2023408**	block5	Control	post	waat3	Verb	Contrastive	r2	112.8004654	3	1	on_focus	3	Contrastive on_focus
**2023408**	block5	Control	post	bui3	Object	Contrastive	r2	96.71247864	4	1	post_focus	3	Contrastive post_focus
**2023408**	block5	Control	post	hok3	Object	Contrastive	r2	118.308768	5	2	post_focus	3	Contrastive post_focus
**2023408**	block5	Control	pre	wai5	Subject	Contrastive	r1	169.321155	1	1	pre_focus	5	Contrastive pre_focus
**2023408**	block5	Control	pre	wai5	Subject	Contrastive	r1	184.3145089	2	2	pre_focus	5	Contrastive pre_focus
**2023408**	block5	Control	pre	waat3	Verb	Contrastive	r1	198.1846183	3	1	on_focus	3	Contrastive on_focus
**2023408**	block5	Control	pre	bui3	Object	Contrastive	r1	130.0682432	4	1	post_focus	3	Contrastive post_focus
**2023408**	block5	Control	pre	hok3	Object	Contrastive	r1	55.03434066	5	2	post_focus	3	Contrastive post_focus
**2023408**	block5	Control	pre	wai5	Subject	Narrow	r1	119.4111995	1	1	on_focus	5	Narrow on_focus
**2023408**	block5	Control	pre	wai5	Subject	Narrow	r1	121.2432559	2	2	on_focus	5	Narrow on_focus
**2023408**	block5	Control	pre	waat3	Verb	Narrow	r1	147.8423006	3	1	post_focus	3	Narrow post_focus
**2023408**	block5	Control	pre	bui3	Object	Narrow	r1	128.9037833	4	1	post_focus	3	Narrow post_focus
**2023408**	block5	Control	pre	hok3	Object	Narrow	r1	82.69551688	5	2	post_focus	3	Narrow post_focus
**2023408**	block5	Control	pre	ceoi3	Subject	Contrastive	r1	110.8320617	1	1	on_focus	3	Contrastive on_focus
**2023408**	block5	Control	pre	ceoi3	Subject	Contrastive	r1	126.8686119	2	2	on_focus	3	Contrastive on_focus
**2023408**	block5	Control	pre	caa4	Verb	Contrastive	r1	67.43197279	3	1	post_focus	4	Contrastive post_focus
**2023408**	block5	Control	pre	ngau4	Object	Contrastive	r1	141.7744204	4	1	post_focus	4	Contrastive post_focus
**2023408**	block5	Control	pre	jau4	Object	Contrastive	r1	104.4444444	5	2	post_focus	4	Contrastive post_focus
**2023408**	block5	Control	pre	wai5	Subject	Narrow	r1	89.16964119	1	1	pre_focus	5	Narrow pre_focus
**2023408**	block5	Control	pre	wai5	Subject	Narrow	r1	121.4040978	2	2	pre_focus	5	Narrow pre_focus
**2023408**	block5	Control	pre	waat3	Verb	Narrow	r1	123.2517465	3	1	pre_focus	3	Narrow pre_focus
**2023408**	block5	Control	pre	bui3	Object	Narrow	r1	116.1517385	4	1	on_focus	3	Narrow on_focus
**2023408**	block5	Control	pre	hok3	Object	Narrow	r1	66.05102041	5	2	on_focus	3	Narrow on_focus
**2023408**	block5	Control	pre	ceoi3	Subject	Narrow	r1	55.43272865	1	1	pre_focus	3	Narrow pre_focus
**2023408**	block5	Control	pre	ceoi3	Subject	Narrow	r1	101.6172087	2	2	pre_focus	3	Narrow pre_focus
**2023408**	block5	Control	pre	caa4	Verb	Narrow	r1	120.7529559	3	1	on_focus	4	Narrow on_focus
**2023408**	block5	Control	pre	ngau4	Object	Narrow	r1	83.77005123	4	1	post_focus	4	Narrow post_focus
**2023408**	block5	Control	pre	jau4	Object	Narrow	r1	76.37229437	5	2	post_focus	4	Narrow post_focus
**2023408**	block5	Control	pre	ceoi3	Subject	Narrow	r1	62.96879387	1	1	on_focus	3	Narrow on_focus
**2023408**	block5	Control	pre	ceoi3	Subject	Narrow	r1	71.81772372	2	2	on_focus	3	Narrow on_focus
**2023408**	block5	Control	pre	caa4	Verb	Narrow	r1	61.61413454	3	1	post_focus	4	Narrow post_focus
**2023408**	block5	Control	pre	ngau4	Object	Narrow	r1	97.54650112	4	1	post_focus	4	Narrow post_focus
**2023408**	block5	Control	pre	jau4	Object	Narrow	r1	92.38630637	5	2	post_focus	4	Narrow post_focus
**2023408**	block5	Control	pre	siu2	Subject	Narrow	r1	97.36205593	1	1	pre_focus	2	Narrow pre_focus
**2023408**	block5	Control	pre	gwong2	Subject	Narrow	r1	155.1918267	2	2	pre_focus	2	Narrow pre_focus
**2023408**	block5	Control	pre	cyun4	Verb	Narrow	r1	75.44609113	3	1	on_focus	4	Narrow on_focus
**2023408**	block5	Control	pre	laam4	Object	Narrow	r1	134.7405788	4	1	post_focus	4	Narrow post_focus
**2023408**	block5	Control	pre	kau4	Object	Narrow	r1	65.85185185	5	2	post_focus	4	Narrow post_focus
**2023408**	block5	Control	pre	wai5	Subject	Contrastive	r1	182.4437467	1	1	pre_focus	5	Contrastive pre_focus
**2023408**	block5	Control	pre	wai5	Subject	Contrastive	r1	123.5985942	2	2	pre_focus	5	Contrastive pre_focus
**2023408**	block5	Control	pre	waat3	Verb	Contrastive	r1	114.7026886	3	1	pre_focus	3	Contrastive pre_focus
**2023408**	block5	Control	pre	bui3	Object	Contrastive	r1	117.2477324	4	1	on_focus	3	Contrastive on_focus
**2023408**	block5	Control	pre	hok3	Object	Contrastive	r1	86.98954397	5	2	on_focus	3	Contrastive on_focus
**2023408**	block5	Control	pre	siu2	Subject	Contrastive	r1	88.14467737	1	1	pre_focus	2	Contrastive pre_focus
**2023408**	block5	Control	pre	gwong2	Subject	Contrastive	r1	174.4272524	2	2	pre_focus	2	Contrastive pre_focus
**2023408**	block5	Control	pre	cyun4	Verb	Contrastive	r1	126.5326909	3	1	pre_focus	4	Contrastive pre_focus
**2023408**	block5	Control	pre	laam4	Object	Contrastive	r1	204.7667758	4	1	on_focus	4	Contrastive on_focus
**2023408**	block5	Control	pre	kau4	Object	Contrastive	r1	97.43441043	5	2	on_focus	4	Contrastive on_focus
**2023408**	block5	Control	pre	siu2	Subject	Contrastive	r1	79.9425548	1	1	pre_focus	2	Contrastive pre_focus
**2023408**	block5	Control	pre	gwong2	Subject	Contrastive	r1	182.7285323	2	2	pre_focus	2	Contrastive pre_focus
**2023408**	block5	Control	pre	cyun4	Verb	Contrastive	r1	98.86190476	3	1	on_focus	4	Contrastive on_focus
**2023408**	block5	Control	pre	laam4	Object	Contrastive	r1	195.6617192	4	1	post_focus	4	Contrastive post_focus
**2023408**	block5	Control	pre	kau4	Object	Contrastive	r1	98.26656589	5	2	post_focus	4	Contrastive post_focus
**2023408**	block5	Control	pre	wai5	Subject	Broad	r1	145.7287933	1	1	broad_focus	5	Broad focus
**2023408**	block5	Control	pre	wai5	Subject	Broad	r1	159.1140009	2	2	broad_focus	5	Broad focus
**2023408**	block5	Control	pre	waat3	Verb	Broad	r1	129.2010234	3	1	broad_focus	3	Broad focus
**2023408**	block5	Control	pre	bui3	Object	Broad	r1	78.52527899	4	1	broad_focus	3	Broad focus
**2023408**	block5	Control	pre	hok3	Object	Broad	r1	63.31628391	5	2	broad_focus	3	Broad focus
**2023408**	block5	Control	pre	ceoi3	Subject	Contrastive	r1	100.227113	1	1	pre_focus	3	Contrastive pre_focus
**2023408**	block5	Control	pre	ceoi3	Subject	Contrastive	r1	96.72004069	2	2	pre_focus	3	Contrastive pre_focus
**2023408**	block5	Control	pre	caa4	Verb	Contrastive	r1	87.64689052	3	1	on_focus	4	Contrastive on_focus
**2023408**	block5	Control	pre	ngau4	Object	Contrastive	r1	158.473167	4	1	post_focus	4	Contrastive post_focus
**2023408**	block5	Control	pre	jau4	Object	Contrastive	r1	79.31790158	5	2	post_focus	4	Contrastive post_focus
**2023408**	block5	Control	pre	siu2	Subject	Broad	r1	151.2239859	1	1	broad_focus	2	Broad focus
**2023408**	block5	Control	pre	gwong2	Subject	Broad	r1	205.9435913	2	2	broad_focus	2	Broad focus
**2023408**	block5	Control	pre	cyun4	Verb	Broad	r1	130.8171634	3	1	broad_focus	4	Broad focus

**2023408**	block5	Control	pre	laam4	Object	Broad	r1	191.2343047	4	1	broad_focus	4	Broad focus
**2023408**	block5	Control	pre	kau4	Object	Broad	r1	91.4760825	5	2	broad_focus	4	Broad focus
**2023408**	block5	Control	pre	siu2	Subject	Narrow	r1	106.1941101	1	1	on_focus	2	Narrow on_focus
**2023408**	block5	Control	pre	gwong2	Subject	Narrow	r1	166.7573696	2	2	on_focus	2	Narrow on_focus
**2023408**	block5	Control	pre	cyun4	Verb	Narrow	r1	110.8163265	3	1	post_focus	4	Narrow post_focus
**2023408**	block5	Control	pre	laam4	Object	Narrow	r1	196.3732318	4	1	post_focus	4	Narrow post_focus
**2023408**	block5	Control	pre	kau4	Object	Narrow	r1	98.35298564	5	2	post_focus	4	Narrow post_focus
**2023408**	block5	Control	pre	wai5	Subject	Contrastive	r1	123.3994616	1	1	on_focus	5	Contrastive on_focus
**2023408**	block5	Control	pre	wai5	Subject	Contrastive	r1	229.0229083	2	2	on_focus	5	Contrastive on_focus
**2023408**	block5	Control	pre	waat3	Verb	Contrastive	r1	135.5881836	3	1	post_focus	3	Contrastive post_focus
**2023408**	block5	Control	pre	bui3	Object	Contrastive	r1	83.69352869	4	1	post_focus	3	Contrastive post_focus
**2023408**	block5	Control	pre	hok3	Object	Contrastive	r1	112.8169879	5	2	post_focus	3	Contrastive post_focus
**2023408**	block5	Control	pre	ceoi3	Subject	Broad	r1	76.01979293	1	1	broad_focus	3	Broad focus
**2023408**	block5	Control	pre	ceoi3	Subject	Broad	r1	92.91640899	2	2	broad_focus	3	Broad focus
**2023408**	block5	Control	pre	caa4	Verb	Broad	r1	153.3272865	3	1	broad_focus	4	Broad focus
**2023408**	block5	Control	pre	ngau4	Object	Broad	r1	56.32125335	4	1	broad_focus	4	Broad focus
**2023408**	block5	Control	pre	jau4	Object	Broad	r1	136.7054216	5	2	broad_focus	4	Broad focus
**2023408**	block5	Control	pre	siu2	Subject	Contrastive	r1	104.5762063	1	1	on_focus	2	Contrastive on_focus
**2023408**	block5	Control	pre	gwong2	Subject	Contrastive	r1	201.2071967	2	2	on_focus	2	Contrastive on_focus
**2023408**	block5	Control	pre	cyun4	Verb	Contrastive	r1	131.1622974	3	1	post_focus	4	Contrastive post_focus
**2023408**	block5	Control	pre	laam4	Object	Contrastive	r1	245.8454271	4	1	post_focus	4	Contrastive post_focus
**2023408**	block5	Control	pre	kau4	Object	Contrastive	r1	89.13252708	5	2	post_focus	4	Contrastive post_focus
**2023408**	block5	Control	pre	siu2	Subject	Narrow	r1	153.6147644	1	1	pre_focus	2	Narrow pre_focus
**2023408**	block5	Control	pre	gwong2	Subject	Narrow	r1	165.5404901	2	2	pre_focus	2	Narrow pre_focus
**2023408**	block5	Control	pre	cyun4	Verb	Narrow	r1	94.93005678	3	1	pre_focus	4	Narrow pre_focus
**2023408**	block5	Control	pre	laam4	Object	Narrow	r1	214.3295572	4	1	on_focus	4	Narrow on_focus
**2023408**	block5	Control	pre	kau4	Object	Narrow	r1	62.36136879	5	2	on_focus	4	Narrow on_focus
**2023408**	block5	Control	pre	ceoi3	Subject	Narrow	r1	85.73008107	1	1	pre_focus	3	Narrow pre_focus
**2023408**	block5	Control	pre	ceoi3	Subject	Narrow	r1	136.9460821	2	2	pre_focus	3	Narrow pre_focus
**2023408**	block5	Control	pre	caa4	Verb	Narrow	r1	91.68801634	3	1	pre_focus	4	Narrow pre_focus
**2023408**	block5	Control	pre	ngau4	Object	Narrow	r1	108.4454972	4	1	on_focus	4	Narrow on_focus
**2023408**	block5	Control	pre	jau4	Object	Narrow	r1	182.0483405	5	2	on_focus	4	Narrow on_focus
**2023408**	block5	Control	pre	wai5	Subject	Narrow	r1	99.41381917	1	1	pre_focus	5	Narrow pre_focus
**2023408**	block5	Control	pre	wai5	Subject	Narrow	r1	179.9629048	2	2	pre_focus	5	Narrow pre_focus
**2023408**	block5	Control	pre	waat3	Verb	Narrow	r1	131.3065132	3	1	on_focus	3	Narrow on_focus
**2023408**	block5	Control	pre	bui3	Object	Narrow	r1	84.68615815	4	1	post_focus	3	Narrow post_focus
**2023408**	block5	Control	pre	hok3	Object	Narrow	r1	65.48627356	5	2	post_focus	3	Narrow post_focus
**2023408**	block5	Control	pre	ceoi3	Subject	Contrastive	r1	53.57057823	1	1	pre_focus	3	Contrastive pre_focus
**2023408**	block5	Control	pre	ceoi3	Subject	Contrastive	r1	65.41522164	2	2	pre_focus	3	Contrastive pre_focus
**2023408**	block5	Control	pre	caa4	Verb	Contrastive	r1	80.37674329	3	1	pre_focus	4	Contrastive pre_focus
**2023408**	block5	Control	pre	ngau4	Object	Contrastive	r1	110.4631729	4	1	on_focus	4	Contrastive on_focus
**2023408**	block5	Control	pre	jau4	Object	Contrastive	r1	144.2748283	5	2	on_focus	4	Contrastive on_focus
**2023408**	block5	Control	pre	wai5	Subject	Narrow	r2	123.4492888	1	1	pre_focus	5	Narrow pre_focus
**2023408**	block5	Control	pre	wai5	Subject	Narrow	r2	167.5338181	2	2	pre_focus	5	Narrow pre_focus
**2023408**	block5	Control	pre	waat3	Verb	Narrow	r2	100.860709	3	1	on_focus	3	Narrow on_focus
**2023408**	block5	Control	pre	bui3	Object	Narrow	r2	125.2496075	4	1	post_focus	3	Narrow post_focus
**2023408**	block5	Control	pre	hok3	Object	Narrow	r2	77.3872512	5	2	post_focus	3	Narrow post_focus
**2023408**	block5	Control	pre	wai5	Subject	Broad	r2	102.7882734	1	1	broad_focus	5	Broad focus
**2023408**	block5	Control	pre	wai5	Subject	Broad	r2	103.0928298	2	2	broad_focus	5	Broad focus
**2023408**	block5	Control	pre	waat3	Verb	Broad	r2	135.3917863	3	1	broad_focus	3	Broad focus
**2023408**	block5	Control	pre	bui3	Object	Broad	r2	132.7156179	4	1	broad_focus	3	Broad focus
**2023408**	block5	Control	pre	hok3	Object	Broad	r2	67.66187957	5	2	broad_focus	3	Broad focus
**2023408**	block5	Control	pre	siu2	Subject	Contrastive	r2	146.704396	1	1	pre_focus	2	Contrastive pre_focus
**2023408**	block5	Control	pre	gwong2	Subject	Contrastive	r2	189.1702075	2	2	pre_focus	2	Contrastive pre_focus
**2023408**	block5	Control	pre	cyun4	Verb	Contrastive	r2	127.0961307	3	1	on_focus	4	Contrastive on_focus
**2023408**	block5	Control	pre	laam4	Object	Contrastive	r2	194.882842	4	1	post_focus	4	Contrastive post_focus
**2023408**	block5	Control	pre	kau4	Object	Contrastive	r2	92.87913145	5	2	post_focus	4	Contrastive post_focus
**2023408**	block5	Control	pre	wai5	Subject	Contrastive	r2	138.4479145	1	1	on_focus	5	Contrastive on_focus
**2023408**	block5	Control	pre	wai5	Subject	Contrastive	r2	156.7898672	2	2	on_focus	5	Contrastive on_focus
**2023408**	block5	Control	pre	waat3	Verb	Contrastive	r2	102.1315844	3	1	post_focus	3	Contrastive post_focus
**2023408**	block5	Control	pre	bui3	Object	Contrastive	r2	84.43304428	4	1	post_focus	3	Contrastive post_focus
**2023408**	block5	Control	pre	hok3	Object	Contrastive	r2	105.2639844	5	2	post_focus	3	Contrastive post_focus
**2023408**	block5	Control	pre	ceoi3	Subject	Contrastive	r2	76.02090487	1	1	on_focus	3	Contrastive on_focus
**2023408**	block5	Control	pre	ceoi3	Subject	Contrastive	r2	82.58563425	2	2	on_focus	3	Contrastive on_focus
**2023408**	block5	Control	pre	caa4	Verb	Contrastive	r2	74.63455914	3	1	post_focus	4	Contrastive post_focus
**2023408**	block5	Control	pre	ngau4	Object	Contrastive	r2	162.9760285	4	1	post_focus	4	Contrastive post_focus

2023408	block5	Control	pre	jau4	Object	Contrastive	r2	159.0621063	5	2	post_focus	4	Contrastive post_focus
2023408	block5	Control	pre	wai5	Subject	Contrastive	r2	157.837791	1	1	pre_focus	5	Contrastive pre_focus
2023408	block5	Control	pre	wai5	Subject	Contrastive	r2	164.1475394	2	2	pre_focus	5	Contrastive pre_focus
2023408	block5	Control	pre	waat3	Verb	Contrastive	r2	106.4382896	3	1	on_focus	3	Contrastive on_focus
2023408	block5	Control	pre	bui3	Object	Contrastive	r2	114.1538244	4	1	post_focus	3	Contrastive post_focus
2023408	block5	Control	pre	hok3	Object	Contrastive	r2	81.41558012	5	2	post_focus	3	Contrastive post_focus
2023408	block5	Control	pre	siu2	Subject	Contrastive	r2	104.2609771	1	1	on_focus	2	Contrastive on_focus
2023408	block5	Control	pre	gwong2	Subject	Contrastive	r2	170.2570465	2	2	on_focus	2	Contrastive on_focus
2023408	block5	Control	pre	cyun4	Verb	Contrastive	r2	123.7058694	3	1	post_focus	4	Contrastive post_focus
2023408	block5	Control	pre	laam4	Object	Contrastive	r2	172.99178	4	1	post_focus	4	Contrastive post_focus
2023408	block5	Control	pre	kau4	Object	Contrastive	r2	81.86559472	5	2	post_focus	4	Contrastive post_focus
2023408	block5	Control	pre	siu2	Subject	Contrastive	r2	134.8328056	1	1	pre_focus	2	Contrastive pre_focus
2023408	block5	Control	pre	gwong2	Subject	Contrastive	r2	192.9183603	2	2	pre_focus	2	Contrastive pre_focus
2023408	block5	Control	pre	cyun4	Verb	Contrastive	r2	98.50696469	3	1	pre_focus	4	Contrastive pre_focus
2023408	block5	Control	pre	laam4	Object	Contrastive	r2	188.9920269	4	1	on_focus	4	Contrastive on_focus
2023408	block5	Control	pre	kau4	Object	Contrastive	r2	52.08238851	5	2	on_focus	4	Contrastive on_focus
2023408	block5	Control	pre	siu2	Subject	Narrow	r2	133.9543151	1	1	pre_focus	2	Narrow pre_focus
2023408	block5	Control	pre	gwong2	Subject	Narrow	r2	130.3471132	2	2	pre_focus	2	Narrow pre_focus
2023408	block5	Control	pre	cyun4	Verb	Narrow	r2	95.20751735	3	1	pre_focus	4	Narrow pre_focus
2023408	block5	Control	pre	laam4	Object	Narrow	r2	210.3613001	4	1	on_focus	4	Narrow on_focus
2023408	block5	Control	pre	kau4	Object	Narrow	r2	79.64778985	5	2	on_focus	4	Narrow on_focus
2023408	block5	Control	pre	ceoi3	Subject	Narrow	r2	88.2972583	1	1	pre_focus	3	Narrow pre_focus
2023408	block5	Control	pre	ceoi3	Subject	Narrow	r2	98.47529052	2	2	pre_focus	3	Narrow pre_focus
2023408	block5	Control	pre	caa4	Verb	Narrow	r2	63.3728453	3	1	pre_focus	4	Narrow pre_focus
2023408	block5	Control	pre	ngau4	Object	Narrow	r2	107.2093628	4	1	on_focus	4	Narrow on_focus
2023408	block5	Control	pre	jau4	Object	Narrow	r2	91.21434121	5	2	on_focus	4	Narrow on_focus
2023408	block5	Control	pre	wai5	Subject	Narrow	r2	107.1215897	1	1	pre_focus	5	Narrow pre_focus
2023408	block5	Control	pre	wai5	Subject	Narrow	r2	133.2131889	2	2	pre_focus	5	Narrow pre_focus
2023408	block5	Control	pre	waat3	Verb	Narrow	r2	115.7263589	3	1	pre_focus	3	Narrow pre_focus
2023408	block5	Control	pre	bui3	Object	Narrow	r2	73.8909405	4	1	on_focus	3	Narrow on_focus
2023408	block5	Control	pre	hok3	Object	Narrow	r2	50.46481921	5	2	on_focus	3	Narrow on_focus
2023408	block5	Control	pre	siu2	Subject	Narrow	r2	112.0831444	1	1	on_focus	2	Narrow on_focus
2023408	block5	Control	pre	gwong2	Subject	Narrow	r2	192.5245654	2	2	on_focus	2	Narrow on_focus
2023408	block5	Control	pre	cyun4	Verb	Narrow	r2	79.78210678	3	1	post_focus	4	Narrow post_focus
2023408	block5	Control	pre	laam4	Object	Narrow	r2	149.5843004	4	1	post_focus	4	Narrow post_focus
2023408	block5	Control	pre	kau4	Object	Narrow	r2	86.30756545	5	2	post_focus	4	Narrow post_focus
2023408	block5	Control	pre	wai5	Subject	Contrastive	r2	112.3110834	1	1	pre_focus	5	Contrastive pre_focus
2023408	block5	Control	pre	wai5	Subject	Contrastive	r2	121.976886	2	2	pre_focus	5	Contrastive pre_focus
2023408	block5	Control	pre	waat3	Verb	Contrastive	r2	152.4373178	3	1	pre_focus	3	Contrastive pre_focus
2023408	block5	Control	pre	bui3	Object	Contrastive	r2	133.2736603	4	1	on_focus	3	Contrastive on_focus
2023408	block5	Control	pre	hok3	Object	Contrastive	r2	73.02535054	5	2	on_focus	3	Contrastive on_focus
2023408	block5	Control	pre	ceoi3	Subject	Broad	r2	86.86271731	1	1	broad_focus	3	Broad focus
2023408	block5	Control	pre	ceoi3	Subject	Broad	r2	105.6009192	2	2	broad_focus	3	Broad focus
2023408	block5	Control	pre	caa4	Verb	Broad	r2	72.69864527	3	1	broad_focus	4	Broad focus
2023408	block5	Control	pre	ngau4	Object	Broad	r2	63.93109095	4	1	broad_focus	4	Broad focus
2023408	block5	Control	pre	jau4	Object	Broad	r2	117.2132101	5	2	broad_focus	4	Broad focus
2023408	block5	Control	pre	siu2	Subject	Broad	r2	84.62090819	1	1	broad_focus	2	Broad focus
2023408	block5	Control	pre	gwong2	Subject	Broad	r2	128.350907	2	2	broad_focus	2	Broad focus
2023408	block5	Control	pre	cyun4	Verb	Broad	r2	86.6606198	3	1	broad_focus	4	Broad focus
2023408	block5	Control	pre	laam4	Object	Broad	r2	201.08311	4	1	broad_focus	4	Broad focus
2023408	block5	Control	pre	kau4	Object	Broad	r2	94.98488284	5	2	broad_focus	4	Broad focus
2023408	block5	Control	pre	siu2	Subject	Narrow	r2	115.6849287	1	1	pre_focus	2	Narrow pre_focus
2023408	block5	Control	pre	gwong2	Subject	Narrow	r2	175.3950811	2	2	pre_focus	2	Narrow pre_focus
2023408	block5	Control	pre	cyun4	Verb	Narrow	r2	104.2257856	3	1	on_focus	4	Narrow on_focus
2023408	block5	Control	pre	laam4	Object	Narrow	r2	176.202437	4	1	post_focus	4	Narrow post_focus
2023408	block5	Control	pre	kau4	Object	Narrow	r2	75.88813303	5	2	post_focus	4	Narrow post_focus
2023408	block5	Control	pre	wai5	Subject	Narrow	r2	106.6430857	1	1	on_focus	5	Narrow on_focus
2023408	block5	Control	pre	wai5	Subject	Narrow	r2	156.6831889	2	2	on_focus	5	Narrow on_focus
2023408	block5	Control	pre	waat3	Verb	Narrow	r2	127.987634	3	1	post_focus	3	Narrow post_focus
2023408	block5	Control	pre	bui3	Object	Narrow	r2	79.73245533	4	1	post_focus	3	Narrow post_focus
2023408	block5	Control	pre	hok3	Object	Narrow	r2	63.83585286	5	2	post_focus	3	Narrow post_focus
2023408	block5	Control	pre	ceoi3	Subject	Narrow	r2	62.24819327	1	1	on_focus	3	Narrow on_focus
2023408	block5	Control	pre	ceoi3	Subject	Narrow	r2	114.8836519	2	2	on_focus	3	Narrow on_focus
2023408	block5	Control	pre	caa4	Verb	Narrow	r2	78.56366843	3	1	post_focus	4	Narrow post_focus
2023408	block5	Control	pre	ngau4	Object	Narrow	r2	147.2137715	4	1	post_focus	4	Narrow post_focus
2023408	block5	Control	pre	jau4	Object	Narrow	r2	102.6657675	5	2	post_focus	4	Narrow post_focus

2023408	block5	Control	pre	ceoi3	Subject	Contrastive	r2	50.47704941	1	1	pre_focus	3	Contrastive pre_focus
2023408	block5	Control	pre	ceoi3	Subject	Contrastive	r2	110.8988543	2	2	pre_focus	3	Contrastive pre_focus
2023408	block5	Control	pre	caa4	Verb	Contrastive	r2	55.46910079	3	1	on_focus	4	Contrastive on_focus
2023408	block5	Control	pre	ngau4	Object	Contrastive	r2	198.4824958	4	1	post_focus	4	Contrastive post_focus
2023408	block5	Control	pre	jau4	Object	Contrastive	r2	191.8690536	5	2	post_focus	4	Contrastive post_focus
2023408	block5	Control	pre	ceoi3	Subject	Contrastive	r2	68.64547351	1	1	pre_focus	3	Contrastive pre_focus
2023408	block5	Control	pre	ceoi3	Subject	Contrastive	r2	81.38277241	2	2	pre_focus	3	Contrastive pre_focus
2023408	block5	Control	pre	caa4	Verb	Contrastive	r2	80.64154802	3	1	pre_focus	4	Contrastive pre_focus
2023408	block5	Control	pre	ngau4	Object	Contrastive	r2	141.4482102	4	1	on_focus	4	Contrastive on_focus
2023408	block5	Control	pre	jau4	Object	Contrastive	r2	164.6378551	5	2	on_focus	4	Contrastive on_focus
2023408	block5	Control	pre	ceoi3	Subject	Narrow	r2	77.25771544	1	1	pre_focus	3	Narrow pre_focus
2023408	block5	Control	pre	ceoi3	Subject	Narrow	r2	119.6720417	2	2	pre_focus	3	Narrow pre_focus
2023408	block5	Control	pre	caa4	Verb	Narrow	r2	75.37036069	3	1	on_focus	4	Narrow on_focus
2023408	block5	Control	pre	ngau4	Object	Narrow	r2	177.5957077	4	1	post_focus	4	Narrow post_focus
2023408	block5	Control	pre	jau4	Object	Narrow	r2	167.2283272	5	2	post_focus	4	Narrow post_focus
2023410	block1	Control	post	zoeng1	Subject	Narrow	r1	220.1013957	1	1	on_focus	1	Narrow on_focus
2023410	block1	Control	post	saang1	Subject	Narrow	r1	236.494821	2	2	on_focus	1	Narrow on_focus
2023410	block1	Control	post	tsa1	Verb	Narrow	r1	167.088541	3	1	post_focus	1	Narrow post_focus
2023410	block1	Control	post	fei1	Object	Narrow	r1	184.0322379	4	1	post_focus	1	Narrow post_focus
2023410	block1	Control	post	gei1	Object	Narrow	r1	406.1264068	5	2	post_focus	1	Narrow post_focus
2023410	block1	Control	post	sau3	Subject	Contrastive	r1	168.4059911	1	1	pre_focus	3	Contrastive pre_focus
2023410	block1	Control	post	sau3	Subject	Contrastive	r1	118.878847	2	2	pre_focus	3	Contrastive pre_focus
2023410	block1	Control	post	sik3	Verb	Contrastive	r1	102.0180324	3	1	pre_focus	3	Contrastive pre_focus
2023410	block1	Control	post	baak3	Object	Contrastive	r1	131.4109779	4	1	on_focus	3	Contrastive on_focus
2023410	block1	Control	post	baak3	Object	Contrastive	r1	106.6359856	5	2	on_focus	3	Contrastive on_focus
2023410	block1	Control	post	zoeng1	Subject	Broad	r1	193.8682813	1	1	broad_focus	1	Broad focus
2023410	block1	Control	post	saang1	Subject	Broad	r1	223.2466078	2	2	broad_focus	1	Broad focus
2023410	block1	Control	post	tsa1	Verb	Broad	r1	163.5436871	3	1	broad_focus	1	Broad focus
2023410	block1	Control	post	fei1	Object	Broad	r1	136.1758749	4	1	broad_focus	1	Broad focus
2023410	block1	Control	post	gei1	Object	Broad	r1	307.7716698	5	2	broad_focus	1	Broad focus
2023410	block1	Control	post	zoeng1	Subject	Contrastive	r1	213.5151182	1	1	pre_focus	1	Contrastive pre_focus
2023410	block1	Control	post	saang1	Subject	Contrastive	r1	228.2075567	2	2	pre_focus	1	Contrastive pre_focus
2023410	block1	Control	post	tsa1	Verb	Contrastive	r1	149.3382272	3	1	pre_focus	1	Contrastive pre_focus
2023410	block1	Control	post	fei1	Object	Contrastive	r1	157.6770617	4	1	on_focus	1	Contrastive on_focus
2023410	block1	Control	post	gei1	Object	Contrastive	r1	366.9165998	5	2	on_focus	1	Contrastive on_focus
2023410	block1	Control	post	jyun2	Subject	Contrastive	r1	210.3108734	1	1	pre_focus	2	Contrastive pre_focus
2023410	block1	Control	post	jyun2	Subject	Contrastive	r1	221.1191063	2	2	pre_focus	2	Contrastive pre_focus
2023410	block1	Control	post	mo2	Verb	Contrastive	r1	162.025463	3	1	pre_focus	2	Contrastive pre_focus
2023410	block1	Control	post	gau2	Object	Contrastive	r1	168.3624976	4	1	on_focus	2	Contrastive on_focus
2023410	block1	Control	post	zai2	Object	Contrastive	r1	371.6738023	5	2	on_focus	2	Contrastive on_focus
2023410	block1	Control	post	sau3	Subject	Narrow	r1	158.7569972	1	1	pre_focus	3	Narrow pre_focus
2023410	block1	Control	post	sau3	Subject	Narrow	r1	114.885816	2	2	pre_focus	3	Narrow pre_focus
2023410	block1	Control	post	sik3	Verb	Narrow	r1	92.23204189	3	1	pre_focus	3	Narrow pre_focus
2023410	block1	Control	post	baak3	Object	Narrow	r1	166.9666818	4	1	on_focus	3	Narrow on_focus
2023410	block1	Control	post	baak3	Object	Narrow	r1	271.1775986	5	2	on_focus	3	Narrow on_focus
2023410	block1	Control	post	jyun2	Subject	Broad	r1	215.193659	1	1	broad_focus	2	Broad focus
2023410	block1	Control	post	jyun2	Subject	Broad	r1	212.7650789	2	2	broad_focus	2	Broad focus
2023410	block1	Control	post	mo2	Verb	Broad	r1	155.0933122	3	1	broad_focus	2	Broad focus
2023410	block1	Control	post	gau2	Object	Broad	r1	146.1456472	4	1	broad_focus	2	Broad focus
2023410	block1	Control	post	zai2	Object	Broad	r1	411.9350404	5	2	broad_focus	2	Broad focus
2023410	block1	Control	post	sau3	Subject	Contrastive	r1	141.7937111	1	1	on_focus	3	Contrastive on_focus
2023410	block1	Control	post	sau3	Subject	Contrastive	r1	113.2111297	2	2	on_focus	3	Contrastive on_focus
2023410	block1	Control	post	sik3	Verb	Contrastive	r1	87.10198581	3	1	post_focus	3	Contrastive post_focus
2023410	block1	Control	post	baak3	Object	Contrastive	r1	123.0314664	4	1	post_focus	3	Contrastive post_focus
2023410	block1	Control	post	baak3	Object	Contrastive	r1	184.2710353	5	2	post_focus	3	Contrastive post_focus
2023410	block1	Control	post	zoeng1	Subject	Contrastive	r1	175.3737747	1	1	on_focus	1	Contrastive on_focus
2023410	block1	Control	post	saang1	Subject	Contrastive	r1	192.6596655	2	2	on_focus	1	Contrastive on_focus
2023410	block1	Control	post	tsa1	Verb	Contrastive	r1	146.8199543	3	1	post_focus	1	Contrastive post_focus
2023410	block1	Control	post	fei1	Object	Contrastive	r1	129.2690507	4	1	post_focus	1	Contrastive post_focus
2023410	block1	Control	post	gei1	Object	Contrastive	r1	279.7451663	5	2	post_focus	1	Contrastive post_focus
2023410	block1	Control	post	sau3	Subject	Contrastive	r1	206.1055857	1	1	pre_focus	3	Contrastive pre_focus
2023410	block1	Control	post	sau3	Subject	Contrastive	r1	119.4581148	2	2	pre_focus	3	Contrastive pre_focus
2023410	block1	Control	post	sik3	Verb	Contrastive	r1	77.86135309	3	1	on_focus	3	Contrastive on_focus
2023410	block1	Control	post	baak3	Object	Contrastive	r1	115.1359637	4	1	post_focus	3	Contrastive post_focus
2023410	block1	Control	post	baak3	Object	Contrastive	r1	191.9999506	5	2	post_focus	3	Contrastive post_focus
2023410	block1	Control	post	sau3	Subject	Narrow	r1	143.307332	1	1	on_focus	3	Narrow on_focus

2023410	block1	Control	post	sau3	Subject	Narrow	r1	123.6407455	2	2	on_focus	3	Narrow on_focus
2023410	block1	Control	post	sik3	Verb	Narrow	r1	83.65187239	3	1	post_focus	3	Narrow post_focus
2023410	block1	Control	post	baak3	Object	Narrow	r1	96.52308645	4	1	post_focus	3	Narrow post_focus
2023410	block1	Control	post	baak3	Object	Narrow	r1	134.1951988	5	2	post_focus	3	Narrow post_focus
2023410	block1	Control	post	zoeng1	Subject	Contrastive	r1	153.5760668	1	1	pre_focus	1	Contrastive pre_focus
2023410	block1	Control	post	saang1	Subject	Contrastive	r1	154.3005945	2	2	pre_focus	1	Contrastive pre_focus
2023410	block1	Control	post	tsa1	Verb	Contrastive	r1	144.4895633	3	1	on_focus	1	Contrastive on_focus
2023410	block1	Control	post	fei1	Object	Contrastive	r1	149.0654765	4	1	post_focus	1	Contrastive post_focus
2023410	block1	Control	post	gei1	Object	Contrastive	r1	306.0266819	5	2	post_focus	1	Contrastive post_focus
2023410	block1	Control	post	jyun2	Subject	Narrow	r1	157.3801527	1	1	pre_focus	2	Narrow pre_focus
2023410	block1	Control	post	jyun2	Subject	Narrow	r1	161.3677861	2	2	pre_focus	2	Narrow pre_focus
2023410	block1	Control	post	mo2	Verb	Narrow	r1	173.3672553	3	1	pre_focus	2	Narrow pre_focus
2023410	block1	Control	post	gau2	Object	Narrow	r1	150.0459589	4	1	on_focus	2	Narrow on_focus
2023410	block1	Control	post	zai2	Object	Narrow	r1	399.0019445	5	2	on_focus	2	Narrow on_focus
2023410	block1	Control	post	sau3	Subject	Broad	r1	167.9663458	1	1	broad_focus	3	Broad focus
2023410	block1	Control	post	sau3	Subject	Broad	r1	105.8271665	2	2	broad_focus	3	Broad focus
2023410	block1	Control	post	sik3	Verb	Broad	r1	96.95636381	3	1	broad_focus	3	Broad focus
2023410	block1	Control	post	baak3	Object	Broad	r1	104.7568033	4	1	broad_focus	3	Broad focus
2023410	block1	Control	post	baak3	Object	Broad	r1	207.0835205	5	2	broad_focus	3	Broad focus
2023410	block1	Control	post	sau3	Subject	Narrow	r1	131.6808027	1	1	pre_focus	3	Narrow pre_focus
2023410	block1	Control	post	sau3	Subject	Narrow	r1	109.6292937	2	2	pre_focus	3	Narrow pre_focus
2023410	block1	Control	post	sik3	Verb	Narrow	r1	90.49033874	3	1	on_focus	3	Narrow on_focus
2023410	block1	Control	post	baak3	Object	Narrow	r1	88.16592381	4	1	post_focus	3	Narrow post_focus
2023410	block1	Control	post	baak3	Object	Narrow	r1	138.2392588	5	2	post_focus	3	Narrow post_focus
2023410	block1	Control	post	zoeng1	Subject	Narrow	r1	186.1492605	1	1	pre_focus	1	Narrow pre_focus
2023410	block1	Control	post	saang1	Subject	Narrow	r1	175.6107306	2	2	pre_focus	1	Narrow pre_focus
2023410	block1	Control	post	tsa1	Verb	Narrow	r1	164.4185131	3	1	on_focus	1	Narrow on_focus
2023410	block1	Control	post	fei1	Object	Narrow	r1	104.1525444	4	1	post_focus	1	Narrow post_focus
2023410	block1	Control	post	gei1	Object	Narrow	r1	325.6998196	5	2	post_focus	1	Narrow post_focus
2023410	block1	Control	post	jyun2	Subject	Contrastive	r1	181.0633836	1	1	pre_focus	2	Contrastive pre_focus
2023410	block1	Control	post	jyun2	Subject	Contrastive	r1	214.0907847	2	2	pre_focus	2	Contrastive pre_focus
2023410	block1	Control	post	mo2	Verb	Contrastive	r1	142.4651477	3	1	on_focus	2	Contrastive on_focus
2023410	block1	Control	post	gau2	Object	Contrastive	r1	156.6581082	4	1	post_focus	2	Contrastive post_focus
2023410	block1	Control	post	zai2	Object	Contrastive	r1	378.7845898	5	2	post_focus	2	Contrastive post_focus
2023410	block1	Control	post	jyun2	Subject	Contrastive	r1	223.4929909	1	1	on_focus	2	Contrastive on_focus
2023410	block1	Control	post	jyun2	Subject	Contrastive	r1	232.2981027	2	2	on_focus	2	Contrastive on_focus
2023410	block1	Control	post	mo2	Verb	Contrastive	r1	188.0361985	3	1	post_focus	2	Contrastive post_focus
2023410	block1	Control	post	gau2	Object	Contrastive	r1	180.861695	4	1	post_focus	2	Contrastive post_focus
2023410	block1	Control	post	zai2	Object	Contrastive	r1	444.135023	5	2	post_focus	2	Contrastive post_focus
2023410	block1	Control	post	jyun2	Subject	Narrow	r1	225.6802034	1	1	on_focus	2	Narrow on_focus
2023410	block1	Control	post	jyun2	Subject	Narrow	r1	156.388353	2	2	on_focus	2	Narrow on_focus
2023410	block1	Control	post	mo2	Verb	Narrow	r1	438.7696114	3	1	post_focus	2	Narrow post_focus
2023410	block1	Control	post	gau2	Object	Narrow	r1	240.7479778	4	1	post_focus	2	Narrow post_focus
2023410	block1	Control	post	zai2	Object	Narrow	r1	365.4859511	5	2	post_focus	2	Narrow post_focus
2023410	block1	Control	post	jyun2	Subject	Narrow	r1	421.2667144	1	1	pre_focus	2	Narrow pre_focus
2023410	block1	Control	post	jyun2	Subject	Narrow	r1	261.2201335	2	2	pre_focus	2	Narrow pre_focus
2023410	block1	Control	post	mo2	Verb	Narrow	r1	204.6447304	3	1	on_focus	2	Narrow on_focus
2023410	block1	Control	post	gau2	Object	Narrow	r1	193.3395458	4	1	post_focus	2	Narrow post_focus
2023410	block1	Control	post	zai2	Object	Narrow	r1	394.5371337	5	2	post_focus	2	Narrow post_focus
2023410	block1	Control	post	jyun2	Subject	Contrastive	r2	217.6590704	1	1	pre_focus	2	Contrastive pre_focus
2023410	block1	Control	post	jyun2	Subject	Contrastive	r2	228.6312979	2	2	pre_focus	2	Contrastive pre_focus
2023410	block1	Control	post	mo2	Verb	Contrastive	r2	205.0081002	3	1	on_focus	2	Contrastive on_focus
2023410	block1	Control	post	gau2	Object	Contrastive	r2	184.0140754	4	1	post_focus	2	Contrastive post_focus
2023410	block1	Control	post	zai2	Object	Contrastive	r2	347.4290133	5	2	post_focus	2	Contrastive post_focus
2023410	block1	Control	post	jyun2	Subject	Narrow	r2	222.5959604	1	1	on_focus	2	Narrow on_focus
2023410	block1	Control	post	jyun2	Subject	Narrow	r2	239.3738471	2	2	on_focus	2	Narrow on_focus
2023410	block1	Control	post	mo2	Verb	Narrow	r2	191.9147277	3	1	post_focus	2	Narrow post_focus
2023410	block1	Control	post	gau2	Object	Narrow	r2	190.1186727	4	1	post_focus	2	Narrow post_focus
2023410	block1	Control	post	zai2	Object	Narrow	r2	332.2567286	5	2	post_focus	2	Narrow post_focus
2023410	block1	Control	post	sau3	Subject	Narrow	r2	151.1520246	1	1	pre_focus	3	Narrow pre_focus
2023410	block1	Control	post	sau3	Subject	Narrow	r2	156.9672831	2	2	pre_focus	3	Narrow pre_focus
2023410	block1	Control	post	sik3	Verb	Narrow	r2	132.1356394	3	1	on_focus	3	Narrow on_focus
2023410	block1	Control	post	baak3	Object	Narrow	r2	154.9025815	4	1	post_focus	3	Narrow post_focus
2023410	block1	Control	post	baak3	Object	Narrow	r2	180.3695485	5	2	post_focus	3	Narrow post_focus
2023410	block1	Control	post	sau3	Subject	Contrastive	r2	147.6135444	1	1	on_focus	3	Contrastive on_focus
2023410	block1	Control	post	sau3	Subject	Contrastive	r2	132.107747	2	2	on_focus	3	Contrastive on_focus

2023410	block1	Control	post	sik3	Verb	Contrastive	r2	80.95337028	3	1	post_focus	3	Contrastive post_focus
2023410	block1	Control	post	baak3	Object	Contrastive	r2	131.138495	4	1	post_focus	3	Contrastive post_focus
2023410	block1	Control	post	baak3	Object	Contrastive	r2	166.947855	5	2	post_focus	3	Contrastive post_focus
2023410	block1	Control	post	sau3	Subject	Contrastive	r2	144.2136805	1	1	pre_focus	3	Contrastive pre_focus
2023410	block1	Control	post	sau3	Subject	Contrastive	r2	117.2108608	2	2	pre_focus	3	Contrastive pre_focus
2023410	block1	Control	post	sik3	Verb	Contrastive	r2	79.27059837	3	1	on_focus	3	Contrastive on_focus
2023410	block1	Control	post	baak3	Object	Contrastive	r2	104.3134061	4	1	post_focus	3	Contrastive post_focus
2023410	block1	Control	post	baak3	Object	Contrastive	r2	122.5128316	5	2	post_focus	3	Contrastive post_focus
2023410	block1	Control	post	zoeng1	Subject	Narrow	r2	166.5852859	1	1	on_focus	1	Narrow on_focus
2023410	block1	Control	post	saang1	Subject	Narrow	r2	161.2535957	2	2	on_focus	1	Narrow on_focus
2023410	block1	Control	post	tsa1	Verb	Narrow	r2	113.3720412	3	1	post_focus	1	Narrow post_focus
2023410	block1	Control	post	fei1	Object	Narrow	r2	110.5442981	4	1	post_focus	1	Narrow post_focus
2023410	block1	Control	post	gei1	Object	Narrow	r2	347.7878464	5	2	post_focus	1	Narrow post_focus
2023410	block1	Control	post	zoeng1	Subject	Contrastive	r2	170.109487	1	1	pre_focus	1	Contrastive pre_focus
2023410	block1	Control	post	saang1	Subject	Contrastive	r2	200.3043844	2	2	pre_focus	1	Contrastive pre_focus
2023410	block1	Control	post	tsa1	Verb	Contrastive	r2	148.837695	3	1	on_focus	1	Contrastive on_focus
2023410	block1	Control	post	fei1	Object	Contrastive	r2	145.3806524	4	1	post_focus	1	Contrastive post_focus
2023410	block1	Control	post	gei1	Object	Contrastive	r2	360.7758331	5	2	post_focus	1	Contrastive post_focus
2023410	block1	Control	post	sau3	Subject	Broad	r2	131.3120883	1	1	broad_focus	3	Broad focus
2023410	block1	Control	post	sau3	Subject	Broad	r2	100.2580692	2	2	broad_focus	3	Broad focus
2023410	block1	Control	post	sik3	Verb	Broad	r2	79.43226353	3	1	broad_focus	3	Broad focus
2023410	block1	Control	post	baak3	Object	Broad	r2	127.2797847	4	1	broad_focus	3	Broad focus
2023410	block1	Control	post	baak3	Object	Broad	r2	185.2409157	5	2	broad_focus	3	Broad focus
2023410	block1	Control	post	zoeng1	Subject	Broad	r2	188.3344774	1	1	broad_focus	1	Broad focus
2023410	block1	Control	post	saang1	Subject	Broad	r2	195.1092614	2	2	broad_focus	1	Broad focus
2023410	block1	Control	post	tsa1	Verb	Broad	r2	136.9556407	3	1	broad_focus	1	Broad focus
2023410	block1	Control	post	fei1	Object	Broad	r2	127.0226886	4	1	broad_focus	1	Broad focus
2023410	block1	Control	post	gei1	Object	Broad	r2	280.5929305	5	2	broad_focus	1	Broad focus
2023410	block1	Control	post	zoeng1	Subject	Contrastive	r2	232.4997919	1	1	pre_focus	1	Contrastive pre_focus
2023410	block1	Control	post	saang1	Subject	Contrastive	r2	280.7398971	2	2	pre_focus	1	Contrastive pre_focus
2023410	block1	Control	post	tsa1	Verb	Contrastive	r2	211.8713994	3	1	pre_focus	1	Contrastive pre_focus
2023410	block1	Control	post	fei1	Object	Contrastive	r2	123.906411	4	1	on_focus	1	Contrastive on_focus
2023410	block1	Control	post	gei1	Object	Contrastive	r2	294.2269069	5	2	on_focus	1	Contrastive on_focus
2023410	block1	Control	post	zoeng1	Subject	Contrastive	r2	194.4275033	1	1	on_focus	1	Contrastive on_focus
2023410	block1	Control	post	saang1	Subject	Contrastive	r2	245.8936038	2	2	on_focus	1	Contrastive on_focus
2023410	block1	Control	post	tsa1	Verb	Contrastive	r2	176.2285381	3	1	post_focus	1	Contrastive post_focus
2023410	block1	Control	post	fei1	Object	Contrastive	r2	134.7774658	4	1	post_focus	1	Contrastive post_focus
2023410	block1	Control	post	gei1	Object	Contrastive	r2	308.1063467	5	2	post_focus	1	Contrastive post_focus
2023410	block1	Control	post	sau3	Subject	Narrow	r2	136.0849193	1	1	on_focus	3	Narrow on_focus
2023410	block1	Control	post	sau3	Subject	Narrow	r2	108.4578778	2	2	on_focus	3	Narrow on_focus
2023410	block1	Control	post	sik3	Verb	Narrow	r2	105.497118	3	1	post_focus	3	Narrow post_focus
2023410	block1	Control	post	baak3	Object	Narrow	r2	92.07086601	4	1	post_focus	3	Narrow post_focus
2023410	block1	Control	post	baak3	Object	Narrow	r2	153.7711568	5	2	post_focus	3	Narrow post_focus
2023410	block1	Control	post	jyun2	Subject	Contrastive	r2	278.6353198	1	1	on_focus	2	Contrastive on_focus
2023410	block1	Control	post	jyun2	Subject	Contrastive	r2	261.4300818	2	2	on_focus	2	Contrastive on_focus
2023410	block1	Control	post	mo2	Verb	Contrastive	r2	157.391171	3	1	post_focus	2	Contrastive post_focus
2023410	block1	Control	post	gau2	Object	Contrastive	r2	186.6264996	4	1	post_focus	2	Contrastive post_focus
2023410	block1	Control	post	zai2	Object	Contrastive	r2	298.6091177	5	2	post_focus	2	Contrastive post_focus
2023410	block1	Control	post	jyun2	Subject	Narrow	r2	269.5944699	1	1	pre_focus	2	Narrow pre_focus
2023410	block1	Control	post	jyun2	Subject	Narrow	r2	216.6132802	2	2	pre_focus	2	Narrow pre_focus
2023410	block1	Control	post	mo2	Verb	Narrow	r2	161.8323317	3	1	on_focus	2	Narrow on_focus
2023410	block1	Control	post	gau2	Object	Narrow	r2	147.3622992	4	1	post_focus	2	Narrow post_focus
2023410	block1	Control	post	zai2	Object	Narrow	r2	319.1747982	5	2	post_focus	2	Narrow post_focus
2023410	block1	Control	post	sau3	Subject	Narrow	r2	188.2135721	1	1	pre_focus	3	Narrow pre_focus
2023410	block1	Control	post	sau3	Subject	Narrow	r2	290.189159	2	2	pre_focus	3	Narrow pre_focus
2023410	block1	Control	post	sik3	Verb	Narrow	r2	123.3412724	3	1	pre_focus	3	Narrow pre_focus
2023410	block1	Control	post	baak3	Object	Narrow	r2	152.3463956	4	1	on_focus	3	Narrow on_focus
2023410	block1	Control	post	baak3	Object	Narrow	r2	188.3355001	5	2	on_focus	3	Narrow on_focus
2023410	block1	Control	post	jyun2	Subject	Contrastive	r2	231.2939083	1	1	pre_focus	2	Contrastive pre_focus
2023410	block1	Control	post	jyun2	Subject	Contrastive	r2	336.0095257	2	2	pre_focus	2	Contrastive pre_focus
2023410	block1	Control	post	mo2	Verb	Contrastive	r2	210.1733912	3	1	pre_focus	2	Contrastive pre_focus
2023410	block1	Control	post	gau2	Object	Contrastive	r2	170.0585677	4	1	on_focus	2	Contrastive on_focus
2023410	block1	Control	post	zai2	Object	Contrastive	r2	306.8100929	5	2	on_focus	2	Contrastive on_focus
2023410	block1	Control	post	jyun2	Subject	Narrow	r2	372.1932734	1	1	pre_focus	2	Narrow pre_focus
2023410	block1	Control	post	jyun2	Subject	Narrow	r2	359.4719765	2	2	pre_focus	2	Narrow pre_focus
2023410	block1	Control	post	mo2	Verb	Narrow	r2	189.8562072	3	1	pre_focus	2	Narrow pre_focus

2023410	block1	Control	post	gau2	Object	Narrow	r2	207.9677215	4	1	on_focus	2	Narrow on_focus
2023410	block1	Control	post	zai2	Object	Narrow	r2	317.1859937	5	2	on_focus	2	Narrow on_focus
2023410	block1	Control	post	sau3	Subject	Contrastive	r2	163.1102203	1	1	pre_focus	3	Contrastive pre_focus
2023410	block1	Control	post	sau3	Subject	Contrastive	r2	131.1004521	2	2	pre_focus	3	Contrastive pre_focus
2023410	block1	Control	post	sik3	Verb	Contrastive	r2	92.41650912	3	1	pre_focus	3	Contrastive pre_focus
2023410	block1	Control	post	baak3	Object	Contrastive	r2	114.2023118	4	1	on_focus	3	Contrastive on_focus
2023410	block1	Control	post	baak3	Object	Contrastive	r2	167.0694187	5	2	on_focus	3	Contrastive on_focus
2023410	block1	Control	post	zoeng1	Subject	Narrow	r2	500.0226213	1	1	pre_focus	1	Narrow pre_focus
2023410	block1	Control	post	saang1	Subject	Narrow	r2	406.5437218	2	2	pre_focus	1	Narrow pre_focus
2023410	block1	Control	post	tsa1	Verb	Narrow	r2	250.2184257	3	1	pre_focus	1	Narrow pre_focus
2023410	block1	Control	post	fei1	Object	Narrow	r2	249.9091628	4	1	on_focus	1	Narrow on_focus
2023410	block1	Control	post	gei1	Object	Narrow	r2	500.3188655	5	2	on_focus	1	Narrow on_focus
2023410	block1	Control	post	jyun2	Subject	Broad	r2	775.1118786	1	1	broad_focus	2	Broad focus
2023410	block1	Control	post	jyun2	Subject	Broad	r2	189.6868684	2	2	broad_focus	2	Broad focus
2023410	block1	Control	post	mo2	Verb	Broad	r2	389.6469718	3	1	broad_focus	2	Broad focus
2023410	block1	Control	post	gau2	Object	Broad	r2	204.1748727	4	1	broad_focus	2	Broad focus
2023410	block1	Control	post	zai2	Object	Broad	r2	344.6955467	5	2	broad_focus	2	Broad focus
2023410	block1	Control	post	zoeng1	Subject	Narrow	r2	232.4162404	1	1	pre_focus	1	Narrow pre_focus
2023410	block1	Control	post	saang1	Subject	Narrow	r2	201.1055311	2	2	pre_focus	1	Narrow pre_focus
2023410	block1	Control	post	tsa1	Verb	Narrow	r2	161.4707674	3	1	on_focus	1	Narrow on_focus
2023410	block1	Control	post	fei1	Object	Narrow	r2	144.9328555	4	1	post_focus	1	Narrow post_focus
2023410	block1	Control	post	gei1	Object	Narrow	r2	334.7190194	5	2	post_focus	1	Narrow post_focus
2023410	block1	Control	pre	sau3	Subject	Contrastive	r1	133.5847606	1	1	pre_focus	3	Contrastive pre_focus
2023410	block1	Control	pre	sau3	Subject	Contrastive	r1	113.7071541	2	2	pre_focus	3	Contrastive pre_focus
2023410	block1	Control	pre	sik3	Verb	Contrastive	r1	91.43156412	3	1	on_focus	3	Contrastive on_focus
2023410	block1	Control	pre	baak3	Object	Contrastive	r1	129.5590829	4	1	post_focus	3	Contrastive post_focus
2023410	block1	Control	pre	baak3	Object	Contrastive	r1	206.0700328	5	2	post_focus	3	Contrastive post_focus
2023410	block1	Control	pre	sau3	Subject	Broad	r1	117.573622	1	1	broad_focus	3	Broad focus
2023410	block1	Control	pre	sau3	Subject	Broad	r1	91.01549509	2	2	broad_focus	3	Broad focus
2023410	block1	Control	pre	sik3	Verb	Broad	r1	77.84933974	3	1	broad_focus	3	Broad focus
2023410	block1	Control	pre	baak3	Object	Broad	r1	128.1559364	4	1	broad_focus	3	Broad focus
2023410	block1	Control	pre	baak3	Object	Broad	r1	217.5471353	5	2	broad_focus	3	Broad focus
2023410	block1	Control	pre	zoeng1	Subject	Narrow	r1	143.8971832	1	1	pre_focus	1	Narrow pre_focus
2023410	block1	Control	pre	saang1	Subject	Narrow	r1	174.4698864	2	2	pre_focus	1	Narrow pre_focus
2023410	block1	Control	pre	tsa1	Verb	Narrow	r1	113.9953659	3	1	pre_focus	1	Narrow pre_focus
2023410	block1	Control	pre	fei1	Object	Narrow	r1	118.3028997	4	1	on_focus	1	Narrow on_focus
2023410	block1	Control	pre	gei1	Object	Narrow	r1	319.0865281	5	2	on_focus	1	Narrow on_focus
2023410	block1	Control	pre	jyun2	Subject	Narrow	r1	204.682268	1	1	pre_focus	2	Narrow pre_focus
2023410	block1	Control	pre	jyun2	Subject	Narrow	r1	164.4668826	2	2	pre_focus	2	Narrow pre_focus
2023410	block1	Control	pre	mo2	Verb	Narrow	r1	148.7949235	3	1	pre_focus	2	Narrow pre_focus
2023410	block1	Control	pre	gau2	Object	Narrow	r1	145.4682814	4	1	on_focus	2	Narrow on_focus
2023410	block1	Control	pre	zai2	Object	Narrow	r1	302.1330186	5	2	on_focus	2	Narrow on_focus
2023410	block1	Control	pre	zoeng1	Subject	Narrow	r1	144.4099826	1	1	on_focus	1	Narrow on_focus
2023410	block1	Control	pre	saang1	Subject	Narrow	r1	185.5589602	2	2	on_focus	1	Narrow on_focus
2023410	block1	Control	pre	tsa1	Verb	Narrow	r1	109.3065566	3	1	post_focus	1	Narrow post_focus
2023410	block1	Control	pre	fei1	Object	Narrow	r1	147.9438397	4	1	post_focus	1	Narrow post_focus
2023410	block1	Control	pre	gei1	Object	Narrow	r1	380.9637961	5	2	post_focus	1	Narrow post_focus
2023410	block1	Control	pre	jyun2	Subject	Contrastive	r1	210.7112623	1	1	pre_focus	2	Contrastive pre_focus
2023410	block1	Control	pre	jyun2	Subject	Contrastive	r1	169.4991938	2	2	pre_focus	2	Contrastive pre_focus
2023410	block1	Control	pre	mo2	Verb	Contrastive	r1	141.3825944	3	1	on_focus	2	Contrastive on_focus
2023410	block1	Control	pre	gau2	Object	Contrastive	r1	81.26669186	4	1	post_focus	2	Contrastive post_focus
2023410	block1	Control	pre	zai2	Object	Contrastive	r1	306.3860831	5	2	post_focus	2	Contrastive post_focus
2023410	block1	Control	pre	zoeng1	Subject	Broad	r1	160.7198786	1	1	broad_focus	1	Broad focus
2023410	block1	Control	pre	saang1	Subject	Broad	r1	173.5503618	2	2	broad_focus	1	Broad focus
2023410	block1	Control	pre	tsa1	Verb	Broad	r1	120.1015211	3	1	broad_focus	1	Broad focus
2023410	block1	Control	pre	fei1	Object	Broad	r1	106.1544085	4	1	broad_focus	1	Broad focus
2023410	block1	Control	pre	gei1	Object	Broad	r1	275.3829945	5	2	broad_focus	1	Broad focus
2023410	block1	Control	pre	zoeng1	Subject	Narrow	r1	152.1461495	1	1	pre_focus	1	Narrow pre_focus
2023410	block1	Control	pre	saang1	Subject	Narrow	r1	185.4968096	2	2	pre_focus	1	Narrow pre_focus
2023410	block1	Control	pre	tsa1	Verb	Narrow	r1	112.5797615	3	1	on_focus	1	Narrow on_focus
2023410	block1	Control	pre	fei1	Object	Narrow	r1	94.65996893	4	1	post_focus	1	Narrow post_focus
2023410	block1	Control	pre	gei1	Object	Narrow	r1	221.3286317	5	2	post_focus	1	Narrow post_focus
2023410	block1	Control	pre	sau3	Subject	Narrow	r1	125.4883479	1	1	pre_focus	3	Narrow pre_focus
2023410	block1	Control	pre	sau3	Subject	Narrow	r1	121.5858754	2	2	pre_focus	3	Narrow pre_focus
2023410	block1	Control	pre	sik3	Verb	Narrow	r1	111.5912339	3	1	pre_focus	3	Narrow pre_focus
2023410	block1	Control	pre	baak3	Object	Narrow	r1	152.4638633	4	1	on_focus	3	Narrow on_focus

**2023410**	block1	Control	pre	baak3	Object	Narrow	r1	185.1412933	5	2	on_focus	3	Narrow on_focus
**2023410**	block1	Control	pre	jyun2	Subject	Narrow	r1	196.3005317	1	1	pre_focus	2	Narrow pre_focus
**2023410**	block1	Control	pre	jyun2	Subject	Narrow	r1	189.3413152	2	2	pre_focus	2	Narrow pre_focus
**2023410**	block1	Control	pre	mo2	Verb	Narrow	r1	151.5804374	3	1	on_focus	2	Narrow on_focus
**2023410**	block1	Control	pre	gau2	Object	Narrow	r1	171.3643505	4	1	post_focus	2	Narrow post_focus
**2023410**	block1	Control	pre	zai2	Object	Narrow	r1	331.0748245	5	2	post_focus	2	Narrow post_focus
**2023410**	block1	Control	pre	sau3	Subject	Contrastive	r1	96.17652632	1	1	on_focus	3	Contrastive on_focus
**2023410**	block1	Control	pre	sau3	Subject	Contrastive	r1	127.2536385	2	2	on_focus	3	Contrastive on_focus
**2023410**	block1	Control	pre	sik3	Verb	Contrastive	r1	83.57496205	3	1	post_focus	3	Contrastive post_focus
**2023410**	block1	Control	pre	baak3	Object	Contrastive	r1	103.1062251	4	1	post_focus	3	Contrastive post_focus
**2023410**	block1	Control	pre	baak3	Object	Contrastive	r1	160.9836073	5	2	post_focus	3	Contrastive post_focus
**2023410**	block1	Control	pre	sau3	Subject	Narrow	r1	48.2757489	1	1	on_focus	3	Narrow on_focus
**2023410**	block1	Control	pre	sau3	Subject	Narrow	r1	79.1337545	2	2	on_focus	3	Narrow on_focus
**2023410**	block1	Control	pre	sik3	Verb	Narrow	r1	60.62460683	3	1	post_focus	3	Narrow post_focus
**2023410**	block1	Control	pre	baak3	Object	Narrow	r1	97.54988461	4	1	post_focus	3	Narrow post_focus
**2023410**	block1	Control	pre	baak3	Object	Narrow	r1	121.96472	5	2	post_focus	3	Narrow post_focus
**2023410**	block1	Control	pre	zoeng1	Subject	Contrastive	r1	144.3547052	1	1	on_focus	1	Contrastive on_focus
**2023410**	block1	Control	pre	saang1	Subject	Contrastive	r1	120.3312688	2	2	on_focus	1	Contrastive on_focus
**2023410**	block1	Control	pre	tsa1	Verb	Contrastive	r1	99.29937186	3	1	post_focus	1	Contrastive post_focus
**2023410**	block1	Control	pre	fei1	Object	Contrastive	r1	93.78769198	4	1	post_focus	1	Contrastive post_focus
**2023410**	block1	Control	pre	gei1	Object	Contrastive	r1	272.8127459	5	2	post_focus	1	Contrastive post_focus
**2023410**	block1	Control	pre	sau3	Subject	Narrow	r1	97.62341517	1	1	pre_focus	3	Narrow pre_focus
**2023410**	block1	Control	pre	sau3	Subject	Narrow	r1	82.33877372	2	2	pre_focus	3	Narrow pre_focus
**2023410**	block1	Control	pre	sik3	Verb	Narrow	r1	79.96882086	3	1	on_focus	3	Narrow on_focus
**2023410**	block1	Control	pre	baak3	Object	Narrow	r1	101.1164893	4	1	post_focus	3	Narrow post_focus
**2023410**	block1	Control	pre	baak3	Object	Narrow	r1	133.6792598	5	2	post_focus	3	Narrow post_focus
**2023410**	block1	Control	pre	jyun2	Subject	Contrastive	r1	194.1978458	1	1	pre_focus	2	Contrastive pre_focus
**2023410**	block1	Control	pre	jyun2	Subject	Contrastive	r1	189.0497296	2	2	pre_focus	2	Contrastive pre_focus
**2023410**	block1	Control	pre	mo2	Verb	Contrastive	r1	208.3878097	3	1	pre_focus	2	Contrastive pre_focus
**2023410**	block1	Control	pre	gau2	Object	Contrastive	r1	178.7738659	4	1	on_focus	2	Contrastive on_focus
**2023410**	block1	Control	pre	zai2	Object	Contrastive	r1	272.0758928	5	2	on_focus	2	Contrastive on_focus
**2023410**	block1	Control	pre	zoeng1	Subject	Contrastive	r1	194.8899187	1	1	pre_focus	1	Contrastive pre_focus
**2023410**	block1	Control	pre	saang1	Subject	Contrastive	r1	150.8199746	2	2	pre_focus	1	Contrastive pre_focus
**2023410**	block1	Control	pre	tsa1	Verb	Contrastive	r1	125.6179201	3	1	on_focus	1	Contrastive on_focus
**2023410**	block1	Control	pre	fei1	Object	Contrastive	r1	88.75429742	4	1	post_focus	1	Contrastive post_focus
**2023410**	block1	Control	pre	gei1	Object	Contrastive	r1	334.853546	5	2	post_focus	1	Contrastive post_focus
**2023410**	block1	Control	pre	jyun2	Subject	Narrow	r1	224.2107623	1	1	on_focus	2	Narrow on_focus
**2023410**	block1	Control	pre	jyun2	Subject	Narrow	r1	186.78641	2	2	on_focus	2	Narrow on_focus
**2023410**	block1	Control	pre	mo2	Verb	Narrow	r1	160.5324105	3	1	post_focus	2	Narrow post_focus
**2023410**	block1	Control	pre	gau2	Object	Narrow	r1	138.2649462	4	1	post_focus	2	Narrow post_focus
**2023410**	block1	Control	pre	zai2	Object	Narrow	r1	290.8842854	5	2	post_focus	2	Narrow post_focus
**2023410**	block1	Control	pre	jyun2	Subject	Broad	r1	259.4676699	1	1	broad_focus	2	Broad focus
**2023410**	block1	Control	pre	jyun2	Subject	Broad	r1	178.6913315	2	2	broad_focus	2	Broad focus
**2023410**	block1	Control	pre	mo2	Verb	Broad	r1	200.4648698	3	1	broad_focus	2	Broad focus
**2023410**	block1	Control	pre	gau2	Object	Broad	r1	158.0782467	4	1	broad_focus	2	Broad focus
**2023410**	block1	Control	pre	zai2	Object	Broad	r1	266.55208	5	2	broad_focus	2	Broad focus
**2023410**	block1	Control	pre	zoeng1	Subject	Contrastive	r1	309.7293754	1	1	pre_focus	1	Contrastive pre_focus
**2023410**	block1	Control	pre	saang1	Subject	Contrastive	r1	270.7112011	2	2	pre_focus	1	Contrastive pre_focus
**2023410**	block1	Control	pre	tsa1	Verb	Contrastive	r1	197.4083485	3	1	pre_focus	1	Contrastive pre_focus
**2023410**	block1	Control	pre	fei1	Object	Contrastive	r1	223.6687066	4	1	on_focus	1	Contrastive on_focus
**2023410**	block1	Control	pre	gei1	Object	Contrastive	r1	292.4277211	5	2	on_focus	1	Contrastive on_focus
**2023410**	block1	Control	pre	jyun2	Subject	Contrastive	r1	276.4259121	1	1	on_focus	2	Contrastive on_focus
**2023410**	block1	Control	pre	jyun2	Subject	Contrastive	r1	188.7679743	2	2	on_focus	2	Contrastive on_focus
**2023410**	block1	Control	pre	mo2	Verb	Contrastive	r1	208.0081463	3	1	post_focus	2	Contrastive post_focus
**2023410**	block1	Control	pre	gau2	Object	Contrastive	r1	188.760478	4	1	post_focus	2	Contrastive post_focus
**2023410**	block1	Control	pre	zai2	Object	Contrastive	r1	314.3594383	5	2	post_focus	2	Contrastive post_focus
**2023410**	block1	Control	pre	sau3	Subject	Contrastive	r1	126.6591235	1	1	pre_focus	3	Contrastive pre_focus
**2023410**	block1	Control	pre	sau3	Subject	Contrastive	r1	85.50629418	2	2	pre_focus	3	Contrastive pre_focus
**2023410**	block1	Control	pre	sik3	Verb	Contrastive	r1	63.86743735	3	1	pre_focus	3	Contrastive pre_focus
**2023410**	block1	Control	pre	baak3	Object	Contrastive	r1	160.817214	4	1	on_focus	3	Contrastive on_focus
**2023410**	block1	Control	pre	baak3	Object	Contrastive	r1	169.5022676	5	2	on_focus	3	Contrastive on_focus
**2023410**	block1	Control	pre	sau3	Subject	Contrastive	r2	103.6284793	1	1	pre_focus	3	Contrastive pre_focus
**2023410**	block1	Control	pre	sau3	Subject	Contrastive	r2	111.3842278	2	2	pre_focus	3	Contrastive pre_focus
**2023410**	block1	Control	pre	sik3	Verb	Contrastive	r2	81.84506239	3	1	on_focus	3	Contrastive on_focus
**2023410**	block1	Control	pre	baak3	Object	Contrastive	r2	102.8349619	4	1	post_focus	3	Contrastive post_focus
**2023410**	block1	Control	pre	baak3	Object	Contrastive	r2	145.9275172	5	2	post_focus	3	Contrastive post_focus

**2023410**	block1	Control	pre	sau3	Subject	Narrow	r2	126.6581874	1	1	on_focus	3	Narrow on_focus
**2023410**	block1	Control	pre	sau3	Subject	Narrow	r2	114.5929449	2	2	on_focus	3	Narrow on_focus
**2023410**	block1	Control	pre	sik3	Verb	Narrow	r2	110.9365017	3	1	post_focus	3	Narrow post_focus
**2023410**	block1	Control	pre	baak3	Object	Narrow	r2	98.27929033	4	1	post_focus	3	Narrow post_focus
**2023410**	block1	Control	pre	baak3	Object	Narrow	r2	106.3064413	5	2	post_focus	3	Narrow post_focus
**2023410**	block1	Control	pre	zoeng1	Subject	Contrastive	r2	154.8381777	1	1	pre_focus	1	Contrastive pre_focus
**2023410**	block1	Control	pre	saang1	Subject	Contrastive	r2	130.215931	2	2	pre_focus	1	Contrastive pre_focus
**2023410**	block1	Control	pre	tsa1	Verb	Contrastive	r2	129.3952507	3	1	on_focus	1	Contrastive on_focus
**2023410**	block1	Control	pre	fei1	Object	Contrastive	r2	143.8892839	4	1	post_focus	1	Contrastive post_focus
**2023410**	block1	Control	pre	gei1	Object	Contrastive	r2	291.4521831	5	2	post_focus	1	Contrastive post_focus
**2023410**	block1	Control	pre	jyun2	Subject	Narrow	r2	195.0526494	1	1	on_focus	2	Narrow on_focus
**2023410**	block1	Control	pre	jyun2	Subject	Narrow	r2	170.6677526	2	2	on_focus	2	Narrow on_focus
**2023410**	block1	Control	pre	mo2	Verb	Narrow	r2	146.7497326	3	1	post_focus	2	Narrow post_focus
**2023410**	block1	Control	pre	gau2	Object	Narrow	r2	154.3570191	4	1	post_focus	2	Narrow post_focus
**2023410**	block1	Control	pre	zai2	Object	Narrow	r2	332.5735278	5	2	post_focus	2	Narrow post_focus
**2023410**	block1	Control	pre	zoeng1	Subject	Contrastive	r2	158.3006451	1	1	pre_focus	1	Contrastive pre_focus
**2023410**	block1	Control	pre	saang1	Subject	Contrastive	r2	214.7299151	2	2	pre_focus	1	Contrastive pre_focus
**2023410**	block1	Control	pre	tsa1	Verb	Contrastive	r2	124.0414654	3	1	pre_focus	1	Contrastive pre_focus
**2023410**	block1	Control	pre	fei1	Object	Contrastive	r2	126.5333768	4	1	on_focus	1	Contrastive on_focus
**2023410**	block1	Control	pre	gei1	Object	Contrastive	r2	305.390325	5	2	on_focus	1	Contrastive on_focus
**2023410**	block1	Control	pre	sau3	Subject	Narrow	r2	138.4326447	1	1	pre_focus	3	Narrow pre_focus
**2023410**	block1	Control	pre	sau3	Subject	Narrow	r2	91.71765844	2	2	pre_focus	3	Narrow pre_focus
**2023410**	block1	Control	pre	sik3	Verb	Narrow	r2	84.93377684	3	1	pre_focus	3	Narrow pre_focus
**2023410**	block1	Control	pre	baak3	Object	Narrow	r2	105.9012585	4	1	on_focus	3	Narrow on_focus
**2023410**	block1	Control	pre	baak3	Object	Narrow	r2	194.5640811	5	2	on_focus	3	Narrow on_focus
**2023410**	block1	Control	pre	jyun2	Subject	Contrastive	r2	213.5746128	1	1	pre_focus	2	Contrastive pre_focus
**2023410**	block1	Control	pre	jyun2	Subject	Contrastive	r2	180.9237843	2	2	pre_focus	2	Contrastive pre_focus
**2023410**	block1	Control	pre	mo2	Verb	Contrastive	r2	153.4692618	3	1	on_focus	2	Contrastive on_focus
**2023410**	block1	Control	pre	gau2	Object	Contrastive	r2	145.5674931	4	1	post_focus	2	Contrastive post_focus
**2023410**	block1	Control	pre	zai2	Object	Contrastive	r2	334.2089897	5	2	post_focus	2	Contrastive post_focus
**2023410**	block1	Control	pre	zoeng1	Subject	Broad	r2	157.5055931	1	1	broad_focus	1	Broad focus
**2023410**	block1	Control	pre	saang1	Subject	Broad	r2	158.0192811	2	2	broad_focus	1	Broad focus
**2023410**	block1	Control	pre	tsa1	Verb	Broad	r2	115.7048465	3	1	broad_focus	1	Broad focus
**2023410**	block1	Control	pre	fei1	Object	Broad	r2	114.4587599	4	1	broad_focus	1	Broad focus
**2023410**	block1	Control	pre	gei1	Object	Broad	r2	308.4140727	5	2	broad_focus	1	Broad focus
**2023410**	block1	Control	pre	zoeng1	Subject	Contrastive	r2	108.1813444	1	1	on_focus	1	Contrastive on_focus
**2023410**	block1	Control	pre	saang1	Subject	Contrastive	r2	135.8420309	2	2	on_focus	1	Contrastive on_focus
**2023410**	block1	Control	pre	tsa1	Verb	Contrastive	r2	121.204896	3	1	post_focus	1	Contrastive post_focus
**2023410**	block1	Control	pre	fei1	Object	Contrastive	r2	133.6024759	4	1	post_focus	1	Contrastive post_focus
**2023410**	block1	Control	pre	gei1	Object	Contrastive	r2	246.5086753	5	2	post_focus	1	Contrastive post_focus
**2023410**	block1	Control	pre	zoeng1	Subject	Narrow	r2	167.5798988	1	1	pre_focus	1	Narrow pre_focus
**2023410**	block1	Control	pre	saang1	Subject	Narrow	r2	164.0626048	2	2	pre_focus	1	Narrow pre_focus
**2023410**	block1	Control	pre	tsa1	Verb	Narrow	r2	129.5678608	3	1	on_focus	1	Narrow on_focus
**2023410**	block1	Control	pre	fei1	Object	Narrow	r2	83.33939373	4	1	post_focus	1	Narrow post_focus
**2023410**	block1	Control	pre	gei1	Object	Narrow	r2	270.933329	5	2	post_focus	1	Narrow post_focus
**2023410**	block1	Control	pre	sau3	Subject	Narrow	r2	124.1363921	1	1	pre_focus	3	Narrow pre_focus
**2023410**	block1	Control	pre	sau3	Subject	Narrow	r2	128.6805874	2	2	pre_focus	3	Narrow pre_focus
**2023410**	block1	Control	pre	sik3	Verb	Narrow	r2	111.1894587	3	1	on_focus	3	Narrow on_focus
**2023410**	block1	Control	pre	baak3	Object	Narrow	r2	90.3941598	4	1	post_focus	3	Narrow post_focus
**2023410**	block1	Control	pre	baak3	Object	Narrow	r2	167.3092087	5	2	post_focus	3	Narrow post_focus
**2023410**	block1	Control	pre	sau3	Subject	Contrastive	r2	99.71280432	1	1	on_focus	3	Contrastive on_focus
**2023410**	block1	Control	pre	sau3	Subject	Contrastive	r2	104.3306608	2	2	on_focus	3	Contrastive on_focus
**2023410**	block1	Control	pre	sik3	Verb	Contrastive	r2	79.91587755	3	1	post_focus	3	Contrastive post_focus
**2023410**	block1	Control	pre	baak3	Object	Contrastive	r2	108.9586653	4	1	post_focus	3	Contrastive post_focus
**2023410**	block1	Control	pre	baak3	Object	Contrastive	r2	201.8539397	5	2	post_focus	3	Contrastive post_focus
**2023410**	block1	Control	pre	jyun2	Subject	Contrastive	r2	228.8789548	1	1	pre_focus	2	Contrastive pre_focus
**2023410**	block1	Control	pre	jyun2	Subject	Contrastive	r2	235.1108293	2	2	pre_focus	2	Contrastive pre_focus
**2023410**	block1	Control	pre	mo2	Verb	Contrastive	r2	216.2143615	3	1	pre_focus	2	Contrastive pre_focus
**2023410**	block1	Control	pre	gau2	Object	Contrastive	r2	148.0863298	4	1	on_focus	2	Contrastive on_focus
**2023410**	block1	Control	pre	zai2	Object	Contrastive	r2	308.1925578	5	2	on_focus	2	Contrastive on_focus
**2023410**	block1	Control	pre	sau3	Subject	Contrastive	r2	85.0663915	1	1	pre_focus	3	Contrastive pre_focus
**2023410**	block1	Control	pre	sau3	Subject	Contrastive	r2	123.3002755	2	2	pre_focus	3	Contrastive pre_focus
**2023410**	block1	Control	pre	sik3	Verb	Contrastive	r2	122.1869609	3	1	pre_focus	3	Contrastive pre_focus
**2023410**	block1	Control	pre	baak3	Object	Contrastive	r2	136.6919664	4	1	on_focus	3	Contrastive on_focus
**2023410**	block1	Control	pre	baak3	Object	Contrastive	r2	157.7269519	5	2	on_focus	3	Contrastive on_focus
**2023410**	block1	Control	pre	sau3	Subject	Broad	r2	130.1679783	1	1	broad_focus	3	Broad focus

2023410	block1	Control	pre	sau3	Subject	Broad	r2	117.3242794	2	2	broad_focus	3	Broad focus
2023410	block1	Control	pre	sik3	Verb	Broad	r2	81.91814071	3	1	broad_focus	3	Broad focus
2023410	block1	Control	pre	baak3	Object	Broad	r2	100.7872947	4	1	broad_focus	3	Broad focus
2023410	block1	Control	pre	baak3	Object	Broad	r2	182.624428	5	2	broad_focus	3	Broad focus
2023410	block1	Control	pre	zoeng1	Subject	Narrow	r2	153.316616	1	1	pre_focus	1	Narrow pre_focus
2023410	block1	Control	pre	saang1	Subject	Narrow	r2	159.7461724	2	2	pre_focus	1	Narrow pre_focus
2023410	block1	Control	pre	tsa1	Verb	Narrow	r2	100.623386	3	1	pre_focus	1	Narrow pre_focus
2023410	block1	Control	pre	fei1	Object	Narrow	r2	134.1229084	4	1	on_focus	1	Narrow on_focus
2023410	block1	Control	pre	gei1	Object	Narrow	r2	272.4402294	5	2	on_focus	1	Narrow on_focus
2023410	block1	Control	pre	jyun2	Subject	Broad	r2	304.1603898	1	1	broad_focus	2	Broad focus
2023410	block1	Control	pre	jyun2	Subject	Broad	r2	284.9429676	2	2	broad_focus	2	Broad focus
2023410	block1	Control	pre	mo2	Verb	Broad	r2	265.5687557	3	1	broad_focus	2	Broad focus
2023410	block1	Control	pre	gau2	Object	Broad	r2	196.8963537	4	1	broad_focus	2	Broad focus
2023410	block1	Control	pre	zai2	Object	Broad	r2	316.5916616	5	2	broad_focus	2	Broad focus
2023410	block1	Control	pre	jyun2	Subject	Contrastive	r2	144.6438357	1	1	on_focus	2	Contrastive on_focus
2023410	block1	Control	pre	jyun2	Subject	Contrastive	r2	225.9821426	2	2	on_focus	2	Contrastive on_focus
2023410	block1	Control	pre	mo2	Verb	Contrastive	r2	181.2826679	3	1	post_focus	2	Contrastive post_focus
2023410	block1	Control	pre	gau2	Object	Contrastive	r2	150.8563726	4	1	post_focus	2	Contrastive post_focus
2023410	block1	Control	pre	zai2	Object	Contrastive	r2	312.2600151	5	2	post_focus	2	Contrastive post_focus
2023410	block1	Control	pre	jyun2	Subject	Narrow	r2	210.6815499	1	1	pre_focus	2	Narrow pre_focus
2023410	block1	Control	pre	jyun2	Subject	Narrow	r2	175.656185	2	2	pre_focus	2	Narrow pre_focus
2023410	block1	Control	pre	mo2	Verb	Narrow	r2	160.4674773	3	1	pre_focus	2	Narrow pre_focus
2023410	block1	Control	pre	gau2	Object	Narrow	r2	141.5875808	4	1	on_focus	2	Narrow on_focus
2023410	block1	Control	pre	zai2	Object	Narrow	r2	319.4789067	5	2	on_focus	2	Narrow on_focus
2023410	block1	Control	pre	zoeng1	Subject	Narrow	r2	159.9420046	1	1	on_focus	1	Narrow on_focus
2023410	block1	Control	pre	saang1	Subject	Narrow	r2	155.5793096	2	2	on_focus	1	Narrow on_focus
2023410	block1	Control	pre	tsa1	Verb	Narrow	r2	117.3711076	3	1	post_focus	1	Narrow post_focus
2023410	block1	Control	pre	fei1	Object	Narrow	r2	131.7140186	4	1	post_focus	1	Narrow post_focus
2023410	block1	Control	pre	gei1	Object	Narrow	r2	363.7474864	5	2	post_focus	1	Narrow post_focus
2023410	block1	Control	pre	jyun2	Subject	Narrow	r2	202.8551738	1	1	pre_focus	2	Narrow pre_focus
2023410	block1	Control	pre	jyun2	Subject	Narrow	r2	202.6249107	2	2	pre_focus	2	Narrow pre_focus
2023410	block1	Control	pre	mo2	Verb	Narrow	r2	161.6996733	3	1	on_focus	2	Narrow on_focus
2023410	block1	Control	pre	gau2	Object	Narrow	r2	146.6963337	4	1	post_focus	2	Narrow post_focus
2023410	block1	Control	pre	zai2	Object	Narrow	r2	225.4811342	5	2	post_focus	2	Narrow post_focus
2023410	block2	Control	post	lok6	Subject	Contrastive	r1	130.0189006	1	1	on_focus	6	Contrastive on_focus
2023410	block2	Control	post	lok6	Subject	Contrastive	r1	215.5906372	2	2	on_focus	6	Contrastive on_focus
2023410	block2	Control	post	waa6	Verb	Contrastive	r1	134.3594605	3	1	post_focus	6	Contrastive post_focus
2023410	block2	Control	post	jyut6	Object	Contrastive	r1	60.25184405	4	1	post_focus	6	Contrastive post_focus
2023410	block2	Control	post	loeng6	Object	Contrastive	r1	472.7170458	5	2	post_focus	6	Contrastive post_focus
2023410	block2	Control	post	ngaa5	Subject	Narrow	r1	206.0746431	1	1	pre_focus	5	Narrow pre_focus
2023410	block2	Control	post	ngaa5	Subject	Narrow	r1	196.1295817	2	2	pre_focus	5	Narrow pre_focus
2023410	block2	Control	post	maai5	Verb	Narrow	r1	189.9051342	3	1	pre_focus	5	Narrow pre_focus
2023410	block2	Control	post	pou5	Object	Narrow	r1	126.5374398	4	1	on_focus	5	Narrow on_focus
2023410	block2	Control	post	pou5	Object	Narrow	r1	231.9664307	5	2	on_focus	5	Narrow on_focus
2023410	block2	Control	post	ngaa5	Subject	Contrastive	r1	167.4230032	1	1	on_focus	5	Contrastive on_focus
2023410	block2	Control	post	ngaa5	Subject	Contrastive	r1	209.2344119	2	2	on_focus	5	Contrastive on_focus
2023410	block2	Control	post	maai5	Verb	Contrastive	r1	292.9338578	3	1	post_focus	5	Contrastive post_focus
2023410	block2	Control	post	pou5	Object	Contrastive	r1	78.2479468	4	1	post_focus	5	Contrastive post_focus
2023410	block2	Control	post	pou5	Object	Contrastive	r1	265.5918291	5	2	post_focus	5	Contrastive post_focus
2023410	block2	Control	post	ma4	Subject	Contrastive	r1	118.3322937	1	1	pre_focus	4	Contrastive pre_focus
2023410	block2	Control	post	ma4	Subject	Contrastive	r1	203.1864951	2	2	pre_focus	4	Contrastive pre_focus
2023410	block2	Control	post	fu4	Verb	Contrastive	r1	133.5398035	3	1	on_focus	4	Contrastive on_focus
2023410	block2	Control	post	maang4	Object	Contrastive	r1	332.4151393	4	1	post_focus	4	Contrastive post_focus
2023410	block2	Control	post	Jan-04	Object	Contrastive	r1	263.215836	5	2	post_focus	4	Contrastive post_focus
2023410	block2	Control	post	lok6	Subject	Contrastive	r1	122.8927987	1	1	pre_focus	6	Contrastive pre_focus
2023410	block2	Control	post	lok6	Subject	Contrastive	r1	149.1940759	2	2	pre_focus	6	Contrastive pre_focus
2023410	block2	Control	post	waa6	Verb	Contrastive	r1	114.6926385	3	1	on_focus	6	Contrastive on_focus
2023410	block2	Control	post	jyut6	Object	Contrastive	r1	67.13779454	4	1	post_focus	6	Contrastive post_focus
2023410	block2	Control	post	loeng6	Object	Contrastive	r1	451.8858244	5	2	post_focus	6	Contrastive post_focus
2023410	block2	Control	post	ngaa5	Subject	Narrow	r1	151.4118292	1	1	on_focus	5	Narrow on_focus
2023410	block2	Control	post	ngaa5	Subject	Narrow	r1	213.071382	2	2	on_focus	5	Narrow on_focus
2023410	block2	Control	post	maai5	Verb	Narrow	r1	209.4849039	3	1	post_focus	5	Narrow post_focus
2023410	block2	Control	post	pou5	Object	Narrow	r1	151.6232484	4	1	post_focus	5	Narrow post_focus
2023410	block2	Control	post	pou5	Object	Narrow	r1	240.611267	5	2	post_focus	5	Narrow post_focus
2023410	block2	Control	post	lok6	Subject	Narrow	r1	141.0014514	1	1	pre_focus	6	Narrow pre_focus
2023410	block2	Control	post	lok6	Subject	Narrow	r1	188.1960452	2	2	pre_focus	6	Narrow pre_focus

**2023410**	block2	Control	post	waa6	Verb	Narrow	r1	112.2361099	3	1	on_focus	6	Narrow on_focus
**2023410**	block2	Control	post	jyut6	Object	Narrow	r1	67.95118792	4	1	post_focus	6	Narrow post_focus
**2023410**	block2	Control	post	loeng6	Object	Narrow	r1	430.7725168	5	2	post_focus	6	Narrow post_focus
**2023410**	block2	Control	post	ngaa5	Subject	Narrow	r1	159.8734305	1	1	pre_focus	5	Narrow pre_focus
**2023410**	block2	Control	post	ngaa5	Subject	Narrow	r1	194.7830929	2	2	pre_focus	5	Narrow pre_focus
**2023410**	block2	Control	post	maai5	Verb	Narrow	r1	195.8654983	3	1	on_focus	5	Narrow on_focus
**2023410**	block2	Control	post	pou5	Object	Narrow	r1	112.100526	4	1	post_focus	5	Narrow post_focus
**2023410**	block2	Control	post	pou5	Object	Narrow	r1	306.6745288	5	2	post_focus	5	Narrow post_focus
**2023410**	block2	Control	post	ma4	Subject	Narrow	r1	150.4385081	1	1	pre_focus	4	Narrow pre_focus
**2023410**	block2	Control	post	ma4	Subject	Narrow	r1	194.4192933	2	2	pre_focus	4	Narrow pre_focus
**2023410**	block2	Control	post	fu4	Verb	Narrow	r1	117.3668379	3	1	on_focus	4	Narrow on_focus
**2023410**	block2	Control	post	maang4	Object	Narrow	r1	250.1755322	4	1	post_focus	4	Narrow post_focus
**2023410**	block2	Control	post	Jan-04	Object	Narrow	r1	241.2296249	5	2	post_focus	4	Narrow post_focus
**2023410**	block2	Control	post	ngaa5	Subject	Contrastive	r1	170.9104094	1	1	pre_focus	5	Contrastive pre_focus
**2023410**	block2	Control	post	ngaa5	Subject	Contrastive	r1	216.2470198	2	2	pre_focus	5	Contrastive pre_focus
**2023410**	block2	Control	post	maai5	Verb	Contrastive	r1	225.1677103	3	1	pre_focus	5	Contrastive pre_focus
**2023410**	block2	Control	post	pou5	Object	Contrastive	r1	129.7602079	4	1	on_focus	5	Contrastive on_focus
**2023410**	block2	Control	post	pou5	Object	Contrastive	r1	333.7138128	5	2	on_focus	5	Contrastive on_focus
**2023410**	block2	Control	post	ngaa5	Subject	Broad	r1	176.6105651	1	1	broad_focus	5	Broad focus
**2023410**	block2	Control	post	ngaa5	Subject	Broad	r1	222.2324908	2	2	broad_focus	5	Broad focus
**2023410**	block2	Control	post	maai5	Verb	Broad	r1	226.9121426	3	1	broad_focus	5	Broad focus
**2023410**	block2	Control	post	pou5	Object	Broad	r1	117.0428223	4	1	broad_focus	5	Broad focus
**2023410**	block2	Control	post	pou5	Object	Broad	r1	306.3255867	5	2	broad_focus	5	Broad focus
**2023410**	block2	Control	post	ngaa5	Subject	Contrastive	r1	358.1174984	1	1	pre_focus	5	Contrastive pre_focus
**2023410**	block2	Control	post	ngaa5	Subject	Contrastive	r1	338.4970118	2	2	pre_focus	5	Contrastive pre_focus
**2023410**	block2	Control	post	maai5	Verb	Contrastive	r1	258.6775297	3	1	on_focus	5	Contrastive on_focus
**2023410**	block2	Control	post	pou5	Object	Contrastive	r1	116.800534	4	1	post_focus	5	Contrastive post_focus
**2023410**	block2	Control	post	pou5	Object	Contrastive	r1	301.8584516	5	2	post_focus	5	Contrastive post_focus
**2023410**	block2	Control	post	ma4	Subject	Contrastive	r1	170.8572945	1	1	pre_focus	4	Contrastive pre_focus
**2023410**	block2	Control	post	ma4	Subject	Contrastive	r1	188.8162144	2	2	pre_focus	4	Contrastive pre_focus
**2023410**	block2	Control	post	fu4	Verb	Contrastive	r1	90.40224844	3	1	pre_focus	4	Contrastive pre_focus
**2023410**	block2	Control	post	maang4	Object	Contrastive	r1	282.1824716	4	1	on_focus	4	Contrastive on_focus
**2023410**	block2	Control	post	Jan-04	Object	Contrastive	r1	119.8980615	5	2	on_focus	4	Contrastive on_focus
**2023410**	block2	Control	post	lok6	Subject	Narrow	r1	150.0866831	1	1	on_focus	6	Narrow on_focus
**2023410**	block2	Control	post	lok6	Subject	Narrow	r1	192.9676493	2	2	on_focus	6	Narrow on_focus
**2023410**	block2	Control	post	waa6	Verb	Narrow	r1	110.3414713	3	1	post_focus	6	Narrow post_focus
**2023410**	block2	Control	post	jyut6	Object	Narrow	r1	59.21903173	4	1	post_focus	6	Narrow post_focus
**2023410**	block2	Control	post	loeng6	Object	Narrow	r1	444.3947648	5	2	post_focus	6	Narrow post_focus
**2023410**	block2	Control	post	ma4	Subject	Narrow	r1	168.9273719	1	1	pre_focus	4	Narrow pre_focus
**2023410**	block2	Control	post	ma4	Subject	Narrow	r1	162.5291954	2	2	pre_focus	4	Narrow pre_focus
**2023410**	block2	Control	post	fu4	Verb	Narrow	r1	115.0031914	3	1	pre_focus	4	Narrow pre_focus
**2023410**	block2	Control	post	maang4	Object	Narrow	r1	218.7735153	4	1	on_focus	4	Narrow on_focus
**2023410**	block2	Control	post	Jan-04	Object	Narrow	r1	230.8102329	5	2	on_focus	4	Narrow on_focus
**2023410**	block2	Control	post	lok6	Subject	Contrastive	r1	174.7172453	1	1	pre_focus	6	Contrastive pre_focus
**2023410**	block2	Control	post	lok6	Subject	Contrastive	r1	227.6231011	2	2	pre_focus	6	Contrastive pre_focus
**2023410**	block2	Control	post	waa6	Verb	Contrastive	r1	107.1329393	3	1	pre_focus	6	Contrastive pre_focus
**2023410**	block2	Control	post	jyut6	Object	Contrastive	r1	193.6428683	4	1	on_focus	6	Contrastive on_focus
**2023410**	block2	Control	post	loeng6	Object	Contrastive	r1	436.7268967	5	2	on_focus	6	Contrastive on_focus
**2023410**	block2	Control	post	ma4	Subject	Contrastive	r1	151.1307909	1	1	on_focus	4	Contrastive on_focus
**2023410**	block2	Control	post	ma4	Subject	Contrastive	r1	194.1995532	2	2	on_focus	4	Contrastive on_focus
**2023410**	block2	Control	post	fu4	Verb	Contrastive	r1	107.6615694	3	1	post_focus	4	Contrastive post_focus
**2023410**	block2	Control	post	maang4	Object	Contrastive	r1	285.1127617	4	1	post_focus	4	Contrastive post_focus
**2023410**	block2	Control	post	Jan-04	Object	Contrastive	r1	336.8380088	5	2	post_focus	4	Contrastive post_focus
**2023410**	block2	Control	post	lok6	Subject	Broad	r1	140.7480732	1	1	broad_focus	6	Broad focus
**2023410**	block2	Control	post	lok6	Subject	Broad	r1	178.1738218	2	2	broad_focus	6	Broad focus
**2023410**	block2	Control	post	waa6	Verb	Broad	r1	138.1061297	3	1	broad_focus	6	Broad focus
**2023410**	block2	Control	post	jyut6	Object	Broad	r1	79.72328019	4	1	broad_focus	6	Broad focus
**2023410**	block2	Control	post	loeng6	Object	Broad	r1	496.9881858	5	2	broad_focus	6	Broad focus
**2023410**	block2	Control	post	ma4	Subject	Narrow	r1	162.1376197	1	1	on_focus	4	Narrow on_focus
**2023410**	block2	Control	post	ma4	Subject	Narrow	r1	202.1655383	2	2	on_focus	4	Narrow on_focus
**2023410**	block2	Control	post	fu4	Verb	Narrow	r1	112.6918525	3	1	post_focus	4	Narrow post_focus
**2023410**	block2	Control	post	maang4	Object	Narrow	r1	258.0686552	4	1	post_focus	4	Narrow post_focus
**2023410**	block2	Control	post	Jan-04	Object	Narrow	r1	248.4206407	5	2	post_focus	4	Narrow post_focus
**2023410**	block2	Control	post	ma4	Subject	Broad	r1	148.3226513	1	1	broad_focus	4	Broad focus
**2023410**	block2	Control	post	ma4	Subject	Broad	r1	224.4620242	2	2	broad_focus	4	Broad focus
**2023410**	block2	Control	post	fu4	Verb	Broad	r1	97.96932122	3	1	broad_focus	4	Broad focus

2023410	block2	Control	post	maang4	Object	Broad	r1	309.4566245	4	1	broad_focus	4	Broad focus
2023410	block2	Control	post	Jan-04	Object	Broad	r1	247.2085849	5	2	broad_focus	4	Broad focus
2023410	block2	Control	post	lok6	Subject	Narrow	r1	127.8025053	1	1	pre_focus	6	Narrow pre_focus
2023410	block2	Control	post	lok6	Subject	Narrow	r1	200.8249277	2	2	pre_focus	6	Narrow pre_focus
2023410	block2	Control	post	waa6	Verb	Narrow	r1	182.5349023	3	1	pre_focus	6	Narrow pre_focus
2023410	block2	Control	post	jyut6	Object	Narrow	r1	76.41415327	4	1	on_focus	6	Narrow on_focus
2023410	block2	Control	post	loeng6	Object	Narrow	r1	507.6521501	5	2	on_focus	6	Narrow on_focus
2023410	block2	Control	post	ma4	Subject	Narrow	r2	190.0533351	1	1	pre_focus	4	Narrow pre_focus
2023410	block2	Control	post	ma4	Subject	Narrow	r2	235.3063863	2	2	pre_focus	4	Narrow pre_focus
2023410	block2	Control	post	fu4	Verb	Narrow	r2	136.7222524	3	1	pre_focus	4	Narrow pre_focus
2023410	block2	Control	post	maang4	Object	Narrow	r2	319.2147408	4	1	on_focus	4	Narrow on_focus
2023410	block2	Control	post	Jan-04	Object	Narrow	r2	316.8252244	5	2	on_focus	4	Narrow on_focus
2023410	block2	Control	post	lok6	Subject	Contrastive	r2	143.8471555	1	1	pre_focus	6	Contrastive pre_focus
2023410	block2	Control	post	lok6	Subject	Contrastive	r2	150.8554398	2	2	pre_focus	6	Contrastive pre_focus
2023410	block2	Control	post	waa6	Verb	Contrastive	r2	107.9493579	3	1	pre_focus	6	Contrastive pre_focus
2023410	block2	Control	post	jyut6	Object	Contrastive	r2	57.80069749	4	1	on_focus	6	Contrastive on_focus
2023410	block2	Control	post	loeng6	Object	Contrastive	r2	480.8782227	5	2	on_focus	6	Contrastive on_focus
2023410	block2	Control	post	ma4	Subject	Narrow	r2	210.2000046	1	1	on_focus	4	Narrow on_focus
2023410	block2	Control	post	ma4	Subject	Narrow	r2	200.826778	2	2	on_focus	4	Narrow on_focus
2023410	block2	Control	post	fu4	Verb	Narrow	r2	99.09680484	3	1	post_focus	4	Narrow post_focus
2023410	block2	Control	post	maang4	Object	Narrow	r2	313.0917392	4	1	post_focus	4	Narrow post_focus
2023410	block2	Control	post	Jan-04	Object	Narrow	r2	249.0072953	5	2	post_focus	4	Narrow post_focus
2023410	block2	Control	post	lok6	Subject	Contrastive	r2	151.8645962	1	1	on_focus	6	Contrastive on_focus
2023410	block2	Control	post	lok6	Subject	Contrastive	r2	153.3560102	2	2	on_focus	6	Contrastive on_focus
2023410	block2	Control	post	waa6	Verb	Contrastive	r2	116.5864254	3	1	post_focus	6	Contrastive post_focus
2023410	block2	Control	post	jyut6	Object	Contrastive	r2	77.82787172	4	1	post_focus	6	Contrastive post_focus
2023410	block2	Control	post	loeng6	Object	Contrastive	r2	481.4331235	5	2	post_focus	6	Contrastive post_focus
2023410	block2	Control	post	ma4	Subject	Contrastive	r2	207.2902008	1	1	pre_focus	4	Contrastive pre_focus
2023410	block2	Control	post	ma4	Subject	Contrastive	r2	190.894216	2	2	pre_focus	4	Contrastive pre_focus
2023410	block2	Control	post	fu4	Verb	Contrastive	r2	100.1721658	3	1	pre_focus	4	Contrastive pre_focus
2023410	block2	Control	post	maang4	Object	Contrastive	r2	373.8327855	4	1	on_focus	4	Contrastive on_focus
2023410	block2	Control	post	Jan-04	Object	Contrastive	r2	244.9971211	5	2	on_focus	4	Contrastive on_focus
2023410	block2	Control	post	ngaa5	Subject	Narrow	r2	161.0661508	1	1	on_focus	5	Narrow on_focus
2023410	block2	Control	post	ngaa5	Subject	Narrow	r2	216.441455	2	2	on_focus	5	Narrow on_focus
2023410	block2	Control	post	maai5	Verb	Narrow	r2	207.2427201	3	1	post_focus	5	Narrow post_focus
2023410	block2	Control	post	pou5	Object	Narrow	r2	128.1136533	4	1	post_focus	5	Narrow post_focus
2023410	block2	Control	post	pou5	Object	Narrow	r2	400.3661121	5	2	post_focus	5	Narrow post_focus
2023410	block2	Control	post	ngaa5	Subject	Narrow	r2	119.9658854	1	1	pre_focus	5	Narrow pre_focus
2023410	block2	Control	post	ngaa5	Subject	Narrow	r2	206.4378659	2	2	pre_focus	5	Narrow pre_focus
2023410	block2	Control	post	maai5	Verb	Narrow	r2	210.2193633	3	1	pre_focus	5	Narrow pre_focus
2023410	block2	Control	post	pou5	Object	Narrow	r2	140.9480831	4	1	on_focus	5	Narrow on_focus
2023410	block2	Control	post	pou5	Object	Narrow	r2	297.788511	5	2	on_focus	5	Narrow on_focus
2023410	block2	Control	post	ngaa5	Subject	Contrastive	r2	185.1292422	1	1	pre_focus	5	Contrastive pre_focus
2023410	block2	Control	post	ngaa5	Subject	Contrastive	r2	228.9343761	2	2	pre_focus	5	Contrastive pre_focus
2023410	block2	Control	post	maai5	Verb	Contrastive	r2	217.4055191	3	1	on_focus	5	Contrastive on_focus
2023410	block2	Control	post	pou5	Object	Contrastive	r2	139.6200918	4	1	post_focus	5	Contrastive post_focus
2023410	block2	Control	post	pou5	Object	Contrastive	r2	307.912203	5	2	post_focus	5	Contrastive post_focus
2023410	block2	Control	post	ngaa5	Subject	Narrow	r2	171.4469812	1	1	pre_focus	5	Narrow pre_focus
2023410	block2	Control	post	ngaa5	Subject	Narrow	r2	211.0371935	2	2	pre_focus	5	Narrow pre_focus
2023410	block2	Control	post	maai5	Verb	Narrow	r2	220.4925674	3	1	on_focus	5	Narrow on_focus
2023410	block2	Control	post	pou5	Object	Narrow	r2	131.2473815	4	1	post_focus	5	Narrow post_focus
2023410	block2	Control	post	pou5	Object	Narrow	r2	266.645269	5	2	post_focus	5	Narrow post_focus
2023410	block2	Control	post	lok6	Subject	Narrow	r2	137.2426172	1	1	pre_focus	6	Narrow pre_focus
2023410	block2	Control	post	lok6	Subject	Narrow	r2	122.8129199	2	2	pre_focus	6	Narrow pre_focus
2023410	block2	Control	post	waa6	Verb	Narrow	r2	109.0440561	3	1	on_focus	6	Narrow on_focus
2023410	block2	Control	post	jyut6	Object	Narrow	r2	77.5269446	4	1	post_focus	6	Narrow post_focus
2023410	block2	Control	post	loeng6	Object	Narrow	r2	395.8490061	5	2	post_focus	6	Narrow post_focus
2023410	block2	Control	post	lok6	Subject	Contrastive	r2	165.7675291	1	1	pre_focus	6	Contrastive pre_focus
2023410	block2	Control	post	lok6	Subject	Contrastive	r2	160.9999401	2	2	pre_focus	6	Contrastive pre_focus
2023410	block2	Control	post	waa6	Verb	Contrastive	r2	103.5788726	3	1	on_focus	6	Contrastive on_focus
2023410	block2	Control	post	jyut6	Object	Contrastive	r2	79.65428877	4	1	post_focus	6	Contrastive post_focus
2023410	block2	Control	post	loeng6	Object	Contrastive	r2	396.8915783	5	2	post_focus	6	Contrastive post_focus
2023410	block2	Control	post	ma4	Subject	Narrow	r2	174.3603684	1	1	pre_focus	4	Narrow pre_focus
2023410	block2	Control	post	ma4	Subject	Narrow	r2	195.3495141	2	2	pre_focus	4	Narrow pre_focus
2023410	block2	Control	post	fu4	Verb	Narrow	r2	77.14060022	3	1	on_focus	4	Narrow on_focus
2023410	block2	Control	post	maang4	Object	Narrow	r2	285.5881758	4	1	post_focus	4	Narrow post_focus

2023410	block2	Control	post	Jan-04	Object	Narrow	r2	275.1546674	5	2	post_focus	4	Narrow post_focus
2023410	block2	Control	post	ma4	Subject	Broad	r2	139.7035655	1	1	broad_focus	4	Broad focus
2023410	block2	Control	post	ma4	Subject	Broad	r2	220.0428541	2	2	broad_focus	4	Broad focus
2023410	block2	Control	post	fu4	Verb	Broad	r2	79.24950417	3	1	broad_focus	4	Broad focus
2023410	block2	Control	post	maang4	Object	Broad	r2	314.7505754	4	1	broad_focus	4	Broad focus
2023410	block2	Control	post	Jan-04	Object	Broad	r2	211.206047	5	2	broad_focus	4	Broad focus
2023410	block2	Control	post	ngaa5	Subject	Contrastive	r2	179.3547094	1	1	on_focus	5	Contrastive on_focus
2023410	block2	Control	post	ngaa5	Subject	Contrastive	r2	207.3158625	2	2	on_focus	5	Contrastive on_focus
2023410	block2	Control	post	maai5	Verb	Contrastive	r2	191.4725041	3	1	post_focus	5	Contrastive post_focus
2023410	block2	Control	post	pou5	Object	Contrastive	r2	164.3283867	4	1	post_focus	5	Contrastive post_focus
2023410	block2	Control	post	pou5	Object	Contrastive	r2	390.2793255	5	2	post_focus	5	Contrastive post_focus
2023410	block2	Control	post	ngaa5	Subject	Contrastive	r2	191.6325839	1	1	pre_focus	5	Contrastive pre_focus
2023410	block2	Control	post	ngaa5	Subject	Contrastive	r2	217.5537704	2	2	pre_focus	5	Contrastive pre_focus
2023410	block2	Control	post	maai5	Verb	Contrastive	r2	259.4254604	3	1	pre_focus	5	Contrastive pre_focus
2023410	block2	Control	post	pou5	Object	Contrastive	r2	159.6988258	4	1	on_focus	5	Contrastive on_focus
2023410	block2	Control	post	pou5	Object	Contrastive	r2	358.0144922	5	2	on_focus	5	Contrastive on_focus
2023410	block2	Control	post	ma4	Subject	Contrastive	r2	183.8511202	1	1	on_focus	4	Contrastive on_focus
2023410	block2	Control	post	ma4	Subject	Contrastive	r2	198.4939182	2	2	on_focus	4	Contrastive on_focus
2023410	block2	Control	post	fu4	Verb	Contrastive	r2	119.4212943	3	1	post_focus	4	Contrastive post_focus
2023410	block2	Control	post	maang4	Object	Contrastive	r2	287.3865499	4	1	post_focus	4	Contrastive post_focus
2023410	block2	Control	post	Jan-04	Object	Contrastive	r2	252.2398908	5	2	post_focus	4	Contrastive post_focus
2023410	block2	Control	post	ngaa5	Subject	Broad	r2	137.1546077	1	1	broad_focus	5	Broad focus
2023410	block2	Control	post	ngaa5	Subject	Broad	r2	247.649529	2	2	broad_focus	5	Broad focus
2023410	block2	Control	post	maai5	Verb	Broad	r2	232.1738693	3	1	broad_focus	5	Broad focus
2023410	block2	Control	post	pou5	Object	Broad	r2	133.4796291	4	1	broad_focus	5	Broad focus
2023410	block2	Control	post	pou5	Object	Broad	r2	305.2176408	5	2	broad_focus	5	Broad focus
2023410	block2	Control	post	lok6	Subject	Broad	r2	146.2074206	1	1	broad_focus	6	Broad focus
2023410	block2	Control	post	lok6	Subject	Broad	r2	175.5147467	2	2	broad_focus	6	Broad focus
2023410	block2	Control	post	waa6	Verb	Broad	r2	108.7394902	3	1	broad_focus	6	Broad focus
2023410	block2	Control	post	jyut6	Object	Broad	r2	88.87224782	4	1	broad_focus	6	Broad focus
2023410	block2	Control	post	loeng6	Object	Broad	r2	359.0434936	5	2	broad_focus	6	Broad focus
2023410	block2	Control	post	lok6	Subject	Narrow	r2	125.6647091	1	1	on_focus	6	Narrow on_focus
2023410	block2	Control	post	lok6	Subject	Narrow	r2	156.5702594	2	2	on_focus	6	Narrow on_focus
2023410	block2	Control	post	waa6	Verb	Narrow	r2	123.2845132	3	1	post_focus	6	Narrow post_focus
2023410	block2	Control	post	jyut6	Object	Narrow	r2	59.99059978	4	1	post_focus	6	Narrow post_focus
2023410	block2	Control	post	loeng6	Object	Narrow	r2	414.5878089	5	2	post_focus	6	Narrow post_focus
2023410	block2	Control	post	ma4	Subject	Contrastive	r2	153.3036111	1	1	pre_focus	4	Contrastive pre_focus
2023410	block2	Control	post	ma4	Subject	Contrastive	r2	206.3050158	2	2	pre_focus	4	Contrastive pre_focus
2023410	block2	Control	post	fu4	Verb	Contrastive	r2	90.07509695	3	1	on_focus	4	Contrastive on_focus
2023410	block2	Control	post	maang4	Object	Contrastive	r2	299.1217944	4	1	post_focus	4	Contrastive post_focus
2023410	block2	Control	post	Jan-04	Object	Contrastive	r2	215.4665389	5	2	post_focus	4	Contrastive post_focus
2023410	block2	Control	post	lok6	Subject	Narrow	r2	129.315239	1	1	pre_focus	6	Narrow pre_focus
2023410	block2	Control	post	lok6	Subject	Narrow	r2	128.8209246	2	2	pre_focus	6	Narrow pre_focus
2023410	block2	Control	post	waa6	Verb	Narrow	r2	119.7232923	3	1	pre_focus	6	Narrow pre_focus
2023410	block2	Control	post	jyut6	Object	Narrow	r2	77.51664257	4	1	on_focus	6	Narrow on_focus
2023410	block2	Control	post	loeng6	Object	Narrow	r2	440.1112485	5	2	on_focus	6	Narrow on_focus
2023410	block2	Control	pre	ma4	Subject	Contrastive	r1	181.7993197	1	1	pre_focus	4	Contrastive pre_focus
2023410	block2	Control	pre	ma4	Subject	Contrastive	r1	329.7316704	2	2	pre_focus	4	Contrastive pre_focus
2023410	block2	Control	pre	fu4	Verb	Contrastive	r1	184.1735954	3	1	on_focus	4	Contrastive on_focus
2023410	block2	Control	pre	maang4	Object	Contrastive	r1	328.2142857	4	1	post_focus	4	Contrastive post_focus
2023410	block2	Control	pre	Jan-04	Object	Contrastive	r1	269.1451247	5	2	post_focus	4	Contrastive post_focus
2023410	block2	Control	pre	lok6	Subject	Contrastive	r1	144.6503401	1	1	pre_focus	6	Contrastive pre_focus
2023410	block2	Control	pre	lok6	Subject	Contrastive	r1	138.066421	2	2	pre_focus	6	Contrastive pre_focus
2023410	block2	Control	pre	waa6	Verb	Contrastive	r1	92.50178167	3	1	pre_focus	6	Contrastive pre_focus
2023410	block2	Control	pre	jyut6	Object	Contrastive	r1	58.01245366	4	1	on_focus	6	Contrastive on_focus
2023410	block2	Control	pre	loeng6	Object	Contrastive	r1	480.4179894	5	2	on_focus	6	Contrastive on_focus
2023410	block2	Control	pre	ma4	Subject	Narrow	r1	177.5260771	1	1	pre_focus	4	Narrow pre_focus
2023410	block2	Control	pre	ma4	Subject	Narrow	r1	212.3097758	2	2	pre_focus	4	Narrow pre_focus
2023410	block2	Control	pre	fu4	Verb	Narrow	r1	119.8866213	3	1	pre_focus	4	Narrow pre_focus
2023410	block2	Control	pre	maang4	Object	Narrow	r1	308.8876291	4	1	on_focus	4	Narrow on_focus
2023410	block2	Control	pre	Jan-04	Object	Narrow	r1	223.590325	5	2	on_focus	4	Narrow on_focus
2023410	block2	Control	pre	ma4	Subject	Broad	r1	138.6408301	1	1	broad_focus	4	Broad focus
2023410	block2	Control	pre	ma4	Subject	Broad	r1	185.2789891	2	2	broad_focus	4	Broad focus
2023410	block2	Control	pre	fu4	Verb	Broad	r1	104.5975057	3	1	broad_focus	4	Broad focus
2023410	block2	Control	pre	maang4	Object	Broad	r1	315.7581255	4	1	broad_focus	4	Broad focus
2023410	block2	Control	pre	Jan-04	Object	Broad	r1	208.6895314	5	2	broad_focus	4	Broad focus

2023410	block2	Control	pre	ngaa5	Subject	Narrow	r1	165.2769997	1	1	pre_focus	5	Narrow pre_focus
2023410	block2	Control	pre	ngaa5	Subject	Narrow	r1	165.2904239	2	2	pre_focus	5	Narrow pre_focus
2023410	block2	Control	pre	maai5	Verb	Narrow	r1	278.5495087	3	1	on_focus	5	Narrow on_focus
2023410	block2	Control	pre	pou5	Object	Narrow	r1	109.4457042	4	1	post_focus	5	Narrow post_focus
2023410	block2	Control	pre	pou5	Object	Narrow	r1	343.4133283	5	2	post_focus	5	Narrow post_focus
2023410	block2	Control	pre	lok6	Subject	Contrastive	r1	127.0910269	1	1	pre_focus	6	Contrastive pre_focus
2023410	block2	Control	pre	lok6	Subject	Contrastive	r1	176.1500492	2	2	pre_focus	6	Contrastive pre_focus
2023410	block2	Control	pre	waa6	Verb	Contrastive	r1	106.9872191	3	1	on_focus	6	Contrastive on_focus
2023410	block2	Control	pre	jyut6	Object	Contrastive	r1	63.66591081	4	1	post_focus	6	Contrastive post_focus
2023410	block2	Control	pre	loeng6	Object	Contrastive	r1	470.4837491	5	2	post_focus	6	Contrastive post_focus
2023410	block2	Control	pre	ngaa5	Subject	Contrastive	r1	140.6708239	1	1	pre_focus	5	Contrastive pre_focus
2023410	block2	Control	pre	ngaa5	Subject	Contrastive	r1	237.390699	2	2	pre_focus	5	Contrastive pre_focus
2023410	block2	Control	pre	maai5	Verb	Contrastive	r1	219.1105651	3	1	pre_focus	5	Contrastive pre_focus
2023410	block2	Control	pre	pou5	Object	Contrastive	r1	116.9157758	4	1	on_focus	5	Contrastive on_focus
2023410	block2	Control	pre	pou5	Object	Contrastive	r1	267.2691934	5	2	on_focus	5	Contrastive on_focus
2023410	block2	Control	pre	lok6	Subject	Narrow	r1	136.4693583	1	1	pre_focus	6	Narrow pre_focus
2023410	block2	Control	pre	lok6	Subject	Narrow	r1	161.2601231	2	2	pre_focus	6	Narrow pre_focus
2023410	block2	Control	pre	waa6	Verb	Narrow	r1	112.5251053	3	1	pre_focus	6	Narrow pre_focus
2023410	block2	Control	pre	jyut6	Object	Narrow	r1	66.38586546	4	1	on_focus	6	Narrow on_focus
2023410	block2	Control	pre	loeng6	Object	Narrow	r1	413.9493575	5	2	on_focus	6	Narrow on_focus
2023410	block2	Control	pre	lok6	Subject	Broad	r1	97.58836868	1	1	broad_focus	6	Broad focus
2023410	block2	Control	pre	lok6	Subject	Broad	r1	170.1120829	2	2	broad_focus	6	Broad focus
2023410	block2	Control	pre	waa6	Verb	Broad	r1	97.99422799	3	1	broad_focus	6	Broad focus
2023410	block2	Control	pre	jyut6	Object	Broad	r1	47.62323391	4	1	broad_focus	6	Broad focus
2023410	block2	Control	pre	loeng6	Object	Broad	r1	364.3329554	5	2	broad_focus	6	Broad focus
2023410	block2	Control	pre	ngaa5	Subject	Contrastive	r1	126.388511	1	1	on_focus	5	Contrastive on_focus
2023410	block2	Control	pre	ngaa5	Subject	Contrastive	r1	270.7981859	2	2	on_focus	5	Contrastive on_focus
2023410	block2	Control	pre	maai5	Verb	Contrastive	r1	241.9965986	3	1	post_focus	5	Contrastive post_focus
2023410	block2	Control	pre	pou5	Object	Contrastive	r1	110.2097506	4	1	post_focus	5	Contrastive post_focus
2023410	block2	Control	pre	pou5	Object	Contrastive	r1	364.1586961	5	2	post_focus	5	Contrastive post_focus
2023410	block2	Control	pre	ngaa5	Subject	Broad	r1	216.2769274	1	1	broad_focus	5	Broad focus
2023410	block2	Control	pre	ngaa5	Subject	Broad	r1	189.7295109	2	2	broad_focus	5	Broad focus
2023410	block2	Control	pre	maai5	Verb	Broad	r1	195.8323435	3	1	broad_focus	5	Broad focus
2023410	block2	Control	pre	pou5	Object	Broad	r1	129.1005291	4	1	broad_focus	5	Broad focus
2023410	block2	Control	pre	pou5	Object	Broad	r1	294.4739229	5	2	broad_focus	5	Broad focus
2023410	block2	Control	pre	ma4	Subject	Narrow	r1	168.5667873	1	1	pre_focus	4	Narrow pre_focus
2023410	block2	Control	pre	ma4	Subject	Narrow	r1	172.6746699	2	2	pre_focus	4	Narrow pre_focus
2023410	block2	Control	pre	fu4	Verb	Narrow	r1	115.4175575	3	1	on_focus	4	Narrow on_focus
2023410	block2	Control	pre	maang4	Object	Narrow	r1	312.6643991	4	1	post_focus	4	Narrow post_focus
2023410	block2	Control	pre	Jan-04	Object	Narrow	r1	251.4533086	5	2	post_focus	4	Narrow post_focus
2023410	block2	Control	pre	lok6	Subject	Contrastive	r1	154.3650794	1	1	on_focus	6	Contrastive on_focus
2023410	block2	Control	pre	lok6	Subject	Contrastive	r1	179.996189	2	2	on_focus	6	Contrastive on_focus
2023410	block2	Control	pre	waa6	Verb	Contrastive	r1	98.79818594	3	1	post_focus	6	Contrastive post_focus
2023410	block2	Control	pre	jyut6	Object	Contrastive	r1	56.66126768	4	1	post_focus	6	Contrastive post_focus
2023410	block2	Control	pre	loeng6	Object	Contrastive	r1	370.0926466	5	2	post_focus	6	Contrastive post_focus
2023410	block2	Control	pre	ma4	Subject	Contrastive	r1	138.8874353	1	1	pre_focus	4	Contrastive pre_focus
2023410	block2	Control	pre	ma4	Subject	Contrastive	r1	182.6199198	2	2	pre_focus	4	Contrastive pre_focus
2023410	block2	Control	pre	fu4	Verb	Contrastive	r1	71.86309444	3	1	pre_focus	4	Contrastive pre_focus
2023410	block2	Control	pre	maang4	Object	Contrastive	r1	351.7310496	4	1	on_focus	4	Contrastive on_focus
2023410	block2	Control	pre	Jan-04	Object	Contrastive	r1	142.0219199	5	2	on_focus	4	Contrastive on_focus
2023410	block2	Control	pre	ngaa5	Subject	Narrow	r1	211.501084	1	1	pre_focus	5	Narrow pre_focus
2023410	block2	Control	pre	ngaa5	Subject	Narrow	r1	168.0468632	2	2	pre_focus	5	Narrow pre_focus
2023410	block2	Control	pre	maai5	Verb	Narrow	r1	194.7661565	3	1	pre_focus	5	Narrow pre_focus
2023410	block2	Control	pre	pou5	Object	Narrow	r1	94.48688877	4	1	on_focus	5	Narrow on_focus
2023410	block2	Control	pre	pou5	Object	Narrow	r1	214.9773243	5	2	on_focus	5	Narrow on_focus
2023410	block2	Control	pre	ngaa5	Subject	Contrastive	r1	156.4777022	1	1	pre_focus	5	Contrastive pre_focus
2023410	block2	Control	pre	ngaa5	Subject	Contrastive	r1	206.9224515	2	2	pre_focus	5	Contrastive pre_focus
2023410	block2	Control	pre	maai5	Verb	Contrastive	r1	231.6696901	3	1	on_focus	5	Contrastive on_focus
2023410	block2	Control	pre	pou5	Object	Contrastive	r1	162.0790411	4	1	post_focus	5	Contrastive post_focus
2023410	block2	Control	pre	pou5	Object	Contrastive	r1	258.6054422	5	2	post_focus	5	Contrastive post_focus
2023410	block2	Control	pre	ma4	Subject	Narrow	r1	137.1776724	1	1	on_focus	4	Narrow on_focus
2023410	block2	Control	pre	ma4	Subject	Narrow	r1	157.2477324	2	2	on_focus	4	Narrow on_focus
2023410	block2	Control	pre	fu4	Verb	Narrow	r1	159.8104956	3	1	post_focus	4	Narrow post_focus
2023410	block2	Control	pre	maang4	Object	Narrow	r1	253.1754535	4	1	post_focus	4	Narrow post_focus
2023410	block2	Control	pre	Jan-04	Object	Narrow	r1	234.0041572	5	2	post_focus	4	Narrow post_focus
2023410	block2	Control	pre	ngaa5	Subject	Narrow	r1	182.0344805	1	1	on_focus	5	Narrow on_focus

2023410	block2	Control	pre	ngaa5	Subject	Narrow	r1	270.8551992	2	2	on_focus	5	Narrow on_focus
2023410	block2	Control	pre	maai5	Verb	Narrow	r1	281.5946311	3	1	post_focus	5	Narrow post_focus
2023410	block2	Control	pre	pou5	Object	Narrow	r1	122.5673973	4	1	post_focus	5	Narrow post_focus
2023410	block2	Control	pre	pou5	Object	Narrow	r1	252.3718821	5	2	post_focus	5	Narrow post_focus
2023410	block2	Control	pre	lok6	Subject	Narrow	r1	118.4189641	1	1	on_focus	6	Narrow on_focus
2023410	block2	Control	pre	lok6	Subject	Narrow	r1	103.0169528	2	2	on_focus	6	Narrow on_focus
2023410	block2	Control	pre	waa6	Verb	Narrow	r1	102.0912069	3	1	post_focus	6	Narrow post_focus
2023410	block2	Control	pre	jyut6	Object	Narrow	r1	83.6781935	4	1	post_focus	6	Narrow post_focus
2023410	block2	Control	pre	loeng6	Object	Narrow	r1	407.1693122	5	2	post_focus	6	Narrow post_focus
2023410	block2	Control	pre	lok6	Subject	Narrow	r1	96.01959832	1	1	pre_focus	6	Narrow pre_focus
2023410	block2	Control	pre	lok6	Subject	Narrow	r1	169.3529856	2	2	pre_focus	6	Narrow pre_focus
2023410	block2	Control	pre	waa6	Verb	Narrow	r1	88.49412492	3	1	on_focus	6	Narrow on_focus
2023410	block2	Control	pre	jyut6	Object	Narrow	r1	55.55933485	4	1	post_focus	6	Narrow post_focus
2023410	block2	Control	pre	loeng6	Object	Narrow	r1	466.7699136	5	2	post_focus	6	Narrow post_focus
2023410	block2	Control	pre	ma4	Subject	Contrastive	r1	152.8163265	1	1	on_focus	4	Contrastive on_focus
2023410	block2	Control	pre	ma4	Subject	Contrastive	r1	217.0408163	2	2	on_focus	4	Contrastive on_focus
2023410	block2	Control	pre	fu4	Verb	Contrastive	r1	117.414966	3	1	post_focus	4	Contrastive post_focus
2023410	block2	Control	pre	maang4	Object	Contrastive	r1	284.2770759	4	1	post_focus	4	Contrastive post_focus
2023410	block2	Control	pre	Jan-04	Object	Contrastive	r1	117.2211915	5	2	post_focus	4	Contrastive post_focus
2023410	block2	Control	pre	ngaa5	Subject	Narrow	r2	150.3963837	1	1	on_focus	5	Narrow on_focus
2023410	block2	Control	pre	ngaa5	Subject	Narrow	r2	184.4717093	2	2	on_focus	5	Narrow on_focus
2023410	block2	Control	pre	maai5	Verb	Narrow	r2	214.8448201	3	1	post_focus	5	Narrow post_focus
2023410	block2	Control	pre	pou5	Object	Narrow	r2	146.6024187	4	1	post_focus	5	Narrow post_focus
2023410	block2	Control	pre	pou5	Object	Narrow	r2	246.8178382	5	2	post_focus	5	Narrow post_focus
2023410	block2	Control	pre	ngaa5	Subject	Contrastive	r2	129.4375431	1	1	on_focus	5	Contrastive on_focus
2023410	block2	Control	pre	ngaa5	Subject	Contrastive	r2	195.0141723	2	2	on_focus	5	Contrastive on_focus
2023410	block2	Control	pre	maai5	Verb	Contrastive	r2	222.6287058	3	1	post_focus	5	Contrastive post_focus
2023410	block2	Control	pre	pou5	Object	Contrastive	r2	117.9807256	4	1	post_focus	5	Contrastive post_focus
2023410	block2	Control	pre	pou5	Object	Contrastive	r2	253.6637512	5	2	post_focus	5	Contrastive post_focus
2023410	block2	Control	pre	ma4	Subject	Contrastive	r2	204.5291005	1	1	pre_focus	4	Contrastive pre_focus
2023410	block2	Control	pre	ma4	Subject	Contrastive	r2	175.8318704	2	2	pre_focus	4	Contrastive pre_focus
2023410	block2	Control	pre	fu4	Verb	Contrastive	r2	142.4979304	3	1	on_focus	4	Contrastive on_focus
2023410	block2	Control	pre	maang4	Object	Contrastive	r2	310.1768707	4	1	post_focus	4	Contrastive post_focus
2023410	block2	Control	pre	Jan-04	Object	Contrastive	r2	157.5696649	5	2	post_focus	4	Contrastive post_focus
2023410	block2	Control	pre	ngaa5	Subject	Broad	r2	311.2468821	1	1	broad_focus	5	Broad focus
2023410	block2	Control	pre	ngaa5	Subject	Broad	r2	283.9504373	2	2	broad_focus	5	Broad focus
2023410	block2	Control	pre	maai5	Verb	Broad	r2	318.9297052	3	1	broad_focus	5	Broad focus
2023410	block2	Control	pre	pou5	Object	Broad	r2	138.7301587	4	1	broad_focus	5	Broad focus
2023410	block2	Control	pre	pou5	Object	Broad	r2	336.4852608	5	2	broad_focus	5	Broad focus
2023410	block2	Control	pre	ngaa5	Subject	Narrow	r2	410.7759961	1	1	pre_focus	5	Narrow pre_focus
2023410	block2	Control	pre	ngaa5	Subject	Narrow	r2	403.9614935	2	2	pre_focus	5	Narrow pre_focus
2023410	block2	Control	pre	maai5	Verb	Narrow	r2	340.8971346	3	1	pre_focus	5	Narrow pre_focus
2023410	block2	Control	pre	pou5	Object	Narrow	r2	186.3707483	4	1	on_focus	5	Narrow on_focus
2023410	block2	Control	pre	pou5	Object	Narrow	r2	276.3307823	5	2	on_focus	5	Narrow on_focus
2023410	block2	Control	pre	ngaa5	Subject	Narrow	r2	202.1055682	1	1	pre_focus	5	Narrow pre_focus
2023410	block2	Control	pre	ngaa5	Subject	Narrow	r2	193.3557305	2	2	pre_focus	5	Narrow pre_focus
2023410	block2	Control	pre	maai5	Verb	Narrow	r2	246.0997732	3	1	on_focus	5	Narrow on_focus
2023410	block2	Control	pre	pou5	Object	Narrow	r2	126.4521193	4	1	post_focus	5	Narrow post_focus
2023410	block2	Control	pre	pou5	Object	Narrow	r2	256.3926682	5	2	post_focus	5	Narrow post_focus
2023410	block2	Control	pre	ngaa5	Subject	Contrastive	r2	163.255228	1	1	pre_focus	5	Contrastive pre_focus
2023410	block2	Control	pre	ngaa5	Subject	Contrastive	r2	223.1216931	2	2	pre_focus	5	Contrastive pre_focus
2023410	block2	Control	pre	maai5	Verb	Contrastive	r2	201.5866537	3	1	pre_focus	5	Contrastive pre_focus
2023410	block2	Control	pre	pou5	Object	Contrastive	r2	143.4507433	4	1	on_focus	5	Contrastive on_focus
2023410	block2	Control	pre	pou5	Object	Contrastive	r2	204.8311917	5	2	on_focus	5	Contrastive on_focus
2023410	block2	Control	pre	ma4	Subject	Narrow	r2	141.9900576	1	1	pre_focus	4	Narrow pre_focus
2023410	block2	Control	pre	ma4	Subject	Narrow	r2	177.8490444	2	2	pre_focus	4	Narrow pre_focus
2023410	block2	Control	pre	fu4	Verb	Narrow	r2	87.11154303	3	1	on_focus	4	Narrow on_focus
2023410	block2	Control	pre	maang4	Object	Narrow	r2	304.6730915	4	1	post_focus	4	Narrow post_focus
2023410	block2	Control	pre	Jan-04	Object	Narrow	r2	196.3497103	5	2	post_focus	4	Narrow post_focus
2023410	block2	Control	pre	lok6	Subject	Contrastive	r2	117.2884858	1	1	pre_focus	6	Contrastive pre_focus
2023410	block2	Control	pre	lok6	Subject	Contrastive	r2	125.2010173	2	2	pre_focus	6	Contrastive pre_focus
2023410	block2	Control	pre	waa6	Verb	Contrastive	r2	96.97656841	3	1	pre_focus	6	Contrastive pre_focus
2023410	block2	Control	pre	jyut6	Object	Contrastive	r2	45.34789354	4	1	on_focus	6	Contrastive on_focus
2023410	block2	Control	pre	loeng6	Object	Contrastive	r2	385.8061339	5	2	on_focus	6	Contrastive on_focus
2023410	block2	Control	pre	ma4	Subject	Contrastive	r2	184.021542	1	1	pre_focus	4	Contrastive pre_focus
2023410	block2	Control	pre	ma4	Subject	Contrastive	r2	146.0695389	2	2	pre_focus	4	Contrastive pre_focus

2023410	block2	Control	pre	fu4	Verb	Contrastive	r2	114.4847569	3	1	pre_focus	4	Contrastive pre_focus
2023410	block2	Control	pre	maang4	Object	Contrastive	r2	271.0442272	4	1	on_focus	4	Contrastive on_focus
2023410	block2	Control	pre	Jan-04	Object	Contrastive	r2	284.0136054	5	2	on_focus	4	Contrastive on_focus
2023410	block2	Control	pre	ma4	Subject	Narrow	r2	199.9909297	1	1	on_focus	4	Narrow on_focus
2023410	block2	Control	pre	ma4	Subject	Narrow	r2	192.7064437	2	2	on_focus	4	Narrow on_focus
2023410	block2	Control	pre	fu4	Verb	Narrow	r2	108.6287658	3	1	post_focus	4	Narrow post_focus
2023410	block2	Control	pre	maang4	Object	Narrow	r2	291.1188358	4	1	post_focus	4	Narrow post_focus
2023410	block2	Control	pre	Jan-04	Object	Narrow	r2	86.76060901	5	2	post_focus	4	Narrow post_focus
2023410	block2	Control	pre	ma4	Subject	Broad	r2	259.5883029	1	1	broad_focus	4	Broad focus
2023410	block2	Control	pre	ma4	Subject	Broad	r2	132.7878559	2	2	broad_focus	4	Broad focus
2023410	block2	Control	pre	fu4	Verb	Broad	r2	82.5474129	3	1	broad_focus	4	Broad focus
2023410	block2	Control	pre	maang4	Object	Broad	r2	233.9740341	4	1	broad_focus	4	Broad focus
2023410	block2	Control	pre	Jan-04	Object	Broad	r2	283.56387	5	2	broad_focus	4	Broad focus
2023410	block2	Control	pre	ma4	Subject	Contrastive	r2	222.4187453	1	1	on_focus	4	Contrastive on_focus
2023410	block2	Control	pre	ma4	Subject	Contrastive	r2	160.7544125	2	2	on_focus	4	Contrastive on_focus
2023410	block2	Control	pre	fu4	Verb	Contrastive	r2	218.9650146	3	1	post_focus	4	Contrastive post_focus
2023410	block2	Control	pre	maang4	Object	Contrastive	r2	375.9268707	4	1	post_focus	4	Contrastive post_focus
2023410	block2	Control	pre	Jan-04	Object	Contrastive	r2	253.7324844	5	2	post_focus	4	Contrastive post_focus
2023410	block2	Control	pre	lok6	Subject	Contrastive	r2	157.5989634	1	1	on_focus	6	Contrastive on_focus
2023410	block2	Control	pre	lok6	Subject	Contrastive	r2	172.292679	2	2	on_focus	6	Contrastive on_focus
2023410	block2	Control	pre	waa6	Verb	Contrastive	r2	97.35806068	3	1	post_focus	6	Contrastive post_focus
2023410	block2	Control	pre	jyut6	Object	Contrastive	r2	48.73771731	4	1	post_focus	6	Contrastive post_focus
2023410	block2	Control	pre	loeng6	Object	Contrastive	r2	482.7947846	5	2	post_focus	6	Contrastive post_focus
2023410	block2	Control	pre	lok6	Subject	Narrow	r2	160.7321135	1	1	pre_focus	6	Narrow pre_focus
2023410	block2	Control	pre	lok6	Subject	Narrow	r2	183.5399521	2	2	pre_focus	6	Narrow pre_focus
2023410	block2	Control	pre	waa6	Verb	Narrow	r2	136.8610301	3	1	on_focus	6	Narrow on_focus
2023410	block2	Control	pre	jyut6	Object	Narrow	r2	60.07227891	4	1	post_focus	6	Narrow post_focus
2023410	block2	Control	pre	loeng6	Object	Narrow	r2	428.1829176	5	2	post_focus	6	Narrow post_focus
2023410	block2	Control	pre	ngaa5	Subject	Contrastive	r2	179.1727135	1	1	pre_focus	5	Contrastive pre_focus
2023410	block2	Control	pre	ngaa5	Subject	Contrastive	r2	218.2006803	2	2	pre_focus	5	Contrastive pre_focus
2023410	block2	Control	pre	maai5	Verb	Contrastive	r2	204.5852903	3	1	on_focus	5	Contrastive on_focus
2023410	block2	Control	pre	pou5	Object	Contrastive	r2	91.93305259	4	1	post_focus	5	Contrastive post_focus
2023410	block2	Control	pre	pou5	Object	Contrastive	r2	289.702192	5	2	post_focus	5	Contrastive post_focus
2023410	block2	Control	pre	ma4	Subject	Narrow	r2	129.2545351	1	1	pre_focus	4	Narrow pre_focus
2023410	block2	Control	pre	ma4	Subject	Narrow	r2	177.0636247	2	2	pre_focus	4	Narrow pre_focus
2023410	block2	Control	pre	fu4	Verb	Narrow	r2	188.6848073	3	1	pre_focus	4	Narrow pre_focus
2023410	block2	Control	pre	maang4	Object	Narrow	r2	487.0947571	4	1	on_focus	4	Narrow on_focus
2023410	block2	Control	pre	Jan-04	Object	Narrow	r2	350.1327839	5	2	on_focus	4	Narrow on_focus
2023410	block2	Control	pre	lok6	Subject	Narrow	r2	85.76679795	1	1	pre_focus	6	Narrow pre_focus
2023410	block2	Control	pre	lok6	Subject	Narrow	r2	144.452003	2	2	pre_focus	6	Narrow pre_focus
2023410	block2	Control	pre	waa6	Verb	Narrow	r2	90.29595563	3	1	pre_focus	6	Narrow pre_focus
2023410	block2	Control	pre	jyut6	Object	Narrow	r2	60.24061476	4	1	on_focus	6	Narrow on_focus
2023410	block2	Control	pre	loeng6	Object	Narrow	r2	354.4333289	5	2	on_focus	6	Narrow on_focus
2023410	block2	Control	pre	lok6	Subject	Narrow	r2	121.7693554	1	1	on_focus	6	Narrow on_focus
2023410	block2	Control	pre	lok6	Subject	Narrow	r2	185.7101285	2	2	on_focus	6	Narrow on_focus
2023410	block2	Control	pre	waa6	Verb	Narrow	r2	74.63728511	3	1	post_focus	6	Narrow post_focus
2023410	block2	Control	pre	jyut6	Object	Narrow	r2	56.66018788	4	1	post_focus	6	Narrow post_focus
2023410	block2	Control	pre	loeng6	Object	Narrow	r2	462.6530612	5	2	post_focus	6	Narrow post_focus
2023410	block2	Control	pre	lok6	Subject	Broad	r2	122.8817082	1	1	broad_focus	6	Broad focus
2023410	block2	Control	pre	lok6	Subject	Broad	r2	174.435941	2	2	broad_focus	6	Broad focus
2023410	block2	Control	pre	waa6	Verb	Broad	r2	94.77796674	3	1	broad_focus	6	Broad focus
2023410	block2	Control	pre	jyut6	Object	Broad	r2	51.8707483	4	1	broad_focus	6	Broad focus
2023410	block2	Control	pre	loeng6	Object	Broad	r2	362.6152683	5	2	broad_focus	6	Broad focus
2023410	block2	Control	pre	lok6	Subject	Contrastive	r2	118.5343915	1	1	pre_focus	6	Contrastive pre_focus
2023410	block2	Control	pre	lok6	Subject	Contrastive	r2	151.5668345	2	2	pre_focus	6	Contrastive pre_focus
2023410	block2	Control	pre	waa6	Verb	Contrastive	r2	106.0037695	3	1	on_focus	6	Contrastive on_focus
2023410	block2	Control	pre	jyut6	Object	Contrastive	r2	65.8307169	4	1	post_focus	6	Contrastive post_focus
2023410	block2	Control	pre	loeng6	Object	Contrastive	r2	441.5368481	5	2	post_focus	6	Contrastive post_focus
2023410	block3	Control	post	suk1	Subject	Narrow	r1	68.55698975	1	1	on_focus	1	Narrow on_focus
2023410	block3	Control	post	suk1	Subject	Narrow	r1	66.87126691	2	2	on_focus	1	Narrow on_focus
2023410	block3	Control	post	sei2	Verb	Narrow	r1	148.3860246	3	1	post_focus	2	Narrow post_focus
2023410	block3	Control	post	svy2	Object	Narrow	r1	144.7872263	4	1	post_focus	2	Narrow post_focus
2023410	block3	Control	post	kwo2	Object	Narrow	r1	316.963043	5	2	post_focus	2	Narrow post_focus
2023410	block3	Control	post	piu2	Subject	Contrastive	r1	183.9773869	1	1	on_focus	2	Contrastive on_focus
2023410	block3	Control	post	tse2	Subject	Contrastive	r1	139.9930867	2	2	on_focus	2	Contrastive on_focus
2023410	block3	Control	post	tsap1	Verb	Contrastive	r1	68.48560733	3	1	post_focus	1	Contrastive post_focus

2023410	block3	Control	post	sy1	Object	Contrastive	r1	127.4349829	4	1	post_focus	1	Contrastive post_focus
2023410	block3	Control	post	pau1	Object	Contrastive	r1	352.0541664	5	2	post_focus	1	Contrastive post_focus
2023410	block3	Control	post	piu2	Subject	Narrow	r1	167.6377508	1	1	on_focus	2	Narrow on_focus
2023410	block3	Control	post	tse2	Subject	Narrow	r1	150.6497199	2	2	on_focus	2	Narrow on_focus
2023410	block3	Control	post	tsap1	Verb	Narrow	r1	59.86600831	3	1	post_focus	1	Narrow post_focus
2023410	block3	Control	post	sy1	Object	Narrow	r1	117.6234914	4	1	post_focus	1	Narrow post_focus
2023410	block3	Control	post	pau1	Object	Narrow	r1	376.2844034	5	2	post_focus	1	Narrow post_focus
2023410	block3	Control	post	piu2	Subject	Narrow	r1	156.6568096	1	1	pre_focus	2	Narrow pre_focus
2023410	block3	Control	post	tse2	Subject	Narrow	r1	149.475165	2	2	pre_focus	2	Narrow pre_focus
2023410	block3	Control	post	tsap1	Verb	Narrow	r1	33.49288182	3	1	pre_focus	1	Narrow pre_focus
2023410	block3	Control	post	sy1	Object	Narrow	r1	112.3934561	4	1	on_focus	1	Narrow on_focus
2023410	block3	Control	post	pau1	Object	Narrow	r1	341.7533374	5	2	on_focus	1	Narrow on_focus
2023410	block3	Control	post	suk1	Subject	Contrastive	r1	69.1175192	1	1	on_focus	1	Contrastive on_focus
2023410	block3	Control	post	suk1	Subject	Contrastive	r1	83.06777109	2	2	on_focus	1	Contrastive on_focus
2023410	block3	Control	post	sei2	Verb	Contrastive	r1	142.3130868	3	1	post_focus	2	Contrastive post_focus
2023410	block3	Control	post	svy2	Object	Contrastive	r1	129.8522126	4	1	post_focus	2	Contrastive post_focus
2023410	block3	Control	post	kwo2	Object	Contrastive	r1	286.4524926	5	2	post_focus	2	Contrastive post_focus
2023410	block3	Control	post	piu2	Subject	Contrastive	r1	137.7384775	1	1	pre_focus	2	Contrastive pre_focus
2023410	block3	Control	post	tse2	Subject	Contrastive	r1	178.200084	2	2	pre_focus	2	Contrastive pre_focus
2023410	block3	Control	post	tsap1	Verb	Contrastive	r1	67.44977759	3	1	pre_focus	1	Contrastive pre_focus
2023410	block3	Control	post	sy1	Object	Contrastive	r1	129.6255837	4	1	on_focus	1	Contrastive on_focus
2023410	block3	Control	post	pau1	Object	Contrastive	r1	407.0568465	5	2	on_focus	1	Contrastive on_focus
2023410	block3	Control	post	suk1	Subject	Narrow	r1	48.25372164	1	1	pre_focus	1	Narrow pre_focus
2023410	block3	Control	post	suk1	Subject	Narrow	r1	62.88443045	2	2	pre_focus	1	Narrow pre_focus
2023410	block3	Control	post	sei2	Verb	Narrow	r1	152.5472065	3	1	on_focus	2	Narrow on_focus
2023410	block3	Control	post	svy2	Object	Narrow	r1	114.243673	4	1	post_focus	2	Narrow post_focus
2023410	block3	Control	post	kwo2	Object	Narrow	r1	294.6870013	5	2	post_focus	2	Narrow post_focus
2023410	block3	Control	post	bui3	Subject	Narrow	r1	173.2352113	1	1	on_focus	3	Narrow on_focus
2023410	block3	Control	post	bui3	Subject	Narrow	r1	134.0749591	2	2	on_focus	3	Narrow on_focus
2023410	block3	Control	post	tsv1	Verb	Narrow	r1	106.453062	3	1	post_focus	1	Narrow post_focus
2023410	block3	Control	post	fug1	Object	Narrow	r1	196.2114058	4	1	post_focus	1	Narrow post_focus
2023410	block3	Control	post	tshe1	Object	Narrow	r1	384.6809857	5	2	post_focus	1	Narrow post_focus
2023410	block3	Control	post	bui3	Subject	Contrastive	r1	165.7626345	1	1	pre_focus	3	Contrastive pre_focus
2023410	block3	Control	post	bui3	Subject	Contrastive	r1	137.7896463	2	2	pre_focus	3	Contrastive pre_focus
2023410	block3	Control	post	tsv1	Verb	Contrastive	r1	141.7727761	3	1	pre_focus	1	Contrastive pre_focus
2023410	block3	Control	post	fug1	Object	Contrastive	r1	162.8455916	4	1	on_focus	1	Contrastive on_focus
2023410	block3	Control	post	tshe1	Object	Contrastive	r1	323.1981376	5	2	on_focus	1	Contrastive on_focus
2023410	block3	Control	post	bui3	Subject	Contrastive	r1	148.3439646	1	1	pre_focus	3	Contrastive pre_focus
2023410	block3	Control	post	bui3	Subject	Contrastive	r1	162.357792	2	2	pre_focus	3	Contrastive pre_focus
2023410	block3	Control	post	tsv1	Verb	Contrastive	r1	114.3098534	3	1	on_focus	1	Contrastive on_focus
2023410	block3	Control	post	fug1	Object	Contrastive	r1	199.8729379	4	1	post_focus	1	Contrastive post_focus
2023410	block3	Control	post	tshe1	Object	Contrastive	r1	298.6964122	5	2	post_focus	1	Contrastive post_focus
2023410	block3	Control	post	piu2	Subject	Broad	r1	432.7471655	1	1	broad_focus	2	Broad focus
2023410	block3	Control	post	tse2	Subject	Broad	r1	379.1588115	2	2	broad_focus	2	Broad focus
2023410	block3	Control	post	tsap1	Verb	Broad	r1	61.76393088	3	1	broad_focus	1	Broad focus
2023410	block3	Control	post	sy1	Object	Broad	r1	126.5943039	4	1	broad_focus	1	Broad focus
2023410	block3	Control	post	pau1	Object	Broad	r1	430.5029673	5	2	broad_focus	1	Broad focus
2023410	block3	Control	post	bui3	Subject	Narrow	r1	126.1439322	1	1	pre_focus	3	Narrow pre_focus
2023410	block3	Control	post	bui3	Subject	Narrow	r1	148.6417476	2	2	pre_focus	3	Narrow pre_focus
2023410	block3	Control	post	tsv1	Verb	Narrow	r1	101.678077	3	1	on_focus	1	Narrow on_focus
2023410	block3	Control	post	fug1	Object	Narrow	r1	145.657468	4	1	post_focus	1	Narrow post_focus
2023410	block3	Control	post	tshe1	Object	Narrow	r1	252.246882	5	2	post_focus	1	Narrow post_focus
2023410	block3	Control	post	suk1	Subject	Contrastive	r1	49.77136312	1	1	pre_focus	1	Contrastive pre_focus
2023410	block3	Control	post	suk1	Subject	Contrastive	r1	50.95591566	2	2	pre_focus	1	Contrastive pre_focus
2023410	block3	Control	post	sei2	Verb	Contrastive	r1	160.2854617	3	1	pre_focus	2	Contrastive pre_focus
2023410	block3	Control	post	svy2	Object	Contrastive	r1	142.0525957	4	1	on_focus	2	Contrastive on_focus
2023410	block3	Control	post	kwo2	Object	Contrastive	r1	307.0066529	5	2	on_focus	2	Contrastive on_focus
2023410	block3	Control	post	piu2	Subject	Narrow	r1	152.0912162	1	1	pre_focus	2	Narrow pre_focus
2023410	block3	Control	post	tse2	Subject	Narrow	r1	121.2387269	2	2	pre_focus	2	Narrow pre_focus
2023410	block3	Control	post	tsap1	Verb	Narrow	r1	39.02073924	3	1	on_focus	1	Narrow on_focus
2023410	block3	Control	post	sy1	Object	Narrow	r1	91.71734412	4	1	post_focus	1	Narrow post_focus
2023410	block3	Control	post	pau1	Object	Narrow	r1	384.9959858	5	2	post_focus	1	Narrow post_focus
2023410	block3	Control	post	suk1	Subject	Contrastive	r1	70.79881269	1	1	pre_focus	1	Contrastive pre_focus
2023410	block3	Control	post	suk1	Subject	Contrastive	r1	55.93116546	2	2	pre_focus	1	Contrastive pre_focus
2023410	block3	Control	post	sei2	Verb	Contrastive	r1	165.9177974	3	1	on_focus	2	Contrastive on_focus
2023410	block3	Control	post	svy2	Object	Contrastive	r1	189.2796237	4	1	post_focus	2	Contrastive post_focus

2023410	block3	Control	post	kwo2	Object	Contrastive	r1	209.6201884	5	2	post_focus	2	Contrastive post_focus
2023410	block3	Control	post	bui3	Subject	Broad	r1	163.1369955	1	1	broad_focus	3	Broad focus
2023410	block3	Control	post	bui3	Subject	Broad	r1	176.0767671	2	2	broad_focus	3	Broad focus
2023410	block3	Control	post	tsv1	Verb	Broad	r1	109.9419821	3	1	broad_focus	1	Broad focus
2023410	block3	Control	post	fug1	Object	Broad	r1	165.3876657	4	1	broad_focus	1	Broad focus
2023410	block3	Control	post	tshe1	Object	Broad	r1	313.8313993	5	2	broad_focus	1	Broad focus
2023410	block3	Control	post	bui3	Subject	Contrastive	r1	231.8200965	1	1	on_focus	3	Contrastive on_focus
2023410	block3	Control	post	bui3	Subject	Contrastive	r1	275.2890327	2	2	on_focus	3	Contrastive on_focus
2023410	block3	Control	post	tsv1	Verb	Contrastive	r1	172.7641233	3	1	post_focus	1	Contrastive post_focus
2023410	block3	Control	post	fug1	Object	Contrastive	r1	192.9129075	4	1	post_focus	1	Contrastive post_focus
2023410	block3	Control	post	tshe1	Object	Contrastive	r1	295.6973136	5	2	post_focus	1	Contrastive post_focus
2023410	block3	Control	post	suk1	Subject	Broad	r1	74.35439732	1	1	broad_focus	1	Broad focus
2023410	block3	Control	post	suk1	Subject	Broad	r1	98.72637925	2	2	broad_focus	1	Broad focus
2023410	block3	Control	post	sei2	Verb	Broad	r1	168.0937907	3	1	broad_focus	2	Broad focus
2023410	block3	Control	post	svy2	Object	Broad	r1	162.1970364	4	1	broad_focus	2	Broad focus
2023410	block3	Control	post	kwo2	Object	Broad	r1	424.1176324	5	2	broad_focus	2	Broad focus
2023410	block3	Control	post	suk1	Subject	Narrow	r1	58.34048491	1	1	pre_focus	1	Narrow pre_focus
2023410	block3	Control	post	suk1	Subject	Narrow	r1	83.31172689	2	2	pre_focus	1	Narrow pre_focus
2023410	block3	Control	post	sei2	Verb	Narrow	r1	188.0096229	3	1	pre_focus	2	Narrow pre_focus
2023410	block3	Control	post	svy2	Object	Narrow	r1	186.1445686	4	1	on_focus	2	Narrow on_focus
2023410	block3	Control	post	kwo2	Object	Narrow	r1	319.1358337	5	2	on_focus	2	Narrow on_focus
2023410	block3	Control	post	piu2	Subject	Contrastive	r1	243.4657636	1	1	pre_focus	2	Contrastive pre_focus
2023410	block3	Control	post	tse2	Subject	Contrastive	r1	172.2288183	2	2	pre_focus	2	Contrastive pre_focus
2023410	block3	Control	post	tsap1	Verb	Contrastive	r1	90.12427178	3	1	on_focus	1	Contrastive on_focus
2023410	block3	Control	post	sy1	Object	Contrastive	r1	130.689565	4	1	post_focus	1	Contrastive post_focus
2023410	block3	Control	post	pau1	Object	Contrastive	r1	411.9628446	5	2	post_focus	1	Contrastive post_focus
2023410	block3	Control	post	bui3	Subject	Narrow	r1	170.3947768	1	1	pre_focus	3	Narrow pre_focus
2023410	block3	Control	post	bui3	Subject	Narrow	r1	182.8057873	2	2	pre_focus	3	Narrow pre_focus
2023410	block3	Control	post	tsv1	Verb	Narrow	r1	148.8202471	3	1	pre_focus	1	Narrow pre_focus
2023410	block3	Control	post	fug1	Object	Narrow	r1	197.7209185	4	1	on_focus	1	Narrow on_focus
2023410	block3	Control	post	tshe1	Object	Narrow	r1	326.9770082	5	2	on_focus	1	Narrow on_focus
2023410	block3	Control	post	suk1	Subject	Contrastive	r2	62.73362488	1	1	pre_focus	1	Contrastive pre_focus
2023410	block3	Control	post	suk1	Subject	Contrastive	r2	124.8373547	2	2	pre_focus	1	Contrastive pre_focus
2023410	block3	Control	post	sei2	Verb	Contrastive	r2	188.3264747	3	1	on_focus	2	Contrastive on_focus
2023410	block3	Control	post	svy2	Object	Contrastive	r2	242.7286044	4	1	post_focus	2	Contrastive post_focus
2023410	block3	Control	post	kwo2	Object	Contrastive	r2	345.5318767	5	2	post_focus	2	Contrastive post_focus
2023410	block3	Control	post	bui3	Subject	Narrow	r2	168.7340242	1	1	pre_focus	3	Narrow pre_focus
2023410	block3	Control	post	bui3	Subject	Narrow	r2	160.5340382	2	2	pre_focus	3	Narrow pre_focus
2023410	block3	Control	post	tsv1	Verb	Narrow	r2	118.1928861	3	1	on_focus	1	Narrow on_focus
2023410	block3	Control	post	fug1	Object	Narrow	r2	191.0983763	4	1	post_focus	1	Narrow post_focus
2023410	block3	Control	post	tshe1	Object	Narrow	r2	265.6470325	5	2	post_focus	1	Narrow post_focus
2023410	block3	Control	post	suk1	Subject	Narrow	r2	73.41596542	1	1	pre_focus	1	Narrow pre_focus
2023410	block3	Control	post	suk1	Subject	Narrow	r2	81.09502183	2	2	pre_focus	1	Narrow pre_focus
2023410	block3	Control	post	sei2	Verb	Narrow	r2	161.7366047	3	1	on_focus	2	Narrow on_focus
2023410	block3	Control	post	svy2	Object	Narrow	r2	148.8226611	4	1	post_focus	2	Narrow post_focus
2023410	block3	Control	post	kwo2	Object	Narrow	r2	232.5212171	5	2	post_focus	2	Narrow post_focus
2023410	block3	Control	post	piu2	Subject	Broad	r2	192.1763414	1	1	broad_focus	2	Broad focus
2023410	block3	Control	post	tse2	Subject	Broad	r2	145.3395744	2	2	broad_focus	2	Broad focus
2023410	block3	Control	post	tsap1	Verb	Broad	r2	28.90103789	3	1	broad_focus	1	Broad focus
2023410	block3	Control	post	sy1	Object	Broad	r2	130.2341555	4	1	broad_focus	1	Broad focus
2023410	block3	Control	post	pau1	Object	Broad	r2	435.7949216	5	2	broad_focus	1	Broad focus
2023410	block3	Control	post	piu2	Subject	Contrastive	r2	144.7131723	1	1	pre_focus	2	Contrastive pre_focus
2023410	block3	Control	post	tse2	Subject	Contrastive	r2	117.5749006	2	2	pre_focus	2	Contrastive pre_focus
2023410	block3	Control	post	tsap1	Verb	Contrastive	r2	36.11305731	3	1	on_focus	1	Contrastive on_focus
2023410	block3	Control	post	sy1	Object	Contrastive	r2	80.76583157	4	1	post_focus	1	Contrastive post_focus
2023410	block3	Control	post	pau1	Object	Contrastive	r2	346.8971196	5	2	post_focus	1	Contrastive post_focus
2023410	block3	Control	post	bui3	Subject	Contrastive	r2	157.0634968	1	1	on_focus	3	Contrastive on_focus
2023410	block3	Control	post	bui3	Subject	Contrastive	r2	148.6837312	2	2	on_focus	3	Contrastive on_focus
2023410	block3	Control	post	tsv1	Verb	Contrastive	r2	121.6436859	3	1	post_focus	1	Contrastive post_focus
2023410	block3	Control	post	fug1	Object	Contrastive	r2	178.9940123	4	1	post_focus	1	Contrastive post_focus
2023410	block3	Control	post	tshe1	Object	Contrastive	r2	255.405821	5	2	post_focus	1	Contrastive post_focus
2023410	block3	Control	post	bui3	Subject	Narrow	r2	141.6173392	1	1	pre_focus	3	Narrow pre_focus
2023410	block3	Control	post	bui3	Subject	Narrow	r2	142.000704	2	2	pre_focus	3	Narrow pre_focus
2023410	block3	Control	post	tsv1	Verb	Narrow	r2	115.5854776	3	1	pre_focus	1	Narrow pre_focus
2023410	block3	Control	post	fug1	Object	Narrow	r2	164.6201608	4	1	on_focus	1	Narrow on_focus
2023410	block3	Control	post	tshe1	Object	Narrow	r2	258.6367813	5	2	on_focus	1	Narrow on_focus

**2023410**	block3	Control	post	bui3	Subject	Narrow	r2	155.1827187	1	1	on_focus	3	Narrow on_focus
**2023410**	block3	Control	post	bui3	Subject	Narrow	r2	149.260828	2	2	on_focus	3	Narrow on_focus
**2023410**	block3	Control	post	tsv1	Verb	Narrow	r2	76.58692749	3	1	post_focus	1	Narrow post_focus
**2023410**	block3	Control	post	fug1	Object	Narrow	r2	113.0845506	4	1	post_focus	1	Narrow post_focus
**2023410**	block3	Control	post	tshe1	Object	Narrow	r2	259.7892556	5	2	post_focus	1	Narrow post_focus
**2023410**	block3	Control	post	piu2	Subject	Contrastive	r2	164.1042118	1	1	pre_focus	2	Contrastive pre_focus
**2023410**	block3	Control	post	tse2	Subject	Contrastive	r2	145.3998659	2	2	pre_focus	2	Contrastive pre_focus
**2023410**	block3	Control	post	tsap1	Verb	Contrastive	r2	36.3598231	3	1	pre_focus	1	Contrastive pre_focus
**2023410**	block3	Control	post	sy1	Object	Contrastive	r2	117.1120747	4	1	on_focus	1	Contrastive on_focus
**2023410**	block3	Control	post	pau1	Object	Contrastive	r2	362.8262017	5	2	on_focus	1	Contrastive on_focus
**2023410**	block3	Control	post	suk1	Subject	Contrastive	r2	80.97655322	1	1	pre_focus	1	Contrastive pre_focus
**2023410**	block3	Control	post	suk1	Subject	Contrastive	r2	125.6808225	2	2	pre_focus	1	Contrastive pre_focus
**2023410**	block3	Control	post	sei2	Verb	Contrastive	r2	137.6141282	3	1	pre_focus	2	Contrastive pre_focus
**2023410**	block3	Control	post	svy2	Object	Contrastive	r2	118.9373889	4	1	on_focus	2	Contrastive on_focus
**2023410**	block3	Control	post	kwo2	Object	Contrastive	r2	318.0010933	5	2	on_focus	2	Contrastive on_focus
**2023410**	block3	Control	post	piu2	Subject	Contrastive	r2	168.7377173	1	1	on_focus	2	Contrastive on_focus
**2023410**	block3	Control	post	tse2	Subject	Contrastive	r2	145.3115596	2	2	on_focus	2	Contrastive on_focus
**2023410**	block3	Control	post	tsap1	Verb	Contrastive	r2	71.91578438	3	1	post_focus	1	Contrastive post_focus
**2023410**	block3	Control	post	sy1	Object	Contrastive	r2	101.0015464	4	1	post_focus	1	Contrastive post_focus
**2023410**	block3	Control	post	pau1	Object	Contrastive	r2	371.5882194	5	2	post_focus	1	Contrastive post_focus
**2023410**	block3	Control	post	bui3	Subject	Contrastive	r2	198.4777977	1	1	pre_focus	3	Contrastive pre_focus
**2023410**	block3	Control	post	bui3	Subject	Contrastive	r2	149.618344	2	2	pre_focus	3	Contrastive pre_focus
**2023410**	block3	Control	post	tsv1	Verb	Contrastive	r2	102.9625711	3	1	pre_focus	1	Contrastive pre_focus
**2023410**	block3	Control	post	fug1	Object	Contrastive	r2	212.459087	4	1	on_focus	1	Contrastive on_focus
**2023410**	block3	Control	post	tshe1	Object	Contrastive	r2	289.9791481	5	2	on_focus	1	Contrastive on_focus
**2023410**	block3	Control	post	suk1	Subject	Broad	r2	65.80935905	1	1	broad_focus	1	Broad focus
**2023410**	block3	Control	post	suk1	Subject	Broad	r2	62.79124969	2	2	broad_focus	1	Broad focus
**2023410**	block3	Control	post	sei2	Verb	Broad	r2	168.5022383	3	1	broad_focus	2	Broad focus
**2023410**	block3	Control	post	svy2	Object	Broad	r2	143.7287039	4	1	broad_focus	2	Broad focus
**2023410**	block3	Control	post	kwo2	Object	Broad	r2	341.9032788	5	2	broad_focus	2	Broad focus
**2023410**	block3	Control	post	piu2	Subject	Narrow	r2	129.0227438	1	1	pre_focus	2	Narrow pre_focus
**2023410**	block3	Control	post	tse2	Subject	Narrow	r2	133.7952541	2	2	pre_focus	2	Narrow pre_focus
**2023410**	block3	Control	post	tsap1	Verb	Narrow	r2	56.748297	3	1	on_focus	1	Narrow on_focus
**2023410**	block3	Control	post	sy1	Object	Narrow	r2	139.1437443	4	1	post_focus	1	Narrow post_focus
**2023410**	block3	Control	post	pau1	Object	Narrow	r2	424.6559365	5	2	post_focus	1	Narrow post_focus
**2023410**	block3	Control	post	piu2	Subject	Narrow	r2	153.4717086	1	1	pre_focus	2	Narrow pre_focus
**2023410**	block3	Control	post	tse2	Subject	Narrow	r2	149.34471	2	2	pre_focus	2	Narrow pre_focus
**2023410**	block3	Control	post	tsap1	Verb	Narrow	r2	48.83593392	3	1	pre_focus	1	Narrow pre_focus
**2023410**	block3	Control	post	sy1	Object	Narrow	r2	120.9659155	4	1	on_focus	1	Narrow on_focus
**2023410**	block3	Control	post	pau1	Object	Narrow	r2	346.3375907	5	2	on_focus	1	Narrow on_focus
**2023410**	block3	Control	post	suk1	Subject	Contrastive	r2	90.31378369	1	1	on_focus	1	Contrastive on_focus
**2023410**	block3	Control	post	suk1	Subject	Contrastive	r2	69.65310837	2	2	on_focus	1	Contrastive on_focus
**2023410**	block3	Control	post	sei2	Verb	Contrastive	r2	154.2361886	3	1	post_focus	2	Contrastive post_focus
**2023410**	block3	Control	post	svy2	Object	Contrastive	r2	127.9303261	4	1	post_focus	2	Contrastive post_focus
**2023410**	block3	Control	post	kwo2	Object	Contrastive	r2	256.3154084	5	2	post_focus	2	Contrastive post_focus
**2023410**	block3	Control	post	suk1	Subject	Narrow	r2	67.53813333	1	1	on_focus	1	Narrow on_focus
**2023410**	block3	Control	post	suk1	Subject	Narrow	r2	146.7740214	2	2	on_focus	1	Narrow on_focus
**2023410**	block3	Control	post	sei2	Verb	Narrow	r2	141.1883437	3	1	post_focus	2	Narrow post_focus
**2023410**	block3	Control	post	svy2	Object	Narrow	r2	168.5632983	4	1	post_focus	2	Narrow post_focus
**2023410**	block3	Control	post	kwo2	Object	Narrow	r2	326.4585857	5	2	post_focus	2	Narrow post_focus
**2023410**	block3	Control	post	suk1	Subject	Narrow	r2	57.47241035	1	1	pre_focus	1	Narrow pre_focus
**2023410**	block3	Control	post	suk1	Subject	Narrow	r2	99.02931604	2	2	pre_focus	1	Narrow pre_focus
**2023410**	block3	Control	post	sei2	Verb	Narrow	r2	148.5560105	3	1	pre_focus	2	Narrow pre_focus
**2023410**	block3	Control	post	svy2	Object	Narrow	r2	144.7296415	4	1	on_focus	2	Narrow on_focus
**2023410**	block3	Control	post	kwo2	Object	Narrow	r2	323.7786643	5	2	on_focus	2	Narrow on_focus
**2023410**	block3	Control	post	bui3	Subject	Broad	r2	194.2949819	1	1	broad_focus	3	Broad focus
**2023410**	block3	Control	post	bui3	Subject	Broad	r2	161.7866526	2	2	broad_focus	3	Broad focus
**2023410**	block3	Control	post	tsv1	Verb	Broad	r2	161.7392957	3	1	broad_focus	1	Broad focus
**2023410**	block3	Control	post	fug1	Object	Broad	r2	165.5292965	4	1	broad_focus	1	Broad focus
**2023410**	block3	Control	post	tshe1	Object	Broad	r2	328.8508919	5	2	broad_focus	1	Broad focus
**2023410**	block3	Control	post	piu2	Subject	Narrow	r2	197.0749388	1	1	on_focus	2	Narrow on_focus
**2023410**	block3	Control	post	tse2	Subject	Narrow	r2	152.9887338	2	2	on_focus	2	Narrow on_focus
**2023410**	block3	Control	post	tsap1	Verb	Narrow	r2	47.16529052	3	1	post_focus	1	Narrow post_focus
**2023410**	block3	Control	post	sy1	Object	Narrow	r2	135.5296785	4	1	post_focus	1	Narrow post_focus
**2023410**	block3	Control	post	pau1	Object	Narrow	r2	361.6818611	5	2	post_focus	1	Narrow post_focus
**2023410**	block3	Control	post	bui3	Subject	Contrastive	r2	148.7527204	1	1	pre_focus	3	Contrastive pre_focus

2023410	block3	Control	post	bui3	Subject	Contrastive	r2	202.3387408	2	2	pre_focus	3	Contrastive pre_focus
2023410	block3	Control	post	tsv1	Verb	Contrastive	r2	131.6408638	3	1	on_focus	1	Contrastive on_focus
2023410	block3	Control	post	fug1	Object	Contrastive	r2	148.5571575	4	1	post_focus	1	Contrastive post_focus
2023410	block3	Control	post	tshe1	Object	Contrastive	r2	255.3676587	5	2	post_focus	1	Contrastive post_focus
2023410	block3	Control	pre	suk1	Subject	Broad	r1	48.22635485	1	1	broad_focus	1	Broad focus
2023410	block3	Control	pre	suk1	Subject	Broad	r1	71.76697796	2	2	broad_focus	1	Broad focus
2023410	block3	Control	pre	sei2	Verb	Broad	r1	125.955282	3	1	broad_focus	2	Broad focus
2023410	block3	Control	pre	svy2	Object	Broad	r1	148.7622287	4	1	broad_focus	2	Broad focus
2023410	block3	Control	pre	kwo2	Object	Broad	r1	297.2272664	5	2	broad_focus	2	Broad focus
2023410	block3	Control	pre	bui3	Subject	Contrastive	r1	125.8763571	1	1	pre_focus	3	Contrastive pre_focus
2023410	block3	Control	pre	bui3	Subject	Contrastive	r1	177.0368296	2	2	pre_focus	3	Contrastive pre_focus
2023410	block3	Control	pre	tsv1	Verb	Contrastive	r1	90.06513744	3	1	pre_focus	1	Contrastive pre_focus
2023410	block3	Control	pre	fug1	Object	Contrastive	r1	119.9003461	4	1	on_focus	1	Contrastive on_focus
2023410	block3	Control	pre	tshe1	Object	Contrastive	r1	279.6422887	5	2	on_focus	1	Contrastive on_focus
2023410	block3	Control	pre	suk1	Subject	Contrastive	r1	56.20449106	1	1	on_focus	1	Contrastive on_focus
2023410	block3	Control	pre	suk1	Subject	Contrastive	r1	58.10348289	2	2	on_focus	1	Contrastive on_focus
2023410	block3	Control	pre	sei2	Verb	Contrastive	r1	126.9469367	3	1	post_focus	2	Contrastive post_focus
2023410	block3	Control	pre	svy2	Object	Contrastive	r1	119.066448	4	1	post_focus	2	Contrastive post_focus
2023410	block3	Control	pre	kwo2	Object	Contrastive	r1	295.2053041	5	2	post_focus	2	Contrastive post_focus
2023410	block3	Control	pre	suk1	Subject	Narrow	r1	62.31421534	1	1	pre_focus	1	Narrow pre_focus
2023410	block3	Control	pre	suk1	Subject	Narrow	r1	47.85445757	2	2	pre_focus	1	Narrow pre_focus
2023410	block3	Control	pre	sei2	Verb	Narrow	r1	122.8997829	3	1	on_focus	2	Narrow on_focus
2023410	block3	Control	pre	svy2	Object	Narrow	r1	165.1269481	4	1	post_focus	2	Narrow post_focus
2023410	block3	Control	pre	kwo2	Object	Narrow	r1	355.9730631	5	2	post_focus	2	Narrow post_focus
2023410	block3	Control	pre	suk1	Subject	Narrow	r1	46.4756015	1	1	pre_focus	1	Narrow pre_focus
2023410	block3	Control	pre	suk1	Subject	Narrow	r1	29.10228663	2	2	pre_focus	1	Narrow pre_focus
2023410	block3	Control	pre	sei2	Verb	Narrow	r1	110.9755291	3	1	pre_focus	2	Narrow pre_focus
2023410	block3	Control	pre	svy2	Object	Narrow	r1	162.2224971	4	1	on_focus	2	Narrow on_focus
2023410	block3	Control	pre	kwo2	Object	Narrow	r1	300.1975773	5	2	on_focus	2	Narrow on_focus
2023410	block3	Control	pre	bui3	Subject	Narrow	r1	99.39832666	1	1	pre_focus	3	Narrow pre_focus
2023410	block3	Control	pre	bui3	Subject	Narrow	r1	106.1031211	2	2	pre_focus	3	Narrow pre_focus
2023410	block3	Control	pre	tsv1	Verb	Narrow	r1	101.866478	3	1	on_focus	1	Narrow on_focus
2023410	block3	Control	pre	fug1	Object	Narrow	r1	145.8528077	4	1	post_focus	1	Narrow post_focus
2023410	block3	Control	pre	tshe1	Object	Narrow	r1	272.0143409	5	2	post_focus	1	Narrow post_focus
2023410	block3	Control	pre	bui3	Subject	Narrow	r1	109.1670365	1	1	pre_focus	3	Narrow pre_focus
2023410	block3	Control	pre	bui3	Subject	Narrow	r1	117.9788454	2	2	pre_focus	3	Narrow pre_focus
2023410	block3	Control	pre	tsv1	Verb	Narrow	r1	96.56838297	3	1	pre_focus	1	Narrow pre_focus
2023410	block3	Control	pre	fug1	Object	Narrow	r1	123.1820309	4	1	on_focus	1	Narrow on_focus
2023410	block3	Control	pre	tshe1	Object	Narrow	r1	268.4848134	5	2	on_focus	1	Narrow on_focus
2023410	block3	Control	pre	piu2	Subject	Narrow	r1	143.3492497	1	1	on_focus	2	Narrow on_focus
2023410	block3	Control	pre	tse2	Subject	Narrow	r1	110.1780564	2	2	on_focus	2	Narrow on_focus
2023410	block3	Control	pre	tsap1	Verb	Narrow	r1	59.8599174	3	1	post_focus	1	Narrow post_focus
2023410	block3	Control	pre	sy1	Object	Narrow	r1	105.6556218	4	1	post_focus	1	Narrow post_focus
2023410	block3	Control	pre	pau1	Object	Narrow	r1	313.8348461	5	2	post_focus	1	Narrow post_focus
2023410	block3	Control	pre	piu2	Subject	Contrastive	r1	110.7818222	1	1	pre_focus	2	Contrastive pre_focus
2023410	block3	Control	pre	tse2	Subject	Contrastive	r1	103.5719386	2	2	pre_focus	2	Contrastive pre_focus
2023410	block3	Control	pre	tsap1	Verb	Contrastive	r1	49.19993682	3	1	on_focus	1	Contrastive on_focus
2023410	block3	Control	pre	sy1	Object	Contrastive	r1	97.18365935	4	1	post_focus	1	Contrastive post_focus
2023410	block3	Control	pre	pau1	Object	Contrastive	r1	356.8001992	5	2	post_focus	1	Contrastive post_focus
2023410	block3	Control	pre	bui3	Subject	Contrastive	r1	106.4806643	1	1	pre_focus	3	Contrastive pre_focus
2023410	block3	Control	pre	bui3	Subject	Contrastive	r1	156.4376075	2	2	pre_focus	3	Contrastive pre_focus
2023410	block3	Control	pre	tsv1	Verb	Contrastive	r1	149.6355969	3	1	on_focus	1	Contrastive on_focus
2023410	block3	Control	pre	fug1	Object	Contrastive	r1	136.8946717	4	1	post_focus	1	Contrastive post_focus
2023410	block3	Control	pre	tshe1	Object	Contrastive	r1	243.8690957	5	2	post_focus	1	Contrastive post_focus
2023410	block3	Control	pre	suk1	Subject	Narrow	r1	62.47493261	1	1	on_focus	1	Narrow on_focus
2023410	block3	Control	pre	suk1	Subject	Narrow	r1	55.08506945	2	2	on_focus	1	Narrow on_focus
2023410	block3	Control	pre	sei2	Verb	Narrow	r1	153.0921197	3	1	post_focus	2	Narrow post_focus
2023410	block3	Control	pre	svy2	Object	Narrow	r1	129.7210398	4	1	post_focus	2	Narrow post_focus
2023410	block3	Control	pre	kwo2	Object	Narrow	r1	313.9390718	5	2	post_focus	2	Narrow post_focus
2023410	block3	Control	pre	piu2	Subject	Contrastive	r1	165.6291025	1	1	on_focus	2	Contrastive on_focus
2023410	block3	Control	pre	tse2	Subject	Contrastive	r1	130.7625664	2	2	on_focus	2	Contrastive on_focus
2023410	block3	Control	pre	tsap1	Verb	Contrastive	r1	55.63132782	3	1	post_focus	1	Contrastive post_focus
2023410	block3	Control	pre	sy1	Object	Contrastive	r1	81.84418233	4	1	post_focus	1	Contrastive post_focus
2023410	block3	Control	pre	pau1	Object	Contrastive	r1	293.7841193	5	2	post_focus	1	Contrastive post_focus
2023410	block3	Control	pre	suk1	Subject	Contrastive	r1	100.1264608	1	1	pre_focus	1	Contrastive pre_focus
2023410	block3	Control	pre	suk1	Subject	Contrastive	r1	71.56304428	2	2	pre_focus	1	Contrastive pre_focus

2023410	block3	Control	pre	sei2	Verb	Contrastive	r1	117.124344	3	1	on_focus	2	Contrastive on_focus
2023410	block3	Control	pre	svy2	Object	Contrastive	r1	133.3490821	4	1	post_focus	2	Contrastive post_focus
2023410	block3	Control	pre	kwo2	Object	Contrastive	r1	226.8183269	5	2	post_focus	2	Contrastive post_focus
2023410	block3	Control	pre	bui3	Subject	Narrow	r1	137.894362	1	1	on_focus	3	Narrow on_focus
2023410	block3	Control	pre	bui3	Subject	Narrow	r1	172.4356906	2	2	on_focus	3	Narrow on_focus
2023410	block3	Control	pre	tsv1	Verb	Narrow	r1	97.24399121	3	1	post_focus	1	Narrow post_focus
2023410	block3	Control	pre	fug1	Object	Narrow	r1	161.8260158	4	1	post_focus	1	Narrow post_focus
2023410	block3	Control	pre	tshe1	Object	Narrow	r1	230.6304834	5	2	post_focus	1	Narrow post_focus
2023410	block3	Control	pre	bui3	Subject	Broad	r1	172.8321071	1	1	broad_focus	3	Broad focus
2023410	block3	Control	pre	bui3	Subject	Broad	r1	144.9019992	2	2	broad_focus	3	Broad focus
2023410	block3	Control	pre	tsv1	Verb	Broad	r1	100.6473975	3	1	broad_focus	1	Broad focus
2023410	block3	Control	pre	fug1	Object	Broad	r1	164.7576463	4	1	broad_focus	1	Broad focus
2023410	block3	Control	pre	tshe1	Object	Broad	r1	254.6837273	5	2	broad_focus	1	Broad focus
2023410	block3	Control	pre	bui3	Subject	Contrastive	r1	127.2955162	1	1	on_focus	3	Contrastive on_focus
2023410	block3	Control	pre	bui3	Subject	Contrastive	r1	133.8601292	2	2	on_focus	3	Contrastive on_focus
2023410	block3	Control	pre	tsv1	Verb	Contrastive	r1	106.9833884	3	1	post_focus	1	Contrastive post_focus
2023410	block3	Control	pre	fug1	Object	Contrastive	r1	151.6039972	4	1	post_focus	1	Contrastive post_focus
2023410	block3	Control	pre	tshe1	Object	Contrastive	r1	312.9094655	5	2	post_focus	1	Contrastive post_focus
2023410	block3	Control	pre	piu2	Subject	Narrow	r1	144.4663727	1	1	pre_focus	2	Narrow pre_focus
2023410	block3	Control	pre	tse2	Subject	Narrow	r1	98.62697083	2	2	pre_focus	2	Narrow pre_focus
2023410	block3	Control	pre	tsap1	Verb	Narrow	r1	88.40464699	3	1	on_focus	1	Narrow on_focus
2023410	block3	Control	pre	sy1	Object	Narrow	r1	114.4085221	4	1	post_focus	1	Narrow post_focus
2023410	block3	Control	pre	pau1	Object	Narrow	r1	356.7570315	5	2	post_focus	1	Narrow post_focus
2023410	block3	Control	pre	suk1	Subject	Contrastive	r1	59.12855883	1	1	pre_focus	1	Contrastive pre_focus
2023410	block3	Control	pre	suk1	Subject	Contrastive	r1	70.58827839	2	2	pre_focus	1	Contrastive pre_focus
2023410	block3	Control	pre	sei2	Verb	Contrastive	r1	118.122323	3	1	pre_focus	2	Contrastive pre_focus
2023410	block3	Control	pre	svy2	Object	Contrastive	r1	135.9758216	4	1	on_focus	2	Contrastive on_focus
2023410	block3	Control	pre	kwo2	Object	Contrastive	r1	279.232652	5	2	on_focus	2	Contrastive on_focus
2023410	block3	Control	pre	piu2	Subject	Broad	r1	116.9791568	1	1	broad_focus	2	Broad focus
2023410	block3	Control	pre	tse2	Subject	Broad	r1	91.73992417	2	2	broad_focus	2	Broad focus
2023410	block3	Control	pre	tsap1	Verb	Broad	r1	55.42591722	3	1	broad_focus	1	Broad focus
2023410	block3	Control	pre	sy1	Object	Broad	r1	99.89458185	4	1	broad_focus	1	Broad focus
2023410	block3	Control	pre	pau1	Object	Broad	r1	320.7172336	5	2	broad_focus	1	Broad focus
2023410	block3	Control	pre	piu2	Subject	Contrastive	r1	107.7076595	1	1	pre_focus	2	Contrastive pre_focus
2023410	block3	Control	pre	tse2	Subject	Contrastive	r1	117.5515166	2	2	pre_focus	2	Contrastive pre_focus
2023410	block3	Control	pre	tsap1	Verb	Contrastive	r1	26.90558449	3	1	pre_focus	1	Contrastive pre_focus
2023410	block3	Control	pre	sy1	Object	Contrastive	r1	93.87764793	4	1	on_focus	1	Contrastive on_focus
2023410	block3	Control	pre	pau1	Object	Contrastive	r1	312.7723596	5	2	on_focus	1	Contrastive on_focus
2023410	block3	Control	pre	piu2	Subject	Narrow	r1	95.4748637	1	1	pre_focus	2	Narrow pre_focus
2023410	block3	Control	pre	tse2	Subject	Narrow	r1	107.0693229	2	2	pre_focus	2	Narrow pre_focus
2023410	block3	Control	pre	tsap1	Verb	Narrow	r1	58.88752379	3	1	pre_focus	1	Narrow pre_focus
2023410	block3	Control	pre	sy1	Object	Narrow	r1	74.38720682	4	1	on_focus	1	Narrow on_focus
2023410	block3	Control	pre	pau1	Object	Narrow	r1	306.067698	5	2	on_focus	1	Narrow on_focus
2023410	block3	Control	pre	piu2	Subject	Narrow	r2	143.268828	1	1	pre_focus	2	Narrow pre_focus
2023410	block3	Control	pre	tse2	Subject	Narrow	r2	150.7005424	2	2	pre_focus	2	Narrow pre_focus
2023410	block3	Control	pre	tsap1	Verb	Narrow	r2	27.79847796	3	1	on_focus	1	Narrow on_focus
2023410	block3	Control	pre	sy1	Object	Narrow	r2	109.0127808	4	1	post_focus	1	Narrow post_focus
2023410	block3	Control	pre	pau1	Object	Narrow	r2	298.579707	5	2	post_focus	1	Narrow post_focus
2023410	block3	Control	pre	piu2	Subject	Broad	r2	139.8347929	1	1	broad_focus	2	Broad focus
2023410	block3	Control	pre	tse2	Subject	Broad	r2	115.9767468	2	2	broad_focus	2	Broad focus
2023410	block3	Control	pre	tsap1	Verb	Broad	r2	44.04116542	3	1	broad_focus	1	Broad focus
2023410	block3	Control	pre	sy1	Object	Broad	r2	100.8276198	4	1	broad_focus	1	Broad focus
2023410	block3	Control	pre	pau1	Object	Broad	r2	306.8537622	5	2	broad_focus	1	Broad focus
2023410	block3	Control	pre	piu2	Subject	Contrastive	r2	125.2999676	1	1	pre_focus	2	Contrastive pre_focus
2023410	block3	Control	pre	tse2	Subject	Contrastive	r2	109.9795114	2	2	pre_focus	2	Contrastive pre_focus
2023410	block3	Control	pre	tsap1	Verb	Contrastive	r2	45.49682445	3	1	on_focus	1	Contrastive on_focus
2023410	block3	Control	pre	sy1	Object	Contrastive	r2	85.0446379	4	1	post_focus	1	Contrastive post_focus
2023410	block3	Control	pre	pau1	Object	Contrastive	r2	289.5186054	5	2	post_focus	1	Contrastive post_focus
2023410	block3	Control	pre	piu2	Subject	Narrow	r2	155.8668316	1	1	pre_focus	2	Narrow pre_focus
2023410	block3	Control	pre	tse2	Subject	Narrow	r2	80.71296212	2	2	pre_focus	2	Narrow pre_focus
2023410	block3	Control	pre	tsap1	Verb	Narrow	r2	38.01620011	3	1	pre_focus	1	Narrow pre_focus
2023410	block3	Control	pre	sy1	Object	Narrow	r2	98.77912153	4	1	on_focus	1	Narrow on_focus
2023410	block3	Control	pre	pau1	Object	Narrow	r2	282.7198737	5	2	on_focus	1	Narrow on_focus
2023410	block3	Control	pre	suk1	Subject	Narrow	r2	50.88915742	1	1	pre_focus	1	Narrow pre_focus
2023410	block3	Control	pre	suk1	Subject	Narrow	r2	66.96844151	2	2	pre_focus	1	Narrow pre_focus
2023410	block3	Control	pre	sei2	Verb	Narrow	r2	109.8247258	3	1	on_focus	2	Narrow on_focus

2023410	block3	Control	pre	svy2	Object	Narrow	r2	113.6391783	4	1	post_focus	2	Narrow post_focus
2023410	block3	Control	pre	kwo2	Object	Narrow	r2	309.477357	5	2	post_focus	2	Narrow post_focus
2023410	block3	Control	pre	bui3	Subject	Narrow	r2	122.4575222	1	1	pre_focus	3	Narrow pre_focus
2023410	block3	Control	pre	bui3	Subject	Narrow	r2	113.7774538	2	2	pre_focus	3	Narrow pre_focus
2023410	block3	Control	pre	tsv1	Verb	Narrow	r2	118.2559461	3	1	on_focus	1	Narrow on_focus
2023410	block3	Control	pre	fug1	Object	Narrow	r2	176.0726029	4	1	post_focus	1	Narrow post_focus
2023410	block3	Control	pre	tshe1	Object	Narrow	r2	215.6919343	5	2	post_focus	1	Narrow post_focus
2023410	block3	Control	pre	piu2	Subject	Contrastive	r2	126.150953	1	1	pre_focus	2	Contrastive pre_focus
2023410	block3	Control	pre	tse2	Subject	Contrastive	r2	110.1073318	2	2	pre_focus	2	Contrastive pre_focus
2023410	block3	Control	pre	tsap1	Verb	Contrastive	r2	53.78037882	3	1	pre_focus	1	Contrastive pre_focus
2023410	block3	Control	pre	sy1	Object	Contrastive	r2	108.3457538	4	1	on_focus	1	Contrastive on_focus
2023410	block3	Control	pre	pau1	Object	Contrastive	r2	332.6130617	5	2	on_focus	1	Contrastive on_focus
2023410	block3	Control	pre	piu2	Subject	Contrastive	r2	139.606324	1	1	on_focus	2	Contrastive on_focus
2023410	block3	Control	pre	tse2	Subject	Contrastive	r2	117.7964853	2	2	on_focus	2	Contrastive on_focus
2023410	block3	Control	pre	tsap1	Verb	Contrastive	r2	36.4909936	3	1	post_focus	1	Contrastive post_focus
2023410	block3	Control	pre	sy1	Object	Contrastive	r2	104.6580319	4	1	post_focus	1	Contrastive post_focus
2023410	block3	Control	pre	pau1	Object	Contrastive	r2	300.0435604	5	2	post_focus	1	Contrastive post_focus
2023410	block3	Control	pre	suk1	Subject	Narrow	r2	52.16753486	1	1	pre_focus	1	Narrow pre_focus
2023410	block3	Control	pre	suk1	Subject	Narrow	r2	65.42232204	2	2	pre_focus	1	Narrow pre_focus
2023410	block3	Control	pre	sei2	Verb	Narrow	r2	117.8695171	3	1	pre_focus	2	Narrow pre_focus
2023410	block3	Control	pre	svy2	Object	Narrow	r2	115.2350371	4	1	on_focus	2	Narrow on_focus
2023410	block3	Control	pre	kwo2	Object	Narrow	r2	290.3232917	5	2	on_focus	2	Narrow on_focus
2023410	block3	Control	pre	bui3	Subject	Narrow	r2	125.7036539	1	1	on_focus	3	Narrow on_focus
2023410	block3	Control	pre	bui3	Subject	Narrow	r2	125.7441102	2	2	on_focus	3	Narrow on_focus
2023410	block3	Control	pre	tsv1	Verb	Narrow	r2	86.78543084	3	1	post_focus	1	Narrow post_focus
2023410	block3	Control	pre	fug1	Object	Narrow	r2	144.3996173	4	1	post_focus	1	Narrow post_focus
2023410	block3	Control	pre	tshe1	Object	Narrow	r2	266.8818837	5	2	post_focus	1	Narrow post_focus
2023410	block3	Control	pre	suk1	Subject	Contrastive	r2	72.48329861	1	1	pre_focus	1	Contrastive pre_focus
2023410	block3	Control	pre	suk1	Subject	Contrastive	r2	79.42833119	2	2	pre_focus	1	Contrastive pre_focus
2023410	block3	Control	pre	sei2	Verb	Contrastive	r2	125.0832917	3	1	on_focus	2	Contrastive on_focus
2023410	block3	Control	pre	svy2	Object	Contrastive	r2	133.7536097	4	1	post_focus	2	Contrastive post_focus
2023410	block3	Control	pre	kwo2	Object	Contrastive	r2	201.6782974	5	2	post_focus	2	Contrastive post_focus
2023410	block3	Control	pre	bui3	Subject	Contrastive	r2	104.7023204	1	1	pre_focus	3	Contrastive pre_focus
2023410	block3	Control	pre	bui3	Subject	Contrastive	r2	117.798698	2	2	pre_focus	3	Contrastive pre_focus
2023410	block3	Control	pre	tsv1	Verb	Contrastive	r2	94.46124775	3	1	on_focus	1	Contrastive on_focus
2023410	block3	Control	pre	fug1	Object	Contrastive	r2	152.6374452	4	1	post_focus	1	Contrastive post_focus
2023410	block3	Control	pre	tshe1	Object	Contrastive	r2	214.9317318	5	2	post_focus	1	Contrastive post_focus
2023410	block3	Control	pre	suk1	Subject	Broad	r2	65.77380811	1	1	broad_focus	1	Broad focus
2023410	block3	Control	pre	suk1	Subject	Broad	r2	79.17847959	2	2	broad_focus	1	Broad focus
2023410	block3	Control	pre	sei2	Verb	Broad	r2	125.1374728	3	1	broad_focus	2	Broad focus
2023410	block3	Control	pre	svy2	Object	Broad	r2	130.869124	4	1	broad_focus	2	Broad focus
2023410	block3	Control	pre	kwo2	Object	Broad	r2	295.0250235	5	2	broad_focus	2	Broad focus
2023410	block3	Control	pre	bui3	Subject	Contrastive	r2	130.457294	1	1	on_focus	3	Contrastive on_focus
2023410	block3	Control	pre	bui3	Subject	Contrastive	r2	235.4494639	2	2	on_focus	3	Contrastive on_focus
2023410	block3	Control	pre	tsv1	Verb	Contrastive	r2	114.3359821	3	1	post_focus	1	Contrastive post_focus
2023410	block3	Control	pre	fug1	Object	Contrastive	r2	156.1715361	4	1	post_focus	1	Contrastive post_focus
2023410	block3	Control	pre	tshe1	Object	Contrastive	r2	312.2211264	5	2	post_focus	1	Contrastive post_focus
2023410	block3	Control	pre	bui3	Subject	Contrastive	r2	125.4033568	1	1	pre_focus	3	Contrastive pre_focus
2023410	block3	Control	pre	bui3	Subject	Contrastive	r2	89.73036643	2	2	pre_focus	3	Contrastive pre_focus
2023410	block3	Control	pre	tsv1	Verb	Contrastive	r2	90.03198131	3	1	pre_focus	1	Contrastive pre_focus
2023410	block3	Control	pre	fug1	Object	Contrastive	r2	152.6547237	4	1	on_focus	1	Contrastive on_focus
2023410	block3	Control	pre	tshe1	Object	Contrastive	r2	269.8455425	5	2	on_focus	1	Contrastive on_focus
2023410	block3	Control	pre	piu2	Subject	Narrow	r2	134.1892382	1	1	on_focus	2	Narrow on_focus
2023410	block3	Control	pre	tse2	Subject	Narrow	r2	120.7433095	2	2	on_focus	2	Narrow on_focus
2023410	block3	Control	pre	tsap1	Verb	Narrow	r2	28.778233	3	1	post_focus	1	Narrow post_focus
2023410	block3	Control	pre	sy1	Object	Narrow	r2	82.62516647	4	1	post_focus	1	Narrow post_focus
2023410	block3	Control	pre	pau1	Object	Narrow	r2	264.9072526	5	2	post_focus	1	Narrow post_focus
2023410	block3	Control	pre	bui3	Subject	Narrow	r2	132.6335605	1	1	pre_focus	3	Narrow pre_focus
2023410	block3	Control	pre	bui3	Subject	Narrow	r2	113.3500252	2	2	pre_focus	3	Narrow pre_focus
2023410	block3	Control	pre	tsv1	Verb	Narrow	r2	102.3685347	3	1	pre_focus	1	Narrow pre_focus
2023410	block3	Control	pre	fug1	Object	Narrow	r2	143.4842882	4	1	on_focus	1	Narrow on_focus
2023410	block3	Control	pre	tshe1	Object	Narrow	r2	253.4614053	5	2	on_focus	1	Narrow on_focus
2023410	block3	Control	pre	suk1	Subject	Narrow	r2	70.34975378	1	1	on_focus	1	Narrow on_focus
2023410	block3	Control	pre	suk1	Subject	Narrow	r2	77.61344098	2	2	on_focus	1	Narrow on_focus
2023410	block3	Control	pre	sei2	Verb	Narrow	r2	109.9558429	3	1	post_focus	2	Narrow post_focus
2023410	block3	Control	pre	svy2	Object	Narrow	r2	132.7693615	4	1	post_focus	2	Narrow post_focus

2023410	block3	Control	pre	kwo2	Object	Narrow	r2	255.6205225	5	2	post_focus	2	Narrow post_focus
2023410	block3	Control	pre	bui3	Subject	Broad	r2	117.2750218	1	1	broad_focus	3	Broad focus
2023410	block3	Control	pre	bui3	Subject	Broad	r2	126.186712	2	2	broad_focus	3	Broad focus
2023410	block3	Control	pre	tsv1	Verb	Broad	r2	101.6098152	3	1	broad_focus	1	Broad focus
2023410	block3	Control	pre	fug1	Object	Broad	r2	121.9156904	4	1	broad_focus	1	Broad focus
2023410	block3	Control	pre	tshe1	Object	Broad	r2	251.8378721	5	2	broad_focus	1	Broad focus
2023410	block3	Control	pre	suk1	Subject	Contrastive	r2	71.80550034	1	1	on_focus	1	Contrastive on_focus
2023410	block3	Control	pre	suk1	Subject	Contrastive	r2	71.17797546	2	2	on_focus	1	Contrastive on_focus
2023410	block3	Control	pre	sei2	Verb	Contrastive	r2	129.4365889	3	1	post_focus	2	Contrastive post_focus
2023410	block3	Control	pre	svy2	Object	Contrastive	r2	130.7017152	4	1	post_focus	2	Contrastive post_focus
2023410	block3	Control	pre	kwo2	Object	Contrastive	r2	284.084872	5	2	post_focus	2	Contrastive post_focus
2023410	block3	Control	pre	suk1	Subject	Contrastive	r2	62.35636577	1	1	pre_focus	1	Contrastive pre_focus
2023410	block3	Control	pre	suk1	Subject	Contrastive	r2	71.33910534	2	2	pre_focus	1	Contrastive pre_focus
2023410	block3	Control	pre	sei2	Verb	Contrastive	r2	112.2152047	3	1	pre_focus	2	Contrastive pre_focus
2023410	block3	Control	pre	svy2	Object	Contrastive	r2	113.4182522	4	1	on_focus	2	Contrastive on_focus
2023410	block3	Control	pre	kwo2	Object	Contrastive	r2	254.0428449	5	2	on_focus	2	Contrastive on_focus
2023410	block4	Control	post	Jan-01	Subject	Broad	r1	301.2422999	1	1	broad_focus	1	Broad focus
2023410	block4	Control	post	Jan-01	Subject	Broad	r1	232.6785414	2	2	broad_focus	1	Broad focus
2023410	block4	Control	post	wei3	Verb	Broad	r1	161.8263416	3	1	broad_focus	3	Broad focus
2023410	block4	Control	post	tsam3	Object	Broad	r1	194.942254	4	1	broad_focus	3	Broad focus
2023410	block4	Control	post	tsam3	Object	Broad	r1	471.8764432	5	2	broad_focus	3	Broad focus
2023410	block4	Control	post	pak3	Subject	Narrow	r1	105.8650213	1	1	pre_focus	3	Narrow pre_focus
2023410	block4	Control	post	pak3	Subject	Narrow	r1	87.60856617	2	2	pre_focus	3	Narrow pre_focus
2023410	block4	Control	post	tsing2	Verb	Narrow	r1	143.2288218	3	1	pre_focus	2	Narrow pre_focus
2023410	block4	Control	post	kau2	Object	Narrow	r1	189.8477471	4	1	on_focus	2	Narrow on_focus
2023410	block4	Control	post	tsi2	Object	Narrow	r1	361.4669751	5	2	on_focus	2	Narrow on_focus
2023410	block4	Control	post	Jan-01	Subject	Contrastive	r1	237.0377696	1	1	pre_focus	1	Contrastive pre_focus
2023410	block4	Control	post	Jan-01	Subject	Contrastive	r1	323.6564838	2	2	pre_focus	1	Contrastive pre_focus
2023410	block4	Control	post	wei3	Verb	Contrastive	r1	193.1876983	3	1	on_focus	3	Contrastive on_focus
2023410	block4	Control	post	tsam3	Object	Contrastive	r1	202.2361919	4	1	post_focus	3	Contrastive post_focus
2023410	block4	Control	post	tsam3	Object	Contrastive	r1	372.0679652	5	2	post_focus	3	Contrastive post_focus
2023410	block4	Control	post	Jan-01	Subject	Contrastive	r1	264.155464	1	1	on_focus	1	Contrastive on_focus
2023410	block4	Control	post	Jan-01	Subject	Contrastive	r1	254.1720739	2	2	on_focus	1	Contrastive on_focus
2023410	block4	Control	post	wei3	Verb	Contrastive	r1	172.7895466	3	1	post_focus	3	Contrastive post_focus
2023410	block4	Control	post	tsam3	Object	Contrastive	r1	214.3409681	4	1	post_focus	3	Contrastive post_focus
2023410	block4	Control	post	tsam3	Object	Contrastive	r1	339.0316778	5	2	post_focus	3	Contrastive post_focus
2023410	block4	Control	post	Jan-01	Subject	Narrow	r1	244.663317	1	1	pre_focus	1	Narrow pre_focus
2023410	block4	Control	post	Jan-01	Subject	Narrow	r1	213.3720679	2	2	pre_focus	1	Narrow pre_focus
2023410	block4	Control	post	wei3	Verb	Narrow	r1	130.5029446	3	1	pre_focus	3	Narrow pre_focus
2023410	block4	Control	post	tsam3	Object	Narrow	r1	175.0920603	4	1	on_focus	3	Narrow on_focus
2023410	block4	Control	post	tsam3	Object	Narrow	r1	351.5228058	5	2	on_focus	3	Narrow on_focus
2023410	block4	Control	post	piu35	Subject	Narrow	r1	128.7761924	1	1	on_focus	35	Narrow on_focus
2023410	block4	Control	post	mui35	Subject	Narrow	r1	251.3535994	2	2	on_focus	35	Narrow on_focus
2023410	block4	Control	post	tsan3	Verb	Narrow	r1	215.6423483	3	1	post_focus	3	Narrow post_focus
2023410	block4	Control	post	jin3	Object	Narrow	r1	224.6787669	4	1	post_focus	3	Narrow post_focus
2023410	block4	Control	post	jin3	Object	Narrow	r1	391.3311755	5	2	post_focus	3	Narrow post_focus
2023410	block4	Control	post	piu35	Subject	Narrow	r1	124.3835559	1	1	pre_focus	35	Narrow pre_focus
2023410	block4	Control	post	mui35	Subject	Narrow	r1	256.4042819	2	2	pre_focus	35	Narrow pre_focus
2023410	block4	Control	post	tsan3	Verb	Narrow	r1	204.1879979	3	1	on_focus	3	Narrow on_focus
2023410	block4	Control	post	jin3	Object	Narrow	r1	230.8738347	4	1	post_focus	3	Narrow post_focus
2023410	block4	Control	post	jin3	Object	Narrow	r1	338.0087995	5	2	post_focus	3	Narrow post_focus
2023410	block4	Control	post	piu35	Subject	Broad	r1	152.6391357	1	1	broad_focus	35	Broad focus
2023410	block4	Control	post	mui35	Subject	Broad	r1	255.3918369	2	2	broad_focus	35	Broad focus
2023410	block4	Control	post	tsan3	Verb	Broad	r1	203.7535356	3	1	broad_focus	3	Broad focus
2023410	block4	Control	post	jin3	Object	Broad	r1	236.5780199	4	1	broad_focus	3	Broad focus
2023410	block4	Control	post	jin3	Object	Broad	r1	352.1773113	5	2	broad_focus	3	Broad focus
2023410	block4	Control	post	pak3	Subject	Broad	r1	125.5291945	1	1	broad_focus	3	Broad focus
2023410	block4	Control	post	pak3	Subject	Broad	r1	121.113056	2	2	broad_focus	3	Broad focus
2023410	block4	Control	post	tsing2	Verb	Broad	r1	173.1006428	3	1	broad_focus	2	Broad focus
2023410	block4	Control	post	kau2	Object	Broad	r1	187.7301284	4	1	broad_focus	2	Broad focus
2023410	block4	Control	post	tsi2	Object	Broad	r1	330.389986	5	2	broad_focus	2	Broad focus
2023410	block4	Control	post	Jan-01	Subject	Narrow	r1	201.2157237	1	1	on_focus	1	Narrow on_focus
2023410	block4	Control	post	Jan-01	Subject	Narrow	r1	244.9839276	2	2	on_focus	1	Narrow on_focus
2023410	block4	Control	post	wei3	Verb	Narrow	r1	148.7937905	3	1	post_focus	3	Narrow post_focus
2023410	block4	Control	post	tsam3	Object	Narrow	r1	150.5642537	4	1	post_focus	3	Narrow post_focus
2023410	block4	Control	post	tsam3	Object	Narrow	r1	367.7650415	5	2	post_focus	3	Narrow post_focus

2023410	block4	Control	post	piu35	Subject	Contrastive	r1	119.2119705	1	1	pre_focus	35	Contrastive pre_focus
2023410	block4	Control	post	mui35	Subject	Contrastive	r1	262.6890618	2	2	pre_focus	35	Contrastive pre_focus
2023410	block4	Control	post	tsan3	Verb	Contrastive	r1	209.6557515	3	1	on_focus	3	Contrastive on_focus
2023410	block4	Control	post	jin3	Object	Contrastive	r1	249.722829	4	1	post_focus	3	Contrastive post_focus
2023410	block4	Control	post	jin3	Object	Contrastive	r1	299.6931437	5	2	post_focus	3	Contrastive post_focus
2023410	block4	Control	post	piu35	Subject	Contrastive	r1	216.500013	1	1	pre_focus	35	Contrastive pre_focus
2023410	block4	Control	post	mui35	Subject	Contrastive	r1	191.3716549	2	2	pre_focus	35	Contrastive pre_focus
2023410	block4	Control	post	tsan3	Verb	Contrastive	r1	219.5983703	3	1	pre_focus	3	Contrastive pre_focus
2023410	block4	Control	post	jin3	Object	Contrastive	r1	241.679765	4	1	on_focus	3	Contrastive on_focus
2023410	block4	Control	post	jin3	Object	Contrastive	r1	366.4451819	5	2	on_focus	3	Contrastive on_focus
2023410	block4	Control	post	Jan-01	Subject	Contrastive	r1	254.763706	1	1	pre_focus	1	Contrastive pre_focus
2023410	block4	Control	post	Jan-01	Subject	Contrastive	r1	343.597563	2	2	pre_focus	1	Contrastive pre_focus
2023410	block4	Control	post	wei3	Verb	Contrastive	r1	552.6865689	3	1	pre_focus	3	Contrastive pre_focus
2023410	block4	Control	post	tsam3	Object	Contrastive	r1	226.8949537	4	1	on_focus	3	Contrastive on_focus
2023410	block4	Control	post	tsam3	Object	Contrastive	r1	426.1120919	5	2	on_focus	3	Contrastive on_focus
2023410	block4	Control	post	pak3	Subject	Contrastive	r1	106.5001751	1	1	pre_focus	3	Contrastive pre_focus
2023410	block4	Control	post	pak3	Subject	Contrastive	r1	140.8507547	2	2	pre_focus	3	Contrastive pre_focus
2023410	block4	Control	post	tsing2	Verb	Contrastive	r1	157.4830415	3	1	pre_focus	2	Contrastive pre_focus
2023410	block4	Control	post	kau2	Object	Contrastive	r1	215.9243567	4	1	on_focus	2	Contrastive on_focus
2023410	block4	Control	post	tsi2	Object	Contrastive	r1	265.608621	5	2	on_focus	2	Contrastive on_focus
2023410	block4	Control	post	pak3	Subject	Narrow	r1	89.62137679	1	1	on_focus	3	Narrow on_focus
2023410	block4	Control	post	pak3	Subject	Narrow	r1	124.9057376	2	2	on_focus	3	Narrow on_focus
2023410	block4	Control	post	tsing2	Verb	Narrow	r1	153.9064731	3	1	post_focus	2	Narrow post_focus
2023410	block4	Control	post	kau2	Object	Narrow	r1	208.8428309	4	1	post_focus	2	Narrow post_focus
2023410	block4	Control	post	tsi2	Object	Narrow	r1	262.564446	5	2	post_focus	2	Narrow post_focus
2023410	block4	Control	post	piu35	Subject	Narrow	r1	227.3969854	1	1	pre_focus	35	Narrow pre_focus
2023410	block4	Control	post	mui35	Subject	Narrow	r1	203.0178681	2	2	pre_focus	35	Narrow pre_focus
2023410	block4	Control	post	tsan3	Verb	Narrow	r1	227.3830096	3	1	pre_focus	3	Narrow pre_focus
2023410	block4	Control	post	jin3	Object	Narrow	r1	279.1033136	4	1	on_focus	3	Narrow on_focus
2023410	block4	Control	post	jin3	Object	Narrow	r1	464.7287607	5	2	on_focus	3	Narrow on_focus
2023410	block4	Control	post	pak3	Subject	Contrastive	r1	107.1318838	1	1	pre_focus	3	Contrastive pre_focus
2023410	block4	Control	post	pak3	Subject	Contrastive	r1	122.6329783	2	2	pre_focus	3	Contrastive pre_focus
2023410	block4	Control	post	tsing2	Verb	Contrastive	r1	200.7334135	3	1	on_focus	2	Contrastive on_focus
2023410	block4	Control	post	kau2	Object	Contrastive	r1	217.3731491	4	1	post_focus	2	Contrastive post_focus
2023410	block4	Control	post	tsi2	Object	Contrastive	r1	346.777066	5	2	post_focus	2	Contrastive post_focus
2023410	block4	Control	post	Jan-01	Subject	Narrow	r1	174.7191331	1	1	pre_focus	1	Narrow pre_focus
2023410	block4	Control	post	Jan-01	Subject	Narrow	r1	205.3565973	2	2	pre_focus	1	Narrow pre_focus
2023410	block4	Control	post	wei3	Verb	Narrow	r1	125.8870775	3	1	on_focus	3	Narrow on_focus
2023410	block4	Control	post	tsam3	Object	Narrow	r1	133.4364723	4	1	post_focus	3	Narrow post_focus
2023410	block4	Control	post	tsam3	Object	Narrow	r1	330.1063789	5	2	post_focus	3	Narrow post_focus
2023410	block4	Control	post	pak3	Subject	Contrastive	r1	62.02243861	1	1	on_focus	3	Contrastive on_focus
2023410	block4	Control	post	pak3	Subject	Contrastive	r1	90.31464849	2	2	on_focus	3	Contrastive on_focus
2023410	block4	Control	post	tsing2	Verb	Contrastive	r1	134.2937651	3	1	post_focus	2	Contrastive post_focus
2023410	block4	Control	post	kau2	Object	Contrastive	r1	168.4412046	4	1	post_focus	2	Contrastive post_focus
2023410	block4	Control	post	tsi2	Object	Contrastive	r1	359.4041096	5	2	post_focus	2	Contrastive post_focus
2023410	block4	Control	post	piu35	Subject	Contrastive	r1	147.7182492	1	1	on_focus	35	Contrastive on_focus
2023410	block4	Control	post	mui35	Subject	Contrastive	r1	195.5226159	2	2	on_focus	35	Contrastive on_focus
2023410	block4	Control	post	tsan3	Verb	Contrastive	r1	200.9697215	3	1	post_focus	3	Contrastive post_focus
2023410	block4	Control	post	jin3	Object	Contrastive	r1	239.0938001	4	1	post_focus	3	Contrastive post_focus
2023410	block4	Control	post	jin3	Object	Contrastive	r1	393.1810875	5	2	post_focus	3	Contrastive post_focus
2023410	block4	Control	post	pak3	Subject	Narrow	r1	92.22322669	1	1	pre_focus	3	Narrow pre_focus
2023410	block4	Control	post	pak3	Subject	Narrow	r1	110.032627	2	2	pre_focus	3	Narrow pre_focus
2023410	block4	Control	post	tsing2	Verb	Narrow	r1	169.9925432	3	1	on_focus	2	Narrow on_focus
2023410	block4	Control	post	kau2	Object	Narrow	r1	190.5656355	4	1	post_focus	2	Narrow post_focus
2023410	block4	Control	post	tsi2	Object	Narrow	r1	306.8858045	5	2	post_focus	2	Narrow post_focus
2023410	block4	Control	post	piu35	Subject	Broad	r2	186.9129059	1	1	broad_focus	35	Broad focus
2023410	block4	Control	post	mui35	Subject	Broad	r2	197.6549476	2	2	broad_focus	35	Broad focus
2023410	block4	Control	post	tsan3	Verb	Broad	r2	212.0225339	3	1	broad_focus	3	Broad focus
2023410	block4	Control	post	jin3	Object	Broad	r2	195.9168232	4	1	broad_focus	3	Broad focus
2023410	block4	Control	post	jin3	Object	Broad	r2	431.7697162	5	2	broad_focus	3	Broad focus
2023410	block4	Control	post	pak3	Subject	Narrow	r2	95.5377812	1	1	pre_focus	3	Narrow pre_focus
2023410	block4	Control	post	pak3	Subject	Narrow	r2	102.2982791	2	2	pre_focus	3	Narrow pre_focus
2023410	block4	Control	post	tsing2	Verb	Narrow	r2	176.453791	3	1	on_focus	2	Narrow on_focus
2023410	block4	Control	post	kau2	Object	Narrow	r2	139.7060861	4	1	post_focus	2	Narrow post_focus
2023410	block4	Control	post	tsi2	Object	Narrow	r2	317.4268406	5	2	post_focus	2	Narrow post_focus
2023410	block4	Control	post	Jan-01	Subject	Contrastive	r2	216.9294926	1	1	on_focus	1	Contrastive on_focus

2023410	block4	Control	post	Jan-01	Subject	Contrastive	r2	182.9306446	2	2	on_focus	1	Contrastive on_focus
2023410	block4	Control	post	wei3	Verb	Contrastive	r2	142.0106293	3	1	post_focus	3	Contrastive post_focus
2023410	block4	Control	post	tsam3	Object	Contrastive	r2	200.6407375	4	1	post_focus	3	Contrastive post_focus
2023410	block4	Control	post	tsam3	Object	Contrastive	r2	217.1952497	5	2	post_focus	3	Contrastive post_focus
2023410	block4	Control	post	piu35	Subject	Contrastive	r2	147.0033058	1	1	pre_focus	35	Contrastive pre_focus
2023410	block4	Control	post	mui35	Subject	Contrastive	r2	216.0172002	2	2	pre_focus	35	Contrastive pre_focus
2023410	block4	Control	post	tsan3	Verb	Contrastive	r2	187.5192207	3	1	on_focus	3	Contrastive on_focus
2023410	block4	Control	post	jin3	Object	Contrastive	r2	234.2773149	4	1	post_focus	3	Contrastive post_focus
2023410	block4	Control	post	jin3	Object	Contrastive	r2	329.9032502	5	2	post_focus	3	Contrastive post_focus
2023410	block4	Control	post	pak3	Subject	Contrastive	r2	116.343759	1	1	pre_focus	3	Contrastive pre_focus
2023410	block4	Control	post	pak3	Subject	Contrastive	r2	87.01907703	2	2	pre_focus	3	Contrastive pre_focus
2023410	block4	Control	post	tsing2	Verb	Contrastive	r2	144.7275636	3	1	pre_focus	2	Contrastive pre_focus
2023410	block4	Control	post	kau2	Object	Contrastive	r2	193.2343834	4	1	on_focus	2	Contrastive on_focus
2023410	block4	Control	post	tsi2	Object	Contrastive	r2	317.7989104	5	2	on_focus	2	Contrastive on_focus
2023410	block4	Control	post	Jan-01	Subject	Contrastive	r2	292.020547	1	1	pre_focus	1	Contrastive pre_focus
2023410	block4	Control	post	Jan-01	Subject	Contrastive	r2	239.1942218	2	2	pre_focus	1	Contrastive pre_focus
2023410	block4	Control	post	wei3	Verb	Contrastive	r2	159.8072929	3	1	pre_focus	3	Contrastive pre_focus
2023410	block4	Control	post	tsam3	Object	Contrastive	r2	201.0120295	4	1	on_focus	3	Contrastive on_focus
2023410	block4	Control	post	tsam3	Object	Contrastive	r2	386.0590189	5	2	on_focus	3	Contrastive on_focus
2023410	block4	Control	post	Jan-01	Subject	Narrow	r2	238.9235419	1	1	pre_focus	1	Narrow pre_focus
2023410	block4	Control	post	Jan-01	Subject	Narrow	r2	232.7014266	2	2	pre_focus	1	Narrow pre_focus
2023410	block4	Control	post	wei3	Verb	Narrow	r2	135.7906043	3	1	on_focus	3	Narrow on_focus
2023410	block4	Control	post	tsam3	Object	Narrow	r2	156.6793221	4	1	post_focus	3	Narrow post_focus
2023410	block4	Control	post	tsam3	Object	Narrow	r2	329.9792949	5	2	post_focus	3	Narrow post_focus
2023410	block4	Control	post	pak3	Subject	Narrow	r2	84.14391804	1	1	pre_focus	3	Narrow pre_focus
2023410	block4	Control	post	pak3	Subject	Narrow	r2	83.27481074	2	2	pre_focus	3	Narrow pre_focus
2023410	block4	Control	post	tsing2	Verb	Narrow	r2	160.6119081	3	1	pre_focus	2	Narrow pre_focus
2023410	block4	Control	post	kau2	Object	Narrow	r2	157.7767352	4	1	on_focus	2	Narrow on_focus
2023410	block4	Control	post	tsi2	Object	Narrow	r2	223.7023324	5	2	on_focus	2	Narrow on_focus
2023410	block4	Control	post	piu35	Subject	Narrow	r2	97.05062043	1	1	pre_focus	35	Narrow pre_focus
2023410	block4	Control	post	mui35	Subject	Narrow	r2	212.7502513	2	2	pre_focus	35	Narrow pre_focus
2023410	block4	Control	post	tsan3	Verb	Narrow	r2	208.2599953	3	1	pre_focus	3	Narrow pre_focus
2023410	block4	Control	post	jin3	Object	Narrow	r2	202.7705244	4	1	on_focus	3	Narrow on_focus
2023410	block4	Control	post	jin3	Object	Narrow	r2	403.5315098	5	2	on_focus	3	Narrow on_focus
2023410	block4	Control	post	Jan-01	Subject	Narrow	r2	204.320217	1	1	on_focus	1	Narrow on_focus
2023410	block4	Control	post	Jan-01	Subject	Narrow	r2	188.6339831	2	2	on_focus	1	Narrow on_focus
2023410	block4	Control	post	wei3	Verb	Narrow	r2	140.9364083	3	1	post_focus	3	Narrow post_focus
2023410	block4	Control	post	tsam3	Object	Narrow	r2	198.8425616	4	1	post_focus	3	Narrow post_focus
2023410	block4	Control	post	tsam3	Object	Narrow	r2	368.9312692	5	2	post_focus	3	Narrow post_focus
2023410	block4	Control	post	pak3	Subject	Contrastive	r2	144.6503142	1	1	pre_focus	3	Contrastive pre_focus
2023410	block4	Control	post	pak3	Subject	Contrastive	r2	101.4256154	2	2	pre_focus	3	Contrastive pre_focus
2023410	block4	Control	post	tsing2	Verb	Contrastive	r2	201.1213026	3	1	on_focus	2	Contrastive on_focus
2023410	block4	Control	post	kau2	Object	Contrastive	r2	211.4292264	4	1	post_focus	2	Contrastive post_focus
2023410	block4	Control	post	tsi2	Object	Contrastive	r2	287.4347733	5	2	post_focus	2	Contrastive post_focus
2023410	block4	Control	post	piu35	Subject	Narrow	r2	137.4710122	1	1	pre_focus	35	Narrow pre_focus
2023410	block4	Control	post	mui35	Subject	Narrow	r2	215.9267707	2	2	pre_focus	35	Narrow pre_focus
2023410	block4	Control	post	tsan3	Verb	Narrow	r2	220.8836538	3	1	on_focus	3	Narrow on_focus
2023410	block4	Control	post	jin3	Object	Narrow	r2	217.9400378	4	1	post_focus	3	Narrow post_focus
2023410	block4	Control	post	jin3	Object	Narrow	r2	312.5449492	5	2	post_focus	3	Narrow post_focus
2023410	block4	Control	post	pak3	Subject	Broad	r2	93.24988371	1	1	broad_focus	3	Broad focus
2023410	block4	Control	post	pak3	Subject	Broad	r2	114.1309826	2	2	broad_focus	3	Broad focus
2023410	block4	Control	post	tsing2	Verb	Broad	r2	176.7334958	3	1	broad_focus	2	Broad focus
2023410	block4	Control	post	kau2	Object	Broad	r2	148.41112	4	1	broad_focus	2	Broad focus
2023410	block4	Control	post	tsi2	Object	Broad	r2	273.236996	5	2	broad_focus	2	Broad focus
2023410	block4	Control	post	piu35	Subject	Contrastive	r2	133.9615966	1	1	pre_focus	35	Contrastive pre_focus
2023410	block4	Control	post	mui35	Subject	Contrastive	r2	250.3200505	2	2	pre_focus	35	Contrastive pre_focus
2023410	block4	Control	post	tsan3	Verb	Contrastive	r2	222.6363415	3	1	pre_focus	3	Contrastive pre_focus
2023410	block4	Control	post	jin3	Object	Contrastive	r2	218.4954865	4	1	on_focus	3	Contrastive on_focus
2023410	block4	Control	post	jin3	Object	Contrastive	r2	368.8206021	5	2	on_focus	3	Contrastive on_focus
2023410	block4	Control	post	piu35	Subject	Narrow	r2	121.2171558	1	1	on_focus	35	Narrow on_focus
2023410	block4	Control	post	mui35	Subject	Narrow	r2	207.8157574	2	2	on_focus	35	Narrow on_focus
2023410	block4	Control	post	tsan3	Verb	Narrow	r2	212.9020304	3	1	post_focus	3	Narrow post_focus
2023410	block4	Control	post	jin3	Object	Narrow	r2	202.3487575	4	1	post_focus	3	Narrow post_focus
2023410	block4	Control	post	jin3	Object	Narrow	r2	333.7293341	5	2	post_focus	3	Narrow post_focus
2023410	block4	Control	post	piu35	Subject	Contrastive	r2	117.8813938	1	1	on_focus	35	Contrastive on_focus
2023410	block4	Control	post	mui35	Subject	Contrastive	r2	220.6984656	2	2	on_focus	35	Contrastive on_focus

2023410	block4	Control	post	tsan3	Verb	Contrastive	r2	193.1959967	3	1	post_focus	3	Contrastive post_focus
2023410	block4	Control	post	jin3	Object	Contrastive	r2	195.2639213	4	1	post_focus	3	Contrastive post_focus
2023410	block4	Control	post	jin3	Object	Contrastive	r2	356.3453295	5	2	post_focus	3	Contrastive post_focus
2023410	block4	Control	post	Jan-01	Subject	Narrow	r2	278.2934838	1	1	pre_focus	1	Narrow pre_focus
2023410	block4	Control	post	Jan-01	Subject	Narrow	r2	359.2079918	2	2	pre_focus	1	Narrow pre_focus
2023410	block4	Control	post	wei3	Verb	Narrow	r2	180.7950159	3	1	pre_focus	3	Narrow pre_focus
2023410	block4	Control	post	tsam3	Object	Narrow	r2	193.3929027	4	1	on_focus	3	Narrow on_focus
2023410	block4	Control	post	tsam3	Object	Narrow	r2	341.6545285	5	2	on_focus	3	Narrow on_focus
2023410	block4	Control	post	Jan-01	Subject	Contrastive	r2	224.8628113	1	1	pre_focus	1	Contrastive pre_focus
2023410	block4	Control	post	Jan-01	Subject	Contrastive	r2	223.4684889	2	2	pre_focus	1	Contrastive pre_focus
2023410	block4	Control	post	wei3	Verb	Contrastive	r2	133.7050415	3	1	on_focus	3	Contrastive on_focus
2023410	block4	Control	post	tsam3	Object	Contrastive	r2	189.6914758	4	1	post_focus	3	Contrastive post_focus
2023410	block4	Control	post	tsam3	Object	Contrastive	r2	351.8644663	5	2	post_focus	3	Contrastive post_focus
2023410	block4	Control	post	pak3	Subject	Narrow	r2	86.59658775	1	1	on_focus	3	Narrow on_focus
2023410	block4	Control	post	pak3	Subject	Narrow	r2	110.6932521	2	2	on_focus	3	Narrow on_focus
2023410	block4	Control	post	tsing2	Verb	Narrow	r2	139.622795	3	1	post_focus	2	Narrow post_focus
2023410	block4	Control	post	kau2	Object	Narrow	r2	164.6370563	4	1	post_focus	2	Narrow post_focus
2023410	block4	Control	post	tsi2	Object	Narrow	r2	268.898799	5	2	post_focus	2	Narrow post_focus
2023410	block4	Control	post	Jan-01	Subject	Broad	r2	211.2068836	1	1	broad_focus	1	Broad focus
2023410	block4	Control	post	Jan-01	Subject	Broad	r2	188.4080904	2	2	broad_focus	1	Broad focus
2023410	block4	Control	post	wei3	Verb	Broad	r2	163.9611484	3	1	broad_focus	3	Broad focus
2023410	block4	Control	post	tsam3	Object	Broad	r2	184.4152482	4	1	broad_focus	3	Broad focus
2023410	block4	Control	post	tsam3	Object	Broad	r2	305.112994	5	2	broad_focus	3	Broad focus
2023410	block4	Control	post	pak3	Subject	Contrastive	r2	107.6101777	1	1	on_focus	3	Contrastive on_focus
2023410	block4	Control	post	pak3	Subject	Contrastive	r2	85.77642836	2	2	on_focus	3	Contrastive on_focus
2023410	block4	Control	post	tsing2	Verb	Contrastive	r2	131.6574851	3	1	post_focus	2	Contrastive post_focus
2023410	block4	Control	post	kau2	Object	Contrastive	r2	188.0234366	4	1	post_focus	2	Contrastive post_focus
2023410	block4	Control	post	tsi2	Object	Contrastive	r2	250.4780901	5	2	post_focus	2	Contrastive post_focus
2023410	block4	Control	pre	pak3	Subject	Contrastive	r1	79.23774638	1	1	pre_focus	3	Contrastive pre_focus
2023410	block4	Control	pre	pak3	Subject	Contrastive	r1	93.73015873	2	2	pre_focus	3	Contrastive pre_focus
2023410	block4	Control	pre	tsing2	Verb	Contrastive	r1	217.2380952	3	1	pre_focus	2	Contrastive pre_focus
2023410	block4	Control	pre	kau2	Object	Contrastive	r1	187.9933084	4	1	on_focus	2	Contrastive on_focus
2023410	block4	Control	pre	tsi2	Object	Contrastive	r1	351.6940837	5	2	on_focus	2	Contrastive on_focus
2023410	block4	Control	pre	piu35	Subject	Contrastive	r1	132.4599125	1	1	pre_focus	35	Contrastive pre_focus
2023410	block4	Control	pre	mui35	Subject	Contrastive	r1	196.4977615	2	2	pre_focus	35	Contrastive pre_focus
2023410	block4	Control	pre	tsan3	Verb	Contrastive	r1	173.9153439	3	1	pre_focus	3	Contrastive pre_focus
2023410	block4	Control	pre	jin3	Object	Contrastive	r1	244.2495728	4	1	on_focus	3	Contrastive on_focus
2023410	block4	Control	pre	jin3	Object	Contrastive	r1	356.2282691	5	2	on_focus	3	Contrastive on_focus
2023410	block4	Control	pre	Jan-01	Subject	Contrastive	r1	219.1836735	1	1	pre_focus	1	Contrastive pre_focus
2023410	block4	Control	pre	Jan-01	Subject	Contrastive	r1	200.4064836	2	2	pre_focus	1	Contrastive pre_focus
2023410	block4	Control	pre	wei3	Verb	Contrastive	r1	139.569161	3	1	pre_focus	3	Contrastive pre_focus
2023410	block4	Control	pre	tsam3	Object	Contrastive	r1	173.5621521	4	1	on_focus	3	Contrastive on_focus
2023410	block4	Control	pre	tsam3	Object	Contrastive	r1	365.6175359	5	2	on_focus	3	Contrastive on_focus
2023410	block4	Control	pre	Jan-01	Subject	Narrow	r1	186.7602041	1	1	pre_focus	1	Narrow pre_focus
2023410	block4	Control	pre	Jan-01	Subject	Narrow	r1	188.7850811	2	2	pre_focus	1	Narrow pre_focus
2023410	block4	Control	pre	wei3	Verb	Narrow	r1	145.2189468	3	1	pre_focus	3	Narrow pre_focus
2023410	block4	Control	pre	tsam3	Object	Narrow	r1	160.2862812	4	1	on_focus	3	Narrow on_focus
2023410	block4	Control	pre	tsam3	Object	Narrow	r1	330.6507778	5	2	on_focus	3	Narrow on_focus
2023410	block4	Control	pre	Jan-01	Subject	Contrastive	r1	192.7569058	1	1	pre_focus	1	Contrastive pre_focus
2023410	block4	Control	pre	Jan-01	Subject	Contrastive	r1	209.2382632	2	2	pre_focus	1	Contrastive pre_focus
2023410	block4	Control	pre	wei3	Verb	Contrastive	r1	167.4284634	3	1	on_focus	3	Contrastive on_focus
2023410	block4	Control	pre	tsam3	Object	Contrastive	r1	111.106576	4	1	post_focus	3	Contrastive post_focus
2023410	block4	Control	pre	tsam3	Object	Contrastive	r1	299.0625644	5	2	post_focus	3	Contrastive post_focus
2023410	block4	Control	pre	pak3	Subject	Broad	r1	119.9168556	1	1	broad_focus	3	Broad focus
2023410	block4	Control	pre	pak3	Subject	Broad	r1	126.5904557	2	2	broad_focus	3	Broad focus
2023410	block4	Control	pre	tsing2	Verb	Broad	r1	205.7105064	3	1	broad_focus	2	Broad focus
2023410	block4	Control	pre	kau2	Object	Broad	r1	235.3408919	4	1	broad_focus	2	Broad focus
2023410	block4	Control	pre	tsi2	Object	Broad	r1	303.611489	5	2	broad_focus	2	Broad focus
2023410	block4	Control	pre	piu35	Subject	Narrow	r1	169.3715042	1	1	on_focus	35	Narrow on_focus
2023410	block4	Control	pre	mui35	Subject	Narrow	r1	197.7250477	2	2	on_focus	35	Narrow on_focus
2023410	block4	Control	pre	tsan3	Verb	Narrow	r1	218.5031314	3	1	post_focus	3	Narrow post_focus
2023410	block4	Control	pre	jin3	Object	Narrow	r1	240.7029478	4	1	post_focus	3	Narrow post_focus
2023410	block4	Control	pre	jin3	Object	Narrow	r1	314.1579056	5	2	post_focus	3	Narrow post_focus
2023410	block4	Control	pre	Jan-01	Subject	Broad	r1	269.3861638	1	1	broad_focus	1	Broad focus
2023410	block4	Control	pre	Jan-01	Subject	Broad	r1	248.0288142	2	2	broad_focus	1	Broad focus
2023410	block4	Control	pre	wei3	Verb	Broad	r1	158.6685563	3	1	broad_focus	3	Broad focus

2023410	block4	Control	pre	tsam3	Object	Broad	r1	177.2096246	4	1	broad_focus	3	Broad focus
2023410	block4	Control	pre	tsam3	Object	Broad	r1	244.2096966	5	2	broad_focus	3	Broad focus
2023410	block4	Control	pre	piu35	Subject	Narrow	r1	134.9406112	1	1	pre_focus	35	Narrow pre_focus
2023410	block4	Control	pre	mui35	Subject	Narrow	r1	255.4859086	2	2	pre_focus	35	Narrow pre_focus
2023410	block4	Control	pre	tsan3	Verb	Narrow	r1	192.6885865	3	1	on_focus	3	Narrow on_focus
2023410	block4	Control	pre	jin3	Object	Narrow	r1	227.5869237	4	1	post_focus	3	Narrow post_focus
2023410	block4	Control	pre	jin3	Object	Narrow	r1	285.1648526	5	2	post_focus	3	Narrow post_focus
2023410	block4	Control	pre	pak3	Subject	Contrastive	r1	118.6547668	1	1	on_focus	3	Contrastive on_focus
2023410	block4	Control	pre	pak3	Subject	Contrastive	r1	121.7100173	2	2	on_focus	3	Contrastive on_focus
2023410	block4	Control	pre	tsing2	Verb	Contrastive	r1	148.7899402	3	1	post_focus	2	Contrastive post_focus
2023410	block4	Control	pre	kau2	Object	Contrastive	r1	210.2721088	4	1	post_focus	2	Contrastive post_focus
2023410	block4	Control	pre	tsi2	Object	Contrastive	r1	285.3602705	5	2	post_focus	2	Contrastive post_focus
2023410	block4	Control	pre	pak3	Subject	Contrastive	r1	113.149311	1	1	pre_focus	3	Contrastive pre_focus
2023410	block4	Control	pre	pak3	Subject	Contrastive	r1	110.5064248	2	2	pre_focus	3	Contrastive pre_focus
2023410	block4	Control	pre	tsing2	Verb	Contrastive	r1	200.0409423	3	1	on_focus	2	Contrastive on_focus
2023410	block4	Control	pre	kau2	Object	Contrastive	r1	189.4132653	4	1	post_focus	2	Contrastive post_focus
2023410	block4	Control	pre	tsi2	Object	Contrastive	r1	282.582475	5	2	post_focus	2	Contrastive post_focus
2023410	block4	Control	pre	Jan-01	Subject	Narrow	r1	211.0657596	1	1	on_focus	1	Narrow on_focus
2023410	block4	Control	pre	Jan-01	Subject	Narrow	r1	205.2032718	2	2	on_focus	1	Narrow on_focus
2023410	block4	Control	pre	wei3	Verb	Narrow	r1	116.8752834	3	1	post_focus	3	Narrow post_focus
2023410	block4	Control	pre	tsam3	Object	Narrow	r1	131.5325019	4	1	post_focus	3	Narrow post_focus
2023410	block4	Control	pre	tsam3	Object	Narrow	r1	201.1088435	5	2	post_focus	3	Narrow post_focus
2023410	block4	Control	pre	Jan-01	Subject	Narrow	r1	227.8209489	1	1	pre_focus	1	Narrow pre_focus
2023410	block4	Control	pre	Jan-01	Subject	Narrow	r1	203.8513659	2	2	pre_focus	1	Narrow pre_focus
2023410	block4	Control	pre	wei3	Verb	Narrow	r1	139.3056646	3	1	on_focus	3	Narrow on_focus
2023410	block4	Control	pre	tsam3	Object	Narrow	r1	183.9531368	4	1	post_focus	3	Narrow post_focus
2023410	block4	Control	pre	tsam3	Object	Narrow	r1	172.1963071	5	2	post_focus	3	Narrow post_focus
2023410	block4	Control	pre	piu35	Subject	Narrow	r1	106.3222114	1	1	pre_focus	35	Narrow pre_focus
2023410	block4	Control	pre	mui35	Subject	Narrow	r1	210.4637188	2	2	pre_focus	35	Narrow pre_focus
2023410	block4	Control	pre	tsan3	Verb	Narrow	r1	200.3050427	3	1	pre_focus	3	Narrow pre_focus
2023410	block4	Control	pre	jin3	Object	Narrow	r1	228.1214024	4	1	on_focus	3	Narrow on_focus
2023410	block4	Control	pre	jin3	Object	Narrow	r1	346.0561854	5	2	on_focus	3	Narrow on_focus
2023410	block4	Control	pre	piu35	Subject	Broad	r1	101.7507559	1	1	broad_focus	35	Broad focus
2023410	block4	Control	pre	mui35	Subject	Broad	r1	257.7045818	2	2	broad_focus	35	Broad focus
2023410	block4	Control	pre	tsan3	Verb	Broad	r1	195.0448282	3	1	broad_focus	3	Broad focus
2023410	block4	Control	pre	jin3	Object	Broad	r1	235.037037	4	1	broad_focus	3	Broad focus
2023410	block4	Control	pre	jin3	Object	Broad	r1	321.7968653	5	2	broad_focus	3	Broad focus
2023410	block4	Control	pre	pak3	Subject	Narrow	r1	130.0056689	1	1	on_focus	3	Narrow on_focus
2023410	block4	Control	pre	pak3	Subject	Narrow	r1	114.8029662	2	2	on_focus	3	Narrow on_focus
2023410	block4	Control	pre	tsing2	Verb	Narrow	r1	149.4362096	3	1	post_focus	2	Narrow post_focus
2023410	block4	Control	pre	kau2	Object	Narrow	r1	82.39455782	4	1	post_focus	2	Narrow post_focus
2023410	block4	Control	pre	tsi2	Object	Narrow	r1	255.0824572	5	2	post_focus	2	Narrow post_focus
2023410	block4	Control	pre	piu35	Subject	Contrastive	r1	130.7964853	1	1	on_focus	35	Contrastive on_focus
2023410	block4	Control	pre	mui35	Subject	Contrastive	r1	232.5439342	2	2	on_focus	35	Contrastive on_focus
2023410	block4	Control	pre	tsan3	Verb	Contrastive	r1	218.7827988	3	1	post_focus	3	Contrastive post_focus
2023410	block4	Control	pre	jin3	Object	Contrastive	r1	288.0804989	4	1	post_focus	3	Contrastive post_focus
2023410	block4	Control	pre	jin3	Object	Contrastive	r1	401.0042112	5	2	post_focus	3	Contrastive post_focus
2023410	block4	Control	pre	pak3	Subject	Narrow	r1	117.452219	1	1	pre_focus	3	Narrow pre_focus
2023410	block4	Control	pre	pak3	Subject	Narrow	r1	101.1366213	2	2	pre_focus	3	Narrow pre_focus
2023410	block4	Control	pre	tsing2	Verb	Narrow	r1	159.3042672	3	1	on_focus	2	Narrow on_focus
2023410	block4	Control	pre	kau2	Object	Narrow	r1	156.845553	4	1	post_focus	2	Narrow post_focus
2023410	block4	Control	pre	tsi2	Object	Narrow	r1	264.1667093	5	2	post_focus	2	Narrow post_focus
2023410	block4	Control	pre	piu35	Subject	Contrastive	r1	84.12838786	1	1	pre_focus	35	Contrastive pre_focus
2023410	block4	Control	pre	mui35	Subject	Contrastive	r1	306.3280423	2	2	pre_focus	35	Contrastive pre_focus
2023410	block4	Control	pre	tsan3	Verb	Contrastive	r1	214.3754454	3	1	on_focus	3	Contrastive on_focus
2023410	block4	Control	pre	jin3	Object	Contrastive	r1	243.6653709	4	1	post_focus	3	Contrastive post_focus
2023410	block4	Control	pre	jin3	Object	Contrastive	r1	395.7823129	5	2	post_focus	3	Contrastive post_focus
2023410	block4	Control	pre	Jan-01	Subject	Contrastive	r1	222.1608088	1	1	on_focus	1	Contrastive on_focus
2023410	block4	Control	pre	Jan-01	Subject	Contrastive	r1	221.9792679	2	2	on_focus	1	Contrastive on_focus
2023410	block4	Control	pre	wei3	Verb	Contrastive	r1	126.8609221	3	1	post_focus	3	Contrastive post_focus
2023410	block4	Control	pre	tsam3	Object	Contrastive	r1	199.4285714	4	1	post_focus	3	Contrastive post_focus
2023410	block4	Control	pre	tsam3	Object	Contrastive	r1	343.9108088	5	2	post_focus	3	Contrastive post_focus
2023410	block4	Control	pre	pak3	Subject	Narrow	r1	84.22839506	1	1	pre_focus	3	Narrow pre_focus
2023410	block4	Control	pre	pak3	Subject	Narrow	r1	81.44525243	2	2	pre_focus	3	Narrow pre_focus
2023410	block4	Control	pre	tsing2	Verb	Narrow	r1	150.3635593	3	1	pre_focus	2	Narrow pre_focus
2023410	block4	Control	pre	kau2	Object	Narrow	r1	161.133031	4	1	on_focus	2	Narrow on_focus

2023410	block4	Control	pre	tsi2	Object	Narrow	r1	281.4229897	5	2	on_focus	2	Narrow on_focus
2023410	block4	Control	pre	piu35	Subject	Contrastive	r2	117.7533842	1	1	pre_focus	35	Contrastive pre_focus
2023410	block4	Control	pre	mui35	Subject	Contrastive	r2	240.4941626	2	2	pre_focus	35	Contrastive pre_focus
2023410	block4	Control	pre	tsan3	Verb	Contrastive	r2	146.3045028	3	1	pre_focus	3	Contrastive pre_focus
2023410	block4	Control	pre	jin3	Object	Contrastive	r2	231.0971817	4	1	on_focus	3	Contrastive on_focus
2023410	block4	Control	pre	jin3	Object	Contrastive	r2	430.7210884	5	2	on_focus	3	Contrastive on_focus
2023410	block4	Control	pre	Jan-01	Subject	Narrow	r2	247.2540414	1	1	pre_focus	1	Narrow pre_focus
2023410	block4	Control	pre	Jan-01	Subject	Narrow	r2	209.5041032	2	2	pre_focus	1	Narrow pre_focus
2023410	block4	Control	pre	wei3	Verb	Narrow	r2	128.5435993	3	1	pre_focus	3	Narrow pre_focus
2023410	block4	Control	pre	tsam3	Object	Narrow	r2	84.93197279	4	1	on_focus	3	Narrow on_focus
2023410	block4	Control	pre	tsam3	Object	Narrow	r2	251.7111966	5	2	on_focus	3	Narrow on_focus
2023410	block4	Control	pre	piu35	Subject	Narrow	r2	90.21348013	1	1	on_focus	35	Narrow on_focus
2023410	block4	Control	pre	mui35	Subject	Narrow	r2	271.2317048	2	2	on_focus	35	Narrow on_focus
2023410	block4	Control	pre	tsan3	Verb	Narrow	r2	179.556928	3	1	post_focus	3	Narrow post_focus
2023410	block4	Control	pre	jin3	Object	Narrow	r2	239.8231293	4	1	post_focus	3	Narrow post_focus
2023410	block4	Control	pre	jin3	Object	Narrow	r2	361.38322	5	2	post_focus	3	Narrow post_focus
2023410	block4	Control	pre	piu35	Subject	Broad	r2	127.7147961	1	1	broad_focus	35	Broad focus
2023410	block4	Control	pre	mui35	Subject	Broad	r2	237.6348397	2	2	broad_focus	35	Broad focus
2023410	block4	Control	pre	tsan3	Verb	Broad	r2	202.3084789	3	1	broad_focus	3	Broad focus
2023410	block4	Control	pre	jin3	Object	Broad	r2	247.9107401	4	1	broad_focus	3	Broad focus
2023410	block4	Control	pre	jin3	Object	Broad	r2	347.084953	5	2	broad_focus	3	Broad focus
2023410	block4	Control	pre	pak3	Subject	Contrastive	r2	147.3778999	1	1	on_focus	3	Contrastive on_focus
2023410	block4	Control	pre	pak3	Subject	Contrastive	r2	133.079851	2	2	on_focus	3	Contrastive on_focus
2023410	block4	Control	pre	tsing2	Verb	Contrastive	r2	161.6783195	3	1	post_focus	2	Contrastive post_focus
2023410	block4	Control	pre	kau2	Object	Contrastive	r2	189.5258566	4	1	post_focus	2	Contrastive post_focus
2023410	block4	Control	pre	tsi2	Object	Contrastive	r2	255.7705275	5	2	post_focus	2	Contrastive post_focus
2023410	block4	Control	pre	piu35	Subject	Narrow	r2	122.3582766	1	1	pre_focus	35	Narrow pre_focus
2023410	block4	Control	pre	mui35	Subject	Narrow	r2	195.1814059	2	2	pre_focus	35	Narrow pre_focus
2023410	block4	Control	pre	tsan3	Verb	Narrow	r2	194.1036785	3	1	on_focus	3	Narrow on_focus
2023410	block4	Control	pre	jin3	Object	Narrow	r2	240.287226	4	1	post_focus	3	Narrow post_focus
2023410	block4	Control	pre	jin3	Object	Narrow	r2	335.4797538	5	2	post_focus	3	Narrow post_focus
2023410	block4	Control	pre	pak3	Subject	Contrastive	r2	135.2879819	1	1	pre_focus	3	Contrastive pre_focus
2023410	block4	Control	pre	pak3	Subject	Contrastive	r2	121.292517	2	2	pre_focus	3	Contrastive pre_focus
2023410	block4	Control	pre	tsing2	Verb	Contrastive	r2	175.8114674	3	1	on_focus	2	Contrastive on_focus
2023410	block4	Control	pre	kau2	Object	Contrastive	r2	193.8936952	4	1	post_focus	2	Contrastive post_focus
2023410	block4	Control	pre	tsi2	Object	Contrastive	r2	274.6642445	5	2	post_focus	2	Contrastive post_focus
2023410	block4	Control	pre	pak3	Subject	Contrastive	r2	112.4457402	1	1	pre_focus	3	Contrastive pre_focus
2023410	block4	Control	pre	pak3	Subject	Contrastive	r2	94.57797214	2	2	pre_focus	3	Contrastive pre_focus
2023410	block4	Control	pre	tsing2	Verb	Contrastive	r2	176.7572362	3	1	pre_focus	2	Contrastive pre_focus
2023410	block4	Control	pre	kau2	Object	Contrastive	r2	179.5011338	4	1	on_focus	2	Contrastive on_focus
2023410	block4	Control	pre	tsi2	Object	Contrastive	r2	253.0045351	5	2	on_focus	2	Contrastive on_focus
2023410	block4	Control	pre	piu35	Subject	Contrastive	r2	126.7254174	1	1	pre_focus	35	Contrastive pre_focus
2023410	block4	Control	pre	mui35	Subject	Contrastive	r2	397.5807148	2	2	pre_focus	35	Contrastive pre_focus
2023410	block4	Control	pre	tsan3	Verb	Contrastive	r2	477.561213	3	1	on_focus	3	Contrastive on_focus
2023410	block4	Control	pre	jin3	Object	Contrastive	r2	302.4675521	4	1	post_focus	3	Contrastive post_focus
2023410	block4	Control	pre	jin3	Object	Contrastive	r2	344.2267574	5	2	post_focus	3	Contrastive post_focus
2023410	block4	Control	pre	piu35	Subject	Contrastive	r2	104.4058957	1	1	on_focus	35	Contrastive on_focus
2023410	block4	Control	pre	mui35	Subject	Contrastive	r2	194.8930118	2	2	on_focus	35	Contrastive on_focus
2023410	block4	Control	pre	tsan3	Verb	Contrastive	r2	228.5780423	3	1	post_focus	3	Contrastive post_focus
2023410	block4	Control	pre	jin3	Object	Contrastive	r2	209.5665571	4	1	post_focus	3	Contrastive post_focus
2023410	block4	Control	pre	jin3	Object	Contrastive	r2	308.2962963	5	2	post_focus	3	Contrastive post_focus
2023410	block4	Control	pre	Jan-01	Subject	Narrow	r2	186.8493323	1	1	on_focus	1	Narrow on_focus
2023410	block4	Control	pre	Jan-01	Subject	Narrow	r2	204.1733731	2	2	on_focus	1	Narrow on_focus
2023410	block4	Control	pre	wei3	Verb	Narrow	r2	120.2322394	3	1	post_focus	3	Narrow post_focus
2023410	block4	Control	pre	tsam3	Object	Narrow	r2	144.803288	4	1	post_focus	3	Narrow post_focus
2023410	block4	Control	pre	tsam3	Object	Narrow	r2	285.0375229	5	2	post_focus	3	Narrow post_focus
2023410	block4	Control	pre	piu35	Subject	Narrow	r2	306.8380305	1	1	pre_focus	35	Narrow pre_focus
2023410	block4	Control	pre	mui35	Subject	Narrow	r2	401.451441	2	2	pre_focus	35	Narrow pre_focus
2023410	block4	Control	pre	tsan3	Verb	Narrow	r2	462.4994846	3	1	pre_focus	3	Narrow pre_focus
2023410	block4	Control	pre	jin3	Object	Narrow	r2	274.0869237	4	1	on_focus	3	Narrow on_focus
2023410	block4	Control	pre	jin3	Object	Narrow	r2	456.5384228	5	2	on_focus	3	Narrow on_focus
2023410	block4	Control	pre	Jan-01	Subject	Contrastive	r2	209.2794155	1	1	on_focus	1	Contrastive on_focus
2023410	block4	Control	pre	Jan-01	Subject	Contrastive	r2	155.8217255	2	2	on_focus	1	Contrastive on_focus
2023410	block4	Control	pre	wei3	Verb	Contrastive	r2	184.4179073	3	1	post_focus	3	Contrastive post_focus
2023410	block4	Control	pre	tsam3	Object	Contrastive	r2	195.8942744	4	1	post_focus	3	Contrastive post_focus
2023410	block4	Control	pre	tsam3	Object	Contrastive	r2	321.1017574	5	2	post_focus	3	Contrastive post_focus

2023410	block4	Control	pre	Jan-01	Subject	Contrastive	r2	355.3957409	1	1	pre_focus	1	Contrastive pre_focus
2023410	block4	Control	pre	Jan-01	Subject	Contrastive	r2	333.8560511	2	2	pre_focus	1	Contrastive pre_focus
2023410	block4	Control	pre	wei3	Verb	Contrastive	r2	264.0179246	3	1	pre_focus	3	Contrastive pre_focus
2023410	block4	Control	pre	tsam3	Object	Contrastive	r2	244.2005183	4	1	on_focus	3	Contrastive on_focus
2023410	block4	Control	pre	tsam3	Object	Contrastive	r2	384.1589423	5	2	on_focus	3	Contrastive on_focus
2023410	block4	Control	pre	pak3	Subject	Broad	r2	112.1882086	1	1	broad_focus	3	Broad focus
2023410	block4	Control	pre	pak3	Subject	Broad	r2	89.70521542	2	2	broad_focus	3	Broad focus
2023410	block4	Control	pre	tsing2	Verb	Broad	r2	234.2533204	3	1	broad_focus	2	Broad focus
2023410	block4	Control	pre	kau2	Object	Broad	r2	167.3718134	4	1	broad_focus	2	Broad focus
2023410	block4	Control	pre	tsi2	Object	Broad	r2	253.6621315	5	2	broad_focus	2	Broad focus
2023410	block4	Control	pre	pak3	Subject	Narrow	r2	105.2615808	1	1	pre_focus	3	Narrow pre_focus
2023410	block4	Control	pre	pak3	Subject	Narrow	r2	72.56727135	2	2	pre_focus	3	Narrow pre_focus
2023410	block4	Control	pre	tsing2	Verb	Narrow	r2	135.1776266	3	1	on_focus	2	Narrow on_focus
2023410	block4	Control	pre	kau2	Object	Narrow	r2	179.402554	4	1	post_focus	2	Narrow post_focus
2023410	block4	Control	pre	tsi2	Object	Narrow	r2	215.0136054	5	2	post_focus	2	Narrow post_focus
2023410	block4	Control	pre	pak3	Subject	Narrow	r2	125.7369615	1	1	pre_focus	3	Narrow pre_focus
2023410	block4	Control	pre	pak3	Subject	Narrow	r2	157.9538927	2	2	pre_focus	3	Narrow pre_focus
2023410	block4	Control	pre	tsing2	Verb	Narrow	r2	361.6448008	3	1	pre_focus	2	Narrow pre_focus
2023410	block4	Control	pre	kau2	Object	Narrow	r2	237.0975057	4	1	on_focus	2	Narrow on_focus
2023410	block4	Control	pre	tsi2	Object	Narrow	r2	228.4211208	5	2	on_focus	2	Narrow on_focus
2023410	block4	Control	pre	Jan-01	Subject	Narrow	r2	178.8057445	1	1	pre_focus	1	Narrow pre_focus
2023410	block4	Control	pre	Jan-01	Subject	Narrow	r2	227.8678328	2	2	pre_focus	1	Narrow pre_focus
2023410	block4	Control	pre	wei3	Verb	Narrow	r2	113.8905086	3	1	on_focus	3	Narrow on_focus
2023410	block4	Control	pre	tsam3	Object	Narrow	r2	133.4167406	4	1	post_focus	3	Narrow post_focus
2023410	block4	Control	pre	tsam3	Object	Narrow	r2	319.4465059	5	2	post_focus	3	Narrow post_focus
2023410	block4	Control	pre	Jan-01	Subject	Contrastive	r2	205.1550858	1	1	pre_focus	1	Contrastive pre_focus
2023410	block4	Control	pre	Jan-01	Subject	Contrastive	r2	215.7237895	2	2	pre_focus	1	Contrastive pre_focus
2023410	block4	Control	pre	wei3	Verb	Contrastive	r2	126.8334953	3	1	on_focus	3	Contrastive on_focus
2023410	block4	Control	pre	tsam3	Object	Contrastive	r2	130.8442933	4	1	post_focus	3	Contrastive post_focus
2023410	block4	Control	pre	tsam3	Object	Contrastive	r2	367.9705215	5	2	post_focus	3	Contrastive post_focus
2023410	block4	Control	pre	Jan-01	Subject	Broad	r2	197.6162132	1	1	broad_focus	1	Broad focus
2023410	block4	Control	pre	Jan-01	Subject	Broad	r2	174.4846423	2	2	broad_focus	1	Broad focus
2023410	block4	Control	pre	wei3	Verb	Broad	r2	185.4957741	3	1	broad_focus	3	Broad focus
2023410	block4	Control	pre	tsam3	Object	Broad	r2	172.0897959	4	1	broad_focus	3	Broad focus
2023410	block4	Control	pre	tsam3	Object	Broad	r2	305.3525537	5	2	broad_focus	3	Broad focus
2023410	block4	Control	pre	pak3	Subject	Narrow	r2	106.2010582	1	1	on_focus	3	Narrow on_focus
2023410	block4	Control	pre	pak3	Subject	Narrow	r2	117.8185941	2	2	on_focus	3	Narrow on_focus
2023410	block4	Control	pre	tsing2	Verb	Narrow	r2	112.7796674	3	1	post_focus	2	Narrow post_focus
2023410	block4	Control	pre	kau2	Object	Narrow	r2	148.7200213	4	1	post_focus	2	Narrow post_focus
2023410	block4	Control	pre	tsi2	Object	Narrow	r2	274.9559737	5	2	post_focus	2	Narrow post_focus
2023410	block5	Control	post	siu2	Subject	Contrastive	r1	146.4100143	1	1	pre_focus	2	Contrastive pre_focus
2023410	block5	Control	post	gwong2	Subject	Contrastive	r1	196.0250866	2	2	pre_focus	2	Contrastive pre_focus
2023410	block5	Control	post	cyun4	Verb	Contrastive	r1	136.3081867	3	1	on_focus	4	Contrastive on_focus
2023410	block5	Control	post	laam4	Object	Contrastive	r1	290.446984	4	1	post_focus	4	Contrastive post_focus
2023410	block5	Control	post	kau4	Object	Contrastive	r1	196.9626478	5	2	post_focus	4	Contrastive post_focus
2023410	block5	Control	post	ceoi3	Subject	Narrow	r1	125.1713687	1	1	pre_focus	3	Narrow pre_focus
2023410	block5	Control	post	ceoi3	Subject	Narrow	r1	146.093468	2	2	pre_focus	3	Narrow pre_focus
2023410	block5	Control	post	caa4	Verb	Narrow	r1	138.1960007	3	1	on_focus	4	Narrow on_focus
2023410	block5	Control	post	ngau4	Object	Narrow	r1	265.2887003	4	1	post_focus	4	Narrow post_focus
2023410	block5	Control	post	jau4	Object	Narrow	r1	271.5579947	5	2	post_focus	4	Narrow post_focus
2023410	block5	Control	post	wai5	Subject	Contrastive	r1	195.5204807	1	1	pre_focus	5	Contrastive pre_focus
2023410	block5	Control	post	wai5	Subject	Contrastive	r1	188.3942338	2	2	pre_focus	5	Contrastive pre_focus
2023410	block5	Control	post	waat3	Verb	Contrastive	r1	140.8660159	3	1	pre_focus	3	Contrastive pre_focus
2023410	block5	Control	post	bui3	Object	Contrastive	r1	133.08921	4	1	on_focus	3	Contrastive on_focus
2023410	block5	Control	post	hok3	Object	Contrastive	r1	152.4396365	5	2	on_focus	3	Contrastive on_focus
2023410	block5	Control	post	ceoi3	Subject	Narrow	r1	102.6332172	1	1	pre_focus	3	Narrow pre_focus
2023410	block5	Control	post	ceoi3	Subject	Narrow	r1	130.0119697	2	2	pre_focus	3	Narrow pre_focus
2023410	block5	Control	post	caa4	Verb	Narrow	r1	105.1237473	3	1	pre_focus	4	Narrow pre_focus
2023410	block5	Control	post	ngau4	Object	Narrow	r1	218.1727677	4	1	on_focus	4	Narrow on_focus
2023410	block5	Control	post	jau4	Object	Narrow	r1	292.3764415	5	2	on_focus	4	Narrow on_focus
2023410	block5	Control	post	siu2	Subject	Narrow	r1	142.0096686	1	1	pre_focus	2	Narrow pre_focus
2023410	block5	Control	post	gwong2	Subject	Narrow	r1	241.6043215	2	2	pre_focus	2	Narrow pre_focus
2023410	block5	Control	post	cyun4	Verb	Narrow	r1	163.1038004	3	1	on_focus	4	Narrow on_focus
2023410	block5	Control	post	laam4	Object	Narrow	r1	193.2877271	4	1	post_focus	4	Narrow post_focus
2023410	block5	Control	post	kau4	Object	Narrow	r1	88.4645973	5	2	post_focus	4	Narrow post_focus
2023410	block5	Control	post	wai5	Subject	Broad	r1	244.9448487	1	1	broad_focus	5	Broad focus

2023410	block5	Control	post	wai5	Subject	Broad	r1	216.0239952	2	2	broad_focus	5	Broad focus
2023410	block5	Control	post	waat3	Verb	Broad	r1	155.3096048	3	1	broad_focus	3	Broad focus
2023410	block5	Control	post	bui3	Object	Broad	r1	153.7412605	4	1	broad_focus	3	Broad focus
2023410	block5	Control	post	hok3	Object	Broad	r1	115.033545	5	2	broad_focus	3	Broad focus
2023410	block5	Control	post	ceoi3	Subject	Narrow	r1	130.2702271	1	1	on_focus	3	Narrow on_focus
2023410	block5	Control	post	ceoi3	Subject	Narrow	r1	112.8571364	2	2	on_focus	3	Narrow on_focus
2023410	block5	Control	post	caa4	Verb	Narrow	r1	82.75407385	3	1	post_focus	4	Narrow post_focus
2023410	block5	Control	post	ngau4	Object	Narrow	r1	199.406794	4	1	post_focus	4	Narrow post_focus
2023410	block5	Control	post	jau4	Object	Narrow	r1	351.8970632	5	2	post_focus	4	Narrow post_focus
2023410	block5	Control	post	ceoi3	Subject	Broad	r1	139.7075346	1	1	broad_focus	3	Broad focus
2023410	block5	Control	post	ceoi3	Subject	Broad	r1	119.5974988	2	2	broad_focus	3	Broad focus
2023410	block5	Control	post	caa4	Verb	Broad	r1	117.5746084	3	1	broad_focus	4	Broad focus
2023410	block5	Control	post	ngau4	Object	Broad	r1	239.3571772	4	1	broad_focus	4	Broad focus
2023410	block5	Control	post	jau4	Object	Broad	r1	270.1554548	5	2	broad_focus	4	Broad focus
2023410	block5	Control	post	siu2	Subject	Contrastive	r1	136.7483596	1	1	on_focus	2	Contrastive on_focus
2023410	block5	Control	post	gwong2	Subject	Contrastive	r1	224.0672714	2	2	on_focus	2	Contrastive on_focus
2023410	block5	Control	post	cyun4	Verb	Contrastive	r1	159.8306191	3	1	post_focus	4	Contrastive post_focus
2023410	block5	Control	post	laam4	Object	Contrastive	r1	240.8354231	4	1	post_focus	4	Contrastive post_focus
2023410	block5	Control	post	kau4	Object	Contrastive	r1	200.2536955	5	2	post_focus	4	Contrastive post_focus
2023410	block5	Control	post	ceoi3	Subject	Contrastive	r1	157.7353961	1	1	pre_focus	3	Contrastive pre_focus
2023410	block5	Control	post	ceoi3	Subject	Contrastive	r1	149.8974102	2	2	pre_focus	3	Contrastive pre_focus
2023410	block5	Control	post	caa4	Verb	Contrastive	r1	141.6562217	3	1	on_focus	4	Contrastive on_focus
2023410	block5	Control	post	ngau4	Object	Contrastive	r1	308.7918378	4	1	post_focus	4	Contrastive post_focus
2023410	block5	Control	post	jau4	Object	Contrastive	r1	214.3787349	5	2	post_focus	4	Contrastive post_focus
2023410	block5	Control	post	wai5	Subject	Contrastive	r1	241.1961605	1	1	pre_focus	5	Contrastive pre_focus
2023410	block5	Control	post	wai5	Subject	Contrastive	r1	177.0658714	2	2	pre_focus	5	Contrastive pre_focus
2023410	block5	Control	post	waat3	Verb	Contrastive	r1	145.2129382	3	1	on_focus	3	Contrastive on_focus
2023410	block5	Control	post	bui3	Object	Contrastive	r1	173.6729731	4	1	post_focus	3	Contrastive post_focus
2023410	block5	Control	post	hok3	Object	Contrastive	r1	165.9331855	5	2	post_focus	3	Contrastive post_focus
2023410	block5	Control	post	siu2	Subject	Narrow	r1	145.9357409	1	1	pre_focus	2	Narrow pre_focus
2023410	block5	Control	post	gwong2	Subject	Narrow	r1	237.019451	2	2	pre_focus	2	Narrow pre_focus
2023410	block5	Control	post	cyun4	Verb	Narrow	r1	74.32773755	3	1	pre_focus	4	Narrow pre_focus
2023410	block5	Control	post	laam4	Object	Narrow	r1	285.5614698	4	1	on_focus	4	Narrow on_focus
2023410	block5	Control	post	kau4	Object	Narrow	r1	199.1266696	5	2	on_focus	4	Narrow on_focus
2023410	block5	Control	post	siu2	Subject	Narrow	r1	160.8216834	1	1	on_focus	2	Narrow on_focus
2023410	block5	Control	post	gwong2	Subject	Narrow	r1	200.6495315	2	2	on_focus	2	Narrow on_focus
2023410	block5	Control	post	cyun4	Verb	Narrow	r1	133.7423958	3	1	post_focus	4	Narrow post_focus
2023410	block5	Control	post	laam4	Object	Narrow	r1	195.53946	4	1	post_focus	4	Narrow post_focus
2023410	block5	Control	post	kau4	Object	Narrow	r1	148.9075704	5	2	post_focus	4	Narrow post_focus
2023410	block5	Control	post	siu2	Subject	Contrastive	r1	149.7376022	1	1	pre_focus	2	Contrastive pre_focus
2023410	block5	Control	post	gwong2	Subject	Contrastive	r1	197.8654099	2	2	pre_focus	2	Contrastive pre_focus
2023410	block5	Control	post	cyun4	Verb	Contrastive	r1	113.9822544	3	1	pre_focus	4	Contrastive pre_focus
2023410	block5	Control	post	laam4	Object	Contrastive	r1	214.7242349	4	1	on_focus	4	Contrastive on_focus
2023410	block5	Control	post	kau4	Object	Contrastive	r1	240.6667848	5	2	on_focus	4	Contrastive on_focus
2023410	block5	Control	post	siu2	Subject	Broad	r1	188.7902653	1	1	broad_focus	2	Broad focus
2023410	block5	Control	post	gwong2	Subject	Broad	r1	228.7356786	2	2	broad_focus	2	Broad focus
2023410	block5	Control	post	cyun4	Verb	Broad	r1	99.01396942	3	1	broad_focus	4	Broad focus
2023410	block5	Control	post	laam4	Object	Broad	r1	287.3366898	4	1	broad_focus	4	Broad focus
2023410	block5	Control	post	kau4	Object	Broad	r1	147.1595278	5	2	broad_focus	4	Broad focus
2023410	block5	Control	post	wai5	Subject	Narrow	r1	177.6750209	1	1	on_focus	5	Narrow on_focus
2023410	block5	Control	post	wai5	Subject	Narrow	r1	208.3447191	2	2	on_focus	5	Narrow on_focus
2023410	block5	Control	post	waat3	Verb	Narrow	r1	149.3926432	3	1	post_focus	3	Narrow post_focus
2023410	block5	Control	post	bui3	Object	Narrow	r1	174.4157711	4	1	post_focus	3	Narrow post_focus
2023410	block5	Control	post	hok3	Object	Narrow	r1	168.4510213	5	2	post_focus	3	Narrow post_focus
2023410	block5	Control	post	wai5	Subject	Narrow	r1	258.982592	1	1	pre_focus	5	Narrow pre_focus
2023410	block5	Control	post	wai5	Subject	Narrow	r1	200.7579518	2	2	pre_focus	5	Narrow pre_focus
2023410	block5	Control	post	waat3	Verb	Narrow	r1	137.7436885	3	1	on_focus	3	Narrow on_focus
2023410	block5	Control	post	bui3	Object	Narrow	r1	151.9266299	4	1	post_focus	3	Narrow post_focus
2023410	block5	Control	post	hok3	Object	Narrow	r1	169.7670569	5	2	post_focus	3	Narrow post_focus
2023410	block5	Control	post	ceoi3	Subject	Contrastive	r1	135.956349	1	1	on_focus	3	Contrastive on_focus
2023410	block5	Control	post	ceoi3	Subject	Contrastive	r1	124.1017582	2	2	on_focus	3	Contrastive on_focus
2023410	block5	Control	post	caa4	Verb	Contrastive	r1	181.8288398	3	1	post_focus	4	Contrastive post_focus
2023410	block5	Control	post	ngau4	Object	Contrastive	r1	196.0102381	4	1	post_focus	4	Contrastive post_focus
2023410	block5	Control	post	jau4	Object	Contrastive	r1	198.4021672	5	2	post_focus	4	Contrastive post_focus
2023410	block5	Control	post	wai5	Subject	Narrow	r1	282.1716665	1	1	pre_focus	5	Narrow pre_focus
2023410	block5	Control	post	wai5	Subject	Narrow	r1	190.1845324	2	2	pre_focus	5	Narrow pre_focus

2023410	block5	Control	post	waat3	Verb	Narrow	r1	123.6103157	3	1	pre_focus	3	Narrow pre_focus
2023410	block5	Control	post	bui3	Object	Narrow	r1	235.0524696	4	1	on_focus	3	Narrow on_focus
2023410	block5	Control	post	hok3	Object	Narrow	r1	169.1594851	5	2	on_focus	3	Narrow on_focus
2023410	block5	Control	post	ceoi3	Subject	Contrastive	r1	128.86853	1	1	pre_focus	3	Contrastive pre_focus
2023410	block5	Control	post	ceoi3	Subject	Contrastive	r1	195.8061527	2	2	pre_focus	3	Contrastive pre_focus
2023410	block5	Control	post	caa4	Verb	Contrastive	r1	220.3101742	3	1	pre_focus	4	Contrastive pre_focus
2023410	block5	Control	post	ngau4	Object	Contrastive	r1	211.5300522	4	1	on_focus	4	Contrastive on_focus
2023410	block5	Control	post	jau4	Object	Contrastive	r1	230.4666598	5	2	on_focus	4	Contrastive on_focus
2023410	block5	Control	post	wai5	Subject	Contrastive	r1	207.6756453	1	1	on_focus	5	Contrastive on_focus
2023410	block5	Control	post	wai5	Subject	Contrastive	r1	246.1296587	2	2	on_focus	5	Contrastive on_focus
2023410	block5	Control	post	waat3	Verb	Contrastive	r1	153.8526154	3	1	post_focus	3	Contrastive post_focus
2023410	block5	Control	post	bui3	Object	Contrastive	r1	141.670247	4	1	post_focus	3	Contrastive post_focus
2023410	block5	Control	post	hok3	Object	Contrastive	r1	103.8811451	5	2	post_focus	3	Contrastive post_focus
2023410	block5	Control	post	ceoi3	Subject	Broad	r2	146.1936604	1	1	broad_focus	3	Broad focus
2023410	block5	Control	post	ceoi3	Subject	Broad	r2	162.2018958	2	2	broad_focus	3	Broad focus
2023410	block5	Control	post	caa4	Verb	Broad	r2	187.000733	3	1	broad_focus	4	Broad focus
2023410	block5	Control	post	ngau4	Object	Broad	r2	107.6337948	4	1	broad_focus	4	Broad focus
2023410	block5	Control	post	jau4	Object	Broad	r2	294.6994435	5	2	broad_focus	4	Broad focus
2023410	block5	Control	post	siu2	Subject	Narrow	r2	135.9137016	1	1	pre_focus	2	Narrow pre_focus
2023410	block5	Control	post	gwong2	Subject	Narrow	r2	248.6411219	2	2	pre_focus	2	Narrow pre_focus
2023410	block5	Control	post	cyun4	Verb	Narrow	r2	86.63573785	3	1	on_focus	4	Narrow on_focus
2023410	block5	Control	post	laam4	Object	Narrow	r2	304.0174842	4	1	post_focus	4	Narrow post_focus
2023410	block5	Control	post	kau4	Object	Narrow	r2	120.1706611	5	2	post_focus	4	Narrow post_focus
2023410	block5	Control	post	wai5	Subject	Broad	r2	217.1248415	1	1	broad_focus	5	Broad focus
2023410	block5	Control	post	wai5	Subject	Broad	r2	191.606737	2	2	broad_focus	5	Broad focus
2023410	block5	Control	post	waat3	Verb	Broad	r2	126.4517552	3	1	broad_focus	3	Broad focus
2023410	block5	Control	post	bui3	Object	Broad	r2	169.3301839	4	1	broad_focus	3	Broad focus
2023410	block5	Control	post	hok3	Object	Broad	r2	102.5677088	5	2	broad_focus	3	Broad focus
2023410	block5	Control	post	siu2	Subject	Contrastive	r2	169.0078636	1	1	on_focus	2	Contrastive on_focus
2023410	block5	Control	post	gwong2	Subject	Contrastive	r2	212.2005021	2	2	on_focus	2	Contrastive on_focus
2023410	block5	Control	post	cyun4	Verb	Contrastive	r2	86.41401109	3	1	post_focus	4	Contrastive post_focus
2023410	block5	Control	post	laam4	Object	Contrastive	r2	230.4141885	4	1	post_focus	4	Contrastive post_focus
2023410	block5	Control	post	kau4	Object	Contrastive	r2	241.9491524	5	2	post_focus	4	Contrastive post_focus
2023410	block5	Control	post	siu2	Subject	Contrastive	r2	270.2457999	1	1	pre_focus	2	Contrastive pre_focus
2023410	block5	Control	post	gwong2	Subject	Contrastive	r2	261.6046882	2	2	pre_focus	2	Contrastive pre_focus
2023410	block5	Control	post	cyun4	Verb	Contrastive	r2	138.571852	3	1	pre_focus	4	Contrastive pre_focus
2023410	block5	Control	post	laam4	Object	Contrastive	r2	290.2187453	4	1	on_focus	4	Contrastive on_focus
2023410	block5	Control	post	kau4	Object	Contrastive	r2	205.0016375	5	2	on_focus	4	Contrastive on_focus
2023410	block5	Control	post	ceoi3	Subject	Contrastive	r2	136.5982464	1	1	pre_focus	3	Contrastive pre_focus
2023410	block5	Control	post	ceoi3	Subject	Contrastive	r2	123.1505899	2	2	pre_focus	3	Contrastive pre_focus
2023410	block5	Control	post	caa4	Verb	Contrastive	r2	134.1243052	3	1	on_focus	4	Contrastive on_focus
2023410	block5	Control	post	ngau4	Object	Contrastive	r2	239.3343089	4	1	post_focus	4	Contrastive post_focus
2023410	block5	Control	post	jau4	Object	Contrastive	r2	315.1604206	5	2	post_focus	4	Contrastive post_focus
2023410	block5	Control	post	ceoi3	Subject	Narrow	r2	132.097329	1	1	pre_focus	3	Narrow pre_focus
2023410	block5	Control	post	ceoi3	Subject	Narrow	r2	149.4252874	2	2	pre_focus	3	Narrow pre_focus
2023410	block5	Control	post	caa4	Verb	Narrow	r2	117.4066924	3	1	pre_focus	4	Narrow pre_focus
2023410	block5	Control	post	ngau4	Object	Narrow	r2	218.667707	4	1	on_focus	4	Narrow on_focus
2023410	block5	Control	post	jau4	Object	Narrow	r2	229.0184092	5	2	on_focus	4	Narrow on_focus
2023410	block5	Control	post	wai5	Subject	Contrastive	r2	181.7408047	1	1	on_focus	5	Contrastive on_focus
2023410	block5	Control	post	wai5	Subject	Contrastive	r2	207.5760889	2	2	on_focus	5	Contrastive on_focus
2023410	block5	Control	post	waat3	Verb	Contrastive	r2	128.6051906	3	1	post_focus	3	Contrastive post_focus
2023410	block5	Control	post	bui3	Object	Contrastive	r2	117.5572077	4	1	post_focus	3	Contrastive post_focus
2023410	block5	Control	post	hok3	Object	Contrastive	r2	101.3491206	5	2	post_focus	3	Contrastive post_focus
2023410	block5	Control	post	ceoi3	Subject	Contrastive	r2	115.7829578	1	1	pre_focus	3	Contrastive pre_focus
2023410	block5	Control	post	ceoi3	Subject	Contrastive	r2	130.1373921	2	2	pre_focus	3	Contrastive pre_focus
2023410	block5	Control	post	caa4	Verb	Contrastive	r2	66.49579078	3	1	pre_focus	4	Contrastive pre_focus
2023410	block5	Control	post	ngau4	Object	Contrastive	r2	200.511075	4	1	on_focus	4	Contrastive on_focus
2023410	block5	Control	post	jau4	Object	Contrastive	r2	332.261733	5	2	on_focus	4	Contrastive on_focus
2023410	block5	Control	post	wai5	Subject	Contrastive	r2	215.618903	1	1	pre_focus	5	Contrastive pre_focus
2023410	block5	Control	post	wai5	Subject	Contrastive	r2	179.0487484	2	2	pre_focus	5	Contrastive pre_focus
2023410	block5	Control	post	waat3	Verb	Contrastive	r2	161.8657385	3	1	on_focus	3	Contrastive on_focus
2023410	block5	Control	post	bui3	Object	Contrastive	r2	145.1452634	4	1	post_focus	3	Contrastive post_focus
2023410	block5	Control	post	hok3	Object	Contrastive	r2	194.7882439	5	2	post_focus	3	Contrastive post_focus
2023410	block5	Control	post	wai5	Subject	Narrow	r2	199.4862325	1	1	pre_focus	5	Narrow pre_focus
2023410	block5	Control	post	wai5	Subject	Narrow	r2	191.6365551	2	2	pre_focus	5	Narrow pre_focus
2023410	block5	Control	post	waat3	Verb	Narrow	r2	135.3238071	3	1	pre_focus	3	Narrow pre_focus

2023410	block5	Control	post	bui3	Object	Narrow	r2	93.67592889	4	1	on_focus	3	Narrow on_focus
2023410	block5	Control	post	hok3	Object	Narrow	r2	153.7984158	5	2	on_focus	3	Narrow on_focus
2023410	block5	Control	post	wai5	Subject	Narrow	r2	166.2884994	1	1	pre_focus	5	Narrow pre_focus
2023410	block5	Control	post	wai5	Subject	Narrow	r2	177.5172575	2	2	pre_focus	5	Narrow pre_focus
2023410	block5	Control	post	waat3	Verb	Narrow	r2	142.6913148	3	1	on_focus	3	Narrow on_focus
2023410	block5	Control	post	bui3	Object	Narrow	r2	134.9941015	4	1	post_focus	3	Narrow post_focus
2023410	block5	Control	post	hok3	Object	Narrow	r2	157.8504053	5	2	post_focus	3	Narrow post_focus
2023410	block5	Control	post	siu2	Subject	Narrow	r2	148.3782024	1	1	pre_focus	2	Narrow pre_focus
2023410	block5	Control	post	gwong2	Subject	Narrow	r2	205.5318003	2	2	pre_focus	2	Narrow pre_focus
2023410	block5	Control	post	cyun4	Verb	Narrow	r2	114.1218298	3	1	pre_focus	4	Narrow pre_focus
2023410	block5	Control	post	laam4	Object	Narrow	r2	236.9593737	4	1	on_focus	4	Narrow on_focus
2023410	block5	Control	post	kau4	Object	Narrow	r2	266.7151702	5	2	on_focus	4	Narrow on_focus
2023410	block5	Control	post	ceoi3	Subject	Contrastive	r2	108.1550061	1	1	on_focus	3	Contrastive on_focus
2023410	block5	Control	post	ceoi3	Subject	Contrastive	r2	141.4100873	2	2	on_focus	3	Contrastive on_focus
2023410	block5	Control	post	caa4	Verb	Contrastive	r2	159.4218854	3	1	post_focus	4	Contrastive post_focus
2023410	block5	Control	post	ngau4	Object	Contrastive	r2	246.8357645	4	1	post_focus	4	Contrastive post_focus
2023410	block5	Control	post	jau4	Object	Contrastive	r2	270.6957028	5	2	post_focus	4	Contrastive post_focus
2023410	block5	Control	post	ceoi3	Subject	Narrow	r2	131.826714	1	1	on_focus	3	Narrow on_focus
2023410	block5	Control	post	ceoi3	Subject	Narrow	r2	171.5358993	2	2	on_focus	3	Narrow on_focus
2023410	block5	Control	post	caa4	Verb	Narrow	r2	124.5927302	3	1	post_focus	4	Narrow post_focus
2023410	block5	Control	post	ngau4	Object	Narrow	r2	242.7201748	4	1	post_focus	4	Narrow post_focus
2023410	block5	Control	post	jau4	Object	Narrow	r2	318.4193919	5	2	post_focus	4	Narrow post_focus
2023410	block5	Control	post	siu2	Subject	Broad	r2	95.71573419	1	1	broad_focus	2	Broad focus
2023410	block5	Control	post	gwong2	Subject	Broad	r2	163.0370829	2	2	broad_focus	2	Broad focus
2023410	block5	Control	post	cyun4	Verb	Broad	r2	124.3181875	3	1	broad_focus	4	Broad focus
2023410	block5	Control	post	laam4	Object	Broad	r2	249.1691429	4	1	broad_focus	4	Broad focus
2023410	block5	Control	post	kau4	Object	Broad	r2	152.0649589	5	2	broad_focus	4	Broad focus
2023410	block5	Control	post	wai5	Subject	Contrastive	r2	210.7131777	1	1	pre_focus	5	Contrastive pre_focus
2023410	block5	Control	post	wai5	Subject	Contrastive	r2	241.1505092	2	2	pre_focus	5	Contrastive pre_focus
2023410	block5	Control	post	waat3	Verb	Contrastive	r2	162.8095961	3	1	pre_focus	3	Contrastive pre_focus
2023410	block5	Control	post	bui3	Object	Contrastive	r2	194.6247471	4	1	on_focus	3	Contrastive on_focus
2023410	block5	Control	post	hok3	Object	Contrastive	r2	261.4026808	5	2	on_focus	3	Contrastive on_focus
2023410	block5	Control	post	ceoi3	Subject	Narrow	r2	153.949417	1	1	pre_focus	3	Narrow pre_focus
2023410	block5	Control	post	ceoi3	Subject	Narrow	r2	164.9759499	2	2	pre_focus	3	Narrow pre_focus
2023410	block5	Control	post	caa4	Verb	Narrow	r2	215.7496862	3	1	on_focus	4	Narrow on_focus
2023410	block5	Control	post	ngau4	Object	Narrow	r2	304.7624455	4	1	post_focus	4	Narrow post_focus
2023410	block5	Control	post	jau4	Object	Narrow	r2	292.656524	5	2	post_focus	4	Narrow post_focus
2023410	block5	Control	post	wai5	Subject	Narrow	r2	361.2393734	1	1	on_focus	5	Narrow on_focus
2023410	block5	Control	post	wai5	Subject	Narrow	r2	307.6011292	2	2	on_focus	5	Narrow on_focus
2023410	block5	Control	post	waat3	Verb	Narrow	r2	171.302328	3	1	post_focus	3	Narrow post_focus
2023410	block5	Control	post	bui3	Object	Narrow	r2	216.0073753	4	1	post_focus	3	Narrow post_focus
2023410	block5	Control	post	hok3	Object	Narrow	r2	216.2315318	5	2	post_focus	3	Narrow post_focus
2023410	block5	Control	post	siu2	Subject	Narrow	r2	115.8821061	1	1	on_focus	2	Narrow on_focus
2023410	block5	Control	post	gwong2	Subject	Narrow	r2	193.5416095	2	2	on_focus	2	Narrow on_focus
2023410	block5	Control	post	cyun4	Verb	Narrow	r2	81.01874875	3	1	post_focus	4	Narrow post_focus
2023410	block5	Control	post	laam4	Object	Narrow	r2	242.8412	4	1	post_focus	4	Narrow post_focus
2023410	block5	Control	post	kau4	Object	Narrow	r2	209.326587	5	2	post_focus	4	Narrow post_focus
2023410	block5	Control	post	siu2	Subject	Contrastive	r2	115.9911211	1	1	pre_focus	2	Contrastive pre_focus
2023410	block5	Control	post	gwong2	Subject	Contrastive	r2	157.0236038	2	2	pre_focus	2	Contrastive pre_focus
2023410	block5	Control	post	cyun4	Verb	Contrastive	r2	102.2036758	3	1	on_focus	4	Contrastive on_focus
2023410	block5	Control	post	laam4	Object	Contrastive	r2	236.1272228	4	1	post_focus	4	Contrastive post_focus
2023410	block5	Control	post	kau4	Object	Contrastive	r2	141.6193225	5	2	post_focus	4	Contrastive post_focus
2023410	block5	Control	pre	siu2	Subject	Contrastive	r1	165.1707552	1	1	pre_focus	2	Contrastive pre_focus
2023410	block5	Control	pre	gwong2	Subject	Contrastive	r1	200.8325552	2	2	pre_focus	2	Contrastive pre_focus
2023410	block5	Control	pre	cyun4	Verb	Contrastive	r1	170.4610733	3	1	on_focus	4	Contrastive on_focus
2023410	block5	Control	pre	laam4	Object	Contrastive	r1	207.8717201	4	1	post_focus	4	Contrastive post_focus
2023410	block5	Control	pre	kau4	Object	Contrastive	r1	195.2248677	5	2	post_focus	4	Contrastive post_focus
2023410	block5	Control	pre	siu2	Subject	Narrow	r1	123.5657596	1	1	on_focus	2	Narrow on_focus
2023410	block5	Control	pre	gwong2	Subject	Narrow	r1	216.6668149	2	2	on_focus	2	Narrow on_focus
2023410	block5	Control	pre	cyun4	Verb	Narrow	r1	161.1030126	3	1	post_focus	4	Narrow post_focus
2023410	block5	Control	pre	laam4	Object	Narrow	r1	235.0430839	4	1	post_focus	4	Narrow post_focus
2023410	block5	Control	pre	kau4	Object	Narrow	r1	67.45275888	5	2	post_focus	4	Narrow post_focus
2023410	block5	Control	pre	siu2	Subject	Narrow	r1	118.7067271	1	1	pre_focus	2	Narrow pre_focus
2023410	block5	Control	pre	gwong2	Subject	Narrow	r1	178.8283123	2	2	pre_focus	2	Narrow pre_focus
2023410	block5	Control	pre	cyun4	Verb	Narrow	r1	118.3248299	3	1	pre_focus	4	Narrow pre_focus
2023410	block5	Control	pre	laam4	Object	Narrow	r1	224.8601663	4	1	on_focus	4	Narrow on_focus

2023410	block5	Control	pre	kau4	Object	Narrow	r1	84.56901375	5	2	on_focus	4	Narrow on_focus
2023410	block5	Control	pre	wai5	Subject	Contrastive	r1	219.6233308	1	1	pre_focus	5	Contrastive pre_focus
2023410	block5	Control	pre	wai5	Subject	Contrastive	r1	139.0454595	2	2	pre_focus	5	Contrastive pre_focus
2023410	block5	Control	pre	waat3	Verb	Contrastive	r1	80.81974589	3	1	on_focus	3	Contrastive on_focus
2023410	block5	Control	pre	bui3	Object	Contrastive	r1	147.8980189	4	1	post_focus	3	Contrastive post_focus
2023410	block5	Control	pre	hok3	Object	Contrastive	r1	89.52587095	5	2	post_focus	3	Contrastive post_focus
2023410	block5	Control	pre	siu2	Subject	Contrastive	r1	173.9297052	1	1	on_focus	2	Contrastive on_focus
2023410	block5	Control	pre	gwong2	Subject	Contrastive	r1	195.2010804	2	2	on_focus	2	Contrastive on_focus
2023410	block5	Control	pre	cyun4	Verb	Contrastive	r1	175.0774754	3	1	post_focus	4	Contrastive post_focus
2023410	block5	Control	pre	laam4	Object	Contrastive	r1	112.6582941	4	1	post_focus	4	Contrastive post_focus
2023410	block5	Control	pre	kau4	Object	Contrastive	r1	128.0857899	5	2	post_focus	4	Contrastive post_focus
2023410	block5	Control	pre	wai5	Subject	Contrastive	r1	237.1728134	1	1	pre_focus	5	Contrastive pre_focus
2023410	block5	Control	pre	wai5	Subject	Contrastive	r1	180.6047943	2	2	pre_focus	5	Contrastive pre_focus
2023410	block5	Control	pre	waat3	Verb	Contrastive	r1	163.5953641	3	1	pre_focus	3	Contrastive pre_focus
2023410	block5	Control	pre	bui3	Object	Contrastive	r1	140.3492063	4	1	on_focus	3	Contrastive on_focus
2023410	block5	Control	pre	hok3	Object	Contrastive	r1	196.4021164	5	2	on_focus	3	Contrastive on_focus
2023410	block5	Control	pre	ceoi3	Subject	Narrow	r1	566.4407191	1	1	pre_focus	3	Narrow pre_focus
2023410	block5	Control	pre	ceoi3	Subject	Narrow	r1	194.5162509	2	2	pre_focus	3	Narrow pre_focus
2023410	block5	Control	pre	caa4	Verb	Narrow	r1	186.7772545	3	1	on_focus	4	Narrow on_focus
2023410	block5	Control	pre	ngau4	Object	Narrow	r1	255.2857143	4	1	post_focus	4	Narrow post_focus
2023410	block5	Control	pre	jau4	Object	Narrow	r1	296.2941367	5	2	post_focus	4	Narrow post_focus
2023410	block5	Control	pre	siu2	Subject	Narrow	r1	149.0075914	1	1	pre_focus	2	Narrow pre_focus
2023410	block5	Control	pre	gwong2	Subject	Narrow	r1	401.6666667	2	2	pre_focus	2	Narrow pre_focus
2023410	block5	Control	pre	cyun4	Verb	Narrow	r1	183.8758791	3	1	on_focus	4	Narrow on_focus
2023410	block5	Control	pre	laam4	Object	Narrow	r1	174.4563222	4	1	post_focus	4	Narrow post_focus
2023410	block5	Control	pre	kau4	Object	Narrow	r1	126.3326356	5	2	post_focus	4	Narrow post_focus
2023410	block5	Control	pre	ceoi3	Subject	Contrastive	r1	153.020683	1	1	on_focus	3	Contrastive on_focus
2023410	block5	Control	pre	ceoi3	Subject	Contrastive	r1	150.4758941	2	2	on_focus	3	Contrastive on_focus
2023410	block5	Control	pre	caa4	Verb	Contrastive	r1	162.9266818	3	1	post_focus	4	Contrastive post_focus
2023410	block5	Control	pre	ngau4	Object	Contrastive	r1	242.6052802	4	1	post_focus	4	Contrastive post_focus
2023410	block5	Control	pre	jau4	Object	Contrastive	r1	140.7515387	5	2	post_focus	4	Contrastive post_focus
2023410	block5	Control	pre	ceoi3	Subject	Contrastive	r1	211.8905896	1	1	pre_focus	3	Contrastive pre_focus
2023410	block5	Control	pre	ceoi3	Subject	Contrastive	r1	288.3333333	2	2	pre_focus	3	Contrastive pre_focus
2023410	block5	Control	pre	caa4	Verb	Contrastive	r1	222.5763956	3	1	pre_focus	4	Contrastive pre_focus
2023410	block5	Control	pre	ngau4	Object	Contrastive	r1	316.4739229	4	1	on_focus	4	Contrastive on_focus
2023410	block5	Control	pre	jau4	Object	Contrastive	r1	175.1253508	5	2	on_focus	4	Contrastive on_focus
2023410	block5	Control	pre	ceoi3	Subject	Broad	r1	129.575266	1	1	broad_focus	3	Broad focus
2023410	block5	Control	pre	ceoi3	Subject	Broad	r1	134.8963395	2	2	broad_focus	3	Broad focus
2023410	block5	Control	pre	caa4	Verb	Broad	r1	246.1124339	3	1	broad_focus	4	Broad focus
2023410	block5	Control	pre	ngau4	Object	Broad	r1	107.0068027	4	1	broad_focus	4	Broad focus
2023410	block5	Control	pre	jau4	Object	Broad	r1	152.0327178	5	2	broad_focus	4	Broad focus
2023410	block5	Control	pre	ceoi3	Subject	Narrow	r1	131.068999	1	1	pre_focus	3	Narrow pre_focus
2023410	block5	Control	pre	ceoi3	Subject	Narrow	r1	118.8106867	2	2	pre_focus	3	Narrow pre_focus
2023410	block5	Control	pre	caa4	Verb	Narrow	r1	93.33144369	3	1	pre_focus	4	Narrow pre_focus
2023410	block5	Control	pre	ngau4	Object	Narrow	r1	233.3268546	4	1	on_focus	4	Narrow on_focus
2023410	block5	Control	pre	jau4	Object	Narrow	r1	166.1814059	5	2	on_focus	4	Narrow on_focus
2023410	block5	Control	pre	wai5	Subject	Narrow	r1	161.2761207	1	1	pre_focus	5	Narrow pre_focus
2023410	block5	Control	pre	wai5	Subject	Narrow	r1	162.6428032	2	2	pre_focus	5	Narrow pre_focus
2023410	block5	Control	pre	waat3	Verb	Narrow	r1	150.4318697	3	1	pre_focus	3	Narrow pre_focus
2023410	block5	Control	pre	bui3	Object	Narrow	r1	116.9309421	4	1	on_focus	3	Narrow on_focus
2023410	block5	Control	pre	hok3	Object	Narrow	r1	93.91987906	5	2	on_focus	3	Narrow on_focus
2023410	block5	Control	pre	wai5	Subject	Broad	r1	151.6950113	1	1	broad_focus	5	Broad focus
2023410	block5	Control	pre	wai5	Subject	Broad	r1	159.0986395	2	2	broad_focus	5	Broad focus
2023410	block5	Control	pre	waat3	Verb	Broad	r1	117.0702948	3	1	broad_focus	3	Broad focus
2023410	block5	Control	pre	bui3	Object	Broad	r1	139.4890401	4	1	broad_focus	3	Broad focus
2023410	block5	Control	pre	hok3	Object	Broad	r1	141.7428251	5	2	broad_focus	3	Broad focus
2023410	block5	Control	pre	wai5	Subject	Narrow	r1	148.6550454	1	1	pre_focus	5	Narrow pre_focus
2023410	block5	Control	pre	wai5	Subject	Narrow	r1	177.7439114	2	2	pre_focus	5	Narrow pre_focus
2023410	block5	Control	pre	waat3	Verb	Narrow	r1	140.4843594	3	1	on_focus	3	Narrow on_focus
2023410	block5	Control	pre	bui3	Object	Narrow	r1	144.1450216	4	1	post_focus	3	Narrow post_focus
2023410	block5	Control	pre	hok3	Object	Narrow	r1	98.02397149	5	2	post_focus	3	Narrow post_focus
2023410	block5	Control	pre	wai5	Subject	Contrastive	r1	168.9880952	1	1	on_focus	5	Contrastive on_focus
2023410	block5	Control	pre	wai5	Subject	Contrastive	r1	129.4008832	2	2	on_focus	5	Contrastive on_focus
2023410	block5	Control	pre	waat3	Verb	Contrastive	r1	128.2575316	3	1	post_focus	3	Contrastive post_focus
2023410	block5	Control	pre	bui3	Object	Contrastive	r1	115.9637188	4	1	post_focus	3	Contrastive post_focus
2023410	block5	Control	pre	hok3	Object	Contrastive	r1	177.7649282	5	2	post_focus	3	Contrastive post_focus

2023410	block5	Control	pre	ceoi3	Subject	Contrastive	r1	84.83155167	1	1	pre_focus	3	Contrastive pre_focus
2023410	block5	Control	pre	ceoi3	Subject	Contrastive	r1	155.8687212	2	2	pre_focus	3	Contrastive pre_focus
2023410	block5	Control	pre	caa4	Verb	Contrastive	r1	93.98904006	3	1	on_focus	4	Contrastive on_focus
2023410	block5	Control	pre	ngau4	Object	Contrastive	r1	234.8783069	4	1	post_focus	4	Contrastive post_focus
2023410	block5	Control	pre	jau4	Object	Contrastive	r1	268.473167	5	2	post_focus	4	Contrastive post_focus
2023410	block5	Control	pre	wai5	Subject	Narrow	r1	123.6997255	1	1	on_focus	5	Narrow on_focus
2023410	block5	Control	pre	wai5	Subject	Narrow	r1	160.1304893	2	2	on_focus	5	Narrow on_focus
2023410	block5	Control	pre	waat3	Verb	Narrow	r1	138.2562358	3	1	post_focus	3	Narrow post_focus
2023410	block5	Control	pre	bui3	Object	Narrow	r1	121.1871253	4	1	post_focus	3	Narrow post_focus
2023410	block5	Control	pre	hok3	Object	Narrow	r1	133.0012958	5	2	post_focus	3	Narrow post_focus
2023410	block5	Control	pre	siu2	Subject	Contrastive	r1	116.2328042	1	1	pre_focus	2	Contrastive pre_focus
2023410	block5	Control	pre	gwong2	Subject	Contrastive	r1	153.471243	2	2	pre_focus	2	Contrastive pre_focus
2023410	block5	Control	pre	cyun4	Verb	Contrastive	r1	96.33786848	3	1	pre_focus	4	Contrastive pre_focus
2023410	block5	Control	pre	laam4	Object	Contrastive	r1	239.4721592	4	1	on_focus	4	Contrastive on_focus
2023410	block5	Control	pre	kau4	Object	Contrastive	r1	171.379932	5	2	on_focus	4	Contrastive on_focus
2023410	block5	Control	pre	siu2	Subject	Broad	r1	122.0634921	1	1	broad_focus	2	Broad focus
2023410	block5	Control	pre	gwong2	Subject	Broad	r1	166.5595868	2	2	broad_focus	2	Broad focus
2023410	block5	Control	pre	cyun4	Verb	Broad	r1	95.67743764	3	1	broad_focus	4	Broad focus
2023410	block5	Control	pre	laam4	Object	Broad	r1	103.291679	4	1	broad_focus	4	Broad focus
2023410	block5	Control	pre	kau4	Object	Broad	r1	102.7069161	5	2	broad_focus	4	Broad focus
2023410	block5	Control	pre	ceoi3	Subject	Narrow	r1	112.1477162	1	1	on_focus	3	Narrow on_focus
2023410	block5	Control	pre	ceoi3	Subject	Narrow	r1	115.5692555	2	2	on_focus	3	Narrow on_focus
2023410	block5	Control	pre	caa4	Verb	Narrow	r1	105.7377755	3	1	post_focus	4	Narrow post_focus
2023410	block5	Control	pre	ngau4	Object	Narrow	r1	225.7048375	4	1	post_focus	4	Narrow post_focus
2023410	block5	Control	pre	jau4	Object	Narrow	r1	141.3856183	5	2	post_focus	4	Narrow post_focus
2023410	block5	Control	pre	wai5	Subject	Contrastive	r2	210.9732243	1	1	on_focus	5	Contrastive on_focus
2023410	block5	Control	pre	wai5	Subject	Contrastive	r2	157.8042328	2	2	on_focus	5	Contrastive on_focus
2023410	block5	Control	pre	waat3	Verb	Contrastive	r2	121.1996095	3	1	post_focus	3	Contrastive post_focus
2023410	block5	Control	pre	bui3	Object	Contrastive	r2	110.8604182	4	1	post_focus	3	Contrastive post_focus
2023410	block5	Control	pre	hok3	Object	Contrastive	r2	107.5170068	5	2	post_focus	3	Contrastive post_focus
2023410	block5	Control	pre	wai5	Subject	Narrow	r2	124.2947846	1	1	pre_focus	5	Narrow pre_focus
2023410	block5	Control	pre	wai5	Subject	Narrow	r2	163.4950537	2	2	pre_focus	5	Narrow pre_focus
2023410	block5	Control	pre	waat3	Verb	Narrow	r2	148.8873772	3	1	pre_focus	3	Narrow pre_focus
2023410	block5	Control	pre	bui3	Object	Narrow	r2	97.61763039	4	1	on_focus	3	Narrow on_focus
2023410	block5	Control	pre	hok3	Object	Narrow	r2	126.1197495	5	2	on_focus	3	Narrow on_focus
2023410	block5	Control	pre	siu2	Subject	Contrastive	r2	98.17721961	1	1	on_focus	2	Contrastive on_focus
2023410	block5	Control	pre	gwong2	Subject	Contrastive	r2	172.7210884	2	2	on_focus	2	Contrastive on_focus
2023410	block5	Control	pre	cyun4	Verb	Contrastive	r2	130.3826531	3	1	post_focus	4	Contrastive post_focus
2023410	block5	Control	pre	laam4	Object	Contrastive	r2	105.5608466	4	1	post_focus	4	Contrastive post_focus
2023410	block5	Control	pre	kau4	Object	Contrastive	r2	143.4325765	5	2	post_focus	4	Contrastive post_focus
2023410	block5	Control	pre	wai5	Subject	Contrastive	r2	201.6470831	1	1	pre_focus	5	Contrastive pre_focus
2023410	block5	Control	pre	wai5	Subject	Contrastive	r2	206.1148904	2	2	pre_focus	5	Contrastive pre_focus
2023410	block5	Control	pre	waat3	Verb	Contrastive	r2	160.4240363	3	1	on_focus	3	Contrastive on_focus
2023410	block5	Control	pre	bui3	Object	Contrastive	r2	150.5045351	4	1	post_focus	3	Contrastive post_focus
2023410	block5	Control	pre	hok3	Object	Contrastive	r2	130.7102365	5	2	post_focus	3	Contrastive post_focus
2023410	block5	Control	pre	ceoi3	Subject	Broad	r2	193.0528907	1	1	broad_focus	3	Broad focus
2023410	block5	Control	pre	ceoi3	Subject	Broad	r2	135.4698546	2	2	broad_focus	3	Broad focus
2023410	block5	Control	pre	caa4	Verb	Broad	r2	157.3602419	3	1	broad_focus	4	Broad focus
2023410	block5	Control	pre	ngau4	Object	Broad	r2	210.6746032	4	1	broad_focus	4	Broad focus
2023410	block5	Control	pre	jau4	Object	Broad	r2	134.0626012	5	2	broad_focus	4	Broad focus
2023410	block5	Control	pre	ceoi3	Subject	Contrastive	r2	149.1303855	1	1	pre_focus	3	Contrastive pre_focus
2023410	block5	Control	pre	ceoi3	Subject	Contrastive	r2	180.8371126	2	2	pre_focus	3	Contrastive pre_focus
2023410	block5	Control	pre	caa4	Verb	Contrastive	r2	93.04391932	3	1	on_focus	4	Contrastive on_focus
2023410	block5	Control	pre	ngau4	Object	Contrastive	r2	170.4535147	4	1	post_focus	4	Contrastive post_focus
2023410	block5	Control	pre	jau4	Object	Contrastive	r2	235.8597884	5	2	post_focus	4	Contrastive post_focus
2023410	block5	Control	pre	wai5	Subject	Broad	r2	233.3225354	1	1	broad_focus	5	Broad focus
2023410	block5	Control	pre	wai5	Subject	Broad	r2	345.4383976	2	2	broad_focus	5	Broad focus
2023410	block5	Control	pre	waat3	Verb	Broad	r2	141.037037	3	1	broad_focus	3	Broad focus
2023410	block5	Control	pre	bui3	Object	Broad	r2	141.2988158	4	1	broad_focus	3	Broad focus
2023410	block5	Control	pre	hok3	Object	Broad	r2	167.1216931	5	2	broad_focus	3	Broad focus
2023410	block5	Control	pre	wai5	Subject	Narrow	r2	190.6786524	1	1	on_focus	5	Narrow on_focus
2023410	block5	Control	pre	wai5	Subject	Narrow	r2	156.2712474	2	2	on_focus	5	Narrow on_focus
2023410	block5	Control	pre	waat3	Verb	Narrow	r2	129.5714867	3	1	post_focus	3	Narrow post_focus
2023410	block5	Control	pre	bui3	Object	Narrow	r2	130.0179516	4	1	post_focus	3	Narrow post_focus
2023410	block5	Control	pre	hok3	Object	Narrow	r2	130.5850813	5	2	post_focus	3	Narrow post_focus
2023410	block5	Control	pre	wai5	Subject	Contrastive	r2	298.0895692	1	1	pre_focus	5	Contrastive pre_focus

2023410	block5	Control	pre	wai5	Subject	Contrastive	r2	387.3316625	2	2	pre_focus	5	Contrastive pre_focus
2023410	block5	Control	pre	waat3	Verb	Contrastive	r2	150.2324263	3	1	pre_focus	3	Contrastive pre_focus
2023410	block5	Control	pre	bui3	Object	Contrastive	r2	179.2610589	4	1	on_focus	3	Contrastive on_focus
2023410	block5	Control	pre	hok3	Object	Contrastive	r2	133.9119875	5	2	on_focus	3	Contrastive on_focus
2023410	block5	Control	pre	siu2	Subject	Contrastive	r2	163.3666417	1	1	pre_focus	2	Contrastive pre_focus
2023410	block5	Control	pre	gwong2	Subject	Contrastive	r2	214.7868481	2	2	pre_focus	2	Contrastive pre_focus
2023410	block5	Control	pre	cyun4	Verb	Contrastive	r2	334.886098	3	1	pre_focus	4	Contrastive pre_focus
2023410	block5	Control	pre	laam4	Object	Contrastive	r2	311.1797198	4	1	on_focus	4	Contrastive on_focus
2023410	block5	Control	pre	kau4	Object	Contrastive	r2	169.6995465	5	2	on_focus	4	Contrastive on_focus
2023410	block5	Control	pre	ceoi3	Subject	Contrastive	r2	102.4245762	1	1	pre_focus	3	Contrastive pre_focus
2023410	block5	Control	pre	ceoi3	Subject	Contrastive	r2	89.49886621	2	2	pre_focus	3	Contrastive pre_focus
2023410	block5	Control	pre	caa4	Verb	Contrastive	r2	126.7261905	3	1	pre_focus	4	Contrastive pre_focus
2023410	block5	Control	pre	ngau4	Object	Contrastive	r2	167.8344671	4	1	on_focus	4	Contrastive on_focus
2023410	block5	Control	pre	jau4	Object	Contrastive	r2	247.2778318	5	2	on_focus	4	Contrastive on_focus
2023410	block5	Control	pre	wai5	Subject	Narrow	r2	246.3667134	1	1	pre_focus	5	Narrow pre_focus
2023410	block5	Control	pre	wai5	Subject	Narrow	r2	160.1716877	2	2	pre_focus	5	Narrow pre_focus
2023410	block5	Control	pre	waat3	Verb	Narrow	r2	125.3435554	3	1	on_focus	3	Narrow on_focus
2023410	block5	Control	pre	bui3	Object	Narrow	r2	129.1164561	4	1	post_focus	3	Narrow post_focus
2023410	block5	Control	pre	hok3	Object	Narrow	r2	142.8964475	5	2	post_focus	3	Narrow post_focus
2023410	block5	Control	pre	siu2	Subject	Broad	r2	135.7734046	1	1	broad_focus	2	Broad focus
2023410	block5	Control	pre	gwong2	Subject	Broad	r2	185.909801	2	2	broad_focus	2	Broad focus
2023410	block5	Control	pre	cyun4	Verb	Broad	r2	103.6446094	3	1	broad_focus	4	Broad focus
2023410	block5	Control	pre	laam4	Object	Broad	r2	152.2260015	4	1	broad_focus	4	Broad focus
2023410	block5	Control	pre	kau4	Object	Broad	r2	74.77090307	5	2	broad_focus	4	Broad focus
2023410	block5	Control	pre	siu2	Subject	Contrastive	r2	180.2947846	1	1	pre_focus	2	Contrastive pre_focus
2023410	block5	Control	pre	gwong2	Subject	Contrastive	r2	180.3896104	2	2	pre_focus	2	Contrastive pre_focus
2023410	block5	Control	pre	cyun4	Verb	Contrastive	r2	78.07350718	3	1	on_focus	4	Contrastive on_focus
2023410	block5	Control	pre	laam4	Object	Contrastive	r2	122.3167045	4	1	post_focus	4	Contrastive post_focus
2023410	block5	Control	pre	kau4	Object	Contrastive	r2	254.1794218	5	2	post_focus	4	Contrastive post_focus
2023410	block5	Control	pre	siu2	Subject	Narrow	r2	129.7782817	1	1	pre_focus	2	Narrow pre_focus
2023410	block5	Control	pre	gwong2	Subject	Narrow	r2	148.7879819	2	2	pre_focus	2	Narrow pre_focus
2023410	block5	Control	pre	cyun4	Verb	Narrow	r2	123.8866213	3	1	on_focus	4	Narrow on_focus
2023410	block5	Control	pre	laam4	Object	Narrow	r2	128.6122449	4	1	post_focus	4	Narrow post_focus
2023410	block5	Control	pre	kau4	Object	Narrow	r2	171.3038549	5	2	post_focus	4	Narrow post_focus
2023410	block5	Control	pre	ceoi3	Subject	Narrow	r2	157.0228602	1	1	pre_focus	3	Narrow pre_focus
2023410	block5	Control	pre	ceoi3	Subject	Narrow	r2	141.4735106	2	2	pre_focus	3	Narrow pre_focus
2023410	block5	Control	pre	caa4	Verb	Narrow	r2	116.733023	3	1	on_focus	4	Narrow on_focus
2023410	block5	Control	pre	ngau4	Object	Narrow	r2	173.6049698	4	1	post_focus	4	Narrow post_focus
2023410	block5	Control	pre	jau4	Object	Narrow	r2	106.4368859	5	2	post_focus	4	Narrow post_focus
2023410	block5	Control	pre	ceoi3	Subject	Contrastive	r2	103.8413778	1	1	on_focus	3	Contrastive on_focus
2023410	block5	Control	pre	ceoi3	Subject	Contrastive	r2	129.8781179	2	2	on_focus	3	Contrastive on_focus
2023410	block5	Control	pre	caa4	Verb	Contrastive	r2	84.77891156	3	1	post_focus	4	Contrastive post_focus
2023410	block5	Control	pre	ngau4	Object	Contrastive	r2	142.5034014	4	1	post_focus	4	Contrastive post_focus
2023410	block5	Control	pre	jau4	Object	Contrastive	r2	328.9206938	5	2	post_focus	4	Contrastive post_focus
2023410	block5	Control	pre	siu2	Subject	Narrow	r2	96.68594104	1	1	on_focus	2	Narrow on_focus
2023410	block5	Control	pre	gwong2	Subject	Narrow	r2	150.8739877	2	2	on_focus	2	Narrow on_focus
2023410	block5	Control	pre	cyun4	Verb	Narrow	r2	74.34938078	3	1	post_focus	4	Narrow post_focus
2023410	block5	Control	pre	laam4	Object	Narrow	r2	258.0952381	4	1	post_focus	4	Narrow post_focus
2023410	block5	Control	pre	kau4	Object	Narrow	r2	84.57671958	5	2	post_focus	4	Narrow post_focus
2023410	block5	Control	pre	ceoi3	Subject	Narrow	r2	74.61904762	1	1	pre_focus	3	Narrow pre_focus
2023410	block5	Control	pre	ceoi3	Subject	Narrow	r2	92.78054009	2	2	pre_focus	3	Narrow pre_focus
2023410	block5	Control	pre	caa4	Verb	Narrow	r2	139.3320341	3	1	pre_focus	4	Narrow pre_focus
2023410	block5	Control	pre	ngau4	Object	Narrow	r2	160.4482924	4	1	on_focus	4	Narrow on_focus
2023410	block5	Control	pre	jau4	Object	Narrow	r2	96.58811144	5	2	on_focus	4	Narrow on_focus
2023410	block5	Control	pre	siu2	Subject	Narrow	r2	96.31408119	1	1	pre_focus	2	Narrow pre_focus
2023410	block5	Control	pre	gwong2	Subject	Narrow	r2	230.0982615	2	2	pre_focus	2	Narrow pre_focus
2023410	block5	Control	pre	cyun4	Verb	Narrow	r2	389.5379819	3	1	pre_focus	4	Narrow pre_focus
2023410	block5	Control	pre	laam4	Object	Narrow	r2	716.8027211	4	1	on_focus	4	Narrow on_focus
2023410	block5	Control	pre	kau4	Object	Narrow	r2	262.3645755	5	2	on_focus	4	Narrow on_focus
2023410	block5	Control	pre	ceoi3	Subject	Narrow	r2	156.0224696	1	1	on_focus	3	Narrow on_focus
2023410	block5	Control	pre	ceoi3	Subject	Narrow	r2	215.7009007	2	2	on_focus	3	Narrow on_focus
2023410	block5	Control	pre	caa4	Verb	Narrow	r2	196.8041383	3	1	post_focus	4	Narrow post_focus
2023410	block5	Control	pre	ngau4	Object	Narrow	r2	241.031746	4	1	post_focus	4	Narrow post_focus
2023410	block5	Control	pre	jau4	Object	Narrow	r2	148.0797774	5	2	post_focus	4	Narrow post_focus

CASD_HumanSpeech_duration_clean_add

Subject	Block	TrainingType	TrainingOrder	word	word_type	focus_type	repetition	Duration	syllable	syll_focus	tone	focus_condition
**2023206**	block1	HumanSpeech	Post	zoeng1	Subject	Contrastive	r1	162.153319807459	1	on_focus	1	Contrastive on_focus
**2023206**	block1	HumanSpeech	Post	saang1	Subject	Contrastive	r1	159.244142101286	2	on_focus	1	Contrastive on_focus
**2023206**	block1	HumanSpeech	Post	tsa1	Verb	Contrastive	r1	217.602968460127	1	post_focus	1	Contrastive post_focus
**2023206**	block1	HumanSpeech	Post	fei1	Object	Contrastive	r1	184.834551104387	1	post_focus	1	Contrastive post_focus
**2023206**	block1	HumanSpeech	Post	gei1	Object	Contrastive	r1	186.205738705738	2	post_focus	1	Contrastive post_focus
**2023206**	block1	HumanSpeech	Post	sau3	Subject	Narrow	r1	195.224517226237	1	on_focus	3	Narrow on_focus
**2023206**	block1	HumanSpeech	Post	sau3	Subject	Narrow	r1	183.979695269912	2	on_focus	3	Narrow on_focus
**2023206**	block1	HumanSpeech	Post	sik3	Verb	Narrow	r1	181.565141207997	1	post_focus	3	Narrow post_focus
**2023206**	block1	HumanSpeech	Post	baak3	Object	Narrow	r1	183.453213453205	1	post_focus	3	Narrow post_focus
**2023206**	block1	HumanSpeech	Post	baak3	Object	Narrow	r1	136.56565355187	2	post_focus	3	Narrow post_focus
**2023206**	block1	HumanSpeech	Post	jyun2	Subject	Contrastive	r1	125.940550133109	1	pre_focus	2	Contrastive pre_focus
**2023206**	block1	HumanSpeech	Post	jyun2	Subject	Contrastive	r1	114.579646394887	2	pre_focus	2	Contrastive pre_focus
**2023206**	block1	HumanSpeech	Post	mo2	Verb	Contrastive	r1	245.570260427399	1	pre_focus	2	Contrastive pre_focus
**2023206**	block1	HumanSpeech	Post	gau2	Object	Contrastive	r1	151.618026720072	1	on_focus	2	Contrastive on_focus
**2023206**	block1	HumanSpeech	Post	zai2	Object	Contrastive	r1	363.635967207401	2	on_focus	2	Contrastive on_focus
**2023206**	block1	HumanSpeech	Post	jyun2	Subject	Contrastive	r1	114.752096187317	1	on_focus	2	Contrastive on_focus
**2023206**	block1	HumanSpeech	Post	jyun2	Subject	Contrastive	r1	119.693877551043	2	on_focus	2	Contrastive on_focus
**2023206**	block1	HumanSpeech	Post	mo2	Verb	Contrastive	r1	202.887188208649	1	post_focus	2	Contrastive post_focus
**2023206**	block1	HumanSpeech	Post	gau2	Object	Contrastive	r1	134.548313949239	1	post_focus	2	Contrastive post_focus
**2023206**	block1	HumanSpeech	Post	zai2	Object	Contrastive	r1	475.613303929634	2	post_focus	2	Contrastive post_focus
**2023206**	block1	HumanSpeech	Post	sau3	Subject	Contrastive	r1	163.587613378695	1	pre_focus	3	Contrastive pre_focus
**2023206**	block1	HumanSpeech	Post	sau3	Subject	Contrastive	r1	140.404040404036	2	pre_focus	3	Contrastive pre_focus
**2023206**	block1	HumanSpeech	Post	sik3	Verb	Contrastive	r1	179.240019240041	1	on_focus	3	Contrastive on_focus
**2023206**	block1	HumanSpeech	Post	baak3	Object	Contrastive	r1	206.404275996107	1	post_focus	3	Contrastive post_focus
**2023206**	block1	HumanSpeech	Post	baak3	Object	Contrastive	r1	155.398631057352	2	post_focus	3	Contrastive post_focus
**2023206**	block1	HumanSpeech	Post	sau3	Subject	Narrow	r1	80.6418493322951	1	pre_focus	3	Narrow pre_focus
**2023206**	block1	HumanSpeech	Post	sau3	Subject	Narrow	r1	160.181439079707	2	pre_focus	3	Narrow pre_focus
**2023206**	block1	HumanSpeech	Post	sik3	Verb	Narrow	r1	86.2352346066473	1	on_focus	3	Narrow on_focus
**2023206**	block1	HumanSpeech	Post	baak3	Object	Narrow	r1	116.085159989922	1	post_focus	3	Narrow post_focus
**2023206**	block1	HumanSpeech	Post	baak3	Object	Narrow	r1	123.846614836395	2	post_focus	3	Narrow post_focus
**2023206**	block1	HumanSpeech	Post	zoeng1	Subject	Narrow	r1	143.235071806515	1	pre_focus	1	Narrow pre_focus
**2023206**	block1	HumanSpeech	Post	saang1	Subject	Narrow	r1	193.055802030955	2	pre_focus	1	Narrow pre_focus
**2023206**	block1	HumanSpeech	Post	tsa1	Verb	Narrow	r1	166.658163265311	1	pre_focus	1	Narrow pre_focus
**2023206**	block1	HumanSpeech	Post	fei1	Object	Narrow	r1	178.298752834479	1	on_focus	1	Narrow on_focus
**2023206**	block1	HumanSpeech	Post	gei1	Object	Narrow	r1	256.093078501237	2	on_focus	1	Narrow on_focus
**2023206**	block1	HumanSpeech	Post	sau3	Subject	Narrow	r1	73.0223517978459	1	pre_focus	3	Narrow pre_focus
**2023206**	block1	HumanSpeech	Post	sau3	Subject	Narrow	r1	72.8329210471941	2	pre_focus	3	Narrow pre_focus
**2023206**	block1	HumanSpeech	Post	sik3	Verb	Narrow	r1	116.660236966368	1	pre_focus	3	Narrow pre_focus
**2023206**	block1	HumanSpeech	Post	baak3	Object	Narrow	r1	102.776614919463	1	on_focus	3	Narrow on_focus
**2023206**	block1	HumanSpeech	Post	baak3	Object	Narrow	r1	486.044142931888	2	on_focus	3	Narrow on_focus
**2023206**	block1	HumanSpeech	Post	sau3	Subject	Broad	r1	116.217183169567	1	broad_focus	3	Broad focus
**2023206**	block1	HumanSpeech	Post	sau3	Subject	Broad	r1	165.523388380535	2	broad_focus	3	Broad focus
**2023206**	block1	HumanSpeech	Post	sik3	Verb	Broad	r1	132.782369212464	1	broad_focus	3	Broad focus
**2023206**	block1	HumanSpeech	Post	baak3	Object	Broad	r1	141.747958442636	1	broad_focus	3	Broad focus
**2023206**	block1	HumanSpeech	Post	baak3	Object	Broad	r1	343.391372421991	2	broad_focus	3	Broad focus
**2023206**	block1	HumanSpeech	Post	jyun2	Subject	Narrow	r1	181.587462245375	1	pre_focus	2	Narrow pre_focus
**2023206**	block1	HumanSpeech	Post	jyun2	Subject	Narrow	r1	109.030815745086	2	pre_focus	2	Narrow pre_focus
**2023206**	block1	HumanSpeech	Post	mo2	Verb	Narrow	r1	187.326970179186	1	pre_focus	2	Narrow pre_focus
**2023206**	block1	HumanSpeech	Post	gau2	Object	Narrow	r1	375.376821884771	1	on_focus	2	Narrow on_focus
**2023206**	block1	HumanSpeech	Post	zai2	Object	Narrow	r1	303.161475116354	2	on_focus	2	Narrow on_focus
**2023206**	block1	HumanSpeech	Post	jyun2	Subject	Broad	r1	308.469063816005	1	broad_focus	2	Broad focus
**2023206**	block1	HumanSpeech	Post	jyun2	Subject	Broad	r1	216.162761400852	2	broad_focus	2	Broad focus
**2023206**	block1	HumanSpeech	Post	mo2	Verb	Broad	r1	320.070454045606	1	broad_focus	2	Broad focus
**2023206**	block1	HumanSpeech	Post	gau2	Object	Broad	r1	237.147598433324	1	broad_focus	2	Broad focus
**2023206**	block1	HumanSpeech	Post	zai2	Object	Broad	r1	452.434191067738	2	broad_focus	2	Broad focus
**2023206**	block1	HumanSpeech	Post	zoeng1	Subject	Contrastive	r1	87.9920424960119	1	pre_focus	1	Contrastive pre_focus
**2023206**	block1	HumanSpeech	Post	saang1	Subject	Contrastive	r1	148.241758241767	2	pre_focus	1	Contrastive pre_focus
**2023206**	block1	HumanSpeech	Post	tsa1	Verb	Contrastive	r1	164.474706064112	1	on_focus	1	Contrastive on_focus
**2023206**	block1	HumanSpeech	Post	fei1	Object	Contrastive	r1	159.083610858232	1	post_focus	1	Contrastive post_focus

**2023206**	block1	HumanSpeech	Post	gei1	Object	Contrastive	r1	244.008780817296	2	post_focus	1	Contrastive post_focus
**2023206**	block1	HumanSpeech	Post	sau3	Subject	Contrastive	r1	98.8281876853421	1	pre_focus	3	Contrastive pre_focus
**2023206**	block1	HumanSpeech	Post	sau3	Subject	Contrastive	r1	145.173454663222	2	pre_focus	3	Contrastive pre_focus
**2023206**	block1	HumanSpeech	Post	sik3	Verb	Contrastive	r1	107.625661375636	1	pre_focus	3	Contrastive pre_focus
**2023206**	block1	HumanSpeech	Post	baak3	Object	Contrastive	r1	124.317110113026	1	on_focus	3	Contrastive on_focus
**2023206**	block1	HumanSpeech	Post	baak3	Object	Contrastive	r1	205.550167936849	2	on_focus	3	Contrastive on_focus
**2023206**	block1	HumanSpeech	Post	zoeng1	Subject	Narrow	r1	94.4217539253884	1	pre_focus	1	Narrow pre_focus
**2023206**	block1	HumanSpeech	Post	saang1	Subject	Narrow	r1	167.636250432395	2	pre_focus	1	Narrow pre_focus
**2023206**	block1	HumanSpeech	Post	tsa1	Verb	Narrow	r1	156.241895063403	1	on_focus	1	Narrow on_focus
**2023206**	block1	HumanSpeech	Post	fei1	Object	Narrow	r1	163.123882772737	1	post_focus	1	Narrow post_focus
**2023206**	block1	HumanSpeech	Post	gei1	Object	Narrow	r1	285.741138560695	2	post_focus	1	Narrow post_focus
**2023206**	block1	HumanSpeech	Post	zoeng1	Subject	Contrastive	r1	110.208097104987	1	pre_focus	1	Contrastive pre_focus
**2023206**	block1	HumanSpeech	Post	saang1	Subject	Contrastive	r1	141.459075452957	2	pre_focus	1	Contrastive pre_focus
**2023206**	block1	HumanSpeech	Post	tsa1	Verb	Contrastive	r1	142.131079443288	1	pre_focus	1	Contrastive pre_focus
**2023206**	block1	HumanSpeech	Post	fei1	Object	Contrastive	r1	137.462401937995	1	on_focus	1	Contrastive on_focus
**2023206**	block1	HumanSpeech	Post	gei1	Object	Contrastive	r1	1525.8393347564	2	on_focus	1	Contrastive on_focus
**2023206**	block1	HumanSpeech	Post	zoeng1	Subject	Narrow	r1	126.682747509165	1	on_focus	1	Narrow on_focus
**2023206**	block1	HumanSpeech	Post	saang1	Subject	Narrow	r1	125.071479719708	2	on_focus	1	Narrow on_focus
**2023206**	block1	HumanSpeech	Post	tsa1	Verb	Narrow	r1	96.6293318674332	1	post_focus	1	Narrow post_focus
**2023206**	block1	HumanSpeech	Post	fei1	Object	Narrow	r1	143.48559891792	1	post_focus	1	Narrow post_focus
**2023206**	block1	HumanSpeech	Post	gei1	Object	Narrow	r1	298.514416212839	2	post_focus	1	Narrow post_focus
**2023206**	block1	HumanSpeech	Post	jyun2	Subject	Narrow	r1	96.1608386044759	1	on_focus	2	Narrow on_focus
**2023206**	block1	HumanSpeech	Post	jyun2	Subject	Narrow	r1	195.548752834469	2	on_focus	2	Narrow on_focus
**2023206**	block1	HumanSpeech	Post	mo2	Verb	Narrow	r1	140.010475925237	1	post_focus	2	Narrow post_focus
**2023206**	block1	HumanSpeech	Post	gau2	Object	Narrow	r1	110.786272180832	1	post_focus	2	Narrow post_focus
**2023206**	block1	HumanSpeech	Post	zai2	Object	Narrow	r1	262.871464375223	2	post_focus	2	Narrow post_focus
**2023206**	block1	HumanSpeech	Post	jyun2	Subject	Narrow	r1	150.125060125049	1	pre_focus	2	Narrow pre_focus
**2023206**	block1	HumanSpeech	Post	jyun2	Subject	Narrow	r1	182.145319080007	2	pre_focus	2	Narrow pre_focus
**2023206**	block1	HumanSpeech	Post	mo2	Verb	Narrow	r1	214.830177962028	1	on_focus	2	Narrow on_focus
**2023206**	block1	HumanSpeech	Post	gau2	Object	Narrow	r1	161.40359609085	1	post_focus	2	Narrow post_focus
**2023206**	block1	HumanSpeech	Post	zai2	Object	Narrow	r1	294.090770636615	2	post_focus	2	Narrow post_focus
**2023206**	block1	HumanSpeech	Post	sau3	Subject	Contrastive	r1	145.591695011348	1	on_focus	3	Contrastive on_focus
**2023206**	block1	HumanSpeech	Post	sau3	Subject	Contrastive	r1	174.560490863001	2	on_focus	3	Contrastive on_focus
**2023206**	block1	HumanSpeech	Post	sik3	Verb	Contrastive	r1	189.550642479219	1	post_focus	3	Contrastive post_focus
**2023206**	block1	HumanSpeech	Post	baak3	Object	Contrastive	r1	208.811949022049	1	post_focus	3	Contrastive post_focus
**2023206**	block1	HumanSpeech	Post	baak3	Object	Contrastive	r1	125.364431486901	2	post_focus	3	Contrastive post_focus
**2023206**	block1	HumanSpeech	Post	jyun2	Subject	Contrastive	r1	140.015489967993	1	pre_focus	2	Contrastive pre_focus
**2023206**	block1	HumanSpeech	Post	jyun2	Subject	Contrastive	r1	123.836692273244	2	pre_focus	2	Contrastive pre_focus
**2023206**	block1	HumanSpeech	Post	mo2	Verb	Contrastive	r1	190.49869824471	1	on_focus	2	Contrastive on_focus
**2023206**	block1	HumanSpeech	Post	gau2	Object	Contrastive	r1	97.7656668728173	1	post_focus	2	Contrastive post_focus
**2023206**	block1	HumanSpeech	Post	zai2	Object	Contrastive	r1	369.821617535933	2	post_focus	2	Contrastive post_focus
**2023206**	block1	HumanSpeech	Post	zoeng1	Subject	Broad	r1	171.908948445321	1	broad_focus	1	Broad focus
**2023206**	block1	HumanSpeech	Post	saang1	Subject	Broad	r1	193.618696178135	2	broad_focus	1	Broad focus
**2023206**	block1	HumanSpeech	Post	tsa1	Verb	Broad	r1	246.054789579148	1	broad_focus	1	Broad focus
**2023206**	block1	HumanSpeech	Post	fei1	Object	Broad	r1	210.531933783386	1	broad_focus	1	Broad focus
**2023206**	block1	HumanSpeech	Post	gei1	Object	Broad	r1	364.553753125222	2	broad_focus	1	Broad focus
**2023206**	block1	HumanSpeech	Post	sau3	Subject	Contrastive	r2	116.880034553503	1	pre_focus	3	Contrastive pre_focus
**2023206**	block1	HumanSpeech	Post	sau3	Subject	Contrastive	r2	93.7883016454748	2	pre_focus	3	Contrastive pre_focus
**2023206**	block1	HumanSpeech	Post	sik3	Verb	Contrastive	r2	80.0437317784031	1	on_focus	3	Contrastive on_focus
**2023206**	block1	HumanSpeech	Post	baak3	Object	Contrastive	r2	112.03857481695	1	post_focus	3	Contrastive post_focus
**2023206**	block1	HumanSpeech	Post	baak3	Object	Contrastive	r2	539.801916465535	2	post_focus	3	Contrastive post_focus
**2023206**	block1	HumanSpeech	Post	zoeng1	Subject	Narrow	r2	99.1443590423273	1	on_focus	1	Narrow on_focus
**2023206**	block1	HumanSpeech	Post	saang1	Subject	Narrow	r2	163.369878913329	2	on_focus	1	Narrow on_focus
**2023206**	block1	HumanSpeech	Post	tsa1	Verb	Narrow	r2	165.802406632622	1	post_focus	1	Narrow post_focus
**2023206**	block1	HumanSpeech	Post	fei1	Object	Narrow	r2	215.308438787645	1	post_focus	1	Narrow post_focus
**2023206**	block1	HumanSpeech	Post	gei1	Object	Narrow	r2	491.136922854309	2	post_focus	1	Narrow post_focus
**2023206**	block1	HumanSpeech	Post	jyun2	Subject	Contrastive	r2	213.283338732253	1	pre_focus	2	Contrastive pre_focus
**2023206**	block1	HumanSpeech	Post	jyun2	Subject	Contrastive	r2	115.064391406236	2	pre_focus	2	Contrastive pre_focus
**2023206**	block1	HumanSpeech	Post	mo2	Verb	Contrastive	r2	158.49459866763	1	pre_focus	2	Contrastive pre_focus
**2023206**	block1	HumanSpeech	Post	gau2	Object	Contrastive	r2	122.166477702194	1	on_focus	2	Contrastive on_focus
**2023206**	block1	HumanSpeech	Post	zai2	Object	Contrastive	r2	578.995616024201	2	on_focus	2	Contrastive on_focus
**2023206**	block1	HumanSpeech	Post	zoeng1	Subject	Contrastive	r2	97.9358685830221	1	on_focus	1	Contrastive on_focus

2023206	block1	HumanSpeech	Post	saang1	Subject	Contrastive	r2	119.630473038626	2	on_focus	1	Contrastive on_focus
2023206	block1	HumanSpeech	Post	tsa1	Verb	Contrastive	r2	177.233597386305	1	post_focus	1	Contrastive post_focus
2023206	block1	HumanSpeech	Post	fei1	Object	Contrastive	r2	118.334278155714	1	post_focus	1	Contrastive post_focus
2023206	block1	HumanSpeech	Post	gei1	Object	Contrastive	r2	910.870670107101	2	post_focus	1	Contrastive post_focus
2023206	block1	HumanSpeech	Post	sau3	Subject	Narrow	r2	110.709408990431	1	pre_focus	3	Narrow pre_focus
2023206	block1	HumanSpeech	Post	sau3	Subject	Narrow	r2	101.241267039882	2	pre_focus	3	Narrow pre_focus
2023206	block1	HumanSpeech	Post	sik3	Verb	Narrow	r2	81.8349281104247	1	pre_focus	3	Narrow pre_focus
2023206	block1	HumanSpeech	Post	baak3	Object	Narrow	r2	184.203092055554	1	on_focus	3	Narrow on_focus
2023206	block1	HumanSpeech	Post	baak3	Object	Narrow	r2	400.689895299706	2	on_focus	3	Narrow on_focus
2023206	block1	HumanSpeech	Post	sau3	Subject	Contrastive	r2	130.95248893211	1	pre_focus	3	Contrastive pre_focus
2023206	block1	HumanSpeech	Post	sau3	Subject	Contrastive	r2	145.823435194131	2	pre_focus	3	Contrastive pre_focus
2023206	block1	HumanSpeech	Post	sik3	Verb	Contrastive	r2	106.950910724549	1	pre_focus	3	Contrastive pre_focus
2023206	block1	HumanSpeech	Post	baak3	Object	Contrastive	r2	152.053584196437	1	on_focus	3	Contrastive on_focus
2023206	block1	HumanSpeech	Post	baak3	Object	Contrastive	r2	333.665513221888	2	on_focus	3	Contrastive on_focus
2023206	block1	HumanSpeech	Post	zoeng1	Subject	Narrow	r2	84.1331592160941	1	pre_focus	1	Narrow pre_focus
2023206	block1	HumanSpeech	Post	saang1	Subject	Narrow	r2	122.456470683517	2	pre_focus	1	Narrow pre_focus
2023206	block1	HumanSpeech	Post	tsa1	Verb	Narrow	r2	115.498110355247	1	pre_focus	1	Narrow pre_focus
2023206	block1	HumanSpeech	Post	fei1	Object	Narrow	r2	154.381708238816	1	on_focus	1	Narrow on_focus
2023206	block1	HumanSpeech	Post	gei1	Object	Narrow	r2	235.223080223022	2	on_focus	1	Narrow on_focus
2023206	block1	HumanSpeech	Post	jyun2	Subject	Narrow	r2	139.622515672897	1	on_focus	2	Narrow on_focus
2023206	block1	HumanSpeech	Post	jyun2	Subject	Narrow	r2	145.302603652908	2	on_focus	2	Narrow on_focus
2023206	block1	HumanSpeech	Post	mo2	Verb	Narrow	r2	125.849330599863	1	post_focus	2	Narrow post_focus
2023206	block1	HumanSpeech	Post	gau2	Object	Narrow	r2	140.809787480919	1	post_focus	2	Narrow post_focus
2023206	block1	HumanSpeech	Post	zai2	Object	Narrow	r2	427.898764769111	2	post_focus	2	Narrow post_focus
2023206	block1	HumanSpeech	Post	sau3	Subject	Narrow	r2	123.534409416436	1	pre_focus	3	Narrow pre_focus
2023206	block1	HumanSpeech	Post	sau3	Subject	Narrow	r2	141.299359509844	2	pre_focus	3	Narrow pre_focus
2023206	block1	HumanSpeech	Post	sik3	Verb	Narrow	r2	160.119186261511	1	on_focus	3	Narrow on_focus
2023206	block1	HumanSpeech	Post	baak3	Object	Narrow	r2	244.793773365188	1	post_focus	3	Narrow post_focus
2023206	block1	HumanSpeech	Post	baak3	Object	Narrow	r2	316.97611228401	2	post_focus	3	Narrow post_focus
2023206	block1	HumanSpeech	Post	jyun2	Subject	Narrow	r2	105.599668438799	1	pre_focus	2	Narrow pre_focus
2023206	block1	HumanSpeech	Post	jyun2	Subject	Narrow	r2	70.2351115884881	2	pre_focus	2	Narrow pre_focus
2023206	block1	HumanSpeech	Post	mo2	Verb	Narrow	r2	176.563198118743	1	pre_focus	2	Narrow pre_focus
2023206	block1	HumanSpeech	Post	gau2	Object	Narrow	r2	112.151753008902	1	on_focus	2	Narrow on_focus
2023206	block1	HumanSpeech	Post	zai2	Object	Narrow	r2	1254.48264591125	2	on_focus	2	Narrow on_focus
2023206	block1	HumanSpeech	Post	sau3	Subject	Broad	r2	121.709613138194	1	broad_focus	3	Broad focus
2023206	block1	HumanSpeech	Post	sau3	Subject	Broad	r2	102.664096749777	2	broad_focus	3	Broad focus
2023206	block1	HumanSpeech	Post	sik3	Verb	Broad	r2	116.612244897965	1	broad_focus	3	Broad focus
2023206	block1	HumanSpeech	Post	baak3	Object	Broad	r2	132.293361789152	1	broad_focus	3	Broad focus
2023206	block1	HumanSpeech	Post	baak3	Object	Broad	r2	245.934411500457	2	broad_focus	3	Broad focus
2023206	block1	HumanSpeech	Post	jyun2	Subject	Narrow	r2	190.358323885107	1	pre_focus	2	Narrow pre_focus
2023206	block1	HumanSpeech	Post	jyun2	Subject	Narrow	r2	74.1138175700371	2	pre_focus	2	Narrow pre_focus
2023206	block1	HumanSpeech	Post	mo2	Verb	Narrow	r2	147.629413670188	1	on_focus	2	Narrow on_focus
2023206	block1	HumanSpeech	Post	gau2	Object	Narrow	r2	91.4998380304155	1	post_focus	2	Narrow post_focus
2023206	block1	HumanSpeech	Post	zai2	Object	Narrow	r2	315.154128965503	2	post_focus	2	Narrow post_focus
2023206	block1	HumanSpeech	Post	zoeng1	Subject	Contrastive	r2	156.26133996966	1	pre_focus	1	Contrastive pre_focus
2023206	block1	HumanSpeech	Post	saang1	Subject	Contrastive	r2	178.477005617083	2	pre_focus	1	Contrastive pre_focus
2023206	block1	HumanSpeech	Post	tsa1	Verb	Contrastive	r2	195.858998144729	1	on_focus	1	Contrastive on_focus
2023206	block1	HumanSpeech	Post	fei1	Object	Contrastive	r2	190.117673331997	1	post_focus	1	Contrastive post_focus
2023206	block1	HumanSpeech	Post	gei1	Object	Contrastive	r2	338.611111111106	2	post_focus	1	Contrastive post_focus
2023206	block1	HumanSpeech	Post	zoeng1	Subject	Narrow	r2	208.476548514113	1	pre_focus	1	Narrow pre_focus
2023206	block1	HumanSpeech	Post	saang1	Subject	Narrow	r2	197.72824001393	2	pre_focus	1	Narrow pre_focus
2023206	block1	HumanSpeech	Post	tsa1	Verb	Narrow	r2	246.587463095409	1	on_focus	1	Narrow on_focus
2023206	block1	HumanSpeech	Post	fei1	Object	Narrow	r2	136.197797823456	1	post_focus	1	Narrow post_focus
2023206	block1	HumanSpeech	Post	gei1	Object	Narrow	r2	397.074829932023	2	post_focus	1	Narrow post_focus
2023206	block1	HumanSpeech	Post	jyun2	Subject	Contrastive	r2	162.809490946188	1	pre_focus	2	Contrastive pre_focus
2023206	block1	HumanSpeech	Post	jyun2	Subject	Contrastive	r2	154.696753906819	2	pre_focus	2	Contrastive pre_focus
2023206	block1	HumanSpeech	Post	mo2	Verb	Contrastive	r2	172.249217147169	1	on_focus	2	Contrastive on_focus
2023206	block1	HumanSpeech	Post	gau2	Object	Contrastive	r2	163.40797430081	1	post_focus	2	Contrastive post_focus
2023206	block1	HumanSpeech	Post	zai2	Object	Contrastive	r2	379.039140293798	2	post_focus	2	Contrastive post_focus
2023206	block1	HumanSpeech	Post	zoeng1	Subject	Broad	r2	151.502222582167	1	broad_focus	1	Broad focus
2023206	block1	HumanSpeech	Post	saang1	Subject	Broad	r2	154.868858654595	2	broad_focus	1	Broad focus
2023206	block1	HumanSpeech	Post	tsa1	Verb	Broad	r2	155.73615802748	1	broad_focus	1	Broad focus

**2023206**	block1	HumanSpeech	Post	fei1	Object	Broad	r2	197.94070714704	1	broad_focus	1	Broad focus
**2023206**	block1	HumanSpeech	Post	gei1	Object	Broad	r2	307.574178607638	2	broad_focus	1	Broad focus
**2023206**	block1	HumanSpeech	Post	sau3	Subject	Narrow	r2	99.0896907933347	1	on_focus	3	Narrow on_focus
**2023206**	block1	HumanSpeech	Post	sau3	Subject	Narrow	r2	133.187605538694	2	on_focus	3	Narrow on_focus
**2023206**	block1	HumanSpeech	Post	sik3	Verb	Narrow	r2	158.115322319418	1	post_focus	3	Narrow post_focus
**2023206**	block1	HumanSpeech	Post	baak3	Object	Narrow	r2	150.056861128292	1	post_focus	3	Narrow post_focus
**2023206**	block1	HumanSpeech	Post	baak3	Object	Narrow	r2	289.554865424407	2	post_focus	3	Narrow post_focus
**2023206**	block1	HumanSpeech	Post	jyun2	Subject	Broad	r2	90.7520786092277	1	broad_focus	2	Broad focus
**2023206**	block1	HumanSpeech	Post	jyun2	Subject	Broad	r2	120.237947993076	2	broad_focus	2	Broad focus
**2023206**	block1	HumanSpeech	Post	mo2	Verb	Broad	r2	171.420357616682	1	broad_focus	2	Broad focus
**2023206**	block1	HumanSpeech	Post	gau2	Object	Broad	r2	196.152683295566	1	broad_focus	2	Broad focus
**2023206**	block1	HumanSpeech	Post	zai2	Object	Broad	r2	309.233884029823	2	broad_focus	2	Broad focus
**2023206**	block1	HumanSpeech	Post	zoeng1	Subject	Contrastive	r2	156.403834260971	1	pre_focus	1	Contrastive pre_focus
**2023206**	block1	HumanSpeech	Post	saang1	Subject	Contrastive	r2	160.005127977115	2	pre_focus	1	Contrastive pre_focus
**2023206**	block1	HumanSpeech	Post	tsa1	Verb	Contrastive	r2	208.22654251964	1	pre_focus	1	Contrastive pre_focus
**2023206**	block1	HumanSpeech	Post	fei1	Object	Contrastive	r2	190.106953892666	1	on_focus	1	Contrastive on_focus
**2023206**	block1	HumanSpeech	Post	gei1	Object	Contrastive	r2	223.511904761892	2	on_focus	1	Contrastive on_focus
**2023206**	block1	HumanSpeech	Post	jyun2	Subject	Contrastive	r2	347.027588813319	1	on_focus	2	Contrastive on_focus
**2023206**	block1	HumanSpeech	Post	jyun2	Subject	Contrastive	r2	250.266439909296	2	on_focus	2	Contrastive on_focus
**2023206**	block1	HumanSpeech	Post	mo2	Verb	Contrastive	r2	265.90864917398	1	post_focus	2	Contrastive post_focus
**2023206**	block1	HumanSpeech	Post	gau2	Object	Contrastive	r2	266.385000054754	1	post_focus	2	Contrastive post_focus
**2023206**	block1	HumanSpeech	Post	zai2	Object	Contrastive	r2	236.139594049007	2	post_focus	2	Contrastive post_focus
**2023206**	block1	HumanSpeech	Post	sau3	Subject	Contrastive	r2	105.719313048212	1	on_focus	3	Contrastive on_focus
**2023206**	block1	HumanSpeech	Post	sau3	Subject	Contrastive	r2	105.527588813288	2	on_focus	3	Contrastive on_focus
**2023206**	block1	HumanSpeech	Post	sik3	Verb	Contrastive	r2	144.433106576003	1	post_focus	3	Contrastive post_focus
**2023206**	block1	HumanSpeech	Post	baak3	Object	Contrastive	r2	155.18903318906	1	post_focus	3	Contrastive post_focus
**2023206**	block1	HumanSpeech	Post	baak3	Object	Contrastive	r2	209.365079365057	2	post_focus	3	Contrastive post_focus
**2023206**	block1	HumanSpeech	Pre	jyun2	Subject	Narrow	r1	219.058201058203	1	pre_focus	2	Narrow pre_focus
**2023206**	block1	HumanSpeech	Pre	jyun2	Subject	Narrow	r1	243.072562358265	2	pre_focus	2	Narrow pre_focus
**2023206**	block1	HumanSpeech	Pre	mo2	Verb	Narrow	r1	303.665466186459	1	on_focus	2	Narrow on_focus
**2023206**	block1	HumanSpeech	Pre	gau2	Object	Narrow	r1	189.185941043092	1	post_focus	2	Narrow post_focus
**2023206**	block1	HumanSpeech	Pre	zai2	Object	Narrow	r1	309.436345966958	2	post_focus	2	Narrow post_focus
**2023206**	block1	HumanSpeech	Pre	jyun2	Subject	Narrow	r1	283.762282690844	1	pre_focus	2	Narrow pre_focus
**2023206**	block1	HumanSpeech	Pre	jyun2	Subject	Narrow	r1	255.528155706742	2	pre_focus	2	Narrow pre_focus
**2023206**	block1	HumanSpeech	Pre	mo2	Verb	Narrow	r1	228.767155985196	1	pre_focus	2	Narrow pre_focus
**2023206**	block1	HumanSpeech	Pre	gau2	Object	Narrow	r1	244.648043614603	1	on_focus	2	Narrow on_focus
**2023206**	block1	HumanSpeech	Pre	zai2	Object	Narrow	r1	395.157699443416	2	on_focus	2	Narrow on_focus
**2023206**	block1	HumanSpeech	Pre	zoeng1	Subject	Narrow	r1	181.080876795164	1	pre_focus	1	Narrow pre_focus
**2023206**	block1	HumanSpeech	Pre	saang1	Subject	Narrow	r1	213.43483425116	2	pre_focus	1	Narrow pre_focus
**2023206**	block1	HumanSpeech	Pre	tsa1	Verb	Narrow	r1	198.262901120046	1	on_focus	1	Narrow on_focus
**2023206**	block1	HumanSpeech	Pre	fei1	Object	Narrow	r1	189.200680272108	1	post_focus	1	Narrow post_focus
**2023206**	block1	HumanSpeech	Pre	gei1	Object	Narrow	r1	313.555967841666	2	post_focus	1	Narrow post_focus
**2023206**	block1	HumanSpeech	Pre	sau3	Subject	Narrow	r1	268.952003023429	1	pre_focus	3	Narrow pre_focus
**2023206**	block1	HumanSpeech	Pre	sau3	Subject	Narrow	r1	191.005145648006	2	pre_focus	3	Narrow pre_focus
**2023206**	block1	HumanSpeech	Pre	sik3	Verb	Narrow	r1	102.160673793321	1	on_focus	3	Narrow on_focus
**2023206**	block1	HumanSpeech	Pre	baak3	Object	Narrow	r1	96.3595724003881	1	post_focus	3	Narrow post_focus
**2023206**	block1	HumanSpeech	Pre	baak3	Object	Narrow	r1	106.712018140598	2	post_focus	3	Narrow post_focus
**2023206**	block1	HumanSpeech	Pre	sau3	Subject	Narrow	r1	181.703312277307	1	on_focus	3	Narrow on_focus
**2023206**	block1	HumanSpeech	Pre	sau3	Subject	Narrow	r1	145.07504589136	2	on_focus	3	Narrow on_focus
**2023206**	block1	HumanSpeech	Pre	sik3	Verb	Narrow	r1	116.167800453496	1	post_focus	3	Narrow post_focus
**2023206**	block1	HumanSpeech	Pre	baak3	Object	Narrow	r1	112.495748299324	1	post_focus	3	Narrow post_focus
**2023206**	block1	HumanSpeech	Pre	baak3	Object	Narrow	r1	111.926789763515	2	post_focus	3	Narrow post_focus
**2023206**	block1	HumanSpeech	Pre	sau3	Subject	Contrastive	r1	151.021352985623	1	pre_focus	3	Contrastive pre_focus
**2023206**	block1	HumanSpeech	Pre	sau3	Subject	Contrastive	r1	143.964589202682	2	pre_focus	3	Contrastive pre_focus
**2023206**	block1	HumanSpeech	Pre	sik3	Verb	Contrastive	r1	132.054421768686	1	on_focus	3	Contrastive on_focus
**2023206**	block1	HumanSpeech	Pre	baak3	Object	Contrastive	r1	140.413022351794	1	post_focus	3	Contrastive post_focus
**2023206**	block1	HumanSpeech	Pre	baak3	Object	Contrastive	r1	165.17762660618	2	post_focus	3	Contrastive post_focus
**2023206**	block1	HumanSpeech	Pre	zoeng1	Subject	Contrastive	r1	317.815030774227	1	on_focus	1	Contrastive on_focus
**2023206**	block1	HumanSpeech	Pre	saang1	Subject	Contrastive	r1	232.312925170078	2	on_focus	1	Contrastive on_focus
**2023206**	block1	HumanSpeech	Pre	tsa1	Verb	Contrastive	r1	191.496598639446	1	post_focus	1	Contrastive post_focus
**2023206**	block1	HumanSpeech	Pre	fei1	Object	Contrastive	r1	201.927437641729	1	post_focus	1	Contrastive post_focus
**2023206**	block1	HumanSpeech	Pre	gei1	Object	Contrastive	r1	239.587301587335	2	post_focus	1	Contrastive post_focus

2023206	block1	HumanSpeech	Pre	jyun2	Subject	Contrastive	r1	301.220710506442	1	pre_focus	2	Contrastive pre_focus
2023206	block1	HumanSpeech	Pre	jyun2	Subject	Contrastive	r1	407.449546485253	2	pre_focus	2	Contrastive pre_focus
2023206	block1	HumanSpeech	Pre	mo2	Verb	Contrastive	r1	362.947845804968	1	on_focus	2	Contrastive on_focus
2023206	block1	HumanSpeech	Pre	gau2	Object	Contrastive	r1	231.543461829148	1	post_focus	2	Contrastive post_focus
2023206	block1	HumanSpeech	Pre	zai2	Object	Contrastive	r1	293.606701940035	2	post_focus	2	Contrastive post_focus
2023206	block1	HumanSpeech	Pre	zoeng1	Subject	Broad	r1	276.876570447996	1	broad_focus	1	Broad focus
2023206	block1	HumanSpeech	Pre	saang1	Subject	Broad	r1	193.563869992431	2	broad_focus	1	Broad focus
2023206	block1	HumanSpeech	Pre	tsa1	Verb	Broad	r1	174.312547241129	1	broad_focus	1	Broad focus
2023206	block1	HumanSpeech	Pre	fei1	Object	Broad	r1	172.098377812659	1	broad_focus	1	Broad focus
2023206	block1	HumanSpeech	Pre	gei1	Object	Broad	r1	195.631141345416	2	broad_focus	1	Broad focus
2023206	block1	HumanSpeech	Pre	sau3	Subject	Narrow	r1	323.164001531381	1	pre_focus	3	Narrow pre_focus
2023206	block1	HumanSpeech	Pre	sau3	Subject	Narrow	r1	301.957381243142	2	pre_focus	3	Narrow pre_focus
2023206	block1	HumanSpeech	Pre	sik3	Verb	Narrow	r1	140.738851095989	1	pre_focus	3	Narrow pre_focus
2023206	block1	HumanSpeech	Pre	baak3	Object	Narrow	r1	164.421768707484	1	on_focus	3	Narrow on_focus
2023206	block1	HumanSpeech	Pre	baak3	Object	Narrow	r1	93.8810395953169	2	on_focus	3	Narrow on_focus
2023206	block1	HumanSpeech	Pre	zoeng1	Subject	Contrastive	r1	185.873015873028	1	pre_focus	1	Contrastive pre_focus
2023206	block1	HumanSpeech	Pre	saang1	Subject	Contrastive	r1	188.711262282652	2	pre_focus	1	Contrastive pre_focus
2023206	block1	HumanSpeech	Pre	tsa1	Verb	Contrastive	r1	177.451247165493	1	pre_focus	1	Contrastive pre_focus
2023206	block1	HumanSpeech	Pre	fei1	Object	Contrastive	r1	194.291383219934	1	on_focus	1	Contrastive on_focus
2023206	block1	HumanSpeech	Pre	gei1	Object	Contrastive	r1	265.301587301565	2	on_focus	1	Contrastive on_focus
2023206	block1	HumanSpeech	Pre	sau3	Subject	Contrastive	r1	247.291383219931	1	on_focus	3	Contrastive on_focus
2023206	block1	HumanSpeech	Pre	sau3	Subject	Contrastive	r1	184.082388510944	2	on_focus	3	Contrastive on_focus
2023206	block1	HumanSpeech	Pre	sik3	Verb	Contrastive	r1	121.041194255497	1	post_focus	3	Contrastive post_focus
2023206	block1	HumanSpeech	Pre	baak3	Object	Contrastive	r1	111.016156462597	1	post_focus	3	Contrastive post_focus
2023206	block1	HumanSpeech	Pre	baak3	Object	Contrastive	r1	110.358451072784	2	post_focus	3	Contrastive post_focus
2023206	block1	HumanSpeech	Pre	jyun2	Subject	Contrastive	r1	316.110571212619	1	on_focus	2	Contrastive on_focus
2023206	block1	HumanSpeech	Pre	jyun2	Subject	Contrastive	r1	252.93607186461	2	on_focus	2	Contrastive on_focus
2023206	block1	HumanSpeech	Pre	mo2	Verb	Contrastive	r1	261.09977324262	1	post_focus	2	Contrastive post_focus
2023206	block1	HumanSpeech	Pre	gau2	Object	Contrastive	r1	192.928262214025	1	post_focus	2	Contrastive post_focus
2023206	block1	HumanSpeech	Pre	zai2	Object	Contrastive	r1	307.392290249425	2	post_focus	2	Contrastive post_focus
2023206	block1	HumanSpeech	Pre	zoeng1	Subject	Narrow	r1	173.500309214546	1	pre_focus	1	Narrow pre_focus
2023206	block1	HumanSpeech	Pre	saang1	Subject	Narrow	r1	184.209784924064	2	pre_focus	1	Narrow pre_focus
2023206	block1	HumanSpeech	Pre	tsa1	Verb	Narrow	r1	176.552532123992	1	pre_focus	1	Narrow pre_focus
2023206	block1	HumanSpeech	Pre	fei1	Object	Narrow	r1	207.82802009694	1	on_focus	1	Narrow on_focus
2023206	block1	HumanSpeech	Pre	gei1	Object	Narrow	r1	302.072400388738	2	on_focus	1	Narrow on_focus
2023206	block1	HumanSpeech	Pre	jyun2	Subject	Narrow	r1	310.604686318982	1	on_focus	2	Narrow on_focus
2023206	block1	HumanSpeech	Pre	jyun2	Subject	Narrow	r1	265.389266817806	2	on_focus	2	Narrow on_focus
2023206	block1	HumanSpeech	Pre	mo2	Verb	Narrow	r1	324.210128495849	1	post_focus	2	Narrow post_focus
2023206	block1	HumanSpeech	Pre	gau2	Object	Narrow	r1	222.639455782314	1	post_focus	2	Narrow post_focus
2023206	block1	HumanSpeech	Pre	zai2	Object	Narrow	r1	283.343051506279	2	post_focus	2	Narrow post_focus
2023206	block1	HumanSpeech	Pre	zoeng1	Subject	Narrow	r1	189.799044756285	1	on_focus	1	Narrow on_focus
2023206	block1	HumanSpeech	Pre	saang1	Subject	Narrow	r1	175.9586797682	2	on_focus	1	Narrow on_focus
2023206	block1	HumanSpeech	Pre	tsa1	Verb	Narrow	r1	156.857375428842	1	post_focus	1	Narrow post_focus
2023206	block1	HumanSpeech	Pre	fei1	Object	Narrow	r1	176.60052910054	1	post_focus	1	Narrow post_focus
2023206	block1	HumanSpeech	Pre	gei1	Object	Narrow	r1	286.198034769484	2	post_focus	1	Narrow post_focus
2023206	block1	HumanSpeech	Pre	sau3	Subject	Broad	r1	370.103433186159	1	broad_focus	3	Broad focus
2023206	block1	HumanSpeech	Pre	sau3	Subject	Broad	r1	212.99571680521	2	broad_focus	3	Broad focus
2023206	block1	HumanSpeech	Pre	sik3	Verb	Broad	r1	140.918367346899	1	broad_focus	3	Broad focus
2023206	block1	HumanSpeech	Pre	baak3	Object	Broad	r1	163.148609037137	1	broad_focus	3	Broad focus
2023206	block1	HumanSpeech	Pre	baak3	Object	Broad	r1	160.26947977258	2	broad_focus	3	Broad focus
2023206	block1	HumanSpeech	Pre	jyun2	Subject	Broad	r1	307.755102040801	1	broad_focus	2	Broad focus
2023206	block1	HumanSpeech	Pre	jyun2	Subject	Broad	r1	271.471088435362	2	broad_focus	2	Broad focus
2023206	block1	HumanSpeech	Pre	mo2	Verb	Broad	r1	210.979179550577	1	broad_focus	2	Broad focus
2023206	block1	HumanSpeech	Pre	gau2	Object	Broad	r1	195.854659324084	1	broad_focus	2	Broad focus
2023206	block1	HumanSpeech	Pre	zai2	Object	Broad	r1	338.367346938753	2	broad_focus	2	Broad focus
2023206	block1	HumanSpeech	Pre	sau3	Subject	Contrastive	r1	127.046755210017	1	pre_focus	3	Contrastive pre_focus
2023206	block1	HumanSpeech	Pre	sau3	Subject	Contrastive	r1	150.034636565238	2	pre_focus	3	Contrastive pre_focus
2023206	block1	HumanSpeech	Pre	sik3	Verb	Contrastive	r1	131.744520030225	1	pre_focus	3	Contrastive pre_focus
2023206	block1	HumanSpeech	Pre	baak3	Object	Contrastive	r1	161.284271284273	1	on_focus	3	Contrastive on_focus
2023206	block1	HumanSpeech	Pre	baak3	Object	Contrastive	r1	163.388476602734	2	on_focus	3	Contrastive on_focus
2023206	block1	HumanSpeech	Pre	jyun2	Subject	Contrastive	r1	240.457294028715	1	pre_focus	2	Contrastive pre_focus
2023206	block1	HumanSpeech	Pre	jyun2	Subject	Contrastive	r1	250.343663391277	2	pre_focus	2	Contrastive pre_focus

**2023206**	block1	HumanSpeech	Pre	mo2	Verb	Contrastive	r1	283.806907378334	1	pre_focus	2	Contrastive pre_focus
**2023206**	block1	HumanSpeech	Pre	gau2	Object	Contrastive	r1	195.951159951164	1	on_focus	2	Contrastive on_focus
**2023206**	block1	HumanSpeech	Pre	zai2	Object	Contrastive	r1	403.96825396823	2	on_focus	2	Contrastive on_focus
**2023206**	block1	HumanSpeech	Pre	zoeng1	Subject	Contrastive	r1	234.546485260751	1	pre_focus	1	Contrastive pre_focus
**2023206**	block1	HumanSpeech	Pre	saang1	Subject	Contrastive	r1	429.440665154971	2	pre_focus	1	Contrastive pre_focus
**2023206**	block1	HumanSpeech	Pre	tsa1	Verb	Contrastive	r1	216.650469711681	1	on_focus	1	Contrastive on_focus
**2023206**	block1	HumanSpeech	Pre	fei1	Object	Contrastive	r1	187.719468739829	1	post_focus	1	Contrastive post_focus
**2023206**	block1	HumanSpeech	Pre	gei1	Object	Contrastive	r1	274.628684807226	2	post_focus	1	Contrastive post_focus
**2023206**	block1	HumanSpeech	Pre	jyun2	Subject	Narrow	r2	318.628117913875	1	on_focus	2	Narrow on_focus
**2023206**	block1	HumanSpeech	Pre	jyun2	Subject	Narrow	r2	261.882086167759	2	on_focus	2	Narrow on_focus
**2023206**	block1	HumanSpeech	Pre	mo2	Verb	Narrow	r2	247.607709750582	1	post_focus	2	Narrow post_focus
**2023206**	block1	HumanSpeech	Pre	gau2	Object	Narrow	r2	213.553287981881	1	post_focus	2	Narrow post_focus
**2023206**	block1	HumanSpeech	Pre	zai2	Object	Narrow	r2	305.108519598321	2	post_focus	2	Narrow post_focus
**2023206**	block1	HumanSpeech	Pre	zoeng1	Subject	Contrastive	r2	180.970017636639	1	on_focus	1	Contrastive on_focus
**2023206**	block1	HumanSpeech	Pre	saang1	Subject	Contrastive	r2	186.324910916767	2	on_focus	1	Contrastive on_focus
**2023206**	block1	HumanSpeech	Pre	tsa1	Verb	Contrastive	r2	172.072162198219	1	post_focus	1	Contrastive post_focus
**2023206**	block1	HumanSpeech	Pre	fei1	Object	Contrastive	r2	179.135487528356	1	post_focus	1	Contrastive post_focus
**2023206**	block1	HumanSpeech	Pre	gei1	Object	Contrastive	r2	246.213151927407	2	post_focus	1	Contrastive post_focus
**2023206**	block1	HumanSpeech	Pre	zoeng1	Subject	Broad	r2	191.926681783855	1	broad_focus	1	Broad focus
**2023206**	block1	HumanSpeech	Pre	saang1	Subject	Broad	r2	196.587301587272	2	broad_focus	1	Broad focus
**2023206**	block1	HumanSpeech	Pre	tsa1	Verb	Broad	r2	183.952191987885	1	broad_focus	1	Broad focus
**2023206**	block1	HumanSpeech	Pre	fei1	Object	Broad	r2	170.929705215428	1	broad_focus	1	Broad focus
**2023206**	block1	HumanSpeech	Pre	gei1	Object	Broad	r2	241.862649821883	2	broad_focus	1	Broad focus
**2023206**	block1	HumanSpeech	Pre	sau3	Subject	Broad	r2	293.669690098227	1	broad_focus	3	Broad focus
**2023206**	block1	HumanSpeech	Pre	sau3	Subject	Broad	r2	252.448979591861	2	broad_focus	3	Broad focus
**2023206**	block1	HumanSpeech	Pre	sik3	Verb	Broad	r2	161.898283122753	1	broad_focus	3	Broad focus
**2023206**	block1	HumanSpeech	Pre	baak3	Object	Broad	r2	133.043781615243	1	broad_focus	3	Broad focus
**2023206**	block1	HumanSpeech	Pre	baak3	Object	Broad	r2	105.65597667636	2	broad_focus	3	Broad focus
**2023206**	block1	HumanSpeech	Pre	zoeng1	Subject	Contrastive	r2	223.251967453621	1	pre_focus	1	Contrastive pre_focus
**2023206**	block1	HumanSpeech	Pre	saang1	Subject	Contrastive	r2	212.559118885622	2	pre_focus	1	Contrastive pre_focus
**2023206**	block1	HumanSpeech	Pre	tsa1	Verb	Contrastive	r2	190.911262282725	1	pre_focus	1	Contrastive pre_focus
**2023206**	block1	HumanSpeech	Pre	fei1	Object	Contrastive	r2	219.426303854902	1	on_focus	1	Contrastive on_focus
**2023206**	block1	HumanSpeech	Pre	gei1	Object	Contrastive	r2	291.787603930459	2	on_focus	1	Contrastive on_focus
**2023206**	block1	HumanSpeech	Pre	zoeng1	Subject	Narrow	r2	208.775728240028	1	pre_focus	1	Narrow pre_focus
**2023206**	block1	HumanSpeech	Pre	saang1	Subject	Narrow	r2	257.288359788333	2	pre_focus	1	Narrow pre_focus
**2023206**	block1	HumanSpeech	Pre	tsa1	Verb	Narrow	r2	454.111866969015	1	pre_focus	1	Narrow pre_focus
**2023206**	block1	HumanSpeech	Pre	fei1	Object	Narrow	r2	200.980347694667	1	on_focus	1	Narrow on_focus
**2023206**	block1	HumanSpeech	Pre	gei1	Object	Narrow	r2	385.769355361163	2	on_focus	1	Narrow on_focus
**2023206**	block1	HumanSpeech	Pre	jyun2	Subject	Contrastive	r2	493.605442176886	1	on_focus	2	Contrastive on_focus
**2023206**	block1	HumanSpeech	Pre	jyun2	Subject	Contrastive	r2	346.317460317437	2	on_focus	2	Contrastive on_focus
**2023206**	block1	HumanSpeech	Pre	mo2	Verb	Contrastive	r2	381.798185941022	1	post_focus	2	Contrastive post_focus
**2023206**	block1	HumanSpeech	Pre	gau2	Object	Contrastive	r2	247.644242882359	1	post_focus	2	Contrastive post_focus
**2023206**	block1	HumanSpeech	Pre	zai2	Object	Contrastive	r2	346.219954648575	2	post_focus	2	Contrastive post_focus
**2023206**	block1	HumanSpeech	Pre	sau3	Subject	Contrastive	r2	220.585077831458	1	pre_focus	3	Contrastive pre_focus
**2023206**	block1	HumanSpeech	Pre	sau3	Subject	Contrastive	r2	194.022202287499	2	pre_focus	3	Contrastive pre_focus
**2023206**	block1	HumanSpeech	Pre	sik3	Verb	Contrastive	r2	166.077097505706	1	pre_focus	3	Contrastive pre_focus
**2023206**	block1	HumanSpeech	Pre	baak3	Object	Contrastive	r2	128.001609245871	1	on_focus	3	Contrastive on_focus
**2023206**	block1	HumanSpeech	Pre	baak3	Object	Contrastive	r2	176.261429302883	2	on_focus	3	Contrastive on_focus
**2023206**	block1	HumanSpeech	Pre	jyun2	Subject	Narrow	r2	226.866213151936	1	pre_focus	2	Narrow pre_focus
**2023206**	block1	HumanSpeech	Pre	jyun2	Subject	Narrow	r2	259.461181297922	2	pre_focus	2	Narrow pre_focus
**2023206**	block1	HumanSpeech	Pre	mo2	Verb	Narrow	r2	216.290249433087	1	on_focus	2	Narrow on_focus
**2023206**	block1	HumanSpeech	Pre	gau2	Object	Narrow	r2	202.749811035517	1	post_focus	2	Narrow post_focus
**2023206**	block1	HumanSpeech	Pre	zai2	Object	Narrow	r2	259.267447719878	2	post_focus	2	Narrow post_focus
**2023206**	block1	HumanSpeech	Pre	sau3	Subject	Contrastive	r2	175.662590433035	1	pre_focus	3	Contrastive pre_focus
**2023206**	block1	HumanSpeech	Pre	sau3	Subject	Contrastive	r2	156.746031746025	2	pre_focus	3	Contrastive pre_focus
**2023206**	block1	HumanSpeech	Pre	sik3	Verb	Contrastive	r2	144.499244142139	1	on_focus	3	Contrastive on_focus
**2023206**	block1	HumanSpeech	Pre	baak3	Object	Contrastive	r2	148.963970773536	1	post_focus	3	Contrastive post_focus
**2023206**	block1	HumanSpeech	Pre	baak3	Object	Contrastive	r2	197.493197278902	2	post_focus	3	Contrastive post_focus
**2023206**	block1	HumanSpeech	Pre	sau3	Subject	Narrow	r2	260.128495842764	1	pre_focus	3	Narrow pre_focus
**2023206**	block1	HumanSpeech	Pre	sau3	Subject	Narrow	r2	158.967888230507	2	pre_focus	3	Narrow pre_focus
**2023206**	block1	HumanSpeech	Pre	sik3	Verb	Narrow	r2	108.219954648519	1	on_focus	3	Narrow on_focus
**2023206**	block1	HumanSpeech	Pre	baak3	Object	Narrow	r2	127.851203973648	1	post_focus	3	Narrow post_focus

**2023206**	block1	HumanSpeech	Pre	baak3	Object	Narrow	r2	147.356009070336	2	post_focus	3	Narrow post_focus
**2023206**	block1	HumanSpeech	Pre	zoeng1	Subject	Contrastive	r2	206.775006298869	1	pre_focus	1	Contrastive pre_focus
**2023206**	block1	HumanSpeech	Pre	saang1	Subject	Contrastive	r2	208.843537414964	2	pre_focus	1	Contrastive pre_focus
**2023206**	block1	HumanSpeech	Pre	tsa1	Verb	Contrastive	r2	192.474624770568	1	on_focus	1	Contrastive on_focus
**2023206**	block1	HumanSpeech	Pre	fei1	Object	Contrastive	r2	227.51613465897	1	post_focus	1	Contrastive post_focus
**2023206**	block1	HumanSpeech	Pre	gei1	Object	Contrastive	r2	335.130213701689	2	post_focus	1	Contrastive post_focus
**2023206**	block1	HumanSpeech	Pre	sau3	Subject	Narrow	r2	272.671201814092	1	pre_focus	3	Narrow pre_focus
**2023206**	block1	HumanSpeech	Pre	sau3	Subject	Narrow	r2	249.368102796666	2	pre_focus	3	Narrow pre_focus
**2023206**	block1	HumanSpeech	Pre	sik3	Verb	Narrow	r2	177.682539682564	1	pre_focus	3	Narrow pre_focus
**2023206**	block1	HumanSpeech	Pre	baak3	Object	Narrow	r2	164.390687309435	1	on_focus	3	Narrow on_focus
**2023206**	block1	HumanSpeech	Pre	baak3	Object	Narrow	r2	187.596028310338	2	on_focus	3	Narrow on_focus
**2023206**	block1	HumanSpeech	Pre	jyun2	Subject	Contrastive	r2	281.507936507978	1	pre_focus	2	Contrastive pre_focus
**2023206**	block1	HumanSpeech	Pre	jyun2	Subject	Contrastive	r2	247.804232804185	2	pre_focus	2	Contrastive pre_focus
**2023206**	block1	HumanSpeech	Pre	mo2	Verb	Contrastive	r2	286.31393298059	1	on_focus	2	Contrastive on_focus
**2023206**	block1	HumanSpeech	Pre	gau2	Object	Contrastive	r2	213.859914336126	1	post_focus	2	Contrastive post_focus
**2023206**	block1	HumanSpeech	Pre	zai2	Object	Contrastive	r2	376.294406651539	2	post_focus	2	Contrastive post_focus
**2023206**	block1	HumanSpeech	Pre	jyun2	Subject	Narrow	r2	310.402494331072	1	pre_focus	2	Narrow pre_focus
**2023206**	block1	HumanSpeech	Pre	jyun2	Subject	Narrow	r2	305.133219954655	2	pre_focus	2	Narrow pre_focus
**2023206**	block1	HumanSpeech	Pre	mo2	Verb	Narrow	r2	219.78231292519	1	pre_focus	2	Narrow pre_focus
**2023206**	block1	HumanSpeech	Pre	gau2	Object	Narrow	r2	223.107371603646	1	on_focus	2	Narrow on_focus
**2023206**	block1	HumanSpeech	Pre	zai2	Object	Narrow	r2	360.621963070912	2	on_focus	2	Narrow on_focus
**2023206**	block1	HumanSpeech	Pre	zoeng1	Subject	Narrow	r2	253.723711706868	1	on_focus	1	Narrow on_focus
**2023206**	block1	HumanSpeech	Pre	saang1	Subject	Narrow	r2	239.708574787983	2	on_focus	1	Narrow on_focus
**2023206**	block1	HumanSpeech	Pre	tsa1	Verb	Narrow	r2	211.866969009861	1	post_focus	1	Narrow post_focus
**2023206**	block1	HumanSpeech	Pre	fei1	Object	Narrow	r2	188.701814058959	1	post_focus	1	Narrow post_focus
**2023206**	block1	HumanSpeech	Pre	gei1	Object	Narrow	r2	236.356764928246	2	post_focus	1	Narrow post_focus
**2023206**	block1	HumanSpeech	Pre	jyun2	Subject	Contrastive	r2	278.225623582784	1	pre_focus	2	Contrastive pre_focus
**2023206**	block1	HumanSpeech	Pre	jyun2	Subject	Contrastive	r2	288.779375386525	2	pre_focus	2	Contrastive pre_focus
**2023206**	block1	HumanSpeech	Pre	mo2	Verb	Contrastive	r2	402.276077097497	1	pre_focus	2	Contrastive pre_focus
**2023206**	block1	HumanSpeech	Pre	gau2	Object	Contrastive	r2	209.067573696132	1	on_focus	2	Contrastive on_focus
**2023206**	block1	HumanSpeech	Pre	zai2	Object	Contrastive	r2	344.326942898419	2	on_focus	2	Contrastive on_focus
**2023206**	block1	HumanSpeech	Pre	sau3	Subject	Narrow	r2	224.76190476192	1	on_focus	3	Narrow on_focus
**2023206**	block1	HumanSpeech	Pre	sau3	Subject	Narrow	r2	183.616780045327	2	on_focus	3	Narrow on_focus
**2023206**	block1	HumanSpeech	Pre	sik3	Verb	Narrow	r2	116.808390022697	1	post_focus	3	Narrow post_focus
**2023206**	block1	HumanSpeech	Pre	baak3	Object	Narrow	r2	139.648033126321	1	post_focus	3	Narrow post_focus
**2023206**	block1	HumanSpeech	Pre	baak3	Object	Narrow	r2	153.670634920672	2	post_focus	3	Narrow post_focus
**2023206**	block1	HumanSpeech	Pre	jyun2	Subject	Broad	r2	280.491307634179	1	broad_focus	2	Broad focus
**2023206**	block1	HumanSpeech	Pre	jyun2	Subject	Broad	r2	269.461451247196	2	broad_focus	2	Broad focus
**2023206**	block1	HumanSpeech	Pre	mo2	Verb	Broad	r2	286.45124716553	1	broad_focus	2	Broad focus
**2023206**	block1	HumanSpeech	Pre	gau2	Object	Broad	r2	233.874716553316	1	broad_focus	2	Broad focus
**2023206**	block1	HumanSpeech	Pre	zai2	Object	Broad	r2	352.514739229036	2	broad_focus	2	Broad focus
**2023206**	block1	HumanSpeech	Pre	zoeng1	Subject	Narrow	r2	191.16213151932	1	pre_focus	1	Narrow pre_focus
**2023206**	block1	HumanSpeech	Pre	saang1	Subject	Narrow	r2	198.711734693859	2	pre_focus	1	Narrow pre_focus
**2023206**	block1	HumanSpeech	Pre	tsa1	Verb	Narrow	r2	184.761904761899	1	on_focus	1	Narrow on_focus
**2023206**	block1	HumanSpeech	Pre	fei1	Object	Narrow	r2	204.106976123796	1	post_focus	1	Narrow post_focus
**2023206**	block1	HumanSpeech	Pre	gei1	Object	Narrow	r2	298.199798437963	2	post_focus	1	Narrow post_focus
**2023206**	block1	HumanSpeech	Pre	sau3	Subject	Contrastive	r2	231.708994708981	1	on_focus	3	Contrastive on_focus
**2023206**	block1	HumanSpeech	Pre	sau3	Subject	Contrastive	r2	179.47341899719	2	on_focus	3	Contrastive on_focus
**2023206**	block1	HumanSpeech	Pre	sik3	Verb	Contrastive	r2	139.532879818603	1	post_focus	3	Contrastive post_focus
**2023206**	block1	HumanSpeech	Pre	baak3	Object	Contrastive	r2	114.342403628086	1	post_focus	3	Contrastive post_focus
**2023206**	block1	HumanSpeech	Pre	baak3	Object	Contrastive	r2	91.8102796674134	2	post_focus	3	Contrastive post_focus
**2023206**	block2	HumanSpeech	Post	ngaa5	Subject	Narrow	r1	219.012398368733	1	on_focus	5	Narrow on_focus
**2023206**	block2	HumanSpeech	Post	ngaa5	Subject	Narrow	r1	251.827514888731	2	on_focus	5	Narrow on_focus
**2023206**	block2	HumanSpeech	Post	maai5	Verb	Narrow	r1	175.835513692661	1	post_focus	5	Narrow post_focus
**2023206**	block2	HumanSpeech	Post	pou5	Object	Narrow	r1	137.459154602013	1	post_focus	5	Narrow post_focus
**2023206**	block2	HumanSpeech	Post	pou5	Object	Narrow	r1	285.857088867289	2	post_focus	5	Narrow post_focus
**2023206**	block2	HumanSpeech	Post	ma4	Subject	Contrastive	r1	238.338057445205	1	on_focus	4	Contrastive on_focus
**2023206**	block2	HumanSpeech	Post	ma4	Subject	Contrastive	r1	192.071995464858	2	on_focus	4	Contrastive on_focus
**2023206**	block2	HumanSpeech	Post	fu4	Verb	Contrastive	r1	131.360071806498	1	post_focus	4	Contrastive post_focus
**2023206**	block2	HumanSpeech	Post	maang4	Object	Contrastive	r1	299.262471655325	1	post_focus	4	Contrastive post_focus
**2023206**	block2	HumanSpeech	Post	jan4	Object	Contrastive	r1	202.113378684814	2	post_focus	4	Contrastive post_focus
**2023206**	block2	HumanSpeech	Post	ngaa5	Subject	Contrastive	r1	168.594402673364	1	pre_focus	5	Contrastive pre_focus

2023206	block2	HumanSpeech	Post	ngaa5	Subject	Contrastive	r1	222.976255636368	2	pre_focus	5	Contrastive pre_focus
2023206	block2	HumanSpeech	Post	maai5	Verb	Contrastive	r1	281.211708925994	1	on_focus	5	Contrastive on_focus
2023206	block2	HumanSpeech	Post	pou5	Object	Contrastive	r1	229.417233560085	1	post_focus	5	Contrastive post_focus
2023206	block2	HumanSpeech	Post	pou5	Object	Contrastive	r1	340.963718820873	2	post_focus	5	Contrastive post_focus
2023206	block2	HumanSpeech	Post	lok6	Subject	Narrow	r1	133.765684051411	1	on_focus	6	Narrow on_focus
2023206	block2	HumanSpeech	Post	lok6	Subject	Narrow	r1	208.390022675729	2	on_focus	6	Narrow on_focus
2023206	block2	HumanSpeech	Post	waa6	Verb	Narrow	r1	172.962423064462	1	post_focus	6	Narrow post_focus
2023206	block2	HumanSpeech	Post	jyut6	Object	Narrow	r1	126.167485512724	1	post_focus	6	Narrow post_focus
2023206	block2	HumanSpeech	Post	loeng6	Object	Narrow	r1	314.831227729201	2	post_focus	6	Narrow post_focus
2023206	block2	HumanSpeech	Post	ma4	Subject	Narrow	r1	217.18909208704	1	on_focus	4	Narrow on_focus
2023206	block2	HumanSpeech	Post	ma4	Subject	Narrow	r1	172.420053491493	2	on_focus	4	Narrow on_focus
2023206	block2	HumanSpeech	Post	fu4	Verb	Narrow	r1	112.847694633388	1	post_focus	4	Narrow post_focus
2023206	block2	HumanSpeech	Post	maang4	Object	Narrow	r1	189.030612244892	1	post_focus	4	Narrow post_focus
2023206	block2	HumanSpeech	Post	jan4	Object	Narrow	r1	139.760807548839	2	post_focus	4	Narrow post_focus
2023206	block2	HumanSpeech	Post	ma4	Subject	Contrastive	r1	175.756781725056	1	pre_focus	4	Contrastive pre_focus
2023206	block2	HumanSpeech	Post	ma4	Subject	Contrastive	r1	201.49433106576	2	pre_focus	4	Contrastive pre_focus
2023206	block2	HumanSpeech	Post	fu4	Verb	Contrastive	r1	168.757942737528	1	on_focus	4	Contrastive on_focus
2023206	block2	HumanSpeech	Post	maang4	Object	Contrastive	r1	299.855820105819	1	post_focus	4	Contrastive post_focus
2023206	block2	HumanSpeech	Post	jan4	Object	Contrastive	r1	505.738938310373	2	post_focus	4	Contrastive post_focus
2023206	block2	HumanSpeech	Post	ngaa5	Subject	Contrastive	r1	242.171147824195	1	on_focus	5	Contrastive on_focus
2023206	block2	HumanSpeech	Post	ngaa5	Subject	Contrastive	r1	314.725376211101	2	on_focus	5	Contrastive on_focus
2023206	block2	HumanSpeech	Post	maai5	Verb	Contrastive	r1	324.467498110351	1	post_focus	5	Contrastive post_focus
2023206	block2	HumanSpeech	Post	pou5	Object	Contrastive	r1	148.648904006052	1	post_focus	5	Contrastive post_focus
2023206	block2	HumanSpeech	Post	pou5	Object	Contrastive	r1	316.09829439023	2	post_focus	5	Contrastive post_focus
2023206	block2	HumanSpeech	Post	lok6	Subject	Broad	r1	125.40629005224	1	broad_focus	6	Broad focus
2023206	block2	HumanSpeech	Post	lok6	Subject	Broad	r1	174.401927437629	2	broad_focus	6	Broad focus
2023206	block2	HumanSpeech	Post	waa6	Verb	Broad	r1	140.823412698381	1	broad_focus	6	Broad focus
2023206	block2	HumanSpeech	Post	jyut6	Object	Broad	r1	185.416012558846	1	broad_focus	6	Broad focus
2023206	block2	HumanSpeech	Post	loeng6	Object	Broad	r1	429.460821365581	2	broad_focus	6	Broad focus
2023206	block2	HumanSpeech	Post	lok6	Subject	Narrow	r1	125.008721437297	1	pre_focus	6	Narrow pre_focus
2023206	block2	HumanSpeech	Post	lok6	Subject	Narrow	r1	153.499622071052	2	pre_focus	6	Narrow pre_focus
2023206	block2	HumanSpeech	Post	waa6	Verb	Narrow	r1	128.997543461821	1	pre_focus	6	Narrow pre_focus
2023206	block2	HumanSpeech	Post	jyut6	Object	Narrow	r1	117.133786848086	1	on_focus	6	Narrow on_focus
2023206	block2	HumanSpeech	Post	loeng6	Object	Narrow	r1	468.424036281192	2	on_focus	6	Narrow on_focus
2023206	block2	HumanSpeech	Post	ma4	Subject	Contrastive	r1	181.678533107089	1	pre_focus	4	Contrastive pre_focus
2023206	block2	HumanSpeech	Post	ma4	Subject	Contrastive	r1	204.517825648765	2	pre_focus	4	Contrastive pre_focus
2023206	block2	HumanSpeech	Post	fu4	Verb	Contrastive	r1	197.423469387758	1	pre_focus	4	Contrastive pre_focus
2023206	block2	HumanSpeech	Post	maang4	Object	Contrastive	r1	175.789871504179	1	on_focus	4	Contrastive on_focus
2023206	block2	HumanSpeech	Post	jan4	Object	Contrastive	r1	212.004535147372	2	on_focus	4	Contrastive on_focus
2023206	block2	HumanSpeech	Post	ngaa5	Subject	Contrastive	r1	237.30684394971	1	pre_focus	5	Contrastive pre_focus
2023206	block2	HumanSpeech	Post	ngaa5	Subject	Contrastive	r1	161.164842748718	2	pre_focus	5	Contrastive pre_focus
2023206	block2	HumanSpeech	Post	maai5	Verb	Contrastive	r1	243.705593348437	1	pre_focus	5	Contrastive pre_focus
2023206	block2	HumanSpeech	Post	pou5	Object	Contrastive	r1	209.309145880553	1	on_focus	5	Contrastive on_focus
2023206	block2	HumanSpeech	Post	pou5	Object	Contrastive	r1	138.010582010565	2	on_focus	5	Contrastive on_focus
2023206	block2	HumanSpeech	Post	lok6	Subject	Contrastive	r1	168.122448979602	1	pre_focus	6	Contrastive pre_focus
2023206	block2	HumanSpeech	Post	lok6	Subject	Contrastive	r1	207.874321445729	2	pre_focus	6	Contrastive pre_focus
2023206	block2	HumanSpeech	Post	waa6	Verb	Contrastive	r1	144.79969765685	1	on_focus	6	Contrastive on_focus
2023206	block2	HumanSpeech	Post	jyut6	Object	Contrastive	r1	104.816559102261	1	post_focus	6	Contrastive post_focus
2023206	block2	HumanSpeech	Post	loeng6	Object	Contrastive	r1	351.775716347163	2	post_focus	6	Contrastive post_focus
2023206	block2	HumanSpeech	Post	ma4	Subject	Narrow	r1	150.586683158082	1	pre_focus	4	Narrow pre_focus
2023206	block2	HumanSpeech	Post	ma4	Subject	Narrow	r1	185.314345518407	2	pre_focus	4	Narrow pre_focus
2023206	block2	HumanSpeech	Post	fu4	Verb	Narrow	r1	104.146636432347	1	on_focus	4	Narrow on_focus
2023206	block2	HumanSpeech	Post	maang4	Object	Narrow	r1	241.187112207513	1	post_focus	4	Narrow post_focus
2023206	block2	HumanSpeech	Post	jan4	Object	Narrow	r1	219.152899254937	2	post_focus	4	Narrow post_focus
2023206	block2	HumanSpeech	Post	lok6	Subject	Contrastive	r1	170.793650793627	1	on_focus	6	Contrastive on_focus
2023206	block2	HumanSpeech	Post	lok6	Subject	Contrastive	r1	171.912320483756	2	on_focus	6	Contrastive on_focus
2023206	block2	HumanSpeech	Post	waa6	Verb	Contrastive	r1	170.204081632647	1	post_focus	6	Contrastive post_focus
2023206	block2	HumanSpeech	Post	jyut6	Object	Contrastive	r1	157.364701436109	1	post_focus	6	Contrastive post_focus
2023206	block2	HumanSpeech	Post	loeng6	Object	Contrastive	r1	240.334845049148	2	post_focus	6	Contrastive post_focus
2023206	block2	HumanSpeech	Post	ma4	Subject	Broad	r1	176.829004329022	1	broad_focus	4	Broad focus
2023206	block2	HumanSpeech	Post	ma4	Subject	Broad	r1	201.985871271575	2	broad_focus	4	Broad focus
2023206	block2	HumanSpeech	Post	fu4	Verb	Broad	r1	156.942554799713	1	broad_focus	4	Broad focus

2023206	block2	HumanSpeech	Post	maang4	Object	Broad	r1	372.202569916851	1	broad_focus	4	Broad focus
2023206	block2	HumanSpeech	Post	jan4	Object	Broad	r1	285.24489795916	2	broad_focus	4	Broad focus
2023206	block2	HumanSpeech	Post	ngaa5	Subject	Broad	r1	223.019995877138	1	broad_focus	5	Broad focus
2023206	block2	HumanSpeech	Post	ngaa5	Subject	Broad	r1	211.149247577822	2	broad_focus	5	Broad focus
2023206	block2	HumanSpeech	Post	maai5	Verb	Broad	r1	273.189090451012	1	broad_focus	5	Broad focus
2023206	block2	HumanSpeech	Post	pou5	Object	Broad	r1	137.536848072585	1	broad_focus	5	Broad focus
2023206	block2	HumanSpeech	Post	pou5	Object	Broad	r1	332.597127740001	2	broad_focus	5	Broad focus
2023206	block2	HumanSpeech	Post	lok6	Subject	Narrow	r1	104.798185941036	1	pre_focus	6	Narrow pre_focus
2023206	block2	HumanSpeech	Post	lok6	Subject	Narrow	r1	168.499622071039	2	pre_focus	6	Narrow pre_focus
2023206	block2	HumanSpeech	Post	waa6	Verb	Narrow	r1	87.6554662268916	1	on_focus	6	Narrow on_focus
2023206	block2	HumanSpeech	Post	jyut6	Object	Narrow	r1	143.667800453528	1	post_focus	6	Narrow post_focus
2023206	block2	HumanSpeech	Post	loeng6	Object	Narrow	r1	442.970521541952	2	post_focus	6	Narrow post_focus
2023206	block2	HumanSpeech	Post	ngaa5	Subject	Narrow	r1	173.399986257152	1	pre_focus	5	Narrow pre_focus
2023206	block2	HumanSpeech	Post	ngaa5	Subject	Narrow	r1	141.561791383197	2	pre_focus	5	Narrow pre_focus
2023206	block2	HumanSpeech	Post	maai5	Verb	Narrow	r1	275.323399200943	1	pre_focus	5	Narrow pre_focus
2023206	block2	HumanSpeech	Post	pou5	Object	Narrow	r1	205.346938775534	1	on_focus	5	Narrow on_focus
2023206	block2	HumanSpeech	Post	pou5	Object	Narrow	r1	239.467120181416	2	on_focus	5	Narrow on_focus
2023206	block2	HumanSpeech	Post	ngaa5	Subject	Narrow	r1	259.376211090483	1	pre_focus	5	Narrow pre_focus
2023206	block2	HumanSpeech	Post	ngaa5	Subject	Narrow	r1	176.487780297293	2	pre_focus	5	Narrow pre_focus
2023206	block2	HumanSpeech	Post	maai5	Verb	Narrow	r1	254.920634920637	1	on_focus	5	Narrow on_focus
2023206	block2	HumanSpeech	Post	pou5	Object	Narrow	r1	195.041572184437	1	post_focus	5	Narrow post_focus
2023206	block2	HumanSpeech	Post	pou5	Object	Narrow	r1	238.714285714309	2	post_focus	5	Narrow post_focus
2023206	block2	HumanSpeech	Post	ma4	Subject	Narrow	r1	210.831443688591	1	pre_focus	4	Narrow pre_focus
2023206	block2	HumanSpeech	Post	ma4	Subject	Narrow	r1	245.96371882086	2	pre_focus	4	Narrow pre_focus
2023206	block2	HumanSpeech	Post	fu4	Verb	Narrow	r1	225.891156462609	1	pre_focus	4	Narrow pre_focus
2023206	block2	HumanSpeech	Post	maang4	Object	Narrow	r1	211.001511715807	1	on_focus	4	Narrow on_focus
2023206	block2	HumanSpeech	Post	jan4	Object	Narrow	r1	239.319727891171	2	on_focus	4	Narrow on_focus
2023206	block2	HumanSpeech	Post	lok6	Subject	Contrastive	r1	166.730914588072	1	pre_focus	6	Contrastive pre_focus
2023206	block2	HumanSpeech	Post	lok6	Subject	Contrastive	r1	178.735827664383	2	pre_focus	6	Contrastive pre_focus
2023206	block2	HumanSpeech	Post	waa6	Verb	Contrastive	r1	179.480347694636	1	pre_focus	6	Contrastive pre_focus
2023206	block2	HumanSpeech	Post	jyut6	Object	Contrastive	r1	157.208994709009	1	on_focus	6	Contrastive on_focus
2023206	block2	HumanSpeech	Post	loeng6	Object	Contrastive	r1	446.201814058952	2	on_focus	6	Contrastive on_focus
2023206	block2	HumanSpeech	Post	ma4	Subject	Contrastive	r2	301.776266062006	1	pre_focus	4	Contrastive pre_focus
2023206	block2	HumanSpeech	Post	ma4	Subject	Contrastive	r2	273.08551992229	2	pre_focus	4	Contrastive pre_focus
2023206	block2	HumanSpeech	Post	fu4	Verb	Contrastive	r2	146.352445740206	1	pre_focus	4	Contrastive pre_focus
2023206	block2	HumanSpeech	Post	maang4	Object	Contrastive	r2	359.433106575977	1	on_focus	4	Contrastive on_focus
2023206	block2	HumanSpeech	Post	mei2	Object	Contrastive	r2	326.481481481494	2	on_focus	2	Contrastive on_focus
2023206	block2	HumanSpeech	Post	lok6	Subject	Contrastive	r2	113.212899974769	1	on_focus	6	Contrastive on_focus
2023206	block2	HumanSpeech	Post	lok6	Subject	Contrastive	r2	181.875283446743	2	on_focus	6	Contrastive on_focus
2023206	block2	HumanSpeech	Post	waa6	Verb	Contrastive	r2	153.185045948192	1	post_focus	6	Contrastive post_focus
2023206	block2	HumanSpeech	Post	jyut6	Object	Contrastive	r2	137.383651873449	1	post_focus	6	Contrastive post_focus
2023206	block2	HumanSpeech	Post	loeng6	Object	Contrastive	r2	348.023088023069	2	post_focus	6	Contrastive post_focus
2023206	block2	HumanSpeech	Post	ma4	Subject	Narrow	r2	356.312763200492	1	on_focus	4	Narrow on_focus
2023206	block2	HumanSpeech	Post	ma4	Subject	Narrow	r2	309.614512471626	2	on_focus	4	Narrow on_focus
2023206	block2	HumanSpeech	Post	fu4	Verb	Narrow	r2	139.674036281178	1	post_focus	4	Narrow post_focus
2023206	block2	HumanSpeech	Post	maang4	Object	Narrow	r2	269.777561818387	1	post_focus	4	Narrow post_focus
2023206	block2	HumanSpeech	Post	jan4	Object	Narrow	r2	212.396825396866	2	post_focus	4	Narrow post_focus
2023206	block2	HumanSpeech	Post	ngaa5	Subject	Contrastive	r2	231.145743145703	1	on_focus	5	Contrastive on_focus
2023206	block2	HumanSpeech	Post	ngaa5	Subject	Contrastive	r2	206.329680020133	2	on_focus	5	Contrastive on_focus
2023206	block2	HumanSpeech	Post	maai5	Verb	Contrastive	r2	282.562358276607	1	post_focus	5	Contrastive post_focus
2023206	block2	HumanSpeech	Post	pou5	Object	Contrastive	r2	155.508314436929	1	post_focus	5	Contrastive post_focus
2023206	block2	HumanSpeech	Post	pou5	Object	Contrastive	r2	278.832199546514	2	post_focus	5	Contrastive post_focus
2023206	block2	HumanSpeech	Post	ngaa5	Subject	Broad	r2	209.981103552536	1	broad_focus	5	Broad focus
2023206	block2	HumanSpeech	Post	ngaa5	Subject	Broad	r2	241.110031314122	2	broad_focus	5	Broad focus
2023206	block2	HumanSpeech	Post	maai5	Verb	Broad	r2	278.335600907042	1	broad_focus	5	Broad focus
2023206	block2	HumanSpeech	Post	pou5	Object	Broad	r2	189.77040816327	1	broad_focus	5	Broad focus
2023206	block2	HumanSpeech	Post	pou5	Object	Broad	r2	354.456592160659	2	broad_focus	5	Broad focus
2023206	block2	HumanSpeech	Post	ngaa5	Subject	Contrastive	r2	172.645340095414	1	pre_focus	5	Contrastive pre_focus
2023206	block2	HumanSpeech	Post	ngaa5	Subject	Contrastive	r2	141.934096389889	2	pre_focus	5	Contrastive pre_focus
2023206	block2	HumanSpeech	Post	maai5	Verb	Contrastive	r2	191.479717813024	1	on_focus	5	Contrastive on_focus
2023206	block2	HumanSpeech	Post	pou5	Object	Contrastive	r2	173.734325639089	1	post_focus	5	Contrastive post_focus
2023206	block2	HumanSpeech	Post	pou5	Object	Contrastive	r2	201.778726994633	2	post_focus	5	Contrastive post_focus

2023206	block2	HumanSpeech	Post	lok6	Subject	Narrow	r2	134.236254888435	1	pre_focus	6	Narrow pre_focus
2023206	block2	HumanSpeech	Post	lok6	Subject	Narrow	r2	162.634794658629	2	pre_focus	6	Narrow pre_focus
2023206	block2	HumanSpeech	Post	waa6	Verb	Narrow	r2	151.628117913845	1	pre_focus	6	Narrow pre_focus
2023206	block2	HumanSpeech	Post	jyut6	Object	Narrow	r2	167.329495900958	1	on_focus	6	Narrow on_focus
2023206	block2	HumanSpeech	Post	loeng6	Object	Narrow	r2	350.412194507442	2	on_focus	6	Narrow on_focus
2023206	block2	HumanSpeech	Post	ngaa5	Subject	Contrastive	r2	136.816324230779	1	pre_focus	5	Contrastive pre_focus
2023206	block2	HumanSpeech	Post	ngaa5	Subject	Contrastive	r2	184.718440396296	2	pre_focus	5	Contrastive pre_focus
2023206	block2	HumanSpeech	Post	maai5	Verb	Contrastive	r2	202.638234781091	1	pre_focus	5	Contrastive pre_focus
2023206	block2	HumanSpeech	Post	pou5	Object	Contrastive	r2	76.130385487545	1	on_focus	5	Contrastive on_focus
2023206	block2	HumanSpeech	Post	pou5	Object	Contrastive	r2	202.288188002512	2	on_focus	5	Contrastive on_focus
2023206	block2	HumanSpeech	Post	ma4	Subject	Contrastive	r2	154.476190476203	1	on_focus	4	Contrastive on_focus
2023206	block2	HumanSpeech	Post	ma4	Subject	Contrastive	r2	163.605346900624	2	on_focus	4	Contrastive on_focus
2023206	block2	HumanSpeech	Post	fu4	Verb	Contrastive	r2	102.137818090227	1	post_focus	4	Contrastive post_focus
2023206	block2	HumanSpeech	Post	maang4	Object	Contrastive	r2	207.041761148901	1	post_focus	4	Contrastive post_focus
2023206	block2	HumanSpeech	Post	jan4	Object	Contrastive	r2	318.556311413488	2	post_focus	4	Contrastive post_focus
2023206	block2	HumanSpeech	Post	lok6	Subject	Broad	r2	96.5088884129273	1	broad_focus	6	Broad focus
2023206	block2	HumanSpeech	Post	lok6	Subject	Broad	r2	184.600347654566	2	broad_focus	6	Broad focus
2023206	block2	HumanSpeech	Post	waa6	Verb	Broad	r2	127.914372098019	1	broad_focus	6	Broad focus
2023206	block2	HumanSpeech	Post	jyut6	Object	Broad	r2	179.397203325721	1	broad_focus	6	Broad focus
2023206	block2	HumanSpeech	Post	loeng6	Object	Broad	r2	455.660264105688	2	broad_focus	6	Broad focus
2023206	block2	HumanSpeech	Post	ngaa5	Subject	Narrow	r2	153.865018831027	1	pre_focus	5	Narrow pre_focus
2023206	block2	HumanSpeech	Post	ngaa5	Subject	Narrow	r2	111.133664935494	2	pre_focus	5	Narrow pre_focus
2023206	block2	HumanSpeech	Post	maai5	Verb	Narrow	r2	240.534333391452	1	on_focus	5	Narrow on_focus
2023206	block2	HumanSpeech	Post	pou5	Object	Narrow	r2	156.834845049104	1	post_focus	5	Narrow post_focus
2023206	block2	HumanSpeech	Post	pou5	Object	Narrow	r2	332.402253830821	2	post_focus	5	Narrow post_focus
2023206	block2	HumanSpeech	Post	lok6	Subject	Narrow	r2	161.499294356474	1	on_focus	6	Narrow on_focus
2023206	block2	HumanSpeech	Post	lok6	Subject	Narrow	r2	192.34013605444	2	on_focus	6	Narrow on_focus
2023206	block2	HumanSpeech	Post	waa6	Verb	Narrow	r2	155.338074623785	1	post_focus	6	Narrow post_focus
2023206	block2	HumanSpeech	Post	jyut6	Object	Narrow	r2	148.556808688397	1	post_focus	6	Narrow post_focus
2023206	block2	HumanSpeech	Post	loeng6	Object	Narrow	r2	353.758003201335	2	post_focus	6	Narrow post_focus
2023206	block2	HumanSpeech	Post	ma4	Subject	Broad	r2	179.345238095209	1	broad_focus	4	Broad focus
2023206	block2	HumanSpeech	Post	ma4	Subject	Broad	r2	145.700331414616	2	broad_focus	4	Broad focus
2023206	block2	HumanSpeech	Post	fu4	Verb	Broad	r2	194.462131519288	1	broad_focus	4	Broad focus
2023206	block2	HumanSpeech	Post	maang4	Object	Broad	r2	185.328235512543	1	broad_focus	4	Broad focus
2023206	block2	HumanSpeech	Post	jan4	Object	Broad	r2	240.967037221367	2	broad_focus	4	Broad focus
2023206	block2	HumanSpeech	Post	ngaa5	Subject	Narrow	r2	213.559906347314	1	pre_focus	5	Narrow pre_focus
2023206	block2	HumanSpeech	Post	ngaa5	Subject	Narrow	r2	107.655378634945	2	pre_focus	5	Narrow pre_focus
2023206	block2	HumanSpeech	Post	maai5	Verb	Narrow	r2	201.362288505095	1	pre_focus	5	Narrow pre_focus
2023206	block2	HumanSpeech	Post	pou5	Object	Narrow	r2	173.515819026022	1	on_focus	5	Narrow on_focus
2023206	block2	HumanSpeech	Post	pou5	Object	Narrow	r2	274.314418889219	2	on_focus	5	Narrow on_focus
2023206	block2	HumanSpeech	Post	lok6	Subject	Contrastive	r2	167.956849406437	1	pre_focus	6	Contrastive pre_focus
2023206	block2	HumanSpeech	Post	lok6	Subject	Contrastive	r2	159.742747673761	2	pre_focus	6	Contrastive pre_focus
2023206	block2	HumanSpeech	Post	waa6	Verb	Contrastive	r2	133.399032231921	1	pre_focus	6	Contrastive pre_focus
2023206	block2	HumanSpeech	Post	jyut6	Object	Contrastive	r2	166.278969707548	1	on_focus	6	Contrastive on_focus
2023206	block2	HumanSpeech	Post	loeng6	Object	Contrastive	r2	372.709780640776	2	on_focus	6	Contrastive on_focus
2023206	block2	HumanSpeech	Post	lok6	Subject	Narrow	r2	116.037674328993	1	pre_focus	6	Narrow pre_focus
2023206	block2	HumanSpeech	Post	lok6	Subject	Narrow	r2	158.923701544097	2	pre_focus	6	Narrow pre_focus
2023206	block2	HumanSpeech	Post	waa6	Verb	Narrow	r2	106.751927437642	1	on_focus	6	Narrow on_focus
2023206	block2	HumanSpeech	Post	jyut6	Object	Narrow	r2	111.372637944044	1	post_focus	6	Narrow post_focus
2023206	block2	HumanSpeech	Post	loeng6	Object	Narrow	r2	407.580309901789	2	post_focus	6	Narrow post_focus
2023206	block2	HumanSpeech	Post	ma4	Subject	Narrow	r2	262.821653529102	1	pre_focus	4	Narrow pre_focus
2023206	block2	HumanSpeech	Post	ma4	Subject	Narrow	r2	300.509491415596	2	pre_focus	4	Narrow pre_focus
2023206	block2	HumanSpeech	Post	fu4	Verb	Narrow	r2	113.409998920247	1	pre_focus	4	Narrow pre_focus
2023206	block2	HumanSpeech	Post	maang4	Object	Narrow	r2	236.530612244849	1	on_focus	4	Narrow on_focus
2023206	block2	HumanSpeech	Post	jan4	Object	Narrow	r2	271.069387755119	2	on_focus	4	Narrow on_focus
2023206	block2	HumanSpeech	Post	lok6	Subject	Contrastive	r2	189.474570780703	1	pre_focus	6	Contrastive pre_focus
2023206	block2	HumanSpeech	Post	lok6	Subject	Contrastive	r2	172.494824016553	2	pre_focus	6	Contrastive pre_focus
2023206	block2	HumanSpeech	Post	waa6	Verb	Contrastive	r2	144.448601662873	1	on_focus	6	Contrastive on_focus
2023206	block2	HumanSpeech	Post	jyut6	Object	Contrastive	r2	141.289115646259	1	post_focus	6	Contrastive post_focus
2023206	block2	HumanSpeech	Post	loeng6	Object	Contrastive	r2	282.778857574783	2	post_focus	6	Contrastive post_focus
2023206	block2	HumanSpeech	Post	ma4	Subject	Narrow	r2	129.901824366129	1	pre_focus	4	Narrow pre_focus
2023206	block2	HumanSpeech	Post	ma4	Subject	Narrow	r2	191.312444169625	2	pre_focus	4	Narrow pre_focus

2023206	block2	HumanSpeech	Post	fu4	Verb	Narrow	r2	89.9535687290722	1	on_focus	4	Narrow on_focus
2023206	block2	HumanSpeech	Post	maang4	Object	Narrow	r2	321.189216427342	1	post_focus	4	Narrow post_focus
2023206	block2	HumanSpeech	Post	jan4	Object	Narrow	r2	256.243386243341	2	post_focus	4	Narrow post_focus
2023206	block2	HumanSpeech	Post	ngaa5	Subject	Narrow	r2	179.122198663038	1	on_focus	5	Narrow on_focus
2023206	block2	HumanSpeech	Post	ngaa5	Subject	Narrow	r2	245.918020623151	2	on_focus	5	Narrow on_focus
2023206	block2	HumanSpeech	Post	maai5	Verb	Narrow	r2	178.542083500076	1	post_focus	5	Narrow post_focus
2023206	block2	HumanSpeech	Post	pou5	Object	Narrow	r2	179.563869992421	1	post_focus	5	Narrow post_focus
2023206	block2	HumanSpeech	Post	pou5	Object	Narrow	r2	252.664399092964	2	post_focus	5	Narrow post_focus
2023206	block2	HumanSpeech	Post	ma4	Subject	Contrastive	r2	320.084404132047	1	pre_focus	4	Contrastive pre_focus
2023206	block2	HumanSpeech	Post	ma4	Subject	Contrastive	r2	393.357583774275	2	pre_focus	4	Contrastive pre_focus
2023206	block2	HumanSpeech	Post	fu4	Verb	Contrastive	r2	176.027210884342	1	on_focus	4	Contrastive on_focus
2023206	block2	HumanSpeech	Post	maang4	Object	Contrastive	r2	322.310405643748	1	post_focus	4	Contrastive post_focus
2023206	block2	HumanSpeech	Post	jan4	Object	Contrastive	r2	217.912572436376	2	post_focus	4	Contrastive post_focus
2023206	block2	HumanSpeech	Pre	ma4	Subject	Contrastive	r1	358.242630385519	1	pre_focus	4	Contrastive pre_focus
2023206	block2	HumanSpeech	Pre	ma4	Subject	Contrastive	r1	416.364323507196	2	pre_focus	4	Contrastive pre_focus
2023206	block2	HumanSpeech	Pre	fu4	Verb	Contrastive	r1	199.203109815357	1	on_focus	4	Contrastive on_focus
2023206	block2	HumanSpeech	Pre	maang4	Object	Contrastive	r1	339.229024943279	1	post_focus	4	Contrastive post_focus
2023206	block2	HumanSpeech	Pre	jan4	Object	Contrastive	r1	310.128495842775	2	post_focus	4	Contrastive post_focus
2023206	block2	HumanSpeech	Pre	ma4	Subject	Narrow	r1	391.258503401332	1	on_focus	4	Narrow on_focus
2023206	block2	HumanSpeech	Pre	ma4	Subject	Narrow	r1	352.851989280623	2	on_focus	4	Narrow on_focus
2023206	block2	HumanSpeech	Pre	fu4	Verb	Narrow	r1	135.7256235828	1	post_focus	4	Narrow post_focus
2023206	block2	HumanSpeech	Pre	maang4	Object	Narrow	r1	275.473760932925	1	post_focus	4	Narrow post_focus
2023206	block2	HumanSpeech	Pre	jan4	Object	Narrow	r1	157.064625850296	2	post_focus	4	Narrow post_focus
2023206	block2	HumanSpeech	Pre	ngaa5	Subject	Narrow	r1	250.596371882068	1	pre_focus	5	Narrow pre_focus
2023206	block2	HumanSpeech	Pre	ngaa5	Subject	Narrow	r1	205.195983155193	2	pre_focus	5	Narrow pre_focus
2023206	block2	HumanSpeech	Pre	maai5	Verb	Narrow	r1	255.697714983455	1	pre_focus	5	Narrow pre_focus
2023206	block2	HumanSpeech	Pre	pou5	Object	Narrow	r1	123.311305474601	1	on_focus	5	Narrow on_focus
2023206	block2	HumanSpeech	Pre	pou5	Object	Narrow	r1	279.346655328823	2	on_focus	5	Narrow on_focus
2023206	block2	HumanSpeech	Pre	ngaa5	Subject	Narrow	r1	270.428949357495	1	pre_focus	5	Narrow pre_focus
2023206	block2	HumanSpeech	Pre	ngaa5	Subject	Narrow	r1	212.032717849013	2	pre_focus	5	Narrow pre_focus
2023206	block2	HumanSpeech	Pre	maai5	Verb	Narrow	r1	311.71135120718	1	on_focus	5	Narrow on_focus
2023206	block2	HumanSpeech	Pre	pou5	Object	Narrow	r1	152.350030921468	1	post_focus	5	Narrow post_focus
2023206	block2	HumanSpeech	Pre	pou5	Object	Narrow	r1	288.284202569912	2	post_focus	5	Narrow post_focus
2023206	block2	HumanSpeech	Pre	lok6	Subject	Narrow	r1	156.645502645517	1	pre_focus	6	Narrow pre_focus
2023206	block2	HumanSpeech	Pre	lok6	Subject	Narrow	r1	173.106575963743	2	pre_focus	6	Narrow pre_focus
2023206	block2	HumanSpeech	Pre	waa6	Verb	Narrow	r1	132.244897959197	1	on_focus	6	Narrow on_focus
2023206	block2	HumanSpeech	Pre	jyut6	Object	Narrow	r1	165.66893424033	1	post_focus	6	Narrow post_focus
2023206	block2	HumanSpeech	Pre	loeng6	Object	Narrow	r1	297.288629737636	2	post_focus	6	Narrow post_focus
2023206	block2	HumanSpeech	Pre	ma4	Subject	Contrastive	r1	394.353741496616	1	on_focus	4	Contrastive on_focus
2023206	block2	HumanSpeech	Pre	ma4	Subject	Contrastive	r1	415.925925925933	2	on_focus	4	Contrastive on_focus
2023206	block2	HumanSpeech	Pre	fu4	Verb	Contrastive	r1	245.526401036614	1	post_focus	4	Contrastive post_focus
2023206	block2	HumanSpeech	Pre	maang4	Object	Contrastive	r1	313.866213151925	1	post_focus	4	Contrastive post_focus
2023206	block2	HumanSpeech	Pre	jan4	Object	Contrastive	r1	204.937641723347	2	post_focus	4	Contrastive post_focus
2023206	block2	HumanSpeech	Pre	lok6	Subject	Contrastive	r1	231.53061224491	1	on_focus	6	Contrastive on_focus
2023206	block2	HumanSpeech	Pre	lok6	Subject	Contrastive	r1	223.95691609978	2	on_focus	6	Contrastive on_focus
2023206	block2	HumanSpeech	Pre	waa6	Verb	Contrastive	r1	176.405895691573	1	post_focus	6	Contrastive post_focus
2023206	block2	HumanSpeech	Pre	jyut6	Object	Contrastive	r1	239.14084152176	1	post_focus	6	Contrastive post_focus
2023206	block2	HumanSpeech	Pre	loeng6	Object	Contrastive	r1	347.34240362809	2	post_focus	6	Contrastive post_focus
2023206	block2	HumanSpeech	Pre	ngaa5	Subject	Contrastive	r1	239.13492063491	1	pre_focus	5	Contrastive pre_focus
2023206	block2	HumanSpeech	Pre	ngaa5	Subject	Contrastive	r1	341.264802217154	2	pre_focus	5	Contrastive pre_focus
2023206	block2	HumanSpeech	Pre	maai5	Verb	Contrastive	r1	645.726883345901	1	pre_focus	5	Contrastive pre_focus
2023206	block2	HumanSpeech	Pre	pou5	Object	Contrastive	r1	158.651794050968	1	on_focus	5	Contrastive on_focus
2023206	block2	HumanSpeech	Pre	pou5	Object	Contrastive	r1	311.072736787025	2	on_focus	5	Contrastive on_focus
2023206	block2	HumanSpeech	Pre	ngaa5	Subject	Contrastive	r1	283.356009070303	1	pre_focus	5	Contrastive pre_focus
2023206	block2	HumanSpeech	Pre	ngaa5	Subject	Contrastive	r1	316.06198034774	2	pre_focus	5	Contrastive pre_focus
2023206	block2	HumanSpeech	Pre	maai5	Verb	Contrastive	r1	337.108843537465	1	on_focus	5	Contrastive on_focus
2023206	block2	HumanSpeech	Pre	pou5	Object	Contrastive	r1	204.648526077108	1	post_focus	5	Contrastive post_focus
2023206	block2	HumanSpeech	Pre	pou5	Object	Contrastive	r1	372.160997732408	2	post_focus	5	Contrastive post_focus
2023206	block2	HumanSpeech	Pre	ma4	Subject	Narrow	r1	422.86281179139	1	pre_focus	4	Narrow pre_focus
2023206	block2	HumanSpeech	Pre	ma4	Subject	Narrow	r1	380.816326530635	2	pre_focus	4	Narrow pre_focus
2023206	block2	HumanSpeech	Pre	fu4	Verb	Narrow	r1	117.472411186725	1	on_focus	4	Narrow on_focus
2023206	block2	HumanSpeech	Pre	maang4	Object	Narrow	r1	320.361781076087	1	post_focus	4	Narrow post_focus

**2023206**	block2	HumanSpeech	Pre	jan4	Object	Narrow	r1	218.621315192763	2	post_focus	4	Narrow post_focus
**2023206**	block2	HumanSpeech	Pre	ma4	Subject	Contrastive	r1	354.136054421815	1	pre_focus	4	Contrastive pre_focus
**2023206**	block2	HumanSpeech	Pre	ma4	Subject	Contrastive	r1	325.234093637448	2	pre_focus	4	Contrastive pre_focus
**2023206**	block2	HumanSpeech	Pre	fu4	Verb	Contrastive	r1	221.751700680272	1	pre_focus	4	Contrastive pre_focus
**2023206**	block2	HumanSpeech	Pre	maang4	Object	Contrastive	r1	357.482659730522	1	on_focus	4	Contrastive on_focus
**2023206**	block2	HumanSpeech	Pre	jan4	Object	Contrastive	r1	271.029169243491	2	on_focus	4	Contrastive on_focus
**2023206**	block2	HumanSpeech	Pre	lok6	Subject	Narrow	r1	189.278155706688	1	on_focus	6	Narrow on_focus
**2023206**	block2	HumanSpeech	Pre	lok6	Subject	Narrow	r1	210.87463556853	2	on_focus	6	Narrow on_focus
**2023206**	block2	HumanSpeech	Pre	waa6	Verb	Narrow	r1	188.784580498918	1	post_focus	6	Narrow post_focus
**2023206**	block2	HumanSpeech	Pre	jyut6	Object	Narrow	r1	160.486988446223	1	post_focus	6	Narrow post_focus
**2023206**	block2	HumanSpeech	Pre	loeng6	Object	Narrow	r1	357.511715797386	2	post_focus	6	Narrow post_focus
**2023206**	block2	HumanSpeech	Pre	lok6	Subject	Narrow	r1	165.48752834467	1	pre_focus	6	Narrow pre_focus
**2023206**	block2	HumanSpeech	Pre	lok6	Subject	Narrow	r1	232.295918367356	2	pre_focus	6	Narrow pre_focus
**2023206**	block2	HumanSpeech	Pre	waa6	Verb	Narrow	r1	175.767195767207	1	pre_focus	6	Narrow pre_focus
**2023206**	block2	HumanSpeech	Pre	jyut6	Object	Narrow	r1	176.87830687828	1	on_focus	6	Narrow on_focus
**2023206**	block2	HumanSpeech	Pre	loeng6	Object	Narrow	r1	353.17460317458	2	on_focus	6	Narrow on_focus
**2023206**	block2	HumanSpeech	Pre	lok6	Subject	Contrastive	r1	169.773242630356	1	pre_focus	6	Contrastive pre_focus
**2023206**	block2	HumanSpeech	Pre	lok6	Subject	Contrastive	r1	213.16326530615	2	pre_focus	6	Contrastive pre_focus
**2023206**	block2	HumanSpeech	Pre	waa6	Verb	Contrastive	r1	240.691609977318	1	pre_focus	6	Contrastive pre_focus
**2023206**	block2	HumanSpeech	Pre	jyut6	Object	Contrastive	r1	244.506802721048	1	on_focus	6	Contrastive on_focus
**2023206**	block2	HumanSpeech	Pre	loeng6	Object	Contrastive	r1	466.168993913357	2	on_focus	6	Contrastive on_focus
**2023206**	block2	HumanSpeech	Pre	ngaa5	Subject	Narrow	r1	294.403628117948	1	on_focus	5	Narrow on_focus
**2023206**	block2	HumanSpeech	Pre	ngaa5	Subject	Narrow	r1	312.460317460307	2	on_focus	5	Narrow on_focus
**2023206**	block2	HumanSpeech	Pre	maai5	Verb	Narrow	r1	329.807796134332	1	post_focus	5	Narrow post_focus
**2023206**	block2	HumanSpeech	Pre	pou5	Object	Narrow	r1	196.893424036261	1	post_focus	5	Narrow post_focus
**2023206**	block2	HumanSpeech	Pre	pou5	Object	Narrow	r1	336.489795918396	2	post_focus	5	Narrow post_focus
**2023206**	block2	HumanSpeech	Pre	lok6	Subject	Contrastive	r1	184.671201814012	1	pre_focus	6	Contrastive pre_focus
**2023206**	block2	HumanSpeech	Pre	lok6	Subject	Contrastive	r1	224.580498866203	2	pre_focus	6	Contrastive pre_focus
**2023206**	block2	HumanSpeech	Pre	waa6	Verb	Contrastive	r1	167.035147392312	1	on_focus	6	Contrastive on_focus
**2023206**	block2	HumanSpeech	Pre	jyut6	Object	Contrastive	r1	194.66364323506	1	post_focus	6	Contrastive post_focus
**2023206**	block2	HumanSpeech	Pre	loeng6	Object	Contrastive	r1	359.168556311431	2	post_focus	6	Contrastive post_focus
**2023206**	block2	HumanSpeech	Pre	lok6	Subject	Broad	r1	216.022675736951	1	broad_focus	6	Broad focus
**2023206**	block2	HumanSpeech	Pre	lok6	Subject	Broad	r1	261.655328798156	2	broad_focus	6	Broad focus
**2023206**	block2	HumanSpeech	Pre	waa6	Verb	Broad	r1	166.746031746015	1	broad_focus	6	Broad focus
**2023206**	block2	HumanSpeech	Pre	jyut6	Object	Broad	r1	160.294784580515	1	broad_focus	6	Broad focus
**2023206**	block2	HumanSpeech	Pre	loeng6	Object	Broad	r1	430.948601662919	2	broad_focus	6	Broad focus
**2023206**	block2	HumanSpeech	Pre	ngaa5	Subject	Contrastive	r1	334.90929705215	1	on_focus	5	Contrastive on_focus
**2023206**	block2	HumanSpeech	Pre	ngaa5	Subject	Contrastive	r1	291.574933003517	2	on_focus	5	Contrastive on_focus
**2023206**	block2	HumanSpeech	Pre	maai5	Verb	Contrastive	r1	302.189828312237	1	post_focus	5	Contrastive post_focus
**2023206**	block2	HumanSpeech	Pre	pou5	Object	Contrastive	r1	147.894935752106	1	post_focus	5	Contrastive post_focus
**2023206**	block2	HumanSpeech	Pre	pou5	Object	Contrastive	r1	294.580498866196	2	post_focus	5	Contrastive post_focus
**2023206**	block2	HumanSpeech	Pre	ma4	Subject	Broad	r1	391.122448979559	1	broad_focus	4	Broad focus
**2023206**	block2	HumanSpeech	Pre	ma4	Subject	Broad	r1	392.459057697181	2	broad_focus	4	Broad focus
**2023206**	block2	HumanSpeech	Pre	fu4	Verb	Broad	r1	154.289493575163	1	broad_focus	4	Broad focus
**2023206**	block2	HumanSpeech	Pre	maang4	Object	Broad	r1	296.572184429294	1	broad_focus	4	Broad focus
**2023206**	block2	HumanSpeech	Pre	jan4	Object	Broad	r1	269.229024943286	2	broad_focus	4	Broad focus
**2023206**	block2	HumanSpeech	Pre	ngaa5	Subject	Broad	r1	369.266817838252	1	broad_focus	5	Broad focus
**2023206**	block2	HumanSpeech	Pre	ngaa5	Subject	Broad	r1	324.152116402104	2	broad_focus	5	Broad focus
**2023206**	block2	HumanSpeech	Pre	maai5	Verb	Broad	r1	343.286237571988	1	broad_focus	5	Broad focus
**2023206**	block2	HumanSpeech	Pre	pou5	Object	Broad	r1	147.13151927441	1	broad_focus	5	Broad focus
**2023206**	block2	HumanSpeech	Pre	pou5	Object	Broad	r1	301.156462585027	2	broad_focus	5	Broad focus
**2023206**	block2	HumanSpeech	Pre	ma4	Subject	Narrow	r1	327.363945578213	1	pre_focus	4	Narrow pre_focus
**2023206**	block2	HumanSpeech	Pre	ma4	Subject	Narrow	r1	321.054421768679	2	pre_focus	4	Narrow pre_focus
**2023206**	block2	HumanSpeech	Pre	fu4	Verb	Narrow	r1	149.991361624018	1	pre_focus	4	Narrow pre_focus
**2023206**	block2	HumanSpeech	Pre	maang4	Object	Narrow	r1	332.383786848084	1	on_focus	4	Narrow on_focus
**2023206**	block2	HumanSpeech	Pre	jan4	Object	Narrow	r1	301.99168556311	2	on_focus	4	Narrow on_focus
**2023206**	block2	HumanSpeech	Pre	lok6	Subject	Contrastive	r2	189.325396825438	1	pre_focus	6	Contrastive pre_focus
**2023206**	block2	HumanSpeech	Pre	lok6	Subject	Contrastive	r2	216.47392290248	2	pre_focus	6	Contrastive pre_focus
**2023206**	block2	HumanSpeech	Pre	waa6	Verb	Contrastive	r2	199.145880574463	1	pre_focus	6	Contrastive pre_focus
**2023206**	block2	HumanSpeech	Pre	jyut6	Object	Contrastive	r2	173.141849332353	1	on_focus	6	Contrastive on_focus
**2023206**	block2	HumanSpeech	Pre	loeng6	Object	Contrastive	r2	395.840262030731	2	on_focus	6	Contrastive on_focus
**2023206**	block2	HumanSpeech	Pre	ma4	Subject	Contrastive	r2	282.173091458787	1	pre_focus	4	Contrastive pre_focus

2023206	block2	HumanSpeech	Pre	ma4	Subject	Contrastive	r2	313.526131345725	2	pre_focus	4	Contrastive pre_focus
2023206	block2	HumanSpeech	Pre	fu4	Verb	Contrastive	r2	142.535147392266	1	on_focus	4	Contrastive on_focus
2023206	block2	HumanSpeech	Pre	maang4	Object	Contrastive	r2	337.910052910047	1	post_focus	4	Contrastive post_focus
2023206	block2	HumanSpeech	Pre	jan4	Object	Contrastive	r2	285.733722060286	2	post_focus	4	Contrastive post_focus
2023206	block2	HumanSpeech	Pre	ma4	Subject	Narrow	r2	412.657596371901	1	on_focus	4	Narrow on_focus
2023206	block2	HumanSpeech	Pre	ma4	Subject	Narrow	r2	274.843098529743	2	on_focus	4	Narrow on_focus
2023206	block2	HumanSpeech	Pre	fu4	Verb	Narrow	r2	135.742630385494	1	post_focus	4	Narrow post_focus
2023206	block2	HumanSpeech	Pre	maang4	Object	Narrow	r2	292.091836734699	1	post_focus	4	Narrow post_focus
2023206	block2	HumanSpeech	Pre	jan4	Object	Narrow	r2	213.425370148059	2	post_focus	4	Narrow post_focus
2023206	block2	HumanSpeech	Pre	lok6	Subject	Contrastive	r2	174.693877551022	1	on_focus	6	Contrastive on_focus
2023206	block2	HumanSpeech	Pre	lok6	Subject	Contrastive	r2	186.003401360495	2	on_focus	6	Contrastive on_focus
2023206	block2	HumanSpeech	Pre	waa6	Verb	Contrastive	r2	166.995464852619	1	post_focus	6	Contrastive post_focus
2023206	block2	HumanSpeech	Pre	jyut6	Object	Contrastive	r2	222.762660619878	1	post_focus	6	Contrastive post_focus
2023206	block2	HumanSpeech	Pre	loeng6	Object	Contrastive	r2	366.575963718788	2	post_focus	6	Contrastive post_focus
2023206	block2	HumanSpeech	Pre	ma4	Subject	Contrastive	r2	271.251700680295	1	pre_focus	4	Contrastive pre_focus
2023206	block2	HumanSpeech	Pre	ma4	Subject	Contrastive	r2	290.527210884363	2	pre_focus	4	Contrastive pre_focus
2023206	block2	HumanSpeech	Pre	fu4	Verb	Contrastive	r2	470.814814814844	1	pre_focus	4	Contrastive pre_focus
2023206	block2	HumanSpeech	Pre	maang4	Object	Contrastive	r2	497.743764172299	1	on_focus	4	Contrastive on_focus
2023206	block2	HumanSpeech	Pre	jan4	Object	Contrastive	r2	267.064436885846	2	on_focus	4	Contrastive on_focus
2023206	block2	HumanSpeech	Pre	ngaa5	Subject	Contrastive	r2	293.930461073387	1	on_focus	5	Contrastive on_focus
2023206	block2	HumanSpeech	Pre	ngaa5	Subject	Contrastive	r2	252.31092856427	2	on_focus	5	Contrastive on_focus
2023206	block2	HumanSpeech	Pre	maai5	Verb	Contrastive	r2	268.654842889532	1	post_focus	5	Contrastive post_focus
2023206	block2	HumanSpeech	Pre	pou5	Object	Contrastive	r2	161.54950869236	1	post_focus	5	Contrastive post_focus
2023206	block2	HumanSpeech	Pre	pou5	Object	Contrastive	r2	303.914399092946	2	post_focus	5	Contrastive post_focus
2023206	block2	HumanSpeech	Pre	lok6	Subject	Broad	r2	179.297052154197	1	broad_focus	6	Broad focus
2023206	block2	HumanSpeech	Pre	lok6	Subject	Broad	r2	205.702947845793	2	broad_focus	6	Broad focus
2023206	block2	HumanSpeech	Pre	waa6	Verb	Broad	r2	156.893424036184	1	broad_focus	6	Broad focus
2023206	block2	HumanSpeech	Pre	jyut6	Object	Broad	r2	209.338084440219	1	broad_focus	6	Broad focus
2023206	block2	HumanSpeech	Pre	loeng6	Object	Broad	r2	433.333333333394	2	broad_focus	6	Broad focus
2023206	block2	HumanSpeech	Pre	lok6	Subject	Narrow	r2	193.492063492045	1	on_focus	6	Narrow on_focus
2023206	block2	HumanSpeech	Pre	lok6	Subject	Narrow	r2	210.045351473923	2	on_focus	6	Narrow on_focus
2023206	block2	HumanSpeech	Pre	waa6	Verb	Narrow	r2	221.273242630332	1	post_focus	6	Narrow post_focus
2023206	block2	HumanSpeech	Pre	jyut6	Object	Narrow	r2	197.61742792366	1	post_focus	6	Narrow post_focus
2023206	block2	HumanSpeech	Pre	loeng6	Object	Narrow	r2	390.046485260768	2	post_focus	6	Narrow post_focus
2023206	block2	HumanSpeech	Pre	ngaa5	Subject	Contrastive	r2	312.263794406704	1	pre_focus	5	Contrastive pre_focus
2023206	block2	HumanSpeech	Pre	ngaa5	Subject	Contrastive	r2	336.235827664382	2	pre_focus	5	Contrastive pre_focus
2023206	block2	HumanSpeech	Pre	maai5	Verb	Contrastive	r2	310.761904761875	1	pre_focus	5	Contrastive pre_focus
2023206	block2	HumanSpeech	Pre	pou5	Object	Contrastive	r2	221.869178440443	1	on_focus	5	Contrastive on_focus
2023206	block2	HumanSpeech	Pre	pou5	Object	Contrastive	r2	399.577979339938	2	on_focus	5	Contrastive on_focus
2023206	block2	HumanSpeech	Pre	ngaa5	Subject	Narrow	r2	203.112244897966	1	pre_focus	5	Narrow pre_focus
2023206	block2	HumanSpeech	Pre	ngaa5	Subject	Narrow	r2	242.007612568841	2	pre_focus	5	Narrow pre_focus
2023206	block2	HumanSpeech	Pre	maai5	Verb	Narrow	r2	346.311858076547	1	on_focus	5	Narrow on_focus
2023206	block2	HumanSpeech	Pre	pou5	Object	Narrow	r2	186.422272612731	1	post_focus	5	Narrow post_focus
2023206	block2	HumanSpeech	Pre	pou5	Object	Narrow	r2	378.93424036281	2	post_focus	5	Narrow post_focus
2023206	block2	HumanSpeech	Pre	lok6	Subject	Narrow	r2	195.975056689349	1	pre_focus	6	Narrow pre_focus
2023206	block2	HumanSpeech	Pre	lok6	Subject	Narrow	r2	234.399092970534	2	pre_focus	6	Narrow pre_focus
2023206	block2	HumanSpeech	Pre	waa6	Verb	Narrow	r2	164.20559334847	1	pre_focus	6	Narrow pre_focus
2023206	block2	HumanSpeech	Pre	jyut6	Object	Narrow	r2	262.806122448978	1	on_focus	6	Narrow on_focus
2023206	block2	HumanSpeech	Pre	loeng6	Object	Narrow	r2	413.261526832912	2	on_focus	6	Narrow on_focus
2023206	block2	HumanSpeech	Pre	ma4	Subject	Narrow	r2	382.09750566898	1	pre_focus	4	Narrow pre_focus
2023206	block2	HumanSpeech	Pre	ma4	Subject	Narrow	r2	418.574667962503	2	pre_focus	4	Narrow pre_focus
2023206	block2	HumanSpeech	Pre	fu4	Verb	Narrow	r2	298.571428571336	1	on_focus	4	Narrow on_focus
2023206	block2	HumanSpeech	Pre	maang4	Object	Narrow	r2	408.553287981817	1	post_focus	4	Narrow post_focus
2023206	block2	HumanSpeech	Pre	jan4	Object	Narrow	r2	262.773998488228	2	post_focus	4	Narrow post_focus
2023206	block2	HumanSpeech	Pre	lok6	Subject	Contrastive	r2	215.275888133078	1	pre_focus	6	Contrastive pre_focus
2023206	block2	HumanSpeech	Pre	lok6	Subject	Contrastive	r2	219.708049886663	2	pre_focus	6	Contrastive pre_focus
2023206	block2	HumanSpeech	Pre	waa6	Verb	Contrastive	r2	170.039682539709	1	on_focus	6	Contrastive on_focus
2023206	block2	HumanSpeech	Pre	jyut6	Object	Contrastive	r2	193.037414965943	1	post_focus	6	Contrastive post_focus
2023206	block2	HumanSpeech	Pre	loeng6	Object	Contrastive	r2	410.164399092878	2	post_focus	6	Contrastive post_focus
2023206	block2	HumanSpeech	Pre	ngaa5	Subject	Narrow	r2	257.120181405867	1	pre_focus	5	Narrow pre_focus
2023206	block2	HumanSpeech	Pre	ngaa5	Subject	Narrow	r2	235.540978295944	2	pre_focus	5	Narrow pre_focus
2023206	block2	HumanSpeech	Pre	maai5	Verb	Narrow	r2	321.324263038605	1	pre_focus	5	Narrow pre_focus

2023206	block2	HumanSpeech	Pre	pou5	Object	Narrow	r2	203.828420256968	1	on_focus	5	Narrow on_focus
2023206	block2	HumanSpeech	Pre	pou5	Object	Narrow	r2	323.888888888973	2	on_focus	5	Narrow on_focus
2023206	block2	HumanSpeech	Pre	ma4	Subject	Broad	r2	352.755102040874	1	broad_focus	4	Broad focus
2023206	block2	HumanSpeech	Pre	ma4	Subject	Broad	r2	414.459561602371	2	broad_focus	4	Broad focus
2023206	block2	HumanSpeech	Pre	fu4	Verb	Broad	r2	211.651549508701	1	broad_focus	4	Broad focus
2023206	block2	HumanSpeech	Pre	maang4	Object	Broad	r2	306.057661159798	1	broad_focus	4	Broad focus
2023206	block2	HumanSpeech	Pre	jan4	Object	Broad	r2	293.662131519227	2	broad_focus	4	Broad focus
2023206	block2	HumanSpeech	Pre	ma4	Subject	Narrow	r2	255.073696145246	1	pre_focus	4	Narrow pre_focus
2023206	block2	HumanSpeech	Pre	ma4	Subject	Narrow	r2	366.022171831787	2	pre_focus	4	Narrow pre_focus
2023206	block2	HumanSpeech	Pre	fu4	Verb	Narrow	r2	179.385487528407	1	pre_focus	4	Narrow pre_focus
2023206	block2	HumanSpeech	Pre	maang4	Object	Narrow	r2	327.831065759597	1	on_focus	4	Narrow on_focus
2023206	block2	HumanSpeech	Pre	jan4	Object	Narrow	r2	251.753590325052	2	on_focus	4	Narrow on_focus
2023206	block2	HumanSpeech	Pre	ngaa5	Subject	Narrow	r2	238.521541950149	1	on_focus	5	Narrow on_focus
2023206	block2	HumanSpeech	Pre	ngaa5	Subject	Narrow	r2	217.743764172269	2	on_focus	5	Narrow on_focus
2023206	block2	HumanSpeech	Pre	maai5	Verb	Narrow	r2	295.596587841487	1	post_focus	5	Narrow post_focus
2023206	block2	HumanSpeech	Pre	pou5	Object	Narrow	r2	188.784958427732	1	post_focus	5	Narrow post_focus
2023206	block2	HumanSpeech	Pre	pou5	Object	Narrow	r2	299.051398337042	2	post_focus	5	Narrow post_focus
2023206	block2	HumanSpeech	Pre	lok6	Subject	Narrow	r2	193.310657596385	1	pre_focus	6	Narrow pre_focus
2023206	block2	HumanSpeech	Pre	lok6	Subject	Narrow	r2	244.941151063585	2	pre_focus	6	Narrow pre_focus
2023206	block2	HumanSpeech	Pre	waa6	Verb	Narrow	r2	170.255911888603	1	on_focus	6	Narrow on_focus
2023206	block2	HumanSpeech	Pre	jyut6	Object	Narrow	r2	205.263038548765	1	post_focus	6	Narrow post_focus
2023206	block2	HumanSpeech	Pre	loeng6	Object	Narrow	r2	408.600907029495	2	post_focus	6	Narrow post_focus
2023206	block2	HumanSpeech	Pre	ngaa5	Subject	Contrastive	r2	302.358276643986	1	pre_focus	5	Contrastive pre_focus
2023206	block2	HumanSpeech	Pre	ngaa5	Subject	Contrastive	r2	443.356009070385	2	pre_focus	5	Contrastive pre_focus
2023206	block2	HumanSpeech	Pre	maai5	Verb	Contrastive	r2	282.947845804983	1	on_focus	5	Contrastive on_focus
2023206	block2	HumanSpeech	Pre	pou5	Object	Contrastive	r2	175.478458049838	1	post_focus	5	Contrastive post_focus
2023206	block2	HumanSpeech	Pre	pou5	Object	Contrastive	r2	294.673469387817	2	post_focus	5	Contrastive post_focus
2023206	block2	HumanSpeech	Pre	ngaa5	Subject	Broad	r2	281.485260770978	1	broad_focus	5	Broad focus
2023206	block2	HumanSpeech	Pre	ngaa5	Subject	Broad	r2	244.952758881254	2	broad_focus	5	Broad focus
2023206	block2	HumanSpeech	Pre	maai5	Verb	Broad	r2	311.831581117303	1	broad_focus	5	Broad focus
2023206	block2	HumanSpeech	Pre	pou5	Object	Broad	r2	187.820294784615	1	broad_focus	5	Broad focus
2023206	block2	HumanSpeech	Pre	pou5	Object	Broad	r2	322.488662131491	2	broad_focus	5	Broad focus
2023206	block2	HumanSpeech	Pre	ma4	Subject	Contrastive	r2	332.897959183697	1	on_focus	4	Contrastive on_focus
2023206	block2	HumanSpeech	Pre	ma4	Subject	Contrastive	r2	327.797933988336	2	on_focus	4	Contrastive on_focus
2023206	block2	HumanSpeech	Pre	fu4	Verb	Contrastive	r2	125.831443688526	1	post_focus	4	Contrastive post_focus
2023206	block2	HumanSpeech	Pre	maang4	Object	Contrastive	r2	320.999999999913	1	post_focus	4	Contrastive post_focus
2023206	block2	HumanSpeech	Pre	jan4	Object	Contrastive	r2	204.095130115547	2	post_focus	4	Contrastive post_focus
2023206	block3	HumanSpeech	Post	bui3	Subject	Broad	r1	134.490996398554	1	broad_focus	3	Broad focus
2023206	block3	HumanSpeech	Post	bui3	Subject	Broad	r1	185.037792894917	2	broad_focus	3	Broad focus
2023206	block3	HumanSpeech	Post	tsv1	Verb	Broad	r1	153.546233308134	1	broad_focus	1	Broad focus
2023206	block3	HumanSpeech	Post	fug1	Object	Broad	r1	203.925736961452	1	broad_focus	1	Broad focus
2023206	block3	HumanSpeech	Post	tshe1	Object	Broad	r1	221.280161637296	2	broad_focus	1	Broad focus
2023206	block3	HumanSpeech	Post	bui3	Subject	Contrastive	r1	176.602090111402	1	on_focus	3	Contrastive on_focus
2023206	block3	HumanSpeech	Post	bui3	Subject	Contrastive	r1	170.052910052902	2	on_focus	3	Contrastive on_focus
2023206	block3	HumanSpeech	Post	tsv1	Verb	Contrastive	r1	127.515935667191	1	post_focus	1	Contrastive post_focus
2023206	block3	HumanSpeech	Post	fug1	Object	Contrastive	r1	158.187349473948	1	post_focus	1	Contrastive post_focus
2023206	block3	HumanSpeech	Post	tshe1	Object	Contrastive	r1	137.101757369621	2	post_focus	1	Contrastive post_focus
2023206	block3	HumanSpeech	Post	bui3	Subject	Narrow	r1	144.008206457187	1	pre_focus	3	Narrow pre_focus
2023206	block3	HumanSpeech	Post	bui3	Subject	Narrow	r1	168.700298279418	2	pre_focus	3	Narrow pre_focus
2023206	block3	HumanSpeech	Post	tsv1	Verb	Narrow	r1	122.547688666117	1	on_focus	1	Narrow on_focus
2023206	block3	HumanSpeech	Post	fug1	Object	Narrow	r1	202.296328876372	1	post_focus	1	Narrow post_focus
2023206	block3	HumanSpeech	Post	tshe1	Object	Narrow	r1	217.097299525889	2	post_focus	1	Narrow post_focus
2023206	block3	HumanSpeech	Post	piu2	Subject	Broad	r1	183.744950087316	1	broad_focus	2	Broad focus
2023206	block3	HumanSpeech	Post	tse2	Subject	Broad	r1	141.038474406145	2	broad_focus	2	Broad focus
2023206	block3	HumanSpeech	Post	tsap1	Verb	Broad	r1	112.481574869321	1	broad_focus	1	Broad focus
2023206	block3	HumanSpeech	Post	sy1	Object	Broad	r1	74.7562358276639	1	broad_focus	1	Broad focus
2023206	block3	HumanSpeech	Post	pau1	Object	Broad	r1	219.017008747457	2	broad_focus	1	Broad focus
2023206	block3	HumanSpeech	Post	suk1	Subject	Contrastive	r1	93.251522831352	1	on_focus	1	Contrastive on_focus
2023206	block3	HumanSpeech	Post	suk1	Subject	Contrastive	r1	76.5934487939148	2	on_focus	1	Contrastive on_focus
2023206	block3	HumanSpeech	Post	sei2	Verb	Contrastive	r1	145.538863693616	1	post_focus	2	Contrastive post_focus
2023206	block3	HumanSpeech	Post	svy2	Object	Contrastive	r1	149.920131015364	1	post_focus	2	Contrastive post_focus
2023206	block3	HumanSpeech	Post	kwo2	Object	Contrastive	r1	187.727193441475	2	post_focus	2	Contrastive post_focus

**2023206**	block3	HumanSpeech	Post	suk1	Subject	Narrow	r1	72.6844890002809	1	pre_focus	1	Narrow pre_focus
**2023206**	block3	HumanSpeech	Post	suk1	Subject	Narrow	r1	97.0724752679644	2	pre_focus	1	Narrow pre_focus
**2023206**	block3	HumanSpeech	Post	sei2	Verb	Narrow	r1	142.778730620719	1	on_focus	2	Narrow on_focus
**2023206**	block3	HumanSpeech	Post	svy2	Object	Narrow	r1	162.761017450464	1	post_focus	2	Narrow post_focus
**2023206**	block3	HumanSpeech	Post	kwo2	Object	Narrow	r1	226.146636432361	2	post_focus	2	Narrow post_focus
**2023206**	block3	HumanSpeech	Post	piu2	Subject	Narrow	r1	133.342523878241	1	on_focus	2	Narrow on_focus
**2023206**	block3	HumanSpeech	Post	tse2	Subject	Narrow	r1	120.796685677632	2	on_focus	2	Narrow on_focus
**2023206**	block3	HumanSpeech	Post	tsap1	Verb	Narrow	r1	84.818075208716	1	post_focus	1	Narrow post_focus
**2023206**	block3	HumanSpeech	Post	sy1	Object	Narrow	r1	101.789218717784	1	post_focus	1	Narrow post_focus
**2023206**	block3	HumanSpeech	Post	pau1	Object	Narrow	r1	238.270932433124	2	post_focus	1	Narrow post_focus
**2023206**	block3	HumanSpeech	Post	piu2	Subject	Narrow	r1	72.4389077384302	1	pre_focus	2	Narrow pre_focus
**2023206**	block3	HumanSpeech	Post	tse2	Subject	Narrow	r1	86.7662998275165	2	pre_focus	2	Narrow pre_focus
**2023206**	block3	HumanSpeech	Post	tsap1	Verb	Narrow	r1	67.7185689090436	1	on_focus	1	Narrow on_focus
**2023206**	block3	HumanSpeech	Post	sy1	Object	Narrow	r1	115.295844245694	1	post_focus	1	Narrow post_focus
**2023206**	block3	HumanSpeech	Post	pau1	Object	Narrow	r1	571.223271917461	2	post_focus	1	Narrow post_focus
**2023206**	block3	HumanSpeech	Post	suk1	Subject	Narrow	r1	80.1056041464108	1	on_focus	1	Narrow on_focus
**2023206**	block3	HumanSpeech	Post	suk1	Subject	Narrow	r1	88.5588106210378	2	on_focus	1	Narrow on_focus
**2023206**	block3	HumanSpeech	Post	sei2	Verb	Narrow	r1	123.811791383247	1	post_focus	2	Narrow post_focus
**2023206**	block3	HumanSpeech	Post	svy2	Object	Narrow	r1	120.336356764938	1	post_focus	2	Narrow post_focus
**2023206**	block3	HumanSpeech	Post	kwo2	Object	Narrow	r1	218.621315192735	2	post_focus	2	Narrow post_focus
**2023206**	block3	HumanSpeech	Post	piu2	Subject	Contrastive	r1	155.38196887951	1	pre_focus	2	Contrastive pre_focus
**2023206**	block3	HumanSpeech	Post	tse2	Subject	Contrastive	r1	171.071119356839	2	pre_focus	2	Contrastive pre_focus
**2023206**	block3	HumanSpeech	Post	tsap1	Verb	Contrastive	r1	113.007999496091	1	on_focus	1	Contrastive on_focus
**2023206**	block3	HumanSpeech	Post	sy1	Object	Contrastive	r1	126.039304610742	1	post_focus	1	Contrastive post_focus
**2023206**	block3	HumanSpeech	Post	pau1	Object	Contrastive	r1	361.570082578481	2	post_focus	1	Contrastive post_focus
**2023206**	block3	HumanSpeech	Post	piu2	Subject	Narrow	r1	152.838694617628	1	pre_focus	2	Narrow pre_focus
**2023206**	block3	HumanSpeech	Post	tse2	Subject	Narrow	r1	149.054975991845	2	pre_focus	2	Narrow pre_focus
**2023206**	block3	HumanSpeech	Post	tsap1	Verb	Narrow	r1	155.39452981335	1	pre_focus	1	Narrow pre_focus
**2023206**	block3	HumanSpeech	Post	sy1	Object	Narrow	r1	116.225099358729	1	on_focus	1	Narrow on_focus
**2023206**	block3	HumanSpeech	Post	pau1	Object	Narrow	r1	434.893384254309	2	on_focus	1	Narrow on_focus
**2023206**	block3	HumanSpeech	Post	suk1	Subject	Contrastive	r1	70.8264833711212	1	pre_focus	1	Contrastive pre_focus
**2023206**	block3	HumanSpeech	Post	suk1	Subject	Contrastive	r1	82.4788915150805	2	pre_focus	1	Contrastive pre_focus
**2023206**	block3	HumanSpeech	Post	sei2	Verb	Contrastive	r1	127.946973661267	1	on_focus	2	Contrastive on_focus
**2023206**	block3	HumanSpeech	Post	svy2	Object	Contrastive	r1	185.56756035747	1	post_focus	2	Contrastive post_focus
**2023206**	block3	HumanSpeech	Post	kwo2	Object	Contrastive	r1	394.338942594572	2	post_focus	2	Contrastive post_focus
**2023206**	block3	HumanSpeech	Post	piu2	Subject	Contrastive	r1	139.726953438014	1	pre_focus	2	Contrastive pre_focus
**2023206**	block3	HumanSpeech	Post	tse2	Subject	Contrastive	r1	148.623363323793	2	pre_focus	2	Contrastive pre_focus
**2023206**	block3	HumanSpeech	Post	tsap1	Verb	Contrastive	r1	100.821985250548	1	pre_focus	1	Contrastive pre_focus
**2023206**	block3	HumanSpeech	Post	sy1	Object	Contrastive	r1	104.223689475788	1	on_focus	1	Contrastive on_focus
**2023206**	block3	HumanSpeech	Post	pau1	Object	Contrastive	r1	243.231093607022	2	on_focus	1	Contrastive on_focus
**2023206**	block3	HumanSpeech	Post	suk1	Subject	Contrastive	r1	80.040103158268	1	pre_focus	1	Contrastive pre_focus
**2023206**	block3	HumanSpeech	Post	suk1	Subject	Contrastive	r1	109.410873458501	2	pre_focus	1	Contrastive pre_focus
**2023206**	block3	HumanSpeech	Post	sei2	Verb	Contrastive	r1	125.181081956583	1	pre_focus	2	Contrastive pre_focus
**2023206**	block3	HumanSpeech	Post	svy2	Object	Contrastive	r1	163.442351299494	1	on_focus	2	Contrastive on_focus
**2023206**	block3	HumanSpeech	Post	kwo2	Object	Contrastive	r1	268.598693445625	2	on_focus	2	Contrastive on_focus
**2023206**	block3	HumanSpeech	Post	suk1	Subject	Broad	r1	118.93914322485	1	broad_focus	1	Broad focus
**2023206**	block3	HumanSpeech	Post	suk1	Subject	Broad	r1	115.18261526831	2	broad_focus	1	Broad focus
**2023206**	block3	HumanSpeech	Post	sei2	Verb	Broad	r1	145.561928475104	1	broad_focus	2	Broad focus
**2023206**	block3	HumanSpeech	Post	svy2	Object	Broad	r1	129.848559371851	1	broad_focus	2	Broad focus
**2023206**	block3	HumanSpeech	Post	kwo2	Object	Broad	r1	151.21273394152	2	broad_focus	2	Broad focus
**2023206**	block3	HumanSpeech	Post	bui3	Subject	Contrastive	r1	120.671201814048	1	pre_focus	3	Contrastive pre_focus
**2023206**	block3	HumanSpeech	Post	bui3	Subject	Contrastive	r1	112.415756613387	2	pre_focus	3	Contrastive pre_focus
**2023206**	block3	HumanSpeech	Post	tsv1	Verb	Contrastive	r1	142.224967693153	1	on_focus	1	Contrastive on_focus
**2023206**	block3	HumanSpeech	Post	fug1	Object	Contrastive	r1	137.86324663576	1	post_focus	1	Contrastive post_focus
**2023206**	block3	HumanSpeech	Post	tshe1	Object	Contrastive	r1	247.73476586401	2	post_focus	1	Contrastive post_focus
**2023206**	block3	HumanSpeech	Post	bui3	Subject	Narrow	r1	141.290897311308	1	on_focus	3	Narrow on_focus
**2023206**	block3	HumanSpeech	Post	bui3	Subject	Narrow	r1	100.761256883715	2	on_focus	3	Narrow on_focus
**2023206**	block3	HumanSpeech	Post	tsv1	Verb	Narrow	r1	67.7324263038486	1	post_focus	1	Narrow post_focus
**2023206**	block3	HumanSpeech	Post	fug1	Object	Narrow	r1	146.987906273608	1	post_focus	1	Narrow post_focus
**2023206**	block3	HumanSpeech	Post	tshe1	Object	Narrow	r1	175.956984814121	2	post_focus	1	Narrow post_focus
**2023206**	block3	HumanSpeech	Post	bui3	Subject	Contrastive	r1	68.6780045351725	1	pre_focus	3	Contrastive pre_focus
**2023206**	block3	HumanSpeech	Post	bui3	Subject	Contrastive	r1	84.215942787381	2	pre_focus	3	Contrastive pre_focus

2023206	block3	HumanSpeech	Post	tsv1	Verb	Contrastive	r1	87.8314076953188	1	pre_focus	1	Contrastive pre_focus
2023206	block3	HumanSpeech	Post	fug1	Object	Contrastive	r1	114.897959183679	1	on_focus	1	Contrastive on_focus
2023206	block3	HumanSpeech	Post	tshe1	Object	Contrastive	r1	188.342403628127	2	on_focus	1	Contrastive on_focus
2023206	block3	HumanSpeech	Post	bui3	Subject	Narrow	r1	116.444720432014	1	pre_focus	3	Narrow pre_focus
2023206	block3	HumanSpeech	Post	bui3	Subject	Narrow	r1	59.0740740740614	2	pre_focus	3	Narrow pre_focus
2023206	block3	HumanSpeech	Post	tsv1	Verb	Narrow	r1	108.149400712648	1	pre_focus	1	Narrow pre_focus
2023206	block3	HumanSpeech	Post	fug1	Object	Narrow	r1	128.174535586709	1	on_focus	1	Narrow on_focus
2023206	block3	HumanSpeech	Post	tshe1	Object	Narrow	r1	204.491475602595	2	on_focus	1	Narrow on_focus
2023206	block3	HumanSpeech	Post	piu2	Subject	Contrastive	r1	138.248271393934	1	on_focus	2	Contrastive on_focus
2023206	block3	HumanSpeech	Post	tse2	Subject	Contrastive	r1	115.5859656524	2	on_focus	2	Contrastive on_focus
2023206	block3	HumanSpeech	Post	tsap1	Verb	Contrastive	r1	111.240779304921	1	post_focus	1	Contrastive post_focus
2023206	block3	HumanSpeech	Post	sy1	Object	Contrastive	r1	140.347173352069	1	post_focus	1	Contrastive post_focus
2023206	block3	HumanSpeech	Post	pau1	Object	Contrastive	r1	283.092997146667	2	post_focus	1	Contrastive post_focus
2023206	block3	HumanSpeech	Post	suk1	Subject	Narrow	r1	92.0326036397228	1	pre_focus	1	Narrow pre_focus
2023206	block3	HumanSpeech	Post	suk1	Subject	Narrow	r1	89.3173191132348	2	pre_focus	1	Narrow pre_focus
2023206	block3	HumanSpeech	Post	sei2	Verb	Narrow	r1	132.365073793636	1	pre_focus	2	Narrow pre_focus
2023206	block3	HumanSpeech	Post	svy2	Object	Narrow	r1	175.524525599712	1	on_focus	2	Narrow on_focus
2023206	block3	HumanSpeech	Post	kwo2	Object	Narrow	r1	263.420207910002	2	on_focus	2	Narrow on_focus
2023206	block3	HumanSpeech	Post	suk1	Subject	Contrastive	r2	51.2188208616919	1	pre_focus	1	Contrastive pre_focus
2023206	block3	HumanSpeech	Post	suk1	Subject	Contrastive	r2	83.9885044926802	2	pre_focus	1	Contrastive pre_focus
2023206	block3	HumanSpeech	Post	sei2	Verb	Contrastive	r2	116.755308183912	1	pre_focus	2	Contrastive pre_focus
2023206	block3	HumanSpeech	Post	svy2	Object	Contrastive	r2	164.555424734004	1	on_focus	2	Contrastive on_focus
2023206	block3	HumanSpeech	Post	kwo2	Object	Contrastive	r2	238.278263686425	2	on_focus	2	Contrastive on_focus
2023206	block3	HumanSpeech	Post	bui3	Subject	Narrow	r2	91.4373897707037	1	on_focus	3	Narrow on_focus
2023206	block3	HumanSpeech	Post	bui3	Subject	Narrow	r2	137.780519615262	2	on_focus	3	Narrow on_focus
2023206	block3	HumanSpeech	Post	tsv1	Verb	Narrow	r2	122.596875787337	1	post_focus	1	Narrow post_focus
2023206	block3	HumanSpeech	Post	fug1	Object	Narrow	r2	133.612244897961	1	post_focus	1	Narrow post_focus
2023206	block3	HumanSpeech	Post	tshe1	Object	Narrow	r2	277.528344671197	2	post_focus	1	Narrow post_focus
2023206	block3	HumanSpeech	Post	suk1	Subject	Narrow	r2	66.6776609633644	1	on_focus	1	Narrow on_focus
2023206	block3	HumanSpeech	Post	suk1	Subject	Narrow	r2	54.2862811791451	2	on_focus	1	Narrow on_focus
2023206	block3	HumanSpeech	Post	sei2	Verb	Narrow	r2	97.1326530612373	1	post_focus	2	Narrow post_focus
2023206	block3	HumanSpeech	Post	svy2	Object	Narrow	r2	135.370370370367	1	post_focus	2	Narrow post_focus
2023206	block3	HumanSpeech	Post	kwo2	Object	Narrow	r2	186.224489795933	2	post_focus	2	Narrow post_focus
2023206	block3	HumanSpeech	Post	piu2	Subject	Contrastive	r2	115.531049846851	1	on_focus	2	Contrastive on_focus
2023206	block3	HumanSpeech	Post	tse2	Subject	Contrastive	r2	123.822278911547	2	on_focus	2	Contrastive on_focus
2023206	block3	HumanSpeech	Post	tsap1	Verb	Contrastive	r2	102.893001356392	1	post_focus	1	Contrastive post_focus
2023206	block3	HumanSpeech	Post	sy1	Object	Contrastive	r2	71.7646374789069	1	post_focus	1	Contrastive post_focus
2023206	block3	HumanSpeech	Post	pau1	Object	Contrastive	r2	205.541068279189	2	post_focus	1	Contrastive post_focus
2023206	block3	HumanSpeech	Post	bui3	Subject	Broad	r2	123.312510189265	1	broad_focus	3	Broad focus
2023206	block3	HumanSpeech	Post	bui3	Subject	Broad	r2	133.242449625698	2	broad_focus	3	Broad focus
2023206	block3	HumanSpeech	Post	tsv1	Verb	Broad	r2	108.455644145351	1	broad_focus	1	Broad focus
2023206	block3	HumanSpeech	Post	fug1	Object	Broad	r2	193.46132037515	1	broad_focus	1	Broad focus
2023206	block3	HumanSpeech	Post	tshe1	Object	Broad	r2	236.539515798938	2	broad_focus	1	Broad focus
2023206	block3	HumanSpeech	Post	bui3	Subject	Narrow	r2	68.5943617212956	1	pre_focus	3	Narrow pre_focus
2023206	block3	HumanSpeech	Post	bui3	Subject	Narrow	r2	131.872476729626	2	pre_focus	3	Narrow pre_focus
2023206	block3	HumanSpeech	Post	tsv1	Verb	Narrow	r2	106.854836002185	1	on_focus	1	Narrow on_focus
2023206	block3	HumanSpeech	Post	fug1	Object	Narrow	r2	152.111682554249	1	post_focus	1	Narrow post_focus
2023206	block3	HumanSpeech	Post	tshe1	Object	Narrow	r2	263.28620899838	2	post_focus	1	Narrow post_focus
2023206	block3	HumanSpeech	Post	bui3	Subject	Contrastive	r2	129.482355442178	1	on_focus	3	Contrastive on_focus
2023206	block3	HumanSpeech	Post	bui3	Subject	Contrastive	r2	179.076607915903	2	on_focus	3	Contrastive on_focus
2023206	block3	HumanSpeech	Post	tsv1	Verb	Contrastive	r2	114.478565010927	1	post_focus	1	Contrastive post_focus
2023206	block3	HumanSpeech	Post	fug1	Object	Contrastive	r2	194.319968508069	1	post_focus	1	Contrastive post_focus
2023206	block3	HumanSpeech	Post	tshe1	Object	Contrastive	r2	250.024628580547	2	post_focus	1	Contrastive post_focus
2023206	block3	HumanSpeech	Post	suk1	Subject	Narrow	r2	44.436318972032	1	pre_focus	1	Narrow pre_focus
2023206	block3	HumanSpeech	Post	suk1	Subject	Narrow	r2	67.7969894425416	2	pre_focus	1	Narrow pre_focus
2023206	block3	HumanSpeech	Post	sei2	Verb	Narrow	r2	111.356656149326	1	on_focus	2	Narrow on_focus
2023206	block3	HumanSpeech	Post	svy2	Object	Narrow	r2	123.567458917734	1	post_focus	2	Narrow post_focus
2023206	block3	HumanSpeech	Post	kwo2	Object	Narrow	r2	273.445578231275	2	post_focus	2	Narrow post_focus
2023206	block3	HumanSpeech	Post	piu2	Subject	Narrow	r2	68.1212570498246	1	pre_focus	2	Narrow pre_focus
2023206	block3	HumanSpeech	Post	tse2	Subject	Narrow	r2	99.9480077745432	2	pre_focus	2	Narrow pre_focus
2023206	block3	HumanSpeech	Post	tsap1	Verb	Narrow	r2	82.1863006969181	1	pre_focus	1	Narrow pre_focus
2023206	block3	HumanSpeech	Post	sy1	Object	Narrow	r2	82.9403952056964	1	on_focus	1	Narrow on_focus

**2023206**	block3	HumanSpeech	Post	pau1	Object	Narrow	r2	254.700018896443	2	on_focus	1	Narrow on_focus
**2023206**	block3	HumanSpeech	Post	suk1	Subject	Contrastive	r2	62.7520786092646	1	on_focus	1	Contrastive on_focus
**2023206**	block3	HumanSpeech	Post	suk1	Subject	Contrastive	r2	94.2055933484198	2	on_focus	1	Contrastive on_focus
**2023206**	block3	HumanSpeech	Post	sei2	Verb	Contrastive	r2	111.056630444409	1	post_focus	2	Contrastive post_focus
**2023206**	block3	HumanSpeech	Post	svy2	Object	Contrastive	r2	157.591512795591	1	post_focus	2	Contrastive post_focus
**2023206**	block3	HumanSpeech	Post	kwo2	Object	Contrastive	r2	200.57090528519	2	post_focus	2	Contrastive post_focus
**2023206**	block3	HumanSpeech	Post	piu2	Subject	Contrastive	r2	119.736313573014	1	pre_focus	2	Contrastive pre_focus
**2023206**	block3	HumanSpeech	Post	tse2	Subject	Contrastive	r2	111.586221790333	2	pre_focus	2	Contrastive pre_focus
**2023206**	block3	HumanSpeech	Post	tsap1	Verb	Contrastive	r2	61.7932728646906	1	on_focus	1	Contrastive on_focus
**2023206**	block3	HumanSpeech	Post	sy1	Object	Contrastive	r2	40.9111523397314	1	post_focus	1	Contrastive post_focus
**2023206**	block3	HumanSpeech	Post	pau1	Object	Contrastive	r2	228.476190476215	2	post_focus	1	Contrastive post_focus
**2023206**	block3	HumanSpeech	Post	piu2	Subject	Contrastive	r2	35.9021893357294	1	pre_focus	2	Contrastive pre_focus
**2023206**	block3	HumanSpeech	Post	tse2	Subject	Contrastive	r2	112.804988662219	2	pre_focus	2	Contrastive pre_focus
**2023206**	block3	HumanSpeech	Post	tsap1	Verb	Contrastive	r2	97.6849152359591	1	pre_focus	1	Contrastive pre_focus
**2023206**	block3	HumanSpeech	Post	sy1	Object	Contrastive	r2	76.4838864839135	1	on_focus	1	Contrastive on_focus
**2023206**	block3	HumanSpeech	Post	pau1	Object	Contrastive	r2	175.789987789983	2	on_focus	1	Contrastive on_focus
**2023206**	block3	HumanSpeech	Post	piu2	Subject	Broad	r2	118.996083281729	1	broad_focus	2	Broad focus
**2023206**	block3	HumanSpeech	Post	tse2	Subject	Broad	r2	104.898217395998	2	broad_focus	2	Broad focus
**2023206**	block3	HumanSpeech	Post	tsap1	Verb	Broad	r2	75.7388669753709	1	broad_focus	1	Broad focus
**2023206**	block3	HumanSpeech	Post	sy1	Object	Broad	r2	58.0876795162339	1	broad_focus	1	Broad focus
**2023206**	block3	HumanSpeech	Post	pau1	Object	Broad	r2	246.896490051427	2	broad_focus	1	Broad focus
**2023206**	block3	HumanSpeech	Post	suk1	Subject	Narrow	r2	65.0158730158523	1	pre_focus	1	Narrow pre_focus
**2023206**	block3	HumanSpeech	Post	suk1	Subject	Narrow	r2	90.5949231544696	2	pre_focus	1	Narrow pre_focus
**2023206**	block3	HumanSpeech	Post	sei2	Verb	Narrow	r2	107.679680567855	1	pre_focus	2	Narrow pre_focus
**2023206**	block3	HumanSpeech	Post	svy2	Object	Narrow	r2	103.825963718805	1	on_focus	2	Narrow on_focus
**2023206**	block3	HumanSpeech	Post	kwo2	Object	Narrow	r2	201.083900226763	2	on_focus	2	Narrow on_focus
**2023206**	block3	HumanSpeech	Post	bui3	Subject	Narrow	r2	53.324514991175	1	pre_focus	3	Narrow pre_focus
**2023206**	block3	HumanSpeech	Post	bui3	Subject	Narrow	r2	80.8017492711315	2	pre_focus	3	Narrow pre_focus
**2023206**	block3	HumanSpeech	Post	tsv1	Verb	Narrow	r2	102.06954751601	1	pre_focus	1	Narrow pre_focus
**2023206**	block3	HumanSpeech	Post	fug1	Object	Narrow	r2	139.088807825829	1	on_focus	1	Narrow on_focus
**2023206**	block3	HumanSpeech	Post	tshe1	Object	Narrow	r2	222.781179138337	2	on_focus	1	Narrow on_focus
**2023206**	block3	HumanSpeech	Post	piu2	Subject	Narrow	r2	98.8095238095639	1	on_focus	2	Narrow on_focus
**2023206**	block3	HumanSpeech	Post	tse2	Subject	Narrow	r2	119.911016597882	2	on_focus	2	Narrow on_focus
**2023206**	block3	HumanSpeech	Post	tsap1	Verb	Narrow	r2	67.7876696572639	1	post_focus	1	Narrow post_focus
**2023206**	block3	HumanSpeech	Post	sy1	Object	Narrow	r2	80.4245136650934	1	post_focus	1	Narrow post_focus
**2023206**	block3	HumanSpeech	Post	pau1	Object	Narrow	r2	210.980210265916	2	post_focus	1	Narrow post_focus
**2023206**	block3	HumanSpeech	Post	piu2	Subject	Narrow	r2	62.1860827664591	1	pre_focus	2	Narrow pre_focus
**2023206**	block3	HumanSpeech	Post	tse2	Subject	Narrow	r2	69.9768612267349	2	pre_focus	2	Narrow pre_focus
**2023206**	block3	HumanSpeech	Post	tsap1	Verb	Narrow	r2	82.6281179138277	1	on_focus	1	Narrow on_focus
**2023206**	block3	HumanSpeech	Post	sy1	Object	Narrow	r2	71.5733472876536	1	post_focus	1	Narrow post_focus
**2023206**	block3	HumanSpeech	Post	pau1	Object	Narrow	r2	242.27974238363	2	post_focus	1	Narrow post_focus
**2023206**	block3	HumanSpeech	Post	bui3	Subject	Contrastive	r2	75.6898823021288	1	pre_focus	3	Contrastive pre_focus
**2023206**	block3	HumanSpeech	Post	bui3	Subject	Contrastive	r2	106.073790627363	2	pre_focus	3	Contrastive pre_focus
**2023206**	block3	HumanSpeech	Post	tsv1	Verb	Contrastive	r2	71.072670338026	1	pre_focus	1	Contrastive pre_focus
**2023206**	block3	HumanSpeech	Post	fug1	Object	Contrastive	r2	106.848726670137	1	on_focus	1	Contrastive on_focus
**2023206**	block3	HumanSpeech	Post	tshe1	Object	Contrastive	r2	162.268375363624	2	on_focus	1	Contrastive on_focus
**2023206**	block3	HumanSpeech	Post	bui3	Subject	Contrastive	r2	84.7978901705915	1	pre_focus	3	Contrastive pre_focus
**2023206**	block3	HumanSpeech	Post	bui3	Subject	Contrastive	r2	120.258071482567	2	pre_focus	3	Contrastive pre_focus
**2023206**	block3	HumanSpeech	Post	tsv1	Verb	Contrastive	r2	102.816245545853	1	on_focus	1	Contrastive on_focus
**2023206**	block3	HumanSpeech	Post	fug1	Object	Contrastive	r2	146.611711351227	1	post_focus	1	Contrastive post_focus
**2023206**	block3	HumanSpeech	Post	tshe1	Object	Contrastive	r2	187.509562747664	2	post_focus	1	Contrastive post_focus
**2023206**	block3	HumanSpeech	Post	suk1	Subject	Contrastive	r2	30.8102869352638	1	pre_focus	1	Contrastive pre_focus
**2023206**	block3	HumanSpeech	Post	suk1	Subject	Contrastive	r2	50.4583963215168	2	pre_focus	1	Contrastive pre_focus
**2023206**	block3	HumanSpeech	Post	sei2	Verb	Contrastive	r2	74.2287669897337	1	on_focus	2	Contrastive on_focus
**2023206**	block3	HumanSpeech	Post	svy2	Object	Contrastive	r2	138.089342403589	1	post_focus	2	Contrastive post_focus
**2023206**	block3	HumanSpeech	Post	kwo2	Object	Contrastive	r2	170.293556311435	2	post_focus	2	Contrastive post_focus
**2023206**	block3	HumanSpeech	Post	suk1	Subject	Broad	r2	121.943985323583	1	broad_focus	1	Broad focus
**2023206**	block3	HumanSpeech	Post	suk1	Subject	Broad	r2	97.1308409403946	2	broad_focus	1	Broad focus
**2023206**	block3	HumanSpeech	Post	sei2	Verb	Broad	r2	102.543684140244	1	broad_focus	2	Broad focus
**2023206**	block3	HumanSpeech	Post	svy2	Object	Broad	r2	112.811403763772	1	broad_focus	2	Broad focus
**2023206**	block3	HumanSpeech	Post	kwo2	Object	Broad	r2	233.151927437632	2	broad_focus	2	Broad focus
**2023206**	block3	HumanSpeech	Pre	suk1	Subject	Narrow	r1	81.4367346938809	1	pre_focus	1	Narrow pre_focus

2023206	block3	HumanSpeech	Pre	suk1	Subject	Narrow	r1	78.1738473167195	2	pre_focus	1	Narrow pre_focus
2023206	block3	HumanSpeech	Pre	sei2	Verb	Narrow	r1	123.611740992715	1	on_focus	2	Narrow on_focus
2023206	block3	HumanSpeech	Pre	svy2	Object	Narrow	r1	177.941636972264	1	post_focus	2	Narrow post_focus
2023206	block3	HumanSpeech	Pre	kwo2	Object	Narrow	r1	316.146636432336	2	post_focus	2	Narrow post_focus
2023206	block3	HumanSpeech	Pre	bui3	Subject	Narrow	r1	120.802559118886	1	pre_focus	3	Narrow pre_focus
2023206	block3	HumanSpeech	Pre	bui3	Subject	Narrow	r1	122.987150415724	2	pre_focus	3	Narrow pre_focus
2023206	block3	HumanSpeech	Pre	tsv1	Verb	Narrow	r1	149.399092970498	1	pre_focus	1	Narrow pre_focus
2023206	block3	HumanSpeech	Pre	fug1	Object	Narrow	r1	333.842277651797	1	on_focus	1	Narrow on_focus
2023206	block3	HumanSpeech	Pre	tshe1	Object	Narrow	r1	212.232300327543	2	on_focus	1	Narrow on_focus
2023206	block3	HumanSpeech	Pre	piu2	Subject	Broad	r1	258.514739229042	1	broad_focus	2	Broad focus
2023206	block3	HumanSpeech	Pre	tse2	Subject	Broad	r1	212.023179642216	2	broad_focus	2	Broad focus
2023206	block3	HumanSpeech	Pre	tsap1	Verb	Broad	r1	103.515243134296	1	broad_focus	1	Broad focus
2023206	block3	HumanSpeech	Pre	sy1	Object	Broad	r1	135.487528344697	1	broad_focus	1	Broad focus
2023206	block3	HumanSpeech	Pre	pau1	Object	Broad	r1	313.581349206345	2	broad_focus	1	Broad focus
2023206	block3	HumanSpeech	Pre	piu2	Subject	Narrow	r1	235.56689342405	1	pre_focus	2	Narrow pre_focus
2023206	block3	HumanSpeech	Pre	tse2	Subject	Narrow	r1	181.077097505664	2	pre_focus	2	Narrow pre_focus
2023206	block3	HumanSpeech	Pre	tsap1	Verb	Narrow	r1	119.235027344303	1	on_focus	1	Narrow on_focus
2023206	block3	HumanSpeech	Pre	sy1	Object	Narrow	r1	146.672335600925	1	post_focus	1	Narrow post_focus
2023206	block3	HumanSpeech	Pre	pau1	Object	Narrow	r1	331.219016342203	2	post_focus	1	Narrow post_focus
2023206	block3	HumanSpeech	Pre	suk1	Subject	Narrow	r1	59.5853579526988	1	pre_focus	1	Narrow pre_focus
2023206	block3	HumanSpeech	Pre	suk1	Subject	Narrow	r1	82.2902494331004	2	pre_focus	1	Narrow pre_focus
2023206	block3	HumanSpeech	Pre	sei2	Verb	Narrow	r1	150.196745364809	1	pre_focus	2	Narrow pre_focus
2023206	block3	HumanSpeech	Pre	svy2	Object	Narrow	r1	184.69329612185	1	on_focus	2	Narrow on_focus
2023206	block3	HumanSpeech	Pre	kwo2	Object	Narrow	r1	338.45049130764	2	on_focus	2	Narrow on_focus
2023206	block3	HumanSpeech	Pre	piu2	Subject	Contrastive	r1	212.816565222568	1	on_focus	2	Contrastive on_focus
2023206	block3	HumanSpeech	Pre	tse2	Subject	Contrastive	r1	207.414965986374	2	on_focus	2	Contrastive on_focus
2023206	block3	HumanSpeech	Pre	tsap1	Verb	Contrastive	r1	115.715041572173	1	post_focus	1	Contrastive post_focus
2023206	block3	HumanSpeech	Pre	sy1	Object	Contrastive	r1	109.442176870772	1	post_focus	1	Contrastive post_focus
2023206	block3	HumanSpeech	Pre	pau1	Object	Contrastive	r1	459.378516838825	2	post_focus	1	Contrastive post_focus
2023206	block3	HumanSpeech	Pre	suk1	Subject	Broad	r1	89.7891156462549	1	broad_focus	1	Broad focus
2023206	block3	HumanSpeech	Pre	suk1	Subject	Broad	r1	121.661807580182	2	broad_focus	1	Broad focus
2023206	block3	HumanSpeech	Pre	sei2	Verb	Broad	r1	495.893630179353	1	broad_focus	2	Broad focus
2023206	block3	HumanSpeech	Pre	svy2	Object	Broad	r1	200.408163265308	1	broad_focus	2	Broad focus
2023206	block3	HumanSpeech	Pre	kwo2	Object	Broad	r1	344.261418853279	2	broad_focus	2	Broad focus
2023206	block3	HumanSpeech	Pre	suk1	Subject	Narrow	r1	92.1830795810195	1	on_focus	1	Narrow on_focus
2023206	block3	HumanSpeech	Pre	suk1	Subject	Narrow	r1	158.338486909912	2	on_focus	1	Narrow on_focus
2023206	block3	HumanSpeech	Pre	sei2	Verb	Narrow	r1	237.670697908783	1	post_focus	2	Narrow post_focus
2023206	block3	HumanSpeech	Pre	svy2	Object	Narrow	r1	227.315192743788	1	post_focus	2	Narrow post_focus
2023206	block3	HumanSpeech	Pre	kwo2	Object	Narrow	r1	393.715581470701	2	post_focus	2	Narrow post_focus
2023206	block3	HumanSpeech	Pre	suk1	Subject	Contrastive	r1	67.5136721355329	1	on_focus	1	Contrastive on_focus
2023206	block3	HumanSpeech	Pre	suk1	Subject	Contrastive	r1	65.8365972651609	2	on_focus	1	Contrastive on_focus
2023206	block3	HumanSpeech	Pre	sei2	Verb	Contrastive	r1	147.888726919348	1	post_focus	2	Contrastive post_focus
2023206	block3	HumanSpeech	Pre	svy2	Object	Contrastive	r1	176.217876039289	1	post_focus	2	Contrastive post_focus
2023206	block3	HumanSpeech	Pre	kwo2	Object	Contrastive	r1	333.492063492088	2	post_focus	2	Contrastive post_focus
2023206	block3	HumanSpeech	Pre	suk1	Subject	Contrastive	r1	71.2232300327571	1	pre_focus	1	Contrastive pre_focus
2023206	block3	HumanSpeech	Pre	suk1	Subject	Contrastive	r1	94.4891991884731	2	pre_focus	1	Contrastive pre_focus
2023206	block3	HumanSpeech	Pre	sei2	Verb	Contrastive	r1	192.30130147298	1	on_focus	2	Contrastive on_focus
2023206	block3	HumanSpeech	Pre	svy2	Object	Contrastive	r1	232.286470143606	1	post_focus	2	Contrastive post_focus
2023206	block3	HumanSpeech	Pre	kwo2	Object	Contrastive	r1	352.970521541948	2	post_focus	2	Contrastive post_focus
2023206	block3	HumanSpeech	Pre	bui3	Subject	Narrow	r1	229.794501133796	1	pre_focus	3	Narrow pre_focus
2023206	block3	HumanSpeech	Pre	bui3	Subject	Narrow	r1	227.535714285693	2	pre_focus	3	Narrow pre_focus
2023206	block3	HumanSpeech	Pre	tsv1	Verb	Narrow	r1	149.151927437657	1	on_focus	1	Narrow on_focus
2023206	block3	HumanSpeech	Pre	fug1	Object	Narrow	r1	174.991496598636	1	post_focus	1	Narrow post_focus
2023206	block3	HumanSpeech	Pre	tshe1	Object	Narrow	r1	194.37452758882	2	post_focus	1	Narrow post_focus
2023206	block3	HumanSpeech	Pre	bui3	Subject	Contrastive	r1	244.930353093622	1	pre_focus	3	Contrastive pre_focus
2023206	block3	HumanSpeech	Pre	bui3	Subject	Contrastive	r1	211.065759637194	2	pre_focus	3	Contrastive pre_focus
2023206	block3	HumanSpeech	Pre	tsv1	Verb	Contrastive	r1	181.654033041809	1	on_focus	1	Contrastive on_focus
2023206	block3	HumanSpeech	Pre	fug1	Object	Contrastive	r1	193.856764928199	1	post_focus	1	Contrastive post_focus
2023206	block3	HumanSpeech	Pre	tshe1	Object	Contrastive	r1	267.541950113355	2	post_focus	1	Contrastive post_focus
2023206	block3	HumanSpeech	Pre	bui3	Subject	Contrastive	r1	270.878684807258	1	on_focus	3	Contrastive on_focus
2023206	block3	HumanSpeech	Pre	bui3	Subject	Contrastive	r1	214.612082928426	2	on_focus	3	Contrastive on_focus
2023206	block3	HumanSpeech	Pre	tsv1	Verb	Contrastive	r1	138.722600151169	1	post_focus	1	Contrastive post_focus

**2023206**	block3	HumanSpeech	Pre	fug1	Object	Contrastive	r1	176.349206349187	1	post_focus	1	Contrastive post_focus
**2023206**	block3	HumanSpeech	Pre	tshe1	Object	Contrastive	r1	277.435122197033	2	post_focus	1	Contrastive post_focus
**2023206**	block3	HumanSpeech	Pre	piu2	Subject	Narrow	r1	234.984539270243	1	on_focus	2	Narrow on_focus
**2023206**	block3	HumanSpeech	Pre	tse2	Subject	Narrow	r1	166.837765409184	2	on_focus	2	Narrow on_focus
**2023206**	block3	HumanSpeech	Pre	tsap1	Verb	Narrow	r1	124.511715797439	1	post_focus	1	Narrow post_focus
**2023206**	block3	HumanSpeech	Pre	sy1	Object	Narrow	r1	136.403061224513	1	post_focus	1	Narrow post_focus
**2023206**	block3	HumanSpeech	Pre	pau1	Object	Narrow	r1	306.381158524033	2	post_focus	1	Narrow post_focus
**2023206**	block3	HumanSpeech	Pre	piu2	Subject	Narrow	r1	231.819098009595	1	pre_focus	2	Narrow pre_focus
**2023206**	block3	HumanSpeech	Pre	tse2	Subject	Narrow	r1	166.247921390777	2	pre_focus	2	Narrow pre_focus
**2023206**	block3	HumanSpeech	Pre	tsap1	Verb	Narrow	r1	125.984504913077	1	pre_focus	1	Narrow pre_focus
**2023206**	block3	HumanSpeech	Pre	sy1	Object	Narrow	r1	178.018707483005	1	on_focus	1	Narrow on_focus
**2023206**	block3	HumanSpeech	Pre	pau1	Object	Narrow	r1	281.730516768135	2	on_focus	1	Narrow on_focus
**2023206**	block3	HumanSpeech	Pre	suk1	Subject	Contrastive	r1	86.5910808768149	1	pre_focus	1	Contrastive pre_focus
**2023206**	block3	HumanSpeech	Pre	suk1	Subject	Contrastive	r1	87.9658530078586	2	pre_focus	1	Contrastive pre_focus
**2023206**	block3	HumanSpeech	Pre	sei2	Verb	Contrastive	r1	375.924036281219	1	pre_focus	2	Contrastive pre_focus
**2023206**	block3	HumanSpeech	Pre	svy2	Object	Contrastive	r1	202.991452991455	1	on_focus	2	Contrastive on_focus
**2023206**	block3	HumanSpeech	Pre	kwo2	Object	Contrastive	r1	346.329365079384	2	on_focus	2	Contrastive on_focus
**2023206**	block3	HumanSpeech	Pre	bui3	Subject	Narrow	r1	218.052046215291	1	on_focus	3	Narrow on_focus
**2023206**	block3	HumanSpeech	Pre	bui3	Subject	Narrow	r1	217.665592552805	2	on_focus	3	Narrow on_focus
**2023206**	block3	HumanSpeech	Pre	tsv1	Verb	Narrow	r1	127.453838678321	1	post_focus	1	Narrow post_focus
**2023206**	block3	HumanSpeech	Pre	fug1	Object	Narrow	r1	186.129251700663	1	post_focus	1	Narrow post_focus
**2023206**	block3	HumanSpeech	Pre	tshe1	Object	Narrow	r1	193.925332037566	2	post_focus	1	Narrow post_focus
**2023206**	block3	HumanSpeech	Pre	bui3	Subject	Broad	r1	228.031530072371	1	broad_focus	3	Broad focus
**2023206**	block3	HumanSpeech	Pre	bui3	Subject	Broad	r1	199.535147392339	2	broad_focus	3	Broad focus
**2023206**	block3	HumanSpeech	Pre	tsv1	Verb	Broad	r1	161.66099773244	1	broad_focus	1	Broad focus
**2023206**	block3	HumanSpeech	Pre	fug1	Object	Broad	r1	180.177626606167	1	broad_focus	1	Broad focus
**2023206**	block3	HumanSpeech	Pre	tshe1	Object	Broad	r1	239.743764172317	2	broad_focus	1	Broad focus
**2023206**	block3	HumanSpeech	Pre	piu2	Subject	Contrastive	r1	306.707482993147	1	pre_focus	2	Contrastive pre_focus
**2023206**	block3	HumanSpeech	Pre	tse2	Subject	Contrastive	r1	473.623582766436	2	pre_focus	2	Contrastive pre_focus
**2023206**	block3	HumanSpeech	Pre	tsap1	Verb	Contrastive	r1	127.868480725624	1	on_focus	1	Contrastive on_focus
**2023206**	block3	HumanSpeech	Pre	sy1	Object	Contrastive	r1	135.680272108857	1	post_focus	1	Contrastive post_focus
**2023206**	block3	HumanSpeech	Pre	pau1	Object	Contrastive	r1	328.042725862247	2	post_focus	1	Contrastive post_focus
**2023206**	block3	HumanSpeech	Pre	piu2	Subject	Contrastive	r1	233.715986394543	1	pre_focus	2	Contrastive pre_focus
**2023206**	block3	HumanSpeech	Pre	tse2	Subject	Contrastive	r1	206.696900982649	2	pre_focus	2	Contrastive pre_focus
**2023206**	block3	HumanSpeech	Pre	tsap1	Verb	Contrastive	r1	121.756885090178	1	pre_focus	1	Contrastive pre_focus
**2023206**	block3	HumanSpeech	Pre	sy1	Object	Contrastive	r1	135.938208616778	1	on_focus	1	Contrastive on_focus
**2023206**	block3	HumanSpeech	Pre	pau1	Object	Contrastive	r1	321.957024079438	2	on_focus	1	Contrastive on_focus
**2023206**	block3	HumanSpeech	Pre	bui3	Subject	Contrastive	r1	248.635676492881	1	pre_focus	3	Contrastive pre_focus
**2023206**	block3	HumanSpeech	Pre	bui3	Subject	Contrastive	r1	318.752834467148	2	pre_focus	3	Contrastive pre_focus
**2023206**	block3	HumanSpeech	Pre	tsv1	Verb	Contrastive	r1	506.394042465445	1	pre_focus	1	Contrastive pre_focus
**2023206**	block3	HumanSpeech	Pre	fug1	Object	Contrastive	r1	268.429877001324	1	on_focus	1	Contrastive on_focus
**2023206**	block3	HumanSpeech	Pre	tshe1	Object	Contrastive	r1	217.458049886602	2	on_focus	1	Contrastive on_focus
**2023206**	block3	HumanSpeech	Pre	piu2	Subject	Narrow	r2	207.118021811937	1	on_focus	2	Narrow on_focus
**2023206**	block3	HumanSpeech	Pre	tse2	Subject	Narrow	r2	147.806932296703	2	on_focus	2	Narrow on_focus
**2023206**	block3	HumanSpeech	Pre	tsap1	Verb	Narrow	r2	115.872206025301	1	post_focus	1	Narrow post_focus
**2023206**	block3	HumanSpeech	Pre	sy1	Object	Narrow	r2	104.459561602425	1	post_focus	1	Narrow post_focus
**2023206**	block3	HumanSpeech	Pre	pau1	Object	Narrow	r2	311.584975870687	2	post_focus	1	Narrow post_focus
**2023206**	block3	HumanSpeech	Pre	suk1	Subject	Narrow	r2	58.7074829931566	1	on_focus	1	Narrow on_focus
**2023206**	block3	HumanSpeech	Pre	suk1	Subject	Narrow	r2	75.1271034729371	2	on_focus	1	Narrow on_focus
**2023206**	block3	HumanSpeech	Pre	sei2	Verb	Narrow	r2	149.248866213156	1	post_focus	2	Narrow post_focus
**2023206**	block3	HumanSpeech	Pre	svy2	Object	Narrow	r2	180.78458049888	1	post_focus	2	Narrow post_focus
**2023206**	block3	HumanSpeech	Pre	kwo2	Object	Narrow	r2	298.084930942082	2	post_focus	2	Narrow post_focus
**2023206**	block3	HumanSpeech	Pre	suk1	Subject	Broad	r2	58.0907029478226	1	broad_focus	1	Broad focus
**2023206**	block3	HumanSpeech	Pre	suk1	Subject	Broad	r2	70.2725534658271	2	broad_focus	1	Broad focus
**2023206**	block3	HumanSpeech	Pre	sei2	Verb	Broad	r2	179.121553383254	1	broad_focus	2	Broad focus
**2023206**	block3	HumanSpeech	Pre	svy2	Object	Broad	r2	211.428571428542	1	broad_focus	2	Broad focus
**2023206**	block3	HumanSpeech	Pre	kwo2	Object	Broad	r2	309.308390022636	2	broad_focus	2	Broad focus
**2023206**	block3	HumanSpeech	Pre	suk1	Subject	Contrastive	r2	71.5797430082716	1	on_focus	1	Contrastive on_focus
**2023206**	block3	HumanSpeech	Pre	suk1	Subject	Contrastive	r2	82.3603380746363	2	on_focus	1	Contrastive on_focus
**2023206**	block3	HumanSpeech	Pre	sei2	Verb	Contrastive	r2	140.442547928274	1	post_focus	2	Contrastive post_focus
**2023206**	block3	HumanSpeech	Pre	svy2	Object	Contrastive	r2	217.894179894188	1	post_focus	2	Contrastive post_focus
**2023206**	block3	HumanSpeech	Pre	kwo2	Object	Contrastive	r2	375.280207320998	2	post_focus	2	Contrastive post_focus

2023206	block3	HumanSpeech	Pre	piu2	Subject	Narrow	r2	192.117913832192	1	pre_focus	2	Narrow pre_focus
2023206	block3	HumanSpeech	Pre	tse2	Subject	Narrow	r2	135.438397581254	2	pre_focus	2	Narrow pre_focus
2023206	block3	HumanSpeech	Pre	tsap1	Verb	Narrow	r2	114.660430838967	1	pre_focus	1	Narrow pre_focus
2023206	block3	HumanSpeech	Pre	sy1	Object	Narrow	r2	130.534769463395	1	on_focus	1	Narrow on_focus
2023206	block3	HumanSpeech	Pre	pau1	Object	Narrow	r2	305.400368480718	2	on_focus	1	Narrow on_focus
2023206	block3	HumanSpeech	Pre	bui3	Subject	Narrow	r2	187.368669690102	1	pre_focus	3	Narrow pre_focus
2023206	block3	HumanSpeech	Pre	bui3	Subject	Narrow	r2	151.598639455756	2	pre_focus	3	Narrow pre_focus
2023206	block3	HumanSpeech	Pre	tsv1	Verb	Narrow	r2	138.221371882139	1	pre_focus	1	Narrow pre_focus
2023206	block3	HumanSpeech	Pre	fug1	Object	Narrow	r2	169.599395313696	1	on_focus	1	Narrow on_focus
2023206	block3	HumanSpeech	Pre	tshe1	Object	Narrow	r2	237.715419501114	2	on_focus	1	Narrow on_focus
2023206	block3	HumanSpeech	Pre	suk1	Subject	Contrastive	r2	79.438775510198	1	pre_focus	1	Contrastive pre_focus
2023206	block3	HumanSpeech	Pre	suk1	Subject	Contrastive	r2	89.998969284693	2	pre_focus	1	Contrastive pre_focus
2023206	block3	HumanSpeech	Pre	sei2	Verb	Contrastive	r2	168.785228377033	1	on_focus	2	Contrastive on_focus
2023206	block3	HumanSpeech	Pre	svy2	Object	Contrastive	r2	197.539682539684	1	post_focus	2	Contrastive post_focus
2023206	block3	HumanSpeech	Pre	kwo2	Object	Contrastive	r2	332.513227513175	2	post_focus	2	Contrastive post_focus
2023206	block3	HumanSpeech	Pre	suk1	Subject	Contrastive	r2	83.1235827664045	1	pre_focus	1	Contrastive pre_focus
2023206	block3	HumanSpeech	Pre	suk1	Subject	Contrastive	r2	112.066326530623	2	pre_focus	1	Contrastive pre_focus
2023206	block3	HumanSpeech	Pre	sei2	Verb	Contrastive	r2	259.682161753631	1	pre_focus	2	Contrastive pre_focus
2023206	block3	HumanSpeech	Pre	svy2	Object	Contrastive	r2	152.738365187304	1	on_focus	2	Contrastive on_focus
2023206	block3	HumanSpeech	Pre	kwo2	Object	Contrastive	r2	351.274880322478	2	on_focus	2	Contrastive on_focus
2023206	block3	HumanSpeech	Pre	bui3	Subject	Broad	r2	202.709750566896	1	broad_focus	3	Broad focus
2023206	block3	HumanSpeech	Pre	bui3	Subject	Broad	r2	197.227891156501	2	broad_focus	3	Broad focus
2023206	block3	HumanSpeech	Pre	tsv1	Verb	Broad	r2	197.694633408958	1	broad_focus	1	Broad focus
2023206	block3	HumanSpeech	Pre	fug1	Object	Broad	r2	184.649556792408	1	broad_focus	1	Broad focus
2023206	block3	HumanSpeech	Pre	tshe1	Object	Broad	r2	208.959183673471	2	broad_focus	1	Broad focus
2023206	block3	HumanSpeech	Pre	piu2	Subject	Contrastive	r2	301.530612244846	1	pre_focus	2	Contrastive pre_focus
2023206	block3	HumanSpeech	Pre	tse2	Subject	Contrastive	r2	338.818270165177	2	pre_focus	2	Contrastive pre_focus
2023206	block3	HumanSpeech	Pre	tsap1	Verb	Contrastive	r2	95.2465986394486	1	on_focus	1	Contrastive on_focus
2023206	block3	HumanSpeech	Pre	sy1	Object	Contrastive	r2	139.164237123396	1	post_focus	1	Contrastive post_focus
2023206	block3	HumanSpeech	Pre	pau1	Object	Contrastive	r2	312.251984126931	2	post_focus	1	Contrastive post_focus
2023206	block3	HumanSpeech	Pre	piu2	Subject	Contrastive	r2	227.953514739227	1	on_focus	2	Contrastive on_focus
2023206	block3	HumanSpeech	Pre	tse2	Subject	Contrastive	r2	205.181405895701	2	on_focus	2	Contrastive on_focus
2023206	block3	HumanSpeech	Pre	tsap1	Verb	Contrastive	r2	100.659187651672	1	post_focus	1	Contrastive post_focus
2023206	block3	HumanSpeech	Pre	sy1	Object	Contrastive	r2	101.626984126995	1	post_focus	1	Contrastive post_focus
2023206	block3	HumanSpeech	Pre	pau1	Object	Contrastive	r2	313.456349206376	2	post_focus	1	Contrastive post_focus
2023206	block3	HumanSpeech	Pre	bui3	Subject	Contrastive	r2	251.716553287963	1	pre_focus	3	Contrastive pre_focus
2023206	block3	HumanSpeech	Pre	bui3	Subject	Contrastive	r2	212.374768089035	2	pre_focus	3	Contrastive pre_focus
2023206	block3	HumanSpeech	Pre	tsv1	Verb	Contrastive	r2	156.785714285718	1	on_focus	1	Contrastive on_focus
2023206	block3	HumanSpeech	Pre	fug1	Object	Contrastive	r2	204.372637944061	1	post_focus	1	Contrastive post_focus
2023206	block3	HumanSpeech	Pre	tshe1	Object	Contrastive	r2	280.064247921359	2	post_focus	1	Contrastive post_focus
2023206	block3	HumanSpeech	Pre	bui3	Subject	Contrastive	r2	265.223922902464	1	on_focus	3	Contrastive on_focus
2023206	block3	HumanSpeech	Pre	bui3	Subject	Contrastive	r2	209.241780045375	2	on_focus	3	Contrastive on_focus
2023206	block3	HumanSpeech	Pre	tsv1	Verb	Contrastive	r2	151.218260197879	1	post_focus	1	Contrastive post_focus
2023206	block3	HumanSpeech	Pre	fug1	Object	Contrastive	r2	207.403628117959	1	post_focus	1	Contrastive post_focus
2023206	block3	HumanSpeech	Pre	tshe1	Object	Contrastive	r2	178.14144849865	2	post_focus	1	Contrastive post_focus
2023206	block3	HumanSpeech	Pre	bui3	Subject	Narrow	r2	217.010925582372	1	pre_focus	3	Narrow pre_focus
2023206	block3	HumanSpeech	Pre	bui3	Subject	Narrow	r2	159.544805576559	2	pre_focus	3	Narrow pre_focus
2023206	block3	HumanSpeech	Pre	tsv1	Verb	Narrow	r2	140.210884353792	1	on_focus	1	Narrow on_focus
2023206	block3	HumanSpeech	Pre	fug1	Object	Narrow	r2	161.893424036236	1	post_focus	1	Narrow post_focus
2023206	block3	HumanSpeech	Pre	tshe1	Object	Narrow	r2	220.418267080049	2	post_focus	1	Narrow post_focus
2023206	block3	HumanSpeech	Pre	bui3	Subject	Narrow	r2	254.818594104336	1	on_focus	3	Narrow on_focus
2023206	block3	HumanSpeech	Pre	bui3	Subject	Narrow	r2	199.972514258263	2	on_focus	3	Narrow on_focus
2023206	block3	HumanSpeech	Pre	tsv1	Verb	Narrow	r2	143.85140722959	1	post_focus	1	Narrow post_focus
2023206	block3	HumanSpeech	Pre	fug1	Object	Narrow	r2	189.079743008278	1	post_focus	1	Narrow post_focus
2023206	block3	HumanSpeech	Pre	tshe1	Object	Narrow	r2	217.828798185963	2	post_focus	1	Narrow post_focus
2023206	block3	HumanSpeech	Pre	piu2	Subject	Contrastive	r2	251.90800129576	1	pre_focus	2	Contrastive pre_focus
2023206	block3	HumanSpeech	Pre	tse2	Subject	Contrastive	r2	360.959183673458	2	pre_focus	2	Contrastive pre_focus
2023206	block3	HumanSpeech	Pre	tsap1	Verb	Contrastive	r2	139.052658100297	1	pre_focus	1	Contrastive pre_focus
2023206	block3	HumanSpeech	Pre	sy1	Object	Contrastive	r2	137.91836734697	1	on_focus	1	Contrastive on_focus
2023206	block3	HumanSpeech	Pre	pau1	Object	Contrastive	r2	337.001763668411	2	on_focus	1	Contrastive on_focus
2023206	block3	HumanSpeech	Pre	piu2	Subject	Broad	r2	242.284580498847	1	broad_focus	2	Broad focus
2023206	block3	HumanSpeech	Pre	tse2	Subject	Broad	r2	186.927437641771	2	broad_focus	2	Broad focus

2023206	block3	HumanSpeech	Pre	tsap1	Verb	Broad	r2	153.683187560773	1	broad_focus	1	Broad focus
2023206	block3	HumanSpeech	Pre	sy1	Object	Broad	r2	176.37188208613	1	broad_focus	1	Broad focus
2023206	block3	HumanSpeech	Pre	pau1	Object	Broad	r2	359.761904761911	2	broad_focus	1	Broad focus
2023206	block3	HumanSpeech	Pre	suk1	Subject	Narrow	r2	72.5736961451275	1	pre_focus	1	Narrow pre_focus
2023206	block3	HumanSpeech	Pre	suk1	Subject	Narrow	r2	57.0263863121454	2	pre_focus	1	Narrow pre_focus
2023206	block3	HumanSpeech	Pre	sei2	Verb	Narrow	r2	169.276895943597	1	on_focus	2	Narrow on_focus
2023206	block3	HumanSpeech	Pre	svy2	Object	Narrow	r2	189.698412698419	1	post_focus	2	Narrow post_focus
2023206	block3	HumanSpeech	Pre	kwo2	Object	Narrow	r2	379.982993197245	2	post_focus	2	Narrow post_focus
2023206	block3	HumanSpeech	Pre	suk1	Subject	Narrow	r2	56.4569160997621	1	pre_focus	1	Narrow pre_focus
2023206	block3	HumanSpeech	Pre	suk1	Subject	Narrow	r2	77.0944012526229	2	pre_focus	1	Narrow pre_focus
2023206	block3	HumanSpeech	Pre	sei2	Verb	Narrow	r2	167.368583797156	1	pre_focus	2	Narrow pre_focus
2023206	block3	HumanSpeech	Pre	svy2	Object	Narrow	r2	249.142101284974	1	on_focus	2	Narrow on_focus
2023206	block3	HumanSpeech	Pre	kwo2	Object	Narrow	r2	390.600907029523	2	on_focus	2	Narrow on_focus
2023206	block3	HumanSpeech	Pre	bui3	Subject	Contrastive	r2	359.319727891148	1	pre_focus	3	Contrastive pre_focus
2023206	block3	HumanSpeech	Pre	bui3	Subject	Contrastive	r2	393.006802721118	2	pre_focus	3	Contrastive pre_focus
2023206	block3	HumanSpeech	Pre	tsv1	Verb	Contrastive	r2	168.082869511409	1	pre_focus	1	Contrastive pre_focus
2023206	block3	HumanSpeech	Pre	fug1	Object	Contrastive	r2	159.818594104308	1	on_focus	1	Contrastive on_focus
2023206	block3	HumanSpeech	Pre	tshe1	Object	Contrastive	r2	198.557823129192	2	on_focus	1	Contrastive on_focus
2023206	block3	HumanSpeech	Pre	piu2	Subject	Narrow	r2	216.179138322047	1	pre_focus	2	Narrow pre_focus
2023206	block3	HumanSpeech	Pre	tse2	Subject	Narrow	r2	170.400604686279	2	pre_focus	2	Narrow pre_focus
2023206	block3	HumanSpeech	Pre	tsap1	Verb	Narrow	r2	96.1154302991076	1	on_focus	1	Narrow on_focus
2023206	block3	HumanSpeech	Pre	sy1	Object	Narrow	r2	135.521541950141	1	post_focus	1	Narrow post_focus
2023206	block3	HumanSpeech	Pre	pau1	Object	Narrow	r2	280.641061980361	2	post_focus	1	Narrow post_focus
2023206	block4	HumanSpeech	Post	piu35	Subject	Narrow	r1	149.376417233555	1	on_focus	35	Narrow on_focus
2023206	block4	HumanSpeech	Post	mui35	Subject	Narrow	r1	131.797052154184	2	on_focus	35	Narrow on_focus
2023206	block4	HumanSpeech	Post	tsan3	Verb	Narrow	r1	226.075322882764	1	post_focus	3	Narrow post_focus
2023206	block4	HumanSpeech	Post	jin3	Object	Narrow	r1	192.138390709829	1	post_focus	3	Narrow post_focus
2023206	block4	HumanSpeech	Post	jin3	Object	Narrow	r1	297.77544351073	2	post_focus	3	Narrow post_focus
2023206	block4	HumanSpeech	Post	pak3	Subject	Broad	r1	96.2547241118727	1	broad_focus	3	Broad focus
2023206	block4	HumanSpeech	Post	pak3	Subject	Broad	r1	107.673469387748	2	broad_focus	3	Broad focus
2023206	block4	HumanSpeech	Post	tsing2	Verb	Broad	r1	100.733182161747	1	broad_focus	2	Broad focus
2023206	block4	HumanSpeech	Post	kau2	Object	Broad	r1	140.377173091451	1	broad_focus	2	Broad focus
2023206	block4	HumanSpeech	Post	tsi2	Object	Broad	r1	101.708238851103	2	broad_focus	2	Broad focus
2023206	block4	HumanSpeech	Post	jan1	Subject	Contrastive	r1	119.631298406802	1	on_focus	1	Contrastive on_focus
2023206	block4	HumanSpeech	Post	jan1	Subject	Contrastive	r1	120.546579743007	2	on_focus	1	Contrastive on_focus
2023206	block4	HumanSpeech	Post	wei3	Verb	Contrastive	r1	96.5253793825269	1	post_focus	3	Contrastive post_focus
2023206	block4	HumanSpeech	Post	tsam3	Object	Contrastive	r1	94.4113909828275	1	post_focus	3	Contrastive post_focus
2023206	block4	HumanSpeech	Post	tsam3	Object	Contrastive	r1	276.413422511865	2	post_focus	3	Contrastive post_focus
2023206	block4	HumanSpeech	Post	jan1	Subject	Contrastive	r1	127.135028614617	1	pre_focus	1	Contrastive pre_focus
2023206	block4	HumanSpeech	Post	jan1	Subject	Contrastive	r1	106.729381407746	2	pre_focus	1	Contrastive pre_focus
2023206	block4	HumanSpeech	Post	wei3	Verb	Contrastive	r1	101.124212648017	1	pre_focus	3	Contrastive pre_focus
2023206	block4	HumanSpeech	Post	tsam3	Object	Contrastive	r1	91.8367346938709	1	on_focus	3	Contrastive on_focus
2023206	block4	HumanSpeech	Post	tsam3	Object	Contrastive	r1	322.552051123481	2	on_focus	3	Contrastive on_focus
2023206	block4	HumanSpeech	Post	piu35	Subject	Narrow	r1	107.649281934997	1	pre_focus	35	Narrow pre_focus
2023206	block4	HumanSpeech	Post	mui35	Subject	Narrow	r1	128.412698412703	2	pre_focus	35	Narrow pre_focus
2023206	block4	HumanSpeech	Post	tsan3	Verb	Narrow	r1	147.954000647886	1	on_focus	3	Narrow on_focus
2023206	block4	HumanSpeech	Post	jin3	Object	Narrow	r1	215.710506424784	1	post_focus	3	Narrow post_focus
2023206	block4	HumanSpeech	Post	jin3	Object	Narrow	r1	397.150690579252	2	post_focus	3	Narrow post_focus
2023206	block4	HumanSpeech	Post	piu35	Subject	Broad	r1	70.2806122449005	1	broad_focus	35	Broad focus
2023206	block4	HumanSpeech	Post	mui35	Subject	Broad	r1	144.691609977329	2	broad_focus	35	Broad focus
2023206	block4	HumanSpeech	Post	tsan3	Verb	Broad	r1	256.026706979085	1	broad_focus	3	Broad focus
2023206	block4	HumanSpeech	Post	jin3	Object	Broad	r1	189.178721296955	1	broad_focus	3	Broad focus
2023206	block4	HumanSpeech	Post	jin3	Object	Broad	r1	129.385309679421	2	broad_focus	3	Broad focus
2023206	block4	HumanSpeech	Post	pak3	Subject	Contrastive	r1	118.997457568895	1	on_focus	3	Contrastive on_focus
2023206	block4	HumanSpeech	Post	pak3	Subject	Contrastive	r1	102.343375445429	2	on_focus	3	Contrastive on_focus
2023206	block4	HumanSpeech	Post	tsing2	Verb	Contrastive	r1	162.153892668186	1	post_focus	2	Contrastive post_focus
2023206	block4	HumanSpeech	Post	kau2	Object	Contrastive	r1	185.878562103042	1	post_focus	2	Contrastive post_focus
2023206	block4	HumanSpeech	Post	tsi2	Object	Contrastive	r1	306.957132059168	2	post_focus	2	Contrastive post_focus
2023206	block4	HumanSpeech	Post	pak3	Subject	Narrow	r1	84.6206538170975	1	pre_focus	3	Narrow pre_focus
2023206	block4	HumanSpeech	Post	pak3	Subject	Narrow	r1	135.514977920991	2	pre_focus	3	Narrow pre_focus
2023206	block4	HumanSpeech	Post	tsing2	Verb	Narrow	r1	162.176870748283	1	on_focus	2	Narrow on_focus
2023206	block4	HumanSpeech	Post	kau2	Object	Narrow	r1	188.595238095218	1	post_focus	2	Narrow post_focus

2023206	block4	HumanSpeech	Post	tsi2	Object	Narrow	r1	181.886033101762	2	post_focus	2	Narrow post_focus
2023206	block4	HumanSpeech	Post	piu35	Subject	Contrastive	r1	68.7054479911353	1	pre_focus	35	Contrastive pre_focus
2023206	block4	HumanSpeech	Post	mui35	Subject	Contrastive	r1	143.22679646574	2	pre_focus	35	Contrastive pre_focus
2023206	block4	HumanSpeech	Post	tsan3	Verb	Contrastive	r1	222.050344358991	1	on_focus	3	Contrastive on_focus
2023206	block4	HumanSpeech	Post	jin3	Object	Contrastive	r1	214.995054529766	1	post_focus	3	Contrastive post_focus
2023206	block4	HumanSpeech	Post	jin3	Object	Contrastive	r1	219.017384731671	2	post_focus	3	Contrastive post_focus
2023206	block4	HumanSpeech	Post	piu35	Subject	Contrastive	r1	240.156840514004	1	pre_focus	35	Contrastive pre_focus
2023206	block4	HumanSpeech	Post	mui35	Subject	Contrastive	r1	175.831966974812	2	pre_focus	35	Contrastive pre_focus
2023206	block4	HumanSpeech	Post	tsan3	Verb	Contrastive	r1	188.791131267322	1	pre_focus	3	Contrastive pre_focus
2023206	block4	HumanSpeech	Post	jin3	Object	Contrastive	r1	230.676312853177	1	on_focus	3	Contrastive on_focus
2023206	block4	HumanSpeech	Post	jin3	Object	Contrastive	r1	390.833333333319	2	on_focus	3	Contrastive on_focus
2023206	block4	HumanSpeech	Post	pak3	Subject	Narrow	r1	111.985238539859	1	pre_focus	3	Narrow pre_focus
2023206	block4	HumanSpeech	Post	pak3	Subject	Narrow	r1	120.319727891143	2	pre_focus	3	Narrow pre_focus
2023206	block4	HumanSpeech	Post	tsing2	Verb	Narrow	r1	181.070294784547	1	pre_focus	2	Narrow pre_focus
2023206	block4	HumanSpeech	Post	kau2	Object	Narrow	r1	224.260977118149	1	on_focus	2	Narrow on_focus
2023206	block4	HumanSpeech	Post	tsi2	Object	Narrow	r1	452.029478458059	2	on_focus	2	Narrow on_focus
2023206	block4	HumanSpeech	Post	jan1	Subject	Narrow	r1	235.396825396833	1	on_focus	1	Narrow on_focus
2023206	block4	HumanSpeech	Post	jan1	Subject	Narrow	r1	187.812469717244	2	on_focus	1	Narrow on_focus
2023206	block4	HumanSpeech	Post	wei3	Verb	Narrow	r1	212.278911564596	1	post_focus	3	Narrow post_focus
2023206	block4	HumanSpeech	Post	tsam3	Object	Narrow	r1	250.363351689856	1	post_focus	3	Narrow post_focus
2023206	block4	HumanSpeech	Post	tsam3	Object	Narrow	r1	396.649049363333	2	post_focus	3	Narrow post_focus
2023206	block4	HumanSpeech	Post	piu35	Subject	Contrastive	r1	127.656420592956	1	on_focus	35	Contrastive on_focus
2023206	block4	HumanSpeech	Post	mui35	Subject	Contrastive	r1	186.235071806493	2	on_focus	35	Contrastive on_focus
2023206	block4	HumanSpeech	Post	tsan3	Verb	Contrastive	r1	211.654884175886	1	post_focus	3	Contrastive post_focus
2023206	block4	HumanSpeech	Post	jin3	Object	Contrastive	r1	185.377389050842	1	post_focus	3	Contrastive post_focus
2023206	block4	HumanSpeech	Post	jin3	Object	Contrastive	r1	239.482993197271	2	post_focus	3	Contrastive post_focus
2023206	block4	HumanSpeech	Post	pak3	Subject	Contrastive	r1	74.2861265718489	1	pre_focus	3	Contrastive pre_focus
2023206	block4	HumanSpeech	Post	pak3	Subject	Contrastive	r1	111.27347094893	2	pre_focus	3	Contrastive pre_focus
2023206	block4	HumanSpeech	Post	tsing2	Verb	Contrastive	r1	84.3152885567235	1	pre_focus	2	Contrastive pre_focus
2023206	block4	HumanSpeech	Post	kau2	Object	Contrastive	r1	108.341317747772	1	on_focus	2	Contrastive on_focus
2023206	block4	HumanSpeech	Post	tsi2	Object	Contrastive	r1	173.784688478577	2	on_focus	2	Contrastive on_focus
2023206	block4	HumanSpeech	Post	jan1	Subject	Broad	r1	74.5191903922091	1	broad_focus	1	Broad focus
2023206	block4	HumanSpeech	Post	jan1	Subject	Broad	r1	101.882626066299	2	broad_focus	1	Broad focus
2023206	block4	HumanSpeech	Post	wei3	Verb	Broad	r1	115.47097145737	1	broad_focus	3	Broad focus
2023206	block4	HumanSpeech	Post	tsam3	Object	Broad	r1	104.998425296031	1	broad_focus	3	Broad focus
2023206	block4	HumanSpeech	Post	tsam3	Object	Broad	r1	221.789763524441	2	broad_focus	3	Broad focus
2023206	block4	HumanSpeech	Post	pak3	Subject	Contrastive	r1	88.076994434175	1	pre_focus	3	Contrastive pre_focus
2023206	block4	HumanSpeech	Post	pak3	Subject	Contrastive	r1	142.579681976287	2	pre_focus	3	Contrastive pre_focus
2023206	block4	HumanSpeech	Post	tsing2	Verb	Contrastive	r1	118.621819098024	1	on_focus	2	Contrastive on_focus
2023206	block4	HumanSpeech	Post	kau2	Object	Contrastive	r1	135.452180872335	1	post_focus	2	Contrastive post_focus
2023206	block4	HumanSpeech	Post	tsi2	Object	Contrastive	r1	181.077097505664	2	post_focus	2	Contrastive post_focus
2023206	block4	HumanSpeech	Post	pak3	Subject	Narrow	r1	47.8672872657739	1	on_focus	3	Narrow on_focus
2023206	block4	HumanSpeech	Post	pak3	Subject	Narrow	r1	104.118345751004	2	on_focus	3	Narrow on_focus
2023206	block4	HumanSpeech	Post	tsing2	Verb	Narrow	r1	94.0610827664443	1	post_focus	2	Narrow post_focus
2023206	block4	HumanSpeech	Post	kau2	Object	Narrow	r1	185.193650793678	1	post_focus	2	Narrow post_focus
2023206	block4	HumanSpeech	Post	tsi2	Object	Narrow	r1	216.951473922904	2	post_focus	2	Narrow post_focus
2023206	block4	HumanSpeech	Post	jan1	Subject	Narrow	r1	148.673527530661	1	pre_focus	1	Narrow pre_focus
2023206	block4	HumanSpeech	Post	jan1	Subject	Narrow	r1	171.733056859125	2	pre_focus	1	Narrow pre_focus
2023206	block4	HumanSpeech	Post	wei3	Verb	Narrow	r1	86.2313036873275	1	on_focus	3	Narrow on_focus
2023206	block4	HumanSpeech	Post	tsam3	Object	Narrow	r1	188.982898715039	1	post_focus	3	Narrow post_focus
2023206	block4	HumanSpeech	Post	tsam3	Object	Narrow	r1	154.483865341007	2	post_focus	3	Narrow post_focus
2023206	block4	HumanSpeech	Post	piu35	Subject	Narrow	r1	128.591269841252	1	pre_focus	35	Narrow pre_focus
2023206	block4	HumanSpeech	Post	mui35	Subject	Narrow	r1	188.464528668618	2	pre_focus	35	Narrow pre_focus
2023206	block4	HumanSpeech	Post	tsan3	Verb	Narrow	r1	166.991301228592	1	pre_focus	3	Narrow pre_focus
2023206	block4	HumanSpeech	Post	jin3	Object	Narrow	r1	159.291173259419	1	on_focus	3	Narrow on_focus
2023206	block4	HumanSpeech	Post	jin3	Object	Narrow	r1	301.484970056407	2	on_focus	3	Narrow on_focus
2023206	block4	HumanSpeech	Post	jan1	Subject	Narrow	r1	156.150793650795	1	pre_focus	1	Narrow pre_focus
2023206	block4	HumanSpeech	Post	jan1	Subject	Narrow	r1	187.882842025687	2	pre_focus	1	Narrow pre_focus
2023206	block4	HumanSpeech	Post	wei3	Verb	Narrow	r1	150.174448567299	1	pre_focus	3	Narrow pre_focus
2023206	block4	HumanSpeech	Post	tsam3	Object	Narrow	r1	211.1124068675	1	on_focus	3	Narrow on_focus
2023206	block4	HumanSpeech	Post	tsam3	Object	Narrow	r1	440.383597883596	2	on_focus	3	Narrow on_focus
2023206	block4	HumanSpeech	Post	jan1	Subject	Contrastive	r1	138.950650435618	1	pre_focus	1	Contrastive pre_focus

**2023206**	block4	HumanSpeech	Post	jan1	Subject	Contrastive	r1	196.947089947088	2	pre_focus	1	Contrastive pre_focus
**2023206**	block4	HumanSpeech	Post	wei3	Verb	Contrastive	r1	393.908730158756	1	on_focus	3	Contrastive on_focus
**2023206**	block4	HumanSpeech	Post	tsam3	Object	Contrastive	r1	304.692197866785	1	post_focus	3	Contrastive post_focus
**2023206**	block4	HumanSpeech	Post	tsam3	Object	Contrastive	r1	455.25963718822	2	post_focus	3	Contrastive post_focus
**2023206**	block4	HumanSpeech	Post	jan1	Subject	Narrow	r2	153.648274124436	1	pre_focus	1	Narrow pre_focus
**2023206**	block4	HumanSpeech	Post	jan1	Subject	Narrow	r2	140.165381708243	2	pre_focus	1	Narrow pre_focus
**2023206**	block4	HumanSpeech	Post	wei3	Verb	Narrow	r2	118.997732426294	1	on_focus	3	Narrow on_focus
**2023206**	block4	HumanSpeech	Post	tsam3	Object	Narrow	r2	150.669690098255	1	post_focus	3	Narrow post_focus
**2023206**	block4	HumanSpeech	Post	tsam3	Object	Narrow	r2	298.321222428342	2	post_focus	3	Narrow post_focus
**2023206**	block4	HumanSpeech	Post	piu35	Subject	Broad	r2	144.49191398171	1	broad_focus	35	Broad focus
**2023206**	block4	HumanSpeech	Post	mui35	Subject	Broad	r2	258.695478191299	2	broad_focus	35	Broad focus
**2023206**	block4	HumanSpeech	Post	tsan3	Verb	Broad	r2	208.420634920657	1	broad_focus	3	Broad focus
**2023206**	block4	HumanSpeech	Post	jin3	Object	Broad	r2	269.180650037782	1	broad_focus	3	Broad focus
**2023206**	block4	HumanSpeech	Post	jin3	Object	Broad	r2	184.225785552314	2	broad_focus	3	Broad focus
**2023206**	block4	HumanSpeech	Post	piu35	Subject	Contrastive	r2	143.291761148902	1	pre_focus	35	Contrastive pre_focus
**2023206**	block4	HumanSpeech	Post	mui35	Subject	Contrastive	r2	254.833711262279	2	pre_focus	35	Contrastive pre_focus
**2023206**	block4	HumanSpeech	Post	tsan3	Verb	Contrastive	r2	324.919663103373	1	pre_focus	3	Contrastive pre_focus
**2023206**	block4	HumanSpeech	Post	jin3	Object	Contrastive	r2	311.918619299547	1	on_focus	3	Contrastive on_focus
**2023206**	block4	HumanSpeech	Post	jin3	Object	Contrastive	r2	510.13832199547	2	on_focus	3	Contrastive on_focus
**2023206**	block4	HumanSpeech	Post	pak3	Subject	Narrow	r2	111.710987425283	1	pre_focus	3	Narrow pre_focus
**2023206**	block4	HumanSpeech	Post	pak3	Subject	Narrow	r2	147.083814657947	2	pre_focus	3	Narrow pre_focus
**2023206**	block4	HumanSpeech	Post	tsing2	Verb	Narrow	r2	171.83609693879	1	pre_focus	2	Narrow pre_focus
**2023206**	block4	HumanSpeech	Post	kau2	Object	Narrow	r2	158.139281353556	1	on_focus	2	Narrow on_focus
**2023206**	block4	HumanSpeech	Post	tsi2	Object	Narrow	r2	547.991071428584	2	on_focus	2	Narrow on_focus
**2023206**	block4	HumanSpeech	Post	jan1	Subject	Broad	r2	162.440413202319	1	broad_focus	1	Broad focus
**2023206**	block4	HumanSpeech	Post	jan1	Subject	Broad	r2	163.243926141888	2	broad_focus	1	Broad focus
**2023206**	block4	HumanSpeech	Post	wei3	Verb	Broad	r2	186.597505668942	1	broad_focus	3	Broad focus
**2023206**	block4	HumanSpeech	Post	tsam3	Object	Broad	r2	214.622071050655	1	broad_focus	3	Broad focus
**2023206**	block4	HumanSpeech	Post	tsam3	Object	Broad	r2	289.018759018745	2	broad_focus	3	Broad focus
**2023206**	block4	HumanSpeech	Post	jan1	Subject	Contrastive	r2	150.22326879469	1	on_focus	1	Contrastive on_focus
**2023206**	block4	HumanSpeech	Post	jan1	Subject	Contrastive	r2	222.83257747543	2	on_focus	1	Contrastive on_focus
**2023206**	block4	HumanSpeech	Post	wei3	Verb	Contrastive	r2	216.325396825397	1	post_focus	3	Contrastive post_focus
**2023206**	block4	HumanSpeech	Post	tsam3	Object	Contrastive	r2	197.299067775276	1	post_focus	3	Contrastive post_focus
**2023206**	block4	HumanSpeech	Post	tsam3	Object	Contrastive	r2	288.752834467118	2	post_focus	3	Contrastive post_focus
**2023206**	block4	HumanSpeech	Post	jan1	Subject	Contrastive	r2	135.207431457445	1	pre_focus	1	Contrastive pre_focus
**2023206**	block4	HumanSpeech	Post	jan1	Subject	Contrastive	r2	200.006086128525	2	pre_focus	1	Contrastive pre_focus
**2023206**	block4	HumanSpeech	Post	wei3	Verb	Contrastive	r2	186.056934137298	1	pre_focus	3	Contrastive pre_focus
**2023206**	block4	HumanSpeech	Post	tsam3	Object	Contrastive	r2	194.033189033206	1	on_focus	3	Contrastive on_focus
**2023206**	block4	HumanSpeech	Post	tsam3	Object	Contrastive	r2	200.970332577469	2	on_focus	3	Contrastive on_focus
**2023206**	block4	HumanSpeech	Post	piu35	Subject	Narrow	r2	132.095993953129	1	pre_focus	35	Narrow pre_focus
**2023206**	block4	HumanSpeech	Post	mui35	Subject	Narrow	r2	255.840136054445	2	pre_focus	35	Narrow pre_focus
**2023206**	block4	HumanSpeech	Post	tsan3	Verb	Narrow	r2	233.552721088444	1	pre_focus	3	Narrow pre_focus
**2023206**	block4	HumanSpeech	Post	jin3	Object	Narrow	r2	322.675736961457	1	on_focus	3	Narrow on_focus
**2023206**	block4	HumanSpeech	Post	jin3	Object	Narrow	r2	175.610355253212	2	on_focus	3	Narrow on_focus
**2023206**	block4	HumanSpeech	Post	pak3	Subject	Contrastive	r2	103.466868228764	1	pre_focus	3	Contrastive pre_focus
**2023206**	block4	HumanSpeech	Post	pak3	Subject	Contrastive	r2	120.401486520564	2	pre_focus	3	Contrastive pre_focus
**2023206**	block4	HumanSpeech	Post	tsing2	Verb	Contrastive	r2	187.368742368733	1	on_focus	2	Contrastive on_focus
**2023206**	block4	HumanSpeech	Post	kau2	Object	Contrastive	r2	192.183589485126	1	post_focus	2	Contrastive post_focus
**2023206**	block4	HumanSpeech	Post	tsi2	Object	Contrastive	r2	224.267848553552	2	post_focus	2	Contrastive post_focus
**2023206**	block4	HumanSpeech	Post	piu35	Subject	Narrow	r2	115.792139077882	1	pre_focus	35	Narrow pre_focus
**2023206**	block4	HumanSpeech	Post	mui35	Subject	Narrow	r2	113.560468631874	2	pre_focus	35	Narrow pre_focus
**2023206**	block4	HumanSpeech	Post	tsan3	Verb	Narrow	r2	198.299924091884	1	on_focus	3	Narrow on_focus
**2023206**	block4	HumanSpeech	Post	jin3	Object	Narrow	r2	212.965662455417	1	post_focus	3	Narrow post_focus
**2023206**	block4	HumanSpeech	Post	jin3	Object	Narrow	r2	417.073871282923	2	post_focus	3	Narrow post_focus
**2023206**	block4	HumanSpeech	Post	piu35	Subject	Narrow	r2	135.011337868491	1	on_focus	35	Narrow on_focus
**2023206**	block4	HumanSpeech	Post	mui35	Subject	Narrow	r2	132.549508692364	2	on_focus	35	Narrow on_focus
**2023206**	block4	HumanSpeech	Post	tsan3	Verb	Narrow	r2	179.947041182061	1	post_focus	3	Narrow post_focus
**2023206**	block4	HumanSpeech	Post	jin3	Object	Narrow	r2	223.251700680237	1	post_focus	3	Narrow post_focus
**2023206**	block4	HumanSpeech	Post	jin3	Object	Narrow	r2	258.928193499628	2	post_focus	3	Narrow post_focus
**2023206**	block4	HumanSpeech	Post	pak3	Subject	Narrow	r2	100.294455946653	1	on_focus	3	Narrow on_focus
**2023206**	block4	HumanSpeech	Post	pak3	Subject	Narrow	r2	116.7281660139	2	on_focus	3	Narrow on_focus
**2023206**	block4	HumanSpeech	Post	tsing2	Verb	Narrow	r2	156.56956412198	1	post_focus	2	Narrow post_focus

2023206	block4	HumanSpeech	Post	kau2	Object	Narrow	r2	174.058895297264	1	post_focus	2	Narrow post_focus
2023206	block4	HumanSpeech	Post	tsi2	Object	Narrow	r2	147.087912087898	2	post_focus	2	Narrow post_focus
2023206	block4	HumanSpeech	Post	piu35	Subject	Contrastive	r2	122.755102040799	1	pre_focus	35	Contrastive pre_focus
2023206	block4	HumanSpeech	Post	mui35	Subject	Contrastive	r2	437.083900226753	2	pre_focus	35	Contrastive pre_focus
2023206	block4	HumanSpeech	Post	tsan3	Verb	Contrastive	r2	339.575963718801	1	on_focus	3	Contrastive on_focus
2023206	block4	HumanSpeech	Post	jin3	Object	Contrastive	r2	300.130385487535	1	post_focus	3	Contrastive post_focus
2023206	block4	HumanSpeech	Post	jin3	Object	Contrastive	r2	255.193823561171	2	post_focus	3	Contrastive post_focus
2023206	block4	HumanSpeech	Post	pak3	Subject	Contrastive	r2	57.7037345549911	1	on_focus	3	Contrastive on_focus
2023206	block4	HumanSpeech	Post	pak3	Subject	Contrastive	r2	83.1632653060979	2	on_focus	3	Contrastive on_focus
2023206	block4	HumanSpeech	Post	tsing2	Verb	Contrastive	r2	119.014636157488	1	post_focus	2	Contrastive post_focus
2023206	block4	HumanSpeech	Post	kau2	Object	Contrastive	r2	113.552968195791	1	post_focus	2	Contrastive post_focus
2023206	block4	HumanSpeech	Post	tsi2	Object	Contrastive	r2	239.89014865208	2	post_focus	2	Contrastive post_focus
2023206	block4	HumanSpeech	Post	jan1	Subject	Narrow	r2	98.8046647229908	1	pre_focus	1	Narrow pre_focus
2023206	block4	HumanSpeech	Post	jan1	Subject	Narrow	r2	80.0230349565823	2	pre_focus	1	Narrow pre_focus
2023206	block4	HumanSpeech	Post	wei3	Verb	Narrow	r2	101.07385811466	1	pre_focus	3	Narrow pre_focus
2023206	block4	HumanSpeech	Post	tsam3	Object	Narrow	r2	105.72533286819	1	on_focus	3	Narrow on_focus
2023206	block4	HumanSpeech	Post	tsam3	Object	Narrow	r2	243.472500111125	2	on_focus	3	Narrow on_focus
2023206	block4	HumanSpeech	Post	piu35	Subject	Contrastive	r2	89.6312493251799	1	on_focus	35	Contrastive on_focus
2023206	block4	HumanSpeech	Post	mui35	Subject	Contrastive	r2	112.169544740937	2	on_focus	35	Contrastive on_focus
2023206	block4	HumanSpeech	Post	tsan3	Verb	Contrastive	r2	193.287400430279	1	post_focus	3	Contrastive post_focus
2023206	block4	HumanSpeech	Post	jin3	Object	Contrastive	r2	141.174603174647	1	post_focus	3	Contrastive post_focus
2023206	block4	HumanSpeech	Post	jin3	Object	Contrastive	r2	166.360544217696	2	post_focus	3	Contrastive post_focus
2023206	block4	HumanSpeech	Post	jan1	Subject	Narrow	r2	48.7250051535852	1	on_focus	1	Narrow on_focus
2023206	block4	HumanSpeech	Post	jan1	Subject	Narrow	r2	117.399092970516	2	on_focus	1	Narrow on_focus
2023206	block4	HumanSpeech	Post	wei3	Verb	Narrow	r2	87.1080876794394	1	post_focus	3	Narrow post_focus
2023206	block4	HumanSpeech	Post	tsam3	Object	Narrow	r2	138.074352548017	1	post_focus	3	Narrow post_focus
2023206	block4	HumanSpeech	Post	tsam3	Object	Narrow	r2	293.16164561061	2	post_focus	3	Narrow post_focus
2023206	block4	HumanSpeech	Post	jan1	Subject	Contrastive	r2	124.605694129514	1	pre_focus	1	Contrastive pre_focus
2023206	block4	HumanSpeech	Post	jan1	Subject	Contrastive	r2	95.6653895274826	2	pre_focus	1	Contrastive pre_focus
2023206	block4	HumanSpeech	Post	wei3	Verb	Contrastive	r2	134.597883597905	1	on_focus	3	Contrastive on_focus
2023206	block4	HumanSpeech	Post	tsam3	Object	Contrastive	r2	145.10049474336	1	post_focus	3	Contrastive post_focus
2023206	block4	HumanSpeech	Post	tsam3	Object	Contrastive	r2	372.494762984616	2	post_focus	3	Contrastive post_focus
2023206	block4	HumanSpeech	Post	pak3	Subject	Broad	r2	54.7355184497746	1	broad_focus	3	Broad focus
2023206	block4	HumanSpeech	Post	pak3	Subject	Broad	r2	75.8816511100804	2	broad_focus	3	Broad focus
2023206	block4	HumanSpeech	Post	tsing2	Verb	Broad	r2	115.028090508474	1	broad_focus	2	Broad focus
2023206	block4	HumanSpeech	Post	kau2	Object	Broad	r2	151.99222546164	1	broad_focus	2	Broad focus
2023206	block4	HumanSpeech	Post	tsi2	Object	Broad	r2	182.854713313873	2	broad_focus	2	Broad focus
2023206	block4	HumanSpeech	Post	pak3	Subject	Contrastive	r2	110.89795918366	1	pre_focus	3	Contrastive pre_focus
2023206	block4	HumanSpeech	Post	pak3	Subject	Contrastive	r2	62.3550372530417	2	pre_focus	3	Contrastive pre_focus
2023206	block4	HumanSpeech	Post	tsing2	Verb	Contrastive	r2	133.757544043249	1	pre_focus	2	Contrastive pre_focus
2023206	block4	HumanSpeech	Post	kau2	Object	Contrastive	r2	140.835834333757	1	on_focus	2	Contrastive on_focus
2023206	block4	HumanSpeech	Post	tsi2	Object	Contrastive	r2	188.363916995399	2	on_focus	2	Contrastive on_focus
2023206	block4	HumanSpeech	Post	pak3	Subject	Narrow	r2	112.982399308976	1	pre_focus	3	Narrow pre_focus
2023206	block4	HumanSpeech	Post	pak3	Subject	Narrow	r2	90.5474265177304	2	pre_focus	3	Narrow pre_focus
2023206	block4	HumanSpeech	Post	tsing2	Verb	Narrow	r2	128.154195011348	1	on_focus	2	Narrow on_focus
2023206	block4	HumanSpeech	Post	kau2	Object	Narrow	r2	156.367346938794	1	post_focus	2	Narrow post_focus
2023206	block4	HumanSpeech	Post	tsi2	Object	Narrow	r2	240.498866213159	2	post_focus	2	Narrow post_focus
2023206	block4	HumanSpeech	Pre	piu35	Subject	Contrastive	r1	279.640211640213	1	pre_focus	35	Contrastive pre_focus
2023206	block4	HumanSpeech	Pre	mui35	Subject	Contrastive	r1	277.210884353735	2	pre_focus	35	Contrastive pre_focus
2023206	block4	HumanSpeech	Pre	tsan3	Verb	Contrastive	r1	423.608646357081	1	on_focus	3	Contrastive on_focus
2023206	block4	HumanSpeech	Pre	jin3	Object	Contrastive	r1	301.944444444445	1	post_focus	3	Contrastive post_focus
2023206	block4	HumanSpeech	Pre	jin3	Object	Contrastive	r1	291.291935577647	2	post_focus	3	Contrastive post_focus
2023206	block4	HumanSpeech	Pre	jan1	Subject	Contrastive	r1	202.528344671208	1	pre_focus	1	Contrastive pre_focus
2023206	block4	HumanSpeech	Pre	jan1	Subject	Contrastive	r1	235.21258503402	2	pre_focus	1	Contrastive pre_focus
2023206	block4	HumanSpeech	Pre	wei3	Verb	Contrastive	r1	485.419501133777	1	on_focus	3	Contrastive on_focus
2023206	block4	HumanSpeech	Pre	tsam3	Object	Contrastive	r1	252.743764172322	1	post_focus	3	Contrastive post_focus
2023206	block4	HumanSpeech	Pre	tsam3	Object	Contrastive	r1	283.819601914843	2	post_focus	3	Contrastive post_focus
2023206	block4	HumanSpeech	Pre	jan1	Subject	Narrow	r1	238.176870748305	1	pre_focus	1	Narrow pre_focus
2023206	block4	HumanSpeech	Pre	jan1	Subject	Narrow	r1	183.9387755102	2	pre_focus	1	Narrow pre_focus
2023206	block4	HumanSpeech	Pre	wei3	Verb	Narrow	r1	223.352985638684	1	pre_focus	3	Narrow pre_focus
2023206	block4	HumanSpeech	Pre	tsam3	Object	Narrow	r1	195.388938183982	1	on_focus	3	Narrow on_focus
2023206	block4	HumanSpeech	Pre	tsam3	Object	Narrow	r1	296.002591512803	2	on_focus	3	Narrow on_focus

**2023206**	block4	HumanSpeech	Pre	piu35	Subject	Narrow	r1	292.354024943279	1	pre_focus	35	Narrow pre_focus
**2023206**	block4	HumanSpeech	Pre	mui35	Subject	Narrow	r1	371.673469387758	2	pre_focus	35	Narrow pre_focus
**2023206**	block4	HumanSpeech	Pre	tsan3	Verb	Narrow	r1	606.479460001083	1	on_focus	3	Narrow on_focus
**2023206**	block4	HumanSpeech	Pre	jin3	Object	Narrow	r1	309.901738473144	1	post_focus	3	Narrow post_focus
**2023206**	block4	HumanSpeech	Pre	jin3	Object	Narrow	r1	356.872492586774	2	post_focus	3	Narrow post_focus
**2023206**	block4	HumanSpeech	Pre	jan1	Subject	Contrastive	r1	202.562358276651	1	on_focus	1	Contrastive on_focus
**2023206**	block4	HumanSpeech	Pre	jan1	Subject	Contrastive	r1	191.686250257675	2	on_focus	1	Contrastive on_focus
**2023206**	block4	HumanSpeech	Pre	wei3	Verb	Contrastive	r1	227.448507180668	1	post_focus	3	Contrastive post_focus
**2023206**	block4	HumanSpeech	Pre	tsam3	Object	Contrastive	r1	234.546485260779	1	post_focus	3	Contrastive post_focus
**2023206**	block4	HumanSpeech	Pre	tsam3	Object	Contrastive	r1	303.633786848081	2	post_focus	3	Contrastive post_focus
**2023206**	block4	HumanSpeech	Pre	pak3	Subject	Broad	r1	162.56338899197	1	broad_focus	3	Broad focus
**2023206**	block4	HumanSpeech	Pre	pak3	Subject	Broad	r1	170.068027210903	2	broad_focus	3	Broad focus
**2023206**	block4	HumanSpeech	Pre	tsing2	Verb	Broad	r1	215.11054421768	1	broad_focus	2	Broad focus
**2023206**	block4	HumanSpeech	Pre	kau2	Object	Broad	r1	251.291257243651	1	broad_focus	2	Broad focus
**2023206**	block4	HumanSpeech	Pre	tsi2	Object	Broad	r1	332.286470143629	2	broad_focus	2	Broad focus
**2023206**	block4	HumanSpeech	Pre	pak3	Subject	Narrow	r1	136.475085760793	1	on_focus	3	Narrow on_focus
**2023206**	block4	HumanSpeech	Pre	pak3	Subject	Narrow	r1	173.121315192759	2	on_focus	3	Narrow on_focus
**2023206**	block4	HumanSpeech	Pre	tsing2	Verb	Narrow	r1	217.727567217366	1	post_focus	2	Narrow post_focus
**2023206**	block4	HumanSpeech	Pre	kau2	Object	Narrow	r1	265.102040816345	1	post_focus	2	Narrow post_focus
**2023206**	block4	HumanSpeech	Pre	tsi2	Object	Narrow	r1	298.870748299294	2	post_focus	2	Narrow post_focus
**2023206**	block4	HumanSpeech	Pre	jan1	Subject	Narrow	r1	202.947845804999	1	pre_focus	1	Narrow pre_focus
**2023206**	block4	HumanSpeech	Pre	jan1	Subject	Narrow	r1	248.59788359791	2	pre_focus	1	Narrow pre_focus
**2023206**	block4	HumanSpeech	Pre	wei3	Verb	Narrow	r1	233.6079617032	1	on_focus	3	Narrow on_focus
**2023206**	block4	HumanSpeech	Pre	tsam3	Object	Narrow	r1	256.349206349199	1	post_focus	3	Narrow post_focus
**2023206**	block4	HumanSpeech	Pre	tsam3	Object	Narrow	r1	346.793650793671	2	post_focus	3	Narrow post_focus
**2023206**	block4	HumanSpeech	Pre	pak3	Subject	Contrastive	r1	177.981687624538	1	pre_focus	3	Contrastive pre_focus
**2023206**	block4	HumanSpeech	Pre	pak3	Subject	Contrastive	r1	185.215419501134	2	pre_focus	3	Contrastive pre_focus
**2023206**	block4	HumanSpeech	Pre	tsing2	Verb	Contrastive	r1	229.246031746015	1	on_focus	2	Contrastive on_focus
**2023206**	block4	HumanSpeech	Pre	kau2	Object	Contrastive	r1	259.997165532866	1	post_focus	2	Contrastive post_focus
**2023206**	block4	HumanSpeech	Pre	tsi2	Object	Contrastive	r1	254.226757369594	2	post_focus	2	Contrastive post_focus
**2023206**	block4	HumanSpeech	Pre	piu35	Subject	Narrow	r1	289.245869776494	1	pre_focus	35	Narrow pre_focus
**2023206**	block4	HumanSpeech	Pre	mui35	Subject	Narrow	r1	243.004535147378	2	pre_focus	35	Narrow pre_focus
**2023206**	block4	HumanSpeech	Pre	tsan3	Verb	Narrow	r1	237.277577697739	1	pre_focus	3	Narrow pre_focus
**2023206**	block4	HumanSpeech	Pre	jin3	Object	Narrow	r1	248.551020408144	1	on_focus	3	Narrow on_focus
**2023206**	block4	HumanSpeech	Pre	jin3	Object	Narrow	r1	301.609977324262	2	on_focus	3	Narrow on_focus
**2023206**	block4	HumanSpeech	Pre	piu35	Subject	Contrastive	r1	251.487150415727	1	on_focus	35	Contrastive on_focus
**2023206**	block4	HumanSpeech	Pre	mui35	Subject	Contrastive	r1	176.873015873014	2	on_focus	35	Contrastive on_focus
**2023206**	block4	HumanSpeech	Pre	tsan3	Verb	Contrastive	r1	266.435941043085	1	post_focus	3	Contrastive post_focus
**2023206**	block4	HumanSpeech	Pre	jin3	Object	Contrastive	r1	245.332577475466	1	post_focus	3	Contrastive post_focus
**2023206**	block4	HumanSpeech	Pre	jin3	Object	Contrastive	r1	337.670877874956	2	post_focus	3	Contrastive post_focus
**2023206**	block4	HumanSpeech	Pre	pak3	Subject	Contrastive	r1	143.16872427986	1	on_focus	3	Contrastive on_focus
**2023206**	block4	HumanSpeech	Pre	pak3	Subject	Contrastive	r1	159.71352985639	2	on_focus	3	Contrastive on_focus
**2023206**	block4	HumanSpeech	Pre	tsing2	Verb	Contrastive	r1	167.176398337119	1	post_focus	2	Contrastive post_focus
**2023206**	block4	HumanSpeech	Pre	kau2	Object	Contrastive	r1	216.613000755842	1	post_focus	2	Contrastive post_focus
**2023206**	block4	HumanSpeech	Pre	tsi2	Object	Contrastive	r1	353.650793650786	2	post_focus	2	Contrastive post_focus
**2023206**	block4	HumanSpeech	Pre	pak3	Subject	Narrow	r1	122.188457800689	1	pre_focus	3	Narrow pre_focus
**2023206**	block4	HumanSpeech	Pre	pak3	Subject	Narrow	r1	126.14253320379	2	pre_focus	3	Narrow pre_focus
**2023206**	block4	HumanSpeech	Pre	tsing2	Verb	Narrow	r1	186.254724111848	1	on_focus	2	Narrow on_focus
**2023206**	block4	HumanSpeech	Pre	kau2	Object	Narrow	r1	223.994708994724	1	post_focus	2	Narrow post_focus
**2023206**	block4	HumanSpeech	Pre	tsi2	Object	Narrow	r1	299.670366392206	2	post_focus	2	Narrow post_focus
**2023206**	block4	HumanSpeech	Pre	pak3	Subject	Contrastive	r1	140.329982739388	1	pre_focus	3	Contrastive pre_focus
**2023206**	block4	HumanSpeech	Pre	pak3	Subject	Contrastive	r1	156.344845630542	2	pre_focus	3	Contrastive pre_focus
**2023206**	block4	HumanSpeech	Pre	tsing2	Verb	Contrastive	r1	153.719360760192	1	pre_focus	2	Contrastive pre_focus
**2023206**	block4	HumanSpeech	Pre	kau2	Object	Contrastive	r1	193.971493359243	1	on_focus	2	Contrastive on_focus
**2023206**	block4	HumanSpeech	Pre	tsi2	Object	Contrastive	r1	344.017384731671	2	on_focus	2	Contrastive on_focus
**2023206**	block4	HumanSpeech	Pre	piu35	Subject	Narrow	r1	170.220458553786	1	on_focus	35	Narrow on_focus
**2023206**	block4	HumanSpeech	Pre	mui35	Subject	Narrow	r1	201.307153164294	2	on_focus	35	Narrow on_focus
**2023206**	block4	HumanSpeech	Pre	tsan3	Verb	Narrow	r1	256.896069538868	1	post_focus	3	Narrow post_focus
**2023206**	block4	HumanSpeech	Pre	jin3	Object	Narrow	r1	229.829931972802	1	post_focus	3	Narrow post_focus
**2023206**	block4	HumanSpeech	Pre	jin3	Object	Narrow	r1	340.173307418183	2	post_focus	3	Narrow post_focus
**2023206**	block4	HumanSpeech	Pre	piu35	Subject	Contrastive	r1	241.190476190525	1	pre_focus	35	Contrastive pre_focus
**2023206**	block4	HumanSpeech	Pre	mui35	Subject	Contrastive	r1	195.098706149111	2	pre_focus	35	Contrastive pre_focus

2023206	block4	HumanSpeech	Pre	tsan3	Verb	Contrastive	r1	262.138218923951	1	pre_focus	3	Contrastive pre_focus
2023206	block4	HumanSpeech	Pre	jin3	Object	Contrastive	r1	265.941043083899	1	on_focus	3	Contrastive on_focus
2023206	block4	HumanSpeech	Pre	jin3	Object	Contrastive	r1	360.201247165492	2	on_focus	3	Contrastive on_focus
2023206	block4	HumanSpeech	Pre	jan1	Subject	Contrastive	r1	204.842889536792	1	pre_focus	1	Contrastive pre_focus
2023206	block4	HumanSpeech	Pre	jan1	Subject	Contrastive	r1	216.622002336294	2	pre_focus	1	Contrastive pre_focus
2023206	block4	HumanSpeech	Pre	wei3	Verb	Contrastive	r1	185.403009688741	1	pre_focus	3	Contrastive pre_focus
2023206	block4	HumanSpeech	Pre	tsam3	Object	Contrastive	r1	205.64021164023	1	on_focus	3	Contrastive on_focus
2023206	block4	HumanSpeech	Pre	tsam3	Object	Contrastive	r1	288.219144800792	2	on_focus	3	Contrastive on_focus
2023206	block4	HumanSpeech	Pre	jan1	Subject	Narrow	r1	209.357520786057	1	on_focus	1	Narrow on_focus
2023206	block4	HumanSpeech	Pre	jan1	Subject	Narrow	r1	205.760686990857	2	on_focus	1	Narrow on_focus
2023206	block4	HumanSpeech	Pre	wei3	Verb	Narrow	r1	201.73201401775	1	post_focus	3	Narrow post_focus
2023206	block4	HumanSpeech	Pre	tsam3	Object	Narrow	r1	236.56259084828	1	post_focus	3	Narrow post_focus
2023206	block4	HumanSpeech	Pre	tsam3	Object	Narrow	r1	265.776482021408	2	post_focus	3	Narrow post_focus
2023206	block4	HumanSpeech	Pre	pak3	Subject	Narrow	r1	154.645433931137	1	pre_focus	3	Narrow pre_focus
2023206	block4	HumanSpeech	Pre	pak3	Subject	Narrow	r1	174.001395429968	2	pre_focus	3	Narrow pre_focus
2023206	block4	HumanSpeech	Pre	tsing2	Verb	Narrow	r1	205.827664399123	1	pre_focus	2	Narrow pre_focus
2023206	block4	HumanSpeech	Pre	kau2	Object	Narrow	r1	246.885865457273	1	on_focus	2	Narrow on_focus
2023206	block4	HumanSpeech	Pre	tsi2	Object	Narrow	r1	314.800777453854	2	on_focus	2	Narrow on_focus
2023206	block4	HumanSpeech	Pre	piu35	Subject	Broad	r1	316.43990929706	1	broad_focus	35	Broad focus
2023206	block4	HumanSpeech	Pre	mui35	Subject	Broad	r1	417.136054421746	2	broad_focus	35	Broad focus
2023206	block4	HumanSpeech	Pre	tsan3	Verb	Broad	r1	612.446613428006	1	broad_focus	3	Broad focus
2023206	block4	HumanSpeech	Pre	jin3	Object	Broad	r1	306.073318216249	1	broad_focus	3	Broad focus
2023206	block4	HumanSpeech	Pre	jin3	Object	Broad	r1	379.766763848409	2	broad_focus	3	Broad focus
2023206	block4	HumanSpeech	Pre	jan1	Subject	Broad	r1	209.826152683263	1	broad_focus	1	Broad focus
2023206	block4	HumanSpeech	Pre	jan1	Subject	Broad	r1	227.510470854952	2	broad_focus	1	Broad focus
2023206	block4	HumanSpeech	Pre	wei3	Verb	Broad	r1	181.498866213133	1	broad_focus	3	Broad focus
2023206	block4	HumanSpeech	Pre	tsam3	Object	Broad	r1	221.122660244191	1	broad_focus	3	Broad focus
2023206	block4	HumanSpeech	Pre	tsam3	Object	Broad	r1	297.26001511716	2	broad_focus	3	Broad focus
2023206	block4	HumanSpeech	Pre	jan1	Subject	Narrow	r2	216.560846560867	1	pre_focus	1	Narrow pre_focus
2023206	block4	HumanSpeech	Pre	jan1	Subject	Narrow	r2	212.029478458078	2	pre_focus	1	Narrow pre_focus
2023206	block4	HumanSpeech	Pre	wei3	Verb	Narrow	r2	182.717996289455	1	on_focus	3	Narrow on_focus
2023206	block4	HumanSpeech	Pre	tsam3	Object	Narrow	r2	187.448979591863	1	post_focus	3	Narrow post_focus
2023206	block4	HumanSpeech	Pre	tsam3	Object	Narrow	r2	252.165532879815	2	post_focus	3	Narrow post_focus
2023206	block4	HumanSpeech	Pre	jan1	Subject	Narrow	r2	172.231670445967	1	pre_focus	1	Narrow pre_focus
2023206	block4	HumanSpeech	Pre	jan1	Subject	Narrow	r2	232.278911564606	2	pre_focus	1	Narrow pre_focus
2023206	block4	HumanSpeech	Pre	wei3	Verb	Narrow	r2	205.988284202533	1	pre_focus	3	Narrow pre_focus
2023206	block4	HumanSpeech	Pre	tsam3	Object	Narrow	r2	234.097105508795	1	on_focus	3	Narrow on_focus
2023206	block4	HumanSpeech	Pre	tsam3	Object	Narrow	r2	250.572126286443	2	on_focus	3	Narrow on_focus
2023206	block4	HumanSpeech	Pre	pak3	Subject	Narrow	r2	108.935424916297	1	on_focus	3	Narrow on_focus
2023206	block4	HumanSpeech	Pre	pak3	Subject	Narrow	r2	134.886255577442	2	on_focus	3	Narrow on_focus
2023206	block4	HumanSpeech	Pre	tsing2	Verb	Narrow	r2	176.948223733916	1	post_focus	2	Narrow post_focus
2023206	block4	HumanSpeech	Pre	kau2	Object	Narrow	r2	216.693594104299	1	post_focus	2	Narrow post_focus
2023206	block4	HumanSpeech	Pre	tsi2	Object	Narrow	r2	300.828924162261	2	post_focus	2	Narrow post_focus
2023206	block4	HumanSpeech	Pre	piu35	Subject	Contrastive	r2	180.09322247417	1	pre_focus	35	Contrastive pre_focus
2023206	block4	HumanSpeech	Pre	mui35	Subject	Contrastive	r2	262.282690854136	2	pre_focus	35	Contrastive pre_focus
2023206	block4	HumanSpeech	Pre	tsan3	Verb	Contrastive	r2	209.843048330413	1	on_focus	3	Contrastive on_focus
2023206	block4	HumanSpeech	Pre	jin3	Object	Contrastive	r2	305.229024943287	1	post_focus	3	Contrastive post_focus
2023206	block4	HumanSpeech	Pre	jin3	Object	Contrastive	r2	474.727891156476	2	post_focus	3	Contrastive post_focus
2023206	block4	HumanSpeech	Pre	pak3	Subject	Narrow	r2	152.398395255545	1	pre_focus	3	Narrow pre_focus
2023206	block4	HumanSpeech	Pre	pak3	Subject	Narrow	r2	157.274008702586	2	pre_focus	3	Narrow pre_focus
2023206	block4	HumanSpeech	Pre	tsing2	Verb	Narrow	r2	201.687452758904	1	on_focus	2	Narrow on_focus
2023206	block4	HumanSpeech	Pre	kau2	Object	Narrow	r2	223.162358276682	1	post_focus	2	Narrow post_focus
2023206	block4	HumanSpeech	Pre	tsi2	Object	Narrow	r2	316.953892668209	2	post_focus	2	Narrow post_focus
2023206	block4	HumanSpeech	Pre	piu35	Subject	Broad	r2	198.78198898607	1	broad_focus	35	Broad focus
2023206	block4	HumanSpeech	Pre	mui35	Subject	Broad	r2	273.340891912312	2	broad_focus	35	Broad focus
2023206	block4	HumanSpeech	Pre	tsan3	Verb	Broad	r2	218.712715855531	1	broad_focus	3	Broad focus
2023206	block4	HumanSpeech	Pre	jin3	Object	Broad	r2	290.694444444455	1	broad_focus	3	Broad focus
2023206	block4	HumanSpeech	Pre	jin3	Object	Broad	r2	377.902494331067	2	broad_focus	3	Broad focus
2023206	block4	HumanSpeech	Pre	jan1	Subject	Contrastive	r2	211.405895691655	1	pre_focus	1	Contrastive pre_focus
2023206	block4	HumanSpeech	Pre	jan1	Subject	Contrastive	r2	192.907353417581	2	pre_focus	1	Contrastive pre_focus
2023206	block4	HumanSpeech	Pre	wei3	Verb	Contrastive	r2	236.410430839044	1	pre_focus	3	Contrastive pre_focus
2023206	block4	HumanSpeech	Pre	tsam3	Object	Contrastive	r2	240.545729402868	1	on_focus	3	Contrastive on_focus

2023206	block4	HumanSpeech	Pre	tsam3	Object	Contrastive	r2	291.802721088402	2	on_focus	3	Contrastive on_focus
2023206	block4	HumanSpeech	Pre	pak3	Subject	Contrastive	r2	133.60303717451	1	pre_focus	3	Contrastive pre_focus
2023206	block4	HumanSpeech	Pre	pak3	Subject	Contrastive	r2	185.772072269799	2	pre_focus	3	Contrastive pre_focus
2023206	block4	HumanSpeech	Pre	tsing2	Verb	Contrastive	r2	203.119533527683	1	pre_focus	2	Contrastive pre_focus
2023206	block4	HumanSpeech	Pre	kau2	Object	Contrastive	r2	239.732664995813	1	on_focus	2	Contrastive on_focus
2023206	block4	HumanSpeech	Pre	tsi2	Object	Contrastive	r2	323.613378684797	2	on_focus	2	Contrastive on_focus
2023206	block4	HumanSpeech	Pre	piu35	Subject	Narrow	r2	256.585240156653	1	pre_focus	35	Narrow pre_focus
2023206	block4	HumanSpeech	Pre	mui35	Subject	Narrow	r2	304.319727891198	2	pre_focus	35	Narrow pre_focus
2023206	block4	HumanSpeech	Pre	tsan3	Verb	Narrow	r2	196.177437641722	1	on_focus	3	Narrow on_focus
2023206	block4	HumanSpeech	Pre	jin3	Object	Narrow	r2	278.743386243377	1	post_focus	3	Narrow post_focus
2023206	block4	HumanSpeech	Pre	jin3	Object	Narrow	r2	369.278155706752	2	post_focus	3	Narrow post_focus
2023206	block4	HumanSpeech	Pre	jan1	Subject	Narrow	r2	169.544973544987	1	on_focus	1	Narrow on_focus
2023206	block4	HumanSpeech	Pre	jan1	Subject	Narrow	r2	212.278371666173	2	on_focus	1	Narrow on_focus
2023206	block4	HumanSpeech	Pre	wei3	Verb	Narrow	r2	173.648993989161	1	post_focus	3	Narrow post_focus
2023206	block4	HumanSpeech	Pre	tsam3	Object	Narrow	r2	154.535147392323	1	post_focus	3	Narrow post_focus
2023206	block4	HumanSpeech	Pre	tsam3	Object	Narrow	r2	213.423280423285	2	post_focus	3	Narrow post_focus
2023206	block4	HumanSpeech	Pre	jan1	Subject	Contrastive	r2	213.699384515735	1	pre_focus	1	Contrastive pre_focus
2023206	block4	HumanSpeech	Pre	jan1	Subject	Contrastive	r2	196.077097505679	2	pre_focus	1	Contrastive pre_focus
2023206	block4	HumanSpeech	Pre	wei3	Verb	Contrastive	r2	213.421516754863	1	on_focus	3	Contrastive on_focus
2023206	block4	HumanSpeech	Pre	tsam3	Object	Contrastive	r2	252.564877802968	1	post_focus	3	Contrastive post_focus
2023206	block4	HumanSpeech	Pre	tsam3	Object	Contrastive	r2	336.314162028486	2	post_focus	3	Contrastive post_focus
2023206	block4	HumanSpeech	Pre	piu35	Subject	Narrow	r2	207.256883705895	1	pre_focus	35	Narrow pre_focus
2023206	block4	HumanSpeech	Pre	mui35	Subject	Narrow	r2	276.700680272086	2	pre_focus	35	Narrow pre_focus
2023206	block4	HumanSpeech	Pre	tsan3	Verb	Narrow	r2	244.466089466073	1	pre_focus	3	Narrow pre_focus
2023206	block4	HumanSpeech	Pre	jin3	Object	Narrow	r2	195.725623582746	1	on_focus	3	Narrow on_focus
2023206	block4	HumanSpeech	Pre	jin3	Object	Narrow	r2	412.62131519278	2	on_focus	3	Narrow on_focus
2023206	block4	HumanSpeech	Pre	piu35	Subject	Contrastive	r2	175.695796267235	1	on_focus	35	Contrastive on_focus
2023206	block4	HumanSpeech	Pre	mui35	Subject	Contrastive	r2	219.467120181378	2	on_focus	35	Contrastive on_focus
2023206	block4	HumanSpeech	Pre	tsan3	Verb	Contrastive	r2	208.624338624361	1	post_focus	3	Contrastive post_focus
2023206	block4	HumanSpeech	Pre	jin3	Object	Contrastive	r2	218.667800453488	1	post_focus	3	Contrastive post_focus
2023206	block4	HumanSpeech	Pre	jin3	Object	Contrastive	r2	325.467372134028	2	post_focus	3	Contrastive post_focus
2023206	block4	HumanSpeech	Pre	piu35	Subject	Contrastive	r2	196.91893424033	1	pre_focus	35	Contrastive pre_focus
2023206	block4	HumanSpeech	Pre	mui35	Subject	Contrastive	r2	247.100340136058	2	pre_focus	35	Contrastive pre_focus
2023206	block4	HumanSpeech	Pre	tsan3	Verb	Contrastive	r2	198.80952380953	1	pre_focus	3	Contrastive pre_focus
2023206	block4	HumanSpeech	Pre	jin3	Object	Contrastive	r2	258.920634920685	1	on_focus	3	Contrastive on_focus
2023206	block4	HumanSpeech	Pre	jin3	Object	Contrastive	r2	391.235827664389	2	on_focus	3	Contrastive on_focus
2023206	block4	HumanSpeech	Pre	pak3	Subject	Narrow	r2	158.853702400506	1	pre_focus	3	Narrow pre_focus
2023206	block4	HumanSpeech	Pre	pak3	Subject	Narrow	r2	164.206842157171	2	pre_focus	3	Narrow pre_focus
2023206	block4	HumanSpeech	Pre	tsing2	Verb	Narrow	r2	189.604794298646	1	pre_focus	2	Narrow pre_focus
2023206	block4	HumanSpeech	Pre	kau2	Object	Narrow	r2	253.994465169569	1	on_focus	2	Narrow on_focus
2023206	block4	HumanSpeech	Pre	tsi2	Object	Narrow	r2	358.622448979645	2	on_focus	2	Narrow on_focus
2023206	block4	HumanSpeech	Pre	jan1	Subject	Broad	r2	146.553287981817	1	broad_focus	1	Broad focus
2023206	block4	HumanSpeech	Pre	jan1	Subject	Broad	r2	227.165532879837	2	broad_focus	1	Broad focus
2023206	block4	HumanSpeech	Pre	wei3	Verb	Broad	r2	196.287226001516	1	broad_focus	3	Broad focus
2023206	block4	HumanSpeech	Pre	tsam3	Object	Broad	r2	156.825396825354	1	broad_focus	3	Broad focus
2023206	block4	HumanSpeech	Pre	tsam3	Object	Broad	r2	237.607709750534	2	broad_focus	3	Broad focus
2023206	block4	HumanSpeech	Pre	pak3	Subject	Contrastive	r2	157.877659000121	1	on_focus	3	Contrastive on_focus
2023206	block4	HumanSpeech	Pre	pak3	Subject	Contrastive	r2	170.511682934034	2	on_focus	3	Contrastive on_focus
2023206	block4	HumanSpeech	Pre	tsing2	Verb	Contrastive	r2	192.363315696639	1	post_focus	2	Contrastive post_focus
2023206	block4	HumanSpeech	Pre	kau2	Object	Contrastive	r2	222.644085411901	1	post_focus	2	Contrastive post_focus
2023206	block4	HumanSpeech	Pre	tsi2	Object	Contrastive	r2	270.935889507314	2	post_focus	2	Contrastive post_focus
2023206	block4	HumanSpeech	Pre	pak3	Subject	Broad	r2	179.858546593266	1	broad_focus	3	Broad focus
2023206	block4	HumanSpeech	Pre	pak3	Subject	Broad	r2	201.08251996453	2	broad_focus	3	Broad focus
2023206	block4	HumanSpeech	Pre	tsing2	Verb	Broad	r2	213.295540438423	1	broad_focus	2	Broad focus
2023206	block4	HumanSpeech	Pre	kau2	Object	Broad	r2	222.111935683415	1	broad_focus	2	Broad focus
2023206	block4	HumanSpeech	Pre	tsi2	Object	Broad	r2	302.287729906766	2	broad_focus	2	Broad focus
2023206	block4	HumanSpeech	Pre	pak3	Subject	Contrastive	r2	182.40101168675	1	pre_focus	3	Contrastive pre_focus
2023206	block4	HumanSpeech	Pre	pak3	Subject	Contrastive	r2	165.932797361336	2	pre_focus	3	Contrastive pre_focus
2023206	block4	HumanSpeech	Pre	tsing2	Verb	Contrastive	r2	229.634353741517	1	on_focus	2	Contrastive on_focus
2023206	block4	HumanSpeech	Pre	kau2	Object	Contrastive	r2	239.749811035551	1	post_focus	2	Contrastive post_focus
2023206	block4	HumanSpeech	Pre	tsi2	Object	Contrastive	r2	267.782312925192	2	post_focus	2	Contrastive post_focus
2023206	block4	HumanSpeech	Pre	jan1	Subject	Contrastive	r2	158.809523809566	1	on_focus	1	Contrastive on_focus

2023206	block4	HumanSpeech	Pre	jan1	Subject	Contrastive	r2	205.136054421757	2	on_focus	1	Contrastive on_focus
2023206	block4	HumanSpeech	Pre	wei3	Verb	Contrastive	r2	211.159108087656	1	post_focus	3	Contrastive post_focus
2023206	block4	HumanSpeech	Pre	tsam3	Object	Contrastive	r2	183.32350718066	1	post_focus	3	Contrastive post_focus
2023206	block4	HumanSpeech	Pre	tsam3	Object	Contrastive	r2	281.906823335362	2	post_focus	3	Contrastive post_focus
2023206	block4	HumanSpeech	Pre	piu35	Subject	Narrow	r2	178.492063492058	1	on_focus	35	Narrow on_focus
2023206	block4	HumanSpeech	Pre	mui35	Subject	Narrow	r2	230.195578231303	2	on_focus	35	Narrow on_focus
2023206	block4	HumanSpeech	Pre	tsan3	Verb	Narrow	r2	194.720462897806	1	post_focus	3	Narrow post_focus
2023206	block4	HumanSpeech	Pre	jin3	Object	Narrow	r2	248.896447467871	1	post_focus	3	Narrow post_focus
2023206	block4	HumanSpeech	Pre	jin3	Object	Narrow	r2	286.967120181373	2	post_focus	3	Narrow post_focus
2023206	block5	HumanSpeech	Post	siu2	Subject	Narrow	r1	241.981211532234	1	pre_focus	2	Narrow pre_focus
2023206	block5	HumanSpeech	Post	gwong2	Subject	Narrow	r1	209.210758377424	2	pre_focus	2	Narrow pre_focus
2023206	block5	HumanSpeech	Post	cyun4	Verb	Narrow	r1	45.6462585034103	1	pre_focus	4	Narrow pre_focus
2023206	block5	HumanSpeech	Post	laam4	Object	Narrow	r1	424.350907029478	1	on_focus	4	Narrow on_focus
2023206	block5	HumanSpeech	Post	kau4	Object	Narrow	r1	154.756235827662	2	on_focus	4	Narrow on_focus
2023206	block5	HumanSpeech	Post	siu2	Subject	Narrow	r1	207.886621315183	1	pre_focus	2	Narrow pre_focus
2023206	block5	HumanSpeech	Post	gwong2	Subject	Narrow	r1	204.674981103537	2	pre_focus	2	Narrow pre_focus
2023206	block5	HumanSpeech	Post	cyun4	Verb	Narrow	r1	82.3096857790802	1	on_focus	4	Narrow on_focus
2023206	block5	HumanSpeech	Post	laam4	Object	Narrow	r1	288.403628117919	1	post_focus	4	Narrow post_focus
2023206	block5	HumanSpeech	Post	kau4	Object	Narrow	r1	169.891156462583	2	post_focus	4	Narrow post_focus
2023206	block5	HumanSpeech	Post	ceoi3	Subject	Contrastive	r1	124.124464600655	1	pre_focus	3	Contrastive pre_focus
2023206	block5	HumanSpeech	Post	ceoi3	Subject	Contrastive	r1	127.731166540698	2	pre_focus	3	Contrastive pre_focus
2023206	block5	HumanSpeech	Post	caa4	Verb	Contrastive	r1	191.30578746649	1	on_focus	4	Contrastive on_focus
2023206	block5	HumanSpeech	Post	ngau4	Object	Contrastive	r1	170.048443619876	1	post_focus	4	Contrastive post_focus
2023206	block5	HumanSpeech	Post	jau4	Object	Contrastive	r1	151.423937996256	2	post_focus	4	Contrastive post_focus
2023206	block5	HumanSpeech	Post	ceoi3	Subject	Narrow	r1	155.822761537038	1	pre_focus	3	Narrow pre_focus
2023206	block5	HumanSpeech	Post	ceoi3	Subject	Narrow	r1	116.704390847246	2	pre_focus	3	Narrow pre_focus
2023206	block5	HumanSpeech	Post	caa4	Verb	Narrow	r1	142.039801217834	1	pre_focus	4	Narrow pre_focus
2023206	block5	HumanSpeech	Post	ngau4	Object	Narrow	r1	214.094871903214	1	on_focus	4	Narrow on_focus
2023206	block5	HumanSpeech	Post	jau4	Object	Narrow	r1	271.815347351051	2	on_focus	4	Narrow on_focus
2023206	block5	HumanSpeech	Post	wai5	Subject	Broad	r1	254.541626174273	1	broad_focus	5	Broad focus
2023206	block5	HumanSpeech	Post	wai5	Subject	Broad	r1	310.653473510598	2	broad_focus	5	Broad focus
2023206	block5	HumanSpeech	Post	waat3	Verb	Broad	r1	190.747219522734	1	broad_focus	3	Broad focus
2023206	block5	HumanSpeech	Post	bui3	Object	Broad	r1	206.221250404923	1	broad_focus	3	Broad focus
2023206	block5	HumanSpeech	Post	hok3	Object	Broad	r1	209.56905839256	2	broad_focus	3	Broad focus
2023206	block5	HumanSpeech	Post	siu2	Subject	Narrow	r1	193.441043083908	1	on_focus	2	Narrow on_focus
2023206	block5	HumanSpeech	Post	gwong2	Subject	Narrow	r1	205.088435374137	2	on_focus	2	Narrow on_focus
2023206	block5	HumanSpeech	Post	cyun4	Verb	Narrow	r1	139.23280423279	1	post_focus	4	Narrow post_focus
2023206	block5	HumanSpeech	Post	laam4	Object	Narrow	r1	242.483680340797	1	post_focus	4	Narrow post_focus
2023206	block5	HumanSpeech	Post	kau4	Object	Narrow	r1	138.915451895059	2	post_focus	4	Narrow post_focus
2023206	block5	HumanSpeech	Post	siu2	Subject	Contrastive	r1	171.080876795145	1	on_focus	2	Contrastive on_focus
2023206	block5	HumanSpeech	Post	gwong2	Subject	Contrastive	r1	169.491394848563	2	on_focus	2	Contrastive on_focus
2023206	block5	HumanSpeech	Post	cyun4	Verb	Contrastive	r1	103.266880826396	1	post_focus	4	Contrastive post_focus
2023206	block5	HumanSpeech	Post	laam4	Object	Contrastive	r1	283.103552532111	1	post_focus	4	Contrastive post_focus
2023206	block5	HumanSpeech	Post	kau4	Object	Contrastive	r1	220.236556634063	2	post_focus	4	Contrastive post_focus
2023206	block5	HumanSpeech	Post	siu2	Subject	Broad	r1	171.554705215414	1	broad_focus	2	Broad focus
2023206	block5	HumanSpeech	Post	gwong2	Subject	Broad	r1	173.93055923668	2	broad_focus	2	Broad focus
2023206	block5	HumanSpeech	Post	cyun4	Verb	Broad	r1	201.249685059224	1	broad_focus	4	Broad focus
2023206	block5	HumanSpeech	Post	laam4	Object	Broad	r1	194.937641723357	1	broad_focus	4	Broad focus
2023206	block5	HumanSpeech	Post	kau4	Object	Broad	r1	179.377497030572	2	broad_focus	4	Broad focus
2023206	block5	HumanSpeech	Post	ceoi3	Subject	Contrastive	r1	181.98934600295	1	pre_focus	3	Contrastive pre_focus
2023206	block5	HumanSpeech	Post	ceoi3	Subject	Contrastive	r1	131.822383965215	2	pre_focus	3	Contrastive pre_focus
2023206	block5	HumanSpeech	Post	caa4	Verb	Contrastive	r1	166.705071135539	1	pre_focus	4	Contrastive pre_focus
2023206	block5	HumanSpeech	Post	ngau4	Object	Contrastive	r1	237.161589273398	1	on_focus	4	Contrastive on_focus
2023206	block5	HumanSpeech	Post	jau4	Object	Contrastive	r1	289.678760393031	2	on_focus	4	Contrastive on_focus
2023206	block5	HumanSpeech	Post	wai5	Subject	Narrow	r1	241.715797430089	1	on_focus	5	Narrow on_focus
2023206	block5	HumanSpeech	Post	wai5	Subject	Narrow	r1	260.146132527069	2	on_focus	5	Narrow on_focus
2023206	block5	HumanSpeech	Post	waat3	Verb	Narrow	r1	169.158163265308	1	post_focus	3	Narrow post_focus
2023206	block5	HumanSpeech	Post	bui3	Object	Narrow	r1	208.127092106679	1	post_focus	3	Narrow post_focus
2023206	block5	HumanSpeech	Post	hok3	Object	Narrow	r1	94.9574829932089	2	post_focus	3	Narrow post_focus
2023206	block5	HumanSpeech	Post	wai5	Subject	Narrow	r1	209.002699492487	1	pre_focus	5	Narrow pre_focus
2023206	block5	HumanSpeech	Post	wai5	Subject	Narrow	r1	217.062461348178	2	pre_focus	5	Narrow pre_focus
2023206	block5	HumanSpeech	Post	waat3	Verb	Narrow	r1	153.655090106213	1	on_focus	3	Narrow on_focus

2023206	block5	HumanSpeech	Post	bui3	Object	Narrow	r1	187.968803683077	1	post_focus	3	Narrow post_focus
2023206	block5	HumanSpeech	Post	hok3	Object	Narrow	r1	141.641628873799	2	post_focus	3	Narrow post_focus
2023206	block5	HumanSpeech	Post	siu2	Subject	Contrastive	r1	203.295540438404	1	pre_focus	2	Contrastive pre_focus
2023206	block5	HumanSpeech	Post	gwong2	Subject	Contrastive	r1	185.797430083142	2	pre_focus	2	Contrastive pre_focus
2023206	block5	HumanSpeech	Post	cyun4	Verb	Contrastive	r1	120.195983155185	1	on_focus	4	Contrastive on_focus
2023206	block5	HumanSpeech	Post	laam4	Object	Contrastive	r1	261.798941798958	1	post_focus	4	Contrastive post_focus
2023206	block5	HumanSpeech	Post	kau4	Object	Contrastive	r1	141.116780045365	2	post_focus	4	Contrastive post_focus
2023206	block5	HumanSpeech	Post	ceoi3	Subject	Narrow	r1	117.162698412727	1	pre_focus	3	Narrow pre_focus
2023206	block5	HumanSpeech	Post	ceoi3	Subject	Narrow	r1	99.1548428895328	2	pre_focus	3	Narrow pre_focus
2023206	block5	HumanSpeech	Post	caa4	Verb	Narrow	r1	112.964852607689	1	on_focus	4	Narrow on_focus
2023206	block5	HumanSpeech	Post	ngau4	Object	Narrow	r1	147.321833495312	1	post_focus	4	Narrow post_focus
2023206	block5	HumanSpeech	Post	jau4	Object	Narrow	r1	284.72789115645	2	post_focus	4	Narrow post_focus
2023206	block5	HumanSpeech	Post	wai5	Subject	Contrastive	r1	228.759385235577	1	on_focus	5	Contrastive on_focus
2023206	block5	HumanSpeech	Post	wai5	Subject	Contrastive	r1	338.247489471968	2	on_focus	5	Contrastive on_focus
2023206	block5	HumanSpeech	Post	waat3	Verb	Contrastive	r1	167.99766371193	1	post_focus	3	Contrastive post_focus
2023206	block5	HumanSpeech	Post	bui3	Object	Contrastive	r1	164.453352769669	1	post_focus	3	Contrastive post_focus
2023206	block5	HumanSpeech	Post	hok3	Object	Contrastive	r1	77.719027004747	2	post_focus	3	Contrastive post_focus
2023206	block5	HumanSpeech	Post	ceoi3	Subject	Narrow	r1	141.479797979798	1	on_focus	3	Narrow on_focus
2023206	block5	HumanSpeech	Post	ceoi3	Subject	Narrow	r1	109.214380196562	2	on_focus	3	Narrow on_focus
2023206	block5	HumanSpeech	Post	caa4	Verb	Narrow	r1	95.0095904550494	1	post_focus	4	Narrow post_focus
2023206	block5	HumanSpeech	Post	ngau4	Object	Narrow	r1	151.668606836665	1	post_focus	4	Narrow post_focus
2023206	block5	HumanSpeech	Post	jau4	Object	Narrow	r1	166.693703122291	2	post_focus	4	Narrow post_focus
2023206	block5	HumanSpeech	Post	ceoi3	Subject	Broad	r1	153.944822373433	1	broad_focus	3	Broad focus
2023206	block5	HumanSpeech	Post	ceoi3	Subject	Broad	r1	104.467120181397	2	broad_focus	3	Broad focus
2023206	block5	HumanSpeech	Post	caa4	Verb	Broad	r1	103.332301196332	1	broad_focus	4	Broad focus
2023206	block5	HumanSpeech	Post	ngau4	Object	Broad	r1	141.698412698418	1	broad_focus	4	Broad focus
2023206	block5	HumanSpeech	Post	jau4	Object	Broad	r1	323.009826152685	2	broad_focus	4	Broad focus
2023206	block5	HumanSpeech	Post	ceoi3	Subject	Contrastive	r1	112.067271352998	1	on_focus	3	Contrastive on_focus
2023206	block5	HumanSpeech	Post	ceoi3	Subject	Contrastive	r1	109.246301695265	2	on_focus	3	Contrastive on_focus
2023206	block5	HumanSpeech	Post	caa4	Verb	Contrastive	r1	111.181376925543	1	post_focus	4	Contrastive post_focus
2023206	block5	HumanSpeech	Post	ngau4	Object	Contrastive	r1	164.226490596235	1	post_focus	4	Contrastive post_focus
2023206	block5	HumanSpeech	Post	jau4	Object	Contrastive	r1	269.160997732428	2	post_focus	4	Contrastive post_focus
2023206	block5	HumanSpeech	Post	wai5	Subject	Contrastive	r1	215.273377416224	1	pre_focus	5	Contrastive pre_focus
2023206	block5	HumanSpeech	Post	wai5	Subject	Contrastive	r1	263.614080552856	2	pre_focus	5	Contrastive pre_focus
2023206	block5	HumanSpeech	Post	waat3	Verb	Contrastive	r1	241.724847833865	1	on_focus	3	Contrastive on_focus
2023206	block5	HumanSpeech	Post	bui3	Object	Contrastive	r1	148.255479969777	1	post_focus	3	Contrastive post_focus
2023206	block5	HumanSpeech	Post	hok3	Object	Contrastive	r1	236.341269841262	2	post_focus	3	Contrastive post_focus
2023206	block5	HumanSpeech	Post	wai5	Subject	Narrow	r1	247.976190476209	1	pre_focus	5	Narrow pre_focus
2023206	block5	HumanSpeech	Post	wai5	Subject	Narrow	r1	271.826341647767	2	pre_focus	5	Narrow pre_focus
2023206	block5	HumanSpeech	Post	waat3	Verb	Narrow	r1	210.274725274701	1	pre_focus	3	Narrow pre_focus
2023206	block5	HumanSpeech	Post	bui3	Object	Narrow	r1	203.169312169308	1	on_focus	3	Narrow on_focus
2023206	block5	HumanSpeech	Post	hok3	Object	Narrow	r1	291.596170319991	2	on_focus	3	Narrow on_focus
2023206	block5	HumanSpeech	Post	wai5	Subject	Contrastive	r1	283.819241982513	1	pre_focus	5	Contrastive pre_focus
2023206	block5	HumanSpeech	Post	wai5	Subject	Contrastive	r1	230.009308986723	2	pre_focus	5	Contrastive pre_focus
2023206	block5	HumanSpeech	Post	waat3	Verb	Contrastive	r1	188.974678760388	1	pre_focus	3	Contrastive pre_focus
2023206	block5	HumanSpeech	Post	bui3	Object	Contrastive	r1	246.068376068365	1	on_focus	3	Contrastive on_focus
2023206	block5	HumanSpeech	Post	hok3	Object	Contrastive	r1	166.102680388377	2	on_focus	3	Contrastive on_focus
2023206	block5	HumanSpeech	Post	siu2	Subject	Contrastive	r1	160.414347557207	1	pre_focus	2	Contrastive pre_focus
2023206	block5	HumanSpeech	Post	gwong2	Subject	Contrastive	r1	307.416099773235	2	pre_focus	2	Contrastive pre_focus
2023206	block5	HumanSpeech	Post	cyun4	Verb	Contrastive	r1	112.558235415378	1	pre_focus	4	Contrastive pre_focus
2023206	block5	HumanSpeech	Post	laam4	Object	Contrastive	r1	313.144368858644	1	on_focus	4	Contrastive on_focus
2023206	block5	HumanSpeech	Post	kau4	Object	Contrastive	r1	179.449460592309	2	on_focus	4	Contrastive on_focus
2023206	block5	HumanSpeech	Post	ceoi3	Subject	Contrastive	r2	184.455782312909	1	pre_focus	3	Contrastive pre_focus
2023206	block5	HumanSpeech	Post	ceoi3	Subject	Contrastive	r2	140.660430839006	2	pre_focus	3	Contrastive pre_focus
2023206	block5	HumanSpeech	Post	caa4	Verb	Contrastive	r2	162.701493640952	1	on_focus	4	Contrastive on_focus
2023206	block5	HumanSpeech	Post	ngau4	Object	Contrastive	r2	212.826908541189	1	post_focus	4	Contrastive post_focus
2023206	block5	HumanSpeech	Post	jau4	Object	Contrastive	r2	404.362244897925	2	post_focus	4	Contrastive post_focus
2023206	block5	HumanSpeech	Post	wai5	Subject	Narrow	r2	271.910030005245	1	on_focus	5	Narrow on_focus
2023206	block5	HumanSpeech	Post	wai5	Subject	Narrow	r2	226.591080876801	2	on_focus	5	Narrow on_focus
2023206	block5	HumanSpeech	Post	waat3	Verb	Narrow	r2	156.338246409689	1	post_focus	3	Narrow post_focus
2023206	block5	HumanSpeech	Post	bui3	Object	Narrow	r2	178.184321347601	1	post_focus	3	Narrow post_focus
2023206	block5	HumanSpeech	Post	hok3	Object	Narrow	r2	190.23866213152	2	post_focus	3	Narrow post_focus

2023206	block5	HumanSpeech	Post	ceoi3	Subject	Narrow	r2	160.38832199547	1	on_focus	3	Narrow on_focus
2023206	block5	HumanSpeech	Post	ceoi3	Subject	Narrow	r2	143.198628657814	2	on_focus	3	Narrow on_focus
2023206	block5	HumanSpeech	Post	caa4	Verb	Narrow	r2	205.098143364182	1	post_focus	4	Narrow post_focus
2023206	block5	HumanSpeech	Post	ngau4	Object	Narrow	r2	187.854084269361	1	post_focus	4	Narrow post_focus
2023206	block5	HumanSpeech	Post	jau4	Object	Narrow	r2	325.346938775482	2	post_focus	4	Narrow post_focus
2023206	block5	HumanSpeech	Post	siu2	Subject	Contrastive	r2	166.118669690093	1	pre_focus	2	Contrastive pre_focus
2023206	block5	HumanSpeech	Post	gwong2	Subject	Contrastive	r2	244.152604764849	2	pre_focus	2	Contrastive pre_focus
2023206	block5	HumanSpeech	Post	cyun4	Verb	Contrastive	r2	123.748196248187	1	pre_focus	4	Contrastive pre_focus
2023206	block5	HumanSpeech	Post	laam4	Object	Contrastive	r2	323.140589569164	1	on_focus	4	Contrastive on_focus
2023206	block5	HumanSpeech	Post	kau4	Object	Contrastive	r2	336.182287729912	2	on_focus	4	Contrastive on_focus
2023206	block5	HumanSpeech	Post	ceoi3	Subject	Narrow	r2	127.515387107223	1	pre_focus	3	Narrow pre_focus
2023206	block5	HumanSpeech	Post	ceoi3	Subject	Narrow	r2	151.982271696539	2	pre_focus	3	Narrow pre_focus
2023206	block5	HumanSpeech	Post	caa4	Verb	Narrow	r2	146.271169128312	1	pre_focus	4	Narrow pre_focus
2023206	block5	HumanSpeech	Post	ngau4	Object	Narrow	r2	135.698088759312	1	on_focus	4	Narrow on_focus
2023206	block5	HumanSpeech	Post	jau4	Object	Narrow	r2	234.165721844306	2	on_focus	4	Narrow on_focus
2023206	block5	HumanSpeech	Post	wai5	Subject	Broad	r2	186.313448456275	1	broad_focus	5	Broad focus
2023206	block5	HumanSpeech	Post	wai5	Subject	Broad	r2	217.119047619065	2	broad_focus	5	Broad focus
2023206	block5	HumanSpeech	Post	waat3	Verb	Broad	r2	190.975056689354	1	broad_focus	3	Broad focus
2023206	block5	HumanSpeech	Post	bui3	Object	Broad	r2	203.134135705568	1	broad_focus	3	Broad focus
2023206	block5	HumanSpeech	Post	hok3	Object	Broad	r2	154.516723356011	2	broad_focus	3	Broad focus
2023206	block5	HumanSpeech	Post	siu2	Subject	Narrow	r2	128.305504019778	1	pre_focus	2	Narrow pre_focus
2023206	block5	HumanSpeech	Post	gwong2	Subject	Narrow	r2	162.216016231042	2	pre_focus	2	Narrow pre_focus
2023206	block5	HumanSpeech	Post	cyun4	Verb	Narrow	r2	93.0926216640557	1	on_focus	4	Narrow on_focus
2023206	block5	HumanSpeech	Post	laam4	Object	Narrow	r2	345.200302343159	1	post_focus	4	Narrow post_focus
2023206	block5	HumanSpeech	Post	kau4	Object	Narrow	r2	208.480038480047	2	post_focus	4	Narrow post_focus
2023206	block5	HumanSpeech	Post	ceoi3	Subject	Narrow	r2	184.275248560965	1	pre_focus	3	Narrow pre_focus
2023206	block5	HumanSpeech	Post	ceoi3	Subject	Narrow	r2	131.206538170801	2	pre_focus	3	Narrow pre_focus
2023206	block5	HumanSpeech	Post	caa4	Verb	Narrow	r2	150.009673372892	1	on_focus	4	Narrow on_focus
2023206	block5	HumanSpeech	Post	ngau4	Object	Narrow	r2	193.845791328584	1	post_focus	4	Narrow post_focus
2023206	block5	HumanSpeech	Post	jau4	Object	Narrow	r2	274.695389266839	2	post_focus	4	Narrow post_focus
2023206	block5	HumanSpeech	Post	siu2	Subject	Broad	r2	179.821170892637	1	broad_focus	2	Broad focus
2023206	block5	HumanSpeech	Post	gwong2	Subject	Broad	r2	166.614008566398	2	broad_focus	2	Broad focus
2023206	block5	HumanSpeech	Post	cyun4	Verb	Broad	r2	96.7687074829655	1	broad_focus	4	Broad focus
2023206	block5	HumanSpeech	Post	laam4	Object	Broad	r2	287.586653709127	1	broad_focus	4	Broad focus
2023206	block5	HumanSpeech	Post	kau4	Object	Broad	r2	168.737244898011	2	broad_focus	4	Broad focus
2023206	block5	HumanSpeech	Post	wai5	Subject	Narrow	r2	217.281431090953	1	pre_focus	5	Narrow pre_focus
2023206	block5	HumanSpeech	Post	wai5	Subject	Narrow	r2	204.06658817376	2	pre_focus	5	Narrow pre_focus
2023206	block5	HumanSpeech	Post	waat3	Verb	Narrow	r2	177.89569160999	1	on_focus	3	Narrow on_focus
2023206	block5	HumanSpeech	Post	bui3	Object	Narrow	r2	232.229780801163	1	post_focus	3	Narrow post_focus
2023206	block5	HumanSpeech	Post	hok3	Object	Narrow	r2	178.516870407293	2	post_focus	3	Narrow post_focus
2023206	block5	HumanSpeech	Post	ceoi3	Subject	Broad	r2	153.354978354969	1	broad_focus	3	Broad focus
2023206	block5	HumanSpeech	Post	ceoi3	Subject	Broad	r2	145.243926141916	2	broad_focus	3	Broad focus
2023206	block5	HumanSpeech	Post	caa4	Verb	Broad	r2	106.192757024814	1	broad_focus	4	Broad focus
2023206	block5	HumanSpeech	Post	ngau4	Object	Broad	r2	213.997050568423	1	broad_focus	4	Broad focus
2023206	block5	HumanSpeech	Post	jau4	Object	Broad	r2	194.494796209085	2	broad_focus	4	Broad focus
2023206	block5	HumanSpeech	Post	siu2	Subject	Contrastive	r2	186.269841269905	1	pre_focus	2	Contrastive pre_focus
2023206	block5	HumanSpeech	Post	gwong2	Subject	Contrastive	r2	192.96292679752	2	pre_focus	2	Contrastive pre_focus
2023206	block5	HumanSpeech	Post	cyun4	Verb	Contrastive	r2	117.299697656847	1	on_focus	4	Contrastive on_focus
2023206	block5	HumanSpeech	Post	laam4	Object	Contrastive	r2	294.424345495827	1	post_focus	4	Contrastive post_focus
2023206	block5	HumanSpeech	Post	kau4	Object	Contrastive	r2	143.854875283409	2	post_focus	4	Contrastive post_focus
2023206	block5	HumanSpeech	Post	wai5	Subject	Narrow	r2	243.799930228477	1	pre_focus	5	Narrow pre_focus
2023206	block5	HumanSpeech	Post	wai5	Subject	Narrow	r2	252.824263038519	2	pre_focus	5	Narrow pre_focus
2023206	block5	HumanSpeech	Post	waat3	Verb	Narrow	r2	217.704821058874	1	pre_focus	3	Narrow pre_focus
2023206	block5	HumanSpeech	Post	bui3	Object	Narrow	r2	219.938631537275	1	on_focus	3	Narrow on_focus
2023206	block5	HumanSpeech	Post	hok3	Object	Narrow	r2	151.487005058414	2	on_focus	3	Narrow on_focus
2023206	block5	HumanSpeech	Post	wai5	Subject	Contrastive	r2	187.074829931987	1	pre_focus	5	Contrastive pre_focus
2023206	block5	HumanSpeech	Post	wai5	Subject	Contrastive	r2	270.425799949578	2	pre_focus	5	Contrastive pre_focus
2023206	block5	HumanSpeech	Post	waat3	Verb	Contrastive	r2	190.248173343434	1	pre_focus	3	Contrastive pre_focus
2023206	block5	HumanSpeech	Post	bui3	Object	Contrastive	r2	254.464852607725	1	on_focus	3	Contrastive on_focus
2023206	block5	HumanSpeech	Post	hok3	Object	Contrastive	r2	116.944220670746	2	on_focus	3	Contrastive on_focus
2023206	block5	HumanSpeech	Post	wai5	Subject	Contrastive	r2	289.611935683411	1	pre_focus	5	Contrastive pre_focus
2023206	block5	HumanSpeech	Post	wai5	Subject	Contrastive	r2	242.32037259344	2	pre_focus	5	Contrastive pre_focus

2023206	block5	HumanSpeech	Post	waat3	Verb	Contrastive	r2	201.2789115646	1	on_focus	3	Contrastive on_focus
2023206	block5	HumanSpeech	Post	bui3	Object	Contrastive	r2	205.622826908552	1	post_focus	3	Contrastive post_focus
2023206	block5	HumanSpeech	Post	hok3	Object	Contrastive	r2	156.954702515918	2	post_focus	3	Contrastive post_focus
2023206	block5	HumanSpeech	Post	siu2	Subject	Narrow	r2	174.115646258485	1	pre_focus	2	Narrow pre_focus
2023206	block5	HumanSpeech	Post	gwong2	Subject	Narrow	r2	368.878306878287	2	pre_focus	2	Narrow pre_focus
2023206	block5	HumanSpeech	Post	cyun4	Verb	Narrow	r2	142.202214219083	1	pre_focus	4	Narrow pre_focus
2023206	block5	HumanSpeech	Post	laam4	Object	Narrow	r2	188.488284202549	1	on_focus	4	Narrow on_focus
2023206	block5	HumanSpeech	Post	kau4	Object	Narrow	r2	117.275132275154	2	on_focus	4	Narrow on_focus
2023206	block5	HumanSpeech	Post	ceoi3	Subject	Contrastive	r2	130.673731030925	1	pre_focus	3	Contrastive pre_focus
2023206	block5	HumanSpeech	Post	ceoi3	Subject	Contrastive	r2	133.884147598451	2	pre_focus	3	Contrastive pre_focus
2023206	block5	HumanSpeech	Post	caa4	Verb	Contrastive	r2	135.228507173508	1	pre_focus	4	Contrastive pre_focus
2023206	block5	HumanSpeech	Post	ngau4	Object	Contrastive	r2	169.800318137163	1	on_focus	4	Contrastive on_focus
2023206	block5	HumanSpeech	Post	jau4	Object	Contrastive	r2	256.286848072591	2	on_focus	4	Contrastive on_focus
2023206	block5	HumanSpeech	Post	ceoi3	Subject	Contrastive	r2	135.648148148164	1	on_focus	3	Contrastive on_focus
2023206	block5	HumanSpeech	Post	ceoi3	Subject	Contrastive	r2	162.764736988379	2	on_focus	3	Contrastive on_focus
2023206	block5	HumanSpeech	Post	caa4	Verb	Contrastive	r2	154.632186207778	1	post_focus	4	Contrastive post_focus
2023206	block5	HumanSpeech	Post	ngau4	Object	Contrastive	r2	237.705432705411	1	post_focus	4	Contrastive post_focus
2023206	block5	HumanSpeech	Post	jau4	Object	Contrastive	r2	332.277021919822	2	post_focus	4	Contrastive post_focus
2023206	block5	HumanSpeech	Post	siu2	Subject	Contrastive	r2	210.578231292573	1	on_focus	2	Contrastive on_focus
2023206	block5	HumanSpeech	Post	gwong2	Subject	Contrastive	r2	213.049559567423	2	on_focus	2	Contrastive on_focus
2023206	block5	HumanSpeech	Post	cyun4	Verb	Contrastive	r2	96.9629629628912	1	post_focus	4	Contrastive post_focus
2023206	block5	HumanSpeech	Post	laam4	Object	Contrastive	r2	241.432350718071	1	post_focus	4	Contrastive post_focus
2023206	block5	HumanSpeech	Post	kau4	Object	Contrastive	r2	110.374149659833	2	post_focus	4	Contrastive post_focus
2023206	block5	HumanSpeech	Post	siu2	Subject	Narrow	r2	138.794750223269	1	on_focus	2	Narrow on_focus
2023206	block5	HumanSpeech	Post	gwong2	Subject	Narrow	r2	191.545297484083	2	on_focus	2	Narrow on_focus
2023206	block5	HumanSpeech	Post	cyun4	Verb	Narrow	r2	77.6732855303521	1	post_focus	4	Narrow post_focus
2023206	block5	HumanSpeech	Post	laam4	Object	Narrow	r2	269.217363135795	1	post_focus	4	Narrow post_focus
2023206	block5	HumanSpeech	Post	kau4	Object	Narrow	r2	177.084833933577	2	post_focus	4	Narrow post_focus
2023206	block5	HumanSpeech	Post	wai5	Subject	Contrastive	r2	196.86394557823	1	on_focus	5	Contrastive on_focus
2023206	block5	HumanSpeech	Post	wai5	Subject	Contrastive	r2	185.232156354573	2	on_focus	5	Contrastive on_focus
2023206	block5	HumanSpeech	Post	waat3	Verb	Contrastive	r2	187.233020192195	1	post_focus	3	Contrastive post_focus
2023206	block5	HumanSpeech	Post	bui3	Object	Contrastive	r2	137.017006802694	1	post_focus	3	Contrastive post_focus
2023206	block5	HumanSpeech	Post	hok3	Object	Contrastive	r2	446.060142238707	2	post_focus	3	Contrastive post_focus
2023206	block5	HumanSpeech	Pre	ceoi3	Subject	Contrastive	r1	211.718820861677	1	pre_focus	3	Contrastive pre_focus
2023206	block5	HumanSpeech	Pre	ceoi3	Subject	Contrastive	r1	330.336356764974	2	pre_focus	3	Contrastive pre_focus
2023206	block5	HumanSpeech	Pre	caa4	Verb	Contrastive	r1	162.681921253352	1	on_focus	4	Contrastive on_focus
2023206	block5	HumanSpeech	Pre	ngau4	Object	Contrastive	r1	239.447895100113	1	post_focus	4	Contrastive post_focus
2023206	block5	HumanSpeech	Pre	jau4	Object	Contrastive	r1	263.643235071754	2	post_focus	4	Contrastive post_focus
2023206	block5	HumanSpeech	Pre	wai5	Subject	Narrow	r1	363.658352229777	1	on_focus	5	Narrow on_focus
2023206	block5	HumanSpeech	Pre	wai5	Subject	Narrow	r1	287.913832199592	2	on_focus	5	Narrow on_focus
2023206	block5	HumanSpeech	Pre	waat3	Verb	Narrow	r1	170.660619803471	1	post_focus	3	Narrow post_focus
2023206	block5	HumanSpeech	Pre	bui3	Object	Narrow	r1	156.604938271585	1	post_focus	3	Narrow post_focus
2023206	block5	HumanSpeech	Pre	hok3	Object	Narrow	r1	231.700680272127	2	post_focus	3	Narrow post_focus
2023206	block5	HumanSpeech	Pre	ceoi3	Subject	Narrow	r1	248.119854002198	1	on_focus	3	Narrow on_focus
2023206	block5	HumanSpeech	Pre	ceoi3	Subject	Narrow	r1	252.36655508769	2	on_focus	3	Narrow on_focus
2023206	block5	HumanSpeech	Pre	caa4	Verb	Narrow	r1	136.473723992538	1	post_focus	4	Narrow post_focus
2023206	block5	HumanSpeech	Pre	ngau4	Object	Narrow	r1	224.944930353104	1	post_focus	4	Narrow post_focus
2023206	block5	HumanSpeech	Pre	jau4	Object	Narrow	r1	294.478458049866	2	post_focus	4	Narrow post_focus
2023206	block5	HumanSpeech	Pre	siu2	Subject	Contrastive	r1	271.864564007387	1	pre_focus	2	Contrastive pre_focus
2023206	block5	HumanSpeech	Pre	gwong2	Subject	Contrastive	r1	292.880358492596	2	pre_focus	2	Contrastive pre_focus
2023206	block5	HumanSpeech	Pre	cyun4	Verb	Contrastive	r1	217.149470899471	1	on_focus	4	Contrastive on_focus
2023206	block5	HumanSpeech	Pre	laam4	Object	Contrastive	r1	349.959183673491	1	post_focus	4	Contrastive post_focus
2023206	block5	HumanSpeech	Pre	kau4	Object	Contrastive	r1	228.962585034026	2	post_focus	4	Contrastive post_focus
2023206	block5	HumanSpeech	Pre	siu2	Subject	Broad	r1	351.90022675738	1	broad_focus	2	Broad focus
2023206	block5	HumanSpeech	Pre	gwong2	Subject	Broad	r1	348.518916338435	2	broad_focus	2	Broad focus
2023206	block5	HumanSpeech	Pre	cyun4	Verb	Broad	r1	201.569160997792	1	broad_focus	4	Broad focus
2023206	block5	HumanSpeech	Pre	laam4	Object	Broad	r1	310.437641723354	1	broad_focus	4	Broad focus
2023206	block5	HumanSpeech	Pre	kau4	Object	Broad	r1	170.213151927442	2	broad_focus	4	Broad focus
2023206	block5	HumanSpeech	Pre	wai5	Subject	Contrastive	r1	373.854875283484	1	pre_focus	5	Contrastive pre_focus
2023206	block5	HumanSpeech	Pre	wai5	Subject	Contrastive	r1	384.235827664384	2	pre_focus	5	Contrastive pre_focus
2023206	block5	HumanSpeech	Pre	waat3	Verb	Contrastive	r1	268.590325018863	1	pre_focus	3	Contrastive pre_focus
2023206	block5	HumanSpeech	Pre	bui3	Object	Contrastive	r1	260.151055293932	1	on_focus	3	Contrastive on_focus

2023206	block5	HumanSpeech	Pre	hok3	Object	Contrastive	r1	111.530612244906	2	on_focus	3	Contrastive on_focus
2023206	block5	HumanSpeech	Pre	ceoi3	Subject	Contrastive	r1	304.135487528299	1	pre_focus	3	Contrastive pre_focus
2023206	block5	HumanSpeech	Pre	ceoi3	Subject	Contrastive	r1	355.444066515474	2	pre_focus	3	Contrastive pre_focus
2023206	block5	HumanSpeech	Pre	caa4	Verb	Contrastive	r1	231.349206349194	1	pre_focus	4	Contrastive pre_focus
2023206	block5	HumanSpeech	Pre	ngau4	Object	Contrastive	r1	156.570730856458	1	on_focus	4	Contrastive on_focus
2023206	block5	HumanSpeech	Pre	jau4	Object	Contrastive	r1	222.176870748342	2	on_focus	4	Contrastive on_focus
2023206	block5	HumanSpeech	Pre	siu2	Subject	Narrow	r1	194.305482876871	1	on_focus	2	Narrow on_focus
2023206	block5	HumanSpeech	Pre	gwong2	Subject	Narrow	r1	251.078841793117	2	on_focus	2	Narrow on_focus
2023206	block5	HumanSpeech	Pre	cyun4	Verb	Narrow	r1	179.187452758867	1	post_focus	4	Narrow post_focus
2023206	block5	HumanSpeech	Pre	laam4	Object	Narrow	r1	292.509448223711	1	post_focus	4	Narrow post_focus
2023206	block5	HumanSpeech	Pre	kau4	Object	Narrow	r1	320.226757369653	2	post_focus	4	Narrow post_focus
2023206	block5	HumanSpeech	Pre	siu2	Subject	Narrow	r1	262.029478458032	1	pre_focus	2	Narrow pre_focus
2023206	block5	HumanSpeech	Pre	gwong2	Subject	Narrow	r1	376.589191232028	2	pre_focus	2	Narrow pre_focus
2023206	block5	HumanSpeech	Pre	cyun4	Verb	Narrow	r1	288.378684807242	1	pre_focus	4	Narrow pre_focus
2023206	block5	HumanSpeech	Pre	laam4	Object	Narrow	r1	286.3340891912	1	on_focus	4	Narrow on_focus
2023206	block5	HumanSpeech	Pre	kau4	Object	Narrow	r1	282.215419501199	2	on_focus	4	Narrow on_focus
2023206	block5	HumanSpeech	Pre	wai5	Subject	Contrastive	r1	514.285714285734	1	on_focus	5	Contrastive on_focus
2023206	block5	HumanSpeech	Pre	wai5	Subject	Contrastive	r1	583.749055177634	2	on_focus	5	Contrastive on_focus
2023206	block5	HumanSpeech	Pre	waat3	Verb	Contrastive	r1	298.541194255506	1	post_focus	3	Contrastive post_focus
2023206	block5	HumanSpeech	Pre	bui3	Object	Contrastive	r1	210.200680272123	1	post_focus	3	Contrastive post_focus
2023206	block5	HumanSpeech	Pre	hok3	Object	Contrastive	r1	162.751322751319	2	post_focus	3	Contrastive post_focus
2023206	block5	HumanSpeech	Pre	ceoi3	Subject	Narrow	r1	386.326530612223	1	pre_focus	3	Narrow pre_focus
2023206	block5	HumanSpeech	Pre	ceoi3	Subject	Narrow	r1	311.111111111131	2	pre_focus	3	Narrow pre_focus
2023206	block5	HumanSpeech	Pre	caa4	Verb	Narrow	r1	219.017384731671	1	pre_focus	4	Narrow pre_focus
2023206	block5	HumanSpeech	Pre	ngau4	Object	Narrow	r1	281.723356009081	1	on_focus	4	Narrow on_focus
2023206	block5	HumanSpeech	Pre	jau4	Object	Narrow	r1	292.106575963771	2	on_focus	4	Narrow on_focus
2023206	block5	HumanSpeech	Pre	ceoi3	Subject	Broad	r1	321.284013605464	1	broad_focus	3	Broad focus
2023206	block5	HumanSpeech	Pre	ceoi3	Subject	Broad	r1	225.999999999999	2	broad_focus	3	Broad focus
2023206	block5	HumanSpeech	Pre	caa4	Verb	Broad	r1	229.886621315188	1	broad_focus	4	Broad focus
2023206	block5	HumanSpeech	Pre	ngau4	Object	Broad	r1	253.052154194961	1	broad_focus	4	Broad focus
2023206	block5	HumanSpeech	Pre	jau4	Object	Broad	r1	351.961451247178	2	broad_focus	4	Broad focus
2023206	block5	HumanSpeech	Pre	wai5	Subject	Contrastive	r1	319.247921390797	1	pre_focus	5	Contrastive pre_focus
2023206	block5	HumanSpeech	Pre	wai5	Subject	Contrastive	r1	337.335276967906	2	pre_focus	5	Contrastive pre_focus
2023206	block5	HumanSpeech	Pre	waat3	Verb	Contrastive	r1	260.743764172332	1	on_focus	3	Contrastive on_focus
2023206	block5	HumanSpeech	Pre	bui3	Object	Contrastive	r1	201.238095238125	1	post_focus	3	Contrastive post_focus
2023206	block5	HumanSpeech	Pre	hok3	Object	Contrastive	r1	138.283446712023	2	post_focus	3	Contrastive post_focus
2023206	block5	HumanSpeech	Pre	siu2	Subject	Narrow	r1	249.013605442201	1	pre_focus	2	Narrow pre_focus
2023206	block5	HumanSpeech	Pre	gwong2	Subject	Narrow	r1	254.507936507878	2	pre_focus	2	Narrow pre_focus
2023206	block5	HumanSpeech	Pre	cyun4	Verb	Narrow	r1	153.401360544194	1	on_focus	4	Narrow on_focus
2023206	block5	HumanSpeech	Pre	laam4	Object	Narrow	r1	300.079365079341	1	post_focus	4	Narrow post_focus
2023206	block5	HumanSpeech	Pre	kau4	Object	Narrow	r1	259.537414965962	2	post_focus	4	Narrow post_focus
2023206	block5	HumanSpeech	Pre	wai5	Subject	Narrow	r1	286.502267573667	1	pre_focus	5	Narrow pre_focus
2023206	block5	HumanSpeech	Pre	wai5	Subject	Narrow	r1	281.597608740469	2	pre_focus	5	Narrow pre_focus
2023206	block5	HumanSpeech	Pre	waat3	Verb	Narrow	r1	253.2426303855	1	pre_focus	3	Narrow pre_focus
2023206	block5	HumanSpeech	Pre	bui3	Object	Narrow	r1	192.396069538916	1	on_focus	3	Narrow on_focus
2023206	block5	HumanSpeech	Pre	hok3	Object	Narrow	r1	140.097721628308	2	on_focus	3	Narrow on_focus
2023206	block5	HumanSpeech	Pre	ceoi3	Subject	Contrastive	r1	210.244250081018	1	on_focus	3	Contrastive on_focus
2023206	block5	HumanSpeech	Pre	ceoi3	Subject	Contrastive	r1	172.162257495586	2	on_focus	3	Contrastive on_focus
2023206	block5	HumanSpeech	Pre	caa4	Verb	Contrastive	r1	117.156462585001	1	post_focus	4	Contrastive post_focus
2023206	block5	HumanSpeech	Pre	ngau4	Object	Contrastive	r1	257.551020408187	1	post_focus	4	Contrastive post_focus
2023206	block5	HumanSpeech	Pre	jau4	Object	Contrastive	r1	165.80498866216	2	post_focus	4	Contrastive post_focus
2023206	block5	HumanSpeech	Pre	siu2	Subject	Contrastive	r1	292.263038548697	1	pre_focus	2	Contrastive pre_focus
2023206	block5	HumanSpeech	Pre	gwong2	Subject	Contrastive	r1	640.397856112145	2	pre_focus	2	Contrastive pre_focus
2023206	block5	HumanSpeech	Pre	cyun4	Verb	Contrastive	r1	226.335348954365	1	pre_focus	4	Contrastive pre_focus
2023206	block5	HumanSpeech	Pre	laam4	Object	Contrastive	r1	207.579365079368	1	on_focus	4	Contrastive on_focus
2023206	block5	HumanSpeech	Pre	kau4	Object	Contrastive	r1	316.712018140549	2	on_focus	4	Contrastive on_focus
2023206	block5	HumanSpeech	Pre	siu2	Subject	Contrastive	r1	239.523053665891	1	on_focus	2	Contrastive on_focus
2023206	block5	HumanSpeech	Pre	gwong2	Subject	Contrastive	r1	231.21315192742	2	on_focus	2	Contrastive on_focus
2023206	block5	HumanSpeech	Pre	cyun4	Verb	Contrastive	r1	199.83803045028	1	post_focus	4	Contrastive post_focus
2023206	block5	HumanSpeech	Pre	laam4	Object	Contrastive	r1	208.191987906218	1	post_focus	4	Contrastive post_focus
2023206	block5	HumanSpeech	Pre	kau4	Object	Contrastive	r1	109.314058956897	2	post_focus	4	Contrastive post_focus
2023206	block5	HumanSpeech	Pre	wai5	Subject	Broad	r1	386.477702191939	1	broad_focus	5	Broad focus

2023206	block5	HumanSpeech	Pre	wai5	Subject	Broad	r1	461.931216931191	2	broad_focus	5	Broad focus
2023206	block5	HumanSpeech	Pre	waat3	Verb	Broad	r1	218.276643990919	1	broad_focus	3	Broad focus
2023206	block5	HumanSpeech	Pre	bui3	Object	Broad	r1	254.253320375767	1	broad_focus	3	Broad focus
2023206	block5	HumanSpeech	Pre	hok3	Object	Broad	r1	163.465608465572	2	broad_focus	3	Broad focus
2023206	block5	HumanSpeech	Pre	ceoi3	Subject	Narrow	r1	222.370874275612	1	pre_focus	3	Narrow pre_focus
2023206	block5	HumanSpeech	Pre	ceoi3	Subject	Narrow	r1	217.938397581293	2	pre_focus	3	Narrow pre_focus
2023206	block5	HumanSpeech	Pre	caa4	Verb	Narrow	r1	202.382842025713	1	on_focus	4	Narrow on_focus
2023206	block5	HumanSpeech	Pre	ngau4	Object	Narrow	r1	230.670889045314	1	post_focus	4	Narrow post_focus
2023206	block5	HumanSpeech	Pre	jau4	Object	Narrow	r1	218.820861677955	2	post_focus	4	Narrow post_focus
2023206	block5	HumanSpeech	Pre	wai5	Subject	Narrow	r1	212.057823129271	1	pre_focus	5	Narrow pre_focus
2023206	block5	HumanSpeech	Pre	wai5	Subject	Narrow	r1	278.775510204127	2	pre_focus	5	Narrow pre_focus
2023206	block5	HumanSpeech	Pre	waat3	Verb	Narrow	r1	235.684051398266	1	on_focus	3	Narrow on_focus
2023206	block5	HumanSpeech	Pre	bui3	Object	Narrow	r1	204.72713529864	1	post_focus	3	Narrow post_focus
2023206	block5	HumanSpeech	Pre	hok3	Object	Narrow	r1	101.682539682542	2	post_focus	3	Narrow post_focus
2023206	block5	HumanSpeech	Pre	ceoi3	Subject	Contrastive	r2	338.230158730198	1	pre_focus	3	Contrastive pre_focus
2023206	block5	HumanSpeech	Pre	ceoi3	Subject	Contrastive	r2	342.069160997767	2	pre_focus	3	Contrastive pre_focus
2023206	block5	HumanSpeech	Pre	caa4	Verb	Contrastive	r2	167.270771556446	1	on_focus	4	Contrastive on_focus
2023206	block5	HumanSpeech	Pre	ngau4	Object	Contrastive	r2	258.073593073618	1	post_focus	4	Contrastive post_focus
2023206	block5	HumanSpeech	Pre	jau4	Object	Contrastive	r2	185.400604686265	2	post_focus	4	Contrastive post_focus
2023206	block5	HumanSpeech	Pre	siu2	Subject	Broad	r2	281.893424036184	1	broad_focus	2	Broad focus
2023206	block5	HumanSpeech	Pre	gwong2	Subject	Broad	r2	298.152958152968	2	broad_focus	2	Broad focus
2023206	block5	HumanSpeech	Pre	cyun4	Verb	Broad	r2	274.448979591853	1	broad_focus	4	Broad focus
2023206	block5	HumanSpeech	Pre	laam4	Object	Broad	r2	263.755102040818	1	broad_focus	4	Broad focus
2023206	block5	HumanSpeech	Pre	kau4	Object	Broad	r2	173.00075585797	2	broad_focus	4	Broad focus
2023206	block5	HumanSpeech	Pre	wai5	Subject	Narrow	r2	341.337868480764	1	pre_focus	5	Narrow pre_focus
2023206	block5	HumanSpeech	Pre	wai5	Subject	Narrow	r2	271.190476190441	2	pre_focus	5	Narrow pre_focus
2023206	block5	HumanSpeech	Pre	waat3	Verb	Narrow	r2	202.44520030235	1	pre_focus	3	Narrow pre_focus
2023206	block5	HumanSpeech	Pre	bui3	Object	Narrow	r2	332.155814706766	1	on_focus	3	Narrow on_focus
2023206	block5	HumanSpeech	Pre	hok3	Object	Narrow	r2	108.677248677168	2	on_focus	3	Narrow on_focus
2023206	block5	HumanSpeech	Pre	wai5	Subject	Contrastive	r2	325.537414965993	1	pre_focus	5	Contrastive pre_focus
2023206	block5	HumanSpeech	Pre	wai5	Subject	Contrastive	r2	432.791950113369	2	pre_focus	5	Contrastive pre_focus
2023206	block5	HumanSpeech	Pre	waat3	Verb	Contrastive	r2	215.884353741558	1	on_focus	3	Contrastive on_focus
2023206	block5	HumanSpeech	Pre	bui3	Object	Contrastive	r2	215.553287981834	1	post_focus	3	Contrastive post_focus
2023206	block5	HumanSpeech	Pre	hok3	Object	Contrastive	r2	173.16704459563	2	post_focus	3	Contrastive post_focus
2023206	block5	HumanSpeech	Pre	ceoi3	Subject	Narrow	r2	206.262428048035	1	on_focus	3	Narrow on_focus
2023206	block5	HumanSpeech	Pre	ceoi3	Subject	Narrow	r2	155.457590443575	2	on_focus	3	Narrow on_focus
2023206	block5	HumanSpeech	Pre	caa4	Verb	Narrow	r2	199.952128999712	1	post_focus	4	Narrow post_focus
2023206	block5	HumanSpeech	Pre	ngau4	Object	Narrow	r2	248.010204081652	1	post_focus	4	Narrow post_focus
2023206	block5	HumanSpeech	Pre	jau4	Object	Narrow	r2	204.055177626628	2	post_focus	4	Narrow post_focus
2023206	block5	HumanSpeech	Pre	ceoi3	Subject	Narrow	r2	162.335600906999	1	pre_focus	3	Narrow pre_focus
2023206	block5	HumanSpeech	Pre	ceoi3	Subject	Narrow	r2	141.215419501123	2	pre_focus	3	Narrow pre_focus
2023206	block5	HumanSpeech	Pre	caa4	Verb	Narrow	r2	191.831065759629	1	pre_focus	4	Narrow pre_focus
2023206	block5	HumanSpeech	Pre	ngau4	Object	Narrow	r2	266.950113378584	1	on_focus	4	Narrow on_focus
2023206	block5	HumanSpeech	Pre	jau4	Object	Narrow	r2	245.616024187484	2	on_focus	4	Narrow on_focus
2023206	block5	HumanSpeech	Pre	siu2	Subject	Narrow	r2	275.171687722718	1	pre_focus	2	Narrow pre_focus
2023206	block5	HumanSpeech	Pre	gwong2	Subject	Narrow	r2	468.850514564792	2	pre_focus	2	Narrow pre_focus
2023206	block5	HumanSpeech	Pre	cyun4	Verb	Narrow	r2	226.594860166301	1	pre_focus	4	Narrow pre_focus
2023206	block5	HumanSpeech	Pre	laam4	Object	Narrow	r2	282.930839002233	1	on_focus	4	Narrow on_focus
2023206	block5	HumanSpeech	Pre	kau4	Object	Narrow	r2	119.188712522032	2	on_focus	4	Narrow on_focus
2023206	block5	HumanSpeech	Pre	wai5	Subject	Broad	r2	448.507936507895	1	broad_focus	5	Broad focus
2023206	block5	HumanSpeech	Pre	wai5	Subject	Broad	r2	415.722789115648	2	broad_focus	5	Broad focus
2023206	block5	HumanSpeech	Pre	waat3	Verb	Broad	r2	233.435374149622	1	broad_focus	3	Broad focus
2023206	block5	HumanSpeech	Pre	bui3	Object	Broad	r2	202.704459561573	1	broad_focus	3	Broad focus
2023206	block5	HumanSpeech	Pre	hok3	Object	Broad	r2	80.7823129250664	2	broad_focus	3	Broad focus
2023206	block5	HumanSpeech	Pre	wai5	Subject	Contrastive	r2	266.955782312948	1	pre_focus	5	Contrastive pre_focus
2023206	block5	HumanSpeech	Pre	wai5	Subject	Contrastive	r2	307.985098801396	2	pre_focus	5	Contrastive pre_focus
2023206	block5	HumanSpeech	Pre	waat3	Verb	Contrastive	r2	236.583522297906	1	pre_focus	3	Contrastive pre_focus
2023206	block5	HumanSpeech	Pre	bui3	Object	Contrastive	r2	278.089754420307	1	on_focus	3	Contrastive on_focus
2023206	block5	HumanSpeech	Pre	hok3	Object	Contrastive	r2	197.58812615953	2	on_focus	3	Contrastive on_focus
2023206	block5	HumanSpeech	Pre	ceoi3	Subject	Broad	r2	156.247165532818	1	broad_focus	3	Broad focus
2023206	block5	HumanSpeech	Pre	ceoi3	Subject	Broad	r2	162.565192743727	2	broad_focus	3	Broad focus
2023206	block5	HumanSpeech	Pre	caa4	Verb	Broad	r2	169.807256235799	1	broad_focus	4	Broad focus

2023206	block5	HumanSpeech	Pre	ngau4	Object	Broad	r2	215.094926859592	1	broad_focus	4	Broad focus
2023206	block5	HumanSpeech	Pre	jau4	Object	Broad	r2	251.625094482165	2	broad_focus	4	Broad focus
2023206	block5	HumanSpeech	Pre	siu2	Subject	Contrastive	r2	283.110936682419	1	on_focus	2	Contrastive on_focus
2023206	block5	HumanSpeech	Pre	gwong2	Subject	Contrastive	r2	250.410509031212	2	on_focus	2	Contrastive on_focus
2023206	block5	HumanSpeech	Pre	cyun4	Verb	Contrastive	r2	192.180110139361	1	post_focus	4	Contrastive post_focus
2023206	block5	HumanSpeech	Pre	laam4	Object	Contrastive	r2	241.624554583723	1	post_focus	4	Contrastive post_focus
2023206	block5	HumanSpeech	Pre	kau4	Object	Contrastive	r2	239.072562358274	2	post_focus	4	Contrastive post_focus
2023206	block5	HumanSpeech	Pre	ceoi3	Subject	Narrow	r2	150.548798644081	1	pre_focus	3	Narrow pre_focus
2023206	block5	HumanSpeech	Pre	ceoi3	Subject	Narrow	r2	134.224489795884	2	pre_focus	3	Narrow pre_focus
2023206	block5	HumanSpeech	Pre	caa4	Verb	Narrow	r2	168.076341647748	1	on_focus	4	Narrow on_focus
2023206	block5	HumanSpeech	Pre	ngau4	Object	Narrow	r2	276.065759637163	1	post_focus	4	Narrow post_focus
2023206	block5	HumanSpeech	Pre	jau4	Object	Narrow	r2	291.83390022672	2	post_focus	4	Narrow post_focus
2023206	block5	HumanSpeech	Pre	ceoi3	Subject	Contrastive	r2	189.055933484497	1	pre_focus	3	Contrastive pre_focus
2023206	block5	HumanSpeech	Pre	ceoi3	Subject	Contrastive	r2	173.111435050146	2	pre_focus	3	Contrastive pre_focus
2023206	block5	HumanSpeech	Pre	caa4	Verb	Contrastive	r2	197.057823129285	1	pre_focus	4	Contrastive pre_focus
2023206	block5	HumanSpeech	Pre	ngau4	Object	Contrastive	r2	279.193121693083	1	on_focus	4	Contrastive on_focus
2023206	block5	HumanSpeech	Pre	jau4	Object	Contrastive	r2	315.416666666692	2	on_focus	4	Contrastive on_focus
2023206	block5	HumanSpeech	Pre	siu2	Subject	Narrow	r2	249.894179894227	1	pre_focus	2	Narrow pre_focus
2023206	block5	HumanSpeech	Pre	gwong2	Subject	Narrow	r2	319.843886272338	2	pre_focus	2	Narrow pre_focus
2023206	block5	HumanSpeech	Pre	cyun4	Verb	Narrow	r2	212.105064247908	1	on_focus	4	Narrow on_focus
2023206	block5	HumanSpeech	Pre	laam4	Object	Narrow	r2	211.135848278786	1	post_focus	4	Narrow post_focus
2023206	block5	HumanSpeech	Pre	kau4	Object	Narrow	r2	163.565759637208	2	post_focus	4	Narrow post_focus
2023206	block5	HumanSpeech	Pre	ceoi3	Subject	Contrastive	r2	190.973748473766	1	on_focus	3	Contrastive on_focus
2023206	block5	HumanSpeech	Pre	ceoi3	Subject	Contrastive	r2	177.624716553282	2	on_focus	3	Contrastive on_focus
2023206	block5	HumanSpeech	Pre	caa4	Verb	Contrastive	r2	127.648202137948	1	post_focus	4	Contrastive post_focus
2023206	block5	HumanSpeech	Pre	ngau4	Object	Contrastive	r2	255.314981806691	1	post_focus	4	Contrastive post_focus
2023206	block5	HumanSpeech	Pre	jau4	Object	Contrastive	r2	280.963718820772	2	post_focus	4	Contrastive post_focus
2023206	block5	HumanSpeech	Pre	wai5	Subject	Contrastive	r2	422.637944066537	1	on_focus	5	Contrastive on_focus
2023206	block5	HumanSpeech	Pre	wai5	Subject	Contrastive	r2	384.518140589648	2	on_focus	5	Contrastive on_focus
2023206	block5	HumanSpeech	Pre	waat3	Verb	Contrastive	r2	192.237339380199	1	post_focus	3	Contrastive post_focus
2023206	block5	HumanSpeech	Pre	bui3	Object	Contrastive	r2	189.764334305096	1	post_focus	3	Contrastive post_focus
2023206	block5	HumanSpeech	Pre	hok3	Object	Contrastive	r2	175.212396069469	2	post_focus	3	Contrastive post_focus
2023206	block5	HumanSpeech	Pre	siu2	Subject	Contrastive	r2	276.929705215366	1	pre_focus	2	Contrastive pre_focus
2023206	block5	HumanSpeech	Pre	gwong2	Subject	Contrastive	r2	305.092703748187	2	pre_focus	2	Contrastive pre_focus
2023206	block5	HumanSpeech	Pre	cyun4	Verb	Contrastive	r2	158.968820861787	1	on_focus	4	Contrastive on_focus
2023206	block5	HumanSpeech	Pre	laam4	Object	Contrastive	r2	247.63200518305	1	post_focus	4	Contrastive post_focus
2023206	block5	HumanSpeech	Pre	kau4	Object	Contrastive	r2	183.10009718175	2	post_focus	4	Contrastive post_focus
2023206	block5	HumanSpeech	Pre	siu2	Subject	Contrastive	r2	275.652305366521	1	pre_focus	2	Contrastive pre_focus
2023206	block5	HumanSpeech	Pre	gwong2	Subject	Contrastive	r2	269.481946624865	2	pre_focus	2	Contrastive pre_focus
2023206	block5	HumanSpeech	Pre	cyun4	Verb	Contrastive	r2	177.346938775486	1	pre_focus	4	Contrastive pre_focus
2023206	block5	HumanSpeech	Pre	laam4	Object	Contrastive	r2	316.45691609981	1	on_focus	4	Contrastive on_focus
2023206	block5	HumanSpeech	Pre	kau4	Object	Contrastive	r2	250.385487528433	2	on_focus	4	Contrastive on_focus
2023206	block5	HumanSpeech	Pre	wai5	Subject	Narrow	r2	387.906273620501	1	on_focus	5	Narrow on_focus
2023206	block5	HumanSpeech	Pre	wai5	Subject	Narrow	r2	397.06916099783	2	on_focus	5	Narrow on_focus
2023206	block5	HumanSpeech	Pre	waat3	Verb	Narrow	r2	213.870748299314	1	post_focus	3	Narrow post_focus
2023206	block5	HumanSpeech	Pre	bui3	Object	Narrow	r2	219.697116942029	1	post_focus	3	Narrow post_focus
2023206	block5	HumanSpeech	Pre	hok3	Object	Narrow	r2	178.939909297014	2	post_focus	3	Narrow post_focus
2023206	block5	HumanSpeech	Pre	wai5	Subject	Narrow	r2	366.791383220061	1	pre_focus	5	Narrow pre_focus
2023206	block5	HumanSpeech	Pre	wai5	Subject	Narrow	r2	262.023809523726	2	pre_focus	5	Narrow pre_focus
2023206	block5	HumanSpeech	Pre	waat3	Verb	Narrow	r2	143.88133030991	1	on_focus	3	Narrow on_focus
2023206	block5	HumanSpeech	Pre	bui3	Object	Narrow	r2	199.998969284707	1	post_focus	3	Narrow post_focus
2023206	block5	HumanSpeech	Pre	hok3	Object	Narrow	r2	179.724111866904	2	post_focus	3	Narrow post_focus
2023206	block5	HumanSpeech	Pre	siu2	Subject	Narrow	r2	222.341785198978	1	on_focus	2	Narrow on_focus
2023206	block5	HumanSpeech	Pre	gwong2	Subject	Narrow	r2	239.763321995497	2	on_focus	2	Narrow on_focus
2023206	block5	HumanSpeech	Pre	cyun4	Verb	Narrow	r2	163.238095238171	1	post_focus	4	Narrow post_focus
2023206	block5	HumanSpeech	Pre	laam4	Object	Narrow	r2	286.209372637927	1	post_focus	4	Narrow post_focus
2023206	block5	HumanSpeech	Pre	kau4	Object	Narrow	r2	153.469387755081	2	post_focus	4	Narrow post_focus
2023207	block1	HumanSpeech	Post	jyun2	Subject	Narrow	r1	79.399848828416	1	pre_focus	2	Narrow pre_focus
2023207	block1	HumanSpeech	Post	jyun2	Subject	Narrow	r1	75.9720554888759	2	pre_focus	2	Narrow pre_focus
2023207	block1	HumanSpeech	Post	mo2	Verb	Narrow	r1	105.419595616013	1	pre_focus	2	Narrow pre_focus
2023207	block1	HumanSpeech	Post	gau2	Object	Narrow	r1	100.905769715297	1	on_focus	2	Narrow on_focus
2023207	block1	HumanSpeech	Post	zai2	Object	Narrow	r1	178.720181405879	2	on_focus	2	Narrow on_focus

2023207	block1	HumanSpeech	Post	zoeng1	Subject	Narrow	r1	165.345717774301	1	on_focus	1	Narrow on_focus
2023207	block1	HumanSpeech	Post	saang1	Subject	Narrow	r1	131.564831993387	2	on_focus	1	Narrow on_focus
2023207	block1	HumanSpeech	Post	tsa1	Verb	Narrow	r1	124.751875109013	1	post_focus	1	Narrow post_focus
2023207	block1	HumanSpeech	Post	fei1	Object	Narrow	r1	140.234315948618	1	post_focus	1	Narrow post_focus
2023207	block1	HumanSpeech	Post	gei1	Object	Narrow	r1	373.959435626119	2	post_focus	1	Narrow post_focus
2023207	block1	HumanSpeech	Post	jyun2	Subject	Narrow	r1	162.910169195897	1	pre_focus	2	Narrow pre_focus
2023207	block1	HumanSpeech	Post	jyun2	Subject	Narrow	r1	159.280423280421	2	pre_focus	2	Narrow pre_focus
2023207	block1	HumanSpeech	Post	mo2	Verb	Narrow	r1	192.760255617401	1	on_focus	2	Narrow on_focus
2023207	block1	HumanSpeech	Post	gau2	Object	Narrow	r1	130.302073210231	1	post_focus	2	Narrow post_focus
2023207	block1	HumanSpeech	Post	zai2	Object	Narrow	r1	211.873687746703	2	post_focus	2	Narrow post_focus
2023207	block1	HumanSpeech	Post	sau3	Subject	Contrastive	r1	85.1571104632285	1	pre_focus	3	Contrastive pre_focus
2023207	block1	HumanSpeech	Post	sau3	Subject	Contrastive	r1	105.35492927329	2	pre_focus	3	Contrastive pre_focus
2023207	block1	HumanSpeech	Post	sik3	Verb	Contrastive	r1	86.5721844293148	1	pre_focus	3	Contrastive pre_focus
2023207	block1	HumanSpeech	Post	baak3	Object	Contrastive	r1	108.071228491411	1	on_focus	3	Contrastive on_focus
2023207	block1	HumanSpeech	Post	baak3	Object	Contrastive	r1	147.994571565988	2	on_focus	3	Contrastive on_focus
2023207	block1	HumanSpeech	Post	sau3	Subject	Contrastive	r1	66.4156543569732	1	pre_focus	3	Contrastive pre_focus
2023207	block1	HumanSpeech	Post	sau3	Subject	Contrastive	r1	116.027940313671	2	pre_focus	3	Contrastive pre_focus
2023207	block1	HumanSpeech	Post	sik3	Verb	Contrastive	r1	49.4714393693982	1	on_focus	3	Contrastive on_focus
2023207	block1	HumanSpeech	Post	baak3	Object	Contrastive	r1	93.185302981226	1	post_focus	3	Contrastive post_focus
2023207	block1	HumanSpeech	Post	baak3	Object	Contrastive	r1	146.570294784567	2	post_focus	3	Contrastive post_focus
2023207	block1	HumanSpeech	Post	zoeng1	Subject	Narrow	r1	122.610589209216	1	pre_focus	1	Narrow pre_focus
2023207	block1	HumanSpeech	Post	saang1	Subject	Narrow	r1	107.350202707352	2	pre_focus	1	Narrow pre_focus
2023207	block1	HumanSpeech	Post	tsa1	Verb	Narrow	r1	131.538278803589	1	on_focus	1	Narrow on_focus
2023207	block1	HumanSpeech	Post	fei1	Object	Narrow	r1	148.207234113158	1	post_focus	1	Narrow post_focus
2023207	block1	HumanSpeech	Post	gei1	Object	Narrow	r1	128.753617118491	2	post_focus	1	Narrow post_focus
2023207	block1	HumanSpeech	Post	sau3	Subject	Contrastive	r1	119.632912648797	1	on_focus	3	Contrastive on_focus
2023207	block1	HumanSpeech	Post	sau3	Subject	Contrastive	r1	118.980725623601	2	on_focus	3	Contrastive on_focus
2023207	block1	HumanSpeech	Post	sik3	Verb	Contrastive	r1	63.1628117913863	1	post_focus	3	Contrastive post_focus
2023207	block1	HumanSpeech	Post	baak3	Object	Contrastive	r1	93.3361678004587	1	post_focus	3	Contrastive post_focus
2023207	block1	HumanSpeech	Post	baak3	Object	Contrastive	r1	146.129843241653	2	post_focus	3	Contrastive post_focus
2023207	block1	HumanSpeech	Post	sau3	Subject	Narrow	r1	129.977324263052	1	on_focus	3	Narrow on_focus
2023207	block1	HumanSpeech	Post	sau3	Subject	Narrow	r1	130.179661608253	2	on_focus	3	Narrow on_focus
2023207	block1	HumanSpeech	Post	sik3	Verb	Narrow	r1	63.6272457701068	1	post_focus	3	Narrow post_focus
2023207	block1	HumanSpeech	Post	baak3	Object	Narrow	r1	93.5779696494023	1	post_focus	3	Narrow post_focus
2023207	block1	HumanSpeech	Post	baak3	Object	Narrow	r1	103.310308738912	2	post_focus	3	Narrow post_focus
2023207	block1	HumanSpeech	Post	sau3	Subject	Broad	r1	129.364970868721	1	broad_focus	3	Broad focus
2023207	block1	HumanSpeech	Post	sau3	Subject	Broad	r1	113.739876903168	2	broad_focus	3	Broad focus
2023207	block1	HumanSpeech	Post	sik3	Verb	Broad	r1	108.329931972804	1	broad_focus	3	Broad focus
2023207	block1	HumanSpeech	Post	baak3	Object	Broad	r1	125.025080739363	1	broad_focus	3	Broad focus
2023207	block1	HumanSpeech	Post	baak3	Object	Broad	r1	261.473922902496	2	broad_focus	3	Broad focus
2023207	block1	HumanSpeech	Post	zoeng1	Subject	Narrow	r1	161.160997732424	1	pre_focus	1	Narrow pre_focus
2023207	block1	HumanSpeech	Post	saang1	Subject	Narrow	r1	157.031997984404	2	pre_focus	1	Narrow pre_focus
2023207	block1	HumanSpeech	Post	tsa1	Verb	Narrow	r1	91.6846182917652	1	pre_focus	1	Narrow pre_focus
2023207	block1	HumanSpeech	Post	fei1	Object	Narrow	r1	127.174981103565	1	on_focus	1	Narrow on_focus
2023207	block1	HumanSpeech	Post	gei1	Object	Narrow	r1	311.48592770441	2	on_focus	1	Narrow on_focus
2023207	block1	HumanSpeech	Post	zoeng1	Subject	Contrastive	r1	218.15354713317	1	pre_focus	1	Contrastive pre_focus
2023207	block1	HumanSpeech	Post	saang1	Subject	Contrastive	r1	228.284889713478	2	pre_focus	1	Contrastive pre_focus
2023207	block1	HumanSpeech	Post	tsa1	Verb	Contrastive	r1	186.98299319729	1	on_focus	1	Contrastive on_focus
2023207	block1	HumanSpeech	Post	fei1	Object	Contrastive	r1	205.115898211119	1	post_focus	1	Contrastive post_focus
2023207	block1	HumanSpeech	Post	gei1	Object	Contrastive	r1	357.5033346672	2	post_focus	1	Contrastive post_focus
2023207	block1	HumanSpeech	Post	zoeng1	Subject	Contrastive	r1	161.146096533855	1	pre_focus	1	Contrastive pre_focus
2023207	block1	HumanSpeech	Post	saang1	Subject	Contrastive	r1	198.131252500986	2	pre_focus	1	Contrastive pre_focus
2023207	block1	HumanSpeech	Post	tsa1	Verb	Contrastive	r1	135.337868480718	1	pre_focus	1	Contrastive pre_focus
2023207	block1	HumanSpeech	Post	fei1	Object	Contrastive	r1	166.478961955136	1	on_focus	1	Contrastive on_focus
2023207	block1	HumanSpeech	Post	gei1	Object	Contrastive	r1	323.510378510377	2	on_focus	1	Contrastive on_focus
2023207	block1	HumanSpeech	Post	sau3	Subject	Narrow	r1	170.179894179881	1	pre_focus	3	Narrow pre_focus
2023207	block1	HumanSpeech	Post	sau3	Subject	Narrow	r1	184.855523161616	2	pre_focus	3	Narrow pre_focus
2023207	block1	HumanSpeech	Post	sik3	Verb	Narrow	r1	100.337868480722	1	on_focus	3	Narrow on_focus
2023207	block1	HumanSpeech	Post	baak3	Object	Narrow	r1	107.252013449056	1	post_focus	3	Narrow post_focus
2023207	block1	HumanSpeech	Post	baak3	Object	Narrow	r1	156.574452003042	2	post_focus	3	Narrow post_focus
2023207	block1	HumanSpeech	Post	jyun2	Subject	Narrow	r1	258.22222222223	1	on_focus	2	Narrow on_focus
2023207	block1	HumanSpeech	Post	jyun2	Subject	Narrow	r1	137.92139077853	2	on_focus	2	Narrow on_focus

2023207	block1	HumanSpeech	Post	mo2	Verb	Narrow	r1	314.111866969	1	post_focus	2	Narrow post_focus
2023207	block1	HumanSpeech	Post	gau2	Object	Narrow	r1	161.681783824633	1	post_focus	2	Narrow post_focus
2023207	block1	HumanSpeech	Post	zai2	Object	Narrow	r1	122.311035525343	2	post_focus	2	Narrow post_focus
2023207	block1	HumanSpeech	Post	jyun2	Subject	Contrastive	r1	175.787981859429	1	on_focus	2	Contrastive on_focus
2023207	block1	HumanSpeech	Post	jyun2	Subject	Contrastive	r1	172.226001511717	2	on_focus	2	Contrastive on_focus
2023207	block1	HumanSpeech	Post	mo2	Verb	Contrastive	r1	164.580498866201	1	post_focus	2	Contrastive post_focus
2023207	block1	HumanSpeech	Post	gau2	Object	Contrastive	r1	154.750566893426	1	post_focus	2	Contrastive post_focus
2023207	block1	HumanSpeech	Post	zai2	Object	Contrastive	r1	243.318216175368	2	post_focus	2	Contrastive post_focus
2023207	block1	HumanSpeech	Post	sau3	Subject	Narrow	r1	103.061224489807	1	pre_focus	3	Narrow pre_focus
2023207	block1	HumanSpeech	Post	sau3	Subject	Narrow	r1	158.880385487493	2	pre_focus	3	Narrow pre_focus
2023207	block1	HumanSpeech	Post	sik3	Verb	Narrow	r1	118.912436769563	1	pre_focus	3	Narrow pre_focus
2023207	block1	HumanSpeech	Post	baak3	Object	Narrow	r1	125.723905723902	1	on_focus	3	Narrow on_focus
2023207	block1	HumanSpeech	Post	baak3	Object	Narrow	r1	217.092916531698	2	on_focus	3	Narrow on_focus
2023207	block1	HumanSpeech	Post	jyun2	Subject	Broad	r1	62.9227405247832	1	broad_focus	2	Broad focus
2023207	block1	HumanSpeech	Post	jyun2	Subject	Broad	r1	68.6400856639011	2	broad_focus	2	Broad focus
2023207	block1	HumanSpeech	Post	mo2	Verb	Broad	r1	183.192743764181	1	broad_focus	2	Broad focus
2023207	block1	HumanSpeech	Post	gau2	Object	Broad	r1	94.4036281179024	1	broad_focus	2	Broad focus
2023207	block1	HumanSpeech	Post	zai2	Object	Broad	r1	310.164399092969	2	broad_focus	2	Broad focus
2023207	block1	HumanSpeech	Post	jyun2	Subject	Contrastive	r1	108.031448089349	1	pre_focus	2	Contrastive pre_focus
2023207	block1	HumanSpeech	Post	jyun2	Subject	Contrastive	r1	88.3936473139784	2	pre_focus	2	Contrastive pre_focus
2023207	block1	HumanSpeech	Post	mo2	Verb	Contrastive	r1	189.978835978849	1	on_focus	2	Contrastive on_focus
2023207	block1	HumanSpeech	Post	gau2	Object	Contrastive	r1	75.253304573863	1	post_focus	2	Contrastive post_focus
2023207	block1	HumanSpeech	Post	zai2	Object	Contrastive	r1	193.654153019253	2	post_focus	2	Contrastive post_focus
2023207	block1	HumanSpeech	Post	zoeng1	Subject	Contrastive	r1	150.489686825466	1	on_focus	1	Contrastive on_focus
2023207	block1	HumanSpeech	Post	saang1	Subject	Contrastive	r1	161.648454968514	2	on_focus	1	Contrastive on_focus
2023207	block1	HumanSpeech	Post	tsa1	Verb	Contrastive	r1	136.430632859202	1	post_focus	1	Contrastive post_focus
2023207	block1	HumanSpeech	Post	fei1	Object	Contrastive	r1	108.002645502637	1	post_focus	1	Contrastive post_focus
2023207	block1	HumanSpeech	Post	gei1	Object	Contrastive	r1	243.087679516265	2	post_focus	1	Contrastive post_focus
2023207	block1	HumanSpeech	Post	jyun2	Subject	Contrastive	r1	85.6331131486741	1	pre_focus	2	Contrastive pre_focus
2023207	block1	HumanSpeech	Post	jyun2	Subject	Contrastive	r1	69.8656462585632	2	pre_focus	2	Contrastive pre_focus
2023207	block1	HumanSpeech	Post	mo2	Verb	Contrastive	r1	162.972657662067	1	pre_focus	2	Contrastive pre_focus
2023207	block1	HumanSpeech	Post	gau2	Object	Contrastive	r1	63.4170391966222	1	on_focus	2	Contrastive on_focus
2023207	block1	HumanSpeech	Post	zai2	Object	Contrastive	r1	227.142857142894	2	on_focus	2	Contrastive on_focus
2023207	block1	HumanSpeech	Post	zoeng1	Subject	Broad	r2	81.3080120937002	1	broad_focus	1	Broad focus
2023207	block1	HumanSpeech	Post	saang1	Subject	Broad	r2	117.673847316723	2	broad_focus	1	Broad focus
2023207	block1	HumanSpeech	Post	tsa1	Verb	Broad	r2	115.576487005058	1	broad_focus	1	Broad focus
2023207	block1	HumanSpeech	Post	fei1	Object	Broad	r2	133.410430839035	1	broad_focus	1	Broad focus
2023207	block1	HumanSpeech	Post	gei1	Object	Broad	r2	171.713852207574	2	broad_focus	1	Broad focus
2023207	block1	HumanSpeech	Post	zoeng1	Subject	Narrow	r2	144.06608357632	1	pre_focus	1	Narrow pre_focus
2023207	block1	HumanSpeech	Post	saang1	Subject	Narrow	r2	161.856818918011	2	pre_focus	1	Narrow pre_focus
2023207	block1	HumanSpeech	Post	tsa1	Verb	Narrow	r2	114.877173091486	1	pre_focus	1	Narrow pre_focus
2023207	block1	HumanSpeech	Post	fei1	Object	Contrastive	r2	109.939058956911	1	on_focus	1	Contrastive on_focus
2023207	block1	HumanSpeech	Post	gei1	Object	Narrow	r2	131.522945686243	2	on_focus	1	Narrow on_focus
2023207	block1	HumanSpeech	Post	jyun2	Subject	Narrow	r2	156.597445995942	1	pre_focus	2	Narrow pre_focus
2023207	block1	HumanSpeech	Post	jyun2	Subject	Narrow	r2	111.552028218682	2	pre_focus	2	Narrow pre_focus
2023207	block1	HumanSpeech	Post	mo2	Verb	Narrow	r2	121.200620599041	1	pre_focus	2	Narrow pre_focus
2023207	block1	HumanSpeech	Post	gau2	Object	Narrow	r2	102.495590828937	1	on_focus	2	Narrow on_focus
2023207	block1	HumanSpeech	Post	zai2	Object	Narrow	r2	317.132496676777	2	on_focus	2	Narrow on_focus
2023207	block1	HumanSpeech	Post	sau3	Subject	Broad	r2	74.6915263801498	1	broad_focus	3	Broad focus
2023207	block1	HumanSpeech	Post	sau3	Subject	Broad	r2	113.223369141792	2	broad_focus	3	Broad focus
2023207	block1	HumanSpeech	Post	sik3	Verb	Broad	r2	71.9954648526482	1	broad_focus	3	Broad focus
2023207	block1	HumanSpeech	Post	baak3	Object	Broad	r2	95.4257805686325	1	broad_focus	3	Broad focus
2023207	block1	HumanSpeech	Post	baak3	Object	Broad	r2	121.225329638037	2	broad_focus	3	Broad focus
2023207	block1	HumanSpeech	Post	sau3	Subject	Narrow	r2	64.333585285965	1	pre_focus	3	Narrow pre_focus
2023207	block1	HumanSpeech	Post	sau3	Subject	Narrow	r2	131.193715581503	2	pre_focus	3	Narrow pre_focus
2023207	block1	HumanSpeech	Post	sik3	Verb	Narrow	r2	101.429104975296	1	on_focus	3	Narrow on_focus
2023207	block1	HumanSpeech	Post	baak3	Object	Narrow	r2	100.027210884377	1	post_focus	3	Narrow post_focus
2023207	block1	HumanSpeech	Post	baak3	Object	Narrow	r2	167.175251052754	2	post_focus	3	Narrow post_focus
2023207	block1	HumanSpeech	Post	jyun2	Subject	Narrow	r2	173.618669690086	1	on_focus	2	Narrow on_focus
2023207	block1	HumanSpeech	Post	jyun2	Subject	Narrow	r2	65.0844041320511	2	on_focus	2	Narrow on_focus
2023207	block1	HumanSpeech	Post	mo2	Verb	Narrow	r2	214.500323939092	1	post_focus	2	Narrow post_focus
2023207	block1	HumanSpeech	Post	gau2	Object	Narrow	r2	132.017573696146	1	post_focus	2	Narrow post_focus

2023207	block1	HumanSpeech	Post	zai2	Object	Narrow	r2	168.371504157165	2	post_focus	2	Narrow post_focus
2023207	block1	HumanSpeech	Post	zoeng1	Subject	Contrastive	r2	134.344438630137	1	pre_focus	1	Contrastive pre_focus
2023207	block1	HumanSpeech	Post	saang1	Subject	Contrastive	r2	338.783446712	2	pre_focus	1	Contrastive pre_focus
2023207	block1	HumanSpeech	Post	tsa1	Verb	Contrastive	r2	103.819601914836	1	pre_focus	1	Contrastive pre_focus
2023207	block1	HumanSpeech	Post	fei1	Object	Contrastive	r2	175.540249433141	1	on_focus	1	Contrastive on_focus
2023207	block1	HumanSpeech	Post	gei1	Object	Contrastive	r2	318.530612244842	2	on_focus	1	Contrastive on_focus
2023207	block1	HumanSpeech	Post	sau3	Subject	Contrastive	r2	155.95871248928	1	on_focus	3	Contrastive on_focus
2023207	block1	HumanSpeech	Post	sau3	Subject	Contrastive	r2	107.043706371428	2	on_focus	3	Contrastive on_focus
2023207	block1	HumanSpeech	Post	sik3	Verb	Contrastive	r2	72.6487093154446	1	post_focus	3	Contrastive post_focus
2023207	block1	HumanSpeech	Post	baak3	Object	Contrastive	r2	120.753968254007	1	post_focus	3	Contrastive post_focus
2023207	block1	HumanSpeech	Post	baak3	Object	Contrastive	r2	263.898337112607	2	post_focus	3	Contrastive post_focus
2023207	block1	HumanSpeech	Post	zoeng1	Subject	Broad	r2	120.820861678055	1	broad_focus	1	Broad focus
2023207	block1	HumanSpeech	Post	saang1	Subject	Broad	r2	182.190098261572	2	broad_focus	1	Broad focus
2023207	block1	HumanSpeech	Post	tsa1	Verb	Broad	r2	122.286470143649	1	broad_focus	1	Broad focus
2023207	block1	HumanSpeech	Post	fei1	Object	Broad	r2	139.064625850324	1	broad_focus	1	Broad focus
2023207	block1	HumanSpeech	Post	gei1	Object	Broad	r2	277.506424792136	2	broad_focus	1	Broad focus
2023207	block1	HumanSpeech	Post	jyun2	Subject	Broad	r2	318.575207860931	1	broad_focus	2	Broad focus
2023207	block1	HumanSpeech	Post	jyun2	Subject	Broad	r2	167.088435374183	2	broad_focus	2	Broad focus
2023207	block1	HumanSpeech	Post	mo2	Verb	Broad	r2	234.889455782309	1	broad_focus	2	Broad focus
2023207	block1	HumanSpeech	Post	gau2	Object	Broad	r2	207.41801848942	1	broad_focus	2	Broad focus
2023207	block1	HumanSpeech	Post	zai2	Object	Broad	r2	218.659863945618	2	broad_focus	2	Broad focus
2023207	block1	HumanSpeech	Post	jyun2	Subject	Narrow	r2	152.946226109464	1	pre_focus	2	Narrow pre_focus
2023207	block1	HumanSpeech	Post	jyun2	Subject	Narrow	r2	97.36281179147	2	pre_focus	2	Narrow pre_focus
2023207	block1	HumanSpeech	Post	mo2	Verb	Narrow	r2	262.836986646562	1	on_focus	2	Narrow on_focus
2023207	block1	HumanSpeech	Post	gau2	Object	Narrow	r2	146.331317712225	1	post_focus	2	Narrow post_focus
2023207	block1	HumanSpeech	Post	zai2	Object	Narrow	r2	217.148526077096	2	post_focus	2	Narrow post_focus
2023207	block1	HumanSpeech	Post	zoeng1	Subject	Contrastive	r2	87.4214938500586	1	pre_focus	1	Contrastive pre_focus
2023207	block1	HumanSpeech	Post	saang1	Subject	Contrastive	r2	80.7867793582204	2	pre_focus	1	Contrastive pre_focus
2023207	block1	HumanSpeech	Post	tsa1	Verb	Contrastive	r2	104.604889574887	1	on_focus	1	Contrastive on_focus
2023207	block1	HumanSpeech	Post	fei1	Object	Contrastive	r2	98.3180518583708	1	post_focus	1	Contrastive post_focus
2023207	block1	HumanSpeech	Post	gei1	Object	Contrastive	r2	269.119562976755	2	post_focus	1	Contrastive post_focus
2023207	block1	HumanSpeech	Post	jyun2	Subject	Contrastive	r2	76.0722384191581	1	on_focus	2	Contrastive on_focus
2023207	block1	HumanSpeech	Post	jyun2	Subject	Contrastive	r2	154.462759462774	2	on_focus	2	Contrastive on_focus
2023207	block1	HumanSpeech	Post	mo2	Verb	Contrastive	r2	181.08876884088	1	post_focus	2	Contrastive post_focus
2023207	block1	HumanSpeech	Post	gau2	Object	Contrastive	r2	139.601662887344	1	post_focus	2	Contrastive post_focus
2023207	block1	HumanSpeech	Post	zai2	Object	Contrastive	r2	356.251700680275	2	post_focus	2	Contrastive post_focus
2023207	block1	HumanSpeech	Post	zoeng1	Subject	Narrow	r2	102.447948876545	1	on_focus	1	Narrow on_focus
2023207	block1	HumanSpeech	Post	saang1	Subject	Narrow	r2	150.700912843831	2	on_focus	1	Narrow on_focus
2023207	block1	HumanSpeech	Post	tsa1	Verb	Narrow	r2	114.863945578236	1	post_focus	1	Narrow post_focus
2023207	block1	HumanSpeech	Post	fei1	Object	Narrow	r2	135.352823669166	1	post_focus	1	Narrow post_focus
2023207	block1	HumanSpeech	Post	gei1	Object	Narrow	r2	151.278998778935	2	post_focus	1	Narrow post_focus
2023207	block1	HumanSpeech	Post	jyun2	Subject	Contrastive	r2	198.46412374477	1	pre_focus	2	Contrastive pre_focus
2023207	block1	HumanSpeech	Post	jyun2	Subject	Contrastive	r2	151.787603930472	2	pre_focus	2	Contrastive pre_focus
2023207	block1	HumanSpeech	Post	mo2	Verb	Contrastive	r2	176.398809523846	1	on_focus	2	Contrastive on_focus
2023207	block1	HumanSpeech	Post	gau2	Object	Contrastive	r2	149.993815708058	1	post_focus	2	Contrastive post_focus
2023207	block1	HumanSpeech	Post	zai2	Object	Contrastive	r2	224.875283446693	2	post_focus	2	Contrastive post_focus
2023207	block1	HumanSpeech	Post	zoeng1	Subject	Narrow	r2	54.6759756534243	1	pre_focus	1	Narrow pre_focus
2023207	block1	HumanSpeech	Post	saang1	Subject	Narrow	r2	64.7235621521531	2	pre_focus	1	Narrow pre_focus
2023207	block1	HumanSpeech	Post	tsa1	Verb	Narrow	r2	111.271227009297	1	on_focus	1	Narrow on_focus
2023207	block1	HumanSpeech	Post	fei1	Object	Narrow	r2	121.650793650758	1	post_focus	1	Narrow post_focus
2023207	block1	HumanSpeech	Post	gei1	Object	Narrow	r2	173.442621774427	2	post_focus	1	Narrow post_focus
2023207	block1	HumanSpeech	Post	zoeng1	Subject	Contrastive	r2	77.9875283446927	1	on_focus	1	Contrastive on_focus
2023207	block1	HumanSpeech	Post	saang1	Subject	Contrastive	r2	142.148526077108	2	on_focus	1	Contrastive on_focus
2023207	block1	HumanSpeech	Post	tsa1	Verb	Contrastive	r2	143.1708238851	1	post_focus	1	Contrastive post_focus
2023207	block1	HumanSpeech	Post	fei1	Object	Contrastive	r2	140.641399416893	1	post_focus	1	Contrastive post_focus
2023207	block1	HumanSpeech	Post	gei1	Object	Contrastive	r2	331.764056049792	2	post_focus	1	Contrastive post_focus
2023207	block1	HumanSpeech	Post	jyun2	Subject	Narrow	r2	76.9674981103208	1	pre_focus	2	Narrow pre_focus
2023207	block1	HumanSpeech	Post	jyun2	Subject	Narrow	r2	124.840136054445	2	pre_focus	2	Narrow pre_focus
2023207	block1	HumanSpeech	Post	mo2	Verb	Narrow	r2	203.161753590337	1	pre_focus	2	Narrow pre_focus
2023207	block1	HumanSpeech	Post	gau2	Object	Narrow	r2	109.603174603194	1	on_focus	2	Narrow on_focus
2023207	block1	HumanSpeech	Post	zai2	Object	Narrow	r2	242.043650793619	2	on_focus	2	Narrow on_focus
2023207	block1	HumanSpeech	Post	sau3	Subject	Narrow	r2	93.8752834467209	1	pre_focus	3	Narrow pre_focus

2023207	block1	HumanSpeech	Post	sau3	Subject	Narrow	r2	122.131519274376	2	pre_focus	3	Narrow pre_focus
2023207	block1	HumanSpeech	Post	sik3	Verb	Narrow	r2	74.0889689209325	1	pre_focus	3	Narrow pre_focus
2023207	block1	HumanSpeech	Post	baak3	Object	Narrow	r2	122.170823885142	1	on_focus	3	Narrow on_focus
2023207	block1	HumanSpeech	Post	baak3	Object	Narrow	r2	182.907029478486	2	on_focus	3	Narrow on_focus
2023207	block1	HumanSpeech	Post	sau3	Subject	Narrow	r2	55.0896722324978	1	on_focus	3	Narrow on_focus
2023207	block1	HumanSpeech	Post	sau3	Subject	Narrow	r2	82.4379852951438	2	on_focus	3	Narrow on_focus
2023207	block1	HumanSpeech	Post	sik3	Verb	Narrow	r2	106.09221466359	1	post_focus	3	Narrow post_focus
2023207	block1	HumanSpeech	Post	baak3	Object	Narrow	r2	154.590576971543	1	post_focus	3	Narrow post_focus
2023207	block1	HumanSpeech	Post	baak3	Object	Narrow	r2	183.878732048356	2	post_focus	3	Narrow post_focus
2023207	block1	HumanSpeech	Post	sau3	Subject	Contrastive	r2	101.523809523826	1	pre_focus	3	Contrastive pre_focus
2023207	block1	HumanSpeech	Post	sau3	Subject	Contrastive	r2	106.865746298581	2	pre_focus	3	Contrastive pre_focus
2023207	block1	HumanSpeech	Post	sik3	Verb	Contrastive	r2	63.8806431663852	1	on_focus	3	Contrastive on_focus
2023207	block1	HumanSpeech	Post	baak3	Object	Contrastive	r2	83.4750566893376	1	post_focus	3	Contrastive post_focus
2023207	block1	HumanSpeech	Post	baak3	Object	Contrastive	r2	102.246315192758	2	post_focus	3	Contrastive post_focus
2023207	block1	HumanSpeech	Post	sau3	Subject	Contrastive	r2	60.9273338965295	1	pre_focus	3	Contrastive pre_focus
2023207	block1	HumanSpeech	Post	sau3	Subject	Contrastive	r2	102.085772949295	2	pre_focus	3	Contrastive pre_focus
2023207	block1	HumanSpeech	Post	sik3	Verb	Contrastive	r2	66.9916855630959	1	pre_focus	3	Contrastive pre_focus
2023207	block1	HumanSpeech	Post	baak3	Object	Contrastive	r2	126.269841269846	1	on_focus	3	Contrastive on_focus
2023207	block1	HumanSpeech	Post	baak3	Object	Contrastive	r2	155.310333657269	2	on_focus	3	Contrastive on_focus
2023207	block1	HumanSpeech	Pre	sau3	Subject	Contrastive	r1	136.056748463346	1	on_focus	3	Contrastive on_focus
2023207	block1	HumanSpeech	Pre	sau3	Subject	Contrastive	r1	131.469647453116	2	on_focus	3	Contrastive on_focus
2023207	block1	HumanSpeech	Pre	sik3	Verb	Contrastive	r1	98.0638681307084	1	post_focus	3	Contrastive post_focus
2023207	block1	HumanSpeech	Pre	baak3	Object	Contrastive	r1	143.796494259078	1	post_focus	3	Contrastive post_focus
2023207	block1	HumanSpeech	Pre	baak3	Object	Contrastive	r1	174.474171381206	2	post_focus	3	Contrastive post_focus
2023207	block1	HumanSpeech	Pre	sau3	Subject	Narrow	r1	160.155107155646	1	pre_focus	3	Narrow pre_focus
2023207	block1	HumanSpeech	Pre	sau3	Subject	Narrow	r1	159.431699754748	2	pre_focus	3	Narrow pre_focus
2023207	block1	HumanSpeech	Pre	sik3	Verb	Narrow	r1	132.879376858995	1	on_focus	3	Narrow on_focus
2023207	block1	HumanSpeech	Pre	baak3	Object	Narrow	r1	133.566704459554	1	post_focus	3	Narrow post_focus
2023207	block1	HumanSpeech	Pre	baak3	Object	Narrow	r1	126.651360544201	2	post_focus	3	Narrow post_focus
2023207	block1	HumanSpeech	Pre	sau3	Subject	Narrow	r1	141.220312686471	1	pre_focus	3	Narrow pre_focus
2023207	block1	HumanSpeech	Pre	sau3	Subject	Narrow	r1	150.241771456081	2	pre_focus	3	Narrow pre_focus
2023207	block1	HumanSpeech	Pre	sik3	Verb	Narrow	r1	131.065431003321	1	pre_focus	3	Narrow pre_focus
2023207	block1	HumanSpeech	Pre	baak3	Object	Narrow	r1	124.941111798279	1	on_focus	3	Narrow on_focus
2023207	block1	HumanSpeech	Pre	baak3	Object	Narrow	r1	141.879337593622	2	on_focus	3	Narrow on_focus
2023207	block1	HumanSpeech	Pre	sau3	Subject	Contrastive	r1	201.451653764934	1	pre_focus	3	Contrastive pre_focus
2023207	block1	HumanSpeech	Pre	sau3	Subject	Contrastive	r1	188.670452161915	2	pre_focus	3	Contrastive pre_focus
2023207	block1	HumanSpeech	Pre	sik3	Verb	Contrastive	r1	113.370271388959	1	pre_focus	3	Contrastive pre_focus
2023207	block1	HumanSpeech	Pre	baak3	Object	Contrastive	r1	120.517190040999	1	on_focus	3	Contrastive on_focus
2023207	block1	HumanSpeech	Pre	baak3	Object	Contrastive	r1	128.316178248639	2	on_focus	3	Contrastive on_focus
2023207	block1	HumanSpeech	Pre	zoeng1	Subject	Narrow	r1	327.086979787993	1	pre_focus	1	Narrow pre_focus
2023207	block1	HumanSpeech	Pre	saang1	Subject	Narrow	r1	242.40779836839	2	pre_focus	1	Narrow pre_focus
2023207	block1	HumanSpeech	Pre	tsa1	Verb	Narrow	r1	158.559354477745	1	on_focus	1	Narrow on_focus
2023207	block1	HumanSpeech	Pre	fei1	Object	Narrow	r1	186.593406593403	1	post_focus	1	Narrow post_focus
2023207	block1	HumanSpeech	Pre	gei1	Object	Narrow	r1	152.019807923182	2	post_focus	1	Narrow post_focus
2023207	block1	HumanSpeech	Pre	jyun2	Subject	Contrastive	r1	219.363639635759	1	on_focus	2	Contrastive on_focus
2023207	block1	HumanSpeech	Pre	jyun2	Subject	Contrastive	r1	197.06746031747	2	on_focus	2	Contrastive on_focus
2023207	block1	HumanSpeech	Pre	mo2	Verb	Contrastive	r1	251.254724111874	1	post_focus	2	Contrastive post_focus
2023207	block1	HumanSpeech	Pre	gau2	Object	Contrastive	r1	179.298777481989	1	post_focus	2	Contrastive post_focus
2023207	block1	HumanSpeech	Pre	zai2	Object	Contrastive	r1	304.498866213152	2	post_focus	2	Contrastive post_focus
2023207	block1	HumanSpeech	Pre	sau3	Subject	Broad	r1	145.808216637306	1	broad_focus	3	Broad focus
2023207	block1	HumanSpeech	Pre	sau3	Subject	Broad	r1	156.379636195993	2	broad_focus	3	Broad focus
2023207	block1	HumanSpeech	Pre	sik3	Verb	Broad	r1	133.820501153707	1	broad_focus	3	Broad focus
2023207	block1	HumanSpeech	Pre	baak3	Object	Broad	r1	121.31793272863	1	broad_focus	3	Broad focus
2023207	block1	HumanSpeech	Pre	baak3	Object	Broad	r1	222.513626207501	2	broad_focus	3	Broad focus
2023207	block1	HumanSpeech	Pre	zoeng1	Subject	Contrastive	r1	232.695652663608	1	pre_focus	1	Contrastive pre_focus
2023207	block1	HumanSpeech	Pre	saang1	Subject	Contrastive	r1	219.97413146795	2	pre_focus	1	Contrastive pre_focus
2023207	block1	HumanSpeech	Pre	tsa1	Verb	Contrastive	r1	192.958296668053	1	on_focus	1	Contrastive on_focus
2023207	block1	HumanSpeech	Pre	fei1	Object	Contrastive	r1	254.325351133105	1	post_focus	1	Contrastive post_focus
2023207	block1	HumanSpeech	Pre	gei1	Object	Contrastive	r1	383.56055730651	2	post_focus	1	Contrastive post_focus
2023207	block1	HumanSpeech	Pre	zoeng1	Subject	Broad	r1	222.129575235613	1	broad_focus	1	Broad focus
2023207	block1	HumanSpeech	Pre	saang1	Subject	Broad	r1	224.854823504188	2	broad_focus	1	Broad focus
2023207	block1	HumanSpeech	Pre	tsa1	Verb	Broad	r1	192.245498265891	1	broad_focus	1	Broad focus

2023207	block1	HumanSpeech	Pre	fei1	Object	Broad	r1	196.555007313407	1	broad_focus	1	Broad focus
2023207	block1	HumanSpeech	Pre	gei1	Object	Broad	r1	396.869830716355	2	broad_focus	1	Broad focus
2023207	block1	HumanSpeech	Pre	jyun2	Subject	Contrastive	r1	220.34398771558	1	pre_focus	2	Contrastive pre_focus
2023207	block1	HumanSpeech	Pre	jyun2	Subject	Contrastive	r1	170.27518163809	2	pre_focus	2	Contrastive pre_focus
2023207	block1	HumanSpeech	Pre	mo2	Verb	Contrastive	r1	236.593936817087	1	on_focus	2	Contrastive on_focus
2023207	block1	HumanSpeech	Pre	gau2	Object	Contrastive	r1	202.092233326823	1	post_focus	2	Contrastive post_focus
2023207	block1	HumanSpeech	Pre	zai2	Object	Contrastive	r1	414.140056562985	2	post_focus	2	Contrastive post_focus
2023207	block1	HumanSpeech	Pre	zoeng1	Subject	Narrow	r1	213.596790377665	1	pre_focus	1	Narrow pre_focus
2023207	block1	HumanSpeech	Pre	saang1	Subject	Narrow	r1	209.328438627267	2	pre_focus	1	Narrow pre_focus
2023207	block1	HumanSpeech	Pre	tsa1	Verb	Narrow	r1	214.903628117895	1	pre_focus	1	Narrow pre_focus
2023207	block1	HumanSpeech	Pre	fei1	Object	Narrow	r1	198.351365943211	1	on_focus	1	Narrow on_focus
2023207	block1	HumanSpeech	Pre	gei1	Object	Narrow	r1	516.023557571145	2	on_focus	1	Narrow on_focus
2023207	block1	HumanSpeech	Pre	jyun2	Subject	Broad	r1	231.734623975285	1	broad_focus	2	Broad focus
2023207	block1	HumanSpeech	Pre	jyun2	Subject	Broad	r1	169.849161518357	2	broad_focus	2	Broad focus
2023207	block1	HumanSpeech	Pre	mo2	Verb	Broad	r1	261.245891772376	1	broad_focus	2	Broad focus
2023207	block1	HumanSpeech	Pre	gau2	Object	Broad	r1	219.288584353308	1	broad_focus	2	Broad focus
2023207	block1	HumanSpeech	Pre	zai2	Object	Broad	r1	562.525408973102	2	broad_focus	2	Broad focus
2023207	block1	HumanSpeech	Pre	zoeng1	Subject	Narrow	r1	205.536466551166	1	on_focus	1	Narrow on_focus
2023207	block1	HumanSpeech	Pre	saang1	Subject	Narrow	r1	216.514464371585	2	on_focus	1	Narrow on_focus
2023207	block1	HumanSpeech	Pre	tsa1	Verb	Narrow	r1	179.229141611074	1	post_focus	1	Narrow post_focus
2023207	block1	HumanSpeech	Pre	fei1	Object	Narrow	r1	203.91351915606	1	post_focus	1	Narrow post_focus
2023207	block1	HumanSpeech	Pre	gei1	Object	Narrow	r1	357.288207352553	2	post_focus	1	Narrow post_focus
2023207	block1	HumanSpeech	Pre	zoeng1	Subject	Contrastive	r1	205.245463264703	1	pre_focus	1	Contrastive pre_focus
2023207	block1	HumanSpeech	Pre	saang1	Subject	Contrastive	r1	190.041414588165	2	pre_focus	1	Contrastive pre_focus
2023207	block1	HumanSpeech	Pre	tsa1	Verb	Contrastive	r1	177.203876612509	1	pre_focus	1	Contrastive pre_focus
2023207	block1	HumanSpeech	Pre	fei1	Object	Contrastive	r1	196.827227119627	1	on_focus	1	Contrastive on_focus
2023207	block1	HumanSpeech	Pre	gei1	Object	Contrastive	r1	401.738882812992	2	on_focus	1	Contrastive on_focus
2023207	block1	HumanSpeech	Pre	jyun2	Subject	Narrow	r1	253.112827618281	1	on_focus	2	Narrow on_focus
2023207	block1	HumanSpeech	Pre	jyun2	Subject	Narrow	r1	163.283072370177	2	on_focus	2	Narrow on_focus
2023207	block1	HumanSpeech	Pre	mo2	Verb	Narrow	r1	265.163049346711	1	post_focus	2	Narrow post_focus
2023207	block1	HumanSpeech	Pre	gau2	Object	Narrow	r1	237.282553341458	1	post_focus	2	Narrow post_focus
2023207	block1	HumanSpeech	Pre	zai2	Object	Narrow	r1	498.357808178241	2	post_focus	2	Narrow post_focus
2023207	block1	HumanSpeech	Pre	zoeng1	Subject	Contrastive	r1	221.769997394347	1	on_focus	1	Contrastive on_focus
2023207	block1	HumanSpeech	Pre	saang1	Subject	Contrastive	r1	201.465448222223	2	on_focus	1	Contrastive on_focus
2023207	block1	HumanSpeech	Pre	tsa1	Verb	Contrastive	r1	189.127029936515	1	post_focus	1	Contrastive post_focus
2023207	block1	HumanSpeech	Pre	fei1	Object	Contrastive	r1	205.967725672963	1	post_focus	1	Contrastive post_focus
2023207	block1	HumanSpeech	Pre	gei1	Object	Contrastive	r1	334.597769266395	2	post_focus	1	Contrastive post_focus
2023207	block1	HumanSpeech	Pre	jyun2	Subject	Narrow	r1	194.875897218765	1	pre_focus	2	Narrow pre_focus
2023207	block1	HumanSpeech	Pre	jyun2	Subject	Narrow	r1	196.562138353045	2	pre_focus	2	Narrow pre_focus
2023207	block1	HumanSpeech	Pre	mo2	Verb	Narrow	r1	282.441899430069	1	on_focus	2	Narrow on_focus
2023207	block1	HumanSpeech	Pre	gau2	Object	Narrow	r1	205.74587449255	1	post_focus	2	Narrow post_focus
2023207	block1	HumanSpeech	Pre	zai2	Object	Narrow	r1	503.647466866994	2	post_focus	2	Narrow post_focus
2023207	block1	HumanSpeech	Pre	jyun2	Subject	Contrastive	r1	215.783145187856	1	pre_focus	2	Contrastive pre_focus
2023207	block1	HumanSpeech	Pre	jyun2	Subject	Contrastive	r1	189.140445684529	2	pre_focus	2	Contrastive pre_focus
2023207	block1	HumanSpeech	Pre	mo2	Verb	Contrastive	r1	249.986370657155	1	pre_focus	2	Contrastive pre_focus
2023207	block1	HumanSpeech	Pre	gau2	Object	Contrastive	r1	218.924921097937	1	on_focus	2	Contrastive on_focus
2023207	block1	HumanSpeech	Pre	zai2	Object	Contrastive	r1	514.347732238321	2	on_focus	2	Contrastive on_focus
2023207	block1	HumanSpeech	Pre	sau3	Subject	Narrow	r1	106.902561168283	1	on_focus	3	Narrow on_focus
2023207	block1	HumanSpeech	Pre	sau3	Subject	Narrow	r1	128.085434847321	2	on_focus	3	Narrow on_focus
2023207	block1	HumanSpeech	Pre	sik3	Verb	Narrow	r1	83.7470252191679	1	post_focus	3	Narrow post_focus
2023207	block1	HumanSpeech	Pre	baak3	Object	Narrow	r1	108.688672664471	1	post_focus	3	Narrow post_focus
2023207	block1	HumanSpeech	Pre	baak3	Object	Narrow	r1	248.460974142802	2	post_focus	3	Narrow post_focus
2023207	block1	HumanSpeech	Pre	sau3	Subject	Contrastive	r1	137.080887149352	1	pre_focus	3	Contrastive pre_focus
2023207	block1	HumanSpeech	Pre	sau3	Subject	Contrastive	r1	155.772788703473	2	pre_focus	3	Contrastive pre_focus
2023207	block1	HumanSpeech	Pre	sik3	Verb	Contrastive	r1	117.599479946364	1	on_focus	3	Contrastive on_focus
2023207	block1	HumanSpeech	Pre	baak3	Object	Contrastive	r1	219.75995643254	1	post_focus	3	Contrastive post_focus
2023207	block1	HumanSpeech	Pre	baak3	Object	Contrastive	r1	175.044514837339	2	post_focus	3	Contrastive post_focus
2023207	block1	HumanSpeech	Pre	jyun2	Subject	Narrow	r1	209.257735662163	1	pre_focus	2	Narrow pre_focus
2023207	block1	HumanSpeech	Pre	jyun2	Subject	Narrow	r1	254.573434603969	2	pre_focus	2	Narrow pre_focus
2023207	block1	HumanSpeech	Pre	mo2	Verb	Narrow	r1	285.857165663174	1	pre_focus	2	Narrow pre_focus
2023207	block1	HumanSpeech	Pre	gau2	Object	Narrow	r1	177.578870781872	1	on_focus	2	Narrow on_focus
2023207	block1	HumanSpeech	Pre	zai2	Object	Narrow	r1	471.709855583754	2	on_focus	2	Narrow on_focus

2023207	block1	HumanSpeech	Pre	jyun2	Subject	Contrastive	r2	246.846724502973	1	on_focus	2	Contrastive on_focus
2023207	block1	HumanSpeech	Pre	jyun2	Subject	Contrastive	r2	161.346205516281	2	on_focus	2	Contrastive on_focus
2023207	block1	HumanSpeech	Pre	mo2	Verb	Contrastive	r2	260.604216978777	1	post_focus	2	Contrastive post_focus
2023207	block1	HumanSpeech	Pre	gau2	Object	Contrastive	r2	227.107743237923	1	post_focus	2	Contrastive post_focus
2023207	block1	HumanSpeech	Pre	zai2	Object	Contrastive	r2	474.331949935674	2	post_focus	2	Contrastive post_focus
2023207	block1	HumanSpeech	Pre	sau3	Subject	Contrastive	r2	186.164768745186	1	pre_focus	3	Contrastive pre_focus
2023207	block1	HumanSpeech	Pre	sau3	Subject	Contrastive	r2	168.364136214961	2	pre_focus	3	Contrastive pre_focus
2023207	block1	HumanSpeech	Pre	sik3	Verb	Contrastive	r2	206.356395734986	1	pre_focus	3	Contrastive pre_focus
2023207	block1	HumanSpeech	Pre	baak3	Object	Contrastive	r2	141.625466193602	1	on_focus	3	Contrastive on_focus
2023207	block1	HumanSpeech	Pre	baak3	Object	Contrastive	r2	254.900259771432	2	on_focus	3	Contrastive on_focus
2023207	block1	HumanSpeech	Pre	sau3	Subject	Contrastive	r2	190.134109433188	1	on_focus	3	Contrastive on_focus
2023207	block1	HumanSpeech	Pre	sau3	Subject	Contrastive	r2	201.393605608814	2	on_focus	3	Contrastive on_focus
2023207	block1	HumanSpeech	Pre	sik3	Verb	Contrastive	r2	153.147458055059	1	post_focus	3	Contrastive post_focus
2023207	block1	HumanSpeech	Pre	baak3	Object	Contrastive	r2	142.746099467502	1	post_focus	3	Contrastive post_focus
2023207	block1	HumanSpeech	Pre	baak3	Object	Contrastive	r2	258.114866942492	2	post_focus	3	Contrastive post_focus
2023207	block1	HumanSpeech	Pre	zoeng1	Subject	Narrow	r2	203.281454803061	1	pre_focus	1	Narrow pre_focus
2023207	block1	HumanSpeech	Pre	saang1	Subject	Narrow	r2	203.324402471537	2	pre_focus	1	Narrow pre_focus
2023207	block1	HumanSpeech	Pre	tsa1	Verb	Narrow	r2	184.246340630295	1	pre_focus	1	Narrow pre_focus
2023207	block1	HumanSpeech	Pre	fei1	Object	Narrow	r2	225.171930285796	1	on_focus	1	Narrow on_focus
2023207	block1	HumanSpeech	Pre	gei1	Object	Narrow	r2	384.763526656741	2	on_focus	1	Narrow on_focus
2023207	block1	HumanSpeech	Pre	sau3	Subject	Narrow	r2	179.799324917212	1	on_focus	3	Narrow on_focus
2023207	block1	HumanSpeech	Pre	sau3	Subject	Narrow	r2	188.751518499089	2	on_focus	3	Narrow on_focus
2023207	block1	HumanSpeech	Pre	sik3	Verb	Narrow	r2	183.511075302476	1	post_focus	3	Narrow post_focus
2023207	block1	HumanSpeech	Pre	baak3	Object	Narrow	r2	177.793720928776	1	post_focus	3	Narrow post_focus
2023207	block1	HumanSpeech	Pre	baak3	Object	Narrow	r2	298.836723709201	2	post_focus	3	Narrow post_focus
2023207	block1	HumanSpeech	Pre	jyun2	Subject	Narrow	r2	245.484779118954	1	pre_focus	2	Narrow pre_focus
2023207	block1	HumanSpeech	Pre	jyun2	Subject	Narrow	r2	151.335851177294	2	pre_focus	2	Narrow pre_focus
2023207	block1	HumanSpeech	Pre	mo2	Verb	Narrow	r2	283.388071698823	1	on_focus	2	Narrow on_focus
2023207	block1	HumanSpeech	Pre	gau2	Object	Narrow	r2	206.726446937523	1	post_focus	2	Narrow post_focus
2023207	block1	HumanSpeech	Pre	zai2	Object	Narrow	r2	455.497873020363	2	post_focus	2	Narrow post_focus
2023207	block1	HumanSpeech	Pre	zoeng1	Subject	Contrastive	r2	206.363236817651	1	on_focus	1	Contrastive on_focus
2023207	block1	HumanSpeech	Pre	saang1	Subject	Contrastive	r2	213.792409861981	2	on_focus	1	Contrastive on_focus
2023207	block1	HumanSpeech	Pre	tsa1	Verb	Contrastive	r2	175.287895604413	1	post_focus	1	Contrastive post_focus
2023207	block1	HumanSpeech	Pre	fei1	Object	Contrastive	r2	219.491354875288	1	post_focus	1	Contrastive post_focus
2023207	block1	HumanSpeech	Pre	gei1	Object	Contrastive	r2	362.980935172402	2	post_focus	1	Contrastive post_focus
2023207	block1	HumanSpeech	Pre	jyun2	Subject	Contrastive	r2	240.528476256941	1	pre_focus	2	Contrastive pre_focus
2023207	block1	HumanSpeech	Pre	jyun2	Subject	Contrastive	r2	173.539230053336	2	pre_focus	2	Contrastive pre_focus
2023207	block1	HumanSpeech	Pre	mo2	Verb	Contrastive	r2	259.585540316948	1	pre_focus	2	Contrastive pre_focus
2023207	block1	HumanSpeech	Pre	gau2	Object	Contrastive	r2	202.355593805692	1	on_focus	2	Contrastive on_focus
2023207	block1	HumanSpeech	Pre	zai2	Object	Contrastive	r2	473.51479428761	2	on_focus	2	Contrastive on_focus
2023207	block1	HumanSpeech	Pre	sau3	Subject	Narrow	r2	159.817683639801	1	pre_focus	3	Narrow pre_focus
2023207	block1	HumanSpeech	Pre	sau3	Subject	Narrow	r2	189.143883144027	2	pre_focus	3	Narrow pre_focus
2023207	block1	HumanSpeech	Pre	sik3	Verb	Narrow	r2	176.175831267983	1	on_focus	3	Narrow on_focus
2023207	block1	HumanSpeech	Pre	baak3	Object	Narrow	r2	178.185417107159	1	post_focus	3	Narrow post_focus
2023207	block1	HumanSpeech	Pre	baak3	Object	Narrow	r2	260.500785852287	2	post_focus	3	Narrow post_focus
2023207	block1	HumanSpeech	Pre	zoeng1	Subject	Broad	r2	176.561969549766	1	broad_focus	1	Broad focus
2023207	block1	HumanSpeech	Pre	saang1	Subject	Broad	r2	209.123464712491	2	broad_focus	1	Broad focus
2023207	block1	HumanSpeech	Pre	tsa1	Verb	Broad	r2	186.494715252536	1	broad_focus	1	Broad focus
2023207	block1	HumanSpeech	Pre	fei1	Object	Broad	r2	220.970589840249	1	broad_focus	1	Broad focus
2023207	block1	HumanSpeech	Pre	gei1	Object	Broad	r2	443.556246870457	2	broad_focus	1	Broad focus
2023207	block1	HumanSpeech	Pre	zoeng1	Subject	Narrow	r2	178.491446761882	1	pre_focus	1	Narrow pre_focus
2023207	block1	HumanSpeech	Pre	saang1	Subject	Narrow	r2	188.856334888726	2	pre_focus	1	Narrow pre_focus
2023207	block1	HumanSpeech	Pre	tsa1	Verb	Narrow	r2	188.736205353507	1	on_focus	1	Narrow on_focus
2023207	block1	HumanSpeech	Pre	fei1	Object	Narrow	r2	209.383401743253	1	post_focus	1	Narrow post_focus
2023207	block1	HumanSpeech	Pre	gei1	Object	Narrow	r2	380.015247801396	2	post_focus	1	Narrow post_focus
2023207	block1	HumanSpeech	Pre	jyun2	Subject	Narrow	r2	195.020726340601	1	on_focus	2	Narrow on_focus
2023207	block1	HumanSpeech	Pre	jyun2	Subject	Narrow	r2	218.576077837099	2	on_focus	2	Narrow on_focus
2023207	block1	HumanSpeech	Pre	mo2	Verb	Narrow	r2	234.50378131048	1	post_focus	2	Narrow post_focus
2023207	block1	HumanSpeech	Pre	gau2	Object	Narrow	r2	200.96678272688	1	post_focus	2	Narrow post_focus
2023207	block1	HumanSpeech	Pre	zai2	Object	Narrow	r2	238.092297157436	2	post_focus	2	Narrow post_focus
2023207	block1	HumanSpeech	Pre	jyun2	Subject	Narrow	r2	104.492673332004	1	pre_focus	2	Narrow pre_focus
2023207	block1	HumanSpeech	Pre	jyun2	Subject	Narrow	r2	101.205753914314	2	pre_focus	2	Narrow pre_focus

2023207	block1	HumanSpeech	Pre	mo2	Verb	Narrow	r2	156.496598639478	1	pre_focus	2	Narrow pre_focus
2023207	block1	HumanSpeech	Pre	gau2	Object	Narrow	r2	94.8785228376892	1	on_focus	2	Narrow on_focus
2023207	block1	HumanSpeech	Pre	zai2	Object	Narrow	r2	354.151464897768	2	on_focus	2	Narrow on_focus
2023207	block1	HumanSpeech	Pre	jyun2	Subject	Contrastive	r2	234.636141636145	1	pre_focus	2	Contrastive pre_focus
2023207	block1	HumanSpeech	Pre	jyun2	Subject	Contrastive	r2	159.181133459185	2	pre_focus	2	Contrastive pre_focus
2023207	block1	HumanSpeech	Pre	mo2	Verb	Contrastive	r2	244.682348809363	1	on_focus	2	Contrastive on_focus
2023207	block1	HumanSpeech	Pre	gau2	Object	Contrastive	r2	231.583773686452	1	post_focus	2	Contrastive post_focus
2023207	block1	HumanSpeech	Pre	zai2	Object	Contrastive	r2	316.09685779074	2	post_focus	2	Contrastive post_focus
2023207	block1	HumanSpeech	Pre	zoeng1	Subject	Contrastive	r2	173.010660541252	1	pre_focus	1	Contrastive pre_focus
2023207	block1	HumanSpeech	Pre	saang1	Subject	Contrastive	r2	243.069686360514	2	pre_focus	1	Contrastive pre_focus
2023207	block1	HumanSpeech	Pre	tsa1	Verb	Contrastive	r2	131.161544341523	1	on_focus	1	Contrastive on_focus
2023207	block1	HumanSpeech	Pre	fei1	Object	Contrastive	r2	147.759313249082	1	post_focus	1	Contrastive post_focus
2023207	block1	HumanSpeech	Pre	gei1	Object	Contrastive	r2	351.327682588192	2	post_focus	1	Contrastive post_focus
2023207	block1	HumanSpeech	Pre	sau3	Subject	Broad	r2	196.691378007642	1	broad_focus	3	Broad focus
2023207	block1	HumanSpeech	Pre	sau3	Subject	Broad	r2	175.865040956978	2	broad_focus	3	Broad focus
2023207	block1	HumanSpeech	Pre	sik3	Verb	Broad	r2	193.963185274129	1	broad_focus	3	Broad focus
2023207	block1	HumanSpeech	Pre	baak3	Object	Broad	r2	199.700238959281	1	broad_focus	3	Broad focus
2023207	block1	HumanSpeech	Pre	baak3	Object	Broad	r2	295.132222914106	2	broad_focus	3	Broad focus
2023207	block1	HumanSpeech	Pre	jyun2	Subject	Broad	r2	236.545525249142	1	broad_focus	2	Broad focus
2023207	block1	HumanSpeech	Pre	jyun2	Subject	Broad	r2	183.615972824498	2	broad_focus	2	Broad focus
2023207	block1	HumanSpeech	Pre	mo2	Verb	Broad	r2	269.421754171788	1	broad_focus	2	Broad focus
2023207	block1	HumanSpeech	Pre	gau2	Object	Broad	r2	226.164220547275	1	broad_focus	2	Broad focus
2023207	block1	HumanSpeech	Pre	zai2	Object	Broad	r2	596.998926267588	2	broad_focus	2	Broad focus
2023207	block1	HumanSpeech	Pre	zoeng1	Subject	Narrow	r2	161.678278406328	1	on_focus	1	Narrow on_focus
2023207	block1	HumanSpeech	Pre	saang1	Subject	Narrow	r2	141.74745363897	2	on_focus	1	Narrow on_focus
2023207	block1	HumanSpeech	Pre	tsa1	Verb	Narrow	r2	164.036321445849	1	post_focus	1	Narrow post_focus
2023207	block1	HumanSpeech	Pre	fei1	Object	Narrow	r2	198.620265018462	1	post_focus	1	Narrow post_focus
2023207	block1	HumanSpeech	Pre	gei1	Object	Narrow	r2	413.626346371871	2	post_focus	1	Narrow post_focus
2023207	block1	HumanSpeech	Pre	sau3	Subject	Narrow	r2	199.137043634664	1	pre_focus	3	Narrow pre_focus
2023207	block1	HumanSpeech	Pre	sau3	Subject	Narrow	r2	174.833113946534	2	pre_focus	3	Narrow pre_focus
2023207	block1	HumanSpeech	Pre	sik3	Verb	Narrow	r2	142.336478734478	1	pre_focus	3	Narrow pre_focus
2023207	block1	HumanSpeech	Pre	baak3	Object	Narrow	r2	161.362982928949	1	on_focus	3	Narrow on_focus
2023207	block1	HumanSpeech	Pre	baak3	Object	Narrow	r2	370.873707355599	2	on_focus	3	Narrow on_focus
2023207	block1	HumanSpeech	Pre	sau3	Subject	Contrastive	r2	185.701632546397	1	pre_focus	3	Contrastive pre_focus
2023207	block1	HumanSpeech	Pre	sau3	Subject	Contrastive	r2	161.58591539704	2	pre_focus	3	Contrastive pre_focus
2023207	block1	HumanSpeech	Pre	sik3	Verb	Contrastive	r2	151.739761357362	1	on_focus	3	Contrastive on_focus
2023207	block1	HumanSpeech	Pre	baak3	Object	Contrastive	r2	162.615121459908	1	post_focus	3	Contrastive post_focus
2023207	block1	HumanSpeech	Pre	baak3	Object	Contrastive	r2	308.141921555602	2	post_focus	3	Contrastive post_focus
2023207	block1	HumanSpeech	Pre	zoeng1	Subject	Contrastive	r2	208.851586538799	1	pre_focus	1	Contrastive pre_focus
2023207	block1	HumanSpeech	Pre	saang1	Subject	Contrastive	r2	206.465522824601	2	pre_focus	1	Contrastive pre_focus
2023207	block1	HumanSpeech	Pre	tsa1	Verb	Contrastive	r2	187.989395613613	1	pre_focus	1	Contrastive pre_focus
2023207	block1	HumanSpeech	Pre	fei1	Object	Contrastive	r2	189.396768540291	1	on_focus	1	Contrastive on_focus
2023207	block1	HumanSpeech	Pre	gei1	Object	Contrastive	r2	379.170574238742	2	on_focus	1	Contrastive on_focus
2023207	block2	HumanSpeech	Post	ngaa5	Subject	Narrow	r1	119.915343915338	1	pre_focus	5	Narrow pre_focus
2023207	block2	HumanSpeech	Post	ngaa5	Subject	Narrow	r1	100.040627362063	2	pre_focus	5	Narrow pre_focus
2023207	block2	HumanSpeech	Post	maai5	Verb	Narrow	r1	251.082766439907	1	on_focus	5	Narrow on_focus
2023207	block2	HumanSpeech	Post	pou5	Object	Narrow	r1	158.425925925926	1	post_focus	5	Narrow post_focus
2023207	block2	HumanSpeech	Post	pou5	Object	Narrow	r1	172.099773242621	2	post_focus	5	Narrow post_focus
2023207	block2	HumanSpeech	Post	lok6	Subject	Contrastive	r1	131.30131969973	1	pre_focus	6	Contrastive pre_focus
2023207	block2	HumanSpeech	Post	lok6	Subject	Contrastive	r1	165.723588161526	2	pre_focus	6	Contrastive pre_focus
2023207	block2	HumanSpeech	Post	waa6	Verb	Contrastive	r1	181.732785273155	1	on_focus	6	Contrastive on_focus
2023207	block2	HumanSpeech	Post	jyut6	Object	Contrastive	r1	206.32304203734	1	post_focus	6	Contrastive post_focus
2023207	block2	HumanSpeech	Post	loeng6	Object	Contrastive	r1	187.607439801326	2	post_focus	6	Contrastive post_focus
2023207	block2	HumanSpeech	Post	ma4	Subject	Narrow	r1	145.291296005581	1	pre_focus	4	Narrow pre_focus
2023207	block2	HumanSpeech	Post	ma4	Subject	Narrow	r1	209.489292013103	2	pre_focus	4	Narrow pre_focus
2023207	block2	HumanSpeech	Post	fu4	Verb	Narrow	r1	218.133030990188	1	pre_focus	4	Narrow pre_focus
2023207	block2	HumanSpeech	Post	maang4	Object	Narrow	r1	209.054337784494	1	on_focus	4	Narrow on_focus
2023207	block2	HumanSpeech	Post	jan4	Object	Narrow	r1	328.231292517003	2	on_focus	4	Narrow on_focus
2023207	block2	HumanSpeech	Post	lok6	Subject	Contrastive	r1	129.95367670878	1	on_focus	6	Contrastive on_focus
2023207	block2	HumanSpeech	Post	lok6	Subject	Contrastive	r1	151.848805163098	2	on_focus	6	Contrastive on_focus
2023207	block2	HumanSpeech	Post	waa6	Verb	Contrastive	r1	180.111003131401	1	post_focus	6	Contrastive post_focus
2023207	block2	HumanSpeech	Post	jyut6	Object	Contrastive	r1	181.730158730161	1	post_focus	6	Contrastive post_focus

2023207	block2	HumanSpeech	Post	loeng6	Object	Contrastive	r1	360.727513227516	2	post_focus	6	Contrastive post_focus
2023207	block2	HumanSpeech	Post	ngaa5	Subject	Narrow	r1	175.435065026903	1	pre_focus	5	Narrow pre_focus
2023207	block2	HumanSpeech	Post	ngaa5	Subject	Narrow	r1	162.593270641594	2	pre_focus	5	Narrow pre_focus
2023207	block2	HumanSpeech	Post	maai5	Verb	Narrow	r1	170.565381708229	1	pre_focus	5	Narrow pre_focus
2023207	block2	HumanSpeech	Post	pou5	Object	Narrow	r1	97.0302343159517	1	on_focus	5	Narrow on_focus
2023207	block2	HumanSpeech	Post	pou5	Object	Narrow	r1	247.927437641721	2	on_focus	5	Narrow on_focus
2023207	block2	HumanSpeech	Post	ngaa5	Subject	Contrastive	r1	163.318336253127	1	on_focus	5	Contrastive on_focus
2023207	block2	HumanSpeech	Post	ngaa5	Subject	Contrastive	r1	136.413994169104	2	on_focus	5	Contrastive on_focus
2023207	block2	HumanSpeech	Post	maai5	Verb	Contrastive	r1	202.744254989156	1	post_focus	5	Contrastive post_focus
2023207	block2	HumanSpeech	Post	pou5	Object	Contrastive	r1	121.459601384402	1	post_focus	5	Contrastive post_focus
2023207	block2	HumanSpeech	Post	pou5	Object	Contrastive	r1	409.103576914646	2	post_focus	5	Contrastive post_focus
2023207	block2	HumanSpeech	Post	ngaa5	Subject	Narrow	r1	146.335514712703	1	on_focus	5	Narrow on_focus
2023207	block2	HumanSpeech	Post	ngaa5	Subject	Narrow	r1	92.4580498866305	2	on_focus	5	Narrow on_focus
2023207	block2	HumanSpeech	Post	maai5	Verb	Narrow	r1	184.240362811792	1	post_focus	5	Narrow post_focus
2023207	block2	HumanSpeech	Post	pou5	Object	Narrow	r1	78.0612244898009	1	post_focus	5	Narrow post_focus
2023207	block2	HumanSpeech	Post	pou5	Object	Narrow	r1	279.759700176371	2	post_focus	5	Narrow post_focus
2023207	block2	HumanSpeech	Post	ngaa5	Subject	Contrastive	r1	155.333778749593	1	pre_focus	5	Contrastive pre_focus
2023207	block2	HumanSpeech	Post	ngaa5	Subject	Contrastive	r1	103.862254607591	2	pre_focus	5	Contrastive pre_focus
2023207	block2	HumanSpeech	Post	maai5	Verb	Contrastive	r1	193.746729461026	1	pre_focus	5	Contrastive pre_focus
2023207	block2	HumanSpeech	Post	pou5	Object	Contrastive	r1	98.5028098195926	1	on_focus	5	Contrastive on_focus
2023207	block2	HumanSpeech	Post	pou5	Object	Contrastive	r1	169.654105028258	2	on_focus	5	Contrastive on_focus
2023207	block2	HumanSpeech	Post	ma4	Subject	Narrow	r1	128.858576935272	1	pre_focus	4	Narrow pre_focus
2023207	block2	HumanSpeech	Post	ma4	Subject	Narrow	r1	110.17391056842	2	pre_focus	4	Narrow pre_focus
2023207	block2	HumanSpeech	Post	fu4	Verb	Narrow	r1	143.330708826738	1	on_focus	4	Narrow on_focus
2023207	block2	HumanSpeech	Post	maang4	Object	Narrow	r1	179.599839935975	1	post_focus	4	Narrow post_focus
2023207	block2	HumanSpeech	Post	jan4	Object	Narrow	r1	164.263960326679	2	post_focus	4	Narrow post_focus
2023207	block2	HumanSpeech	Post	ngaa5	Subject	Contrastive	r1	161.706647571322	1	pre_focus	5	Contrastive pre_focus
2023207	block2	HumanSpeech	Post	ngaa5	Subject	Contrastive	r1	158.527298271082	2	pre_focus	5	Contrastive pre_focus
2023207	block2	HumanSpeech	Post	maai5	Verb	Contrastive	r1	187.798773830508	1	on_focus	5	Contrastive on_focus
2023207	block2	HumanSpeech	Post	pou5	Object	Contrastive	r1	82.0297349708881	1	post_focus	5	Contrastive post_focus
2023207	block2	HumanSpeech	Post	pou5	Object	Contrastive	r1	207.29952587098	2	post_focus	5	Contrastive post_focus
2023207	block2	HumanSpeech	Post	lok6	Subject	Narrow	r1	87.3500273672789	1	pre_focus	6	Narrow pre_focus
2023207	block2	HumanSpeech	Post	lok6	Subject	Narrow	r1	70.3955451497222	2	pre_focus	6	Narrow pre_focus
2023207	block2	HumanSpeech	Post	waa6	Verb	Narrow	r1	134.665767808627	1	pre_focus	6	Narrow pre_focus
2023207	block2	HumanSpeech	Post	jyut6	Object	Narrow	r1	124.404761904771	1	on_focus	6	Narrow on_focus
2023207	block2	HumanSpeech	Post	loeng6	Object	Narrow	r1	201.006841483036	2	on_focus	6	Narrow on_focus
2023207	block2	HumanSpeech	Post	ma4	Subject	Narrow	r1	115.772577146799	1	on_focus	4	Narrow on_focus
2023207	block2	HumanSpeech	Post	ma4	Subject	Narrow	r1	139.957168052405	2	on_focus	4	Narrow on_focus
2023207	block2	HumanSpeech	Post	fu4	Verb	Narrow	r1	138.163265306133	1	post_focus	4	Narrow post_focus
2023207	block2	HumanSpeech	Post	maang4	Object	Narrow	r1	77.162257495587	1	post_focus	4	Narrow post_focus
2023207	block2	HumanSpeech	Post	jan4	Object	Narrow	r1	108.605694129494	2	post_focus	4	Narrow post_focus
2023207	block2	HumanSpeech	Post	ma4	Subject	Contrastive	r1	142.229510851962	1	on_focus	4	Contrastive on_focus
2023207	block2	HumanSpeech	Post	ma4	Subject	Contrastive	r1	160.801079052334	2	on_focus	4	Contrastive on_focus
2023207	block2	HumanSpeech	Post	fu4	Verb	Contrastive	r1	73.2350718064936	1	post_focus	4	Contrastive post_focus
2023207	block2	HumanSpeech	Post	maang4	Object	Contrastive	r1	153.417172743929	1	post_focus	4	Contrastive post_focus
2023207	block2	HumanSpeech	Post	jan4	Object	Contrastive	r1	197.639879800164	2	post_focus	4	Contrastive post_focus
2023207	block2	HumanSpeech	Post	lok6	Subject	Narrow	r1	116.478647014361	1	on_focus	6	Narrow on_focus
2023207	block2	HumanSpeech	Post	lok6	Subject	Narrow	r1	188.188208616793	2	on_focus	6	Narrow on_focus
2023207	block2	HumanSpeech	Post	waa6	Verb	Narrow	r1	185.162234590791	1	post_focus	6	Narrow post_focus
2023207	block2	HumanSpeech	Post	jyut6	Object	Narrow	r1	119.179894179865	1	post_focus	6	Narrow post_focus
2023207	block2	HumanSpeech	Post	loeng6	Object	Narrow	r1	172.126984126976	2	post_focus	6	Narrow post_focus
2023207	block2	HumanSpeech	Post	ma4	Subject	Broad	r1	99.2625575482862	1	broad_focus	4	Broad focus
2023207	block2	HumanSpeech	Post	ma4	Subject	Broad	r1	115.978857270619	2	broad_focus	4	Broad focus
2023207	block2	HumanSpeech	Post	fu4	Verb	Broad	r1	90.4100529100731	1	broad_focus	4	Broad focus
2023207	block2	HumanSpeech	Post	maang4	Object	Broad	r1	189.834467120164	1	broad_focus	4	Broad focus
2023207	block2	HumanSpeech	Post	jan4	Object	Broad	r1	183.7603930461	2	broad_focus	4	Broad focus
2023207	block2	HumanSpeech	Post	ngaa5	Subject	Broad	r1	236.734693877537	1	broad_focus	5	Broad focus
2023207	block2	HumanSpeech	Post	ngaa5	Subject	Broad	r1	213.091458805735	2	broad_focus	5	Broad focus
2023207	block2	HumanSpeech	Post	maai5	Verb	Broad	r1	145.797430083121	1	broad_focus	5	Broad focus
2023207	block2	HumanSpeech	Post	pou5	Object	Broad	r1	147.016520894056	1	broad_focus	5	Broad focus
2023207	block2	HumanSpeech	Post	pou5	Object	Broad	r1	135.17460317459	2	broad_focus	5	Broad focus
2023207	block2	HumanSpeech	Post	lok6	Subject	Narrow	r1	87.8798185941037	1	pre_focus	6	Narrow pre_focus

**2023207**	block2	HumanSpeech	Post	lok6	Subject	Narrow	r1	91.2528344671273	2	pre_focus	6	Narrow pre_focus
**2023207**	block2	HumanSpeech	Post	waa6	Verb	Narrow	r1	144.31287792894	1	on_focus	6	Narrow on_focus
**2023207**	block2	HumanSpeech	Post	jyut6	Object	Narrow	r1	160.770975056693	1	post_focus	6	Narrow post_focus
**2023207**	block2	HumanSpeech	Post	loeng6	Object	Narrow	r1	232.448979591823	2	post_focus	6	Narrow post_focus
**2023207**	block2	HumanSpeech	Post	lok6	Subject	Contrastive	r1	116.353741496596	1	pre_focus	6	Contrastive pre_focus
**2023207**	block2	HumanSpeech	Post	lok6	Subject	Contrastive	r1	137.146744412064	2	pre_focus	6	Contrastive pre_focus
**2023207**	block2	HumanSpeech	Post	waa6	Verb	Contrastive	r1	160.606575963754	1	pre_focus	6	Contrastive pre_focus
**2023207**	block2	HumanSpeech	Post	jyut6	Object	Contrastive	r1	122.033730158734	1	on_focus	6	Contrastive on_focus
**2023207**	block2	HumanSpeech	Post	loeng6	Object	Contrastive	r1	280.673370797587	2	on_focus	6	Contrastive on_focus
**2023207**	block2	HumanSpeech	Post	lok6	Subject	Broad	r1	97.4603174603033	1	broad_focus	6	Broad focus
**2023207**	block2	HumanSpeech	Post	lok6	Subject	Broad	r1	164.854442681246	2	broad_focus	6	Broad focus
**2023207**	block2	HumanSpeech	Post	waa6	Verb	Broad	r1	143.718511083875	1	broad_focus	6	Broad focus
**2023207**	block2	HumanSpeech	Post	jyut6	Object	Broad	r1	187.104794298676	1	broad_focus	6	Broad focus
**2023207**	block2	HumanSpeech	Post	loeng6	Object	Broad	r1	356.216264283489	2	broad_focus	6	Broad focus
**2023207**	block2	HumanSpeech	Post	ma4	Subject	Contrastive	r1	127.185420238561	1	pre_focus	4	Contrastive pre_focus
**2023207**	block2	HumanSpeech	Post	ma4	Subject	Contrastive	r1	75.255102040785	2	pre_focus	4	Contrastive pre_focus
**2023207**	block2	HumanSpeech	Post	fu4	Verb	Contrastive	r1	180.570042831931	1	pre_focus	4	Contrastive pre_focus
**2023207**	block2	HumanSpeech	Post	maang4	Object	Contrastive	r1	222.569573283835	1	on_focus	4	Contrastive on_focus
**2023207**	block2	HumanSpeech	Post	jan4	Object	Contrastive	r1	275.679624230662	2	on_focus	4	Contrastive on_focus
**2023207**	block2	HumanSpeech	Post	ma4	Subject	Contrastive	r1	135.943472627133	1	pre_focus	4	Contrastive pre_focus
**2023207**	block2	HumanSpeech	Post	ma4	Subject	Contrastive	r1	84.1298185941071	2	pre_focus	4	Contrastive pre_focus
**2023207**	block2	HumanSpeech	Post	fu4	Verb	Contrastive	r1	163.558201058208	1	on_focus	4	Contrastive on_focus
**2023207**	block2	HumanSpeech	Post	maang4	Object	Contrastive	r1	196.892471274225	1	post_focus	4	Contrastive post_focus
**2023207**	block2	HumanSpeech	Post	jan4	Object	Contrastive	r1	262.19605791033	2	post_focus	4	Contrastive post_focus
**2023207**	block2	HumanSpeech	Post	ngaa5	Subject	Narrow	r2	154.507921082285	1	pre_focus	5	Narrow pre_focus
**2023207**	block2	HumanSpeech	Post	ngaa5	Subject	Narrow	r2	155.618835383081	2	pre_focus	5	Narrow pre_focus
**2023207**	block2	HumanSpeech	Post	maai5	Verb	Narrow	r2	215.472411186681	1	pre_focus	5	Narrow pre_focus
**2023207**	block2	HumanSpeech	Post	pou5	Object	Narrow	r2	146.591080876817	1	on_focus	5	Narrow on_focus
**2023207**	block2	HumanSpeech	Post	pou5	Object	Narrow	r2	299.540438397571	2	on_focus	5	Narrow on_focus
**2023207**	block2	HumanSpeech	Post	ngaa5	Subject	Contrastive	r2	105.208848107992	1	on_focus	5	Contrastive on_focus
**2023207**	block2	HumanSpeech	Post	ngaa5	Subject	Contrastive	r2	126.454192066461	2	on_focus	5	Contrastive on_focus
**2023207**	block2	HumanSpeech	Post	maai5	Verb	Contrastive	r2	265.362085004938	1	post_focus	5	Contrastive post_focus
**2023207**	block2	HumanSpeech	Post	pou5	Object	Contrastive	r2	207.046485260747	1	post_focus	5	Contrastive post_focus
**2023207**	block2	HumanSpeech	Post	pou5	Object	Contrastive	r2	280.338435374176	2	post_focus	5	Contrastive post_focus
**2023207**	block2	HumanSpeech	Post	ma4	Subject	Narrow	r2	137.991307634138	1	pre_focus	4	Narrow pre_focus
**2023207**	block2	HumanSpeech	Post	ma4	Subject	Narrow	r2	153.814652845256	2	pre_focus	4	Narrow pre_focus
**2023207**	block2	HumanSpeech	Post	fu4	Verb	Narrow	r2	102.388079041134	1	on_focus	4	Narrow on_focus
**2023207**	block2	HumanSpeech	Post	maang4	Object	Narrow	r2	266.325004360738	1	post_focus	4	Narrow post_focus
**2023207**	block2	HumanSpeech	Post	jan4	Object	Narrow	r2	179.565381708244	2	post_focus	4	Narrow post_focus
**2023207**	block2	HumanSpeech	Post	lok6	Subject	Contrastive	r2	162.895205701318	1	pre_focus	6	Contrastive pre_focus
**2023207**	block2	HumanSpeech	Post	lok6	Subject	Contrastive	r2	237.760334903186	2	pre_focus	6	Contrastive pre_focus
**2023207**	block2	HumanSpeech	Post	waa6	Verb	Contrastive	r2	152.244897959207	1	on_focus	6	Contrastive on_focus
**2023207**	block2	HumanSpeech	Post	jyut6	Object	Contrastive	r2	89.2498110355291	1	post_focus	6	Contrastive post_focus
**2023207**	block2	HumanSpeech	Post	loeng6	Object	Contrastive	r2	150.360839988167	2	post_focus	6	Contrastive post_focus
**2023207**	block2	HumanSpeech	Post	ngaa5	Subject	Narrow	r2	106.151171579739	1	pre_focus	5	Narrow pre_focus
**2023207**	block2	HumanSpeech	Post	ngaa5	Subject	Narrow	r2	169.836950653263	2	pre_focus	5	Narrow pre_focus
**2023207**	block2	HumanSpeech	Post	maai5	Verb	Narrow	r2	149.213151927455	1	on_focus	5	Narrow on_focus
**2023207**	block2	HumanSpeech	Post	pou5	Object	Narrow	r2	182.403628117925	1	post_focus	5	Narrow post_focus
**2023207**	block2	HumanSpeech	Post	pou5	Object	Narrow	r2	203.395691609956	2	post_focus	5	Narrow post_focus
**2023207**	block2	HumanSpeech	Post	ngaa5	Subject	Contrastive	r2	164.64404076649	1	pre_focus	5	Contrastive pre_focus
**2023207**	block2	HumanSpeech	Post	ngaa5	Subject	Contrastive	r2	101.384894867039	2	pre_focus	5	Contrastive pre_focus
**2023207**	block2	HumanSpeech	Post	maai5	Verb	Contrastive	r2	306.758629377697	1	on_focus	5	Contrastive on_focus
**2023207**	block2	HumanSpeech	Post	pou5	Object	Contrastive	r2	181.126228269079	1	post_focus	5	Contrastive post_focus
**2023207**	block2	HumanSpeech	Post	pou5	Object	Contrastive	r2	183.036281179113	2	post_focus	5	Contrastive post_focus
**2023207**	block2	HumanSpeech	Post	ma4	Subject	Narrow	r2	151.867508908339	1	on_focus	4	Narrow on_focus
**2023207**	block2	HumanSpeech	Post	ma4	Subject	Narrow	r2	162.940287225979	2	on_focus	4	Narrow on_focus
**2023207**	block2	HumanSpeech	Post	fu4	Verb	Narrow	r2	134.148585750069	1	post_focus	4	Narrow post_focus
**2023207**	block2	HumanSpeech	Post	maang4	Object	Narrow	r2	167.052802073215	1	post_focus	4	Narrow post_focus
**2023207**	block2	HumanSpeech	Post	jan4	Object	Narrow	r2	212.111758989323	2	post_focus	4	Narrow post_focus
**2023207**	block2	HumanSpeech	Post	ma4	Subject	Narrow	r2	73.6079617031749	1	pre_focus	4	Narrow pre_focus
**2023207**	block2	HumanSpeech	Post	ma4	Subject	Narrow	r2	63.3560090703043	2	pre_focus	4	Narrow pre_focus
**2023207**	block2	HumanSpeech	Post	fu4	Verb	Narrow	r2	124.004157218451	1	pre_focus	4	Narrow pre_focus

**2023207**	block2	HumanSpeech	Post	maang4	Object	Narrow	r2	137.781732848168	1	on_focus	4	Narrow on_focus
**2023207**	block2	HumanSpeech	Post	jan4	Object	Narrow	r2	185.635028614655	2	on_focus	4	Narrow on_focus
**2023207**	block2	HumanSpeech	Post	lok6	Subject	Narrow	r2	127.32237339381	1	pre_focus	6	Narrow pre_focus
**2023207**	block2	HumanSpeech	Post	lok6	Subject	Narrow	r2	159.478458049875	2	pre_focus	6	Narrow pre_focus
**2023207**	block2	HumanSpeech	Post	waa6	Verb	Narrow	r2	170.598433312705	1	on_focus	6	Narrow on_focus
**2023207**	block2	HumanSpeech	Post	jyut6	Object	Narrow	r2	82.7210884353633	1	post_focus	6	Narrow post_focus
**2023207**	block2	HumanSpeech	Post	loeng6	Object	Narrow	r2	117.416855631149	2	post_focus	6	Narrow post_focus
**2023207**	block2	HumanSpeech	Post	lok6	Subject	Narrow	r2	97.2826908541151	1	on_focus	6	Narrow on_focus
**2023207**	block2	HumanSpeech	Post	lok6	Subject	Narrow	r2	120.622323003289	2	on_focus	6	Narrow on_focus
**2023207**	block2	HumanSpeech	Post	waa6	Verb	Narrow	r2	117.622826908558	1	post_focus	6	Narrow post_focus
**2023207**	block2	HumanSpeech	Post	jyut6	Object	Narrow	r2	94.8091458805891	1	post_focus	6	Narrow post_focus
**2023207**	block2	HumanSpeech	Post	loeng6	Object	Narrow	r2	173.645124716558	2	post_focus	6	Narrow post_focus
**2023207**	block2	HumanSpeech	Post	lok6	Subject	Narrow	r2	82.4303957171821	1	pre_focus	6	Narrow pre_focus
**2023207**	block2	HumanSpeech	Post	lok6	Subject	Narrow	r2	82.3234811165889	2	pre_focus	6	Narrow pre_focus
**2023207**	block2	HumanSpeech	Post	waa6	Verb	Narrow	r2	154.07772304323	1	pre_focus	6	Narrow pre_focus
**2023207**	block2	HumanSpeech	Post	jyut6	Object	Narrow	r2	114.678760393048	1	on_focus	6	Narrow on_focus
**2023207**	block2	HumanSpeech	Post	loeng6	Object	Narrow	r2	152.503401360548	2	on_focus	6	Narrow on_focus
**2023207**	block2	HumanSpeech	Post	ma4	Subject	Contrastive	r2	142.991135848291	1	pre_focus	4	Contrastive pre_focus
**2023207**	block2	HumanSpeech	Post	ma4	Subject	Contrastive	r2	87.8130511463553	2	pre_focus	4	Contrastive pre_focus
**2023207**	block2	HumanSpeech	Post	fu4	Verb	Contrastive	r2	103.203757693535	1	pre_focus	4	Contrastive pre_focus
**2023207**	block2	HumanSpeech	Post	maang4	Object	Contrastive	r2	152.233216518937	1	on_focus	4	Contrastive on_focus
**2023207**	block2	HumanSpeech	Post	jan4	Object	Contrastive	r2	211.725875535393	2	on_focus	4	Contrastive on_focus
**2023207**	block2	HumanSpeech	Post	lok6	Subject	Contrastive	r2	81.2747003563175	1	pre_focus	6	Contrastive pre_focus
**2023207**	block2	HumanSpeech	Post	lok6	Subject	Contrastive	r2	180.41246128081	2	pre_focus	6	Contrastive pre_focus
**2023207**	block2	HumanSpeech	Post	waa6	Verb	Contrastive	r2	145.518032609857	1	pre_focus	6	Contrastive pre_focus
**2023207**	block2	HumanSpeech	Post	jyut6	Object	Contrastive	r2	109.653992549397	1	on_focus	6	Contrastive on_focus
**2023207**	block2	HumanSpeech	Post	loeng6	Object	Contrastive	r2	197.864701436146	2	on_focus	6	Contrastive on_focus
**2023207**	block2	HumanSpeech	Post	ma4	Subject	Contrastive	r2	146.931846812805	1	pre_focus	4	Contrastive pre_focus
**2023207**	block2	HumanSpeech	Post	ma4	Subject	Contrastive	r2	146.624149659857	2	pre_focus	4	Contrastive pre_focus
**2023207**	block2	HumanSpeech	Post	fu4	Verb	Contrastive	r2	78.8072221370157	1	on_focus	4	Contrastive on_focus
**2023207**	block2	HumanSpeech	Post	maang4	Object	Contrastive	r2	218.580498866231	1	post_focus	4	Contrastive post_focus
**2023207**	block2	HumanSpeech	Post	jan4	Object	Contrastive	r2	237.494870964213	2	post_focus	4	Contrastive post_focus
**2023207**	block2	HumanSpeech	Post	ngaa5	Subject	Narrow	r2	223.734638546262	1	on_focus	5	Narrow on_focus
**2023207**	block2	HumanSpeech	Post	ngaa5	Subject	Narrow	r2	132.112390509633	2	on_focus	5	Narrow on_focus
**2023207**	block2	HumanSpeech	Post	maai5	Verb	Narrow	r2	236.272539258948	1	post_focus	5	Narrow post_focus
**2023207**	block2	HumanSpeech	Post	pou5	Object	Narrow	r2	218.619311786199	1	post_focus	5	Narrow post_focus
**2023207**	block2	HumanSpeech	Post	pou5	Object	Narrow	r2	384.203901715694	2	post_focus	5	Narrow post_focus
**2023207**	block2	HumanSpeech	Post	ma4	Subject	Contrastive	r2	199.711907911365	1	on_focus	4	Contrastive on_focus
**2023207**	block2	HumanSpeech	Post	ma4	Subject	Contrastive	r2	198.069211675318	2	on_focus	4	Contrastive on_focus
**2023207**	block2	HumanSpeech	Post	fu4	Verb	Contrastive	r2	129.968865933051	1	post_focus	4	Contrastive post_focus
**2023207**	block2	HumanSpeech	Post	maang4	Object	Contrastive	r2	252.531853872995	1	post_focus	4	Contrastive post_focus
**2023207**	block2	HumanSpeech	Post	jan4	Object	Contrastive	r2	427.839412867911	2	post_focus	4	Contrastive post_focus
**2023207**	block2	HumanSpeech	Post	lok6	Subject	Contrastive	r2	198.090382231214	1	on_focus	6	Contrastive on_focus
**2023207**	block2	HumanSpeech	Post	lok6	Subject	Contrastive	r2	255.974310232432	2	on_focus	6	Contrastive on_focus
**2023207**	block2	HumanSpeech	Post	waa6	Verb	Contrastive	r2	227.388052044716	1	post_focus	6	Contrastive post_focus
**2023207**	block2	HumanSpeech	Post	jyut6	Object	Contrastive	r2	139.473658576662	1	post_focus	6	Contrastive post_focus
**2023207**	block2	HumanSpeech	Post	loeng6	Object	Contrastive	r2	498.824724107862	2	post_focus	6	Contrastive post_focus
**2023207**	block2	HumanSpeech	Post	ma4	Subject	Broad	r2	199.771144227043	1	broad_focus	4	Broad focus
**2023207**	block2	HumanSpeech	Post	ma4	Subject	Broad	r2	205.472482917912	2	broad_focus	4	Broad focus
**2023207**	block2	HumanSpeech	Post	fu4	Verb	Broad	r2	97.3276598089114	1	broad_focus	4	Broad focus
**2023207**	block2	HumanSpeech	Post	maang4	Object	Broad	r2	274.996585424958	1	broad_focus	4	Broad focus
**2023207**	block2	HumanSpeech	Post	jan4	Object	Broad	r2	605.507366668064	2	broad_focus	4	Broad focus
**2023207**	block2	HumanSpeech	Post	ngaa5	Subject	Contrastive	r2	132.854362275452	1	pre_focus	5	Contrastive pre_focus
**2023207**	block2	HumanSpeech	Post	ngaa5	Subject	Contrastive	r2	282.024485948909	2	pre_focus	5	Contrastive pre_focus
**2023207**	block2	HumanSpeech	Post	maai5	Verb	Contrastive	r2	186.098700496814	1	pre_focus	5	Contrastive pre_focus
**2023207**	block2	HumanSpeech	Post	pou5	Object	Contrastive	r2	189.280225448442	1	on_focus	5	Contrastive on_focus
**2023207**	block2	HumanSpeech	Post	pou5	Object	Contrastive	r2	531.703249981263	2	on_focus	5	Contrastive on_focus
**2023207**	block2	HumanSpeech	Post	lok6	Subject	Broad	r2	141.935022161761	1	broad_focus	6	Broad focus
**2023207**	block2	HumanSpeech	Post	lok6	Subject	Broad	r2	195.667623058284	2	broad_focus	6	Broad focus
**2023207**	block2	HumanSpeech	Post	waa6	Verb	Broad	r2	181.01134940656	1	broad_focus	6	Broad focus
**2023207**	block2	HumanSpeech	Post	jyut6	Object	Broad	r2	169.272544195621	1	broad_focus	6	Broad focus
**2023207**	block2	HumanSpeech	Post	loeng6	Object	Broad	r2	463.792129350736	2	broad_focus	6	Broad focus

2023207	block2	HumanSpeech	Post	ngaa5	Subject	Broad	r2	113.077648492947	1	broad_focus	5	Broad focus
2023207	block2	HumanSpeech	Post	ngaa5	Subject	Broad	r2	144.197544991698	2	broad_focus	5	Broad focus
2023207	block2	HumanSpeech	Post	maai5	Verb	Broad	r2	243.275530348512	1	broad_focus	5	Broad focus
2023207	block2	HumanSpeech	Post	pou5	Object	Broad	r2	159.759742280755	1	broad_focus	5	Broad focus
2023207	block2	HumanSpeech	Post	pou5	Object	Broad	r2	535.278354279569	2	broad_focus	5	Broad focus
2023207	block2	HumanSpeech	Pre	ngaa5	Subject	Contrastive	r1	193.049857172298	1	pre_focus	5	Contrastive pre_focus
2023207	block2	HumanSpeech	Pre	ngaa5	Subject	Contrastive	r1	174.872094292283	2	pre_focus	5	Contrastive pre_focus
2023207	block2	HumanSpeech	Pre	maai5	Verb	Contrastive	r1	314.227387251208	1	on_focus	5	Contrastive on_focus
2023207	block2	HumanSpeech	Pre	pou5	Object	Contrastive	r1	193.046107331838	1	post_focus	5	Contrastive post_focus
2023207	block2	HumanSpeech	Pre	pou5	Object	Contrastive	r1	267.821617535901	2	post_focus	5	Contrastive post_focus
2023207	block2	HumanSpeech	Pre	ma4	Subject	Narrow	r1	143.739682539689	1	on_focus	4	Narrow on_focus
2023207	block2	HumanSpeech	Pre	ma4	Subject	Narrow	r1	237.593174021754	2	on_focus	4	Narrow on_focus
2023207	block2	HumanSpeech	Pre	fu4	Verb	Narrow	r1	128.875977823327	1	post_focus	4	Narrow post_focus
2023207	block2	HumanSpeech	Pre	maang4	Object	Narrow	r1	277.935341755722	1	post_focus	4	Narrow post_focus
2023207	block2	HumanSpeech	Pre	jan4	Object	Narrow	r1	266.866024187465	2	post_focus	4	Narrow post_focus
2023207	block2	HumanSpeech	Pre	lok6	Subject	Narrow	r1	107.310176595888	1	pre_focus	6	Narrow pre_focus
2023207	block2	HumanSpeech	Pre	lok6	Subject	Narrow	r1	152.165285807627	2	pre_focus	6	Narrow pre_focus
2023207	block2	HumanSpeech	Pre	waa6	Verb	Narrow	r1	184.329176114886	1	on_focus	6	Narrow on_focus
2023207	block2	HumanSpeech	Pre	jyut6	Object	Narrow	r1	127.328634588054	1	post_focus	6	Narrow post_focus
2023207	block2	HumanSpeech	Pre	loeng6	Object	Narrow	r1	358.37468320662	2	post_focus	6	Narrow post_focus
2023207	block2	HumanSpeech	Pre	ngaa5	Subject	Narrow	r1	176.998275093496	1	pre_focus	5	Narrow pre_focus
2023207	block2	HumanSpeech	Pre	ngaa5	Subject	Narrow	r1	151.935086203821	2	pre_focus	5	Narrow pre_focus
2023207	block2	HumanSpeech	Pre	maai5	Verb	Narrow	r1	245.034013605448	1	pre_focus	5	Narrow pre_focus
2023207	block2	HumanSpeech	Pre	pou5	Object	Narrow	r1	208.771982867233	1	on_focus	5	Narrow on_focus
2023207	block2	HumanSpeech	Pre	pou5	Object	Narrow	r1	354.993197278901	2	on_focus	5	Narrow on_focus
2023207	block2	HumanSpeech	Pre	lok6	Subject	Contrastive	r1	122.175453514757	1	pre_focus	6	Contrastive pre_focus
2023207	block2	HumanSpeech	Pre	lok6	Subject	Contrastive	r1	139.539007092225	2	pre_focus	6	Contrastive pre_focus
2023207	block2	HumanSpeech	Pre	waa6	Verb	Contrastive	r1	147.142857142853	1	pre_focus	6	Contrastive pre_focus
2023207	block2	HumanSpeech	Pre	jyut6	Object	Contrastive	r1	106.456916099773	1	on_focus	6	Contrastive on_focus
2023207	block2	HumanSpeech	Pre	loeng6	Object	Contrastive	r1	412.131519274368	2	on_focus	6	Contrastive on_focus
2023207	block2	HumanSpeech	Pre	ngaa5	Subject	Narrow	r1	154.590466223112	1	pre_focus	5	Narrow pre_focus
2023207	block2	HumanSpeech	Pre	ngaa5	Subject	Narrow	r1	136.617146716844	2	pre_focus	5	Narrow pre_focus
2023207	block2	HumanSpeech	Pre	maai5	Verb	Narrow	r1	267.993197278912	1	on_focus	5	Narrow on_focus
2023207	block2	HumanSpeech	Pre	pou5	Object	Narrow	r1	131.93184681279	1	post_focus	5	Narrow post_focus
2023207	block2	HumanSpeech	Pre	pou5	Object	Narrow	r1	331.796616082357	2	post_focus	5	Narrow post_focus
2023207	block2	HumanSpeech	Pre	ngaa5	Subject	Contrastive	r1	156.039413211005	1	on_focus	5	Contrastive on_focus
2023207	block2	HumanSpeech	Pre	ngaa5	Subject	Contrastive	r1	190.034300294457	2	on_focus	5	Contrastive on_focus
2023207	block2	HumanSpeech	Pre	maai5	Verb	Contrastive	r1	259.482459650542	1	post_focus	5	Contrastive post_focus
2023207	block2	HumanSpeech	Pre	pou5	Object	Contrastive	r1	219.243278263662	1	post_focus	5	Contrastive post_focus
2023207	block2	HumanSpeech	Pre	pou5	Object	Contrastive	r1	314.827264239028	2	post_focus	5	Contrastive post_focus
2023207	block2	HumanSpeech	Pre	lok6	Subject	Broad	r1	133.088159426023	1	broad_focus	6	Broad focus
2023207	block2	HumanSpeech	Pre	lok6	Subject	Broad	r1	228.507630847531	2	broad_focus	6	Broad focus
2023207	block2	HumanSpeech	Pre	waa6	Verb	Broad	r1	130.84759156186	1	broad_focus	6	Broad focus
2023207	block2	HumanSpeech	Pre	jyut6	Object	Broad	r1	151.313114134553	1	broad_focus	6	Broad focus
2023207	block2	HumanSpeech	Pre	loeng6	Object	Broad	r1	238.548752834475	2	broad_focus	6	Broad focus
2023207	block2	HumanSpeech	Pre	ma4	Subject	Contrastive	r1	198.308216428472	1	pre_focus	4	Contrastive pre_focus
2023207	block2	HumanSpeech	Pre	ma4	Subject	Contrastive	r1	254.076547790874	2	pre_focus	4	Contrastive pre_focus
2023207	block2	HumanSpeech	Pre	fu4	Verb	Contrastive	r1	143.418997228537	1	pre_focus	4	Contrastive pre_focus
2023207	block2	HumanSpeech	Pre	maang4	Object	Contrastive	r1	262.582828003019	1	on_focus	4	Contrastive on_focus
2023207	block2	HumanSpeech	Pre	jan4	Object	Contrastive	r1	226.85324129651	2	on_focus	4	Contrastive on_focus
2023207	block2	HumanSpeech	Pre	lok6	Subject	Contrastive	r1	128.112760255647	1	on_focus	6	Contrastive on_focus
2023207	block2	HumanSpeech	Pre	lok6	Subject	Contrastive	r1	181.863132888907	2	on_focus	6	Contrastive on_focus
2023207	block2	HumanSpeech	Pre	waa6	Verb	Contrastive	r1	164.795918367361	1	post_focus	6	Contrastive post_focus
2023207	block2	HumanSpeech	Pre	jyut6	Object	Contrastive	r1	191.057365253812	1	post_focus	6	Contrastive post_focus
2023207	block2	HumanSpeech	Pre	loeng6	Object	Contrastive	r1	329.614512471665	2	post_focus	6	Contrastive post_focus
2023207	block2	HumanSpeech	Pre	ma4	Subject	Broad	r1	150.441343203966	1	broad_focus	4	Broad focus
2023207	block2	HumanSpeech	Pre	ma4	Subject	Broad	r1	284.734164777035	2	broad_focus	4	Broad focus
2023207	block2	HumanSpeech	Pre	fu4	Verb	Broad	r1	163.691232048393	1	broad_focus	4	Broad focus
2023207	block2	HumanSpeech	Pre	maang4	Object	Broad	r1	286.50912996784	1	broad_focus	4	Broad focus
2023207	block2	HumanSpeech	Pre	jan4	Object	Broad	r1	265.135226577399	2	broad_focus	4	Broad focus
2023207	block2	HumanSpeech	Pre	ngaa5	Subject	Contrastive	r1	186.815486688488	1	pre_focus	5	Contrastive pre_focus
2023207	block2	HumanSpeech	Pre	ngaa5	Subject	Contrastive	r1	250.122017060733	2	pre_focus	5	Contrastive pre_focus

**2023207**	block2	HumanSpeech	Pre	maai5	Verb	Contrastive	r1	321.198506069095	1	pre_focus	5	Contrastive pre_focus
**2023207**	block2	HumanSpeech	Pre	pou5	Object	Contrastive	r1	164.541761148882	1	on_focus	5	Contrastive on_focus
**2023207**	block2	HumanSpeech	Pre	pou5	Object	Contrastive	r1	224.618893011723	2	on_focus	5	Contrastive on_focus
**2023207**	block2	HumanSpeech	Pre	ma4	Subject	Contrastive	r1	129.735591206213	1	pre_focus	4	Contrastive pre_focus
**2023207**	block2	HumanSpeech	Pre	ma4	Subject	Contrastive	r1	197.507663144279	2	pre_focus	4	Contrastive pre_focus
**2023207**	block2	HumanSpeech	Pre	fu4	Verb	Contrastive	r1	148.411504952833	1	on_focus	4	Contrastive on_focus
**2023207**	block2	HumanSpeech	Pre	maang4	Object	Contrastive	r1	246.63228212097	1	post_focus	4	Contrastive post_focus
**2023207**	block2	HumanSpeech	Pre	jan4	Object	Contrastive	r1	269.201814058931	2	post_focus	4	Contrastive post_focus
**2023207**	block2	HumanSpeech	Pre	ngaa5	Subject	Narrow	r1	236.196153320748	1	on_focus	5	Narrow on_focus
**2023207**	block2	HumanSpeech	Pre	ngaa5	Subject	Narrow	r1	224.809968284092	2	on_focus	5	Narrow on_focus
**2023207**	block2	HumanSpeech	Pre	maai5	Verb	Narrow	r1	378.949556430769	1	post_focus	5	Narrow post_focus
**2023207**	block2	HumanSpeech	Pre	pou5	Object	Narrow	r1	137.791320231827	1	post_focus	5	Narrow post_focus
**2023207**	block2	HumanSpeech	Pre	pou5	Object	Narrow	r1	184.00874635563	2	post_focus	5	Narrow post_focus
**2023207**	block2	HumanSpeech	Pre	ma4	Subject	Narrow	r1	141.762627756407	1	pre_focus	4	Narrow pre_focus
**2023207**	block2	HumanSpeech	Pre	ma4	Subject	Narrow	r1	294.19522729728	2	pre_focus	4	Narrow pre_focus
**2023207**	block2	HumanSpeech	Pre	fu4	Verb	Narrow	r1	112.640005497099	1	pre_focus	4	Narrow pre_focus
**2023207**	block2	HumanSpeech	Pre	maang4	Object	Narrow	r1	248.40939884723	1	on_focus	4	Narrow on_focus
**2023207**	block2	HumanSpeech	Pre	jan4	Object	Narrow	r1	279.461380258738	2	on_focus	4	Narrow on_focus
**2023207**	block2	HumanSpeech	Pre	lok6	Subject	Narrow	r1	97.184523809517	1	pre_focus	6	Narrow pre_focus
**2023207**	block2	HumanSpeech	Pre	lok6	Subject	Narrow	r1	162.319576719597	2	pre_focus	6	Narrow pre_focus
**2023207**	block2	HumanSpeech	Pre	waa6	Verb	Narrow	r1	152.449126836871	1	pre_focus	6	Narrow pre_focus
**2023207**	block2	HumanSpeech	Pre	jyut6	Object	Narrow	r1	136.573048266882	1	on_focus	6	Narrow on_focus
**2023207**	block2	HumanSpeech	Pre	loeng6	Object	Narrow	r1	196.353674803277	2	on_focus	6	Narrow on_focus
**2023207**	block2	HumanSpeech	Pre	lok6	Subject	Contrastive	r1	148.078529657482	1	pre_focus	6	Contrastive pre_focus
**2023207**	block2	HumanSpeech	Pre	lok6	Subject	Contrastive	r1	182.100418871244	2	pre_focus	6	Contrastive pre_focus
**2023207**	block2	HumanSpeech	Pre	waa6	Verb	Contrastive	r1	146.384164776975	1	on_focus	6	Contrastive on_focus
**2023207**	block2	HumanSpeech	Pre	jyut6	Object	Contrastive	r1	151.633156966454	1	post_focus	6	Contrastive post_focus
**2023207**	block2	HumanSpeech	Pre	loeng6	Object	Contrastive	r1	325.940316297476	2	post_focus	6	Contrastive post_focus
**2023207**	block2	HumanSpeech	Pre	ngaa5	Subject	Broad	r1	228.57774220796	1	broad_focus	5	Broad focus
**2023207**	block2	HumanSpeech	Pre	ngaa5	Subject	Broad	r1	259.811707398967	2	broad_focus	5	Broad focus
**2023207**	block2	HumanSpeech	Pre	maai5	Verb	Broad	r1	210.847100745127	1	broad_focus	5	Broad focus
**2023207**	block2	HumanSpeech	Pre	pou5	Object	Broad	r1	207.530103090619	1	broad_focus	5	Broad focus
**2023207**	block2	HumanSpeech	Pre	pou5	Object	Broad	r1	331.747165532931	2	broad_focus	5	Broad focus
**2023207**	block2	HumanSpeech	Pre	lok6	Subject	Narrow	r1	117.502309565793	1	on_focus	6	Narrow on_focus
**2023207**	block2	HumanSpeech	Pre	lok6	Subject	Narrow	r1	149.324248217795	2	on_focus	6	Narrow on_focus
**2023207**	block2	HumanSpeech	Pre	waa6	Verb	Narrow	r1	155.111377884452	1	post_focus	6	Narrow post_focus
**2023207**	block2	HumanSpeech	Pre	jyut6	Object	Narrow	r1	164.289715886355	1	post_focus	6	Narrow post_focus
**2023207**	block2	HumanSpeech	Pre	loeng6	Object	Narrow	r1	296.294115936973	2	post_focus	6	Narrow post_focus
**2023207**	block2	HumanSpeech	Pre	ma4	Subject	Contrastive	r1	127.685296340815	1	on_focus	4	Contrastive on_focus
**2023207**	block2	HumanSpeech	Pre	ma4	Subject	Contrastive	r1	158.403443009831	2	on_focus	4	Contrastive on_focus
**2023207**	block2	HumanSpeech	Pre	fu4	Verb	Contrastive	r1	78.4626881055601	1	post_focus	4	Contrastive post_focus
**2023207**	block2	HumanSpeech	Pre	maang4	Object	Contrastive	r1	198.070249376315	1	post_focus	4	Contrastive post_focus
**2023207**	block2	HumanSpeech	Pre	jan4	Object	Contrastive	r1	485.368084417132	2	post_focus	4	Contrastive post_focus
**2023207**	block2	HumanSpeech	Pre	ma4	Subject	Narrow	r1	99.5430397267114	1	pre_focus	4	Narrow pre_focus
**2023207**	block2	HumanSpeech	Pre	ma4	Subject	Narrow	r1	133.111163845399	2	pre_focus	4	Narrow pre_focus
**2023207**	block2	HumanSpeech	Pre	fu4	Verb	Narrow	r1	83.7938806599254	1	on_focus	4	Narrow on_focus
**2023207**	block2	HumanSpeech	Pre	maang4	Object	Narrow	r1	236.547112892822	1	post_focus	4	Narrow post_focus
**2023207**	block2	HumanSpeech	Pre	jan4	Object	Narrow	r1	390.792711370295	2	post_focus	4	Narrow post_focus
**2023207**	block2	HumanSpeech	Pre	ngaa5	Subject	Narrow	r2	160.78840319085	1	pre_focus	5	Narrow pre_focus
**2023207**	block2	HumanSpeech	Pre	ngaa5	Subject	Narrow	r2	138.046813963683	2	pre_focus	5	Narrow pre_focus
**2023207**	block2	HumanSpeech	Pre	maai5	Verb	Narrow	r2	316.722325293767	1	on_focus	5	Narrow on_focus
**2023207**	block2	HumanSpeech	Pre	pou5	Object	Narrow	r2	201.492219876457	1	post_focus	5	Narrow post_focus
**2023207**	block2	HumanSpeech	Pre	pou5	Object	Narrow	r2	299.555555555571	2	post_focus	5	Narrow post_focus
**2023207**	block2	HumanSpeech	Pre	ngaa5	Subject	Contrastive	r2	203.216941162452	1	on_focus	5	Contrastive on_focus
**2023207**	block2	HumanSpeech	Pre	ngaa5	Subject	Contrastive	r2	138.567520849961	2	on_focus	5	Contrastive on_focus
**2023207**	block2	HumanSpeech	Pre	maai5	Verb	Contrastive	r2	277.905889628585	1	post_focus	5	Contrastive post_focus
**2023207**	block2	HumanSpeech	Pre	pou5	Object	Contrastive	r2	179.568081200728	1	post_focus	5	Contrastive post_focus
**2023207**	block2	HumanSpeech	Pre	pou5	Object	Contrastive	r2	494.867346938804	2	post_focus	5	Contrastive post_focus
**2023207**	block2	HumanSpeech	Pre	ngaa5	Subject	Contrastive	r2	157.772544915417	1	pre_focus	5	Contrastive pre_focus
**2023207**	block2	HumanSpeech	Pre	ngaa5	Subject	Contrastive	r2	145.492337285816	2	pre_focus	5	Contrastive pre_focus
**2023207**	block2	HumanSpeech	Pre	maai5	Verb	Contrastive	r2	315.073608930732	1	on_focus	5	Contrastive on_focus
**2023207**	block2	HumanSpeech	Pre	pou5	Object	Contrastive	r2	194.111695183153	1	post_focus	5	Contrastive post_focus

2023207	block2	HumanSpeech	Pre	pou5	Object	Contrastive	r2	443.946570294827	2	post_focus	5	Contrastive post_focus
2023207	block2	HumanSpeech	Pre	ngaa5	Subject	Narrow	r2	132.232768314339	1	on_focus	5	Narrow on_focus
2023207	block2	HumanSpeech	Pre	ngaa5	Subject	Narrow	r2	108.112433862402	2	on_focus	5	Narrow on_focus
2023207	block2	HumanSpeech	Pre	maai5	Verb	Narrow	r2	188.732615268293	1	post_focus	5	Narrow post_focus
2023207	block2	HumanSpeech	Pre	pou5	Object	Narrow	r2	138.595324892378	1	post_focus	5	Narrow post_focus
2023207	block2	HumanSpeech	Pre	pou5	Object	Narrow	r2	406.243278920783	2	post_focus	5	Narrow post_focus
2023207	block2	HumanSpeech	Pre	lok6	Subject	Broad	r2	172.309685779055	1	broad_focus	6	Broad focus
2023207	block2	HumanSpeech	Pre	lok6	Subject	Broad	r2	152.542676329801	2	broad_focus	6	Broad focus
2023207	block2	HumanSpeech	Pre	waa6	Verb	Broad	r2	150.490617147739	1	broad_focus	6	Broad focus
2023207	block2	HumanSpeech	Pre	jyut6	Object	Broad	r2	115.90562646461	1	broad_focus	6	Broad focus
2023207	block2	HumanSpeech	Pre	loeng6	Object	Broad	r2	274.841539790486	2	broad_focus	6	Broad focus
2023207	block2	HumanSpeech	Pre	ma4	Subject	Contrastive	r2	190.551579428018	1	on_focus	4	Contrastive on_focus
2023207	block2	HumanSpeech	Pre	ma4	Subject	Contrastive	r2	208.891699129765	2	on_focus	4	Contrastive on_focus
2023207	block2	HumanSpeech	Pre	fu4	Verb	Contrastive	r2	102.927904771263	1	post_focus	4	Contrastive post_focus
2023207	block2	HumanSpeech	Pre	maang4	Object	Contrastive	r2	218.620527576149	1	post_focus	4	Contrastive post_focus
2023207	block2	HumanSpeech	Pre	jan4	Object	Contrastive	r2	288.973922902471	2	post_focus	4	Contrastive post_focus
2023207	block2	HumanSpeech	Pre	lok6	Subject	Narrow	r2	156.52099374546	1	pre_focus	6	Narrow pre_focus
2023207	block2	HumanSpeech	Pre	lok6	Subject	Narrow	r2	244.326530612227	2	pre_focus	6	Narrow pre_focus
2023207	block2	HumanSpeech	Pre	waa6	Verb	Narrow	r2	110.467768059607	1	on_focus	6	Narrow on_focus
2023207	block2	HumanSpeech	Pre	jyut6	Object	Narrow	r2	168.201058201021	1	post_focus	6	Narrow post_focus
2023207	block2	HumanSpeech	Pre	loeng6	Object	Narrow	r2	299.778425655973	2	post_focus	6	Narrow post_focus
2023207	block2	HumanSpeech	Pre	lok6	Subject	Narrow	r2	115.792258423767	1	on_focus	6	Narrow on_focus
2023207	block2	HumanSpeech	Pre	lok6	Subject	Narrow	r2	125.959448714525	2	on_focus	6	Narrow on_focus
2023207	block2	HumanSpeech	Pre	waa6	Verb	Narrow	r2	142.818594104313	1	post_focus	6	Narrow post_focus
2023207	block2	HumanSpeech	Pre	jyut6	Object	Narrow	r2	119.815759637163	1	post_focus	6	Narrow post_focus
2023207	block2	HumanSpeech	Pre	loeng6	Object	Narrow	r2	244.918867980061	2	post_focus	6	Narrow post_focus
2023207	block2	HumanSpeech	Pre	lok6	Subject	Narrow	r2	169.044217687087	1	pre_focus	6	Narrow pre_focus
2023207	block2	HumanSpeech	Pre	lok6	Subject	Narrow	r2	198.854551344368	2	pre_focus	6	Narrow pre_focus
2023207	block2	HumanSpeech	Pre	waa6	Verb	Narrow	r2	178.625598387441	1	pre_focus	6	Narrow pre_focus
2023207	block2	HumanSpeech	Pre	jyut6	Object	Narrow	r2	81.4856043427312	1	on_focus	6	Narrow on_focus
2023207	block2	HumanSpeech	Pre	loeng6	Object	Narrow	r2	221.275510204066	2	on_focus	6	Narrow on_focus
2023207	block2	HumanSpeech	Pre	ma4	Subject	Narrow	r2	158.41521793908	1	pre_focus	4	Narrow pre_focus
2023207	block2	HumanSpeech	Pre	ma4	Subject	Narrow	r2	215.411627614003	2	pre_focus	4	Narrow pre_focus
2023207	block2	HumanSpeech	Pre	fu4	Verb	Narrow	r2	94.090307068484	1	on_focus	4	Narrow on_focus
2023207	block2	HumanSpeech	Pre	maang4	Object	Narrow	r2	230.338927629532	1	post_focus	4	Narrow post_focus
2023207	block2	HumanSpeech	Pre	jan4	Object	Narrow	r2	272.134038800743	2	post_focus	4	Narrow post_focus
2023207	block2	HumanSpeech	Pre	ngaa5	Subject	Contrastive	r2	143.656997390167	1	pre_focus	5	Contrastive pre_focus
2023207	block2	HumanSpeech	Pre	ngaa5	Subject	Contrastive	r2	143.369163649822	2	pre_focus	5	Contrastive pre_focus
2023207	block2	HumanSpeech	Pre	maai5	Verb	Contrastive	r2	273.091458805709	1	pre_focus	5	Contrastive pre_focus
2023207	block2	HumanSpeech	Pre	pou5	Object	Contrastive	r2	227.307886117387	1	on_focus	5	Contrastive on_focus
2023207	block2	HumanSpeech	Pre	pou5	Object	Contrastive	r2	389.147680236135	2	on_focus	5	Contrastive on_focus
2023207	block2	HumanSpeech	Pre	ma4	Subject	Contrastive	r2	161.980482669208	1	pre_focus	4	Contrastive pre_focus
2023207	block2	HumanSpeech	Pre	ma4	Subject	Contrastive	r2	247.068351150006	2	pre_focus	4	Contrastive pre_focus
2023207	block2	HumanSpeech	Pre	fu4	Verb	Contrastive	r2	168.107909830553	1	pre_focus	4	Contrastive pre_focus
2023207	block2	HumanSpeech	Pre	maang4	Object	Contrastive	r2	228.102928127782	1	on_focus	4	Contrastive on_focus
2023207	block2	HumanSpeech	Pre	jan4	Object	Contrastive	r2	317.800075585808	2	on_focus	4	Contrastive on_focus
2023207	block2	HumanSpeech	Pre	lok6	Subject	Contrastive	r2	145.129149404113	1	pre_focus	6	Contrastive pre_focus
2023207	block2	HumanSpeech	Pre	lok6	Subject	Contrastive	r2	153.796836194772	2	pre_focus	6	Contrastive pre_focus
2023207	block2	HumanSpeech	Pre	waa6	Verb	Contrastive	r2	176.482741244627	1	pre_focus	6	Contrastive pre_focus
2023207	block2	HumanSpeech	Pre	jyut6	Object	Contrastive	r2	120.045351473948	1	on_focus	6	Contrastive on_focus
2023207	block2	HumanSpeech	Pre	loeng6	Object	Contrastive	r2	261.637512147729	2	on_focus	6	Contrastive on_focus
2023207	block2	HumanSpeech	Pre	ma4	Subject	Broad	r2	173.067379332679	1	broad_focus	4	Broad focus
2023207	block2	HumanSpeech	Pre	ma4	Subject	Broad	r2	324.474474474471	2	broad_focus	4	Broad focus
2023207	block2	HumanSpeech	Pre	fu4	Verb	Broad	r2	125.644935752121	1	broad_focus	4	Broad focus
2023207	block2	HumanSpeech	Pre	maang4	Object	Broad	r2	333.607042817107	1	broad_focus	4	Broad focus
2023207	block2	HumanSpeech	Pre	jan4	Object	Broad	r2	212.602040816307	2	broad_focus	4	Broad focus
2023207	block2	HumanSpeech	Pre	lok6	Subject	Contrastive	r2	152.432876532259	1	on_focus	6	Contrastive on_focus
2023207	block2	HumanSpeech	Pre	lok6	Subject	Contrastive	r2	163.513689426395	2	on_focus	6	Contrastive on_focus
2023207	block2	HumanSpeech	Pre	waa6	Verb	Contrastive	r2	167.545179688034	1	post_focus	6	Contrastive post_focus
2023207	block2	HumanSpeech	Pre	jyut6	Object	Contrastive	r2	161.476442428864	1	post_focus	6	Contrastive post_focus
2023207	block2	HumanSpeech	Pre	loeng6	Object	Contrastive	r2	179.789218717758	2	post_focus	6	Contrastive post_focus
2023207	block2	HumanSpeech	Pre	ma4	Subject	Contrastive	r2	136.514134542665	1	pre_focus	4	Contrastive pre_focus

2023207	block2	HumanSpeech	Pre	ma4	Subject	Contrastive	r2	312.447288161593	2	pre_focus	4	Contrastive pre_focus
2023207	block2	HumanSpeech	Pre	fu4	Verb	Contrastive	r2	190.831928212845	1	on_focus	4	Contrastive on_focus
2023207	block2	HumanSpeech	Pre	maang4	Object	Contrastive	r2	269.181153943066	1	post_focus	4	Contrastive post_focus
2023207	block2	HumanSpeech	Pre	jan4	Object	Contrastive	r2	169.997408487234	2	post_focus	4	Contrastive post_focus
2023207	block2	HumanSpeech	Pre	lok6	Subject	Contrastive	r2	117.437787080632	1	pre_focus	6	Contrastive pre_focus
2023207	block2	HumanSpeech	Pre	lok6	Subject	Contrastive	r2	123.27610409244	2	pre_focus	6	Contrastive pre_focus
2023207	block2	HumanSpeech	Pre	waa6	Verb	Contrastive	r2	175.903430155131	1	on_focus	6	Contrastive on_focus
2023207	block2	HumanSpeech	Pre	jyut6	Object	Contrastive	r2	145.504535147381	1	post_focus	6	Contrastive post_focus
2023207	block2	HumanSpeech	Pre	loeng6	Object	Contrastive	r2	224.162737963411	2	post_focus	6	Contrastive post_focus
2023207	block2	HumanSpeech	Pre	ngaa5	Subject	Narrow	r2	180.616298289749	1	pre_focus	5	Narrow pre_focus
2023207	block2	HumanSpeech	Pre	ngaa5	Subject	Narrow	r2	139.622401897554	2	pre_focus	5	Narrow pre_focus
2023207	block2	HumanSpeech	Pre	maai5	Verb	Narrow	r2	295.504220206567	1	pre_focus	5	Narrow pre_focus
2023207	block2	HumanSpeech	Pre	pou5	Object	Narrow	r2	206.483288967775	1	on_focus	5	Narrow on_focus
2023207	block2	HumanSpeech	Pre	pou5	Object	Narrow	r2	319.909297052163	2	on_focus	5	Narrow on_focus
2023207	block2	HumanSpeech	Pre	ma4	Subject	Narrow	r2	172.020926465791	1	on_focus	4	Narrow on_focus
2023207	block2	HumanSpeech	Pre	ma4	Subject	Narrow	r2	255.121207213051	2	on_focus	4	Narrow on_focus
2023207	block2	HumanSpeech	Pre	fu4	Verb	Narrow	r2	115.387874448061	1	post_focus	4	Narrow post_focus
2023207	block2	HumanSpeech	Pre	maang4	Object	Narrow	r2	266.967949235095	1	post_focus	4	Narrow post_focus
2023207	block2	HumanSpeech	Pre	jan4	Object	Narrow	r2	254.996220710552	2	post_focus	4	Narrow post_focus
2023207	block2	HumanSpeech	Pre	ma4	Subject	Narrow	r2	213.849773242657	1	pre_focus	4	Narrow pre_focus
2023207	block2	HumanSpeech	Pre	ma4	Subject	Narrow	r2	258.172244289199	2	pre_focus	4	Narrow pre_focus
2023207	block2	HumanSpeech	Pre	fu4	Verb	Narrow	r2	158.132653061216	1	pre_focus	4	Narrow pre_focus
2023207	block2	HumanSpeech	Pre	maang4	Object	Narrow	r2	297.439081452694	1	on_focus	4	Narrow on_focus
2023207	block2	HumanSpeech	Pre	jan4	Object	Narrow	r2	369.426423200821	2	on_focus	4	Narrow on_focus
2023207	block2	HumanSpeech	Pre	ngaa5	Subject	Broad	r2	147.245193617891	1	broad_focus	5	Broad focus
2023207	block2	HumanSpeech	Pre	ngaa5	Subject	Broad	r2	266.971705348851	2	broad_focus	5	Broad focus
2023207	block2	HumanSpeech	Pre	maai5	Verb	Broad	r2	179.244931183689	1	broad_focus	5	Broad focus
2023207	block2	HumanSpeech	Pre	pou5	Object	Broad	r2	195.793650793689	1	broad_focus	5	Broad focus
2023207	block2	HumanSpeech	Pre	pou5	Object	Broad	r2	242.123115913046	2	broad_focus	5	Broad focus
2023207	block3	HumanSpeech	Post	piu2	Subject	Contrastive	r1	84.2467776584357	1	pre_focus	2	Contrastive pre_focus
2023207	block3	HumanSpeech	Post	tse2	Subject	Contrastive	r1	103.507180650041	2	pre_focus	2	Contrastive pre_focus
2023207	block3	HumanSpeech	Post	tsap1	Verb	Contrastive	r1	50.5901990425741	1	pre_focus	1	Contrastive pre_focus
2023207	block3	HumanSpeech	Post	sy1	Object	Contrastive	r1	64.8458737030211	1	on_focus	1	Contrastive on_focus
2023207	block3	HumanSpeech	Post	pau1	Object	Contrastive	r1	207.851851851856	2	on_focus	1	Contrastive on_focus
2023207	block3	HumanSpeech	Post	suk1	Subject	Contrastive	r1	63.1956592160634	1	pre_focus	1	Contrastive pre_focus
2023207	block3	HumanSpeech	Post	suk1	Subject	Contrastive	r1	73.1775684632936	2	pre_focus	1	Contrastive pre_focus
2023207	block3	HumanSpeech	Post	sei2	Verb	Contrastive	r1	266.169690098266	1	on_focus	2	Contrastive on_focus
2023207	block3	HumanSpeech	Post	svy2	Object	Contrastive	r1	164.069664902996	1	post_focus	2	Contrastive post_focus
2023207	block3	HumanSpeech	Post	kwo2	Object	Contrastive	r1	403.50659922089	2	post_focus	2	Contrastive post_focus
2023207	block3	HumanSpeech	Post	suk1	Subject	Narrow	r1	64.5998152347431	1	on_focus	1	Narrow on_focus
2023207	block3	HumanSpeech	Post	suk1	Subject	Narrow	r1	77.0618103700826	2	on_focus	1	Narrow on_focus
2023207	block3	HumanSpeech	Post	sei2	Verb	Narrow	r1	295.158422064475	1	post_focus	2	Narrow post_focus
2023207	block3	HumanSpeech	Post	svy2	Object	Narrow	r1	136.848072562358	1	post_focus	2	Narrow post_focus
2023207	block3	HumanSpeech	Post	kwo2	Object	Narrow	r1	413.012633624888	2	post_focus	2	Narrow post_focus
2023207	block3	HumanSpeech	Post	piu2	Subject	Broad	r1	115.144368858665	1	broad_focus	2	Broad focus
2023207	block3	HumanSpeech	Post	tse2	Subject	Broad	r1	109.668367346941	2	broad_focus	2	Broad focus
2023207	block3	HumanSpeech	Post	tsap1	Verb	Broad	r1	47.2328931572576	1	broad_focus	1	Broad focus
2023207	block3	HumanSpeech	Post	sy1	Object	Broad	r1	105.328798185937	1	broad_focus	1	Broad focus
2023207	block3	HumanSpeech	Post	pau1	Object	Broad	r1	240.409941754478	2	broad_focus	1	Broad focus
2023207	block3	HumanSpeech	Post	suk1	Subject	Broad	r1	68.9650145772589	1	broad_focus	1	Broad focus
2023207	block3	HumanSpeech	Post	suk1	Subject	Broad	r1	69.4145537002697	2	broad_focus	1	Broad focus
2023207	block3	HumanSpeech	Post	sei2	Verb	Broad	r1	101.051673194519	1	broad_focus	2	Broad focus
2023207	block3	HumanSpeech	Post	svy2	Object	Broad	r1	138.070294784583	1	broad_focus	2	Broad focus
2023207	block3	HumanSpeech	Post	kwo2	Object	Broad	r1	192.643166357456	2	broad_focus	2	Broad focus
2023207	block3	HumanSpeech	Post	piu2	Subject	Contrastive	r1	107.954648526075	1	on_focus	2	Contrastive on_focus
2023207	block3	HumanSpeech	Post	tse2	Subject	Contrastive	r1	119.933484504912	2	on_focus	2	Contrastive on_focus
2023207	block3	HumanSpeech	Post	tsap1	Verb	Contrastive	r1	86.3129251700627	1	post_focus	1	Contrastive post_focus
2023207	block3	HumanSpeech	Post	sy1	Object	Contrastive	r1	64.3764172335608	1	post_focus	1	Contrastive post_focus
2023207	block3	HumanSpeech	Post	pau1	Object	Contrastive	r1	142.058956916102	2	post_focus	1	Contrastive post_focus
2023207	block3	HumanSpeech	Post	bui3	Subject	Narrow	r1	99.1763474620626	1	pre_focus	3	Narrow pre_focus
2023207	block3	HumanSpeech	Post	bui3	Subject	Narrow	r1	57.9002267573685	2	pre_focus	3	Narrow pre_focus
2023207	block3	HumanSpeech	Post	tsv1	Verb	Narrow	r1	1083.40702947847	1	on_focus	1	Narrow on_focus

**2023207**	block3	HumanSpeech	Post	fug1	Object	Narrow	r1	243.29705215419	1	post_focus	1	Narrow post_focus
**2023207**	block3	HumanSpeech	Post	tshe1	Object	Narrow	r1	435.544217687081	2	post_focus	1	Narrow post_focus
**2023207**	block3	HumanSpeech	Post	bui3	Subject	Contrastive	r1	130.730158730159	1	on_focus	3	Contrastive on_focus
**2023207**	block3	HumanSpeech	Post	bui3	Subject	Contrastive	r1	72.787952787948	2	on_focus	3	Contrastive on_focus
**2023207**	block3	HumanSpeech	Post	tsv1	Verb	Contrastive	r1	106.680272108832	1	post_focus	1	Contrastive post_focus
**2023207**	block3	HumanSpeech	Post	fug1	Object	Contrastive	r1	145.549886621311	1	post_focus	1	Contrastive post_focus
**2023207**	block3	HumanSpeech	Post	tshe1	Object	Contrastive	r1	380.038872691927	2	post_focus	1	Contrastive post_focus
**2023207**	block3	HumanSpeech	Post	piu2	Subject	Narrow	r1	125.521541950107	1	pre_focus	2	Narrow pre_focus
**2023207**	block3	HumanSpeech	Post	tse2	Subject	Narrow	r1	78.1043083900244	2	pre_focus	2	Narrow pre_focus
**2023207**	block3	HumanSpeech	Post	tsap1	Verb	Narrow	r1	62.6731205302775	1	pre_focus	1	Narrow pre_focus
**2023207**	block3	HumanSpeech	Post	sy1	Object	Narrow	r1	62.2873983901684	1	on_focus	1	Narrow on_focus
**2023207**	block3	HumanSpeech	Post	pau1	Object	Narrow	r1	301.427398545627	2	on_focus	1	Narrow on_focus
**2023207**	block3	HumanSpeech	Post	suk1	Subject	Contrastive	r1	56.6270018979367	1	pre_focus	1	Contrastive pre_focus
**2023207**	block3	HumanSpeech	Post	suk1	Subject	Contrastive	r1	83.7152688079499	2	pre_focus	1	Contrastive pre_focus
**2023207**	block3	HumanSpeech	Post	sei2	Verb	Contrastive	r1	102.013156911113	1	pre_focus	2	Contrastive pre_focus
**2023207**	block3	HumanSpeech	Post	svy2	Object	Contrastive	r1	83.0975056689311	1	on_focus	2	Contrastive on_focus
**2023207**	block3	HumanSpeech	Post	kwo2	Object	Contrastive	r1	263.703703703698	2	on_focus	2	Contrastive on_focus
**2023207**	block3	HumanSpeech	Post	piu2	Subject	Contrastive	r1	103.510616367771	1	pre_focus	2	Contrastive pre_focus
**2023207**	block3	HumanSpeech	Post	tse2	Subject	Contrastive	r1	107.736961451224	2	pre_focus	2	Contrastive pre_focus
**2023207**	block3	HumanSpeech	Post	tsap1	Verb	Contrastive	r1	192.213178922344	1	on_focus	1	Contrastive on_focus
**2023207**	block3	HumanSpeech	Post	sy1	Object	Contrastive	r1	92.2956484180872	1	post_focus	1	Contrastive post_focus
**2023207**	block3	HumanSpeech	Post	pau1	Object	Contrastive	r1	324.216130896787	2	post_focus	1	Contrastive post_focus
**2023207**	block3	HumanSpeech	Post	bui3	Subject	Contrastive	r1	98.8319088319258	1	pre_focus	3	Contrastive pre_focus
**2023207**	block3	HumanSpeech	Post	bui3	Subject	Contrastive	r1	106.92932728648	2	pre_focus	3	Contrastive pre_focus
**2023207**	block3	HumanSpeech	Post	tsv1	Verb	Contrastive	r1	198.049433106576	1	pre_focus	1	Contrastive pre_focus
**2023207**	block3	HumanSpeech	Post	fug1	Object	Contrastive	r1	139.625850340138	1	on_focus	1	Contrastive on_focus
**2023207**	block3	HumanSpeech	Post	tshe1	Object	Contrastive	r1	257.496850592105	2	on_focus	1	Contrastive on_focus
**2023207**	block3	HumanSpeech	Post	suk1	Subject	Narrow	r1	91.3514739229129	1	pre_focus	1	Narrow pre_focus
**2023207**	block3	HumanSpeech	Post	suk1	Subject	Narrow	r1	53.5449735449731	2	pre_focus	1	Narrow pre_focus
**2023207**	block3	HumanSpeech	Post	sei2	Verb	Narrow	r1	241.357709750559	1	on_focus	2	Narrow on_focus
**2023207**	block3	HumanSpeech	Post	svy2	Object	Narrow	r1	139.871504157242	1	post_focus	2	Narrow post_focus
**2023207**	block3	HumanSpeech	Post	kwo2	Object	Narrow	r1	127.743764172351	2	post_focus	2	Narrow post_focus
**2023207**	block3	HumanSpeech	Post	bui3	Subject	Narrow	r1	249.106575963708	1	pre_focus	3	Narrow pre_focus
**2023207**	block3	HumanSpeech	Post	bui3	Subject	Narrow	r1	338.494163097323	2	pre_focus	3	Narrow pre_focus
**2023207**	block3	HumanSpeech	Post	tsv1	Verb	Narrow	r1	274.962207105062	1	pre_focus	1	Narrow pre_focus
**2023207**	block3	HumanSpeech	Post	fug1	Object	Narrow	r1	119.770003239381	1	on_focus	1	Narrow on_focus
**2023207**	block3	HumanSpeech	Post	tshe1	Object	Narrow	r1	236.003887269192	2	on_focus	1	Narrow on_focus
**2023207**	block3	HumanSpeech	Post	suk1	Subject	Narrow	r1	42.3963844797015	1	pre_focus	1	Narrow pre_focus
**2023207**	block3	HumanSpeech	Post	suk1	Subject	Narrow	r1	76.8660968661266	2	pre_focus	1	Narrow pre_focus
**2023207**	block3	HumanSpeech	Post	sei2	Verb	Narrow	r1	193.234606663196	1	pre_focus	2	Narrow pre_focus
**2023207**	block3	HumanSpeech	Post	svy2	Object	Narrow	r1	126.698412698403	1	on_focus	2	Narrow on_focus
**2023207**	block3	HumanSpeech	Post	kwo2	Object	Narrow	r1	423.341389187243	2	on_focus	2	Narrow on_focus
**2023207**	block3	HumanSpeech	Post	bui3	Subject	Narrow	r1	47.9072184429299	1	on_focus	3	Narrow on_focus
**2023207**	block3	HumanSpeech	Post	bui3	Subject	Narrow	r1	81.0471331389522	2	on_focus	3	Narrow on_focus
**2023207**	block3	HumanSpeech	Post	tsv1	Verb	Narrow	r1	296.563753706607	1	post_focus	1	Narrow post_focus
**2023207**	block3	HumanSpeech	Post	fug1	Object	Narrow	r1	124.928674500097	1	post_focus	1	Narrow post_focus
**2023207**	block3	HumanSpeech	Post	tshe1	Object	Narrow	r1	234.979124168916	2	post_focus	1	Narrow post_focus
**2023207**	block3	HumanSpeech	Post	bui3	Subject	Contrastive	r1	63.7146224023297	1	pre_focus	3	Contrastive pre_focus
**2023207**	block3	HumanSpeech	Post	bui3	Subject	Contrastive	r1	49.992961657324	2	pre_focus	3	Contrastive pre_focus
**2023207**	block3	HumanSpeech	Post	tsv1	Verb	Contrastive	r1	259.580540094817	1	on_focus	1	Contrastive on_focus
**2023207**	block3	HumanSpeech	Post	fug1	Object	Contrastive	r1	123.784162787928	1	post_focus	1	Contrastive post_focus
**2023207**	block3	HumanSpeech	Post	tshe1	Object	Contrastive	r1	207.420005039069	2	post_focus	1	Contrastive post_focus
**2023207**	block3	HumanSpeech	Post	bui3	Subject	Broad	r1	76.5598061086621	1	broad_focus	3	Broad focus
**2023207**	block3	HumanSpeech	Post	bui3	Subject	Broad	r1	86.3304988662037	2	broad_focus	3	Broad focus
**2023207**	block3	HumanSpeech	Post	tsv1	Verb	Broad	r1	224.214337250061	1	broad_focus	1	Broad focus
**2023207**	block3	HumanSpeech	Post	fug1	Object	Broad	r1	157.02822816678	1	broad_focus	1	Broad focus
**2023207**	block3	HumanSpeech	Post	tshe1	Object	Broad	r1	268.621463077977	2	broad_focus	1	Broad focus
**2023207**	block3	HumanSpeech	Post	piu2	Subject	Narrow	r1	118.202947845788	1	on_focus	2	Narrow on_focus
**2023207**	block3	HumanSpeech	Post	tse2	Subject	Narrow	r1	89.4048645615158	2	on_focus	2	Narrow on_focus
**2023207**	block3	HumanSpeech	Post	tsap1	Verb	Narrow	r1	85.7026739842297	1	post_focus	1	Narrow post_focus
**2023207**	block3	HumanSpeech	Post	sy1	Object	Narrow	r1	86.3728679877909	1	post_focus	1	Narrow post_focus
**2023207**	block3	HumanSpeech	Post	pau1	Object	Narrow	r1	291.96671525753	2	post_focus	1	Narrow post_focus

**2023207**	block3	HumanSpeech	Post	piu2	Subject	Narrow	r1	24.8771730914825	1	pre_focus	2	Narrow pre_focus
**2023207**	block3	HumanSpeech	Post	tse2	Subject	Narrow	r1	72.7301587301667	2	pre_focus	2	Narrow pre_focus
**2023207**	block3	HumanSpeech	Post	tsap1	Verb	Narrow	r1	70.8045927365788	1	on_focus	1	Narrow on_focus
**2023207**	block3	HumanSpeech	Post	sy1	Object	Narrow	r1	89.4741118669629	1	post_focus	1	Narrow post_focus
**2023207**	block3	HumanSpeech	Post	pau1	Object	Narrow	r1	313.032879818593	2	post_focus	1	Narrow post_focus
**2023207**	block3	HumanSpeech	Post	suk1	Subject	Contrastive	r1	89.2397937295755	1	on_focus	1	Contrastive on_focus
**2023207**	block3	HumanSpeech	Post	suk1	Subject	Contrastive	r1	97.9370156022696	2	on_focus	1	Contrastive on_focus
**2023207**	block3	HumanSpeech	Post	sei2	Verb	Contrastive	r1	131.143123916246	1	post_focus	2	Contrastive post_focus
**2023207**	block3	HumanSpeech	Post	svy2	Object	Contrastive	r1	116.10458324742	1	post_focus	2	Contrastive post_focus
**2023207**	block3	HumanSpeech	Post	kwo2	Object	Contrastive	r1	121.86507936508	2	post_focus	2	Contrastive post_focus
**2023207**	block3	HumanSpeech	Post	suk1	Subject	Narrow	r2	63.0510722807287	1	pre_focus	1	Narrow pre_focus
**2023207**	block3	HumanSpeech	Post	suk1	Subject	Narrow	r2	76.6161898549456	2	pre_focus	1	Narrow pre_focus
**2023207**	block3	HumanSpeech	Post	sei2	Verb	Narrow	r2	98.9493197279216	1	pre_focus	2	Narrow pre_focus
**2023207**	block3	HumanSpeech	Post	svy2	Object	Narrow	r2	112.671705719322	1	on_focus	2	Narrow on_focus
**2023207**	block3	HumanSpeech	Post	kwo2	Object	Narrow	r2	190.284706475211	2	on_focus	2	Narrow on_focus
**2023207**	block3	HumanSpeech	Post	suk1	Subject	Narrow	r2	51.2899617786786	1	on_focus	1	Narrow on_focus
**2023207**	block3	HumanSpeech	Post	suk1	Subject	Narrow	r2	70.587622254294	2	on_focus	1	Narrow on_focus
**2023207**	block3	HumanSpeech	Post	sei2	Verb	Narrow	r2	88.7921390778672	1	post_focus	2	Narrow post_focus
**2023207**	block3	HumanSpeech	Post	svy2	Object	Narrow	r2	109.691358024719	1	post_focus	2	Narrow post_focus
**2023207**	block3	HumanSpeech	Post	kwo2	Object	Narrow	r2	152.068027210873	2	post_focus	2	Narrow post_focus
**2023207**	block3	HumanSpeech	Post	suk1	Subject	Contrastive	r2	90.0306463139771	1	on_focus	1	Contrastive on_focus
**2023207**	block3	HumanSpeech	Post	suk1	Subject	Contrastive	r2	87.0935161534874	2	on_focus	1	Contrastive on_focus
**2023207**	block3	HumanSpeech	Post	sei2	Verb	Contrastive	r2	110.298927263244	1	post_focus	2	Contrastive post_focus
**2023207**	block3	HumanSpeech	Post	svy2	Object	Contrastive	r2	103.7782551617	1	post_focus	2	Contrastive post_focus
**2023207**	block3	HumanSpeech	Post	kwo2	Object	Contrastive	r2	190.212396069569	2	post_focus	2	Contrastive post_focus
**2023207**	block3	HumanSpeech	Post	bui3	Subject	Contrastive	r2	73.520030234306	1	pre_focus	3	Contrastive pre_focus
**2023207**	block3	HumanSpeech	Post	bui3	Subject	Contrastive	r2	75.7027727435684	2	pre_focus	3	Contrastive pre_focus
**2023207**	block3	HumanSpeech	Post	tsv1	Verb	Contrastive	r2	107.679157538712	1	on_focus	1	Contrastive on_focus
**2023207**	block3	HumanSpeech	Post	fug1	Object	Contrastive	r2	81.0691609977425	1	post_focus	1	Contrastive post_focus
**2023207**	block3	HumanSpeech	Post	tshe1	Object	Contrastive	r2	213.335222978088	2	post_focus	1	Contrastive post_focus
**2023207**	block3	HumanSpeech	Post	bui3	Subject	Narrow	r2	70.787528344681	1	on_focus	3	Narrow on_focus
**2023207**	block3	HumanSpeech	Post	bui3	Subject	Narrow	r2	72.1641156462738	2	on_focus	3	Narrow on_focus
**2023207**	block3	HumanSpeech	Post	tsv1	Verb	Narrow	r2	100.565381708265	1	post_focus	1	Narrow post_focus
**2023207**	block3	HumanSpeech	Post	fug1	Object	Narrow	r2	103.051398337101	1	post_focus	1	Narrow post_focus
**2023207**	block3	HumanSpeech	Post	tshe1	Object	Narrow	r2	176.334089191243	2	post_focus	1	Narrow post_focus
**2023207**	block3	HumanSpeech	Post	piu2	Subject	Contrastive	r2	128.303552532117	1	pre_focus	2	Contrastive pre_focus
**2023207**	block3	HumanSpeech	Post	tse2	Subject	Contrastive	r2	107.514339068956	2	pre_focus	2	Contrastive pre_focus
**2023207**	block3	HumanSpeech	Post	tsap1	Verb	Contrastive	r2	97.3707117255458	1	on_focus	1	Contrastive on_focus
**2023207**	block3	HumanSpeech	Post	sy1	Object	Contrastive	r2	103.231292516995	1	post_focus	1	Contrastive post_focus
**2023207**	block3	HumanSpeech	Post	pau1	Object	Contrastive	r2	174.127524025465	2	post_focus	1	Contrastive post_focus
**2023207**	block3	HumanSpeech	Post	piu2	Subject	Broad	r2	98.3236961451155	1	broad_focus	2	Broad focus
**2023207**	block3	HumanSpeech	Post	tse2	Subject	Broad	r2	99.4571995464923	2	broad_focus	2	Broad focus
**2023207**	block3	HumanSpeech	Post	tsap1	Verb	Broad	r2	72.1590540978241	1	broad_focus	1	Broad focus
**2023207**	block3	HumanSpeech	Post	sy1	Object	Broad	r2	66.7687074829928	1	broad_focus	1	Broad focus
**2023207**	block3	HumanSpeech	Post	pau1	Object	Broad	r2	158.016502897453	2	broad_focus	1	Broad focus
**2023207**	block3	HumanSpeech	Post	bui3	Subject	Narrow	r2	61.5561224489909	1	pre_focus	3	Narrow pre_focus
**2023207**	block3	HumanSpeech	Post	bui3	Subject	Narrow	r2	131.65739022881	2	pre_focus	3	Narrow pre_focus
**2023207**	block3	HumanSpeech	Post	tsv1	Verb	Narrow	r2	135.952380952403	1	pre_focus	1	Narrow pre_focus
**2023207**	block3	HumanSpeech	Post	fug1	Object	Narrow	r2	143.021541950105	1	on_focus	1	Narrow on_focus
**2023207**	block3	HumanSpeech	Post	tshe1	Object	Narrow	r2	135.257369614521	2	on_focus	1	Narrow on_focus
**2023207**	block3	HumanSpeech	Post	suk1	Subject	Narrow	r2	59.1754866448753	1	pre_focus	1	Narrow pre_focus
**2023207**	block3	HumanSpeech	Post	suk1	Subject	Narrow	r2	45.3610216016216	2	pre_focus	1	Narrow pre_focus
**2023207**	block3	HumanSpeech	Post	sei2	Verb	Narrow	r2	96.066790352495	1	on_focus	2	Narrow on_focus
**2023207**	block3	HumanSpeech	Post	svy2	Object	Narrow	r2	93.4758125472683	1	post_focus	2	Narrow post_focus
**2023207**	block3	HumanSpeech	Post	kwo2	Object	Narrow	r2	220.615484288942	2	post_focus	2	Narrow post_focus
**2023207**	block3	HumanSpeech	Post	piu2	Subject	Narrow	r2	74.935752078602	1	on_focus	2	Narrow on_focus
**2023207**	block3	HumanSpeech	Post	tse2	Subject	Narrow	r2	108.134783704145	2	on_focus	2	Narrow on_focus
**2023207**	block3	HumanSpeech	Post	tsap1	Verb	Narrow	r2	103.734693877527	1	post_focus	1	Narrow post_focus
**2023207**	block3	HumanSpeech	Post	sy1	Object	Narrow	r2	100.854371378176	1	post_focus	1	Narrow post_focus
**2023207**	block3	HumanSpeech	Post	pau1	Object	Narrow	r2	155.146258503407	2	post_focus	1	Narrow post_focus
**2023207**	block3	HumanSpeech	Post	suk1	Subject	Broad	r2	62.4843159486375	1	broad_focus	1	Broad focus
**2023207**	block3	HumanSpeech	Post	suk1	Subject	Broad	r2	64.4525429219129	2	broad_focus	1	Broad focus

2023207	block3	HumanSpeech	Post	sei2	Verb	Broad	r2	93.4705506134037	1	broad_focus	2	Broad focus
2023207	block3	HumanSpeech	Post	svy2	Object	Broad	r2	86.2914862914863	1	broad_focus	2	Broad focus
2023207	block3	HumanSpeech	Post	kwo2	Object	Broad	r2	142.468820861666	2	broad_focus	2	Broad focus
2023207	block3	HumanSpeech	Post	piu2	Subject	Narrow	r2	104.546485260784	1	pre_focus	2	Narrow pre_focus
2023207	block3	HumanSpeech	Post	tse2	Subject	Narrow	r2	133.285462333077	2	pre_focus	2	Narrow pre_focus
2023207	block3	HumanSpeech	Post	tsap1	Verb	Narrow	r2	75.8503401360429	1	pre_focus	1	Narrow pre_focus
2023207	block3	HumanSpeech	Post	sy1	Object	Narrow	r2	110.235935644113	1	on_focus	1	Narrow on_focus
2023207	block3	HumanSpeech	Post	pau1	Object	Narrow	r2	123.397581254721	2	on_focus	1	Narrow on_focus
2023207	block3	HumanSpeech	Post	piu2	Subject	Contrastive	r2	102.297808012111	1	pre_focus	2	Contrastive pre_focus
2023207	block3	HumanSpeech	Post	tse2	Subject	Contrastive	r2	64.2947845804827	2	pre_focus	2	Contrastive pre_focus
2023207	block3	HumanSpeech	Post	tsap1	Verb	Contrastive	r2	93.6402656300572	1	pre_focus	1	Contrastive pre_focus
2023207	block3	HumanSpeech	Post	sy1	Object	Contrastive	r2	90.7790735341507	1	on_focus	1	Contrastive on_focus
2023207	block3	HumanSpeech	Post	pau1	Object	Contrastive	r2	214.496882086166	2	on_focus	1	Contrastive on_focus
2023207	block3	HumanSpeech	Post	piu2	Subject	Contrastive	r2	155.262832405697	1	on_focus	2	Contrastive on_focus
2023207	block3	HumanSpeech	Post	tse2	Subject	Contrastive	r2	120.332037576929	2	on_focus	2	Contrastive on_focus
2023207	block3	HumanSpeech	Post	tsap1	Verb	Contrastive	r2	73.7868480725865	1	post_focus	1	Contrastive post_focus
2023207	block3	HumanSpeech	Post	sy1	Object	Contrastive	r2	97.3242630385016	1	post_focus	1	Contrastive post_focus
2023207	block3	HumanSpeech	Post	pau1	Object	Contrastive	r2	250.811830479279	2	post_focus	1	Contrastive post_focus
2023207	block3	HumanSpeech	Post	bui3	Subject	Contrastive	r2	105.183673469412	1	pre_focus	3	Contrastive pre_focus
2023207	block3	HumanSpeech	Post	bui3	Subject	Contrastive	r2	170.687830687825	2	pre_focus	3	Contrastive pre_focus
2023207	block3	HumanSpeech	Post	tsv1	Verb	Contrastive	r2	110.288817281287	1	pre_focus	1	Contrastive pre_focus
2023207	block3	HumanSpeech	Post	fug1	Object	Contrastive	r2	176.465824425009	1	on_focus	1	Contrastive on_focus
2023207	block3	HumanSpeech	Post	tshe1	Object	Contrastive	r2	157.970521541927	2	on_focus	1	Contrastive on_focus
2023207	block3	HumanSpeech	Post	bui3	Subject	Narrow	r2	97.3023431595266	1	pre_focus	3	Narrow pre_focus
2023207	block3	HumanSpeech	Post	bui3	Subject	Narrow	r2	112.979394656463	2	pre_focus	3	Narrow pre_focus
2023207	block3	HumanSpeech	Post	tsv1	Verb	Narrow	r2	121.339380196559	1	on_focus	1	Narrow on_focus
2023207	block3	HumanSpeech	Post	fug1	Object	Narrow	r2	161.028974552778	1	post_focus	1	Narrow post_focus
2023207	block3	HumanSpeech	Post	tshe1	Object	Narrow	r2	215.848396501485	2	post_focus	1	Narrow post_focus
2023207	block3	HumanSpeech	Post	bui3	Subject	Contrastive	r2	161.906829461373	1	on_focus	3	Contrastive on_focus
2023207	block3	HumanSpeech	Post	bui3	Subject	Contrastive	r2	141.688947687953	2	on_focus	3	Contrastive on_focus
2023207	block3	HumanSpeech	Post	tsv1	Verb	Contrastive	r2	169.900066047717	1	post_focus	1	Contrastive post_focus
2023207	block3	HumanSpeech	Post	fug1	Object	Contrastive	r2	223.038828079495	1	post_focus	1	Contrastive post_focus
2023207	block3	HumanSpeech	Post	tshe1	Object	Contrastive	r2	318.555105881501	2	post_focus	1	Contrastive post_focus
2023207	block3	HumanSpeech	Post	piu2	Subject	Narrow	r2	173.663989072168	1	pre_focus	2	Narrow pre_focus
2023207	block3	HumanSpeech	Post	tse2	Subject	Narrow	r2	107.972917341556	2	pre_focus	2	Narrow pre_focus
2023207	block3	HumanSpeech	Post	tsap1	Verb	Narrow	r2	148.593474756751	1	on_focus	1	Narrow on_focus
2023207	block3	HumanSpeech	Post	sy1	Object	Narrow	r2	175.416510524769	1	post_focus	1	Narrow post_focus
2023207	block3	HumanSpeech	Post	pau1	Object	Narrow	r2	337.65084608018	2	post_focus	1	Narrow post_focus
2023207	block3	HumanSpeech	Post	suk1	Subject	Contrastive	r2	149.393751344576	1	pre_focus	1	Contrastive pre_focus
2023207	block3	HumanSpeech	Post	suk1	Subject	Contrastive	r2	134.166383219963	2	pre_focus	1	Contrastive pre_focus
2023207	block3	HumanSpeech	Post	sei2	Verb	Contrastive	r2	151.900759524494	1	pre_focus	2	Contrastive pre_focus
2023207	block3	HumanSpeech	Post	svy2	Object	Contrastive	r2	139.190252802962	1	on_focus	2	Contrastive on_focus
2023207	block3	HumanSpeech	Post	kwo2	Object	Contrastive	r2	444.781701040199	2	on_focus	2	Contrastive on_focus
2023207	block3	HumanSpeech	Post	suk1	Subject	Contrastive	r2	116.6471823297	1	pre_focus	1	Contrastive pre_focus
2023207	block3	HumanSpeech	Post	suk1	Subject	Contrastive	r2	164.566241212185	2	pre_focus	1	Contrastive pre_focus
2023207	block3	HumanSpeech	Post	sei2	Verb	Contrastive	r2	152.062293220581	1	on_focus	2	Contrastive on_focus
2023207	block3	HumanSpeech	Post	svy2	Object	Contrastive	r2	163.500481000483	1	post_focus	2	Contrastive post_focus
2023207	block3	HumanSpeech	Post	kwo2	Object	Contrastive	r2	400.523323615118	2	post_focus	2	Contrastive post_focus
2023207	block3	HumanSpeech	Post	bui3	Subject	Broad	r2	128.468550540731	1	broad_focus	3	Broad focus
2023207	block3	HumanSpeech	Post	bui3	Subject	Broad	r2	122.378261964968	2	broad_focus	3	Broad focus
2023207	block3	HumanSpeech	Post	tsv1	Verb	Broad	r2	127.766013093265	1	broad_focus	1	Broad focus
2023207	block3	HumanSpeech	Post	fug1	Object	Broad	r2	190.737392783205	1	broad_focus	1	Broad focus
2023207	block3	HumanSpeech	Post	tshe1	Object	Broad	r2	363.531182994734	2	broad_focus	1	Broad focus
2023207	block3	HumanSpeech	Pre	bui3	Subject	Narrow	r1	150.802437641744	1	pre_focus	3	Narrow pre_focus
2023207	block3	HumanSpeech	Pre	bui3	Subject	Narrow	r1	160.450026164312	2	pre_focus	3	Narrow pre_focus
2023207	block3	HumanSpeech	Pre	tsv1	Verb	Narrow	r1	143.298361768956	1	pre_focus	1	Narrow pre_focus
2023207	block3	HumanSpeech	Pre	fug1	Object	Narrow	r1	187.74287595221	1	on_focus	1	Narrow on_focus
2023207	block3	HumanSpeech	Pre	tshe1	Object	Narrow	r1	189.801646974587	2	on_focus	1	Narrow on_focus
2023207	block3	HumanSpeech	Pre	piu2	Subject	Broad	r1	183.11755579424	1	broad_focus	2	Broad focus
2023207	block3	HumanSpeech	Pre	tse2	Subject	Broad	r1	239.165615337043	2	broad_focus	2	Broad focus
2023207	block3	HumanSpeech	Pre	tsap1	Verb	Broad	r1	77.6372961883283	1	broad_focus	1	Broad focus
2023207	block3	HumanSpeech	Pre	sy1	Object	Broad	r1	136.088171702795	1	broad_focus	1	Broad focus

**2023207**	block3	HumanSpeech	Pre	pau1	Object	Broad	r1	258.741626919004	2	broad_focus	1	Broad focus
**2023207**	block3	HumanSpeech	Pre	suk1	Subject	Contrastive	r1	64.902865908266	1	pre_focus	1	Contrastive pre_focus
**2023207**	block3	HumanSpeech	Pre	suk1	Subject	Contrastive	r1	78.2753040610089	2	pre_focus	1	Contrastive pre_focus
**2023207**	block3	HumanSpeech	Pre	sei2	Verb	Contrastive	r1	131.793206171352	1	pre_focus	2	Contrastive pre_focus
**2023207**	block3	HumanSpeech	Pre	svy2	Object	Contrastive	r1	182.847611091233	1	on_focus	2	Contrastive on_focus
**2023207**	block3	HumanSpeech	Pre	kwo2	Object	Contrastive	r1	296.570582406446	2	on_focus	2	Contrastive on_focus
**2023207**	block3	HumanSpeech	Pre	suk1	Subject	Contrastive	r1	52.9376643130206	1	on_focus	1	Contrastive on_focus
**2023207**	block3	HumanSpeech	Pre	suk1	Subject	Contrastive	r1	63.8563734564173	2	on_focus	1	Contrastive on_focus
**2023207**	block3	HumanSpeech	Pre	sei2	Verb	Contrastive	r1	118.280565358958	1	post_focus	2	Contrastive post_focus
**2023207**	block3	HumanSpeech	Pre	svy2	Object	Contrastive	r1	177.391254578737	1	post_focus	2	Contrastive post_focus
**2023207**	block3	HumanSpeech	Pre	kwo2	Object	Contrastive	r1	243.242630385481	2	post_focus	2	Contrastive post_focus
**2023207**	block3	HumanSpeech	Pre	suk1	Subject	Narrow	r1	63.359101216264	1	pre_focus	1	Narrow pre_focus
**2023207**	block3	HumanSpeech	Pre	suk1	Subject	Narrow	r1	49.0064935064822	2	pre_focus	1	Narrow pre_focus
**2023207**	block3	HumanSpeech	Pre	sei2	Verb	Narrow	r1	150.973533023233	1	pre_focus	2	Narrow pre_focus
**2023207**	block3	HumanSpeech	Pre	svy2	Object	Narrow	r1	143.667697381971	1	on_focus	2	Narrow on_focus
**2023207**	block3	HumanSpeech	Pre	kwo2	Object	Narrow	r1	402.201814058941	2	on_focus	2	Narrow on_focus
**2023207**	block3	HumanSpeech	Pre	piu2	Subject	Narrow	r1	228.633835300514	1	on_focus	2	Narrow on_focus
**2023207**	block3	HumanSpeech	Pre	tse2	Subject	Narrow	r1	119.321915900855	2	on_focus	2	Narrow on_focus
**2023207**	block3	HumanSpeech	Pre	tsap1	Verb	Narrow	r1	46.4904362776792	1	post_focus	1	Narrow post_focus
**2023207**	block3	HumanSpeech	Pre	sy1	Object	Narrow	r1	129.532375913328	1	post_focus	1	Narrow post_focus
**2023207**	block3	HumanSpeech	Pre	pau1	Object	Narrow	r1	211.28254611537	2	post_focus	1	Narrow post_focus
**2023207**	block3	HumanSpeech	Pre	bui3	Subject	Contrastive	r1	164.623330813782	1	on_focus	3	Contrastive on_focus
**2023207**	block3	HumanSpeech	Pre	bui3	Subject	Contrastive	r1	135.826530612235	2	on_focus	3	Contrastive on_focus
**2023207**	block3	HumanSpeech	Pre	tsv1	Verb	Contrastive	r1	132.245842781572	1	post_focus	1	Contrastive post_focus
**2023207**	block3	HumanSpeech	Pre	fug1	Object	Contrastive	r1	133.430432001859	1	post_focus	1	Contrastive post_focus
**2023207**	block3	HumanSpeech	Pre	tshe1	Object	Contrastive	r1	237.721678414971	2	post_focus	1	Contrastive post_focus
**2023207**	block3	HumanSpeech	Pre	suk1	Subject	Contrastive	r1	38.0103660511395	1	pre_focus	1	Contrastive pre_focus
**2023207**	block3	HumanSpeech	Pre	suk1	Subject	Contrastive	r1	121.062225756106	2	pre_focus	1	Contrastive pre_focus
**2023207**	block3	HumanSpeech	Pre	sei2	Verb	Contrastive	r1	165.457425254061	1	on_focus	2	Contrastive on_focus
**2023207**	block3	HumanSpeech	Pre	svy2	Object	Contrastive	r1	200.942055393568	1	post_focus	2	Contrastive post_focus
**2023207**	block3	HumanSpeech	Pre	kwo2	Object	Contrastive	r1	247.625850340143	2	post_focus	2	Contrastive post_focus
**2023207**	block3	HumanSpeech	Pre	piu2	Subject	Contrastive	r1	170.696469063813	1	pre_focus	2	Contrastive pre_focus
**2023207**	block3	HumanSpeech	Pre	tse2	Subject	Contrastive	r1	144.308183879588	2	pre_focus	2	Contrastive pre_focus
**2023207**	block3	HumanSpeech	Pre	tsap1	Verb	Contrastive	r1	64.7810374149742	1	pre_focus	1	Contrastive pre_focus
**2023207**	block3	HumanSpeech	Pre	sy1	Object	Contrastive	r1	108.944318468133	1	on_focus	1	Contrastive on_focus
**2023207**	block3	HumanSpeech	Pre	pau1	Object	Contrastive	r1	330.82905676892	2	on_focus	1	Contrastive on_focus
**2023207**	block3	HumanSpeech	Pre	bui3	Subject	Narrow	r1	183.654195011343	1	pre_focus	3	Narrow pre_focus
**2023207**	block3	HumanSpeech	Pre	bui3	Subject	Narrow	r1	128.284632034649	2	pre_focus	3	Narrow pre_focus
**2023207**	block3	HumanSpeech	Pre	tsv1	Verb	Narrow	r1	159.232513518219	1	on_focus	1	Narrow on_focus
**2023207**	block3	HumanSpeech	Pre	fug1	Object	Narrow	r1	177.658730158726	1	post_focus	1	Narrow post_focus
**2023207**	block3	HumanSpeech	Pre	tshe1	Object	Narrow	r1	195.63265306121	2	post_focus	1	Narrow post_focus
**2023207**	block3	HumanSpeech	Pre	bui3	Subject	Broad	r1	147.654825794348	1	broad_focus	3	Broad focus
**2023207**	block3	HumanSpeech	Pre	bui3	Subject	Broad	r1	158.445452254995	2	broad_focus	3	Broad focus
**2023207**	block3	HumanSpeech	Pre	tsv1	Verb	Broad	r1	183.415741476949	1	broad_focus	1	Broad focus
**2023207**	block3	HumanSpeech	Pre	fug1	Object	Broad	r1	183.623078861189	1	broad_focus	1	Broad focus
**2023207**	block3	HumanSpeech	Pre	tshe1	Object	Broad	r1	153.769841269849	2	broad_focus	1	Broad focus
**2023207**	block3	HumanSpeech	Pre	bui3	Subject	Contrastive	r1	204.949210976906	1	pre_focus	3	Contrastive pre_focus
**2023207**	block3	HumanSpeech	Pre	bui3	Subject	Contrastive	r1	178.550092764397	2	pre_focus	3	Contrastive pre_focus
**2023207**	block3	HumanSpeech	Pre	tsv1	Verb	Contrastive	r1	152.282432271136	1	on_focus	1	Contrastive on_focus
**2023207**	block3	HumanSpeech	Pre	fug1	Object	Contrastive	r1	187.449303530968	1	post_focus	1	Contrastive post_focus
**2023207**	block3	HumanSpeech	Pre	tshe1	Object	Contrastive	r1	231.052154194998	2	post_focus	1	Contrastive post_focus
**2023207**	block3	HumanSpeech	Pre	bui3	Subject	Narrow	r1	202.869629333179	1	on_focus	3	Narrow on_focus
**2023207**	block3	HumanSpeech	Pre	bui3	Subject	Narrow	r1	152.890795981591	2	on_focus	3	Narrow on_focus
**2023207**	block3	HumanSpeech	Pre	tsv1	Verb	Narrow	r1	163.286237571981	1	post_focus	1	Narrow post_focus
**2023207**	block3	HumanSpeech	Pre	fug1	Object	Narrow	r1	180.768140589578	1	post_focus	1	Narrow post_focus
**2023207**	block3	HumanSpeech	Pre	tshe1	Object	Narrow	r1	201.087301587279	2	post_focus	1	Narrow post_focus
**2023207**	block3	HumanSpeech	Pre	piu2	Subject	Contrastive	r1	155.554925673982	1	pre_focus	2	Contrastive pre_focus
**2023207**	block3	HumanSpeech	Pre	tse2	Subject	Contrastive	r1	128.032171201824	2	pre_focus	2	Contrastive pre_focus
**2023207**	block3	HumanSpeech	Pre	tsap1	Verb	Contrastive	r1	85.2718928210265	1	on_focus	1	Contrastive on_focus
**2023207**	block3	HumanSpeech	Pre	sy1	Object	Contrastive	r1	135.576457576917	1	post_focus	1	Contrastive post_focus
**2023207**	block3	HumanSpeech	Pre	pau1	Object	Contrastive	r1	339.39681545138	2	post_focus	1	Contrastive post_focus
**2023207**	block3	HumanSpeech	Pre	piu2	Subject	Narrow	r1	158.155994673052	1	pre_focus	2	Narrow pre_focus

**2023207**	block3	HumanSpeech	Pre	tse2	Subject	Narrow	r1	97.0860344137918	2	pre_focus	2	Narrow pre_focus
**2023207**	block3	HumanSpeech	Pre	tsap1	Verb	Narrow	r1	101.88919866124	1	on_focus	1	Narrow on_focus
**2023207**	block3	HumanSpeech	Pre	sy1	Object	Narrow	r1	121.535460160999	1	post_focus	1	Narrow post_focus
**2023207**	block3	HumanSpeech	Pre	pau1	Object	Narrow	r1	335.446226109468	2	on_focus	1	Narrow on_focus
**2023207**	block3	HumanSpeech	Pre	suk1	Subject	Narrow	r1	72.5518984249334	1	pre_focus	1	Narrow pre_focus
**2023207**	block3	HumanSpeech	Pre	suk1	Subject	Narrow	r1	95.5673782671056	2	pre_focus	1	Narrow pre_focus
**2023207**	block3	HumanSpeech	Pre	sei2	Verb	Narrow	r1	157.2215061463	1	on_focus	2	Narrow on_focus
**2023207**	block3	HumanSpeech	Pre	svy2	Object	Narrow	r1	169.168556311433	1	post_focus	2	Narrow post_focus
**2023207**	block3	HumanSpeech	Pre	kwo2	Object	Narrow	r1	335.592403628141	2	post_focus	2	Narrow post_focus
**2023207**	block3	HumanSpeech	Pre	piu2	Subject	Contrastive	r1	270.530612244954	1	on_focus	2	Contrastive on_focus
**2023207**	block3	HumanSpeech	Pre	tse2	Subject	Contrastive	r1	150.334646139186	2	on_focus	2	Contrastive on_focus
**2023207**	block3	HumanSpeech	Pre	tsap1	Verb	Contrastive	r1	106.621200668826	1	post_focus	1	Contrastive post_focus
**2023207**	block3	HumanSpeech	Pre	sy1	Object	Contrastive	r1	137.936507936558	1	post_focus	1	Contrastive post_focus
**2023207**	block3	HumanSpeech	Pre	pau1	Object	Contrastive	r1	612.839357965413	2	post_focus	1	Contrastive post_focus
**2023207**	block3	HumanSpeech	Pre	suk1	Subject	Narrow	r1	64.6491027868592	1	on_focus	1	Narrow on_focus
**2023207**	block3	HumanSpeech	Pre	suk1	Subject	Narrow	r1	82.9100007819079	2	on_focus	1	Narrow on_focus
**2023207**	block3	HumanSpeech	Pre	sei2	Verb	Narrow	r1	152.403516728384	1	post_focus	2	Narrow post_focus
**2023207**	block3	HumanSpeech	Pre	svy2	Object	Narrow	r1	222.365538808106	1	post_focus	2	Narrow post_focus
**2023207**	block3	HumanSpeech	Pre	kwo2	Object	Narrow	r1	388.996083281768	2	post_focus	2	Narrow post_focus
**2023207**	block3	HumanSpeech	Pre	suk1	Subject	Broad	r1	64.2353237590783	1	broad_focus	1	Broad focus
**2023207**	block3	HumanSpeech	Pre	suk1	Subject	Broad	r1	70.8491654920635	2	broad_focus	1	Broad focus
**2023207**	block3	HumanSpeech	Pre	sei2	Verb	Broad	r1	135.239154269755	1	broad_focus	2	Broad focus
**2023207**	block3	HumanSpeech	Pre	svy2	Object	Broad	r1	128.21394064872	1	broad_focus	2	Broad focus
**2023207**	block3	HumanSpeech	Pre	kwo2	Object	Broad	r1	479.873236331571	2	broad_focus	2	Broad focus
**2023207**	block3	HumanSpeech	Pre	bui3	Subject	Contrastive	r1	212.535700062574	1	pre_focus	3	Contrastive pre_focus
**2023207**	block3	HumanSpeech	Pre	bui3	Subject	Contrastive	r1	161.741556271636	2	pre_focus	3	Contrastive pre_focus
**2023207**	block3	HumanSpeech	Pre	tsv1	Verb	Contrastive	r1	212.249459170664	1	pre_focus	1	Contrastive pre_focus
**2023207**	block3	HumanSpeech	Pre	fug1	Object	Contrastive	r1	161.50566893424	1	on_focus	1	Contrastive on_focus
**2023207**	block3	HumanSpeech	Pre	tshe1	Object	Contrastive	r1	194.213777465052	2	on_focus	1	Contrastive on_focus
**2023207**	block3	HumanSpeech	Pre	piu2	Subject	Narrow	r1	179.015306122494	1	pre_focus	2	Narrow pre_focus
**2023207**	block3	HumanSpeech	Pre	tse2	Subject	Narrow	r1	158.44907608431	2	pre_focus	2	Narrow pre_focus
**2023207**	block3	HumanSpeech	Pre	tsap1	Verb	Narrow	r1	86.0622208900281	1	on_focus	1	Narrow on_focus
**2023207**	block3	HumanSpeech	Pre	sy1	Object	Narrow	r1	210.98266812561	1	post_focus	1	Narrow post_focus
**2023207**	block3	HumanSpeech	Pre	pau1	Object	Narrow	r1	374.633376055783	2	post_focus	1	Narrow post_focus
**2023207**	block3	HumanSpeech	Pre	bui3	Subject	Contrastive	r2	227.341426225678	1	on_focus	3	Contrastive on_focus
**2023207**	block3	HumanSpeech	Pre	bui3	Subject	Contrastive	r2	184.545135514497	2	on_focus	3	Contrastive on_focus
**2023207**	block3	HumanSpeech	Pre	tsv1	Verb	Contrastive	r2	159.040382110277	1	post_focus	1	Contrastive post_focus
**2023207**	block3	HumanSpeech	Pre	fug1	Object	Contrastive	r2	108.835385691918	1	post_focus	1	Contrastive post_focus
**2023207**	block3	HumanSpeech	Pre	tshe1	Object	Contrastive	r2	291.969283687706	2	post_focus	1	Contrastive post_focus
**2023207**	block3	HumanSpeech	Pre	suk1	Subject	Broad	r2	105.921982319501	1	broad_focus	1	Broad focus
**2023207**	block3	HumanSpeech	Pre	suk1	Subject	Broad	r2	79.0763416477489	2	broad_focus	1	Broad focus
**2023207**	block3	HumanSpeech	Pre	sei2	Verb	Broad	r2	141.072162198157	1	broad_focus	2	Broad focus
**2023207**	block3	HumanSpeech	Pre	svy2	Object	Broad	r2	183.26272933416	1	broad_focus	2	Broad focus
**2023207**	block3	HumanSpeech	Pre	kwo2	Object	Broad	r2	238.040438397604	2	broad_focus	2	Broad focus
**2023207**	block3	HumanSpeech	Pre	piu2	Subject	Contrastive	r2	151.177063417947	1	pre_focus	2	Contrastive pre_focus
**2023207**	block3	HumanSpeech	Pre	tse2	Subject	Contrastive	r2	150.210540781984	2	pre_focus	2	Contrastive pre_focus
**2023207**	block3	HumanSpeech	Pre	tsap1	Verb	Contrastive	r2	132.487697206557	1	pre_focus	1	Contrastive pre_focus
**2023207**	block3	HumanSpeech	Pre	sy1	Object	Contrastive	r2	134.387400793628	1	on_focus	1	Contrastive on_focus
**2023207**	block3	HumanSpeech	Pre	pau1	Object	Contrastive	r2	462.688455092177	2	on_focus	1	Contrastive on_focus
**2023207**	block3	HumanSpeech	Pre	bui3	Subject	Contrastive	r2	149.004487575894	1	pre_focus	3	Contrastive pre_focus
**2023207**	block3	HumanSpeech	Pre	bui3	Subject	Contrastive	r2	188.124241149467	2	pre_focus	3	Contrastive pre_focus
**2023207**	block3	HumanSpeech	Pre	tsv1	Verb	Contrastive	r2	167.530571752536	1	on_focus	1	Contrastive on_focus
**2023207**	block3	HumanSpeech	Pre	fug1	Object	Contrastive	r2	182.397392290227	1	post_focus	1	Contrastive post_focus
**2023207**	block3	HumanSpeech	Pre	tshe1	Object	Contrastive	r2	206.193964765419	2	post_focus	1	Contrastive post_focus
**2023207**	block3	HumanSpeech	Pre	suk1	Subject	Narrow	r2	93.4202079100146	1	pre_focus	1	Narrow pre_focus
**2023207**	block3	HumanSpeech	Pre	suk1	Subject	Narrow	r2	75.1108018965283	2	pre_focus	1	Narrow pre_focus
**2023207**	block3	HumanSpeech	Pre	sei2	Verb	Narrow	r2	122.561494439026	1	on_focus	2	Narrow on_focus
**2023207**	block3	HumanSpeech	Pre	svy2	Object	Narrow	r2	158.068310657598	1	post_focus	2	Narrow post_focus
**2023207**	block3	HumanSpeech	Pre	kwo2	Object	Narrow	r2	243.284202569896	2	post_focus	2	Narrow post_focus
**2023207**	block3	HumanSpeech	Pre	bui3	Subject	Narrow	r2	151.401772830297	1	pre_focus	3	Narrow pre_focus
**2023207**	block3	HumanSpeech	Pre	bui3	Subject	Narrow	r2	146.84729064038	2	pre_focus	3	Narrow pre_focus
**2023207**	block3	HumanSpeech	Pre	tsv1	Verb	Narrow	r2	112.079016221855	1	pre_focus	1	Narrow pre_focus

2023207	block3	HumanSpeech	Pre	fug1	Object	Narrow	r2	174.126689636921	1	on_focus	1	Narrow on_focus
2023207	block3	HumanSpeech	Pre	tshe1	Object	Narrow	r2	139.899092970495	2	on_focus	1	Narrow on_focus
2023207	block3	HumanSpeech	Pre	suk1	Subject	Contrastive	r2	46.7814196385348	1	pre_focus	1	Contrastive pre_focus
2023207	block3	HumanSpeech	Pre	suk1	Subject	Contrastive	r2	66.6767210028638	2	pre_focus	1	Contrastive pre_focus
2023207	block3	HumanSpeech	Pre	sei2	Verb	Contrastive	r2	292.192849010235	1	on_focus	2	Contrastive on_focus
2023207	block3	HumanSpeech	Pre	svy2	Object	Contrastive	r2	330.776985926661	1	post_focus	2	Contrastive post_focus
2023207	block3	HumanSpeech	Pre	kwo2	Object	Contrastive	r2	268.93088099439	2	post_focus	2	Contrastive post_focus
2023207	block3	HumanSpeech	Pre	piu2	Subject	Contrastive	r2	175.108819541947	1	pre_focus	2	Contrastive pre_focus
2023207	block3	HumanSpeech	Pre	tse2	Subject	Contrastive	r2	146.43343051506	2	pre_focus	2	Contrastive pre_focus
2023207	block3	HumanSpeech	Pre	tsap1	Verb	Contrastive	r2	81.5440893338746	1	on_focus	1	Contrastive on_focus
2023207	block3	HumanSpeech	Pre	sy1	Object	Contrastive	r2	179.347262714543	1	post_focus	1	Contrastive post_focus
2023207	block3	HumanSpeech	Pre	pau1	Object	Contrastive	r2	355.19686662542	2	post_focus	1	Contrastive post_focus
2023207	block3	HumanSpeech	Pre	bui3	Subject	Broad	r2	238.913110698888	1	broad_focus	3	Broad focus
2023207	block3	HumanSpeech	Pre	bui3	Subject	Broad	r2	148.592936164334	2	broad_focus	3	Broad focus
2023207	block3	HumanSpeech	Pre	tsv1	Verb	Broad	r2	174.103967401493	1	broad_focus	1	Broad focus
2023207	block3	HumanSpeech	Pre	fug1	Object	Broad	r2	208.562701270978	1	broad_focus	1	Broad focus
2023207	block3	HumanSpeech	Pre	tshe1	Object	Broad	r2	279.118968710804	2	broad_focus	1	Broad focus
2023207	block3	HumanSpeech	Pre	bui3	Subject	Contrastive	r2	200.94217687074	1	pre_focus	3	Contrastive pre_focus
2023207	block3	HumanSpeech	Pre	bui3	Subject	Contrastive	r2	135.600670823919	2	pre_focus	3	Contrastive pre_focus
2023207	block3	HumanSpeech	Pre	tsv1	Verb	Contrastive	r2	195.552532123941	1	pre_focus	1	Contrastive pre_focus
2023207	block3	HumanSpeech	Pre	fug1	Object	Contrastive	r2	196.950034092879	1	on_focus	1	Contrastive on_focus
2023207	block3	HumanSpeech	Pre	tshe1	Object	Contrastive	r2	188.428790871228	2	on_focus	1	Contrastive on_focus
2023207	block3	HumanSpeech	Pre	suk1	Subject	Narrow	r2	92.582065553529	1	pre_focus	1	Narrow pre_focus
2023207	block3	HumanSpeech	Pre	suk1	Subject	Narrow	r2	81.5315279958213	2	pre_focus	1	Narrow pre_focus
2023207	block3	HumanSpeech	Pre	sei2	Verb	Narrow	r2	304.806638102377	1	pre_focus	2	Narrow pre_focus
2023207	block3	HumanSpeech	Pre	svy2	Object	Narrow	r2	205.53725583801	1	on_focus	2	Narrow on_focus
2023207	block3	HumanSpeech	Pre	kwo2	Object	Narrow	r2	464.566566566589	2	on_focus	2	Narrow on_focus
2023207	block3	HumanSpeech	Pre	suk1	Subject	Contrastive	r2	88.8586545729027	1	on_focus	1	Contrastive on_focus
2023207	block3	HumanSpeech	Pre	suk1	Subject	Contrastive	r2	88.5694849368406	2	on_focus	1	Contrastive on_focus
2023207	block3	HumanSpeech	Pre	sei2	Verb	Contrastive	r2	168.750099859267	1	post_focus	2	Contrastive post_focus
2023207	block3	HumanSpeech	Pre	svy2	Object	Contrastive	r2	142.277075909703	1	post_focus	2	Contrastive post_focus
2023207	block3	HumanSpeech	Pre	kwo2	Object	Contrastive	r2	292.197026958888	2	post_focus	2	Contrastive post_focus
2023207	block3	HumanSpeech	Pre	bui3	Subject	Narrow	r2	196.547388025238	1	on_focus	3	Narrow on_focus
2023207	block3	HumanSpeech	Pre	bui3	Subject	Narrow	r2	215.499310835469	2	on_focus	3	Narrow on_focus
2023207	block3	HumanSpeech	Pre	tsv1	Verb	Narrow	r2	131.00262832404	1	post_focus	1	Narrow post_focus
2023207	block3	HumanSpeech	Pre	fug1	Object	Narrow	r2	187.39815739815	1	post_focus	1	Narrow post_focus
2023207	block3	HumanSpeech	Pre	tshe1	Object	Narrow	r2	175.343915343944	2	post_focus	1	Narrow post_focus
2023207	block3	HumanSpeech	Pre	piu2	Subject	Contrastive	r2	174.369843175327	1	on_focus	2	Contrastive on_focus
2023207	block3	HumanSpeech	Pre	tse2	Subject	Contrastive	r2	122.589270140338	2	on_focus	2	Contrastive on_focus
2023207	block3	HumanSpeech	Pre	tsap1	Verb	Contrastive	r2	78.045027534813	1	post_focus	1	Contrastive post_focus
2023207	block3	HumanSpeech	Pre	sy1	Object	Contrastive	r2	115.266955266975	1	post_focus	1	Contrastive post_focus
2023207	block3	HumanSpeech	Pre	pau1	Object	Contrastive	r2	243.955978381337	2	post_focus	1	Contrastive post_focus
2023207	block3	HumanSpeech	Pre	piu2	Subject	Broad	r2	167.585093250125	1	broad_focus	2	Broad focus
2023207	block3	HumanSpeech	Pre	tse2	Subject	Broad	r2	136.38699924411	2	broad_focus	2	Broad focus
2023207	block3	HumanSpeech	Pre	tsap1	Verb	Broad	r2	96.2156714537628	1	broad_focus	1	Broad focus
2023207	block3	HumanSpeech	Pre	sy1	Object	Broad	r2	148.409716973163	1	broad_focus	1	Broad focus
2023207	block3	HumanSpeech	Pre	pau1	Object	Broad	r2	277.912860382287	2	broad_focus	1	Broad focus
2023207	block3	HumanSpeech	Pre	piu2	Subject	Narrow	r2	144.915986394551	1	pre_focus	2	Narrow pre_focus
2023207	block3	HumanSpeech	Pre	tse2	Subject	Narrow	r2	148.517978620021	2	pre_focus	2	Narrow pre_focus
2023207	block3	HumanSpeech	Pre	tsap1	Verb	Narrow	r2	84.2309685779128	1	on_focus	1	Narrow on_focus
2023207	block3	HumanSpeech	Pre	sy1	Object	Narrow	r2	148.590864917367	1	post_focus	1	Narrow post_focus
2023207	block3	HumanSpeech	Pre	pau1	Object	Narrow	r2	285.007960631049	2	post_focus	1	Narrow post_focus
2023207	block3	HumanSpeech	Pre	piu2	Subject	Narrow	r2	160.149281934991	1	on_focus	2	Narrow on_focus
2023207	block3	HumanSpeech	Pre	tse2	Subject	Narrow	r2	123.349206349189	2	on_focus	2	Narrow on_focus
2023207	block3	HumanSpeech	Pre	tsap1	Verb	Narrow	r2	61.6555955715512	1	post_focus	1	Narrow post_focus
2023207	block3	HumanSpeech	Pre	sy1	Object	Narrow	r2	153.489633948766	1	post_focus	1	Narrow post_focus
2023207	block3	HumanSpeech	Pre	pau1	Object	Narrow	r2	261.431270921094	2	post_focus	1	Narrow post_focus
2023207	block3	HumanSpeech	Pre	bui3	Subject	Narrow	r2	266.053076597871	1	pre_focus	3	Narrow pre_focus
2023207	block3	HumanSpeech	Pre	bui3	Subject	Narrow	r2	114.55500310899	2	pre_focus	3	Narrow pre_focus
2023207	block3	HumanSpeech	Pre	tsv1	Verb	Narrow	r2	139.478309842445	1	on_focus	1	Narrow on_focus
2023207	block3	HumanSpeech	Pre	fug1	Object	Narrow	r2	181.569796624615	1	post_focus	1	Narrow post_focus
2023207	block3	HumanSpeech	Pre	tshe1	Object	Narrow	r2	136.782116355334	2	post_focus	1	Narrow post_focus

2023207	block3	HumanSpeech	Pre	suk1	Subject	Contrastive	r2	94.4703974670915	1	pre_focus	1	Contrastive pre_focus
2023207	block3	HumanSpeech	Pre	suk1	Subject	Contrastive	r2	168.133296177018	2	pre_focus	1	Contrastive pre_focus
2023207	block3	HumanSpeech	Pre	sei2	Verb	Contrastive	r2	211.868248735868	1	pre_focus	2	Contrastive pre_focus
2023207	block3	HumanSpeech	Pre	svy2	Object	Contrastive	r2	259.345695763557	1	on_focus	2	Contrastive on_focus
2023207	block3	HumanSpeech	Pre	kwo2	Object	Contrastive	r2	333.088055809128	2	on_focus	2	Contrastive on_focus
2023207	block3	HumanSpeech	Pre	piu2	Subject	Narrow	r2	120.97610584118	1	pre_focus	2	Narrow pre_focus
2023207	block3	HumanSpeech	Pre	tse2	Subject	Narrow	r2	106.732574091723	2	pre_focus	2	Narrow pre_focus
2023207	block3	HumanSpeech	Pre	tsap1	Verb	Narrow	r2	68.0407102850609	1	pre_focus	1	Narrow pre_focus
2023207	block3	HumanSpeech	Pre	sy1	Object	Narrow	r2	123.616693177155	1	on_focus	1	Narrow on_focus
2023207	block3	HumanSpeech	Pre	pau1	Object	Narrow	r2	308.354813051608	2	on_focus	1	Narrow on_focus
2023207	block3	HumanSpeech	Pre	suk1	Subject	Narrow	r2	79.3309508488278	1	on_focus	1	Narrow on_focus
2023207	block3	HumanSpeech	Pre	suk1	Subject	Narrow	r2	96.8229334317243	2	on_focus	1	Narrow on_focus
2023207	block3	HumanSpeech	Pre	sei2	Verb	Narrow	r2	148.31809770692	1	post_focus	2	Narrow post_focus
2023207	block3	HumanSpeech	Pre	svy2	Object	Narrow	r2	140.490996516291	1	post_focus	2	Narrow post_focus
2023207	block3	HumanSpeech	Pre	kwo2	Object	Narrow	r2	409.805588902202	2	post_focus	2	Narrow post_focus
2023207	block4	HumanSpeech	Post	pak3	Subject	Narrow	r1	87.6316452506813	1	pre_focus	3	Narrow pre_focus
2023207	block4	HumanSpeech	Post	pak3	Subject	Narrow	r1	76.7878444306973	2	pre_focus	3	Narrow pre_focus
2023207	block4	HumanSpeech	Post	tsing2	Verb	Narrow	r1	176.911976911981	1	pre_focus	2	Narrow pre_focus
2023207	block4	HumanSpeech	Post	kau2	Object	Narrow	r1	199.863258434675	1	on_focus	2	Narrow on_focus
2023207	block4	HumanSpeech	Post	tsi2	Object	Narrow	r1	398.602657437237	2	on_focus	2	Narrow on_focus
2023207	block4	HumanSpeech	Post	piu35	Subject	Narrow	r1	179.367484367489	1	pre_focus	35	Narrow pre_focus
2023207	block4	HumanSpeech	Post	mui35	Subject	Narrow	r1	148.413576183174	2	pre_focus	35	Narrow pre_focus
2023207	block4	HumanSpeech	Post	tsan3	Verb	Narrow	r1	215.881062102255	1	on_focus	3	Narrow on_focus
2023207	block4	HumanSpeech	Post	jin3	Object	Narrow	r1	233.493323255217	1	post_focus	3	Narrow post_focus
2023207	block4	HumanSpeech	Post	jin3	Object	Narrow	r1	327.222222222204	2	post_focus	3	Narrow post_focus
2023207	block4	HumanSpeech	Post	pak3	Subject	Contrastive	r1	114.33590564026	1	on_focus	3	Contrastive on_focus
2023207	block4	HumanSpeech	Post	pak3	Subject	Contrastive	r1	100.894325387955	2	on_focus	3	Contrastive on_focus
2023207	block4	HumanSpeech	Post	tsing2	Verb	Contrastive	r1	160.694444444445	1	post_focus	2	Contrastive post_focus
2023207	block4	HumanSpeech	Post	kau2	Object	Contrastive	r1	173.152977240278	1	post_focus	2	Contrastive post_focus
2023207	block4	HumanSpeech	Post	tsi2	Object	Contrastive	r1	435.655976676387	2	post_focus	2	Contrastive post_focus
2023207	block4	HumanSpeech	Post	pak3	Subject	Contrastive	r1	112.990677752578	1	pre_focus	3	Contrastive pre_focus
2023207	block4	HumanSpeech	Post	pak3	Subject	Contrastive	r1	145.501700680271	2	pre_focus	3	Contrastive pre_focus
2023207	block4	HumanSpeech	Post	tsing2	Verb	Contrastive	r1	67.040816326525	1	on_focus	2	Contrastive on_focus
2023207	block4	HumanSpeech	Post	kau2	Object	Contrastive	r1	258.756613756617	1	post_focus	2	Contrastive post_focus
2023207	block4	HumanSpeech	Post	tsi2	Object	Contrastive	r1	783.195767195764	2	post_focus	2	Contrastive post_focus
2023207	block4	HumanSpeech	Post	jan1	Subject	Narrow	r1	228.84137782097	1	pre_focus	1	Narrow pre_focus
2023207	block4	HumanSpeech	Post	jan1	Subject	Narrow	r1	204.486747079784	2	pre_focus	1	Narrow pre_focus
2023207	block4	HumanSpeech	Post	wei3	Verb	Narrow	r1	139.964726631376	1	on_focus	3	Narrow on_focus
2023207	block4	HumanSpeech	Post	tsam3	Object	Narrow	r1	466.459684649038	1	post_focus	3	Narrow post_focus
2023207	block4	HumanSpeech	Post	tsam3	Object	Narrow	r1	856.014739229011	2	post_focus	3	Narrow post_focus
2023207	block4	HumanSpeech	Post	jan1	Subject	Contrastive	r1	183.441739268801	1	pre_focus	1	Contrastive pre_focus
2023207	block4	HumanSpeech	Post	jan1	Subject	Contrastive	r1	269.165729184778	2	pre_focus	1	Contrastive pre_focus
2023207	block4	HumanSpeech	Post	wei3	Verb	Contrastive	r1	774.033523319247	1	pre_focus	3	Contrastive pre_focus
2023207	block4	HumanSpeech	Post	tsam3	Object	Contrastive	r1	265.958766648396	1	on_focus	3	Contrastive on_focus
2023207	block4	HumanSpeech	Post	tsam3	Object	Contrastive	r1	407.58566389519	2	on_focus	3	Contrastive on_focus
2023207	block4	HumanSpeech	Post	piu35	Subject	Narrow	r1	320.479969765699	1	on_focus	35	Narrow on_focus
2023207	block4	HumanSpeech	Post	mui35	Subject	Narrow	r1	272.559118885653	2	on_focus	35	Narrow on_focus
2023207	block4	HumanSpeech	Post	tsan3	Verb	Narrow	r1	347.897959183683	1	post_focus	3	Narrow post_focus
2023207	block4	HumanSpeech	Post	jin3	Object	Narrow	r1	793.969333765261	1	post_focus	3	Narrow post_focus
2023207	block4	HumanSpeech	Post	jin3	Object	Narrow	r1	802.003563330089	2	post_focus	3	Narrow post_focus
2023207	block4	HumanSpeech	Post	pak3	Subject	Broad	r1	113.302739085043	1	broad_focus	3	Broad focus
2023207	block4	HumanSpeech	Post	pak3	Subject	Broad	r1	128.125832343528	2	broad_focus	3	Broad focus
2023207	block4	HumanSpeech	Post	tsing2	Verb	Broad	r1	189.940476190486	1	broad_focus	2	Broad focus
2023207	block4	HumanSpeech	Post	kau2	Object	Broad	r1	617.029478458051	1	broad_focus	2	Broad focus
2023207	block4	HumanSpeech	Post	tsi2	Object	Broad	r1	878.966117848108	2	broad_focus	2	Broad focus
2023207	block4	HumanSpeech	Post	pak3	Subject	Contrastive	r1	102.232189579126	1	pre_focus	3	Contrastive pre_focus
2023207	block4	HumanSpeech	Post	pak3	Subject	Contrastive	r1	164.422769107631	2	pre_focus	3	Contrastive pre_focus
2023207	block4	HumanSpeech	Post	tsing2	Verb	Contrastive	r1	282.081632653075	1	pre_focus	2	Contrastive pre_focus
2023207	block4	HumanSpeech	Post	kau2	Object	Contrastive	r1	311.614512471664	1	on_focus	2	Contrastive on_focus
2023207	block4	HumanSpeech	Post	tsi2	Object	Contrastive	r1	740.739795918387	2	on_focus	2	Contrastive on_focus
2023207	block4	HumanSpeech	Post	jan1	Subject	Contrastive	r1	187.894935752098	1	on_focus	1	Contrastive on_focus
2023207	block4	HumanSpeech	Post	jan1	Subject	Contrastive	r1	187.871504157215	2	on_focus	1	Contrastive on_focus

2023207	block4	HumanSpeech	Post	wei3	Verb	Contrastive	r1	190.65111758988	1	post_focus	3	Contrastive post_focus
2023207	block4	HumanSpeech	Post	tsam3	Object	Contrastive	r1	580.260770975059	1	post_focus	3	Contrastive post_focus
2023207	block4	HumanSpeech	Post	tsam3	Object	Contrastive	r1	537.356009070294	2	post_focus	3	Contrastive post_focus
2023207	block4	HumanSpeech	Post	piu35	Subject	Contrastive	r1	131.929015084296	1	pre_focus	35	Contrastive pre_focus
2023207	block4	HumanSpeech	Post	mui35	Subject	Contrastive	r1	243.210236475562	2	pre_focus	35	Contrastive pre_focus
2023207	block4	HumanSpeech	Post	tsan3	Verb	Contrastive	r1	462.334240362793	1	on_focus	3	Contrastive on_focus
2023207	block4	HumanSpeech	Post	jin3	Object	Contrastive	r1	222.224651765487	1	post_focus	3	Contrastive post_focus
2023207	block4	HumanSpeech	Post	jin3	Object	Contrastive	r1	307.466796242295	2	post_focus	3	Contrastive post_focus
2023207	block4	HumanSpeech	Post	pak3	Subject	Narrow	r1	78.7792894935819	1	on_focus	3	Narrow on_focus
2023207	block4	HumanSpeech	Post	pak3	Subject	Narrow	r1	85.6265446253417	2	on_focus	3	Narrow on_focus
2023207	block4	HumanSpeech	Post	tsing2	Verb	Narrow	r1	166.669096209915	1	post_focus	2	Narrow post_focus
2023207	block4	HumanSpeech	Post	kau2	Object	Narrow	r1	243.56311413456	1	post_focus	2	Narrow post_focus
2023207	block4	HumanSpeech	Post	tsi2	Object	Narrow	r1	221.90539178635	2	post_focus	2	Narrow post_focus
2023207	block4	HumanSpeech	Post	jan1	Subject	Contrastive	r1	253.798185941037	1	pre_focus	1	Contrastive pre_focus
2023207	block4	HumanSpeech	Post	jan1	Subject	Contrastive	r1	180.595238095265	2	pre_focus	1	Contrastive pre_focus
2023207	block4	HumanSpeech	Post	wei3	Verb	Contrastive	r1	206.424792139074	1	on_focus	3	Contrastive on_focus
2023207	block4	HumanSpeech	Post	tsam3	Object	Contrastive	r1	179.462666018793	1	post_focus	3	Contrastive post_focus
2023207	block4	HumanSpeech	Post	tsam3	Object	Contrastive	r1	215.598581312861	2	post_focus	3	Contrastive post_focus
2023207	block4	HumanSpeech	Post	jan1	Subject	Broad	r1	114.895726495718	1	broad_focus	1	Broad focus
2023207	block4	HumanSpeech	Post	jan1	Subject	Broad	r1	119.867913832223	2	broad_focus	1	Broad focus
2023207	block4	HumanSpeech	Post	wei3	Verb	Broad	r1	126.891752092547	1	broad_focus	3	Broad focus
2023207	block4	HumanSpeech	Post	tsam3	Object	Broad	r1	104.543650793659	1	broad_focus	3	Broad focus
2023207	block4	HumanSpeech	Post	tsam3	Object	Broad	r1	396.889644746807	2	broad_focus	3	Broad focus
2023207	block4	HumanSpeech	Post	pak3	Subject	Narrow	r1	153.706958091874	1	pre_focus	3	Narrow pre_focus
2023207	block4	HumanSpeech	Post	pak3	Subject	Narrow	r1	154.193391642366	2	pre_focus	3	Narrow pre_focus
2023207	block4	HumanSpeech	Post	tsing2	Verb	Narrow	r1	406.877227081281	1	on_focus	2	Narrow on_focus
2023207	block4	HumanSpeech	Post	kau2	Object	Narrow	r1	249.243764172348	1	post_focus	2	Narrow post_focus
2023207	block4	HumanSpeech	Post	tsi2	Object	Narrow	r1	356.062736205587	2	post_focus	2	Narrow post_focus
2023207	block4	HumanSpeech	Post	piu35	Subject	Contrastive	r1	288.782123960686	1	pre_focus	35	Contrastive pre_focus
2023207	block4	HumanSpeech	Post	mui35	Subject	Contrastive	r1	219.573696145119	2	pre_focus	35	Contrastive pre_focus
2023207	block4	HumanSpeech	Post	tsan3	Verb	Contrastive	r1	406.870748299326	1	pre_focus	3	Contrastive pre_focus
2023207	block4	HumanSpeech	Post	jin3	Object	Contrastive	r1	283.383219954629	1	on_focus	3	Contrastive on_focus
2023207	block4	HumanSpeech	Post	jin3	Object	Contrastive	r1	523.900226757348	2	on_focus	3	Contrastive on_focus
2023207	block4	HumanSpeech	Post	jan1	Subject	Narrow	r1	204.421768707505	1	pre_focus	1	Narrow pre_focus
2023207	block4	HumanSpeech	Post	jan1	Subject	Narrow	r1	266.834575099864	2	pre_focus	1	Narrow pre_focus
2023207	block4	HumanSpeech	Post	wei3	Verb	Narrow	r1	1410.20231796421	1	pre_focus	3	Narrow pre_focus
2023207	block4	HumanSpeech	Post	tsam3	Object	Narrow	r1	172.704931972788	1	on_focus	3	Narrow on_focus
2023207	block4	HumanSpeech	Post	tsam3	Object	Narrow	r1	283.442392830153	2	on_focus	3	Narrow on_focus
2023207	block4	HumanSpeech	Post	piu35	Subject	Contrastive	r1	267.277399848837	1	on_focus	35	Contrastive on_focus
2023207	block4	HumanSpeech	Post	mui35	Subject	Contrastive	r1	223.414965986393	2	on_focus	35	Contrastive on_focus
2023207	block4	HumanSpeech	Post	tsan3	Verb	Contrastive	r1	263.121005978149	1	post_focus	3	Contrastive post_focus
2023207	block4	HumanSpeech	Post	jin3	Object	Contrastive	r1	542.492063492091	1	post_focus	3	Contrastive post_focus
2023207	block4	HumanSpeech	Post	jin3	Object	Contrastive	r1	421.303854875276	2	post_focus	3	Contrastive post_focus
2023207	block4	HumanSpeech	Post	piu35	Subject	Broad	r1	167.909297052148	1	broad_focus	35	Broad focus
2023207	block4	HumanSpeech	Post	mui35	Subject	Broad	r1	232.28539034659	2	broad_focus	35	Broad focus
2023207	block4	HumanSpeech	Post	tsan3	Verb	Broad	r1	261.668556311406	1	broad_focus	3	Broad focus
2023207	block4	HumanSpeech	Post	jin3	Object	Broad	r1	601.946334089178	1	broad_focus	3	Broad focus
2023207	block4	HumanSpeech	Post	jin3	Object	Broad	r1	508.916099773245	2	broad_focus	3	Broad focus
2023207	block4	HumanSpeech	Post	jan1	Subject	Narrow	r1	282.656840513994	1	on_focus	1	Narrow on_focus
2023207	block4	HumanSpeech	Post	jan1	Subject	Narrow	r1	210.379818594106	2	on_focus	1	Narrow on_focus
2023207	block4	HumanSpeech	Post	wei3	Verb	Narrow	r1	170.820105820155	1	post_focus	3	Narrow post_focus
2023207	block4	HumanSpeech	Post	tsam3	Object	Narrow	r1	433.9520570133	1	post_focus	3	Narrow post_focus
2023207	block4	HumanSpeech	Post	tsam3	Object	Narrow	r1	489.219104308347	2	post_focus	3	Narrow post_focus
2023207	block4	HumanSpeech	Post	piu35	Subject	Narrow	r1	115.942302847088	1	pre_focus	35	Narrow pre_focus
2023207	block4	HumanSpeech	Post	mui35	Subject	Narrow	r1	283.360544217658	2	pre_focus	35	Narrow pre_focus
2023207	block4	HumanSpeech	Post	tsan3	Verb	Narrow	r1	233.835222978087	1	pre_focus	3	Narrow pre_focus
2023207	block4	HumanSpeech	Post	jin3	Object	Narrow	r1	226.341647770198	1	on_focus	3	Narrow on_focus
2023207	block4	HumanSpeech	Post	jin3	Object	Narrow	r1	410.456034265565	2	on_focus	3	Narrow on_focus
2023207	block4	HumanSpeech	Post	piu35	Subject	Contrastive	r2	180.735341755735	1	pre_focus	35	Contrastive pre_focus
2023207	block4	HumanSpeech	Post	mui35	Subject	Contrastive	r2	259.845271441861	2	pre_focus	35	Contrastive pre_focus
2023207	block4	HumanSpeech	Post	tsan3	Verb	Contrastive	r2	217.323390894876	1	pre_focus	3	Contrastive pre_focus
2023207	block4	HumanSpeech	Post	jin3	Object	Contrastive	r2	749.015495086951	1	on_focus	3	Contrastive on_focus

2023207	block4	HumanSpeech	Post	jin3	Object	Contrastive	r2	379.879062736165	2	on_focus	3	Contrastive on_focus
2023207	block4	HumanSpeech	Post	pak3	Subject	Broad	r2	104.005794910563	1	broad_focus	3	Broad focus
2023207	block4	HumanSpeech	Post	pak3	Subject	Broad	r2	104.022423784329	2	broad_focus	3	Broad focus
2023207	block4	HumanSpeech	Post	tsing2	Verb	Broad	r2	134.946334089193	1	broad_focus	2	Broad focus
2023207	block4	HumanSpeech	Post	kau2	Object	Broad	r2	135.855008670148	1	broad_focus	2	Broad focus
2023207	block4	HumanSpeech	Post	tsi2	Object	Broad	r2	424.635928445412	2	broad_focus	2	Broad focus
2023207	block4	HumanSpeech	Post	jan1	Subject	Narrow	r2	50.5833564355953	1	pre_focus	1	Narrow pre_focus
2023207	block4	HumanSpeech	Post	jan1	Subject	Narrow	r2	48.2477676704889	2	pre_focus	1	Narrow pre_focus
2023207	block4	HumanSpeech	Post	wei3	Verb	Narrow	r2	107.881533565831	1	on_focus	3	Narrow on_focus
2023207	block4	HumanSpeech	Post	tsam3	Object	Narrow	r2	110.317460317447	1	post_focus	3	Narrow post_focus
2023207	block4	HumanSpeech	Post	tsam3	Object	Narrow	r2	123.925601986798	2	post_focus	3	Narrow post_focus
2023207	block4	HumanSpeech	Post	jan1	Subject	Contrastive	r2	111.248632786442	1	pre_focus	1	Contrastive pre_focus
2023207	block4	HumanSpeech	Post	jan1	Subject	Contrastive	r2	87.3368283974969	2	pre_focus	1	Contrastive pre_focus
2023207	block4	HumanSpeech	Post	wei3	Verb	Contrastive	r2	91.8087679516475	1	pre_focus	3	Contrastive pre_focus
2023207	block4	HumanSpeech	Post	tsam3	Object	Contrastive	r2	99.9977324262886	1	on_focus	3	Contrastive on_focus
2023207	block4	HumanSpeech	Post	tsam3	Object	Contrastive	r2	328.433754454124	2	on_focus	3	Contrastive on_focus
2023207	block4	HumanSpeech	Post	jan1	Subject	Broad	r2	165.129623277892	1	broad_focus	1	Broad focus
2023207	block4	HumanSpeech	Post	jan1	Subject	Broad	r2	120.534677733019	2	broad_focus	1	Broad focus
2023207	block4	HumanSpeech	Post	wei3	Verb	Broad	r2	258.422373962105	1	broad_focus	3	Broad focus
2023207	block4	HumanSpeech	Post	tsam3	Object	Broad	r2	108.787603930466	1	broad_focus	3	Broad focus
2023207	block4	HumanSpeech	Post	tsam3	Object	Broad	r2	244.425547997025	2	broad_focus	3	Broad focus
2023207	block4	HumanSpeech	Post	piu35	Subject	Contrastive	r2	85.1155440071238	1	pre_focus	35	Contrastive pre_focus
2023207	block4	HumanSpeech	Post	mui35	Subject	Contrastive	r2	129.020624122631	2	pre_focus	35	Contrastive pre_focus
2023207	block4	HumanSpeech	Post	tsan3	Verb	Contrastive	r2	173.056044256782	1	on_focus	3	Contrastive on_focus
2023207	block4	HumanSpeech	Post	jin3	Object	Contrastive	r2	208.025521319598	1	post_focus	3	Contrastive post_focus
2023207	block4	HumanSpeech	Post	jin3	Object	Contrastive	r2	204.918535315358	2	post_focus	3	Contrastive post_focus
2023207	block4	HumanSpeech	Post	jan1	Subject	Contrastive	r2	86.0837467819238	1	pre_focus	1	Contrastive pre_focus
2023207	block4	HumanSpeech	Post	jan1	Subject	Contrastive	r2	89.4473110882359	2	pre_focus	1	Contrastive pre_focus
2023207	block4	HumanSpeech	Post	wei3	Verb	Contrastive	r2	194.480158730187	1	on_focus	3	Contrastive on_focus
2023207	block4	HumanSpeech	Post	tsam3	Object	Contrastive	r2	113.06873398712	1	post_focus	3	Contrastive post_focus
2023207	block4	HumanSpeech	Post	tsam3	Object	Contrastive	r2	150.617010089036	2	post_focus	3	Contrastive post_focus
2023207	block4	HumanSpeech	Post	pak3	Subject	Contrastive	r2	69.8858697672335	1	pre_focus	3	Contrastive pre_focus
2023207	block4	HumanSpeech	Post	pak3	Subject	Contrastive	r2	60.0920080262313	2	pre_focus	3	Contrastive pre_focus
2023207	block4	HumanSpeech	Post	tsing2	Verb	Contrastive	r2	49.3832199546773	1	pre_focus	2	Contrastive pre_focus
2023207	block4	HumanSpeech	Post	kau2	Object	Contrastive	r2	101.257328328131	1	on_focus	2	Contrastive on_focus
2023207	block4	HumanSpeech	Post	tsi2	Object	Contrastive	r2	212.191043083919	2	on_focus	2	Contrastive on_focus
2023207	block4	HumanSpeech	Post	pak3	Subject	Narrow	r2	103.767006802741	1	pre_focus	3	Narrow pre_focus
2023207	block4	HumanSpeech	Post	pak3	Subject	Narrow	r2	105.818060557567	2	pre_focus	3	Narrow pre_focus
2023207	block4	HumanSpeech	Post	tsing2	Verb	Narrow	r2	175.660745779794	1	pre_focus	2	Narrow pre_focus
2023207	block4	HumanSpeech	Post	kau2	Object	Narrow	r2	109.001927437646	1	on_focus	2	Narrow on_focus
2023207	block4	HumanSpeech	Post	tsi2	Object	Narrow	r2	92.533286819048	2	on_focus	2	Narrow on_focus
2023207	block4	HumanSpeech	Post	piu35	Subject	Narrow	r2	73.1034676626336	1	pre_focus	35	Narrow pre_focus
2023207	block4	HumanSpeech	Post	mui35	Subject	Narrow	r2	61.1728829462663	2	pre_focus	35	Narrow pre_focus
2023207	block4	HumanSpeech	Post	tsan3	Verb	Narrow	r2	134.490881272768	1	on_focus	3	Narrow on_focus
2023207	block4	HumanSpeech	Post	jin3	Object	Narrow	r2	123.826817977601	1	post_focus	3	Narrow post_focus
2023207	block4	HumanSpeech	Post	jin3	Object	Narrow	r2	211.393094872392	2	post_focus	3	Narrow post_focus
2023207	block4	HumanSpeech	Post	pak3	Subject	Narrow	r2	76.7138083303394	1	on_focus	3	Narrow on_focus
2023207	block4	HumanSpeech	Post	pak3	Subject	Narrow	r2	69.6970754113408	2	on_focus	3	Narrow on_focus
2023207	block4	HumanSpeech	Post	tsing2	Verb	Narrow	r2	92.7634164776805	1	post_focus	2	Narrow post_focus
2023207	block4	HumanSpeech	Post	kau2	Object	Narrow	r2	141.388888888855	1	post_focus	2	Narrow post_focus
2023207	block4	HumanSpeech	Post	tsi2	Object	Narrow	r2	332.59016304936	2	post_focus	2	Narrow post_focus
2023207	block4	HumanSpeech	Post	piu35	Subject	Narrow	r2	86.9609208205588	1	pre_focus	35	Narrow pre_focus
2023207	block4	HumanSpeech	Post	mui35	Subject	Narrow	r2	80.4039640547671	2	pre_focus	35	Narrow pre_focus
2023207	block4	HumanSpeech	Post	tsan3	Verb	Narrow	r2	135.628598914309	1	pre_focus	3	Narrow pre_focus
2023207	block4	HumanSpeech	Post	jin3	Object	Narrow	r2	189.747417485478	1	on_focus	3	Narrow on_focus
2023207	block4	HumanSpeech	Post	jin3	Object	Narrow	r2	188.057616986157	2	on_focus	3	Narrow on_focus
2023207	block4	HumanSpeech	Post	pak3	Subject	Contrastive	r2	49.1089480055393	1	pre_focus	3	Contrastive pre_focus
2023207	block4	HumanSpeech	Post	pak3	Subject	Contrastive	r2	60.1671608814627	2	pre_focus	3	Contrastive pre_focus
2023207	block4	HumanSpeech	Post	tsing2	Verb	Contrastive	r2	72.8960986103857	1	on_focus	2	Contrastive on_focus
2023207	block4	HumanSpeech	Post	kau2	Object	Contrastive	r2	110.887188208608	1	post_focus	2	Contrastive post_focus
2023207	block4	HumanSpeech	Post	tsi2	Object	Contrastive	r2	109.380102040802	2	post_focus	2	Contrastive post_focus
2023207	block4	HumanSpeech	Post	piu35	Subject	Narrow	r2	162.811791383206	1	on_focus	35	Narrow on_focus

**2023207**	block4	HumanSpeech	Post	mui35	Subject	Narrow	r2	140.221088435339	2	on_focus	35	Narrow on_focus
**2023207**	block4	HumanSpeech	Post	tsan3	Verb	Narrow	r2	204.817838246413	1	post_focus	3	Narrow post_focus
**2023207**	block4	HumanSpeech	Post	jin3	Object	Narrow	r2	102.274502393527	1	post_focus	3	Narrow post_focus
**2023207**	block4	HumanSpeech	Post	jin3	Object	Narrow	r2	108.544973544952	2	post_focus	3	Narrow post_focus
**2023207**	block4	HumanSpeech	Post	piu35	Subject	Broad	r2	124.749433106558	1	broad_focus	35	Broad focus
**2023207**	block4	HumanSpeech	Post	mui35	Subject	Broad	r2	131.202623906688	2	broad_focus	35	Broad focus
**2023207**	block4	HumanSpeech	Post	tsan3	Verb	Broad	r2	120.084353741504	1	broad_focus	3	Broad focus
**2023207**	block4	HumanSpeech	Post	jin3	Object	Broad	r2	230.551776266054	1	broad_focus	3	Broad focus
**2023207**	block4	HumanSpeech	Post	jin3	Object	Broad	r2	197.451247165532	2	broad_focus	3	Broad focus
**2023207**	block4	HumanSpeech	Post	pak3	Subject	Contrastive	r2	98.9072746215243	1	on_focus	3	Contrastive on_focus
**2023207**	block4	HumanSpeech	Post	pak3	Subject	Contrastive	r2	183.86937155816	2	on_focus	3	Contrastive on_focus
**2023207**	block4	HumanSpeech	Post	tsing2	Verb	Contrastive	r2	267.85525321236	1	post_focus	2	Contrastive post_focus
**2023207**	block4	HumanSpeech	Post	kau2	Object	Contrastive	r2	249.218559218605	1	post_focus	2	Contrastive post_focus
**2023207**	block4	HumanSpeech	Post	tsi2	Object	Contrastive	r2	648.073372206056	2	post_focus	2	Contrastive post_focus
**2023207**	block4	HumanSpeech	Post	jan1	Subject	Contrastive	r2	143.060252672456	1	on_focus	1	Contrastive on_focus
**2023207**	block4	HumanSpeech	Post	jan1	Subject	Contrastive	r2	150.830999066272	2	on_focus	1	Contrastive on_focus
**2023207**	block4	HumanSpeech	Post	wei3	Verb	Contrastive	r2	131.729564841805	1	post_focus	3	Contrastive post_focus
**2023207**	block4	HumanSpeech	Post	tsam3	Object	Contrastive	r2	163.36889301175	1	post_focus	3	Contrastive post_focus
**2023207**	block4	HumanSpeech	Post	tsam3	Object	Contrastive	r2	267.066731454463	2	post_focus	3	Contrastive post_focus
**2023207**	block4	HumanSpeech	Post	pak3	Subject	Narrow	r2	100.578128221002	1	pre_focus	3	Narrow pre_focus
**2023207**	block4	HumanSpeech	Post	pak3	Subject	Narrow	r2	94.8917233560564	2	pre_focus	3	Narrow pre_focus
**2023207**	block4	HumanSpeech	Post	tsing2	Verb	Narrow	r2	81.4172335601029	1	on_focus	2	Narrow on_focus
**2023207**	block4	HumanSpeech	Post	kau2	Object	Narrow	r2	403.703703703684	1	post_focus	2	Narrow post_focus
**2023207**	block4	HumanSpeech	Post	tsi2	Object	Narrow	r2	294.441580140813	2	post_focus	2	Narrow post_focus
**2023207**	block4	HumanSpeech	Post	jan1	Subject	Narrow	r2	175.725443616557	1	pre_focus	1	Narrow pre_focus
**2023207**	block4	HumanSpeech	Post	jan1	Subject	Narrow	r2	225.548958977527	2	pre_focus	1	Narrow pre_focus
**2023207**	block4	HumanSpeech	Post	wei3	Verb	Narrow	r2	149.246976568406	1	pre_focus	3	Narrow pre_focus
**2023207**	block4	HumanSpeech	Post	tsam3	Object	Narrow	r2	158.746220710498	1	on_focus	3	Narrow on_focus
**2023207**	block4	HumanSpeech	Post	tsam3	Object	Narrow	r2	226.358654572891	2	on_focus	3	Narrow on_focus
**2023207**	block4	HumanSpeech	Post	piu35	Subject	Contrastive	r2	77.5931324911312	1	on_focus	35	Contrastive on_focus
**2023207**	block4	HumanSpeech	Post	mui35	Subject	Contrastive	r2	242.214663643267	2	on_focus	35	Contrastive on_focus
**2023207**	block4	HumanSpeech	Post	tsan3	Verb	Contrastive	r2	226.369047619073	1	post_focus	3	Contrastive post_focus
**2023207**	block4	HumanSpeech	Post	jin3	Object	Contrastive	r2	249.891156462525	1	post_focus	3	Contrastive post_focus
**2023207**	block4	HumanSpeech	Post	jin3	Object	Contrastive	r2	339.202569916893	2	post_focus	3	Contrastive post_focus
**2023207**	block4	HumanSpeech	Post	jan1	Subject	Narrow	r2	382.576894005467	1	on_focus	1	Narrow on_focus
**2023207**	block4	HumanSpeech	Post	jan1	Subject	Narrow	r2	398.742756361798	2	on_focus	1	Narrow on_focus
**2023207**	block4	HumanSpeech	Post	wei3	Verb	Narrow	r2	163.900893690823	1	post_focus	3	Narrow post_focus
**2023207**	block4	HumanSpeech	Post	tsam3	Object	Narrow	r2	199.621711118255	1	post_focus	3	Narrow post_focus
**2023207**	block4	HumanSpeech	Post	tsam3	Object	Narrow	r2	308.108843537411	2	post_focus	3	Narrow post_focus
**2023207**	block4	HumanSpeech	Pre	piu35	Subject	Contrastive	r1	194.096418118022	1	pre_focus	35	Contrastive pre_focus
**2023207**	block4	HumanSpeech	Pre	mui35	Subject	Contrastive	r1	197.265054169861	2	pre_focus	35	Contrastive pre_focus
**2023207**	block4	HumanSpeech	Pre	tsan3	Verb	Contrastive	r1	296.747032146186	1	pre_focus	3	Contrastive pre_focus
**2023207**	block4	HumanSpeech	Pre	jin3	Object	Contrastive	r1	259.830556208783	1	on_focus	3	Contrastive on_focus
**2023207**	block4	HumanSpeech	Pre	jin3	Object	Contrastive	r1	407.039016664839	2	on_focus	3	Contrastive on_focus
**2023207**	block4	HumanSpeech	Pre	jan1	Subject	Broad	r1	197.865889212835	1	broad_focus	1	Broad focus
**2023207**	block4	HumanSpeech	Pre	jan1	Subject	Broad	r1	152.68017976814	2	broad_focus	1	Broad focus
**2023207**	block4	HumanSpeech	Pre	wei3	Verb	Broad	r1	138.235638699882	1	broad_focus	3	Broad focus
**2023207**	block4	HumanSpeech	Pre	tsam3	Object	Broad	r1	120.547119641515	1	broad_focus	3	Broad focus
**2023207**	block4	HumanSpeech	Pre	tsam3	Object	Broad	r1	602.459687578744	2	broad_focus	3	Broad focus
**2023207**	block4	HumanSpeech	Pre	pak3	Subject	Narrow	r1	138.871072238373	1	on_focus	3	Narrow on_focus
**2023207**	block4	HumanSpeech	Pre	pak3	Subject	Narrow	r1	160.286350865874	2	on_focus	3	Narrow on_focus
**2023207**	block4	HumanSpeech	Pre	tsing2	Verb	Narrow	r1	209.210419210422	1	post_focus	2	Narrow post_focus
**2023207**	block4	HumanSpeech	Pre	kau2	Object	Narrow	r1	265.088648986193	1	post_focus	2	Narrow post_focus
**2023207**	block4	HumanSpeech	Pre	tsi2	Object	Narrow	r1	371.870111042085	2	post_focus	2	Narrow post_focus
**2023207**	block4	HumanSpeech	Pre	piu35	Subject	Broad	r1	41.3496183275015	1	broad_focus	35	Broad focus
**2023207**	block4	HumanSpeech	Pre	mui35	Subject	Broad	r1	154.485466914082	2	broad_focus	35	Broad focus
**2023207**	block4	HumanSpeech	Pre	tsan3	Verb	Broad	r1	216.825505192105	1	broad_focus	3	Broad focus
**2023207**	block4	HumanSpeech	Pre	jin3	Object	Broad	r1	231.776369888621	1	broad_focus	3	Broad focus
**2023207**	block4	HumanSpeech	Pre	jin3	Object	Broad	r1	499.513068385227	2	broad_focus	3	Broad focus
**2023207**	block4	HumanSpeech	Pre	piu35	Subject	Contrastive	r1	210.733833763356	1	pre_focus	35	Contrastive pre_focus
**2023207**	block4	HumanSpeech	Pre	mui35	Subject	Contrastive	r1	263.791848002143	2	pre_focus	35	Contrastive pre_focus
**2023207**	block4	HumanSpeech	Pre	tsan3	Verb	Contrastive	r1	289.502796674242	1	on_focus	3	Contrastive on_focus

**2023207**	block4	HumanSpeech	Pre	jin3	Object	Contrastive	r1	368.033736750533	1	post_focus	3	Contrastive post_focus
**2023207**	block4	HumanSpeech	Pre	jin3	Object	Contrastive	r1	333.413570556388	2	post_focus	3	Contrastive post_focus
**2023207**	block4	HumanSpeech	Pre	jan1	Subject	Narrow	r1	210.88253968253	1	pre_focus	1	Narrow pre_focus
**2023207**	block4	HumanSpeech	Pre	jan1	Subject	Narrow	r1	251.666726655344	2	pre_focus	1	Narrow pre_focus
**2023207**	block4	HumanSpeech	Pre	wei3	Verb	Narrow	r1	296.998176082013	1	on_focus	3	Narrow on_focus
**2023207**	block4	HumanSpeech	Pre	tsam3	Object	Narrow	r1	211.602583062188	1	post_focus	3	Narrow post_focus
**2023207**	block4	HumanSpeech	Pre	tsam3	Object	Narrow	r1	235.503350969964	2	post_focus	3	Narrow post_focus
**2023207**	block4	HumanSpeech	Pre	jan1	Subject	Narrow	r1	245.377127281927	1	pre_focus	1	Narrow pre_focus
**2023207**	block4	HumanSpeech	Pre	jan1	Subject	Narrow	r1	198.244374672925	2	pre_focus	1	Narrow pre_focus
**2023207**	block4	HumanSpeech	Pre	wei3	Verb	Narrow	r1	216.350574712635	1	pre_focus	3	Narrow pre_focus
**2023207**	block4	HumanSpeech	Pre	tsam3	Object	Narrow	r1	233.2286277408	1	on_focus	3	Narrow on_focus
**2023207**	block4	HumanSpeech	Pre	tsam3	Object	Narrow	r1	255.7179138322	2	on_focus	3	Narrow on_focus
**2023207**	block4	HumanSpeech	Pre	piu35	Subject	Narrow	r1	141.299703241998	1	pre_focus	35	Narrow pre_focus
**2023207**	block4	HumanSpeech	Pre	mui35	Subject	Narrow	r1	296.820987654314	2	pre_focus	35	Narrow pre_focus
**2023207**	block4	HumanSpeech	Pre	tsan3	Verb	Narrow	r1	235.667530504315	1	on_focus	3	Narrow on_focus
**2023207**	block4	HumanSpeech	Pre	jin3	Object	Narrow	r1	289.615142353227	1	post_focus	3	Narrow post_focus
**2023207**	block4	HumanSpeech	Pre	jin3	Object	Narrow	r1	372.601857947416	2	post_focus	3	Narrow post_focus
**2023207**	block4	HumanSpeech	Pre	piu35	Subject	Narrow	r1	180.68119876159	1	pre_focus	35	Narrow pre_focus
**2023207**	block4	HumanSpeech	Pre	mui35	Subject	Narrow	r1	263.915257531607	2	pre_focus	35	Narrow pre_focus
**2023207**	block4	HumanSpeech	Pre	tsan3	Verb	Narrow	r1	225.790511076298	1	pre_focus	3	Narrow pre_focus
**2023207**	block4	HumanSpeech	Pre	jin3	Object	Narrow	r1	273.096362108049	1	on_focus	3	Narrow on_focus
**2023207**	block4	HumanSpeech	Pre	jin3	Object	Narrow	r1	298.157023382828	2	on_focus	3	Narrow on_focus
**2023207**	block4	HumanSpeech	Pre	jan1	Subject	Contrastive	r1	179.446963970747	1	pre_focus	1	Contrastive pre_focus
**2023207**	block4	HumanSpeech	Pre	jan1	Subject	Contrastive	r1	188.759508262592	2	pre_focus	1	Contrastive pre_focus
**2023207**	block4	HumanSpeech	Pre	wei3	Verb	Contrastive	r1	239.00251139878	1	on_focus	3	Contrastive on_focus
**2023207**	block4	HumanSpeech	Pre	tsam3	Object	Contrastive	r1	225.371607228738	1	post_focus	3	Contrastive post_focus
**2023207**	block4	HumanSpeech	Pre	tsam3	Object	Contrastive	r1	568.847565341969	2	post_focus	3	Contrastive post_focus
**2023207**	block4	HumanSpeech	Pre	pak3	Subject	Narrow	r1	124.331065759634	1	pre_focus	3	Narrow pre_focus
**2023207**	block4	HumanSpeech	Pre	pak3	Subject	Narrow	r1	130.748428159109	2	pre_focus	3	Narrow pre_focus
**2023207**	block4	HumanSpeech	Pre	tsing2	Verb	Narrow	r1	212.795918367306	1	on_focus	2	Narrow on_focus
**2023207**	block4	HumanSpeech	Pre	kau2	Object	Narrow	r1	234.321853741505	1	post_focus	2	Narrow post_focus
**2023207**	block4	HumanSpeech	Pre	tsi2	Object	Narrow	r1	201.95454993592	2	post_focus	2	Narrow post_focus
**2023207**	block4	HumanSpeech	Pre	jan1	Subject	Contrastive	r1	239.164202254983	1	on_focus	1	Contrastive on_focus
**2023207**	block4	HumanSpeech	Pre	jan1	Subject	Contrastive	r1	181.311930699735	2	on_focus	1	Contrastive on_focus
**2023207**	block4	HumanSpeech	Pre	wei3	Verb	Contrastive	r1	235.222673999374	1	post_focus	3	Contrastive post_focus
**2023207**	block4	HumanSpeech	Pre	tsam3	Object	Contrastive	r1	238.321537765444	1	post_focus	3	Contrastive post_focus
**2023207**	block4	HumanSpeech	Pre	tsam3	Object	Contrastive	r1	294.759950884156	2	post_focus	3	Contrastive post_focus
**2023207**	block4	HumanSpeech	Pre	pak3	Subject	Broad	r1	123.709671002928	1	broad_focus	3	Broad focus
**2023207**	block4	HumanSpeech	Pre	pak3	Subject	Broad	r1	132.056621287973	2	broad_focus	3	Broad focus
**2023207**	block4	HumanSpeech	Pre	tsing2	Verb	Broad	r1	201.458553791895	1	broad_focus	2	Broad focus
**2023207**	block4	HumanSpeech	Pre	kau2	Object	Broad	r1	293.7760089373	1	broad_focus	2	Broad focus
**2023207**	block4	HumanSpeech	Pre	tsi2	Object	Broad	r1	252.956191735962	2	broad_focus	2	Broad focus
**2023207**	block4	HumanSpeech	Pre	jan1	Subject	Narrow	r1	214.300368943213	1	on_focus	1	Narrow on_focus
**2023207**	block4	HumanSpeech	Pre	jan1	Subject	Narrow	r1	190.317679711256	2	on_focus	1	Narrow on_focus
**2023207**	block4	HumanSpeech	Pre	wei3	Verb	Narrow	r1	226.070213799858	1	post_focus	3	Narrow post_focus
**2023207**	block4	HumanSpeech	Pre	tsam3	Object	Narrow	r1	165.539103584706	1	post_focus	3	Narrow post_focus
**2023207**	block4	HumanSpeech	Pre	tsam3	Object	Narrow	r1	387.318994938028	2	post_focus	3	Narrow post_focus
**2023207**	block4	HumanSpeech	Pre	pak3	Subject	Contrastive	r1	141.956945171216	1	on_focus	3	Contrastive on_focus
**2023207**	block4	HumanSpeech	Pre	pak3	Subject	Contrastive	r1	141.433898576793	2	on_focus	3	Contrastive on_focus
**2023207**	block4	HumanSpeech	Pre	tsing2	Verb	Contrastive	r1	249.138478379791	1	post_focus	2	Contrastive post_focus
**2023207**	block4	HumanSpeech	Pre	kau2	Object	Contrastive	r1	250.152908010079	1	post_focus	2	Contrastive post_focus
**2023207**	block4	HumanSpeech	Pre	tsi2	Object	Contrastive	r1	233.772486772466	2	post_focus	2	Contrastive post_focus
**2023207**	block4	HumanSpeech	Pre	pak3	Subject	Contrastive	r1	125.220521541962	1	pre_focus	3	Contrastive pre_focus
**2023207**	block4	HumanSpeech	Pre	pak3	Subject	Contrastive	r1	142.339748091956	2	pre_focus	3	Contrastive pre_focus
**2023207**	block4	HumanSpeech	Pre	tsing2	Verb	Contrastive	r1	203.22339036619	1	on_focus	2	Contrastive on_focus
**2023207**	block4	HumanSpeech	Pre	kau2	Object	Contrastive	r1	302.516608982785	1	post_focus	2	Contrastive post_focus
**2023207**	block4	HumanSpeech	Pre	tsi2	Object	Contrastive	r1	255.965338516319	2	post_focus	2	Contrastive post_focus
**2023207**	block4	HumanSpeech	Pre	pak3	Subject	Contrastive	r1	144.965940677366	1	pre_focus	3	Contrastive pre_focus
**2023207**	block4	HumanSpeech	Pre	pak3	Subject	Contrastive	r1	136.245197588494	2	pre_focus	3	Contrastive pre_focus
**2023207**	block4	HumanSpeech	Pre	tsing2	Verb	Contrastive	r1	194.157269299637	1	pre_focus	2	Contrastive pre_focus
**2023207**	block4	HumanSpeech	Pre	kau2	Object	Contrastive	r1	330.084028949557	1	on_focus	2	Contrastive on_focus
**2023207**	block4	HumanSpeech	Pre	tsi2	Object	Contrastive	r1	556.610959818954	2	on_focus	2	Contrastive on_focus

**2023207**	block4	HumanSpeech	Pre	jan1	Subject	Contrastive	r1	243.128518594347	1	pre_focus	1	Contrastive pre_focus
**2023207**	block4	HumanSpeech	Pre	jan1	Subject	Contrastive	r1	193.491752154728	2	pre_focus	1	Contrastive pre_focus
**2023207**	block4	HumanSpeech	Pre	wei3	Verb	Contrastive	r1	235.031711412262	1	pre_focus	3	Contrastive pre_focus
**2023207**	block4	HumanSpeech	Pre	tsam3	Object	Contrastive	r1	249.263466392904	1	on_focus	3	Contrastive on_focus
**2023207**	block4	HumanSpeech	Pre	tsam3	Object	Contrastive	r1	344.147500269969	2	on_focus	3	Contrastive on_focus
**2023207**	block4	HumanSpeech	Pre	piu35	Subject	Contrastive	r1	138.776302635733	1	on_focus	35	Contrastive on_focus
**2023207**	block4	HumanSpeech	Pre	mui35	Subject	Contrastive	r1	248.79482227675	2	on_focus	35	Contrastive on_focus
**2023207**	block4	HumanSpeech	Pre	tsan3	Verb	Contrastive	r1	230.866717057211	1	post_focus	3	Contrastive post_focus
**2023207**	block4	HumanSpeech	Pre	jin3	Object	Contrastive	r1	296.123859464387	1	post_focus	3	Contrastive post_focus
**2023207**	block4	HumanSpeech	Pre	jin3	Object	Contrastive	r1	369.516533886269	2	post_focus	3	Contrastive post_focus
**2023207**	block4	HumanSpeech	Pre	piu35	Subject	Narrow	r1	181.598816397241	1	on_focus	35	Narrow on_focus
**2023207**	block4	HumanSpeech	Pre	mui35	Subject	Narrow	r1	220.695569232987	2	on_focus	35	Narrow on_focus
**2023207**	block4	HumanSpeech	Pre	tsan3	Verb	Narrow	r1	219.123803402113	1	post_focus	3	Narrow post_focus
**2023207**	block4	HumanSpeech	Pre	jin3	Object	Narrow	r1	271.846011131743	1	post_focus	3	Narrow post_focus
**2023207**	block4	HumanSpeech	Pre	jin3	Object	Narrow	r1	343.774342060101	2	post_focus	3	Narrow post_focus
**2023207**	block4	HumanSpeech	Pre	pak3	Subject	Narrow	r1	111.846984076692	1	pre_focus	3	Narrow pre_focus
**2023207**	block4	HumanSpeech	Pre	pak3	Subject	Narrow	r1	101.890532376501	2	pre_focus	3	Narrow pre_focus
**2023207**	block4	HumanSpeech	Pre	tsing2	Verb	Narrow	r1	131.732023727352	1	pre_focus	2	Narrow pre_focus
**2023207**	block4	HumanSpeech	Pre	kau2	Object	Narrow	r1	169.953765609364	1	on_focus	2	Narrow on_focus
**2023207**	block4	HumanSpeech	Pre	tsi2	Object	Narrow	r1	326.891945183831	2	on_focus	2	Narrow on_focus
**2023207**	block4	HumanSpeech	Pre	jan1	Subject	Narrow	r2	257.685455134379	1	pre_focus	1	Narrow pre_focus
**2023207**	block4	HumanSpeech	Pre	jan1	Subject	Narrow	r2	272.016647829446	2	pre_focus	1	Narrow pre_focus
**2023207**	block4	HumanSpeech	Pre	wei3	Verb	Narrow	r2	314.149774503676	1	pre_focus	3	Narrow pre_focus
**2023207**	block4	HumanSpeech	Pre	tsam3	Object	Narrow	r2	197.325893320283	1	on_focus	3	Narrow on_focus
**2023207**	block4	HumanSpeech	Pre	tsam3	Object	Narrow	r2	214.008547008518	2	on_focus	3	Narrow on_focus
**2023207**	block4	HumanSpeech	Pre	pak3	Subject	Broad	r2	113.907000029428	1	broad_focus	3	Broad focus
**2023207**	block4	HumanSpeech	Pre	pak3	Subject	Broad	r2	167.640463592875	2	broad_focus	3	Broad focus
**2023207**	block4	HumanSpeech	Pre	tsing2	Verb	Broad	r2	224.338538445693	1	broad_focus	2	Broad focus
**2023207**	block4	HumanSpeech	Pre	kau2	Object	Broad	r2	244.585285966252	1	broad_focus	2	Broad focus
**2023207**	block4	HumanSpeech	Pre	tsi2	Object	Broad	r2	165.496654802268	2	broad_focus	2	Broad focus
**2023207**	block4	HumanSpeech	Pre	pak3	Subject	Contrastive	r2	137.451247165529	1	pre_focus	3	Contrastive pre_focus
**2023207**	block4	HumanSpeech	Pre	pak3	Subject	Contrastive	r2	129.703945632173	2	pre_focus	3	Contrastive pre_focus
**2023207**	block4	HumanSpeech	Pre	tsing2	Verb	Contrastive	r2	212.965505042632	1	pre_focus	2	Contrastive pre_focus
**2023207**	block4	HumanSpeech	Pre	kau2	Object	Contrastive	r2	274.697914519322	1	on_focus	2	Contrastive on_focus
**2023207**	block4	HumanSpeech	Pre	tsi2	Object	Contrastive	r2	430.525772213628	2	on_focus	2	Contrastive on_focus
**2023207**	block4	HumanSpeech	Pre	jan1	Subject	Contrastive	r2	241.481481481458	1	pre_focus	1	Contrastive pre_focus
**2023207**	block4	HumanSpeech	Pre	jan1	Subject	Contrastive	r2	211.799886621293	2	pre_focus	1	Contrastive pre_focus
**2023207**	block4	HumanSpeech	Pre	wei3	Verb	Contrastive	r2	236.545827566204	1	pre_focus	3	Contrastive pre_focus
**2023207**	block4	HumanSpeech	Pre	tsam3	Object	Contrastive	r2	226.418797133078	1	on_focus	3	Contrastive on_focus
**2023207**	block4	HumanSpeech	Pre	tsam3	Object	Contrastive	r2	351.407229558504	2	on_focus	3	Contrastive on_focus
**2023207**	block4	HumanSpeech	Pre	pak3	Subject	Narrow	r2	128.916099773278	1	on_focus	3	Narrow on_focus
**2023207**	block4	HumanSpeech	Pre	pak3	Subject	Narrow	r2	151.005291005276	2	on_focus	3	Narrow on_focus
**2023207**	block4	HumanSpeech	Pre	tsing2	Verb	Narrow	r2	183.524199423346	1	post_focus	2	Narrow post_focus
**2023207**	block4	HumanSpeech	Pre	kau2	Object	Narrow	r2	226.009880142556	1	post_focus	2	Narrow post_focus
**2023207**	block4	HumanSpeech	Pre	tsi2	Object	Narrow	r2	217.300615484305	2	post_focus	2	Narrow post_focus
**2023207**	block4	HumanSpeech	Pre	jan1	Subject	Contrastive	r2	209.605442176894	1	on_focus	1	Contrastive on_focus
**2023207**	block4	HumanSpeech	Pre	jan1	Subject	Contrastive	r2	195.158536349027	2	on_focus	1	Contrastive on_focus
**2023207**	block4	HumanSpeech	Pre	wei3	Verb	Contrastive	r2	189.420678527824	1	post_focus	3	Contrastive post_focus
**2023207**	block4	HumanSpeech	Pre	tsam3	Object	Contrastive	r2	180.023688046674	1	post_focus	3	Contrastive post_focus
**2023207**	block4	HumanSpeech	Pre	tsam3	Object	Contrastive	r2	194.616780045351	2	post_focus	3	Contrastive post_focus
**2023207**	block4	HumanSpeech	Pre	piu35	Subject	Contrastive	r2	164.728438781538	1	pre_focus	35	Contrastive pre_focus
**2023207**	block4	HumanSpeech	Pre	mui35	Subject	Contrastive	r2	222.276212072188	2	pre_focus	35	Contrastive pre_focus
**2023207**	block4	HumanSpeech	Pre	tsan3	Verb	Contrastive	r2	220.307634164783	1	on_focus	3	Contrastive on_focus
**2023207**	block4	HumanSpeech	Pre	jin3	Object	Contrastive	r2	192.644041320193	1	post_focus	3	Contrastive post_focus
**2023207**	block4	HumanSpeech	Pre	jin3	Object	Contrastive	r2	191.38170823885	2	post_focus	3	Contrastive post_focus
**2023207**	block4	HumanSpeech	Pre	piu35	Subject	Narrow	r2	125.84574990592	1	pre_focus	35	Narrow pre_focus
**2023207**	block4	HumanSpeech	Pre	mui35	Subject	Narrow	r2	210.951815344458	2	pre_focus	35	Narrow pre_focus
**2023207**	block4	HumanSpeech	Pre	tsan3	Verb	Narrow	r2	204.954648526098	1	on_focus	3	Narrow on_focus
**2023207**	block4	HumanSpeech	Pre	jin3	Object	Narrow	r2	183.87566137568	1	post_focus	3	Narrow post_focus
**2023207**	block4	HumanSpeech	Pre	jin3	Object	Narrow	r2	156.753762110895	2	post_focus	3	Narrow post_focus
**2023207**	block4	HumanSpeech	Pre	jan1	Subject	Broad	r2	191.027261381805	1	broad_focus	1	Broad focus
**2023207**	block4	HumanSpeech	Pre	jan1	Subject	Broad	r2	115.400035993275	2	broad_focus	1	Broad focus

2023207	block4	HumanSpeech	Pre	wei3	Verb	Broad	r2	155.713841091995	1	broad_focus	3	Broad focus
2023207	block4	HumanSpeech	Pre	tsam3	Object	Broad	r2	209.864485476771	1	broad_focus	3	Broad focus
2023207	block4	HumanSpeech	Pre	tsam3	Object	Broad	r2	356.176303854909	2	broad_focus	3	Broad focus
2023207	block4	HumanSpeech	Pre	pak3	Subject	Contrastive	r2	140.426303854838	1	on_focus	3	Contrastive on_focus
2023207	block4	HumanSpeech	Pre	pak3	Subject	Contrastive	r2	156.245545837351	2	on_focus	3	Contrastive on_focus
2023207	block4	HumanSpeech	Pre	tsing2	Verb	Contrastive	r2	143.467876039324	1	post_focus	2	Contrastive post_focus
2023207	block4	HumanSpeech	Pre	kau2	Object	Contrastive	r2	218.962315084752	1	post_focus	2	Contrastive post_focus
2023207	block4	HumanSpeech	Pre	tsi2	Object	Contrastive	r2	261.040004638232	2	post_focus	2	Contrastive post_focus
2023207	block4	HumanSpeech	Pre	piu35	Subject	Contrastive	r2	145.408826581161	1	pre_focus	35	Contrastive pre_focus
2023207	block4	HumanSpeech	Pre	mui35	Subject	Contrastive	r2	219.205936920275	2	pre_focus	35	Contrastive pre_focus
2023207	block4	HumanSpeech	Pre	tsan3	Verb	Contrastive	r2	246.68167016182	1	pre_focus	3	Contrastive pre_focus
2023207	block4	HumanSpeech	Pre	jin3	Object	Contrastive	r2	198.430705615579	1	on_focus	3	Contrastive on_focus
2023207	block4	HumanSpeech	Pre	jin3	Object	Contrastive	r2	219.067460317433	2	on_focus	3	Contrastive on_focus
2023207	block4	HumanSpeech	Pre	pak3	Subject	Narrow	r2	130.453514739202	1	pre_focus	3	Narrow pre_focus
2023207	block4	HumanSpeech	Pre	pak3	Subject	Narrow	r2	162.553636839448	2	pre_focus	3	Narrow pre_focus
2023207	block4	HumanSpeech	Pre	tsing2	Verb	Narrow	r2	150.194093510436	1	pre_focus	2	Narrow pre_focus
2023207	block4	HumanSpeech	Pre	kau2	Object	Narrow	r2	110.924757781845	1	on_focus	2	Narrow on_focus
2023207	block4	HumanSpeech	Pre	tsi2	Object	Narrow	r2	223.767951625064	2	on_focus	2	Narrow on_focus
2023207	block4	HumanSpeech	Pre	piu35	Subject	Broad	r2	123.091247541197	1	broad_focus	35	Broad focus
2023207	block4	HumanSpeech	Pre	mui35	Subject	Broad	r2	225.440709617146	2	broad_focus	35	Broad focus
2023207	block4	HumanSpeech	Pre	tsan3	Verb	Broad	r2	265.813492063444	1	broad_focus	3	Broad focus
2023207	block4	HumanSpeech	Pre	jin3	Object	Broad	r2	195.104591836753	1	broad_focus	3	Broad focus
2023207	block4	HumanSpeech	Pre	jin3	Object	Broad	r2	229.62320483748	2	broad_focus	3	Broad focus
2023207	block4	HumanSpeech	Pre	pak3	Subject	Contrastive	r2	137.546485260714	1	pre_focus	3	Contrastive pre_focus
2023207	block4	HumanSpeech	Pre	pak3	Subject	Contrastive	r2	154.296579743004	2	pre_focus	3	Contrastive pre_focus
2023207	block4	HumanSpeech	Pre	tsing2	Verb	Contrastive	r2	226.286785776551	1	on_focus	2	Contrastive on_focus
2023207	block4	HumanSpeech	Pre	kau2	Object	Contrastive	r2	258.022675736925	1	post_focus	2	Contrastive post_focus
2023207	block4	HumanSpeech	Pre	tsi2	Object	Contrastive	r2	539.208146151509	2	post_focus	2	Contrastive post_focus
2023207	block4	HumanSpeech	Pre	piu35	Subject	Narrow	r2	126.344482237414	1	on_focus	35	Narrow on_focus
2023207	block4	HumanSpeech	Pre	mui35	Subject	Narrow	r2	221.325558795002	2	on_focus	35	Narrow on_focus
2023207	block4	HumanSpeech	Pre	tsan3	Verb	Narrow	r2	212.733351844008	1	post_focus	3	Narrow post_focus
2023207	block4	HumanSpeech	Pre	jin3	Object	Narrow	r2	183.222726127497	1	post_focus	3	Narrow post_focus
2023207	block4	HumanSpeech	Pre	jin3	Object	Narrow	r2	132.971034567618	2	post_focus	3	Narrow post_focus
2023207	block4	HumanSpeech	Pre	piu35	Subject	Contrastive	r2	131.840035253617	1	on_focus	35	Contrastive on_focus
2023207	block4	HumanSpeech	Pre	mui35	Subject	Contrastive	r2	243.547327856504	2	on_focus	35	Contrastive on_focus
2023207	block4	HumanSpeech	Pre	tsan3	Verb	Contrastive	r2	261.665243233438	1	post_focus	3	Contrastive post_focus
2023207	block4	HumanSpeech	Pre	jin3	Object	Contrastive	r2	276.303099017355	1	post_focus	3	Contrastive post_focus
2023207	block4	HumanSpeech	Pre	jin3	Object	Contrastive	r2	324.495302883179	2	post_focus	3	Contrastive post_focus
2023207	block4	HumanSpeech	Pre	piu35	Subject	Narrow	r2	113.056935817781	1	pre_focus	35	Narrow pre_focus
2023207	block4	HumanSpeech	Pre	mui35	Subject	Narrow	r2	251.077997336438	2	pre_focus	35	Narrow pre_focus
2023207	block4	HumanSpeech	Pre	tsan3	Verb	Narrow	r2	225.052726155013	1	pre_focus	3	Narrow pre_focus
2023207	block4	HumanSpeech	Pre	jin3	Object	Narrow	r2	199.73970826743	1	on_focus	3	Narrow on_focus
2023207	block4	HumanSpeech	Pre	jin3	Object	Narrow	r2	225.514977920966	2	on_focus	3	Narrow on_focus
2023207	block4	HumanSpeech	Pre	jan1	Subject	Contrastive	r2	178.846324243978	1	pre_focus	1	Contrastive pre_focus
2023207	block4	HumanSpeech	Pre	jan1	Subject	Contrastive	r2	188.907984246384	2	pre_focus	1	Contrastive pre_focus
2023207	block4	HumanSpeech	Pre	wei3	Verb	Contrastive	r2	212.264472740685	1	on_focus	3	Contrastive on_focus
2023207	block4	HumanSpeech	Pre	tsam3	Object	Contrastive	r2	199.060003488512	1	post_focus	3	Contrastive post_focus
2023207	block4	HumanSpeech	Pre	tsam3	Object	Contrastive	r2	120.651273329827	2	post_focus	3	Contrastive post_focus
2023207	block4	HumanSpeech	Pre	jan1	Subject	Narrow	r2	194.102767918821	1	pre_focus	1	Narrow pre_focus
2023207	block4	HumanSpeech	Pre	jan1	Subject	Narrow	r2	166.418846274155	2	pre_focus	1	Narrow pre_focus
2023207	block4	HumanSpeech	Pre	wei3	Verb	Narrow	r2	185.370370370379	1	on_focus	3	Narrow on_focus
2023207	block4	HumanSpeech	Pre	tsam3	Object	Narrow	r2	198.556533724627	1	post_focus	3	Narrow post_focus
2023207	block4	HumanSpeech	Pre	tsam3	Object	Narrow	r2	188.927761580771	2	post_focus	3	Narrow post_focus
2023207	block4	HumanSpeech	Pre	jan1	Subject	Narrow	r2	159.313144612611	1	on_focus	1	Narrow on_focus
2023207	block4	HumanSpeech	Pre	jan1	Subject	Narrow	r2	111.499078449128	2	on_focus	1	Narrow on_focus
2023207	block4	HumanSpeech	Pre	wei3	Verb	Narrow	r2	140.495950761306	1	post_focus	3	Narrow post_focus
2023207	block4	HumanSpeech	Pre	tsam3	Object	Narrow	r2	115.680820715284	1	post_focus	3	Narrow post_focus
2023207	block4	HumanSpeech	Pre	tsam3	Object	Narrow	r2	308.730158730214	2	post_focus	3	Narrow post_focus
2023207	block4	HumanSpeech	Pre	pak3	Subject	Narrow	r2	120.286686102986	1	pre_focus	3	Narrow pre_focus
2023207	block4	HumanSpeech	Pre	pak3	Subject	Narrow	r2	148.639250106726	2	pre_focus	3	Narrow pre_focus
2023207	block4	HumanSpeech	Pre	tsing2	Verb	Narrow	r2	161.759362330827	1	on_focus	2	Narrow on_focus
2023207	block4	HumanSpeech	Pre	kau2	Object	Narrow	r2	211.035309361819	1	post_focus	2	Narrow post_focus

2023207	block4	HumanSpeech	Pre	tsi2	Object	Narrow	r2	124.267573696102	2	post_focus	2	Narrow post_focus
2023207	block5	HumanSpeech	Post	siu2	Subject	Narrow	r1	90.9479128268771	1	on_focus	2	Narrow on_focus
2023207	block5	HumanSpeech	Post	gwong2	Subject	Narrow	r1	74.9648482301666	2	on_focus	2	Narrow on_focus
2023207	block5	HumanSpeech	Post	cyun4	Verb	Narrow	r1	149.20210508447	1	post_focus	4	Narrow post_focus
2023207	block5	HumanSpeech	Post	laam4	Object	Narrow	r1	91.2511773940281	1	post_focus	4	Narrow post_focus
2023207	block5	HumanSpeech	Post	kau4	Object	Narrow	r1	122.869425548004	2	post_focus	4	Narrow post_focus
2023207	block5	HumanSpeech	Post	siu2	Subject	Contrastive	r1	76.9883497616775	1	on_focus	2	Contrastive on_focus
2023207	block5	HumanSpeech	Post	gwong2	Subject	Contrastive	r1	106.866061980355	2	on_focus	2	Contrastive on_focus
2023207	block5	HumanSpeech	Post	cyun4	Verb	Contrastive	r1	95.0919627110096	1	post_focus	4	Contrastive post_focus
2023207	block5	HumanSpeech	Post	laam4	Object	Contrastive	r1	163.010008601148	1	post_focus	4	Contrastive post_focus
2023207	block5	HumanSpeech	Post	kau4	Object	Contrastive	r1	104.693877551014	2	post_focus	4	Contrastive post_focus
2023207	block5	HumanSpeech	Post	ceoi3	Subject	Broad	r1	73.0299103768459	1	broad_focus	3	Broad focus
2023207	block5	HumanSpeech	Post	ceoi3	Subject	Broad	r1	198.362035973986	2	broad_focus	3	Broad focus
2023207	block5	HumanSpeech	Post	caa4	Verb	Broad	r1	198.0157218443	1	broad_focus	4	Broad focus
2023207	block5	HumanSpeech	Post	ngau4	Object	Broad	r1	115.712053502364	1	broad_focus	4	Broad focus
2023207	block5	HumanSpeech	Post	jau4	Object	Broad	r1	139.304108589826	2	broad_focus	4	Broad focus
2023207	block5	HumanSpeech	Post	ceoi3	Subject	Narrow	r1	54.2784464212929	1	on_focus	3	Narrow on_focus
2023207	block5	HumanSpeech	Post	ceoi3	Subject	Narrow	r1	100.656724228159	2	on_focus	3	Narrow on_focus
2023207	block5	HumanSpeech	Post	caa4	Verb	Narrow	r1	117.754112226905	1	post_focus	4	Narrow post_focus
2023207	block5	HumanSpeech	Post	ngau4	Object	Narrow	r1	153.226969863695	1	post_focus	4	Narrow post_focus
2023207	block5	HumanSpeech	Post	jau4	Object	Narrow	r1	209.79501133786	2	post_focus	4	Narrow post_focus
2023207	block5	HumanSpeech	Post	siu2	Subject	Contrastive	r1	134.320181405897	1	pre_focus	2	Contrastive pre_focus
2023207	block5	HumanSpeech	Post	gwong2	Subject	Contrastive	r1	186.09518655947	2	pre_focus	2	Contrastive pre_focus
2023207	block5	HumanSpeech	Post	cyun4	Verb	Contrastive	r1	78.9189720332644	1	on_focus	4	Contrastive on_focus
2023207	block5	HumanSpeech	Post	laam4	Object	Contrastive	r1	216.818236066359	1	post_focus	4	Contrastive post_focus
2023207	block5	HumanSpeech	Post	kau4	Object	Contrastive	r1	143.30635702558	2	post_focus	4	Contrastive post_focus
2023207	block5	HumanSpeech	Post	wai5	Subject	Narrow	r1	55.6144571649924	1	pre_focus	5	Narrow pre_focus
2023207	block5	HumanSpeech	Post	wai5	Subject	Narrow	r1	63.3410199096573	2	pre_focus	5	Narrow pre_focus
2023207	block5	HumanSpeech	Post	waat3	Verb	Narrow	r1	196.496197089928	1	pre_focus	3	Narrow pre_focus
2023207	block5	HumanSpeech	Post	bui3	Object	Narrow	r1	92.3007697410014	1	on_focus	3	Narrow on_focus
2023207	block5	HumanSpeech	Post	hok3	Object	Narrow	r1	272.011715797419	2	on_focus	3	Narrow on_focus
[truncated: 3,424,838 more chars]
